# A reanalysis of a genome-wide association study on breast cancer in Asian populations using the SG10K_Health reference panel for imputation: a multi-Centre case–control analysis

**DOI:** 10.1093/hmg/ddag015

**Published:** 2026-03-23

**Authors:** Xuling Chang, Shivaani Mariapun, Mengyu Li, Ling Wang, Peh Joo Ho, Alexis Jiaying Khng, Kenneth R Muir, Artitaya Lophatananon, Kristan J Aronson, Rachel A Murphy, Ava Kwong, Chun Hang Au, Sung-Won Kim, Sue K Park, Daniel O Stram, Anna H Wu, Soo-Hwang Teo, Cheng-Har Yip, Nur Aishah Mohd Tai, MyBrCa Investigators, Esther M John, Allison W Kurian, Motoki Iwasaki, Taiki Yamaji, Ji-Yeob Choi, Daehee Kang, Xiao-Ou Shu, Wei Zheng, Mikael Hartman, Ern Yu Tan, Veronique Kiak-Mien Tan, Geok Hoon Lim, Manjeet K Bolla, Alison M Dunning, Joe Dennis, Qin Wang, Marc Naven, Douglas F Easton, SG10K_Health Consortium, Rajkumar s/o Dorajoo, Weang-Kee Ho, Jingmei Li

**Affiliations:** Department of Infectious Diseases, University of Melbourne at the Peter Doherty Institute for Infection and Immunity, 792 Elizabeth St, Melbourne VIC 3000, Australia; Department of Paediatrics, Yong Loo Lin School of Medicine, National University of Singapore, 1E Kent Ridge Road, NUHS Tower Block, Level 12, Singapore 119228, Singapore; Khoo Teck Puat – National University Children’s Medical Institute, National University Health System, 5 Lower Kent Ridge Rd, Singapore 119074, Singapore; Cancer Research Malaysia, Level 1, Subang Jaya Medical Centre South Tower, 1, Jalan SS12/1A, Ss 12, 47500 Subang Jaya, Selangor, Malaysia; Genome Institute of Singapore (GIS), Agency for Science, Technology and Research (A*STAR), 60 Biopolis St, Genome, #02-01, Singapore 138672, Singapore; Genome Institute of Singapore (GIS), Agency for Science, Technology and Research (A*STAR), 60 Biopolis St, Genome, #02-01, Singapore 138672, Singapore; Genome Institute of Singapore (GIS), Agency for Science, Technology and Research (A*STAR), 60 Biopolis St, Genome, #02-01, Singapore 138672, Singapore; Saw Swee Hock School of Public Health, National University of Singapore and National University Health System, 12 Science Drive 2, #10-01, Singapore 117549, Singapore; Genome Institute of Singapore (GIS), Agency for Science, Technology and Research (A*STAR), 60 Biopolis St, Genome, #02-01, Singapore 138672, Singapore; Division of Population Health, Health Services Research and Primary Care, School of Health Sciences, Faculty of Biology, Medicine and Health, University of Manchester, 46 Grafton Street, Manchester M13 9NT, United Kingdom; Division of Population Health, Health Services Research and Primary Care, School of Health Sciences, Faculty of Biology, Medicine and Health, University of Manchester, 46 Grafton Street, Manchester M13 9NT, United Kingdom; Department of Public Health Sciences and Sinclair Cancer Research Institute, Queen’s University, 355 King St. West, Kingston, ON K7L 2X3, Canada; School of Population and Public Health, University of British Columbia, 2206 East Mall, Vancouver, BC V6T 1Z3, Canada; BC Cancer, 600 W 10th Ave, Vancouver, BC V5Z 4E6, Canada; Hong Kong Hereditary Breast Cancer Family Registry, 6/F, No. 3 Tung Wong Road, Shau Kei Wan, Hong Kong; Department of Surgery, University of Hong Kong, 2/F, Professorial Block, Queen Mary Hospital, 102 Pokfulam Road, Hong Kong; Breast Surgery Centre and Cancer Genetics Centre, Hong Kong Sanatorium and Hospital, 2 Village Rd, Happy Valley, Hong Kong; Molecular Pathology Division, Department of Pathology, Hong Kong Sanatorium and Hospital, 2 Village Rd, Happy Valley, Hong Kong; Department of Surgery, 657 Siheung-daero, Yeongdeungpo District, Seoul, Republic of Korea; Department of Preventive Medicine, Seoul National University College of Medicine, 103 Daehak-ro, Jongno-gu, Seoul 03080, Republic of Korea; Integrated Major in Innovative Medical Science, Seoul National University College of Medicine, 103 Daehak-ro, Jongno-gu, Seoul 03080, Republic of Korea; Cancer Research Institute, Seoul National University, 103 Daehak-ro, Jongno-gu, Seoul 03080, Republic of Korea; Department of Preventive Medicine, Keck School of Medicine, University of Southern California, 1975 Zonal Ave, Los Angeles, CA 90033, USA; Department of Population Health and Public Health Sciences, Keck School of Medicine, University of Southern California, Norris Comprehensive Cancer Center, 1441 Eastlake Ave, Los Angeles, CA 90033, USA; Cancer Research Malaysia, Level 1, Subang Jaya Medical Centre South Tower, 1, Jalan SS12/1A, Ss 12, 47500 Subang Jaya, Selangor, Malaysia; Department of Surgery, Faculty of Medicine, University of Malaya, UM Cancer Research Institute, Kuala Lumpur 50603, Malaysia; Sime Darby Medical Centre, No. 1, Jalan SS 12/1A, Ss 12, 47500 Subang Jaya, Selangor, Malaysia; Picaso Hospital, 110, Jalan Professor Khoo Kay Kim, Seksyen 19, 46300 Petaling Jaya, Selangor, Malaysia; Department of Surgery, Faculty of Medicine, University of Malaya, UM Cancer Research Institute, Kuala Lumpur 50603, Malaysia; Cancer Research Malaysia, Level 1, Subang Jaya Medical Centre South Tower, 1, Jalan SS12/1A, Ss 12, 47500 Subang Jaya, Selangor, Malaysia; School of Mathematical Sciences, Faculty of Science and Engineering, University of Nottingham Malaysia, Jalan Broga, 43500 Semenyih, Selangor Darul Ehsan, Malaysia; Breast Cancer Research Unit, University Malaya Cancer Research Institute, Faculty of Medicine, University of Malaya, Kuala Lumpur 50603, Malaysia; Department of Epidemiology and Population Health, Stanford University School of Medicine, Alway Building, 300 Pasteur Drive, Stanford, CA 94305, USA; Department of Medicine, Division of Oncology, Stanford Cancer Institute, Stanford University School of Medicine, Edwards Building, Suite R106, 300 Pasteur Drive, Stanford, CA 94305, USA; Department of Epidemiology and Population Health, Stanford University School of Medicine, Alway Building, 300 Pasteur Drive, Stanford, CA 94305, USA; Department of Medicine, Division of Oncology, Stanford Cancer Institute, Stanford University School of Medicine, Edwards Building, Suite R106, 300 Pasteur Drive, Stanford, CA 94305, USA; Division of Epidemiology, National Cancer Center Institute for Cancer Control, 5-1-1 Tsukiji, Chuo-ku, Tokyo 104-0045, Japan; Division of Epidemiology, National Cancer Center Institute for Cancer Control, 5-1-1 Tsukiji, Chuo-ku, Tokyo 104-0045, Japan; Cancer Research Institute, Seoul National University, 103 Daehak-ro, Jongno-gu, Seoul 03080, Republic of Korea; Department of Biomedical Sciences, Seoul National University Graduate School, 1 Gwanak-ro, Gwanak-gu, Seoul 08826, Republic of Korea; Institute of Health Policy and Management, Seoul National University Medical Research Center, 103 Daehak-ro, Jongno-gu, Seoul 03080, Republic of Korea; Department of Preventive Medicine, Seoul National University College of Medicine, 103 Daehak-ro, Jongno-gu, Seoul 03080, Republic of Korea; Cancer Research Institute, Seoul National University, 103 Daehak-ro, Jongno-gu, Seoul 03080, Republic of Korea; Division of Epidemiology, Department of Medicine, Vanderbilt Epidemiology Center, Vanderbilt-Ingram Cancer Center, Vanderbilt University School of Medicine, 2525 West End Avenue, Suite 600 (IMPH), Nashville, TN 37203-1738, USA; Division of Epidemiology, Department of Medicine, Vanderbilt Epidemiology Center, Vanderbilt-Ingram Cancer Center, Vanderbilt University School of Medicine, 2525 West End Avenue, Suite 600 (IMPH), Nashville, TN 37203-1738, USA; Saw Swee Hock School of Public Health, National University of Singapore and National University Health System, 12 Science Drive 2, #10-01, Singapore 117549, Singapore; Department of Surgery, Yong Loo Lin School of Medicine, National University of Singapore and National University Health Systems, NUHS Tower Block, 1E Kent Ridge Road, Level 8, Singapore 119228, Singapore; Department of Surgery, National University Hospital and National University Health Systems, NUHS Tower Block, 1E Kent Ridge Road, Level 8, Singapore 119228, Singapore; Department of General Surgery, Tan Tock Seng Hospital, 11 Jalan Tan Tock Seng, Level 2, TTSH Medical Centre, Singapore 308433, Singapore; Lee Kong Chian School of Medicine, Nanyang Technological University, 11 Mandalay Rd, Singapore 308232, Singapore; Institute of Molecular and Cell Biology, Agency for Science (IMCB), Technology and Research (A*STAR), 61 Biopolis Dr, Proteos, Singapore 138673, Singapore; Division of Surgery and Surgical Oncology, National Cancer Centre Singapore, 30 Hospital Boulevard, Singapore 168583, Singapore; Department of Breast Surgery, Singapore General Hospital, SGH Block 3, Level 1, Clinic M or J, Outram Road, Singapore 169608, Singapore; SingHealth Duke-NUS Breast Centre, Singapore Health Services (SingHealth), 5 Hospital Drive, Singapore 169609, Singapore, Singapore; SingHealth Duke-NUS Breast Centre, Singapore Health Services (SingHealth), 5 Hospital Drive, Singapore 169609, Singapore, Singapore; Breast Department, KK Women’s and Children’s Hospital, 100 Bukit Timah Rd, Singapore 229899, Singapore; Centre for Cancer Genetic Epidemiology, Department of Public Health and Primary Care, University of Cambridge, CambridgeWorts Causeway, Cambridge, CB1 8RN, United Kingdom; Centre for Cancer Genetic Epidemiology, Department of Oncology, University of Cambridge, CambridgeWorts Causeway, Cambridge, CB1 8RN, United Kingdom; Centre for Cancer Genetic Epidemiology, Department of Public Health and Primary Care, University of Cambridge, CambridgeWorts Causeway, Cambridge, CB1 8RN, United Kingdom; Centre for Cancer Genetic Epidemiology, Department of Public Health and Primary Care, University of Cambridge, CambridgeWorts Causeway, Cambridge, CB1 8RN, United Kingdom; Centre for Cancer Genetic Epidemiology, Department of Public Health and Primary Care, University of Cambridge, CambridgeWorts Causeway, Cambridge, CB1 8RN, United Kingdom; Centre for Cancer Genetic Epidemiology, Department of Public Health and Primary Care, University of Cambridge, CambridgeWorts Causeway, Cambridge, CB1 8RN, United Kingdom; Centre for Cancer Genetic Epidemiology, Department of Oncology, University of Cambridge, CambridgeWorts Causeway, Cambridge, CB1 8RN, United Kingdom; Department of Paediatrics, Yong Loo Lin School of Medicine, National University of Singapore, 1E Kent Ridge Road, NUHS Tower Block, Level 12, Singapore 119228, Singapore; Genome Institute of Singapore (GIS), Agency for Science, Technology and Research (A*STAR), 60 Biopolis St, Genome, #02-01, Singapore 138672, Singapore; School of Mathematical Sciences, Faculty of Science and Engineering, University of Nottingham Malaysia, Jalan Broga, 43500 Semenyih, Selangor Darul Ehsan, Malaysia; Genome Institute of Singapore (GIS), Agency for Science, Technology and Research (A*STAR), 60 Biopolis St, Genome, #02-01, Singapore 138672, Singapore; Department of Surgery, Yong Loo Lin School of Medicine, National University of Singapore and National University Health Systems, NUHS Tower Block, 1E Kent Ridge Road, Level 8, Singapore 119228, Singapore; National Cancer Centre Singapore (NCCS), Singapore Health Services (SingHealth), Singapore30 Hospital Boulevard, Singapore 168583, Singapore

**Keywords:** genome-wide association study, breast neoplasms, genotype imputation, reference panels, Asian ancestry

## Abstract

Genome-wide association studies (GWAS) have identified numerous genetic variants linked to breast cancer risk, but most discoveries come from European populations, limiting their applicability to other populations. Here, we show that the choice of genotype imputation reference panel, an essential step for GWAS, affects variant detection in Asian populations. Using two large breast cancer datasets from the Breast Cancer Association Consortium (n = 38 954 Asian samples), we compared the 1000 Genomes (1KG) reference panel with SG10K_Health (SG10K), an Asian-specific panel. SG10K imputed more rare variants and achieved higher accuracy for rare alleles (MAF < 0.001), while 1KG performed better for common variants in some contexts. Differences in panel performance influenced association signals, including breast cancer candidate loci such as *FGFR2*, *TOX3*, and *ESR1*. Together, these findings support the use of population-specific imputation panels as a means to improve variant discovery in underrepresented populations.

## Introduction

Genome-wide association studies (GWAS) have advanced our understanding of the genetic architecture underlying complex diseases, including breast cancer [1–11]. However, the predominance of European-ancestry populations in GWAS has limited the applicability of findings across diverse ethnic groups, resulting in gaps in precision medicine and risk prediction globally [12]. For instance, genetic risk factors associated with breast cancer risk identified in European populations are often not fully transferable to populations of other ancestries [13]. This lack of representation limits the generalizability of GWAS findings and reduces our ability to capture the genetic diversity of human populations comprehensively.

In recent years, efforts to include more diverse populations in genetic studies, including those of Asian descent, have been underway but remain insufficient [14–22]. Asian populations are very heterogeneous and exhibit distinct genetic architectures and allele frequencies compared to European populations. There is thus a need for tailored GWAS approaches, including using a population-specific reference panel for genotype imputation [23]. Imputation, a widely used step in GWAS, allows for the prediction of genotypes at untyped variants based on linkage disequilibrium patterns observed in a reference panel [24]. However, the lack of population-specific reference panels for genotype imputation remains a major challenge [25–30]. While imputation using mixed-ancestry reference panels is largely effective, its accuracy declines if the population studied is not well represented due to differences in linkage disequilibrium patterns and allele frequencies [31, 32].

The inclusion of diverse Asian ethnic groups, which are increasingly represented in contemporary studies, adds to the complexity of addressing population-specific differences in imputation [33–35]. To address these gaps, this study examines the need for population-specific imputation panels by re-analyzing a breast cancer GWAS dataset comprising Asian samples.

## Results

We analyzed genotype data from 12 109 Asian samples in the iCOGS array (199 174 variants) and 26 845 Asian samples in the OncoArray (483 469 variants) from the Breast Cancer Association Consortium (BCAC) (Supplementary Table 1). After quality control, we performed genotype imputation using two reference panels: the 1000 Genomes Project Phase 3 panel (1KG), a diverse panel with 26 global populations including East Asians, and the SG10K_Health panel (SG10K), a population-specific resource comprising 9770 whole-genome sequences from Chinese, Indian, and Malay individuals from Singapore. We then conducted genome-wide association analyses to identify breast cancer susceptibility loci, comparing the performance of these two imputation strategies.

### Comparison of variant imputation between reference panels

**Figure 1:**
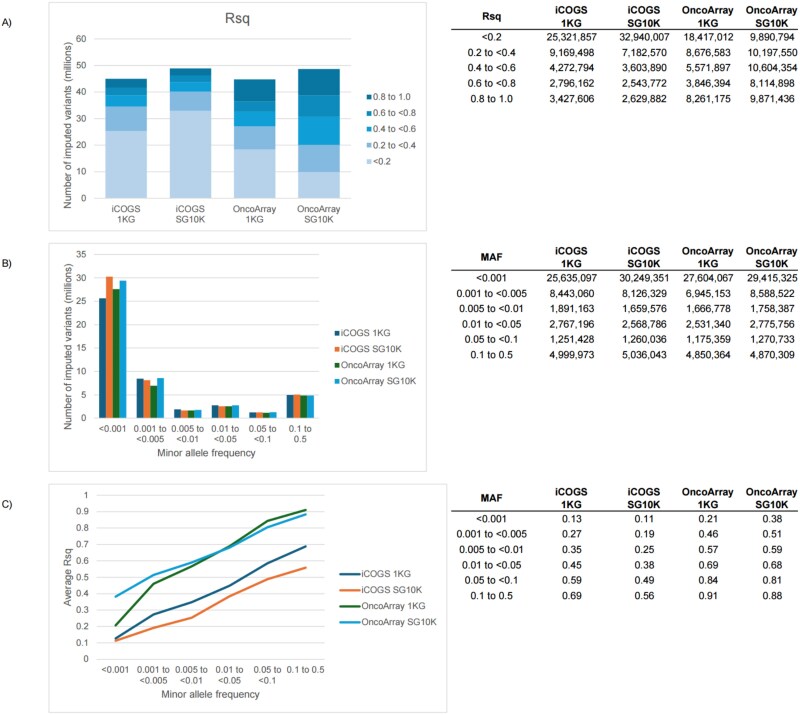
Comparison of the breast cancer association consortium (BCAC) Asian dataset imputed by 1KG and SG10K. A) Total number of variants imputed by different panels and their distribution across Rsq score bins. B) Number of imputed variants across allele frequency bins. C) Average Rsq score across allele frequency bins. MAF: Minor allele frequency.

The two reference panels yielded different imputation outcomes in terms of variant coverage and quality (Fig. 1). Although both panels are comparable in number of sites (~49 million, Supplementary Table 2), 1KG yielded ~ 3.9 million fewer imputed variants than SG10K (Fig. 1A). An advantage of SG10K over 1KG was observed for rare variants (MAF < 0.001), both in the number of imputed variants (Fig. 1B) and average imputation quality scores (Rsq) (Fig. 1C). For the less dense iCOGS, the average Rsq scores were similar between 1KG and SG10K for rare variants (MAF < 0.001) but higher for 1KG compared to SG10K across all other MAF bins.

### Quality control and variant filtering for further analyses

To ensure high-quality data for association testing, we applied stringent filtering criteria to the imputed variants. We excluded non-biallelic variants, extremely rare variants (MAF < 0.0001), and SNPs with low imputation quality (Rsq < 0.3 for common variants and Rsq < 0.6 for rare variants) from further analysis. Detailed information for the exclusion in each step is shown in Supplementary Table 3. A total of 6 768 661 and 5 322 463 common variants (MAF ≥ 0.01) were retained after quality control using 1KG and SG10K, respectively, for iCOGS. For OncoArray, the numbers were 7 675 795 and 7 464 119, respectively. A total of 1 266 334 and 1 296 394 rare variants (0.0001 < MAF < 0.01) were retained after quality control using 1KG and SG10K, respectively, for iCOGS. For OncoArray, the numbers were 3 879 283 and 9 071 436, respectively. While the majority of the variants included in both panels had an absolute MAF difference of 10% and below, there were variants with a range of absolute MAF differences up to more than 40% (Supplementary Table 4).

### Sample exclusions and final dataset composition

After imputation and variant quality control, we further refined our study population to focus on invasive breast cancer cases with complete clinical information. Further exclusions for the iCOGS dataset included 1524 duplicates across the two genotyping arrays (PI_HAT>0.9), non-invasive breast cancer (*n* = 447), cancers of unknown invasiveness (*n* = 53), and missing information on age at diagnosis for cases or age at interview for controls (*n* = 5), resulting in 10 164 unique samples for association analyses (4441 cases and 5723 controls) (Supplementary Table 1). For OncoArray, 24 601 unique samples (12 636 invasive breast cancer cases and 11 965 controls) remained after excluding non-invasive breast cancer (*n* = 1388), cancers of unknown invasiveness (*n* = 589), missing information on age at diagnosis for cases or age at interview for controls (*n* = 440), and a single Asian in the European KARMA study (Supplementary Table 1).

### Genome-wide association signals across arrays and imputation panels

**Figure 2:**
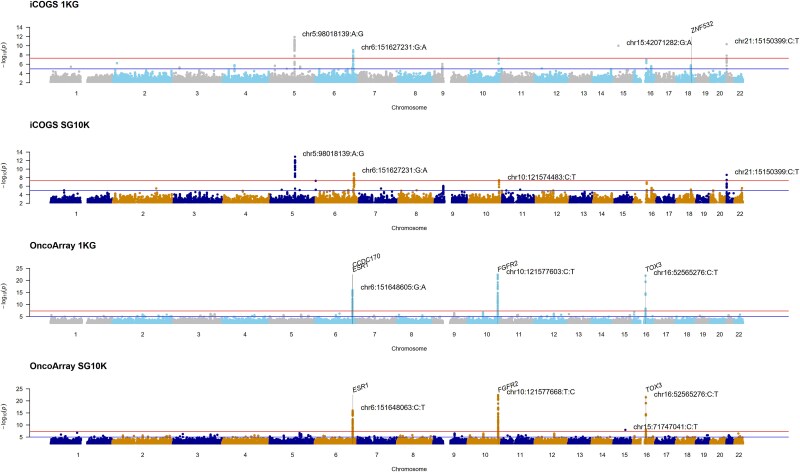
Manhattan plots by array and imputation panel. Grey vertical lines denote significant genes from the MAGMA analysis (*P* < 0.05/20000).

We performed genome-wide association testing for breast cancer using logistic regression adjusted for age, study site, and population structure. Manhattan plots by array and imputation panel reveal similar signal peaks (Fig. 2). However, chr15:42071282:G:A, which surpassed the genome-wide association significance threshold found in the iCOGS array imputed by 1KG (OR [95% CI] = 8.7 [4.52 to 16.8], *P* = 1.00e-10), was not found on the SG10K panel. This variant was not replicated on the OncoArray (OR = 0.77 [0.57 to 1.03], *P* = 0.079).

### Comparison of top signals and replication across panels

**Figure 3:**
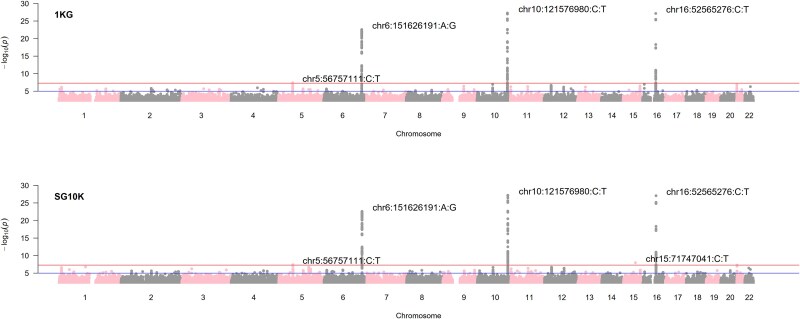
Manhattan plot of meta-analysis results by imputation panel.

In the iCOGs data imputed with the SG10K panels, the *P*-value of chr10:121574483:C:T was marginally improved from 5.46e-8 to 4.02e-8, as compared to imputation done with the 1KG. This region was replicated by the OncoArray by different top variants after 1KG imputation (chr10:121577603:C:T (0.83 [0.80 to 0.86], *P* = 4.66e-23)) and SG10K imputation (chr10:121577668:T:C (0.83 [0.80 to 0.86], *P* = 3.69e-23)). For OncoArray, a rare variant chr15:71747041:C:T (MAF = 0.0006) was present on SG10K (OR = 12.0 [5.13 to 28.2], *P* = 1.06e-8), but was not imputed on 1KG. Overall, the observed -log10P of SG10K imputed variants were smaller for iCOGS as seen in the quantile-quantile and scatter plots (Supplementary Fig. 1 and Supplementary Fig. 2). The converse was true for the OncoArray. To assess the consistency of findings across both arrays, we conducted meta-analyses combining results from iCOGS and OncoArray for each imputation panel. Meta-analysis results of the iCOGS and OncoArray arrays for each imputation panel are shown in Fig. 3 and Supplementary Fig. 3.

### Overlap of candidate variants between imputation panels

Beyond genome-wide significant loci, we examined the broader landscape of suggestive associations to understand panel-specific variant discovery. We explored candidate breast cancer-associated variants (*P* < 0.01) uniquely imputed by a single panel or an intersection of 1KG and SG10K (Supplementary Fig. 4). The overlap of candidate breast cancer-associated variants imputed by both panels was 33.5% (iCOGS, 27 269 of 81 388) and 47.6% (OncoArray, 40 563 of 85 165) for common variants and 12.4% (iCOGS, 2066 of 16 691) and 10.7% (OncoArray, 9686 of 90 254) for rare variants.

### Gene-based association findings by imputation panel

Beyond genome-wide significant loci, we examined the broader landscape of suggestive associations to understand panel-specific variant discovery (Supplementary Table 5). Gene-set analysis on mixed rare and common variant data found *ZNF532* on chromosome 18 to be significant after multiple testing corrections for 20 000 genes (480 variants, *P* = 8.77e-7) when imputed by 1KG but not on SG10K (429 variants, *P* = 8.92e-6) (Fig. 2, Supplementary Tables 6–7). For the OncoArray data, *FGFR2* (639 variants, *P* = 2.01e-13), *CCDC17* (788 variants, *P* = 6.05e-7), *TOX3* (421 variants, *P* = 7.89e-7), and *ESR1* (2069 variants, *P* = 8.90e-7) were found to be significant when imputed by 1KG (Fig. 2, Supplementary Table 8). When imputed by SG10K, *FGFR2* (735 variants, *P* = 3.74e-14), *TOX3* (566 variants, *P* = 8.13e-7), and *ESR1* (2415 variants, *P* = 1.88e-6) were significant, but not *CCDC17* (887 variants, *P* = 3.72e-6) (Fig. 2, Supplementary Table 9).

## Discussion

The comparison between 1KG, a diverse reference panel with 26 populations, and SG10K, a population-specific reference panel comprising East Asians, South Asians and South East Asians, in combination with a dense array (OncoArray) and less dense array (iCOGS) reveals that the choice of reference panel for improving imputation quality may depend on the density of the array and whether the research focus is on rare or common variants.

In our study, SG10K showed advantages in imputing rare variants (MAF < 0.001), yielding a higher number of imputed variants and higher average imputation quality scores for these variants. However, this was only observed in the OncoArray, suggesting that using a larger and population-specific reference panel for imputation in a more densely populated array is beneficial for rare variant analysis. This is consistent with previous observations that the accuracy of identifying low-frequency and rare variants increases as reference panels grow larger and genome-wide genotyping arrays become more densely populated [36]. Notably, we also showed that the higher imputation quality of the rare variants resulted in a higher proportion of sub-genome-wide significant rare variants. By contrast, for dense arrays, using a population-specific reference panel did not result in improved imputation accuracy and higher yield of significant variants for common variants.

Conversely, 1KG demonstrated higher average imputation quality scores in the less dense iCOGS array for both rare and common variants, suggesting that diverse reference panels better capture long-range haplotypes when genotyped markers are sparse. This density-dependent performance has important implications: while population-specific panels excel for rare variant imputation in modern high-density arrays, diverse panels such as 1KG or TOPMed may be preferable when array density is a limiting factor. Manhattan plots indicated similar signal peaks, though some significant variants were only identified by one panel. Gene-set analysis identified significant genes such as *ZNF532*, *FGFR2*, *TOX3*, and *ESR1*, with some differences in statistical significance levels and variants between the panels. Overall, SG10K showed smaller -log10P values for iCOGS-imputed variants, while 1KG showed smaller values for OncoArray. The overlap of breast cancer-associated variants between the panels was moderate, with a higher overlap for common variants than for rare variants. These differences demonstrate the impact of reference panel choice on genetic association results, as relying on a single panel may lead to missed associations.

While the SG10K_Health panel focuses on three Singaporean populations (i.e. Chinese, Indian, and Malay), this focus is both a strength and a limitation. These groups collectively represent a substantial portion of the global Asian population and are underrepresented in most large-scale genomic reference panels. Their distinct demographic histories and genetic architectures allow SG10K to capture population-specific variants, providing clear advantages for imputing Asian-specific rare variants that are absent or extremely rare in the 1KG global sample. However, this same specificity may result in reduced imputation accuracy for variants that are common elsewhere but rare in SG10K. For instance, the failure to impute chr15:42071282:G:A in SG10K (genome-wide significant in 1KG-imputed iCOGS data) may reflect either true absence/extreme rarity of this variant in Asian populations or limitations in SG10K's coverage. The inability to replicate this signal in OncoArray (*P* = 0.079) suggests it may be a false positive, but panel-specific ascertainment could also contribute to such discrepancies. This suggests that panel choice can influence variant discovery.

More broadly, while SG10K provides excellent coverage and is representative of our study samples, its applicability to other Asian populations remains to be established. This point is especially pertinent considering the migration history of Singaporean ethnic groups, many of whom originated from southern regions [37]. In contrast, the 1KG panel, despite having fewer Asian samples overall, captures greater diversity across Asian populations (e.g. Han Chinese [Beijing], Japanese [Tokyo], Southern Han Chinese, Chinese Dai, Kinh Vietnamese), which may explain some of its advantages for certain variants and in populations not represented in SG10K.

Our choice of imputation quality thresholds (Rsq ≥ 0.3 for common variants and Rsq ≥ 0.6 for rare variants) follows established guidelines to strike a balance between retaining potentially informative variants and excluding poorly imputed genotypes. These thresholds impact rare variant analysis, where we observed that SG10K imputed more rare variants meeting quality criteria in OncoArray (9 071 436 vs. 3 879 283 for 1KG). At the same time, this means that variants just below threshold (essentially an arbitrary value) are excluded in a way that differs between panels. Our MAF threshold (≥0.0001) also excluded extremely rare variants where we observed the most pronounced differences between panels, which may underestimate the full potential of SG10K for rare variant imputation.

From our results, several future directions can be explored for improving imputation in Asian populations. First, the development of larger, more comprehensive Asian reference panels that include currently underrepresented populations would enhance imputation across the full diversity of Asian ancestry. Second, methods for combining multiple reference panels (such as using SG10K for Asian-specific variants alongside 1KG or TOPMed for broader coverage) may offer advantages over relying on any single panel. Third, array design could be optimized for Asian populations by including variants identified in Asian-specific sequencing projects, reducing dependence on imputation for the most informative variants.

Based on our findings, we offer the following guidance for researchers conducting GWAS in Asian populations. For studies focused on common variant associations using modern high-density arrays, both 1KG and population-specific panels such as SG10K are likely to perform well, with panel choice having modest impact on results. For rare variant analyses in densely genotyped Asian cohorts, population-specific panels matched to the study population's ancestry provide clear advantages in variant discovery and imputation quality. For lower-density arrays, diverse reference panels may offer better overall imputation quality across the allele frequency spectrum. For multi-ethnic studies, consider performing imputation separately for each major ancestral group using population-matched reference panels, then combining results in downstream analyses. Finally, when the study population's ancestry is heterogeneous or includes underrepresented populations in available reference panels, sensitivity analyses using multiple reference panels can help identify robust associations and flag panel-specific findings that require additional validation.

Overall, our comparative analysis indicates that selecting an appropriate reference panel requires considering array density, variant frequency, and population ancestry together. While no single panel is universally superior, SG10K excels for rare variant imputation in high-density arrays of Asian populations, whereas 1KG offers advantages for common variants and less dense arrays through broader haplotype coverage. These findings emphasize the importance of matching reference panel characteristics to specific study objectives and highlight opportunities for future methodological advances through panel integration and expanded population representation.

## Materials and methods

### Ethics statement

This study was approved by the A*STAR institutional review board (IRB Reference: 2021–159; approval date 10 November 2021). Each of the participating studies in BCAC was approved by the respective local ethics review boards. Written informed consent was obtained from all study participants by the respective studies.

### Study population

As described in the Results, genotype data were obtained from BCAC for two large genotyping arrays, iCOGS and OncoArray (concept 710) [38, 39]. There were 113 547 samples with genotype (*n* = 199 961 variants) and phenotype data in the iCOGS dataset. Of the 164 248 samples with genotype data (*n* = 494 444 variants) in the OncoArray dataset, 164 205 had corresponding phenotype data. A subset of 12 500 and 27 501 samples genotyped using the iCOGS and OncoArray, respectively, were Asians (i.e. EthnicityGeno = ‘Asian’) (Supplementary Table 1). Ancestral lineage was determined through a principal component analysis utilizing informative markers (details described in [2]).

### Pre-imputation processing of genotype data

Standard quality control (QC) procedures were applied to both datasets. In brief, samples with extremes in heterozygosity (outside 3 standard deviations, n_iCOGS_ = 373 and n_OncoArray_ = 287) were removed. All samples had call rates above 95%. Identity-by-state and identity-by-descent measures were performed by pair-wise comparison of samples to detect first-degree familial relationships. One sample from each pair with the lower call rate was excluded from further analysis (n_iCOGS_ = 18 and n_OncoArray_ = 369). A total of 12 109 and 26 845 samples from iCOGS and OncoArray, respectively, remained for imputation (Supplementary Fig. 5).

Variants with identical positions (n_iCOGS_ = 19 and n_OncoArray_ = 93), indels (n_iCOGS_ = 49 and n_OncoArray_ = 9960), and strand orientation ‘U’ (n_iCOGS_ = 139 and n_OncoArray_ = 492) were removed. Additionally, variants located on the reverse strand were flipped. Further exclusions were made for chromosome Y and MT variants and call rates below 95%, resulting in 199 258 and 483 711 variants post-QC for iCOGS and OncoArray, respectively. Finally, 199 174 and 483 469 variants were successfully lifted over from hg19 to hg38 using the UCSC liftOver tool.

### Phasing and imputation

Phasing was performed by Eagle v2.4 [40]. Imputation (Minimac4 [41]) using the 1000 Genomes (1KG) reference panel (GRCh38/hg38, Phase 3, Version 5, [26]) was performed with the Michigan Imputation Server (v1.7.3) (Supplementary Table 2).

The SG10K_Health reference panel (r5.5.1) (SG10K) comprises 9770 whole-genome sequences from healthy Asian volunteers of Chinese, Indian, and Malay ethnicities from Singapore [42]. Phasing by Eagle 2.4.1 and SG10K imputation using Minimac4 was performed on the RAPTOR analytics platform [43].

### Statistical analyses

The associations between genetic variants and breast cancer were examined using SAIGE v0.45 with adjustment for age, study site, and the first 10 principal components as covariates. SAIGE is an R package that can adjust for sample-relatedness and case–control imbalance and produce accurate *P*-values using saddlepoint approximation [44].

Generalized gene-set analysis on mixed rare and common variant data was performed using MAGMA (v1.10) using SNP-wise ‘mean’ models (i.e. test of mean variant association, uses the sum of squared variant Z-statistics as test statistic) on default settings [45]. Summary statistics (*P*-values from the single variant association analyses) and a reference LD panel (1KG, Phase 3, build 38, 585 East Asians, 70 692 015 variants across chromosomes 1 to 22, https://www.cog-genomics.org/plink/2.0/resources, updated 2022-08-04, accessed 2024-05-09) were used [46]. Gene locations (build 38, updated 19/09/2018, accessed 2024-05-09) were downloaded from the Magma website (https://cncr.nl/research/magma/). To capture variants in regulatory regions, a window (35 kilobase pair (kb) upstream and 10 kb downstream) was added (−-annotate window = 35,10). Supplementary Table 5 provides an overview of the number of variants and genes mapped.

Meta-analyses of iCOGS and OncoArray GWAS results (1KG: 11780092 variants; SG10K: 16558953 variants) were performed using Metal (2011-03-25 release) with genomic control set to ‘ON’ [47].

## Supplementary Material

Supplementary_merged_ddag015

## Data Availability

The BCAC dataset was approved under concept #710 (individual-level data) is not made publicly available due to restraints imposed by the ethics committees of individual studies. Access to the BCAC data is governed by a Data Access Co-ordinating Committee (DACC), which can be reached at https://www.ccge.medschl.cam.ac.uk/breast-cancer-association-consortium-bcac/data-data-access. The SG10K_Health panel is available through the SG10K_Health Data Access Portal (https://www.npm.sg/phase-i-sg10k-health/). Requestors should be bona fide researchers and are required to submit a Data Access Request outlining the proposed research for approval by the Data Access Committee, which convenes monthly. Data for this study were obtained under Data Access Application NPM00028.

## Computer code

No custom code was generated for this study. All analyses were conducted using publicly available software, which is cited in the Methods. Custom parameters and non-default settings are detailed in the Methods section.
