# Supplementary material for: A reanalysis of a genome-wide association study on breast cancer in Asian populations using the SG10K_Health reference panel for imputation: a multi-Centre case–control analysis
Source: Hum Mol Genet. 2026 Mar 23;35(6):ddag015. doi: 10.1093/hmg/ddag015 (PMC13017919; doi:10.1093/hmg/ddag015)
Supplement: Supplementary_merged_ddag015 [file supplementary_merged_ddag015.pdf]

SUPPLEMENTARY FILES

Supplementary Table 1. Description of analytical samples. *Invasive breast cancer.

Supplementary Table 2. Overview of imputation reference panels compared.

Supplementary Table 3. Number of imputed variants by genotyping array and reference panel. MAF: Minor allele frequency

Supplementary Table 4. Minor allele frequency (MAF) comparisons of variants in datasets with Rsq>0.3 imputed by both reference panels. A) iCOGS, B) OncoArray.

Supplementary Table 5. Number of variants and genes mapped for MAGMA analysis. MAF: minor allele frequency.

Supplementary Table 6. MAGMA results for iCOGS imputed by 1KG.

Supplementary Table 7. MAGMA results for iCOGS imputed by SG10K.

Supplementary Table 8. MAGMA results for OncoArray imputed by 1KG.

Supplementary Table 9. MAGMA results for OncoArray imputed by SG10K.

Supplementary Figure 1. Quantile-quantile plots by array.

Supplementary Figure 2. Pvalue comparisons of variants P<0.01 in datasets imputed by both reference panels. A) iCOGS MAF≥0.01, B) iCOGS MAF≥0.0001 and <0.01, C) OncoArray MAF≥0.01, D) OncoArray MAF≥0.0001 and <0.01.

Supplementary Figure 3. Quantile-quantile plot of meta-analysis results.

Supplementary Figure 4. Overlap of panel-specific candidate loci (P<0.01) in datasets imputed by both reference panels. A) iCOGS, MAF ≥ 0.01; B) iCOGS, 0.0001 ≤ MAF < 0.01; C) OncoArray, MAF ≥ 0.01; D) OncoArray, 0.0001 ≤ MAF < 0.01.

Supplementary Figure 5. Principal analysis component plot of A) 12,109 women genotyped on iCOGS and B) 26,845 women genotyped on OncoArray.

Supplementary Table 1. Description of analytical samples. *Invasive breast cancer.

Acronym	Name	Reference	Design	Country	Controls	Cases*	Controls	Cases*	Median age at interview or diagnosis (IQR)
iCOGS									
ACP	Asia Cancer Program		Hospital based case-control study	Thailand	636	416	627	413	46 (40 to 53)
HERPACC	Hospital-based Epidemiologic Research Program at Aichi Cancer Center	10.1002/ijc.24505	Hospital-based case-control study	Japan	1376	561	1376	561	51 (44 to 59)
LAABC	Los Angeles County Asian-American Breast Cancer Case-Control Study	10.3945/ajcn.2008.26915, 10.1007/s10549-011-1640-x, 10.1002/ijc.31018	Population-based case-control	USA	990	808	982	805	53 (46 to 61)
MYBRCA	Malaysian Breast Cancer Genetic Study	10.1186/bcr3347	Hospital-based case-control study	Malaysia	610	769	422	10	49 (37 to 57)
SBCGS	Shanghai Breast Cancer Genetic Study	10.1038/ng.318	Population-based case-control study, cohort study	China	892	829	892	827	52 (46 to 61)
SEBCS	Seoul Breast Cancer Study	10.1158/1055-9965.EPI-04-0330, 10.1007/s10549-007-9730-5	Hospital-based case-control study	Korea	1129	1027	1129	1021	49 (44 to 56)
SGBCC	Singapore Breast Cancer Cohort	PMID: 33901219	Hospital based breast cancer cohort and population-based controls	Singapore	502	441	59	28	51 (44.5 to 61.5)
TWBCS	Taiwanese Breast Cancer Study	10.1158/1055-9965.EPI-07-0116, 10.1093/carcin/bgn233	Hospital-based case-control study	Taiwan	236	776	236	776	51 (45 to 58)
OncoArray									
ACP	Asia Cancer Program		Hospital based case-control study	Thailand	642	448	426	413	47 (38 to 53)
CBCS	Canadian Breast Cancer Study	10.1016/j.canep.2013.04.006, 10.1007/s10549-013-2596-9, 10.1136/oemed-2013-101482, 10.1007/s10552-013-0312-z	Population-based case-control study	Canada	987	821	170	253	51 (45 to 61)
HERPACC	Hospital-based Epidemiologic Research Program at Aichi Cancer Center	10.1002/ijc.24505	Hospital-based case-control study	Japan	283	231	283	231	51 (45 to 60)
HKBCS	Hong Kong Breast Cancer Study	10.1007/s10549-012-2292-1, 10.1371/journal.pone.0043994	Hospital-based case-control study	Hong Kong	454	478	451	477	46 (39 to 52)
KOHBRA	Korean Hereditary Breast Cancer Study	10.1016/j.clon.2010.11.007	Population-based Case-Control study	Korea	665	1292	665	1290	40 (34 to 48)
MYBRCA	Malaysian Breast Cancer Genetic Study	10.1186/bcr3347	Hospital-based case-control study	Malaysia	3590	3359	3349	3234	52 (46 to 59)
NC-BCFR	Northern California Breast Cancer Family Registry	10.1186/bcr801	Population-based familial case-control study	USA	207	1450	52	445	48 (42 to 55)
NGOBCS	Nagano Breast Cancer Study	10.1038/jhg.2009.13	Hospital-based case-control study	Japan	366	366	366	366	53 (46 to 61)
SBCGS	Shanghai Breast Cancer Genetic Study	10.1038/ng.318	Population-based case-control study, cohort study	China	935	815	935	815	55 (50 to 63)
SEBCS	Seoul Breast Cancer Study	10.1158/1055-9965.EPI-04-0330, 10.1007/s10549-007-9730-5	Hospital-based case-control study	Korea	1107	1102	1104	1098	48 (42 to 56)

SGBCC	Singapore Breast Cancer Cohort	PMID: 33901219	Hospital based breast cancer cohort and population-based controls	Singapore	4047	3607	3908	3500	51 (45 to 59)
TWBCS	Taiwanese Breast Cancer Study	10.1158/1055-9965.EPI-07-0116, 10.1093/carcin/bgn233	Hospital-based case-control study	Taiwan	256	514	256	514	49.5 (43 to 57)

Supplementary Table 2. Overview of imputation reference panels compared.

Reference panel	Samples	Sites (millions)	Ancestry distribution	Panel content	Phasing and imputation
1KG (Phase 3 (Version 5)) (10.1038/nature15393)	2,504	49,143,605	26 populations: Africans (26%), Admixed American (14%), East Asian (20%), European (20%), South Asian (20%)	chr1-22 and X; SNVs and indels	EAGLE2+ Minimac4 on Michigan Imputation Server (10.1038/ng.3656)
SG10K_Health r5.5.1 (10.21203/rs.3.rs-3376868/v1)	9,770	49,090,104	Chinese (58%), Indians (24%) and Malays (18%).	chr1-22 and X; SNVs and indels	EAGLE2 (2.4.1) + Minimac4 (1.0.2) on Raptor (10.1016/j.isci.2023.106546)

Supplementary Table 3. Number of imputed variants by genotyping array and reference panel. MAF: Minor allele frequency

	iCOGS		OncoArray	
	**1KG**	**SG10K**	**1KG**	**SG10K**
**Common (MAF≥0.01)**				
Total imputed + genotyped	9,181,634	9,028,118	8,889,225	9,249,980
Excluded	2,412,973	3,705,655	1,213,430	1,785,861
Non-bi-allelic	986,587	1,395,955	958,819	1,433,955
Low imputation quality (Rsq<0.3)	1,426,386	2,309,700	254,611	351,906
Retained	6,768,661	5,322,463	7,675,795	7,464,119
Overlap	5,003,471		6,900,391	
Unique	1,765,190	318,992	775,404	563,728
**Rare (MAF≥0.0001 and <0.01)**				
Total imputed + genotyped	35,995,702	40,061,973	36,312,359	39,840,111
Excluded	34,729,368	38,765,579	32,433,076	30,768,675
Non-bi-allelic	2,710,339	5,092,062	2,739,609	5,054,062
Low imputation quality (Rsq<0.6)	31,851,162	33,543,391	29,030,291	25,177,510
Extremely low MAF (<0.01%)	167,867	130,126	663,176	537,103
Retained	1,266,334	1,296,394	3,879,283	9,071,436
Overlap	558,156		2,668,527	
Unique	708,178	738,238	1,210,756	6,402,909

Supplementary Table 4. Minor allele frequency (MAF) comparisons of variants in datasets with Rsq>0.3 imputed by both reference panels. A) iCOGS, B) OncoArray.

Absolute MAF difference	iCOGS	OncoArray
>0.1	41,819	60,489
>0.2	8,207	28,910
>0.3	3,384	14,830
>0.4	982	5,719

Supplementary Table 5. Number of variants and genes mapped for MAGMA analysis. MAF: minor allele frequency.

	All		MAF≥0.01		MAF<0.01	
	iCOGS	OncoArray	iCOGS	OncoArray	iCOGS	OncoArray
1KG						
Variants with MAF >0.01% after exclusion of samples with missing covariates	7,998,306	11,528,223	6,768,661	7,675,795	1,229,643	3,852,427
Valid pvalues	7,732,699	11,117,370	6,534,354	7,366,114	1,198,345	3,751,256
Genes containing valid SNPs	18,921	19,035	18,893	19,022	18,027	18,909
SG10K						
Variants with MAF >0.01% after exclusion of samples with missing covariates	6,558,058	16,450,325	5,322,463	7,474,119	1,235,594	8,986,205
Valid pvalues	6,111,941	12,189,134	5,260,075	7,343,596	851,866	4,845,538
Genes containing valid SNPs	18,498	19,037	18,362	18,988	18,575	18,887

Supplementary Table 6. MAGMA results for iCOGS imputed by 1KG.

STRAND	SYMBOL	CHR	START	STOP	NSNPS	NPARAM	N	ZSTAT	P
+	ZNF532	18	58827600	58996477	480	47	10164	4.7798	8.7718e-07
-	PRIMA1	14	93708298	93824035	352	63	10164	4.2136	1.2569e-05
+	TAB2	6	149182924	149421613	839	52	10164	3.7903	7.5228e-05
-	FILIP1	6	75284948	75528829	509	35	10164	3.7822	7.7725e-05
+	LSP1	11	1817970	1902263	436	74	10164	3.6856	0.00011407
-	COMMD8	4	47440793	47498665	117	19	10164	3.5712	0.00017768
-	DCAF12	9	34076383	34161773	259	22	10164	3.5698	0.00017861
-	ZNF821	16	71849680	71919867	186	28	10164	3.5486	0.00019368
+	FAM120B	6	170255703	170417071	156	11	10164	3.5209	0.00021501
+	IST1	16	71859408	71940639	237	32	10164	3.4952	0.00023683
-	PKD1L3	16	71919538	72034978	530	42	10164	3.4381	0.00029288
-	IMPA1	8	81646916	81721354	203	19	10164	3.437	0.00029408
-	AP1G1	16	71719000	71844073	366	29	10164	3.4342	0.00029717
+	ATXN1L	16	71810991	71867333	145	26	10164	3.4143	0.00031978
-	SLC10A5	8	81683656	81729972	163	13	10164	3.3795	0.00036309
+	LAMA2	6	128848141	129526569	1592	91	10164	3.3665	0.00038071
-	FGFR2	10	121468330	121633656	629	68	10164	3.357	0.00039396
-	DLL1	6	170272200	170341555	34	4	10164	3.3541	0.00039805
-	UBAP2	9	33911693	34083949	528	24	10164	3.3468	0.0004087
-	RNF170	8	42839637	42932931	28	5	10164	3.3227	0.00044577
+	MYO6	6	75714176	75929537	561	28	10164	3.2897	0.00050145
-	PHLPP2	16	71634924	71759701	333	21	10164	3.2521	0.00057281
-	THAP1	8	42826674	42878331	21	4	10164	3.25	0.00057696
+	CORT	1	10414719	10462003	158	20	10164	3.2254	0.00062906
-	TMEM30A	6	75242922	75319916	145	17	10164	3.2197	0.00064157
+	CCDC170	6	151459040	151631193	783	63	10164	3.2147	0.00065296
-	ZFAND1	8	81691331	81756304	195	17	10164	3.1942	0.00070113
-	TOX3	16	52426425	52582802	362	41	10164	3.1926	0.00070495
+	CHMP4C	8	81697453	81769515	201	20	10164	3.1739	0.00075195
+	ARF6	14	49858018	49907054	61	12	10164	3.1586	0.0007927
+	HABP2	10	113515831	113599602	341	45	10164	3.1576	0.00079541
+	UBE2R2	9	33782164	33930403	386	20	10164	3.1455	0.00082905
-	PIGZ	3	196936343	197003871	120	18	10164	3.1438	0.00083397
+	ATOH1	4	93793927	93839991	126	13	10164	3.1278	0.00088049
-	CDH22	20	46163733	46343829	300	48	10164	3.1219	0.00089857
-	CADM1	11	115163625	115539523	595	64	10164	3.1045	0.00095305
-	NCBP2	3	196925402	196977593	113	17	10164	3.1016	0.00096235
-	SHCBP1L	1	182889865	182988418	211	23	10164	3.097	0.00097752
+	CASS4	20	56376548	56469340	182	26	10164	3.0908	0.00099804
-	CHRNA6	8	42742620	42803786	84	4	10164	3.0618	0.0011002
+	HOOK3	8	42861890	43040539	49	6	10164	3.0541	0.0011287
-	TOP2B	3	25587905	25699905	254	20	10164	3.0396	0.0011846
-	TAT	16	71556851	71612095	140	17	10164	3.0371	0.0011941
-	COX7A2	6	75227675	75289885	141	12	10164	3.0258	0.0012399
+	SENP6	6	75566509	75728285	455	25	10164	3.0124	0.0012959
+	PRSS3	9	33715466	33809231	234	23	10164	3.0013	0.0013443
-	COL12A1	6	75074326	75241202	387	34	10164	2.964	0.0015183
-	CPXM2	10	123712291	123926984	367	41	10164	2.9572	0.0015521
+	MARVELD3	16	71591138	71651965	160	22	10164	2.9528	0.0015747
+	TEX37	2	88489317	88539585	167	13	10164	2.9431	0.0016245
+	CHRNB3	8	42662419	42747407	95	5	10164	2.9422	0.0016297
+	FBXW8	12	116875951	117042498	381	25	10164	2.9094	0.0018108
-	CDKN2A	9	21957752	22030043	192	26	10164	2.9073	0.0018227
+	APITD1-COR	1	10395102	10462003	204	24	10164	2.8945	0.0018987
+	SETD9	5	56874259	56935532	187	23	10164	2.8922	0.001913
-	NXPH2	2	138659157	138815241	337	32	10164	2.8738	0.0020276
-	LOC1053751	7	4116653	4172174	243	26	10164	2.8618	0.0021062
+	RTFDC1	20	56433585	56528886	210	14	10164	2.8576	0.0021343
-	DTNBP1	6	15512801	15698058	335	24	10164	2.8513	0.0021771
+	APITD1	1	10395102	10452815	183	21	10164	2.8454	0.0022179

STRAND	SYMBOL	CHR	START	STOP	NSNPS	NPARAM	N	ZSTAT	P
-	LAMA4	6	112097931	112289722	551	62	10164	2.8331	0.0023047
+	NUBPL	14	31526385	31871224	953	19	10164	2.8247	0.002366
-	PLA2G16	11	63564462	63651926	65	13	10164	2.8148	0.0024406
-	LMOD3	3	69098672	69157595	241	20	10164	2.8112	0.0024682
+	STYX	14	52695165	52784989	181	21	10164	2.789	0.0026432
+	KLHL5	4	39009801	39152823	418	13	10164	2.7834	0.0026897
-	KRIT1	7	92188969	92281128	134	21	10164	2.7756	0.002755
-	GNPNAT1	14	52765193	52826668	140	20	10164	2.7686	0.0028147
-	SBSPON	8	73054543	73128272	230	25	10164	2.7669	0.0028294
-	MIER3	5	56909602	56987130	295	31	10164	2.7634	0.0028597
-	NEMF	14	49773278	49888073	107	15	10164	2.7386	0.0030855
+	XKR9	8	70634363	71075788	936	11	10164	2.7359	0.0031107
+	CLEC2L	7	139488928	139554985	42	9	10164	2.7311	0.0031564
-	PLA2G2E	1	19910307	19958617	153	21	10164	2.7282	0.0031839
+	ANKIB1	7	92211216	92411770	265	29	10164	2.7247	0.0032178
+	ARHGEF4	2	130801916	131057253	496	44	10164	2.7143	0.0033203
-	SUN2	22	38724714	38791019	122	23	10164	2.7097	0.0033671
-	MEGF9	9	120590918	120749487	221	15	10164	2.7077	0.0033873
+	MAK16	8	33450167	33511260	161	9	10164	2.7062	0.0034033
+	UBLCP1	5	159228081	159296040	141	22	10164	2.704	0.0034255
-	FUT10	8	33298611	33508478	573	32	10164	2.6994	0.0034731
-	SERPINA6	14	94294248	94358351	251	37	10164	2.6974	0.0034942
+	GAR1	4	109780510	109834737	202	19	10164	2.6944	0.0035258
-	MGAT4D	4	140432258	140534829	156	9	10164	2.6939	0.0035314
-	FABP12	8	81514981	81625596	312	25	10164	2.6912	0.0035602
+	GORAB	1	170497122	170563834	91	16	10164	2.6866	0.0036094
+	FNTA	8	43021299	43095788	25	3	10164	2.678	0.0037028
+	SIGLEC15	18	45790580	45852556	111	10	10164	2.677	0.0037147
+	GTPBP1	22	38669557	38748173	178	12	10164	2.6746	0.0037409
+	FAM209A	20	56489729	56536152	87	8	10164	2.6728	0.0037607
-	LOC1053789	1	1055635	1104326	66	4	10164	2.6698	0.0037945
-	RNF223	1	1061746	1109307	77	4	10164	2.6682	0.0038129
+	GOLGA7B	10	97815238	97881578	157	21	10164	2.663	0.0038724
+	TSNAX	1	231493653	231576524	123	22	10164	2.6604	0.0039022
-	DFFA	1	10450531	10507556	159	25	10164	2.6597	0.0039103
-	JOSD1	22	38675543	38736418	136	12	10164	2.6525	0.003995
+	DAAM2	6	39757366	39914877	573	63	10164	2.6448	0.0040864
-	ZNF286B	17	18648429	18717259	63	8	10164	2.6439	0.0040978
-	COX7B2	4	46724827	46944267	618	33	10164	2.639	0.004157
-	CDKN2B	9	21992903	22044313	152	18	10164	2.6122	0.0044978
-	MTERF1	7	91862876	91915720	130	13	10164	2.6029	0.0046218
-	ZNF417	19	57895774	57951616	99	9	10164	2.6019	0.0046354
-	CPSF1	8	144383231	144444400	101	7	10164	2.5992	0.0046718
-	ZNF814	19	57859379	57924074	128	12	10164	2.5986	0.0046798
-	LACTB2	8	70627266	70704212	155	10	10164	2.5937	0.0047479
-	KANSL2	12	48643211	48717252	51	8	10164	2.5919	0.0047722
+	PAPPA	9	116118792	116412321	549	82	10164	2.5824	0.004906
+	DHRS7B	17	21091275	21201972	153	14	10164	2.5821	0.0049103
+	HGSNAT	8	43105449	43212827	45	3	10164	2.5788	0.0049566
+	C15orf26	15	81064408	81159175	112	12	10164	2.5592	0.0052451
-	NFXL1	4	47837233	47949667	297	19	10164	2.5588	0.0052511
-	MYSM1	1	58644739	58735092	385	19	10164	2.5576	0.0052698
+	CELF2	10	10969641	11346675	847	88	10164	2.5552	0.0053062
-	PITX1	5	135017734	135069274	89	18	10164	2.5442	0.0054767
+	AGTR1	3	148662871	148753003	202	18	10164	2.5406	0.0055335
+	TRIM16L	17	18662998	18746118	74	9	10164	2.5332	0.0056512
+	ICOS	2	203901731	203971579	215	27	10164	2.533	0.0056545
+	FAM209B	20	56491195	56546521	123	13	10164	2.5329	0.0056568
+	PLEKHS1	10	113716262	113792433	285	30	10164	2.5299	0.0057039
+	NXPH1	7	8398955	8762963	1245	89	10164	2.527	0.0057526
+	CNDP2	18	74461265	74533454	117	8	10164	2.5228	0.0058206
+	ELMOD2	4	140489158	140563770	82	6	10164	2.5222	0.0058314
-	C10orf111	10	15085385	15132319	159	20	10164	2.5208	0.0058552

STRAND	SYMBOL	CHR	START	STOP	NSNPS	NPARAM	N	ZSTAT	P
-	PTCH2	1	44809844	44877944	197	19	10164	2.5152	0.0059477
+	RRH	4	109792994	109854707	196	18	10164	2.5063	0.0061003
+	POTEA	8	43257370	43376407	101	4	10164	2.5056	0.0061122
-	CFI	4	109721221	109837225	386	29	10164	2.5043	0.0061344
-	ZNF622	5	16441519	16505327	134	16	10164	2.5025	0.006166
+	JARID2	6	15210975	15532042	696	44	10164	2.5001	0.0062072
+	ADCK5	8	144339015	144403242	33	2	10164	2.4926	0.0063403
-	CCR7	17	40543769	40600484	62	14	10164	2.4883	0.0064177
-	TCTEX1D4	1	44795908	44842442	134	16	10164	2.4874	0.0064338
+	BCL2L11	2	111085618	111178445	224	30	10164	2.4847	0.0064839
+	RNMT	18	13691647	13774556	168	16	10164	2.4843	0.0064907
+	ADGRL2	1	81271199	82002738	2323	179	10164	2.4816	0.0065406
+	ODF3L1	15	75688944	75737688	118	22	10164	2.4778	0.0066103
-	FAM227A	22	38568120	38691629	327	15	10164	2.4739	0.0066832
-	PDCD2	6	170565572	170619774	64	3	10164	2.4736	0.0066887
+	CNDP1	18	74499457	74595025	168	28	10164	2.4721	0.0067154
-	METTL21A	2	207570635	207660928	141	15	10164	2.4689	0.0067765
-	RNF19A	8	100247059	100371218	298	19	10164	2.4679	0.0067956
-	OSBPL3	7	24786537	25015212	418	61	10164	2.4651	0.0068495
-	MARCH8	10	45444585	45629906	684	19	10164	2.4583	0.0069805
+	DNASE2B	1	84363532	84425895	181	21	10164	2.4566	0.0070133
-	FBXL6	8	144345428	144393523	24	2	10164	2.4485	0.0071717
+	IYD	6	150333861	150414629	405	25	10164	2.4468	0.0072056
-	CSPG4	15	75664322	75747848	217	29	10164	2.4359	0.0074273
+	CBY1	22	38621653	38683850	210	9	10164	2.4358	0.00743
-	TTI2	8	33488509	33548185	167	14	10164	2.433	0.0074866
+	PAK2	3	196704857	196842647	364	20	10164	2.4325	0.0074967
+	LOC1053785	10	46248257	46295118	270	27	10164	2.4279	0.0075928
-	HRASLS2	11	63542759	63600089	57	13	10164	2.4267	0.0076195
+	PTER	10	16401943	16523745	426	31	10164	2.4243	0.0076699
-	SORCS1	10	106563663	107216126	1550	101	10164	2.4228	0.0077012
+	TOMM22	22	38646949	38694760	168	8	10164	2.4155	0.0078578
-	CFAP46	10	132798392	132978163	574	35	10164	2.4121	0.0079302
-	RASGRF1	15	78949947	79125873	311	43	10164	2.4111	0.007952
-	TMEM249	8	144343226	144390609	19	1	10164	2.4067	0.0080479
+	SLC52A2	8	144323557	144371286	4	1	10164	2.4052	0.0080822
-	SCRT1	8	144320565	144371281	4	1	10164	2.4052	0.0080822
-	CD300LF	17	74684308	74748059	155	30	10164	2.4015	0.0081647
+	LMX1B	9	126579443	126711032	392	37	10164	2.401	0.0081753
+	GBP6	1	89328877	89398160	195	27	10164	2.4002	0.0081938
-	SAA2-SAA4	11	18221355	18283674	204	29	10164	2.399	0.008219
-	TESC	12	117028923	117134446	282	37	10164	2.3906	0.0084093
-	PTAFR	1	28137166	28228936	122	13	10164	2.3871	0.0084905
+	ATPIF1	1	28201091	28248105	114	5	10164	2.383	0.0085853
-	PSMB1	6	170525116	170588329	92	3	10164	2.3819	0.0086115
+	SPATA8	15	96748407	96795615	131	20	10164	2.3761	0.0087481
+	LOC644277	11	111410886	111466605	215	33	10164	2.3717	0.0088522
+	POMK	8	43058506	43133440	24	4	10164	2.371	0.00887
-	LOC1053781	6	170451022	170498114	122	10	10164	2.3667	0.0089735
-	CCR10	17	42669402	42716827	59	10	10164	2.3653	0.0090086
+	TBP	6	170519333	170582870	89	3	10164	2.363	0.0090647
+	RPS8	1	44740574	44788740	108	15	10164	2.3582	0.009181
-	ZNF418	19	57911884	57970408	120	13	10164	2.3561	0.0092339
-	CCNT1	12	48678458	48751998	72	12	10164	2.3521	0.0093338
-	SLC39A4	8	144402414	144453034	96	7	10164	2.3516	0.0093468
-	FAM210A	18	13653347	13761592	228	17	10164	2.3496	0.0093957
+	PSMC6	14	52672163	52737998	169	29	10164	2.3495	0.009399
+	SPON1	11	13927637	14278133	862	49	10164	2.3491	0.0094098
+	KIF2C	1	44704712	44777767	211	27	10164	2.3466	0.0094733
+	ABRACL	6	138993682	139053302	166	30	10164	2.3455	0.0095003
-	FAM25C	10	47985322	48034791	12	3	10164	2.3455	0.0095016
-	PPP3R2	9	101581615	101630001	171	22	10164	2.3428	0.0095692
+	FAM21C	10	45692200	45802964	245	21	10164	2.3397	0.0096486

STRAND	SYMBOL	CHR	START	STOP	NSNPS	NPARAM	N	ZSTAT	P
-	RASSF2	20	4770023	4858645	382	53	10164	2.3366	0.0097298
+	CHRNA7	15	31995483	32182521	281	26	10164	2.3349	0.0097733
+	LOC1053691	1	1026207	1076390	34	3	10164	2.3301	0.0099004
-	SLC5A8	12	101146216	101245274	340	32	10164	2.3299	0.0099045
-	MORN4	10	97604553	97669156	171	19	10164	2.3289	0.009931
+	SCPEP1	17	56943107	57016768	108	10	10164	2.3256	0.010019
+	MYZAP	15	57556904	57695364	654	51	10164	2.3238	0.010067
-	PLEKHM3	2	207811288	208075372	416	23	10164	2.3237	0.010071
-	LHFP	13	39332892	39638219	935	79	10164	2.3215	0.010129
-	RGS8	1	182633341	182788307	361	32	10164	2.3049	0.010587
-	EPG5	18	45790580	46002339	427	22	10164	2.3013	0.010687
-	SAA4	11	18221355	18271837	174	27	10164	2.3005	0.01071
-	FAM13B	5	137927949	138086961	233	10	10164	2.2955	0.010852
-	LRRC2	3	46505385	46615060	468	31	10164	2.2933	0.010915
-	DMC1	22	38499154	38605286	164	19	10164	2.2929	0.010927
-	ZFAND4	10	45604567	45707962	302	14	10164	2.2922	0.010946
+	TMEM253	14	21063937	21113724	43	6	10164	2.2867	0.011106
-	SAA2	11	18228233	18283674	177	26	10164	2.2853	0.011148
-	C1orf159	1	1071818	1151356	156	8	10164	2.2851	0.011154
+	SAA1	11	18231261	18279977	164	24	10164	2.2821	0.011241
+	ZFP1	16	75097509	75182234	316	28	10164	2.2783	0.011355
+	B3GALT1	2	167258061	167880856	1661	115	10164	2.2774	0.01138
-	ASIC2	17	33003087	34191806	3158	243	10164	2.2754	0.011441
+	LOC1019288	6	154475097	154532300	184	24	10164	2.2746	0.011465
+	ENPP7	17	79695919	79752926	4	2	10164	2.2735	0.011498
-	PLEKHH3	17	42657914	42712030	64	11	10164	2.2722	0.011536
-	LRIG3	12	58862149	58955538	330	35	10164	2.2712	0.011569
+	TDGF1	3	46539555	46592463	252	18	10164	2.2696	0.011614
+	ACTN4	19	38612616	38740531	446	40	10164	2.2657	0.011736
-	PDGFA	7	487258	555668	3	2	10164	2.2594	0.011931
-	DUSP8	11	1544051	1607831	210	20	10164	2.2563	0.012027
+	CLIP4	2	29062676	29193813	389	31	10164	2.2485	0.012272
-	GCNT7	20	56481492	56560925	178	18	10164	2.2476	0.012301
+	LACC1	13	43844284	43903933	264	28	10164	2.2442	0.01241
+	PEX14	1	10439946	10640758	530	44	10164	2.2392	0.012573
+	CORO2B	15	68524275	68737806	246	41	10164	2.2391	0.012575
-	PPIAL4G	1	148472548	148518285	1	1	10164	2.2365	0.012661
-	CLGN	4	140378453	140462661	126	11	10164	2.2326	0.012788
+	KCNB2	8	72502391	72948349	1041	45	10164	2.2315	0.012825
-	OR51G1	11	4913374	4959339	152	19	10164	2.2232	0.013101
-	EIF4G3	1	20796292	21212320	721	22	10164	2.2222	0.013134
-	CST9L	20	23554732	23603749	159	6	10164	2.221	0.013174
-	HP1BP3	1	20732677	20822310	152	16	10164	2.2209	0.013178
-	CEP162	6	84114241	84262890	248	29	10164	2.2202	0.013201
-	ST8SIA6	10	17305414	17490510	780	36	10164	2.2201	0.013205
+	NTS	12	85839295	85892992	155	8	10164	2.2179	0.013281
+	PLEKHO1	1	150114434	150170065	32	15	10164	2.2166	0.013325
-	VPS28	8	144413601	144463563	125	10	10164	2.2092	0.01358
-	DNAJC8	1	28190278	28268031	184	8	10164	2.2083	0.013612
-	TRIM25	17	56877909	56949048	61	9	10164	2.2062	0.013684
+	GCOM1	15	57556904	57727557	706	53	10164	2.2006	0.013882
+	SENP5	3	196832856	196944714	243	16	10164	2.2001	0.013901
+	PKD2L2	5	137854211	137952747	103	11	10164	2.1993	0.013929
-	KLF7	2	207064137	207208851	355	35	10164	2.1986	0.013953
+	BTBD19	1	44772457	44824199	124	17	10164	2.1979	0.01398
-	TBC1D28	17	18624434	18679433	27	7	10164	2.1946	0.014098
-	ZZEF1	17	3994445	4178020	491	18	10164	2.1945	0.0141
-	COX5A	15	74910275	74973154	188	35	10164	2.194	0.014117
+	KAAG1	6	24321903	24368284	125	19	10164	2.1868	0.014379
+	C16orf78	16	49338808	49409431	189	20	10164	2.1843	0.01447
+	MAFK	7	1495732	1553043	180	5	10164	2.1819	0.014557
+	CDC40	6	110145421	110242232	185	11	10164	2.18	0.014631
-	SYCE1	10	133543900	133603291	236	7	10164	2.1798	0.014637

STRAND	SYMBOL	CHR	START	STOP	NSNPS	NPARAM	N	ZSTAT	P
-	ATL2	2	38284094	38413139	388	13	10164	2.178	0.014704
+	CROCC	1	16860707	16982979	63	8	10164	2.1762	0.014768
+	IGLL5	22	22852780	22905834	19	5	10164	2.176	0.014776
-	RNF166	16	88686495	88741421	256	40	10164	2.1751	0.014812
-	DBNDD1	16	89994865	90055131	274	31	10164	2.1736	0.014867
+	HEXDC	17	82383376	82452640	145	16	10164	2.1733	0.01488
+	ATP10D	4	47450271	47603486	431	40	10164	2.1685	0.01506
-	DEFA3	8	7005869	7053339	188	16	10164	2.1677	0.015092
+	MMP26	11	4953194	5002429	170	25	10164	2.1654	0.015177
+	CYB5D2	17	4108168	4167701	169	15	10164	2.1645	0.015215
-	SH2D5	1	20709731	20767640	148	20	10164	2.1637	0.015245
+	ENDOV	17	80380117	80448086	9	5	10164	2.1624	0.015294
-	RASSF9	12	85790703	85871540	218	6	10164	2.1617	0.015319
-	HERC1	15	63598618	63868949	692	26	10164	2.1607	0.015358
-	LOC1053721	18	74444602	74491948	104	18	10164	2.1573	0.01549
-	CPM	12	68841175	68998240	442	56	10164	2.157	0.015504
-	ACBD7	10	15065475	15123776	206	24	10164	2.1557	0.015554
-	RSL24D1	15	55171314	55232033	232	12	10164	2.1549	0.015587
-	BEST4	1	44772045	44827828	133	18	10164	2.1515	0.01572
+	GBA3	4	22657914	22829575	299	28	10164	2.1508	0.015746
+	MRPS26	20	3011029	3058250	145	22	10164	2.1501	0.015775
-	HPS5	11	18268670	18357498	199	19	10164	2.1475	0.015877
-	HSPG2	1	21812232	21972297	411	40	10164	2.1462	0.01593
+	OR8G1	11	124214676	124260611	305	13	10164	2.1393	0.016205
-	PGGT1B	5	115190241	115297887	198	28	10164	2.1381	0.016253
-	ADAMTS6	5	65138736	65517027	881	45	10164	2.1365	0.016321
+	DHODH	16	71973744	72035417	301	32	10164	2.1358	0.016348
+	PCNT	21	46289100	46455769	691	36	10164	2.1353	0.016369
+	RALGPS1	9	126879774	127233166	726	28	10164	2.1344	0.016407
+	CYP2E1	10	133492363	133549116	169	11	10164	2.1343	0.016408
+	UTRN	6	144250298	144863034	1627	86	10164	2.1321	0.016498
-	RPP25	15	74945102	74992434	131	23	10164	2.1311	0.016542
-	NEK10	3	27097336	27404460	1093	39	10164	2.1292	0.016618
-	PKHD1	6	51604685	52122625	1625	79	10164	2.1285	0.01665
-	TRIM49	11	89787655	89843575	39	9	10164	2.1277	0.016682
-	INTS1	7	1460277	1539382	215	6	10164	2.1234	0.016859
+	MOGAT2	11	75682819	75741286	249	35	10164	2.1189	0.017051
-	FRMD4B	3	69158782	69576340	1525	99	10164	2.1185	0.017066
-	TPTE2	13	19412877	19596574	299	11	10164	2.1183	0.017074
+	LOC1053691	11	89786830	89836934	42	9	10164	2.1182	0.017079
+	CNTNAP1	17	42647614	42709993	84	9	10164	2.1177	0.017102
+	SND1	7	127617119	128102607	993	40	10164	2.1163	0.01716
+	PI4K2A	10	97605686	97686434	221	20	10164	2.1161	0.01717
-	C21orf58	21	46290430	46358899	264	31	10164	2.1156	0.017188
-	WDR61	15	78273233	78334726	186	36	10164	2.1113	0.017375
-	TONSL	8	144418780	144479488	140	11	10164	2.1107	0.017401
-	LOC1019275	11	1544607	1591457	142	12	10164	2.1086	0.017491
+	ATP1A1	1	116337185	116414774	62	8	10164	2.1074	0.017541
-	EGR3	8	22677659	22728302	81	11	10164	2.1073	0.017547
-	NGF	1	115275916	115373384	326	46	10164	2.1054	0.01763
-	CCDC122	13	43813931	43914690	457	28	10164	2.1046	0.017663
+	SCOC	4	140222286	140392556	297	31	10164	2.0983	0.01794
-	MEGF6	1	3477942	3659757	159	17	10164	2.0979	0.017958
+	PHC1	12	8879684	8951467	189	19	10164	2.0976	0.017971
+	LAMP5	20	9479318	9540524	172	26	10164	2.0968	0.018007
+	SLC9A3R1	17	74713612	74779360	145	24	10164	2.0963	0.018026
+	STX11	6	144105051	144201939	313	31	10164	2.0962	0.018033
+	OR8G5	11	124206158	124333224	675	21	10164	2.0922	0.018209
+	FSCN1	7	5557805	5616656	192	23	10164	2.092	0.018218
-	ADAMTS10	19	8570240	8645753	16	2	10164	2.0911	0.018258
+	PLK3	1	44764952	44815995	115	16	10164	2.0903	0.018294
+	FBXL22	15	63559830	63612428	125	16	10164	2.0901	0.018306
-	VIPR2	7	159018175	159179957	405	21	10164	2.09	0.018309

STRAND	SYMBOL	CHR	START	STOP	NSNPS	NPARAM	N	ZSTAT	P
+	SLC6A17	1	110115510	110212202	184	19	10164	2.0888	0.018365
-	PRODH2	19	35789990	35848299	156	37	10164	2.0881	0.018393
+	C18orf8	18	23468470	23541807	184	26	10164	2.0875	0.018422
-	EEA1	12	92762509	92964331	405	26	10164	2.0871	0.018439
+	ZHX2	8	122746349	122984515	511	93	10164	2.0861	0.018486
+	CCDC109B	4	109525199	109698645	689	37	10164	2.0853	0.018521
+	OGDH	7	44571522	44719070	307	18	10164	2.0831	0.018619
-	HRK	12	116850420	116916427	159	23	10164	2.08	0.018761
+	GAS8	16	89984629	90054971	328	30	10164	2.0789	0.018812
-	SFXN4	10	119130913	119200709	238	26	10164	2.0778	0.018863
+	PIM3	22	49925495	49974072	148	21	10164	2.0753	0.018978
-	TPSG1	16	1211488	1261384	83	7	10164	2.075	0.018992
+	KIFC2	8	144431149	144484116	124	10	10164	2.0735	0.019063
-	LOC1019272	13	80031700	80110306	195	24	10164	2.0734	0.019067
-	OGFOD3	17	82379210	82453637	166	16	10164	2.0714	0.019161
+	SPAG1	8	100123003	100251904	299	16	10164	2.0707	0.019195
+	LOC1053715	17	15782863	15940886	246	11	10164	2.0703	0.019213
+	C20orf144	20	33627286	33673915	71	19	10164	2.0703	0.019214
-	PTHLH	12	27948084	28007731	207	26	10164	2.0683	0.019304
-	ATXN1	6	16289112	16796490	1432	192	10164	2.0666	0.019388
+	NHLRC2	10	113819632	113922506	128	30	10164	2.0652	0.019452
-	TPSB2	16	1218336	1265184	68	6	10164	2.0641	0.019506
+	ARL6IP5	3	69049939	69116088	200	23	10164	2.064	0.01951
+	LOC1053781	14	105446769	105496591	26	6	10164	2.0624	0.019583
+	MDFI	6	41602037	41664246	131	32	10164	2.0617	0.01962
-	OR51A4	11	4936159	4982100	146	16	10164	2.0599	0.019705
+	TPSAB1	16	1205677	1252554	91	8	10164	2.0573	0.019831
+	RAB37	17	74636117	74757335	323	54	10164	2.0565	0.019868
-	TAAR1	6	132634984	132681003	229	22	10164	2.0562	0.019881
-	B9D2	19	41344417	41399540	98	7	10164	2.0553	0.019927
-	KRT222	17	40645620	40700164	73	7	10164	2.0541	0.019982
-	SERPINA10	14	94273313	94328271	204	39	10164	2.0524	0.020067
+	OR2J2	6	29138534	29184574	213	23	10164	2.0513	0.020119
-	TOMM7	7	22802632	22857852	247	22	10164	2.0512	0.020126
-	HSD17B3	9	96225306	96348983	505	46	10164	2.0497	0.020197
-	FBXL17	5	107849033	108417098	1766	67	10164	2.0477	0.020295
+	KLHDC2	14	49732589	49795660	80	11	10164	2.0471	0.020323
-	CTTNBP2	7	117700648	117908507	323	44	10164	2.0468	0.02034
-	ANXA8	10	47457993	48028768	46	10	10164	2.0457	0.020391
+	OR51L1	11	4963983	5009930	164	27	10164	2.0431	0.020523
+	KIAA1109	4	122117333	122372759	464	20	10164	2.0429	0.020532
-	ELFN2	22	37330644	37462470	369	40	10164	2.0398	0.020683
+	LHX1	17	36902475	36954615	76	13	10164	2.0376	0.020797
+	LOC646670	15	72602065	72659548	55	13	10164	2.0364	0.020857
-	EBF1	5	158685908	159134780	1013	85	10164	2.0361	0.020869
+	ATRNL1	10	115058365	115958999	1939	40	10164	2.0357	0.020888
-	LOC1053709	15	32018535	32065596	101	9	10164	2.0344	0.020957
-	ZNF219	14	21080046	21139704	81	14	10164	2.0312	0.021117
+	PANX3	11	124576557	124630356	130	19	10164	2.0309	0.021135
-	DBX1	11	20146214	20195324	159	34	10164	2.0302	0.021169
-	PRSS22	16	2842727	2893630	124	10	10164	2.03	0.021176
-	LOC1053778	13	113731875	113787839	166	16	10164	2.0293	0.021214
-	CCBE1	18	59420939	59732643	1305	99	10164	2.0278	0.021291
-	SMARCE1	17	40617720	40682851	90	8	10164	2.0269	0.021339
+	SPDL1	5	169548634	169614778	165	15	10164	2.0256	0.021403
-	IL2	4	122441470	122491495	104	20	10164	2.0251	0.021428
-	TMEM184A	7	1532235	1595821	110	11	10164	2.0246	0.021455
+	VWA5B1	1	20255907	20370221	252	20	10164	2.0207	0.021655
+	SLC14A1	18	45688756	45762520	347	21	10164	2.0204	0.021671
-	UBA3	3	69044730	69115373	204	23	10164	2.0196	0.021713
+	LRRC8B	1	89489144	89611539	309	18	10164	2.0175	0.02182
+	ADAD1	4	122343966	122439815	162	22	10164	2.0154	0.021932
+	SALL3	18	78945275	79008969	181	24	10164	2.0147	0.021969

STRAND	SYMBOL	CHR	START	STOP	NSNPS	NPARAM	N	ZSTAT	P
+	CPS1	2	210442682	210689107	568	43	10164	2.0131	0.02205
+	OR6C4	12	55516227	55562156	140	6	10164	2.0127	0.022073
+	LOC1002892	9	61295592	61471342	3	2	10164	2.0126	0.022076
+	LOC1053782	10	43877405	43924496	176	27	10164	2.0117	0.022125
+	C11orf84	11	63778374	63837718	90	18	10164	2.0064	0.022409
-	LOC1053785	10	119188557	119242578	176	18	10164	2.0053	0.022464
-	MLLT3	9	20331665	20657543	675	62	10164	2.005	0.022481
+	ANKRD30B	18	14713160	14865465	285	13	10164	2.004	0.022536
+	XIRP2	2	166853487	167269753	1756	66	10164	2.0038	0.022547
+	GJA8	1	147867820	147925287	124	23	10164	2.002	0.022644
-	NOC2L	1	934203	994299	1	1	10164	2.0007	0.022713
+	PLEKHN1	1	931497	985108	1	1	10164	2.0007	0.022713
+	SAMD11	1	890741	954581	1	1	10164	2.0007	0.022713
+	KLHL17	1	924952	975720	1	1	10164	2.0007	0.022713
+	CCDC70	13	51826981	51876236	105	18	10164	2.0005	0.022726
-	OR2B3	6	29076208	29122313	215	36	10164	2.0001	0.022744
-	WASF1	6	110089817	110224874	281	12	10164	1.9993	0.022786
+	ANTXRL	10	46251997	46340419	341	27	10164	1.9988	0.022815
+	ASB7	15	100567550	100661701	311	32	10164	1.9983	0.02284
-	OR51G2	11	4904719	4950663	159	23	10164	1.9977	0.022873
+	UGGT1	2	128056180	128205677	430	15	10164	1.9964	0.022944
+	IRF8	16	85864168	85932606	323	46	10164	1.9955	0.022996
+	BCKDHA	19	41362789	41435005	140	5	10164	1.9935	0.023103
+	LOC1053750	6	36643946	36691307	237	25	10164	1.9931	0.023127
-	IKZF2	2	212989684	213189266	630	41	10164	1.9929	0.023136
+	LOC1053750	6	29073630	29124417	230	36	10164	1.9921	0.023179
-	NPC1	18	23496184	23621617	286	34	10164	1.9913	0.023224
+	NEFM	8	24878761	24929093	77	9	10164	1.9907	0.023256
+	OR2J3	6	29076810	29122884	216	36	10164	1.9895	0.023321
+	ARHGAP15	2	143035968	143778352	1646	129	10164	1.9883	0.023387
-	JPH2	20	44101697	44222578	435	51	10164	1.986	0.023515
+	CDKN1A	6	36641460	36697339	259	29	10164	1.9834	0.023664
-	FAM69C	18	74425728	74499715	172	30	10164	1.9825	0.023713
-	SERPINA2	14	94354318	94401702	201	27	10164	1.9822	0.023728
-	HLA-B	6	31343868	31392212	1724	57	10164	1.9814	0.023774
+	ESR1	6	151655496	152113274	2021	126	10164	1.9811	0.023791
+	GNRH2	20	3004061	3055860	156	21	10164	1.9805	0.023821
+	CD22	19	35294166	35357361	230	50	10164	1.9794	0.023888
-	PRB2	12	11381540	11430564	66	12	10164	1.9789	0.023916
+	PVR	19	44608798	44676161	129	13	10164	1.9737	0.024206
+	CATSPER3	5	134932906	135021707	204	26	10164	1.973	0.024247
+	TPSD1	16	1221272	1268493	58	7	10164	1.9718	0.024318
+	FOXG1	14	28732072	28780277	140	19	10164	1.9715	0.024335
-	EXOSC5	19	41376370	41432351	110	4	10164	1.9709	0.02437
+	TMEM91	19	41328966	41394083	101	10	10164	1.9701	0.024412
-	ABCB4	7	87392044	87512523	312	23	10164	1.9672	0.02458
-	B4GALT1	9	33100637	33202358	272	28	10164	1.9668	0.024602
+	SIGLECL1	19	51216026	51279330	184	27	10164	1.9658	0.02466
-	PRDX3	10	119157699	119213865	166	15	10164	1.9653	0.024689
+	COASY	17	42527074	42576281	87	10	10164	1.9624	0.024857
+	AGRN	1	985101	1066119	60	3	10164	1.962	0.024884
-	IL12B	5	159304783	159365473	135	23	10164	1.9604	0.024973
+	FEM1A	19	4756716	4805559	182	21	10164	1.9578	0.025127
+	TICRR	15	89540438	89638023	252	10	10164	1.9577	0.025134
+	H2AFY2	10	70017601	70122284	289	34	10164	1.9574	0.025148
+	KCNIP1	5	170318487	170746632	1179	131	10164	1.9572	0.025164
+	ACTL10	20	33631498	33678525	76	18	10164	1.9571	0.025169
-	ERO1L	14	52629915	52730931	254	40	10164	1.9552	0.025277
-	ADGRE3	19	14609117	14709918	308	23	10164	1.9545	0.025321
+	SESN2	1	28224452	28292491	157	10	10164	1.9545	0.025322
-	DEFA5	8	7045300	7091739	126	16	10164	1.9534	0.025387
-	KCNK9	8	139590838	139738068	334	40	10164	1.9527	0.025429
+	BAIAP2	17	81000144	81127432	447	58	10164	1.9504	0.025567

STRAND	SYMBOL	CHR	START	STOP	NSNPS	NPARAM	N	ZSTAT	P
-	ORC4	2	147920397	148056604	291	24	10164	1.9497	0.025603
+	BIRC2	11	102312182	102388670	168	16	10164	1.9496	0.025613
+	GTF2H1	11	18287269	18377043	187	17	10164	1.9479	0.025713
+	GRK5	10	119172685	119465619	942	75	10164	1.9478	0.025719
-	KIAA0226L	13	46332000	46425042	210	16	10164	1.9434	0.025983
-	DNAJB3	2	233732750	233779015	159	22	10164	1.9421	0.026062
+	TSLP	5	111035080	111088024	137	36	10164	1.9414	0.026108
-	CCL27	9	34651883	34697692	130	17	10164	1.9413	0.026111
+	SLC29A4	7	5239374	5314073	277	45	10164	1.9413	0.026113
+	ARHGEF40	14	21035226	21100248	82	21	10164	1.9412	0.026118
+	MLX	17	42532060	42583203	87	10	10164	1.9384	0.026285
+	TMEM183A	1	202972383	203034848	179	29	10164	1.9383	0.026291
-	PLA2G4D	15	42057565	42129559	284	40	10164	1.9365	0.026401
+	RIOK1	6	7354419	7428037	171	10	10164	1.9352	0.026483
+	C6orf136	6	30612039	30663210	232	47	10164	1.9332	0.026606
+	CA14	1	150222159	150275078	78	11	10164	1.9312	0.026732
+	FLYWCH2	16	2847804	2909382	175	11	10164	1.9305	0.02677
-	CNKSR3	6	154395299	154545619	512	68	10164	1.9304	0.026781
-	ATP2A3	17	3913869	3999464	154	23	10164	1.9285	0.026896
-	CAPN12	19	38720192	38779474	289	27	10164	1.9259	0.02706
-	PPP1R10	6	30590400	30653607	329	41	10164	1.9237	0.027197
+	SCARA3	8	27598894	27722743	430	34	10164	1.9225	0.02727
+	IQCF2	3	51826629	51873424	125	9	10164	1.9224	0.027278
+	ALDH5A1	6	24459969	24547207	499	40	10164	1.9224	0.02728
-	LPAR1	9	110863261	111074243	542	45	10164	1.9223	0.027282
+	HSD17B1	17	42516943	42565214	78	11	10164	1.9217	0.027322
-	LANCL1	2	210421249	210512618	265	22	10164	1.9211	0.02736
-	LOC1053746	5	6329488	6404751	157	14	10164	1.9207	0.027385
-	CORIN	4	47583998	47873106	741	39	10164	1.9199	0.027438
+	UGT1A5	2	233677992	233783299	415	25	10164	1.9195	0.027463
+	UGT1A3	2	233694127	233783299	335	24	10164	1.9157	0.027703
-	TGFB1	19	41320531	41388933	118	14	10164	1.9154	0.027719
-	PLXNA2	1	208012242	208279384	936	84	10164	1.9153	0.027729
+	STON1-GTF2	2	48494925	48786517	841	74	10164	1.9151	0.027739
-	APTX	9	32962606	33060138	396	15	10164	1.9142	0.0278
+	ACVR2A	2	147809517	147940827	275	27	10164	1.914	0.02781
-	CAGE1	6	7316654	7424709	286	23	10164	1.9137	0.02783
-	LOC1027249	2	45638733	45685986	152	27	10164	1.9131	0.027868
+	UGT1A4	2	233683792	233783299	382	24	10164	1.913	0.027871
-	MTUS1	8	17633794	17836220	1101	78	10164	1.9114	0.027979
+	RASAL2	1	178058729	178489514	736	27	10164	1.9109	0.028009
+	CEACAM19	19	44631592	44694359	109	13	10164	1.9097	0.028087
+	C1orf54	1	150234004	150290924	85	12	10164	1.909	0.028129
-	TRPM1	15	30991061	31196273	510	37	10164	1.9089	0.028136
+	LOC729254	2	191757840	191830249	113	6	10164	1.9086	0.028156
-	MOB2	11	1459448	1521779	188	15	10164	1.9073	0.02824
-	SRPK1	6	35823034	35956180	395	26	10164	1.9071	0.028252
-	ANP32E	1	150208417	150271156	80	9	10164	1.9068	0.028276
+	MED15	22	20472542	20597632	66	4	10164	1.9055	0.028356
-	KRTAP5-1	11	1574342	1620283	200	22	10164	1.9033	0.028501
-	ACSBG1	15	78160845	78269707	414	54	10164	1.9021	0.028579
-	BTG4	11	111374095	111549733	422	55	10164	1.9016	0.028613
+	PRKCI	3	170187432	170315982	561	34	10164	1.9011	0.028642
-	ATL3	11	63614082	63706974	51	19	10164	1.9011	0.028644
-	DCDC2	6	24161755	24418292	820	52	10164	1.9009	0.028655
+	C11orf63	11	122846639	122969722	388	45	10164	1.9007	0.028671
-	LOC1053705	14	77022368	77069940	153	26	10164	1.9001	0.028709
+	MICA	6	31364784	31425315	1460	38	10164	1.8998	0.028728
+	SETBP1	18	44645173	45078510	940	107	10164	1.899	0.028782
-	GPLD1	6	24415832	24530043	751	45	10164	1.8986	0.028808
+	DNAJA1	9	32990211	33049064	285	13	10164	1.8985	0.028815
-	IFI27L2	14	94117781	94164620	143	23	10164	1.8975	0.028879
+	SLC2A8	9	127362138	127417898	193	28	10164	1.8967	0.028934

STRAND	SYMBOL	CHR	START	STOP	NSNPS	NPARAM	N	ZSTAT	P
+	NELFCD	20	58946208	59005134	59	10	10164	1.8961	0.028973
-	TUBA3C	13	19163770	19216852	282	19	10164	1.8953	0.029027
-	SSBP3	1	54215431	54448485	703	76	10164	1.8925	0.029211
-	DKK3	11	11952996	12044370	337	61	10164	1.8921	0.029239
+	OR2AP1	12	55539415	55585344	207	12	10164	1.8905	0.029347
+	SDHAF3	7	97081593	97191763	36	4	10164	1.8898	0.029393
+	TUBG2	17	42624248	42677006	93	9	10164	1.8893	0.029423
+	B3GNT5	3	183218226	183283391	85	10	10164	1.8869	0.029589
-	COIL	17	56928199	56996050	96	9	10164	1.8863	0.029628
+	LRCH3	3	197756226	197894310	221	16	10164	1.8857	0.02967
+	ASH2L	8	38069965	38150080	114	11	10164	1.8849	0.029719
-	BIN1	2	127038023	127142400	415	42	10164	1.8845	0.029751
-	RSRP1	1	25232249	25302915	5	3	10164	1.8821	0.029912
-	LDHD	16	75101860	75151772	203	27	10164	1.882	0.029915
+	RPP38	10	15062180	15114257	187	22	10164	1.8801	0.030049
+	CCDC136	7	128756410	128832133	185	18	10164	1.8787	0.03014
+	ZNF808	19	52492652	52580178	204	9	10164	1.8786	0.030153
-	TMEM156	4	38956016	39067669	356	31	10164	1.8779	0.030194
+	MANEA	6	95542497	95619452	236	16	10164	1.8767	0.030276
-	CEP19	3	196696273	196747294	149	12	10164	1.8756	0.030354
-	RAB27A	15	55192966	55324828	424	29	10164	1.8755	0.030364
+	ERICH4	19	41408158	41454765	90	6	10164	1.8754	0.030368
+	CTU2	16	88671483	88725386	211	37	10164	1.8747	0.030419
+	DISC1	1	231591815	232051272	1166	130	10164	1.8728	0.030546
-	C2orf71	2	29051373	29109838	197	25	10164	1.8722	0.030591
-	RNF145	5	159147409	159245053	203	25	10164	1.8715	0.030641
+	FAN1	15	30868852	30953108	110	7	10164	1.8709	0.030679
+	ATG101	12	52034974	52087495	62	7	10164	1.8679	0.030886
+	OXT	20	3033871	3082517	139	28	10164	1.8674	0.030919
+	USP3	15	63469511	63604640	385	26	10164	1.8656	0.031045
-	GPR84	12	54340784	54400253	99	20	10164	1.8656	0.031048
-	CLU	8	27586917	27650031	227	34	10164	1.8655	0.031053
-	SCTR	2	119429843	119559452	332	32	10164	1.8654	0.031064
-	SYF2	1	25212276	25267522	7	4	10164	1.8654	0.031065
+	CYP46A1	14	99649262	99737321	35	10	10164	1.8618	0.031313
-	LRRC4	7	128017071	128067107	94	21	10164	1.8613	0.031352
-	MTMR10	15	30909764	31026604	219	10	10164	1.8611	0.031367
+	ATAT1	6	30591836	30656823	328	41	10164	1.86	0.031444
+	OR6C68	12	55457378	55503316	205	13	10164	1.86	0.031446
+	NFKB1	4	102466329	102627302	330	18	10164	1.8598	0.031456
-	PIEZO1	16	88705338	88819964	608	61	10164	1.8597	0.031462
-	TRIM27	6	28893002	28958991	361	38	10164	1.8594	0.031485
+	FAM212A	3	49768233	49815033	90	15	10164	1.8582	0.031567
+	HEPHL1	11	93986212	94124208	377	33	10164	1.858	0.031587
-	HS3ST6	16	1901463	1956422	222	29	10164	1.8563	0.031703
-	OR8D1	11	124299840	124345766	170	16	10164	1.8556	0.031759
+	PFKP	10	3032474	3146805	415	44	10164	1.8554	0.031768
-	IGFBP3	7	45902245	45956272	148	20	10164	1.8549	0.031804
+	DNAJA4	15	78229145	78292196	233	29	10164	1.8531	0.031933
-	ZNF585A	19	37137438	37207741	172	25	10164	1.8526	0.031971
-	SLC44A5	1	75192131	75759322	1493	47	10164	1.8526	0.031973
-	HES4	1	988962	1036285	47	3	10164	1.8518	0.03203
+	OR8G2	11	124189639	124235663	251	18	10164	1.8515	0.032049
+	LDLRAD3	11	35908981	36242136	1254	127	10164	1.8514	0.032053
-	PSMC3IP	17	42562310	42612831	86	7	10164	1.8512	0.032073
-	TUBGCP3	13	112475005	112641442	243	11	10164	1.8511	0.032074
+	TRIM60	4	164990762	165051818	220	22	10164	1.8486	0.032256
+	NLRC5	16	56954547	57093526	485	94	10164	1.8478	0.032319
-	CSTB	21	43763665	43811375	194	18	10164	1.8474	0.032342
+	SOX4	6	21558741	21608619	62	7	10164	1.847	0.032373
-	SNRPB	20	2451635	2505853	105	16	10164	1.8466	0.032401
+	ADAMTS14	10	70637539	70772441	655	54	10164	1.8453	0.032497
+	OPTC	1	203459143	203518949	168	20	10164	1.8449	0.032525

STRAND	SYMBOL	CHR	START	STOP	NSNPS	NPARAM	N	ZSTAT	P
-	SLC46A2	9	112868920	112925942	243	32	10164	1.8445	0.032559
+	FAT4	4	125280897	125502932	726	55	10164	1.8428	0.032682
+	UGT1A1	2	233725273	233783299	210	25	10164	1.8424	0.032709
+	BASP1	5	17181823	17286845	307	44	10164	1.8417	0.032757
+	GADD45A	1	67650177	67698338	149	20	10164	1.8405	0.032849
-	DLX5	7	97010390	97059831	11	4	10164	1.8401	0.032877
+	CRIP2	14	105437938	105490170	31	6	10164	1.8399	0.032889
+	BNIP3L	8	26348007	26423129	140	22	10164	1.8368	0.033117
+	ZNF79	9	127389374	127456964	219	21	10164	1.8368	0.033118
-	EXOC7	17	76070993	76138787	144	14	10164	1.8363	0.033156
-	PANK1	10	89569497	89680572	455	35	10164	1.8362	0.033163
-	EPHA2	1	16114337	16191087	172	24	10164	1.8358	0.033193
-	HMGCLL1	6	55424373	55714668	1074	48	10164	1.8348	0.033271
-	B3GNT8	19	41415359	41463856	89	7	10164	1.8347	0.033275
-	GNG8	19	46623934	46670534	84	11	10164	1.8344	0.0333
-	EIF2B3	1	44840522	45021722	556	21	10164	1.8342	0.03331
+	C5orf66	5	134998280	135354680	534	57	10164	1.8328	0.033417
+	FAM19A1	3	67956592	68555621	1850	86	10164	1.8328	0.033418
+	GPC5	13	91363619	92877237	3295	144	10164	1.8305	0.033587
-	RSU1	10	16580616	16852454	700	61	10164	1.8296	0.033657
+	SSC5D	19	55453497	55529099	311	35	10164	1.828	0.033775
+	C15orf53	15	38661598	38710038	146	27	10164	1.8268	0.033868
-	LDOC1L	22	44482570	44533125	189	12	10164	1.8248	0.034015
+	PCSK5	9	75855601	76372339	1040	109	10164	1.8241	0.034065
+	NOS3	7	150956056	151024599	152	27	10164	1.8234	0.034124
-	OSBPL11	3	125518858	125630537	387	22	10164	1.8214	0.03427
-	DSTYK	1	205132503	205246599	326	17	10164	1.8194	0.034427
-	ERCC8	5	60863832	60980078	244	18	10164	1.8172	0.034593
+	FAM46C	1	117570982	117638389	259	33	10164	1.8171	0.034599
-	RPL12	9	127437674	127486432	170	16	10164	1.8161	0.034674
-	RNF113B	13	98165785	98212267	23	9	10164	1.8158	0.034702
-	GRIN3A	9	101559352	101773580	755	36	10164	1.8157	0.034706
+	UTP20	12	101245127	101396619	359	20	10164	1.8117	0.035014
+	UGT1A7	2	233646938	233783299	483	28	10164	1.811	0.035068
-	GABRA4	4	46908900	47029407	404	32	10164	1.8092	0.035209
-	CLSTN1	1	9719021	9859526	369	18	10164	1.8082	0.035287
+	WDR19	4	39147189	39295810	314	13	10164	1.8082	0.03529
+	LRRC28	15	99216362	99396309	416	20	10164	1.8068	0.035398
+	ATP1B1	1	169071709	169142722	222	34	10164	1.8016	0.035807
+	LRSAM1	9	127416486	127513501	326	20	10164	1.8016	0.035807
-	GOLGA3	12	132758909	132864118	354	22	10164	1.8008	0.035865
+	FLRT1	11	64000970	64129183	339	64	10164	1.8001	0.035921
-	FAM134C	17	42569508	42644427	132	9	10164	1.7992	0.035991
-	RORA	15	60478284	61264303	2058	232	10164	1.7987	0.03603
+	STRIP2	7	129399433	129498399	164	15	10164	1.7984	0.036054
-	EZH1	17	42690275	42780078	141	17	10164	1.7981	0.036077
+	CYP27A1	2	218746749	218825293	179	7	10164	1.798	0.036091
-	CLPS	6	35784982	35832344	213	31	10164	1.797	0.036165
-	CANT1	17	78981716	79044817	209	30	10164	1.796	0.036245
-	BLID	11	122105354	122151215	108	14	10164	1.7948	0.036344
+	TUBG1	17	42574340	42625238	79	6	10164	1.7941	0.036399
+	NNAT	20	37486205	37533690	93	13	10164	1.794	0.036408
+	ARHGEF1	19	41848173	41917454	108	10	10164	1.7937	0.036428
-	MACROD1	11	63988556	64201130	585	70	10164	1.7935	0.036447
+	LOC1053748	6	562631	612734	219	25	10164	1.7933	0.036466
+	UGT1A9	2	233636898	233783305	503	31	10164	1.7924	0.036534
-	OR56A1	11	6016671	6062741	105	15	10164	1.7922	0.036554
+	UGT1A6	2	233656675	233783305	468	26	10164	1.7917	0.03659
+	AADACL4	1	12609547	12677086	96	25	10164	1.7911	0.03664
+	OR6C76	12	55391254	55437192	129	10	10164	1.7905	0.036688
+	C5orf58	5	170197552	170256231	160	25	10164	1.7903	0.0367
-	SNAI3	16	88667682	88721474	213	38	10164	1.7902	0.036707
-	TMF1	3	69009827	69087333	164	17	10164	1.7898	0.036743

STRAND	SYMBOL	CHR	START	STOP	NSNPS	NPARAM	N	ZSTAT	P
+	EIF1B	3	40274682	40322424	105	8	10164	1.7889	0.036819
+	ISG15	1	978467	1024540	34	2	10164	1.7877	0.036916
+	TMCC2	1	205192910	205283343	263	24	10164	1.7876	0.036921
+	OPTN	10	13065075	13148291	262	36	10164	1.7864	0.037017
-	AK3	9	4699556	4777043	400	48	10164	1.7856	0.037082
-	IRF2BPL	14	77014543	77063699	171	28	10164	1.7851	0.037124
+	C11orf70	11	102012411	102094560	170	17	10164	1.7846	0.037163
-	PTPN3	9	109365694	109573353	633	33	10164	1.7839	0.037222
-	TMEM123	11	102386325	102488044	198	32	10164	1.7835	0.037253
+	FAM32A	19	16150184	16202046	5	1	10164	1.7796	0.037573
-	CIB3	19	16151368	16208525	5	1	10164	1.7796	0.037573
+	RAB44	6	36662827	36743183	371	39	10164	1.7795	0.037575
-	NRXN1	2	49908505	51067536	3794	155	10164	1.7795	0.037581
+	KIRREL2	19	35816635	35877146	107	22	10164	1.7782	0.037684
-	ASXL2	2	25723753	25913474	320	11	10164	1.7757	0.037895
+	MTA1	14	105384849	105480729	49	10	10164	1.7756	0.037903
+	YTHDF3	8	63133553	63222788	247	19	10164	1.7742	0.038016
-	LOC1019287	5	177098798	177156834	107	12	10164	1.7696	0.038394
+	MRPS18B	6	30582320	30636397	279	38	10164	1.7694	0.038413
+	POLR1D	13	27585743	27677422	244	30	10164	1.769	0.038449
-	LOC1053763	10	941571	991386	139	24	10164	1.7659	0.038708
+	PLEKHG7	12	92701489	92782745	150	13	10164	1.7658	0.038717
+	MAN2A1	5	109654366	109879625	550	18	10164	1.7651	0.038774
+	MTO1	6	73426731	73511456	229	29	10164	1.7637	0.03889
-	ZDHHC11B	5	700355	819749	327	29	10164	1.7637	0.038895
+	LOC1053722	19	8595645	8687993	86	11	10164	1.7633	0.038921
+	JDP2	14	75392806	75482701	229	38	10164	1.7623	0.039011
+	ZNF701	19	52535273	52597174	116	10	10164	1.762	0.039033
-	CCDC112	5	115257188	115331761	166	30	10164	1.7619	0.039047
+	PRMT2	21	46600153	46675685	259	34	10164	1.7612	0.039102
-	AVEN	15	33856227	34109458	708	41	10164	1.7599	0.039212
-	GATA2	3	128469422	128528187	206	36	10164	1.7581	0.039366
+	RRP1	21	43754535	43814102	236	23	10164	1.7576	0.039404
-	KDR	4	55068259	55160595	229	27	10164	1.7538	0.039734
+	ADAM22	7	87899157	88212889	610	36	10164	1.7534	0.039767
-	C10orf128	10	49144724	49223585	212	23	10164	1.7528	0.039819
-	HTR1B	6	77452231	77498403	153	24	10164	1.7522	0.039873
+	INHBB	2	120311143	120361807	107	27	10164	1.7495	0.040098
+	CCDC132	7	93197340	93371123	233	30	10164	1.7488	0.040164
-	MED10	5	6361926	6413526	130	11	10164	1.7482	0.040217
+	WNT8A	5	138048892	138106730	91	7	10164	1.7458	0.040427
-	CMTM6	3	32471312	32537911	163	24	10164	1.7456	0.040441
+	SPN	16	29627950	29680507	2	1	10164	1.744	0.040579
+	PAM	5	102720276	103041105	828	29	10164	1.744	0.04058
+	CACNG8	19	53928036	54000215	1	1	10164	1.7431	0.040658
+	PLEKHG6	12	6275436	6338506	219	30	10164	1.7431	0.040661
-	PPP2R2B	5	146579504	147116520	1253	80	10164	1.7425	0.040712
+	RNF20	9	101498849	101573344	182	19	10164	1.742	0.040752
-	LOC1019275	15	30529463	30660778	23	2	10164	1.7408	0.040858
-	PTGIR	19	46600040	46660118	112	12	10164	1.7396	0.040966
-	CSGALNACT	8	19394161	19792271	1478	83	10164	1.7389	0.041024
+	DDX18	2	117779679	117842377	133	14	10164	1.7389	0.041028
-	LNX2	13	27535911	27655583	264	24	10164	1.7379	0.041117
+	SMIM4	3	52500986	52550570	132	20	10164	1.737	0.04119
+	CDH6	5	31158655	31339146	472	55	10164	1.7368	0.041213
+	SOX2	3	181676924	181724436	42	10	10164	1.7363	0.041257
+	LOC1053785	10	85113131	85166883	223	15	10164	1.7357	0.041312
+	APLP2	11	130034821	130154811	351	20	10164	1.7344	0.041423
+	GMCL1	2	69794642	69891386	316	23	10164	1.7342	0.041442
+	WWC1	5	168257060	168482303	648	78	10164	1.7342	0.041444
-	TRIM61	4	164944446	165012666	230	14	10164	1.7336	0.041491
-	PIK3C2B	1	204412628	204529815	295	19	10164	1.7335	0.041505
-	FGF1	5	142582178	142733070	288	47	10164	1.7334	0.041509

STRAND	SYMBOL	CHR	START	STOP	NSNPS	NPARAM	N	ZSTAT	P
+	MSI2	17	57221570	57710477	1021	136	10164	1.7327	0.041575
+	AQP8	16	25181916	25238932	112	18	10164	1.7327	0.041578
+	GOLGA8H	15	30510870	30627827	6	1	10164	1.7309	0.041733
-	DSCAML1	11	117417772	117832261	1574	150	10164	1.7305	0.041767
+	GPR26	10	123631355	123707399	316	36	10164	1.7301	0.041802
-	OR51A2	11	4944772	4990713	127	18	10164	1.7297	0.041839
+	NANS	9	98021677	98093083	148	18	10164	1.7288	0.041924
-	TM4SF20	2	227352156	227414306	346	39	10164	1.7273	0.042054
-	TRG	7	38230024	38403055	244	21	10164	1.7273	0.042059
+	CPT1C	19	49655878	49723731	148	25	10164	1.7272	0.042062
+	PIGX	3	196677374	196746007	154	12	10164	1.7254	0.042223
-	TFCP2	12	51083756	51208143	357	23	10164	1.7253	0.042235
+	APOBEC3G	22	39042005	39097743	132	16	10164	1.7248	0.042282
-	ZNF256	19	57930833	57982747	106	11	10164	1.7239	0.042365
-	PGPEP1L	15	98958230	99042795	315	27	10164	1.7222	0.042515
-	IQCF5	3	51863721	51910584	102	16	10164	1.7222	0.042516
+	ACTR5	20	38713258	38782446	20	6	10164	1.722	0.042535
-	GPHB5	14	63302835	63353879	208	31	10164	1.7212	0.042606
+	NDUFAF2	5	60910129	61163037	523	21	10164	1.721	0.042625
+	LBP	20	38311411	38387011	316	42	10164	1.721	0.04263
+	FAM231A	1	16638003	16683512	4	1	10164	1.7208	0.042646
+	MRRF	9	122229603	122335297	114	17	10164	1.7203	0.042686
+	FAS	10	88934801	89027059	300	26	10164	1.7202	0.042697
-	ZNF20	19	12121350	12175407	81	8	10164	1.72	0.042719
+	RAB18	10	27469174	27552237	254	23	10164	1.7191	0.042798
-	CDHR4	3	49780732	49838164	93	14	10164	1.717	0.04299
-	LINS	15	100559223	100637240	304	24	10164	1.716	0.043079
-	TICAM1	19	4805924	4866742	216	29	10164	1.716	0.043079
+	ZNF587	19	57814813	57875123	150	15	10164	1.7148	0.043189
+	LOC1001321	3	46577435	46636543	314	28	10164	1.7143	0.043237
-	OR8D2	11	124309262	124355197	188	17	10164	1.7138	0.043283
-	NOTCH1	9	136484444	136580786	128	17	10164	1.7132	0.043341
+	ZNRF4	19	5420415	5466856	107	24	10164	1.713	0.043358
+	LIPF	10	88629389	88688815	162	23	10164	1.7112	0.043519
+	MTAP	9	21767636	21875971	464	33	10164	1.7108	0.043556
-	RRP7A	22	42498335	42554823	141	20	10164	1.7092	0.043708
-	POLE3	9	113397235	113445749	81	6	10164	1.7056	0.04404
+	TERF1	8	72973862	73058123	202	20	10164	1.7048	0.044116
+	ADM5	19	49653685	49700990	84	18	10164	1.7035	0.044235
+	ARHGAP11B	15	30591131	30648810	18	2	10164	1.7016	0.044415
+	BPI	20	38269150	38347505	250	45	10164	1.7008	0.044491
-	CYHR1	8	144439582	144500691	115	10	10164	1.7004	0.044528
-	SLC38A11	2	164887745	164990560	345	31	10164	1.7001	0.044559
-	NPC2	14	74469940	74528381	155	17	10164	1.6985	0.044703
-	CEP295NL	17	78880579	78938217	354	37	10164	1.6983	0.044726
+	SLC4A3	2	219592570	219651980	5	3	10164	1.6982	0.044739
+	LYZL1	10	29254051	29328328	316	27	10164	1.6946	0.045078
-	PHF21B	22	44858432	45044928	672	56	10164	1.6917	0.045356
-	OR2W1	6	29034213	29080175	231	34	10164	1.6916	0.045357
+	SARS	1	109178893	109248182	139	15	10164	1.6916	0.04536
-	NRIP2	12	2814882	2870536	111	11	10164	1.6915	0.045371
+	TMEM255B	13	113724210	113823797	318	20	10164	1.6912	0.045401
+	CBFA2T2	20	33455122	33660031	321	32	10164	1.6904	0.045477
+	HOGA1	10	97549345	97622802	186	27	10164	1.6894	0.045568
+	GLP2R	17	9790543	9902104	82	2	10164	1.689	0.045608
-	CBR4	4	168884623	169045317	199	11	10164	1.689	0.045608
+	A2ML1	12	8787554	8896918	283	46	10164	1.6882	0.045685
-	PSMD5	9	120804779	120878021	122	14	10164	1.6878	0.04572
+	MYF6	12	80672629	80719478	92	4	10164	1.6874	0.045767
+	PITHD1	1	23743386	23798232	30	9	10164	1.6871	0.04579
+	SMKR1	7	129467479	129522932	121	17	10164	1.6869	0.045812
+	MAST2	1	45751992	46046124	834	24	10164	1.6866	0.045842
-	LRRD1	7	92134884	92200276	140	19	10164	1.6863	0.045866

STRAND	SYMBOL	CHR	START	STOP	NSNPS	NPARAM	N	ZSTAT	P
-	ZNF311	6	28984785	29040628	272	44	10164	1.686	0.045894
-	CHFR	12	132830352	132922618	355	20	10164	1.684	0.046092
+	GJA4	1	34757958	34805750	31	13	10164	1.6827	0.046216
+	TBRG1	11	124587599	124645926	121	18	10164	1.6825	0.046237
+	PRMT1	19	49642152	49698450	94	18	10164	1.6824	0.046241
+	UROD	1	44977133	45025669	163	13	10164	1.6823	0.04626
-	ZNF585B	19	37171579	37245549	171	26	10164	1.6821	0.046275
+	PCK2	14	24059131	24114132	77	20	10164	1.681	0.046383
-	EEF2	19	3966056	4020463	61	6	10164	1.68	0.046478
-	ATOH7	10	68220595	68267113	122	10	10164	1.6789	0.046583
-	PLAT	8	42164718	42242676	166	29	10164	1.6788	0.046594
-	TEC	4	48125783	48304797	661	37	10164	1.6786	0.046618
-	CHN1	2	174789314	175040379	327	14	10164	1.6777	0.046705
-	IL21	4	122602628	122656057	137	21	10164	1.6768	0.046788
+	GPR37L1	1	202083705	202141425	200	27	10164	1.6768	0.046796
+	LARP1B	4	128026266	128232931	443	16	10164	1.6763	0.04684
-	HIP1	7	75523298	75773972	622	48	10164	1.6757	0.0469
+	ADCY7	16	50209749	50328135	184	31	10164	1.6756	0.046907
-	DAZL	3	16576792	16640499	301	30	10164	1.675	0.046972
-	C8orf37	8	95234913	95305337	246	20	10164	1.674	0.047066
-	BEGAIN	14	100527147	100622404	303	31	10164	1.6736	0.047108
-	PHF19	9	120845651	120929896	229	22	10164	1.6728	0.047179
-	ZCCHC11	1	52413275	52588463	322	18	10164	1.6726	0.047205
-	KCNV1	8	109953636	110010734	59	5	10164	1.6704	0.047419
-	PARP9	3	122517910	122599676	289	34	10164	1.6697	0.047491
-	BRD7	16	50309018	50403966	201	27	10164	1.6692	0.047543
-	GGH	8	63005079	63074445	260	24	10164	1.6682	0.047642
-	GUCY1A2	11	106664012	107053445	1044	63	10164	1.6664	0.047817
+	OR6C65	12	55365529	55411467	153	11	10164	1.6664	0.047819
+	IQCF3	3	51791883	51840858	88	9	10164	1.6658	0.047872
+	PRR20E	13	57132197	57180218	52	16	10164	1.6653	0.047923
+	ADORA2B	17	15892783	15985896	159	14	10164	1.665	0.047953
+	MMP23B	1	1597181	1644654	1	1	10164	1.665	0.047955
+	MIB2	1	1579975	1640610	1	1	10164	1.665	0.047955
-	C1orf233	1	1588008	1635096	1	1	10164	1.665	0.047955
-	GLI3	7	41950949	42299112	825	86	10164	1.6642	0.04804
+	ZNF548	19	57354850	57412551	106	13	10164	1.6638	0.048076
+	C4orf29	4	127930261	128046326	171	13	10164	1.6638	0.04808
-	SBK2	19	55519336	55572174	157	30	10164	1.6635	0.048109
-	ESCO1	18	21519281	21635736	123	10	10164	1.6629	0.048162
-	CARD17	11	105082469	105136465	139	12	10164	1.6628	0.04818
-	DDX56	7	44555417	44609538	149	16	10164	1.6626	0.048197
+	BTD	3	15566352	15657642	256	33	10164	1.6624	0.048216
+	RTN4RL2	11	57425537	57487539	144	26	10164	1.6623	0.048223
+	C10orf142	10	44257750	44304649	179	18	10164	1.6619	0.048265
+	COPZ1	12	54290090	54361851	112	15	10164	1.6616	0.048298
-	KCNC3	19	50301942	50368515	129	22	10164	1.6615	0.048309
+	LOC1053784	10	89754148	89814646	220	15	10164	1.6613	0.048325
+	LTC4S	5	179758985	179806512	81	13	10164	1.6587	0.048585
-	ZCCHC8	12	122461599	122536073	102	9	10164	1.6568	0.04878
+	YBEY	21	46251310	46323225	312	22	10164	1.6562	0.048837
+	LOC1053709	15	31356503	31414730	107	13	10164	1.656	0.048858
-	SLC4A7	3	27362721	27519420	545	32	10164	1.6557	0.048896
+	STON1	2	48495169	48608515	306	35	10164	1.6552	0.048944
+	IGSF23	19	44578590	44646781	172	22	10164	1.6552	0.048945
+	GPX7	1	52567371	52619051	111	11	10164	1.655	0.048966
+	NARF	17	82423184	82498267	156	12	10164	1.6529	0.049176
-	HSPA5	9	125224848	125276387	119	16	10164	1.6521	0.04926
+	LOC1053707	15	30764309	30810017	107	5	10164	1.6511	0.049363
+	PPP1R15A	19	48837392	48886062	140	15	10164	1.6508	0.049389
+	CUL9	6	43147174	43234587	190	22	10164	1.6505	0.04942
+	RHPN1	8	143333842	143394220	158	18	10164	1.6504	0.049429
-	NXNL1	19	17445425	17495916	109	8	10164	1.6504	0.04943

STRAND	SYMBOL	CHR	START	STOP	NSNPS	NPARAM	N	ZSTAT	P
-	UHRF1BP1L	12	100026794	100177864	268	14	10164	1.6496	0.049513
+	SERPINI1	3	167700644	167835569	390	26	10164	1.6493	0.049547
-	LOC1053792	8	12975151	13073442	637	43	10164	1.6474	0.049733
+	HSPA4L	4	127747209	127844073	125	15	10164	1.6474	0.049738
+	IRF5	7	128902737	128960042	170	30	10164	1.6472	0.049754
-	ACOT12	5	81319991	81429169	290	45	10164	1.6466	0.049818
-	TLE3	15	70037790	70132935	218	43	10164	1.6459	0.049891
-	TAAR2	6	132589627	132659358	397	40	10164	1.6453	0.049952
+	DTNA	18	34458290	34901844	677	49	10164	1.6446	0.050029
+	UGT1A10	2	233601477	233783305	596	34	10164	1.6444	0.050043
-	ARL1	12	101383120	101442820	158	22	10164	1.6443	0.050056
-	HECTD3	1	44992546	45046355	136	15	10164	1.6442	0.05007
-	TP53RK	20	46674365	46724637	58	6	10164	1.6432	0.05017
+	MYF5	12	80681929	80729668	80	5	10164	1.6414	0.050354
+	C14orf80	14	105454855	105509248	31	7	10164	1.6413	0.050366
+	GTF2A1L	2	48582780	48689609	235	22	10164	1.6408	0.050421
+	C17orf112	17	52948991	52997658	132	16	10164	1.6403	0.050472
+	PRELP	1	203440755	203501352	183	17	10164	1.6399	0.050514
-	MFSD8	4	127907805	128000984	130	9	10164	1.6397	0.050536
-	EBNA1BP2	1	43154174	43207570	123	2	10164	1.6374	0.050778
-	DHX36	3	154265668	154359497	139	14	10164	1.6361	0.050912
+	MAPK6	15	51969265	52076265	288	23	10164	1.6353	0.050995
-	KCTD9	8	25417842	25493468	183	8	10164	1.6339	0.051138
+	SNRNP27	2	69858943	69915236	161	25	10164	1.6337	0.051162
-	RGS22	8	99950936	100141116	404	27	10164	1.6331	0.051226
-	LOC1053793	8	21217720	21332077	301	26	10164	1.6316	0.051378
+	CD79A	19	41842120	41891372	66	6	10164	1.6308	0.051468
+	IRG1	13	76913559	76968659	154	27	10164	1.6304	0.051506
-	CYP4F11	19	15902370	15969866	270	18	10164	1.6304	0.051511
+	GRM7	3	6826097	7751531	2822	195	10164	1.6303	0.051521
-	AQP3	9	33431154	33482633	128	23	10164	1.6299	0.051563
-	EOGT	3	68965212	69048961	246	23	10164	1.6295	0.051605
+	C15orf41	15	36544603	36820260	658	54	10164	1.6293	0.051625
+	NAGLU	17	42500933	42554449	82	16	10164	1.6292	0.051637
+	SRF	6	43136295	43191506	132	14	10164	1.6291	0.051646
+	UNC5B	10	71177535	71312879	424	55	10164	1.6291	0.05165
+	PLCG2	16	81744258	81972693	1006	94	10164	1.6282	0.051737
+	MAML1	5	179697850	179787286	147	14	10164	1.6279	0.051775
+	MAP3K1	5	56780073	56906152	365	24	10164	1.6267	0.051896
+	GUCA1A	6	42120386	42190056	78	15	10164	1.626	0.05197
-	IP6K1	3	49714295	49821540	196	19	10164	1.6248	0.052099
+	TMEM121	14	105491031	105540202	58	4	10164	1.6242	0.052162
-	TNFRSF1A	12	6318757	6377117	173	42	10164	1.624	0.052188
+	POLD3	11	74557530	74679341	337	17	10164	1.624	0.052189
-	BARD1	2	214715646	214844711	403	22	10164	1.6227	0.052324
+	LYPLA2	1	23755785	23805539	31	9	10164	1.6211	0.052499
+	RGS1	1	192540727	192590029	111	16	10164	1.6208	0.052526
-	ATP13A3	3	194392674	194521654	80	13	10164	1.6203	0.052589
-	RBM18	9	122229555	122299864	94	13	10164	1.6189	0.052737
+	RGS3	9	113409729	113607743	210	15	10164	1.6186	0.052769
-	USP37	2	218440251	218603366	267	10	10164	1.6171	0.052928
-	IRF3	19	49649569	49700875	91	19	10164	1.6134	0.053334
-	RPS20	8	56058180	56109581	336	11	10164	1.612	0.053486
+	FRY	13	31996300	32307498	776	91	10164	1.6116	0.053521
-	ZNF287	17	16539913	16604206	12	2	10164	1.6115	0.053532
+	TTK	6	79969605	80052527	283	17	10164	1.6108	0.053613
-	PAWR	12	79577791	79726010	157	11	10164	1.6099	0.053705
-	TNRC18	7	5296790	5458546	188	32	10164	1.6089	0.053818
-	BICD2	9	92701363	92799801	236	17	10164	1.6085	0.053868
+	AKIRIN1	1	38956244	39016065	163	17	10164	1.6077	0.053953
+	TMPRSS12	12	50807918	50897884	220	18	10164	1.6051	0.054236
+	ITGB3	17	47218842	47322711	381	26	10164	1.605	0.05425
+	TTLL4	2	218675835	218765416	170	7	10164	1.6049	0.05426

STRAND	SYMBOL	CHR	START	STOP	NSNPS	NPARAM	N	ZSTAT	P
+	TNNT3	11	1884562	1948706	240	44	10164	1.6046	0.054288
+	VIL1	2	218384115	218459525	114	15	10164	1.6045	0.054303
+	CRABP1	15	78305324	78358230	142	35	10164	1.6033	0.054432
+	CDCA2	8	25423574	25521308	222	10	10164	1.6032	0.05445
-	FBXO43	8	100123360	100185569	146	13	10164	1.6028	0.054491
+	LRIT3	4	109813184	109882315	219	21	10164	1.6024	0.054534
+	KIF20A	5	138143728	138197715	51	6	10164	1.6021	0.054571
-	DEFB134	8	11983177	12035752	20	2	10164	1.6017	0.054615
+	SRGN	10	69053072	69114811	198	23	10164	1.6011	0.054682
+	RARRES3	11	63501801	63556458	64	8	10164	1.6005	0.054744
+	FAM71A	1	212589447	212636778	140	24	10164	1.6005	0.054745
+	FBXO7	22	32439682	32508831	257	30	10164	1.6004	0.054755
-	CALN1	7	71769491	72482151	1617	71	10164	1.6003	0.054761
+	TLR3	4	186034155	186095098	225	37	10164	1.6002	0.054779
-	PNMAL2	19	46481191	46530912	139	20	10164	1.5995	0.054855
-	AMT	3	49406778	49457678	104	16	10164	1.5983	0.05499
+	CACNA1H	16	1118241	1231772	138	18	10164	1.5981	0.055008
+	ZNF526	19	42185340	42238201	64	7	10164	1.5981	0.055008
-	RAB38	11	88103247	88210467	363	20	10164	1.5979	0.055028
+	ITM2B	13	48198138	48272096	145	24	10164	1.5979	0.055037
+	FAM45A	10	119069065	119147984	266	21	10164	1.5976	0.055068
+	C1QTNF1	17	78987934	79059788	232	35	10164	1.5974	0.055093
-	ATP5SL	19	41421318	41482827	132	14	10164	1.5949	0.055367
+	MCM10	10	13126554	13221104	264	43	10164	1.5947	0.055386
-	EEF1A1	6	73505750	73556032	139	20	10164	1.5943	0.055431
-	WASF2	1	27394226	27525187	148	7	10164	1.5936	0.055511
-	RHOA	3	49349136	49447093	221	24	10164	1.5929	0.055587
+	NEK11	3	130991850	131360465	887	30	10164	1.5922	0.055675
-	DEDD2	19	42188593	42255152	77	8	10164	1.5914	0.05576
-	LOC1027234	17	36851674	36898150	129	21	10164	1.5911	0.05579
+	TMEM236	10	17717210	17810887	365	34	10164	1.5891	0.056023
-	USH2A	1	215612894	216458396	1860	111	10164	1.589	0.056033
+	KLF13	15	31291855	31445665	226	24	10164	1.5881	0.056131
-	DIP2C	10	264190	724689	1011	42	10164	1.5847	0.056518
-	TESK2	1	45333883	45526342	618	28	10164	1.5841	0.056585
-	DOLK	9	128935530	128982733	61	4	10164	1.5839	0.056605
-	TRAM1	8	70563218	70643459	130	13	10164	1.5833	0.056681
-	ZNF251	8	144710909	144790585	141	10	10164	1.5831	0.056704
-	TMED4	7	44567894	44617295	128	14	10164	1.583	0.056713
+	ZNF283	19	43792292	43858898	281	24	10164	1.5821	0.056815
-	BMP4	14	53939736	53991836	139	28	10164	1.5819	0.056836
+	INSM2	14	35499042	35547054	87	20	10164	1.5817	0.056859
-	TCF20	22	42150013	42318922	351	25	10164	1.5806	0.056988
-	LYPD5	19	43785927	43862199	316	21	10164	1.5805	0.057002
+	EPAS1	2	46262402	46396703	420	71	10164	1.58	0.057055
-	C16orf47	16	73116638	73179447	179	21	10164	1.5798	0.057079
+	RAMP2	17	42726194	42773041	79	12	10164	1.5796	0.0571
+	ANP32B	9	97948207	98025943	141	23	10164	1.5795	0.057111
+	CREB1	2	207494892	207615560	117	16	10164	1.5794	0.057117
-	CDC23	5	138177648	138248343	75	6	10164	1.5792	0.057146
-	ME3	11	86431108	86707636	1201	54	10164	1.5783	0.05725
-	BPIFC	22	32403845	32499469	335	48	10164	1.5776	0.057328
-	EGLN1	1	231353751	231460044	390	17	10164	1.5762	0.057495
+	GABARAPL1	12	10177841	10233128	211	27	10164	1.5757	0.057543
+	MIA3	1	222583102	222678012	202	22	10164	1.5751	0.057622
-	HSD17B14	19	48803017	48871703	111	13	10164	1.5745	0.057681
-	SMU1	9	33031852	33111716	256	27	10164	1.5743	0.057704
-	EMB	5	50386192	50478307	275	17	10164	1.5742	0.057721
+	LCMT1	16	25076726	25188231	183	14	10164	1.5737	0.057775
+	HAUS2	15	42513813	42579994	106	18	10164	1.5735	0.057802
+	GJB3	1	34746189	34796366	34	12	10164	1.5731	0.057853
-	LRRC57	15	42518444	42583804	102	17	10164	1.5719	0.057986
-	STEAP4	7	88266429	88341913	124	25	10164	1.5719	0.057991

STRAND	SYMBOL	CHR	START	STOP	NSNPS	NPARAM	N	ZSTAT	P
+	LOC1053722	19	10002577	10051022	21	5	10164	1.5716	0.058025
-	C12orf43	12	120992422	121051502	237	29	10164	1.5711	0.058074
+	PGLS	19	17476623	17531288	110	13	10164	1.5711	0.058083
-	ZNF385A	12	54359129	54426305	83	20	10164	1.5705	0.05815
-	TNPO3	7	128944180	129090173	324	25	10164	1.5703	0.058168
+	PPP4R4	14	94139312	94289755	392	22	10164	1.5702	0.058179
+	SNRPD1	18	21577269	21640247	80	8	10164	1.5698	0.058235
-	CPSF7	11	61392648	61464992	86	7	10164	1.5695	0.05826
-	DCLRE1A	10	113824724	113889404	81	23	10164	1.5679	0.058447
+	DNTT	10	96269328	96348564	172	10	10164	1.5678	0.05847
+	QPRT	16	29634840	29707994	3	1	10164	1.5672	0.058532
-	ZNF625	19	12134890	12191731	90	8	10164	1.5668	0.058579
-	TIMP2	17	78842977	78960390	566	57	10164	1.5668	0.058584
-	MSANTD4	11	105997902	106057254	127	16	10164	1.5665	0.058613
+	NUP188	9	128912693	129017096	155	11	10164	1.566	0.058673
+	KCNK15	20	44710847	44762313	99	12	10164	1.5657	0.058706
+	SYNGR1	22	39314949	39395588	302	28	10164	1.5634	0.058978
-	ZNF34	8	144762224	144822359	149	11	10164	1.5621	0.059136
-	CLDN7	17	7249903	7298193	99	14	10164	1.5606	0.059309
+	SDHAF2	11	61395125	61456767	76	7	10164	1.5601	0.059366
+	CCDC129	7	31479071	31668720	469	22	10164	1.5596	0.059428
+	KLHL2	4	165172618	165333156	400	26	10164	1.5577	0.059653
-	ZNF768	16	30514001	30561589	12	4	10164	1.5551	0.059963
+	NRG1	8	31604304	32777957	3432	130	10164	1.5548	0.059996
-	PSKH2	8	86037109	86104622	141	13	10164	1.5545	0.060038
+	R3HDM1	2	135496455	135735270	477	32	10164	1.554	0.060088
+	LOC1053733	2	522813	571982	136	16	10164	1.554	0.060089
-	FYCO1	3	45907899	46030824	337	20	10164	1.5533	0.060172
-	NME5	5	138105172	138174443	80	9	10164	1.5529	0.060224
+	MYOT	5	137832856	137897851	52	8	10164	1.5528	0.060239
-	SPRY2	13	80325976	80375951	155	34	10164	1.5527	0.060249
-	C17orf62	17	82432586	82485831	103	8	10164	1.5523	0.060292
+	ABHD12B	14	50837090	50914970	230	31	10164	1.5522	0.060304
+	CNTNAP5	2	123990287	124931201	2652	148	10164	1.5518	0.060349
+	LYRM5	12	25160216	25215015	140	12	10164	1.5512	0.060427
-	NPHS1	19	35815372	35886993	128	25	10164	1.551	0.060447
-	RPL8	8	144779767	144827420	135	11	10164	1.5475	0.060872
-	TRAM2	6	52487402	52612064	467	43	10164	1.5468	0.060958
-	RAB3GAP2	1	220138268	220307501	397	38	10164	1.5466	0.060975
-	COL24A1	1	85719233	86191791	1920	79	10164	1.5465	0.060992
+	RPEL1	10	103210887	103258016	139	32	10164	1.5459	0.061063
-	SPARC	5	151651096	151722054	214	28	10164	1.5458	0.061072
+	SLC6A5	11	20564400	20669285	505	41	10164	1.5446	0.061217
-	UCP1	4	140549394	140603902	70	14	10164	1.5443	0.061258
-	GPANK1	6	31651229	31701283	238	51	10164	1.543	0.061415
-	EXT1	8	117789363	118146819	825	63	10164	1.5423	0.061504
-	GNRH1	8	25409258	25460040	116	7	10164	1.5422	0.061516
-	TRAF1	9	120892393	120964173	209	17	10164	1.5413	0.06162
+	FAM129C	19	17488301	17565545	158	24	10164	1.5409	0.061666
-	STK39	2	167944020	168282595	1095	54	10164	1.5408	0.061688
+	BBS4	15	72651179	72748476	118	17	10164	1.5398	0.06181
+	SLC2A4	17	7246735	7298048	106	15	10164	1.539	0.061902
+	CFAP57	1	43137330	43264358	232	6	10164	1.5376	0.062071
+	ZNF420	19	36972813	37140324	772	20	10164	1.5373	0.062108
-	DHX58	17	42091404	42147733	117	9	10164	1.5372	0.062126
-	CMBL	5	10267595	10343056	177	21	10164	1.5371	0.062134
+	FLNC	7	128795429	128869274	194	19	10164	1.5367	0.062178
-	UBA7	3	49795205	49848958	76	11	10164	1.5362	0.06224
+	LY6G5B	6	31635951	31682450	249	53	10164	1.5361	0.062253
-	ZSWIM5	1	45006399	45241578	526	31	10164	1.5356	0.062319
+	HIC2	22	21382404	21461463	11	2	10164	1.5341	0.0625
+	MYRIP	3	39773914	40270321	1585	82	10164	1.5336	0.062561
-	KIF7	15	89598913	89698074	169	14	10164	1.5326	0.062683

STRAND	SYMBOL	CHR	START	STOP	NSNPS	NPARAM	N	ZSTAT	P
-	CLINT1	5	157775743	157894175	345	19	10164	1.5324	0.062707
-	BLCAP	20	37507417	37562931	149	11	10164	1.5321	0.062746
-	LOC1053777	5	170632233	170716437	182	26	10164	1.5321	0.062746
-	SAP130	2	127931217	128063093	286	24	10164	1.532	0.062757
-	PERM1	1	965199	1017117	18	2	10164	1.532	0.062759
+	TMEM177	2	119644167	119692118	112	10	10164	1.5316	0.062811
+	PLK4	4	127845861	127909224	76	8	10164	1.5316	0.062813
-	NUP160	11	47768113	47883505	198	14	10164	1.5308	0.062915
-	LOC1019299	1	41365004	41415923	183	31	10164	1.5307	0.062924
-	DACT3	19	46637564	46696138	108	20	10164	1.5304	0.062953
-	CST9	20	23592410	23640973	74	8	10164	1.529	0.063129
+	NSD1	5	177098025	177310213	327	16	10164	1.5281	0.063242
-	NPIPA5	16	15353624	15416972	79	11	10164	1.528	0.063256
-	GPX1	3	49347176	49393358	96	16	10164	1.5279	0.06327
-	NICN1	3	49412333	49464324	107	17	10164	1.5278	0.063281
-	NCOA4	10	45995088	46065714	261	32	10164	1.5277	0.063298
+	GINM1	6	149531392	149600931	110	17	10164	1.527	0.063383
-	ELOVL2	6	10970759	11079391	289	28	10164	1.5266	0.063434
+	SH3GL2	9	17543954	17807129	1120	70	10164	1.5263	0.063463
+	FOXF2	6	1354834	1405597	67	16	10164	1.5257	0.063544
-	DBT	1	100176922	100284863	238	11	10164	1.5251	0.063615
+	UGT1A8	2	233582645	233783299	629	34	10164	1.5249	0.063645
+	LDHAL6B	15	59171816	59218588	122	13	10164	1.5244	0.063701
+	DRD5	4	9746634	9794009	116	15	10164	1.5244	0.063708
+	IL13	5	132623173	132671109	120	18	10164	1.5244	0.063708
-	CTDNEP1	17	7233587	7286940	98	16	10164	1.5241	0.06374
-	DHRS3	1	12557910	12653361	293	38	10164	1.5222	0.063976
-	GABARAP	17	7230419	7277434	83	15	10164	1.5219	0.064018
-	NR6A1	9	124507275	124806310	415	26	10164	1.5218	0.064028
-	WFDC8	20	45541152	45614350	439	17	10164	1.5216	0.064056
+	GABRB1	4	46958858	47437343	1293	86	10164	1.5215	0.064063
-	STAT6	12	57085404	57146433	119	20	10164	1.5205	0.064192
+	ZBTB34	9	126825665	126895878	178	20	10164	1.5205	0.064199
-	SLC43A1	11	57474531	57550719	184	29	10164	1.5197	0.064297
+	GOLGA6B	15	72573556	72677397	90	15	10164	1.5188	0.064409
+	GTPBP4	10	953409	1027768	240	27	10164	1.5185	0.064445
+	ZNF383	19	37182926	37253926	177	27	10164	1.5183	0.064464
-	GJA5	1	147746199	147816067	230	35	10164	1.518	0.064503
-	FBXW2	9	120746976	120828462	111	13	10164	1.5175	0.064571
+	NR4A1	12	51987832	52069507	104	19	10164	1.5173	0.064595
+	RNF138	18	32056855	32141561	147	20	10164	1.5172	0.064602
-	UBD	6	29545612	29594925	302	41	10164	1.517	0.064634
-	IGF2BP3	7	23300209	23505527	402	49	10164	1.5157	0.064796
+	LOC1019285	1	169068700	169116473	128	19	10164	1.5142	0.064987
+	TCTA	3	49377206	49426476	117	17	10164	1.5138	0.065042
+	MROH2A	2	233740662	233843423	447	70	10164	1.5134	0.065087
+	TMEM191C	22	21432140	21479940	13	2	10164	1.5133	0.065099
+	SORCS2	4	7157607	7752837	2365	238	10164	1.5131	0.065128
+	CHST4	16	71491120	71548590	114	14	10164	1.5119	0.065282
-	CLIC5	6	45888451	46115348	746	72	10164	1.5117	0.065304
+	LOC1005063	17	296205	343488	25	6	10164	1.5109	0.065402
-	ACTA2	10	88925074	89026390	332	32	10164	1.5101	0.065506
-	TREM1	6	41257385	41321719	182	27	10164	1.5094	0.065603
+	LOC1053787	1	52517934	52575732	137	16	10164	1.5091	0.065634
-	MCF2L2	3	183168004	183463619	718	48	10164	1.5085	0.065708
-	S100A7	1	153447744	153495661	105	7	10164	1.5083	0.06574
+	HTR6	1	19630287	19690966	198	28	10164	1.5083	0.065741
+	NR2C2	3	14912584	15059279	223	31	10164	1.5076	0.065825
+	LRRC55	11	57146747	57201714	142	24	10164	1.5062	0.066009
+	UCHL1	4	41221881	41278429	137	15	10164	1.5056	0.066081
+	FFAR1	19	35316542	35362464	174	40	10164	1.5055	0.066095
-	LRRC38	1	13464977	13548781	383	58	10164	1.505	0.066165
+	GRIK4	11	120476746	120998805	1310	134	10164	1.5049	0.066173

STRAND	SYMBOL	CHR	START	STOP	NSNPS	NPARAM	N	ZSTAT	P
-	BRD8	5	138129766	138213669	80	7	10164	1.5047	0.066199
+	SYCP2L	6	10851831	10984309	550	53	10164	1.5045	0.066232
+	LOC1053700	12	126390958	126444206	172	12	10164	1.5043	0.066256
-	HEPACAM2	7	93178586	93267297	140	20	10164	1.5041	0.066274
-	PABPC4	1	39550813	39611849	128	14	10164	1.5041	0.066283
+	ZNF136	19	12128057	12199249	112	9	10164	1.5037	0.066333
-	ADARB2	10	1167313	1772498	2049	187	10164	1.5036	0.06634
-	DAPK3	19	3948453	4006123	51	7	10164	1.5027	0.066462
-	MICALL2	7	1418465	1494497	148	8	10164	1.5023	0.066511
-	RASA4B	7	102473139	102552777	9	3	10164	1.502	0.066554
+	PTK7	6	43041268	43171720	318	27	10164	1.5016	0.066595
+	LOC1053724	19	51236237	51305425	207	28	10164	1.5014	0.066632
+	PTH2R	2	208324845	208504506	387	19	10164	1.5007	0.06672
-	OR6C70	12	55459200	55694844	876	39	10164	1.5002	0.066782
-	PIWIL3	22	24709034	24809720	408	31	10164	1.5002	0.066784
+	MMACHC	1	45465184	45521067	193	23	10164	1.4999	0.066815
+	CROT	7	87310635	87409796	212	30	10164	1.4998	0.066833
-	LAD1	1	201370838	201434541	158	34	10164	1.4991	0.06692
-	OPN1SW	7	128762489	128810790	108	14	10164	1.4989	0.066945
-	EIF2AK3	2	88546741	88662576	220	10	10164	1.4989	0.066948
-	ERC2	3	55498308	56503366	3026	117	10164	1.4989	0.066949
+	KIF20B	10	89666590	89784943	458	20	10164	1.4989	0.066955
+	CFAP54	12	96454546	96885555	1553	42	10164	1.4985	0.066998
+	BCL2L12	19	49630142	49683916	97	20	10164	1.4984	0.067009
-	LYPD8	1	248711230	248790792	237	11	10164	1.4982	0.067044
+	HIST1H4C	6	26068948	26114337	231	39	10164	1.4979	0.067076
+	BSN	3	49519489	49681549	277	26	10164	1.4969	0.067203
+	SCAMP5	15	74960535	75031495	232	24	10164	1.4967	0.067233
-	GALE	1	23785599	23835804	61	8	10164	1.4961	0.067319
-	TMEM11	17	21187950	21249595	87	9	10164	1.4957	0.067369
+	LOC1053692	17	20886024	21009752	206	12	10164	1.4955	0.067397
+	PWP1	12	107650813	107722480	202	26	10164	1.4952	0.067435
-	GZMB	14	24620948	24669226	189	27	10164	1.495	0.067452
+	CDK14	7	90561362	91220590	1825	68	10164	1.4923	0.067815
-	KCNH2	7	150934956	151013314	181	29	10164	1.4918	0.06787
+	CIART	1	150247533	150297093	87	12	10164	1.4911	0.067965
+	MRS2	6	24367908	24436194	342	29	10164	1.4909	0.067999
-	KAT2A	17	42103111	42156393	118	9	10164	1.4903	0.068068
-	FNBP4	11	47706517	47802460	166	14	10164	1.4901	0.0681
-	GNG12	1	67691466	67868779	501	39	10164	1.4889	0.068254
-	OR52A1	11	5141431	5187369	212	18	10164	1.4888	0.068276
+	CRIP1	14	105451920	105498789	20	5	10164	1.4885	0.068312
-	MCM3AP	21	46225125	46321559	392	25	10164	1.4882	0.068354
+	HIST1H2AC	6	26089145	26134690	233	32	10164	1.488	0.068375
+	XRCC6BP1	12	57906541	57967269	174	15	10164	1.4879	0.068384
+	IL36RN	2	113023638	113074744	237	22	10164	1.4868	0.068536
-	S100A7L2	1	153426995	153475027	89	7	10164	1.4865	0.068571
+	SRC	20	37309685	37415432	176	19	10164	1.486	0.068645
-	C6orf47	6	31648298	31695772	229	52	10164	1.4854	0.068723
-	FOXJ1	17	76126333	76176299	92	13	10164	1.4853	0.068737
-	RMND1	6	151394761	151487181	368	32	10164	1.4834	0.068984
+	SMIM17	19	56608159	56667246	133	17	10164	1.4827	0.069072
-	NMT2	10	15095700	15203705	355	48	10164	1.4826	0.069092
+	SNAP23	15	42456244	42543061	160	24	10164	1.4826	0.069095
-	NETO2	16	47071520	47179025	53	3	10164	1.4825	0.069105
+	AIF1L	9	131061476	131133152	231	26	10164	1.4821	0.069153
+	CALCOCO2	17	48795988	48875245	245	24	10164	1.4821	0.06916
-	LOC1019274	13	33002924	33051442	196	25	10164	1.4796	0.069492
-	NDRG2	14	21006763	21105872	159	41	10164	1.4794	0.069511
-	TNP1	2	216849458	216895059	151	25	10164	1.479	0.069568
-	MB21D1	6	73406258	73487330	230	40	10164	1.4784	0.069651
+	ITGAL	16	30437662	30533185	43	9	10164	1.4781	0.069689
+	C12orf54	12	48420581	48506514	347	18	10164	1.4767	0.069874

STRAND	SYMBOL	CHR	START	STOP	NSNPS	NPARAM	N	ZSTAT	P
+	GPR3	1	27357644	27405814	50	9	10164	1.4766	0.069897
+	SLC27A1	19	17433774	17516168	167	13	10164	1.4766	0.069898
-	NT5DC2	3	52514369	52570077	130	16	10164	1.4765	0.069904
+	LHFPL5	6	35762420	35834075	352	40	10164	1.4761	0.069959
+	ZNF517	8	144763876	144819144	139	10	10164	1.4754	0.070049
-	HNRNPA0	5	137741384	137789350	29	7	10164	1.4746	0.070157
-	ALAD	9	113376312	113436338	128	16	10164	1.4741	0.070222
-	NT5C1A	1	39649121	39707038	190	34	10164	1.4739	0.07025
-	ATP5J	21	25714480	25770654	141	18	10164	1.4737	0.070287
+	HSPB9	17	42087738	42133353	83	9	10164	1.4736	0.070295
+	RHD	1	25237393	25340445	5	3	10164	1.4734	0.070317
-	BRINP1	9	119156630	119404461	214	29	10164	1.4731	0.070368
+	CAPN7	3	15171226	15262916	114	12	10164	1.473	0.070379
+	RPIA	2	88656648	88760935	227	9	10164	1.4727	0.07042
+	CALM3	19	46566255	46620782	49	12	10164	1.4725	0.070442
-	TSG101	11	18470311	18561956	221	16	10164	1.4722	0.070481
-	ZNF404	19	43862363	43918964	134	9	10164	1.472	0.070513
-	APOBEC1	12	7639400	7705599	299	31	10164	1.4716	0.070564
-	TMEM240	1	1524778	1575360	1	1	10164	1.4714	0.070591
+	CACNG3	16	24220553	24372416	444	73	10164	1.4708	0.070669
+	METTL7A	12	50888811	50942517	148	19	10164	1.4703	0.070737
+	C9orf43	9	113375644	113439684	138	18	10164	1.47	0.07078
-	GDF3	12	7679785	7730764	246	43	10164	1.4697	0.07082
-	CLASP1	2	121327776	121684476	794	13	10164	1.4696	0.070829
+	BSND	1	54963944	55018792	101	20	10164	1.4696	0.070837
-	DPP9	19	4665226	4758843	80	7	10164	1.4694	0.070859
-	C7orf31	7	25115200	25215748	325	21	10164	1.4688	0.070938
-	FCN3	1	27359110	27409825	50	9	10164	1.4686	0.070966
-	FAM53A	4	1564062	1719313	471	40	10164	1.4686	0.070969
+	MPV17L	16	15360698	15419686	89	11	10164	1.4681	0.071039
+	LARS2	3	45353561	45558842	566	50	10164	1.468	0.071046
+	MMRN1	4	89859876	89964629	224	17	10164	1.468	0.071052
-	ZMYM5	13	19813482	19898636	242	7	10164	1.4678	0.071082
-	SSR1	6	7271143	7348314	422	28	10164	1.4672	0.071165
+	PINLYP	19	43541800	43592104	168	30	10164	1.4669	0.071198
+	TOE1	1	45304670	45353978	140	23	10164	1.4661	0.071317
+	POLR2K	8	100115611	100164002	116	11	10164	1.465	0.071454
-	IGSF6	16	21631284	21687666	101	11	10164	1.4647	0.071498
-	ASPN	9	92446205	92517562	130	10	10164	1.4642	0.071569
-	PLEKHA4	19	48827092	48903627	217	21	10164	1.4637	0.071635
+	ISG20	15	88600622	88666344	235	31	10164	1.4636	0.071648
+	LOC1053699	12	45955062	46014827	96	16	10164	1.4627	0.071772
-	ZNF624	17	16589870	16690626	153	5	10164	1.4626	0.071783
+	GARNL3	9	127189471	127403549	515	46	10164	1.4624	0.071818
+	DPPA3	12	7676454	7727559	235	41	10164	1.4623	0.071824
+	PARP2	14	20308614	20367910	188	16	10164	1.4623	0.071828
-	HMGCL	1	23791877	23860459	102	10	10164	1.4617	0.071915
-	WARS2	1	119021216	119175788	534	44	10164	1.4603	0.072103
-	FABP9	8	81448383	81496523	129	9	10164	1.46	0.072148
-	RAX2	19	3759089	3807221	21	4	10164	1.4592	0.072259
+	RNF180	5	64130311	64384375	377	16	10164	1.4589	0.072293
+	OR10H4	19	15914008	15959958	159	12	10164	1.4587	0.072324
-	CDK5RAP1	20	33348839	33436902	162	19	10164	1.4582	0.072397
-	MUTYH	1	45319242	45375470	153	21	10164	1.4581	0.072413
+	WBSCR22	7	73648566	73708221	100	8	10164	1.4579	0.072437
+	LOC1053775	4	186069788	186134062	219	24	10164	1.4578	0.072453
-	SCRT2	20	651596	711179	148	30	10164	1.4576	0.072472
+	TMEM37	2	119394912	119448520	135	20	10164	1.4573	0.072511
+	LOC1053732	1	227751481	227800651	191	32	10164	1.456	0.0727
+	APOBEC3H	22	39062223	39114067	194	14	10164	1.4555	0.072765
-	SPTBN5	15	41838146	41929077	351	43	10164	1.4552	0.072807
-	HS6ST1	2	128255480	128353597	175	31	10164	1.4548	0.072856
+	OR5AK2	11	56953914	56999843	164	19	10164	1.4542	0.072951

STRAND	SYMBOL	CHR	START	STOP	NSNPS	NPARAM	N	ZSTAT	P
+	TBC1D10C	11	67368686	67420090	111	18	10164	1.4538	0.072996
+	RAP2A	13	97399221	97477998	85	20	10164	1.4526	0.073163
+	LOC1053729	22	23459294	23520197	30	2	10164	1.451	0.073394
-	PLIN3	19	4828334	4902768	183	27	10164	1.4492	0.073642
-	ST3GAL5	2	85829147	85924034	314	14	10164	1.4489	0.073678
-	KSR2	12	117443012	118004103	1878	185	10164	1.4477	0.073851
-	LOC1053789	5	56760327	56808143	125	19	10164	1.4454	0.074173
-	ZC3H3	8	143427655	143577245	562	44	10164	1.4454	0.074176
-	SLC8A1	2	40102146	40547486	1514	77	10164	1.4452	0.074203
+	LOC1053790	5	79478678	79525303	146	22	10164	1.4451	0.07421
-	ZNF671	19	57709747	57762627	101	12	10164	1.4445	0.074297
-	GRIP2	3	14479111	14637996	517	67	10164	1.444	0.074365
+	MPHOSPH8	13	19598647	19683459	142	9	10164	1.4426	0.074571
-	CYTH3	7	6151779	6307611	370	38	10164	1.4423	0.074609
+	ZNF365	10	62339157	62682011	1201	100	10164	1.4419	0.074671
-	CDO1	5	115794733	115851954	67	9	10164	1.4406	0.074845
+	FAM84A	2	14597686	14650046	71	14	10164	1.4403	0.074887
+	SNED1	2	240962597	241104228	378	47	10164	1.4401	0.074915
+	PLCD4	2	218572765	218647186	136	8	10164	1.4398	0.074967
+	HSCB	22	28707001	28767518	188	33	10164	1.4397	0.074979
-	MPZ	1	161294735	161344972	171	8	10164	1.4383	0.07517
-	AGBL2	11	47649169	47750382	156	14	10164	1.4381	0.075205
-	EIF3A	10	119025029	119115822	225	18	10164	1.4372	0.075331
-	GPR33	14	31472944	31522945	167	23	10164	1.4364	0.075437
+	TGIF2	20	36538473	36603952	47	18	10164	1.4356	0.075561
+	PTDSS2	11	413271	501399	237	21	10164	1.4338	0.07581
+	STK32A	5	147199963	147411533	716	43	10164	1.4329	0.075937
-	LOC390937	19	41897535	41963122	97	12	10164	1.4329	0.075945
-	GNAT3	7	80447292	80562915	249	26	10164	1.4326	0.075981
-	CCNB1IP1	14	20301368	20368312	205	17	10164	1.4325	0.075997
+	ACTR3	2	113854934	113971554	441	24	10164	1.4322	0.07605
+	EIF4EBP3	5	140512666	140559578	46	11	10164	1.4306	0.076277
+	PCMT1	6	149714695	149821421	233	8	10164	1.4301	0.07635
+	CACNA1D	3	53459976	53823151	1006	107	10164	1.4299	0.076374
-	MAP3K6	1	27345179	27403014	71	15	10164	1.4299	0.076379
-	HAND1	5	154464972	154513264	171	31	10164	1.4296	0.076421
+	SNX33	15	75614007	75668627	138	32	10164	1.4288	0.07653
+	PCNP	3	101538534	101604437	193	22	10164	1.4282	0.076619
-	CYB5R1	1	202951873	203002276	152	27	10164	1.4279	0.076658
+	ARMT1	6	151417258	151480101	245	29	10164	1.4274	0.076731
-	GPR149	3	154327672	154464715	311	28	10164	1.4272	0.076767
-	LRRC39	1	100138448	100213273	110	12	10164	1.4269	0.076808
+	PPP1R16A	8	144442987	144512121	115	10	10164	1.4257	0.076975
+	PIM1	6	37135146	37185428	176	25	10164	1.4254	0.07702
+	ZNF274	19	58147989	58223562	145	20	10164	1.4254	0.07702
-	ASTN2	9	116415225	117450039	3581	244	10164	1.4247	0.077115
-	SEL1L	14	81461547	81568861	191	32	10164	1.4247	0.07712
-	HIST1H1T	6	26097412	26143136	229	32	10164	1.4247	0.077122
+	CDRT15L2	17	20544724	20590911	3	1	10164	1.4246	0.077133
+	MGAT5B	17	76815503	76960393	437	46	10164	1.4245	0.077146
-	ACTB	7	5517148	5565601	106	18	10164	1.4239	0.077234
-	SLC39A3	19	2722524	2775076	94	5	10164	1.4239	0.077245
+	PDHX	11	34880829	35006128	564	15	10164	1.4236	0.077277
-	MAX	14	64996101	65137695	353	26	10164	1.4234	0.077316
-	PHF23	17	7225028	7274717	87	17	10164	1.4232	0.077334
+	CELSR2	1	109214715	109285756	117	17	10164	1.4232	0.07734
-	SHPRH	6	145852721	145999285	341	11	10164	1.4231	0.077348
+	CSNK2B	6	31630880	31680070	272	56	10164	1.4225	0.077439
-	FBXO30	6	145788121	145849786	96	12	10164	1.4219	0.077533
-	C1QTNF9B	13	23881099	23933503	201	24	10164	1.4218	0.07754
+	STOX2	4	183761331	184033526	426	60	10164	1.4213	0.077615
+	SUMO4	6	149365359	149411046	120	11	10164	1.4204	0.077749
+	PGD	1	10363992	10430511	190	17	10164	1.4203	0.077757

STRAND	SYMBOL	CHR	START	STOP	NSNPS	NPARAM	N	ZSTAT	P
+	GALNT8	12	4685586	4782726	271	18	10164	1.4201	0.077794
-	FANCG	9	35063838	35115016	148	28	10164	1.4191	0.07793
-	SLC25A40	7	87823568	87911377	201	22	10164	1.4189	0.077971
+	GPNMB	7	23211686	23285110	149	13	10164	1.4183	0.078046
+	VAMP3	1	7736269	7791432	152	24	10164	1.4183	0.078051
+	GRB7	17	39702909	39757285	58	12	10164	1.418	0.078094
+	RQCD1	2	218533575	218606435	128	7	10164	1.4177	0.078141
-	IQCB1	3	121759763	121870079	364	15	10164	1.4173	0.078202
-	PSPC1	13	19664752	19818019	261	12	10164	1.4171	0.078221
+	RAB3GAP1	2	135017265	135185584	332	17	10164	1.4164	0.078325
+	ANP32D	12	48437665	48483060	179	13	10164	1.4161	0.078372
+	PLA2G2F	1	20104326	20160386	98	28	10164	1.4157	0.078425
-	TRA2B	3	185904568	185973136	157	22	10164	1.4151	0.078513
-	TNNT2	1	201349008	201412765	198	29	10164	1.4143	0.078643
+	KIAA1324	1	109078963	109216781	305	26	10164	1.4142	0.078655
-	SHFM1	7	96678767	96744891	139	17	10164	1.4139	0.078696
-	MAP3K19	2	134954290	135082444	343	28	10164	1.4134	0.078765
-	LOC1019272	19	31139950	31267298	220	19	10164	1.4132	0.078805
+	INA	10	103242163	103300351	192	28	10164	1.4114	0.079067
+	UTS2R	17	82339277	82385586	122	7	10164	1.4105	0.079196
+	OLFML2A	9	124742158	124824891	166	32	10164	1.4101	0.079252
-	FAM188A	10	15768169	15895520	98	14	10164	1.4097	0.079312
-	FAM219B	15	74889987	74942307	152	28	10164	1.4095	0.079338
-	PM20D1	1	205818022	205885148	143	23	10164	1.4094	0.079357
-	LOXHD1	18	46466961	46692114	803	82	10164	1.4083	0.079522
-	TUBE1	6	112060657	112122548	175	11	10164	1.4082	0.079529
-	FBXO41	2	73244682	73319453	33	6	10164	1.4078	0.079596
+	F11	4	186230945	186299681	242	35	10164	1.4078	0.079599
+	C5orf47	5	173954159	174016140	158	16	10164	1.4052	0.079988
-	CNGA1	4	47925015	48051679	459	28	10164	1.405	0.080007
+	HOXC13	12	53903792	53956544	159	26	10164	1.4044	0.080102
+	ZNF17	19	57376161	57431953	93	13	10164	1.4038	0.08019
+	PRR20B	13	57112488	57160509	83	18	10164	1.4036	0.080215
-	CD164L2	1	27369100	27418333	49	6	10164	1.4029	0.080329
+	IARS2	1	220059113	220158041	238	30	10164	1.4017	0.0805
-	NSMCE1	16	27214994	27303809	181	19	10164	1.4017	0.080505
+	LOC1010605	15	30480862	30528467	4	1	10164	1.4014	0.080545
-	GAREM	18	32253521	32505496	776	45	10164	1.4011	0.080593
-	RPL7	8	73280639	73329494	143	23	10164	1.4011	0.080598
+	ARHGEF12	11	120301406	120499936	372	21	10164	1.4011	0.080598
+	SCNN1G	16	23147719	23226879	219	21	10164	1.401	0.080611
+	C1orf94	1	34131883	34229131	272	34	10164	1.4009	0.080616
-	LOC1053720	18	23123586	23170761	116	17	10164	1.4007	0.080647
+	LOC1053769	19	43866660	43945219	214	16	10164	1.4005	0.080684
+	OR6C2	12	55417214	55463152	135	13	10164	1.4004	0.080696
+	LTB4R2	14	24275151	24322053	105	14	10164	1.4002	0.080733
-	OASL	12	121010292	121074242	227	37	10164	1.3992	0.080871
-	HACL1	3	15550704	15636852	203	29	10164	1.3972	0.081181
+	ST14	11	130124787	130220362	196	28	10164	1.3968	0.081235
+	NUS1	6	117640454	117720727	223	38	10164	1.3962	0.081322
+	LETMD1	12	51013230	51077369	145	19	10164	1.3961	0.081339
+	EIF3K	19	38583419	38646959	183	36	10164	1.3955	0.081427
-	MIEN1	17	39719156	39765541	46	11	10164	1.3952	0.081472
+	NPL	1	182754449	182840384	192	25	10164	1.3944	0.081602
+	KL	13	32981063	33076145	312	30	10164	1.3942	0.081624
+	GABPA	21	25699947	25782460	211	23	10164	1.3941	0.081649
-	LY6G5C	6	31666684	31715373	228	55	10164	1.3934	0.081746
+	ATAD3A	1	1477143	1544687	3	2	10164	1.3929	0.081819
-	LOC1053708	15	62239533	62291938	156	21	10164	1.3926	0.081865
-	GRB10	7	50580063	50828462	804	35	10164	1.3918	0.081995
+	PER3	1	7749380	7855181	337	26	10164	1.3916	0.08202
-	USP22	17	20989593	21078409	213	9	10164	1.3916	0.082028
-	ORC1	1	52362829	52439471	98	17	10164	1.391	0.082105

STRAND	SYMBOL	CHR	START	STOP	NSNPS	NPARAM	N	ZSTAT	P
+	PHKB	16	47426268	47711523	128	4	10164	1.3887	0.082465
-	HARS	5	140663904	140726727	110	7	10164	1.3883	0.082522
-	CCDC82	11	96342765	96424931	202	24	10164	1.3882	0.082538
-	NUP43	6	149714315	149781572	143	7	10164	1.3878	0.082604
-	SLC39A8	4	102241041	102380498	411	29	10164	1.3876	0.082634
-	CPA6	8	67412170	67781571	871	65	10164	1.3874	0.082666
-	POLDIP2	17	28336628	28392589	100	8	10164	1.3871	0.082709
+	HARS2	5	140656426	140709318	85	7	10164	1.3858	0.0829
+	SLC1A4	2	64953445	65033865	129	19	10164	1.3854	0.082965
-	TMEM196	7	19709315	19808576	451	27	10164	1.385	0.083022
-	ARHGAP17	16	24909384	25050601	285	23	10164	1.385	0.083024
+	SERHL2	22	42483735	42584382	253	36	10164	1.3847	0.083065
-	MPDZ	9	13095296	13314662	165	12	10164	1.3846	0.083085
-	CTSV	9	97019677	97074643	139	11	10164	1.3845	0.083106
-	GRIK3	1	36785527	37069548	340	30	10164	1.3843	0.083131
+	SLC47A1	17	19498854	19589033	140	18	10164	1.3843	0.083137
-	KIF17	1	20651643	20753017	306	52	10164	1.3842	0.083143
-	OR8B4	11	124413942	124459871	218	25	10164	1.3842	0.083145
-	NOL6	9	33451353	33508943	160	18	10164	1.3841	0.083162
+	SETD5	3	9362700	9488154	287	22	10164	1.3839	0.083193
+	CHGB	20	5876328	5935361	251	34	10164	1.3835	0.083257
+	ZNF527	19	37336048	37403066	205	24	10164	1.3834	0.083266
-	FGA	4	154573128	154625745	60	8	10164	1.3832	0.083305
-	LOC1019294	12	2838243	2892215	118	14	10164	1.383	0.083325
+	LOC653602	2	14600091	14660819	80	13	10164	1.3828	0.083359
+	PLCB4	20	9033710	9490816	724	60	10164	1.3828	0.083361
-	ZNF142	2	218627916	218694632	126	10	10164	1.3828	0.083361
+	GSDMD	8	143518387	143573062	232	28	10164	1.3825	0.083414
-	BAG6	6	31629028	31695767	352	51	10164	1.3824	0.083421
-	ANO9	11	407930	477011	133	15	10164	1.382	0.083493
-	JOSD2	19	50495997	50546355	164	37	10164	1.3816	0.083543
+	LHX4	1	180193803	180285053	255	38	10164	1.3815	0.083556
+	AMBN	4	70557258	70617288	187	26	10164	1.3815	0.08356
+	OR2H2	6	29552378	29601012	285	49	10164	1.3812	0.08361
+	ZMAT2	5	140663743	140716663	89	7	10164	1.3809	0.083655
-	ZNF154	19	57686275	57744211	126	16	10164	1.3805	0.083714
+	USP5	12	6817118	6876632	61	16	10164	1.3804	0.083732
+	PRR20D	13	57125632	57173653	52	14	10164	1.3796	0.083852
+	FNDC3A	13	48940912	49219779	400	26	10164	1.3795	0.083866
-	FAM102A	9	127930582	128016027	280	19	10164	1.3792	0.083918
+	MAG	19	35257086	35323807	213	37	10164	1.3791	0.083931
-	VTN	17	28357277	28405352	93	7	10164	1.3782	0.084071
+	SLITRK5	13	87636232	87689616	108	10	10164	1.3781	0.084085
+	OPN4	10	86619557	86676460	256	24	10164	1.3779	0.084114
+	FOXC1	6	1575446	1623897	85	27	10164	1.3775	0.084179
-	FRK	6	115931528	116135728	569	41	10164	1.3766	0.084311
+	VPS37D	7	73632844	73682110	75	10	10164	1.3766	0.084325
+	CAPN2	1	223666593	223786018	439	32	10164	1.3763	0.084359
-	MAP4K1	19	38577640	38653035	222	43	10164	1.3758	0.084436
-	SEBOX	17	28354268	28400151	95	8	10164	1.3756	0.084467
-	PPP5D1	19	46470788	46636200	345	39	10164	1.3755	0.084483
+	C22orf15	22	23725033	23775863	170	23	10164	1.3754	0.084504
+	ZNF776	19	57711796	57768159	116	13	10164	1.3747	0.084605
-	HOXB2	17	48532655	48580031	167	41	10164	1.3745	0.084646
-	TOP1MT	8	143299324	143394977	333	24	10164	1.3745	0.084647
+	WFDC2	20	45413648	45491532	256	44	10164	1.3743	0.084667
+	NGDN	14	23434628	23488193	123	7	10164	1.3737	0.084762
+	LOC1053768	18	12396366	12446206	182	31	10164	1.3724	0.084966
-	EPM2A	6	145490777	145771303	768	17	10164	1.3719	0.08504
-	TFRC	3	196039284	196117161	271	21	10164	1.3717	0.085086
+	GFRAL	6	55292469	55412493	636	38	10164	1.371	0.085184
-	DND1	5	140660796	140708586	81	7	10164	1.3706	0.085246
+	ARHGEF16	1	3419583	3491115	197	21	10164	1.3704	0.085288

STRAND	SYMBOL	CHR	START	STOP	NSNPS	NPARAM	N	ZSTAT	P
+	TOR4A	9	137242828	137292641	126	11	10164	1.3703	0.085293
+	LOC1019277	11	65419421	65476720	154	22	10164	1.3694	0.085431
+	DBF4	7	87841229	87919541	167	20	10164	1.3687	0.085542
-	CLDND1	3	98505473	98558066	136	28	10164	1.3686	0.085563
+	MARK2	11	63803928	63921020	187	20	10164	1.3681	0.085633
+	FAM229B	6	112052471	112112790	170	11	10164	1.3681	0.085634
-	SNTA1	20	33397957	33478892	143	25	10164	1.368	0.085653
-	STK25	2	241484707	241544730	239	34	10164	1.3677	0.085705
-	HIST1H4H	6	26275126	26320499	214	22	10164	1.3673	0.085768
-	ALKBH8	11	107492726	107600737	312	20	10164	1.367	0.085813
-	FKBP1A	20	1358977	1428172	246	22	10164	1.3669	0.085823
+	GABRA5	15	26831719	26959210	177	21	10164	1.3669	0.085825
-	SBSN	19	35513367	35563351	164	28	10164	1.3651	0.086117
+	SERPING1	11	57562554	57624853	158	22	10164	1.3649	0.086143
+	OPRD1	1	28777142	28873696	177	19	10164	1.3647	0.086179
+	HKR1	19	37299678	37374455	250	28	10164	1.3638	0.086316
-	SLC1A5	19	46749698	46823649	203	26	10164	1.363	0.086449
+	C10orf2	10	100952378	101004403	81	9	10164	1.3625	0.086523
-	MTCH2	11	47595204	47677654	105	15	10164	1.3624	0.086542
-	ZNF747	16	30520367	30569970	11	4	10164	1.362	0.086594
+	HPR	16	72028226	72087246	171	27	10164	1.3618	0.086625
+	PSMA6	14	35243558	35327479	244	23	10164	1.3617	0.086643
-	DPF1	19	38201006	38264714	268	55	10164	1.3616	0.086662
-	CCDC102A	16	57502178	57572130	154	17	10164	1.3614	0.086693
-	LATS1	6	149648153	149753256	184	10	10164	1.3612	0.086724
-	PHOX2A	11	72229077	72279176	122	19	10164	1.3607	0.086811
+	CD19	16	28896916	28949347	54	11	10164	1.3606	0.086813
-	KRTAP5-2	11	1587177	1633294	248	23	10164	1.3606	0.086823
-	ANKRD34A	1	145949441	145999591	2	1	10164	1.3602	0.086881
-	DHX16	6	30643119	30708053	358	30	10164	1.3602	0.086891
-	ADPGK	15	72741294	72818785	147	13	10164	1.3601	0.086893
-	GZMH	14	24596480	24644774	174	28	10164	1.3599	0.086936
+	TMEM199	17	28322581	28373683	76	11	10164	1.359	0.087078
-	PPP1R18	6	30666389	30722895	356	26	10164	1.3585	0.087145
+	MYO16	13	108460729	109218007	2691	180	10164	1.3583	0.087181
-	LOC1053700	13	18932475	18979377	82	11	10164	1.358	0.087227
-	NT5C3B	17	41815082	41871271	139	6	10164	1.3579	0.087243
-	LHX6	9	122175652	122263893	174	32	10164	1.3571	0.087381
-	OTUD7A	15	31471682	31905685	823	25	10164	1.3567	0.087433
+	PKN3	9	128667499	128730920	74	11	10164	1.3556	0.08762
+	SEZ6L	22	26134474	26393597	416	47	10164	1.3553	0.087655
-	NXPE4	11	114560591	114630840	186	24	10164	1.3545	0.087782
-	PNMAL1	19	46456491	46506563	175	18	10164	1.3542	0.087835
-	DERL1	8	123003164	123077423	143	23	10164	1.3541	0.087853
+	APOBEC3A	22	38922522	38973183	121	12	10164	1.3539	0.087876
-	CADPS	3	62388346	62910389	1694	117	10164	1.3535	0.087946
-	RAPH1	2	203423682	203570335	254	26	10164	1.3534	0.087957
+	SARM1	17	28336662	28411045	130	10	10164	1.3533	0.087983
-	CFAP126	1	161354731	161402883	183	3	10164	1.3524	0.088127
-	EXOC2	6	475138	728141	891	65	10164	1.3523	0.088144
-	FAM71E1	19	50456643	50511746	187	31	10164	1.3519	0.088208
+	CANX	5	179643646	179741641	212	29	10164	1.3512	0.088318
+	FKBP9	7	32922404	33016931	275	34	10164	1.3511	0.088334
+	RDH10	8	73259602	73335285	233	35	10164	1.3502	0.08847
+	FMN2	1	240056885	240485189	1441	105	10164	1.3501	0.088489
-	CHCHD3	7	132774862	133117079	766	31	10164	1.3501	0.08849
+	THUMPD3	3	9327976	9396791	176	22	10164	1.3496	0.088565
+	ANLN	7	36354803	36463791	294	21	10164	1.3496	0.088579
-	KIF13A	6	17749183	18022629	528	19	10164	1.3493	0.088618
-	OMD	9	92404245	92459554	133	10	10164	1.349	0.088667
-	POLD4	11	67340765	67388596	81	16	10164	1.3489	0.088683
+	GPX3	5	150985438	151038993	189	32	10164	1.3475	0.088917
-	KATNA1	6	149584873	149684018	194	8	10164	1.3471	0.088974

STRAND	SYMBOL	CHR	START	STOP	NSNPS	NPARAM	N	ZSTAT	P
-	KRTAP21-1	21	30745139	30790378	62	4	10164	1.347	0.088984
+	IGF1R	15	98613539	98974530	1056	96	10164	1.3469	0.089005
-	DHRS1	14	24280598	24334833	140	22	10164	1.346	0.089149
+	FAM20C	7	157777	270774	253	24	10164	1.3459	0.089173
+	THAP3	1	6589865	6645586	107	14	10164	1.3454	0.089253
-	ZNF19	16	71464073	71524351	124	15	10164	1.3447	0.089363
-	ANGPT2	8	6489651	6598263	666	67	10164	1.3446	0.089379
-	UEVLD	11	18519609	18623938	251	24	10164	1.3446	0.089381
+	PDIA5	3	123032009	123172106	411	56	10164	1.3442	0.089444
-	TRMT10A	4	99536707	99599057	204	16	10164	1.3439	0.089483
-	NSFL1C	20	1432162	1502720	211	16	10164	1.3438	0.08951
+	ACTR10	14	58165115	58245636	93	14	10164	1.3435	0.08955
-	GABRB3	15	26533546	26808788	535	43	10164	1.3429	0.089648
-	PMP2	8	81430326	81482484	130	8	10164	1.3428	0.089676
-	DVL2	17	7215342	7269548	85	16	10164	1.3421	0.08979
+	ELP5	17	7217053	7269940	85	16	10164	1.3421	0.08979
+	SLC35A4	5	140529565	140579104	60	12	10164	1.3414	0.08989
+	LTB4R	14	24276499	24328036	121	17	10164	1.3414	0.089894
-	APIP	11	34872295	34951411	436	11	10164	1.3411	0.089941
-	SLC18A1	8	20134855	20218338	330	35	10164	1.3405	0.090038
+	TRIM77	11	89675299	89727872	168	13	10164	1.3402	0.090087
-	NLRP10	11	7949023	8000424	121	20	10164	1.3401	0.090106
+	RAB10	2	25998860	26147454	199	7	10164	1.3401	0.090112
+	GPR137C	14	52518148	52647713	395	34	10164	1.3398	0.090152
-	GPR151	5	146504854	146551113	125	17	10164	1.3395	0.090198
+	CYR61	1	85545761	85593967	158	33	10164	1.3394	0.090227
-	SEC16A	9	136430096	136519549	162	13	10164	1.3393	0.090234
+	KNTC1	12	122492249	122637311	230	17	10164	1.3392	0.090245
+	GDAP1	8	74314251	74377110	210	27	10164	1.3391	0.09027
-	PRKG2	4	81077370	81251743	404	25	10164	1.3387	0.090341
+	KLRD1	12	10269446	10370876	160	19	10164	1.3361	0.090751
+	POLD1	19	50349290	50428018	269	29	10164	1.336	0.090777
+	LOC1053781	6	164149366	164347805	563	55	10164	1.3359	0.090791
-	RCAN1	21	34506442	34650142	740	40	10164	1.3358	0.090811
-	NUP153	6	17605035	17741834	315	26	10164	1.3358	0.090813
-	SLPI	20	45242239	45289564	179	6	10164	1.3357	0.090829
-	DDX17	22	38473438	38541340	104	13	10164	1.3349	0.090949
-	FBXW7	4	152311258	152571095	468	11	10164	1.3349	0.090963
+	DAG1	3	49433703	49545618	216	21	10164	1.3347	0.090992
-	PEX11B	1	145901348	145953924	7	2	10164	1.3344	0.091044
+	CFAP221	2	119509423	119670321	302	20	10164	1.3343	0.091052
-	ZNF605	12	132911433	132991306	266	20	10164	1.3342	0.091073
+	CIPC	14	77063235	77127287	213	30	10164	1.3338	0.091135
+	LOC1053702	13	39566324	39650412	175	30	10164	1.3337	0.091154
+	NR1H2	19	50341423	50393028	144	18	10164	1.3322	0.091405
-	CYP27C1	2	127173832	127261356	183	15	10164	1.332	0.091429
+	SDK1	7	3266448	4279000	4467	93	10164	1.3318	0.091465
-	MRPL43	10	100967822	101022515	106	13	10164	1.3317	0.091478
-	ANGPT1	8	107239482	107533026	955	66	10164	1.3315	0.09152
-	EHHADH	3	185180624	185317886	289	31	10164	1.3308	0.091628
-	LATS2	13	20963036	21096583	355	20	10164	1.3305	0.091669
+	ZNF547	19	57328435	57389559	186	16	10164	1.3305	0.091673
+	SDHC	1	161279376	161374751	339	12	10164	1.3303	0.091706
+	STX16	20	58616253	58689526	139	21	10164	1.3303	0.09171
+	LOC1053739	2	121575402	121659344	232	10	10164	1.3299	0.091771
+	IRAK2	3	10129879	10253745	556	64	10164	1.3298	0.0918
+	TMEM72	10	44876308	44947010	265	27	10164	1.3296	0.09183
-	PAIP2B	2	71172738	71262114	236	22	10164	1.3295	0.091834
-	DUOX2	15	45082653	45149161	106	17	10164	1.3293	0.091877
-	KCNE1	21	34408715	34547275	233	19	10164	1.3293	0.091878
-	SLC16A12	10	89420294	89570556	348	42	10164	1.3291	0.091902
-	FOXH1	8	144463732	144511335	56	7	10164	1.3277	0.092136
-	ZNF823	19	11711265	11774009	128	13	10164	1.3274	0.092192

STRAND	SYMBOL	CHR	START	STOP	NSNPS	NPARAM	N	ZSTAT	P
-	TBC1D32	6	121069494	121369956	1357	30	10164	1.3271	0.092231
-	ADD2	2	70651202	70803243	559	41	10164	1.327	0.092256
+	ICAM5	19	10254435	10306778	129	24	10164	1.3269	0.092268
-	RSRC2	12	122494643	122562013	109	12	10164	1.3269	0.09227
+	MLLT4	6	167791922	167982023	189	7	10164	1.3268	0.092286
-	ACAP2	3	195264736	195478176	516	10	10164	1.3265	0.092335
+	ZFPM1	16	88416431	88545166	168	22	10164	1.3261	0.0924
+	SUDS3	12	118338189	118428035	209	17	10164	1.326	0.092415
+	SH2D4B	10	80502902	80656560	494	43	10164	1.3256	0.092479
-	NIFK	2	121716945	121771927	100	8	10164	1.3253	0.09254
+	SQSTM1	5	179771388	179848078	198	36	10164	1.3251	0.092572
+	CCDC7	10	32411082	32892864	1511	27	10164	1.3251	0.092575
+	HNRNPAB	5	178169507	178221183	192	33	10164	1.325	0.092578
-	HIST1H2BC	6	26113467	26158904	205	39	10164	1.325	0.092579
+	LOC1009968	17	19542660	19643825	161	20	10164	1.325	0.092588
+	RAD50	5	132521924	132654621	342	26	10164	1.3239	0.092762
-	OBFC1	10	103860258	103953287	258	24	10164	1.3237	0.092794
+	SEMA4G	10	100934482	100995616	84	11	10164	1.3232	0.092889
+	CDADC1	13	49212875	49303486	148	11	10164	1.3231	0.092898
+	PRR20A	13	57105918	57153939	114	20	10164	1.3227	0.092965
+	SLC24A4	14	92287581	92511481	697	54	10164	1.3227	0.092974
+	ANXA4	2	69609100	69836464	792	51	10164	1.3223	0.093042
-	IQSEC1	3	12887043	13317998	955	103	10164	1.3219	0.093093
-	LOC1053781	13	112003841	112071593	123	14	10164	1.3218	0.09311
+	PMPCA	9	136375573	136433761	182	12	10164	1.3207	0.093304
+	SLC34A1	5	177349431	177408848	61	8	10164	1.3203	0.093372
+	HFE	6	26052281	26106117	249	41	10164	1.3193	0.093541
-	IRS2	13	109743837	109821567	226	29	10164	1.319	0.093585
+	TMEM147	19	35510600	35557527	156	26	10164	1.3188	0.093612
+	PNPO	17	47906523	47959308	141	20	10164	1.3184	0.093678
-	LOC1053776	4	102408707	102536891	267	37	10164	1.3176	0.093821
-	TXNL1	18	56592387	56673689	185	16	10164	1.3168	0.093952
-	PRCP	11	82814367	82936691	375	27	10164	1.3164	0.094016
-	VNN1	6	132670858	132749055	321	26	10164	1.3162	0.094058
+	DTX3L	3	122529338	122585203	175	24	10164	1.3161	0.094078
-	HMX1	4	8836077	8906817	1	1	10164	1.316	0.094087
-	LOC1053744	4	8837235	8888617	1	1	10164	1.316	0.094087
-	AMIGO3	3	49706834	49754805	88	10	10164	1.3154	0.094191
+	CCND2	12	4238733	4315356	120	30	10164	1.3144	0.094354
-	MFI2	3	196977559	197064816	80	11	10164	1.3139	0.094436
+	GNL3	3	52650920	52704497	110	10	10164	1.313	0.094584
+	PARP15	3	122542454	122649047	393	46	10164	1.3125	0.094675
-	HOXB1	17	48519445	48565910	158	33	10164	1.3121	0.094745
-	IFT20	17	28318325	28370489	79	12	10164	1.3117	0.094804
-	TXK	4	48056393	48169256	281	38	10164	1.3117	0.094813
+	NIPAL1	4	47981774	48050173	266	26	10164	1.3115	0.094838
+	EMC10	19	50441457	50515909	252	35	10164	1.3104	0.095023
+	PGBD4	15	34067073	34114390	85	9	10164	1.3103	0.095051
+	RBM33	7	155609494	155791485	414	10	10164	1.3097	0.095155
+	LZTS2	10	100962107	101017836	102	11	10164	1.3091	0.095251
-	TMCC3	12	94557124	94685562	353	41	10164	1.3089	0.095288
-	ADIPOR1	1	202930825	202993572	208	32	10164	1.3087	0.095316
-	TMEM64	8	90611995	90680905	154	13	10164	1.3081	0.095418
+	NOP9	14	24264854	24319126	125	17	10164	1.3076	0.095512
+	UHRF2	9	6378148	6517056	299	17	10164	1.3075	0.095526
-	ITFG1	16	47144387	47496274	186	6	10164	1.3073	0.095562
+	UBE2Q2L	15	84137490	84192233	60	18	10164	1.3071	0.095582
+	ZNF419	19	57452711	57504680	208	12	10164	1.3068	0.095637
+	HCRTR1	1	31582689	31642519	78	6	10164	1.3066	0.095668
+	OCLM	1	186365572	186411455	101	15	10164	1.3063	0.095728
-	CCNH	5	87381493	87448033	104	10	10164	1.3061	0.095751
+	TSN	2	121720545	121777853	106	9	10164	1.3059	0.095793
-	KRT24	17	40687991	40738750	118	16	10164	1.3054	0.095877

STRAND	SYMBOL	CHR	START	STOP	NSNPS	NPARAM	N	ZSTAT	P
-	SCAF11	12	45909131	46027040	194	26	10164	1.3051	0.095929
-	FABP4	8	81468497	81518238	114	9	10164	1.305	0.095951
+	GJB4	1	34724741	34773724	49	17	10164	1.304	0.096114
+	HSD3B2	1	119379931	119433039	190	23	10164	1.3039	0.09614
+	ZNF793	19	37471447	37553337	220	35	10164	1.3019	0.096469
+	USP8	15	50389377	50511083	416	38	10164	1.3017	0.096511
-	FOXI3	2	88438207	88487535	86	11	10164	1.3017	0.096516
-	LOC1053753	7	92402550	92472456	139	27	10164	1.3012	0.096592
-	GMPPB	3	49711476	49758974	85	12	10164	1.3011	0.096609
-	REPS1	6	138894015	139023282	385	19	10164	1.3009	0.096641
-	GFRA3	5	138242380	138309564	95	10	10164	1.3006	0.096704
-	HIST1H2BB	6	26033227	26078657	188	32	10164	1.2995	0.09689
-	CEND1	11	777110	825126	134	20	10164	1.2992	0.096944
+	DEFB125	20	52672	106573	38	9	10164	1.299	0.096972
-	SDCCAG3	9	136391922	136445602	147	11	10164	1.2986	0.097041
-	NDUFA2	5	140635363	140682785	76	6	10164	1.2977	0.0972
-	KLHL11	17	41838518	41900431	109	6	10164	1.2973	0.097268
+	RTCA	1	100231158	100302769	176	10	10164	1.2966	0.09739
-	EMC7	15	34074022	34136852	118	15	10164	1.2956	0.097552
+	PRR20C	13	57119061	57167082	69	17	10164	1.2955	0.097574
+	AP4E1	15	50873625	51015900	397	20	10164	1.2955	0.097581
-	NAPSA	19	50348472	50404331	193	20	10164	1.295	0.097659
-	AGMO	7	15149124	15597015	1226	81	10164	1.295	0.097665
-	TTC39B	9	15160844	15342360	534	61	10164	1.2949	0.09768
+	C7orf33	7	148555565	148625860	153	28	10164	1.294	0.097833
+	IL1R1	2	102034638	102189874	595	62	10164	1.2938	0.097867
-	EVA1B	1	36312030	36359154	80	19	10164	1.2938	0.097867
+	CD38	4	15743298	15859083	349	19	10164	1.2938	0.097868
-	LOC1053724	19	51549870	51599820	292	20	10164	1.2936	0.097895
-	MOCS1	6	39893705	39969551	252	38	10164	1.2936	0.097905
+	PTGER4	5	40644498	40751764	362	26	10164	1.293	0.098003
-	RERG	12	15097782	15256477	404	35	10164	1.2928	0.098037
+	ICAM4	19	10251967	10298584	119	25	10164	1.2928	0.098038
-	KLHL21	1	6580724	6637898	103	14	10164	1.2921	0.098164
+	HSPA1A	6	31780514	31827942	188	62	10164	1.292	0.098173
+	LDHA	11	18359389	18418218	167	16	10164	1.2914	0.098278
-	SLU7	5	160391641	160454161	141	21	10164	1.2914	0.098279
+	MTRNR2L5	10	55563990	55610728	76	6	10164	1.2911	0.09833
-	RHOG	11	3816978	3875983	129	20	10164	1.2907	0.098398
+	STXBP4	17	54933735	55174285	906	35	10164	1.2902	0.098489
-	KLHL22	22	20431519	20530883	27	4	10164	1.2901	0.098514
-	SHISA7	19	55418738	55477863	151	29	10164	1.2895	0.098603
+	FOXI2	10	127702274	127751186	119	7	10164	1.2895	0.098605
-	TRPM6	9	74712495	74923094	246	28	10164	1.2891	0.098677
+	PIAS4	19	3972598	4049386	129	11	10164	1.2887	0.098751
+	CPSF6	12	69204537	69284358	118	18	10164	1.288	0.09888
+	PSMD14	2	161273275	161421717	189	17	10164	1.2874	0.098981
+	PDE4DIP	1	148773167	149043019	4	2	10164	1.2865	0.099131
-	KRTAP26-1	21	30309131	30355289	249	17	10164	1.2865	0.099133
+	RXFP4	1	155906689	155952834	79	14	10164	1.2864	0.099156
+	MAP2K4	17	11985818	12153831	394	27	10164	1.286	0.099216
+	RNFT2	12	116703291	116863631	466	38	10164	1.2856	0.099288
+	STK35	20	2064799	2158555	251	12	10164	1.2853	0.099348
-	KRTAP21-2	21	30736951	30782202	75	4	10164	1.285	0.099402
-	LMTK3	19	48475271	48548905	123	18	10164	1.2848	0.099423
-	PIK3R3	1	46030140	46211495	489	37	10164	1.2844	0.099502
-	HLA-DMB	6	32924629	32976070	375	42	10164	1.284	0.099572
-	NMNAT2	1	183238237	183453499	721	50	10164	1.2831	0.099734
-	LOC1053721	18	49007679	49054771	108	28	10164	1.2803	0.10021
-	GEM	8	94239253	94297319	150	20	10164	1.2801	0.10026
+	CD33	19	51180537	51250019	186	35	10164	1.2798	0.10031
+	STK36	2	218637026	218712717	129	11	10164	1.2797	0.10032
+	SLC22A5	5	132334704	132405614	303	23	10164	1.2797	0.10033

STRAND	SYMBOL	CHR	START	STOP	NSNPS	NPARAM	N	ZSTAT	P
+	ZNF37A	10	38059336	38133352	230	14	10164	1.2795	0.10036
+	CELA2A	1	15421728	15482091	144	19	10164	1.2791	0.10043
-	TNFSF10	3	172495508	172558507	252	32	10164	1.2789	0.10047
+	NUTM1	15	34308251	34367737	150	6	10164	1.2789	0.10047
+	DIP2A	21	46423942	46594263	722	33	10164	1.2786	0.10053
-	DPM2	9	127925095	127973484	138	13	10164	1.2785	0.10054
-	ACBD6	1	180277385	180537967	377	12	10164	1.2785	0.10054
-	LRRTM2	5	138859346	138910368	26	6	10164	1.2783	0.10058
-	NINJ2	12	554296	698741	513	42	10164	1.2782	0.1006
-	NPC1L1	7	44502535	44576330	155	25	10164	1.2781	0.1006
+	NPEPL1	20	58654131	58725844	180	17	10164	1.2779	0.10065
+	ZNF92	7	65338799	65411135	182	12	10164	1.2778	0.10065
-	ACBD5	10	27185214	27277139	598	16	10164	1.2774	0.10073
+	FAM177B	1	222702216	222763727	135	12	10164	1.2772	0.10076
+	GLI2	2	120762291	121002653	125	17	10164	1.2771	0.10078
+	SNCAIP	5	122276349	122474099	365	27	10164	1.277	0.1008
-	BMP5	6	55743653	55910593	444	39	10164	1.2768	0.10083
+	HMP19	5	174010604	174119179	51	14	10164	1.2768	0.10084
+	ATP6V0A1	17	42423844	42532579	194	23	10164	1.2764	0.1009
-	IL36B	2	113012091	113087867	358	24	10164	1.2765	0.1009
-	C11orf95	11	63749892	63803641	38	18	10164	1.2762	0.10093
+	CPNE6	14	24035837	24088100	92	24	10164	1.2761	0.10096
+	GTF2H2C	5	69525143	69602903	6	2	10164	1.2755	0.10106
-	AHDC1	1	27524245	27639178	63	20	10164	1.2754	0.10109
-	S100B	21	46588618	46640122	254	28	10164	1.2753	0.10111
+	SRGAP1	12	63809512	64157863	545	62	10164	1.2751	0.10113
-	PSG3	19	42711642	42775516	33	10	10164	1.275	0.10115
+	DMRTA1	9	22411841	22462473	227	24	10164	1.275	0.10115
-	AGO2	8	140510156	140677326	408	37	10164	1.2748	0.10119
+	TRHDE	12	72237580	72675644	717	26	10164	1.2744	0.10126
+	IL1F10	2	113032970	113085850	275	27	10164	1.2744	0.10126
+	HIGD1C	12	50901223	50982571	306	22	10164	1.2742	0.10129
-	RAB20	13	110513066	110596737	414	51	10164	1.2738	0.10137
-	AURKA	20	56359389	56427295	101	22	10164	1.2736	0.1014
+	ABCF1	6	30536393	30601532	269	45	10164	1.2734	0.10144
+	PTTG1	5	160386807	160438744	123	17	10164	1.2734	0.10144
-	BLNK	10	96181699	96306576	264	25	10164	1.2729	0.10152
+	PIWIL2	8	22240297	22367568	124	6	10164	1.2719	0.10171
+	MEPE	4	87786398	87856817	292	45	10164	1.2717	0.10175
-	LOC1053771	3	75383739	75431371	53	6	10164	1.2713	0.10182
+	ADGRG5	16	57494333	57587210	225	24	10164	1.271	0.10186
+	PACSIN1	6	34431061	34546262	557	63	10164	1.2703	0.10198
-	GPR157	1	9093391	9164170	187	28	10164	1.2702	0.10202
-	UCMA	10	13211766	13269328	270	41	10164	1.2683	0.10235
-	MRPS10	6	42196801	42252907	2	1	10164	1.2679	0.10241
+	DYNLL2	17	58048419	58100257	38	3	10164	1.2677	0.10246
-	OGN	9	92373967	92439655	132	9	10164	1.2673	0.10252
+	TTL	2	112447166	112542770	128	17	10164	1.2672	0.10254
-	ZRANB3	2	135186969	135566322	666	15	10164	1.2668	0.10262
-	KIAA0907	1	155903043	155969442	109	15	10164	1.2667	0.10264
-	GOLGA1	9	124868294	124976114	219	24	10164	1.2665	0.10267
-	CSRNP2	12	51051205	51118671	170	18	10164	1.2663	0.1027
-	KIAA0753	17	6568151	6676744	325	18	10164	1.2655	0.10285
-	ZNF827	4	145747627	145973455	357	47	10164	1.2649	0.10295
+	PTPRA	20	2829195	3048669	625	28	10164	1.2642	0.10307
-	CABP5	19	48019383	48079054	240	24	10164	1.264	0.10311
+	SLC25A31	4	127695375	127784299	133	17	10164	1.264	0.10312
+	FFAR3	19	35323209	35370489	172	41	10164	1.2638	0.10315
+	NDUFS3	11	47544010	47594563	49	8	10164	1.2637	0.10317
+	PIKFYVE	2	208230753	208368751	294	13	10164	1.2635	0.10321
-	MMADHC	2	149559633	149622816	102	7	10164	1.2633	0.10323
+	SLC25A34	1	15700088	15751392	162	19	10164	1.263	0.1033
-	MBD3L3	19	7046205	7104615	112	14	10164	1.2616	0.10355

STRAND	SYMBOL	CHR	START	STOP	NSNPS	NPARAM	N	ZSTAT	P
-	KRAS	12	25194789	25285931	245	25	10164	1.2611	0.10363
-	HEYL	1	39613431	39674676	151	27	10164	1.2608	0.10369
-	TIGD6	5	149983123	150036167	124	11	10164	1.2607	0.10371
-	SERPINB1	6	2822332	2877049	229	25	10164	1.2607	0.10372
+	JRKL	11	96354994	96403563	128	18	10164	1.2606	0.10372
+	RPP14	3	58271245	58330193	158	5	10164	1.2599	0.10384
+	TRAK1	3	41978802	42235890	665	47	10164	1.2598	0.10388
-	RNH1	11	484512	542283	265	20	10164	1.2595	0.10393
-	OR9I1	11	58108500	58154444	188	25	10164	1.2594	0.10394
+	ECM1	1	150473011	150523789	160	15	10164	1.2593	0.10395
-	LOC1053739	2	110012300	110064293	2	1	10164	1.2594	0.10395
-	PBRM1	3	52535352	52720898	415	15	10164	1.2593	0.10396
+	MYBPC2	19	50397903	50476326	277	51	10164	1.2591	0.10399
-	BBS10	12	76334486	76383442	26	7	10164	1.2585	0.1041
-	C8orf82	8	144516219	144564074	30	5	10164	1.2582	0.10415
-	NIN	14	50709763	50866165	287	33	10164	1.2579	0.10421
-	TIMM10	11	57518463	57565759	141	20	10164	1.2578	0.10422
+	PHF13	1	6578696	6634033	95	13	10164	1.2578	0.10422
+	DTWD1	15	49585980	49664448	139	20	10164	1.2576	0.10427
-	C6orf132	6	42091119	42177977	139	16	10164	1.2571	0.10435
+	TMEM136	11	120289630	120343688	126	21	10164	1.2565	0.10446
+	SRFBP1	5	121926961	122038600	260	12	10164	1.2547	0.10479
+	MSMO1	4	165292666	165353164	142	21	10164	1.2542	0.10488
-	PAX3	2	222189887	222356263	363	33	10164	1.2541	0.1049
-	OR8B3	11	124386410	124434522	205	21	10164	1.254	0.10492
+	CTNNBL1	20	37658955	37882129	644	26	10164	1.2539	0.10495
-	PTAR1	9	69699522	69795044	201	26	10164	1.2535	0.10501
-	LOC1053741	3	150861019	150913949	63	14	10164	1.2532	0.10506
+	GRAMD3	5	126325096	126504364	471	82	10164	1.2527	0.10516
+	VSNL1	2	17505540	17666439	397	17	10164	1.2525	0.10519
+	TARS2	1	150452364	150517609	221	18	10164	1.2525	0.10519
+	POMP	13	28624004	28688957	175	18	10164	1.2523	0.10524
-	MCAT	22	43122206	43178397	239	49	10164	1.2522	0.10525
+	ETNK1	12	22590142	22700674	219	23	10164	1.2518	0.10532
+	RGS14	5	177322711	177382601	76	9	10164	1.2517	0.10534
+	MRPL41	9	137516857	137562555	63	14	10164	1.2514	0.1054
-	INPP5E	9	136418615	136474859	105	8	10164	1.2504	0.10557
-	JMJD4	1	227721189	227770499	202	31	10164	1.2499	0.10566
-	DEFA1B	8	6986766	7034202	50	8	10164	1.2495	0.10574
-	ZNF772	19	57459586	57512570	240	14	10164	1.2493	0.10578
-	KCNMB1	5	170368161	170424677	211	34	10164	1.2492	0.1058
+	CYP26C1	10	93026264	93078697	134	17	10164	1.2492	0.1058
+	FAM180B	11	47551678	47599194	39	8	10164	1.2491	0.10582
+	MYL12B	18	3227113	3288284	216	17	10164	1.2489	0.10584
-	FRG2B	10	133614796	133662886	61	7	10164	1.2489	0.10585
-	BPNT1	1	220047482	220124927	185	26	10164	1.2489	0.10586
+	LOC1053763	10	630959	676681	72	9	10164	1.2487	0.10588
-	SAMD9L	7	93120054	93183393	116	16	10164	1.2487	0.10589
+	UBAP1	9	34144005	34262523	280	28	10164	1.2486	0.10591
+	EMILIN2	18	2811231	2924092	186	35	10164	1.2484	0.10595
+	CSF3	17	39980361	40027813	181	13	10164	1.2482	0.10598
+	GNAS	20	58804740	58921195	189	29	10164	1.2482	0.10598
-	DNAJC30	7	73670918	73718451	70	5	10164	1.2481	0.10599
-	LOC729159	16	60348455	60394763	102	19	10164	1.2481	0.106
+	SNAP47	1	227699996	227791231	353	29	10164	1.2469	0.10622
+	NUB1	7	151306699	151388461	226	39	10164	1.2468	0.10623
-	PRF1	10	70587348	70637775	159	22	10164	1.2465	0.10629
+	CCNA1	13	36396030	36452882	66	5	10164	1.2465	0.10629
+	TUBB	6	30685201	30735426	320	26	10164	1.2461	0.10636
+	ZNF277	7	112171588	112353934	312	17	10164	1.2459	0.10639
-	CYP51A1	7	92102149	92169745	162	13	10164	1.2452	0.10653
-	ECM2	9	92483547	92571092	203	15	10164	1.2445	0.10666
+	UBE2E1	3	23770893	23901640	402	27	10164	1.2442	0.1067

STRAND	SYMBOL	CHR	START	STOP	NSNPS	NPARAM	N	ZSTAT	P
-	MAP6	11	75576914	75703730	359	28	10164	1.2436	0.10682
+	LNP1	3	100366193	100466326	220	28	10164	1.2435	0.10684
+	VPS45	1	150032293	150155327	56	27	10164	1.2434	0.10686
+	RASSF5	1	206472530	206599284	283	43	10164	1.2425	0.10703
+	LOC1053699	12	114370713	114417019	130	25	10164	1.2424	0.10705
+	ENGASE	17	79036523	79098603	169	25	10164	1.2419	0.10714
-	ABL2	1	179089327	179264693	481	27	10164	1.2413	0.10725
+	KCNA6	12	4771885	4861114	135	20	10164	1.241	0.1073
+	BHLHE40	3	4944412	4995181	90	7	10164	1.2406	0.10737
-	LMAN2	5	177321562	177386884	81	10	10164	1.2403	0.10744
+	SPPL2C	17	45809890	45857072	10	4	10164	1.24	0.10748
-	VGLL4	3	11546067	11755768	743	67	10164	1.24	0.10749
+	APOBEC3F	22	39005604	39065972	129	16	10164	1.2397	0.10754
-	ZNF521	18	25051924	25387250	638	59	10164	1.239	0.10768
-	RIT1	1	155887808	155946402	103	14	10164	1.2386	0.10774
+	DGCR8	22	20045232	20121877	269	29	10164	1.2385	0.10777
-	LOC1019293	7	65489303	65590350	99	13	10164	1.2384	0.10778
-	PKDREJ	22	46245663	46298322	95	3	10164	1.2378	0.1079
-	MSMB	10	46023305	46081269	229	23	10164	1.237	0.10804
+	DHX38	16	72058665	72122912	220	24	10164	1.2364	0.10815
+	AKAP9	7	91905867	92120673	489	16	10164	1.2362	0.10818
-	TMEM105	17	81295814	81365674	162	15	10164	1.236	0.10823
+	TMEM98	17	32892910	32951649	31	6	10164	1.2359	0.10824
-	CHIT1	1	203202823	203265099	286	37	10164	1.2356	0.1083
+	HPDL	1	45291873	45338675	145	26	10164	1.2356	0.1083
+	THSD4	15	71080628	71793383	1852	142	10164	1.2348	0.10846
-	ENPP2	8	119547077	119708404	406	49	10164	1.2342	0.10857
+	NTM	11	131335361	132346822	2562	209	10164	1.2342	0.10857
-	MFAP2	1	16964500	17016643	80	8	10164	1.2338	0.10865
+	ARID2	12	45694689	45918037	369	30	10164	1.2333	0.10873
-	VCP	9	35046068	35107742	182	28	10164	1.2329	0.1088
+	GAPVD1	9	125206663	125375011	371	26	10164	1.2327	0.10884
-	MYH7	14	23402738	23470686	156	18	10164	1.2327	0.10885
-	CSTF2T	10	51685486	51734595	142	9	10164	1.2313	0.10911
-	FAM166A	9	137233583	137286193	151	14	10164	1.2312	0.10913
+	PRAMEF17	1	13354632	13402629	193	10	10164	1.231	0.10916
-	GRIN2B	12	13527337	14017002	1307	104	10164	1.2308	0.1092
+	ZNF304	19	57316245	57369898	218	20	10164	1.2306	0.10923
+	CCT7	2	73198425	73263022	89	7	10164	1.2306	0.10924
+	ZC3H15	2	186451158	186519361	160	15	10164	1.2304	0.10928
-	CYP4F2	19	15868024	15933120	285	14	10164	1.23	0.10934
+	UBXN4	2	135706619	135795063	268	30	10164	1.2301	0.10934
-	PAMR1	11	35421827	35565300	554	67	10164	1.2292	0.1095
-	ASPDH	19	50501600	50549690	156	36	10164	1.2291	0.10953
+	KIAA1328	18	36794109	37257316	570	12	10164	1.2289	0.10955
-	SMIM15	5	61147709	61197475	109	13	10164	1.2289	0.10956
+	C12orf75	12	105295636	105381518	311	31	10164	1.2283	0.10967
-	SRA1	5	140540067	140593093	69	15	10164	1.227	0.10992
+	ACPT	19	50755415	50805224	241	39	10164	1.2267	0.10996
-	DIRAS1	19	2704567	2756392	100	6	10164	1.2266	0.10999
+	PTPMT1	11	47530430	47583461	53	9	10164	1.2263	0.11004
+	POLR3GL	1	145924007	145988848	5	1	10164	1.2255	0.1102
-	ATP6V1G3	1	198513222	198575945	139	18	10164	1.2254	0.1102
-	LIX1L	1	145923424	145993019	5	1	10164	1.2255	0.1102
-	UBE2L6	11	57541655	57603330	189	23	10164	1.2252	0.11025
-	FAM134B	5	16462816	16652058	519	55	10164	1.2251	0.11027
+	PIGW	17	36500002	36549368	48	10	10164	1.225	0.1103
-	EQTN	9	27274654	27332139	205	32	10164	1.2239	0.1105
+	KLHDC1	14	49658105	49763152	176	15	10164	1.2237	0.11054
-	ABHD3	18	21640897	21739805	139	14	10164	1.2232	0.11063
+	CSTF1	20	56357371	56414526	99	20	10164	1.2231	0.11064
+	NUDT16	3	131346671	131398830	177	15	10164	1.2229	0.11069
+	LOC1010599	11	493484	542263	218	18	10164	1.2228	0.11071

STRAND	SYMBOL	CHR	START	STOP	NSNPS	NPARAM	N	ZSTAT	P
+	CSNK1G3	5	123477099	123627045	301	20	10164	1.2222	0.11081
-	NFE2	12	54282107	54336037	76	10	10164	1.2222	0.11081
-	PRAMEF19	1	13358431	13406947	188	11	10164	1.2215	0.11095
+	GRM1	6	145929769	146447598	1059	43	10164	1.2212	0.111
+	PCP4	21	39832421	39939397	455	50	10164	1.2208	0.11108
+	CHRM5	15	33933245	34075094	369	24	10164	1.2206	0.11113
+	ERBB2IP	5	65891465	66091023	239	18	10164	1.2204	0.11116
+	SYT9	11	7203796	7479045	938	94	10164	1.2203	0.11117
+	REG1A	2	79085458	79133419	152	27	10164	1.2202	0.1112
-	SERTAD2	2	64621621	64786091	325	28	10164	1.2199	0.11125
-	MST1	3	49673947	49724528	79	15	10164	1.2196	0.1113
-	SPATA6L	9	4585837	4701801	545	50	10164	1.2194	0.11134
-	PRDM15	21	41788225	41914482	601	47	10164	1.2185	0.11151
+	WDR55	5	140629799	140682345	89	7	10164	1.2182	0.11158
+	KLHL10	17	41802490	41858347	135	8	10164	1.2178	0.11165
+	NELFB	9	137220307	137283548	173	18	10164	1.2173	0.11174
+	B4GALT2	1	43944202	44001171	88	13	10164	1.2173	0.11175
+	HADHB	2	26209748	26300465	156	7	10164	1.217	0.1118
-	SPAG11B	8	7437754	7498670	1	1	10164	1.2169	0.11181
-	DEFB103B	8	7418888	7465348	1	1	10164	1.2169	0.11181
-	DEFB4B	8	7404863	7451832	1	1	10164	1.2169	0.11181
+	EPC2	2	148609811	148798491	478	21	10164	1.2168	0.11185
+	ANKRD66	6	46711917	46769218	103	17	10164	1.2161	0.11197
+	SLC39A14	8	22332249	22444129	42	3	10164	1.2154	0.11211
+	TANK	2	161101927	161246176	235	14	10164	1.2151	0.11216
+	RP1	8	54581067	54640834	152	22	10164	1.215	0.11219
-	FAM129B	9	127495338	127614007	436	37	10164	1.2147	0.11225
+	BCS1L	2	218624656	218673443	100	7	10164	1.2143	0.11231
+	DLL4	15	40894333	40949060	105	21	10164	1.2142	0.11233
+	FBXW10	17	18705223	18789349	36	9	10164	1.2137	0.11244
-	TMEM221	19	17425509	17483660	126	13	10164	1.2134	0.11249
+	SLC2A10	20	46673347	46746347	108	7	10164	1.2133	0.11251
+	HIST1H4I	6	27104309	27149678	105	33	10164	1.2132	0.11254
+	APOBR	16	28459649	28508970	49	9	10164	1.2127	0.11262
+	BBX	3	107487936	107821329	588	37	10164	1.2124	0.11269
-	JAZF1	7	27820573	28215818	1351	111	10164	1.2118	0.11279
+	C3orf80	3	160188784	160300143	199	36	10164	1.2115	0.11284
+	ACSM2A	16	20416526	20497669	203	13	10164	1.2112	0.11291
+	ZNF557	19	7034460	7097968	92	10	10164	1.2097	0.1132
-	ADGRB2	1	31717105	31799063	134	10	10164	1.2097	0.11321
-	MED31	17	6633313	6686634	183	21	10164	1.2094	0.11326
+	MOBP	3	39432573	39539497	488	18	10164	1.2085	0.11343
-	RBBP5	1	205076142	205157022	259	25	10164	1.2085	0.11343
-	APH1A	1	150255399	150304209	103	12	10164	1.2083	0.11346
+	FLVCR2	14	75543597	75658169	313	39	10164	1.2082	0.11348
-	MDC1	6	30689807	30752966	412	30	10164	1.208	0.11352
-	SNX16	8	81789583	81877325	308	12	10164	1.2079	0.11355
-	CST3	20	23617897	23673048	37	11	10164	1.2076	0.1136
-	GTDC1	2	143935787	144367534	442	29	10164	1.2075	0.11361
-	PIP5KL1	9	127911529	127965797	135	13	10164	1.2074	0.11364
-	AIDA	1	222658013	222747901	199	14	10164	1.207	0.11371
+	WHSC1	4	1836396	1992207	134	17	10164	1.2069	0.11374
-	HAVCR2	5	157075832	157144237	201	18	10164	1.2065	0.11381
+	MSRA	8	10019224	10438891	1935	109	10164	1.2061	0.11389
+	PARVA	11	12341441	12545356	482	78	10164	1.206	0.1139
+	ZCCHC17	1	31261982	31375722	241	13	10164	1.2058	0.11395
-	TRAIP	3	49818595	49891564	121	15	10164	1.2055	0.114
+	IFRD1	7	112388144	112487203	263	24	10164	1.2053	0.11404
-	RRAS	19	49625295	49675143	101	18	10164	1.2052	0.11405
-	TNFAIP8L3	15	51046596	51140276	229	26	10164	1.2051	0.11407
-	KLHL38	8	123635505	123688866	198	28	10164	1.2044	0.11421
-	KLHL12	1	202881096	202963644	265	28	10164	1.2042	0.11426
+	MARCH6	5	10318639	10450388	92	2	10164	1.204	0.1143

STRAND	SYMBOL	CHR	START	STOP	NSNPS	NPARAM	N	ZSTAT	P
+	FOXI1	5	170070897	170119725	201	23	10164	1.2037	0.11435
-	GSK3A	19	42220186	42277584	69	5	10164	1.203	0.11448
+	GPR15	3	98496899	98543150	122	25	10164	1.2019	0.11469
-	SLC25A22	11	780475	833269	149	23	10164	1.2017	0.11473
-	SH3GLB2	9	128997792	129063353	56	12	10164	1.2017	0.11475
+	LTBR	12	6340368	6401571	188	49	10164	1.2012	0.11485
-	ATG12	5	115818196	115876851	79	9	10164	1.2011	0.11485
-	KLRG2	7	139416933	139518711	104	9	10164	1.2011	0.11485
+	SPRTN	1	231302936	231380551	161	11	10164	1.2007	0.11494
+	SGPP2	2	222389052	222571842	384	54	10164	1.1998	0.1151
-	PRAMEF10	1	12882896	12933270	13	4	10164	1.1998	0.11511
-	CLEC4C	12	7719415	7784473	212	26	10164	1.1997	0.11512
+	NFAT5	16	69530966	69714666	361	19	10164	1.1996	0.11515
-	TXNL4B	16	72074857	72129316	194	21	10164	1.1992	0.11523
-	LOC1027244	5	157351616	157495056	473	25	10164	1.1991	0.11525
-	SAMD10	20	63964113	64015986	93	7	10164	1.1988	0.1153
-	APMAP	20	24952925	25027974	208	12	10164	1.1987	0.11533
-	XRCC1	19	43533312	43610578	239	38	10164	1.1983	0.11539
+	CENPP	9	92290457	92625164	694	18	10164	1.1979	0.11547
-	UBE2S	19	55391282	55443146	154	29	10164	1.1975	0.11556
-	SDPR	2	191824305	191882280	18	4	10164	1.1969	0.11567
-	ASTN1	1	176847305	177199904	592	41	10164	1.1968	0.1157
-	UCHL5	1	193002366	193095107	64	7	10164	1.1967	0.11572
+	RPS6KB2	11	67393460	67445408	110	19	10164	1.1965	0.11576
+	PRAMEF7	1	12881647	12930480	14	4	10164	1.1964	0.11578
-	USP4	3	49267144	49375103	196	21	10164	1.1961	0.11584
+	FGD4	12	32364541	32656050	1258	66	10164	1.1961	0.11584
+	KIR2DL3	19	54703509	54763052	118	9	10164	1.1953	0.11598
-	SCGB1D4	11	62286282	62334064	200	15	10164	1.1953	0.11598
+	AP3M2	8	42117946	42181183	122	16	10164	1.1952	0.116
+	GRIK2	6	101358708	102080083	1893	116	10164	1.1951	0.11602
+	PLEKHM2	1	15649267	15744769	279	21	10164	1.1951	0.11602
-	NKX6-2	10	132774816	132821033	140	23	10164	1.195	0.11603
-	RBM8A	1	145911556	145962536	5	1	10164	1.195	0.11605
-	POU2F2	19	42076110	42167473	89	9	10164	1.1949	0.11606
-	CTXN1	19	7914496	7961166	127	12	10164	1.1944	0.11616
+	ZNF430	19	20985620	21070055	212	15	10164	1.1941	0.11623
-	USP31	16	23051406	23184270	387	18	10164	1.1939	0.11627
+	SELT	3	150568279	150640447	136	13	10164	1.1937	0.1163
+	COLGALT1	19	17520702	17593162	181	20	10164	1.1935	0.11633
+	TRIP6	7	100831221	100883455	122	21	10164	1.1934	0.11635
+	ITGAV	2	186555063	186690902	275	26	10164	1.1934	0.11636
-	LOC1053739	2	237475700	237522338	264	31	10164	1.1931	0.11642
+	APAF1	12	98610228	98745433	354	28	10164	1.193	0.11643
-	MYO19	17	36485755	36570457	67	12	10164	1.1922	0.11658
-	TTC5	14	20279131	20340994	175	18	10164	1.1921	0.1166
-	BNC1	15	83245884	83319716	117	20	10164	1.1921	0.11661
-	ZNF569	19	37400876	37504064	268	34	10164	1.1917	0.11668
-	ELAVL2	9	23680104	23885706	657	57	10164	1.1917	0.1167
-	AP3M1	10	74110257	74186068	183	15	10164	1.191	0.11683
-	NEB	2	151475339	151769487	696	41	10164	1.1909	0.11684
-	ERF	19	42237561	42290164	61	3	10164	1.1909	0.11685
+	ZNF761	19	53396974	53468261	119	16	10164	1.1909	0.11685
-	MMP1	11	102779910	102833235	271	46	10164	1.1907	0.11688
-	GFRA2	8	21682014	21824294	339	44	10164	1.1907	0.11689
-	CERS3	15	100390395	100579786	976	43	10164	1.1906	0.1169
-	IQCG	3	197877208	197995015	278	25	10164	1.1906	0.11691
-	LRRN2	1	204607170	204720733	332	40	10164	1.1905	0.11692
-	PPP1R15B	1	204390275	204446817	120	15	10164	1.19	0.11702
-	LSM2	6	31787392	31841984	238	70	10164	1.1899	0.11705
+	KIF2B	17	53787878	53835212	193	19	10164	1.1899	0.11705
-	NRM	6	30678047	30726420	293	23	10164	1.1892	0.11717
-	PLA2G2D	1	20102650	20154566	97	28	10164	1.1886	0.1173

STRAND	SYMBOL	CHR	START	STOP	NSNPS	NPARAM	N	ZSTAT	P
+	LYZ	12	69313354	69364233	101	11	10164	1.1876	0.1175
+	LOC1006528	22	23900477	23952861	196	19	10164	1.1875	0.11752
+	LOC1053692	5	10427873	10474841	51	2	10164	1.1874	0.11753
+	CD36	7	80567188	80689277	116	9	10164	1.1873	0.11755
+	TROVE2	1	193024422	193101777	58	7	10164	1.1869	0.11764
+	TCF7L1	2	85098460	85320388	676	58	10164	1.1868	0.11766
+	GOLGA6D	15	75247841	75305807	49	24	10164	1.1859	0.11783
+	PPP1R32	11	61446113	61500928	95	9	10164	1.1854	0.11793
-	FAM171A2	17	44343733	44398867	58	11	10164	1.1854	0.11793
-	PWWP2A	5	160033562	160155808	193	16	10164	1.1851	0.118
-	PRADC1	2	73218006	73268228	53	6	10164	1.1849	0.11802
-	KIF6	6	39325100	39760472	1038	53	10164	1.185	0.11802
-	KRTAP21-3	21	30708525	30753777	199	6	10164	1.1848	0.11805
+	KHNYN	14	24388171	24451342	236	25	10164	1.1847	0.11806
+	CDC5L	6	44352514	44460424	251	33	10164	1.1845	0.11811
+	UVRAG	11	75780167	76154238	874	52	10164	1.1842	0.11816
-	SNAPC4	9	136365571	136435208	206	13	10164	1.1839	0.11824
+	LOC1053739	2	236518699	236579257	82	6	10164	1.1834	0.11833
-	ME1	6	83200389	83466741	761	28	10164	1.1828	0.11844
+	LOC1027234	10	1003454	1050858	167	26	10164	1.1827	0.11846
-	GRM8	7	126428598	127288294	2638	92	10164	1.1814	0.11873
+	RPS19	19	41824918	41881416	88	6	10164	1.1812	0.11876
-	PPP2R3C	14	35075472	35157501	222	21	10164	1.1812	0.11876
+	RNF17	13	24712672	24899262	693	44	10164	1.1812	0.11876
+	GJB5	1	34720046	34768512	54	18	10164	1.1811	0.11879
+	GTSE1	22	46261014	46343699	141	4	10164	1.1807	0.11886
-	HADHA	2	26180635	26279726	171	7	10164	1.1806	0.11889
+	MED21	12	26987522	27040673	156	27	10164	1.1803	0.11894
+	LRP3	19	33159693	33218867	191	31	10164	1.1803	0.11895
-	LOC1019277	2	119700868	119795452	141	15	10164	1.1796	0.11908
+	SLMO1	18	12372896	12442238	237	34	10164	1.1795	0.1191
-	SLC43A3	11	57396954	57462580	136	26	10164	1.1794	0.11911
+	NCR3LG1	11	17316732	17391661	196	20	10164	1.1788	0.11923
-	APPL2	12	105163297	105271230	414	25	10164	1.1788	0.11924
+	SPIB	19	50383938	50441053	217	33	10164	1.178	0.11939
-	CARD18	11	105127721	105174734	187	12	10164	1.178	0.1194
+	ZNF175	19	51536278	51602510	367	21	10164	1.1778	0.11944
-	ELOVL7	5	60741789	60879283	326	29	10164	1.1776	0.11948
-	POC5	5	75664198	75752488	363	22	10164	1.1774	0.11953
+	LOC1053698	12	80901093	80947633	124	20	10164	1.1764	0.11971
-	HFM1	1	91250766	91443007	492	38	10164	1.1761	0.11977
+	TXNDC17	17	6605902	6654541	145	11	10164	1.1761	0.11978
+	ZNF7	8	144792518	144857508	208	15	10164	1.176	0.1198
+	NOL12	22	37651337	37703478	171	23	10164	1.1757	0.11986
-	LCTL	15	66537468	66600979	273	43	10164	1.1754	0.11992
-	ACTL9	19	8687397	8733830	73	9	10164	1.175	0.11999
-	KRTAP25-1	21	30279145	30324514	221	22	10164	1.1748	0.12004
+	SLC5A9	1	48187270	48258644	231	24	10164	1.1743	0.12013
-	SLC36A3	5	151266365	151347981	319	22	10164	1.1743	0.12013
+	MCTP2	15	94196538	94493952	410	41	10164	1.1734	0.12032
+	HP	16	72019609	72071056	151	22	10164	1.1734	0.12033
-	MBD3L4	19	7027744	7075826	37	6	10164	1.1731	0.12038
+	PSD2	5	139686903	139854466	220	27	10164	1.173	0.12041
+	LY6G6F	6	31671907	31720595	232	52	10164	1.1729	0.12043
+	PSMA3	14	58209805	58282009	109	14	10164	1.1727	0.12045
-	TBC1D4	13	75274664	75517646	639	59	10164	1.1725	0.12049
+	LOC1053739	2	120293666	120356060	125	32	10164	1.1724	0.12051
+	COL6A6	3	130482177	130688135	619	64	10164	1.1724	0.12052
+	ATP5G1	17	48857786	48905871	147	17	10164	1.1722	0.12057
+	GPR142	17	74332407	74383008	125	25	10164	1.1721	0.12057
-	MUC6	11	1002824	1071706	140	20	10164	1.1718	0.12065
-	C2orf80	2	208155347	208225258	198	24	10164	1.1715	0.12069
+	ITFG2	12	2777621	2846956	134	12	10164	1.1714	0.12073

STRAND	SYMBOL	CHR	START	STOP	NSNPS	NPARAM	N	ZSTAT	P
+	KDM8	16	27168486	27232305	73	12	10164	1.1711	0.12079
+	C15orf48	15	45395529	45443449	190	16	10164	1.1708	0.12085
+	C15orf54	15	39215669	39264845	136	30	10164	1.1707	0.12085
+	C10orf91	10	132410210	132458321	55	10	10164	1.1701	0.12098
-	RAB5C	17	42114976	42190044	152	17	10164	1.17	0.121
-	PEX2	8	76970258	77036044	107	12	10164	1.1698	0.12105
-	TTC23	15	99126323	99286226	358	22	10164	1.1693	0.12115
+	HSPA1B	6	31792735	31840254	216	65	10164	1.1687	0.12127
+	SPACA7	13	112340881	112444695	417	19	10164	1.1684	0.12132
-	GOLGA6L2	15	23429386	23482243	107	6	10164	1.1684	0.12132
-	INPP4B	4	142018028	142881535	2256	65	10164	1.1683	0.12134
-	RNF25	2	218653864	218707058	93	9	10164	1.168	0.12139
-	LHCGR	2	48676774	48790741	397	44	10164	1.1675	0.1215
+	OR6C75	12	55330111	55376049	176	10	10164	1.1672	0.12156
+	HIST1H1E	6	26121331	26167115	216	39	10164	1.1671	0.12158
-	OR10A3	11	7928576	7974520	156	19	10164	1.167	0.1216
-	GFPT1	2	69309769	69422254	302	14	10164	1.1666	0.12169
+	C1orf228	1	44639694	44735591	252	40	10164	1.1665	0.12171
-	SIGIRR	11	395716	452454	101	15	10164	1.1662	0.12176
-	ZNF70	22	23731584	23786092	188	26	10164	1.1658	0.12184
-	EXOC8	1	231322731	231372872	100	6	10164	1.1658	0.12184
-	STEAP1B	7	22409444	22535282	388	38	10164	1.1658	0.12184
-	LOC1053691	16	15126205	15189766	19	5	10164	1.1654	0.12192
-	PRB3	12	11255914	11304707	23	6	10164	1.1652	0.12197
-	RHOV	15	40862214	40909289	144	16	10164	1.1652	0.12197
-	EPRS	1	219958600	220081658	413	21	10164	1.1641	0.12219
+	CDC73	1	193086958	193264815	180	10	10164	1.1637	0.12226
-	NTHL1	16	2029815	2082866	59	21	10164	1.1637	0.12228
+	GAB1	4	143301762	143484565	261	23	10164	1.1636	0.12229
+	ZNF570	19	37432465	37495358	175	28	10164	1.1635	0.12231
+	ANKHD1-EIF	5	140366814	140559578	183	26	10164	1.1633	0.12235
+	HIST1H2AG	6	27098038	27143535	97	28	10164	1.1632	0.12238
-	CASC1	12	25097057	25230162	384	17	10164	1.1628	0.12246
+	ZMIZ2	7	44713547	44779881	118	19	10164	1.1628	0.12246
+	DAPP1	4	99781824	99882249	228	24	10164	1.1625	0.12252
+	ZNF587B	19	57795294	57856238	138	13	10164	1.1623	0.12256
+	LOC113230	19	14038009	14085062	59	8	10164	1.1619	0.12264
-	TRAF6	11	36473767	36545313	195	34	10164	1.1617	0.12268
-	CLN3	16	28456653	28527302	77	9	10164	1.1616	0.1227
+	NAA20	20	19982290	20043629	142	21	10164	1.1612	0.12278
-	PPIL4	6	149494495	149581102	137	15	10164	1.161	0.12282
+	MRAP2	6	83989786	84156282	334	32	10164	1.1604	0.12294
+	ATP6V0B	1	43939648	43988300	82	12	10164	1.1599	0.12304
+	PTPRG	3	61526569	62307609	2552	172	10164	1.1598	0.12306
+	PHYHD1	9	128885895	128952041	127	18	10164	1.1598	0.12306
+	ABCD3	1	94383086	94528663	217	35	10164	1.1597	0.12309
+	ROPN1L	5	10406862	10492298	64	2	10164	1.1585	0.12332
-	SLC12A6	15	34219996	34373064	313	8	10164	1.1584	0.12336
+	TPI1	12	6832420	6880946	31	7	10164	1.1583	0.12338
-	PRR14L	22	31671347	31785173	313	25	10164	1.1579	0.12345
+	MAP3K4	6	160956727	161127385	462	42	10164	1.1569	0.12366
-	SFMBT1	3	52889982	53081655	587	29	10164	1.1565	0.12373
-	ZNF44	19	12237244	12329899	221	8	10164	1.1558	0.12388
+	CDK5RAP3	17	47932904	47991786	185	25	10164	1.1546	0.12412
+	AP3S1	5	115806922	115924081	181	20	10164	1.1543	0.12418
+	C2orf49	2	105302359	105369940	235	25	10164	1.1542	0.12421
+	RTP3	3	46462995	46510949	146	21	10164	1.1542	0.12421
-	RANBP9	6	13611498	13746580	238	29	10164	1.154	0.12424
+	FAM53C	5	138302535	138359729	101	11	10164	1.154	0.12425
+	RFC3	13	33783049	33983946	611	31	10164	1.1537	0.1243
+	DUOXA2	15	45079325	45128106	73	8	10164	1.1537	0.12431
-	CAMSAP1	9	135798487	135942251	161	13	10164	1.1535	0.12436
+	REP15	12	27661495	27707633	108	23	10164	1.1533	0.12439

STRAND	SYMBOL	CHR	START	STOP	NSNPS	NPARAM	N	ZSTAT	P
-	PTPRB	12	70506850	70672440	497	61	10164	1.153	0.12446
+	SLC26A7	8	91174494	91408155	423	36	10164	1.153	0.12446
+	VWA2	10	114204137	114304500	193	18	10164	1.1526	0.12453
+	EGFR	7	54984032	55217338	672	74	10164	1.1525	0.12456
-	TSKS	19	49729753	49798328	139	28	10164	1.1525	0.12457
+	IL17C	16	88603593	88650474	115	19	10164	1.1517	0.12472
-	BRD9	5	853735	927824	160	12	10164	1.1517	0.12472
+	TDRD7	9	97376989	97506125	368	22	10164	1.1516	0.12473
-	ZDHHC11	5	785605	893973	322	21	10164	1.1516	0.12475
-	MORC2	22	30915138	31003497	189	27	10164	1.1506	0.12494
+	CGN	1	151475877	151548692	138	20	10164	1.1505	0.12497
-	OLA1	2	174062447	174283637	539	17	10164	1.1498	0.12512
+	DTL	1	212000553	212115006	277	21	10164	1.1491	0.12525
-	GFOD1	6	13347830	13522637	435	38	10164	1.1491	0.12525
+	RTP1	3	187162486	187211465	146	25	10164	1.1486	0.12535
-	ZBTB22	6	33304405	33352942	188	35	10164	1.1486	0.12537
+	OR8S1	12	48490632	48538103	216	19	10164	1.148	0.12549
-	SLC36A2	5	151304978	151382590	307	26	10164	1.1466	0.12577
-	FNDC9	5	157331599	157380721	163	13	10164	1.1463	0.12583
+	TMEM8B	9	35794225	35864847	153	24	10164	1.1461	0.12588
+	WASF3	13	26522703	26698945	460	21	10164	1.1457	0.12597
-	TRIM50	7	73302472	73363279	22	7	10164	1.1456	0.12597
+	RBM5	3	50053908	50128964	153	16	10164	1.1453	0.12604
+	PHF14	7	10922892	11179828	677	27	10164	1.1447	0.12616
-	MYO1F	19	8510790	8612447	54	3	10164	1.1446	0.12619
-	BDH1	3	197499783	197608323	333	32	10164	1.1445	0.1262
-	DAXX	6	33308558	33358016	200	39	10164	1.1445	0.1262
+	ZFP36	19	39371847	39419412	37	9	10164	1.1445	0.12621
-	KIAA1161	9	34356666	34411896	136	27	10164	1.1443	0.12624
+	WNT2B	1	112431541	112531288	182	21	10164	1.144	0.12631
+	C4BPB	1	207053850	207109993	93	14	10164	1.1433	0.12645
-	PLA2G3	22	31124807	31175757	151	35	10164	1.1425	0.12662
-	SNRNP40	1	31249568	31331797	206	12	10164	1.1422	0.12668
-	NPIPB6	16	28332512	28399929	12	3	10164	1.1419	0.12675
+	LRRTM3	10	66891034	67111551	594	41	10164	1.1416	0.12681
+	PRPF6	20	63946069	64043100	172	13	10164	1.1414	0.12684
-	LOC1053768	1	20153076	20198980	62	9	10164	1.1414	0.12685
-	IRX4	5	1867427	1922179	199	31	10164	1.1411	0.12692
-	OR7A17	19	14870426	14916355	198	26	10164	1.1402	0.12711
+	SNRNP25	16	18829	67669	101	9	10164	1.14	0.12714
-	CLCF1	11	67354168	67409177	116	19	10164	1.1393	0.12728
-	PRC1	15	90956038	91029651	240	22	10164	1.1392	0.12732
-	IFNLR1	1	24144157	24222275	278	47	10164	1.1389	0.12737
+	SRRT	7	100839971	100898664	139	20	10164	1.1388	0.12738
-	C9orf92	9	16193935	16311313	563	76	10164	1.1385	0.12745
+	EIF3F	11	7952320	8006171	119	20	10164	1.1385	0.12746
+	TRIOBP	22	37661988	37786556	307	28	10164	1.1384	0.12749
+	PCDHB8	5	141142790	141190500	144	14	10164	1.1381	0.12753
-	LOC1053693	11	63826865	63874417	84	10	10164	1.1381	0.12754
+	LOC1053708	15	51059593	51106596	119	15	10164	1.1378	0.1276
-	VN1R1	19	57445174	57491739	186	13	10164	1.1371	0.12775
-	NSMCE4A	10	121947088	122010228	269	26	10164	1.1367	0.12782
-	NSUN5	7	73292516	73343867	24	7	10164	1.1366	0.12784
-	CINP	14	102338282	102397916	149	18	10164	1.1366	0.12785
-	DDX6	11	118737763	118826263	230	20	10164	1.1363	0.12791
+	STIP1	11	64150272	64214548	183	30	10164	1.1361	0.12796
-	SUCLG2	3	67350460	67689614	1358	69	10164	1.136	0.12797
-	CEP170	1	243114428	243290406	234	22	10164	1.136	0.12797
-	PAX4	7	127600292	127653192	140	25	10164	1.1357	0.12804
+	APOM	6	31617410	31668210	288	55	10164	1.1357	0.12805
-	ZBED8	5	160383148	160435097	119	16	10164	1.1355	0.12808
+	LOC1027243	12	24867309	24914261	125	14	10164	1.1354	0.12811
+	ANKHD1	5	140366814	140549856	170	25	10164	1.1351	0.12816

STRAND	SYMBOL	CHR	START	STOP	NSNPS	NPARAM	N	ZSTAT	P
-	USP41	22	20353284	20425758	80	14	10164	1.1349	0.1282
-	TMEM30B	14	61267370	61316812	124	20	10164	1.1348	0.12823
+	TTC1	5	159974100	160075545	162	11	10164	1.1345	0.12829
-	ZNF692	1	248840004	248895798	61	8	10164	1.1336	0.12847
+	PXK	3	58297890	58436127	311	11	10164	1.1335	0.12849
-	TAPBP	6	33289694	33349387	217	41	10164	1.1331	0.12859
-	PALM3	19	14043367	14097111	48	7	10164	1.1326	0.1287
+	BRSK2	11	1354899	1472689	576	58	10164	1.1321	0.1288
+	NXPE2	11	114613490	114717117	340	20	10164	1.132	0.12881
+	ATP5S	14	50277329	50336715	127	5	10164	1.1318	0.12886
+	ATP2C1	3	130815483	131026712	418	40	10164	1.1317	0.12887
-	HIST1H3B	6	26021589	26067060	184	36	10164	1.1315	0.12891
+	LOC1010600	2	90324288	90372907	4	2	10164	1.1316	0.12891
+	NANOGNB	12	7730216	7784121	139	18	10164	1.1314	0.12893
-	E2F6	2	11434375	11501177	103	7	10164	1.1314	0.12894
+	SOX9	17	72086020	72136420	134	18	10164	1.1314	0.12894
+	CCT5	5	10215170	10276389	256	16	10164	1.1314	0.12895
+	PELO	5	52752940	52812617	259	30	10164	1.1307	0.1291
+	GADD45G	9	89570012	89616555	195	19	10164	1.1306	0.12911
+	ATP6V1C1	8	102986020	103083057	334	26	10164	1.1303	0.12918
+	GPR88	1	100503172	100552027	117	17	10164	1.1303	0.12919
-	GLRX2	1	193086465	193141549	54	4	10164	1.1299	0.12925
+	SLC22A13	3	38230807	38288315	74	15	10164	1.1299	0.12926
-	TIMM44	19	7916718	7978823	168	14	10164	1.1297	0.1293
+	MRPS21	1	150258842	150318979	155	14	10164	1.1294	0.12937
+	GAREML	2	26138091	26212529	137	10	10164	1.1292	0.12941
-	TSSK1B	5	113422554	113470031	202	18	10164	1.129	0.12946
-	RABEP2	16	28894421	28960211	72	13	10164	1.1287	0.1295
+	PPFIA4	1	202991521	203088736	320	49	10164	1.1287	0.12952
+	TBKBP1	17	47659065	47722063	111	18	10164	1.1286	0.12953
+	DDIAS	11	82866695	82944659	221	24	10164	1.1282	0.12963
+	IGSF5	21	39690838	39812096	531	27	10164	1.1278	0.12971
-	NHLRC1	6	18110487	18157620	21	5	10164	1.1278	0.12971
-	PRAMEF4	1	12869212	12921201	19	5	10164	1.1276	0.12975
-	RHCG	15	89461407	89531613	136	12	10164	1.1272	0.12982
-	CHEK2	22	28677743	28776905	332	40	10164	1.1271	0.12984
-	RFC1	4	39277449	39401381	217	15	10164	1.1266	0.12995
+	SLC32A1	20	38689462	38739372	60	5	10164	1.1258	0.13012
-	LYPD4	19	41823686	41879862	92	6	10164	1.1251	0.13026
+	AP2A2	11	890809	1022245	319	10	10164	1.1248	0.13034
+	TMEM108	3	133003288	133407775	1276	48	10164	1.1247	0.13036
+	FHDC1	4	152876570	152989696	193	22	10164	1.1246	0.13038
+	TRIM49C	11	89995149	90052029	12	6	10164	1.1244	0.13041
+	NRGN	11	124704933	124757206	112	21	10164	1.1244	0.13043
+	MRPL23	11	1912272	2024017	144	31	10164	1.124	0.13051
+	NDFIP1	5	142073759	142164443	315	19	10164	1.1237	0.13056
+	MALSU1	7	23264321	23319561	119	12	10164	1.1237	0.13057
-	TGFBRAP1	2	105239404	105364714	413	35	10164	1.1236	0.13059
-	LRRC24	8	144512377	144562032	33	5	10164	1.1231	0.1307
-	MGAT4B	5	179787597	179841952	154	32	10164	1.1223	0.13087
-	PRB1	12	11341823	11390590	95	14	10164	1.1219	0.13094
-	DYM	18	49031474	49495709	1359	31	10164	1.1219	0.13095
+	ZNF773	19	57464854	57528437	322	11	10164	1.1216	0.13101
-	LOC1053781	14	60565033	60678945	200	16	10164	1.1206	0.13123
+	BTRC	10	101319033	101567321	608	24	10164	1.1204	0.13127
+	DNAJB5	9	34954369	35008433	146	36	10164	1.1195	0.13146
-	ZBTB2	6	151354115	151426700	268	39	10164	1.1194	0.13148
+	ZNF540	19	37516371	37624179	279	34	10164	1.1194	0.13148
+	PAX1	20	21670659	21728486	91	10	10164	1.1194	0.13149
-	NRL	14	24068693	24149802	101	16	10164	1.1192	0.13152
+	ZNF169	9	94224266	94313008	309	32	10164	1.1193	0.13152
+	RAB22A	20	58274715	58377507	168	7	10164	1.1188	0.13161
+	PTBP1	19	762392	822327	101	18	10164	1.1187	0.13164

STRAND	SYMBOL	CHR	START	STOP	NSNPS	NPARAM	N	ZSTAT	P
+	C6orf48	6	31799915	31849766	227	67	10164	1.1187	0.13164
-	ZNF883	9	112987120	113047192	103	17	10164	1.1186	0.13166
+	RBPJL	20	45271824	45329677	220	16	10164	1.1181	0.13176
+	MARK3	14	103350364	103513831	482	25	10164	1.1181	0.13177
+	AIG1	6	143024363	143350343	893	50	10164	1.118	0.13178
-	CLPB	11	72282425	72469680	472	37	10164	1.1176	0.13188
+	CCR4	3	32916574	32964911	142	6	10164	1.117	0.132
+	ZNRD1	6	30026240	30074909	532	25	10164	1.1168	0.13204
-	SCNN1A	12	6336843	6412357	221	55	10164	1.1167	0.13206
-	IL5	5	132531444	132591890	154	20	10164	1.1165	0.1321
+	NPSR1	7	34623239	34888332	948	51	10164	1.1165	0.13211
+	CYP3A43	7	99793013	99877092	127	15	10164	1.1164	0.13213
+	RPLP2	11	774936	822876	135	20	10164	1.1158	0.13226
-	DEFB136	8	11963937	12009599	15	4	10164	1.1153	0.13236
+	TRIP13	5	857854	929072	146	11	10164	1.1143	0.13257
-	CYP1B1	2	38057603	38111181	226	28	10164	1.1141	0.13262
+	ACVR1B	12	51916667	52007079	143	21	10164	1.114	0.13263
-	SECISBP2L	15	48978638	49081563	197	22	10164	1.1139	0.13265
+	ZNRF1	16	74964017	75120994	388	28	10164	1.1136	0.13272
-	PBLD	10	68272660	68367928	251	17	10164	1.1135	0.13276
+	CREB3L1	11	46242638	46331422	151	25	10164	1.1132	0.13281
-	NRG2	5	139836779	140078437	354	33	10164	1.1131	0.13284
-	LOC1053767	16	88376927	88426933	2	1	10164	1.1126	0.13294
+	KCNK18	10	117162489	117220299	115	12	10164	1.1124	0.13298
+	RBM6	3	49905038	50087252	421	20	10164	1.1123	0.133
+	CCDC24	1	43956608	44006528	75	12	10164	1.1122	0.13303
-	ZNF281	1	200394947	200445058	97	15	10164	1.1119	0.13308
+	SPCS1	3	52670841	52718182	95	9	10164	1.1119	0.13308
-	AGPAT4	6	161120020	161309075	581	83	10164	1.1113	0.13322
-	METTL4	18	2527525	2606490	279	25	10164	1.1108	0.13333
+	CCDC144A	17	16655235	16784999	102	7	10164	1.1103	0.13344
-	NAP1L1	12	76034892	76119958	74	5	10164	1.1102	0.13345
-	P3H4	17	41791952	41847616	131	10	10164	1.11	0.13351
+	CLDN4	7	73795863	73842693	71	2	10164	1.1092	0.13367
+	LIPJ	10	88548277	88616976	204	17	10164	1.1089	0.13373
+	COMMD10	5	116048645	116422683	2034	47	10164	1.1089	0.13374
-	RNF115	1	145736082	145859123	124	7	10164	1.1087	0.13377
+	TMEM45B	11	129780781	129870003	279	25	10164	1.1087	0.13379
-	KCNN4	19	43756533	43817139	225	27	10164	1.1084	0.13385
+	CIC	19	42233537	42305796	82	5	10164	1.1083	0.13386
-	KRTAP8-1	21	30802697	30848252	180	21	10164	1.1083	0.13386
-	MED13L	12	115945771	116312203	579	39	10164	1.1082	0.13389
-	CBLB	3	105645461	105904552	773	32	10164	1.1078	0.13396
+	PICK1	22	38021420	38085701	131	24	10164	1.1077	0.13401
+	SEC62	3	169931792	170008373	251	32	10164	1.1074	0.13405
-	HIST1H4B	6	26016896	26062252	194	37	10164	1.1068	0.13419
+	LAMC3	9	130974117	131103059	398	47	10164	1.1067	0.13421
+	C2orf47	2	199920317	199974125	109	19	10164	1.1067	0.13421
-	ATP4B	13	113638804	113693198	81	20	10164	1.1058	0.13441
+	FOLH1B	11	89602520	89708718	540	16	10164	1.1056	0.13444
+	ITCH	20	34328235	34521393	318	18	10164	1.1052	0.13454
-	COL22A1	8	138578235	138949094	839	74	10164	1.1048	0.13463
+	DPH2	1	43934981	43983371	84	12	10164	1.1045	0.13468
-	SGTA	19	2744713	2818356	105	5	10164	1.1045	0.13469
-	DIDO1	20	62867734	62972952	262	25	10164	1.1044	0.1347
-	FBN1	15	48398306	48680788	484	16	10164	1.1043	0.13473
+	PCDHB16	5	141146690	141196221	144	13	10164	1.1041	0.13478
-	NCAPD3	11	134142442	134260345	198	22	10164	1.1036	0.13487
+	C17orf100	17	6616739	6663298	147	11	10164	1.1037	0.13487
-	LOC1019295	17	82703905	82751473	223	23	10164	1.1035	0.1349
+	SCGB1A1	11	62384035	62433206	205	26	10164	1.1032	0.13498
-	CBLN3	14	24416532	24464525	272	16	10164	1.1031	0.13499
-	B3GALT2	1	193168730	193221613	59	6	10164	1.103	0.13502

STRAND	SYMBOL	CHR	START	STOP	NSNPS	NPARAM	N	ZSTAT	P
+	LCN1	9	135486297	135536540	117	11	10164	1.1029	0.13504
+	ZNF586	19	57734652	57790616	116	13	10164	1.1027	0.13507
+	SPO11	20	57294759	57353993	124	15	10164	1.1026	0.1351
+	LRRC42	1	53911326	53978168	123	12	10164	1.1024	0.13513
+	KIAA0754	1	39374504	39426482	71	11	10164	1.1021	0.1352
+	RUNDC3B	7	87592675	87842298	519	30	10164	1.102	0.13522
-	CDCA3	12	6834798	6887066	29	7	10164	1.1018	0.13528
+	PRMT3	11	20352530	20519333	551	25	10164	1.1017	0.1353
+	PTGS1	9	122335530	122405705	131	21	10164	1.1016	0.13532
+	UBXN10	1	20148302	20206048	78	11	10164	1.1014	0.13536
+	CTNNA1	5	138718396	138945034	172	16	10164	1.1013	0.13538
+	WFDC10A	20	45594746	45641192	180	14	10164	1.1006	0.13554
-	TMEM194A	12	57045643	57123857	146	20	10164	1.1003	0.13559
+	FGFR4	5	177051872	177108142	146	17	10164	1.1003	0.1356
+	LFNG	7	2477529	2539177	82	12	10164	1.1002	0.13562
-	CHTF8	16	69108009	69167590	114	24	10164	1.1	0.13566
+	TMEM200A	6	130331281	130453424	296	15	10164	1.0998	0.13572
-	UBE2H	7	129820732	129987960	475	19	10164	1.0994	0.13579
+	FAM178A	10	100877569	100975134	164	17	10164	1.0993	0.13581
+	C1orf27	1	186340758	186431375	217	24	10164	1.0991	0.13586
+	OR9K2	12	55094769	55140776	225	5	10164	1.0989	0.13591
+	LOC1053785	1	2245383	2294676	167	38	10164	1.098	0.13609
+	LAMA3	18	23654443	23965066	651	30	10164	1.0975	0.1362
+	RAPGEFL1	17	40142010	40205656	44	7	10164	1.0975	0.13622
+	RASL11A	13	27235327	27283767	71	15	10164	1.0974	0.13623
-	C4orf27	4	169719468	169792942	30	3	10164	1.097	0.13632
+	ISCA2	14	74458720	74505568	113	8	10164	1.0969	0.13633
-	OR10A6	11	7917718	7963662	172	19	10164	1.0969	0.13635
+	GDA	9	72079595	72269059	372	20	10164	1.0963	0.13648
+	TSPO	22	43116514	43173242	229	44	10164	1.0959	0.13655
+	OR4A47	11	48448466	48499774	147	14	10164	1.0958	0.13658
+	DAAM1	14	59153611	59381405	676	35	10164	1.0955	0.13664
+	SP9	2	174299960	174356005	63	6	10164	1.0955	0.13665
-	FUCA1	1	23835077	23903369	270	14	10164	1.095	0.13676
-	HSPB11	1	53911561	53981303	129	13	10164	1.0947	0.13682
-	NECAB3	20	33647087	33709458	110	25	10164	1.0946	0.13684
+	DNAH11	7	21508215	21911568	2249	176	10164	1.0945	0.13688
+	FAM171B	2	186659062	186775959	273	25	10164	1.0943	0.13692
-	ATP9A	20	51586775	51803411	842	41	10164	1.0936	0.13706
+	DSG1	18	31283089	31369246	398	22	10164	1.0934	0.13711
-	MKKS	20	10394780	10469239	299	13	10164	1.0934	0.13712
+	PFKFB2	1	206999416	207091023	186	28	10164	1.0932	0.13714
+	GPT	8	144467221	144517174	57	7	10164	1.0931	0.13718
+	PPP1R11	6	30031880	30080333	534	26	10164	1.093	0.1372
-	CRK	17	1411353	1491267	208	20	10164	1.0928	0.13725
-	TTC33	5	40701576	40790970	257	29	10164	1.0927	0.13726
+	FKBP6	7	73293152	73368637	25	7	10164	1.0926	0.13728
-	SLC11A2	12	50969410	51063335	246	23	10164	1.0925	0.13731
-	FAM173B	5	10215507	10285036	280	18	10164	1.092	0.13741
+	CDH15	16	89136755	89205492	347	27	10164	1.0919	0.13743
+	COL19A1	6	69831556	70222454	1222	72	10164	1.0919	0.13745
-	MOK	14	102214450	102340200	281	24	10164	1.0919	0.13745
-	TMEM158	3	45214464	45261322	237	26	10164	1.0918	0.13746
+	AP2A1	19	49731923	49817112	187	33	10164	1.0917	0.13749
-	DLG4	17	7179890	7255050	118	24	10164	1.0913	0.13758
-	CCDC17	1	45610044	45659787	180	16	10164	1.0908	0.13767
+	RPL10A	6	35433401	35480781	256	47	10164	1.0908	0.13768
+	MAPKAPK2	1	206649912	206744285	184	23	10164	1.0908	0.13769
+	SEMG2	20	45186369	45234458	114	11	10164	1.0904	0.13776
-	SNUPN	15	75588083	75661378	182	31	10164	1.0903	0.13779
+	DHRS11	17	36556786	36610804	25	7	10164	1.0902	0.13782
-	EPPIN	20	45530626	45582426	318	8	10164	1.0897	0.13791
-	OR5AU1	14	21144937	21191025	103	12	10164	1.0897	0.13793

STRAND	SYMBOL	CHR	START	STOP	NSNPS	NPARAM	N	ZSTAT	P
+	PNPLA2	11	783891	835573	146	23	10164	1.0893	0.138
-	NCKAP5	2	132661788	133709923	2550	204	10164	1.0892	0.13802
-	LRP11	6	149785188	149899348	288	19	10164	1.0888	0.13813
+	MRPS35	12	27675773	27766304	220	21	10164	1.0887	0.13815
-	FBXO46	19	45700629	45765893	120	20	10164	1.0882	0.13825
+	DEFB115	20	31222664	31269632	102	4	10164	1.0882	0.13825
-	C10orf67	10	23192851	23380216	474	28	10164	1.0874	0.13844
-	TMC6	17	78102918	78167407	215	47	10164	1.0871	0.1385
+	DNAH8	6	38680332	39040798	1602	75	10164	1.087	0.13851
-	PCDH9	13	66292834	67265336	2393	106	10164	1.0869	0.13855
-	AVPI1	10	97667424	97722258	146	20	10164	1.0867	0.13858
-	SRXN1	20	636615	688370	133	27	10164	1.0866	0.13862
-	LOC1010601	19	12237244	12302038	160	8	10164	1.0865	0.13862
-	CLNK	4	10479900	10769853	1220	78	10164	1.0864	0.13864
-	ZC3H8	2	112205677	112290101	80	12	10164	1.0861	0.13871
+	NUCB1	19	48865050	48933283	198	20	10164	1.0857	0.1388
+	IQSEC3	12	31796	188460	425	50	10164	1.0853	0.1389
+	LOC1053771	3	52964045	53058123	300	24	10164	1.0852	0.13892
-	CDK11B	1	1625226	1694839	2	1	10164	1.0851	0.13893
+	WNK2	9	93149155	93330572	534	47	10164	1.085	0.13897
-	DAB2	5	39361674	39460233	291	40	10164	1.0849	0.13899
+	OXSR1	3	38130260	38265488	166	23	10164	1.0848	0.139
+	NBL1	1	19608229	19668456	168	27	10164	1.0841	0.13915
+	LOC1053751	7	141197	190728	179	16	10164	1.0841	0.13916
-	C9orf57	9	72041381	72095605	120	14	10164	1.0838	0.13924
-	CEP83	12	94255652	94494988	339	26	10164	1.0837	0.13925
-	GSG1	12	13073537	13138720	108	20	10164	1.0836	0.13928
+	LIMD1	3	45559188	45691263	422	35	10164	1.0834	0.13932
+	SAMD13	1	84239952	84360798	98	18	10164	1.0834	0.13932
+	TRMU	22	46300401	46367340	104	4	10164	1.0831	0.13938
+	PRPF38A	1	52369547	52428320	76	14	10164	1.0829	0.13943
+	B3GNT2	2	62161127	62234731	161	22	10164	1.0827	0.13948
+	NDUFB10	16	1924516	1971975	168	35	10164	1.0826	0.1395
-	VSIG2	11	124737472	124788025	78	12	10164	1.0824	0.13953
+	PCDHB7	5	141137621	141186376	139	15	10164	1.0819	0.13965
-	BTBD18	11	57733514	57788256	88	17	10164	1.0818	0.13967
-	SETD7	4	139485660	139592157	190	31	10164	1.0817	0.13968
+	C18orf21	18	35937625	35989287	103	7	10164	1.0816	0.13972
-	C19orf18	19	57948437	58009534	158	10	10164	1.0812	0.1398
+	LOC1027244	19	20212268	20267291	73	4	10164	1.0811	0.13982
+	GAPDHS	19	35498412	35555319	193	34	10164	1.0809	0.13988
-	IKBIP	12	98603404	98680051	214	24	10164	1.0807	0.13991
-	SPPL2A	15	50697533	50801158	380	25	10164	1.0806	0.13994
-	TRIM64B	11	89860127	89913437	4	2	10164	1.0805	0.13997
-	ALDH4A1	1	18861430	18937799	179	34	10164	1.0803	0.14001
+	ZNF678	1	227528519	227687443	587	26	10164	1.0802	0.14002
+	TDRD1	10	114139442	114242669	181	15	10164	1.0802	0.14003
+	MAST4	5	66561348	67179598	1568	98	10164	1.08	0.14007
+	KRTAP5-5	11	1594803	1640930	267	26	10164	1.0797	0.14013
-	IARS	9	92200207	92328756	256	15	10164	1.0797	0.14014
-	SFTA3	14	36463288	36548785	167	28	10164	1.0795	0.14019
+	C14orf177	14	98676613	98727766	219	24	10164	1.0793	0.14022
-	SIGLEC10	19	51400021	51452803	140	22	10164	1.0791	0.14028
+	NXNL2	9	88500073	88594466	49	8	10164	1.0788	0.14034
+	FBLN2	3	13514125	13648422	494	77	10164	1.0786	0.14039
+	BROX	1	222677553	222745196	156	11	10164	1.0783	0.14044
-	CASQ2	1	115690003	115803805	498	25	10164	1.078	0.14051
+	TSC2	16	2012936	2098712	102	29	10164	1.078	0.14051
-	ESF1	20	13704322	13819933	291	30	10164	1.0775	0.14063
+	VPS18	15	40859430	40913975	166	19	10164	1.0773	0.14067
+	LRRC56	11	484000	564916	372	20	10164	1.0773	0.14067
-	TRIM29	11	120101286	120220423	436	27	10164	1.0772	0.14069
+	TMEM82	1	15707492	15757797	163	20	10164	1.077	0.14074

STRAND	SYMBOL	CHR	START	STOP	NSNPS	NPARAM	N	ZSTAT	P
+	ACKR3	2	236502126	236592358	105	10	10164	1.0763	0.1409
+	ZNF714	19	21047147	21135094	287	13	10164	1.0761	0.14094
+	ADGB	6	146563965	146825462	424	51	10164	1.076	0.14096
-	DIAPH3	13	59655583	60198985	1434	37	10164	1.0759	0.14099
+	RCAN3	1	24467351	24547020	161	18	10164	1.0758	0.14102
-	TTLL6	17	48752231	48852107	335	33	10164	1.0757	0.14104
+	APEH	3	49638130	49693504	70	8	10164	1.0756	0.14105
-	TNIP1	5	151019943	151122660	394	45	10164	1.0756	0.14106
+	INTU	4	127588271	127726779	224	19	10164	1.0756	0.14106
-	ZFP62	5	180835338	180896286	101	4	10164	1.0751	0.14115
-	KCNA3	1	110643560	110710033	111	13	10164	1.0751	0.14117
+	IFNK	9	27489314	27536498	185	17	10164	1.075	0.14118
-	HIST1H1C	6	26045740	26091471	203	31	10164	1.0745	0.14129
-	SIRPB2	20	1460688	1526587	230	18	10164	1.0745	0.1413
+	CCDC81	11	86339736	86433109	365	44	10164	1.0744	0.14131
+	OR1K1	9	122765123	122811073	68	12	10164	1.0743	0.14134
-	HIST1H2AB	6	26023092	26068568	182	35	10164	1.074	0.14141
+	TGIF2-C20or	20	36539554	36622557	62	21	10164	1.0734	0.14155
-	PCDH1	5	141842319	141913813	123	17	10164	1.0732	0.14158
+	JAM3	11	134033925	134161757	355	20	10164	1.073	0.14164
-	FAM219A	9	34388184	34493610	302	27	10164	1.0729	0.14166
-	C1orf52	1	85239953	85294672	172	25	10164	1.0727	0.14171
-	NOL8	9	92287358	92360994	146	10	10164	1.0726	0.14173
+	KRTAP13-1	21	30361074	30406822	150	27	10164	1.0724	0.14177
+	GHRHR	7	30929021	30989531	115	10	10164	1.0722	0.14181
+	LOC1053747	5	28774035	28908807	246	15	10164	1.0721	0.14184
+	LOC1010600	1	227934793	227981378	221	16	10164	1.0719	0.14188
+	AZU1	19	792831	842017	77	9	10164	1.0718	0.14191
+	HADH	4	107954714	108045175	225	13	10164	1.0718	0.14191
+	RAD9A	11	67356952	67408412	108	18	10164	1.0714	0.14199
-	EPPIN-WFD(	20	45526280	45582426	331	10	10164	1.0713	0.14201
+	LOC1053748	2	38040387	38105569	276	34	10164	1.0712	0.14205
+	DMRT1	9	806647	979090	688	43	10164	1.071	0.14208
+	PSMD3	17	39945768	40007960	238	20	10164	1.0707	0.14215
-	SLC7A4	22	21018718	21067558	108	19	10164	1.0706	0.14218
+	FLYWCH1	16	2876921	2961208	323	23	10164	1.0699	0.14233
-	GATM	15	45351124	45432043	226	14	10164	1.0697	0.14238
-	TJP1	15	29689367	30003799	797	51	10164	1.0697	0.14238
+	PGBD2	1	248837850	248929146	77	10	10164	1.0694	0.14245
-	MYT1L	2	1779113	2366375	1432	107	10164	1.0688	0.14258
+	TOMM40	19	44856220	44913689	155	19	10164	1.0687	0.1426
+	C9orf106	9	129286016	129332603	3	2	10164	1.0687	0.14261
+	RPL21	13	27216555	27266568	83	12	10164	1.0686	0.14263
+	PCDHB6	5	141115022	141163287	105	16	10164	1.0685	0.14265
+	LOC1019275	11	1004200	1065749	109	18	10164	1.0685	0.14265
-	DENND4A	15	65649123	65827293	344	14	10164	1.0684	0.14266
+	SLC28A2	15	45217066	45285934	294	8	10164	1.0683	0.14269
-	KRTAP13-2	21	30361391	30407239	150	27	10164	1.0681	0.14273
-	NDUFA4	7	10921953	10975186	187	27	10164	1.0681	0.14274
+	PRSS38	1	227780692	227870439	341	42	10164	1.068	0.14275
+	KIAA0141	5	141888785	141961465	152	17	10164	1.0678	0.14282
-	ACP5	19	11564660	11613986	57	9	10164	1.067	0.14299
+	ARMC4P1	10	27245762	27339145	500	26	10164	1.0662	0.14317
+	FAM218A	4	164921948	164969121	146	12	10164	1.0653	0.14338
+	SRD5A1	5	6598360	6679562	304	20	10164	1.0643	0.14359
+	PRDM16	1	3033227	3448621	1184	128	10164	1.064	0.14366
-	SUN3	7	47977148	48064127	234	20	10164	1.064	0.14366
-	MED27	9	131850110	132114887	416	28	10164	1.064	0.14367
+	LOC1053699	12	51812781	51863515	75	15	10164	1.0638	0.14372
+	CRISPLD2	16	84784981	84919510	639	66	10164	1.0633	0.14383
+	BHMG1	19	45688892	45774539	163	27	10164	1.0629	0.14391
+	FPGS	9	127767858	127824520	175	26	10164	1.0629	0.14392
+	TMEM175	4	897387	968656	195	31	10164	1.0627	0.14395

STRAND	SYMBOL	CHR	START	STOP	NSNPS	NPARAM	N	ZSTAT	P
-	RABGGTA	14	24255538	24306627	123	18	10164	1.0617	0.14418
+	SSH3	11	67268448	67322607	110	21	10164	1.0615	0.14422
+	NAALAD2	11	90096573	90209743	444	22	10164	1.0607	0.14441
+	RAE1	20	57316089	57388463	185	15	10164	1.0606	0.14443
+	BAALC	8	103105693	103240305	486	48	10164	1.0598	0.14461
+	C9orf163	9	136448495	136496067	74	6	10164	1.0596	0.14467
-	GPBP1L1	1	45617304	45723110	341	17	10164	1.0591	0.14477
+	TMEM125	1	43234951	43284002	52	5	10164	1.0584	0.14493
-	DEFB116	20	31293212	31343585	68	5	10164	1.058	0.14504
+	MED9	17	17441986	17503220	144	18	10164	1.0579	0.14506
-	SRP68	17	76028775	76107526	125	6	10164	1.0569	0.14527
-	ACLY	17	41856916	41965542	153	9	10164	1.0569	0.14528
-	ZNF112	19	44316553	44391704	248	26	10164	1.0569	0.14529
+	LRRC74B	22	21010960	21074168	154	29	10164	1.0567	0.14533
+	RNF165	18	46299224	46471412	433	64	10164	1.0567	0.14533
-	C9orf116	9	135485180	135536832	126	12	10164	1.0564	0.14538
-	TMEM246	9	101463158	101522221	192	24	10164	1.0564	0.14539
-	PARP16	15	65224464	65321884	26	10	10164	1.056	0.14548
+	CAND2	3	12761653	12844818	349	30	10164	1.0559	0.1455
-	SAMSN1	21	14475228	14618402	420	44	10164	1.0558	0.14553
+	FERMT3	11	64171655	64233891	188	33	10164	1.0555	0.14561
+	AMDHD1	12	95908293	95978592	299	17	10164	1.0552	0.14566
-	LOC1019284	7	56803396	56849205	83	10	10164	1.0546	0.14581
-	GBP2	1	89096132	89161159	168	11	10164	1.0543	0.14587
+	SCGB1D1	11	62155238	62203537	211	19	10164	1.0542	0.14589
+	TP53INP2	20	34669311	34723439	118	16	10164	1.0542	0.14589
+	GLIS2	16	4281181	4349597	94	13	10164	1.0541	0.14591
-	C8B	1	56919210	57001140	264	20	10164	1.0536	0.14603
-	WFDC9	20	45597939	45666268	263	19	10164	1.0531	0.14615
+	STAB1	3	52460338	52534495	183	24	10164	1.0524	0.1463
+	GPR17	2	127610864	127662639	162	21	10164	1.0522	0.14636
-	CCDC92	12	123926409	124007985	124	5	10164	1.052	0.1464
-	C9orf24	9	34369019	34433577	187	30	10164	1.0516	0.14648
+	CPZ	4	8557660	8629761	146	18	10164	1.0516	0.1465
-	COMMD5	8	144827978	144888640	159	13	10164	1.0516	0.1465
+	ACSF3	16	89058809	89165846	523	29	10164	1.0514	0.14653
-	RND3	2	150458193	150522695	136	18	10164	1.0514	0.14655
+	OTUD3	1	19846702	19922945	188	21	10164	1.0514	0.14655
-	OXER1	2	42752499	42799261	228	27	10164	1.0506	0.14673
+	LOC1053756	8	100071005	100117768	98	13	10164	1.0504	0.14677
-	ENO1	1	8851000	8914092	132	15	10164	1.05	0.14685
+	LOC1053709	15	40869922	40918532	140	19	10164	1.05	0.14686
-	PDHB	3	58417630	58468852	103	7	10164	1.0498	0.14691
+	PCDHB9	5	141152127	141201538	140	13	10164	1.0497	0.14693
-	ASTE1	3	131003873	131061854	127	16	10164	1.0494	0.147
+	NKD1	16	50513330	50659249	188	24	10164	1.0491	0.14706
-	TTPA	8	63049488	63121100	229	27	10164	1.0487	0.14716
-	XKR6	8	10886045	11236381	1309	67	10164	1.0486	0.14718
+	FCGR2B	1	161628115	161688654	88	19	10164	1.0484	0.14723
-	COX6C	8	99867995	99929014	106	13	10164	1.0481	0.1473
-	HPCAL4	1	39668648	39726710	182	32	10164	1.0476	0.14742
-	ANKRD26	10	26956584	27135498	640	21	10164	1.0473	0.14747
+	PAX9	14	36622568	36687807	238	23	10164	1.0472	0.1475
-	MLXIPL	7	73583194	73659540	62	8	10164	1.0471	0.14752
+	ARFIP1	4	152744937	152921912	314	16	10164	1.0468	0.14761
-	LRRC40	1	70134802	70240678	189	22	10164	1.0468	0.14761
+	SPAG6	10	22310416	22427610	47	25	10164	1.0464	0.14769
-	GLT8D1	3	52684484	52741083	116	11	10164	1.046	0.14779
-	ANKRD35	1	145856560	145920866	82	9	10164	1.0458	0.14783
+	GIMAP8	7	150415630	150489395	171	16	10164	1.0456	0.14787
+	SPATA5L1	15	45367273	45431419	165	14	10164	1.0454	0.14792
-	ZNF552	19	57797082	57850188	120	12	10164	1.0454	0.14793
+	NTN1	17	8986542	9254000	803	94	10164	1.0448	0.14805

STRAND	SYMBOL	CHR	START	STOP	NSNPS	NPARAM	N	ZSTAT	P
+	TMEM69	1	45653175	45704436	155	15	10164	1.0448	0.14805
-	PLA2G4E	15	41971582	42085703	509	49	10164	1.0446	0.1481
+	STK38L	12	27209145	27335959	359	36	10164	1.0444	0.14815
-	SIRT6	19	4164109	4217599	3	2	10164	1.0443	0.14817
+	UAP1	1	162526506	162609843	313	20	10164	1.0438	0.14829
-	KIAA1257	3	128900186	129061519	403	23	10164	1.0438	0.14829
-	DNAJC11	1	6624168	6736906	245	20	10164	1.0438	0.1483
+	ZC2HC1A	8	78631047	78729765	202	12	10164	1.0435	0.14835
+	GCA	2	162283793	162389989	139	9	10164	1.0434	0.14837
+	GPRC5C	17	74389503	74461658	51	16	10164	1.0434	0.14838
-	SERP1	3	150531993	150638177	199	15	10164	1.043	0.14848
+	CALM1	14	90361983	90418275	8	2	10164	1.0429	0.1485
-	LOC1053691	15	45222945	45310437	304	9	10164	1.0428	0.14852
-	SCAMP2	15	74833730	74908365	207	40	10164	1.0426	0.14856
-	LRRC37A3	17	64844147	64954480	2	1	10164	1.0424	0.14862
+	ZNF749	19	57393568	57457485	139	26	10164	1.0423	0.14864
+	HLA-DQA1	6	32602396	32664774	2917	37	10164	1.0422	0.14866
+	LOC1053767	15	45413410	45479925	230	12	10164	1.0421	0.14867
+	NANOS1	10	118994716	119044342	127	19	10164	1.0421	0.14868
+	ZNF74	22	20359115	20418463	67	12	10164	1.0417	0.14878
-	RAD51AP2	2	17500584	17555580	61	11	10164	1.0416	0.14879
-	PRDM7	16	90046566	90112339	161	16	10164	1.0415	0.14882
+	LOC1053786	1	42944698	42995699	204	18	10164	1.0414	0.14885
+	COL4A2	13	110272284	110523027	1399	174	10164	1.0413	0.14886
-	CC2D1B	1	52340593	52401205	89	14	10164	1.0406	0.14904
-	ABI3BP	3	100739335	101028493	977	71	10164	1.0405	0.14905
+	ADK	10	74116185	74719303	1013	21	10164	1.04	0.14917
-	IL22RA1	1	24109771	24178285	229	33	10164	1.0397	0.14923
-	NOL10	2	10560766	10724987	651	22	10164	1.0395	0.14929
-	TRPM7	15	50547158	50721815	582	26	10164	1.0394	0.1493
-	TSR3	16	1339240	1386872	48	11	10164	1.0392	0.14936
-	CHRM1	11	62898679	62956861	106	24	10164	1.0385	0.14952
-	CELF1	11	47455937	47600569	154	23	10164	1.038	0.14964
-	CCDC73	11	32592080	32829658	529	17	10164	1.0377	0.14971
+	FAM183A	1	43107979	43166396	81	5	10164	1.0371	0.14984
-	TCL1A	14	95699967	95749196	133	12	10164	1.0371	0.14985
-	USP50	15	50483681	50582439	310	21	10164	1.0368	0.14992
-	PRAMEF11	1	12814380	12866410	249	6	10164	1.0363	0.15002
-	PRSS57	19	675546	730531	1	1	10164	1.0361	0.15007
-	TULP2	19	48870822	48935184	186	19	10164	1.036	0.15011
-	FGG	4	154594134	154647750	66	10	10164	1.0359	0.15013
+	METTL9	16	21562221	21667473	168	12	10164	1.0358	0.15015
-	OLR1	12	10148300	10211261	230	22	10164	1.0356	0.15019
-	APLNR	11	57223577	57272453	108	12	10164	1.0354	0.15023
-	MON1A	3	49898869	49965357	137	16	10164	1.0353	0.15026
+	CXCR6	3	45905399	45958354	137	15	10164	1.0352	0.15029
-	RAB3B	1	51897956	52025764	284	25	10164	1.0349	0.15035
-	OPALIN	10	96333216	96394365	164	17	10164	1.0349	0.15036
+	MC5R	18	13790544	13836862	233	20	10164	1.0348	0.15037
-	NELFA	4	1972714	2076914	151	20	10164	1.0345	0.15046
+	PTPRQ	12	80409274	80690189	532	29	10164	1.0343	0.15049
+	MTTP	4	99529078	99633997	334	20	10164	1.0341	0.15054
+	MASTL	10	27119824	27196920	271	16	10164	1.0337	0.15063
+	CHAC1	15	40918438	40966519	123	19	10164	1.0337	0.15064
-	ZNF561	19	9597326	9656240	110	11	10164	1.0333	0.15073
+	HOXC12	12	53919930	53966566	117	19	10164	1.0328	0.15085
+	RNF123	3	49654143	49731529	110	17	10164	1.0323	0.15096
+	ZNF672	1	248803178	248859517	72	6	10164	1.0323	0.15096
+	SPTBN1	2	54421317	54681446	975	61	10164	1.0322	0.15099
+	LOC1053730	22	49814635	49861067	165	18	10164	1.0321	0.151
+	PYDC2	3	191426163	191471456	177	13	10164	1.0318	0.15108
-	ANKRD60	20	58206126	58263653	110	16	10164	1.0317	0.15111
-	PEPD	19	33376949	33556893	676	45	10164	1.0315	0.15115

STRAND	SYMBOL	CHR	START	STOP	NSNPS	NPARAM	N	ZSTAT	P
-	RPL3	22	39302882	39354665	222	22	10164	1.031	0.15128
-	DDC	7	50448436	50600457	650	28	10164	1.0308	0.15131
-	C16orf89	16	5032772	5101145	55	8	10164	1.0308	0.15131
+	TMOD1	9	97466180	97611743	445	52	10164	1.0298	0.15156
+	WDR75	2	189406433	189485562	337	12	10164	1.0295	0.15162
-	TYW5	2	199918911	199990736	148	25	10164	1.0293	0.15166
+	ASRGL1	11	62302302	62411378	428	20	10164	1.0289	0.15177
-	PADI2	1	17056761	17154453	227	9	10164	1.0285	0.15186
-	STOML2	9	35089776	35138195	155	30	10164	1.028	0.15198
+	KLHL7	7	23070734	23185421	268	13	10164	1.0278	0.15202
+	LOC1053710	16	1395309	1443421	93	15	10164	1.0276	0.15206
+	CLDN16	3	190352872	190422143	259	31	10164	1.0276	0.15207
+	MDM4	1	204481379	204591681	244	15	10164	1.0274	0.15212
-	RASD1	17	17484437	17531395	131	26	10164	1.0254	0.15258
-	SEP15	1	86852445	86949424	186	8	10164	1.0252	0.15263
+	CD160	1	145684433	145749288	38	4	10164	1.0251	0.15265
-	SERPINA1	14	94366747	94425692	207	31	10164	1.0246	0.15279
+	HMGXB3	5	149965606	150063143	230	18	10164	1.0242	0.15286
+	PQLC3	2	11120372	11188874	167	13	10164	1.024	0.15291
-	HIST1H3F	6	26240142	26285607	172	30	10164	1.0237	0.15299
+	RBL2	16	53399439	53501648	192	15	10164	1.0229	0.15319
+	CTRB1	16	75183986	75234924	183	18	10164	1.0227	0.15323
+	CARNS1	11	67380678	67435607	122	20	10164	1.0226	0.15326
+	OTOA	16	21629155	21770729	222	28	10164	1.0225	0.15328
+	IK	5	140612799	140672480	123	10	10164	1.0223	0.15332
-	CARD9	9	136353956	136408681	153	10	10164	1.022	0.15338
-	LOC1005074	6	138715211	138808679	257	32	10164	1.0219	0.1534
+	NCAPD2	12	6459132	6541966	321	20	10164	1.0219	0.15341
-	ATP5G2	12	53655160	53711728	232	27	10164	1.0219	0.15343
-	PPP1CA	11	67388181	67436905	111	18	10164	1.0218	0.15344
+	POLR3C	1	145789069	145854402	60	5	10164	1.0214	0.15353
-	ZNF106	15	42402437	42526197	240	26	10164	1.0205	0.15375
+	LGALS9	17	27596148	27659560	170	15	10164	1.0202	0.15383
-	CER1	9	14709733	14757717	150	24	10164	1.0197	0.15394
+	SRSF11	1	70170682	70262020	166	20	10164	1.0193	0.15404
-	ZDHHC6	10	112415439	112482234	95	18	10164	1.0191	0.15407
-	BTBD1	15	83006423	83102434	212	29	10164	1.019	0.1541
-	GUCA1B	6	42173284	42241551	5	2	10164	1.0186	0.15419
+	LGALS1	22	37640606	37689802	161	19	10164	1.0185	0.15422
-	POLL	10	101568882	101623270	114	20	10164	1.018	0.15433
-	NSUN2	5	6589239	6668360	323	22	10164	1.0179	0.15437
+	KIR3DL3	19	54689447	54746536	144	12	10164	1.0176	0.15443
-	CBY3	5	179668558	179715974	112	14	10164	1.0174	0.15449
+	PRAMEF2	1	12822086	12871909	251	5	10164	1.0171	0.15455
-	ZNF571	19	37554253	37629792	184	23	10164	1.0169	0.15459
+	TMEM145	19	42278325	42335064	45	4	10164	1.0167	0.15464
-	IFNW1	9	21130632	21177145	216	44	10164	1.0167	0.15465
+	NUPL2	7	23146827	23211011	105	10	10164	1.0165	0.1547
-	CCDC25	8	27723316	27807655	326	13	10164	1.0164	0.15473
+	DNAI1	9	34423752	34530989	271	31	10164	1.0161	0.15478
+	MAP10	1	232769892	232820346	194	18	10164	1.0155	0.15494
+	PRDM9	5	23472615	23538597	55	11	10164	1.0152	0.15501
+	NOSTRIN	2	168751539	168875339	296	32	10164	1.0145	0.15517
+	CIRH1A	16	69097596	69179034	161	29	10164	1.0142	0.15524
+	RASA1	5	87232801	87401926	270	17	10164	1.0141	0.15526
+	SUGCT	7	40099965	40870767	904	48	10164	1.014	0.1553
-	METTL24	6	110235946	110393272	298	32	10164	1.0139	0.15532
-	ICA1	7	8103184	8297687	418	63	10164	1.0135	0.15541
-	LALBA	12	48557684	48635111	155	21	10164	1.0132	0.15549
-	CRTAC1	10	97844711	98065828	308	26	10164	1.0131	0.15551
-	FAM221B	9	35807017	35868805	139	24	10164	1.0127	0.1556
+	RANBP1	22	20080938	20137357	200	20	10164	1.0125	0.15564
-	LOC1010599	4	164919491	164965626	137	11	10164	1.0124	0.15567

STRAND	SYMBOL	CHR	START	STOP	NSNPS	NPARAM	N	ZSTAT	P
+	CTNND1	11	57726762	57829180	174	24	10164	1.0121	0.15575
+	ABCB8	7	150993422	151057782	167	41	10164	1.012	0.15578
-	ABCA12	2	214921542	215173580	335	30	10164	1.0118	0.15582
-	COQ2	4	83128601	83320089	317	14	10164	1.0117	0.15585
+	CCDC117	22	28737674	28799301	204	32	10164	1.0113	0.15593
-	NMU	4	55585229	55671698	255	22	10164	1.0113	0.15594
-	CA10	17	51620313	52195017	1334	88	10164	1.011	0.15601
+	OSBPL9	1	51581874	51799219	308	19	10164	1.0109	0.15604
+	WISP3	6	112018516	112079686	164	34	10164	1.0096	0.15635
+	NDC80	18	2536511	2626635	277	25	10164	1.0096	0.15635
-	FAM124B	2	224368698	224436994	335	29	10164	1.0095	0.15637
+	GPR45	2	105206743	105253467	123	18	10164	1.0091	0.15646
+	ALG1	16	5036809	5097379	51	7	10164	1.0088	0.15654
-	ALG12	22	49849147	49953458	551	22	10164	1.0087	0.15656
-	LPPR3	19	802488	859243	63	8	10164	1.0086	0.15658
+	MFSD3	8	144472267	144521228	57	7	10164	1.0084	0.15664
+	PNPLA1	6	36204766	36323955	404	85	10164	1.0079	0.15674
+	LOC1053780	6	150565272	150611061	163	33	10164	1.0078	0.15677
+	FGD5	3	14783962	14944565	585	28	10164	1.0078	0.15678
-	SFT2D1	6	166309728	166377606	90	5	10164	1.0077	0.1568
-	TOMM70A	3	100353459	100436398	191	24	10164	1.0076	0.15682
-	IPP	1	45684324	45785813	314	15	10164	1.0074	0.15687
-	KIAA0895	7	36314150	36425125	282	29	10164	1.0074	0.15688
-	SH3YL1	2	208136	299866	152	9	10164	1.0068	0.15702
-	SEPHS2	16	30433625	30480975	17	3	10164	1.0066	0.15706
+	CD200	3	112297347	112372812	338	26	10164	1.006	0.15721
-	KCTD12	13	76870169	76921405	104	15	10164	1.0057	0.15727
+	HN1L	16	1643277	1712072	171	19	10164	1.0056	0.1573
+	PRAMEF20	1	13375450	13431354	143	16	10164	1.0055	0.15732
+	CHRAC1	8	140476298	140527153	48	12	10164	1.0054	0.15735
+	FOXR1	11	118936543	118991285	168	24	10164	1.0052	0.1574
-	ZDHHC22	14	77121270	77179268	269	51	10164	1.0051	0.15743
+	SACM1L	3	45654262	45755409	332	18	10164	1.0045	0.15757
+	LOC1027235	9	127781066	127827166	148	20	10164	1.0045	0.15757
+	SYNGR2	17	78133551	78182928	185	33	10164	1.0042	0.15764
+	LRRN1	3	3764402	3859834	374	47	10164	1.0038	0.15774
+	CEP164	11	117286778	117423266	359	34	10164	1.0034	0.15783
-	PTGR1	9	111539722	111634855	319	36	10164	1.0034	0.15784
+	ASCL1	12	102922674	102970516	94	15	10164	1.0033	0.15786
-	HIST1H4G	6	26236611	26281977	170	30	10164	1.0029	0.15795
+	FNDC3B	3	172004628	172410703	753	70	10164	1.0028	0.15797
-	SH3BP5L	1	248800446	248860955	74	7	10164	1.0026	0.15804
-	CD14	5	140621728	140668701	102	9	10164	1.0023	0.1581
-	SGSH	17	80196966	80255400	245	23	10164	1.0022	0.15812
-	RABIF	1	202868282	202924257	161	15	10164	1.0019	0.1582
+	NPR2	9	35717945	35819731	188	31	10164	1.0018	0.15823
+	CCDC37	3	126359893	126446556	191	27	10164	1.0014	0.15832
-	CKAP4	12	106227881	106282935	91	17	10164	1.0013	0.15833
+	C2orf57	2	231557864	231604283	159	18	10164	1.0008	0.15846
+	ANKRD13D	11	67254291	67312484	113	24	10164	1.0007	0.15849
-	VPREB3	22	23742743	23789443	167	24	10164	1.0007	0.1585
+	AMY1C	1	103714656	103768692	13	5	10164	1.0006	0.15851
+	LOC388780	20	2171468	2268031	276	30	10164	1.0004	0.15855
-	RABL2B	22	50757492	50818675	7	2	10164	1.0004	0.15857
-	IFIH1	2	162257079	162353764	152	7	10164	1.0001	0.15863
-	WFDC6	20	45524197	45574495	315	10	10164	0.99985	0.15869
+	LOC389602	7	155927632	155976343	49	6	10164	0.99982	0.1587
+	MLPH	2	237451410	237565318	601	37	10164	0.99939	0.1588
+	C1orf53	1	197867552	197917367	83	15	10164	0.99915	0.15886
-	PRR18	6	166295680	166343383	153	9	10164	0.99899	0.1589
-	PLA2G2C	1	20153991	20210194	70	10	10164	0.99897	0.15891
-	IQCA1	2	236314147	236542552	314	15	10164	0.99861	0.15899
+	COL18A1	21	45370137	45523720	637	60	10164	0.99856	0.159

STRAND	SYMBOL	CHR	START	STOP	NSNPS	NPARAM	N	ZSTAT	P
-	ANKLE2	12	132715667	132796888	259	35	10164	0.99841	0.15904
-	HNRNPCL1	1	12837377	12883756	208	3	10164	0.99782	0.15918
+	CYP26A1	10	93038475	93087884	128	19	10164	0.9973	0.15931
-	TPMT	6	18118311	18190165	93	8	10164	0.99731	0.15931
-	BTBD10	11	13377996	13498297	345	19	10164	0.99719	0.15934
-	HIST1H3G	6	26260918	26306384	189	23	10164	0.99637	0.15954
-	LOC1053767	17	82691905	82740329	220	24	10164	0.99632	0.15955
+	AZIN2	1	33046110	33188892	302	16	10164	0.99626	0.15956
-	TRIM14	9	98073189	98154408	156	31	10164	0.99617	0.15958
-	APBB3	5	140548268	140599604	77	13	10164	0.99596	0.15963
-	IMP3	15	75629085	75675323	128	35	10164	0.99591	0.15965
+	GNG11	7	93886704	93936514	62	7	10164	0.99545	0.15976
-	CDK5RAP2	9	120378869	120615170	322	20	10164	0.99534	0.15979
+	DSE	6	116245120	116453475	571	45	10164	0.99496	0.15988
+	SLC22A14	3	38246430	38328522	260	17	10164	0.99482	0.15991
+	HIST1H2BI	6	26237976	26283412	164	29	10164	0.9945	0.15999
+	LINC01272	20	50232483	50289795	174	28	10164	0.99436	0.16002
+	BTNL8	5	180864077	180962166	243	10	10164	0.99429	0.16004
+	PPIH	1	42622770	42691654	141	17	10164	0.99397	0.16012
-	TGM1	14	24239114	24298210	135	22	10164	0.99375	0.16017
-	SPAG8	9	35797785	35847272	113	20	10164	0.99377	0.16017
-	PIGO	9	35078688	35131601	167	33	10164	0.99328	0.16029
-	HGF	7	81689006	81805438	236	24	10164	0.99243	0.16049
-	LIN7A	12	80782520	80972923	546	33	10164	0.99242	0.1605
-	ARRDC5	19	4880437	4951820	122	21	10164	0.99213	0.16057
+	MGST1	12	16312142	16387189	251	39	10164	0.99186	0.16063
-	CHI3L1	1	203168931	203221960	207	42	10164	0.99168	0.16068
-	SSH2	17	29615938	29965239	325	10	10164	0.99151	0.16072
+	C7orf57	7	48000511	48071297	183	16	10164	0.99145	0.16073
-	PDCD10	3	167673735	167770422	193	21	10164	0.99134	0.16076
+	OR10A7	12	55186025	55231975	144	7	10164	0.99103	0.16084
-	OC90	8	132014220	132094380	266	36	10164	0.99066	0.16092
-	PTCHD3	10	27388188	27449368	273	25	10164	0.98979	0.16114
-	CDC25C	5	138275265	138373355	173	14	10164	0.98976	0.16115
-	KIF3C	2	25916586	26017574	133	22	10164	0.9897	0.16116
+	ATOH8	2	85718786	85801383	275	51	10164	0.98913	0.1613
-	TRIM66	11	8602037	8717703	246	28	10164	0.98892	0.16135
-	MRPL51	12	6482150	6528305	189	16	10164	0.9887	0.16141
-	L2HGDH	14	50232434	50347389	259	12	10164	0.98851	0.16145
+	TRMT11	6	125951430	126049276	46	17	10164	0.98837	0.16148
-	DEFB119	20	31367163	31425649	114	8	10164	0.98829	0.1615
+	KDM1B	6	18120388	18233854	160	13	10164	0.98793	0.16159
+	EFCAB5	17	29894856	30118452	230	11	10164	0.98774	0.16164
-	TRIM64C	11	49043714	49094112	166	13	10164	0.98766	0.16166
+	ZNF664	12	123938215	124025439	126	6	10164	0.98692	0.16184
-	MSMP	9	35742990	35789277	80	19	10164	0.9868	0.16187
+	CDC37L1	9	4644553	4718399	368	29	10164	0.98624	0.16201
+	ZBTB8B	1	32430057	32497859	116	5	10164	0.98605	0.16205
-	TAPT1	4	16150505	16262158	380	33	10164	0.98593	0.16208
+	ZNF576	19	43561392	43610435	139	29	10164	0.98588	0.1621
+	PDXP	22	37623730	37676932	190	19	10164	0.9856	0.16216
-	ULK4	3	41229987	41997887	2665	55	10164	0.98506	0.1623
-	YES1	18	711588	847326	537	30	10164	0.98481	0.16236
+	EXO1	1	241812967	241919165	453	44	10164	0.98471	0.16238
-	ZNF235	19	44276348	44340707	127	13	10164	0.98466	0.1624
-	DYNC1LI1	3	32515971	32605874	246	30	10164	0.98463	0.1624
-	FBXO3	11	33730944	33809531	159	14	10164	0.98375	0.16262
-	COL7A1	3	48554073	48630302	77	13	10164	0.98333	0.16272
-	ZDHHC12	9	128710869	128759141	52	8	10164	0.98315	0.16277
+	UBE2Z	17	48873369	48939060	214	16	10164	0.98284	0.16284
+	RTP4	3	187333380	187381584	158	23	10164	0.98237	0.16296
-	CYCS	7	25108651	25160361	153	11	10164	0.98216	0.16301
-	DRICH1	22	23570872	23672723	306	29	10164	0.98199	0.16305

STRAND	SYMBOL	CHR	START	STOP	NSNPS	NPARAM	N	ZSTAT	P
+	SEMG1	20	45171997	45219773	127	9	10164	0.98091	0.16332
+	ZNF839	14	102282377	102353174	148	22	10164	0.98009	0.16352
-	DMKN	19	35487217	35548668	223	42	10164	0.97965	0.16363
+	MLNR	13	49185338	49232377	46	10	10164	0.97957	0.16365
+	ZNF551	19	57646969	57699811	152	17	10164	0.97921	0.16374
+	CABLES1	18	23099564	23270470	406	39	10164	0.9792	0.16374
-	YME1L1	10	27100111	27190278	317	13	10164	0.97891	0.16381
-	UNKL	16	1353205	1449749	143	24	10164	0.97888	0.16382
-	KIAA1211L	2	98783846	98971221	503	35	10164	0.97881	0.16384
-	TBL2	7	73558944	73613683	19	4	10164	0.97855	0.1639
-	IDI2	10	1008907	1060859	172	26	10164	0.97838	0.16394
-	FAM227B	15	49318393	49655931	971	28	10164	0.97814	0.164
-	PTGS2	1	186661812	186715427	209	17	10164	0.97763	0.16413
-	MAP4K3	2	39239266	39472312	596	24	10164	0.97763	0.16413
+	DNAH2	17	7682354	7843744	302	52	10164	0.97762	0.16413
+	TCERG1	5	146412310	146521508	263	20	10164	0.9774	0.16419
-	KLHL3	5	137607500	137771090	217	21	10164	0.97728	0.16422
-	RGL2	6	33281654	33334452	189	39	10164	0.97716	0.16424
-	VARS	6	31767518	31830935	261	74	10164	0.97692	0.1643
-	AKR7A2	1	19292708	19347146	180	23	10164	0.97663	0.16438
-	NSMAF	8	58573504	58694845	387	42	10164	0.97653	0.1644
+	MPI	15	74855011	74909457	145	29	10164	0.97563	0.16462
+	HS2ST1	1	86879652	87119998	376	7	10164	0.97562	0.16463
+	EAF2	3	121800187	121896526	273	18	10164	0.97549	0.16466
+	EIF4B	12	52971258	53052209	264	27	10164	0.97502	0.16477
+	SPX	12	21491322	21541276	182	25	10164	0.9747	0.16485
-	EYA3	1	27960344	28123675	251	23	10164	0.97456	0.16489
-	INTS7	1	211930399	212070677	378	16	10164	0.97444	0.16492
-	SLC35C2	20	46339528	46399458	70	10	10164	0.97428	0.16496
+	DNAJC10	2	182681041	182790025	167	12	10164	0.97386	0.16506
+	BTBD11	12	107282168	107669642	1108	92	10164	0.97366	0.16511
-	LOC1053722	18	76043684	76091412	236	25	10164	0.97364	0.16512
-	ABCB9	12	122908708	123010125	76	10	10164	0.9735	0.16515
+	KDM2A	11	67084268	67268082	305	31	10164	0.97338	0.16518
-	VAMP1	12	6452237	6505899	226	15	10164	0.97328	0.16521
-	WBSCR16	7	75017119	75108881	28	5	10164	0.97306	0.16526
-	MCM9	6	118803449	118970162	467	16	10164	0.97301	0.16527
-	WASL	7	123671927	123784071	230	15	10164	0.97298	0.16528
+	ERVV-2	19	53009738	53061127	103	14	10164	0.97299	0.16528
-	HINT2	9	35802960	35850479	103	21	10164	0.97238	0.16543
-	PRR15L	17	47941967	47992894	163	22	10164	0.97227	0.16546
-	AHCY	20	34224840	34346976	223	22	10164	0.97184	0.16556
-	ARHGEF2	1	155936640	156037635	160	27	10164	0.97176	0.16559
-	ZNF414	19	8500578	8549164	93	4	10164	0.97139	0.16568
+	IL23R	1	67103640	67269979	419	33	10164	0.97133	0.16569
-	RIMBP3C	22	21535666	21586461	55	4	10164	0.97119	0.16573
+	YTHDF2	1	28701621	28779775	85	5	10164	0.97109	0.16575
-	PRAMEF14	1	13331892	13382134	125	3	10164	0.97087	0.16581
-	LOC1053706	14	95453143	95500924	134	21	10164	0.9708	0.16582
-	IGDCC3	15	65317127	65413040	73	16	10164	0.97052	0.16589
+	THOP1	19	2750508	2823601	107	6	10164	0.97048	0.1659
-	SLC7A11	4	138154094	138347660	502	56	10164	0.96992	0.16604
+	LRRC14	8	144482965	144535178	58	7	10164	0.96918	0.16623
+	TCEB3	1	23708366	23772059	65	8	10164	0.96895	0.16628
+	C11orf31	11	57706250	57753411	93	17	10164	0.96898	0.16628
+	YEATS4	12	69324700	69434838	264	22	10164	0.96888	0.1663
-	C15orf40	15	82978308	83047298	145	27	10164	0.96847	0.1664
-	PTPN9	15	75457121	75614291	313	28	10164	0.96819	0.16648
-	HOXB4	17	48565507	48613381	160	36	10164	0.96807	0.1665
-	KIF21B	1	200959386	201058700	313	30	10164	0.96808	0.1665
-	FASTKD3	5	7849159	7904037	178	24	10164	0.96771	0.16659
+	ZDBF2	2	206239641	206324438	173	18	10164	0.96734	0.16669
-	LOC1053769	1	28667907	28716528	62	6	10164	0.96726	0.16671

STRAND	SYMBOL	CHR	START	STOP	NSNPS	NPARAM	N	ZSTAT	P
+	SET	9	128648432	128706396	79	16	10164	0.96706	0.16676
+	SRD5A3	4	55311221	55383100	164	20	10164	0.96678	0.16683
+	MARVELD1	10	97678708	97728152	131	23	10164	0.96672	0.16684
+	ZNF804B	7	88724368	89347057	2370	109	10164	0.96642	0.16692
-	CYP11B1	8	142862354	142914846	41	2	10164	0.96636	0.16693
-	WNT4	1	22107305	22178981	213	34	10164	0.96619	0.16697
+	POLR1B	2	112506915	112587153	162	12	10164	0.96573	0.16709
-	TP53BP2	1	223769893	223880981	289	25	10164	0.96553	0.16714
+	SMTNL1	11	57502552	57560274	162	24	10164	0.96534	0.16719
+	TRIM49B	11	48996600	49047977	159	10	10164	0.96483	0.16732
+	NTN3	16	2436499	2484145	7	2	10164	0.96449	0.1674
-	TRAPPC6B	14	39137811	39205430	215	31	10164	0.96428	0.16745
+	GPR32	19	50735464	50781732	191	51	10164	0.96416	0.16748
+	SLC45A1	1	8283085	8354167	91	20	10164	0.96416	0.16748
+	ZDHHC8	22	20096841	20158007	235	23	10164	0.96395	0.16753
-	LOC1053791	5	138734429	138789097	33	6	10164	0.96393	0.16754
+	LOC1053705	14	70188259	70262008	227	18	10164	0.96386	0.16756
-	MTRNR2L3	20	57348440	57394822	139	14	10164	0.9638	0.16757
-	CDC42EP3	2	37631882	37707535	256	24	10164	0.96328	0.1677
+	ACP1	2	229140	288283	98	7	10164	0.96326	0.16771
-	SOX7	8	10713768	10765574	245	24	10164	0.96324	0.16771
-	FAM65B	6	24794281	25077288	1113	91	10164	0.96291	0.1678
-	LOC1053792	8	10657199	10707600	219	30	10164	0.96282	0.16782
-	ABCA4	1	93982837	94156149	573	78	10164	0.96233	0.16794
+	ZSCAN4	19	57616504	57689152	250	18	10164	0.96209	0.168
-	SLC2A1	1	42915375	42994176	283	28	10164	0.96189	0.16805
+	MACF1	1	39046417	39497138	700	29	10164	0.96156	0.16814
+	TRMT44	4	8405778	8526547	287	26	10164	0.96125	0.16821
+	PROC	2	127383405	127439246	126	14	10164	0.96099	0.16828
-	MRPS18A	6	43661197	43722812	169	20	10164	0.961	0.16828
-	ST6GALNAC	9	127897886	127952051	121	17	10164	0.96087	0.16831
-	FAM104A	17	73197353	73267394	229	10	10164	0.96079	0.16833
-	FAP	2	162160684	162278535	204	11	10164	0.96072	0.16835
+	SOCS6	18	70253901	70340199	244	28	10164	0.96066	0.16836
+	EFCAB13	17	47288961	47451312	551	26	10164	0.96061	0.16837
-	PRR23C	3	139032102	139079892	92	8	10164	0.96061	0.16838
+	RPL27A	11	8647448	8699872	112	18	10164	0.96046	0.16841
+	ISX	22	35031136	35097387	264	16	10164	0.96022	0.16847
+	MSRB3	12	65243643	65476907	417	32	10164	0.96012	0.1685
-	TSTD2	9	97590080	97668680	239	11	10164	0.96001	0.16852
-	LOC1027243	22	37719416	37782434	128	14	10164	0.95991	0.16855
+	EPHX2	8	27456002	27554922	297	35	10164	0.95971	0.1686
-	ITGA2B	17	44362181	44424505	83	10	10164	0.9597	0.1686
-	NEGR1	1	71392942	72317847	2048	114	10164	0.9597	0.1686
-	TFAP4	16	4247186	4308000	147	17	10164	0.9594	0.16868
+	PARD6B	20	50696544	50763741	82	10	10164	0.95938	0.16868
+	MAPK8IP3	16	1671183	1780317	279	34	10164	0.95908	0.16876
-	ZNF512B	20	63946704	64004870	111	11	10164	0.95896	0.16879
-	TCERG1L	10	131082391	131346721	1319	130	10164	0.95878	0.16883
-	NGB	14	77255491	77306312	153	22	10164	0.95828	0.16896
-	CD59	11	33693010	33771479	107	10	10164	0.95768	0.16911
-	LINC00083	1	178468153	178534619	130	23	10164	0.95766	0.16912
-	MASP1	3	187206085	187327022	365	48	10164	0.95761	0.16913
-	SLIT3	5	168651733	169336129	2173	200	10164	0.95754	0.16915
+	NCBP1	9	97598423	97683748	258	10	10164	0.95749	0.16916
-	SLC30A1	1	211565039	211613757	156	33	10164	0.95724	0.16922
-	PCYT1A	3	196228382	196322713	234	13	10164	0.95695	0.1693
-	STX1A	7	73689205	73754702	71	13	10164	0.95662	0.16938
+	MBD5	2	147986011	148523477	1090	47	10164	0.95644	0.16943
+	OIT3	10	72858556	72943036	84	12	10164	0.95641	0.16943
+	PGAP2	11	3762724	3836371	122	14	10164	0.95636	0.16945
-	GPR135	14	59442773	59500396	198	14	10164	0.95627	0.16947
-	SELM	22	31094777	31142565	164	30	10164	0.95628	0.16947

STRAND	SYMBOL	CHR	START	STOP	NSNPS	NPARAM	N	ZSTAT	P
-	HERPUD2	7	35622659	35730187	210	16	10164	0.95582	0.16958
+	BBOX1	11	27005705	27137807	409	32	10164	0.9558	0.16959
-	SLCO2A1	3	133922696	134065076	419	68	10164	0.95559	0.16964
+	LGALS7B	19	38754210	38801754	229	23	10164	0.95558	0.16964
-	TRIM62	1	33135399	33217070	196	14	10164	0.95533	0.1697
-	C10orf90	10	126414997	126706038	415	50	10164	0.95526	0.16972
+	CLPSL1	6	35746054	35798064	326	39	10164	0.95502	0.16978
-	LAMA1	18	6931744	7152814	344	36	10164	0.95501	0.16979
+	MRC1	10	17774343	17921162	516	58	10164	0.95497	0.1698
-	TRMT2A	22	20101866	20152295	190	17	10164	0.95492	0.16981
+	SAP30L	5	154410957	154471053	196	34	10164	0.95471	0.16986
-	LOC643802	16	53359362	53406129	119	20	10164	0.95461	0.16989
-	ZNF329	19	58116328	58190110	196	15	10164	0.95455	0.1699
+	TBC1D19	4	26548924	26779771	209	15	10164	0.95414	0.17001
+	MTRR	5	7816186	7911124	249	35	10164	0.95399	0.17005
+	ZNF578	19	52418506	52526878	367	11	10164	0.95399	0.17005
-	DEFB121	20	31394845	31454068	135	8	10164	0.95367	0.17012
+	CD300A	17	74431366	74494798	131	20	10164	0.95361	0.17014
-	PIAS3	1	145838522	145894081	81	9	10164	0.95345	0.17018
-	KBTBD4	11	47562197	47614015	49	10	10164	0.95338	0.1702
-	ACTL7B	9	108844588	108890995	250	33	10164	0.95309	0.17027
+	LOC1053723	19	20323181	20381817	108	5	10164	0.9528	0.17034
-	C3orf36	3	133918145	133964812	125	24	10164	0.95258	0.1704
+	NECAP2	1	16405672	16470090	106	13	10164	0.9525	0.17042
+	PRSS48	4	151241779	151301611	196	16	10164	0.95234	0.17046
+	B3GNT3	19	17759828	17823576	139	18	10164	0.95196	0.17056
+	WDR20	14	102104842	102234839	260	23	10164	0.95178	0.1706
-	TPR	1	186301652	186410848	261	24	10164	0.95177	0.17061
+	RHNO1	12	2841258	2899525	158	12	10164	0.95157	0.17066
+	CRELD2	22	49883630	49937540	316	14	10164	0.95143	0.17069
-	MED19	11	57693709	57747438	116	19	10164	0.95143	0.17069
+	CEP350	1	179919773	180124880	438	17	10164	0.95114	0.17077
-	KRTAP5-3	11	1597565	1643463	291	27	10164	0.95111	0.17077
+	TTC38	22	46232964	46304008	132	3	10164	0.95091	0.17082
+	SCGB2A1	11	62173668	62223939	218	23	10164	0.95079	0.17085
+	MYL9	20	36506484	36559823	39	17	10164	0.95034	0.17097
-	GSDMC	8	129695903	129821888	496	34	10164	0.95031	0.17098
-	KRTAP27-1	21	30327013	30372694	212	18	10164	0.95015	0.17102
-	IL27	16	28489069	28561399	114	9	10164	0.95009	0.17103
+	FN3K	17	82700576	82761197	255	25	10164	0.95006	0.17104
-	C5orf67	5	56501394	56641232	460	69	10164	0.94966	0.17114
-	ZNF563	19	12307477	12383130	93	11	10164	0.94929	0.17124
+	HERC3	4	88557423	88719297	442	24	10164	0.94918	0.17126
+	GATAD1	7	92412448	92501328	189	28	10164	0.94898	0.17132
-	C1orf127	1	10936473	11017037	185	36	10164	0.94852	0.17143
+	FFAR2	19	35414715	35461767	170	37	10164	0.94842	0.17146
-	CENPJ	13	24872274	24957889	452	27	10164	0.94836	0.17147
-	PISD	22	31608491	31697564	309	21	10164	0.94833	0.17148
+	TMCO6	5	140561540	140657248	188	17	10164	0.94832	0.17148
-	PREP	6	105267567	105438124	429	47	10164	0.94822	0.17151
+	DFNB59	2	178415592	178471390	56	7	10164	0.94744	0.17171
-	APOL3	22	36130323	36201177	277	31	10164	0.94699	0.17182
+	MXD1	2	69880041	69952945	212	25	10164	0.94685	0.17186
-	OSER1	20	44185939	44245906	218	27	10164	0.94654	0.17194
+	GLE1	9	128469692	128552301	134	10	10164	0.94635	0.17199
-	RPL37	5	40821328	40870285	179	22	10164	0.94625	0.17201
+	CCR9	3	45851504	45913177	151	20	10164	0.94622	0.17202
+	OR2L13	1	247902029	248110922	538	27	10164	0.94515	0.17229
-	C5orf60	5	179631542	179680167	122	22	10164	0.94439	0.17248
-	AKAP10	17	19894452	20012856	337	12	10164	0.94432	0.1725
-	CEBPE	14	23107306	23154985	94	29	10164	0.94409	0.17256
+	VPS25	17	42738436	42789600	89	11	10164	0.94403	0.17258
-	MT1HL1	1	236994103	237039418	77	20	10164	0.94325	0.17278

STRAND	SYMBOL	CHR	START	STOP	NSNPS	NPARAM	N	ZSTAT	P
-	STARD5	15	81302666	81359183	162	18	10164	0.94321	0.17279
+	OR14J1	6	29271690	29317655	217	33	10164	0.94296	0.17285
-	ZBTB7C	18	48016864	48444422	1031	121	10164	0.94238	0.173
+	DEFB123	20	31405519	31460257	121	8	10164	0.94231	0.17302
-	ZKSCAN2	16	25226001	25292889	243	8	10164	0.94198	0.1731
+	FNTB	14	64951789	65072652	285	38	10164	0.94192	0.17312
-	OR7A10	19	14830948	14876877	161	19	10164	0.94166	0.17318
-	OR8B8	11	124430150	124476085	200	23	10164	0.94113	0.17332
-	PLXND1	3	129545213	129641739	306	26	10164	0.94088	0.17338
+	OR6C3	12	55296701	55342636	150	18	10164	0.94077	0.17341
+	DGKZ	11	46297905	46390554	137	24	10164	0.94003	0.1736
+	LOC1053723	19	35412902	35458862	165	35	10164	0.93991	0.17363
+	KCTD16	5	144135760	144495686	782	43	10164	0.93989	0.17364
-	C9orf50	9	129602225	129657275	73	13	10164	0.93974	0.17368
-	PLA2G7	6	46694316	46770701	131	23	10164	0.93944	0.17375
-	ZBTB14	18	5279019	5332053	83	8	10164	0.93935	0.17378
+	BIRC3	11	102282373	102349403	172	20	10164	0.9392	0.17381
-	MCTP1	5	94693690	95319575	1399	114	10164	0.93919	0.17382
+	DPCD	10	101553332	101619653	149	23	10164	0.93903	0.17386
+	KIAA1958	9	112451968	112675311	589	37	10164	0.93893	0.17388
+	PRTN3	19	805965	858175	58	8	10164	0.93859	0.17397
+	PRELID1	5	177268762	177316959	85	9	10164	0.93858	0.17397
+	MOV10	1	112639312	112710746	131	15	10164	0.93816	0.17408
-	MMP3	11	102825797	102878611	192	33	10164	0.93809	0.1741
-	C3orf84	3	49167539	49227181	111	13	10164	0.93792	0.17414
-	DNLZ	9	136351900	136398811	122	9	10164	0.93749	0.17425
+	HIST1H2BD	6	26122843	26181349	272	42	10164	0.93723	0.17432
+	FGB	4	154527980	154582763	131	8	10164	0.93721	0.17433
-	GRIFIN	7	2464636	2511855	77	8	10164	0.93675	0.17444
-	LDB2	4	16491534	16933809	912	93	10164	0.93673	0.17445
+	LOC1053709	15	93171194	93216739	171	28	10164	0.93665	0.17447
-	ITGA5	12	54385261	54454266	78	18	10164	0.93642	0.17453
+	CDKN2AIP	4	183409635	183459064	165	10	10164	0.93643	0.17453
-	STARD13	13	33093135	33711835	1751	138	10164	0.9364	0.17453
+	VCL	10	73963078	74130160	281	28	10164	0.93621	0.17458
+	HIVEP1	6	11972653	12174999	619	47	10164	0.93595	0.17465
-	GBP4	1	89171148	89233950	104	11	10164	0.93582	0.17468
-	ANGPTL2	9	127077349	127157797	126	16	10164	0.93576	0.1747
+	SLX4IP	20	10399547	10648546	732	43	10164	0.93531	0.17481
-	LRRC75B	22	24575623	24628104	99	11	10164	0.93486	0.17493
-	CSNK1D	17	82229725	82308742	15	3	10164	0.93467	0.17498
+	SLC44A2	19	10567445	10654559	276	35	10164	0.93448	0.17503
+	C17orf80	17	73197233	73258959	213	9	10164	0.93405	0.17514
+	SEPW1	19	47743585	47794682	80	7	10164	0.93393	0.17517
+	OR4D2	17	58134656	58180579	134	20	10164	0.93323	0.17535
+	ACAN	15	88768442	88885354	74	14	10164	0.93312	0.17538
-	HSPA1L	6	31799619	31856999	269	73	10164	0.93273	0.17548
+	TMEM61	1	54945595	55002293	166	14	10164	0.93272	0.17548
+	ERC1	12	956208	1505933	1686	50	10164	0.93271	0.17549
+	SIX6	14	60474220	60521807	91	7	10164	0.93257	0.17552
-	SNTB1	8	120525745	120847069	879	60	10164	0.93203	0.17566
+	CRACR2B	11	791144	841991	164	21	10164	0.932	0.17567
+	RGL4	22	23655861	23709705	217	16	10164	0.9319	0.17569
-	SLITRK1	13	83867205	83917393	210	26	10164	0.93181	0.17572
+	DCAF11	14	24079697	24135242	73	11	10164	0.93169	0.17575
+	N4BP3	5	178078506	178137034	160	19	10164	0.93158	0.17578
-	TUBB2A	6	3143666	3192549	102	11	10164	0.93116	0.17589
+	USP18	22	18114959	18187397	18	3	10164	0.93111	0.1759
-	TARP	7	38247879	38340646	106	7	10164	0.93083	0.17597
+	ASF1A	6	118859077	118919171	146	10	10164	0.93066	0.17601
+	FGF7	15	49388178	49497326	332	21	10164	0.93063	0.17602
-	GSTA3	6	52886639	52944798	158	10	10164	0.93052	0.17605
-	PRKRA	2	178421414	178486231	70	10	10164	0.92984	0.17623

STRAND	SYMBOL	CHR	START	STOP	NSNPS	NPARAM	N	ZSTAT	P
-	CNR2	1	23863463	23948327	270	16	10164	0.92974	0.17625
+	APOBEC3B	22	38947399	39002779	90	19	10164	0.92976	0.17625
+	SREK1	5	66109218	66193616	112	9	10164	0.92973	0.17626
+	INPPL1	11	72188866	72249147	168	30	10164	0.92957	0.1763
-	C1orf234	1	23000834	23050850	57	6	10164	0.9295	0.17631
+	LOC1019280	5	172720503	172772565	148	28	10164	0.92947	0.17632
+	RPL35A	3	197915181	197965851	119	10	10164	0.92908	0.17642
+	TJP2	9	69086264	69265208	661	49	10164	0.92909	0.17642
-	LHFPL4	3	9488361	9588802	255	24	10164	0.92909	0.17642
+	SLC24A1	15	65576405	65671242	179	13	10164	0.929	0.17645
-	GOLGA8B	15	34515276	34618651	178	15	10164	0.92896	0.17646
-	SEC61G	7	54742247	54794246	163	9	10164	0.92822	0.17665
-	DCTPP1	16	30413698	30465052	17	3	10164	0.92821	0.17665
+	RARB	3	25139332	25607932	1600	107	10164	0.92789	0.17673
-	FBXW11	5	171851549	172041873	17	6	10164	0.92788	0.17673
+	HMSD	18	63914250	63971821	183	11	10164	0.92775	0.17677
-	SPDYE6	7	102335752	102392047	39	7	10164	0.92757	0.17681
+	ZNHIT3	17	36451627	36509310	40	8	10164	0.9275	0.17683
-	CYP11B2	8	142900558	142952862	15	6	10164	0.92736	0.17687
-	GJA9	1	38864067	38916626	138	11	10164	0.92738	0.17687
-	STK10	5	172032070	172223342	399	37	10164	0.92695	0.17698
+	DAPK1	9	87462228	87718634	966	79	10164	0.92663	0.17706
+	NASP	1	45548988	45628906	287	21	10164	0.92651	0.17709
+	LOC1019300	7	35659329	35709413	107	10	10164	0.92651	0.17709
-	ZBTB12	6	31889204	31937367	223	62	10164	0.9264	0.17712
+	OR4D9	11	59479913	59525857	80	9	10164	0.92632	0.17714
-	PAFAH1B3	19	42287033	42337800	38	4	10164	0.92615	0.17718
+	DDIT4	10	72238919	72286039	114	12	10164	0.92606	0.17721
-	C16orf91	16	1409744	1455800	117	16	10164	0.92606	0.17721
+	LOC1010601	11	45178066	45224744	127	19	10164	0.92548	0.17736
+	KDM1A	1	22984443	23093691	128	8	10164	0.92527	0.17741
+	MINOS1-NBL	1	19561977	19668456	305	26	10164	0.92519	0.17743
+	ASCL4	12	107739385	107786644	138	17	10164	0.92507	0.17746
+	SH3BP1	22	37604677	37666043	217	19	10164	0.92466	0.17757
-	LOC1053692	19	15845340	15898863	255	17	10164	0.9244	0.17764
+	FAM175B	10	124766785	124846670	199	42	10164	0.92437	0.17765
-	PARL	3	183816488	183919905	351	21	10164	0.92425	0.17768
-	PEF1	1	31619862	31680237	90	9	10164	0.9242	0.17769
-	RUNX1	21	34777801	35084310	530	79	10164	0.92378	0.1778
+	GPR78	4	8545237	8600023	84	15	10164	0.92375	0.17781
-	PCED1A	20	2825314	2876243	179	22	10164	0.92374	0.17781
-	PPAP2A	5	55414842	55570370	401	15	10164	0.92323	0.17794
+	EXOC6	10	92799713	93069494	544	29	10164	0.92323	0.17794
+	YAP1	11	102075254	102243423	392	22	10164	0.92286	0.17804
+	ANKRD42	11	83158739	83266099	234	28	10164	0.92286	0.17804
+	MMP11	22	23737849	23794316	205	26	10164	0.92248	0.17814
-	TFEB	6	41673978	41771259	273	48	10164	0.92228	0.17819
+	HIST1H3C	6	26010411	26055869	184	35	10164	0.92224	0.1782
-	ZFAND5	9	72341425	72400247	95	6	10164	0.92185	0.1783
-	KCNG4	16	84211135	84274750	346	62	10164	0.92179	0.17832
+	CD151	11	797952	848835	170	21	10164	0.92164	0.17836
+	FBLN7	2	112103385	112198218	143	20	10164	0.92133	0.17844
-	PRKDC	8	47763108	47995183	249	45	10164	0.921	0.17853
-	TFF3	21	42301667	42350596	60	12	10164	0.92092	0.17855
-	POLE2	14	49633552	49723417	202	13	10164	0.92075	0.17859
-	ENPP5	6	46149182	46206010	132	20	10164	0.92069	0.17861
+	EWSR1	22	29233009	29310525	217	36	10164	0.92055	0.17864
+	ZNF486	19	20132214	20210490	123	4	10164	0.92029	0.17871
-	GTSF1	12	54445952	54508602	144	14	10164	0.92006	0.17877
-	PHLDA2	11	2918273	2964420	114	13	10164	0.91976	0.17885
+	ISLR	15	74138746	74186871	60	23	10164	0.91959	0.17889
-	PENK	8	56430954	56481723	182	19	10164	0.91945	0.17893
-	PKD1	16	2078708	2170898	60	35	10164	0.91915	0.17901

STRAND	SYMBOL	CHR	START	STOP	NSNPS	NPARAM	N	ZSTAT	P
-	ETNK2	1	204121061	204187182	167	28	10164	0.91897	0.17906
+	JKAMP	14	59449443	59515407	213	13	10164	0.91878	0.1791
+	EFCC1	3	128966251	129050742	159	20	10164	0.9185	0.17918
-	ARF4	3	57561363	57632488	146	14	10164	0.91823	0.17925
+	HIPK1	1	113894197	113987869	208	33	10164	0.91731	0.17949
-	CDPF1	22	46234013	46285679	98	3	10164	0.91726	0.1795
-	ERCC3	2	127247290	127329176	127	14	10164	0.91699	0.17957
-	HIST1H1A	6	26007032	26052812	185	35	10164	0.91624	0.17977
+	LOC1053714	17	319716	367581	78	17	10164	0.91604	0.17982
-	LOC1053788	1	74469955	74572447	176	21	10164	0.91536	0.18
+	PCDHB10	5	141157379	141205640	147	14	10164	0.91534	0.18001
-	RECQL4	8	144501284	144552827	43	6	10164	0.91516	0.18005
-	NSMF	9	137437570	137494357	40	15	10164	0.91504	0.18009
+	ACOXL	2	110697539	111130610	1323	77	10164	0.91498	0.1801
+	ZNF431	19	21107004	21196002	239	18	10164	0.91493	0.18011
+	SUCO	1	172497349	172621833	236	19	10164	0.91477	0.18016
-	SLC38A4	12	46754761	46867422	395	37	10164	0.91436	0.18026
-	MESP1	15	89739867	89786309	145	7	10164	0.91433	0.18027
-	PTN	7	137217341	137378860	354	40	10164	0.91431	0.18028
-	WBSCR27	7	73824590	73877525	94	3	10164	0.91422	0.1803
-	B4GALNT1	12	57615895	57668239	155	14	10164	0.91416	0.18032
-	PTGER3	1	70842353	71082814	662	51	10164	0.91298	0.18063
+	CELA2B	1	15441101	15501400	212	23	10164	0.9129	0.18065
-	KRTAP19-6	21	30531535	30576864	149	12	10164	0.91279	0.18068
+	MTCL1	18	8670578	8842778	351	44	10164	0.9124	0.18078
-	ARL5A	2	151790966	151863495	146	28	10164	0.91234	0.18079
-	TMEM110	3	52826756	52932581	363	19	10164	0.91215	0.18084
+	NOTO	2	73167258	73221212	122	9	10164	0.9115	0.18102
+	RPRD2	1	150329111	150486566	568	22	10164	0.91129	0.18107
+	RAB6C	2	129944662	129992738	84	6	10164	0.91102	0.18114
-	SLC16A9	10	59640764	59744891	227	33	10164	0.91085	0.18119
-	EDC3	15	74620558	74731045	177	40	10164	0.91003	0.1814
+	OR5C1	9	122753933	122799895	45	14	10164	0.90996	0.18142
+	MGMT	10	129432190	129778042	914	35	10164	0.90968	0.1815
+	TMC8	17	78093851	78152968	195	43	10164	0.90933	0.18159
+	ACAD9	3	128844490	128923114	264	19	10164	0.90912	0.18164
-	TRPV4	12	109773087	109868407	215	29	10164	0.90902	0.18167
-	CCDC90B	11	83249093	83321407	180	16	10164	0.90872	0.18175
-	NFU1	2	69386113	69472628	264	21	10164	0.90843	0.18183
+	GPSM1	9	136292476	136369605	89	10	10164	0.90822	0.18188
+	DRD4	11	602305	650706	162	20	10164	0.90798	0.18194
-	OR52A5	11	5121692	5167642	122	17	10164	0.9079	0.18197
+	LOC1002895	7	102328861	102390633	64	9	10164	0.9075	0.18207
-	MTIF3	13	27425639	27485854	184	18	10164	0.90741	0.18209
+	ZNF26	12	132951348	133022568	154	12	10164	0.90723	0.18214
-	KCP	7	128858934	128945719	226	32	10164	0.90721	0.18215
-	SPC24	19	11135498	11191002	203	36	10164	0.90718	0.18216
-	C9orf72	9	27536546	27608866	240	33	10164	0.90664	0.1823
+	EIF5A	17	7271999	7322463	117	21	10164	0.90656	0.18232
-	LOC1019285	7	56816850	56862843	47	7	10164	0.90608	0.18245
-	VAX1	10	117118521	117173301	101	16	10164	0.90586	0.1825
-	LZTS3	20	3152617	3208592	119	16	10164	0.90575	0.18253
+	FABP6	5	160152367	160248722	323	19	10164	0.90574	0.18254
-	HBB	11	5215466	5262071	161	19	10164	0.90556	0.18258
+	SLC25A4	4	185108263	185160384	164	18	10164	0.90549	0.1826
+	GSDMA	17	39927973	39987880	215	20	10164	0.90552	0.1826
+	ARL4A	7	12651827	12700934	188	25	10164	0.90528	0.18266
+	BRMS1L	14	35791318	35881963	214	25	10164	0.90515	0.18269
+	ABHD6	3	58202506	58304738	222	10	10164	0.90502	0.18273
-	SSRP1	11	57315985	57370877	87	13	10164	0.90495	0.18275
+	TNRC6C	17	77969170	78118835	329	20	10164	0.90426	0.18293
+	GAS2L3	12	100538646	100638288	285	29	10164	0.90422	0.18294
+	NDUFAF5	20	13750026	13828421	204	25	10164	0.90404	0.18299

STRAND	SYMBOL	CHR	START	STOP	NSNPS	NPARAM	N	ZSTAT	P
-	C1orf110	1	162836566	162903900	311	30	10164	0.90397	0.18301
-	REEP1	2	86203993	86373418	528	26	10164	0.9037	0.18308
+	UPF3A	13	114246584	114315817	215	24	10164	0.90345	0.18314
+	OR4D1	17	58120154	58166086	114	12	10164	0.90263	0.18336
+	LOC1027249	11	15588774	15715368	248	17	10164	0.90196	0.18354
+	FOXQ1	6	1277440	1324758	92	18	10164	0.9019	0.18355
-	IRF7	11	602553	650999	164	20	10164	0.90142	0.18368
+	FST	5	53445341	53497134	184	32	10164	0.90143	0.18368
+	PAFAH1B1	17	2558629	2695617	247	18	10164	0.9007	0.18387
-	FERMT2	14	52847271	52986097	391	33	10164	0.9002	0.18401
-	KCTD19	16	67279432	67361761	88	17	10164	0.90019	0.18401
-	LOC1053737	2	184583627	184633985	132	24	10164	0.90017	0.18401
+	PCDHB11	5	141164775	141213045	142	15	10164	0.90013	0.18403
+	HNF1A	12	120942787	121012512	264	35	10164	0.90004	0.18405
+	MESP2	15	89741358	89788754	149	7	10164	0.90001	0.18406
+	PRAC2	17	48685809	48734758	171	34	10164	0.89989	0.18409
+	KCNS2	8	98392022	98440797	76	15	10164	0.89939	0.18422
+	LOC1053739	2	96834182	96880390	88	25	10164	0.89938	0.18423
+	SMC4	3	160364304	160444962	161	22	10164	0.89932	0.18424
+	ASIP	20	34151493	34279344	189	20	10164	0.89923	0.18426
-	TMEM110-MI	3	52823115	52932581	380	19	10164	0.89914	0.18429
-	CSMD3	8	112212928	113472467	3143	66	10164	0.89897	0.18434
-	ESAM	11	124743123	124797327	90	16	10164	0.89881	0.18438
+	TRIB3	20	345629	407559	63	19	10164	0.89814	0.18456
+	TEX35	1	178478077	178533500	111	20	10164	0.89801	0.18459
+	IAH1	2	9439532	9527208	167	10	10164	0.89801	0.18459
+	RBMS3	3	29245860	30020395	3033	179	10164	0.89795	0.18461
-	ZNF780A	19	40059152	40126736	184	6	10164	0.89789	0.18462
+	COX4I2	20	31602879	31655009	74	8	10164	0.89757	0.18471
-	LOC1019271	2	239869393	239948187	349	34	10164	0.89745	0.18474
+	HECA	6	139100112	139190809	223	16	10164	0.89738	0.18476
-	MMEL1	1	2580639	2668042	175	9	10164	0.89736	0.18476
+	ACTR6	12	100165087	100234424	143	11	10164	0.89686	0.1849
-	VSIG8	1	159844316	159897657	117	14	10164	0.89675	0.18493
-	CNTFR	9	34541432	34625140	247	35	10164	0.8966	0.18497
-	RABL3	3	120676681	120777537	166	19	10164	0.89635	0.18503
-	KRTAP23-1	21	30338399	30383606	183	19	10164	0.89629	0.18505
+	TNFAIP1	17	28300522	28357009	71	16	10164	0.89614	0.18509
+	DERA	12	15876172	16047381	362	33	10164	0.89605	0.18511
-	CPNE1	20	35616031	35699956	215	18	10164	0.89575	0.18519
+	SMTN	22	31029180	31114624	313	34	10164	0.89565	0.18522
-	SPRN	10	133410666	133459617	104	10	10164	0.89529	0.18532
-	NOP10	15	34331716	34378161	92	9	10164	0.89518	0.18535
+	ZBTB18	1	244013939	244067476	17	3	10164	0.89493	0.18541
+	ESRP1	8	94606136	94717466	404	34	10164	0.89464	0.18549
+	DHX30	3	47767909	47860196	110	17	10164	0.89442	0.18555
-	RBM12	20	35638925	35699956	155	15	10164	0.8943	0.18558
-	LOC1027242	19	15841993	15899904	265	18	10164	0.89426	0.18559
+	KIAA1033	12	105072714	105179135	293	18	10164	0.89411	0.18563
+	MRM1	17	36566581	36644698	35	7	10164	0.89339	0.18582
-	MYLK4	6	2653629	2805123	583	44	10164	0.89333	0.18584
+	ARID3B	15	74506177	74608131	245	24	10164	0.8931	0.1859
+	CST7	20	24914230	24969928	315	11	10164	0.89289	0.18596
+	CNNM3	2	96779952	96847466	136	24	10164	0.89286	0.18597
-	INTS4	11	77868720	78029842	443	30	10164	0.89284	0.18597
+	TEX12	11	112132372	112182556	103	13	10164	0.89239	0.18609
-	PDGFB	22	39213680	39279952	148	20	10164	0.89235	0.1861
+	TMEM87B	2	112020223	112129318	194	21	10164	0.8922	0.18614
-	HBS1L	6	134950378	135089898	272	11	10164	0.89144	0.18635
+	PCDHA1	5	140751136	141022344	520	21	10164	0.89143	0.18635
-	KRT79	12	52811447	52869293	291	25	10164	0.89126	0.18639
-	MANBA	4	102621486	102795994	371	25	10164	0.89017	0.18669
-	POMC	2	25150853	25203871	121	16	10164	0.88934	0.18691

STRAND	SYMBOL	CHR	START	STOP	NSNPS	NPARAM	N	ZSTAT	P
-	PAF1	19	39375629	39426195	43	11	10164	0.88903	0.18699
-	WDR72	15	53503741	53797878	1306	59	10164	0.88841	0.18716
-	VWA9	15	65568757	65646289	150	10	10164	0.88814	0.18723
-	SEL1L3	4	25711867	25898595	208	35	10164	0.88809	0.18725
+	WBSCR28	7	73826159	73875894	90	3	10164	0.88735	0.18745
+	CARD6	5	40806308	40865354	231	25	10164	0.88698	0.18755
-	GBP7	1	89121751	89211040	169	15	10164	0.88659	0.18765
-	AP4B1	1	113884748	113940206	123	27	10164	0.88651	0.18767
-	IQCF1	3	51884876	51938370	89	16	10164	0.88645	0.18769
+	LOC400682	19	21602371	21667708	609	16	10164	0.88637	0.18771
-	OR51V1	11	5189735	5235700	214	23	10164	0.88633	0.18772
+	TPRG1	3	189064459	189333482	277	30	10164	0.88584	0.18785
-	PIP4K2A	10	22524837	22749574	698	34	10164	0.88529	0.188
-	GIPC1	19	14467759	14531149	218	35	10164	0.8853	0.188
+	LOC1053777	5	181260347	181328407	115	10	10164	0.88516	0.18804
+	RAMP3	7	45122768	45194250	362	22	10164	0.88496	0.18809
+	LOC1019280	17	67208959	67282108	103	10	10164	0.88483	0.18812
+	TRAPPC5	19	7645821	7692862	90	20	10164	0.88419	0.1883
-	IFT80	3	160246986	160434532	314	26	10164	0.88412	0.18831
+	REG3G	2	78990686	79038504	320	19	10164	0.88415	0.18831
+	MADCAM1	19	461490	515343	15	3	10164	0.88376	0.18841
-	ODF3L2	19	453346	509983	15	3	10164	0.88376	0.18841
+	KCNK2	1	214967639	215247093	790	33	10164	0.88341	0.18851
-	RASGEF1C	5	180090795	180244130	683	90	10164	0.88326	0.18855
+	RPS7	2	3540263	3590919	161	14	10164	0.88307	0.1886
-	ZNF45	19	43902624	43970280	225	22	10164	0.88308	0.1886
+	C4orf48	4	2000608	2053970	85	12	10164	0.88302	0.18861
-	SIGMAR1	9	34624722	34672826	113	21	10164	0.88267	0.18871
-	BCL7B	7	73526353	73592735	19	6	10164	0.88257	0.18873
-	MC2R	18	13872044	13950707	327	19	10164	0.88235	0.18879
-	UBL7	15	74435977	74496188	165	14	10164	0.88231	0.1888
-	PIDD1	11	789179	844501	180	23	10164	0.8823	0.18881
+	LRRC36	16	67291844	67395206	106	19	10164	0.8821	0.18886
+	SFXN3	10	100996229	101051241	120	29	10164	0.88155	0.18901
+	SH3BGR	21	39410856	39525506	291	20	10164	0.88152	0.18902
+	NIT2	3	100299718	100365634	151	26	10164	0.88115	0.18912
+	GGNBP2	17	36509888	36599848	56	11	10164	0.88099	0.18916
+	OSTN	3	191164142	191273338	479	35	10164	0.88097	0.18917
-	WDR46	6	33269103	33324527	211	41	10164	0.88089	0.18919
-	KLK6	19	50948631	51004673	215	42	10164	0.87993	0.18945
+	HIST1H2AH	6	27112082	27157562	110	33	10164	0.8794	0.18959
+	ZACN	17	76044182	76092804	69	3	10164	0.8792	0.18965
-	H2AFV	7	44816889	44883126	145	12	10164	0.8785	0.18984
+	KCNN1	19	17916302	18009121	173	24	10164	0.87844	0.18985
+	CDK9	9	127751026	127800773	159	23	10164	0.87832	0.18989
+	PPAPDC2	9	4627294	4675274	331	31	10164	0.87815	0.18993
+	TNNI2	11	1804003	1851680	203	37	10164	0.87791	0.19
-	EPSTI1	13	42876388	43027271	522	61	10164	0.87767	0.19006
+	C2orf50	2	11098053	11164564	194	17	10164	0.87746	0.19012
+	LOC1053751	6	32931705	32982249	372	38	10164	0.87727	0.19017
+	CYFIP2	5	157231080	157405598	463	25	10164	0.87704	0.19023
-	ITGB1	10	32890318	32993365	305	26	10164	0.87702	0.19024
+	TBC1D31	8	123037680	123175173	282	31	10164	0.87676	0.19031
+	KIAA0391	14	35086283	35284078	604	33	10164	0.87671	0.19032
-	GALNT18	11	11260874	11657045	1607	195	10164	0.87672	0.19032
-	LRRC34	3	169783006	169847866	179	23	10164	0.87656	0.19036
+	VTI1A	10	112411998	112865364	672	77	10164	0.87623	0.19045
+	SEMA3F	3	50120063	50199075	174	28	10164	0.87607	0.1905
-	TMEM40	3	12723530	12804457	315	23	10164	0.87597	0.19052
-	BCHE	3	165762904	165872472	298	18	10164	0.875	0.19079
-	ZNF573	19	37725131	37814636	242	36	10164	0.87499	0.19079
+	ZNF727	7	64009801	64095184	355	16	10164	0.87485	0.19083
+	RUFY1	5	179515561	179620026	292	23	10164	0.87473	0.19086

STRAND	SYMBOL	CHR	START	STOP	NSNPS	NPARAM	N	ZSTAT	P
-	L3HYPDH	14	59455511	59519438	216	13	10164	0.87456	0.19091
+	LRP1	12	57093499	57223359	267	40	10164	0.87439	0.19095
+	SPSB1	1	9257882	9379532	337	43	10164	0.8742	0.19101
+	C1QTNF9B-A	13	23853889	23902103	124	10	10164	0.874	0.19106
+	ICAM1	19	10235841	10296615	157	31	10164	0.87363	0.19116
+	HGFAC	4	3406933	3459495	196	34	10164	0.87342	0.19122
-	FAM170B	10	49121154	49169020	208	20	10164	0.87328	0.19125
-	POU2AF1	11	111342251	111414432	246	22	10164	0.87326	0.19126
+	LOC1027247	1	12995809	13042199	1	1	10164	0.87289	0.19136
-	GGACT	13	100520164	100623792	237	30	10164	0.8725	0.19147
-	CASKIN2	17	75490260	75550583	182	18	10164	0.87216	0.19156
-	HBG1	11	5238272	5284857	102	8	10164	0.87164	0.1917
+	LIPG	18	49525699	49602908	236	36	10164	0.87159	0.19172
-	LOC1053778	13	18952129	19054869	393	16	10164	0.87156	0.19172
-	HLA-DOA	6	32994182	33044612	485	51	10164	0.87152	0.19173
+	ENTPD3	3	40352156	40438626	195	13	10164	0.87151	0.19174
+	MRPS2	9	135464986	135514673	120	16	10164	0.87135	0.19178
+	CTLA4	2	203832788	203883960	106	12	10164	0.87132	0.19179
+	OR6C74	12	55204762	55258226	176	11	10164	0.87121	0.19182
-	ZBTB11	3	101639439	101712144	149	22	10164	0.87087	0.19191
+	CLIP2	7	74254475	74415943	31	6	10164	0.87081	0.19193
-	LSM7	19	2311521	2363615	149	17	10164	0.87077	0.19194
+	SMPD1	11	6355301	6404998	58	9	10164	0.87061	0.19198
+	PRR19	19	42267132	42320821	45	3	10164	0.87058	0.19199
-	HAAO	2	42757089	42828138	325	34	10164	0.87038	0.19205
+	PRPF3	1	150286479	150363228	256	17	10164	0.87015	0.19211
-	TNFRSF13B	17	16929084	17007088	293	32	10164	0.87005	0.19214
-	SLC28A3	9	84265850	84403632	562	46	10164	0.87005	0.19214
-	MACC1	7	20124655	20252390	360	21	10164	0.87004	0.19214
+	HSH2D	19	16099028	16168575	84	6	10164	0.8698	0.19221
-	ANKRD23	2	96827914	96879021	98	27	10164	0.86968	0.19224
-	C15orf59	15	73725431	73788187	173	23	10164	0.8693	0.19234
-	TPPP	5	649862	728395	281	35	10164	0.86912	0.19239
+	EGF	4	109877883	110022962	517	32	10164	0.86904	0.19241
-	RAB7B	1	205966740	206038495	15	8	10164	0.86892	0.19245
-	MATN4	20	45283446	45343566	191	24	10164	0.86844	0.19258
+	CISD1	10	58234102	58299259	186	4	10164	0.86817	0.19265
+	SLC35A5	3	112526709	112594439	192	11	10164	0.86787	0.19273
-	PRB4	12	11297081	11345435	67	14	10164	0.86738	0.19287
-	HOXB3	17	48538870	48625282	303	44	10164	0.86729	0.19289
+	SLC17A6	11	22303121	22389503	178	25	10164	0.86715	0.19293
-	ETF1	5	138496093	138578300	82	7	10164	0.86706	0.19295
-	MRLN	10	59726990	59788471	192	20	10164	0.86696	0.19298
+	LRRIQ4	3	169777947	169848007	185	23	10164	0.86693	0.19299
+	PRSS55	8	10490477	10564166	334	23	10164	0.86639	0.19314
-	ANXA6	5	151090706	151192882	455	57	10164	0.86621	0.19319
-	TFEC	7	115925148	116194891	637	31	10164	0.86606	0.19323
-	BTBD17	17	74346416	74398652	98	24	10164	0.86604	0.19323
-	NOVA1	14	26435883	26632754	15	6	10164	0.86554	0.19337
+	PHF20	20	35737001	35960377	531	21	10164	0.86523	0.19346
-	PHOX2B	4	41734082	41783970	152	11	10164	0.86519	0.19347
-	FEZF2	3	62359672	62408515	34	15	10164	0.86516	0.19348
+	OCLN	5	69457292	69568104	80	11	10164	0.86506	0.1935
-	MXD3	5	177295500	177347291	75	14	10164	0.86473	0.19359
+	PIPOX	17	29007900	29067218	79	12	10164	0.86455	0.19364
-	PLA2G12A	4	109699989	109765086	179	38	10164	0.86453	0.19365
-	FLOT1	6	30717734	30777685	413	43	10164	0.86438	0.19369
+	EML1	14	99702722	99952060	668	50	10164	0.86373	0.19387
-	SMIM12	1	34840362	34894816	68	16	10164	0.86371	0.19387
+	SDCBP	8	58518169	58592860	209	16	10164	0.86362	0.1939
+	PKP3	11	357599	414908	150	16	10164	0.86326	0.194
-	PCBD1	10	70872280	70923786	161	39	10164	0.86307	0.19405
-	CELF4	18	37180090	37601037	877	93	10164	0.86278	0.19413

STRAND	SYMBOL	CHR	START	STOP	NSNPS	NPARAM	N	ZSTAT	P
+	ZNF233	19	44224880	44285317	130	16	10164	0.86268	0.19416
+	PMAIP1	18	59864960	59914306	130	20	10164	0.86258	0.19418
-	DTD2	14	31436036	31492474	175	18	10164	0.86162	0.19445
-	LOC1053692	15	96071411	96187989	246	27	10164	0.86154	0.19447
-	MRPS25	3	15032251	15100311	119	9	10164	0.86138	0.19452
-	LSAMP	3	115792363	116480542	1361	68	10164	0.86116	0.19457
-	YOD1	1	207033849	207088133	89	15	10164	0.86095	0.19463
-	SLA2	20	36602519	36681216	55	21	10164	0.86057	0.19474
+	PDXDC1	16	14939591	15149349	59	11	10164	0.8603	0.19481
+	DISP1	1	222779514	223015995	50	8	10164	0.85976	0.19496
+	BMP8A	1	39456634	39539869	143	17	10164	0.85976	0.19496
+	ZNF549	19	57492081	57550886	245	11	10164	0.85944	0.19505
+	TBX1	22	19721703	19793593	198	32	10164	0.8593	0.19509
-	KBTBD6	13	41117573	41167800	72	13	10164	0.85921	0.19511
+	ESCO2	8	27736477	27822396	358	14	10164	0.85912	0.19514
-	ADAMTS20	12	43342843	43586940	759	32	10164	0.85908	0.19515
+	CWC27	5	64733918	65028763	543	23	10164	0.85902	0.19516
+	FPR3	19	51760158	51836474	267	33	10164	0.85898	0.19518
-	ACSS1	20	24996230	25093182	183	24	10164	0.85888	0.1952
+	SH3TC1	4	8147072	8251103	170	32	10164	0.85842	0.19533
-	PBK	8	27799621	27873095	382	14	10164	0.85809	0.19542
+	LOC1053709	15	83172433	83237217	129	10	10164	0.85792	0.19547
-	FBL	19	39824453	39881414	163	19	10164	0.85788	0.19548
+	TMEM212	3	171808349	171877846	173	30	10164	0.85777	0.19551
+	CCDC140	2	222263147	222315217	87	15	10164	0.85765	0.19554
+	LOC1053749	6	26222024	26272753	210	27	10164	0.85752	0.19558
-	ENHO	9	34511042	34558039	108	24	10164	0.85743	0.1956
+	RIN2	20	19722610	20012459	906	99	10164	0.85736	0.19562
-	POLR2J	7	102463100	102513999	40	5	10164	0.85735	0.19563
+	VPS26B	11	134189605	134257792	95	15	10164	0.85698	0.19573
-	DFNA5	7	24688355	24793020	326	30	10164	0.85683	0.19577
+	RHOBTB3	5	95696066	95806367	227	30	10164	0.8568	0.19578
-	NOB1	16	69731854	69789968	124	15	10164	0.85668	0.19581
+	LOC1019293	7	44835946	44882158	86	8	10164	0.85652	0.19586
-	IPMK	10	58181517	58302937	327	5	10164	0.85646	0.19587
+	OR8A1	11	124535003	124612563	264	32	10164	0.85641	0.19588
-	TMC3	15	81322419	81409077	142	15	10164	0.85618	0.19595
+	VANGL1	1	115606953	115708224	400	43	10164	0.85583	0.19605
-	IPPK	9	92603184	92705265	236	17	10164	0.85524	0.19621
-	RHBDL2	1	38875806	38976830	317	30	10164	0.85514	0.19624
+	LOC1005069	2	127343128	127399411	116	15	10164	0.85507	0.19626
-	PRAM1	19	8480055	8537654	115	7	10164	0.85494	0.19629
+	FYTTD1	3	197714553	197794446	67	3	10164	0.85479	0.19633
+	GPD1L	3	32071511	32178715	486	23	10164	0.85469	0.19636
-	ALDOB	9	101410560	101470780	164	21	10164	0.85458	0.19639
+	PNN	14	39140183	39193218	168	27	10164	0.85455	0.1964
-	LOC1053739	2	110031483	110090097	2	1	10164	0.85457	0.1964
+	APBA2	15	28850994	29128315	517	25	10164	0.85334	0.19673
+	PPP2R2A	8	26256491	26382680	245	34	10164	0.85336	0.19673
+	TMPRSS11E	4	68412449	68507604	115	20	10164	0.85322	0.19677
-	MTPN	7	135916755	136012456	342	24	10164	0.85297	0.19684
-	LUZP6	7	135916755	136012456	342	24	10164	0.85297	0.19684
-	C3orf70	3	185068050	185188014	441	41	10164	0.85261	0.19694
-	SH2D3C	9	127728317	127820159	270	32	10164	0.85253	0.19696
-	MYCBP	1	38852490	38908378	137	12	10164	0.85251	0.19697
+	LCE6A	1	152807854	152853983	43	11	10164	0.85224	0.19704
-	NFS1	20	35658688	35734365	228	18	10164	0.85195	0.19712
+	ILK	11	6568708	6620874	141	25	10164	0.85135	0.19729
-	SRCIN1	17	38520024	38640930	243	29	10164	0.85122	0.19732
-	LXN	3	158656414	158707693	166	11	10164	0.85107	0.19737
+	TPGS1	19	472497	529654	21	5	10164	0.85101	0.19738
-	ASCC1	10	72086032	72252134	379	14	10164	0.85097	0.19739
+	CCDC124	19	17898015	17953985	67	14	10164	0.85091	0.19741

STRAND	SYMBOL	CHR	START	STOP	NSNPS	NPARAM	N	ZSTAT	P
-	PPEF2	4	75849872	75937528	206	30	10164	0.85085	0.19743
-	CHST1	11	45637689	45700656	157	20	10164	0.85081	0.19744
-	LARP4B	10	796914	1023323	459	46	10164	0.85036	0.19756
+	C9orf173	9	137215840	137263483	138	18	10164	0.85024	0.1976
+	MNDA	1	158796378	158859480	137	15	10164	0.8502	0.19761
-	RNF130	5	179910270	180107130	582	47	10164	0.85	0.19766
+	UBE2L3	22	21514447	21634034	136	7	10164	0.84993	0.19768
-	DYRK1B	19	39815347	39869233	165	17	10164	0.84969	0.19775
+	PSMB9	6	32819161	32869851	503	68	10164	0.84959	0.19778
+	SNX21	20	45798622	45853275	176	27	10164	0.84953	0.19779
-	PPY	17	43930804	43979248	90	11	10164	0.84942	0.19782
-	SNRPD2	19	45677454	45727311	115	23	10164	0.8493	0.19786
-	FAM214B	9	35094121	35151351	201	36	10164	0.84925	0.19787
+	PDE12	3	57521252	57664979	301	16	10164	0.84919	0.19789
-	GPRIN2	10	46533963	46591947	2	1	10164	0.84881	0.19799
+	SYT15	10	46543434	46605194	2	1	10164	0.84881	0.19799
-	C1orf210	1	43271865	43320840	40	10	10164	0.8486	0.19805
-	RHEB	7	151456012	151554924	219	27	10164	0.84784	0.19826
+	ATIC	2	215276956	215359773	232	18	10164	0.8478	0.19828
+	LOC1053789	11	90036054	90083512	8	4	10164	0.84757	0.19834
-	LOC388849	22	20138417	20186259	215	30	10164	0.8475	0.19836
+	PCDHA2	5	140759859	141022344	479	20	10164	0.84734	0.1984
+	LOC1053767	16	90067673	90116120	104	12	10164	0.84723	0.19843
+	LY6E	8	142983485	143032410	10	6	10164	0.84701	0.1985
-	SFTPD	10	79927740	80017236	466	17	10164	0.84694	0.19851
-	KIAA1715	2	175913862	176040388	448	37	10164	0.84688	0.19853
-	CRYBA2	2	218980190	219028556	61	20	10164	0.84659	0.19861
-	SDR39U1	14	24429766	24477905	374	14	10164	0.84621	0.19872
+	FAM213B	1	2551460	2601469	108	7	10164	0.84594	0.19879
-	PTK2	8	140648382	141036313	654	17	10164	0.84571	0.19886
-	NOXRED1	14	77384021	77461011	212	17	10164	0.84569	0.19886
+	LARP4	12	50365809	50490005	243	18	10164	0.84507	0.19904
+	LPPR1	9	100993749	101335135	1050	72	10164	0.84455	0.19918
-	CH25H	10	89195937	89242314	119	20	10164	0.84446	0.19921
-	HBG2	11	5243191	5289781	105	10	10164	0.84438	0.19923
+	ITGA9	3	37417322	37829790	977	75	10164	0.84423	0.19927
-	KLHL9	9	21321019	21370430	121	25	10164	0.84407	0.19932
-	DPH3	3	16247061	16299989	183	17	10164	0.84396	0.19935
-	KIAA0226	3	197661393	197784727	88	3	10164	0.8439	0.19936
-	CIDEB	14	24295187	24346377	139	23	10164	0.84386	0.19937
-	TRABD2B	1	47750528	48032371	836	55	10164	0.84388	0.19937
-	HIST1H1D	6	26224212	26269988	182	24	10164	0.84364	0.19943
+	PLEKHA3	2	178445410	178515586	102	13	10164	0.84353	0.19947
-	RECQL	12	21458910	21536669	278	32	10164	0.84317	0.19957
-	C6orf10	6	32278541	32407114	1804	44	10164	0.84285	0.19966
-	UCN2	3	48551718	48598768	40	8	10164	0.84267	0.19971
+	LIMCH1	4	41324607	41710044	443	41	10164	0.84258	0.19973
-	EN1	2	118832171	118883183	141	18	10164	0.8423	0.19981
-	PITPNM3	17	6440344	6591557	381	37	10164	0.84214	0.19985
-	ARHGAP39	8	144519179	144735520	213	12	10164	0.84198	0.1999
+	OR2T4	1	248326581	248372627	71	4	10164	0.84114	0.20014
-	TMEM41A	3	185472671	185534057	113	12	10164	0.84087	0.20021
+	CAMSAP3	19	7560902	7628310	257	39	10164	0.84032	0.20036
+	AKR1A1	1	45515783	45580051	243	22	10164	0.84029	0.20037
-	SIGLEC5	19	51600957	51665474	181	26	10164	0.84022	0.20039
+	FN3KRP	17	82681706	82738017	232	26	10164	0.84018	0.2004
+	OR1L1	9	122626716	122672648	111	21	10164	0.83994	0.20047
-	GNL1	6	30531377	30592594	233	45	10164	0.83987	0.20049
+	LOC1053772	3	75246933	75352180	383	32	10164	0.83987	0.20049
+	CCDC84	11	118959353	119025793	219	17	10164	0.83964	0.20056
+	DHDH	19	48898639	48954969	150	27	10164	0.83952	0.20059
-	ATG9B	7	151002209	151059499	155	37	10164	0.83946	0.20061
-	TST	22	37000859	37055183	112	9	10164	0.83917	0.20069

STRAND	SYMBOL	CHR	START	STOP	NSNPS	NPARAM	N	ZSTAT	P
-	DDX60	4	168206291	168353807	502	22	10164	0.83913	0.2007
+	H1F0	22	37770107	37817436	92	9	10164	0.83909	0.20071
+	IL11RA	9	34617185	34671902	125	22	10164	0.839	0.20073
+	OR2T6	1	248352609	248398535	42	2	10164	0.83899	0.20074
+	NBPF3	1	21400318	21494900	161	16	10164	0.83844	0.20089
+	ASAH2B	10	50704899	50767188	155	13	10164	0.83788	0.20105
-	PINX1	8	10754961	10874899	588	26	10164	0.8374	0.20118
-	STC2	5	173304723	173364503	52	9	10164	0.83737	0.20119
-	C8orf89	8	73231364	73320712	275	39	10164	0.83698	0.2013
+	ZNF544	19	58192783	58273644	209	16	10164	0.83681	0.20135
+	DNAJC25	9	111596352	111664351	265	30	10164	0.83676	0.20136
+	TCEA2	20	64007350	64082347	136	20	10164	0.83662	0.2014
-	DUSP16	12	12463282	12597839	321	35	10164	0.83658	0.20141
-	TSPYL1	6	116264859	116315117	180	17	10164	0.83581	0.20163
-	C21orf91	21	17778967	17854386	109	7	10164	0.83581	0.20163
-	KDM7A	7	140074746	140211956	207	13	10164	0.83521	0.2018
-	FAM150B	2	269561	323880	69	8	10164	0.83498	0.20186
+	IL17RE	3	9867062	9926402	150	18	10164	0.83472	0.20194
-	CLDN5	22	19513024	19560337	146	29	10164	0.83421	0.20208
+	PCDHA3	5	140766198	141022344	464	19	10164	0.834	0.20214
+	COG1	17	73158034	73218507	124	10	10164	0.83385	0.20218
-	DPT	1	168685457	168764204	283	27	10164	0.83366	0.20224
-	ATG3	3	112522507	112596963	207	11	10164	0.83365	0.20224
+	GSTZ1	14	77285887	77341597	153	20	10164	0.83359	0.20226
-	OR7D4	19	9203850	9249871	134	21	10164	0.83359	0.20226
-	PLRG1	4	154524997	154585433	149	9	10164	0.83313	0.20239
+	GMEB1	1	28633722	28725603	108	9	10164	0.83296	0.20243
-	NT5C3A	7	33004113	33097797	304	15	10164	0.83276	0.20249
-	AZIN1	8	102816302	102899200	285	25	10164	0.83272	0.2025
+	PIK3CD	1	9594889	9739114	385	37	10164	0.83247	0.20257
+	C10orf62	10	97554693	97600934	113	15	10164	0.83204	0.20269
+	SP2	17	47861054	47942271	267	37	10164	0.83181	0.20276
-	PAH	12	102828321	102952603	348	21	10164	0.83179	0.20277
+	MYLK2	20	31784312	31844697	107	9	10164	0.83121	0.20293
-	FABP3	1	31350418	31408283	138	12	10164	0.83101	0.20298
+	CRTAM	11	122803431	122882643	246	37	10164	0.831	0.20299
-	CAMKV	3	49847981	49904955	102	14	10164	0.83049	0.20313
+	OVOS	12	9482803	9583297	4	1	10164	0.83021	0.20321
+	ADRBK1	11	67231400	67296558	105	23	10164	0.83018	0.20322
+	C17orf99	17	78111040	78176904	247	49	10164	0.83002	0.20326
+	YLPM1	14	74728322	74847310	234	21	10164	0.82973	0.20335
-	B3GAT2	6	70851366	70992189	398	36	10164	0.82955	0.2034
-	HDGFRP3	15	83105376	83243018	236	15	10164	0.82908	0.20353
+	YBX1	1	42647389	42712363	121	17	10164	0.82879	0.20361
+	GGA1	22	37572235	37643564	257	24	10164	0.82835	0.20374
+	WDR88	19	33097029	33185799	284	35	10164	0.82821	0.20378
+	GAPDH	12	6499405	6548375	168	22	10164	0.82803	0.20383
-	MYCBP2	13	77034657	77362042	663	32	10164	0.82799	0.20384
+	ILF3	19	10619261	10702419	167	16	10164	0.82792	0.20386
-	CDK19	6	110599978	110851516	557	29	10164	0.82787	0.20387
+	RMND5B	5	178095532	178158570	164	18	10164	0.82777	0.2039
+	ZNF804A	2	184563366	184949487	923	31	10164	0.82704	0.20411
+	PQLC2	1	19277246	19342431	238	26	10164	0.82698	0.20412
+	CAPZA1	1	112584453	112681619	166	15	10164	0.82689	0.20415
-	CYP1A1	15	74709542	74760610	151	29	10164	0.82677	0.20418
-	PRR34	22	46040459	46089144	25	6	10164	0.82623	0.20434
+	LOC1053728	21	26360033	26423109	196	41	10164	0.82621	0.20434
-	SLMO2	20	59023145	59077846	66	6	10164	0.82568	0.20449
-	ZGLP1	19	10294803	10344557	157	20	10164	0.82568	0.20449
-	PDZK1	1	145660852	145742400	25	3	10164	0.82553	0.20453
+	NOMO1	16	14798786	14906157	10	4	10164	0.82541	0.20457
-	FAM131C	1	16047769	16108632	284	31	10164	0.82519	0.20463
-	KLF15	3	126332635	126392393	41	15	10164	0.82514	0.20465

STRAND	SYMBOL	CHR	START	STOP	NSNPS	NPARAM	N	ZSTAT	P
+	ACO1	9	32349603	32460836	283	34	10164	0.82445	0.20484
+	ISL1	5	51348124	51404738	107	14	10164	0.82446	0.20484
-	ILVBL	19	15104974	15160799	171	14	10164	0.82433	0.20488
-	TAP1	6	32835209	32888971	552	52	10164	0.82421	0.20491
+	CAST	5	96627037	96784683	407	39	10164	0.82407	0.20495
-	RBM39	20	35693609	35777336	251	14	10164	0.82407	0.20495
+	CD1A	1	158213329	158268269	245	7	10164	0.82363	0.20507
+	MCM4	8	47925203	47988160	60	19	10164	0.82358	0.20509
-	KCNH5	14	62696573	63080256	1146	68	10164	0.82357	0.20509
+	TES	7	116175493	116268786	298	17	10164	0.82325	0.20518
-	FUOM	10	133342775	133393025	61	5	10164	0.82326	0.20518
+	ALOX5	10	45339176	45456121	455	39	10164	0.82321	0.20519
-	LOC1053768	17	76690190	76742304	144	18	10164	0.82322	0.20519
+	ZC2HC1B	6	143829436	143948346	380	38	10164	0.82305	0.20524
+	BRD2	6	32933660	32991505	427	40	10164	0.823	0.20525
-	DRAM2	1	111107332	111175216	134	15	10164	0.82265	0.20535
+	MYB	6	135145981	135229173	165	17	10164	0.82234	0.20544
+	KIAA1456	8	12910674	13039777	676	38	10164	0.82222	0.20548
-	OTX2	14	56790707	56845476	89	16	10164	0.82204	0.20553
+	ARL11	13	49593299	49643872	216	24	10164	0.82139	0.20571
+	RASA2	3	141452047	141625363	473	24	10164	0.8211	0.2058
-	MOS	8	56102942	56148982	156	18	10164	0.82098	0.20583
+	ADIPOR2	12	1655981	1798679	420	27	10164	0.82089	0.20585
+	PROSC	8	37727583	37789768	41	16	10164	0.82083	0.20587
-	TRERF1	6	42214933	42487205	301	35	10164	0.82074	0.2059
-	ZNF14	19	19700472	19768112	212	30	10164	0.82069	0.20591
+	CDC34	19	496719	552088	84	10	10164	0.82056	0.20595
-	SLC26A5	7	103342730	103481177	258	25	10164	0.82038	0.206
+	METTL23	17	76690873	76743881	146	19	10164	0.82031	0.20602
-	RRAS2	11	14267920	14399506	177	29	10164	0.8201	0.20608
-	MTCH1	6	36958135	37021551	332	44	10164	0.82007	0.20609
-	TMCO1	1	165714291	165803922	144	16	10164	0.82005	0.20609
-	MKI67	10	128086659	128161363	319	30	10164	0.81994	0.20613
-	AKAP8L	19	15370048	15454121	27	11	10164	0.81993	0.20613
+	NEIL2	8	11734663	11797345	417	42	10164	0.81956	0.20623
+	RSC1A1	1	15624869	15671722	121	10	10164	0.81949	0.20625
+	SPA17	11	124638844	124707545	97	8	10164	0.81935	0.20629
+	MTRNR2L6	7	142631299	142677693	132	6	10164	0.8193	0.20631
-	TLR10	4	38762238	38817990	328	25	10164	0.81879	0.20645
-	CD24	6	106959831	107011855	101	17	10164	0.8188	0.20645
+	NCF4	22	36825988	36888017	124	18	10164	0.81876	0.20646
+	IPO13	1	43911806	43978022	134	14	10164	0.81838	0.20657
-	SETD3	14	99387746	99516418	131	7	10164	0.8183	0.20659
-	LOC1053785	1	2198942	2250211	144	30	10164	0.81814	0.20664
+	NDUFA9	12	4614098	4697554	282	30	10164	0.81764	0.20678
-	MEOX2	7	15601212	15721683	362	29	10164	0.81715	0.20692
+	PRPF38B	1	108657292	108711803	58	11	10164	0.81706	0.20695
+	UBE2B	5	134336176	134402108	93	5	10164	0.81697	0.20697
-	PRRT1	6	32138363	32186943	211	59	10164	0.81699	0.20697
-	IL18	11	112133251	112199117	148	17	10164	0.81694	0.20698
+	SLC26A11	17	80185401	80263509	305	24	10164	0.81639	0.20714
-	CTRB2	16	75194096	75242174	172	18	10164	0.81632	0.20716
-	SMARCC1	3	47575888	47816915	336	18	10164	0.81612	0.20721
+	SLC44A1	9	105209608	105448504	90	23	10164	0.81604	0.20724
-	GAS1	9	86934362	86982189	131	7	10164	0.81592	0.20727
+	LOC285500	4	177409513	177687123	957	59	10164	0.81591	0.20728
+	ARL6	3	97729521	97811242	156	20	10164	0.81583	0.2073
+	MEGF8	19	42290609	42388769	115	8	10164	0.81576	0.20732
-	ZNF23	16	71437597	71497252	116	19	10164	0.81561	0.20736
-	SIAE	11	124625789	124711303	132	11	10164	0.81548	0.2074
+	C11orf96	11	43907556	43953883	169	37	10164	0.81535	0.20744
-	HLA-DMA	6	32938614	32988122	356	39	10164	0.81517	0.20749
+	PCDHA4	5	140772074	141022344	456	20	10164	0.81497	0.20755

STRAND	SYMBOL	CHR	START	STOP	NSNPS	NPARAM	N	ZSTAT	P
-	PPP1R12C	19	55080913	55152600	149	27	10164	0.81436	0.20772
+	LOC1019272	2	251352	304727	88	7	10164	0.81432	0.20773
+	OR5K2	3	98462681	98508631	123	23	10164	0.81394	0.20784
+	LOC1053696	12	9368060	9418752	167	17	10164	0.81381	0.20788
-	HENMT1	1	108638288	108696526	72	11	10164	0.81378	0.20789
+	JADE2	5	134489162	134593230	282	41	10164	0.81346	0.20798
+	SDHAF1	19	35960188	36006318	62	15	10164	0.81328	0.20803
-	SMARCD3	7	151228973	151312149	183	21	10164	0.81217	0.20835
-	CDH18	5	19462930	20610873	2815	95	10164	0.81193	0.20842
-	ATP5H	17	75028860	75081979	113	12	10164	0.81192	0.20842
+	ADAMTSL1	9	17871603	18920950	3399	240	10164	0.81174	0.20847
-	KLF14	7	130722554	130769033	115	15	10164	0.81139	0.20857
-	ALK	2	29182774	29956611	2574	167	10164	0.81099	0.20868
+	CELA3B	1	21941925	22008642	410	25	10164	0.811	0.20868
+	GZMM	19	509034	559920	83	10	10164	0.81088	0.20872
+	DCC	18	52305172	53545903	5298	140	10164	0.81079	0.20874
+	FAM159A	1	52596439	52679120	207	31	10164	0.81063	0.20879
+	NR2E1	6	108123115	108198809	65	12	10164	0.81059	0.2088
+	LCE3C	1	152565662	152611086	29	7	10164	0.81051	0.20882
+	GLS	2	190845821	190975552	285	31	10164	0.81044	0.20884
-	PKMYT1	16	2962791	3015539	46	6	10164	0.80991	0.209
-	KLF4	9	107474852	107524766	118	14	10164	0.80965	0.20907
-	EIF3CL	16	28369579	28438841	2	1	10164	0.80959	0.20909
+	DDX43	6	73359562	73427566	193	23	10164	0.80948	0.20912
-	TRPV1	17	3555446	3644411	362	34	10164	0.80896	0.20927
-	NUPR1	16	28527341	28574174	79	7	10164	0.80878	0.20932
-	HELZ	17	67060438	67280963	252	15	10164	0.80869	0.20935
-	HRAS	11	522242	570561	230	22	10164	0.8086	0.20937
-	FIGNL1	7	50434129	50485390	180	17	10164	0.80846	0.20941
-	THPO	3	184361935	184414688	142	30	10164	0.80831	0.20946
-	PLIN1	15	89654365	89714417	114	7	10164	0.80684	0.20988
+	ATP6V1B2	8	20162193	20231696	259	39	10164	0.80654	0.20996
-	GCLM	1	93875205	93944598	20	2	10164	0.80656	0.20996
+	LSMEM1	7	112445238	112501062	181	16	10164	0.80641	0.21
+	PRIM2	6	57279805	57656850	880	32	10164	0.80592	0.21015
-	FEV	2	218971087	219020657	77	17	10164	0.80562	0.21023
-	COL28A1	7	7328549	7572277	887	32	10164	0.80564	0.21023
-	FKBPL	6	32118707	32165308	166	56	10164	0.80559	0.21024
-	KRTAP24-1	21	30271309	30317958	201	24	10164	0.80506	0.21039
-	TCFL5	20	62831014	62896763	211	28	10164	0.80492	0.21043
+	QSOX1	1	180119833	180208034	350	27	10164	0.80488	0.21044
-	PRDX1	1	45501035	45557890	237	22	10164	0.80476	0.21048
+	PTPRZ1	7	121838105	122072036	557	40	10164	0.80474	0.21049
-	TRIM36	5	115114762	115215546	259	36	10164	0.8047	0.2105
+	VIP	6	152712923	152769765	191	26	10164	0.80454	0.21054
+	OLFML3	1	113944391	113992254	117	20	10164	0.80452	0.21055
+	ATXN2L	16	28788048	28847237	67	6	10164	0.80411	0.21067
+	RXRA	9	134291463	134450586	273	20	10164	0.80387	0.21074
-	FBP2	9	94548714	94628832	376	21	10164	0.80384	0.21075
-	SNF8	17	48920096	48980414	223	16	10164	0.80327	0.21091
+	OTOL1	3	161394601	161513975	448	29	10164	0.80304	0.21098
-	PSMB8	6	32830717	32879935	474	48	10164	0.803	0.21099
-	IDH1	2	208226227	208290143	95	16	10164	0.80281	0.21104
+	WDR93	15	89655085	89753790	196	8	10164	0.8025	0.21113
-	PDE4C	19	18197961	18283200	309	29	10164	0.80213	0.21124
+	FANCE	6	35417339	35477106	315	47	10164	0.80198	0.21128
+	ZC3H6	2	112240559	112350063	148	8	10164	0.80193	0.2113
+	RNF5	6	32143385	32190793	204	59	10164	0.80174	0.21135
+	NABP1	2	191643072	191698522	116	17	10164	0.80166	0.21137
-	ZNHIT6	1	85639423	85743463	376	27	10164	0.80136	0.21146
-	FBXL14	12	1555993	1629180	197	40	10164	0.80134	0.21147
+	RBFA	18	79999346	80060652	1	1	10164	0.8013	0.21148
+	WDR37	10	1021836	1142297	423	37	10164	0.80099	0.21157

STRAND	SYMBOL	CHR	START	STOP	NSNPS	NPARAM	N	ZSTAT	P
+	COPS7B	2	231746671	231819254	102	7	10164	0.80093	0.21159
+	C11orf52	11	111883877	111936871	91	14	10164	0.80075	0.21164
+	LGALS12	11	63471052	63526774	69	11	10164	0.80051	0.21171
-	RNASEH1	2	3521813	3593371	207	18	10164	0.80031	0.21176
-	CPEB3	10	92038638	92326118	401	21	10164	0.80013	0.21182
-	CRNKL1	20	20024361	20091046	202	18	10164	0.79989	0.21189
-	MXRA7	17	76663650	76746005	219	25	10164	0.79985	0.2119
-	SPATA21	1	16377117	16472842	155	17	10164	0.79949	0.212
-	PPP2R2C	4	6310578	6598600	709	68	10164	0.79948	0.21201
+	PCDHAC2	5	140931162	141022344	194	15	10164	0.79942	0.21202
-	WFDC11	20	45638563	45705239	244	18	10164	0.79916	0.2121
-	LCT	2	135777845	135872180	269	26	10164	0.79896	0.21216
+	ANXA1	9	73116731	73180393	210	27	10164	0.79883	0.21219
+	RABEPK	9	125165542	125244159	260	24	10164	0.79885	0.21219
+	LDLRAD2	1	21777265	21835221	89	14	10164	0.79825	0.21236
-	AGPAT1	6	32158206	32213111	266	68	10164	0.79818	0.21238
-	FKBP7	2	178453664	178515393	94	13	10164	0.79811	0.2124
+	CLLU1	12	92386531	92441002	121	15	10164	0.79812	0.2124
+	TMX2	11	57677523	57750973	155	23	10164	0.79791	0.21246
-	CSRP2	12	76848715	76914046	119	17	10164	0.79786	0.21248
+	LOC1053739	2	105107926	105155520	68	12	10164	0.79729	0.21264
-	LCE3D	1	152569384	152615504	26	6	10164	0.7972	0.21267
+	NAT8L	4	2024512	2079089	70	12	10164	0.79707	0.2127
+	APOE	19	44870749	44919395	98	14	10164	0.79654	0.21286
-	ENPP6	4	184078706	184252961	546	67	10164	0.7965	0.21287
-	APOBEC4	1	183636275	183688313	285	7	10164	0.79635	0.21291
-	C1orf131	1	231213763	231276187	142	17	10164	0.79585	0.21306
+	DOT1L	19	2129127	2242578	328	21	10164	0.79566	0.21311
-	ASAH2	10	50172778	50286577	382	20	10164	0.79556	0.21314
-	GPRIN3	4	89231276	89343010	249	40	10164	0.79552	0.21316
-	ULK3	15	74826116	74878211	141	34	10164	0.79507	0.21329
+	PAOX	10	133344237	133401696	69	8	10164	0.79504	0.21329
-	ASGR2	17	7091322	7150522	265	40	10164	0.7948	0.21336
+	NPY5R	4	163308868	163362403	201	36	10164	0.79471	0.21339
+	ATP2A1	16	28843488	28914509	96	6	10164	0.7946	0.21342
-	JMJD6	17	76702832	76761799	131	19	10164	0.79452	0.21345
+	SLC39A2	14	20964255	21011875	162	20	10164	0.79392	0.21362
+	DMRTC2	19	41809743	41862330	96	5	10164	0.79386	0.21364
+	FAM124A	13	51187334	51294241	294	41	10164	0.79377	0.21366
+	ANKFN1	17	55930604	56521407	1135	50	10164	0.79347	0.21375
-	RAB13	1	153971617	154021377	61	11	10164	0.79343	0.21376
-	DNPH1	6	43215629	43264473	117	17	10164	0.79303	0.21388
+	TMEM141	9	136756325	136803317	186	9	10164	0.79288	0.21392
-	ZNF608	5	124626913	124783824	374	44	10164	0.79232	0.21409
-	GPATCH8	17	44385277	44538602	220	13	10164	0.79216	0.21413
+	CACYBP	1	174964435	175022027	79	16	10164	0.79218	0.21413
-	HSPA9	5	138544882	138610629	70	5	10164	0.79199	0.21418
-	IGF1	12	102385867	102516786	241	24	10164	0.79201	0.21418
-	RAB24	5	177291190	177338744	74	13	10164	0.79201	0.21418
-	DNAJB8	3	128452432	128502248	237	58	10164	0.79176	0.21425
+	AATF	17	36913875	37066871	212	19	10164	0.79159	0.2143
-	C12orf71	12	27071057	27119144	157	16	10164	0.79153	0.21432
+	TTC4	1	54680822	54752657	263	9	10164	0.79134	0.21437
+	SPDYA	2	28775834	28860610	142	14	10164	0.7913	0.21438
-	PRDM4	12	107722866	107796272	200	21	10164	0.79116	0.21443
+	TRMT10C	3	101526836	101576446	146	16	10164	0.79103	0.21446
-	BRD1	22	49763278	49862548	311	24	10164	0.79066	0.21457
+	C18orf25	18	46138553	46276992	314	14	10164	0.79048	0.21463
-	TRPT1	11	64213799	64261254	149	33	10164	0.79035	0.21466
+	BLZF1	1	169332956	169406544	193	16	10164	0.79033	0.21467
+	SAMD15	14	77342419	77401244	154	18	10164	0.79027	0.21469
+	KCTD6	3	58457096	58512360	87	12	10164	0.79016	0.21472
-	LOC1019280	8	1962618	2008870	143	11	10164	0.79004	0.21475

STRAND	SYMBOL	CHR	START	STOP	NSNPS	NPARAM	N	ZSTAT	P
+	CCDC36	3	49163428	49268157	199	20	10164	0.78928	0.21497
-	C1QTNF4	11	47579664	47629409	51	12	10164	0.78926	0.21498
+	PRICKLE4	6	41745762	41797372	121	21	10164	0.789	0.21506
+	GNPAT	1	231206173	231287973	204	25	10164	0.78838	0.21524
+	CCDC101	16	28518928	28601790	148	9	10164	0.78826	0.21527
+	SPPL2B	19	2293614	2365102	214	24	10164	0.78799	0.21535
+	MAB21L3	1	116040792	116145240	118	12	10164	0.78785	0.21539
-	TSPYL4	6	116239964	116289098	162	14	10164	0.78783	0.2154
-	PITRM1	10	3127727	3207841	329	20	10164	0.78755	0.21548
+	UPK1A	19	35631516	35688485	203	25	10164	0.7874	0.21552
+	ZNF69	19	11852786	11924551	32	5	10164	0.7872	0.21558
+	MLH1	3	36958350	37060846	161	10	10164	0.78711	0.21561
+	FKBP2	11	64205941	64254135	157	34	10164	0.7868	0.2157
+	SF3A2	19	2201817	2258679	129	23	10164	0.78634	0.21583
+	USP38	4	143149917	143233874	144	8	10164	0.78595	0.21595
-	LZTFL1	3	45813316	45950724	307	25	10164	0.78589	0.21597
+	PTPN13	4	86559307	86825179	854	25	10164	0.7853	0.21614
-	FHIT	3	59737587	61286459	5566	343	10164	0.78521	0.21616
+	COMMD1	2	61853728	62146070	582	30	10164	0.78514	0.21619
+	TFG	3	100674284	100758967	228	21	10164	0.7851	0.2162
-	GYS2	12	21521527	21665773	325	18	10164	0.78454	0.21636
+	HECTD2	10	91374280	91524829	160	3	10164	0.78448	0.21638
-	SELL	1	169680665	169746702	341	24	10164	0.78445	0.21639
-	RAET1E	6	149854397	149933321	241	18	10164	0.78442	0.2164
+	C9orf131	9	35006095	35055991	136	24	10164	0.78424	0.21645
+	PI3	20	45139899	45186544	122	8	10164	0.78403	0.21651
+	PPT2	6	32118452	32173681	209	62	10164	0.78371	0.2166
-	CD209	19	7729988	7782605	122	15	10164	0.78348	0.21667
-	ADGRF1	6	46987703	47077363	226	31	10164	0.7833	0.21673
+	EPHB2	1	22675770	22925330	663	81	10164	0.78319	0.21676
-	C1orf116	1	207008521	207067761	129	19	10164	0.78297	0.21682
+	POLR3D	8	22210104	22264355	55	12	10164	0.78237	0.217
+	ATP1B3	3	141841628	141936540	306	20	10164	0.7821	0.21708
-	FAM69A	1	92822729	92996522	346	20	10164	0.7821	0.21708
-	LOC1053745	2	42403741	42460087	183	37	10164	0.78202	0.2171
+	FAM73B	9	129001605	129082082	65	16	10164	0.78187	0.21715
-	PIGU	20	34550542	34712285	312	18	10164	0.7818	0.21717
+	RBM38	20	57356398	57419333	176	22	10164	0.78154	0.21724
-	LIMS2	2	127628421	127716786	258	33	10164	0.78126	0.21732
+	PCDHA5	5	140786776	141022344	424	19	10164	0.78099	0.21741
+	KCNU1	8	36749324	36946125	346	23	10164	0.78057	0.21753
-	ARPC5	1	183616193	183670941	292	6	10164	0.7803	0.21761
+	PTCHD2	1	11444238	11547583	241	21	10164	0.78016	0.21765
+	TRMT61A	14	103494172	103547073	177	19	10164	0.78014	0.21766
+	MOCOS	18	36152517	36278722	266	39	10164	0.78008	0.21767
-	LOC1053709	15	91260022	91307820	115	11	10164	0.7801	0.21767
+	PRR3	6	30521709	30574696	204	36	10164	0.77973	0.21777
-	YIPF5	5	144148159	144205714	172	12	10164	0.7796	0.21781
-	ZNF18	17	11967439	12032510	147	19	10164	0.77907	0.21797
-	INSR	19	7102255	7329000	880	85	10164	0.77902	0.21798
-	NT5C	17	75120225	75166795	83	12	10164	0.77898	0.21799
-	PHYHIP	8	22209703	22267338	56	12	10164	0.77884	0.21804
-	TP53TG5	20	45363880	45442838	178	19	10164	0.77884	0.21804
-	LOC441239	7	64877751	64925100	217	17	10164	0.77856	0.21812
-	TCTE3	6	169715090	169786549	180	13	10164	0.77833	0.21819
-	COX18	4	73044696	73104759	77	23	10164	0.77784	0.21833
-	KCTD10	12	109438655	109512350	253	19	10164	0.77723	0.21851
-	LOC1053776	4	143167737	143220297	92	6	10164	0.7771	0.21855
+	MVK	12	109538353	109607270	250	21	10164	0.77704	0.21857
+	MCEMP1	19	7642057	7689833	103	20	10164	0.77612	0.21884
+	IGFL1	19	46194752	46241243	142	20	10164	0.77612	0.21884
-	DNAH17	17	78413697	78612432	647	70	10164	0.77573	0.21896
-	TEX261	2	70975938	71029873	154	24	10164	0.77568	0.21897

STRAND	SYMBOL	CHR	START	STOP	NSNPS	NPARAM	N	ZSTAT	P
-	FAM184A	6	118949829	119184193	599	42	10164	0.77554	0.21901
+	FSCN2	17	81478051	81549790	290	26	10164	0.77548	0.21903
+	SCYL1	11	65490065	65548711	106	36	10164	0.77516	0.21912
+	RNF185	22	31125152	31217019	267	35	10164	0.77517	0.21912
-	PRKAA1	5	40749379	40833195	314	28	10164	0.7751	0.21914
+	TOMM6	6	41752443	41799896	100	17	10164	0.77492	0.21919
-	PRR23B	3	139009031	139055926	100	12	10164	0.77469	0.21926
-	SP8	7	20771467	20821889	112	23	10164	0.77458	0.21929
+	CEACAM21	19	41514250	41596844	156	16	10164	0.77447	0.21933
+	PKP4	2	158421880	158691429	895	34	10164	0.77427	0.21939
+	C11orf86	11	66940283	66987008	74	26	10164	0.77412	0.21943
-	C3orf62	3	49258597	49312075	95	15	10164	0.77355	0.2196
+	PDPN	1	13548757	13627957	323	55	10164	0.7732	0.2197
+	SLK	10	103932182	104039233	327	19	10164	0.77311	0.21973
+	ZNF211	19	57598167	57652779	185	11	10164	0.77301	0.21976
-	MFSD9	2	102704630	102771882	140	12	10164	0.77298	0.21977
+	CACNA1C	12	1935786	2707949	1666	157	10164	0.77279	0.21982
-	TCP10	6	167362379	167419552	286	35	10164	0.77231	0.21996
-	PSMD12	17	67330486	67401605	45	8	10164	0.77179	0.22012
-	UGT2B15	4	68636597	68705776	242	26	10164	0.77174	0.22013
+	RBM17	10	6053986	6127459	402	62	10164	0.77171	0.22014
+	SLC4A9	5	140323559	140385137	81	14	10164	0.77122	0.22029
+	ACVRL1	12	51871913	51933361	156	17	10164	0.77109	0.22033
+	KRTAP10-8	21	44577079	44622954	211	7	10164	0.77064	0.22046
+	ERMARD	6	169716622	169791584	201	14	10164	0.7706	0.22047
+	PRKRIP1	7	102361357	102436682	78	11	10164	0.77046	0.22051
+	VIT	2	36661690	36824794	524	59	10164	0.77037	0.22054
-	GBX2	2	236155236	236203009	1	1	10164	0.77028	0.22057
-	LGALS2	22	37560246	37615017	197	24	10164	0.77019	0.22059
+	NUDT22	11	64191258	64240269	165	36	10164	0.77021	0.22059
+	ELMOD1	11	107556091	107676779	280	32	10164	0.7701	0.22062
-	HUS1	7	47953288	48014625	184	18	10164	0.76989	0.22068
-	ZNF750	17	82819434	82875055	171	16	10164	0.76983	0.2207
+	LOC1053781	14	104181110	104228144	31	5	10164	0.76962	0.22076
+	KCNMB2	3	178501298	178854429	1120	58	10164	0.76948	0.22081
+	GTF3C6	6	110923560	110977888	249	14	10164	0.76938	0.22083
-	RBSN	3	15060069	15134157	171	12	10164	0.76889	0.22098
+	ZFPM2	8	105211436	105814539	1523	102	10164	0.76867	0.22104
+	SH2D7	15	78042805	78114051	216	29	10164	0.76865	0.22105
-	KLK12	19	51018960	51070652	209	47	10164	0.76854	0.22108
-	CCDC148	2	158161356	158491824	1276	45	10164	0.76847	0.2211
+	CNTNAP2	7	146081361	148430998	6424	259	10164	0.76841	0.22112
-	RGS5	1	163132299	163356791	792	41	10164	0.76829	0.22116
-	GMEB2	20	63577602	63662028	312	31	10164	0.76827	0.22116
+	C4BPA	1	207069238	207154972	142	19	10164	0.76796	0.22125
+	EXOSC7	3	44941230	45022668	174	18	10164	0.76798	0.22125
+	RPL5	1	92797037	92851924	131	14	10164	0.76762	0.22136
+	OR10H3	19	15706393	15752343	216	15	10164	0.76751	0.22139
-	TMEM200B	1	29109425	29164134	109	14	10164	0.76749	0.2214
-	IL2RA	10	6000694	6097370	510	72	10164	0.76714	0.2215
-	MMAB	12	109543715	109608553	225	19	10164	0.76664	0.22165
-	KRTAP10-5	21	44569455	44615604	220	9	10164	0.7666	0.22166
+	GTF2E1	3	120707711	120793070	141	14	10164	0.76625	0.22176
-	CDCA4	14	104999573	105056088	199	14	10164	0.76608	0.22181
+	C16orf59	16	2425111	2474963	9	3	10164	0.76594	0.22186
-	CEP152	15	48652534	48846918	305	35	10164	0.7657	0.22193
+	PCDHA6	5	140792922	141022344	411	19	10164	0.76566	0.22194
-	SULT1A1	16	28595587	28658586	99	8	10164	0.76544	0.222
+	IRAK3	12	66154198	66264622	277	33	10164	0.76547	0.222
+	CDC123	10	12160962	12260590	296	27	10164	0.76544	0.22201
+	PRKCH	14	61286443	61560980	832	83	10164	0.76495	0.22215
+	HSPB2	11	111877736	111924093	94	13	10164	0.76455	0.22227
-	RNF6	13	26122115	26257559	512	43	10164	0.76455	0.22227

STRAND	SYMBOL	CHR	START	STOP	NSNPS	NPARAM	N	ZSTAT	P
-	TRIM52	5	181246327	181296160	142	11	10164	0.76452	0.22228
+	ZSCAN2	15	84565986	84633718	168	25	10164	0.76441	0.22231
+	BOC	3	113176003	113297463	435	58	10164	0.76436	0.22233
+	PCDHB12	5	141173719	141222126	135	15	10164	0.76406	0.22242
+	RASSF7	11	524776	574025	244	24	10164	0.76389	0.22247
+	FTO	16	53668963	54124467	1302	115	10164	0.7635	0.22258
-	SLC35D2	9	96303437	96418710	422	25	10164	0.7629	0.22276
+	RGS12	4	3257978	3449913	664	47	10164	0.76282	0.22278
-	SHISA2	13	26034597	26094587	283	26	10164	0.76283	0.22278
+	SAMD7	3	169870106	169949175	337	31	10164	0.76239	0.22291
+	PIGB	15	55283865	55365648	256	22	10164	0.76227	0.22295
+	POLR2M	15	57671521	57727557	115	21	10164	0.76202	0.22302
+	UBTFL1	11	90050950	90097131	25	8	10164	0.76193	0.22305
+	TOX2	20	43879852	44079616	536	63	10164	0.76179	0.22309
+	FCHO1	19	17712718	17798568	214	17	10164	0.76173	0.22311
-	MED24	17	40009097	40089636	172	17	10164	0.76154	0.22317
-	ACSM2B	16	20526261	20611373	197	16	10164	0.76154	0.22317
-	LRRC31	3	169829241	169904935	287	39	10164	0.76136	0.22322
-	TM7SF3	12	26961570	27049427	243	39	10164	0.76094	0.22335
-	CCDC181	1	169384870	169495669	284	26	10164	0.76087	0.22337
+	OBP2A	9	135511139	135559969	103	12	10164	0.76015	0.22358
+	FAM159B	5	64655308	64728190	98	13	10164	0.76016	0.22358
-	BCL2L1	20	31654452	31758964	134	9	10164	0.76012	0.22359
+	FOXO3	6	108524823	108694769	255	24	10164	0.76003	0.22362
-	ABHD16A	6	31676949	31738360	266	61	10164	0.75998	0.22363
+	TMEM216	11	61356687	61408863	45	13	10164	0.75985	0.22367
-	FCRL5	1	157503377	157587520	323	19	10164	0.75954	0.22377
+	SUZ12	17	31902025	32011045	58	2	10164	0.75925	0.22385
-	LCN8	9	136744388	136793279	207	10	10164	0.75907	0.22391
-	TMEM41B	11	9270654	9349768	206	18	10164	0.75873	0.22401
-	NPHP3	3	132670609	132757459	181	36	10164	0.75863	0.22404
-	RNF216	7	5610041	5816730	645	25	10164	0.75856	0.22406
+	PANO1	11	762511	809185	140	21	10164	0.75843	0.2241
-	SIX5	19	45754785	45804239	87	13	10164	0.75824	0.22416
+	HEATR3	16	50030970	50115481	229	12	10164	0.75809	0.2242
+	CLN5	13	76956924	77012517	155	23	10164	0.75777	0.22429
-	HNRNPK	9	83958083	84015654	114	16	10164	0.75776	0.2243
+	STIM1	11	3820703	4103210	648	31	10164	0.75773	0.22431
+	MPP6	7	24538346	24703703	401	40	10164	0.75765	0.22433
+	SPP1	4	87940650	87993411	155	25	10164	0.75758	0.22435
+	OR14C36	1	248313775	248359713	115	3	10164	0.75734	0.22442
+	SOX1	13	112032599	112081706	57	12	10164	0.75728	0.22444
-	OR2M7	1	248313630	248359568	116	3	10164	0.75706	0.22451
+	NDUFS5	1	38991295	39044636	170	9	10164	0.75691	0.22455
+	CAP2	6	17358216	17567792	469	46	10164	0.75685	0.22457
-	MTRNR2L7	10	37591438	37637931	151	11	10164	0.75681	0.22458
-	HNRNPCL2	1	13105488	13151854	70	3	10164	0.75675	0.2246
+	DGKA	12	55896162	55964027	78	12	10164	0.75644	0.22469
-	RNF133	7	122687712	122734154	111	14	10164	0.75641	0.2247
+	ANKRD6	6	89398178	89643834	533	38	10164	0.75637	0.22472
+	PTDSS1	8	96226886	96344552	103	12	10164	0.75568	0.22492
+	CPA5	7	130309790	130384709	235	24	10164	0.75557	0.22495
+	ZDHHC16	10	97411131	97467370	96	17	10164	0.75552	0.22497
+	PCDHA7	5	140799384	141022344	407	18	10164	0.75492	0.22515
+	P2RX3	11	57300946	57382399	131	20	10164	0.75477	0.22519
+	SERPINB10	18	63872969	63946111	304	10	10164	0.75469	0.22522
+	SLC39A13	11	47372132	47426500	132	13	10164	0.75463	0.22523
+	KLK2	19	50838433	50890567	278	41	10164	0.75453	0.22527
-	TDRP	8	447018	580781	591	39	10164	0.75444	0.22529
-	LOX	5	122053195	122113360	136	18	10164	0.75442	0.2253
-	RRN3	16	15050022	15129335	37	8	10164	0.75436	0.22532
-	ZMYM6	1	34976165	35066968	155	5	10164	0.75421	0.22536
+	U2SURP	3	142966524	143070725	23	9	10164	0.75422	0.22536

STRAND	SYMBOL	CHR	START	STOP	NSNPS	NPARAM	N	ZSTAT	P
-	RAF1	3	12573601	12699201	378	20	10164	0.75398	0.22543
+	BCR	22	23145365	23328037	426	35	10164	0.75379	0.22549
+	MARS	12	57452953	57526655	160	22	10164	0.75377	0.22549
+	CCDC50	3	191294082	191408670	465	27	10164	0.75375	0.2255
+	OR13F1	9	104469263	104515222	205	17	10164	0.75355	0.22556
+	GTF3A	13	27389544	27445709	216	19	10164	0.75337	0.22562
-	AKAP8	19	15343521	15414828	25	10	10164	0.75331	0.22563
+	EXT2	11	44060549	44255430	593	37	10164	0.75291	0.22575
-	VPS52	6	33240272	33306965	265	38	10164	0.75276	0.2258
-	MRPS24	7	43856558	43904546	130	18	10164	0.75276	0.2258
-	CST2	20	23813767	23861675	205	16	10164	0.75271	0.22581
-	ATP2B2	3	10314023	10742993	1635	150	10164	0.75257	0.22586
-	CCL19	9	34679570	34726277	134	28	10164	0.75176	0.2261
+	PDCD11	10	103361624	103456295	197	37	10164	0.75011	0.2266
-	WIBG	12	55891413	55962913	85	13	10164	0.74987	0.22667
+	GREB1L	18	21207242	21535423	282	15	10164	0.74983	0.22668
+	CEP126	11	101880015	102011065	329	25	10164	0.74933	0.22683
+	DHRS4	14	23918735	23979279	228	13	10164	0.7493	0.22684
+	GID8	20	62903089	62958475	130	16	10164	0.74923	0.22686
+	LOC1053692	15	100820307	100887011	200	27	10164	0.74914	0.22689
+	CCDC183	9	136761338	136817741	184	11	10164	0.74891	0.22696
+	OR2T8	1	247877253	247933152	182	18	10164	0.74884	0.22698
-	RPRD1A	18	35979824	36102594	263	25	10164	0.7485	0.22708
-	OR2T33	1	248262852	248308814	112	2	10164	0.74837	0.22712
-	SLC7A8	14	23115295	23218660	242	58	10164	0.74831	0.22714
-	ACTG1	17	81499971	81547866	197	21	10164	0.7479	0.22726
+	ALG1L2	3	130046831	130108467	144	10	10164	0.74775	0.2273
+	PRRC2A	6	31585673	31647777	401	80	10164	0.74758	0.22736
-	HN1	17	75125243	75189683	105	14	10164	0.74756	0.22736
-	CA5A	16	87878019	87971569	205	15	10164	0.74754	0.22737
+	NPIPB8	16	28589517	28668725	111	8	10164	0.74742	0.2274
-	LDHB	12	21625341	21692971	92	5	10164	0.7472	0.22747
-	CASP6	4	109678628	109748904	209	34	10164	0.74703	0.22752
-	LCN10	9	136728167	136777959	151	8	10164	0.747	0.22753
-	SAMD12	8	118121828	118656995	1470	101	10164	0.74693	0.22755
+	CDK5R2	2	218924628	218972155	110	11	10164	0.74668	0.22763
+	EED	11	86209544	86295420	223	29	10164	0.74659	0.22766
-	ZER1	9	128719786	128807422	135	14	10164	0.74631	0.22774
+	ZNF546	19	39960630	40031041	160	8	10164	0.74622	0.22777
-	MYBPH	1	203157811	203210966	214	44	10164	0.74606	0.22781
-	TSC22D1	13	44422143	44611565	323	36	10164	0.74597	0.22784
-	HNRNPH1	5	179604178	179669784	200	20	10164	0.74561	0.22795
-	HBEGF	5	140322843	140381603	80	14	10164	0.74557	0.22796
+	ARFGAP1	20	63237785	63299793	209	21	10164	0.7454	0.22802
-	JAG1	20	10627684	10709046	214	48	10164	0.74511	0.2281
-	CARS	11	2990922	3092451	259	18	10164	0.74508	0.22811
+	MFSD11	17	76701565	76814356	277	33	10164	0.7449	0.22817
-	ANKRD36B	2	97482663	97625245	173	12	10164	0.7448	0.2282
+	TMEM159	16	21123377	21190616	165	7	10164	0.74452	0.22828
-	SKAP2	7	26657062	26899743	766	21	10164	0.74443	0.22831
+	SOD3	4	24760463	24810845	88	14	10164	0.74425	0.22836
+	MYLIP	6	16094086	16158248	158	35	10164	0.74418	0.22838
-	KTI12	1	52022105	52068800	91	8	10164	0.74273	0.22882
-	UFSP1	7	100878723	100924718	131	19	10164	0.74268	0.22884
+	IL17RB	3	53811550	53875800	198	33	10164	0.74251	0.22889
-	STRA6	15	74169466	74247259	189	31	10164	0.74241	0.22892
+	PTPRU	1	29201516	29336813	261	28	10164	0.74232	0.22895
+	TRAPPC4	11	118983531	119033674	175	17	10164	0.74229	0.22896
-	IL1B	2	112819751	112871779	103	18	10164	0.74223	0.22897
-	FAM187B	19	35214801	35263725	212	26	10164	0.74217	0.22899
+	TRIM44	11	35627692	35821053	344	24	10164	0.74205	0.22903
-	FECH	18	57534841	57621737	288	44	10164	0.74157	0.22917
-	AAED1	9	96631251	96690323	78	14	10164	0.74129	0.22926

STRAND	SYMBOL	CHR	START	STOP	NSNPS	NPARAM	N	ZSTAT	P
+	CHST2	3	143084776	143134014	42	8	10164	0.74073	0.22943
-	ANKRD39	2	96837985	96893019	122	29	10164	0.74007	0.22963
+	PFDN6	6	33254597	33300934	190	32	10164	0.73987	0.22969
-	SLC6A20	3	45745449	45831561	203	58	10164	0.73988	0.22969
+	NAB2	12	57053894	57105476	94	14	10164	0.73957	0.22978
-	TCEA1	8	53956554	54057456	102	16	10164	0.73947	0.22981
-	SEC24D	4	118712823	118873683	622	26	10164	0.73941	0.22983
+	BTF3L4	1	52021185	52100716	169	10	10164	0.73915	0.22991
+	GNB3	12	6805854	6857395	86	14	10164	0.73877	0.23002
-	AP3D1	19	2090988	2186620	284	27	10164	0.7386	0.23007
-	LCN15	9	136749634	136805114	210	11	10164	0.73849	0.23011
+	LOC1053703	13	113782492	113830631	200	17	10164	0.73826	0.23018
+	SMYD5	2	73179238	73237227	101	8	10164	0.7382	0.2302
+	ITPA	20	3173868	3237449	192	24	10164	0.73798	0.23026
-	LOC730183	16	30687704	30733489	14	2	10164	0.73778	0.23032
+	RYR2	1	237007238	237843988	2502	192	10164	0.7377	0.23035
-	PLSCR5	3	146566608	146641216	359	23	10164	0.73767	0.23036
+	ZBED4	22	49818849	49900078	340	17	10164	0.73764	0.23037
+	KRTAP10-7	21	44565597	44612174	229	10	10164	0.73753	0.2304
-	BOLA3	2	74125401	74182912	68	4	10164	0.73729	0.23047
+	TLL1	4	165838238	166115178	777	66	10164	0.737	0.23056
-	RANBP3L	5	36237874	36336926	308	28	10164	0.73696	0.23057
+	APOBEC3D	22	38986113	39043251	136	19	10164	0.73655	0.2307
+	SLC6A6	3	14367576	14499349	286	50	10164	0.7365	0.23071
-	CELA1	12	51318443	51381679	179	24	10164	0.73625	0.23079
-	CNOT4	7	135351795	135545127	429	19	10164	0.736	0.23086
+	MRPL19	2	75611783	75672208	218	30	10164	0.73595	0.23088
-	RFTN1	3	16305845	16548715	788	94	10164	0.73583	0.23092
-	SPATA9	5	95642181	95718010	177	12	10164	0.73552	0.23101
+	PRG4	1	186261273	186324562	160	22	10164	0.73548	0.23102
-	TFPI2	7	93875397	93925991	92	7	10164	0.7353	0.23108
-	PPID	4	158699127	158758400	76	11	10164	0.73523	0.2311
+	USF2	19	35233993	35289815	202	23	10164	0.73475	0.23124
+	ZNF536	19	30190044	30723538	845	106	10164	0.73456	0.2313
+	OR10H2	19	15693024	15739052	256	16	10164	0.73434	0.23137
+	NFKBIB	19	38864657	38918894	133	32	10164	0.73431	0.23138
-	SORBS2	4	185575444	185991716	1037	82	10164	0.7343	0.23138
-	GSTA5	6	52821742	52881095	162	9	10164	0.73407	0.23145
-	ST18	8	52100832	52444902	674	62	10164	0.73395	0.23149
+	PCDHAC1	5	140891717	141022344	258	17	10164	0.73377	0.23154
+	LOC1053714	1	150515283	150561350	99	21	10164	0.7337	0.23157
+	PCDHB5	5	141100206	141148127	106	13	10164	0.7335	0.23163
+	OR2M5	1	248110148	248156086	97	9	10164	0.73344	0.23165
-	COPB2	3	139347587	139424680	205	15	10164	0.73309	0.23175
-	ST7L	1	112507804	112655838	263	20	10164	0.733	0.23178
+	IL37	2	112876064	112928882	96	15	10164	0.73251	0.23193
-	PDDC1	11	757217	812501	159	23	10164	0.73223	0.23201
+	OR2T1	1	248370995	248417104	35	2	10164	0.73209	0.23206
-	PGC	6	41726711	41782401	115	25	10164	0.73203	0.23208
+	PCDHA8	5	140806322	141022344	386	17	10164	0.73193	0.23211
+	ROMO1	20	35664310	35710980	126	14	10164	0.73147	0.23225
-	FAM126A	7	22885848	23049195	470	21	10164	0.73133	0.23229
-	SIGLEC6	19	51509530	51566863	296	21	10164	0.73127	0.23231
+	ATP6V0D2	8	86063910	86164225	337	15	10164	0.73121	0.23233
+	PLEKHG4	16	67242820	67299500	52	12	10164	0.73117	0.23234
+	ZFYVE9	1	52107094	52356686	419	20	10164	0.73112	0.23235
-	TXNDC12	1	52010131	52091171	159	11	10164	0.7311	0.23236
+	DTHD1	4	36246611	36355756	360	43	10164	0.73104	0.23238
-	OOEP	6	73358555	73404792	123	17	10164	0.73078	0.23246
-	VWA7	6	31755401	31812331	235	70	10164	0.73066	0.23249
+	BIK	22	43075748	43139712	273	55	10164	0.73045	0.23256
+	GP9	3	129020449	129072411	60	18	10164	0.73028	0.23261
+	OR2Z1	19	8695954	8742009	81	11	10164	0.73016	0.23265

STRAND	SYMBOL	CHR	START	STOP	NSNPS	NPARAM	N	ZSTAT	P
-	RFT1	3	53061152	53165459	316	21	10164	0.73008	0.23267
-	HPSE	4	83282461	83370153	107	9	10164	0.73006	0.23268
-	TRPV3	17	3500502	3592995	461	57	10164	0.72998	0.2327
-	MANSC4	12	27752641	27808083	179	19	10164	0.72985	0.23274
+	C11orf87	11	109386728	109439167	77	20	10164	0.72969	0.23279
-	OR2T12	1	248284616	248330578	178	1	10164	0.72955	0.23283
-	KHDRBS2	6	61620484	62321195	1938	49	10164	0.72946	0.23286
+	PPIA	7	44761636	44813123	114	11	10164	0.72856	0.23314
+	STRAP	12	15847354	15913478	116	5	10164	0.72828	0.23322
-	CYB5R2	11	7655095	7709341	303	31	10164	0.72815	0.23326
-	COPG2	7	130496238	130703758	358	17	10164	0.72807	0.23329
-	LOC1053691	1	53631740	53774323	369	21	10164	0.72798	0.23331
-	MAPK11	22	50253713	50305393	175	34	10164	0.72785	0.23335
+	CD40	20	46083250	46139745	149	21	10164	0.72772	0.23339
-	SLC46A1	17	28384642	28441212	93	16	10164	0.72756	0.23344
-	SLC13A5	17	6674713	6748421	126	26	10164	0.72743	0.23348
+	FAM122A	9	68745048	68793693	141	26	10164	0.72727	0.23353
+	NDUFV2	18	9067630	9144345	145	25	10164	0.727	0.23361
-	GTSCR1	18	70620519	70705875	253	29	10164	0.72688	0.23365
-	WIF1	12	65040624	65156566	181	22	10164	0.72665	0.23372
-	SETX	9	132251440	132391726	323	13	10164	0.72641	0.23379
+	FLJ20712	7	33691517	33738456	123	22	10164	0.72633	0.23382
+	SOBP	6	107455098	107671309	505	60	10164	0.72605	0.2339
+	CEPT1	1	111104627	111195102	177	20	10164	0.72586	0.23396
+	SWI5	9	128240384	128298989	109	14	10164	0.72551	0.23407
-	TUFM	16	28832411	28881511	81	5	10164	0.72541	0.2341
-	TMPRSS11F	4	68043198	68164869	311	11	10164	0.7253	0.23413
+	LRMP	12	25017101	25118335	330	40	10164	0.72523	0.23416
+	GALR2	17	76037088	76087541	63	3	10164	0.72521	0.23416
+	TNS2	12	53011980	53074379	189	20	10164	0.72515	0.23418
+	ZNF362	1	33221307	33310720	175	24	10164	0.72474	0.23431
+	OLFM1	9	135040243	135131184	423	43	10164	0.72469	0.23432
-	SULT1A2	16	28581943	28632083	103	7	10164	0.72461	0.23434
+	ANXA9	1	150947023	151006063	111	15	10164	0.72455	0.23437
+	FAM102B	1	108525349	108649327	298	14	10164	0.72449	0.23438
-	C2orf42	2	70139885	70226019	259	29	10164	0.72421	0.23447
-	MRPS33	7	140996161	141049981	76	7	10164	0.72404	0.23452
+	LYN	8	55843463	56022447	644	43	10164	0.72396	0.23455
+	HNRNPH3	10	68297011	68353196	138	13	10164	0.72381	0.23459
+	RPL15	3	23881804	23933696	178	21	10164	0.72366	0.23464
+	TNFAIP3	6	137831317	137893314	136	23	10164	0.72298	0.23485
+	RAG1	11	36475400	36589762	280	42	10164	0.72261	0.23496
-	HS3ST1	4	11388364	11464567	272	39	10164	0.7226	0.23496
-	LOC1053764	10	15006256	15108080	451	36	10164	0.72228	0.23506
+	TTC25	17	41895617	41975651	107	8	10164	0.72217	0.23509
+	TECPR2	14	102327963	102512481	539	25	10164	0.72161	0.23527
+	LOC1019275	19	35979499	36055997	120	20	10164	0.72156	0.23528
-	BTNL2	6	32383339	32443879	1253	39	10164	0.72133	0.23535
-	TMED8	14	77325021	77412053	211	24	10164	0.72124	0.23538
-	DDHD1	14	53026740	53188328	360	29	10164	0.7212	0.23539
+	EDRF1	10	125684515	125774143	238	36	10164	0.72094	0.23547
+	LY9	1	160761074	160838256	98	7	10164	0.72063	0.23557
-	ALKBH4	7	102446220	102499874	75	6	10164	0.72055	0.23559
-	MUSTN1	3	52823115	52870219	228	14	10164	0.72056	0.23559
-	NQO1	16	69699401	69761668	134	16	10164	0.72027	0.23568
-	KBTBD3	11	106036806	106112765	147	20	10164	0.71923	0.236
-	HBD	11	5222829	5269628	134	12	10164	0.7192	0.23601
+	QRSL1	6	106594566	106678417	344	26	10164	0.7191	0.23604
+	MYL12A	18	3212482	3266237	156	23	10164	0.71858	0.2362
+	TMEM182	2	102701908	102853838	250	20	10164	0.71849	0.23623
+	P2RY11	19	10076521	10125389	76	17	10164	0.71832	0.23628
-	PIK3IP1	22	31271593	31327534	157	24	10164	0.71822	0.23631
+	CCDC146	7	77087617	77305204	630	35	10164	0.71767	0.23648

STRAND	SYMBOL	CHR	START	STOP	NSNPS	NPARAM	N	ZSTAT	P
+	MAP3K8	10	30399003	30471833	175	16	10164	0.7176	0.2365
-	PFKFB4	3	48507684	48597774	95	14	10164	0.71758	0.23651
+	PRDX6	1	173442347	173498807	99	10	10164	0.71718	0.23663
-	ZNF780B	19	40018260	40094027	161	9	10164	0.71683	0.23674
-	INO80C	18	35458327	35532991	135	20	10164	0.71661	0.23681
-	PEX11A	15	89671531	89725784	124	4	10164	0.71638	0.23688
+	INPP5J	22	31072736	31144697	262	42	10164	0.71638	0.23688
-	FCGR3B	1	161613196	161666963	90	17	10164	0.71613	0.23696
-	ELAVL1	19	7948573	8040645	231	19	10164	0.71588	0.23703
+	SRCAP	16	30664141	30750129	23	2	10164	0.71576	0.23707
-	PCGF6	10	103292796	103386134	202	33	10164	0.71562	0.23711
+	TGFBI	5	135993895	136073818	220	29	10164	0.71517	0.23725
+	KISS1R	19	882342	931015	26	4	10164	0.71515	0.23726
+	C16orf71	16	4699288	4759396	107	3	10164	0.71503	0.23729
+	CCNK	14	99446402	99521515	61	4	10164	0.71503	0.2373
+	CA6	1	8910785	8985092	213	47	10164	0.71496	0.23732
-	LRP2	2	169117109	169397613	429	33	10164	0.71442	0.23748
+	UBE3B	12	109442410	109553628	388	21	10164	0.71428	0.23753
-	OR51S1	11	4838203	4894600	133	14	10164	0.71426	0.23753
-	CDKL3	5	134226120	134406469	291	11	10164	0.71421	0.23755
-	HSF2BP	21	43519186	43694499	152	7	10164	0.71389	0.23765
+	C9orf47	9	88955863	89006142	140	15	10164	0.7138	0.23768
-	CCNO	5	55221152	55268717	129	13	10164	0.7137	0.23771
+	DEFB118	20	31333618	31383923	54	8	10164	0.71325	0.23784
-	SCARF2	22	20414584	20472859	25	5	10164	0.71307	0.2379
-	SHROOM1	5	132812141	132866621	116	13	10164	0.71282	0.23798
-	CERS2	1	150955173	151010003	101	14	10164	0.71278	0.23799
+	GCAT	22	37772900	37827327	103	10	10164	0.71198	0.23824
+	ZNF534	19	52394321	52461939	235	12	10164	0.71198	0.23824
+	CLDN9	16	2977456	3024505	27	3	10164	0.71193	0.23825
+	OR2L3	1	248025682	248071620	69	5	10164	0.71181	0.23829
+	SULF1	8	69431624	69670912	269	41	10164	0.71175	0.23831
+	HIST1H3A	6	25985490	26030958	174	36	10164	0.7117	0.23832
+	LOC1001328	9	61947796	61996927	5	2	10164	0.71136	0.23843
-	SHF	15	45157214	45236329	228	16	10164	0.71133	0.23844
-	PTGER1	19	14462466	14510362	215	33	10164	0.71117	0.23849
+	SERINC2	1	31374565	31444680	248	15	10164	0.71105	0.23853
+	MED25	19	49783279	49850384	168	22	10164	0.7106	0.23867
-	USMG5	10	103379050	103431513	109	26	10164	0.7104	0.23873
+	ATRN	20	3436018	3661122	467	37	10164	0.71028	0.23877
-	OR7A5	19	14815742	14868245	152	23	10164	0.71026	0.23877
-	STC1	8	23831921	23889807	208	27	10164	0.71019	0.23879
+	OGFOD2	12	122939703	122990041	39	6	10164	0.70992	0.23888
-	ZNF787	19	56077363	56156373	120	10	10164	0.70989	0.23888
-	XCR1	3	46010799	46062487	227	16	10164	0.70887	0.2392
-	TAOK3	12	118139801	118407945	548	25	10164	0.70881	0.23922
-	TOLLIP	11	1264368	1344662	225	33	10164	0.70833	0.23937
-	MAPKAP1	9	125427394	125742234	511	26	10164	0.70825	0.23939
-	AFG3L2	18	12318944	12412309	231	19	10164	0.70824	0.2394
-	OR52E2	11	5048650	5094627	266	18	10164	0.70824	0.2394
-	ENTPD8	9	137424364	137476662	51	9	10164	0.70763	0.23959
-	OR1B1	9	122618579	122664535	135	24	10164	0.70748	0.23963
-	ALDH1A2	15	57943424	58100923	361	12	10164	0.70709	0.23976
-	MTRF1L	6	152977366	153037815	191	27	10164	0.70694	0.2398
-	UQCRC1	3	48588999	48644665	67	11	10164	0.70683	0.23984
+	LRRC16B	14	24016997	24079728	127	25	10164	0.70679	0.23985
-	ZNF606	19	57967072	58038346	184	12	10164	0.70662	0.2399
+	PCDHA9	5	140812772	141022344	374	17	10164	0.70659	0.23991
-	USP12	13	27056150	27206896	392	33	10164	0.70643	0.23996
-	SLC38A7	16	58655109	58719777	217	24	10164	0.70627	0.24001
-	SDHB	1	17008722	17089170	295	11	10164	0.70589	0.24013
-	NDRG1	8	133227171	133332587	232	37	10164	0.70575	0.24017
+	CLP1	11	57622744	57671865	93	17	10164	0.70569	0.24019

STRAND	SYMBOL	CHR	START	STOP	NSNPS	NPARAM	N	ZSTAT	P
+	EVPLL	17	18342692	18399652	163	16	10164	0.70559	0.24022
-	CLECL1	12	9705860	9768299	252	18	10164	0.70551	0.24025
+	LOC1053711	16	28562305	28613054	122	8	10164	0.70549	0.24025
+	MINOS1	1	19561977	19639821	211	20	10164	0.70536	0.24029
-	PDE6D	2	231722425	231816327	139	7	10164	0.70531	0.24031
+	GPATCH2L	14	76116885	76224473	396	23	10164	0.70527	0.24032
-	PSMC3	11	47408769	47461473	135	13	10164	0.70493	0.24043
-	CHRNA1	2	174737592	174799472	114	16	10164	0.70486	0.24045
-	P2RY14	3	151202115	151313628	278	29	10164	0.70482	0.24046
+	BAX	19	48919860	48971798	150	25	10164	0.70476	0.24048
-	CYP26B1	2	72119238	72182862	113	17	10164	0.70451	0.24056
-	DZIP1	13	95568202	95679706	176	28	10164	0.70349	0.24088
-	CCDC108	2	218992846	219076551	166	17	10164	0.70299	0.24103
-	LOC644215	10	97391237	97461151	154	16	10164	0.703	0.24103
+	LYPD6	2	149294985	149484148	316	23	10164	0.70264	0.24114
+	TSEN15	1	184016651	184105846	138	17	10164	0.70217	0.24129
-	ASB2	14	93924153	94011786	240	43	10164	0.7019	0.24137
+	SSFA2	2	181856716	181940738	130	12	10164	0.70188	0.24138
-	PDIA4	7	148993051	149063690	190	22	10164	0.70185	0.24139
-	POMT2	14	77264956	77355884	241	27	10164	0.70177	0.24141
+	PCDHA10	5	140820883	141022344	365	18	10164	0.70137	0.24153
-	CHRNA2	8	27449761	27514296	186	27	10164	0.70118	0.24159
-	PROP1	5	177982235	178031242	55	10	10164	0.70118	0.24159
+	PAQR8	6	52327128	52417777	287	44	10164	0.70117	0.2416
-	PCDH18	4	137508918	137567498	174	22	10164	0.70111	0.24162
+	PVRL2	19	44811136	44899228	246	32	10164	0.701	0.24165
+	ACSM5	16	20374520	20450987	215	27	10164	0.70099	0.24165
+	OR56A3	11	5912347	5958294	194	22	10164	0.70099	0.24166
+	QTRT1	19	10666436	10723367	95	13	10164	0.70093	0.24167
-	CFAP45	1	159862364	159935116	127	17	10164	0.70067	0.24176
-	RBM26	13	79301827	79441258	369	9	10164	0.70056	0.24179
+	PSME1	14	24101158	24148967	63	8	10164	0.70044	0.24183
+	LOC1009966	2	459739	506631	138	22	10164	0.70008	0.24194
+	VWA5A	11	124080404	124156912	268	49	10164	0.69982	0.24202
-	HSP90AA1	14	102070738	102174759	219	24	10164	0.69956	0.2421
+	AMD1	6	110779621	110905713	316	21	10164	0.69934	0.24217
-	XPA	9	97644398	97732409	311	14	10164	0.69919	0.24222
-	ALDH1L1	3	126093561	126216526	509	35	10164	0.69894	0.24229
+	TDRD9	14	103893438	104062667	564	22	10164	0.6989	0.24231
-	STMN3	20	63629705	63688610	261	34	10164	0.6982	0.24253
-	EPM2AIP1	3	36975866	37028304	74	7	10164	0.69793	0.24261
-	LOC1001304	2	213266553	213319205	46	11	10164	0.6978	0.24265
+	C14orf180	14	104544684	104599847	232	16	10164	0.6973	0.24281
+	SC5D	11	121257679	121323410	214	14	10164	0.69727	0.24282
-	CHDH	3	53806297	53881393	222	36	10164	0.69724	0.24283
+	TSPAN9	12	3042355	3296564	685	71	10164	0.6971	0.24287
+	DLAT	11	111989814	112074278	89	9	10164	0.69702	0.2429
+	OR5K4	3	98318854	98364819	171	18	10164	0.69668	0.243
+	TGS1	8	55738232	55835448	294	15	10164	0.69663	0.24302
-	IRS1	2	226721317	226833790	246	27	10164	0.69659	0.24303
-	FHOD1	16	67219389	67282522	59	12	10164	0.69659	0.24303
+	BCO2	11	112140485	112228929	190	19	10164	0.69646	0.24307
+	ACPP	3	132282367	132378302	407	28	10164	0.6963	0.24312
-	TXNDC16	14	52420590	52587583	552	35	10164	0.69598	0.24322
-	ARIH2OS	3	48907788	48954385	67	9	10164	0.69575	0.24329
-	HEG1	3	124955710	125090958	375	43	10164	0.6956	0.24334
-	ZNF488	10	47355496	47419333	44	6	10164	0.69521	0.24346
+	OAF	11	120176039	120239941	194	30	10164	0.69507	0.24351
-	ZMYM6NB	1	34971526	35020347	84	4	10164	0.6947	0.24362
-	LMF1	16	843634	1016318	473	15	10164	0.69453	0.24367
-	AKTIP	16	53481280	53539411	164	28	10164	0.69444	0.2437
+	RPGRIP1	14	21252962	21361301	19	2	10164	0.69413	0.2438
-	FCGBP	19	39853323	39969626	235	34	10164	0.69383	0.24389

STRAND	SYMBOL	CHR	START	STOP	NSNPS	NPARAM	N	ZSTAT	P
-	FDXACB1	11	111864056	111914457	108	13	10164	0.69375	0.24392
+	CHST11	12	104421914	104772014	1231	122	10164	0.69359	0.24397
+	VEGFB	11	64199584	64249264	166	37	10164	0.69345	0.24401
-	PIRT	17	10812475	10873101	183	27	10164	0.69346	0.24401
+	UBL4B	1	110077440	110123947	122	18	10164	0.69331	0.24406
-	ZSCAN5A	19	56211303	56265399	43	8	10164	0.69315	0.24411
-	GABRR2	6	89246061	89350299	276	50	10164	0.69293	0.24418
+	MRPS9	2	105003025	105109960	249	9	10164	0.6929	0.24419
-	AREL1	14	74651252	74748115	169	19	10164	0.69267	0.24426
-	BCL10	1	85255776	85311904	191	30	10164	0.69259	0.24428
+	MYCT1	6	152662895	152757175	398	26	10164	0.69259	0.24428
-	FRMPD2	10	48140001	48309898	330	21	10164	0.69239	0.24434
+	DIXDC1	11	111892144	112032650	167	20	10164	0.69172	0.24456
-	TEX33	22	36981119	37042863	183	12	10164	0.69124	0.24471
+	C2orf69	2	199876256	199938273	147	26	10164	0.69107	0.24476
-	GALNT6	12	51341870	51426955	229	40	10164	0.69101	0.24478
+	CHAMP1	13	114279490	114337328	172	17	10164	0.69092	0.24481
+	VEGFA	6	43735209	43796487	175	48	10164	0.69071	0.24487
+	HIST1H2BH	6	26216651	26262075	201	23	10164	0.69038	0.24498
-	TNKS1BP1	11	57289629	57359952	111	19	10164	0.68989	0.24513
-	RERE	1	8342404	8852691	632	19	10164	0.68985	0.24515
-	SIRT2	19	38868555	38934862	161	30	10164	0.68982	0.24515
+	YTHDC2	5	113478683	113605287	520	21	10164	0.68933	0.24531
+	ACADVL	17	7182125	7235267	81	17	10164	0.6892	0.24535
+	MPST	22	36984642	37039822	156	9	10164	0.68921	0.24535
+	PXYLP1	3	141196825	141304903	376	39	10164	0.68905	0.2454
+	PCDHA13	5	140847269	141022344	328	15	10164	0.68862	0.24553
-	LOC1027237	16	71419996	71477046	138	21	10164	0.68836	0.24561
-	AGA	4	177420774	177477503	202	28	10164	0.68828	0.24564
+	GRP	18	59184118	59240771	147	20	10164	0.68817	0.24567
+	MTHFD2	2	74163563	74225298	72	4	10164	0.68766	0.24583
+	HTRA4	8	38939149	38998662	112	16	10164	0.68743	0.24591
-	CIDEC	3	9856710	9915262	174	25	10164	0.6874	0.24592
+	SH3BGRL3	1	26244722	26291522	135	11	10164	0.68717	0.24599
-	CRYGA	2	208150740	208198573	167	20	10164	0.6871	0.24601
+	DYNLRB1	20	34481355	34550958	108	19	10164	0.68692	0.24607
-	DENND6A	3	57615454	57728089	232	19	10164	0.68665	0.24615
-	HOMER1	5	79363824	79548836	532	53	10164	0.68632	0.24626
-	TIGD4	4	152759354	152814789	102	11	10164	0.68626	0.24628
+	RSPH9	6	43610030	43681013	156	17	10164	0.68616	0.24631
+	DNAJC4	11	64195234	64244281	160	37	10164	0.68603	0.24635
+	HIST1H4A	6	25986679	26032050	179	37	10164	0.68586	0.2464
+	PHRF1	11	541220	622222	303	24	10164	0.68571	0.24645
+	COL20A1	20	63258169	63344847	176	17	10164	0.68557	0.24649
+	KIF1B	1	10175706	10391603	522	18	10164	0.68545	0.24653
+	PRSS23	11	86756059	86962910	570	46	10164	0.68536	0.24656
+	EGFL8	6	32129605	32178285	191	55	10164	0.6852	0.24661
+	FITM1	14	24096466	24142849	63	9	10164	0.68511	0.24664
+	RPF2	6	110947017	111038263	464	18	10164	0.68507	0.24665
-	TAF1A	1	222547902	222625096	177	19	10164	0.68468	0.24677
+	LMTK2	7	98071875	98219636	504	34	10164	0.68431	0.24689
-	SLC13A3	20	46547823	46719485	266	44	10164	0.68415	0.24694
-	PAPOLB	7	4847738	4896994	152	14	10164	0.684	0.24699
-	CDHR5	11	606565	660067	191	23	10164	0.68388	0.24703
-	MAP9	4	155332658	155411970	278	24	10164	0.68384	0.24704
+	ALCAM	3	105331713	105586913	714	32	10164	0.68371	0.24708
+	ZNF806	2	132272144	132328747	1	1	10164	0.68356	0.24713
-	STAR	8	38132700	38186265	74	12	10164	0.68348	0.24715
+	SPATS1	6	44307660	44387167	181	27	10164	0.68332	0.2472
-	SCAND1	20	35943617	35990359	100	15	10164	0.68321	0.24724
-	ZFP37	9	113031815	113091791	78	14	10164	0.68313	0.24726
+	LRRC37B	17	31972372	32063500	18	4	10164	0.68297	0.24731
-	TMEM68	8	55728744	55808407	247	21	10164	0.68268	0.2474

STRAND	SYMBOL	CHR	START	STOP	NSNPS	NPARAM	N	ZSTAT	P
-	BEND5	1	48717523	48811969	168	24	10164	0.68266	0.24741
+	PBX1	1	164524360	164896047	409	56	10164	0.68244	0.24748
+	NUDT17	1	145809985	145858753	27	4	10164	0.68225	0.24754
+	PIGT	20	45381067	45436245	116	14	10164	0.68199	0.24762
+	C18orf63	18	74280875	74369187	357	16	10164	0.68176	0.2477
-	STMN4	8	27225323	27293865	217	36	10164	0.68158	0.24775
-	ITIH4	3	52802990	52865701	306	12	10164	0.68151	0.24777
-	MROH6	8	143556189	143607758	234	28	10164	0.68148	0.24778
-	CCDC71	3	49152535	49201352	98	12	10164	0.68103	0.24793
-	ADH7	4	99402261	99470510	188	20	10164	0.68043	0.24812
-	CCDC93	2	117905478	118049163	423	22	10164	0.68008	0.24823
-	KIF24	9	34242380	34394914	334	23	10164	0.68005	0.24824
+	KRTAP13-4	21	30395276	30440758	134	14	10164	0.67999	0.24826
-	MOXD1	6	132286055	132436534	396	32	10164	0.67975	0.24833
-	SELK	3	53875199	53926962	118	13	10164	0.67942	0.24844
+	PPAN	19	10071223	10121299	75	17	10164	0.67921	0.2485
-	SLC19A2	1	169453909	169521079	167	24	10164	0.67899	0.24857
-	EPS15	1	51344263	51554354	245	23	10164	0.67881	0.24863
+	ZNF215	11	6891418	7015354	417	25	10164	0.67852	0.24872
+	SNTG2	2	915868	1377645	2058	65	10164	0.67815	0.24884
-	RXRG	1	165390922	165480355	382	46	10164	0.67794	0.2489
-	KRTAP10-6	21	44581268	44627505	189	7	10164	0.67765	0.249
-	FRS3	6	41760176	41814905	102	15	10164	0.67759	0.24902
-	ZNF25	10	37939572	38011980	153	16	10164	0.67757	0.24902
-	SDCBP2	20	1299909	1364235	174	24	10164	0.6766	0.24933
+	PSMC4	19	39936005	39991764	120	8	10164	0.67639	0.2494
-	NRD1	1	51779192	51913937	307	16	10164	0.67614	0.24947
+	QKI	6	163379643	163588596	160	14	10164	0.67607	0.2495
+	HSPB3	5	54420601	54466384	179	23	10164	0.67596	0.24953
+	KRTAP20-2	21	30600264	30645461	96	10	10164	0.67562	0.24964
-	CDKL4	2	39164937	39281810	329	18	10164	0.67561	0.24964
-	ASB3	2	53659980	53822009	555	35	10164	0.67539	0.24971
+	LRRC16A	6	25244428	25630530	1907	123	10164	0.67533	0.24973
+	TPX2	20	31698819	31811802	152	11	10164	0.67513	0.2498
-	MOB1A	2	74144409	74213886	86	5	10164	0.67495	0.24985
+	CRTAP	3	33078958	33157773	242	34	10164	0.67492	0.24986
+	PCDHA11	5	140832513	141022344	352	17	10164	0.67494	0.24986
+	IFNL1	19	39261325	39308672	119	4	10164	0.6746	0.24996
-	TP53I3	2	24067428	24120215	182	9	10164	0.67453	0.24999
-	IFNA6	9	21340318	21385887	115	24	10164	0.67425	0.25008
-	MAFA	8	143418060	143465432	201	22	10164	0.67424	0.25008
+	ZNF441	19	11732000	11794078	101	8	10164	0.67399	0.25016
+	SLC22A15	1	115941498	116080054	158	13	10164	0.67379	0.25022
-	YBX2	17	7278252	7329557	106	20	10164	0.67361	0.25028
-	XKR3	22	16773412	16856694	266	30	10164	0.6734	0.25035
+	TCAF2	7	143585952	143740080	30	5	10164	0.67321	0.25041
-	PHF10	6	169693905	169759010	144	14	10164	0.67171	0.25089
-	CCDC80	3	112594386	112676143	186	30	10164	0.67152	0.25095
-	SLC17A2	6	25902754	25965726	287	28	10164	0.67122	0.25104
+	FKBP10	17	41777262	41833217	112	14	10164	0.67114	0.25107
+	CYP20A1	2	203203441	203315840	205	19	10164	0.66985	0.25148
+	SYT8	11	1792479	1847521	220	35	10164	0.66977	0.2515
-	AGTPBP1	9	85536539	85777029	417	28	10164	0.66965	0.25154
+	BUB3	10	123119244	123175370	150	28	10164	0.66944	0.25161
+	PPIF	10	79312464	79365334	112	17	10164	0.66944	0.25161
-	ZNF121	19	9555616	9619545	149	9	10164	0.66928	0.25166
-	NKX2-6	8	23692451	23741598	248	33	10164	0.66917	0.25169
+	CREBL2	12	12576833	12655108	142	17	10164	0.66914	0.2517
+	ERLIN2	8	37701579	37767801	29	17	10164	0.66896	0.25176
-	ANGEL2	1	212982182	213050875	117	18	10164	0.66877	0.25182
+	UPK2	11	118921298	118968559	146	30	10164	0.66871	0.25184
-	ESD	13	46761256	46832681	123	14	10164	0.66866	0.25186
+	TRIM38	6	25927689	25997329	284	33	10164	0.66862	0.25187

STRAND	SYMBOL	CHR	START	STOP	NSNPS	NPARAM	N	ZSTAT	P
+	PCDHA12	5	140840302	141022344	336	16	10164	0.66856	0.25189
+	SPINT2	19	38229458	38302614	336	44	10164	0.6678	0.25213
+	LOC1053736	2	127302789	127385404	140	16	10164	0.66778	0.25214
-	FAM19A4	3	68721764	68967610	900	42	10164	0.66764	0.25218
+	CYP1A2	15	74713843	74766600	171	31	10164	0.66726	0.2523
+	OR2T3	1	248438351	248484307	31	2	10164	0.66715	0.25234
-	TSGA10	2	98987261	99189920	564	21	10164	0.66705	0.25237
-	MST1R	3	49876981	49938873	93	12	10164	0.66702	0.25238
+	AMH	19	2214114	2262073	113	23	10164	0.66675	0.25246
+	SLCO1B3	12	20775704	20926911	865	30	10164	0.66673	0.25247
+	TOR3A	1	179046977	179105996	200	25	10164	0.66664	0.2525
+	SLC37A2	11	125028067	125100516	257	35	10164	0.66609	0.25268
+	GATA3	10	8010428	8085201	303	56	10164	0.66583	0.25276
-	POTEC	18	14499778	14578600	130	14	10164	0.66559	0.25284
-	GOLGB1	3	121653199	121785811	338	24	10164	0.66528	0.25294
+	TYR	11	89142858	89305759	509	32	10164	0.66507	0.253
-	VEPH1	3	157249742	157538626	972	46	10164	0.665	0.25302
-	RNF148	7	122691666	122737967	108	15	10164	0.66498	0.25303
-	RTBDN	19	12815477	12873267	73	5	10164	0.6647	0.25312
-	RAET1G	6	149906878	149958152	178	22	10164	0.66471	0.25312
+	LOC1053771	21	13992416	14070261	150	18	10164	0.66454	0.25317
-	ACBD3	1	226134679	226221722	250	34	10164	0.66447	0.2532
-	HOXA4	7	27118507	27165780	103	14	10164	0.66415	0.2533
-	RAB11FIP2	10	117994916	118081877	75	13	10164	0.66413	0.2533
-	LOC1002890	20	28547090	28637838	32	7	10164	0.66393	0.25337
+	EIF2A	3	150511690	150596016	166	17	10164	0.66351	0.2535
-	FAM174B	15	92607448	92847338	946	73	10164	0.66338	0.25354
-	SEMA3A	7	83946805	84527724	2026	82	10164	0.66328	0.25357
+	PCYOX1	2	70223099	70291185	170	14	10164	0.66327	0.25358
+	PPFIA1	11	70235687	70394501	330	15	10164	0.66303	0.25365
+	FARP2	2	241321249	241504842	519	31	10164	0.6627	0.25376
+	FAM160B2	8	22054184	22114380	192	20	10164	0.66252	0.25382
-	TNNC2	20	45813214	45868323	194	28	10164	0.66245	0.25384
+	TBR1	2	161381109	161435062	75	9	10164	0.66242	0.25385
-	DDRGK1	20	3180366	3239649	193	26	10164	0.66215	0.25394
-	SGCZ	8	14079864	15273283	7070	234	10164	0.66214	0.25394
+	OR10G8	11	123994623	124040558	254	19	10164	0.66191	0.25401
-	KANK2	19	11154267	11232567	184	31	10164	0.6616	0.25411
-	FASTKD5	20	3136519	3194886	86	12	10164	0.66149	0.25415
-	TMPPE	3	33080416	33131801	156	23	10164	0.6614	0.25418
-	OR13J1	9	35859463	35905401	138	22	10164	0.66101	0.2543
+	ZNF8	19	58243952	58305888	187	16	10164	0.66083	0.25436
+	PSAT1	9	78262075	78340093	249	37	10164	0.66073	0.25439
+	ALX1	12	85244795	85311784	109	17	10164	0.6595	0.25479
+	CMAS	12	22011174	22075674	33	3	10164	0.65948	0.25479
-	SLC26A8	6	35933514	36059636	466	39	10164	0.65936	0.25483
-	TMEM217	6	37202178	37294005	207	21	10164	0.65928	0.25486
+	SCAF1	19	49605503	49668649	147	18	10164	0.65903	0.25494
+	FNIP2	4	158734030	158918050	253	15	10164	0.659	0.25495
-	TBC1D2B	15	77984985	78112667	346	28	10164	0.65886	0.25499
+	LOC1027246	7	32737427	32843146	376	17	10164	0.65887	0.25499
-	CMTM4	16	66588214	66731707	325	19	10164	0.65864	0.25506
+	GTF2IRD1	7	74418790	74612605	303	20	10164	0.65852	0.2551
+	SV2B	15	91064721	91311309	421	26	10164	0.65852	0.2551
-	LOC1053789	1	39478834	39526993	81	11	10164	0.65834	0.25516
+	DNAJC25-GN	9	111596352	111680246	331	28	10164	0.65796	0.25528
-	DUSP3	17	43756121	43814000	124	12	10164	0.65775	0.25535
-	NME7	1	169121998	169402963	925	21	10164	0.65765	0.25538
-	KCNA2	1	110583580	110666514	168	22	10164	0.65712	0.25555
+	ADAMTSL4	1	150514369	150570937	117	24	10164	0.65699	0.25559
-	LTBP2	14	74488183	74653609	428	36	10164	0.65698	0.2556
+	AADACL3	1	12681115	12738759	146	16	10164	0.65665	0.2557
+	CLPSL2	6	35741594	35789552	278	40	10164	0.65662	0.25571

STRAND	SYMBOL	CHR	START	STOP	NSNPS	NPARAM	N	ZSTAT	P
+	REM2	14	22848165	22897680	106	19	10164	0.65643	0.25577
-	FSHR	2	48856435	49189527	1352	72	10164	0.65603	0.2559
+	TIGD5	8	143562904	143610315	212	24	10164	0.6557	0.25601
+	NTF3	12	5397114	5505299	360	43	10164	0.65567	0.25602
+	LY6G6D	6	31680356	31727804	210	52	10164	0.65563	0.25603
-	GCFC2	2	75652705	75745986	253	29	10164	0.65542	0.2561
+	LOC1053726	20	56523911	56606608	169	23	10164	0.65542	0.2561
-	C1orf86	1	2174460	2247720	204	41	10164	0.65539	0.25611
+	MIXL1	1	226188549	226237056	123	21	10164	0.65519	0.25617
+	IL17RC	3	9882074	9943621	162	23	10164	0.65517	0.25618
-	UTP6	17	31853171	31936710	109	3	10164	0.65462	0.25636
+	ZDHHC5	11	57633002	57711187	143	22	10164	0.65432	0.25645
-	ZNF607	19	37686363	37754790	177	26	10164	0.65417	0.2565
+	USO1	4	75689522	75824289	334	15	10164	0.65403	0.25654
-	SEMA3D	7	84985192	85221855	369	36	10164	0.65376	0.25663
+	ADO	10	62769756	62818479	127	25	10164	0.6536	0.25669
+	ZNF268	12	133146409	133217112	126	8	10164	0.653	0.25688
+	OR2T5	1	248453589	248499536	26	2	10164	0.65293	0.2569
-	LSS	21	46178446	46263824	323	29	10164	0.65243	0.25706
-	LOC1053749	6	27115897	27165449	127	35	10164	0.6523	0.2571
-	LOC1053719	17	77247737	77317699	217	24	10164	0.65218	0.25714
+	CCDC28A	6	138738520	138818327	225	25	10164	0.65204	0.25719
+	HK1	10	69234984	69411882	524	68	10164	0.65198	0.25721
-	SHOX2	3	158086011	158141202	163	16	10164	0.65198	0.25721
+	PRAMEF1	1	12756397	12806628	167	14	10164	0.65181	0.25726
-	ANGPTL5	11	101880674	101951715	180	25	10164	0.65161	0.25732
-	SIN3A	15	75359379	75490815	237	34	10164	0.65148	0.25737
+	PHLPP1	18	62680439	62990443	329	38	10164	0.65121	0.25746
-	TAAR5	6	132578592	132624738	255	41	10164	0.6509	0.25756
+	BPHL	6	3083376	3163198	130	11	10164	0.65066	0.25763
+	AHSA1	14	77422875	77479472	180	14	10164	0.65056	0.25767
+	KIT	4	54622928	54750715	359	36	10164	0.6504	0.25772
+	LOC1009965	12	132515729	132564890	179	23	10164	0.65033	0.25774
+	KIAA1522	1	32706911	32784970	146	15	10164	0.65013	0.2578
+	SGSM1	22	24771221	24936846	741	80	10164	0.64999	0.25785
+	TMEM59L	19	18572435	18631039	127	11	10164	0.64972	0.25794
+	FOXK2	17	82484718	82614607	160	24	10164	0.64944	0.25803
+	MRPS12	19	38895708	38943020	129	19	10164	0.64936	0.25805
+	UBE2F	2	237931945	238052782	401	29	10164	0.64908	0.25814
-	WNT9A	1	227908658	227982975	358	34	10164	0.64872	0.25826
-	DCLRE1C	10	14894611	14989432	374	39	10164	0.64859	0.2583
-	ZNF562	19	9638654	9710302	218	15	10164	0.64843	0.25835
-	SPOCK2	10	72049034	72124032	199	24	10164	0.64821	0.25843
-	PMFBP1	16	72106698	72212002	325	28	10164	0.64787	0.25853
+	MURC	9	100541867	100598402	172	22	10164	0.64789	0.25853
-	OR2AE1	7	99866062	99912033	102	10	10164	0.64774	0.25858
+	LRTM2	12	1785267	1846753	164	34	10164	0.64772	0.25858
-	POLN	4	2061958	2277164	535	16	10164	0.6473	0.25872
+	OR4D11	11	59468576	59514511	82	12	10164	0.64671	0.25891
+	SRP9	1	225742813	225800466	248	43	10164	0.64666	0.25892
-	SNCA	4	89714099	89873314	531	24	10164	0.64666	0.25893
-	FLRT3	20	14313993	14372667	173	22	10164	0.64646	0.25899
-	SIGLEC14	19	51632553	51681879	228	18	10164	0.6463	0.25904
-	DNAJB1	19	14504764	14564711	153	23	10164	0.64611	0.25911
-	CPOX	3	98566414	98628668	177	22	10164	0.64594	0.25916
-	FBXO17	19	38931401	39010813	267	29	10164	0.6457	0.25924
+	WIPF2	17	40184304	40294136	119	4	10164	0.64563	0.25926
-	SPINT3	20	45502461	45550624	184	16	10164	0.64542	0.25933
+	ULBP2	6	149906938	149959235	179	22	10164	0.64542	0.25933
-	FAM71B	5	157152332	157201268	160	17	10164	0.64535	0.25935
-	HDAC2	6	113926156	114006195	223	33	10164	0.64474	0.25955
+	BMI1	10	22286210	22341485	26	14	10164	0.64448	0.25963
+	ECT2L	6	138761051	138914070	445	50	10164	0.64449	0.25963

STRAND	SYMBOL	CHR	START	STOP	NSNPS	NPARAM	N	ZSTAT	P
+	COMMD3-BM	10	22281383	22341485	26	14	10164	0.64448	0.25963
+	ADGRD1	12	130918907	131151465	720	57	10164	0.64446	0.25964
-	PPAP2C	19	271040	326435	50	4	10164	0.64436	0.25967
-	HRASLS5	11	63451404	63526208	116	18	10164	0.64396	0.2598
-	HIST1H2BG	6	26206200	26251644	228	21	10164	0.64391	0.25982
-	ABCB1	7	87493863	87748323	608	45	10164	0.64386	0.25983
-	ATP13A2	1	16975958	17046972	137	10	10164	0.64388	0.25983
-	SYT6	1	114079292	114188850	319	53	10164	0.64379	0.25986
-	EHMT2	6	31869759	31932707	324	59	10164	0.64359	0.25992
-	KRTAP10-3	21	44548023	44593760	214	18	10164	0.64344	0.25997
-	NHP2	5	178139463	178188960	185	19	10164	0.64323	0.26004
+	PRKCZ	1	2015470	2195395	748	56	10164	0.64307	0.26009
-	LOC1001313	16	68003436	68050911	58	5	10164	0.64234	0.26032
-	RHBDD3	22	29249855	29303185	123	34	10164	0.64216	0.26039
-	MAGI2	7	78007055	79488805	5295	288	10164	0.64184	0.26049
+	B3GALT4	6	33242140	33288825	194	28	10164	0.6417	0.26053
-	MZB1	5	139377568	139424916	59	9	10164	0.64167	0.26054
+	JAM2	21	25604282	25727562	457	37	10164	0.64161	0.26056
-	SYNC	1	32669906	32737760	184	11	10164	0.6414	0.26063
+	MYO7B	2	127500693	127647728	317	23	10164	0.64125	0.26068
+	ZNF696	8	143256389	143309952	188	9	10164	0.64112	0.26072
+	NOL11	17	67682833	67754208	247	21	10164	0.64101	0.26076
+	RNF31	14	24112450	24170661	82	14	10164	0.64086	0.26081
-	STON2	14	81250650	81462404	619	51	10164	0.64076	0.26084
-	CLEC4G	19	7718957	7767171	55	10	10164	0.6407	0.26086
+	SPAG16	2	213249379	214420501	2451	75	10164	0.64058	0.2609
-	REG1B	2	79075023	79123024	173	30	10164	0.64032	0.26098
+	IRX5	16	54896199	54944485	36	6	10164	0.64034	0.26098
+	GDF10	10	47265224	47323583	69	15	10164	0.64007	0.26107
-	LOC1027233	20	29447393	29531909	9	3	10164	0.63984	0.26114
-	LOC1053716	1	182054295	182279444	473	47	10164	0.63982	0.26114
-	AGAP4	10	45815592	45888877	77	13	10164	0.63976	0.26117
+	L3MBTL2	22	41170232	41241272	215	20	10164	0.63964	0.2612
-	ADAM17	2	9479263	9590788	206	15	10164	0.6395	0.26125
-	PLCZ1	12	18631990	18773059	395	32	10164	0.63937	0.26129
-	EIF2D	1	206561297	206647551	191	34	10164	0.63918	0.26135
-	PIK3CB	3	138642698	138869938	255	9	10164	0.63919	0.26135
+	LOC1053717	1	155966153	156043225	124	23	10164	0.6389	0.26144
-	FAM208A	3	56610132	56718242	424	21	10164	0.63878	0.26148
-	NPVF	7	25214570	25263486	205	27	10164	0.63873	0.2615
-	YPEL4	11	57635087	57684944	94	17	10164	0.63868	0.26152
+	LSR	19	35213656	35277964	261	32	10164	0.63858	0.26155
-	C1orf204	1	159824474	159890347	151	23	10164	0.63765	0.26185
-	GRM6	5	178968327	179030123	280	27	10164	0.63762	0.26186
-	GCNT4	5	75017464	75065899	146	24	10164	0.63701	0.26206
+	LRRC10B	11	61473800	61521018	103	14	10164	0.63644	0.26225
+	GATSL2	7	74929818	75041532	68	6	10164	0.63639	0.26226
-	AP1M2	19	10562671	10622315	200	25	10164	0.63628	0.2623
+	BFSP2	3	133364995	133485212	279	38	10164	0.63624	0.26231
+	MUC22	6	30970952	31045570	901	44	10164	0.63623	0.26231
+	HIST1H4F	6	26205426	26250793	231	21	10164	0.63613	0.26235
-	AXIN2	17	65518563	65596622	183	37	10164	0.63573	0.26248
-	ENOX1	13	43203130	43821980	1717	97	10164	0.63545	0.26257
-	C12orf77	12	24983424	25032439	159	29	10164	0.63538	0.26259
+	SLC9A5	16	67212551	67282191	67	12	10164	0.63503	0.2627
-	KRTAP6-1	21	30603686	30648904	88	9	10164	0.63499	0.26272
-	CSMD2	1	33503999	34200842	1694	150	10164	0.63483	0.26277
-	WFDC10B	20	45674651	45740019	302	18	10164	0.63444	0.2629
+	MRPS7	17	75226668	75276376	154	9	10164	0.63398	0.26305
-	ITGA10	1	145881208	145945411	21	4	10164	0.63387	0.26308
+	CRELD1	3	9898788	9955413	156	23	10164	0.6333	0.26327
+	PALM	19	673953	758330	4	1	10164	0.63323	0.26329
-	ELMO2	20	46356049	46442051	96	8	10164	0.63324	0.26329

STRAND	SYMBOL	CHR	START	STOP	NSNPS	NPARAM	N	ZSTAT	P
-	SF3B6	2	24057584	24111444	178	10	10164	0.6329	0.2634
+	ALG10	12	33987281	34038302	131	15	10164	0.63264	0.26348
+	FCF1	14	74678117	74746691	126	16	10164	0.63261	0.26349
+	ANO7	2	241153509	241249595	350	38	10164	0.63243	0.26355
+	MGAM	7	141953888	142116747	40	7	10164	0.63171	0.26379
-	TMEM114	16	8516549	8625193	571	51	10164	0.63166	0.2638
+	ZNF33A	10	37975483	38070067	187	17	10164	0.63151	0.26385
+	TEKT4	2	94832767	94886829	82	11	10164	0.6313	0.26392
+	ZNF596	8	197137	257340	155	19	10164	0.63131	0.26392
+	RPS18	6	33237075	33286504	203	30	10164	0.63122	0.26395
+	PCDHB13	5	141178919	141227421	129	14	10164	0.63096	0.26403
+	LOC1019291	6	32220717	32417822	2642	56	10164	0.63071	0.26412
-	LOC440292	15	77931393	77979561	190	29	10164	0.63042	0.26421
-	IFNA21	9	21155637	21201660	261	21	10164	0.6303	0.26425
+	C17orf97	17	375327	424666	55	8	10164	0.63027	0.26426
+	ENAM	4	70593744	70656819	117	25	10164	0.63023	0.26427
+	RGMB	5	98734295	98806494	155	17	10164	0.62993	0.26437
+	NOC4L	12	132109422	132162473	11	6	10164	0.62987	0.26439
-	RNF208	9	137210247	137256323	124	19	10164	0.62963	0.26447
-	GLIS1	1	53496233	53769204	747	54	10164	0.62954	0.2645
+	CCDC97	19	41275189	41334883	113	8	10164	0.62951	0.26451
-	CRYAB	11	111898620	111948213	71	13	10164	0.62911	0.26464
-	LOC1019272	16	50090478	50188014	198	19	10164	0.62898	0.26468
-	ZNF214	11	6986613	7055376	181	10	10164	0.6288	0.26474
-	AKR7A3	1	19264325	19324346	246	16	10164	0.62831	0.2649
-	CLIP1	12	122261432	122457632	361	25	10164	0.62813	0.26496
+	GEMIN2	14	39079284	39146973	216	32	10164	0.62777	0.26508
+	ZNF507	19	32310608	32397667	166	14	10164	0.62772	0.26509
-	TUBGCP5	15	22981677	23074673	61	7	10164	0.62769	0.2651
-	ARHGAP9	12	57462255	57518959	126	18	10164	0.6276	0.26513
+	NFATC2IP	16	28915988	28976465	76	11	10164	0.62762	0.26513
+	GFM1	3	158609497	158702572	338	13	10164	0.62713	0.26529
+	LOC1053739	2	97056741	97110360	93	14	10164	0.62711	0.26529
-	CCDC8	19	46400329	46448662	212	24	10164	0.62709	0.2653
-	SPTSSA	14	34422938	34497262	162	20	10164	0.62702	0.26532
+	RWDD2A	6	83158313	83208932	125	24	10164	0.62699	0.26533
-	KCNAB3	17	7911859	7964435	59	11	10164	0.62693	0.26535
-	ANKS3	16	4686510	4769377	141	4	10164	0.62653	0.26549
-	ADI1	2	3487919	3554579	234	27	10164	0.62648	0.2655
-	LOC1053756	8	98913917	98977358	65	10	10164	0.62637	0.26553
+	CDH13	16	82591794	83806610	5321	349	10164	0.62627	0.26557
-	SEC23A	14	39021919	39138243	351	28	10164	0.62577	0.26573
-	FAM53B	10	124609294	124779361	605	52	10164	0.62574	0.26574
+	ARL6IP4	12	122945060	122992913	37	6	10164	0.62558	0.2658
-	PRR23A	3	138993962	139041268	83	12	10164	0.62497	0.266
+	FAM103A1	15	82951203	83001057	79	17	10164	0.62479	0.26605
+	TMEM50A	1	25303298	25372361	20	2	10164	0.62477	0.26606
-	AHNAK	11	62423542	62581860	175	39	10164	0.62437	0.26619
+	CPSF3	2	9388537	9483110	180	11	10164	0.62401	0.26631
+	FANK1	10	125861224	126019592	565	36	10164	0.62402	0.26631
+	ODF1	8	102516620	102571017	301	38	10164	0.62375	0.2664
-	CTDSP2	12	57809927	57881964	127	16	10164	0.62358	0.26645
+	CHMP1B	18	11816390	11864449	105	7	10164	0.62352	0.26647
+	SMIM13	6	11059033	11148738	339	31	10164	0.62338	0.26652
-	GPR85	7	113070413	113122778	129	13	10164	0.62312	0.2666
+	TMEM52B	12	10135599	10201804	203	19	10164	0.6231	0.26661
+	LOC1053693	11	57287395	57335956	70	14	10164	0.62274	0.26673
-	PDCD7	15	65107379	65168836	79	9	10164	0.62253	0.2668
+	FAM19A3	1	112683994	112737235	94	16	10164	0.62232	0.26687
-	CARD16	11	105030306	105080416	57	9	10164	0.62178	0.26704
+	SNTB2	16	69152147	69319052	348	23	10164	0.6217	0.26707
-	RGS7	1	240765514	241392230	1398	119	10164	0.62141	0.26717
-	MAN2C1	15	75345792	75404114	92	21	10164	0.62126	0.26721

STRAND	SYMBOL	CHR	START	STOP	NSNPS	NPARAM	N	ZSTAT	P
+	FAM118A	22	45274200	45351955	324	25	10164	0.62103	0.26729
+	C19orf60	19	18553685	18602337	107	8	10164	0.62073	0.26739
+	SH2B1	16	28811600	28884213	124	6	10164	0.62039	0.2675
-	DPF3	14	72599032	72929116	1097	106	10164	0.62027	0.26754
-	KRT78	12	52827804	52883994	332	21	10164	0.62004	0.26762
-	GOLGA2	9	128245829	128311007	124	14	10164	0.6199	0.26766
+	NIPAL4	5	157425019	157484722	188	16	10164	0.61953	0.26778
-	GRPEL1	4	7050053	7103073	129	14	10164	0.61948	0.2678
+	NPIPB15	16	74340001	74402115	43	10	10164	0.61949	0.2678
-	UCKL1	20	63929829	63994435	119	15	10164	0.61927	0.26787
+	PCTP	17	55715979	55853209	249	17	10164	0.61871	0.26805
-	MBD2	18	54141601	54259788	260	29	10164	0.61845	0.26814
-	PPP1R27	17	81823492	81870050	1	1	10164	0.61844	0.26814
-	FAM195B	17	81812361	81868294	1	1	10164	0.61844	0.26814
-	ZNF582	19	56364832	56428585	144	25	10164	0.61831	0.26819
-	CFC1	2	130582165	130634575	1	1	10164	0.61807	0.26826
+	POTEJ	2	130576413	130668448	1	1	10164	0.61807	0.26826
-	PPM1F	22	21909419	21987878	206	18	10164	0.61768	0.26839
-	CBLN1	16	49267917	49316831	97	18	10164	0.61766	0.2684
-	GRXCR2	5	145848387	145972176	274	38	10164	0.61741	0.26848
-	GSTA1	6	52781380	52838866	140	9	10164	0.61728	0.26853
+	HIST1H2BE	6	26148796	26194230	230	30	10164	0.61725	0.26854
+	KIAA1045	9	34775040	34992544	657	42	10164	0.61694	0.26864
-	ELOVL4	6	79904812	79982598	255	19	10164	0.6169	0.26865
-	ADAM20	14	70510877	70592701	277	26	10164	0.61683	0.26867
+	GRASP	12	51971937	52025889	56	11	10164	0.61677	0.26869
+	TAB1	22	39364754	39447127	192	15	10164	0.61671	0.26871
+	LDB3	10	86631717	86746072	425	45	10164	0.61637	0.26883
-	SYCE3	22	50541112	50597899	246	21	10164	0.61612	0.26891
+	FAM231B	1	16503724	16550035	47	7	10164	0.6159	0.26898
-	CCDC14	3	123877353	123996409	315	17	10164	0.61552	0.26911
+	HK2	2	74797655	74903354	57	9	10164	0.61535	0.26916
-	CDR2	16	22335936	22409617	69	4	10164	0.61533	0.26917
-	GBA2	9	35726866	35784228	93	26	10164	0.61527	0.26919
+	SP5	2	170679738	170727988	151	23	10164	0.61501	0.26927
-	STK33	11	8381866	8629289	696	23	10164	0.61487	0.26932
-	EIF6	20	35268906	35319816	114	17	10164	0.61448	0.26945
+	ANKRD33B	5	10529323	10667816	284	23	10164	0.61405	0.26959
-	TERF2	16	69345561	69421000	155	35	10164	0.61382	0.26967
+	EN2	7	155423129	155474831	87	14	10164	0.61356	0.26975
+	ANKRD12	18	9101753	9295985	407	19	10164	0.61299	0.26994
+	PRRC2C	1	171450513	171603511	483	35	10164	0.61266	0.27005
+	LOC1053743	3	125721192	125779389	370	11	10164	0.61236	0.27015
-	MAP3K2	2	127288669	127378229	150	17	10164	0.61227	0.27018
-	THEMIS	6	127698194	127953631	460	29	10164	0.61213	0.27023
+	PPAN-P2RY1	19	10071223	10125389	86	18	10164	0.61205	0.27025
-	RAPSN	11	47427757	47484178	116	15	10164	0.61169	0.27037
-	LCN6	9	136734017	136783528	183	10	10164	0.61141	0.27047
-	PTRF	17	42392449	42458320	203	24	10164	0.61136	0.27048
+	EXTL3	8	28666473	28763690	86	7	10164	0.61134	0.27049
-	CA12	15	63313531	63417110	330	47	10164	0.6113	0.2705
+	ARID4A	14	58263385	58383995	176	15	10164	0.61122	0.27053
+	TIAM2	6	155055289	155267723	634	55	10164	0.61105	0.27058
+	OS9	12	57658955	57731557	225	18	10164	0.61091	0.27063
-	AK2	1	32997940	33071911	100	8	10164	0.61088	0.27064
+	SLC12A9	7	100799036	100877012	199	28	10164	0.61042	0.27079
+	ZNF528	19	52362868	52428408	194	30	10164	0.6103	0.27083
+	DUS2	16	67987944	68089323	132	10	10164	0.60984	0.27098
+	PPCDC	15	74988555	75060726	227	23	10164	0.60986	0.27098
+	PRDM2	1	13665240	13835079	533	42	10164	0.60974	0.27102
-	DNTTIP2	1	93859458	93914206	18	1	10164	0.60965	0.27105
-	AGER	6	32170968	32219322	269	62	10164	0.60959	0.27107
-	ZSWIM2	2	186817480	186884170	286	24	10164	0.60894	0.27128

STRAND	SYMBOL	CHR	START	STOP	NSNPS	NPARAM	N	ZSTAT	P
+	LMCD1	3	8466807	8578125	340	24	10164	0.6089	0.27129
-	NKAIN4	20	63230784	63289540	206	22	10164	0.60881	0.27133
-	YWHAZ	8	100908576	100989596	155	14	10164	0.60844	0.27145
-	LOC1019278	10	3042510	3104273	71	10	10164	0.60841	0.27146
+	NUBP2	16	1747923	1799191	228	27	10164	0.60816	0.27154
+	KDM3B	5	138317596	138447027	259	18	10164	0.60815	0.27154
-	EZR	6	158755741	158854424	381	20	10164	0.60797	0.2716
+	ARHGEF25	12	57575180	57627245	118	21	10164	0.60762	0.27172
+	IL31RA	5	55805334	55932854	265	27	10164	0.60743	0.27178
-	ZNF160	19	53054882	53138471	310	26	10164	0.60712	0.27189
-	LMNTD2	11	544850	595780	204	28	10164	0.60616	0.27221
-	MS4A4E	11	60191253	60278114	240	19	10164	0.6058	0.27232
-	AMFR	16	56351452	56460538	307	21	10164	0.60577	0.27233
+	LOC1053761	9	92586621	92641713	117	14	10164	0.60563	0.27238
+	GNGT1	7	93871508	93921173	102	6	10164	0.60548	0.27243
-	ANKRD17	4	73064785	73294142	246	43	10164	0.60538	0.27246
+	GOLT1B	12	21466765	21528403	207	26	10164	0.60535	0.27247
-	SLC35F4	14	57553922	58017274	1786	90	10164	0.60537	0.27247
-	GOLGA6L10	15	82329993	82384475	47	12	10164	0.60529	0.27249
+	TTBK1	6	43208484	43298259	212	28	10164	0.60522	0.27252
-	ALX4	11	44250438	44354220	301	49	10164	0.60515	0.27254
-	FRRS1L	9	109126188	109202291	212	31	10164	0.60512	0.27255
+	PCSK2	20	17191107	17494578	1155	89	10164	0.60493	0.27261
+	SLC39A12	10	17916839	18053292	621	45	10164	0.6049	0.27262
+	ASXL1	20	32323344	32449319	219	14	10164	0.6048	0.27266
-	C17orf98	17	38815392	38876500	176	21	10164	0.60477	0.27267
-	RPS6	9	19366256	19415237	88	11	10164	0.60465	0.2727
+	FOLR2	11	72181775	72231950	153	27	10164	0.60453	0.27275
+	LEP	7	128206202	128267629	156	21	10164	0.60451	0.27275
+	ZNF638	2	71296755	71445061	427	21	10164	0.60421	0.27285
-	PLA2G4C	19	48037843	48145853	444	47	10164	0.60414	0.27288
-	ZFP30	19	37622487	37692285	185	23	10164	0.60396	0.27293
-	ZNF248	10	37734363	37895851	290	23	10164	0.60396	0.27293
-	ZSCAN18	19	58073834	58153433	263	14	10164	0.60355	0.27307
+	ADPRM	17	10662610	10721558	149	11	10164	0.60349	0.27309
+	MROH7	1	54606740	54720267	367	42	10164	0.60349	0.27309
+	C8orf74	8	10637592	10710613	312	33	10164	0.60342	0.27312
+	PPP2R2D	10	131865644	131979980	237	19	10164	0.60309	0.27322
+	SLC22A4	5	132259384	132354206	391	32	10164	0.60244	0.27344
-	NUDT21	16	56419136	56486349	183	24	10164	0.60243	0.27345
+	SPEM1	17	7385360	7431632	107	31	10164	0.60216	0.27354
+	OR7E24	19	9216044	9262063	109	18	10164	0.6021	0.27355
+	AARD	8	116903225	116954000	175	19	10164	0.60198	0.27359
-	ADAP1	7	887900	990407	364	40	10164	0.60164	0.27371
+	MARK1	1	220493183	220674457	231	18	10164	0.60143	0.27378
-	DDX28	16	68011274	68058867	54	4	10164	0.60099	0.27392
+	NLK	17	28007647	28215140	193	56	10164	0.60064	0.27404
-	MKL1	22	40400281	40671719	552	23	10164	0.60051	0.27408
+	TBC1D12	10	94367429	94546332	406	23	10164	0.60035	0.27414
+	SSTR1	14	38172999	38223063	153	16	10164	0.60032	0.27415
+	ZFYVE19	15	40772076	40824569	166	25	10164	0.59999	0.27426
+	RFX6	6	116842213	116942163	376	25	10164	0.59976	0.27433
-	TMEM119	12	108579846	108633118	160	28	10164	0.59943	0.27444
+	ZNF554	19	2784874	2846735	120	12	10164	0.5994	0.27445
+	EXOG	3	38461127	38536305	174	17	10164	0.59928	0.27449
-	TCF3	19	1599290	1687426	83	12	10164	0.59923	0.27451
+	GINS3	16	58357394	58416144	155	21	10164	0.5988	0.27465
+	LEAP2	5	132838666	132884890	122	15	10164	0.59866	0.2747
+	OGFOD1	16	56416417	56487495	196	26	10164	0.59853	0.27474
+	FAM81A	15	59367276	59533552	488	55	10164	0.59845	0.27477
-	STK3	8	98361355	98977571	708	39	10164	0.59839	0.27479
+	FTL	19	48930309	48976879	132	20	10164	0.59832	0.27481
-	DNAJC27	2	24933636	25007094	137	8	10164	0.59824	0.27484

STRAND	SYMBOL	CHR	START	STOP	NSNPS	NPARAM	N	ZSTAT	P
+	FAM8A1	6	17565287	17621719	141	20	10164	0.59823	0.27484
+	RTEL1	20	63622810	63706253	336	36	10164	0.59807	0.2749
+	RNF182	6	13889446	13990009	205	17	10164	0.59791	0.27495
+	SLC1A3	5	36571355	36698334	356	51	10164	0.59784	0.27497
-	EXOSC1	10	97425909	97481011	87	15	10164	0.59768	0.27503
-	DDIT3	12	57506588	57556698	84	12	10164	0.59756	0.27507
-	KIAA1683	19	18247096	18309509	229	25	10164	0.59751	0.27508
+	VPS13D	1	12195039	12522047	567	25	10164	0.59746	0.2751
-	OTUD7B	1	149930330	150045772	82	13	10164	0.59746	0.2751
-	FAM178B	2	96865882	97021601	536	41	10164	0.59745	0.27511
-	COL5A3	19	9949561	10045538	106	16	10164	0.59741	0.27512
+	TECR	19	14492768	14575980	216	35	10164	0.597	0.27525
+	REM1	20	31440288	31494905	67	9	10164	0.59697	0.27526
+	SOX17	8	54422935	54470896	108	16	10164	0.59693	0.27528
+	PRAMEF15	1	13280581	13332595	59	3	10164	0.59668	0.27536
-	ZCCHC14	16	87396246	87527269	293	29	10164	0.5966	0.27539
-	HLA-C	6	31258749	31307136	1682	46	10164	0.59657	0.2754
-	NAT8	2	73630723	73677410	1	1	10164	0.59569	0.27569
-	CDC37	19	10381131	10438595	150	18	10164	0.5956	0.27572
+	OR10G9	11	123988013	124033948	238	20	10164	0.59547	0.27576
-	FGL2	7	77183371	77234833	137	18	10164	0.5954	0.27579
+	TPBGL	11	75205905	75253704	76	12	10164	0.5954	0.27579
+	TDRD6	6	46652875	46714319	75	13	10164	0.59528	0.27583
+	SGIP1	1	66498311	66758999	902	34	10164	0.59514	0.27588
+	KLHDC8B	3	49136120	49186486	102	11	10164	0.5951	0.27589
+	HIST1H3E	6	26190155	26235616	262	22	10164	0.59495	0.27594
-	NAP1L4	11	2934430	3027377	265	16	10164	0.59481	0.27599
+	SERPINB2	18	63852705	63913890	214	11	10164	0.59463	0.27604
+	LOC1009966	6	109182301	109295010	433	17	10164	0.59465	0.27604
+	ZMYM2	13	19922432	20101845	215	14	10164	0.59463	0.27605
-	PBX2	6	32174733	32225186	311	58	10164	0.59458	0.27606
-	UNC93B1	11	67981100	68039125	181	27	10164	0.59404	0.27624
-	TM2D2	8	38978808	39031816	99	8	10164	0.59384	0.27631
+	ZNF879	5	178988732	179044387	200	14	10164	0.59376	0.27633
-	ECHS1	10	133352483	133408404	73	9	10164	0.59303	0.27658
+	DMRTB1	1	53424399	53477488	190	21	10164	0.59295	0.27661
-	ARAP2	4	36055998	36279357	511	54	10164	0.59272	0.27668
-	TMEM26	10	61396642	61488450	276	24	10164	0.59268	0.2767
-	CSNK1A1	5	149485894	149586552	230	25	10164	0.59259	0.27673
-	CTAGE6	7	143745089	143792750	3	2	10164	0.59254	0.27674
-	ANKRD28	3	15657236	15894555	678	25	10164	0.59247	0.27677
+	PTTG2	4	37925435	37971010	146	22	10164	0.592	0.27692
+	CABP4	11	67417415	67471774	106	20	10164	0.59195	0.27694
-	PNPLA7	9	137449472	137585895	155	28	10164	0.59195	0.27694
+	COL4A3	2	227129565	227324792	931	81	10164	0.59188	0.27696
+	DIO3	14	101526351	101573452	85	18	10164	0.59173	0.27702
+	LRP10	14	22836613	22891580	123	27	10164	0.59152	0.27708
-	LOC1053691	6	9835518	10194438	1278	83	10164	0.59151	0.27709
-	ALG9	11	111772195	111906581	281	23	10164	0.59136	0.27714
+	POLR3E	16	22262366	22345103	108	5	10164	0.59122	0.27719
+	PPP1R37	19	45058173	45157285	220	26	10164	0.59116	0.27721
-	AZGP1	7	99956727	100011112	114	17	10164	0.591	0.27726
+	TPP2	13	102561936	102689174	488	29	10164	0.59074	0.27735
-	APBB1	11	6385124	6454472	108	23	10164	0.59056	0.27741
-	ZNF812	19	9678855	9735817	255	13	10164	0.59017	0.27754
-	HRSP12	8	98092339	98152190	100	9	10164	0.59012	0.27755
+	ZSCAN1	19	57998989	58068907	146	18	10164	0.59001	0.27759
-	PPFIA2	12	81247975	81794548	1352	78	10164	0.58959	0.27773
-	TPRX1	19	47791243	47838604	197	24	10164	0.58929	0.27783
+	FOXJ2	12	7997708	8065522	36	3	10164	0.58927	0.27784
-	IER3	6	30733199	30779550	331	36	10164	0.58888	0.27797
+	TUBB4B	9	137206259	137253707	129	18	10164	0.58887	0.27798
+	SLC26A10	12	57584910	57636151	121	23	10164	0.58886	0.27798

STRAND	SYMBOL	CHR	START	STOP	NSNPS	NPARAM	N	ZSTAT	P
-	TPM2	9	35671993	35725056	119	25	10164	0.58881	0.27799
-	ZNF629	16	30768449	30822277	30	2	10164	0.58879	0.278
-	TAP2	6	32811833	32873823	620	67	10164	0.58868	0.27804
-	VIPAS39	14	77416675	77492640	231	18	10164	0.58833	0.27815
-	GGA3	17	75215480	75297393	247	12	10164	0.58818	0.27821
+	GCC2	2	108414121	108519398	281	11	10164	0.58789	0.2783
-	UBE2T	1	202321657	202376966	111	14	10164	0.58791	0.2783
+	XRCC5	2	216074297	216216293	347	30	10164	0.5878	0.27833
+	SPIDR	8	47225878	47746001	592	39	10164	0.58779	0.27834
+	TALDO1	11	712432	775024	167	25	10164	0.58768	0.27838
+	MRPL30	2	99146079	99209557	171	18	10164	0.58752	0.27843
-	FCHSD2	11	72826743	73177454	302	29	10164	0.58745	0.27845
+	ABHD17C	15	80660311	80765621	335	36	10164	0.58746	0.27845
-	BOD1L1	4	13558738	13662705	196	15	10164	0.58745	0.27845
+	ENO4	10	116814212	116953974	141	24	10164	0.58739	0.27847
-	EIF2AK1	7	6012247	6094229	315	22	10164	0.58737	0.27848
-	XIRP1	3	39173215	39227620	127	28	10164	0.58725	0.27852
-	NAPRT	8	143564664	143614626	225	27	10164	0.58705	0.27859
+	OR2L2	1	248000510	248049332	74	5	10164	0.58682	0.27866
-	LOC1053714	17	391242	452165	133	22	10164	0.58547	0.27911
-	SGCE	7	94575224	94691209	241	36	10164	0.58547	0.27912
-	BNC2	9	16399503	16905838	1715	129	10164	0.58543	0.27913
+	LMLN	3	197925200	198053720	230	20	10164	0.58522	0.2792
+	HERPUD1	16	56896765	56953881	254	33	10164	0.58509	0.27924
-	FRMD4A	10	13633706	14365867	3179	237	10164	0.58496	0.27929
+	SERPINB8	18	63935028	64016821	169	19	10164	0.58478	0.27935
+	HLA-E	6	30454406	30504205	372	43	10164	0.5846	0.27941
-	CHRM4	11	46374792	46421608	39	12	10164	0.58431	0.27951
+	CHST14	15	40435961	40483158	101	26	10164	0.58427	0.27952
-	NAT9	17	74760547	74811395	126	14	10164	0.58415	0.27956
+	NTMT1	9	129573884	129646131	109	19	10164	0.58416	0.27956
-	C3orf67	3	58724472	59085078	395	20	10164	0.5841	0.27958
+	PTPN4	2	119724631	119994899	321	18	10164	0.5839	0.27964
+	TM4SF4	3	149439581	149513394	422	31	10164	0.58376	0.27969
+	PPP1R7	2	241114573	241193650	376	32	10164	0.58349	0.27978
+	METTL14	4	118650364	118722270	125	14	10164	0.58349	0.27978
-	SPDYE18	7	77041689	77095538	37	10	10164	0.58325	0.27986
-	GRIK1	21	29526933	29974964	1129	80	10164	0.58313	0.2799
+	CNNM4	2	96725902	96821891	184	22	10164	0.58308	0.27992
+	WNK4	17	42744673	42807066	106	11	10164	0.58296	0.27996
-	GHDC	17	42179087	42229532	68	13	10164	0.58288	0.27999
-	C22orf31	22	29048672	29108966	275	33	10164	0.5828	0.28001
+	RMI1	9	83945720	84014074	138	17	10164	0.58268	0.28005
-	NUTM2F	9	94308196	94589847	868	34	10164	0.58239	0.28015
-	LOC1053724	19	58009302	58060640	97	13	10164	0.58215	0.28023
+	LOC1053711	16	22347084	22417457	61	4	10164	0.5821	0.28025
+	GNAL	18	11654015	11895685	376	23	10164	0.58207	0.28026
-	RFC2	7	74221485	74289458	42	2	10164	0.58194	0.2803
+	OR1L3	9	122640130	122686104	89	12	10164	0.58185	0.28033
-	DUOXA1	15	45107366	45164938	121	21	10164	0.58153	0.28044
+	DRAM1	12	101842327	101933623	414	28	10164	0.58149	0.28046
+	ADGRB1	8	142414888	142555008	421	56	10164	0.58137	0.2805
-	METTL10	10	124747831	124826941	223	42	10164	0.58131	0.28052
-	CCDC38	12	95857048	95978254	502	34	10164	0.58067	0.28073
+	C2orf27A	2	131687491	131777404	138	7	10164	0.58056	0.28077
-	TMPRSS6	22	37055436	37145635	217	32	10164	0.58056	0.28077
+	PRRG4	11	32794774	32868123	113	15	10164	0.58014	0.28091
+	PPP1R12B	1	202313702	202598569	509	25	10164	0.58006	0.28094
+	RRM1	11	4059561	4148881	291	18	10164	0.57993	0.28098
+	FAM24A	10	122875701	122923111	122	13	10164	0.57978	0.28103
+	KRTAP20-3	21	30607864	30653136	86	8	10164	0.57974	0.28104
-	TIA1	2	70199444	70283707	215	16	10164	0.57962	0.28108
-	MAML2	11	95966204	96378245	1383	172	10164	0.57929	0.2812

STRAND	SYMBOL	CHR	START	STOP	NSNPS	NPARAM	N	ZSTAT	P
-	UBXN7	3	196343490	196489318	251	25	10164	0.57916	0.28124
-	HIST1H2AD	6	26188784	26234293	262	22	10164	0.57889	0.28133
-	IL2RB	22	37115838	37184922	153	32	10164	0.57861	0.28142
+	RNF125	18	31983223	32098528	240	35	10164	0.57859	0.28143
-	GBP1	1	89042304	89100360	174	16	10164	0.57856	0.28144
+	SYNM	15	99070080	99145595	162	16	10164	0.57814	0.28158
-	LOC400736	1	13186188	13236409	1	1	10164	0.57814	0.28158
-	MBOAT1	6	20089684	20247464	366	55	10164	0.57786	0.28168
-	GRID1	10	85589555	86401493	2375	164	10164	0.57776	0.28171
+	GLI4	8	143232437	143286931	158	8	10164	0.57754	0.28179
+	TNFRSF12A	16	2985312	3032383	24	3	10164	0.57736	0.28185
+	LOC1053735	2	110281888	110357069	1	1	10164	0.57729	0.28187
+	LOC1001343	17	73702854	73766831	252	32	10164	0.5772	0.2819
-	OVCH2	11	7668692	7741454	306	32	10164	0.57674	0.28206
-	IDI1	10	1030023	1091699	211	30	10164	0.57619	0.28224
+	TBX21	17	47698244	47756122	124	22	10164	0.57597	0.28232
-	TTF1	9	132365548	132441871	105	22	10164	0.57587	0.28235
-	PTK6	20	63518413	63572370	218	43	10164	0.57584	0.28236
+	PBX3	9	125712338	125977377	612	11	10164	0.57577	0.28238
+	NOG	17	56558699	56605590	69	6	10164	0.57572	0.2824
+	RNF34	12	121365083	121434352	240	23	10164	0.5755	0.28248
-	SLC35F2	11	107780991	107894188	436	24	10164	0.57485	0.2827
-	ZNF829	19	36878124	36951387	253	22	10164	0.57477	0.28272
+	LOC1053788	1	87184918	87271689	194	14	10164	0.57469	0.28275
-	CORO2A	9	98110975	98227674	422	52	10164	0.57391	0.28302
+	DDX1	2	15556621	15641111	285	30	10164	0.57382	0.28304
+	MIA2	14	39198921	39263371	190	22	10164	0.57382	0.28305
-	VSTM4	10	49004245	49164144	639	26	10164	0.57341	0.28318
-	CHID1	11	857859	950058	210	17	10164	0.57307	0.2833
-	TMCO4	1	19672213	19835353	557	69	10164	0.57308	0.2833
-	WNT7B	22	45910362	46012128	122	9	10164	0.57298	0.28333
+	ORAI1	12	121591550	121652040	161	18	10164	0.57292	0.28335
-	DYNLT1	6	158626474	158679786	146	18	10164	0.57281	0.28339
-	ZRANB2	1	71053291	71116289	100	18	10164	0.57278	0.2834
+	PQLC2L	3	157508344	157687750	590	40	10164	0.57277	0.2834
+	ZNF205	16	3077562	3130527	88	8	10164	0.57264	0.28344
-	POLR2J2	7	102626748	102706729	28	6	10164	0.57256	0.28347
-	MAP4	3	47840690	48124279	369	22	10164	0.57241	0.28352
+	SKI	1	2193695	2320213	393	56	10164	0.57232	0.28355
-	MYH11	16	15693135	15892033	747	51	10164	0.57216	0.28361
+	PGAM1	10	97391270	97443441	124	12	10164	0.57207	0.28364
-	COPRS	17	31841865	31894307	63	3	10164	0.57205	0.28364
+	TM6SF1	15	83072475	83148015	128	20	10164	0.57188	0.2837
-	COPB1	11	14447503	14534895	143	12	10164	0.5718	0.28373
-	STRN4	19	46709509	46781475	196	25	10164	0.57166	0.28377
+	BAHCC1	17	81360464	81476332	196	36	10164	0.57155	0.28381
-	PURB	7	44866293	44920385	101	15	10164	0.57149	0.28383
+	S1PR3	9	88956409	89015155	161	19	10164	0.57142	0.28386
+	KIF19	17	74291212	74365820	245	39	10164	0.5711	0.28397
+	IPO5	13	97918638	98034296	200	25	10164	0.57078	0.28407
+	OR2G6	1	248486647	248532597	20	1	10164	0.57079	0.28407
-	SMG9	19	43717992	43789990	164	22	10164	0.57059	0.28414
-	EHD4	15	41889440	42007557	417	57	10164	0.57051	0.28416
-	ZSCAN10	16	3078890	3134317	94	8	10164	0.57032	0.28423
-	LOC1053751	6	30454493	30502855	361	40	10164	0.57007	0.28432
+	MYO9B	19	17040779	17225277	899	75	10164	0.56935	0.28456
+	DENND4C	9	19195765	19384268	368	17	10164	0.56916	0.28462
-	PARP4	13	24410930	24547993	735	28	10164	0.56894	0.2847
-	ZWINT	10	56347438	56396302	77	14	10164	0.56865	0.2848
+	SPINT4	20	45687349	45735696	211	20	10164	0.56861	0.28481
-	GLUL	1	182368093	182427206	143	10	10164	0.5685	0.28485
+	HACD3	15	65495489	65588355	213	15	10164	0.5685	0.28485
-	CDNF	10	14809245	14873098	151	27	10164	0.56846	0.28486

STRAND	SYMBOL	CHR	START	STOP	NSNPS	NPARAM	N	ZSTAT	P
-	SPI1	11	47344858	47430640	213	17	10164	0.56836	0.2849
-	KRT16	17	41599778	41647827	39	4	10164	0.56827	0.28492
-	COMMD9	11	36262292	36324449	222	32	10164	0.56799	0.28502
-	ZNF786	7	149059641	149125777	120	6	10164	0.56797	0.28503
-	TEP1	14	20355667	20449645	352	67	10164	0.5676	0.28515
+	TMEM265	16	30705191	30755196	11	2	10164	0.56755	0.28517
+	MDM2	12	68773168	68855544	148	20	10164	0.56747	0.2852
-	CFAP74	1	1911951	2038837	415	49	10164	0.56741	0.28522
-	TRAPPC1	17	7920345	7966999	50	11	10164	0.56726	0.28527
+	MAST1	19	12798934	12884953	100	7	10164	0.56713	0.28531
+	SLC5A2	16	31447535	31500770	42	9	10164	0.56692	0.28538
-	SARDH	9	133651740	133774955	223	26	10164	0.56676	0.28544
+	VSTM2A	7	54507325	54581080	178	11	10164	0.56645	0.28554
-	DUXA	19	57141726	57202488	215	26	10164	0.56646	0.28554
-	BEND3	6	107055180	107150182	165	28	10164	0.5656	0.28583
-	PIGR	1	206918522	206981466	136	15	10164	0.56525	0.28595
+	CCDC116	22	21597147	21647327	83	9	10164	0.5651	0.286
+	FAM110B	8	57959509	58159718	490	53	10164	0.5649	0.28607
-	LOC1019298	10	131874018	131924796	117	16	10164	0.56486	0.28608
+	ZMYM1	1	35024786	35125859	148	11	10164	0.56483	0.28609
+	MRPS30	5	44773925	44825514	146	15	10164	0.56482	0.2861
+	NSG1	4	4351256	4429058	260	36	10164	0.56479	0.28611
+	ANKRD36	2	97078186	97274521	284	24	10164	0.56477	0.28612
-	ZNF425	7	149092784	149161346	156	7	10164	0.56461	0.28617
+	LOC1053703	13	110778203	110830209	129	25	10164	0.56455	0.28619
-	ACOT8	20	45831721	45892409	224	27	10164	0.56419	0.28631
-	SERPINI2	3	167431789	167509132	182	14	10164	0.56401	0.28637
+	TRMT13	1	100098147	100160498	76	12	10164	0.56393	0.2864
+	MEIG1	10	14919228	14982857	285	23	10164	0.56379	0.28645
-	NEK4	3	52700780	52805949	240	20	10164	0.56368	0.28648
-	UBE3A	15	25327234	25474043	125	7	10164	0.56369	0.28648
-	TMBIM4	12	66126936	66205072	207	18	10164	0.56368	0.28648
+	CYLD	16	50707050	50811936	204	20	10164	0.56313	0.28667
+	ZNF398	7	149091416	149193042	225	9	10164	0.56303	0.28671
+	PPP1R17	7	31652017	31719911	20	3	10164	0.56297	0.28673
+	LOC1027249	1	16500773	16546956	55	8	10164	0.56244	0.28691
-	BNIP3	10	131957683	132017013	175	18	10164	0.56194	0.28708
+	TUBA8	22	18075687	18141731	17	3	10164	0.56171	0.28716
-	TSPAN5	4	98460367	98693661	621	53	10164	0.56165	0.28718
-	FBXO5	6	152960519	153018605	212	22	10164	0.56151	0.28723
-	RBMS1	2	160262151	160528856	628	34	10164	0.56143	0.28725
+	C11orf30	11	76409928	76562899	380	27	10164	0.56143	0.28725
-	MYH6	14	23371990	23443277	169	26	10164	0.56132	0.28729
-	VGLL3	3	86927973	87026287	390	32	10164	0.56128	0.2873
+	COMMD4	15	75300996	75351927	162	22	10164	0.56124	0.28732
+	TBC1D30	12	64789572	64891022	90	13	10164	0.56121	0.28733
+	LHPP	10	124426772	124624141	1014	95	10164	0.56113	0.28735
+	ANKRD32	5	94580858	94724918	363	17	10164	0.56115	0.28735
-	VSTM5	11	93810569	93885502	282	24	10164	0.56066	0.28751
+	UQCRQ	5	132831627	132878844	116	15	10164	0.56051	0.28757
-	AVIL	12	57787372	57854824	133	19	10164	0.56042	0.2876
-	SLC1A6	19	14940033	15045643	578	51	10164	0.56039	0.28761
+	ALPK3	15	84781680	84883482	291	40	10164	0.56039	0.28761
-	LGALS9B	17	20439395	20502535	28	4	10164	0.56039	0.28761
+	LOC1019278	1	87177669	87261331	162	14	10164	0.56004	0.28772
-	BBS7	4	121814329	121905497	192	27	10164	0.55993	0.28776
-	OR6C6	12	55284288	55330232	131	21	10164	0.55974	0.28783
-	HELZ2	20	63548086	63609239	226	39	10164	0.55968	0.28785
+	HNRNPUL1	19	41227476	41317693	169	13	10164	0.55953	0.2879
-	SYNE4	19	35993000	36043793	80	15	10164	0.55954	0.2879
+	PHF20L1	8	132740358	132858807	286	18	10164	0.55934	0.28796
+	GNAT1	3	50156610	50207696	106	16	10164	0.55913	0.28804
-	SMC1B	22	45334063	45448629	376	18	10164	0.5591	0.28805

STRAND	SYMBOL	CHR	START	STOP	NSNPS	NPARAM	N	ZSTAT	P
+	SLCO1B7	12	20980696	21100106	579	24	10164	0.55902	0.28807
-	ZNF782	9	96806699	97048673	411	14	10164	0.55896	0.2881
+	CCDC185	1	223358373	223405470	1	1	10164	0.55886	0.28813
+	RGS20	8	53816808	53969304	265	32	10164	0.55869	0.28819
+	NUDT2	9	34294506	34353713	138	14	10164	0.55861	0.28821
+	CCDC60	12	119299712	119551047	613	70	10164	0.55848	0.28826
-	DEK	6	18214169	18299568	154	10	10164	0.55771	0.28852
-	FCER2	19	7678757	7737310	14	7	10164	0.55765	0.28854
-	SLC2A14	12	7802512	7926196	533	64	10164	0.55764	0.28855
+	CCDC157	22	30321638	30388383	218	20	10164	0.55757	0.28857
+	PLCB3	11	64216523	64279452	194	38	10164	0.55747	0.2886
-	B4GALT5	20	49622945	49748884	368	21	10164	0.55715	0.28871
-	S100A8	1	153380032	153457578	147	7	10164	0.55668	0.28887
-	TTC34	1	2626988	2824665	168	19	10164	0.55666	0.28888
-	SLC39A1	1	153949099	154003184	56	10	10164	0.55656	0.28891
+	RSBN1L	7	77661426	77789803	315	16	10164	0.55617	0.28905
+	CREB5	7	28264321	28835894	2084	223	10164	0.55578	0.28918
-	MYO1C	17	1454186	1527707	202	27	10164	0.55521	0.28938
-	FOXM1	12	2847681	2912155	190	9	10164	0.55505	0.28943
-	KRTDAP	19	35477324	35525531	182	38	10164	0.55473	0.28954
+	C10orf107	10	61627952	61776766	438	25	10164	0.55436	0.28967
+	L3MBTL1	20	43472680	43551895	156	24	10164	0.55376	0.28987
+	ZSWIM3	20	45822581	45889131	245	29	10164	0.55364	0.28991
+	NCKAP1L	12	54462711	54553115	205	30	10164	0.55357	0.28994
+	SNX2	5	122739996	122844539	304	16	10164	0.5535	0.28996
-	BET1	7	93952770	94039390	108	16	10164	0.55332	0.29002
-	LOXL4	10	98237686	98303250	83	13	10164	0.55316	0.29008
+	KLHL42	12	27744958	27813040	244	27	10164	0.55313	0.29009
+	EFR3A	8	131869109	132023642	396	20	10164	0.55302	0.29013
+	SLC38A3	3	50170246	50230979	111	18	10164	0.55233	0.29036
+	MAP1LC3A	20	34511830	34570345	100	18	10164	0.55214	0.29043
+	HIATL1	9	94339322	94470920	286	17	10164	0.55213	0.29043
-	JTB	1	153964269	154012975	56	12	10164	0.55209	0.29044
-	FKBP11	12	48911959	48960547	113	4	10164	0.55107	0.29079
-	KIFAP3	1	169911329	170109741	516	40	10164	0.55106	0.2908
-	KCTD18	2	200478952	200545094	176	37	10164	0.55089	0.29086
+	CFB	6	31910944	31962084	236	62	10164	0.5508	0.29089
-	POLR1A	2	86015168	86141155	325	14	10164	0.55079	0.29089
+	LYPD6B	2	149003467	149225262	679	34	10164	0.55065	0.29094
-	SLC26A9	1	205903048	205978472	195	30	10164	0.55056	0.29097
+	UBA52	19	18528766	18587460	88	7	10164	0.55018	0.2911
-	GLYAT	11	58698757	58766974	308	17	10164	0.55002	0.29115
-	PDZD7	10	100997683	101066157	151	38	10164	0.55002	0.29115
-	ACHE	7	100879994	100931686	165	26	10164	0.54998	0.29117
+	GGT1	22	24548750	24639005	166	21	10164	0.54976	0.29124
-	LOC1053782	12	51829120	51881959	72	13	10164	0.54942	0.29136
-	NCAPG2	7	158621169	158739831	359	14	10164	0.54938	0.29137
-	HIST1H3D	6	26186784	26234293	273	23	10164	0.54925	0.29142
-	DHRS12	13	51757993	51839199	162	29	10164	0.54919	0.29144
-	AKNAD1	1	108805898	108893242	263	37	10164	0.54915	0.29145
+	LOC1053752	7	27079130	27126665	86	21	10164	0.54897	0.29151
-	PPIC	5	123013383	123071730	181	26	10164	0.54872	0.2916
+	OR6C1	12	55285600	55331538	131	20	10164	0.54867	0.29162
+	C2orf15	2	99106722	99161487	156	14	10164	0.54853	0.29166
+	IL4	5	132638986	132692678	140	28	10164	0.54838	0.29172
-	LIN7C	11	27484418	27541779	120	20	10164	0.54838	0.29172
-	TBC1D10A	22	30281990	30361966	205	27	10164	0.54824	0.29176
+	SOCS7	17	38317124	38415593	126	12	10164	0.54802	0.29184
+	METTL11B	1	170111047	170178923	229	23	10164	0.5477	0.29195
-	SMIM2	13	44133151	44196257	159	27	10164	0.54759	0.29199
+	RGCC	13	41422406	41480877	194	33	10164	0.54746	0.29203
+	GPR65	14	87970124	88024811	317	23	10164	0.54743	0.29204
+	PRR29	17	63963344	64014305	56	12	10164	0.54735	0.29207

STRAND	SYMBOL	CHR	START	STOP	NSNPS	NPARAM	N	ZSTAT	P
-	C14orf2	14	103902288	103956566	217	15	10164	0.54703	0.29218
-	LOC1019290	19	291437	339505	52	2	10164	0.54704	0.29218
-	GTPBP2	6	43610481	43664246	116	13	10164	0.54699	0.29219
+	SOX8	16	946808	996979	188	10	10164	0.54692	0.29222
+	BCL9	1	147506401	147636219	276	39	10164	0.5464	0.29239
+	IL27RA	19	13996740	14062919	119	13	10164	0.54629	0.29243
-	FAM78A	9	131248078	131316920	115	11	10164	0.54601	0.29253
+	MRC2	17	62591515	62703601	159	20	10164	0.5456	0.29267
-	TFPI	2	187454230	187589494	365	34	10164	0.54547	0.29271
-	PANK3	5	168545623	168614609	133	19	10164	0.54517	0.29282
+	RPL14	3	40422292	40472372	135	10	10164	0.54514	0.29283
-	SOCS3	17	78346777	78395079	144	18	10164	0.54484	0.29293
-	AKAP3	12	4605508	4684047	264	26	10164	0.54482	0.29294
+	TPD52L3	9	6293349	6341900	55	11	10164	0.54481	0.29294
+	DAGLA	11	61645433	61757002	202	26	10164	0.54476	0.29296
+	GNPTG	16	1316899	1373351	97	20	10164	0.54477	0.29296
+	ARNT2	15	80369350	80607937	776	83	10164	0.54418	0.29316
-	SCGB3A1	5	180580105	180626487	178	27	10164	0.54397	0.29323
+	ZWILCH	15	66470083	66559485	254	29	10164	0.54338	0.29344
-	BTC	4	74734759	74829672	334	22	10164	0.54318	0.2935
+	LOC1053740	3	101652198	101719759	145	20	10164	0.54293	0.29359
+	LOC1053705	14	70309946	70357628	146	14	10164	0.54285	0.29362
-	TWISTNB	7	19685462	19744037	221	23	10164	0.54243	0.29376
+	NDUFS8	11	67995617	68046647	162	26	10164	0.54217	0.29385
-	ZDHHC3	3	44905261	45011182	202	16	10164	0.54209	0.29388
-	RRP12	10	97346701	97436370	254	12	10164	0.54202	0.2939
-	XXYLT1	3	195058276	195306166	753	37	10164	0.54203	0.2939
-	TPGS2	18	36770024	36864393	63	16	10164	0.54187	0.29395
-	RTN4IP1	6	106561028	106665500	459	27	10164	0.54179	0.29398
+	CCL20	2	227778842	227827564	94	17	10164	0.54164	0.29403
+	FXR1	3	180877446	180992753	191	16	10164	0.54159	0.29405
+	PRRC1	5	127482609	127565089	187	12	10164	0.54129	0.29415
-	NKX3-2	4	13530830	13579490	77	13	10164	0.54102	0.29425
+	ASIC3	7	151013292	151062756	131	31	10164	0.54097	0.29426
-	SLC19A3	2	227675210	227753029	185	26	10164	0.54091	0.29429
-	LOC1053761	9	94351251	94409815	112	10	10164	0.54087	0.2943
-	KLK7	19	50966479	51019064	249	37	10164	0.54073	0.29435
+	RBM48	7	92493692	92549405	127	18	10164	0.54036	0.29447
+	NAALADL2	3	174405995	175819510	3612	192	10164	0.54035	0.29448
-	ACTR8	3	53854931	53917202	161	14	10164	0.54028	0.2945
+	ZDHHC23	3	113912901	114002167	144	32	10164	0.54027	0.29451
-	SF3A1	22	30321988	30391947	226	21	10164	0.54001	0.29459
-	ZIM3	19	57124096	57180202	234	26	10164	0.53969	0.2947
+	HOXC11	12	53938126	53986419	107	17	10164	0.53932	0.29483
-	ILDR2	1	166903204	167024093	152	11	10164	0.53897	0.29495
-	HIST1H4D	6	26178710	26224076	276	25	10164	0.53892	0.29497
+	HAMP	19	35247507	35295143	153	22	10164	0.53838	0.29516
+	PHF3	6	63600802	63725522	260	25	10164	0.53823	0.29521
+	STK11	19	1170799	1238435	178	27	10164	0.53808	0.29526
+	HIST1H2AE	6	26181920	26227483	272	24	10164	0.53806	0.29527
-	TMEM54	1	32884595	32936435	54	7	10164	0.53793	0.29531
-	ARF3	12	48921156	48992526	133	7	10164	0.53782	0.29535
+	CHCHD5	2	112548606	112599040	95	11	10164	0.53766	0.2954
-	MMP7	11	102510508	102565747	214	38	10164	0.53744	0.29548
-	WFDC5	20	45099452	45151321	155	16	10164	0.53742	0.29549
-	FOXS1	20	31834300	31880617	60	11	10164	0.53708	0.2956
+	TSPAN16	19	11261139	11336996	36	5	10164	0.53696	0.29565
-	SYNJ2BP-CC	14	70315081	70452090	398	19	10164	0.53668	0.29574
+	CPA1	7	130345371	130398108	177	21	10164	0.53655	0.29579
+	ARHGEF26	3	154086003	154267827	383	26	10164	0.53621	0.29591
-	APBB2	4	40800027	41251714	1216	82	10164	0.53565	0.2961
-	RBP2	3	139442884	139511510	183	14	10164	0.53552	0.29615
-	RNF157	17	76132453	76275368	308	29	10164	0.53534	0.29621

STRAND	SYMBOL	CHR	START	STOP	NSNPS	NPARAM	N	ZSTAT	P
-	KLF12	13	73676012	74203169	1153	102	10164	0.53522	0.29625
-	COL16A1	1	31642247	31739167	182	25	10164	0.53504	0.29631
-	LOC285556	4	99626529	99857027	577	58	10164	0.53499	0.29633
-	RPRM	2	153467338	153513808	129	24	10164	0.53492	0.29635
+	OTUD1	10	23404269	23452381	181	12	10164	0.53491	0.29636
+	WNT1	12	48943453	48992613	85	6	10164	0.53431	0.29656
-	LETM1	4	1801479	1891247	87	9	10164	0.53426	0.29658
-	DPEP2	16	67977390	68035611	83	11	10164	0.53405	0.29665
-	CRLF1	19	18583225	18641850	138	13	10164	0.53394	0.29669
+	OR4K17	14	20082407	20128438	75	5	10164	0.53371	0.29677
+	IKBKB	8	42236302	42342653	187	19	10164	0.53325	0.29693
-	CNGB3	8	86563843	86778675	689	52	10164	0.53324	0.29693
-	KCNJ4	22	38416327	38490199	161	25	10164	0.53308	0.29699
+	MIOS	7	7531891	7617514	242	24	10164	0.53298	0.29702
+	C11orf1	11	111844198	111894074	97	12	10164	0.53276	0.2971
+	AIMP2	7	5974251	6033834	219	25	10164	0.53258	0.29716
+	ZBTB10	8	80450619	80536265	122	8	10164	0.53242	0.29722
-	LINGO1	15	77603024	77855900	823	51	10164	0.53233	0.29725
+	GIPC2	1	77989370	78148449	333	24	10164	0.53229	0.29726
+	USP30	12	108988089	109098026	228	30	10164	0.53229	0.29726
-	CIB2	15	78094606	78166721	241	32	10164	0.53211	0.29732
-	CBX5	12	54220946	54315131	141	15	10164	0.53192	0.29739
+	CCDC159	19	11311505	11364944	35	5	10164	0.53185	0.29742
-	ATP8B4	15	49848238	50216793	1125	87	10164	0.53179	0.29743
-	C6orf229	6	24787321	24833896	181	20	10164	0.5315	0.29754
+	FZD3	8	28459205	28584268	363	25	10164	0.53127	0.29762
-	GFOD2	16	67664533	67754370	87	10	10164	0.53095	0.29773
+	GPATCH1	19	33045880	33140412	311	15	10164	0.53087	0.29776
-	ADAR	1	154572057	154663013	168	15	10164	0.53065	0.29783
-	NEUROG3	10	69562035	69608454	94	16	10164	0.53051	0.29788
+	LOC645382	1	13268516	13319027	13	2	10164	0.53027	0.29796
+	LOC1019288	4	53540693	53586543	131	23	10164	0.52967	0.29817
+	TMPRSS9	19	2350235	2436261	450	31	10164	0.52958	0.2982
-	PSMA1	11	14494876	14678634	270	33	10164	0.52937	0.29828
-	TSGA13	7	130658646	130722432	121	15	10164	0.5292	0.29833
+	LOXL1	15	73891448	73962137	259	48	10164	0.52917	0.29834
+	ARIH2	3	48883821	48995541	151	14	10164	0.52908	0.29838
-	DNAH12	3	57283699	57579344	905	22	10164	0.52905	0.29838
-	TBC1D7	6	13294951	13363583	315	23	10164	0.52905	0.29839
-	IMPDH2	3	49014325	49064701	57	13	10164	0.52849	0.29858
-	PMS2	7	5960925	6044106	254	27	10164	0.52847	0.29859
-	BSX	11	122967649	123016671	145	18	10164	0.52838	0.29862
+	ADAM21	14	70417157	70469905	164	15	10164	0.52833	0.29863
-	ACTA1	1	229421245	229469096	104	9	10164	0.5283	0.29865
+	KDELR3	22	38433062	38493447	120	22	10164	0.52823	0.29867
+	NME8	7	37813597	37910401	486	42	10164	0.52819	0.29868
-	TLR1	4	38777555	38840800	339	27	10164	0.52816	0.29869
-	NUP210L	1	153982690	154190116	314	24	10164	0.52757	0.2989
-	DCDC5	11	30853603	31027686	200	15	10164	0.52758	0.2989
+	HAO2	1	119333776	119404130	217	31	10164	0.52746	0.29894
-	CCT6B	17	34917859	35015919	155	37	10164	0.5274	0.29896
+	HIST1H2BF	6	26164559	26209988	267	30	10164	0.52733	0.29898
-	CSH1	17	63884908	63931661	114	11	10164	0.52718	0.29903
-	CCDC68	18	54891509	54994508	302	18	10164	0.52712	0.29905
-	ERICH3	1	74558111	74708738	208	23	10164	0.52713	0.29905
-	ABTB2	11	34140987	34393008	426	54	10164	0.52708	0.29907
+	VPS16	20	2805697	2876732	232	32	10164	0.52705	0.29908
-	SYNJ2BP	14	70356496	70452090	277	22	10164	0.52697	0.29911
+	CCDC94	19	4212114	4279088	172	13	10164	0.52668	0.29921
+	CYP2C18	10	94648494	94746190	330	20	10164	0.52639	0.29931
+	TEX26	13	30897684	30985502	160	20	10164	0.52599	0.29945
-	CEACAM4	19	41606986	41662084	134	19	10164	0.52565	0.29956
-	PPP1R14A	19	38241237	38291591	239	39	10164	0.52558	0.29959

STRAND	SYMBOL	CHR	START	STOP	NSNPS	NPARAM	N	ZSTAT	P
-	TBX15	1	118873043	119024556	577	46	10164	0.52553	0.29961
-	TMEM39A	3	119418960	119498682	327	26	10164	0.52549	0.29962
+	ZNF574	19	42039905	42091567	38	10	10164	0.52544	0.29964
-	RALGAPA2	20	20379558	20747638	602	23	10164	0.52518	0.29973
-	KLK9	19	50992513	51044634	255	39	10164	0.52505	0.29978
-	FAM151A	1	54599177	54658527	206	32	10164	0.52505	0.29978
-	CREG2	2	101338354	101422988	227	22	10164	0.52491	0.29982
+	NUP50	22	45128845	45198011	211	17	10164	0.52481	0.29986
+	TBCD	17	82717060	82953186	716	26	10164	0.52439	0.3
+	CD6	11	60936641	61030377	156	23	10164	0.52431	0.30003
-	CLEC10A	17	7064537	7115601	255	34	10164	0.52422	0.30006
+	C6orf25	6	31685808	31736710	222	54	10164	0.52362	0.30027
-	LOC286238	9	88617068	88687179	56	3	10164	0.52362	0.30027
+	EBI3	19	4194302	4247542	58	6	10164	0.52353	0.3003
+	UBE2E3	2	180945385	181073427	407	14	10164	0.52328	0.30039
+	MAP2K6	17	69379697	69552329	452	54	10164	0.52315	0.30044
+	LLGL2	17	75490080	75585209	356	28	10164	0.52304	0.30047
+	TMEM169	2	216046866	216112783	143	27	10164	0.52273	0.30058
+	GALR3	22	37787009	37835865	83	9	10164	0.52261	0.30062
+	NFATC3	16	68050366	68239259	239	9	10164	0.52258	0.30063
-	ATF6B	6	32105268	32163240	202	65	10164	0.52251	0.30066
+	OR7C2	19	14906489	14952448	208	22	10164	0.52245	0.30068
-	PNPLA8	7	108460422	108563161	264	24	10164	0.52232	0.30072
-	LOC1053776	4	139590624	139643579	164	33	10164	0.52212	0.30079
+	TMEM170B	6	11503196	11593524	270	19	10164	0.52202	0.30083
+	LOC1053766	11	539016	587669	219	27	10164	0.52143	0.30103
+	CNTROB	17	7897124	7959919	65	14	10164	0.52138	0.30105
-	C7orf76	7	96471626	96538523	151	21	10164	0.52099	0.30119
+	PCDH17	13	57595104	57739311	353	47	10164	0.52087	0.30123
+	TIE1	1	43265895	43333110	59	14	10164	0.52058	0.30133
+	FRMPD1	9	37567978	37756904	533	65	10164	0.52056	0.30134
-	LSM5	7	32475333	32530258	196	16	10164	0.52047	0.30137
+	RAB39A	11	107893475	107973482	175	24	10164	0.5199	0.30157
+	KBTBD11	8	1935710	2016943	212	21	10164	0.51985	0.30158
-	HAVCR1	5	156969480	157104527	395	26	10164	0.51985	0.30158
+	CNBD2	20	35919562	36040700	224	24	10164	0.51984	0.30159
+	LOC1053706	14	104044941	104094033	111	15	10164	0.51956	0.30168
-	CCER2	19	38898980	38949370	138	20	10164	0.51947	0.30172
-	MCIDAS	5	55209597	55262315	142	13	10164	0.51924	0.3018
+	OR5H6	3	98228260	98275350	289	17	10164	0.51885	0.30193
-	TBC1D5	3	17147162	17777739	1561	31	10164	0.51878	0.30196
-	LOC728026	9	94337117	94382586	62	8	10164	0.5187	0.30199
+	EREG	4	74330143	74398760	363	20	10164	0.51853	0.30204
-	ICK	6	52991299	53096833	115	11	10164	0.51769	0.30234
-	ANO2	12	5550241	5983791	1197	92	10164	0.51759	0.30237
+	TIPRL	1	168143845	168212114	132	5	10164	0.51743	0.30243
-	PDZK1IP1	1	47173589	47225099	138	22	10164	0.51735	0.30246
-	ATP4A	19	35540193	35598658	134	29	10164	0.51717	0.30252
-	LRIG1	3	66368797	66536263	460	48	10164	0.51717	0.30252
+	INTS10	8	19782149	19862078	350	26	10164	0.51708	0.30255
-	SIAH2	3	150731123	150798476	156	10	10164	0.5165	0.30275
+	TGFB1I1	16	31437155	31487960	47	9	10164	0.51622	0.30285
-	SFRP5	10	97756751	97806999	172	19	10164	0.51607	0.3029
+	SNRPN	15	24788647	24988582	540	53	10164	0.51603	0.30292
-	ARHGAP29	1	94158907	94310068	247	32	10164	0.5158	0.303
+	LY96	8	73956329	74042562	246	19	10164	0.5157	0.30303
+	PRR16	5	120429278	120697332	924	54	10164	0.51547	0.30311
-	FH	1	241487557	241554785	180	22	10164	0.51533	0.30316
-	MITD1	2	99151427	99216061	170	20	10164	0.51531	0.30317
+	LOC1053709	15	78094038	78153173	194	26	10164	0.5152	0.30321
-	EVC2	4	5524397	5744548	749	66	10164	0.51496	0.30329
-	C14orf39	14	60343122	60521069	359	15	10164	0.51493	0.3033
+	MYD88	3	38103478	38153022	60	18	10164	0.51465	0.3034

STRAND	SYMBOL	CHR	START	STOP	NSNPS	NPARAM	N	ZSTAT	P
+	SMARCB1	22	23751963	23844518	486	25	10164	0.51462	0.30341
+	ITGA2	5	52954326	53104779	732	53	10164	0.51429	0.30352
+	DNTTIP1	20	45756937	45821427	238	24	10164	0.51391	0.30366
-	OR8B2	11	124372402	124418343	200	17	10164	0.51388	0.30367
+	KCNH8	3	19112093	19545643	574	49	10164	0.51368	0.30374
-	GLYATL2	11	58824065	58879808	293	11	10164	0.51366	0.30375
+	KCNA1	12	4874907	4928256	14	5	10164	0.51355	0.30378
-	FUZ	19	49796866	49852376	132	18	10164	0.51337	0.30385
-	NTAN1	16	15027853	15091079	34	8	10164	0.51337	0.30385
-	HAPLN1	5	83628198	83756077	296	36	10164	0.51312	0.30393
+	NPAP1	15	24640394	24693446	189	18	10164	0.51313	0.30393
-	RPL23	17	38840068	38888800	145	24	10164	0.5129	0.30401
+	TACC2	10	121954174	122264545	1207	114	10164	0.51291	0.30401
-	KRTAP7-1	21	30819039	30864732	180	26	10164	0.51283	0.30403
-	SLC2A3	12	7909228	7971296	176	31	10164	0.51254	0.30414
-	HLA-DRB1	6	32568769	32624836	1995	32	10164	0.51239	0.30419
-	PPP2R1B	11	111685074	111801445	221	19	10164	0.51236	0.3042
-	TXN	9	110233812	110291640	146	25	10164	0.51231	0.30422
+	PEG10	7	94621325	94679695	128	21	10164	0.51218	0.30426
-	LOC728715	12	9387062	9438060	106	14	10164	0.51218	0.30426
-	PROB1	5	139381946	139430196	65	9	10164	0.51205	0.30431
-	HOXA2	7	27090354	27137775	93	16	10164	0.51203	0.30432
-	TNFSF18	1	173031220	173085963	110	12	10164	0.51202	0.30432
-	ALG14	1	94971565	95107964	382	32	10164	0.51197	0.30434
+	SLC5A5	19	17836411	17905174	114	9	10164	0.51192	0.30435
-	NAGPA	16	5014844	5068941	94	11	10164	0.51172	0.30442
-	MEF2C	5	88708241	88939105	394	31	10164	0.51164	0.30445
-	GPR179	17	38315610	38378810	69	12	10164	0.51161	0.30446
-	LAMTOR3	4	99868338	99929546	72	9	10164	0.51158	0.30447
-	CPSF4L	17	73232343	73297532	186	15	10164	0.51129	0.30457
+	AEN	15	88569681	88642281	264	32	10164	0.51114	0.30463
+	RBCK1	20	373065	440966	144	27	10164	0.51102	0.30467
-	BACE1	11	117275686	117351256	173	34	10164	0.51101	0.30467
+	C7	5	40874473	41026004	766	59	10164	0.511	0.30468
+	SLC5A11	16	24810863	24921628	237	31	10164	0.51094	0.3047
+	PTX3	3	157401791	157453628	163	23	10164	0.51088	0.30472
-	OSBPL8	12	76341797	76594809	249	23	10164	0.5108	0.30475
+	CCDC66	3	56522111	56631836	424	23	10164	0.51069	0.30478
+	SGPL1	10	70780948	70891189	285	26	10164	0.51054	0.30484
-	FCAMR	1	206947647	207005659	137	17	10164	0.51035	0.3049
-	CASP4	11	104932866	105003598	233	29	10164	0.51024	0.30494
+	GAS2L1	22	29271618	29322789	103	25	10164	0.51007	0.305
-	ANGPTL6	19	10082337	10141209	90	18	10164	0.50964	0.30515
-	TCAF1	7	143841368	143937198	127	18	10164	0.50956	0.30518
+	HMGA2	12	65789460	65976291	302	25	10164	0.50946	0.30522
-	HOXA3	7	27096190	27187581	182	25	10164	0.50927	0.30528
-	PRG2	11	57376787	57425657	85	19	10164	0.50927	0.30528
+	SLMAP	3	57721221	57940003	396	26	10164	0.50909	0.30535
-	ETHE1	19	43496719	43562244	221	29	10164	0.50892	0.3054
-	GABRA2	4	46233548	46425039	499	34	10164	0.5087	0.30548
+	UPK3A	22	45249987	45305879	319	36	10164	0.50861	0.30551
+	GCNT3	15	59576783	59630011	165	21	10164	0.50856	0.30553
+	UBE2V2	8	47963288	48074708	124	34	10164	0.50853	0.30554
+	ENPP4	6	46094964	46156699	231	19	10164	0.50838	0.30559
-	EDNRB	13	77885481	78010529	516	21	10164	0.50837	0.3056
+	P3H3	12	6793373	6849854	121	18	10164	0.50837	0.3056
+	PCBP1	2	70052453	70099203	112	11	10164	0.50824	0.30564
-	INO80	15	40968880	41151354	505	20	10164	0.50814	0.30568
+	SYTL3	6	158609899	158774876	534	57	10164	0.50768	0.30584
+	TBC1D15	12	71804707	71936849	310	24	10164	0.50764	0.30585
+	PLEKHA2	8	38866235	38983912	249	43	10164	0.50726	0.30599
+	IL1RN	2	113064365	113144016	420	25	10164	0.5072	0.30601
-	RBBP8NL	20	62400237	62462680	275	37	10164	0.50682	0.30614

STRAND	SYMBOL	CHR	START	STOP	NSNPS	NPARAM	N	ZSTAT	P
-	DPP8	15	65432467	65552702	282	18	10164	0.50638	0.30629
-	FKBP8	19	18521758	18579077	89	9	10164	0.50624	0.30634
-	ERVW-1	7	92458380	92512915	125	18	10164	0.50583	0.30649
-	VTCN1	1	117133587	117305368	389	37	10164	0.50578	0.3065
-	LYPD3	19	43450794	43500679	141	26	10164	0.50571	0.30653
-	NPY1R	4	163313962	163379832	302	35	10164	0.50552	0.3066
+	EPB41	1	28852091	29130046	493	26	10164	0.50544	0.30662
+	LOC1053753	7	76489383	76554090	55	5	10164	0.50516	0.30672
+	FBXO9	6	53022783	53110873	88	10	10164	0.50503	0.30677
+	C19orf66	19	10051119	10103252	56	8	10164	0.50491	0.30681
+	SLC16A5	17	75052727	75116162	172	21	10164	0.5048	0.30685
-	KLHL31	6	53637901	53700846	253	19	10164	0.50477	0.30686
-	PRKD1	14	29566479	29962693	981	59	10164	0.50457	0.30693
+	GATA4	8	11641924	11770001	559	60	10164	0.50456	0.30694
+	DYRK3	1	206600534	206659197	109	21	10164	0.50449	0.30696
-	SOX10	22	37962312	38019532	69	12	10164	0.50437	0.307
+	PRKCA	17	66267782	66820744	2029	158	10164	0.50432	0.30702
+	GJA1	6	121400577	121459744	213	16	10164	0.50416	0.30707
+	METTL17	14	20954806	21007035	190	22	10164	0.50393	0.30716
+	BLOC1S4	4	6681115	6727660	93	17	10164	0.50356	0.30729
+	FBXL7	5	15465165	15949796	1006	46	10164	0.50346	0.30732
+	COX6B1	19	35613223	35668784	136	20	10164	0.50336	0.30735
-	FLOT2	17	28869339	28932697	66	17	10164	0.50313	0.30744
-	HOXA5	7	27131052	27178668	93	16	10164	0.50307	0.30746
+	ZFYVE27	10	97702121	97770907	221	25	10164	0.50276	0.30757
-	SCN3A	2	165077520	165239088	371	25	10164	0.50249	0.30766
+	ACAD8	11	134218537	134275944	86	13	10164	0.50246	0.30767
+	TSGA10IP	11	65910445	65970392	147	26	10164	0.50221	0.30776
+	TGM3	20	2260967	2351079	37	9	10164	0.50217	0.30777
-	DCHS1	11	6611324	6690849	175	30	10164	0.50181	0.3079
-	KLRB1	12	9585274	9642901	138	10	10164	0.50172	0.30793
-	C17orf59	17	8178333	8225246	123	25	10164	0.50156	0.30799
+	UGCG	9	111861766	111945374	469	41	10164	0.50149	0.30801
+	LMNB1	5	126741623	126847020	198	22	10164	0.50137	0.30806
+	CYB5B	16	69389595	69476264	261	33	10164	0.50114	0.30814
-	RIPK4	21	41729369	41802089	255	35	10164	0.50111	0.30815
+	PPAPDC3	9	131254682	131319262	112	11	10164	0.50078	0.30826
-	FIGNL2	12	51807892	51883760	121	23	10164	0.50063	0.30831
-	PASK	2	241096099	241185364	392	33	10164	0.50042	0.30839
+	CHD7	8	60643762	60878028	516	33	10164	0.50037	0.30841
+	LCE3B	1	152578811	152624098	59	5	10164	0.50015	0.30849
-	INS	11	2149779	2196209	188	22	10164	0.49978	0.30861
-	CORO6	17	29604756	29657908	38	7	10164	0.49967	0.30865
-	SEC63	6	107857756	107993278	296	26	10164	0.49959	0.30868
+	DOPEY1	6	83032660	83178471	321	30	10164	0.49957	0.30869
+	SAMD4A	14	54532612	54803315	681	114	10164	0.49949	0.30872
+	LIMK2	22	31177264	31290080	296	20	10164	0.49946	0.30873
+	HIST1H4E	6	26169645	26215021	264	27	10164	0.49927	0.30879
+	AXL	19	41184199	41271767	166	23	10164	0.49916	0.30883
+	MOGAT1	2	222636738	222719930	222	29	10164	0.49902	0.30888
+	PHKG2	16	30713299	30771176	13	2	10164	0.49901	0.30889
-	RD3L	14	103930426	103977308	199	11	10164	0.49895	0.30891
+	OR5H2	3	98247888	98293832	308	14	10164	0.49883	0.30895
-	IFITM10	11	1722410	1785594	207	28	10164	0.49875	0.30898
-	DCTN2	12	57520050	57582331	112	20	10164	0.49853	0.30906
+	OR11H4	14	20207792	20253766	84	14	10164	0.49842	0.30909
-	MMP21	10	125754939	125820224	228	29	10164	0.49836	0.30911
+	CMTM7	3	32356671	32464841	429	37	10164	0.49833	0.30913
+	VPS13B	8	98978071	99887586	1425	22	10164	0.49832	0.30913
+	CDC16	13	114199845	114282675	329	18	10164	0.49799	0.30925
+	PARVB	22	43964211	44179233	271	43	10164	0.49797	0.30925
+	ZNF227	19	44172547	44247268	159	15	10164	0.49764	0.30937
-	TBATA	10	70761238	70820401	271	31	10164	0.49745	0.30944

STRAND	SYMBOL	CHR	START	STOP	NSNPS	NPARAM	N	ZSTAT	P
-	CKB	14	103509658	103557859	163	20	10164	0.49731	0.30949
-	RCOR2	11	63901221	63951844	80	11	10164	0.49712	0.30955
-	SLC22A18AS	11	2877344	2938945	147	33	10164	0.49702	0.30959
+	APOBEC3C	22	38979260	39028838	118	16	10164	0.49689	0.30963
+	CDCA7	2	173319017	173378990	144	22	10164	0.49688	0.30964
+	LOC1053756	7	157393233	157451783	300	23	10164	0.49651	0.30977
+	CCDC65	12	48869110	48931576	136	8	10164	0.49637	0.30982
+	FBLN1	22	45467839	45611134	567	76	10164	0.49616	0.30989
-	HCRT	17	42174060	42220452	56	11	10164	0.49558	0.31009
+	DSG4	18	31341777	31425791	215	28	10164	0.49505	0.31028
+	C2	6	31862785	31955674	448	74	10164	0.49485	0.31035
+	CCDC42B	12	113114181	113169276	110	7	10164	0.49426	0.31056
-	KCNS1	20	45082072	45136112	162	21	10164	0.49415	0.3106
+	ZNF273	7	64835177	64942239	470	22	10164	0.49415	0.3106
-	BCAT2	19	48785062	48846063	72	11	10164	0.49359	0.3108
-	CSHL1	17	63899602	63946323	105	11	10164	0.49331	0.3109
+	TRH	3	129939393	129987938	242	21	10164	0.49331	0.3109
+	RBX1	22	40916347	40983015	71	19	10164	0.49329	0.3109
+	SART1	11	65926689	65990136	189	25	10164	0.49323	0.31093
-	CDC42SE1	1	151040971	151094649	110	15	10164	0.49317	0.31095
-	VMO1	17	4775285	4821434	135	28	10164	0.49317	0.31095
-	RPL4	15	66489315	66539855	150	20	10164	0.49311	0.31097
-	SLC2A9	4	9761125	10075248	1267	31	10164	0.49295	0.31103
-	RANBP10	16	67713070	67841652	134	14	10164	0.4929	0.31104
-	ZNF296	19	45061500	45111430	118	12	10164	0.49277	0.31109
+	NUDT16L1	16	4658558	4705859	64	3	10164	0.49269	0.31112
+	APOC1	19	44879325	44929349	110	16	10164	0.49232	0.31125
+	LOC1053746	5	25155596	25530991	903	39	10164	0.49226	0.31127
-	RDH13	19	55031319	55104774	262	31	10164	0.49223	0.31128
+	SPECC1	17	19974336	20324759	684	12	10164	0.49181	0.31143
-	LUZP1	1	23074023	23212808	266	13	10164	0.49166	0.31148
+	SAXO2	15	82227811	82294927	184	22	10164	0.49125	0.31162
+	OR52J3	11	5011526	5057461	354	14	10164	0.49118	0.31165
-	ADCY4	14	24308349	24370071	188	23	10164	0.49112	0.31167
+	EPS8L1	19	55040853	55097923	198	27	10164	0.49107	0.31169
+	RPS27	1	153955763	154002155	51	11	10164	0.49102	0.31171
-	DDAH1	1	85308485	85613363	1018	84	10164	0.49101	0.31171
+	ZNF771	16	30372414	30428595	1	1	10164	0.49098	0.31172
-	SPATA24	5	139385940	139439155	67	11	10164	0.49066	0.31183
+	LOC1053708	15	59994652	60051899	101	15	10164	0.49061	0.31185
-	EIF4E	4	98868456	98965635	151	8	10164	0.49047	0.3119
+	NUP214	9	131089959	131244670	297	19	10164	0.49049	0.3119
+	CPEB4	5	173853328	173970310	275	20	10164	0.49039	0.31193
-	RRM2B	8	102194501	102274118	249	28	10164	0.49019	0.312
+	ZBTB8A	1	32504145	32615950	210	13	10164	0.49008	0.31204
+	MEST	7	130451175	130516297	100	10	10164	0.48959	0.31221
+	KIAA0232	4	6747732	6894172	143	16	10164	0.48955	0.31222
+	NTSR1	20	62673783	62772771	449	53	10164	0.48946	0.31226
-	CWC25	17	38790434	38860350	176	17	10164	0.48943	0.31227
+	SHANK3	22	50639642	50743212	105	19	10164	0.48935	0.3123
-	BCL7C	16	30824041	30929376	89	5	10164	0.4892	0.31235
+	GRHPR	9	37387666	37446993	198	26	10164	0.48866	0.31254
-	OR6B2	2	240018965	240068858	43	11	10164	0.48865	0.31254
+	LRRC8C	1	89580873	89729535	255	21	10164	0.48861	0.31256
-	DMPK	19	45759709	45817557	125	18	10164	0.48795	0.31279
-	GLTP	12	109840943	109915488	185	19	10164	0.48783	0.31284
-	RFFL	17	34999112	35124329	227	39	10164	0.4876	0.31291
+	ELFN1	7	1631056	1757954	263	27	10164	0.48749	0.31295
+	SPAG4	20	35580853	35631045	119	17	10164	0.48742	0.31298
+	PTCD2	5	72285367	72378397	68	17	10164	0.48742	0.31298
-	TAC3	12	56999997	57051560	91	7	10164	0.4872	0.31306
-	PIH1D2	11	112042409	112109171	110	17	10164	0.48719	0.31306
-	EEF1D	8	143569697	143632675	198	21	10164	0.48663	0.31326

STRAND	SYMBOL	CHR	START	STOP	NSNPS	NPARAM	N	ZSTAT	P
+	NRG3	10	81840309	82997179	3605	218	10164	0.48654	0.31329
+	HTATIP2	11	20328685	20393783	292	42	10164	0.48641	0.31334
+	CLEC3B	3	44991267	45046071	165	14	10164	0.48621	0.31341
+	C9orf135	9	69785800	69916237	371	37	10164	0.48608	0.31345
+	GPC1	2	240400698	240478078	309	30	10164	0.48599	0.31349
+	C15orf32	15	92436654	92511117	234	42	10164	0.48538	0.3137
+	DPYSL4	10	132149987	132215776	265	30	10164	0.48522	0.31376
+	KIF3B	20	32242651	32345011	151	12	10164	0.48515	0.31379
+	LDHAL6A	11	18420827	18489600	209	14	10164	0.48502	0.31383
+	AIF1	6	31580192	31627021	292	82	10164	0.48486	0.31389
+	WISP2	20	44679993	44737812	117	23	10164	0.48484	0.31389
+	OR51T1	11	4846819	4892883	98	10	10164	0.4848	0.31391
+	OR2L5	1	247986948	248032886	79	5	10164	0.48472	0.31394
-	ELOF1	19	11543043	11594236	84	15	10164	0.48455	0.314
-	OVCH1	12	29412128	29532800	443	50	10164	0.48442	0.31405
+	UGT2B28	4	69245468	69305050	63	10	10164	0.4837	0.3143
+	NSRP1	17	30081807	30196475	161	10	10164	0.48348	0.31438
-	LOC1053791	5	132948513	132997693	130	13	10164	0.48348	0.31438
-	SNX4	3	125436644	125555214	311	21	10164	0.48345	0.31439
+	UHRF1	19	4868080	4972154	219	39	10164	0.48343	0.31439
+	DMBT1	10	122525665	122653736	523	21	10164	0.48321	0.31447
+	CASP7	10	113644162	113740909	372	29	10164	0.4832	0.31448
-	LOC388813	21	14581392	14678107	171	22	10164	0.48316	0.31449
-	TRPC3	4	121869028	121986891	241	37	10164	0.48313	0.3145
+	RREB1	6	7072597	7261980	405	38	10164	0.48261	0.31469
-	NUDT5	10	12155330	12231144	184	27	10164	0.48251	0.31472
-	NELL2	12	44498275	44956848	1258	52	10164	0.48233	0.31478
+	FBRS	16	30623431	30680810	34	2	10164	0.48232	0.31479
+	ANKRD62	18	12037533	12139764	350	11	10164	0.48221	0.31483
-	BEND7	10	13428484	13563597	428	34	10164	0.48196	0.31492
+	LOC1053745	2	42773519	42981692	1035	108	10164	0.48188	0.31494
+	MAP1B	5	72072291	72219570	363	53	10164	0.48149	0.31508
+	LIMS1	2	108499355	108697246	447	14	10164	0.48142	0.31511
+	WFDC13	20	45667016	45718817	164	17	10164	0.48138	0.31512
-	PRAMEF8	1	13271304	13320137	15	2	10164	0.48128	0.31516
-	QRICH1	3	49019707	49129071	137	16	10164	0.48116	0.3152
+	RECK	9	36001913	36134455	304	21	10164	0.48114	0.31521
-	C17orf96	17	38661703	38709934	61	12	10164	0.4808	0.31533
-	LCA5	6	79474991	79572430	275	28	10164	0.48078	0.31534
+	PKN1	19	14398053	14481867	219	38	10164	0.48063	0.31539
-	CHORDC1	11	90183947	90258364	257	27	10164	0.48038	0.31548
+	AASDHPPT	11	106042565	106108692	116	17	10164	0.4802	0.31554
-	PPAP2B	1	56484747	56614584	426	48	10164	0.47992	0.31564
-	ACSM1	16	20613233	20732744	279	19	10164	0.47966	0.31573
-	GPR75-ASB3	2	53660293	53895033	784	32	10164	0.47951	0.31579
+	LOC1001290	19	51380906	51427425	82	20	10164	0.47937	0.31584
-	COL9A1	6	70205061	70338083	564	52	10164	0.47932	0.31585
-	LGALS7	19	38760968	38808517	200	21	10164	0.47922	0.31589
+	LIPT1	2	99119955	99173157	148	14	10164	0.47873	0.31607
+	TM9SF4	20	32074506	32177258	213	14	10164	0.47859	0.31612
-	PGM3	6	83141972	83228936	220	33	10164	0.47836	0.3162
+	TGM6	20	2345908	2442753	309	26	10164	0.47834	0.31621
+	ZC3H18	16	88535379	88641964	317	26	10164	0.47803	0.31632
-	PRKAG2	7	151546114	151912231	1140	131	10164	0.47747	0.31652
-	DDX60L	4	168346735	168515515	602	54	10164	0.4774	0.31654
+	NKAIN3	8	62213310	63009652	2717	101	10164	0.47682	0.31674
+	RANGRF	17	8253387	8300092	153	25	10164	0.47666	0.3168
+	ZNF221	19	43916223	43977600	221	22	10164	0.47652	0.31685
-	DDX52	17	37599739	37678464	138	31	10164	0.47638	0.3169
-	NCR3	6	31578883	31628019	305	76	10164	0.47637	0.31691
+	ARMC7	17	75074952	75140265	166	25	10164	0.47605	0.31702
+	SETDB1	1	150891339	150974901	161	15	10164	0.476	0.31704
-	MIER2	19	295573	379793	57	3	10164	0.47599	0.31704

STRAND	SYMBOL	CHR	START	STOP	NSNPS	NPARAM	N	ZSTAT	P
-	KAT6A	8	41919479	42086989	310	21	10164	0.47596	0.31705
-	OPCML	11	132404981	133567508	3234	255	10164	0.47573	0.31713
+	RIMKLA	1	42345797	42434229	261	18	10164	0.47568	0.31715
-	GTF3C1	16	27450613	27584930	117	16	10164	0.47552	0.31721
-	RINL	19	38857830	38913279	125	32	10164	0.47536	0.31727
+	NOM1	7	156914723	156983182	384	24	10164	0.47521	0.31732
-	NBPF11	1	148092046	148187190	28	1	10164	0.47481	0.31746
-	DENND4B	1	153919501	153981681	84	9	10164	0.47479	0.31747
+	TAPBPL	12	6416655	6472322	172	28	10164	0.47411	0.31771
-	SLC25A20	3	48846923	48933993	118	11	10164	0.47408	0.31772
-	LIN9	1	226221149	226345099	249	31	10164	0.47408	0.31772
-	USP19	3	49098046	49155938	90	9	10164	0.47359	0.3179
+	SLC12A2	5	128048791	128199688	219	16	10164	0.47351	0.31792
-	POP5	12	120569045	120616452	142	25	10164	0.47333	0.31799
-	TOP3B	22	21947025	22017843	142	19	10164	0.47327	0.31801
+	PCSK9	1	55004476	55074853	216	37	10164	0.47321	0.31803
+	THBS1	15	39546079	39607467	166	15	10164	0.47315	0.31805
-	MIF4GD	17	75256228	75306292	155	10	10164	0.47314	0.31806
-	UBE2G1	17	4259217	4401674	240	15	10164	0.47308	0.31808
+	GPX5	6	28491012	28544951	236	31	10164	0.473	0.31811
-	USP49	6	41779896	41930361	237	21	10164	0.473	0.31811
+	C4orf51	4	145645104	145810392	105	16	10164	0.47244	0.31831
+	OR4X1	11	48228861	48274778	189	14	10164	0.47235	0.31834
-	CD300C	17	74531065	74581171	252	27	10164	0.47186	0.31851
+	UBE2Q2	15	75808286	75911047	154	34	10164	0.47187	0.31851
-	APRT	16	88799469	88846934	315	23	10164	0.47185	0.31852
-	ODC1	2	10430371	10483554	33	5	10164	0.47154	0.31863
-	RSPH3	6	158966739	159035432	195	7	10164	0.47146	0.31866
+	SRRM4	12	118946486	119173053	878	81	10164	0.47115	0.31877
-	IHH	2	219044420	219095516	117	10	10164	0.47102	0.31881
+	WDR36	5	111057172	111140502	282	32	10164	0.47067	0.31894
+	EPHA6	3	96779581	97774977	1820	72	10164	0.47056	0.31898
-	C19orf48	19	50787693	50839853	227	26	10164	0.4705	0.319
-	LLPH	12	66113069	66165753	130	11	10164	0.47012	0.31914
-	TPPP3	16	67379807	67428535	47	12	10164	0.47004	0.31916
+	DEPDC5	22	31718951	31917034	597	32	10164	0.46991	0.31921
+	GRHL1	2	9916663	10012284	369	27	10164	0.46934	0.31941
+	CDC42	1	22017627	22102943	263	22	10164	0.46922	0.31946
-	MYLK	3	123602296	123919302	507	50	10164	0.46878	0.31961
+	SOD1	21	31624622	31678931	75	16	10164	0.46872	0.31963
+	MAP1LC3B2	12	116524208	116586620	165	27	10164	0.4687	0.31964
-	LOC1053719	17	80875804	80926713	269	25	10164	0.46865	0.31966
+	PRND	20	4686854	4738462	167	32	10164	0.46832	0.31978
-	LRRFIP2	3	37042626	37211817	276	15	10164	0.46793	0.31992
+	KXD1	19	18522762	18579387	88	9	10164	0.46792	0.31992
-	MRPL3	3	131452201	131538016	177	25	10164	0.46777	0.31998
-	FAM192A	16	57142466	57221230	86	6	10164	0.46769	0.32
+	LOC1019275	2	241232602	241287002	147	25	10164	0.46768	0.32001
+	LOC1053777	5	175996325	176045274	20	6	10164	0.46762	0.32003
+	MSX2	5	174689572	174740899	156	24	10164	0.46747	0.32008
-	CSNK2A3	11	11341942	11388357	147	36	10164	0.46738	0.32012
-	SLC10A1	14	69765835	69832289	90	6	10164	0.46681	0.32032
-	RNF19B	1	32922498	33000266	107	9	10164	0.4668	0.32032
+	DLST	14	74846891	74913747	136	12	10164	0.46673	0.32035
-	FAM101B	17	429978	480940	71	13	10164	0.46657	0.3204
+	RBFOX1	16	5204782	7723340	13005	622	10164	0.46643	0.32045
+	LOC1053779	6	109148930	109213797	190	17	10164	0.46635	0.32048
+	TSHZ1	18	75175755	75299950	280	42	10164	0.46625	0.32052
-	PIGP	21	37055364	37108158	230	15	10164	0.46618	0.32054
+	PDCD2L	19	34369398	34436168	76	7	10164	0.46615	0.32055
-	ARRB1	11	75250122	75386831	306	53	10164	0.46589	0.32065
+	SLC13A2	17	28438357	28507781	124	11	10164	0.4658	0.32068
-	ATP5E	20	59018678	59067367	50	6	10164	0.46563	0.32074

STRAND	SYMBOL	CHR	START	STOP	NSNPS	NPARAM	N	ZSTAT	P
-	ACAA1	3	38112710	38172242	70	19	10164	0.46557	0.32076
-	LTBP3	11	65528559	65593361	135	32	10164	0.46554	0.32077
+	DUOX1	15	45094992	45175578	165	25	10164	0.46554	0.32077
-	ZFP14	19	36324453	36414203	236	20	10164	0.4655	0.32079
-	ESR2	14	64162925	64373550	571	40	10164	0.46547	0.3208
-	FRMD1	6	168045745	168136514	87	16	10164	0.46519	0.3209
-	GAK	4	839277	967390	450	35	10164	0.46457	0.32112
+	KAT6B	10	74789927	75042623	417	27	10164	0.46434	0.3212
+	CAPN11	6	44123811	44194402	80	13	10164	0.46421	0.32125
+	NRSN1	6	24091186	24157529	182	39	10164	0.46407	0.3213
+	TBC1D24	16	2440146	2515733	12	4	10164	0.46403	0.32131
-	MORN3	12	121641118	121707639	217	21	10164	0.46404	0.32131
-	NETO1	18	72732314	72902575	577	61	10164	0.46402	0.32132
+	RPL13	16	89525657	89576829	194	20	10164	0.46389	0.32136
+	ARL2BP	16	57210126	57263635	57	3	10164	0.46389	0.32136
+	ELF3	1	201975519	202027188	225	30	10164	0.4638	0.3214
+	NIPBL	5	36841759	37075819	260	25	10164	0.46378	0.3214
-	DALRD3	3	49005488	49056386	58	14	10164	0.46371	0.32143
-	CAPZB	1	19328773	19520641	641	35	10164	0.46349	0.32151
-	MAPK12	22	50242902	50296759	190	34	10164	0.46342	0.32153
+	OR2L8	1	247913858	247959796	146	11	10164	0.46333	0.32156
-	TBC1D16	17	79922343	80070867	403	53	10164	0.46302	0.32167
-	MOB3A	19	2061032	2131502	169	33	10164	0.46285	0.32173
+	LOC1053781	6	163376037	163424279	65	5	10164	0.46271	0.32179
-	COPE	19	18889514	18954464	122	24	10164	0.46246	0.32188
+	ITGA4	2	181421892	181547747	305	33	10164	0.46242	0.32189
+	TAF5	10	103332953	103399065	115	21	10164	0.46241	0.32189
-	OR13A1	10	45292294	45350608	251	25	10164	0.46233	0.32192
-	BCAT1	12	24800024	24984459	667	44	10164	0.46206	0.32202
-	POLR3K	16	36979	88632	152	15	10164	0.46207	0.32202
+	OR2T2	1	248414062	248558832	67	5	10164	0.46205	0.32202
+	LOC1053792	1	248410607	248558838	67	5	10164	0.46205	0.32202
-	RALGAPA1	14	35528352	35844304	624	30	10164	0.46174	0.32213
+	HLA-DRA	6	32404842	32455046	894	41	10164	0.4614	0.32225
+	SLC30A8	8	116915180	117186714	623	40	10164	0.46133	0.32228
-	CCDC158	4	76303039	76456893	335	29	10164	0.46087	0.32244
+	FREM2	13	38652036	38897131	507	57	10164	0.46089	0.32244
-	CYB5A	18	74243292	74327016	383	18	10164	0.46077	0.32248
+	CCDC180	9	97272628	97387295	372	22	10164	0.46061	0.32254
-	NME3	16	1760320	1806758	282	27	10164	0.46015	0.3227
+	PNLIP	10	116510916	116577855	130	9	10164	0.46004	0.32274
+	TUBB3	16	89887009	89946097	242	19	10164	0.45954	0.32292
+	ANKRD61	7	5996376	6046552	201	23	10164	0.45943	0.32296
-	CIR1	2	174338150	174430715	139	4	10164	0.45938	0.32298
+	IGFBP1	7	45853360	45903668	191	26	10164	0.4592	0.32305
+	C19orf33	19	38269164	38315006	210	45	10164	0.45918	0.32305
+	TCP10L2	6	167135593	167203779	189	21	10164	0.45897	0.32313
-	MYO1A	12	57018517	57085765	127	10	10164	0.45888	0.32316
-	UNC13A	19	17591328	17723353	148	24	10164	0.45877	0.3232
+	TBPL1	6	133917170	133997500	154	12	10164	0.45861	0.32326
+	CYP2C19	10	94727681	94863205	463	24	10164	0.45855	0.32328
+	POP1	8	98082293	98169841	117	11	10164	0.45779	0.32355
+	EFR3B	2	25007104	25169135	408	25	10164	0.45778	0.32355
-	SPOCD1	1	31780422	31851051	150	21	10164	0.45767	0.32359
+	PDE6H	12	14938022	14991865	218	28	10164	0.45757	0.32363
-	THEM5	1	151837101	151888697	132	18	10164	0.45758	0.32363
+	FBXL8	16	67124988	67174174	41	9	10164	0.45752	0.32365
+	LOC1053753	7	76475102	76534424	66	5	10164	0.45749	0.32366
+	OSTF1	9	75053482	75158137	287	29	10164	0.45745	0.32367
+	ADAM29	4	174883358	174988180	404	18	10164	0.45728	0.32373
-	ZNF510	9	96744553	96813119	193	9	10164	0.45728	0.32373
+	TMCO2	1	40212901	40261693	130	10	10164	0.45726	0.32374
-	THSD7A	7	11360435	11867198	2016	131	10164	0.45726	0.32374

STRAND	SYMBOL	CHR	START	STOP	NSNPS	NPARAM	N	ZSTAT	P
+	LOC1019273	13	24506663	24577321	303	26	10164	0.45708	0.32381
-	ACTRT3	3	169756921	169804895	93	11	10164	0.45665	0.32396
-	C2CD4B	15	62153538	62200283	96	14	10164	0.45651	0.32401
-	OR10G7	11	124028066	124074001	230	24	10164	0.45635	0.32407
+	DSG3	18	31412769	31488702	217	23	10164	0.45613	0.32415
-	ACOX3	4	8356263	8475725	335	31	10164	0.45597	0.3242
+	LOC1053691	11	744359	790898	126	21	10164	0.45581	0.32426
-	SCRG1	4	173378148	173511691	266	21	10164	0.45572	0.32429
+	VAPA	18	9878958	9970021	23	6	10164	0.45558	0.32435
+	LDHC	11	18377306	18461246	282	16	10164	0.45501	0.32455
+	GLTSCR2	19	47710536	47767066	95	8	10164	0.45497	0.32457
+	GABPB2	1	151035604	151139119	201	19	10164	0.45428	0.32481
+	C14orf79	14	104951279	105010307	192	24	10164	0.45426	0.32482
+	C5orf64	5	61602781	61740997	417	39	10164	0.45425	0.32482
-	NRXN2	11	64596174	64758188	273	28	10164	0.45424	0.32483
-	KIAA1407	3	113954137	114091641	255	26	10164	0.45398	0.32492
+	SYTL1	1	27306992	27363932	77	13	10164	0.45377	0.325
-	HNRNPC	14	21199136	21304479	14	6	10164	0.45354	0.32508
+	EXOC3	5	408209	477305	411	29	10164	0.45344	0.32511
-	TCF4	18	55212331	55670993	813	71	10164	0.45331	0.32516
-	TRMT112	11	64306492	64353084	167	32	10164	0.45326	0.32518
+	PCDHGB7	5	141382647	141522981	284	27	10164	0.45322	0.32519
-	SARS2	19	38905264	38965896	178	27	10164	0.4532	0.3252
-	CDC42BPB	14	102922379	103092405	449	40	10164	0.45308	0.32525
+	R3HCC1L	10	98099624	98254897	238	14	10164	0.45269	0.32538
-	BBS2	16	56460389	56555096	356	32	10164	0.45261	0.32542
-	ADAMTS1	21	26826287	26880409	182	22	10164	0.45249	0.32546
+	ATP6V1F	7	128827803	128875849	141	13	10164	0.45235	0.32551
-	UBR5	8	102242273	102447689	525	40	10164	0.45235	0.32551
-	CACNA1S	1	201029507	201147566	435	74	10164	0.45217	0.32557
-	PATZ1	22	31315804	31381263	159	26	10164	0.45218	0.32557
+	RIBC2	22	45378691	45442422	211	17	10164	0.45219	0.32557
+	CREB3L4	1	153932839	153984364	62	9	10164	0.45181	0.3257
-	OR4F4	15	101912142	101958059	1	1	10164	0.45167	0.32575
+	LOC1053753	7	1581880	1652739	170	18	10164	0.45167	0.32575
+	PLXDC2	10	19781227	20299856	2211	114	10164	0.45162	0.32577
-	PPP2R5E	14	63362479	63578361	697	36	10164	0.4516	0.32578
-	LYG2	2	99188503	99291732	248	26	10164	0.4515	0.32582
-	HOXA1	7	27082993	27131006	94	19	10164	0.45112	0.32595
+	PRKCE	2	45616315	46197990	1940	210	10164	0.45075	0.32609
+	TBC1D13	9	128752207	128820432	153	15	10164	0.45073	0.32609
+	C8orf22	8	49019336	49086083	150	20	10164	0.45052	0.32617
-	C9orf129	9	93308199	93381414	284	32	10164	0.45039	0.32622
+	RABL6	9	136772922	136851187	195	15	10164	0.45036	0.32623
-	MAP6D1	3	183805876	183860605	173	21	10164	0.45023	0.32627
+	PCDHGA11	5	141385970	141522979	277	27	10164	0.45006	0.32634
-	PER2	2	238234038	238325102	272	36	10164	0.4498	0.32643
+	TARDBP	1	10977622	11035492	106	13	10164	0.44977	0.32644
-	REV1	2	99390475	99525030	306	18	10164	0.44976	0.32644
+	HELLS	10	94510767	94612099	242	20	10164	0.44971	0.32646
-	SIRPG	20	1619152	1721516	319	13	10164	0.44971	0.32646
+	TAF4B	18	26191445	26401685	567	36	10164	0.44957	0.32651
+	RALGPS2	1	178690147	178931842	565	33	10164	0.44957	0.32651
-	ARHGDIB	12	14932015	14996628	263	31	10164	0.44906	0.32669
-	DENND1B	1	197494748	197817175	457	18	10164	0.44906	0.3267
+	PDGFRL	8	17541433	17653133	574	57	10164	0.44841	0.32693
-	C11orf68	11	65906810	65954060	109	25	10164	0.44828	0.32697
-	KLK8	19	50986008	51036702	225	29	10164	0.44816	0.32702
+	TP73	1	3617565	3746201	179	26	10164	0.44801	0.32707
-	C9orf84	9	111676175	111830008	615	26	10164	0.44795	0.32709
-	METTL21C	13	102675747	102729521	193	32	10164	0.4479	0.32711
+	EPB41L5	2	119978028	120189121	316	11	10164	0.44783	0.32714
+	AMOTL1	11	94671436	94886753	461	33	10164	0.44782	0.32714

STRAND	SYMBOL	CHR	START	STOP	NSNPS	NPARAM	N	ZSTAT	P
-	SLC17A5	6	73583379	73689014	522	20	10164	0.44773	0.32717
+	AHSP	16	31492852	31538803	124	8	10164	0.44769	0.32719
-	ISYNA1	19	18424388	18473301	86	6	10164	0.44737	0.32731
-	IFFO2	1	18894280	18991667	279	33	10164	0.44721	0.32736
+	AMY1A	1	103620519	103674551	1	1	10164	0.44684	0.3275
+	KDM3A	2	86405647	86502716	168	16	10164	0.44674	0.32753
-	PGRMC2	4	128259237	128323829	120	21	10164	0.44614	0.32775
-	SEC13	3	10290929	10356188	239	41	10164	0.44601	0.32779
+	C6orf120	6	169667161	169716306	110	13	10164	0.44592	0.32783
-	N4BP2L2	13	32422488	32573885	286	36	10164	0.44588	0.32784
+	RHBG	1	156331043	156395234	157	34	10164	0.44585	0.32785
-	SSBP2	5	81407360	81786253	815	50	10164	0.44583	0.32786
-	CCNA2	4	121806444	121858933	139	21	10164	0.44565	0.32793
+	ZNF610	19	52296863	52377123	312	28	10164	0.4456	0.32794
-	DHX32	10	125826340	125916315	202	28	10164	0.44553	0.32797
+	LCLAT1	2	30412226	30654225	830	24	10164	0.4454	0.32802
+	OTOGL	12	80095502	80389090	343	22	10164	0.44539	0.32802
-	PDILT	16	20349170	20439742	285	39	10164	0.44524	0.32807
+	SLC31A1	9	113186528	113274492	276	29	10164	0.44501	0.32816
-	EEF2KMT	16	5074300	5132820	8	5	10164	0.4447	0.32827
-	SARAF	8	30053012	30118208	125	12	10164	0.44455	0.32832
+	KRTAP5-10	11	71530563	71576620	200	12	10164	0.44439	0.32838
-	RAB40B	17	82644959	82733722	338	35	10164	0.44428	0.32842
-	KATNAL1	13	30192630	30342487	359	35	10164	0.44428	0.32842
+	DNM3	1	171806478	172428466	1660	82	10164	0.44401	0.32852
+	RAB26	16	2113144	2164140	23	15	10164	0.44392	0.32855
+	LOC1053742	3	184360351	184461071	296	44	10164	0.44388	0.32856
+	C1orf106	1	200856074	200925736	229	46	10164	0.44381	0.32859
+	SERPINA5	14	94546369	94603120	299	24	10164	0.44378	0.3286
-	CCDC177	14	69559814	69609883	81	11	10164	0.44363	0.32865
-	CLDN8	21	30204006	30251151	218	21	10164	0.44359	0.32867
-	WDR26	1	224375143	224469299	222	20	10164	0.4436	0.32867
-	CORO1B	11	67428047	67478821	93	19	10164	0.44309	0.32885
+	ORAI2	7	102398530	102466821	110	18	10164	0.4431	0.32885
+	PSEN2	1	226835572	226906103	271	33	10164	0.44301	0.32888
-	CT62	15	71100244	71150500	57	10	10164	0.44288	0.32892
+	OTUB1	11	63950853	64008420	162	16	10164	0.44287	0.32893
-	PC	11	66838522	66993376	324	39	10164	0.44284	0.32894
+	LOC1053756	7	158398391	158457848	81	13	10164	0.44269	0.32899
-	DFNB31	9	114392078	114540509	511	42	10164	0.4425	0.32906
-	DNAAF2	14	49615174	49670230	194	14	10164	0.44247	0.32907
-	DNAJC17	15	40757867	40842478	245	34	10164	0.44241	0.3291
-	P4HA2	5	132182009	132262863	273	38	10164	0.44236	0.32911
-	RDX	11	110164880	110331712	280	22	10164	0.44219	0.32917
+	CEMIP	15	80744343	80961662	476	74	10164	0.44215	0.32919
+	SLC7A6	16	68228830	68311823	123	13	10164	0.44199	0.32925
-	GDF9	5	132851181	132901884	113	14	10164	0.44194	0.32927
+	MARC2	1	220712502	220794815	532	21	10164	0.44188	0.32929
-	DHX29	5	55246245	55342693	190	17	10164	0.44184	0.3293
-	TAL1	1	47206290	47267373	192	28	10164	0.44157	0.3294
+	BTNL9	5	181005225	181071523	232	18	10164	0.44132	0.32949
-	CHST15	10	123944351	124128684	427	35	10164	0.44102	0.3296
+	DGAT2	11	75733733	75811536	232	25	10164	0.44077	0.32969
-	CRABP2	1	156689606	156740816	99	25	10164	0.44061	0.32975
-	TOX	8	58795418	59154208	896	71	10164	0.44041	0.32982
-	CCDC154	16	1423425	1479844	240	30	10164	0.44027	0.32987
-	FAHD2B	2	97072404	97129882	97	13	10164	0.43963	0.3301
+	LOC645177	12	24962562	25076516	415	45	10164	0.43946	0.33017
+	MBD3L2	19	7014340	7061735	20	4	10164	0.43939	0.33019
+	PRPF18	10	13551939	13640868	315	20	10164	0.43935	0.3302
+	GNAO1	16	56156339	56367444	686	60	10164	0.43882	0.3304
+	XAF1	17	6720572	6785647	202	27	10164	0.43871	0.33043
+	SIPA1L3	19	37872221	38218372	1462	89	10164	0.43861	0.33047

STRAND	SYMBOL	CHR	START	STOP	NSNPS	NPARAM	N	ZSTAT	P
+	C10orf126	10	28811408	28891898	362	29	10164	0.43861	0.33047
+	ADRB2	5	148791593	148838634	177	28	10164	0.4386	0.33048
-	BTLA	3	112453966	112534702	196	18	10164	0.43859	0.33048
+	SCRN3	2	174360729	174439575	138	4	10164	0.43843	0.33054
-	NR5A1	9	124471232	124542448	123	20	10164	0.43836	0.33056
+	MYRF	11	61717637	61798518	144	22	10164	0.43807	0.33067
-	ORMDL3	17	39911042	39962631	165	14	10164	0.43801	0.33069
-	TXNL4A	18	79962221	80023592	82	8	10164	0.43794	0.33072
-	SCT	11	614989	662240	169	22	10164	0.43758	0.33085
-	SUMO2	17	75157730	75218003	135	18	10164	0.43744	0.3309
-	CTSD	11	1742752	1798992	172	32	10164	0.43718	0.33099
-	CPNE4	3	131523560	132320547	3158	126	10164	0.43708	0.33103
-	TMEM220	17	10703322	10765329	181	12	10164	0.43694	0.33108
-	PTPRCAP	11	67425510	67472682	87	17	10164	0.4368	0.33113
+	CDC14A	1	100310042	100530277	520	40	10164	0.43656	0.33121
-	TMEM81	1	205073129	205119460	145	16	10164	0.43631	0.33131
+	RSPRY1	16	57151291	57250473	99	6	10164	0.43586	0.33147
+	NR5A2	1	199992602	200187424	554	60	10164	0.43575	0.33151
-	RPH3AL	17	202389	421254	181	36	10164	0.4356	0.33156
-	ANGPTL1	1	178839535	178906353	130	18	10164	0.43557	0.33158
-	FN1	2	215350454	215471167	275	24	10164	0.43519	0.33171
+	ICE1	5	5387673	5500234	326	33	10164	0.43516	0.33172
-	SLC22A24	11	63069255	63179221	403	15	10164	0.435	0.33178
+	HSPB1	7	76267558	76314297	96	14	10164	0.43471	0.33189
-	ZNF280B	22	22474421	22544187	89	4	10164	0.43461	0.33192
-	ST6GALNAC	17	76607774	76678838	272	24	10164	0.43408	0.33212
+	CERS4	19	8174329	8272421	363	55	10164	0.4338	0.33222
+	KDM6B	17	7799200	7864800	112	26	10164	0.43377	0.33223
+	OXNAD1	3	16230160	16396979	597	43	10164	0.43351	0.33232
-	SASS6	1	100073544	100168095	137	16	10164	0.4329	0.33254
+	CYP2W1	7	948156	999640	226	25	10164	0.43282	0.33257
+	RRP1B	21	43624551	43706079	139	8	10164	0.4325	0.33269
-	DSCR3	21	37213425	37303124	258	30	10164	0.43246	0.3327
+	ZFP41	8	143211821	143272705	216	19	10164	0.43248	0.3327
+	UBE2D2	5	139526166	139638434	106	7	10164	0.43239	0.33273
+	DAPL1	2	158760317	158837457	177	21	10164	0.43163	0.33301
+	CEP170B	14	104830313	104906770	247	16	10164	0.43151	0.33305
-	ZPBP	7	49840322	50128264	698	13	10164	0.43133	0.33311
+	SLC22A10	11	63233720	63321774	133	7	10164	0.43131	0.33312
+	TIGAR	12	4286193	4370028	244	41	10164	0.43105	0.33322
-	RAB3D	19	11312046	11374668	44	7	10164	0.43092	0.33326
+	ARTN	1	43896897	43947241	127	14	10164	0.43087	0.33328
-	TKTL2	4	163461095	163508895	127	25	10164	0.43083	0.3333
-	DNAH5	5	13680328	14046759	1369	80	10164	0.43037	0.33346
+	DYNAP	18	54497713	54609493	307	23	10164	0.43033	0.33348
+	SHD	19	4243601	4300723	168	17	10164	0.43018	0.33353
-	DBX2	12	45004756	45086099	249	25	10164	0.43009	0.33356
-	TOR1AIP2	1	179829967	179912806	173	15	10164	0.42995	0.33361
+	TRIM13	13	49962007	50028467	134	20	10164	0.42924	0.33388
-	C9	5	39274275	39399553	350	41	10164	0.42873	0.33406
+	PRPF4	9	113240634	113303914	163	16	10164	0.42858	0.33411
-	LMAN1	18	59317823	59394276	191	19	10164	0.42856	0.33412
+	CLRN2	4	17480165	17537104	202	23	10164	0.42857	0.33412
+	KCNRG	13	49980254	50030922	111	20	10164	0.42845	0.33416
+	RAB8A	19	16076680	16143635	108	9	10164	0.42838	0.33419
+	MAEL	1	166940582	167032214	138	8	10164	0.42815	0.33427
-	GOPC	6	117550269	117637542	220	25	10164	0.42792	0.33435
+	OR4C3	11	48289941	48335930	167	14	10164	0.42787	0.33437
-	PAQR6	1	156233321	156283488	132	19	10164	0.42775	0.33442
+	FAM177A1	14	35009907	35093383	260	22	10164	0.42757	0.33448
-	SLC44A4	6	31853192	31914046	292	68	10164	0.42754	0.33449
+	MT4	16	56530049	56578957	253	48	10164	0.4271	0.33465
-	JAK3	19	17814780	17883071	141	14	10164	0.42688	0.33473

STRAND	SYMBOL	CHR	START	STOP	NSNPS	NPARAM	N	ZSTAT	P
+	ZDHHC17	12	76729074	76863701	245	18	10164	0.42685	0.33474
+	PLA2G15	16	68210343	68271062	86	6	10164	0.42671	0.3348
+	KRTAP15-1	21	30405328	30450780	164	13	10164	0.4267	0.3348
+	KLK3	19	50819915	50887702	334	46	10164	0.42655	0.33485
+	FAM151B	5	80452981	80552563	232	31	10164	0.4264	0.33491
-	RPRML	17	46968156	47014248	168	11	10164	0.42581	0.33512
-	ZFP36L1	14	68777655	68831243	216	27	10164	0.4257	0.33516
-	PMP22	17	15219777	15300357	207	25	10164	0.42565	0.33518
-	GP5	3	194384821	194434266	15	2	10164	0.42554	0.33522
-	TRAPPC9	8	139718081	140493579	1908	147	10164	0.4253	0.33531
-	RRAGC	1	38828197	38894823	157	15	10164	0.42522	0.33534
+	RNF40	16	30726612	30812340	30	3	10164	0.42513	0.33537
-	HNRNPDL	4	82412564	82465225	101	11	10164	0.42499	0.33542
-	CCNL1	3	157136364	157195693	190	16	10164	0.42485	0.33547
-	ESRP2	16	68218547	68271233	76	6	10164	0.42485	0.33547
-	CYP2A7	19	40865439	40917752	72	12	10164	0.42474	0.33551
+	PCDHB4	5	141086750	141135623	119	8	10164	0.42474	0.33551
+	IGFN1	1	201155806	201238952	309	48	10164	0.42458	0.33557
+	EIF3M	11	32548767	32614498	147	17	10164	0.42455	0.33558
-	C1QTNF8	16	1078226	1131306	43	5	10164	0.42447	0.33561
-	SLC7A6OS	16	68290615	68345965	103	8	10164	0.42423	0.3357
-	SLC38A10	17	81234811	81330433	246	23	10164	0.42398	0.33579
+	MAT2B	5	163468064	163529353	171	21	10164	0.4239	0.33582
+	LPL	8	19904071	19977259	271	17	10164	0.42379	0.33586
+	ADAM9	8	38961965	39115261	275	18	10164	0.42359	0.33593
+	TSHB	1	114994824	115044309	142	8	10164	0.4235	0.33597
-	LOC1053756	7	156927417	156974681	283	27	10164	0.42347	0.33598
-	FOXN4	12	109267978	109345490	163	22	10164	0.42342	0.33599
+	STXBP3	1	108711663	108819526	298	16	10164	0.42323	0.33607
+	MTR	1	236759304	236913981	488	31	10164	0.42308	0.33612
-	CHADL	22	41199869	41276762	255	23	10164	0.42307	0.33612
+	ZKSCAN1	7	99980572	100055143	137	20	10164	0.42258	0.3363
-	LRP6	12	12106025	12302007	541	42	10164	0.42243	0.33635
+	TTC3	21	37038254	37213107	592	22	10164	0.42236	0.33638
+	NR2E3	15	71772197	71828666	118	19	10164	0.42237	0.33638
-	AFF4	5	132865379	132998662	340	17	10164	0.42233	0.33639
-	SLC22A16	6	110414685	110511641	371	25	10164	0.42216	0.33645
-	IRF2BP1	19	45873607	45921170	172	23	10164	0.42203	0.3365
+	SLC4A4	4	71152286	71582087	985	68	10164	0.4216	0.33666
-	LMAN2L	2	96695929	96775082	127	18	10164	0.42114	0.33683
+	CC2D1A	19	13871143	13940880	113	7	10164	0.4211	0.33684
-	TXNDC9	2	99309024	99371397	152	18	10164	0.42104	0.33686
+	PRR5L	11	36261175	36475204	816	106	10164	0.42101	0.33687
-	HDGF	1	156732107	156787448	115	14	10164	0.421	0.33688
+	TSPAN4	11	807824	877116	192	25	10164	0.42065	0.33701
-	OCM2	7	97974700	98025104	245	36	10164	0.42022	0.33716
+	GPI	19	34324592	34412413	121	11	10164	0.42019	0.33717
-	QARS	3	49085932	49140129	73	10	10164	0.41991	0.33728
+	YPEL2	17	59296692	59411734	272	33	10164	0.41977	0.33733
-	RGPD3	2	106381360	106503345	78	14	10164	0.41969	0.33736
-	KRTAP19-7	21	30551098	30596289	177	13	10164	0.41938	0.33747
+	ALDH3A2	17	19613136	19687596	93	8	10164	0.41912	0.33756
-	MRPS34	16	1761895	1808139	292	28	10164	0.41897	0.33762
+	ECHDC3	10	11707357	11774070	270	25	10164	0.41897	0.33762
-	MUC8	12	132461100	132509630	181	35	10164	0.41892	0.33764
-	CASP1	11	105015508	105071662	67	10	10164	0.41879	0.33768
-	SDK2	17	73324384	73679484	561	58	10164	0.41872	0.33771
+	RLF	1	40126369	40250921	345	11	10164	0.41861	0.33775
+	PCDHGA12	5	141395591	141522979	257	26	10164	0.41862	0.33775
-	PDZD9	16	21947540	22036127	75	14	10164	0.41853	0.33778
-	ATE1	10	121730421	121963801	1017	40	10164	0.41851	0.33779
-	EMC9	14	24128965	24176588	74	15	10164	0.41851	0.33779
+	BCAM	19	44774059	44831421	113	24	10164	0.41831	0.33786

STRAND	SYMBOL	CHR	START	STOP	NSNPS	NPARAM	N	ZSTAT	P
-	UBQLN3	11	5497300	5544985	216	26	10164	0.41785	0.33803
+	TMEM181	6	158501432	158645435	395	26	10164	0.41759	0.33812
+	PARM1	4	74898075	75060115	216	34	10164	0.41751	0.33815
+	FAT3	11	92279283	92906470	1005	47	10164	0.41748	0.33816
+	EPX	17	58157728	58215174	125	20	10164	0.41745	0.33817
+	DDR1	6	30845909	30910156	355	26	10164	0.41721	0.33826
+	RANBP2	2	108684446	108795811	185	13	10164	0.4171	0.3383
+	GAN	16	81279966	81390198	475	34	10164	0.41709	0.33831
-	SLC39A11	17	72635946	73127714	1630	178	10164	0.41708	0.33831
-	METTL16	17	2406049	2546906	138	15	10164	0.41694	0.33836
+	MICB	6	31459277	31521124	884	52	10164	0.41673	0.33844
-	SMARCD2	17	63822081	63877991	152	6	10164	0.41644	0.33854
+	PAPD7	5	6678953	6767048	331	22	10164	0.41562	0.33884
-	STXBP6	14	24799654	25085297	934	58	10164	0.41563	0.33884
+	MUC7	4	70395492	70492997	380	20	10164	0.4154	0.33892
+	TNFRSF6B	20	63661651	63708698	197	27	10164	0.41535	0.33894
+	LEPROTL1	8	30060406	30147706	170	13	10164	0.41532	0.33895
+	LOC1053772	3	71545821	71594038	56	9	10164	0.41526	0.33898
+	BARHL1	9	132547606	132600253	103	21	10164	0.41458	0.33923
-	RFWD2	1	175934826	176242279	546	21	10164	0.41455	0.33923
+	MAP1S	19	17684494	17744515	127	12	10164	0.4145	0.33925
-	WDR33	2	127694234	127846187	387	22	10164	0.41452	0.33925
+	ZNF618	9	113841282	114066595	568	47	10164	0.41443	0.33928
+	PCDHGB6	5	141373203	141522979	304	23	10164	0.4143	0.33933
-	OAT	10	124387303	124453976	180	21	10164	0.4142	0.33937
+	TCL1B	14	95651417	95702643	220	37	10164	0.4141	0.3394
+	EPHB1	3	134760257	135270467	1288	91	10164	0.41402	0.33943
+	PCDHGB5	5	141363128	141522979	319	21	10164	0.41393	0.33946
+	FAM69B	9	136677572	136734718	106	9	10164	0.41391	0.33947
-	PPP1R14B	11	64234479	64281941	141	29	10164	0.41364	0.33957
+	PCDHGA10	5	141378176	141522979	294	21	10164	0.41316	0.33975
-	QDPR	4	17476393	17547234	263	31	10164	0.41256	0.33996
+	PARP3	3	51907287	51958867	80	15	10164	0.41249	0.33999
-	EPHB4	7	100792565	100862521	182	26	10164	0.41244	0.34001
+	CFAP99	4	2383893	2472963	379	31	10164	0.41234	0.34005
+	LOC1053793	8	20370485	20423289	241	18	10164	0.41171	0.34028
+	LOC1053792	9	64797222	64845363	8	1	10164	0.41168	0.34029
+	ZNF263	16	3248380	3311401	76	12	10164	0.41154	0.34034
-	MTERF3	8	96229398	96296613	14	8	10164	0.41144	0.34037
-	LTK	15	41493637	41548887	144	18	10164	0.41136	0.3404
-	MRGPRX1	11	18923813	18970002	137	15	10164	0.41132	0.34042
-	CBX4	17	79823156	79874414	189	28	10164	0.4113	0.34043
-	HLA-DOB	6	32802763	32852048	546	65	10164	0.41119	0.34047
-	TMEM256	17	7392974	7439131	113	33	10164	0.41085	0.34059
-	DEFB124	20	31455506	31508013	47	10	10164	0.41066	0.34066
+	LAMC1	1	182988460	183155592	622	9	10164	0.41053	0.34071
-	GOLT1A	1	204188160	204249092	168	18	10164	0.41049	0.34072
-	NUP155	5	37281633	37406455	213	16	10164	0.41032	0.34079
-	HIST1H2BJ	6	27122316	27167796	121	36	10164	0.41019	0.34083
-	NRG4	15	75931973	76047444	128	32	10164	0.41018	0.34084
-	MMS19	10	97448324	97533794	187	21	10164	0.41011	0.34086
+	DMTN	8	22014149	22092527	233	34	10164	0.41007	0.34088
+	PPP1CB	2	28716748	28812940	173	17	10164	0.40983	0.34096
+	VHL	3	10106635	10163670	316	19	10164	0.40944	0.34111
+	PDS5B	13	32551427	32788020	646	23	10164	0.40941	0.34112
-	TOR2A	9	127721524	127771377	140	14	10164	0.40932	0.34115
-	THEG	19	352057	411703	16	3	10164	0.40928	0.34117
-	HGD	3	120618168	120717571	193	27	10164	0.40909	0.34124
+	LOC1053752	7	48812489	48861619	185	27	10164	0.40901	0.34127
+	SLC17A9	20	62917647	62979585	151	19	10164	0.40881	0.34134
-	LOC1053790	5	83497040	83597372	358	44	10164	0.40872	0.34137
+	GRM3	7	86608914	86874879	552	44	10164	0.40868	0.34139
+	PRKACB	1	84042975	84248498	391	26	10164	0.40863	0.3414

STRAND	SYMBOL	CHR	START	STOP	NSNPS	NPARAM	N	ZSTAT	P
-	COX16	14	70315081	70394731	244	16	10164	0.40853	0.34144
+	USP29	19	57085141	57141926	218	24	10164	0.40819	0.34157
-	UQCRB	8	96216676	96270634	7	4	10164	0.40816	0.34158
+	TMEM70	8	73941142	73992783	152	14	10164	0.40807	0.34161
+	AIP	11	67448029	67501108	87	23	10164	0.40764	0.34177
-	ST6GALNAC	9	127875321	127941731	134	23	10164	0.40739	0.34186
-	CSH2	17	63862012	63908729	140	8	10164	0.40732	0.34189
-	ARGLU1	13	106533314	106603166	23	4	10164	0.40731	0.34189
+	ZBTB43	9	126769072	126848210	117	11	10164	0.40728	0.3419
+	COLEC11	2	3559832	3654644	329	29	10164	0.40688	0.34205
-	RPS6KL1	14	74892849	74958282	112	11	10164	0.40658	0.34216
-	PDGFRB	5	150103839	150190884	282	49	10164	0.40645	0.34221
+	SERPIND1	22	20739095	20797720	8	3	10164	0.40629	0.34227
+	SCAMP1	5	78325503	78490739	421	15	10164	0.40625	0.34228
+	TCF7	5	134079703	134158229	171	23	10164	0.40579	0.34245
-	LEO1	15	51928025	52006801	295	23	10164	0.40547	0.34257
-	TIMD4	5	156909282	157007310	246	25	10164	0.40538	0.3426
+	SIRPA	20	1859117	1949896	92	17	10164	0.40539	0.3426
-	IFNGR1	6	137187484	137254844	135	26	10164	0.40534	0.34261
-	KLK11	19	51012231	51063034	239	48	10164	0.40534	0.34261
-	SALL1	16	51125975	51187316	101	20	10164	0.40519	0.34267
+	PCDHGA9	5	141367953	141522979	314	20	10164	0.40517	0.34268
+	NUP85	17	75170450	75245759	192	16	10164	0.40511	0.3427
-	TMEM134	11	67454348	67504277	80	23	10164	0.40501	0.34273
+	LRGUK	7	134092352	134274181	661	20	10164	0.40499	0.34274
+	MUC21	6	30948708	30998637	356	34	10164	0.40484	0.3428
-	ZBTB39	12	56988833	57041513	98	9	10164	0.40477	0.34282
-	VEGFC	4	176673534	176827745	203	14	10164	0.40457	0.3429
+	MBD6	12	57485710	57540148	106	11	10164	0.40421	0.34303
-	SLC7A5	16	87820023	87904497	370	26	10164	0.40412	0.34306
+	H3F3A	1	226027707	226082002	132	20	10164	0.40369	0.34322
+	CMC1	3	28206589	28329776	249	20	10164	0.40368	0.34322
+	HOXD13	2	176052505	176105938	220	43	10164	0.4036	0.34325
+	SDCCAG8	1	243220419	243510091	483	38	10164	0.4031	0.34344
-	PFN3	5	177390107	177435636	40	11	10164	0.40285	0.34353
+	MUS81	11	65825244	65876443	143	26	10164	0.40282	0.34354
+	STATH	4	69960931	70012456	119	15	10164	0.40279	0.34355
-	FANCC	9	95089054	95352709	663	33	10164	0.40273	0.34357
-	IFNL4	19	39236314	39283856	95	12	10164	0.40274	0.34357
+	HBQ1	16	145334	191179	106	20	10164	0.4026	0.34362
+	RPL31	2	100967229	101029693	219	36	10164	0.40254	0.34364
-	ERICH6	3	150649882	150739563	144	14	10164	0.40245	0.34368
+	NPIPA3	16	14671473	14736338	2	1	10164	0.40242	0.34369
-	CD70	19	6575839	6626152	141	13	10164	0.4022	0.34377
-	THRB	3	24107153	24530281	1316	149	10164	0.40166	0.34397
-	MTL5	11	68696674	68786564	242	32	10164	0.4016	0.34399
-	LOC1053781	6	163701976	163749377	163	14	10164	0.40156	0.344
-	GH2	17	63870212	63916942	122	8	10164	0.4015	0.34403
+	EIF4EBP1	8	37995502	38070365	115	16	10164	0.40136	0.34408
-	CBWD1	9	111038	214075	149	14	10164	0.40135	0.34408
+	ZMYND11	10	99465	264637	190	19	10164	0.40103	0.3442
+	AVL9	7	32460426	32598741	481	20	10164	0.40093	0.34423
+	GPX8	5	55125156	55177301	109	9	10164	0.40082	0.34428
-	PEX1	7	92477023	92563531	194	24	10164	0.40071	0.34432
+	LIPK	10	88671234	88763092	310	18	10164	0.40045	0.34441
+	HAUS1	18	46069332	46138333	247	17	10164	0.40013	0.34453
-	GIP	17	48948556	49003593	161	21	10164	0.39997	0.34459
-	KRT81	12	52275913	52343410	261	22	10164	0.39987	0.34463
+	NR1H3	11	47213300	47279033	157	14	10164	0.39946	0.34478
-	DRD2	11	113399595	113510279	358	40	10164	0.3991	0.34491
-	UBAP1L	15	65082760	65141647	61	9	10164	0.39909	0.34491
-	LONRF2	2	100263291	100357733	444	24	10164	0.39897	0.34496
-	EFNA5	5	107366889	107705895	740	79	10164	0.39882	0.34501

STRAND	SYMBOL	CHR	START	STOP	NSNPS	NPARAM	N	ZSTAT	P
-	SCO1	17	10670332	10732568	163	8	10164	0.39877	0.34503
-	CDH8	16	61637244	62072035	995	59	10164	0.39862	0.34509
-	ZNF500	16	4735987	4802218	132	8	10164	0.39843	0.34515
+	NAT1	8	18135462	18233689	482	41	10164	0.39842	0.34516
+	FGF5	4	80231588	80301017	150	25	10164	0.39814	0.34526
-	FAM83C	20	35275731	35327422	128	19	10164	0.39762	0.34546
-	DHRS13	17	28887781	28938071	50	13	10164	0.39756	0.34548
+	HNRNPA1	12	54245696	54297087	79	9	10164	0.39753	0.34549
+	OR51M1	11	5354377	5400434	432	16	10164	0.39725	0.34559
+	IDH3A	15	78114362	78180542	213	34	10164	0.39715	0.34563
+	XRN2	20	21268304	21399825	260	18	10164	0.39712	0.34564
-	TAF12	1	28593096	28678109	90	10	10164	0.3969	0.34572
+	ZNF700	19	11890068	11960773	111	8	10164	0.39682	0.34575
+	JAGN1	3	9855587	9904349	144	29	10164	0.39668	0.3458
-	TINF2	14	24224403	24278027	89	18	10164	0.39661	0.34583
+	MS4A6E	11	60299882	60350968	244	14	10164	0.3965	0.34587
+	EML6	2	54688670	54982908	1187	89	10164	0.39639	0.34591
+	CD101	1	116966750	117046551	168	18	10164	0.39625	0.34596
+	RNPEP	1	201947638	202016147	276	40	10164	0.39614	0.346
+	ISM1	20	13186771	13310650	397	47	10164	0.39609	0.34602
-	ST8SIA4	5	100796935	100938285	310	18	10164	0.39596	0.34607
+	WNK1	12	717593	921452	361	27	10164	0.39559	0.3462
+	HOXD11	2	176072356	176119588	161	39	10164	0.39536	0.34629
-	ZFHX3	16	72772885	73093635	956	97	10164	0.39519	0.34635
-	C12orf49	12	116703320	116773070	180	28	10164	0.39521	0.34635
-	NFRKB	11	129853773	129930595	237	30	10164	0.39508	0.34639
+	TARS	5	33405696	33478091	187	17	10164	0.39488	0.34647
+	SAC3D1	11	65005904	65054828	116	14	10164	0.3945	0.3466
+	ANAPC16	10	72181000	72245860	190	7	10164	0.39434	0.34666
+	TMEM79	1	156247913	156302443	142	17	10164	0.39392	0.34682
-	ADAMTS17	15	99961438	100377087	2254	158	10164	0.39365	0.34692
+	ERBB2	17	39653084	39738662	146	12	10164	0.39355	0.34695
+	C4orf33	4	129058674	129122688	250	22	10164	0.39334	0.34704
+	FOS	14	75243778	75292234	102	20	10164	0.39318	0.34709
-	KLK13	19	51046206	51100110	230	62	10164	0.39308	0.34713
+	MTHFD1L	6	150830679	151111887	971	72	10164	0.39298	0.34717
+	FNDC8	17	35086612	35140732	134	27	10164	0.39284	0.34722
+	NT5DC1	6	116065823	116255690	658	30	10164	0.39284	0.34722
+	HSD11B2	16	67396133	67447553	54	11	10164	0.39258	0.34731
+	POLR2F	22	37918663	38051915	165	20	10164	0.39245	0.34736
+	SPNS1	16	28939771	28994762	98	11	10164	0.39246	0.34736
-	CFAP43	10	104119888	104267366	338	25	10164	0.39211	0.34749
+	LOC1053730	22	49746805	49793435	138	20	10164	0.39171	0.34764
+	ERCC4	16	13885145	13962348	103	3	10164	0.39137	0.34776
-	CLLU1OS	12	92410094	92463545	94	13	10164	0.39125	0.34781
+	OR10G4	11	123980575	124026510	240	23	10164	0.39112	0.34786
+	TRIM22	11	5654587	5720863	307	26	10164	0.39094	0.34792
-	F3	1	94519176	94576857	188	33	10164	0.39078	0.34798
+	C5AR2	19	47296841	47352080	78	10	10164	0.39058	0.34805
+	SLAIN2	4	48306480	48436201	408	27	10164	0.39055	0.34807
-	GPX6	6	28493296	28550793	254	34	10164	0.39037	0.34813
-	ATXN7L1	7	105594774	105912232	786	99	10164	0.39033	0.34815
-	CRTC2	1	153937672	153993656	61	10	10164	0.39024	0.34818
+	ZNF619	3	40442113	40500237	139	11	10164	0.3901	0.34823
-	RIC3	11	8082965	8204083	349	18	10164	0.38998	0.34828
+	LOC1053724	19	58270337	58325653	117	15	10164	0.38996	0.34828
-	RGPD8	2	112358369	112468645	32	10	10164	0.38991	0.3483
-	COLQ	3	15440133	15556751	393	24	10164	0.38981	0.34834
+	PCDHB14	5	141187326	141236288	131	16	10164	0.3898	0.34834
-	GNA14	9	77413079	77683316	627	86	10164	0.38974	0.34836
-	DZIP1L	3	138051985	138150818	22	5	10164	0.38969	0.34838
-	GPR152	11	67441301	67487729	78	19	10164	0.38966	0.34839
-	ZHX1	8	123238450	123310541	37	19	10164	0.38963	0.34841

STRAND	SYMBOL	CHR	START	STOP	NSNPS	NPARAM	N	ZSTAT	P
+	SLC16A3	17	82193406	82249499	113	5	10164	0.3893	0.34853
-	ZBED9	6	28561338	28651203	317	43	10164	0.3893	0.34853
-	ZDHHC1	16	67384419	67451436	69	14	10164	0.38917	0.34858
-	MAS1L	6	29476766	29522902	282	45	10164	0.38916	0.34858
-	KNOP1	16	19696347	19753361	110	7	10164	0.38913	0.34859
-	PSG2	19	43054210	43117741	303	14	10164	0.38897	0.34865
+	HOXD10	2	176081764	176129942	166	45	10164	0.38891	0.34867
+	CNN2	19	991275	1049068	345	32	10164	0.38889	0.34868
-	PROX2	14	74842806	74911419	141	13	10164	0.3888	0.34871
-	RASGEF1A	10	43184533	43301919	339	26	10164	0.38876	0.34873
-	SYNE1	6	152111684	152672399	2133	165	10164	0.38859	0.34879
-	LINGO3	19	2279775	2343157	196	27	10164	0.38847	0.34883
+	ATF5	19	49893702	49943936	159	25	10164	0.38839	0.34886
-	LCA5L	21	39395844	39480793	243	20	10164	0.38827	0.34891
+	LOC1053734	1	210258123	210331710	216	31	10164	0.38805	0.34899
-	C19orf57	19	13872348	13941099	112	7	10164	0.38761	0.34915
+	MAN1A2	1	117332393	117538872	377	18	10164	0.3875	0.34919
-	LOC1019284	15	52790166	52841418	159	21	10164	0.38745	0.34921
-	ATP5A1	18	46074144	46139233	229	17	10164	0.38739	0.34923
+	KLHL40	3	42650519	42702446	137	30	10164	0.38732	0.34926
-	IGF2	11	2119112	2184603	222	35	10164	0.38723	0.34929
-	C2CD4D	1	151827818	151880329	139	21	10164	0.38695	0.3494
+	POGLUT1	3	119433938	119504708	279	33	10164	0.3868	0.34945
-	METTL6	3	15371063	15462547	278	27	10164	0.38654	0.34955
+	CUL1	7	148663178	148811110	179	12	10164	0.38651	0.34956
-	WFDC12	20	45113426	45159465	130	13	10164	0.3865	0.34956
+	NCOA3	20	47466857	47666877	471	22	10164	0.38646	0.34958
-	KLHL6	3	183477531	183590712	281	33	10164	0.38639	0.3496
+	THSD7B	2	136730562	137687717	3837	172	10164	0.38638	0.34961
-	TMEM243	7	87186160	87255573	195	21	10164	0.38609	0.34971
-	HAUS3	4	2221836	2277164	133	13	10164	0.38604	0.34974
-	DEPDC4	12	100193669	100302172	175	13	10164	0.38558	0.3499
-	SLAMF9	1	159941492	159989254	73	15	10164	0.38536	0.34999
-	C1QL3	10	16503743	16557005	164	21	10164	0.38533	0.35
-	TMEM173	5	139465528	139517790	51	13	10164	0.38517	0.35005
-	ARL15	5	53874748	54345586	1285	82	10164	0.38504	0.3501
+	AQP1	7	30876800	30935516	116	25	10164	0.38489	0.35016
+	USPL1	13	30582693	30669557	114	9	10164	0.38458	0.35027
-	PRAME	22	22537696	22594361	1	1	10164	0.38441	0.35034
-	MROH2B	5	40988020	41106342	578	48	10164	0.38434	0.35036
-	TFDP2	3	141934428	142184544	821	20	10164	0.38432	0.35037
-	BDNF	11	27644893	27757058	206	30	10164	0.38418	0.35042
+	NUCB2	11	17225337	17359980	310	24	10164	0.38415	0.35043
-	ITGB1BP1	2	9395684	9458569	125	9	10164	0.3841	0.35045
-	FTSJ3	17	63809433	63862671	110	5	10164	0.38389	0.35053
+	ZNF710	15	89964046	90092200	185	31	10164	0.3839	0.35053
-	KRTAP10-2	21	44540357	44586505	188	19	10164	0.38381	0.35056
-	AMMECR1L	2	127851630	127923570	178	21	10164	0.38359	0.35064
+	LCE4A	1	152674047	152719434	134	10	10164	0.38331	0.35074
+	OSR2	8	98903104	98962110	66	10	10164	0.38308	0.35083
-	RERGL	12	18070869	18125193	159	17	10164	0.38266	0.35099
+	ZNF226	19	44130062	44187685	107	12	10164	0.38256	0.35102
+	SPSB4	3	141015795	141158611	317	32	10164	0.38254	0.35103
+	BYSL	6	41872847	41943046	105	18	10164	0.38245	0.35106
+	SPINT1	15	40809018	40867655	196	32	10164	0.38225	0.35114
-	LPPR5	1	98880245	99040809	498	42	10164	0.38221	0.35115
+	NBEAL1	2	202979617	203236353	412	24	10164	0.38218	0.35116
+	KCTD2	17	74997567	75075889	137	14	10164	0.38217	0.35117
-	LAMB2	3	49111114	49168166	87	10	10164	0.38208	0.3512
+	FAM98B	15	38419127	38497710	151	17	10164	0.38202	0.35122
+	UQCRC2	16	21918288	21993347	58	12	10164	0.38178	0.35131
+	CPB1	3	148756180	148870192	293	29	10164	0.38157	0.35139
+	PANX1	11	94093928	94195596	292	33	10164	0.38145	0.35143

STRAND	SYMBOL	CHR	START	STOP	NSNPS	NPARAM	N	ZSTAT	P
-	THBS2	6	169205780	169289114	321	43	10164	0.38109	0.35157
-	IFNA4	9	21176618	21222599	255	18	10164	0.3809	0.35164
-	ADAT1	16	75588349	75658323	234	24	10164	0.38083	0.35166
-	LOC1053751	6	20390748	20437772	118	32	10164	0.38082	0.35167
-	LOC1001293	13	77651374	77697803	145	22	10164	0.38079	0.35168
+	CEACAM3	19	41761590	41821553	96	7	10164	0.38077	0.35169
-	MEFV	16	3232028	3291627	112	16	10164	0.38067	0.35172
+	TMEM88	17	7819896	7866099	76	15	10164	0.38066	0.35173
-	MAPK10	4	86002299	86629110	1824	67	10164	0.3806	0.35175
+	TCTEX1D1	1	66717457	66789047	181	18	10164	0.38046	0.3518
-	CLCN7	16	1434933	1510084	352	43	10164	0.38043	0.35181
+	TCHP	12	109845680	109928069	197	20	10164	0.38028	0.35187
-	IQCA1L	7	151180873	151240519	99	15	10164	0.38027	0.35187
+	PLA2G4A	1	186793900	186998981	517	50	10164	0.38009	0.35194
+	CNOT11	2	101217883	101280316	88	12	10164	0.37999	0.35198
-	ZNF891	12	133109969	133165473	105	7	10164	0.37999	0.35198
+	C1orf68	1	152684522	152730429	170	12	10164	0.37991	0.35201
+	ARMC5	16	31423273	31477167	45	8	10164	0.37986	0.35202
+	TINAG	6	54272792	54400152	514	42	10164	0.37981	0.35204
+	DNAH9	17	11563431	11980168	960	93	10164	0.37978	0.35205
+	SFT2D2	1	168191017	168252850	65	8	10164	0.3798	0.35205
-	NAP1L5	4	88685915	88732872	111	14	10164	0.37954	0.35214
+	RPL18A	19	17824878	17873324	79	7	10164	0.37926	0.35225
+	ZNF568	19	36881329	37007932	557	24	10164	0.37909	0.35231
+	PPP1R14C	6	150108052	150260392	516	60	10164	0.37907	0.35232
+	DDI2	1	15582458	15671057	321	12	10164	0.37904	0.35233
+	TMEM120B	12	121677752	121792924	281	40	10164	0.37898	0.35235
+	SOAT1	1	179258714	179368680	500	31	10164	0.37892	0.35238
+	ARHGAP25	2	68675505	68836833	634	50	10164	0.37871	0.35245
-	MARCH10	17	62691314	62843381	308	23	10164	0.37869	0.35246
-	HSPA12A	10	116661192	116885814	553	59	10164	0.37861	0.35249
+	CASP10	2	201147898	201239406	217	44	10164	0.37852	0.35252
+	SOWAHA	5	132778324	132826797	91	19	10164	0.37846	0.35254
+	PCDHGA8	5	141356916	141522979	334	24	10164	0.37839	0.35257
+	ASB1	2	238391741	238462250	268	26	10164	0.37819	0.35264
+	AOX1	2	200550952	200692241	452	45	10164	0.37804	0.3527
+	HOXC8	12	53974106	54022763	54	11	10164	0.37789	0.35276
-	MSTN	2	190045700	190097729	125	14	10164	0.3776	0.35286
-	CCDC186	10	114110856	114209605	127	13	10164	0.37753	0.35289
+	KCNJ18	17	21657523	21715700	1	1	10164	0.37749	0.35291
+	DBH	9	133601363	133669344	184	33	10164	0.37711	0.35305
+	PSMC5	17	63792410	63842027	81	5	10164	0.3771	0.35305
-	LILRB3	19	54206278	54258087	56	9	10164	0.37683	0.35315
-	IQCF6	3	51768561	51814773	48	16	10164	0.37681	0.35316
+	RPL38	17	74168656	74219880	50	14	10164	0.37673	0.35319
-	RPL35	9	124847879	124896961	147	20	10164	0.37658	0.35324
+	HSPA14	10	14803160	14881741	195	33	10164	0.37624	0.35337
-	ZNF250	8	144866951	144937305	119	13	10164	0.37593	0.35349
+	C1orf105	1	172385515	172478829	168	17	10164	0.37575	0.35355
+	LCE2A	1	152663364	152709442	118	8	10164	0.37574	0.35356
-	TMEM89	3	48610842	48656756	58	8	10164	0.37534	0.3537
+	MS4A12	11	60457742	60517430	238	18	10164	0.37519	0.35376
-	C16orf58	16	31479469	31543419	151	11	10164	0.37501	0.35383
+	OR4X2	11	48210104	48256015	178	13	10164	0.375	0.35383
-	LOC1053702	13	44360118	44409008	100	17	10164	0.37493	0.35386
-	AMBRA1	11	46386412	46629069	207	20	10164	0.37467	0.35395
+	BTBD3	20	11855723	11936595	199	13	10164	0.37428	0.3541
+	DCAF10	9	37765554	37877669	300	11	10164	0.37402	0.35419
+	NEIL1	15	75311649	75365251	143	19	10164	0.3735	0.35439
-	RNF39	6	30060266	30110851	492	31	10164	0.37347	0.3544
-	PSG7	19	42914132	42972178	214	9	10164	0.37312	0.35453
-	TLL2	10	96354606	96548926	491	48	10164	0.37305	0.35455
+	GOLGA6L9	15	82382678	82449387	103	17	10164	0.37302	0.35457

STRAND	SYMBOL	CHR	START	STOP	NSNPS	NPARAM	N	ZSTAT	P
-	HOXA6	7	27135583	27182774	94	18	10164	0.37267	0.3547
-	LOC645202	15	28339390	28393086	1	1	10164	0.37259	0.35473
-	NCEH1	3	172620645	172746218	197	14	10164	0.37256	0.35474
+	HLA-F	6	29688340	29750355	837	32	10164	0.37233	0.35482
+	SF3B2	11	66016775	66078911	183	26	10164	0.37207	0.35492
+	CYP2S1	19	41158207	41217539	92	16	10164	0.37208	0.35492
+	ATP11B	3	182758503	182931635	379	19	10164	0.37137	0.35518
-	FLG2	1	152338735	152395010	119	10	10164	0.37129	0.35521
-	MS4A6A	11	60161607	60219666	143	13	10164	0.37119	0.35525
+	GMPR	6	16203580	16305549	368	32	10164	0.37114	0.35527
+	PCDHGA7	5	141347739	141522979	350	22	10164	0.37101	0.35531
+	MLLT6	17	38670620	38739803	81	17	10164	0.37055	0.35548
-	CEACAM20	19	44494833	44564945	213	38	10164	0.37027	0.35559
+	PCDHGB4	5	141352885	141522979	343	22	10164	0.37003	0.35568
-	PPP1R14D	15	40805445	40863709	201	33	10164	0.37001	0.35569
+	ARL4D	17	43363985	43411136	96	16	10164	0.36971	0.3558
-	PYY	17	43942733	44059260	204	22	10164	0.36972	0.3558
-	LUC7L	16	178971	264450	217	25	10164	0.36955	0.35586
+	PLEKHG2	19	39377582	39438415	70	12	10164	0.36953	0.35587
+	BSG	19	536277	593493	228	29	10164	0.36947	0.35589
-	NBPF14	1	148521385	148630947	1	1	10164	0.36941	0.35591
+	THOC6	16	2989031	3037755	22	3	10164	0.36941	0.35591
+	SLC22A9	11	63334789	63420259	170	15	10164	0.36913	0.35601
-	FAM65C	20	50576108	50727004	244	37	10164	0.36869	0.35618
-	ALDH1L2	12	105009784	105119583	410	35	10164	0.36834	0.35631
+	MC1R	16	89882879	89930977	175	13	10164	0.36831	0.35632
-	LGSN	6	63265951	63602921	821	30	10164	0.36803	0.35643
-	ERN2	16	23680304	23748500	142	26	10164	0.36787	0.35649
+	SFT2D3	2	127666023	127713833	138	16	10164	0.36772	0.35654
+	KIAA1024L	5	129713191	129775063	228	30	10164	0.36759	0.35659
+	CYP4F8	19	15580218	15641867	426	15	10164	0.36755	0.3566
-	CPB2	13	46043187	46140076	233	18	10164	0.36731	0.35669
+	ROPN1B	3	125934144	125993454	133	15	10164	0.36731	0.3567
-	CP	3	149152410	149257050	275	39	10164	0.36714	0.35676
+	SLC22A11	11	64520626	64582875	157	20	10164	0.36684	0.35687
+	SUV39H2	10	14843783	14914315	140	28	10164	0.36685	0.35687
+	DYSF	2	71418119	71696763	654	74	10164	0.36678	0.35689
-	CD5L	1	157817071	157876844	158	19	10164	0.36671	0.35692
-	SKP1	5	134146391	134212033	142	11	10164	0.36671	0.35692
+	MC3R	20	56213732	56259815	178	19	10164	0.3665	0.357
+	TMEM104	17	74741483	74849783	220	24	10164	0.36643	0.35702
+	MINK1	17	4798340	4908061	329	27	10164	0.36624	0.35709
-	SLC30A10	1	219903919	219994315	280	20	10164	0.36618	0.35712
+	GLI1	12	57425135	57482268	124	24	10164	0.36606	0.35716
+	NLRP14	11	6985446	7099025	315	13	10164	0.36503	0.35754
-	ITGAE	17	3704625	3836243	284	25	10164	0.36502	0.35755
-	BTBD2	19	1975438	2050703	292	45	10164	0.36419	0.35786
+	CCDC105	19	14975727	15033271	341	35	10164	0.36408	0.3579
+	HOXC6	12	53981852	54040823	79	14	10164	0.36382	0.358
-	ZFP82	19	36380332	36453658	179	27	10164	0.36368	0.35805
-	TACSTD2	1	58565423	58612494	217	23	10164	0.36356	0.35809
+	LOC1053743	3	195960072	196015093	130	17	10164	0.36358	0.35809
+	FBXO22	15	75868859	75945268	104	27	10164	0.36346	0.35813
-	TMEM71	8	132699945	132795712	269	22	10164	0.36334	0.35817
+	NEK7	1	198121978	198332420	624	25	10164	0.36326	0.3582
-	EBLN1	10	22198814	22244983	109	12	10164	0.36299	0.35831
-	EMCN	4	100385341	100553093	421	25	10164	0.36236	0.35854
+	CCDC88B	11	64305197	64367534	213	35	10164	0.3621	0.35864
-	FBXL3	13	76991339	77062196	177	18	10164	0.36205	0.35866
-	TRIL	7	28943358	28993413	173	19	10164	0.36198	0.35868
-	SCN5A	3	38538058	38684673	368	49	10164	0.36192	0.35871
-	CDH17	8	94117162	94252303	382	38	10164	0.36187	0.35872
-	LOC1053734	1	178528473	178577606	169	10	10164	0.36187	0.35872

STRAND	SYMBOL	CHR	START	STOP	NSNPS	NPARAM	N	ZSTAT	P
+	COX8A	11	63939607	63986543	124	13	10164	0.36176	0.35876
-	LOC1053766	13	94702307	94748712	99	12	10164	0.36174	0.35877
-	RPS25	11	119005712	119053347	158	22	10164	0.36171	0.35878
-	CX3CR1	3	39253494	39316735	193	33	10164	0.36158	0.35883
+	ZNF264	19	57156479	57232846	224	22	10164	0.36157	0.35884
+	CLDN20	6	155229013	155286548	196	17	10164	0.3615	0.35886
+	UBASH3B	11	122620690	122824479	591	64	10164	0.36105	0.35903
+	MARCH5	10	92256163	92363964	162	16	10164	0.36097	0.35906
-	NOTCH3	19	15149633	15235981	168	14	10164	0.36087	0.3591
-	RBP1	3	139507434	139574829	200	28	10164	0.36079	0.35913
+	TTC12	11	113279529	113395952	309	30	10164	0.36068	0.35917
-	ZBTB20	3	114304500	115182280	1627	106	10164	0.35996	0.35944
+	LAYN	11	111503985	111571745	91	19	10164	0.35994	0.35945
-	CSRNP1	3	39131851	39189597	138	22	10164	0.3597	0.35954
+	KIAA0556	16	27515119	27790371	266	27	10164	0.3596	0.35957
+	LRWD1	7	102429943	102483168	110	13	10164	0.3596	0.35957
+	DDB2	11	47179942	47249218	152	15	10164	0.35957	0.35958
+	ADGRF4	6	47663553	47732021	261	9	10164	0.35952	0.3596
+	ODF2	9	128420155	128511292	190	11	10164	0.35925	0.35971
+	OR10C1	6	29404939	29450977	487	23	10164	0.35913	0.35975
-	TBX5	12	114343930	114443442	344	42	10164	0.35907	0.35977
+	PCDHGA6	5	141339084	141522979	363	24	10164	0.35889	0.35984
-	TCTEX1D2	3	196281219	196353294	185	14	10164	0.35875	0.35989
+	EGR1	5	138430492	138479315	76	10	10164	0.35868	0.35992
-	RNF183	9	113287093	113338274	120	14	10164	0.35863	0.35993
-	GSTA2	6	52740087	52798563	164	9	10164	0.35858	0.35996
+	NR1I2	3	119745484	119828485	220	36	10164	0.35856	0.35996
+	UMAD1	7	7605636	7889223	1137	83	10164	0.35825	0.36008
-	FAM186A	12	50316852	50431635	210	18	10164	0.35803	0.36016
+	FAM193A	4	2561288	2742575	418	28	10164	0.35795	0.36019
-	SGMS1	10	50295585	50658977	993	38	10164	0.35794	0.36019
+	LOC1053771	3	37140864	37206722	105	10	10164	0.35793	0.3602
+	TERF2IP	16	75612704	75667442	184	21	10164	0.35756	0.36034
-	TBC1D27	17	16912923	16964592	245	30	10164	0.35753	0.36035
+	MFSD2A	1	39920112	39979968	119	16	10164	0.35686	0.3606
+	RARA	17	40274171	40367643	43	6	10164	0.35678	0.36063
-	TMEM186	16	8785180	8832648	176	18	10164	0.35673	0.36065
-	NMUR1	2	231509907	231565471	221	20	10164	0.35663	0.36069
-	ELL	19	18432663	18557129	214	11	10164	0.35653	0.36072
+	VWC2	7	49738661	49931950	388	38	10164	0.35617	0.36086
-	ZNF28	19	52787408	52856678	131	13	10164	0.35594	0.36094
+	TMEM97	17	28284095	28338685	68	19	10164	0.35585	0.36098
-	CBX7	22	39120772	39187662	197	23	10164	0.35571	0.36103
+	SSTR5	16	1037756	1091454	26	5	10164	0.35568	0.36104
-	TCP10L	21	32564841	32620535	173	25	10164	0.35568	0.36104
-	TCEB1	8	73935138	74007287	222	19	10164	0.35564	0.36105
-	NCOA5	20	46050985	46124952	161	21	10164	0.35513	0.36125
-	RIPK3	14	24326021	24375036	160	17	10164	0.35507	0.36127
-	KCTD4	13	45182853	45246102	113	11	10164	0.35506	0.36127
-	ZNF781	19	37657749	37727315	191	22	10164	0.35504	0.36128
+	H1FOO	3	129508214	129561467	136	24	10164	0.35491	0.36133
+	ARHGAP40	20	38566934	38660652	91	15	10164	0.35416	0.36161
+	GPC6	13	93191825	94418020	3634	210	10164	0.35414	0.36162
-	CTSK	1	150786208	150843441	75	11	10164	0.354	0.36167
-	KCNG1	20	50993656	51058176	125	20	10164	0.35397	0.36168
+	AHR	7	17263652	17356151	153	37	10164	0.35359	0.36182
+	CD28	2	203671475	203748912	163	24	10164	0.35358	0.36183
+	SUPV3L1	10	69145204	69219098	206	21	10164	0.35351	0.36185
+	IRF9	14	24126213	24176565	75	15	10164	0.35337	0.36191
-	COL5A2	2	189021915	189260314	672	26	10164	0.35302	0.36204
-	PCMTD1	8	51807575	51934186	447	18	10164	0.353	0.36205
+	RGL1	1	183601047	183938551	1164	41	10164	0.35294	0.36207
+	REEP2	5	138404001	138456969	116	13	10164	0.35282	0.36211

STRAND	SYMBOL	CHR	START	STOP	NSNPS	NPARAM	N	ZSTAT	P
-	KRTAP6-2	21	30588686	30633874	140	14	10164	0.35266	0.36217
-	ZFP64	20	52074011	52226985	516	38	10164	0.35264	0.36218
+	ZFP28	19	56502716	56566810	188	18	10164	0.35256	0.36221
+	ITIH1	3	52742586	52802068	138	15	10164	0.35201	0.36241
+	HOXD12	2	176063513	176113770	192	43	10164	0.35171	0.36253
-	GATAD2B	1	153794725	153957975	280	21	10164	0.35164	0.36256
-	UGT2B4	4	69470165	69561014	563	13	10164	0.35139	0.36265
+	NAPG	18	10490876	10562769	294	17	10164	0.35117	0.36273
+	KLHDC7A	1	18445930	18495999	201	27	10164	0.35118	0.36273
+	C17orf74	17	7389881	7437568	114	32	10164	0.35101	0.36279
-	UBOX5	20	3097573	3194910	120	17	10164	0.35098	0.3628
-	ZNF350	19	51954340	52021856	206	17	10164	0.35098	0.3628
+	RSPO3	6	127083903	127209481	238	18	10164	0.35092	0.36282
-	BARHL2	1	90702022	90752237	70	11	10164	0.35079	0.36287
+	MDK	11	46345784	46393837	54	11	10164	0.35073	0.3629
-	GPR132	14	105039389	105100550	189	18	10164	0.35072	0.3629
-	KDM2B	12	121392501	121616113	494	41	10164	0.3504	0.36302
-	NUDCD2	5	163443488	163495137	126	20	10164	0.3504	0.36302
+	IGF2R	6	159934099	160116551	633	73	10164	0.35013	0.36312
-	RASGRP1	15	38478101	38599806	164	28	10164	0.35008	0.36314
-	EXOC5	14	57192476	57303899	161	8	10164	0.35007	0.36314
+	TRMT10B	9	37718204	37788972	191	26	10164	0.34972	0.36327
-	SECTM1	17	82311024	82369045	74	4	10164	0.3495	0.36336
+	GPR89B	1	147893393	148003521	122	13	10164	0.34864	0.36368
-	LOC1053705	14	68791294	68863141	278	38	10164	0.34862	0.36369
-	VCPKMT	14	50098632	50151579	121	11	10164	0.34827	0.36382
+	GNAZ	22	23035483	23135034	158	11	10164	0.34824	0.36383
-	CDC20B	5	55102971	55208177	258	16	10164	0.34817	0.36386
-	FOXL2	3	138934224	138982140	118	13	10164	0.34813	0.36387
-	WFDC3	20	45764208	45826908	210	23	10164	0.3479	0.36396
+	STBD1	4	76271026	76321130	170	25	10164	0.34748	0.36411
-	BCL11A	2	60441167	60588498	229	49	10164	0.34744	0.36413
+	FDFT1	8	11760573	11849309	612	52	10164	0.3474	0.36415
-	AIM2	1	159052479	159111895	109	14	10164	0.34688	0.36434
-	FLT4	5	180591506	180685271	313	37	10164	0.34685	0.36435
-	NPHP1	2	110113336	110240062	244	18	10164	0.34684	0.36436
+	RBBP4	1	32616148	32696211	218	8	10164	0.34681	0.36437
+	AJAP1	1	4620045	4802534	781	94	10164	0.34668	0.36441
+	HMMR	5	163425511	163501945	152	23	10164	0.34651	0.36448
+	CXCL13	4	77476753	77621834	414	27	10164	0.3464	0.36452
-	ZUFSP	6	116625618	116703810	236	24	10164	0.34639	0.36452
+	CLK3	15	74563476	74655418	181	28	10164	0.34634	0.36454
+	OR2AK2	1	247930332	247976339	197	11	10164	0.34627	0.36457
+	LPPR2	19	11320386	11375698	42	7	10164	0.34615	0.36461
+	MAP3K13	3	185247941	185499094	599	29	10164	0.34603	0.36466
+	PCDHB2	5	141059607	141107380	119	9	10164	0.34603	0.36466
-	SLC16A11	17	7031617	7079400	194	36	10164	0.34603	0.36466
+	HSDL2	9	112344909	112482405	580	36	10164	0.34572	0.36478
-	CLDN19	1	42723093	42775254	106	16	10164	0.34534	0.36492
+	SNX32	11	65798939	65865049	233	30	10164	0.34512	0.365
-	WNT10B	12	48955340	49006858	66	9	10164	0.34477	0.36513
-	SPINK1	5	147814580	147874231	194	29	10164	0.34462	0.36519
+	KPNB1	17	47614838	47693638	126	16	10164	0.34447	0.36525
-	XPO6	16	28087976	28246960	354	7	10164	0.34441	0.36527
+	DSG2	18	31463064	31558851	279	33	10164	0.34426	0.36533
+	CPNE2	16	57057543	57158369	168	29	10164	0.34414	0.36537
-	CALML4	15	68180705	68241110	188	18	10164	0.34393	0.36545
+	LOC1053758	8	54338207	54405505	254	34	10164	0.34393	0.36545
+	PRR14	16	30615717	30666413	35	2	10164	0.34377	0.36551
-	HTR1D	1	23181895	23229729	139	8	10164	0.34362	0.36557
+	LAT	16	28949826	29000783	92	11	10164	0.34357	0.36558
-	PEAK1	15	77090829	77455104	703	29	10164	0.34347	0.36562
-	LOC1019271	7	76061322	76107392	102	11	10164	0.34312	0.36575

STRAND	SYMBOL	CHR	START	STOP	NSNPS	NPARAM	N	ZSTAT	P
-	C2orf27B	2	131784961	131836661	68	5	10164	0.34308	0.36577
-	AKT1S1	19	49859033	49913356	146	31	10164	0.34273	0.3659
+	RBPJ	4	26128494	26445131	510	60	10164	0.34245	0.36601
-	LOC1053692	16	88079867	88125794	58	9	10164	0.34232	0.36605
+	PCDHGB3	5	141335264	141522979	369	24	10164	0.34228	0.36607
-	LOC1053727	20	61990728	62037317	164	16	10164	0.34224	0.36608
-	MAB21L1	13	35463789	35511695	101	13	10164	0.34221	0.36609
-	FBXL5	4	15594384	15694917	318	21	10164	0.34208	0.36615
-	MYH3	17	10618526	10714030	262	23	10164	0.34196	0.36619
+	MS4A2	11	60053261	60108467	187	19	10164	0.34186	0.36623
-	PI4KA	22	20697691	20893812	19	5	10164	0.34162	0.36632
-	SLC35D1	1	66989332	67089397	285	25	10164	0.34136	0.36642
-	C5orf45	5	179827266	179893840	169	29	10164	0.34114	0.3665
+	KREMEN1	22	29038078	29178333	529	53	10164	0.34113	0.3665
+	LRRIQ1	12	85001321	85282867	467	25	10164	0.34105	0.36653
+	KRBA1	7	149679969	149744575	152	17	10164	0.34105	0.36653
-	PITPNM2	12	122973480	123185810	181	16	10164	0.341	0.36655
-	PIAS2	18	46798246	46955167	512	28	10164	0.34093	0.36658
-	LTA4H	12	95990753	96078520	292	36	10164	0.34083	0.36661
-	RRP8	11	6504830	6638678	350	42	10164	0.34065	0.36668
+	CCNF	16	2394394	2468858	9	3	10164	0.34044	0.36676
-	HPX	11	6421038	6476024	132	24	10164	0.33998	0.36694
+	CLEC4M	19	7728054	7780558	107	13	10164	0.33996	0.36694
+	GTPBP8	3	112955482	113011374	137	23	10164	0.33995	0.36695
-	SLC35B4	7	134279332	134355314	386	24	10164	0.3399	0.36697
-	FAM155A	13	107158531	107902255	1921	160	10164	0.33988	0.36697
+	ZNF555	19	2806435	2870474	112	7	10164	0.33981	0.367
+	C1orf158	1	12711186	12771169	174	10	10164	0.33937	0.36717
-	ZBTB7A	19	4033303	4101818	132	18	10164	0.33914	0.36725
-	LOC1019277	11	5504393	5556635	281	22	10164	0.33903	0.36729
+	KCNJ15	21	38194915	38313126	436	25	10164	0.33872	0.36741
+	PKNOX1	21	42939510	43043931	312	14	10164	0.33856	0.36747
+	HSF4	16	67128385	67179945	46	11	10164	0.33854	0.36748
-	COL4A4	2	226957532	227199559	744	52	10164	0.3383	0.36757
-	SLC41A3	3	125996357	126136550	511	24	10164	0.33801	0.36768
-	EPC1	10	32257716	32413798	443	28	10164	0.33794	0.3677
-	SLC8B1	12	113288766	113370142	107	7	10164	0.33787	0.36773
+	C20orf195	20	63518020	63566708	198	38	10164	0.33777	0.36777
-	MRPS27	5	72209409	72355257	128	20	10164	0.33775	0.36778
+	EGLN2	19	40764143	40818441	120	12	10164	0.33762	0.36782
-	DDAH2	6	31717037	31765265	191	52	10164	0.33755	0.36785
-	COA5	2	98589323	98643518	106	11	10164	0.33743	0.3679
+	DTX3	12	57569327	57619804	115	20	10164	0.3374	0.36791
+	RPTOR	17	80509825	80976373	1511	64	10164	0.33728	0.36795
+	ZSCAN20	1	33437631	33510693	205	26	10164	0.33724	0.36797
+	NAA40	11	63903970	63967319	116	14	10164	0.33678	0.36814
+	KRTAP10-9	21	44592123	44638378	155	8	10164	0.33668	0.36818
-	ODF3B	22	50520409	50567579	187	16	10164	0.33665	0.36819
+	IL12A	3	159953836	160006019	187	28	10164	0.33659	0.36821
+	KIF5A	12	57515064	57594771	153	24	10164	0.33618	0.36837
-	LOC1053719	17	40308289	40358603	20	3	10164	0.33577	0.36852
-	CPT1B	22	50558861	50613667	160	18	10164	0.33568	0.36856
-	SEMA5B	3	122899193	123063605	528	62	10164	0.3354	0.36866
-	OGDHL	10	49724641	49797379	232	21	10164	0.33527	0.36871
+	FGD2	6	36970647	37039070	334	43	10164	0.33478	0.3689
+	NUTM2G	9	96894004	96952290	89	6	10164	0.33442	0.36903
+	PROM2	2	95239416	95301320	79	22	10164	0.33434	0.36906
-	PHC3	3	170076731	170216749	399	34	10164	0.33426	0.36909
-	PPP6C	9	125136573	125224939	313	19	10164	0.33425	0.3691
-	OR52E8	11	5846749	5892702	240	10	10164	0.33422	0.36911
-	GCC1	7	127570628	127620600	152	25	10164	0.33416	0.36913
-	CYBA	16	88633289	88686085	188	39	10164	0.33396	0.3692
-	MRPL24	1	156727302	156776266	84	12	10164	0.33391	0.36922

STRAND	SYMBOL	CHR	START	STOP	NSNPS	NPARAM	N	ZSTAT	P
+	GRXCR1	4	42858266	43040658	682	53	10164	0.33377	0.36928
+	SMC3	10	110532691	110614634	268	23	10164	0.33361	0.36934
-	KARS	16	75617724	75682687	220	26	10164	0.33351	0.36937
+	G0S2	1	209640325	209686390	73	19	10164	0.33343	0.3694
+	C20orf85	20	58115927	58171130	83	15	10164	0.33337	0.36943
+	OR4S1	11	48271223	48317152	171	12	10164	0.33335	0.36944
-	SAMHD1	20	36881824	36986843	202	19	10164	0.33327	0.36946
+	SYCP1	1	114819416	115005370	342	14	10164	0.33303	0.36955
+	SDHD	11	112051824	112105801	94	16	10164	0.33298	0.36957
+	SPRED1	15	38217087	38367249	433	18	10164	0.33299	0.36957
+	ZBTB40	1	22393838	22541157	343	32	10164	0.3326	0.36972
+	SEC23B	20	18472544	18571415	392	20	10164	0.3325	0.36975
+	PTOV1	19	49813844	49870742	133	17	10164	0.33228	0.36984
-	KIAA1462	10	30002800	30150486	406	46	10164	0.33227	0.36984
+	C1orf186	1	206017723	206112701	53	6	10164	0.33226	0.36984
-	ZNF835	19	56652652	56706783	131	18	10164	0.33221	0.36987
+	SNTN	3	63617668	63675217	284	32	10164	0.33217	0.36988
-	LOC1053781	6	130277921	130418090	501	17	10164	0.33201	0.36994
+	HINT3	6	125921715	125990244	65	15	10164	0.33178	0.37003
-	MLKL	16	74661855	74736148	192	32	10164	0.33175	0.37004
+	ERLEC1	2	53751931	53836928	283	31	10164	0.33167	0.37007
+	TOR1AIP1	1	179847042	179930077	174	14	10164	0.33138	0.37018
-	ZNF285	19	44375646	44436611	238	19	10164	0.33133	0.3702
+	RTN3	11	63646360	63769891	51	23	10164	0.33129	0.37021
+	NDUFAF3	3	48985475	49033495	51	11	10164	0.33118	0.37026
+	MGRN1	16	4581493	4700974	71	4	10164	0.33115	0.37027
+	KANK1	9	435294	756106	1540	59	10164	0.33109	0.37029
-	UBQLNL	11	5504393	5551726	242	22	10164	0.33102	0.37031
-	MRPL2	6	43044029	43095186	120	15	10164	0.33093	0.37035
-	TMCC1	3	129637792	129928597	369	26	10164	0.33067	0.37045
-	ERP44	9	99969179	100134052	238	13	10164	0.33021	0.37062
-	GGTLC1	20	23975053	24023779	182	14	10164	0.33014	0.37065
-	HHLA1	8	132051486	132140265	361	35	10164	0.32997	0.37071
+	HOXC4	12	53981852	54066030	123	20	10164	0.3299	0.37074
-	XPO4	13	20767329	20937774	380	20	10164	0.32973	0.3708
+	LOC1053716	1	205406393	205462029	110	15	10164	0.32966	0.37083
-	CLIC1	6	31720581	31772318	199	55	10164	0.32962	0.37084
+	HOXC5	12	53981852	54045361	86	15	10164	0.3296	0.37085
+	PRCC	1	156732482	156810817	174	17	10164	0.32948	0.3709
-	NLRC3	16	3529033	3613373	233	13	10164	0.32943	0.37092
-	GRAMD2	15	72149806	72232795	132	19	10164	0.32928	0.37097
+	MS4A5	11	60394589	60457792	280	32	10164	0.32917	0.37101
-	FDX1L	19	10300211	10351015	152	21	10164	0.32918	0.37101
+	CCNG2	4	77122204	77180060	145	20	10164	0.32903	0.37106
-	KIDINS220	2	8718857	8872625	36	7	10164	0.32883	0.37114
+	ZBTB47	3	42618684	42677580	118	15	10164	0.32883	0.37114
-	KPNA1	3	122411901	122549939	312	20	10164	0.32879	0.37116
-	PTH	11	13482054	13531020	155	12	10164	0.32877	0.37117
+	RDH8	19	9978249	10032278	18	5	10164	0.3287	0.37119
+	UBE2C	20	45777576	45826957	173	20	10164	0.32853	0.37126
+	ZNF583	19	56369014	56435031	135	22	10164	0.3283	0.37134
+	PCDHB3	5	141065242	141113827	128	9	10164	0.32804	0.37144
+	GDF2	10	47287490	47337594	36	10	10164	0.32801	0.37145
-	SLC41A1	1	205779093	205848759	169	24	10164	0.32797	0.37147
-	PRDX2	19	12786820	12836910	30	8	10164	0.32793	0.37148
+	TNFRSF11A	18	62290287	62397710	489	62	10164	0.32795	0.37148
-	SYPL1	7	106080368	106147647	162	12	10164	0.32742	0.37168
-	WDPCP	2	63110762	63875825	1806	63	10164	0.32738	0.37169
-	SHC2	19	406583	496024	19	4	10164	0.32718	0.37177
-	TMED6	16	69333246	69386809	96	19	10164	0.3271	0.37179
-	GRB14	2	164482406	164656850	370	43	10164	0.32689	0.37188
-	NDUFA10	2	239947372	240060402	328	23	10164	0.32674	0.37193
-	FBP1	9	94593133	94675249	311	27	10164	0.32667	0.37196

STRAND	SYMBOL	CHR	START	STOP	NSNPS	NPARAM	N	ZSTAT	P
+	MT3	16	56554355	56601088	208	41	10164	0.32667	0.37196
-	GREM2	1	240479573	240647272	142	34	10164	0.32655	0.37201
+	CA2	8	85428902	85491492	97	12	10164	0.32646	0.37204
+	CSDC2	22	41526010	41586666	142	16	10164	0.32644	0.37205
-	GH1	17	63907193	63953852	104	12	10164	0.32636	0.37208
-	FAM162B	6	116742197	116800723	187	28	10164	0.32635	0.37208
+	SLC34A3	9	137195116	137246554	123	17	10164	0.32627	0.37211
-	NUP133	1	229431297	229543341	241	10	10164	0.32624	0.37212
-	TMEM252	9	68520662	68575886	118	21	10164	0.32614	0.37216
+	CYB5D1	17	7822746	7873486	91	16	10164	0.32598	0.37222
+	LMOD2	7	123620807	123674093	63	9	10164	0.32585	0.37227
+	GOLGA8N	15	32552328	32617310	14	4	10164	0.32571	0.37232
-	RSPH10B	7	5916136	6005684	121	21	10164	0.3253	0.37248
+	CACNA2D3	3	54087593	55084557	2982	207	10164	0.32516	0.37253
-	GDF6	8	96132330	96195792	11	7	10164	0.32462	0.37273
+	SSSCA1	11	65535459	65581888	95	24	10164	0.32458	0.37275
+	MAP4K4	2	101662703	101904690	572	53	10164	0.32422	0.37288
-	GSDMB	17	39894595	39954377	145	13	10164	0.32415	0.37291
-	GGCT	7	30486621	30539841	114	13	10164	0.32391	0.373
-	LOC149373	1	231174099	231222627	157	25	10164	0.3235	0.37316
-	ASPRV1	2	69950089	69997265	115	21	10164	0.32318	0.37328
+	VPS26A	10	69089152	69182861	263	25	10164	0.3231	0.37331
+	ZNF10	12	133095628	133169465	131	8	10164	0.32304	0.37333
-	DDX39A	19	14398798	14454383	87	23	10164	0.32301	0.37334
-	WDR27	6	169416407	169737063	950	27	10164	0.32282	0.37341
+	ASPG	14	104050670	104122761	148	20	10164	0.32255	0.37352
+	GPBP1	5	57138849	57274679	280	35	10164	0.32252	0.37353
-	PLA2G2A	1	19965431	20015439	162	26	10164	0.32244	0.37356
-	SLC2A7	1	8950853	9061384	269	48	10164	0.32234	0.3736
+	C20orf24	20	36570734	36622557	42	16	10164	0.32228	0.37362
-	CYP7B1	8	64585972	64833791	453	32	10164	0.32218	0.37366
-	CMC2	16	80965802	81041897	395	16	10164	0.32214	0.37367
+	RUVBL2	19	48958353	49025926	169	19	10164	0.32188	0.37377
+	PDP2	16	66845457	66901101	104	13	10164	0.32176	0.37382
+	NAA30	14	57355553	57422748	170	14	10164	0.32165	0.37386
+	MXI1	10	110172605	110297365	314	32	10164	0.32158	0.37389
+	SNX15	11	64992408	65050572	135	19	10164	0.32124	0.37401
+	CYLC2	9	102960311	103028488	293	23	10164	0.32121	0.37402
-	CNFN	19	42377019	42425317	58	7	10164	0.32123	0.37402
-	RPL24	3	101671090	101721719	106	18	10164	0.32121	0.37403
+	RAP1A	1	111584783	111726695	477	30	10164	0.32115	0.37405
+	C16orf70	16	67074898	67158539	98	12	10164	0.32056	0.37427
-	EGR2	10	62801996	62954895	611	38	10164	0.32033	0.37436
-	IGF2BP2	3	185633131	185860039	338	33	10164	0.32029	0.37438
-	G3BP2	4	75632769	75759525	314	18	10164	0.32007	0.37446
+	TBK1	12	64417060	64512119	166	19	10164	0.31977	0.37457
-	C16orf97	16	52016352	52113517	274	35	10164	0.31966	0.37461
-	HEATR5A	14	31281788	31455582	446	25	10164	0.31956	0.37465
+	HMG20A	15	77385651	77495603	218	23	10164	0.31946	0.37469
+	DYRK1A	21	37330790	37525376	441	26	10164	0.31944	0.3747
+	NAT14	19	55450190	55497568	141	27	10164	0.31936	0.37473
+	ZFP69B	1	40415086	40473718	160	27	10164	0.31933	0.37474
+	UBB	17	16345793	16392745	34	4	10164	0.31908	0.37483
-	KIAA1644	22	44233665	44366695	572	104	10164	0.31868	0.37498
-	TEDDM1	1	182388117	182435616	122	14	10164	0.31855	0.37503
+	ABCG1	21	42164689	42314387	304	28	10164	0.31853	0.37504
-	SF3B4	1	149913317	149963252	28	6	10164	0.31852	0.37504
-	LOC1053747	5	41194	92883	63	12	10164	0.31818	0.37517
+	ATF3	1	212530334	212630777	283	43	10164	0.31811	0.3752
-	CSF1R	5	150043291	150148372	364	62	10164	0.31806	0.37522
-	LOC1053750	6	29480320	29529236	313	33	10164	0.31798	0.37525
+	IL1RL2	2	102151157	102250002	364	29	10164	0.31784	0.3753
-	LRRC4B	19	50506892	50603219	311	61	10164	0.31777	0.37533

STRAND	SYMBOL	CHR	START	STOP	NSNPS	NPARAM	N	ZSTAT	P
+	PLD3	19	40313425	40388490	161	22	10164	0.31758	0.3754
-	CCDC3	10	12886625	13134773	937	95	10164	0.31752	0.37543
-	NDNF	4	121025627	121107518	189	24	10164	0.31741	0.37547
+	PRUNE	1	150973391	151045713	149	25	10164	0.31735	0.37549
+	PRKAB1	12	119632864	119691624	117	15	10164	0.31724	0.37553
+	HMX2	10	123107421	123160672	151	32	10164	0.31694	0.37564
-	TMEM231	16	75528117	75591286	191	17	10164	0.31695	0.37564
-	COX19	7	954850	1010599	261	27	10164	0.31672	0.37573
+	NCAN	19	19176964	19262252	274	44	10164	0.31661	0.37577
+	CEP85	1	26199153	26289038	304	15	10164	0.31662	0.37577
+	KCTD17	22	37016725	37073390	67	9	10164	0.3166	0.37577
-	SOST	17	43743731	43793788	127	14	10164	0.31614	0.37595
-	ALKBH6	19	35999116	36049243	79	15	10164	0.31609	0.37597
+	LAMC2	1	183151039	183255127	373	29	10164	0.31603	0.37599
+	BTF3	5	73463425	73515623	105	3	10164	0.316	0.376
+	IL36A	2	112970461	113021071	136	22	10164	0.31557	0.37616
-	TMEM59	1	54021673	54088462	132	15	10164	0.31533	0.37626
+	SRRM5	19	43577101	43624498	121	25	10164	0.31514	0.37633
-	PLSCR3	17	7379727	7429843	124	33	10164	0.31497	0.37639
+	ITK	5	157145896	157265099	323	37	10164	0.31492	0.37641
-	KIF12	9	114081623	114135084	177	30	10164	0.31483	0.37644
+	WDR6	3	48972204	49025953	55	13	10164	0.31465	0.37651
-	SLC30A4	15	45472478	45558669	136	13	10164	0.31461	0.37653
+	ID1	20	31570283	31616515	17	7	10164	0.31451	0.37657
-	SOX6	11	15956449	16511388	1119	45	10164	0.31451	0.37657
-	TSPAN19	12	85004311	85071278	113	14	10164	0.31438	0.37662
+	CDT1	16	88768778	88819258	242	34	10164	0.31425	0.37667
+	LRRC46	17	47796627	47847713	174	27	10164	0.3138	0.37684
+	P4HTM	3	48954100	49017149	75	13	10164	0.31366	0.37689
+	NAT10	11	34070564	34161034	30	13	10164	0.31362	0.37691
-	PLA2R1	2	159922002	160097902	611	38	10164	0.31322	0.37706
-	ZNF843	16	31425564	31478027	44	8	10164	0.31309	0.37711
-	PECR	2	216028388	216116816	226	33	10164	0.31286	0.37719
+	TEX38	1	46634346	46683594	164	11	10164	0.31236	0.37739
-	CHCHD10	22	23755834	23802972	179	19	10164	0.3122	0.37745
+	SBK1	16	28223703	28333849	208	23	10164	0.31206	0.3775
-	RBP4	10	93581836	93636236	166	27	10164	0.31196	0.37753
+	WAC	10	28497588	28633112	258	17	10164	0.31182	0.37759
+	CYSLTR2	13	48671815	48719362	101	16	10164	0.31176	0.37761
-	STYXL1	7	75985834	76083003	232	20	10164	0.31106	0.37788
-	LRRC18	10	48873696	48975126	272	33	10164	0.31094	0.37792
-	KISS1	1	204180341	204231491	122	19	10164	0.31079	0.37798
+	CRX	19	47786842	47853330	302	23	10164	0.31048	0.3781
+	MMP24	20	35191736	35287663	194	29	10164	0.3103	0.37817
-	PSG11	19	42997657	43061479	200	17	10164	0.31006	0.37826
+	TWIST2	2	238812934	238920548	86	15	10164	0.31002	0.37827
+	TCEANC2	1	54018572	54122519	231	21	10164	0.31001	0.37828
+	C3orf52	3	112051335	112141095	475	27	10164	0.30955	0.37845
+	HSD11B1L	19	5645765	5698523	97	10	10164	0.30955	0.37845
-	IL15RA	10	5938900	6013634	441	76	10164	0.30951	0.37847
+	TSNAXIP1	16	67771656	67838068	80	12	10164	0.30947	0.37848
-	TMEM65	8	124296189	124407699	309	15	10164	0.30939	0.37851
+	SLC5A3	21	34038523	34116262	69	15	10164	0.30898	0.37867
-	ANKRD34B	5	80546755	80607753	207	38	10164	0.3089	0.3787
-	DNAJB14	4	99886248	99981726	107	11	10164	0.30881	0.37873
-	TTC30B	2	177540152	177587796	152	11	10164	0.3088	0.37874
-	TYK2	19	10340528	10415572	219	27	10164	0.30843	0.37888
-	PLCXD3	5	41296946	41545628	768	51	10164	0.30826	0.37894
-	LOC1053691	16	67200434	67261998	77	12	10164	0.30819	0.37897
-	ALDH8A1	6	134907390	134985122	143	12	10164	0.30803	0.37903
+	PCDHB1	5	141016376	141063962	55	8	10164	0.30793	0.37907
-	FCRL3	1	157666481	157735985	190	21	10164	0.3079	0.37908
+	TAS2R5	7	141755217	141801366	159	9	10164	0.30751	0.37923

STRAND	SYMBOL	CHR	START	STOP	NSNPS	NPARAM	N	ZSTAT	P
-	COLCA1	11	111283389	111340048	200	33	10164	0.3074	0.37927
-	LIPA	10	89203569	89287039	196	40	10164	0.30728	0.37931
+	HSP90AB1	6	44211166	44263888	122	20	10164	0.30725	0.37933
-	KRT8	12	52887187	52984866	375	42	10164	0.30711	0.37938
-	GOT2	16	58697131	58769357	379	21	10164	0.30709	0.37939
-	PDC	1	186433566	186496108	138	15	10164	0.30708	0.37939
+	PHGR1	15	40316033	40366434	142	32	10164	0.30705	0.3794
+	TEX19	17	82324247	82373776	69	3	10164	0.30678	0.3795
-	MAP3K7	6	90503573	90622301	340	17	10164	0.30676	0.37951
+	TTC36	11	118492495	118541025	69	9	10164	0.30674	0.37952
-	SATB2	2	199259500	199506266	367	33	10164	0.3066	0.37957
-	NKX3-1	8	23668693	23717937	201	34	10164	0.30643	0.37964
+	C1orf226	1	162343906	162396818	184	38	10164	0.30625	0.37971
-	TUBB2B	6	3214261	3266665	131	4	10164	0.30621	0.37972
-	SIGLEC11	19	49938993	49996172	213	19	10164	0.30595	0.37982
+	MIB1	18	21705793	21880957	246	17	10164	0.30569	0.37992
-	IFNL3	19	39233606	39282034	91	13	10164	0.30552	0.37998
-	DSCAM	21	40000999	40882113	2195	174	10164	0.30531	0.38007
-	TMPRSS11D	4	67810876	67918998	161	19	10164	0.30494	0.38021
-	SLC25A1	22	19165575	19213863	196	21	10164	0.30489	0.38023
+	TAS2R4	7	141743489	141789388	166	10	10164	0.30425	0.38047
-	MALL	2	110073870	110153139	135	17	10164	0.30419	0.38049
+	ZNF556	19	2832335	2888517	85	6	10164	0.3037	0.38068
-	ZNF615	19	51981332	52043238	170	15	10164	0.30359	0.38072
+	SLC29A1	6	44184505	44244151	110	23	10164	0.3033	0.38083
+	OR52M1	11	4510191	4556144	378	9	10164	0.30331	0.38083
-	PSMB2	1	35589541	35676844	200	13	10164	0.30323	0.38086
+	RASD2	22	35505518	35564002	144	25	10164	0.30256	0.38111
+	KRTAP10-4	21	44538729	44585371	188	21	10164	0.30242	0.38117
-	TRMT61B	2	28839821	28905309	118	12	10164	0.30238	0.38118
+	KRT86	12	52239631	52319163	254	25	10164	0.30233	0.3812
-	JPH1	8	74224700	74356500	418	43	10164	0.30228	0.38122
-	SLC4A1	17	44238390	44303161	157	31	10164	0.30169	0.38144
+	SFN	1	26828142	26874456	45	6	10164	0.30162	0.38147
+	TMX1	14	51205168	51267655	188	21	10164	0.30159	0.38148
+	ACTN2	1	236651454	236774631	534	38	10164	0.30153	0.3815
-	UBL3	13	29754405	29885683	356	22	10164	0.30122	0.38162
+	CAPZA3	12	18703111	18749188	104	10	10164	0.30055	0.38188
+	UGT2B10	4	68780993	68842017	178	17	10164	0.30022	0.38201
-	PRKD2	19	46664316	46752127	197	22	10164	0.30019	0.38202
+	DHX9	1	182804304	182897982	175	16	10164	0.30013	0.38204
-	ELL2	5	95875098	95997071	254	14	10164	0.29997	0.3821
-	SLC2A13	12	39745021	40141074	1179	62	10164	0.29973	0.38219
-	C6orf7	6	79793582	79913494	475	48	10164	0.29964	0.38223
+	FMO4	1	171279137	171352084	78	13	10164	0.29953	0.38227
-	PELI1	2	64082652	64179471	220	25	10164	0.29951	0.38228
+	KLC4	6	43024594	43085099	139	14	10164	0.29942	0.38231
+	UBA2	19	34393176	34479893	87	7	10164	0.29939	0.38232
+	TCAIM	3	44303119	44419448	164	7	10164	0.29919	0.3824
+	XPOT	12	64369350	64461125	157	21	10164	0.29904	0.38246
+	C17orf77	17	74549679	74604209	253	27	10164	0.29892	0.3825
-	INS-IGF2	11	2119112	2196209	291	38	10164	0.29847	0.38267
-	RPP40	6	4982414	5039063	212	14	10164	0.29844	0.38269
+	OR52E4	11	5845630	5895294	258	11	10164	0.29813	0.3828
+	CDH20	18	61298582	61565773	515	89	10164	0.2981	0.38281
+	AKAP6	14	32294273	32843999	1740	159	10164	0.29771	0.38296
+	PLG	6	160667193	160764054	303	43	10164	0.29753	0.38303
-	C19orf47	19	40285271	40383527	206	23	10164	0.29709	0.3832
+	MSH5	6	31704948	31772678	284	63	10164	0.29694	0.38325
-	FAM98A	2	33573661	33634362	160	17	10164	0.2969	0.38327
-	CUL7	6	43027617	43088950	142	15	10164	0.29688	0.38328
+	C17orf105	17	43743391	43794842	130	14	10164	0.29682	0.3833
-	EVPL	17	75996845	76062452	117	20	10164	0.29678	0.38332

STRAND	SYMBOL	CHR	START	STOP	NSNPS	NPARAM	N	ZSTAT	P
+	LOC1053730	22	45847056	45904406	108	18	10164	0.29661	0.38338
+	SGOL2	2	200489802	200594095	265	35	10164	0.29622	0.38353
+	FAM157B	9	138180351	138262775	35	6	10164	0.29608	0.38358
+	TAS2R3	7	141729097	141775197	163	12	10164	0.29595	0.38363
-	ASB10	7	151165698	151222832	114	21	10164	0.29591	0.38365
+	SH3RF3	2	109094498	109514636	1551	79	10164	0.2956	0.38377
+	ZC3H12C	11	110053412	110181841	284	28	10164	0.2954	0.38384
+	FAM20B	1	178990809	179086567	324	29	10164	0.29539	0.38385
+	MS4A3	11	60021628	60081116	147	19	10164	0.29519	0.38392
-	ERV3-1	7	64980355	65041746	244	19	10164	0.29472	0.3841
+	PCDHGA5	5	141329331	141522979	377	27	10164	0.29462	0.38414
+	SERPINA3	14	94577377	94634053	275	39	10164	0.29457	0.38416
-	GNB2L1	5	181226928	181278906	131	9	10164	0.29445	0.38421
+	GSX1	13	27757643	27803952	114	18	10164	0.29442	0.38422
+	MDH2	7	76013019	76077508	162	15	10164	0.2944	0.38423
-	CCPG1	15	55345223	55443510	354	20	10164	0.29394	0.3844
+	SNPH	20	1231297	1319327	151	17	10164	0.29381	0.38445
+	UNC50	2	98573579	98628515	98	11	10164	0.29378	0.38446
-	RTCB	22	32377575	32447287	257	35	10164	0.29357	0.38454
+	CYP4F22	19	15473484	15562317	206	29	10164	0.29348	0.38458
+	PTP4A1	6	63537012	63593588	88	10	10164	0.29303	0.38475
-	TMEM183B	3	149971661	150018366	120	18	10164	0.29287	0.38481
+	POLI	18	54234479	54331266	393	24	10164	0.29278	0.38485
+	C16orf52	16	21973135	22094654	96	14	10164	0.29249	0.38496
-	CLDN3	7	73758997	73805270	50	4	10164	0.2922	0.38507
+	SS18L2	3	42546840	42604998	137	13	10164	0.29191	0.38518
+	BANP	16	87914172	88087318	486	15	10164	0.29138	0.38538
+	HBP1	7	107133961	107212529	150	12	10164	0.29122	0.38544
+	PRAP1	10	133312340	133362683	39	5	10164	0.2912	0.38545
-	RPS6KA2	6	166399366	166897550	1383	121	10164	0.29082	0.3856
+	ZSWIM1	20	45845942	45895266	183	22	10164	0.29078	0.38561
+	LOC730159	1	173568008	173647134	116	15	10164	0.29074	0.38562
-	UBE2W	8	73770097	73913910	265	20	10164	0.29068	0.38565
-	TRPS1	8	115398496	115704580	517	26	10164	0.29064	0.38566
+	AGPAT5	8	6673357	6771503	340	34	10164	0.2906	0.38568
+	DOK5	20	54440472	54661171	308	33	10164	0.29055	0.3857
+	SCGB3A2	5	147843711	147892193	226	24	10164	0.29037	0.38577
-	SCML4	6	107687297	107883659	799	59	10164	0.29015	0.38585
+	C5AR1	19	47272745	47332070	114	10	10164	0.29011	0.38587
+	NAV3	12	77536862	78223012	1532	104	10164	0.28986	0.38596
+	RALY	20	33958652	34093185	252	23	10164	0.28973	0.38601
-	ZNF764	16	30543764	30593321	10	4	10164	0.2896	0.38606
+	TMEM102	17	7393825	7447679	128	37	10164	0.28949	0.3861
-	NKX2-4	20	21385367	21432409	90	16	10164	0.28916	0.38623
-	SOS2	14	50107128	50266878	397	17	10164	0.289	0.38629
+	LOC1005064	9	26031675	26128408	204	27	10164	0.28884	0.38635
-	LOC1027233	11	17685267	17732526	103	11	10164	0.28871	0.3864
+	PCDHGB2	5	141325136	141522979	384	25	10164	0.28851	0.38648
-	FMNL3	12	49627941	49742414	194	12	10164	0.28838	0.38653
+	RNPC3	1	103490956	103565239	70	11	10164	0.28832	0.38655
-	EXOSC3	9	37769714	37820092	136	10	10164	0.28771	0.38678
-	FBXL18	7	5444465	5548805	231	18	10164	0.28758	0.38683
+	ELN	7	73992772	74079907	5	2	10164	0.28694	0.38708
+	MYT1	20	64129474	64252253	380	49	10164	0.28692	0.38709
-	BOD1	5	173597145	173652077	172	22	10164	0.28691	0.38709
+	RLN3	19	13993205	14040971	88	11	10164	0.28664	0.38719
+	TYRP1	9	12658386	12720266	206	23	10164	0.28652	0.38724
-	SLC15A5	12	16178485	16312685	279	32	10164	0.28649	0.38725
-	LOC400499	16	11362601	11558533	714	59	10164	0.28648	0.38726
-	UGT2B11	4	69189655	69251768	265	20	10164	0.2862	0.38736
-	HLA-DQB1	6	32649464	32701689	2127	30	10164	0.28618	0.38737
+	ARRDC2	19	17966132	18024102	116	16	10164	0.28592	0.38747
+	TIMM17A	1	201920491	201980661	192	41	10164	0.28562	0.38758

STRAND	SYMBOL	CHR	START	STOP	NSNPS	NPARAM	N	ZSTAT	P
-	TCP11	6	35108071	35176642	283	32	10164	0.28544	0.38766
-	BNIP2	15	59649146	59724529	283	14	10164	0.28537	0.38768
+	CDS2	20	5091761	5207887	207	18	10164	0.28528	0.38771
-	SLC2A2	3	170985365	171061979	154	14	10164	0.28483	0.38789
+	UBE2QL1	5	6402347	6506721	294	33	10164	0.28465	0.38795
+	AQP11	11	77554635	77620356	144	10	10164	0.28461	0.38797
+	TPPP2	14	20995186	21046352	113	29	10164	0.28448	0.38802
+	OPN5	6	47745302	47867840	388	30	10164	0.28443	0.38804
-	FHL2	2	105350815	105473773	461	76	10164	0.28386	0.38826
+	C4orf17	4	99476004	99552303	287	27	10164	0.28366	0.38834
+	TANGO2	22	19982000	20075926	375	39	10164	0.28338	0.38844
+	TSPAN11	12	30891449	31026490	442	76	10164	0.28334	0.38846
-	AAGAB	15	67190675	67290198	339	26	10164	0.28329	0.38848
+	TIMM10B	11	6446447	6494681	137	14	10164	0.28323	0.3885
+	GABARAPL2	16	75531351	75587881	175	16	10164	0.2832	0.38851
+	TMEM109	11	60878899	60933443	79	5	10164	0.28318	0.38852
+	KCNK6	19	38284814	38339014	282	45	10164	0.28301	0.38858
+	ADCK2	7	140637925	140705111	62	3	10164	0.28288	0.38864
+	TMC5	16	19375522	19509113	494	34	10164	0.28282	0.38866
+	MLLT11	1	151024675	151078497	112	17	10164	0.28258	0.38875
-	ITGA11	15	68287428	68467160	614	53	10164	0.28256	0.38876
+	LRRC3C	17	39892722	39955034	152	13	10164	0.28249	0.38878
-	B4GALT6	18	31612246	31760286	372	47	10164	0.28246	0.3888
-	RBM23	14	22890645	22954187	134	8	10164	0.28229	0.38886
-	TIMM8B	11	112074800	112121798	104	17	10164	0.28214	0.38892
+	IL1R2	2	101956816	102038544	318	29	10164	0.28199	0.38897
+	CRAMP1L	16	1579640	1687908	294	19	10164	0.28167	0.3891
+	ALDH3B1	11	67973547	68039282	226	29	10164	0.28165	0.38911
+	ATAD3B	1	1436732	1519479	5	3	10164	0.2816	0.38913
-	PSG5	19	43022810	43221536	611	21	10164	0.28133	0.38923
-	ZBED5	11	10842704	10893073	38	7	10164	0.281	0.38935
+	QTRTD1	3	114021731	114098422	172	18	10164	0.2809	0.38939
+	APCDD1	18	10419628	10498701	288	45	10164	0.28085	0.38941
-	MSRB1	16	1928233	1978293	168	36	10164	0.28083	0.38942
-	CALY	10	133314072	133371971	48	5	10164	0.28067	0.38948
-	LOC1019284	1	103408079	103560483	148	18	10164	0.28061	0.3895
+	STX4	16	30998095	31050168	69	6	10164	0.28055	0.38953
+	TPM1	15	63007639	63081915	305	32	10164	0.28055	0.38953
+	CHST8	19	33586955	33783509	269	41	10164	0.28043	0.38958
-	ACP2	11	47229302	47283847	131	13	10164	0.28013	0.38969
-	RPL32	3	12824945	12876582	161	12	10164	0.2801	0.3897
+	MAATS1	3	119668022	119777102	267	25	10164	0.28004	0.38972
+	LOC1053766	12	9413794	9513089	144	8	10164	0.27994	0.38976
-	MCEE	2	71099676	71165288	234	21	10164	0.27992	0.38977
-	NAPA	19	47477634	47550258	74	8	10164	0.27987	0.38979
-	TMEM132D	12	129061726	129938730	2997	292	10164	0.27966	0.38987
+	MTHFD2L	4	74079183	74313099	391	34	10164	0.27958	0.3899
+	ZMIZ1	10	79033964	79326528	1185	164	10164	0.27956	0.38991
+	ADORA1	1	203092705	203177405	260	39	10164	0.27951	0.38993
-	GABRG1	4	46025769	46159065	394	21	10164	0.27946	0.38994
-	MVD	16	88641935	88698157	206	37	10164	0.2791	0.39009
-	RILPL1	12	123461362	123568718	122	10	10164	0.27884	0.39018
+	ZNF543	19	57285497	57340776	239	29	10164	0.2786	0.39027
+	ENDOG	9	128783411	128832678	135	12	10164	0.27846	0.39033
+	E2F5	8	85142056	85224518	112	10	10164	0.27818	0.39044
+	TGM4	3	44839592	44924601	151	14	10164	0.27803	0.39049
-	SPARCL1	4	87463330	87564503	377	26	10164	0.27791	0.39054
-	KRT4	12	52796543	52849116	240	48	10164	0.27749	0.3907
+	SNAPC2	19	7885309	7933251	140	27	10164	0.27733	0.39076
-	TIAM1	21	31108418	31594977	1224	102	10164	0.27729	0.39078
+	APOL6	22	35613360	35678409	140	12	10164	0.27724	0.3908
+	PDHA2	4	95805088	95851474	318	17	10164	0.27719	0.39082
+	ACSS3	12	81043018	81267795	797	38	10164	0.27718	0.39082

STRAND	SYMBOL	CHR	START	STOP	NSNPS	NPARAM	N	ZSTAT	P
+	ZNF778	16	89167038	89242637	414	27	10164	0.27701	0.39089
+	PAEP	9	135526754	135576776	131	19	10164	0.27673	0.39099
+	CLEC18C	16	70138322	70197147	46	7	10164	0.27669	0.39101
+	NAA35	9	85906142	86035462	175	26	10164	0.2766	0.39105
-	TTLL12	22	43156622	43222131	441	53	10164	0.27657	0.39106
-	ASGR1	17	7163431	7214564	96	23	10164	0.27632	0.39115
+	NIPSNAP3B	9	104728741	104787764	156	18	10164	0.27584	0.39134
-	BAZ2B	2	159305312	159747439	1470	53	10164	0.27566	0.3914
-	ITPR2	12	26325352	26868198	1815	128	10164	0.27565	0.39141
+	ZNF703	8	37660751	37710021	14	6	10164	0.27565	0.39141
-	AGXT2	5	34988101	35083188	337	31	10164	0.27559	0.39143
-	SRI	7	88195115	88261993	110	17	10164	0.2751	0.39162
+	PRDX5	11	64283088	64331823	156	28	10164	0.27465	0.39179
-	DIRAS3	1	68035962	68086733	53	20	10164	0.27464	0.3918
+	CACNG1	17	67009536	67066797	112	25	10164	0.2745	0.39185
+	PLK1	16	23643822	23700367	163	11	10164	0.27437	0.3919
+	IL33	9	6180149	6267983	178	23	10164	0.2741	0.392
-	ZNF415	19	53097879	53168077	227	25	10164	0.27388	0.39209
+	LOC1053702	13	83871573	84078784	751	38	10164	0.27371	0.39215
-	ABCF2	7	151197830	151262374	100	11	10164	0.2737	0.39216
-	CTNNBIP1	1	9838276	9945258	185	22	10164	0.2734	0.39227
-	GRIK5	19	41988316	42106804	81	16	10164	0.27333	0.3923
+	ATG10	5	81937025	82268502	520	35	10164	0.27316	0.39236
+	TMEM5	12	63744803	63819558	168	21	10164	0.27302	0.39242
+	SERPINA4	14	94526420	94579913	193	27	10164	0.27226	0.39271
-	SCN4A	17	63928554	64025150	145	24	10164	0.27217	0.39275
-	SMG5	1	156239224	156317885	211	19	10164	0.27213	0.39276
-	PIGY	4	88510978	88558801	105	15	10164	0.27184	0.39287
-	PYURF	4	88510978	88558801	105	15	10164	0.27184	0.39287
-	ENC1	5	74617406	74676424	74	9	10164	0.2718	0.39289
+	LOC402269	7	63597667	63650929	7	3	10164	0.27164	0.39295
-	TYMP	22	50515752	50565085	194	18	10164	0.27162	0.39296
+	ANKRD18B	9	33489353	33585969	201	29	10164	0.27161	0.39296
+	POLR3F	20	18432389	18494648	260	24	10164	0.27152	0.39299
+	PSMF1	20	1078263	1178378	202	29	10164	0.27144	0.39302
+	CRYBA1	17	29211857	29264494	111	13	10164	0.27137	0.39305
+	EXOSC9	4	121766295	121827021	175	21	10164	0.27111	0.39315
-	POLR3H	22	41515799	41579606	140	15	10164	0.27112	0.39315
-	FAM163B	9	133567076	133644920	263	32	10164	0.27098	0.3932
+	PCCB	3	136215325	136340171	310	16	10164	0.27073	0.3933
-	ISOC2	19	55442978	55496682	151	30	10164	0.27054	0.39337
-	CLPX	15	65140446	65220404	118	14	10164	0.27046	0.3934
+	BIRC6	2	32322028	32628899	370	39	10164	0.27003	0.39357
+	KIAA1549L	11	33341568	33684102	278	44	10164	0.26977	0.39367
+	BHLHE22	8	64545238	64593634	96	16	10164	0.26962	0.39373
+	PRMT7	16	68275974	68368829	189	12	10164	0.26934	0.39383
+	RCC1	1	28470943	28549196	88	6	10164	0.26933	0.39384
-	INO80D	2	205983721	206121182	295	19	10164	0.26933	0.39384
+	DOCK8	9	179865	475259	1093	67	10164	0.26882	0.39403
+	CPA4	7	130258134	130334180	167	24	10164	0.26864	0.3941
+	FAM181A	14	93883894	93939608	197	34	10164	0.2685	0.39416
-	IRGQ	19	43574367	43631229	136	26	10164	0.26845	0.39418
-	GPRC5B	16	19848971	19921045	157	21	10164	0.26831	0.39423
+	CELF5	19	3189676	3307076	128	12	10164	0.26825	0.39425
-	DNAJC9	10	73231954	73282331	124	3	10164	0.26814	0.39429
-	HAPLN3	15	88867288	88930626	189	24	10164	0.26802	0.39434
+	MYOD1	11	17684563	17732131	105	12	10164	0.26778	0.39444
-	SLC25A19	17	75262980	75324957	159	11	10164	0.26763	0.39449
-	UROS	10	125774980	125858268	268	28	10164	0.2674	0.39458
-	C2CD5	12	22438546	22579546	262	12	10164	0.26727	0.39463
+	MYO18B	22	25707147	26068788	1120	114	10164	0.26718	0.39467
+	IGFBP2	2	216598404	216674436	185	33	10164	0.2671	0.3947
+	MUC2	11	1039875	1120508	218	31	10164	0.26676	0.39483

STRAND	SYMBOL	CHR	START	STOP	NSNPS	NPARAM	N	ZSTAT	P
+	VSTM2L	20	37868097	37955350	269	46	10164	0.26672	0.39484
-	LGALS3BP	17	78961253	79014979	198	35	10164	0.2666	0.39489
+	CREB3	9	35697320	35747008	69	20	10164	0.26657	0.3949
-	C6	5	41132146	41296486	600	41	10164	0.2664	0.39496
+	LOC1053753	7	63730655	63777001	96	12	10164	0.26636	0.39498
+	F2R	5	76681043	76745770	218	26	10164	0.26622	0.39504
+	THTPA	14	23476760	23569581	136	21	10164	0.26617	0.39505
-	LOC1053729	1	214009269	214147021	268	46	10164	0.26599	0.39512
+	LOC1027247	7	63728951	63774555	95	12	10164	0.26562	0.39527
+	RSPH4A	6	116558900	116643051	280	31	10164	0.26558	0.39528
+	POFUT1	20	32172822	32248664	75	8	10164	0.2655	0.39531
+	CYTH4	22	37247454	37325348	154	25	10164	0.26544	0.39534
-	PGM2L1	11	74320312	74433465	323	21	10164	0.26541	0.39535
-	SLC23A1	5	139357196	139419553	72	11	10164	0.2653	0.39539
-	FAM96A	15	64062559	64129008	80	5	10164	0.26519	0.39543
+	TEAD1	11	12639422	12954737	590	42	10164	0.265	0.3955
-	TIMELESS	12	56406373	56484416	132	12	10164	0.26499	0.39551
-	NFE2L2	2	177220303	177300131	216	25	10164	0.26482	0.39558
+	FBLIM1	1	15721171	15796589	218	27	10164	0.26474	0.3956
-	A4GNT	3	138113718	138167387	42	2	10164	0.26454	0.39568
-	ENKD1	16	67652947	67701725	58	11	10164	0.26433	0.39576
-	HAS2	8	121602116	121676390	184	13	10164	0.2643	0.39578
+	MKLN1	7	131075096	131506639	867	83	10164	0.26427	0.39579
+	TMED2	12	123549529	123608144	150	13	10164	0.26417	0.39582
-	LOC1001305	17	74547246	74602534	266	29	10164	0.26403	0.39588
-	ENOSF1	18	660324	747662	412	23	10164	0.26393	0.39592
-	DPYS	8	104369424	104502049	517	49	10164	0.26391	0.39593
-	FAM63A	1	150986825	151043378	119	22	10164	0.26387	0.39594
-	ZC3HAV1L	7	139011498	139071489	162	9	10164	0.26376	0.39598
+	CHSY3	5	129868408	130196678	701	35	10164	0.26376	0.39598
+	NPY2R	4	155138754	155227096	240	15	10164	0.26367	0.39602
+	KRT18	12	52913871	52962901	140	27	10164	0.26353	0.39607
+	LCE2B	1	152651123	152697401	113	7	10164	0.26322	0.39619
-	TARBP1	1	234381313	234514158	144	11	10164	0.26297	0.39629
-	DENND5A	11	9128825	9300335	330	18	10164	0.26274	0.39638
+	LOC1053698	12	80365695	80433307	149	6	10164	0.2625	0.39647
+	TRAF3IP1	2	238285544	238410900	370	25	10164	0.26248	0.39648
+	SPG7	16	89473381	89567768	356	18	10164	0.26234	0.39653
-	MTMR11	1	149918650	149971898	31	6	10164	0.26209	0.39663
+	NFATC4	14	24331911	24389604	175	18	10164	0.26198	0.39667
-	ASB13	10	5628857	5701595	430	54	10164	0.2618	0.39674
+	ABCC1	16	15914577	16153074	597	49	10164	0.26172	0.39677
+	RAB36	22	23110326	23179285	159	6	10164	0.26163	0.39681
+	MORN2	2	38840962	38892709	167	23	10164	0.26144	0.39688
+	FAM89B	11	65537349	65584198	98	24	10164	0.26113	0.397
-	ZBBX	3	167168425	167415405	579	38	10164	0.26088	0.39709
-	GYS1	19	48958125	49028353	187	21	10164	0.26076	0.39714
+	HYAL4	7	123810169	123887481	100	14	10164	0.2607	0.39716
+	NKTR	3	42565655	42658741	205	14	10164	0.26047	0.39725
+	PACS2	14	105265811	105408147	53	21	10164	0.26023	0.39734
-	YWHAE	17	1334540	1435375	299	19	10164	0.25991	0.39747
-	CYP7A1	8	58480178	58535959	171	22	10164	0.25987	0.39748
-	ZBTB8OS	1	32610820	32686013	194	8	10164	0.25977	0.39752
+	SEMA4B	15	90149920	90239664	385	38	10164	0.2596	0.39759
-	CD300LB	17	74511174	74566474	212	20	10164	0.25915	0.39776
+	ATP6AP1L	5	82243665	82354260	239	22	10164	0.25879	0.3979
-	THUMPD2	2	39726060	39814276	79	15	10164	0.25874	0.39792
-	RAB30	11	82963133	83106923	367	37	10164	0.25846	0.39802
+	FAM149A	4	186109287	186182663	241	30	10164	0.25834	0.39807
+	SLAMF7	1	160704057	160764821	7	4	10164	0.25808	0.39817
+	PRAMEF12	1	12728223	12787906	150	9	10164	0.25758	0.39837
+	ZAK	2	173040837	173278009	742	40	10164	0.25716	0.39853
+	PHTF2	7	77763792	77967504	406	17	10164	0.25715	0.39853

STRAND	SYMBOL	CHR	START	STOP	NSNPS	NPARAM	N	ZSTAT	P
+	SMAD5	5	136097845	136192734	234	11	10164	0.25648	0.39879
+	NPR3	5	32654076	32801724	304	25	10164	0.25649	0.39879
+	GJC2	1	228114714	228169826	180	28	10164	0.25643	0.39881
-	MED7	5	157128440	157177910	129	11	10164	0.25611	0.39893
-	MYO9A	15	71812291	72153464	504	20	10164	0.25605	0.39896
+	ZFP69	1	40442220	40506343	165	33	10164	0.25588	0.39902
-	MRPS16	10	73238843	73287693	126	3	10164	0.25583	0.39904
+	MAP1LC3B	16	87357195	87414774	95	25	10164	0.25583	0.39904
-	DHX15	4	24517464	24619603	196	17	10164	0.25569	0.3991
+	IAPP	12	21337868	21389980	136	15	10164	0.25564	0.39911
+	LOC1053724	19	44707615	44758454	176	31	10164	0.25562	0.39912
-	ZNF668	16	31050843	31109320	74	5	10164	0.25539	0.39921
-	TTLL1	22	43029516	43124428	419	55	10164	0.25537	0.39922
+	NDUFB2	7	140661681	140716646	73	2	10164	0.25523	0.39927
-	RSPH14	22	23049408	23180230	259	12	10164	0.25505	0.39934
-	ZNF709	19	12451184	12519818	111	2	10164	0.25505	0.39934
-	SCO2	22	50513568	50561439	185	17	10164	0.2549	0.3994
+	MUC5B	11	1188065	1272176	224	34	10164	0.25487	0.39941
-	SUSD5	3	33140045	33254215	404	40	10164	0.25478	0.39945
-	HNRNPU	1	244840300	244899525	43	6	10164	0.25439	0.3996
+	FSHB	11	30195688	30245277	117	8	10164	0.25433	0.39962
-	PPP2CA	5	134186457	134261259	167	10	10164	0.25432	0.39962
+	PIP5K1B	9	68670217	69019176	1119	65	10164	0.25424	0.39966
-	PRTG	15	55601540	55778649	291	13	10164	0.25416	0.39969
+	ADSSL1	14	104689197	104757325	273	34	10164	0.25411	0.39971
+	SAPCD1	6	31727996	31774850	195	54	10164	0.25392	0.39978
-	SELE	1	169712640	169769079	397	19	10164	0.2539	0.39979
+	UTP18	17	51225536	51307933	199	15	10164	0.25363	0.39989
-	SOX21	13	94699622	94747543	104	13	10164	0.25332	0.40001
-	L3MBTL4	18	5944703	6449911	536	61	10164	0.25331	0.40001
-	ZFYVE28	4	2257524	2453690	805	58	10164	0.25301	0.40013
+	CMTM3	16	66569032	66623892	115	11	10164	0.25289	0.40017
+	FAM26F	6	116426370	116473782	142	24	10164	0.25281	0.40021
-	RIOK2	5	97150867	97218301	182	20	10164	0.25263	0.40028
+	C1orf50	1	42732245	42785742	114	16	10164	0.25235	0.40038
-	TRIM59	3	160425503	160484838	141	20	10164	0.25231	0.4004
-	GBX1	7	151136738	151202548	116	17	10164	0.25204	0.4005
+	EMC2	8	108408624	108496907	270	16	10164	0.25185	0.40058
+	CXXC5	5	139613716	139693882	99	14	10164	0.2518	0.4006
+	CTF1	16	30860828	30913560	48	3	10164	0.25175	0.40062
+	CCDC83	11	85820055	85930021	380	25	10164	0.25164	0.40066
+	ZNF628	19	55441332	55494487	150	30	10164	0.25158	0.40068
-	PHLDA1	12	76015447	76066776	65	12	10164	0.25157	0.40069
+	FARP1	13	98107562	98459773	968	118	10164	0.25135	0.40077
+	ZDHHC14	6	157346525	157688114	319	34	10164	0.25119	0.40083
-	CD79B	17	63918738	63967353	113	15	10164	0.25105	0.40089
-	RASAL1	12	113088819	113171239	167	10	10164	0.25097	0.40092
+	PLBD2	12	113323566	113399715	58	6	10164	0.25095	0.40093
+	MS4A13	11	60480376	60553424	283	22	10164	0.2509	0.40095
+	GADD45B	19	2441125	2488259	191	37	10164	0.2508	0.40099
-	SHMT1	17	18317860	18398563	244	22	10164	0.25038	0.40115
+	PDE4A	19	10381773	10479631	252	28	10164	0.25034	0.40116
-	CDC14B	9	96480218	96654830	185	25	10164	0.25017	0.40123
-	ZC3H12D	6	149437630	149520012	299	21	10164	0.25014	0.40124
-	CFAP70	10	73243758	73396064	374	4	10164	0.25008	0.40126
+	RAI1	17	17646473	17821453	382	44	10164	0.24993	0.40132
+	C16orf82	16	27031898	27079166	90	13	10164	0.24879	0.40176
+	CHPF2	7	151197489	151248827	85	9	10164	0.24867	0.40181
+	LOC1019293	13	114137978	114233074	444	24	10164	0.24852	0.40187
-	BCL11B	14	99159287	99306713	268	43	10164	0.24824	0.40197
-	MCRS1	12	49548294	49603225	92	9	10164	0.24802	0.40206
+	ARHGEF15	17	8275238	8332516	167	25	10164	0.24796	0.40208
-	CILP	15	65185999	65246502	64	13	10164	0.2477	0.40218

STRAND	SYMBOL	CHR	START	STOP	NSNPS	NPARAM	N	ZSTAT	P
+	PDSS1	10	26662424	26758564	518	32	10164	0.24769	0.40219
-	C14orf37	14	57983531	58187239	467	39	10164	0.24769	0.40219
-	MMP16	8	87993095	88362488	904	87	10164	0.24759	0.40222
-	RFX3	9	3208297	3561001	850	44	10164	0.24713	0.4024
-	DDX54	12	113147173	113220479	115	6	10164	0.24695	0.40247
-	DNAJC19	3	180973709	181024774	88	13	10164	0.24691	0.40249
-	LOC1027252	22	23996088	24043824	78	18	10164	0.24685	0.40251
+	VLDLR	9	2586679	2666100	282	42	10164	0.24678	0.40254
-	CSPG5	3	47552238	47615240	91	15	10164	0.24646	0.40266
+	SAMM50	22	43920381	44006532	249	15	10164	0.2461	0.4028
+	SLC7A2	8	17462088	17580568	481	75	10164	0.24541	0.40307
-	SLC2A5	1	9026943	9106704	329	31	10164	0.24525	0.40313
-	VPS39	15	42148701	42243326	272	16	10164	0.24502	0.40322
-	WT1	11	32377775	32470535	348	18	10164	0.24497	0.40324
+	DCAF15	19	13917507	13971444	45	13	10164	0.24491	0.40326
-	BATF2	11	64977945	65032653	124	22	10164	0.24426	0.40351
-	TM4SF19	3	196313546	196373420	127	15	10164	0.24424	0.40352
-	SRMS	20	63529924	63582504	189	41	10164	0.24356	0.40379
+	FXYD7	19	35108250	35164302	126	13	10164	0.24338	0.40385
-	ARHGAP12	10	31795398	31963876	314	14	10164	0.24303	0.40399
-	FARSB	2	222561443	222691355	323	21	10164	0.24294	0.40402
-	YIF1B	19	38293560	38350976	311	36	10164	0.24293	0.40403
+	OTUB2	14	93981095	94058930	260	30	10164	0.24276	0.4041
-	SLC25A35	17	8277764	8330400	151	23	10164	0.2423	0.40427
-	LTF	3	46426005	46500142	318	34	10164	0.24215	0.40433
+	ADGRV1	5	90523800	91174223	1720	82	10164	0.24201	0.40439
-	LOC1005065	1	2171811	2219360	131	31	10164	0.24201	0.40439
+	KRTAP20-1	21	30581455	30626625	150	14	10164	0.24184	0.40445
-	MOCS2	5	53085679	53144772	276	35	10164	0.24181	0.40446
-	LOC1053740	3	126422796	126470544	159	16	10164	0.24176	0.40448
+	TTR	18	31556767	31609024	172	25	10164	0.24081	0.40485
+	SYNJ2	6	157946856	158109176	517	44	10164	0.24052	0.40496
-	TMEM207	3	190418655	190484876	241	31	10164	0.24024	0.40507
+	ZNF831	20	59088428	59269113	255	29	10164	0.23984	0.40523
+	LHX9	1	197877505	197945476	112	20	10164	0.23982	0.40524
+	IFNL2	19	39230883	39280092	87	14	10164	0.23971	0.40528
-	SBK3	19	55530559	55580543	124	27	10164	0.23959	0.40533
+	CCRN4L	4	138980759	139055939	95	15	10164	0.23953	0.40535
+	BCCIP	10	125788535	125863695	224	25	10164	0.23952	0.40535
+	SCEL	13	77500674	77655263	397	33	10164	0.23929	0.40544
-	ZNF519	18	14046324	14167490	234	23	10164	0.23929	0.40544
+	EPHB6	7	142819643	142881093	145	8	10164	0.23894	0.40558
+	STK4	20	44931479	45089975	300	19	10164	0.23892	0.40559
+	GRIN3B	19	965438	1020223	314	29	10164	0.23879	0.40563
+	PFAS	17	8214255	8280491	224	32	10164	0.23875	0.40565
-	PRG3	11	57366769	57416150	88	18	10164	0.23869	0.40567
+	NPEPPS	17	47487950	47633276	215	16	10164	0.23851	0.40574
+	SLC30A5	5	69058949	69141072	245	16	10164	0.23848	0.40575
+	LOC1053782	12	80389259	80445269	116	8	10164	0.238	0.40594
+	ANKRD53	2	70942210	70996674	163	32	10164	0.23787	0.40599
-	ALDH1A1	9	72890662	72988317	187	28	10164	0.23766	0.40607
+	LRRC8A	9	128847112	128928042	164	18	10164	0.23757	0.40611
+	TMEM215	9	32748499	32799201	105	12	10164	0.23744	0.40616
+	MRPS17	7	55916918	55965341	147	22	10164	0.23742	0.40617
+	CAPN3	15	42324500	42422317	346	19	10164	0.23733	0.4062
-	SNRPG	2	70271378	70328737	172	18	10164	0.23731	0.40621
+	ARHGAP28	18	6694718	6925716	513	39	10164	0.23729	0.40622
-	E2F1	20	33665486	33721404	92	19	10164	0.23711	0.40629
+	RAPGEF2	4	159069071	159370162	562	42	10164	0.23702	0.40632
+	HPS3	3	149094584	149183518	263	27	10164	0.237	0.40633
-	KLHL35	11	75412394	75468444	101	16	10164	0.237	0.40633
-	NUP62	19	49896825	49964731	211	37	10164	0.23682	0.4064
+	CHRD	3	184345073	184399835	120	32	10164	0.2366	0.40648

STRAND	SYMBOL	CHR	START	STOP	NSNPS	NPARAM	N	ZSTAT	P
-	KIAA1598	10	116873377	117161586	381	27	10164	0.23656	0.4065
-	RPL36AL	14	49608688	49655685	180	13	10164	0.23628	0.40661
-	MED16	19	857962	928218	37	6	10164	0.23603	0.40671
-	GK5	3	142147527	142260607	290	33	10164	0.2359	0.40675
-	MRPL39	21	25575656	25642846	332	20	10164	0.23588	0.40676
-	GAL3ST3	11	66030765	66084180	145	23	10164	0.23577	0.4068
-	CD7	17	82304865	82352604	53	3	10164	0.23546	0.40692
-	LAMB1	7	107913801	108038359	188	26	10164	0.2354	0.40695
+	RRNAD1	1	156693471	156746960	93	21	10164	0.23527	0.407
-	LOC1053764	10	22215994	22281083	66	18	10164	0.23526	0.407
-	EIF2S2	20	34078309	34147279	100	12	10164	0.23516	0.40704
-	SEZ6	17	28944905	29041440	131	25	10164	0.23507	0.40708
-	LIPH	3	185496866	185587655	235	17	10164	0.23495	0.40712
-	ZNF846	19	9742145	9821177	148	12	10164	0.23488	0.40715
-	LOC1006528	3	28565591	28611491	64	10	10164	0.23477	0.40719
+	CTXN3	5	127614021	127668630	43	9	10164	0.23474	0.40721
+	TMCO5A	15	37899604	38005033	204	15	10164	0.23459	0.40726
-	EVA1A	2	75482318	75604722	334	44	10164	0.23439	0.40734
+	INF2	14	104654606	104729610	298	41	10164	0.23424	0.4074
-	RPE65	1	68418824	68484959	193	28	10164	0.23421	0.40741
+	C2orf70	2	26527556	26589529	131	16	10164	0.23422	0.40741
-	C11orf91	11	33688108	33735740	45	9	10164	0.23419	0.40742
-	RCBTB1	13	49521944	49620606	538	26	10164	0.234	0.40749
-	FOXN3	14	89146172	89654150	970	107	10164	0.23376	0.40758
-	CDKL2	4	75566494	75665537	241	20	10164	0.23344	0.40771
+	CHURC1-FN	14	64879361	65072655	584	42	10164	0.2334	0.40773
+	C12orf40	12	39591170	39731914	378	34	10164	0.2333	0.40776
+	LOC728763	2	240871359	241003307	514	34	10164	0.23279	0.40796
+	OR1F1	16	3156095	3215344	167	14	10164	0.23253	0.40806
+	B4GALNT4	11	334779	392117	194	24	10164	0.23246	0.40809
-	DKK2	4	106911802	107071296	355	48	10164	0.23207	0.40824
+	BATF	14	75487441	75556992	217	32	10164	0.2319	0.40831
+	CD53	1	110836199	110909936	243	15	10164	0.23161	0.40842
-	UTS2	1	7837612	7948610	389	36	10164	0.23145	0.40848
-	IGSF9	1	159917039	159980596	93	19	10164	0.23144	0.40849
+	NCMAP	1	24521076	24619328	370	32	10164	0.23132	0.40853
-	HIST2H3C	1	149830687	149876193	7	3	10164	0.23127	0.40855
+	HIST2H3A	1	149817619	149863125	7	3	10164	0.23127	0.40855
+	HIST2H2AA4	1	149816061	149861594	7	3	10164	0.23127	0.40855
-	HNF1B	17	37676431	37780078	307	72	10164	0.23106	0.40863
+	NPS	10	127514349	127562671	246	31	10164	0.23063	0.4088
-	MYOM1	18	3056807	3255108	496	62	10164	0.23036	0.40891
+	ATP12A	13	24645411	24721785	387	39	10164	0.23014	0.40899
-	ADAM12	10	126002385	126423558	1769	144	10164	0.23015	0.40899
-	TNFRSF10D	8	23125588	23199030	259	57	10164	0.23003	0.40903
+	EP300	22	41057610	41190077	311	24	10164	0.22988	0.40909
-	ADAMTS8	11	130394923	130463644	148	23	10164	0.22978	0.40913
+	GALR1	18	77215052	77280142	322	32	10164	0.22961	0.4092
+	ABCE1	4	145063004	145139524	56	9	10164	0.22947	0.40925
-	GNA12	7	2718105	2905656	829	52	10164	0.22944	0.40927
-	KLRC3	12	10402315	10455595	86	8	10164	0.22922	0.40935
-	RGS10	10	119489827	119577710	292	42	10164	0.22904	0.40942
+	BFAR	16	14597811	14679236	23	12	10164	0.22869	0.40956
-	OR2Y1	5	180729123	180775058	83	12	10164	0.22806	0.4098
+	IKBKE	1	206435243	206506890	184	26	10164	0.22785	0.40988
+	C8orf33	8	145017438	145075864	157	6	10164	0.22785	0.40988
+	ZSCAN21	7	100014767	100075042	92	12	10164	0.22776	0.40991
+	TPD52L1	6	125118729	125273498	442	33	10164	0.22776	0.40992
-	DZANK1	20	18373367	18502185	525	28	10164	0.22775	0.40992
+	FUT9	6	95980969	96225612	933	53	10164	0.22771	0.40994
+	KRTAP6-3	21	30557440	30603055	193	12	10164	0.22736	0.41007
-	PTPN22	1	113803811	113906757	216	29	10164	0.2269	0.41025
-	KCNMA1	10	76859601	77672819	2456	180	10164	0.22686	0.41027

STRAND	SYMBOL	CHR	START	STOP	NSNPS	NPARAM	N	ZSTAT	P
-	FAM180A	7	135719598	135783867	216	35	10164	0.22681	0.41028
-	LOC158434	9	96096661	96151411	152	26	10164	0.22675	0.41031
+	ANKEF1	20	9999999	10066764	195	16	10164	0.22666	0.41034
-	KLHDC4	16	87686569	87800997	479	33	10164	0.22661	0.41036
+	ADAMTS15	11	130413974	130486645	170	27	10164	0.22649	0.41041
+	SRPRB	3	133749033	133831492	229	26	10164	0.22624	0.41051
+	DOCK2	5	169602247	170093382	1536	129	10164	0.22581	0.41067
+	RGS21	1	192281992	192377285	183	13	10164	0.22572	0.41071
-	SF3B5	6	144084881	144130617	127	23	10164	0.22555	0.41078
-	ABLIM2	4	7955310	8193832	750	124	10164	0.22544	0.41082
-	FAM49A	2	16539459	16700866	387	56	10164	0.22539	0.41084
+	CENPN	16	80971498	81043107	344	13	10164	0.22486	0.41105
-	RND1	12	48847133	48900870	110	16	10164	0.22475	0.41109
+	DEGS1	1	224148208	224203441	174	21	10164	0.22471	0.4111
-	ATPAF1	1	46625039	46703427	255	17	10164	0.22473	0.4111
+	CNIH4	1	224321811	224389452	181	21	10164	0.22458	0.41115
+	ANXA3	4	78516588	78620451	331	27	10164	0.22424	0.41129
-	ECHDC2	1	52885910	52962212	148	20	10164	0.22417	0.41131
-	PRMT5	14	22910511	22964585	101	5	10164	0.22413	0.41133
+	COL3A1	2	188939373	189022746	205	27	10164	0.22365	0.41152
+	WWP1	8	86307736	86478265	405	16	10164	0.22363	0.41152
-	NKX2-1	14	36506397	36555225	108	17	10164	0.22361	0.41153
-	SEMA4C	2	96849736	96905943	151	28	10164	0.22358	0.41154
+	TSPAN18	11	44692130	44942427	852	102	10164	0.22356	0.41155
+	PCF11	11	83122095	83195794	122	18	10164	0.22324	0.41167
-	HTR7	10	90730818	90892914	425	22	10164	0.22314	0.41171
+	MRPS6	21	34038523	34153034	119	17	10164	0.2227	0.41188
+	GSE1	16	85135003	85686206	1170	141	10164	0.22259	0.41193
+	ECT2	3	172715682	172839265	240	12	10164	0.22252	0.41196
-	HAL	12	95962662	96031365	276	31	10164	0.22249	0.41197
-	HACD2	3	123484516	123620077	306	18	10164	0.22249	0.41197
+	TPBG	6	82328206	82377417	101	13	10164	0.22237	0.41201
-	KIN	10	7740962	7823027	359	38	10164	0.22237	0.41201
+	ZNF75A	16	3270406	3328851	66	7	10164	0.22234	0.41202
+	MYL4	17	47174013	47237697	124	24	10164	0.22233	0.41203
+	ZNF646	16	31038756	31093512	63	4	10164	0.22234	0.41203
+	LOC1019292	10	30014323	30121844	240	38	10164	0.22233	0.41203
+	DHRS2	14	23595115	23655639	12	4	10164	0.22195	0.41218
+	HOXC10	12	53950162	54000279	97	16	10164	0.22187	0.41221
+	SLC12A3	16	56830207	56925850	349	40	10164	0.22175	0.41226
+	LOR	1	153224635	153272125	170	18	10164	0.22152	0.41234
-	PKM	15	72189029	72266812	116	14	10164	0.22147	0.41236
-	ILF2	1	153651788	153706028	93	12	10164	0.22127	0.41244
-	FUT6	19	5820626	5874753	139	26	10164	0.22108	0.41251
+	COLCA2	11	111263546	111318735	163	21	10164	0.22108	0.41251
-	NEU1	6	31849052	31897932	257	60	10164	0.2209	0.41258
-	NTN4	12	95647807	95825787	656	50	10164	0.22068	0.41267
+	NAGS	17	43969546	44019068	102	16	10164	0.22069	0.41267
-	C4orf46	4	158656675	158707255	28	9	10164	0.22057	0.41271
-	ZNF600	19	52755471	52868628	144	15	10164	0.22048	0.41275
-	HECTD1	14	31090115	31243060	480	22	10164	0.22032	0.41281
-	RBBP9	20	18476540	18532255	202	19	10164	0.22019	0.41286
-	TRIM31	6	30092892	30148090	622	39	10164	0.22019	0.41286
-	LY6G6C	6	31708648	31756734	211	52	10164	0.22012	0.41289
+	HS6ST3	13	96055536	96849562	1662	79	10164	0.21993	0.41296
+	TMEM56	1	95057517	95207607	457	39	10164	0.21987	0.41299
-	TREH	11	118648232	118714690	271	13	10164	0.21948	0.41314
-	ADCY9	16	3943387	4151185	647	57	10164	0.21933	0.4132
+	ANKRD33	12	51853009	51901721	102	15	10164	0.2193	0.41321
-	TLR6	4	38813704	38892711	475	22	10164	0.21927	0.41322
+	PCYOX1L	5	149323007	149379658	78	9	10164	0.21922	0.41324
-	GLRA3	4	174614141	174864315	872	58	10164	0.21898	0.41333
+	CCR8	3	39294706	39343680	126	19	10164	0.21894	0.41335

STRAND	SYMBOL	CHR	START	STOP	NSNPS	NPARAM	N	ZSTAT	P
+	RPL36	19	5655261	5701667	94	11	10164	0.21834	0.41358
-	OR11A1	6	29415504	29462732	472	24	10164	0.2183	0.4136
-	LIPT2	11	74480727	74528719	131	13	10164	0.21827	0.41361
-	PLEKHJ1	19	2219952	2272405	138	26	10164	0.21804	0.4137
-	NCF2	1	183545562	183625921	283	28	10164	0.21781	0.41379
-	LOC1053765	11	10291745	10339753	120	18	10164	0.21778	0.4138
-	ROCK2	2	11169764	11380363	315	12	10164	0.21758	0.41388
+	C15orf65	15	55373525	55428712	216	19	10164	0.21737	0.41396
-	CFAP97	4	185141723	185244495	356	15	10164	0.2173	0.41399
+	ADGRG7	3	100574589	100705479	395	23	10164	0.21727	0.414
-	PCNA	20	5104953	5161622	85	11	10164	0.21719	0.41403
+	RITA1	12	113150526	113202368	83	5	10164	0.21716	0.41404
+	ZNF813	19	53432735	53504292	263	34	10164	0.21716	0.41404
-	COL10A1	6	116108909	116253169	497	30	10164	0.21711	0.41406
+	KCTD3	1	215532185	215631807	171	10	10164	0.21702	0.4141
-	PMM1	22	41566886	41624867	137	12	10164	0.21683	0.41417
-	GPATCH2	1	217416992	217666102	541	49	10164	0.21666	0.41424
+	CHRNA9	4	40300329	40365217	231	17	10164	0.21652	0.41429
-	NINJ1	9	93111489	93169304	191	30	10164	0.21641	0.41433
-	MOB3B	9	27315209	27564852	704	68	10164	0.21636	0.41435
+	KIAA0195	17	75406159	75510452	261	18	10164	0.21596	0.41451
-	CEP85L	6	118450772	118745082	780	35	10164	0.2156	0.41465
-	CALCRL	2	187331963	187483294	423	25	10164	0.21559	0.41466
-	CTBP2	10	124977849	125196055	976	116	10164	0.21482	0.41495
+	HSD11B1	1	209651180	209744950	225	31	10164	0.21445	0.4151
-	PROKR2	20	5289874	5352547	356	18	10164	0.21433	0.41514
+	ABCF3	3	184151075	184204452	98	13	10164	0.21426	0.41517
+	DHRS4L2	14	23934874	24016408	300	21	10164	0.21422	0.41519
-	MAGOH	1	53216892	53273610	118	20	10164	0.21417	0.41521
+	C17orf78	17	37340935	37402708	120	25	10164	0.21404	0.41526
-	LRRC25	19	18381137	18432625	115	17	10164	0.21401	0.41527
+	NDUFS4	5	53525623	53693341	614	29	10164	0.21361	0.41542
-	ZNF200	16	3212325	3271221	137	14	10164	0.21363	0.41542
+	LOC1009963	7	63721809	63767407	79	11	10164	0.21353	0.41546
-	MDP1	14	24203937	24251070	65	13	10164	0.21343	0.41549
+	LOC1019303	7	26363614	26472008	356	51	10164	0.21333	0.41553
-	CLIP3	19	36004660	36067895	115	19	10164	0.21328	0.41555
+	PPP5C	19	46311994	46400975	142	19	10164	0.21271	0.41578
-	KLK10	19	51002744	51055175	243	47	10164	0.21269	0.41578
+	C11orf57	11	112039244	112095150	70	12	10164	0.21261	0.41581
+	WDFY4	10	48649830	48992956	946	71	10164	0.21239	0.4159
+	HAGHL	16	691936	739715	128	17	10164	0.21238	0.41591
-	ROR2	9	91712596	91985162	982	56	10164	0.21235	0.41592
-	SPSB2	12	6860935	6908471	17	4	10164	0.21231	0.41593
-	CCDC79	16	66744411	66836620	157	9	10164	0.21217	0.41599
+	RAB4B	19	40743219	40806944	122	12	10164	0.21209	0.41602
-	LYG1	2	99274238	99339742	163	15	10164	0.21207	0.41603
-	FCRL4	1	157563749	157633080	292	18	10164	0.21204	0.41604
-	CACNG2	22	36550869	36737645	339	38	10164	0.21193	0.41608
+	DTX2	7	76426655	76515998	107	9	10164	0.21189	0.4161
-	KCNJ6	21	37607482	37954731	951	71	10164	0.2118	0.41613
+	PLEKHB1	11	73611178	73672819	155	24	10164	0.21168	0.41618
+	NOXA1	9	137388395	137444406	71	10	10164	0.21156	0.41623
+	FZD2	17	44522444	44571262	73	16	10164	0.21143	0.41628
+	HIAT1	1	100003078	100093377	155	11	10164	0.21128	0.41633
+	PKD2	4	87972639	88087779	299	23	10164	0.21124	0.41635
+	ZNRF3	22	28765654	29067488	974	54	10164	0.21095	0.41646
-	MFAP4	17	19373442	19422219	52	2	10164	0.21076	0.41654
-	DYNC1LI2	16	66710893	66786622	140	8	10164	0.21064	0.41659
+	PRAMEF9	1	13140281	13189132	14	1	10164	0.21055	0.41662
-	PRAMEF26	1	13138751	13188111	14	1	10164	0.21055	0.41662
-	HNRNPCL4	1	13154555	13200482	14	1	10164	0.21055	0.41662
+	SLC28A1	15	84849577	84985651	486	71	10164	0.21045	0.41666

STRAND	SYMBOL	CHR	START	STOP	NSNPS	NPARAM	N	ZSTAT	P
-	GPSM3	6	32180766	32230523	356	63	10164	0.21042	0.41667
-	FZD5	2	207752586	207804419	60	13	10164	0.21036	0.41669
+	DEFB131	4	9409534	9460514	91	14	10164	0.21038	0.41669
+	METTL13	1	171746621	171807716	197	16	10164	0.21002	0.41683
-	ELF2	4	139047717	139212218	266	25	10164	0.20987	0.41688
-	NES	1	156658763	156712397	126	23	10164	0.20972	0.41694
-	MYO18A	17	29063510	29215389	244	18	10164	0.20949	0.41703
+	NCR2	6	41300790	41360887	282	27	10164	0.20932	0.4171
+	LOC1053691	22	37951323	37998098	61	10	10164	0.20928	0.41711
+	PDCD6IP	3	33763571	33879707	369	19	10164	0.20916	0.41716
+	TRIM40	6	30099510	30158773	683	38	10164	0.20884	0.41728
-	EVX2	2	176066021	176120797	194	45	10164	0.20882	0.4173
-	ZNF518B	4	10429873	10492408	225	25	10164	0.20874	0.41732
-	IKBKAP	9	108857520	108969328	417	29	10164	0.2086	0.41738
+	PTCD3	2	86071182	86152157	199	15	10164	0.20859	0.41738
-	MYO1D	17	32482522	32912173	408	52	10164	0.20809	0.41758
-	SERPINB9	6	2877265	2938312	207	33	10164	0.20792	0.41764
-	LRRC29	16	67197139	67262059	89	13	10164	0.20786	0.41767
+	KLHL26	19	18602028	18680492	171	15	10164	0.20775	0.41771
+	CAMK1D	10	12314517	12845545	2464	231	10164	0.20775	0.41771
+	HBA1	16	141651	187522	111	21	10164	0.20744	0.41783
+	EXOSC2	9	130658760	130714894	140	16	10164	0.20717	0.41794
-	DGKG	3	186137201	186397234	117	20	10164	0.20703	0.41799
-	ROS1	6	117277918	117460922	698	59	10164	0.20701	0.418
+	CD86	3	122020362	122131143	226	42	10164	0.20652	0.41819
-	PROSER1	13	38999865	39073095	136	11	10164	0.2065	0.4182
-	ZFAT	8	134467788	134748049	842	44	10164	0.20616	0.41833
+	PDE6C	10	93577588	93675673	254	41	10164	0.20614	0.41834
+	PKIB	6	122436917	122736373	915	42	10164	0.20609	0.41836
-	HLTF	3	149020117	149121554	228	22	10164	0.20582	0.41847
-	LRRC6	8	132560416	132720097	207	26	10164	0.20578	0.41848
+	LOC1053740	3	126178254	126257330	410	15	10164	0.20511	0.41874
-	PLAGL2	20	32182504	32242743	56	7	10164	0.20502	0.41878
-	MROH5	8	141423829	141542230	496	51	10164	0.20498	0.41879
+	UNC5D	8	35200457	35806550	794	49	10164	0.20467	0.41892
+	RAB12	18	8574445	8649383	242	22	10164	0.20456	0.41896
-	KRTAP22-2	21	30580105	30625397	152	13	10164	0.20439	0.41902
+	ABL1	9	130678881	130897675	545	33	10164	0.20424	0.41908
+	DEPTOR	8	119838655	120060918	869	36	10164	0.20408	0.41915
+	STAM	10	17609125	17726824	449	23	10164	0.20381	0.41925
-	CALHM2	10	103436785	103487405	138	27	10164	0.20339	0.41941
-	C1orf123	1	53204099	53255639	114	22	10164	0.2032	0.41949
-	SIL1	5	138936720	139233376	391	16	10164	0.20319	0.41949
-	C18orf42	18	5133673	5233203	339	38	10164	0.20313	0.41952
-	LOC1001299	12	65841227	65918544	109	13	10164	0.2031	0.41953
+	NPR1	1	153643617	153703992	106	13	10164	0.20211	0.41991
+	LCE1A	1	152792473	152838097	76	14	10164	0.20203	0.41995
-	LCE1C	1	152794835	152841631	76	14	10164	0.20203	0.41995
-	CHKB	22	50568958	50617999	134	15	10164	0.20191	0.42
-	GNPDA1	5	141990669	142048055	104	22	10164	0.20179	0.42004
-	C9orf64	9	83928307	83992067	134	18	10164	0.20159	0.42012
-	WDR45B	17	82604562	82683535	249	30	10164	0.20149	0.42016
+	CUX1	7	101780904	102293958	1259	63	10164	0.20147	0.42017
+	DR1	1	93310921	93372592	144	14	10164	0.20134	0.42022
-	PITPNM1	11	67481767	67540372	103	22	10164	0.20122	0.42026
+	CCDC85B	11	65855404	65901635	118	19	10164	0.20106	0.42033
-	ANAPC10	4	144957117	145133541	149	20	10164	0.20102	0.42034
-	FAT2	5	151494092	151687140	659	67	10164	0.20079	0.42043
+	ZNF880	19	52334917	52395795	260	32	10164	0.20073	0.42046
+	SLC25A25	9	128033200	128119258	50	15	10164	0.20069	0.42047
-	HOXA7	7	27143716	27191677	89	19	10164	0.20066	0.42048
+	KAT8	16	31082664	31141393	74	5	10164	0.20064	0.42049
-	SPDEF	6	34527802	34591333	288	40	10164	0.20062	0.4205

STRAND	SYMBOL	CHR	START	STOP	NSNPS	NPARAM	N	ZSTAT	P
+	SNX1	15	64059076	64154234	112	6	10164	0.20038	0.42059
+	RB1	13	48268747	48491890	362	27	10164	0.19999	0.42074
-	ABCC4	13	95009829	95336446	1278	88	10164	0.19996	0.42076
+	TFAP2B	6	50782710	50857619	159	21	10164	0.19983	0.4208
-	GPN2	1	26869382	26925378	62	10	10164	0.19968	0.42086
+	ARL2	11	64979113	65032185	114	21	10164	0.19962	0.42089
-	CFL1	11	65844811	65893333	121	21	10164	0.19961	0.42089
-	ANKRD46	8	100499752	100594786	188	28	10164	0.19947	0.42095
-	SULT6B1	2	37157820	37223547	230	24	10164	0.19947	0.42095
-	DPEP3	16	67965663	68015549	83	12	10164	0.1993	0.42101
-	RPS23	5	82263320	82313416	112	14	10164	0.19914	0.42108
-	TPM3	1	154145304	154227135	109	19	10164	0.19908	0.4211
+	NHLRC3	13	39003311	39060109	115	10	10164	0.19908	0.4211
+	NBPF6	1	108386489	108481903	14	5	10164	0.19886	0.42119
+	ZNF592	15	84713561	84816432	255	30	10164	0.19859	0.42129
+	RAB2A	8	60481910	60633644	468	22	10164	0.19834	0.42139
+	EAF1	3	15392557	15452613	162	18	10164	0.1981	0.42148
+	HRCT1	9	35871192	35917141	142	24	10164	0.198	0.42152
-	CLN6	15	68196992	68264742	224	20	10164	0.19795	0.42154
-	CTC1	17	8214821	8283095	233	32	10164	0.19795	0.42154
-	PLAG1	8	56150909	56246314	160	23	10164	0.19793	0.42155
-	DCAF8	1	160205715	160297560	232	11	10164	0.19793	0.42155
+	PMM2	16	8762813	8865197	473	29	10164	0.19755	0.4217
+	CMYA5	5	79654836	79810226	455	55	10164	0.19753	0.42171
+	TEX22	14	105363583	105423859	22	11	10164	0.19753	0.42171
+	C17orf107	17	4864462	4915329	180	23	10164	0.1974	0.42176
+	FAM228B	2	24041874	24179638	429	20	10164	0.19738	0.42177
+	C15orf62	15	40734961	40782449	109	21	10164	0.19734	0.42178
+	TRHR	8	109051342	109129584	257	26	10164	0.19726	0.42181
+	NOV	8	119381312	119434438	182	14	10164	0.19724	0.42182
+	TCOF1	5	150322639	150410308	169	17	10164	0.19725	0.42182
-	RAB23	6	57176992	57257314	124	22	10164	0.19712	0.42187
+	GPR162	12	6786797	6837418	139	20	10164	0.1969	0.42195
-	NCALD	8	101676542	102159907	1380	98	10164	0.19687	0.42196
+	GDF15	19	18351158	18399176	142	20	10164	0.19682	0.42198
+	TMA16	4	163459289	163530539	193	27	10164	0.19649	0.42211
+	KLHDC7B	22	50513033	50561023	182	17	10164	0.1964	0.42215
+	CTSW	11	65844813	65893741	122	21	10164	0.19618	0.42223
+	ARPC5L	9	124827135	124887733	281	28	10164	0.19608	0.42227
+	PCBP3	21	45814961	45952454	491	25	10164	0.19586	0.42236
+	EFHC1	6	52385196	52505785	351	25	10164	0.1956	0.42246
+	GSTO1	10	104219194	104277464	140	24	10164	0.19559	0.42247
+	GCHFR	15	40729087	40777713	113	22	10164	0.19542	0.42253
+	PIP4K2C	12	57556159	57613428	119	19	10164	0.19534	0.42256
-	MCM6	2	135829626	135911477	209	20	10164	0.19529	0.42258
-	MLH3	14	75003764	75086532	169	11	10164	0.19524	0.4226
+	PSMD7	16	74261775	74316288	57	8	10164	0.19522	0.42261
+	ZFAND6	15	80024568	80148393	374	26	10164	0.19521	0.42261
+	FUK	16	70419584	70490274	53	16	10164	0.19509	0.42266
+	AMY2B	1	103519644	103589534	68	12	10164	0.19498	0.4227
+	IBA57	1	228130728	228207905	270	40	10164	0.19473	0.4228
+	ACR	22	50703224	50755299	41	9	10164	0.1946	0.42285
+	HCN2	19	554893	627159	190	21	10164	0.19461	0.42285
+	BMP3	4	80995622	81067531	157	13	10164	0.19461	0.42285
-	OR4C11	11	55593354	55639398	24	5	10164	0.19461	0.42285
+	SMIM18	8	30603600	30655956	103	14	10164	0.19447	0.4229
-	FAM20A	17	68524561	68635954	237	38	10164	0.1942	0.42301
+	CD52	1	26282920	26330523	153	11	10164	0.19407	0.42306
-	SEPT12	16	4767609	4823521	73	3	10164	0.19398	0.4231
+	LMNA	1	156047546	156150089	174	32	10164	0.19373	0.42319
-	DMXL2	15	51437724	51657833	655	25	10164	0.1937	0.4232
-	LOC1053691	1	223941266	224018034	88	7	10164	0.19361	0.42324
+	ATL1	14	50498082	50643068	183	13	10164	0.19351	0.42328

STRAND	SYMBOL	CHR	START	STOP	NSNPS	NPARAM	N	ZSTAT	P
+	AGAP1	2	235459089	236141800	1355	100	10164	0.19337	0.42334
+	CNEP1R1	16	49990206	50047088	124	13	10164	0.19336	0.42334
+	CXADR	21	17477906	17643323	475	36	10164	0.19272	0.42359
-	ATP5B	12	56628175	56681068	78	7	10164	0.19264	0.42362
-	PBOV1	6	138205986	138253490	128	13	10164	0.19263	0.42362
+	BPIFB4	20	33046512	33121751	271	24	10164	0.19256	0.42365
-	RNF168	3	196458783	196538768	227	18	10164	0.19257	0.42365
+	SLC22A18	11	2864721	2935246	158	35	10164	0.1922	0.42379
+	C1orf115	1	220655286	220709157	145	14	10164	0.19218	0.4238
-	XBP1	22	28784560	28835572	176	26	10164	0.19184	0.42393
+	KIF26A	14	104103723	104190898	87	15	10164	0.19183	0.42394
+	IL18RAP	2	102383558	102462568	318	15	10164	0.19175	0.42397
+	OR10H5	19	15759049	15804996	169	16	10164	0.19169	0.42399
+	CFLAR	2	201081154	201182688	247	44	10164	0.19156	0.42404
+	ADCYAP1R1	7	31017341	31121479	147	30	10164	0.19148	0.42407
+	POU2F3	11	120201640	120329945	296	38	10164	0.19142	0.4241
-	OR2T11	1	248616178	248662128	14	3	10164	0.19142	0.4241
+	OR9Q1	11	57988881	58191587	883	36	10164	0.19124	0.42417
+	RNF224	9	137192566	137239640	103	15	10164	0.19121	0.42418
+	PCDHGA4	5	141320025	141522979	406	31	10164	0.19088	0.42431
+	ARIH1	15	72439282	72596560	226	20	10164	0.19079	0.42434
-	ERRFI1	1	8001719	8061333	90	10	10164	0.19065	0.4244
-	DDT	22	23961365	24014828	81	15	10164	0.19055	0.42444
-	RUSC1-AS1	1	155310460	155359147	74	17	10164	0.19041	0.4245
-	MKX	10	27662874	27780849	348	27	10164	0.19029	0.42454
-	HES6	2	238228267	238275124	169	18	10164	0.19019	0.42458
+	LOC1027235	14	38221229	38396634	501	30	10164	0.19019	0.42458
-	KPTN	19	47465141	47521791	57	6	10164	0.19011	0.42461
+	GM2A	5	151218052	151280394	224	26	10164	0.18999	0.42466
-	C1QTNF2	5	160337754	160405652	148	21	10164	0.18985	0.42471
-	ANKFY1	17	4153371	4299008	323	17	10164	0.18976	0.42475
-	CD180	5	67171009	67231789	196	31	10164	0.18947	0.42486
-	PRPF19	11	60880547	60941589	101	6	10164	0.18944	0.42487
-	MAP4K5	14	50408480	50596126	179	14	10164	0.18926	0.42494
-	TECRL	4	64266298	64444494	297	9	10164	0.18928	0.42494
-	SPRYD7	13	49902702	50030962	264	26	10164	0.18894	0.42507
-	NXN	17	789313	1014767	879	104	10164	0.18879	0.42513
+	SLC25A18	22	17528165	17600890	203	40	10164	0.18848	0.42525
-	RAD21	8	116835934	116909866	211	15	10164	0.18838	0.42529
-	HDAC10	22	50235183	50286405	196	30	10164	0.18814	0.42538
+	RNF146	6	127231340	127298562	234	25	10164	0.18801	0.42543
-	RFTN2	2	197560800	197711045	189	11	10164	0.18786	0.42549
-	TNFSF4	1	173173731	173243072	152	18	10164	0.18779	0.42552
-	AP1S3	2	223745330	223872602	454	39	10164	0.18776	0.42553
+	CEBPB	20	50155583	50202690	169	15	10164	0.18769	0.42556
-	CTSC	11	88283592	88372773	230	23	10164	0.18761	0.42559
+	KLF17	1	44008927	44145140	497	46	10164	0.18731	0.42571
+	GTF2F2	13	45085464	45294889	451	29	10164	0.18715	0.42577
-	CEBPD	8	47726914	47773164	54	13	10164	0.18709	0.42579
-	WDR87	19	37874573	37941706	201	27	10164	0.18702	0.42582
+	LOC1053719	17	78213227	78262491	232	35	10164	0.18687	0.42588
-	MYOM3	1	24046041	24147175	317	39	10164	0.18684	0.42589
-	C5orf49	5	7820378	7886490	160	30	10164	0.18683	0.4259
-	CYP3A4	7	99746960	99819188	73	17	10164	0.18678	0.42591
-	HEMGN	9	97916791	97979915	130	15	10164	0.18641	0.42606
+	RHOQ	2	46507728	46594688	236	33	10164	0.18621	0.42614
+	ZNF470	19	56532081	56598903	185	19	10164	0.18568	0.42635
+	TRAPPC11	4	183624267	183723594	133	22	10164	0.1856	0.42638
+	C3orf35	3	37351269	37445497	135	16	10164	0.18555	0.4264
+	RNF112	17	19375790	19427276	59	2	10164	0.18553	0.42641
-	FGF6	12	4424142	4480614	159	22	10164	0.18529	0.4265
-	MCM7	7	100082728	100136940	98	17	10164	0.18506	0.42659
+	FAM222A	12	109679382	109780509	202	18	10164	0.18487	0.42666

STRAND	SYMBOL	CHR	START	STOP	NSNPS	NPARAM	N	ZSTAT	P
-	TLN1	9	35687337	35767395	133	30	10164	0.18458	0.42678
-	CATSPER1	11	66006752	66061519	185	27	10164	0.18452	0.4268
-	PDIA6	2	10773391	10872977	436	27	10164	0.18447	0.42682
+	FADD	11	70168163	70217402	160	22	10164	0.18442	0.42684
-	IGDCC4	15	65371480	65458072	162	19	10164	0.18427	0.4269
+	TNFRSF1B	1	12131949	12219222	234	33	10164	0.18418	0.42694
-	ZNF83	19	52602364	52725581	118	10	10164	0.18406	0.42698
+	DNHD1	11	6462296	6582025	350	28	10164	0.18406	0.42698
-	PPP1R3A	7	113866827	113954027	97	5	10164	0.18405	0.42699
-	HES7	17	8110590	8160739	147	33	10164	0.18399	0.42701
-	GC	4	71731693	71840520	340	34	10164	0.18398	0.42702
+	MBLAC1	7	100091694	100138724	95	17	10164	0.18393	0.42703
+	LOC1053714	1	149798133	149850737	17	4	10164	0.18391	0.42704
+	POLR2H	3	184326714	184378595	107	27	10164	0.18389	0.42705
+	RCN3	19	49492579	49553638	209	15	10164	0.18379	0.42709
-	KRT17	17	41609440	41659630	15	4	10164	0.18332	0.42727
-	GSC2	22	19138991	19185283	169	32	10164	0.18332	0.42728
+	SLIRP	14	77673071	77727598	231	21	10164	0.18321	0.42732
-	MSANTD2	11	124756492	124835436	216	32	10164	0.18316	0.42734
-	LIPE	19	42391507	42462426	61	11	10164	0.18314	0.42735
-	ZNF117	7	64964452	65026036	241	18	10164	0.18312	0.42735
+	LRRC7	1	69533773	70158192	1752	88	10164	0.18306	0.42737
+	ISL2	15	76300942	76352476	151	26	10164	0.18289	0.42744
-	RAB3A	19	18186799	18239064	140	21	10164	0.18284	0.42746
+	MFSD5	12	53216251	53264406	104	17	10164	0.18265	0.42753
-	TAGLN2	1	159908107	159960542	80	16	10164	0.18236	0.42765
+	LSM11	5	157708695	157770709	195	22	10164	0.18236	0.42765
-	EBPL	13	49650676	49726487	296	26	10164	0.18234	0.42766
-	CWF19L2	11	107316345	107492846	537	30	10164	0.18224	0.4277
-	TMEM205	19	11332776	11381414	17	4	10164	0.18202	0.42778
+	ZNF234	19	44106530	44170309	142	16	10164	0.18197	0.4278
-	ZNF426	19	9516551	9573645	258	4	10164	0.18166	0.42793
-	LOC1001303	17	81365498	81421797	106	15	10164	0.18161	0.42794
+	TARBP2	12	53465203	53516431	54	19	10164	0.18153	0.42797
+	RAC1	7	6339495	6413967	302	23	10164	0.18128	0.42807
+	CSF2RB	22	36878575	36950449	144	9	10164	0.18118	0.42811
+	KAT2B	3	20005032	20164404	644	46	10164	0.1812	0.42811
+	KPRP	1	152723030	152772053	112	14	10164	0.18112	0.42814
+	TFAP2E	1	35538370	35605328	124	12	10164	0.18106	0.42816
-	PFN4	2	24104809	24158477	191	18	10164	0.18091	0.42822
+	ACSM4	12	7269332	7338373	160	22	10164	0.18033	0.42845
-	TTN	2	178515989	178865754	700	46	10164	0.18026	0.42847
+	NLGN2	17	7373183	7429864	138	36	10164	0.1801	0.42854
+	KRTAP12-3	21	44622932	44668341	156	9	10164	0.17982	0.42865
-	FCMR	1	206893285	206957033	129	17	10164	0.17977	0.42867
-	METTL1	12	57758471	57807131	101	14	10164	0.17972	0.42868
-	OR52E6	11	5830956	5876897	266	10	10164	0.17962	0.42872
-	EIF3D	22	36500850	36564431	59	11	10164	0.17962	0.42873
-	KDF1	1	26939556	26995484	44	10	10164	0.17924	0.42887
-	LOC1053788	1	89890426	89941621	47	9	10164	0.17918	0.4289
+	DBNDD2	20	45370993	45420610	105	10	10164	0.17898	0.42898
+	COG6	13	39620627	39801665	395	13	10164	0.17883	0.42904
-	DGCR6L	22	20304238	20355105	23	9	10164	0.17878	0.42906
+	SLC22A12	11	64555810	64612353	136	24	10164	0.17853	0.42915
-	IFNA7	9	21191469	21237205	260	18	10164	0.17838	0.42921
-	FTCD	21	46126151	46190856	384	55	10164	0.17749	0.42956
+	ARL6IP6	2	152682893	152771253	186	20	10164	0.17728	0.42964
-	POC1A	3	52065233	52189690	199	25	10164	0.17723	0.42966
+	ZNF134	19	57579462	57632268	124	13	10164	0.17712	0.42971
+	EIF5B	2	99302371	99410265	267	21	10164	0.17712	0.42971
+	CARD14	17	80134992	80219331	300	31	10164	0.17705	0.42974
+	LOH12CR1	12	12322079	12479410	515	45	10164	0.177	0.42975
+	BPIFB3	20	33020331	33083637	217	22	10164	0.17697	0.42977

STRAND	SYMBOL	CHR	START	STOP	NSNPS	NPARAM	N	ZSTAT	P
+	TMEM25	11	118496088	118556598	91	9	10164	0.17685	0.42981
-	NLE1	17	35121322	35177315	179	31	10164	0.17653	0.42994
+	SSBP1	7	141703321	141760488	159	13	10164	0.17641	0.42998
-	TMEM194B	2	190444092	190578625	316	21	10164	0.17621	0.43007
-	IFNB1	9	21067105	21112944	187	41	10164	0.17614	0.43009
-	NID1	1	235965830	236100181	178	14	10164	0.17611	0.4301
+	SSBP4	19	18383661	18444562	119	16	10164	0.17585	0.43021
+	TRAPPC12	2	3344672	3489571	507	39	10164	0.17532	0.43041
+	C5orf30	5	103223741	103288660	108	9	10164	0.17523	0.43045
-	PCDH8	13	52833974	52883640	144	18	10164	0.17502	0.43053
+	ZNF620	3	40469989	40528221	98	13	10164	0.17476	0.43063
+	GAPT	5	58456435	58506358	221	27	10164	0.17473	0.43064
+	GEMIN7	19	45040739	45101524	120	12	10164	0.17423	0.43084
-	IL10	1	206757602	206809607	190	34	10164	0.17406	0.43091
+	GUCY1A3	4	155631568	155747062	300	29	10164	0.17369	0.43105
+	LOC1019273	6	871470	925491	227	27	10164	0.17361	0.43109
+	GRK6	5	177391496	177452849	60	9	10164	0.17346	0.43115
-	SLC6A4	17	30184319	30270968	136	20	10164	0.17341	0.43116
+	SLC29A3	10	71284253	71373396	349	41	10164	0.17342	0.43116
+	CASD1	7	94474858	94641872	398	38	10164	0.17291	0.43136
-	GHSR	3	172433291	172483456	161	21	10164	0.17286	0.43138
+	HABP4	9	96415155	96501336	169	12	10164	0.17267	0.43146
+	HNRNPA3	2	177177314	177233959	112	10	10164	0.17239	0.43157
-	MRVI1	11	10563091	10728988	236	20	10164	0.17233	0.43159
+	NIPSNAP3A	9	104712688	104770122	132	14	10164	0.17217	0.43165
+	HES3	1	6209158	6255578	81	11	10164	0.17216	0.43166
-	MPPED2	11	30374493	30621714	609	47	10164	0.17199	0.43172
-	CHUK	10	100176113	100264610	247	19	10164	0.17186	0.43177
+	CNPY4	7	100084642	100135508	94	16	10164	0.17177	0.43181
+	CDC6	17	40252689	40313161	48	4	10164	0.17123	0.43202
-	COPS2	15	49115274	49190657	227	21	10164	0.17095	0.43213
+	MERTK	2	111863479	112049953	364	13	10164	0.17087	0.43216
+	HKDC1	10	69185303	69277559	293	40	10164	0.17067	0.43224
-	BAK1	6	33562546	33615299	353	41	10164	0.17065	0.43225
+	SLC4A2	7	151023212	151086527	177	35	10164	0.17064	0.43225
+	C10orf71	10	49262056	49337492	218	26	10164	0.17063	0.43226
-	PRKAR2A	3	48734601	48882837	127	17	10164	0.17039	0.43235
+	PHOSPHO2	2	169659454	169711708	193	11	10164	0.16974	0.43261
-	CDC27	17	47107697	47224312	229	24	10164	0.16961	0.43266
-	LMBRD1	6	69665749	69832157	604	20	10164	0.16942	0.43273
-	RUFY2	10	68331107	68442294	223	11	10164	0.16936	0.43276
-	CYP3A7	7	99695037	99770200	159	10	10164	0.16928	0.43279
-	DPPA5	6	73343062	73389276	145	22	10164	0.16927	0.43279
-	LOC1002873	2	238834546	238882679	17	4	10164	0.16918	0.43283
+	PCGF3	4	670748	780640	308	32	10164	0.16916	0.43284
-	LOC1001321	9	64868467	64924049	4	2	10164	0.16895	0.43292
+	ZNF763	19	11930054	11990399	204	9	10164	0.16888	0.43295
-	SPSB3	16	1766712	1817605	380	26	10164	0.16861	0.43305
-	OTOF	2	26447203	26593698	228	35	10164	0.16837	0.43315
-	CEP70	3	138484339	138629383	162	5	10164	0.16836	0.43315
-	ZC3HC1	7	130008286	130086451	83	17	10164	0.16806	0.43327
-	VKORC1	16	31080854	31131337	68	5	10164	0.16803	0.43328
+	LOC1053769	19	36234531	36288351	144	14	10164	0.16801	0.43329
+	KPNA5	6	116646086	116751867	282	28	10164	0.16791	0.43333
+	MTX2	2	176234395	176348025	320	32	10164	0.16752	0.43348
-	WDR34	9	128623661	128691850	121	17	10164	0.16739	0.43353
+	PDPR	16	70078626	70172534	141	12	10164	0.1673	0.43357
-	ABHD11	7	73726094	73773885	51	6	10164	0.1672	0.43361
+	FSIP2	2	185703628	185843290	316	12	10164	0.16706	0.43366
+	DHX34	19	47314281	47392704	159	10	10164	0.16702	0.43368
-	NID2	14	51994802	52104231	418	27	10164	0.1667	0.4338
-	ARHGAP21	10	24573609	24758668	440	22	10164	0.16667	0.43382
-	SDE2	1	225972702	226034365	171	31	10164	0.1665	0.43388

STRAND	SYMBOL	CHR	START	STOP	NSNPS	NPARAM	N	ZSTAT	P
-	BRD4	19	15226836	15367543	96	10	10164	0.16633	0.43395
+	NR1H4	12	100438759	100573867	247	26	10164	0.16628	0.43397
+	CYYR1-AS1	21	26370943	26579263	372	66	10164	0.16627	0.43397
-	PRL	6	22277244	22337883	173	28	10164	0.16616	0.43401
-	ANK1	8	41643225	41931762	854	71	10164	0.16591	0.43411
-	SEC22C	3	42537967	42636080	224	16	10164	0.16584	0.43414
+	MTG1	10	133359117	133430670	117	12	10164	0.16577	0.43417
-	C9orf114	9	128809651	128864847	122	12	10164	0.16569	0.4342
-	NKIRAS1	3	23882081	23952046	288	24	10164	0.16557	0.43425
+	TPRG1L	1	3589992	3640131	3	2	10164	0.16553	0.43426
-	URGCP-MRF	7	43856558	43941632	244	25	10164	0.16547	0.43429
+	HHAT	1	210293252	210686298	1473	123	10164	0.16545	0.4343
+	ADGRF2	6	47621590	47707797	357	5	10164	0.16535	0.43433
+	RXFP3	5	33901386	33948918	165	24	10164	0.16534	0.43434
-	LOC1053734	1	149973141	150025952	41	9	10164	0.16526	0.43437
+	SRRM3	7	76131675	76297292	418	16	10164	0.16493	0.4345
+	DIS3L2	2	231926583	232353968	787	32	10164	0.16491	0.43451
+	EFCAB9	5	172153430	172213454	130	14	10164	0.1648	0.43455
+	MAPK8IP2	22	50565685	50621551	145	16	10164	0.16469	0.43459
-	DOK4	16	57461958	57521473	128	22	10164	0.16469	0.43459
-	KIF18A	11	28010616	28143199	190	19	10164	0.16423	0.43477
+	SNRPE	1	203826585	203881152	193	18	10164	0.16401	0.43486
+	LRAT	4	154591961	154763119	218	18	10164	0.16387	0.43492
+	KLF5	13	73019976	73087542	196	32	10164	0.16353	0.43505
-	CAPG	2	85384748	85453467	232	32	10164	0.16353	0.43505
+	MRPL35	2	86164355	86223794	183	13	10164	0.16331	0.43514
-	AOAH	7	36502941	36759549	658	72	10164	0.16304	0.43524
-	ANP32A	15	68768535	68855922	148	25	10164	0.16287	0.43531
-	TRADD	16	67144185	67194909	51	13	10164	0.16286	0.43531
+	DPY19L4	8	94684875	94803848	389	24	10164	0.16256	0.43543
-	CPVL	7	28985631	29181537	622	61	10164	0.16252	0.43545
+	GLB1L3	11	134241322	134331361	203	27	10164	0.16242	0.43549
-	GPR171	3	151187754	151238222	147	21	10164	0.16238	0.4355
-	IMMT	2	86133932	86230770	273	19	10164	0.16226	0.43555
-	MPP7	10	28040993	28338061	1253	38	10164	0.16222	0.43557
-	LOC1019269	2	9639308	9700708	99	7	10164	0.16216	0.43559
-	TOMM34	20	44932130	44995473	110	17	10164	0.16208	0.43562
-	KCNJ11	11	17354824	17424331	233	33	10164	0.16192	0.43569
+	SNAP29	22	20824004	20901214	6	2	10164	0.16181	0.43573
-	RAD51D	17	35089792	35154869	164	35	10164	0.16174	0.43576
-	MCOLN2	1	84915583	85032122	335	19	10164	0.16163	0.4358
+	C1QTNF7	4	15304818	15456169	480	30	10164	0.1615	0.43585
+	GRK7	3	141714984	141827370	336	26	10164	0.16147	0.43586
-	SERTAD3	19	40430841	40479375	118	14	10164	0.16144	0.43587
+	DEC1	9	115077617	115412644	1397	77	10164	0.16115	0.43599
-	GPRC6A	6	116782085	116864057	224	25	10164	0.16103	0.43604
+	LOC1053730	22	50571999	50620205	126	14	10164	0.16094	0.43607
+	LOC1027241	17	42836312	42884494	74	11	10164	0.16054	0.43623
+	PRLH	2	237531574	237577175	182	25	10164	0.16018	0.43637
+	FAM86KP	4	9118296	9171952	122	7	10164	0.16017	0.43637
-	CFL2	14	34700382	34749823	199	18	10164	0.16011	0.4364
-	ZSCAN12	6	28368821	28434767	291	27	10164	0.15996	0.43645
-	ETV1	7	13881229	14026425	505	62	10164	0.15978	0.43653
-	RAD18	3	8867196	8998475	554	25	10164	0.15978	0.43653
-	RAVER1	19	10306212	10368638	199	28	10164	0.15967	0.43657
-	PEX19	1	160266809	160320151	97	8	10164	0.15957	0.43661
+	WDR18	19	949328	1004570	251	26	10164	0.15947	0.43665
+	TNKS	8	9520214	9792346	1172	33	10164	0.15943	0.43666
-	RHOC	1	112691127	112742403	97	16	10164	0.15929	0.43672
+	NCR1	19	54870992	54948208	288	28	10164	0.15917	0.43677
-	KLF10	8	102638777	102690902	149	26	10164	0.15855	0.43701
-	LOC1053702	13	24528602	24576913	209	15	10164	0.15845	0.43705
+	FOXD2	1	47401017	47450691	126	18	10164	0.15806	0.43721

STRAND	SYMBOL	CHR	START	STOP	NSNPS	NPARAM	N	ZSTAT	P
+	FGF11	17	7403273	7454932	122	30	10164	0.15786	0.43729
-	FBXO32	8	123487887	123576253	252	48	10164	0.15748	0.43743
+	B3GNT4	12	122168681	122218135	132	21	10164	0.15739	0.43747
+	SKIL	3	170322678	170406849	302	30	10164	0.15715	0.43756
-	WDR53	3	196544183	196603610	101	13	10164	0.1571	0.43758
+	KCNQ5	6	72586280	73208851	1786	135	10164	0.15696	0.43764
+	DBI	2	119331924	119382551	150	10	10164	0.15692	0.43765
-	ZNF334	20	46471908	46548581	139	16	10164	0.15692	0.43765
-	AKT2	19	40220317	40320531	188	20	10164	0.15692	0.43766
-	ARNT	1	150799705	150911768	168	19	10164	0.1568	0.4377
-	BCLAF1	6	136246863	136325645	146	13	10164	0.15674	0.43773
+	ESRRA	11	64270528	64326743	178	31	10164	0.15662	0.43777
+	SCD	10	100312015	100374831	153	26	10164	0.1563	0.4379
-	PRELID2	5	145327932	145870383	1552	53	10164	0.15626	0.43791
-	HS3ST3A1	17	13484032	13636974	504	47	10164	0.15594	0.43804
+	ITIH3	3	52759768	52819009	171	19	10164	0.15591	0.43805
+	HMCN2	9	130230759	130444123	500	69	10164	0.15573	0.43812
+	TRIM15	6	30128206	30182696	486	29	10164	0.15535	0.43827
+	ENDOD1	11	95054810	95142651	174	20	10164	0.15532	0.43828
-	CFAP53	18	50217193	50301495	235	26	10164	0.15528	0.4383
+	HM13	20	31479410	31579567	38	18	10164	0.15511	0.43837
+	LOC1053691	8	144965545	145045864	219	12	10164	0.15511	0.43837
+	CCDC138	2	108751750	108886769	222	13	10164	0.15504	0.4384
+	TGIF1	18	3376927	3468411	119	24	10164	0.15433	0.43867
+	GPR137	11	64234260	64299500	196	34	10164	0.15431	0.43868
+	TSACC	1	156302314	156356995	132	12	10164	0.15424	0.43871
-	KLHL1	13	69690593	70143493	1692	71	10164	0.15413	0.43875
-	CACNA2D2	3	50352613	50539244	363	27	10164	0.15405	0.43878
+	C1orf146	1	92182912	92255812	89	9	10164	0.15403	0.43879
-	CRNN	1	152399243	152449274	136	15	10164	0.15378	0.43889
+	KCNV2	9	2682239	2740037	253	41	10164	0.15373	0.43891
-	OR9A2	7	142987851	143062284	60	2	10164	0.15368	0.43893
+	FOXF1	16	86475527	86524464	275	47	10164	0.15364	0.43895
-	VNN3	6	132712787	132769765	183	19	10164	0.15347	0.43902
-	ERH	14	69370123	69433304	149	17	10164	0.15293	0.43923
+	KLRF2	12	9846489	9905833	155	17	10164	0.1524	0.43943
-	LMNB2	19	2417907	2491968	301	37	10164	0.15191	0.43963
-	PTPRN2	7	157529056	158622796	3442	229	10164	0.15183	0.43966
-	FOSL1	11	65882136	65935526	144	24	10164	0.15178	0.43968
-	C16orf93	16	30747423	30797277	12	3	10164	0.151	0.43999
+	KCNH3	12	49504041	49568308	101	14	10164	0.15074	0.44009
+	C10orf53	10	49644638	49718910	256	34	10164	0.15034	0.44025
+	SIRT5	6	13539274	13625158	137	22	10164	0.15003	0.44037
-	PGAP3	17	39661122	39723070	120	9	10164	0.14981	0.44046
-	IL1A	2	112763915	112820394	143	11	10164	0.14948	0.44059
-	RASEF	9	82969585	83098177	227	22	10164	0.14933	0.44065
-	GIMAP6	7	150615375	150667648	233	21	10164	0.1491	0.44074
+	FAM135A	6	70378404	70571174	561	21	10164	0.14906	0.44075
-	VPS33B	15	90988416	91057621	250	36	10164	0.14905	0.44076
+	RBP3	10	47313371	47367875	28	11	10164	0.14898	0.44078
+	DOK7	4	3428306	3511476	401	40	10164	0.149	0.44078
+	CCSER1	4	90092394	91612219	4543	181	10164	0.14897	0.44079
-	F12	5	177392138	177451524	58	9	10164	0.14885	0.44084
+	ORAI3	16	30914084	30964938	48	4	10164	0.14848	0.44098
-	CTNND2	5	10961840	11939043	2706	207	10164	0.14835	0.44104
-	FAM168A	11	73390478	73633189	424	22	10164	0.14792	0.4412
-	RIMS4	20	44741804	44845338	139	24	10164	0.14787	0.44122
-	FBXL4	6	98863725	98983006	230	23	10164	0.14771	0.44129
-	ZHX1-C8orf7	8	123216189	123309487	41	21	10164	0.14753	0.44136
-	PDCL2	4	55546523	55627268	234	15	10164	0.14744	0.44139
-	ST3GAL2	16	70369435	70474088	82	22	10164	0.14738	0.44141
-	B3GNT9	16	67138102	67185999	47	12	10164	0.14708	0.44153
-	ZNF280A	22	22503736	22555281	39	4	10164	0.14697	0.44158

STRAND	SYMBOL	CHR	START	STOP	NSNPS	NPARAM	N	ZSTAT	P
+	CNP	17	41931741	41987736	77	9	10164	0.1469	0.44161
+	TRIML1	4	188103189	188157743	51	4	10164	0.14682	0.44164
+	SQLE	8	124963478	125032284	141	14	10164	0.14649	0.44177
-	ZNF443	19	12419706	12492521	87	2	10164	0.14633	0.44183
+	DRAP1	11	65884257	65931577	127	20	10164	0.14621	0.44188
-	ADAM10	15	58585204	58784978	373	16	10164	0.14583	0.44203
-	P3H2	3	189946728	190157437	672	41	10164	0.14568	0.44209
-	ZNF195	11	3347927	3414222	1	1	10164	0.1454	0.4422
-	ADAMTS5	21	26907912	27002120	185	19	10164	0.14512	0.44231
-	ALKBH1	14	77662406	77743032	338	19	10164	0.14502	0.44235
+	LOC1009964	7	63719798	63765530	61	9	10164	0.14497	0.44237
-	TMEM203	9	137194082	137240638	107	15	10164	0.14482	0.44243
-	KLRK1	12	10362353	10425054	73	7	10164	0.14475	0.44246
-	GLMN	1	92236398	92476946	291	24	10164	0.14455	0.44253
+	IKZF1	7	50268465	50415101	358	47	10164	0.14449	0.44256
+	LOC1053703	13	100638259	100686985	112	11	10164	0.14444	0.44258
-	CHIC2	4	53998373	54100427	130	14	10164	0.14429	0.44264
-	WDSUB1	2	159225793	159321793	394	22	10164	0.14413	0.4427
-	LOC1005062	5	21471307	21519694	60	9	10164	0.14411	0.44271
+	BNIPL	1	151001553	151057600	108	20	10164	0.14407	0.44272
+	TIGD2	4	89077817	89124901	191	16	10164	0.14379	0.44283
+	KRTAP10-11	21	44611414	44657650	135	12	10164	0.14327	0.44304
-	SIM1	6	100375009	100499929	291	35	10164	0.14311	0.4431
+	TOPAZ1	3	44206886	44346610	249	8	10164	0.14299	0.44315
+	SEC24B	4	109398772	109550459	326	14	10164	0.14296	0.44316
+	HIST2H4A	1	149797657	149843052	10	3	10164	0.14267	0.44328
-	SCAI	9	124932608	125178559	614	27	10164	0.1426	0.4433
+	GPIHBP1	8	143178193	143227170	187	25	10164	0.14257	0.44331
-	EPB41L4B	9	109161974	109355964	532	68	10164	0.14247	0.44335
+	ETAA1	2	67362310	67420413	144	12	10164	0.14212	0.44349
-	TRIML2	4	188081255	188144603	52	4	10164	0.14213	0.44349
-	PAQR9	3	142951231	142998336	19	3	10164	0.14209	0.4435
-	LOC1019297	1	189256081	189370533	210	3	10164	0.14207	0.44351
+	NBPF19	1	149428737	149566256	7	2	10164	0.14196	0.44356
-	RASL10A	22	29302933	29354618	109	25	10164	0.14182	0.44361
-	SLC25A17	22	40759630	40854399	203	29	10164	0.14179	0.44362
-	CCT4	2	61858127	61923671	168	19	10164	0.14176	0.44363
+	BDP1	5	71420383	71591062	359	14	10164	0.1416	0.4437
+	CDK1	10	60743331	60804852	195	22	10164	0.14147	0.44375
-	CATSPERB	14	91570774	91767086	489	48	10164	0.14109	0.4439
+	ZFYVE16	5	80372592	80490520	355	17	10164	0.14091	0.44397
+	NPIPB5	16	22478523	22546535	3	1	10164	0.14063	0.44408
-	CCT3	1	156298961	156373415	189	19	10164	0.14059	0.4441
+	LOC1053765	10	8011827	8060862	189	46	10164	0.14045	0.44415
+	PCDHGC5	5	141454241	141522979	153	19	10164	0.14039	0.44417
-	ZFYVE1	14	72959445	73062212	416	26	10164	0.14022	0.44424
+	CHRNB1	17	7410087	7467613	144	29	10164	0.14017	0.44426
+	PLEKHG1	6	150564863	150853665	1153	102	10164	0.14016	0.44426
-	LOC1019278	11	65786550	65868497	277	38	10164	0.14016	0.44427
-	PRPF40A	2	152641593	152752916	241	18	10164	0.14013	0.44428
+	TSSC4	11	2365445	2413878	189	17	10164	0.1398	0.44441
+	TSFM	12	57747745	57812856	128	16	10164	0.13944	0.44455
-	LRRC4C	11	40099938	41495212	3606	160	10164	0.13932	0.4446
+	TBCEL	11	120989094	121099647	142	10	10164	0.13887	0.44478
-	C15orf56	15	40240664	40287909	136	30	10164	0.13882	0.4448
+	SPRYD4	12	56433517	56480985	89	11	10164	0.13873	0.44483
+	SLC1A1	9	4455427	4597469	392	47	10164	0.13864	0.44487
+	SERF1B	5	69990251	70053108	2	1	10164	0.13859	0.44489
+	LOC1027243	5	69944017	70483520	2	1	10164	0.13859	0.44489
-	MXD4	4	2237432	2297143	164	20	10164	0.13815	0.44506
+	NXPH4	12	57181795	57236449	130	18	10164	0.13814	0.44507
+	ENO2	12	6879450	6933696	12	6	10164	0.13796	0.44514
-	TH	11	2153929	2207137	235	29	10164	0.13786	0.44517

STRAND	SYMBOL	CHR	START	STOP	NSNPS	NPARAM	N	ZSTAT	P
-	GABRB2	5	161278419	161583124	856	57	10164	0.13759	0.44528
+	ART1	11	3607234	3675075	71	12	10164	0.13743	0.44535
-	PSMG3	7	1557332	1605493	76	11	10164	0.13742	0.44535
-	ART5	11	3628506	3677316	71	12	10164	0.13743	0.44535
+	TFAP2D	6	50678544	50783033	164	19	10164	0.13733	0.44538
-	SSUH2	3	8580122	8780035	391	74	10164	0.13723	0.44543
-	FAM110C	2	28814	82115	74	5	10164	0.1372	0.44544
-	TAS2R1	5	9618997	9665351	88	15	10164	0.13711	0.44547
-	BLVRB	19	40437784	40500818	134	21	10164	0.13709	0.44548
-	BCL2L10	15	52099269	52148042	175	20	10164	0.13683	0.44558
+	SMIM22	16	4753391	4806491	86	5	10164	0.13669	0.44564
-	MPHOSPH6	16	82138161	82205224	416	17	10164	0.13659	0.44568
+	SEPT11	4	76914742	77050154	351	33	10164	0.13656	0.44569
+	WFS1	4	6234850	6313265	217	8	10164	0.13641	0.44575
+	DCTN5	16	23606366	23683747	227	18	10164	0.1362	0.44583
+	TEK	9	27074141	27240178	757	70	10164	0.13618	0.44584
+	GRIA4	11	105574994	105992092	814	40	10164	0.13612	0.44586
+	LCE2C	1	152640315	152686574	104	5	10164	0.13612	0.44586
+	ACYP2	2	54080273	54315300	778	45	10164	0.1361	0.44587
+	LOC1053767	16	89197741	89244453	277	24	10164	0.13609	0.44588
+	LOC1053743	4	3610103	3663896	2	1	10164	0.13594	0.44594
+	RELT	11	73341360	73407474	118	10	10164	0.13591	0.44595
+	CDX1	5	150131781	150194558	184	34	10164	0.13563	0.44606
-	TMEM238	19	55369244	55419259	171	28	10164	0.13557	0.44608
-	ARPP19	15	52537187	52604221	174	27	10164	0.13547	0.44612
-	RPAP1	15	41507176	41579277	191	22	10164	0.13525	0.44621
+	FAM21A	10	50032888	50143509	231	25	10164	0.13519	0.44623
-	ZNF699	19	9283925	9344766	107	7	10164	0.13489	0.44635
-	GALC	14	87923014	88028665	606	25	10164	0.13483	0.44637
-	GOLGA8K	15	32379585	32438292	1	1	10164	0.13477	0.4464
-	SLCO4C1	5	102223986	102331549	408	23	10164	0.13459	0.44647
-	CCDC69	5	151171052	151259093	318	38	10164	0.13421	0.44662
-	PNMA2	8	26494679	26548967	189	19	10164	0.13401	0.4467
+	AGL	1	99815077	99934023	328	25	10164	0.13397	0.44671
+	TMEM132A	11	60889441	60947159	104	6	10164	0.13396	0.44672
+	TAAR6	6	132535322	132581359	185	29	10164	0.13388	0.44675
+	NFIA	1	61042257	61472788	856	108	10164	0.13384	0.44676
-	PPA1	10	70192830	70268434	235	30	10164	0.13374	0.4468
+	TMEM92	17	50236406	50291485	9	4	10164	0.13359	0.44686
+	C16orf45	16	15399468	15604689	325	23	10164	0.13343	0.44693
+	NT5M	17	17268366	17357663	161	14	10164	0.13327	0.44699
+	MARC1	1	220751697	220824399	524	17	10164	0.13324	0.447
+	CCDC85A	2	56149123	56396174	737	74	10164	0.13308	0.44707
-	EFEMP2	11	65856441	65907934	135	22	10164	0.13302	0.44709
-	ALS2CR12	2	201278271	201392398	365	29	10164	0.13303	0.44709
-	CDC26	9	113257008	113310589	140	15	10164	0.13282	0.44717
+	PCDHGA1	5	141295685	141522979	497	37	10164	0.13258	0.44726
+	TMEM38B	9	105659525	105786611	320	10	10164	0.13252	0.44729
-	RARRES1	3	158686892	158767691	246	24	10164	0.13204	0.44748
+	MS4A1	11	60420809	60480752	207	29	10164	0.13202	0.44749
-	POLR2D	2	127836266	127893155	137	15	10164	0.13176	0.44759
+	ZNF213	16	3100056	3152804	86	6	10164	0.13174	0.44759
+	MELK	9	36537862	36687683	36	15	10164	0.1316	0.44765
-	LOC1053794	11	73570220	73683096	245	37	10164	0.13156	0.44767
-	OR2AT4	11	75078751	75124713	130	12	10164	0.13133	0.44776
-	CCDC168	13	102719367	102794072	168	19	10164	0.13105	0.44787
+	CEACAM6	19	41720421	41782211	94	10	10164	0.13043	0.44811
-	ETFA	15	76206287	76346469	366	27	10164	0.13026	0.44818
-	PNKP	19	49851203	49902565	139	24	10164	0.13012	0.44823
+	CST8	20	23456076	23517128	184	12	10164	0.13004	0.44827
-	DCUN1D1	3	182928158	183020947	141	19	10164	0.13003	0.44827
+	LOC1053777	8	4493043	4547829	285	46	10164	0.12987	0.44833
-	OR6T1	11	123932432	124028780	514	28	10164	0.12977	0.44837

STRAND	SYMBOL	CHR	START	STOP	NSNPS	NPARAM	N	ZSTAT	P
+	ALMS1	2	73350758	73619919	45	5	10164	0.12972	0.44839
+	ITPKC	19	40681980	40750860	103	11	10164	0.12969	0.44841
-	LMBR1	7	156658946	156928208	919	28	10164	0.12958	0.44845
-	CDKN2D	19	10556462	10603979	188	22	10164	0.12955	0.44846
-	FAM13A	4	88715954	89146398	1267	39	10164	0.12949	0.44848
+	ZNF716	7	57415177	57483559	401	10	10164	0.1293	0.44856
-	AHCTF1	1	246829098	246966978	269	23	10164	0.12867	0.44881
-	CISH	3	50596454	50646831	93	13	10164	0.12831	0.44895
-	C5orf42	5	37055171	37284428	278	23	10164	0.12817	0.44901
+	C3orf14	3	62283973	62346213	61	16	10164	0.12812	0.44903
-	FAM204A	10	118295889	118377327	84	14	10164	0.12798	0.44908
-	AGAP9	10	47491854	47558638	1	1	10164	0.12783	0.44914
-	ZNF33B	10	42579107	42673836	216	19	10164	0.12777	0.44916
-	FTH1	11	61954285	62002660	145	19	10164	0.12746	0.44929
+	NEU3	11	74953905	75017698	87	11	10164	0.12726	0.44937
-	DCT	13	94427304	94585265	470	24	10164	0.12716	0.44941
+	KIAA1467	12	13009381	13093449	217	25	10164	0.12691	0.44951
-	PDK4	7	95573497	95631613	178	15	10164	0.12684	0.44953
+	RNF10	12	120499308	120587594	318	20	10164	0.12655	0.44965
-	NHP2L1	22	41663933	41724292	157	11	10164	0.12642	0.4497
-	FOXD1	5	73436256	73483527	59	5	10164	0.12636	0.44973
-	FAM214A	15	52571321	52744817	498	52	10164	0.12634	0.44973
+	DDX19A	16	70311861	70383383	64	19	10164	0.126	0.44987
-	DLG1	3	197032560	197334272	447	14	10164	0.12583	0.44993
+	CCDC30	1	42428318	42664664	679	19	10164	0.12584	0.44993
-	TNFRSF25	1	6451151	6501195	76	10	10164	0.12581	0.44994
-	SOWAHB	4	76884929	76932849	133	17	10164	0.12575	0.44996
-	ADCK4	19	40681529	40751960	109	11	10164	0.12568	0.44999
-	OSBPL7	17	47797367	47856826	204	30	10164	0.12567	0.45
+	BLOC1S6	15	45552219	45619711	122	13	10164	0.12541	0.4501
+	FZR1	19	3471065	3548539	1	1	10164	0.1253	0.45014
-	DOHH	19	3480821	3540918	1	1	10164	0.1253	0.45014
-	IFNA17	9	21217243	21263222	215	15	10164	0.12496	0.45028
-	FKBP15	9	113155519	113256361	332	26	10164	0.1249	0.4503
-	PYGL	14	50895217	50979530	318	34	10164	0.12452	0.45045
+	PGS1	17	78343618	78434659	244	23	10164	0.12435	0.45052
+	C1orf56	1	151012783	151061420	96	17	10164	0.1242	0.45058
-	QRFP	9	130883428	130928838	150	19	10164	0.12419	0.45058
+	PCDHGB1	5	141315261	141522979	422	32	10164	0.12415	0.4506
+	C10orf12	10	96946284	96995828	27	8	10164	0.12409	0.45062
-	LOC1053697	12	9637014	9688535	179	15	10164	0.12403	0.45065
-	B9D1	17	19325263	19413182	88	3	10164	0.12397	0.45067
-	LOC1053781	6	167347356	167406630	242	38	10164	0.12379	0.45074
+	FOXL1	16	86543509	86591698	198	30	10164	0.12371	0.45077
+	TULP3	12	2855867	2951140	294	9	10164	0.12363	0.45081
+	TADA2A	17	37371877	37487481	268	22	10164	0.12333	0.45092
-	RDH14	2	18544723	18595693	92	14	10164	0.12335	0.45092
-	PRICKLE1	12	42448338	42624770	695	49	10164	0.12335	0.45092
+	OR2M2	1	248144986	248191029	81	7	10164	0.12323	0.45096
-	AURKB	17	8194731	8245776	153	26	10164	0.12304	0.45104
+	ZBTB9	6	33419579	33467544	147	35	10164	0.12265	0.45119
-	ZNF689	16	30592558	30645775	38	3	10164	0.12257	0.45122
+	IL21R	16	27367162	27462042	240	32	10164	0.12255	0.45123
-	SEC11A	15	84659537	84751460	216	29	10164	0.12235	0.45131
+	SH2B2	7	102250059	102331711	205	36	10164	0.12217	0.45138
-	SLC26A6	3	48615723	48670493	68	9	10164	0.12208	0.45142
+	RWDD3	1	95199155	95257225	152	20	10164	0.12202	0.45144
-	CSN2	4	69945256	70028629	211	20	10164	0.12159	0.45161
-	ZNF676	19	22168936	22283018	338	19	10164	0.12155	0.45163
+	DNAH10	12	123726133	123945720	447	32	10164	0.12147	0.45166
-	MED6	14	70573221	70635690	288	23	10164	0.12134	0.45171
+	SPATA31D4	9	81893428	81944998	31	5	10164	0.12125	0.45175
+	OR1S1	11	58179745	58225722	185	20	10164	0.12107	0.45182

STRAND	SYMBOL	CHR	START	STOP	NSNPS	NPARAM	N	ZSTAT	P
+	PCDHGC4	5	141450174	141522979	159	20	10164	0.121	0.45185
+	CA7	16	66809379	66864149	81	8	10164	0.12088	0.45189
-	ZNF335	20	45938653	46007194	209	35	10164	0.12089	0.45189
+	ATP5D	19	1206750	1254825	108	15	10164	0.12066	0.45198
+	GTF2H4	6	30873200	30924103	272	27	10164	0.12064	0.45199
-	CHST6	16	75463124	75530434	183	25	10164	0.12049	0.45205
-	RMDN3	15	40725884	40790336	158	26	10164	0.1204	0.45208
-	ITPK1	14	92926914	93150918	558	46	10164	0.12033	0.45211
+	SENP2	3	185551228	185641101	226	13	10164	0.11991	0.45228
-	RAP1GAP	1	21586215	21704375	314	36	10164	0.11979	0.45232
-	EIF3H	8	116634816	116790823	269	18	10164	0.1198	0.45232
-	ILDR1	3	121977323	122095516	261	43	10164	0.11939	0.45248
-	CIT	12	119675790	119912318	536	43	10164	0.11937	0.45249
+	GGTLC2	22	22609620	22657898	16	2	10164	0.11928	0.45253
+	SLAIN1	13	77662854	77774242	244	31	10164	0.1191	0.4526
+	FAM174A	5	100500305	100596741	164	14	10164	0.11906	0.45261
+	LOC1053719	17	62309040	62363998	143	18	10164	0.11885	0.4527
+	MZT2B	2	130146544	130214794	102	3	10164	0.11877	0.45273
+	AFMID	17	78150102	78217701	302	34	10164	0.11867	0.45277
+	MED29	19	39356323	39410563	23	7	10164	0.11829	0.45292
+	CTH	1	70376218	70450114	208	32	10164	0.11814	0.45298
+	TMTC2	12	82651952	83144868	1117	72	10164	0.11804	0.45302
-	RFX1	19	13951530	14042514	127	17	10164	0.11794	0.45306
-	TAF4	20	61964798	62100810	414	26	10164	0.11784	0.4531
+	MAN1C1	1	25582468	25796207	113	4	10164	0.11778	0.45312
+	PRAMEF25	1	13038678	13088038	3	1	10164	0.11777	0.45313
+	HNRNPCL3	1	13026232	13072237	3	1	10164	0.11777	0.45313
-	LOC1019299	1	13039808	13088659	3	1	10164	0.11777	0.45313
-	NAE1	16	66792878	66865977	114	9	10164	0.11774	0.45314
-	GLS2	12	56460944	56523414	69	11	10164	0.11716	0.45337
+	ATP6V1B1	2	70900868	70975431	225	40	10164	0.11708	0.4534
-	CCDC96	4	7030849	7078001	106	10	10164	0.11686	0.45348
-	FSTL1	3	120384214	120486071	340	39	10164	0.11664	0.45357
+	PLA2G5	1	19993339	20101901	324	23	10164	0.11648	0.45364
+	KRTAP5-9	11	71513420	71559607	171	13	10164	0.11624	0.45373
-	CYP27B1	12	57752334	57802193	97	13	10164	0.11601	0.45382
+	ASS1	9	130409707	130511274	255	36	10164	0.11598	0.45384
+	FCGR2C	1	161546339	161611220	84	20	10164	0.11553	0.45401
+	FXYD1	19	35103789	35153055	123	11	10164	0.11541	0.45406
+	TTF2	1	117025288	117112870	136	15	10164	0.11537	0.45408
+	MADD	11	47234376	47340031	237	19	10164	0.11494	0.45425
-	TFAP2A	6	10386677	10454624	177	34	10164	0.11483	0.45429
+	TDRD12	19	32684773	32837415	456	29	10164	0.11455	0.4544
-	ALG1L	3	125918840	125987098	217	17	10164	0.11456	0.4544
-	CACUL1	10	118662993	118790246	284	16	10164	0.1145	0.45442
-	EIF2B1	12	123611023	123668776	129	12	10164	0.11445	0.45444
+	SLC35G5	8	11295986	11342186	165	14	10164	0.11433	0.45449
+	WDR38	9	124818347	124867891	256	27	10164	0.11406	0.4546
+	MS4A4A	11	60245541	60318972	197	24	10164	0.1137	0.45474
-	MBTD1	17	51167419	51295493	325	17	10164	0.11345	0.45484
-	NUDT8	11	67617938	67664937	179	25	10164	0.1134	0.45486
+	PRR9	1	153170162	153229317	150	21	10164	0.11334	0.45488
+	CDR2L	17	74952632	75015800	103	14	10164	0.11317	0.45495
+	CEACAM16	19	44662504	44720714	116	20	10164	0.11312	0.45497
-	LOC1053691	7	81462689	81605356	322	17	10164	0.11311	0.45497
-	ERN1	17	64033030	64165142	113	13	10164	0.11309	0.45498
-	CCDC175	14	59495067	59617114	474	15	10164	0.11305	0.45499
+	OR51A7	11	4872370	4918308	115	16	10164	0.11282	0.45509
-	ADPRHL1	13	113411940	113488524	221	34	10164	0.1128	0.4551
+	ETFDH	4	158637101	158718713	45	11	10164	0.11276	0.45511
+	ZFP42	4	187959054	188015050	31	6	10164	0.11251	0.45521
+	LOC1027234	14	36238320	36486196	543	47	10164	0.11239	0.45526
-	COPS3	17	17236624	17316303	175	8	10164	0.11227	0.4553

STRAND	SYMBOL	CHR	START	STOP	NSNPS	NPARAM	N	ZSTAT	P
-	GPA33	1	167042836	167125631	184	17	10164	0.1121	0.45537
-	IFNA14	9	21229202	21274979	192	16	10164	0.11203	0.4554
+	HEY2	6	125714586	125771271	130	19	10164	0.1119	0.45545
-	LOC1053754	7	102397507	102456280	93	16	10164	0.1119	0.45545
-	RNF149	2	101261219	101343716	145	19	10164	0.1118	0.45549
-	CWF19L1	10	100222296	100302681	233	19	10164	0.11177	0.4555
+	DNAH14	1	224894595	225409294	1886	38	10164	0.11169	0.45553
-	MSL2	3	136138918	136232393	140	23	10164	0.11091	0.45585
+	LOC1053769	19	36231808	36279037	158	15	10164	0.11087	0.45586
+	DDX55	12	123567092	123630938	190	14	10164	0.11042	0.45604
+	EIF2AK4	15	39899124	40045596	595	50	10164	0.11041	0.45604
+	ZNF687	1	151246555	151301905	76	14	10164	0.1101	0.45616
+	ZC2HC1C	14	75034232	75089987	112	8	10164	0.10996	0.45622
-	HPD	12	121829527	121923611	259	28	10164	0.10988	0.45625
+	COA6	1	234338437	234394049	16	4	10164	0.10977	0.4563
-	C2orf44	2	24019336	24082426	177	21	10164	0.10942	0.45644
-	OR2T10	1	248582830	248628768	3	1	10164	0.1094	0.45644
+	FAM43B	1	20517439	20565020	123	19	10164	0.10927	0.45649
+	GLP1R	6	39013781	39101303	366	48	10164	0.10911	0.45656
-	COX6A2	16	31417731	31463428	39	8	10164	0.10903	0.45659
+	POTED	21	13575061	13651585	22	3	10164	0.10901	0.4566
-	HBE1	11	5258345	5316945	204	31	10164	0.10873	0.45671
-	HID1	17	74940742	75007805	121	18	10164	0.10864	0.45674
-	DPM3	1	155129891	155175520	80	8	10164	0.10846	0.45681
+	NUDC	1	26876128	26956871	70	13	10164	0.10846	0.45682
-	HDLBP	2	241217267	241350700	383	31	10164	0.1084	0.45684
+	ANXA10	4	168057526	168197742	381	30	10164	0.10814	0.45694
-	CARD11	7	2896075	3078945	846	124	10164	0.10812	0.45695
-	DCP1A	3	53273423	53382627	239	29	10164	0.10778	0.45708
-	BIN2	12	51271038	51359680	290	27	10164	0.10771	0.45711
-	AMBP	9	114050127	114113472	188	26	10164	0.10757	0.45717
-	PRRT4	7	128340325	128397069	108	27	10164	0.10755	0.45718
+	RUNX2	6	45292800	45674032	1154	89	10164	0.1073	0.45728
+	EFNA4	1	155028737	155079553	73	19	10164	0.10704	0.45738
-	MPPE1	18	11873471	11943797	238	34	10164	0.10687	0.45744
-	RBL1	20	36986349	37131000	203	19	10164	0.10668	0.45752
+	KIAA1211	4	56014229	56340724	1150	124	10164	0.10656	0.45757
+	BOD1L2	18	57112062	57160408	214	30	10164	0.10621	0.45771
-	PACSIN2	22	42859766	43050690	674	34	10164	0.10605	0.45777
+	C12orf74	12	92667843	92718549	115	12	10164	0.10594	0.45782
-	ZFAND2A	7	1142907	1195219	301	17	10164	0.10571	0.45791
+	EEF1B2	2	206124594	206172929	143	11	10164	0.10553	0.45798
+	MFSD4	1	205533984	205612918	239	38	10164	0.10547	0.458
+	TMEM56-RW	1	95082923	95254955	488	35	10164	0.10538	0.45804
-	SLC9C2	1	173490465	173638094	317	16	10164	0.10532	0.45806
-	CYP3A7-CYF	7	99674679	99770200	182	12	10164	0.10527	0.45808
+	GSTCD	4	105673778	105857725	256	20	10164	0.10515	0.45813
+	KIF2A	5	62271162	62397184	366	22	10164	0.1051	0.45815
+	DNAJB9	7	108534745	108584850	116	14	10164	0.10509	0.45815
-	PARP11	12	3798861	3908448	211	21	10164	0.10503	0.45818
-	CHMP4A	14	24199581	24248830	77	14	10164	0.10501	0.45819
-	POSTN	13	37552582	37633844	285	22	10164	0.10496	0.45821
+	THRA	17	40027193	40103867	122	20	10164	0.10478	0.45828
+	RGSL1	1	182414217	182570597	440	26	10164	0.10475	0.45829
+	CYP4F12	19	15638018	15708819	467	25	10164	0.10469	0.45831
-	STX1B	16	30979256	31045638	83	7	10164	0.10468	0.45832
-	ST8SIA1	12	22119135	22369714	701	49	10164	0.10446	0.4584
+	FAM83A	8	123144047	123220079	136	10	10164	0.10391	0.45862
-	NOX4	11	89314353	89624611	878	54	10164	0.10384	0.45865
-	ID3	1	23547930	23594794	82	11	10164	0.10378	0.45867
-	SREK1IP1	5	64708148	64803731	132	11	10164	0.10364	0.45873
+	TCF7L2	10	112915219	113177678	641	100	10164	0.10361	0.45874
+	BICD1	12	32071736	32393650	1467	80	10164	0.1034	0.45882

STRAND	SYMBOL	CHR	START	STOP	NSNPS	NPARAM	N	ZSTAT	P
+	SERP2	13	44338842	44407714	132	22	10164	0.10341	0.45882
+	CBLC	19	44742861	44810646	149	24	10164	0.10338	0.45883
-	CKAP2L	2	112726349	112799677	162	13	10164	0.10325	0.45888
-	CBLN4	20	55987357	56040474	80	10	10164	0.10312	0.45894
+	NBR1	17	43135471	43221689	168	6	10164	0.10303	0.45897
+	CLEC2D	12	9634708	9709555	277	20	10164	0.10291	0.45902
+	TSHZ2	20	52937309	53505330	1031	117	10164	0.10284	0.45904
-	UBXN11	1	26272282	26353265	229	15	10164	0.10282	0.45905
-	NARS2	11	78425961	78609864	410	10	10164	0.10234	0.45924
+	KRTAP22-1	21	30566121	30611267	187	13	10164	0.10222	0.45929
-	FIGN	2	163594937	163771197	413	54	10164	0.10218	0.45931
-	FRA10AC1	10	93657883	93737572	200	11	10164	0.10215	0.45932
-	MYBPC3	11	47321406	47387702	152	20	10164	0.10191	0.45941
-	MRPL50	9	101377633	101433641	156	20	10164	0.10187	0.45943
+	FKRP	19	46709606	46768575	134	21	10164	0.10181	0.45946
+	FAM200B	4	15646729	15700447	160	20	10164	0.10176	0.45947
-	NRAP	10	113578824	113699070	458	35	10164	0.10166	0.45951
+	ALPL	1	21473982	21588412	327	28	10164	0.10157	0.45955
-	FLG	1	152292163	152360257	234	22	10164	0.10129	0.45966
+	LUC7L3	17	50684565	50762711	60	13	10164	0.10112	0.45973
+	AP2M1	3	184139846	184194091	92	14	10164	0.10079	0.45986
+	ARID5A	2	96501727	96562634	97	29	10164	0.10069	0.4599
-	MKNK2	19	2027471	2086244	155	34	10164	0.10064	0.45992
+	ZAR1	4	48455252	48508122	122	14	10164	0.10055	0.45995
+	AQP12A	2	240656845	240708483	122	15	10164	0.10047	0.45999
-	VPS37B	12	122855328	122931220	68	6	10164	0.1004	0.46002
-	CDKN1C	11	2873218	2920816	124	27	10164	0.10001	0.46017
-	SCIMP	17	5198920	5269860	252	30	10164	0.099905	0.46021
+	LOC1053753	7	57735209	57850981	254	14	10164	0.099892	0.46022
+	SNX24	5	122810465	123039276	700	24	10164	0.099695	0.46029
+	PCDHGC3	5	141441002	141522977	176	21	10164	0.099509	0.46037
-	EFTUD1	15	82120220	82297763	461	27	10164	0.099462	0.46039
-	PSG8	19	42742687	42800692	33	12	10164	0.099193	0.46049
-	PLSCR1	3	146505178	146579841	291	26	10164	0.099039	0.46055
+	RAB11B	19	8355321	8414434	88	8	10164	0.098999	0.46057
-	BRAF	7	140705951	140959764	407	14	10164	0.098932	0.4606
+	GXYLT2	3	72813522	72985374	206	21	10164	0.098872	0.46062
+	TNFAIP8L2	1	151121629	151169749	61	11	10164	0.098787	0.46065
-	ACACA	17	37074992	37441822	617	33	10164	0.098644	0.46071
+	PRRX1	1	170627728	170749419	215	24	10164	0.098437	0.46079
-	DSCC1	8	119823941	119890930	285	23	10164	0.09825	0.46087
+	RETN	19	7634086	7680454	160	19	10164	0.098218	0.46088
+	ZNF613	19	51892147	51955758	191	18	10164	0.098118	0.46092
+	ZNF627	19	11540272	11629159	155	21	10164	0.098082	0.46093
+	ORC6	16	46654646	46708394	13	3	10164	0.097732	0.46107
+	DNAJC6	1	65213148	65425869	491	34	10164	0.097549	0.46115
-	NEK9	14	75072115	75162075	176	10	10164	0.097386	0.46121
-	PDE1C	7	31606777	32334404	2065	121	10164	0.097324	0.46123
+	TTLL5	14	75626208	75965082	725	40	10164	0.097304	0.46124
-	SLC16A14	2	230024974	230103999	274	33	10164	0.097229	0.46127
-	PSME2	14	24133365	24182316	76	17	10164	0.097172	0.46129
-	OAZ2	15	64677574	64738281	79	9	10164	0.097142	0.46131
-	ZDHHC19	3	196187443	196246437	238	29	10164	0.09707	0.46134
-	TNIP2	4	2731660	2791376	240	31	10164	0.096714	0.46148
-	IL31	12	122162030	122217963	151	22	10164	0.096529	0.46155
-	NEDD8	14	24206851	24267370	94	19	10164	0.096477	0.46157
+	CSN1S1	4	69896062	69956570	175	23	10164	0.096235	0.46167
-	LACTBL1	1	22943043	23007316	137	13	10164	0.09574	0.46186
+	ARL14EPL	5	116015393	116069426	328	36	10164	0.095459	0.46197
+	SLC25A47	14	100288342	100340378	151	34	10164	0.095399	0.462
+	NSUN7	4	40714897	40819985	142	14	10164	0.095354	0.46202
-	IL7	8	78665872	78840523	389	38	10164	0.09525	0.46206
-	T	6	166147656	166203669	333	32	10164	0.095003	0.46216

STRAND	SYMBOL	CHR	START	STOP	NSNPS	NPARAM	N	ZSTAT	P
+	ARL9	4	56470209	56533983	199	20	10164	0.094927	0.46219
+	PLEKHD1	14	69449754	69539327	202	28	10164	0.094712	0.46227
-	PTRH1	9	127703948	127751714	120	15	10164	0.094071	0.46253
-	COQ5	12	120493279	120564161	251	24	10164	0.093923	0.46259
+	FBXO45	3	196533854	196599059	140	14	10164	0.093878	0.4626
+	TNPO1	5	72781591	72924388	252	20	10164	0.093639	0.4627
+	SPDYE1	7	43962440	44020124	95	13	10164	0.093519	0.46275
+	LRRC69	8	91067619	91229236	394	17	10164	0.093486	0.46276
+	LRRC49	15	70818239	71060097	269	24	10164	0.093237	0.46286
+	AAMDC	11	77786107	77882354	207	14	10164	0.092775	0.46304
-	MYPOP	19	45880023	45937696	221	27	10164	0.092517	0.46314
-	CD1B	1	158317950	158366531	80	10	10164	0.092501	0.46315
+	CD1E	1	158318435	158367553	80	10	10164	0.092501	0.46315
+	SCLY	2	238025924	238109413	293	21	10164	0.092396	0.46319
+	SKOR1	15	67784704	67844561	254	27	10164	0.092382	0.4632
-	FADS3	11	61863523	61926545	139	24	10164	0.092361	0.46321
+	EPCAM	2	47334148	47397028	231	36	10164	0.092319	0.46322
-	IFNA10	9	21196181	21242143	238	17	10164	0.092251	0.46325
-	CCDC57	17	82091470	82248520	471	5	10164	0.092205	0.46327
-	ZNF343	20	2470071	2559522	226	38	10164	0.091865	0.4634
+	EHMT1	9	137583992	137846127	374	12	10164	0.091709	0.46346
+	RSAD1	17	50443800	50512164	117	10	10164	0.091643	0.46349
+	HOXC9	12	53965093	54013337	63	11	10164	0.091286	0.46363
-	ASCC2	22	29778608	29873304	268	32	10164	0.091147	0.46369
+	TTLL9	20	31835702	31954959	131	25	10164	0.091043	0.46373
-	HIST2H2AA3	1	149832218	149877751	8	3	10164	0.091005	0.46374
+	SMCHD1	18	2620887	2815017	622	34	10164	0.090882	0.46379
+	ZFAND3	6	37784496	38164624	1051	25	10164	0.090746	0.46385
+	FSCN3	7	127558635	127611797	154	26	10164	0.090606	0.4639
+	SWSAP1	19	11339707	11386951	22	4	10164	0.090028	0.46413
-	FAM136A	2	70285976	70337088	162	16	10164	0.089601	0.4643
-	ZNF468	19	52828532	52892649	159	18	10164	0.089551	0.46432
-	ZMYND19	9	137572076	137625485	71	4	10164	0.089435	0.46437
-	CDK17	12	96268261	96435588	353	33	10164	0.089378	0.46439
+	C17orf50	17	35725593	35775079	131	18	10164	0.089377	0.46439
-	VPS13C	15	61842389	62095465	708	25	10164	0.089307	0.46442
+	GLYATL1	11	58892629	58967074	251	23	10164	0.089263	0.46444
+	SYNE2	14	63817586	64236449	1268	61	10164	0.089229	0.46445
-	ATP8B1	18	57636426	57838822	556	55	10164	0.089101	0.4645
-	DIMT1	5	62378524	62438901	147	18	10164	0.089021	0.46453
+	FAM153B	5	176028709	176124798	36	6	10164	0.088822	0.46461
+	TMPRSS7	3	112004067	112091269	542	34	10164	0.088776	0.46463
+	TRAF7	16	2120753	2188129	41	22	10164	0.088471	0.46475
+	TFAP2C	20	56594302	56649283	83	13	10164	0.088368	0.46479
-	C11orf16	11	8910076	8968006	168	12	10164	0.088366	0.46479
-	TCEB3C	18	47018202	47065078	140	24	10164	0.088272	0.46483
-	KLK15	19	50815289	50871035	291	49	10164	0.088191	0.46486
+	BCL3	19	44713045	44770044	202	36	10164	0.088123	0.46489
+	LIMK1	7	74048777	74132525	32	4	10164	0.088085	0.4649
+	LAT2	7	74174392	74239834	17	3	10164	0.088106	0.4649
+	RNASEH2A	19	12767054	12823648	25	9	10164	0.087924	0.46497
+	DDX49	19	18884675	18938633	117	21	10164	0.087674	0.46507
-	HLA-DRB5	6	32507374	32565316	254	25	10164	0.087639	0.46508
-	SVIL	10	29447338	29771935	1040	87	10164	0.087634	0.46508
+	LIN28B	6	104901999	105093332	262	28	10164	0.087584	0.4651
-	LOC1019287	1	26682063	26731405	60	3	10164	0.0875	0.46514
+	SORD	15	44988104	45085089	172	23	10164	0.087256	0.46523
+	C1orf95	1	226513248	226619214	318	38	10164	0.087248	0.46524
-	KRT83	12	52304301	52356398	210	18	10164	0.087218	0.46525
-	SAMD9	7	93089513	93153023	153	17	10164	0.08722	0.46525
-	GABRR1	6	89167504	89266288	341	32	10164	0.086745	0.46544
-	HAPLN4	19	19245641	19297787	153	24	10164	0.086604	0.46549
-	AAK1	2	69447995	69678845	710	33	10164	0.086443	0.46556

STRAND	SYMBOL	CHR	START	STOP	NSNPS	NPARAM	N	ZSTAT	P
-	LOC1053705	14	69469602	69519436	128	20	10164	0.08643	0.46556
+	MRGBP	20	62761453	62810593	185	30	10164	0.086252	0.46563
-	CDCP2	1	54128995	54188006	172	28	10164	0.086235	0.46564
-	COG8	16	69318621	69374623	132	20	10164	0.085854	0.46579
-	WDR5B	3	122401853	122451035	125	17	10164	0.085525	0.46592
-	GPS2	17	7302659	7350339	103	18	10164	0.085165	0.46607
-	NOC3L	10	94304907	94397976	295	30	10164	0.084984	0.46614
-	ARSJ	4	113890284	114016430	406	27	10164	0.084953	0.46615
+	DDX42	17	63738884	63829317	175	9	10164	0.084721	0.46624
-	C7orf66	7	108873975	108931211	235	7	10164	0.084533	0.46632
+	GALK2	15	49120759	49377894	743	22	10164	0.084507	0.46633
-	PLXNB2	22	50264979	50342642	262	38	10164	0.084349	0.46639
-	SLX4	16	3571165	3646598	228	14	10164	0.084282	0.46642
-	CDKL1	14	50319404	50432404	199	20	10164	0.084256	0.46643
-	INCA1	17	4978130	5032922	155	23	10164	0.084151	0.46647
+	BANF1	11	65967079	66014146	189	19	10164	0.084077	0.4665
-	GATB	4	151660052	151796023	303	12	10164	0.083817	0.4666
-	SNX14	6	85495496	85629156	226	28	10164	0.083748	0.46663
-	RPP25L	9	34600485	34647113	106	20	10164	0.08367	0.46666
-	SYNCRIP	6	85597784	85678850	118	22	10164	0.083594	0.46669
-	PTPRD	9	8304246	10647723	10351	504	10164	0.083409	0.46676
-	LOC1019272	2	111211749	111283237	106	15	10164	0.083425	0.46676
-	ZADH2	18	75187323	75244326	122	22	10164	0.083226	0.46684
+	ARHGEF37	5	149516632	149644968	406	31	10164	0.083175	0.46686
-	TMEM218	11	125084370	125146763	221	31	10164	0.083031	0.46691
+	CPE	4	165343945	165508330	713	35	10164	0.082904	0.46696
-	SH3D19	4	151110281	151263030	402	20	10164	0.082683	0.46705
+	TCIRG1	11	68003995	68060899	155	26	10164	0.082581	0.46709
+	KMT2E	7	104979190	105124085	346	15	10164	0.082566	0.4671
+	ANKK1	11	113351038	113410418	196	24	10164	0.082491	0.46713
+	LOC1053785	10	129733365	129780983	118	12	10164	0.082437	0.46715
+	CSGALNACT	10	43103445	43195308	214	19	10164	0.082273	0.46721
+	CHAC2	2	53732792	53785150	183	25	10164	0.082281	0.46721
+	PCDHGA3	5	141309034	141522979	442	31	10164	0.08221	0.46724
-	TRPA1	8	72011251	72125009	402	28	10164	0.082098	0.46728
+	LOC1053768	18	79645361	79698319	215	23	10164	0.0821	0.46728
+	HBM	16	130974	176768	111	23	10164	0.08206	0.4673
-	NEDD8-MDP	14	24203937	24267370	102	20	10164	0.081963	0.46734
+	LRRC23	12	6869733	6924242	17	6	10164	0.081936	0.46735
+	FER	5	108711085	109197674	1407	36	10164	0.081844	0.46739
+	ANO1	11	70043302	70199545	522	59	10164	0.081612	0.46748
-	C3orf17	3	112992444	113054851	125	21	10164	0.081442	0.46755
-	FAXC	6	99262915	99385122	365	24	10164	0.08141	0.46756
+	NRBF2	10	63098247	63165031	225	27	10164	0.081392	0.46757
-	LBX1	10	101216212	101265327	104	21	10164	0.081312	0.4676
+	HAS3	16	69070564	69128719	126	25	10164	0.081138	0.46767
+	GUK1	1	228104643	228158955	184	26	10164	0.081082	0.46769
+	ZNF707	8	143649452	143705385	130	17	10164	0.080575	0.46789
-	EFNB2	13	106479731	106570040	64	10	10164	0.080494	0.46792
+	RPF1	1	84444237	84508350	175	17	10164	0.080118	0.46807
+	C22orf46	22	41654936	41708136	146	12	10164	0.079971	0.46813
+	YEATS2	3	183662804	183822625	447	19	10164	0.079765	0.46821
-	COL6A3	2	237314012	237449370	486	56	10164	0.079615	0.46827
+	DNAJC13	3	132382532	132549032	417	33	10164	0.079076	0.46849
+	FBXO44	1	11619375	11673327	152	27	10164	0.079076	0.46849
-	OR6X1	11	123743580	123789518	165	18	10164	0.078904	0.46855
+	RASL10B	17	35696660	35753525	140	26	10164	0.07888	0.46856
+	INHBE	12	57420313	57468008	104	19	10164	0.078867	0.46857
-	MTMR12	5	32217007	32348008	219	16	10164	0.078782	0.4686
-	LOC1019288	13	113389610	113455858	204	32	10164	0.078292	0.4688
+	ACSL3	2	222825984	222953401	342	26	10164	0.0782	0.46883
-	LTB	6	31570558	31617425	297	72	10164	0.078027	0.4689
+	BCKDK	16	31073294	31122791	67	4	10164	0.078016	0.46891

STRAND	SYMBOL	CHR	START	STOP	NSNPS	NPARAM	N	ZSTAT	P
+	ZNF718	4	89476	212306	136	5	10164	0.077907	0.46895
+	TCTN2	12	123636113	123718405	219	8	10164	0.077825	0.46898
-	ROGDI	16	4786962	4837950	47	3	10164	0.077735	0.46902
-	TP53I11	11	44922348	44986306	146	29	10164	0.077692	0.46904
-	RPL10L	14	46641013	46686825	119	16	10164	0.077621	0.46906
-	ZIM2	19	56764547	56875729	140	14	10164	0.07704	0.4693
-	PDF	16	69318621	69365595	116	18	10164	0.076912	0.46935
-	HIBADH	7	27515440	27698001	619	26	10164	0.076815	0.46939
+	NOL3	16	67135497	67185737	49	13	10164	0.076504	0.46951
+	GPR182	12	56959355	57010007	115	9	10164	0.076462	0.46953
-	INSL3	19	17806513	17856574	91	18	10164	0.076429	0.46954
+	ANKRD13B	17	29558509	29624761	74	15	10164	0.07634	0.46957
-	SMPD4	2	130141392	130217750	117	3	10164	0.076276	0.4696
-	EEF1E1	6	8063360	8137595	280	35	10164	0.076054	0.46969
+	MAPK7	17	19342721	19393544	46	2	10164	0.075906	0.46975
-	MORC1	3	108948239	109153146	622	16	10164	0.075857	0.46977
-	TMEM120A	7	75976837	76029674	122	17	10164	0.075753	0.46981
-	CCDC47	17	63735250	63808728	150	9	10164	0.075636	0.46985
-	MARCH3	5	126857714	127067402	580	13	10164	0.075382	0.46996
+	PDCD5	19	32546170	32597452	182	22	10164	0.07521	0.47002
+	AK5	1	77246977	77569969	1115	59	10164	0.075151	0.47005
-	LOC1019301	18	47011726	47059244	122	21	10164	0.075145	0.47005
+	LCE1E	1	152751277	152798426	113	10	10164	0.074624	0.47026
+	ADAM15	1	155016272	155072776	90	19	10164	0.074529	0.47029
+	FCGRT	19	49477279	49536428	161	17	10164	0.073999	0.47051
+	AP4M1	7	100066412	100117180	104	18	10164	0.073874	0.47056
-	C1GALT1C1L	2	43665153	43711322	203	23	10164	0.073736	0.47061
-	EIF3E	8	108191743	108283730	240	10	10164	0.073645	0.47065
+	TIMM23	10	45937453	46013742	191	17	10164	0.073648	0.47065
+	C20orf196	20	5715335	5874407	356	40	10164	0.073545	0.47069
+	SEMA4A	1	156112359	156187751	148	22	10164	0.073404	0.47074
+	GIMAP1	7	150681557	150734280	160	20	10164	0.072721	0.47101
+	CGB2	19	48996873	49043238	162	17	10164	0.072717	0.47102
-	PALB2	16	23593162	23676357	249	20	10164	0.072548	0.47108
+	PRRX2	9	129630641	129732674	282	32	10164	0.072529	0.47109
-	TNXB	6	32031155	32144374	409	65	10164	0.072475	0.47111
+	OR2M4	1	248203929	248249864	45	3	10164	0.072388	0.47115
-	CNR1	6	88129864	88201745	194	33	10164	0.072342	0.47116
-	EBF3	10	129825232	129999281	51	12	10164	0.072343	0.47116
-	BOLL	2	197716879	197821574	108	8	10164	0.07191	0.47134
-	PSME4	2	53854067	54005840	451	28	10164	0.071623	0.47145
-	LOC1027237	15	74588919	74650079	115	23	10164	0.071499	0.4715
+	CASR	3	122148667	122296503	540	31	10164	0.071126	0.47165
+	MSTO1	1	155575170	155624967	90	10	10164	0.071114	0.47165
+	KIAA1217	10	23659746	24557848	2495	144	10164	0.071038	0.47168
+	RPP30	10	90836952	90918558	173	19	10164	0.07096	0.47171
-	TMEM259	19	999651	1056169	313	28	10164	0.070649	0.47184
+	MSH2	2	47368067	47574579	621	44	10164	0.070542	0.47188
+	GSKIP	14	96328452	96397290	191	14	10164	0.070535	0.47188
-	ZNF649	19	51878538	51940052	198	15	10164	0.070511	0.47189
+	WRN	8	30998262	31183761	420	38	10164	0.070258	0.47199
+	C12orf45	12	104951320	105004727	179	18	10164	0.070227	0.47201
+	SLC27A2	15	50147196	50246392	428	35	10164	0.069838	0.47216
-	SLC22A31	16	89185756	89236671	286	28	10164	0.069671	0.47223
-	SMCO1	3	196496879	196550366	176	12	10164	0.069272	0.47239
-	PCDH20	13	61399685	61450522	193	19	10164	0.069149	0.47244
+	FAIM	3	138573594	138643376	69	5	10164	0.069056	0.47247
+	PAQR5	15	69263902	69417637	443	26	10164	0.06869	0.47262
+	AGPAT3	21	43830160	43997594	458	38	10164	0.068524	0.47268
-	TRPM5	11	2393952	2470017	123	28	10164	0.068078	0.47286
-	PSTPIP2	18	45973536	46107297	453	21	10164	0.067976	0.4729
+	SGCD	5	156082934	156777788	1808	76	10164	0.067969	0.47291
-	SLCO6A1	5	102361782	102534656	520	17	10164	0.067828	0.47296

STRAND	SYMBOL	CHR	START	STOP	NSNPS	NPARAM	N	ZSTAT	P
+	FBXO39	17	6741175	6806474	236	32	10164	0.067635	0.47304
+	AK4	1	65112549	65242145	336	28	10164	0.067555	0.47307
-	TMEM244	6	129821244	129896503	249	19	10164	0.067337	0.47316
+	IL18R1	2	102321283	102408777	381	19	10164	0.067304	0.47317
-	GALNT4	12	89509412	89559806	80	9	10164	0.067229	0.4732
-	POC1B-GALI	12	89509412	89561262	80	9	10164	0.067229	0.4732
-	MED20	6	41895354	41956139	100	18	10164	0.066817	0.47336
+	PRPF40B	12	49588414	49654669	69	11	10164	0.066719	0.4734
+	HLA-DQA2	6	32706386	32756887	1579	26	10164	0.066645	0.47343
+	OXA1L	14	22731522	22781789	178	21	10164	0.066454	0.47351
-	TSPEAR	21	44487892	44746580	793	41	10164	0.0664	0.47353
+	C11orf74	11	36559229	36669291	394	17	10164	0.066379	0.47354
-	RAX	18	59257035	59308393	208	24	10164	0.066289	0.47357
-	STPG1	1	24346999	24450497	289	22	10164	0.066275	0.47358
+	MIER1	1	66889895	66998619	236	18	10164	0.066239	0.47359
+	FAM149B1	10	73133095	73252181	281	5	10164	0.066127	0.47364
-	DYX1C1	15	55407755	55543234	436	32	10164	0.065837	0.47375
-	MARCH4	2	216247862	216407027	505	55	10164	0.065633	0.47383
+	SNX18	5	54482759	54556586	210	15	10164	0.065618	0.47384
-	THAP8	19	36024984	36089762	141	18	10164	0.065539	0.47387
-	GPATCH3	1	26880488	26936065	56	10	10164	0.065449	0.47391
-	CLEC2B	12	9842369	9904859	161	18	10164	0.065265	0.47398
+	SH3D21	1	36270726	36331962	86	16	10164	0.065194	0.47401
-	FOLH1	11	49136635	49243670	364	17	10164	0.064955	0.4741
-	MEIOB	16	1823983	1907178	616	13	10164	0.064921	0.47412
+	GMPR2	14	24197422	24249242	89	16	10164	0.064857	0.47414
+	CBFB	16	66994147	67111058	124	15	10164	0.064843	0.47415
-	NAIF1	9	128051233	128102321	27	10	10164	0.064811	0.47416
-	COA7	1	52676342	52733366	178	15	10164	0.064668	0.47422
+	GLDN	15	51306516	51418013	499	51	10164	0.064478	0.47429
+	PLCB1	20	8097254	8894900	1911	163	10164	0.064436	0.47431
-	WDR91	7	135173838	135246564	200	13	10164	0.064002	0.47448
+	NLGN1	3	173361490	174296644	2314	117	10164	0.063954	0.4745
-	MAF	16	79202772	79635786	1927	163	10164	0.063934	0.47451
+	ATP8B2	1	154290560	154361304	187	19	10164	0.063883	0.47453
+	JMY	5	79201102	79337215	332	22	10164	0.063884	0.47453
-	PARP1	1	226350691	226443100	368	23	10164	0.063857	0.47454
+	LCE1D	1	152761751	152808181	118	10	10164	0.063797	0.47457
+	P2RY1	3	152799947	152848056	150	21	10164	0.063745	0.47459
+	CYSRT1	9	137189635	137236311	105	16	10164	0.063607	0.47464
-	LCE3A	1	152612834	152658103	186	7	10164	0.063527	0.47467
+	IPO11	5	62377746	62638589	494	22	10164	0.063515	0.47468
+	KIFC1	6	33356524	33420226	259	50	10164	0.063348	0.47474
+	RORB	9	74462336	74697201	420	34	10164	0.063017	0.47488
-	GIT1	17	29563469	29624592	71	14	10164	0.063018	0.47488
-	CCDC144NL	17	20837348	20931647	89	6	10164	0.06277	0.47497
+	GABRP	5	170737566	170824047	209	27	10164	0.062755	0.47498
+	EPHA3	3	89072524	89492134	1288	67	10164	0.062698	0.475
+	CERS6	2	168421249	168785134	988	53	10164	0.062581	0.47505
+	CC2D2A	4	15434861	15611971	445	41	10164	0.062524	0.47507
+	GLB1L2	11	134296874	134386328	166	36	10164	0.062542	0.47507
-	PLVAP	19	17341448	17412349	258	40	10164	0.062336	0.47515
+	C4orf19	4	37418454	37603510	608	56	10164	0.062283	0.47517
-	MRPL17	11	6670385	6718401	179	22	10164	0.061739	0.47539
+	PDIK1L	1	26076165	26135548	148	8	10164	0.061739	0.47539
+	RAB7A	3	128691136	128824798	293	24	10164	0.06137	0.47553
+	EIF2B2	14	74967909	75019591	94	9	10164	0.061332	0.47555
-	KIAA1549	7	138821381	139016318	458	42	10164	0.061193	0.4756
+	PLD2	17	4772101	4833432	184	35	10164	0.060741	0.47578
+	VAX2	2	70865590	70943446	204	32	10164	0.060755	0.47578
+	CERCAM	9	128376575	128447351	135	13	10164	0.060566	0.47585
-	LCP2	5	170237754	170332818	213	56	10164	0.060528	0.47587
-	DUSP10	1	221691420	221777176	132	24	10164	0.060489	0.47588

STRAND	SYMBOL	CHR	START	STOP	NSNPS	NPARAM	N	ZSTAT	P
+	ZNF658	9	66865729	66931653	12	3	10164	0.060481	0.47589
+	SLC27A3	1	153740292	153790157	72	24	10164	0.060415	0.47591
-	DPPA4	3	109316141	109372572	179	13	10164	0.060185	0.476
-	DNAJC1	10	21746548	22038768	430	36	10164	0.060129	0.47603
-	CASP5	11	104982633	105058221	143	24	10164	0.059955	0.4761
+	CTNNA2	2	79477934	80658868	4227	291	10164	0.059731	0.47618
+	GTF2H3	12	123598739	123672604	175	15	10164	0.059536	0.47626
+	THG1L	5	157696315	157749818	173	20	10164	0.059152	0.47642
-	HIST1H2BK	6	27128293	27181858	143	37	10164	0.058997	0.47648
-	AGRP	16	67472571	67519083	43	10	10164	0.058908	0.47651
+	ZNF391	6	27339938	27413908	292	17	10164	0.058828	0.47654
+	ARNTL2	12	27297854	27435813	371	38	10164	0.058742	0.47658
+	HSD17B4	5	119417443	119552335	498	35	10164	0.058637	0.47662
+	ARHGAP31	3	119259289	119429476	828	84	10164	0.058417	0.47671
-	NKAIN1	1	31169745	31274887	348	17	10164	0.058379	0.47672
+	GANC	15	42238658	42363666	514	17	10164	0.058057	0.47685
-	GLMP	1	156282687	156330689	128	11	10164	0.057777	0.47696
+	MX2	21	41326986	41418943	244	42	10164	0.057711	0.47699
+	VPS4A	16	69276384	69335043	143	19	10164	0.05731	0.47715
+	KMT2A	11	118401490	118536824	167	24	10164	0.057264	0.47717
-	CHST5	16	75518530	75570170	165	14	10164	0.057065	0.47725
+	MYO3A	10	25899073	26222536	630	14	10164	0.056919	0.4773
-	FAM186B	12	49572875	49640650	85	11	10164	0.056713	0.47739
+	SETD1A	16	30913505	30994664	83	5	10164	0.056606	0.47743
+	SFI1	22	31453688	31628551	602	30	10164	0.056599	0.47743
+	CYP2C9	10	94903658	94999391	409	26	10164	0.056201	0.47759
-	SRP14	15	40025690	40074188	179	27	10164	0.056146	0.47761
-	TMEM241	18	23285781	23472969	320	24	10164	0.05606	0.47765
-	CYP2C8	10	95026772	95104498	283	22	10164	0.056016	0.47766
+	WIPI2	7	5155204	5243855	265	29	10164	0.056019	0.47766
-	MUT	6	49420360	49498328	171	17	10164	0.056012	0.47767
-	PRSS53	16	31073424	31125035	69	4	10164	0.055855	0.47773
-	C19orf84	19	51378289	51425574	74	19	10164	0.055715	0.47778
-	PPP1R13B	14	103723751	103883967	434	21	10164	0.055636	0.47782
+	SYNGAP1	6	33385070	33463689	280	53	10164	0.055598	0.47783
+	CDK8	13	26219125	26415238	521	26	10164	0.055567	0.47784
-	DCP1B	12	1936048	2039511	207	26	10164	0.055463	0.47788
-	ASPH	8	61490556	61749640	641	40	10164	0.055381	0.47792
+	SNAPIN	1	153623654	153671852	77	6	10164	0.055235	0.47798
-	EFCAB14	1	46665159	46754064	263	26	10164	0.05514	0.47801
+	TIMMDC1	3	119463521	119534281	295	25	10164	0.054874	0.47812
+	ATF6	1	161731244	161974070	601	17	10164	0.054639	0.47821
+	ZNF730	19	23081975	23157212	139	14	10164	0.054422	0.4783
+	TUFT1	1	151505300	151593583	192	30	10164	0.054338	0.47833
-	ISG20L2	1	156711891	156763472	82	16	10164	0.05424	0.47837
-	LOC1053748	2	39138212	39185198	132	17	10164	0.053859	0.47852
+	NANOS3	19	13841406	13890757	85	12	10164	0.053684	0.47859
-	GLB1	3	32986608	33132202	536	38	10164	0.053548	0.47865
+	ZSCAN25	7	99581611	99748530	269	16	10164	0.053501	0.47867
-	CAB39L	13	49298647	49479134	578	63	10164	0.053378	0.47872
-	MAD1L1	7	1805792	2267948	1887	39	10164	0.053093	0.47883
-	HAUS4	14	22936228	22992142	80	6	10164	0.052621	0.47902
-	ATG2B	14	96271258	96398341	428	16	10164	0.052604	0.47902
-	FAM84B	8	126542438	126593478	149	27	10164	0.052579	0.47903
+	PLAC9	10	80097502	80155028	223	25	10164	0.052425	0.4791
-	C16orf90	16	3483484	3530721	181	17	10164	0.052283	0.47915
+	RPAP2	1	92263965	92398175	156	16	10164	0.051956	0.47928
+	ITGA1	5	52753302	52963655	863	92	10164	0.051886	0.47931
+	KCNJ2	17	70133616	70190044	122	10	10164	0.051752	0.47936
+	DDX4	5	55702995	55827147	340	20	10164	0.051692	0.47939
-	PODNL1	19	13921187	13988392	56	16	10164	0.051527	0.47945
-	P4HA3	11	74237863	74346676	230	24	10164	0.051461	0.47948
+	YPEL5	2	30111884	30170533	116	16	10164	0.0514	0.4795

STRAND	SYMBOL	CHR	START	STOP	NSNPS	NPARAM	N	ZSTAT	P
+	C14orf166	14	51954510	52014702	193	27	10164	0.051395	0.47951
-	ATP8B3	19	1772070	1847276	55	11	10164	0.051388	0.47951
-	MMP10	11	102760502	102815628	244	55	10164	0.051315	0.47954
-	PHTF1	1	113687202	113794892	198	23	10164	0.051268	0.47956
-	P2RX1	17	3886591	3951666	100	20	10164	0.051064	0.47964
+	CACNG6	19	53956149	54022666	56	9	10164	0.05093	0.47969
+	PKP1	1	201248452	201342993	381	39	10164	0.05081	0.47974
+	BHMT	5	79076781	79142290	160	19	10164	0.050694	0.47978
-	FAM24B	10	122839094	122914641	188	17	10164	0.05069	0.47979
-	IFNE	9	21470839	21517313	150	15	10164	0.050396	0.4799
+	S100A7A	1	153381524	153433225	98	3	10164	0.050334	0.47993
+	KRTAP10-10	21	44602356	44648455	133	12	10164	0.049958	0.48008
-	DICER1	14	95076228	95193010	143	12	10164	0.049804	0.48014
+	HTN1	4	70015415	70068848	168	16	10164	0.049777	0.48015
-	LOC1053791	5	127208521	127264743	175	11	10164	0.049777	0.48015
-	ABHD17B	9	71852452	71946233	45	8	10164	0.04957	0.48023
+	UBE2D1	10	58299979	58380753	89	5	10164	0.049536	0.48025
-	RPL9	4	39444124	39493948	191	24	10164	0.049459	0.48028
+	PSMD9	12	121853731	121927865	225	24	10164	0.049389	0.4803
+	PROL1	4	70362882	70420197	203	17	10164	0.049395	0.4803
+	COL9A3	20	62782062	62851159	193	37	10164	0.049387	0.48031
-	MPC2	1	167906675	167972069	23	4	10164	0.049196	0.48038
+	CATSPERG	19	38300783	38380949	417	39	10164	0.04899	0.48046
-	CLNS1A	11	77606151	77672806	128	11	10164	0.048969	0.48047
+	RBBP6	16	24504587	24582863	82	4	10164	0.048815	0.48053
+	EIF5	14	103299002	103355025	185	18	10164	0.048659	0.4806
+	PTS	11	112191365	112243973	92	13	10164	0.048427	0.48069
-	DUSP11	2	73752198	73815157	14	3	10164	0.048419	0.48069
-	SPAG7	17	4949226	5002837	137	21	10164	0.048432	0.48069
+	TSEN54	17	75480707	75534739	124	14	10164	0.048184	0.48078
+	LOC1053775	4	186123229	186168843	173	22	10164	0.048171	0.48079
-	PEX12	17	35564795	35613637	146	18	10164	0.047966	0.48087
+	TCF12	15	56883289	57301129	1023	24	10164	0.047922	0.48089
+	CLUAP1	16	3465945	3549048	320	22	10164	0.047887	0.4809
-	GKAP1	9	83729421	83852837	290	18	10164	0.047899	0.4809
+	KIF22	16	29755062	29815385	1	1	10164	0.04782	0.48093
-	C16orf54	16	29732463	29781329	1	1	10164	0.04782	0.48093
+	ZG16	16	29725827	29791648	1	1	10164	0.04782	0.48093
-	ANK3	10	60016298	60768526	2505	110	10164	0.047773	0.48095
+	PACRGL	4	20661287	20776315	464	23	10164	0.047674	0.48099
-	TMEM170A	16	75433244	75499737	195	12	10164	0.047628	0.48101
+	ZNF57	19	2865898	2928646	160	7	10164	0.047506	0.48105
-	ARAP1	11	72675069	72787389	299	22	10164	0.047326	0.48113
+	LOC1019273	18	10554366	10616403	218	15	10164	0.047085	0.48122
+	ARID1A	1	26661031	26792110	138	6	10164	0.047056	0.48123
+	CDKAL1	6	20499457	21242404	2528	127	10164	0.047068	0.48123
-	ZNF594	17	5169535	5226883	139	19	10164	0.046991	0.48126
+	HDAC9	7	18051942	19009521	2850	236	10164	0.046974	0.48127
-	NOTCH2	1	119901553	120104703	131	10	10164	0.046603	0.48141
+	TBC1D17	19	49842425	49898750	137	21	10164	0.046248	0.48156
-	EMP2	16	10518422	10615682	215	36	10164	0.045923	0.48169
-	MPP3	17	43790799	43868179	94	26	10164	0.04584	0.48172
-	HCN1	5	45244950	45731118	1075	43	10164	0.045746	0.48176
+	MPG	16	42019	95851	144	15	10164	0.045637	0.4818
-	LOC1005055	19	9777406	9828381	70	7	10164	0.045491	0.48186
+	C7orf34	7	142904506	142950870	74	3	10164	0.044879	0.4821
+	PDE3B	11	14608656	14882059	397	28	10164	0.044768	0.48215
+	LOC1053783	10	80614552	80663734	104	14	10164	0.044768	0.48215
-	ANKRD45	1	173598336	173740267	167	19	10164	0.044661	0.48219
+	URM1	9	128336319	128402016	108	11	10164	0.044336	0.48232
+	SLC35G1	10	93858973	93924255	22	12	10164	0.044313	0.48233
+	WBSCR17	7	71097414	71723601	2021	120	10164	0.044063	0.48243
-	ZBTB26	9	122908029	122966551	94	11	10164	0.043978	0.48246

STRAND	SYMBOL	CHR	START	STOP	NSNPS	NPARAM	N	ZSTAT	P
+	FBXO25	8	371808	479876	438	29	10164	0.043949	0.48247
-	ANKRD10	13	110868540	110950107	266	32	10164	0.043837	0.48252
+	LOC1019294	8	11642071	11692039	203	36	10164	0.043715	0.48257
+	LOC1053751	6	25238478	25289431	252	35	10164	0.043692	0.48257
+	TMEM106A	17	43176827	43230041	92	5	10164	0.043509	0.48265
+	SIK2	11	111567391	111742388	366	16	10164	0.043351	0.48271
-	CCDC39	3	180604008	180714495	120	16	10164	0.043262	0.48275
-	LOC1019300	12	92136489	92398858	397	45	10164	0.0428	0.48293
-	ABCA8	17	68857287	68990408	281	25	10164	0.042736	0.48296
+	SCN8A	12	51555193	51822864	329	24	10164	0.042642	0.48299
+	ZBTB48	1	6544991	6599280	179	19	10164	0.042629	0.483
+	TTC24	1	156544727	156597797	155	19	10164	0.042415	0.48308
+	CAPRIN1	11	34016617	34112610	111	11	10164	0.042399	0.48309
+	SCYL2	12	100232137	100351722	182	13	10164	0.04228	0.48314
-	HGC6.3	6	167965924	168011939	100	14	10164	0.042126	0.4832
+	PSORS1C1	6	31079831	31150092	1105	56	10164	0.041833	0.48332
-	ALOXE3	17	8085900	8153916	196	53	10164	0.041425	0.48348
-	AARS2	6	44287826	44348358	131	22	10164	0.04126	0.48354
-	DERL3	22	23824503	23874213	275	39	10164	0.040729	0.48376
+	AMY2A	1	103582332	103635780	20	7	10164	0.040363	0.4839
-	SEMA7A	15	74399289	74468958	223	32	10164	0.040379	0.4839
+	PEBP1	12	118101065	118155585	131	18	10164	0.040324	0.48392
+	LOC1053719	17	82906490	82952075	173	20	10164	0.040154	0.48399
-	ZNF432	19	52021378	52083820	168	13	10164	0.039864	0.4841
-	OR52H1	11	5534561	5580523	291	21	10164	0.039869	0.4841
+	PLN	6	118513279	118570424	144	15	10164	0.03983	0.48411
-	ATP6V1E2	2	46479355	46577412	303	31	10164	0.039557	0.48422
-	RWDD4	4	183629636	183694178	74	12	10164	0.039096	0.48441
-	SNX19	11	130865871	130951487	345	18	10164	0.03862	0.4846
-	S100A13	1	153608799	153669092	102	6	10164	0.038534	0.48463
-	GJD2	15	34742441	34789581	146	18	10164	0.038141	0.48479
-	PSG4	19	43182702	43240774	64	13	10164	0.03812	0.4848
+	P2RY6	11	73229496	73308625	138	14	10164	0.038075	0.48481
+	CSNK1G2	19	1906149	1991338	333	34	10164	0.037995	0.48485
+	UNCX	7	1198018	1246977	14	2	10164	0.037962	0.48486
-	STRADA	17	63692832	63776970	158	9	10164	0.037395	0.48508
-	CAMTA2	17	4957992	5022673	169	24	10164	0.037317	0.48512
-	TNIK	3	171052503	171495408	464	68	10164	0.036894	0.48528
+	FAM63B	15	58736194	58871900	242	21	10164	0.036842	0.48531
+	KYNU	2	142713928	143052316	847	61	10164	0.036764	0.48534
+	RCN1	11	32055931	32115726	136	15	10164	0.036682	0.48537
+	HBA2	16	137847	183710	106	21	10164	0.036407	0.48548
-	IFNA16	9	21206373	21252311	224	16	10164	0.036366	0.4855
+	EMID1	22	29170912	29269597	323	39	10164	0.036282	0.48553
-	VRK3	19	49966467	50061017	288	20	10164	0.036265	0.48554
-	DCST2	1	155007448	155068781	99	20	10164	0.036057	0.48562
+	CD83	6	14082256	14146918	196	27	10164	0.035859	0.4857
-	CYP3A5	7	99638189	99715026	119	11	10164	0.035368	0.48589
-	LYST	1	235651031	235918708	322	12	10164	0.035244	0.48594
+	MINPP1	10	87467792	87563461	140	18	10164	0.035197	0.48596
-	FIBP	11	65873740	65923539	134	22	10164	0.035121	0.48599
+	CLEC16A	16	10909488	11203278	678	24	10164	0.035033	0.48603
-	IL4I1	19	49879654	49964539	248	45	10164	0.035029	0.48603
+	OR7D2	19	9150594	9198817	169	18	10164	0.034699	0.48616
-	CSMD1	8	2925353	5029806	16866	629	10164	0.034646	0.48618
-	PDE4D	5	58959038	60557120	4520	206	10164	0.033817	0.48651
+	RBMS2	12	56486824	56606196	177	18	10164	0.033678	0.48657
-	PHYH	10	13267796	13335138	228	28	10164	0.033515	0.48663
+	CALU	7	128704292	128783423	229	29	10164	0.033469	0.48665
-	VPS33A	12	122221547	122301521	185	20	10164	0.033312	0.48671
+	AGPS	2	177357743	177553836	484	20	10164	0.033268	0.48673
-	SMIM7	19	16620743	16695157	148	15	10164	0.033168	0.48677
+	CLEC12A	12	9916316	10009354	397	27	10164	0.033155	0.48678

STRAND	SYMBOL	CHR	START	STOP	NSNPS	NPARAM	N	ZSTAT	P
+	USP25	21	15694947	15890072	279	11	10164	0.0331	0.4868
-	TTLL8	22	50008575	50094132	354	33	10164	0.032981	0.48684
+	ZBTB32	19	35669492	35727048	206	26	10164	0.03259	0.487
+	WDR62	19	36019881	36115111	211	24	10164	0.032595	0.487
-	CRP	1	159702289	159749609	141	19	10164	0.03249	0.48704
+	FBXL19	16	30888055	30958783	62	5	10164	0.03235	0.4871
-	NPPC	2	231912095	231961389	87	18	10164	0.032274	0.48713
-	TBC1D9B	5	179852071	179942856	202	27	10164	0.032204	0.48715
-	SLC8A3	14	70034217	70258612	865	72	10164	0.032189	0.48716
+	NUF2	1	163286933	163365764	294	19	10164	0.032185	0.48716
-	LOC1053788	1	74459617	74511533	49	7	10164	0.032057	0.48721
+	TCF25	16	89838586	89921387	319	16	10164	0.031895	0.48728
-	SEPSECS	4	25110005	25195582	207	31	10164	0.031786	0.48732
+	MAP2K3	17	21249656	21325240	103	15	10164	0.03175	0.48734
-	KHDC1	6	73231314	73345365	407	25	10164	0.031588	0.4874
+	MARCKS	6	113822335	113873475	139	28	10164	0.031194	0.48756
+	GLRX3	10	130101375	130190382	397	39	10164	0.031195	0.48756
+	ACSM6	10	95159200	95238929	227	13	10164	0.031075	0.4876
-	MIPEP	13	23720188	23924448	694	40	10164	0.031053	0.48761
+	PPP2R3A	3	135930673	136157910	458	29	10164	0.031069	0.48761
+	MED12L	3	151050665	151446678	1011	55	10164	0.030877	0.48768
+	INCENP	11	62088973	62163163	290	21	10164	0.030671	0.48777
-	ZNF433	19	12004717	12070710	152	12	10164	0.03065	0.48777
+	DNAL1	14	73609875	73713728	326	25	10164	0.030633	0.48778
-	SUSD1	9	112030781	112210520	548	65	10164	0.030475	0.48784
-	TAMM41	3	11780442	11881919	223	24	10164	0.030488	0.48784
-	C22orf23	22	37933050	37988669	76	14	10164	0.030432	0.48786
+	ZNF354B	5	178824953	178894434	410	18	10164	0.03016	0.48797
-	RSBN1	1	113751832	113847476	162	22	10164	0.030083	0.488
-	SESTD1	2	179091692	179299623	495	29	10164	0.029652	0.48817
-	PHLDB3	19	43464341	43539920	219	29	10164	0.029623	0.48818
+	MPHOSPH1(	2	71095314	71160102	212	18	10164	0.02937	0.48828
+	ACCSL	11	43886084	44070307	808	80	10164	0.029354	0.48829
-	GSTM3	1	109723932	109776038	69	11	10164	0.029285	0.48832
+	BAIAP3	16	1298605	1359441	126	21	10164	0.029283	0.48832
-	UBAC1	9	135922969	135996380	118	10	10164	0.029265	0.48833
-	C1D	2	68032200	68098027	66	9	10164	0.02917	0.48836
+	USP53	4	119177618	119305518	347	19	10164	0.029038	0.48842
+	ZNF439	19	11813530	11879491	3	2	10164	0.028872	0.48848
+	CHL1	3	161596	419417	795	45	10164	0.028757	0.48853
+	RHOBTB2	8	22952258	23030197	220	42	10164	0.02873	0.48854
+	KIAA0513	16	84992751	85104230	580	42	10164	0.028595	0.48859
-	LILRA4	19	54323185	54374169	32	4	10164	0.028106	0.48879
-	DCTN3	9	34603545	34655523	115	21	10164	0.027933	0.48886
-	GP2	16	20299574	20362820	147	26	10164	0.027814	0.48891
-	OR5V1	6	29345230	29492067	1156	41	10164	0.0276	0.48899
+	C1GALT1	7	7147547	7258651	381	22	10164	0.027418	0.48906
+	TTC6	14	37560199	37852628	449	43	10164	0.027427	0.48906
+	ARMC8	3	138152248	138308389	91	8	10164	0.02736	0.48909
-	ZNF792	19	34946354	34999282	195	24	10164	0.027332	0.4891
+	GIMAP7	7	150479857	150531073	215	26	10164	0.027336	0.4891
+	CDH7	18	65715252	65890939	320	17	10164	0.027245	0.48913
-	SBNO2	19	1097634	1209283	333	30	10164	0.026888	0.48927
-	ODF2L	1	86336824	86431342	337	16	10164	0.026798	0.48931
+	TMEM38A	19	16626127	16699019	154	14	10164	0.026713	0.48934
+	HTRA1	10	122426525	122524908	345	35	10164	0.026629	0.48938
+	POU4F3	5	146304024	146350520	63	5	10164	0.026245	0.48953
+	FBXO6	1	11629093	11684354	169	31	10164	0.026171	0.48956
-	GPR87	3	151284086	151351848	201	20	10164	0.025978	0.48964
-	BRCA1	17	43034295	43160483	268	7	10164	0.025612	0.48978
+	CAMK4	5	111189249	111504886	801	61	10164	0.025558	0.4898
+	BCKDHB	6	80071627	80356270	1201	32	10164	0.025557	0.48981
+	MFF	2	227290151	227367836	283	29	10164	0.025514	0.48982

STRAND	SYMBOL	CHR	START	STOP	NSNPS	NPARAM	N	ZSTAT	P
+	FOSL2	2	28357759	28427312	150	27	10164	0.025423	0.48986
+	CCDC54	3	107342341	107388634	154	24	10164	0.02517	0.48996
-	CYP17A1	10	102820531	102872533	174	26	10164	0.025109	0.48998
-	ACYP1	14	75043221	75104483	123	9	10164	0.024755	0.49013
-	PLEK2	14	67375659	67447174	213	29	10164	0.024488	0.49023
+	SMCP	1	152843322	152895047	86	9	10164	0.024448	0.49025
-	CASP12	11	104875718	104933670	160	18	10164	0.024292	0.49031
+	WBP1L	10	102704283	102826265	396	33	10164	0.02413	0.49037
-	LPIN2	18	2906994	3048315	281	31	10164	0.024069	0.4904
+	OGFR	20	62769825	62824000	191	29	10164	0.024045	0.49041
-	ASCL3	11	8927572	8978033	136	12	10164	0.023982	0.49043
+	FEN1	11	61757637	61807244	62	10	10164	0.023912	0.49046
+	ZBED6	1	203762523	203810462	180	20	10164	0.023874	0.49048
+	ARHGAP44	17	12754512	13001643	916	67	10164	0.023851	0.49049
+	OR5H14	3	98113718	98160399	220	8	10164	0.023727	0.49054
-	SIX1	14	60634699	60684437	84	10	10164	0.023619	0.49058
-	COLGALT2	1	183919662	184086611	395	34	10164	0.023388	0.49067
-	TNNT1	19	55122698	55184354	245	30	10164	0.023299	0.49071
-	CLEC2A	12	9870903	9967407	296	23	10164	0.023118	0.49078
+	FAM71C	12	99612750	99660114	90	11	10164	0.023004	0.49082
-	SLC6A9	1	43986483	44066492	164	38	10164	0.022974	0.49084
-	SF3B1	2	197381974	197470091	74	8	10164	0.022957	0.49084
-	LOC1010604	17	48624804	48682072	210	34	10164	0.022924	0.49086
+	WTIP	19	34446901	34519133	93	13	10164	0.022891	0.49087
+	EPO	7	100685800	100733700	83	11	10164	0.022816	0.4909
+	FKBP1B	2	24014706	24077743	177	22	10164	0.022778	0.49091
+	IFNAR2	21	33194895	33274513	237	23	10164	0.02253	0.49101
+	MAPT	17	45859382	46038334	32	11	10164	0.02253	0.49101
-	RRP9	3	51923426	51976941	69	10	10164	0.022045	0.49121
+	LCE2D	1	152628411	152674659	134	4	10164	0.02171	0.49134
+	OR4B1	11	48181810	48227739	176	14	10164	0.0215	0.49142
+	AKAP7	6	131100686	131293535	562	55	10164	0.021487	0.49143
+	DCTN6	8	30121297	30193639	175	15	10164	0.021412	0.49146
+	ZNF720	16	31678229	31804641	274	13	10164	0.021219	0.49154
+	EXOC4	7	133218067	134075761	2208	80	10164	0.02107	0.49159
-	ZNF3	7	100053847	100117548	118	20	10164	0.020951	0.49164
-	IL17REL	22	49984513	50048198	157	22	10164	0.020799	0.4917
-	ZNF614	19	52003324	52063427	158	12	10164	0.020696	0.49174
+	CHD3	17	7849776	7922757	100	19	10164	0.020432	0.49185
-	NIPAL2	8	98179826	98330442	294	33	10164	0.020412	0.49186
+	LRRC63	13	46176911	46286818	387	30	10164	0.020375	0.49187
+	SYT1	12	78828993	79462008	922	60	10164	0.020296	0.4919
-	PSAPL1	4	7420294	7469973	224	41	10164	0.020257	0.49192
-	GOLGA6A	15	74057654	74117747	36	14	10164	0.020202	0.49194
-	ATP5G3	2	175166258	175216762	144	13	10164	0.020049	0.492
+	ATP6V0A2	12	123677318	123771755	270	10	10164	0.019922	0.49205
-	TEKT5	16	10571369	10777416	700	42	10164	0.019688	0.49215
+	SUGT1	13	52617696	52698298	146	8	10164	0.019573	0.49219
-	RC3H2	9	122839453	122940283	154	14	10164	0.019534	0.49221
+	MIA	19	40740177	40787493	90	10	10164	0.019503	0.49222
-	RAB29	1	205757986	205810487	156	16	10164	0.019444	0.49224
+	PAICS	4	56400749	56471368	208	23	10164	0.019433	0.49225
-	SPRYD3	12	53054316	53114420	142	18	10164	0.019342	0.49228
+	ARRDC1	9	137570640	137625360	74	5	10164	0.019293	0.4923
+	PCDHGA2	5	141303760	141522979	460	34	10164	0.019084	0.49239
-	AGPAT2	9	136663143	136722459	65	6	10164	0.018987	0.49243
-	SMAD7	18	48909853	48985711	258	52	10164	0.018688	0.49254
-	CXXC1	18	50272343	50323322	151	22	10164	0.018692	0.49254
+	OR5H1	3	98097698	98143639	220	8	10164	0.018275	0.49271
+	SDF2L1	22	21607253	21654299	92	14	10164	0.018082	0.49279
+	SNX22	15	64116698	64167481	47	5	10164	0.017963	0.49283
-	BAAT	9	101350417	101420005	242	23	10164	0.017907	0.49286
-	LOC1019280	15	32563958	32649746	52	11	10164	0.017784	0.49291

STRAND	SYMBOL	CHR	START	STOP	NSNPS	NPARAM	N	ZSTAT	P
+	IL4R	16	27278668	27374778	206	21	10164	0.017696	0.49294
+	LOC1010604	9	32424521	32484910	124	22	10164	0.01757	0.49299
+	PPIAL4C	1	149548823	149594607	8	2	10164	0.017236	0.49312
+	STIM2	4	26825691	27035381	289	26	10164	0.017188	0.49314
-	LOC1053728	21	45045877	45108903	212	32	10164	0.017144	0.49316
-	PTTG1IP	21	44839585	44908903	254	30	10164	0.01694	0.49324
+	CCDC33	15	74182197	74346472	565	70	10164	0.016641	0.49336
-	SHISA5	3	48457798	48539826	103	14	10164	0.016474	0.49343
-	DDX51	12	132126594	132179335	14	7	10164	0.016207	0.49353
+	CDHR3	7	105842200	106046431	629	74	10164	0.016198	0.49354
-	MKNK1	1	46547407	46639294	250	32	10164	0.016102	0.49358
-	ACER1	19	6296142	6388444	100	2	10164	0.015623	0.49377
-	TSNARE1	8	142202080	142438291	1091	54	10164	0.015446	0.49384
+	PSMG4	6	3219449	3313019	309	14	10164	0.015437	0.49384
+	EDN1	6	12226214	12307194	212	41	10164	0.01536	0.49387
-	VHLL	1	156288624	156334637	124	11	10164	0.015156	0.49395
-	TUBA3E	2	130181745	130233461	88	3	10164	0.01485	0.49408
-	KCNQ3	8	132110858	132515757	1167	119	10164	0.014709	0.49413
-	TRIT1	1	39831031	39918511	207	18	10164	0.014703	0.49413
-	MBOAT4	8	30121454	30193699	176	15	10164	0.014508	0.49421
-	LGI4	19	35114513	35174451	157	23	10164	0.01449	0.49422
+	TM9SF2	13	99466374	99574048	315	24	10164	0.014392	0.49426
+	TUBB1	20	58984254	59036654	51	7	10164	0.014375	0.49427
+	MIDN	19	1213553	1269143	102	15	10164	0.014371	0.49427
-	GMNC	3	190833268	190897743	157	21	10164	0.014351	0.49428
-	POGZ	1	151392724	151494465	187	17	10164	0.014184	0.49434
+	OR2F1	7	143872126	143971015	238	20	10164	0.014106	0.49437
-	ARID3C	9	34611286	34663950	114	21	10164	0.014049	0.4944
+	CA9	9	35638918	35691159	198	34	10164	0.013976	0.49442
-	ORC5	7	104116341	104243047	554	27	10164	0.013775	0.4945
-	TNFRSF9	1	7905871	7978165	130	12	10164	0.013446	0.49464
+	KLHDC10	7	130035509	130145720	188	18	10164	0.013333	0.49468
+	LRRC27	10	132295063	132391506	173	15	10164	0.01322	0.49473
+	ANKRD24	19	4147625	4234814	37	7	10164	0.013196	0.49474
+	NMS	2	100435482	100493280	350	8	10164	0.013123	0.49476
+	MAPKAPK3	3	50576862	50659297	151	15	10164	0.012904	0.49485
+	GPER1	7	1051807	1103815	232	28	10164	0.012894	0.49486
-	MMP19	12	55825430	55877983	50	16	10164	0.012856	0.49487
-	ZNF445	3	44429770	44512670	60	6	10164	0.012421	0.49504
+	WDR43	2	28859643	28958220	292	14	10164	0.01213	0.49516
+	FOXC2	16	86532251	86578933	192	26	10164	0.011788	0.4953
-	GGT5	22	24209654	24280142	169	21	10164	0.011699	0.49533
+	TWSG1	18	9299767	9412420	292	18	10164	0.011527	0.4954
+	FAM132B	2	238124008	238178890	199	38	10164	0.01107	0.49558
-	TMED7	5	115603208	115661179	203	12	10164	0.011065	0.49559
+	ANAPC4	4	25342226	25428498	272	29	10164	0.010834	0.49568
-	TBC1D3F	17	36418612	36474523	11	2	10164	0.010541	0.49579
+	SYNPO2	4	118815688	119071247	1065	85	10164	0.010284	0.4959
-	CCL26	7	75759524	75824746	224	21	10164	0.010173	0.49594
-	ZNF503	10	75269726	75436894	274	41	10164	0.010087	0.49598
-	C19orf35	19	2264632	2317182	171	25	10164	0.0099942	0.49601
+	IL6	7	22690442	22742002	249	31	10164	0.0099673	0.49602
-	MGAT4A	2	98609106	98766126	407	41	10164	0.0099166	0.49604
-	MYOF	10	93296429	93517486	704	53	10164	0.009881	0.49606
-	BIN3	8	22610418	22704148	135	12	10164	0.0097849	0.4961
-	FSD2	15	82745365	82841211	235	31	10164	0.0097507	0.49611
-	ADGRA3	4	22377374	22551058	780	55	10164	0.0097427	0.49611
-	LOC1027238	4	186406044	186522637	528	55	10164	0.0095453	0.49619
+	SEC11C	18	59104884	59168837	267	22	10164	0.009262	0.49631
+	LOC1005055	18	57595302	57679276	308	32	10164	0.0092052	0.49633
+	ZNF71	19	56560296	56634481	176	19	10164	0.0090745	0.49638
+	PIBF1	13	72747092	73026454	764	30	10164	0.0089903	0.49641
-	UQCRHL	1	15797162	15842699	191	13	10164	0.0089292	0.49644

STRAND	SYMBOL	CHR	START	STOP	NSNPS	NPARAM	N	ZSTAT	P
+	CLPP	19	6326452	6378904	81	1	10164	0.0087774	0.4965
-	SUPT16H	14	21341472	21419639	30	1	10164	0.0087052	0.49653
-	ZNF641	12	48325491	48386414	196	27	10164	0.0085703	0.49658
+	RALBP1	18	9440532	9548108	244	19	10164	0.007985	0.49681
-	TRAPPC8	18	31819173	31978212	413	23	10164	0.0079709	0.49682
-	SEPT10	2	109532797	109649206	366	23	10164	0.0078258	0.49688
-	DENND2A	7	140508418	140676732	54	7	10164	0.0075958	0.49697
+	STARD9	15	42540634	42730998	388	38	10164	0.007589	0.49697
-	CAPN13	2	30712771	30842484	463	57	10164	0.0072072	0.49712
-	CTSZ	20	58985187	59042254	56	7	10164	0.0071395	0.49715
-	SMNDC1	10	110283040	110339949	127	24	10164	0.0069563	0.49722
+	OR2M3	1	248168068	248214006	85	6	10164	0.0067611	0.4973
+	LASP1	17	38834859	38931770	331	50	10164	0.0067496	0.49731
-	KLRC4-KLRK	12	10362353	10445146	94	9	10164	0.006421	0.49744
+	TNFRSF14	1	2521365	2575622	98	12	10164	0.006147	0.49755
-	TUBGCP2	10	133268630	133347337	76	6	10164	0.005879	0.49765
-	MAPK1	22	21749657	21902680	355	24	10164	0.0057069	0.49772
+	RPUSD2	15	40534293	40584706	213	19	10164	0.0056707	0.49774
-	SNX20	16	50656300	50716353	144	23	10164	0.0056559	0.49774
+	ZNF282	7	149160462	149236248	176	16	10164	0.0055812	0.49777
+	BMP6	6	7691778	7891728	547	49	10164	0.0055766	0.49778
+	PNLIPRP3	10	116392879	116487961	307	25	10164	0.0054896	0.49781
+	CTSA	20	45855952	45908820	185	24	10164	0.0053547	0.49786
-	DBR1	3	138150988	138209949	57	3	10164	0.0047685	0.4981
+	LONP2	16	48209167	48363979	228	6	10164	0.004643	0.49815
+	PCIF1	20	45899674	45958023	170	32	10164	0.0043464	0.49827
+	CNOT2	12	70207994	70364993	579	35	10164	0.0042491	0.4983
+	PPP1R1B	17	39591208	39646625	80	12	10164	0.0042362	0.49831
-	DCN	12	91135258	91218029	177	20	10164	0.0042155	0.49832
+	ZMPSTE24	1	40223050	40304184	175	19	10164	0.0039673	0.49842
-	CELSR3	3	48626463	48697915	97	10	10164	0.003938	0.49843
+	ERVV-1	19	52979091	53026580	145	11	10164	0.0038179	0.49848
-	LOC1053698	12	71420074	71485832	168	16	10164	0.0037902	0.49849
+	ATG16L1	2	233216571	233305674	271	25	10164	0.0037385	0.49851
-	UIMC1	5	176895005	177057571	359	18	10164	0.0035867	0.49857
-	GNS	12	64703442	64794446	110	8	10164	0.0034279	0.49863
-	NEUROD2	17	39593768	39642922	71	10	10164	0.00343	0.49863
-	MSR1	8	16097878	16227791	655	32	10164	0.0033737	0.49865
-	NPFF	12	53496690	53542638	85	15	10164	0.0032327	0.49871
+	DDX19B	16	70254767	70343832	66	22	10164	0.0031529	0.49874
-	USP48	1	21668298	21818199	220	16	10164	0.0028879	0.49885
+	TAS2R41	7	143442873	143488796	104	16	10164	0.0028796	0.49885
-	ZNF43	19	21794949	21887068	285	34	10164	0.0026826	0.49893
+	BBS12	4	122665444	122754943	213	18	10164	0.0026732	0.49893
-	SREBF1	17	17801349	17872017	198	27	10164	0.0025627	0.49898
-	C15orf57	15	40521341	40600057	305	18	10164	0.0023722	0.49905
-	ZNF506	19	19782711	19856751	122	14	10164	0.0023344	0.49907
+	OSBPL6	2	178159481	178409433	783	47	10164	0.0020266	0.49919
-	CD226	18	69849459	69996787	195	18	10164	0.0019473	0.49922
+	CD55	1	207286472	207370966	159	10	10164	0.0018846	0.49925
-	FOXD4	9	106231	153417	60	11	10164	0.0015654	0.49938
+	KMO	1	241496889	241605647	269	39	10164	0.0015394	0.49939
-	COX11	17	54941898	55003780	253	28	10164	0.0015019	0.4994
+	STX17	9	99871633	99989337	166	11	10164	0.00139	0.49945
+	C7orf55-LUC	7	139306132	139433457	251	3	10164	0.0013733	0.49945
+	GCNT2	6	10486283	10639368	461	49	10164	0.0012602	0.4995
-	DIABLO	12	122197662	122262534	171	22	10164	0.0011737	0.49953
+	CDH4	20	61217426	61950617	1888	189	10164	0.00094018	0.49962
-	ZFP36L2	2	43212402	43261606	114	31	10164	0.0009181	0.49963
+	ADAMTS19	5	129425399	129748763	1092	57	10164	0.00087244	0.49965
+	USP42	7	6043743	6171564	398	34	10164	0.00079927	0.49968
+	RGS18	1	192123462	192197172	130	16	10164	0.00063424	0.49975
-	LECT2	5	135936911	135990034	208	16	10164	0.00043747	0.49983

STRAND	SYMBOL	CHR	START	STOP	NSNPS	NPARAM	N	ZSTAT	P
-	ZBTB6	9	122898056	122948328	75	9	10164	0.00011655	0.49995
+	HEMK1	3	50534180	50599050	100	13	10164	-7.01E-05	0.50003
-	CXCL3	4	74026589	74073773	42	13	10164	-9.48E-05	0.50004
+	CRHR1	17	45749280	45845828	16	6	10164	-0.00059685	0.50024
-	PRLHR	10	118581925	118634895	127	20	10164	-0.00059594	0.50024
+	PTF1A	10	23157531	23204252	66	18	10164	-0.0013558	0.50054
+	L3MBTL3	6	129943804	130151449	569	55	10164	-0.0013997	0.50056
+	TMEM233	12	119557636	119664442	268	23	10164	-0.0014674	0.50059
+	BMPR1B	4	94722977	95168450	1492	68	10164	-0.0017179	0.50069
+	MGA	15	41586232	41779943	373	27	10164	-0.0017844	0.50071
+	PRKG1	10	50956151	52308350	3799	238	10164	-0.001994	0.5008
-	PIP4K2B	17	38755689	38834965	158	17	10164	-0.0020894	0.50083
+	SPCS2	11	74914247	74989031	109	12	10164	-0.0021014	0.50084
-	ZNF98	19	22381011	22457362	253	21	10164	-0.0022065	0.50088
-	NAALADL1	11	65034818	65094021	140	22	10164	-0.0022801	0.50091
-	NATD1	17	21228870	21288266	93	14	10164	-0.0023433	0.50093
-	USP6NL	10	11450510	11646789	453	26	10164	-0.0023713	0.50095
+	DNAH1	3	52281319	52410497	269	28	10164	-0.0025852	0.50103
+	CNST	1	246531337	246678587	463	39	10164	-0.002596	0.50104
-	MCOLN3	1	85008082	85083902	210	24	10164	-0.0026273	0.50105
+	GLTSCR1L	6	42747052	42878560	225	27	10164	-0.00283	0.50113
-	PLK2	5	58443982	58495139	224	28	10164	-0.0028473	0.50114
-	PRSS37	7	141826278	141877499	176	13	10164	-0.0028881	0.50115
-	C4orf45	4	158883532	159117867	558	33	10164	-0.0028793	0.50115
+	LUC7L2	7	139305359	139433457	252	3	10164	-0.003107	0.50124
+	ZNF114	19	48235099	48297608	259	23	10164	-0.0031502	0.50126
-	DNAJB12	10	72322830	72390149	199	13	10164	-0.0031899	0.50127
+	BHLHA15	7	98177254	98222959	154	27	10164	-0.0032373	0.50129
-	CHAD	17	50454492	50504881	83	8	10164	-0.0033634	0.50134
+	C12orf60	12	14750217	14833858	278	37	10164	-0.0033982	0.50136
+	SLC22A7	6	43260710	43315538	127	22	10164	-0.0035952	0.50143
-	PPAT	4	56383362	56470636	257	25	10164	-0.0036366	0.50145
+	CHODL	21	17784595	18277373	880	62	10164	-0.003708	0.50148
-	CHD1	5	98845204	98963623	217	23	10164	-0.0039423	0.50157
-	MYCL	1	39885424	39937015	104	14	10164	-0.0039404	0.50157
-	PLA2G10	16	14662548	14729669	4	2	10164	-0.0040078	0.5016
+	AP5M1	14	57233888	57308742	87	9	10164	-0.0040157	0.5016
-	YWHAG	7	76316791	76394025	127	11	10164	-0.0040457	0.50161
-	DLC1	8	13073361	13550658	2474	109	10164	-0.0040766	0.50163
-	PSG1	19	42856461	42914719	140	4	10164	-0.0041444	0.50165
+	LOC1001289	2	173989721	174037158	110	15	10164	-0.0042886	0.50171
-	RBM47	4	40413255	40665786	414	58	10164	-0.0043468	0.50173
+	ZNF346	5	176987680	177091188	259	15	10164	-0.0043833	0.50175
-	C1orf109	1	37671570	37727336	186	16	10164	-0.0046149	0.50184
+	RBAK	7	5010821	5079488	308	19	10164	-0.0047449	0.50189
-	SOHLH1	9	135683407	135736316	94	15	10164	-0.0047364	0.50189
+	NT5E	6	85414584	85505791	219	15	10164	-0.0047985	0.50191
+	EYA4	6	133206335	133542120	985	44	10164	-0.0048866	0.50195
-	TUBA4A	2	219239711	219289608	95	9	10164	-0.0049219	0.50196
+	NCAM2	21	20963315	21550630	2111	115	10164	-0.0049525	0.50198
-	LOC1053768	17	60109031	60155552	56	11	10164	-0.0049726	0.50198
-	IFNA5	9	21294614	21340313	137	16	10164	-0.0050873	0.50203
+	TBX4	17	61409626	61495110	143	23	10164	-0.0053	0.50211
-	NUDCD3	7	44372366	44525878	251	8	10164	-0.0053996	0.50215
-	MDGA2	14	46829623	47710334	2765	92	10164	-0.0056142	0.50224
-	LOC1053707	14	104124783	104173629	44	8	10164	-0.0061499	0.50245
-	C9orf152	9	110189561	110243133	205	26	10164	-0.0061665	0.50246
+	KDM4A	1	43615126	43715518	202	18	10164	-0.0065601	0.50262
+	PHF7	3	52375558	52433641	109	17	10164	-0.006629	0.50264
+	PTPDC1	9	93995794	94119856	260	29	10164	-0.0066454	0.50265
+	WRB	21	39344676	39407889	140	15	10164	-0.0067062	0.50268
+	TTC7A	2	46881129	47086136	735	53	10164	-0.0068204	0.50272
-	NDUFS1	2	206113079	206194519	224	14	10164	-0.0068657	0.50274

STRAND	SYMBOL	CHR	START	STOP	NSNPS	NPARAM	N	ZSTAT	P
-	FAM78B	1	166047426	166201755	439	27	10164	-0.0069692	0.50278
+	LOC1053724	19	40408184	40455263	107	12	10164	-0.0070127	0.5028
+	IQCJ	3	159034252	159276307	561	40	10164	-0.0073642	0.50294
-	ZNF560	19	9435826	9541689	396	14	10164	-0.0075044	0.50299
+	LOC441155	6	65266418	65315613	75	9	10164	-0.007923	0.50316
+	SOX13	1	204038109	204137743	253	41	10164	-0.0080887	0.50323
+	CASQ1	1	160155495	160211886	192	45	10164	-0.0081329	0.50324
+	FKBP4	12	2759942	2815423	90	15	10164	-0.0082419	0.50329
-	TSPAN8	12	71115093	71192999	295	23	10164	-0.0083367	0.50333
-	SGOL1	3	20149046	20223131	297	21	10164	-0.0083957	0.50335
-	OR11L1	1	247830928	247876896	145	11	10164	-0.008821	0.50352
+	PKIA	8	78481101	78615267	183	16	10164	-0.0089808	0.50358
-	TAC4	17	49828309	49883017	118	18	10164	-0.0091469	0.50365
-	VPS35	16	46647995	46724232	16	3	10164	-0.0095461	0.50381
+	TBC1D21	15	73838575	73919783	279	28	10164	-0.0099758	0.50398
-	RBM12B	8	93721500	93776012	72	9	10164	-0.010062	0.50401
-	SP6	17	47834908	47911190	277	50	10164	-0.010264	0.50409
+	LOC1001307	7	128831410	128882047	138	11	10164	-0.010243	0.50409
-	LOC1019285	3	44670777	44720782	44	8	10164	-0.01036	0.50413
-	S100A14	1	153604255	153651332	80	5	10164	-0.010547	0.50421
-	PNMA1	14	73701783	73749425	157	26	10164	-0.01069	0.50426
+	ZNF621	3	40489316	40549794	94	14	10164	-0.010802	0.50431
+	SKIV2L	6	31923804	31979755	253	62	10164	-0.010899	0.50435
-	SAV1	14	50623580	50703357	171	13	10164	-0.010987	0.50438
-	RNF215	22	30368812	30422313	157	22	10164	-0.011106	0.50443
+	ZNF480	19	52262169	52335927	302	23	10164	-0.011122	0.50444
+	PGM5	9	68321899	68541061	427	40	10164	-0.011226	0.50448
-	CUTA	6	33406542	33453288	134	39	10164	-0.011364	0.50453
+	QPCTL	19	45657381	45713990	193	27	10164	-0.011365	0.50453
-	DTNB	2	25367198	25708647	564	65	10164	-0.011543	0.5046
-	ATF7	12	53497856	53661415	442	25	10164	-0.011554	0.50461
+	SLC39A9	14	69363379	69478403	270	24	10164	-0.012021	0.5048
-	PGAM2	7	44052727	44100972	130	16	10164	-0.012364	0.50493
-	SYNE3	14	95406083	95551668	533	85	10164	-0.012474	0.50498
-	C11orf71	11	114381448	114435550	92	8	10164	-0.012783	0.5051
+	PITPNC1	17	67342281	67707263	558	84	10164	-0.013153	0.50525
-	ARHGAP20	11	110566855	110748189	583	31	10164	-0.013346	0.50532
-	NUPR1L	7	56104681	56151397	177	22	10164	-0.013479	0.50538
-	CALCR	7	93414487	93609730	601	44	10164	-0.014167	0.50565
-	NUAK1	12	106053347	106175033	233	24	10164	-0.014176	0.50566
-	TSPAN12	7	120777320	120893366	290	11	10164	-0.014468	0.50577
+	MS4A14	11	60361014	60427756	271	29	10164	-0.014598	0.50582
-	SERTAD1	19	40412501	40461025	118	12	10164	-0.01485	0.50592
-	TFF2	21	42336357	42386099	127	19	10164	-0.014893	0.50594
-	CENPC	4	67462271	67580538	302	14	10164	-0.01516	0.50605
+	AMN	14	102887548	102940847	105	19	10164	-0.015172	0.50605
+	CD1D	1	158143038	158196427	136	11	10164	-0.015463	0.50617
-	SF1	11	64754604	64813844	110	20	10164	-0.015557	0.50621
-	ZNF367	9	96375941	96453720	175	11	10164	-0.015616	0.50623
-	MTRNR2L2	5	80640000	80686035	321	14	10164	-0.015707	0.50627
+	TNFSF9	19	6493562	6545928	12	2	10164	-0.015916	0.50635
-	TUBB4A	19	6484319	6537848	12	2	10164	-0.015916	0.50635
+	LCE1F	1	152741372	152786969	121	10	10164	-0.015951	0.50636
-	PACSIN3	11	47167522	47221459	92	12	10164	-0.015971	0.50637
+	ZNF830	17	34926530	34973186	79	20	10164	-0.016367	0.50653
-	INPP5K	17	1484575	1551888	183	15	10164	-0.016858	0.50672
-	LOC1053767	1	9986423	10033089	62	13	10164	-0.016947	0.50676
+	DNASE1	16	3576745	3672875	273	18	10164	-0.017296	0.5069
+	FCRLA	1	161671972	161724352	183	29	10164	-0.017619	0.50703
-	ACY3	11	67632555	67685807	187	27	10164	-0.017617	0.50703
+	RPL11	1	23656779	23706425	124	15	10164	-0.01817	0.50725
-	MMS22L	6	97132161	97318437	414	21	10164	-0.018337	0.50732
+	ENO3	17	4914182	4967131	137	24	10164	-0.018566	0.50741

STRAND	SYMBOL	CHR	START	STOP	NSNPS	NPARAM	N	ZSTAT	P
+	ARHGAP11A	15	32578733	32649949	53	11	10164	-0.018844	0.50752
+	RPA1	17	1794979	1909554	396	30	10164	-0.019111	0.50762
+	SENP8	15	72079258	72150970	113	13	10164	-0.019118	0.50763
+	RNF11	1	51201273	51283447	84	16	10164	-0.019153	0.50764
+	MAP2K1	15	66351873	66501544	444	29	10164	-0.019178	0.50765
-	FIBCD1	9	130892438	130974068	159	22	10164	-0.01938	0.50773
+	DNAJB11	3	186535676	186595800	113	6	10164	-0.019838	0.50791
-	SRSF4	1	29137738	29217125	155	17	10164	-0.019929	0.50795
-	MICU1	10	72357326	72661191	584	24	10164	-0.020078	0.50801
+	PROCR	20	35136937	35225989	221	20	10164	-0.020182	0.50805
+	CTRC	1	15403442	15459247	114	20	10164	-0.0202	0.50806
-	OSTM1	6	108031409	108109737	170	12	10164	-0.020337	0.50811
-	PTPN14	1	214326942	214586681	736	54	10164	-0.020472	0.50817
-	SLC12A7	5	1040374	1190747	503	63	10164	-0.020841	0.50831
-	MBD1	18	50256623	50316774	163	25	10164	-0.020851	0.50832
-	SLCO5A1	8	69657046	69870064	508	58	10164	-0.020914	0.50834
-	ABLIM1	10	114421110	114856643	1076	86	10164	-0.020933	0.50835
+	SEC22A	3	123166927	123284136	341	11	10164	-0.021003	0.50838
+	INADL	1	61707477	62173920	1234	91	10164	-0.021064	0.5084
+	ZYG11A	1	52807511	52904575	221	23	10164	-0.021069	0.5084
+	KIF25	6	167967543	168055089	177	18	10164	-0.021857	0.50872
+	PLCE1	10	93958989	94338392	814	78	10164	-0.021906	0.50874
+	PRPH	12	49260013	49308698	158	21	10164	-0.022238	0.50887
+	MOB4	2	197480571	197563699	82	8	10164	-0.022393	0.50893
+	SDHAF4	6	70531922	70598903	252	19	10164	-0.022503	0.50898
-	UMOD	16	20323051	20391301	186	30	10164	-0.022752	0.50908
+	SUSD6	14	69576593	69725144	217	25	10164	-0.022901	0.50914
+	EVL	14	99984387	100154236	495	36	10164	-0.022966	0.50916
-	MRPS14	1	175002958	175058455	137	25	10164	-0.023118	0.50922
+	LOC1019270	14	90013134	90071730	130	14	10164	-0.02317	0.50924
+	SAFB	19	5588035	5678478	173	16	10164	-0.023377	0.50933
-	WDR47	1	108960211	109077228	149	18	10164	-0.023443	0.50935
+	ZFP90	16	68504289	68586072	273	11	10164	-0.023521	0.50938
-	PRSS33	16	2773953	2822607	4	1	10164	-0.02354	0.50939
-	CD69	12	9742486	9795901	203	16	10164	-0.023588	0.50941
+	UTP23	8	116731503	116858959	275	33	10164	-0.02387	0.50952
+	GPR62	3	51920314	51967504	65	10	10164	-0.023859	0.50952
-	GATSL3	22	30275118	30324627	113	29	10164	-0.023965	0.50956
+	TMEM45A	3	100457619	100587453	397	23	10164	-0.024036	0.50959
+	FXYD5	19	35119716	35179885	183	25	10164	-0.024072	0.5096
-	GCLC	6	53487341	53580129	252	40	10164	-0.024282	0.50969
-	C15orf52	15	40321452	40375967	166	36	10164	-0.024306	0.5097
+	LCE1B	1	152776971	152823109	101	11	10164	-0.024364	0.50972
+	ALDH1A3	15	100844692	100926626	311	28	10164	-0.024486	0.50977
-	KLLN	10	87849161	87898437	147	10	10164	-0.02466	0.50984
+	SOWAHC	2	109579334	109628987	137	19	10164	-0.024706	0.50986
-	TAF6	7	100097070	100154858	104	23	10164	-0.024917	0.50994
-	COBLL1	2	164670185	164877168	647	41	10164	-0.024995	0.50997
+	OR4C16	11	55537128	55583060	312	13	10164	-0.024985	0.50997
+	PCCA	13	100054015	100540437	1048	55	10164	-0.025313	0.5101
+	LVRN	5	115919903	116037606	595	44	10164	-0.025339	0.51011
-	LEF1	4	108037545	108203956	305	22	10164	-0.025864	0.51032
+	ELANE	19	815997	866250	36	5	10164	-0.025932	0.51034
+	KLHDC3	6	42979103	43031298	118	11	10164	-0.025956	0.51035
+	TMEM208	16	67192108	67239279	80	12	10164	-0.026086	0.51041
-	ACVR1C	2	157516767	157663887	92	11	10164	-0.026328	0.5105
+	AGBL3	7	134951508	135145778	533	25	10164	-0.026361	0.51052
-	NRDE2	14	90268054	90367137	8	2	10164	-0.026394	0.51053
+	CST6	11	65976991	66023505	190	22	10164	-0.027157	0.51083
+	SPATA5	4	122888070	123329450	1303	28	10164	-0.027538	0.51098
+	STAP1	4	67523728	67617337	223	19	10164	-0.027595	0.51101
+	TSPAN33	7	129109716	129179694	205	30	10164	-0.02763	0.51102
+	RIOK3	18	23417823	23493140	160	18	10164	-0.028318	0.5113

STRAND	SYMBOL	CHR	START	STOP	NSNPS	NPARAM	N	ZSTAT	P
+	RASL11B	4	52827328	52876835	144	14	10164	-0.028338	0.5113
+	KCNJ3	2	154663581	154868352	585	59	10164	-0.028373	0.51132
+	EVA1C	21	32376692	32525400	561	53	10164	-0.02846	0.51135
-	RASGEF1B	4	81416393	81506928	121	11	10164	-0.028449	0.51135
-	RDH16	12	56941431	56994374	130	12	10164	-0.028477	0.51136
+	CPNE7	16	89540768	89607246	296	39	10164	-0.028493	0.51137
-	CPEB1	15	82533201	82683016	305	23	10164	-0.028504	0.51137
-	CTSS	1	150720196	150800957	122	12	10164	-0.028538	0.51138
-	PARP6	15	72231181	72307583	139	13	10164	-0.028554	0.51139
-	C2orf76	2	119290442	119401826	296	15	10164	-0.028703	0.51145
+	C6orf203	6	106993172	107061586	185	18	10164	-0.02885	0.51151
-	FRMD5	15	43860761	44230577	774	40	10164	-0.028981	0.51156
+	MARS2	2	197670304	197718390	52	6	10164	-0.029002	0.51157
-	ZNF653	19	11473427	11540923	150	11	10164	-0.028995	0.51157
-	TMEM87A	15	42200452	42309526	336	12	10164	-0.029152	0.51163
-	LOC1053718	17	76943181	77004199	117	14	10164	-0.029291	0.51168
+	LOC1053715	17	2673663	2723146	66	12	10164	-0.029794	0.51188
+	CCDC91	12	28153259	28560166	1113	26	10164	-0.030001	0.51197
+	SRP72	4	56432596	56513681	256	20	10164	-0.030072	0.512
-	KLRC1	12	10432264	10489685	144	9	10164	-0.03027	0.51207
-	RAB11FIP5	2	73063382	73148018	179	13	10164	-0.030493	0.51216
+	HECW1	7	43077599	43573654	1476	127	10164	-0.030538	0.51218
+	TRIM55	8	66077667	66185489	145	11	10164	-0.030605	0.51221
+	CASP14	19	15014480	15068293	216	25	10164	-0.031307	0.51249
-	CDK2AP2	11	67496490	67543728	88	19	10164	-0.031366	0.51251
-	NUMBL	19	40655905	40726675	123	13	10164	-0.031393	0.51252
+	PNLIPRP2	10	116585953	116655143	180	18	10164	-0.031462	0.51255
+	MICU3	8	16992238	17132639	417	36	10164	-0.031465	0.51255
-	SIX4	14	60699538	60759321	106	10	10164	-0.031521	0.51257
-	OR9G4	11	56732828	56778811	159	23	10164	-0.031754	0.51267
+	LIPM	10	88767730	88832022	246	27	10164	-0.031899	0.51272
+	PHYHIPL	10	59138371	59257774	10	5	10164	-0.03195	0.51274
-	HRNR	1	152202076	152259196	209	12	10164	-0.032036	0.51278
+	XRCC4	5	83042409	83363760	1103	39	10164	-0.032253	0.51286
+	MAPRE2	18	34941928	35153470	433	42	10164	-0.032252	0.51286
+	BDKRB2	14	96169798	96254329	327	55	10164	-0.032399	0.51292
+	HTN3	4	69993413	70046538	148	17	10164	-0.032548	0.51298
+	DLGAP4	20	36271323	36538637	271	50	10164	-0.032554	0.51298
-	CNTD2	19	40212208	40262095	73	13	10164	-0.032627	0.51301
-	UPF2	10	11910022	12078172	527	28	10164	-0.032679	0.51303
-	SPICE1	3	113432718	113550187	418	18	10164	-0.032671	0.51303
-	RAG2	11	36581943	36633279	193	12	10164	-0.032734	0.51306
+	WNT9B	17	46798201	46896775	190	25	10164	-0.032969	0.51315
-	MRO	18	50785120	50860421	306	41	10164	-0.033043	0.51318
-	KRTAP13-3	21	30415393	30460912	169	11	10164	-0.033131	0.51321
+	SMG7	1	183437371	183564193	227	30	10164	-0.033215	0.51325
-	MCHR2	6	99909910	100029238	185	20	10164	-0.033594	0.5134
+	THEGL	4	56495575	56651550	481	56	10164	-0.033664	0.51343
-	SYT5	19	55163096	55215441	193	21	10164	-0.033797	0.51348
+	CNTLN	9	17099990	17513923	1581	41	10164	-0.033857	0.5135
-	DNA2	10	68404064	68507516	307	12	10164	-0.033898	0.51352
-	OVOL2	20	18014152	18094188	254	40	10164	-0.033966	0.51355
+	PFDN4	20	54172963	54229953	212	29	10164	-0.034114	0.51361
-	LOC1053750	6	53054693	53100792	42	8	10164	-0.034242	0.51366
-	TRIM49D1	11	89901409	89955388	23	6	10164	-0.034294	0.51368
+	TRIM49D2	11	89889064	89943063	23	6	10164	-0.034294	0.51368
+	FOXD4L3	9	68267867	68315084	1	1	10164	-0.034442	0.51374
+	CBWD3	9	68206866	68310035	1	1	10164	-0.034442	0.51374
+	LOC1019284	7	128496355	128650002	545	21	10164	-0.034493	0.51376
-	ASH1L	1	155325261	155597850	452	19	10164	-0.034532	0.51377
-	TOM1L2	17	17833508	18007470	473	25	10164	-0.034521	0.51377
+	HSPE1-MOB	2	197464997	197563699	95	8	10164	-0.034613	0.51381
-	H2AFZ	4	99938087	99985355	48	7	10164	-0.034811	0.51388

STRAND	SYMBOL	CHR	START	STOP	NSNPS	NPARAM	N	ZSTAT	P
+	NEK6	9	124222606	124388316	415	31	10164	-0.034943	0.51394
+	MCU	10	72657131	72897694	261	25	10164	-0.035045	0.51398
-	LOC1053777	5	177996273	178042707	31	7	10164	-0.035038	0.51398
+	UBFD1	16	23522541	23584394	174	14	10164	-0.035133	0.51401
-	NKPD1	19	45139111	45197841	134	23	10164	-0.035251	0.51406
+	DISP2	15	40319359	40381055	177	37	10164	-0.03541	0.51412
-	CCDC179	11	22836922	22895486	208	16	10164	-0.035534	0.51417
+	GYPC	2	126620935	126706675	222	34	10164	-0.035548	0.51418
-	TEX2	17	64137433	64298323	322	16	10164	-0.035866	0.51431
-	ABCA9	17	68964632	69102443	213	23	10164	-0.035922	0.51433
+	ZCCHC2	18	62488410	62597709	222	24	10164	-0.03627	0.51447
-	RFX2	19	5983164	6145653	247	9	10164	-0.036451	0.51454
+	DBNL	7	44009640	44071716	151	13	10164	-0.036558	0.51458
+	OR51E1	11	4608926	4665488	201	26	10164	-0.03656	0.51458
-	LOC1053768	18	498396	566912	8	2	10164	-0.036561	0.51458
-	ERO1LB	1	236205122	236317039	471	16	10164	-0.036616	0.5146
+	LY86	6	6553701	6664983	481	64	10164	-0.036751	0.51466
-	CNOT6L	4	77703387	77855238	207	12	10164	-0.037065	0.51478
+	MYNN	3	169734649	169799716	143	21	10164	-0.037088	0.51479
-	TBC1D3J	17	36439777	36491035	17	4	10164	-0.037569	0.51498
-	IRF1	5	132471609	132525777	146	14	10164	-0.037612	0.515
-	CLEC18B	16	74398631	74457248	85	9	10164	-0.037847	0.5151
+	MAS1	6	159871942	159918076	120	24	10164	-0.037897	0.51511
+	PSMC1	14	90221550	90282622	6	3	10164	-0.037951	0.51514
+	SLC4A8	12	51356317	51525763	397	34	10164	-0.038178	0.51523
-	CASP9	1	15481401	15561529	291	16	10164	-0.038328	0.51529
-	VSIG10	12	118053593	118139005	216	34	10164	-0.038378	0.51531
+	COMMD3	10	22281383	22330317	23	12	10164	-0.038443	0.51533
-	KLRC2	12	10420599	10470993	100	9	10164	-0.038616	0.5154
+	FHAD1	1	15201602	15410283	512	56	10164	-0.038651	0.51542
+	CFAP61	20	20017514	20370714	955	47	10164	-0.038724	0.51544
-	ARHGEF19	1	16187856	16247671	156	12	10164	-0.038915	0.51552
+	REXO2	11	114404386	114460279	124	12	10164	-0.039012	0.51556
+	TMEM62	15	43098144	43195147	137	18	10164	-0.039177	0.51563
-	ICAM2	17	63992595	64055634	36	10	10164	-0.039214	0.51564
-	GNA13	17	64999289	65091802	62	8	10164	-0.040066	0.51598
-	LOC1053759	9	11600222	11653749	194	15	10164	-0.040177	0.51602
-	OR13C3	9	104525770	104571813	120	20	10164	-0.040194	0.51603
-	IQCD	12	113185441	113256094	100	4	10164	-0.040328	0.51608
+	RRP36	6	42986636	43039599	116	10	10164	-0.040804	0.51627
-	LOC1053776	4	185560102	185615930	94	15	10164	-0.040815	0.51628
-	AHNAK2	14	104927253	105013357	328	31	10164	-0.040836	0.51629
+	RBAK-RBAKI	7	5010821	5083223	325	21	10164	-0.041214	0.51644
-	EPB41L3	18	5382381	5665666	780	91	10164	-0.041297	0.51647
+	LGALS9C	17	18441785	18504945	15	4	10164	-0.041305	0.51647
-	USP24	1	55056359	55250368	278	24	10164	-0.041821	0.51668
+	FOXL2NB	3	138912234	138963990	128	15	10164	-0.042243	0.51685
+	ATXN10	22	45636798	45855307	401	22	10164	-0.042411	0.51691
-	KIF16B	20	16262097	16608434	1307	75	10164	-0.042573	0.51698
+	ABHD14A	3	51940026	51991200	72	12	10164	-0.042594	0.51699
-	STX2	12	130764883	130874274	328	22	10164	-0.042639	0.51701
+	HMOX1	22	35346067	35404214	167	24	10164	-0.042739	0.51705
-	ISCA1	9	86254546	86317575	121	9	10164	-0.042909	0.51711
+	PSEN1	14	73101435	73233691	330	28	10164	-0.042975	0.51714
-	KNCN	1	46535638	46586657	136	21	10164	-0.0432	0.51723
-	SLC33A1	3	155816511	155889459	242	23	10164	-0.043394	0.51731
-	KCNJ8	12	21754955	21810502	27	7	10164	-0.044026	0.51756
-	LPAR3	1	84801602	84928213	343	43	10164	-0.044085	0.51758
-	MCC	5	113012099	113523830	2066	61	10164	-0.044298	0.51767
+	MX1	21	41385329	41469214	343	30	10164	-0.044296	0.51767
+	HNRNPA1L2	13	52582470	52653784	225	7	10164	-0.044416	0.51771
-	FAM90A1	12	8211260	8262703	10	2	10164	-0.044596	0.51779
+	DYNC1I1	7	95737506	96120322	1130	105	10164	-0.044837	0.51788

STRAND	SYMBOL	CHR	START	STOP	NSNPS	NPARAM	N	ZSTAT	P
+	OR6V1	7	143017341	143063282	39	1	10164	-0.044992	0.51794
+	XRCC6	22	41586163	41674048	205	17	10164	-0.045409	0.51811
+	FAM114A1	4	38832733	38955744	566	41	10164	-0.045902	0.51831
+	NPAS3	14	32899909	33814176	2239	244	10164	-0.046394	0.5185
+	HDGFL1	6	22534449	22580521	181	26	10164	-0.046706	0.51863
-	CCDC74B	2	130129283	130180134	67	3	10164	-0.04686	0.51869
+	SRRM1	1	24608012	24683281	110	15	10164	-0.046898	0.5187
-	CHRNE	17	4887769	4938128	192	28	10164	-0.047213	0.51883
+	USP43	17	9608493	9739687	117	15	10164	-0.047492	0.51894
-	SLC37A4	11	119014351	119065906	167	26	10164	-0.04759	0.51898
+	TBC1D1	4	37856084	38149175	1014	91	10164	-0.047665	0.51901
-	NOS1	12	117198142	117396802	588	63	10164	-0.047683	0.51902
+	ATMIN	16	81000853	81057346	213	15	10164	-0.047803	0.51906
-	DDO	6	110382180	110450550	192	27	10164	-0.047827	0.51907
+	OR1M1	19	9058245	9104186	121	10	10164	-0.047834	0.51908
-	BAD	11	64259828	64319704	175	30	10164	-0.048105	0.51918
-	NMNAT3	3	139550180	139713043	317	33	10164	-0.048362	0.51929
+	COL6A2	21	46062606	46142849	402	47	10164	-0.048392	0.5193
-	INPP5B	1	37850697	37982084	43	8	10164	-0.048425	0.51931
+	RNF220	1	44369783	44661724	532	74	10164	-0.048411	0.51931
+	CTDSP1	2	218363338	218415941	119	21	10164	-0.04842	0.51931
-	CCDC78	16	712582	761881	127	20	10164	-0.048439	0.51932
-	DNAL4	22	38768508	38829156	119	17	10164	-0.049047	0.51956
+	LOC1053727	20	49573935	49643617	186	24	10164	-0.049159	0.5196
-	MRPL55	1	228096679	228145953	167	25	10164	-0.04942	0.51971
+	GP1BA	17	4897275	4945030	168	25	10164	-0.049532	0.51975
-	C7orf49	7	135155780	135205826	172	13	10164	-0.049548	0.51976
-	CENPBD1	16	89959775	90007832	202	23	10164	-0.049891	0.5199
-	MANSC1	12	12319284	12385235	256	32	10164	-0.050044	0.51996
-	NR4A2	2	156314432	156369683	75	16	10164	-0.05007	0.51997
-	SPTSSB	3	161334792	161407938	279	32	10164	-0.050111	0.51998
-	RGS17	6	153000823	153166254	595	31	10164	-0.050118	0.51999
-	TMEM150A	2	85588547	85637699	108	14	10164	-0.050404	0.5201
-	TOB2	22	41423488	41483230	121	12	10164	-0.05048	0.52013
+	LOC1053782	5	148203565	148254034	161	10	10164	-0.050599	0.52018
+	TPCN1	12	113186080	113308589	178	6	10164	-0.05064	0.52019
-	C1orf141	1	67082176	67176645	271	22	10164	-0.050705	0.52022
-	TMEM53	1	44643247	44709608	162	21	10164	-0.050874	0.52029
+	ZDHHC18	1	26791710	26865720	65	7	10164	-0.050972	0.52033
+	EBLN2	3	73026659	73073337	130	7	10164	-0.05124	0.52043
+	OR4L1	14	20025045	20070983	62	7	10164	-0.051255	0.52044
-	RHCE	1	25350659	25465192	102	2	10164	-0.051285	0.52045
+	ZNF575	19	43496805	43546138	162	23	10164	-0.051415	0.5205
+	PTH1R	3	46842717	46913799	161	33	10164	-0.051667	0.5206
-	TTC39A	1	51277258	51380116	113	25	10164	-0.051741	0.52063
-	SRPK2	7	105103601	105434366	975	17	10164	-0.052306	0.52086
-	LYRM2	6	89622224	89673755	101	21	10164	-0.052362	0.52088
+	PAPPA2	1	176428171	176855601	599	35	10164	-0.052726	0.52103
-	PITPNA	17	1507989	1597816	224	24	10164	-0.052818	0.52106
-	MIP	12	56439502	56491553	74	13	10164	-0.052846	0.52107
+	E2F4	16	67157165	67208918	75	13	10164	-0.052903	0.5211
+	CMTM2	16	66544448	66598275	123	7	10164	-0.053144	0.52119
+	MYBPC1	12	101559931	101695883	578	53	10164	-0.053228	0.52122
-	BAZ1B	7	73430398	73557298	10	5	10164	-0.053328	0.52126
-	LOC1053743	3	160022259	160075364	249	23	10164	-0.053438	0.52131
+	KHDC3L	6	73327211	73374175	144	25	10164	-0.053584	0.52137
+	RPS6KA4	11	64324153	64382215	202	29	10164	-0.053818	0.52146
-	NUP210	3	13306230	13455319	253	11	10164	-0.055092	0.52197
-	OR2T29	1	248548544	248594491	1	1	10164	-0.055369	0.52208
-	CENPV	17	16332534	16388498	79	9	10164	-0.055525	0.52214
+	TGFBR3L	19	7880455	7929097	132	26	10164	-0.055515	0.52214
-	TP53AIP1	11	128924732	128978399	170	35	10164	-0.055571	0.52216
+	PPDPF	20	63485078	63532171	259	34	10164	-0.055663	0.5222

STRAND	SYMBOL	CHR	START	STOP	NSNPS	NPARAM	N	ZSTAT	P
-	LOC1053777	8	641299	697434	243	28	10164	-0.055706	0.52221
-	BCAS1	20	53933540	54105765	756	65	10164	-0.055756	0.52223
+	FAM170A	5	119594559	119645822	231	15	10164	-0.055741	0.52223
+	OR52I2	11	4551791	4597905	186	27	10164	-0.056184	0.5224
-	ACVR1	2	157726444	157910862	140	22	10164	-0.056265	0.52243
+	DOCK5	8	25149772	25425716	597	50	10164	-0.056323	0.52246
+	EFCAB8	20	32825995	32971845	351	31	10164	-0.056497	0.52253
-	PXDNL	8	51309575	51846029	1712	75	10164	-0.05667	0.5226
-	OR13C4	9	104516253	104562209	144	21	10164	-0.056758	0.52263
+	CPD	17	30343910	30479657	193	12	10164	-0.056788	0.52264
-	OSM	22	30252826	30301840	129	44	10164	-0.056769	0.52264
+	PAQR4	16	2934245	2983489	153	15	10164	-0.056985	0.52272
-	TMEM189	20	50113737	50188798	226	20	10164	-0.057287	0.52284
+	TLN2	15	62355575	62854631	1130	126	10164	-0.057787	0.52304
-	DIRAS2	9	90599832	90678105	277	37	10164	-0.057812	0.52305
-	MATN1	1	30701277	30758585	115	16	10164	-0.05787	0.52307
-	HDHD3	9	113363416	113412009	134	29	10164	-0.0581	0.52317
-	GPR55	2	230897318	230996227	277	43	10164	-0.058249	0.52322
-	SRSF2	17	76724115	76772629	68	9	10164	-0.058431	0.5233
-	S100A12	1	153363708	153410599	88	2	10164	-0.059151	0.52358
-	C3orf18	3	50548025	50609002	99	13	10164	-0.059198	0.5236
-	C19orf26	19	1219948	1276763	85	14	10164	-0.059429	0.52369
+	PPP4R2	3	72961968	73075860	362	17	10164	-0.05943	0.5237
-	NTSR2	2	11648178	11705203	9	2	10164	-0.059533	0.52374
+	TRPC1	3	142689032	142817888	348	24	10164	-0.059709	0.52381
-	TBX10	11	67621303	67674560	204	29	10164	-0.059725	0.52381
-	NANOS2	19	45903214	45949778	144	21	10164	-0.059746	0.52382
-	STK40	1	36329619	36420927	272	27	10164	-0.060158	0.52399
+	MITF	3	69704435	69978337	777	44	10164	-0.060667	0.52419
+	ZNF429	19	21470635	21548277	253	11	10164	-0.060986	0.52431
-	NEDD4	15	55816917	56028746	817	27	10164	-0.061001	0.52432
-	BAZ2A	12	56585596	56671816	92	10	10164	-0.061088	0.52436
+	EPN2	17	19202377	19346715	115	4	10164	-0.061289	0.52444
+	CAPN15	16	492756	564636	93	16	10164	-0.061328	0.52445
-	OR1D5	17	3052669	3098607	34	4	10164	-0.061445	0.5245
+	RAB3C	5	58547127	58869394	1024	74	10164	-0.061497	0.52452
+	PDE3A	12	20333510	20697641	1150	111	10164	-0.061669	0.52459
+	ANKRD13A	12	109964169	110049432	148	13	10164	-0.061702	0.5246
-	C19orf70	19	5668419	5715900	127	15	10164	-0.061851	0.52466
-	PIK3R4	3	130668934	130781852	333	15	10164	-0.062001	0.52472
-	CERK	22	46674410	46773255	451	44	10164	-0.062027	0.52473
+	APOA1BP	1	156556766	156604299	165	18	10164	-0.06212	0.52477
-	DRD3	3	114117797	114234407	288	36	10164	-0.062304	0.52484
+	SLC25A3	12	98558625	98612000	176	31	10164	-0.062392	0.52487
+	TATDN2	3	10213493	10291222	283	36	10164	-0.062452	0.5249
-	CHRNB4	15	78613297	78690586	291	47	10164	-0.062547	0.52494
+	KCNAB1	3	156085548	156549138	1595	108	10164	-0.062696	0.525
-	OR12D3	6	29363423	29410291	351	31	10164	-0.062696	0.525
-	RPS27L	15	63143340	63192542	168	22	10164	-0.062733	0.52501
+	TRNAU1AP	1	28518017	28588545	82	6	10164	-0.062722	0.52501
-	MGC50722	9	136256538	136341901	16	7	10164	-0.062877	0.52507
-	GNPTAB	12	101735497	101865867	322	26	10164	-0.06335	0.52526
+	ALKBH7	19	6337433	6385250	77	1	10164	-0.063419	0.52528
+	CCDC18	1	93145330	93289037	312	14	10164	-0.06366	0.52538
-	ARL3	10	102663727	102749433	209	32	10164	-0.064103	0.52556
-	IGSF9B	11	133898302	133991963	248	37	10164	-0.064359	0.52566
-	NADK	1	1741232	1815514	12	2	10164	-0.06443	0.52569
-	KPNA3	13	49689307	49827921	483	38	10164	-0.064692	0.52579
+	FAM157A	3	198116974	198221810	7	3	10164	-0.065006	0.52592
+	DCST1	1	154998806	155060930	100	21	10164	-0.065198	0.52599
-	NUP98	11	3665010	3832792	245	22	10164	-0.065284	0.52603
+	GOSR1	17	30442408	30536814	104	8	10164	-0.065335	0.52605
-	CEACAM7	19	41663307	41723277	96	12	10164	-0.065538	0.52613

STRAND	SYMBOL	CHR	START	STOP	NSNPS	NPARAM	N	ZSTAT	P
+	PCK1	20	57526081	57576461	202	22	10164	-0.065783	0.52622
-	DDOST	1	20641767	20696544	194	36	10164	-0.065916	0.52628
+	LOC1019293	16	12535192	12584287	728	33	10164	-0.066015	0.52632
-	MEX3A	1	156062013	156116998	90	22	10164	-0.066327	0.52644
-	ICAM3	19	10323776	10374829	171	24	10164	-0.066467	0.5265
-	CFAP44	3	113276930	113480190	706	22	10164	-0.066536	0.52652
-	MGAT4C	12	85966235	86873904	2069	51	10164	-0.06663	0.52656
-	CACNA2D1	7	81936444	82478806	1976	121	10164	-0.066706	0.52659
+	MIF	22	23859378	23905222	235	32	10164	-0.06678	0.52662
+	SEC14L2	22	30361941	30435303	219	27	10164	-0.066767	0.52662
-	FAM181B	11	82722004	82768864	113	16	10164	-0.066791	0.52663
-	C8orf88	8	90948478	91020257	119	14	10164	-0.066789	0.52663
+	RALA	7	39587955	39718124	246	28	10164	-0.066897	0.52667
-	COLEC12	18	309355	535729	711	78	10164	-0.067144	0.52677
-	OCA2	15	27744873	28134340	980	56	10164	-0.06723	0.5268
+	SMIM8	6	87287588	87352328	256	17	10164	-0.067416	0.52687
+	DIP2B	12	50469985	50758667	531	25	10164	-0.06746	0.52689
-	FAM120AOS	9	93433332	93488592	165	24	10164	-0.067646	0.52697
+	LRR1	14	49563697	49624672	186	15	10164	-0.06772	0.527
+	COL5A1	9	134606786	134854843	786	63	10164	-0.067971	0.5271
+	ZNF623	8	143601013	143663775	149	30	10164	-0.068527	0.52732
-	SLC7A7	14	22763222	22854811	361	49	10164	-0.068707	0.52739
-	ASCC3	6	100498194	100916372	1150	26	10164	-0.068727	0.5274
+	DHTKD1	10	12033917	12133228	320	27	10164	-0.06889	0.52746
-	PTBP3	9	112207715	112368664	530	22	10164	-0.069258	0.52761
+	KLHL32	6	96863013	97152614	913	52	10164	-0.069288	0.52762
-	KRT14	17	41572279	41621895	102	3	10164	-0.069327	0.52764
-	FSBP	8	94417712	94471952	168	14	10164	-0.069403	0.52767
+	FRAS1	4	78021968	78554269	1744	60	10164	-0.069566	0.52773
+	PIWIL1	12	130302887	130436260	455	55	10164	-0.069647	0.52776
-	LYZL2	10	30596232	30664762	279	31	10164	-0.069647	0.52776
+	CAT	11	34403925	34482060	347	13	10164	-0.069795	0.52782
-	CD8B	2	86805337	86896924	11	2	10164	-0.069782	0.52782
-	SNAPC5	15	66480328	66532808	138	18	10164	-0.070015	0.52791
+	HSBP1L1	18	79929582	79980822	125	11	10164	-0.07001	0.52791
+	F2	11	46684192	46749506	115	15	10164	-0.070449	0.52808
-	TAGAP	6	159024468	159080152	150	12	10164	-0.070522	0.52811
-	BCAR1	16	75219030	75303053	238	24	10164	-0.070533	0.52812
-	FAM167A	8	11411464	11510915	353	25	10164	-0.070604	0.52814
-	ANXA7	10	73365431	73449083	112	5	10164	-0.07088	0.52825
+	BBS9	7	33094244	33645769	1641	53	10164	-0.070878	0.52825
+	ABCA7	19	1005103	1075572	338	28	10164	-0.070926	0.52827
-	OPN3	1	241583150	241675399	188	42	10164	-0.070935	0.52828
+	SPEN	1	15812864	15950455	325	21	10164	-0.071016	0.52831
-	FBXL16	16	682500	740825	161	21	10164	-0.071075	0.52833
+	OR5K1	3	98434577	98480528	130	19	10164	-0.071193	0.52838
-	LOC1053792	5	132485146	132532253	116	15	10164	-0.071356	0.52844
-	GAS7	17	9900609	10233551	900	104	10164	-0.071371	0.52845
+	SLC35A1	6	87437925	87522339	245	19	10164	-0.071483	0.52849
-	TRA2A	7	23494780	23567041	161	22	10164	-0.071617	0.52855
+	OLIG1	21	33035144	33082422	116	17	10164	-0.071628	0.52855
-	CLCN2	3	184336185	184396651	123	31	10164	-0.07177	0.52861
+	C19orf40	19	32937217	32987495	212	28	10164	-0.07177	0.52861
-	PTCHD4	6	47867421	48146132	818	51	10164	-0.071842	0.52864
+	PTGDR	14	52232256	52286724	131	16	10164	-0.072001	0.5287
+	BNIP1	5	173109442	173174387	101	8	10164	-0.072281	0.52881
-	METTL18	1	169782529	169829959	195	23	10164	-0.072308	0.52882
-	TPRKB	2	73719830	73775104	24	3	10164	-0.072331	0.52883
+	LACE1	6	108259894	108535548	362	19	10164	-0.072474	0.52889
+	KIF1C	17	4962948	5038399	214	30	10164	-0.072593	0.52893
-	EARS2	16	23512013	23592375	235	16	10164	-0.072757	0.529
-	CCL23	17	36003056	36052975	123	25	10164	-0.07285	0.52904
+	FAM208B	10	5649772	5775779	567	54	10164	-0.072866	0.52904

STRAND	SYMBOL	CHR	START	STOP	NSNPS	NPARAM	N	ZSTAT	P
-	SLAMF6	1	160475030	160558262	215	19	10164	-0.072925	0.52907
-	CDK4	12	57737727	57787447	92	13	10164	-0.073103	0.52914
-	EPHA7	6	93230020	93454584	506	53	10164	-0.073194	0.52917
-	SPATC1L	21	46151148	46220149	336	33	10164	-0.073216	0.52918
+	SHMT2	12	57194573	57244935	110	19	10164	-0.073326	0.52923
+	OR5AC2	3	98052173	98098102	323	14	10164	-0.073351	0.52924
-	UTS2B	3	191257155	191365536	452	33	10164	-0.073441	0.52927
+	PIGV	1	26752963	26808403	47	5	10164	-0.073707	0.52938
+	ATP11AUN	13	112612044	112694497	149	20	10164	-0.073781	0.52941
-	BOLA2B	16	30182935	30229306	2	1	10164	-0.073824	0.52942
-	MED26	19	16564907	16663204	124	15	10164	-0.074193	0.52957
+	SLC10A4	4	48448343	48499524	140	17	10164	-0.074383	0.52965
+	DCAF6	1	167901559	168085843	92	10	10164	-0.074636	0.52975
+	G6PC	17	42865797	42924433	108	19	10164	-0.074824	0.52982
-	PITPNB	22	27841669	27954306	297	28	10164	-0.074854	0.52983
+	PINK1	1	20598455	20661511	298	23	10164	-0.074864	0.52984
-	PTX4	16	1475939	1523467	218	34	10164	-0.075062	0.52992
-	BTG3	21	17583650	17647950	157	13	10164	-0.075423	0.53006
-	MAP3K12	12	53469669	53535136	104	23	10164	-0.075447	0.53007
+	CCDC64	12	119954799	120104495	326	19	10164	-0.075591	0.53013
-	LMX1A	1	165191867	165391715	558	67	10164	-0.075762	0.5302
-	KLRC4	12	10397384	10444757	61	7	10164	-0.075933	0.53026
+	ZC3H11A	1	203760537	203864128	389	25	10164	-0.075999	0.53029
-	RPGRIP1L	16	53589906	53738859	340	27	10164	-0.076049	0.53031
-	ALDH6A1	14	74047662	74119493	264	20	10164	-0.076069	0.53032
+	LOC1027242	6	167148455	167206978	159	19	10164	-0.076147	0.53035
-	LCE3E	1	152555654	152601770	66	14	10164	-0.076366	0.53044
+	KLHL30	2	238103722	238162906	241	41	10164	-0.076553	0.53051
+	C9orf85	9	71876321	71983459	58	9	10164	-0.076705	0.53057
-	DBP	19	48620560	48672550	86	14	10164	-0.076754	0.53059
-	NKX2-8	14	36570011	36617581	149	13	10164	-0.07688	0.53064
-	P3H1	1	42736335	42802084	122	16	10164	-0.076986	0.53068
-	RPS17	15	82526750	82575544	112	20	10164	-0.076997	0.53069
+	ATP8A2	13	25337011	26035851	1530	76	10164	-0.07712	0.53074
-	E2F8	11	19214063	19276655	175	24	10164	-0.07715	0.53075
+	CEP135	4	55913808	56043363	237	27	10164	-0.077212	0.53077
-	EMC1	1	19205664	19286559	283	16	10164	-0.077338	0.53082
+	UGT2B7	4	69016249	69122987	517	14	10164	-0.077402	0.53085
+	GALM	2	38630910	38744767	401	36	10164	-0.077522	0.5309
+	C10orf32	10	102819210	102874961	188	28	10164	-0.077967	0.53107
-	NDUFAB1	16	23571014	23631341	181	13	10164	-0.078293	0.5312
-	SCGB2B2	19	34566740	34710731	568	33	10164	-0.078323	0.53121
-	HACD1	10	17579959	17652390	275	28	10164	-0.07835	0.53123
+	USP10	16	84664949	84789922	488	43	10164	-0.078436	0.53126
-	RPS29	14	49566672	49621416	162	14	10164	-0.078551	0.53131
+	NOS1AP	1	162034791	162380023	1067	63	10164	-0.078692	0.53136
-	URB1	21	32301019	32428003	406	36	10164	-0.078836	0.53142
-	TIAF1	17	29063521	29110609	73	6	10164	-0.078875	0.53143
+	ZNF729	19	22251450	22327176	193	28	10164	-0.078964	0.53147
+	ETV2	19	35606213	35654871	101	16	10164	-0.079086	0.53152
+	EPN3	17	50497687	50553750	150	17	10164	-0.079159	0.53155
+	PHF1	6	33375412	33426453	207	45	10164	-0.079327	0.53161
+	ZIC1	3	147374394	147426719	91	16	10164	-0.079453	0.53166
+	ARFGEF3	6	138126916	138354663	614	38	10164	-0.079505	0.53168
-	FAM3C	7	121338245	121431369	185	14	10164	-0.079573	0.53171
+	DDX47	12	12778346	12839981	191	28	10164	-0.0796	0.53172
-	PSD3	8	18517303	19121921	3057	187	10164	-0.079712	0.53177
+	FAM160B1	10	114786744	114909827	347	18	10164	-0.079777	0.53179
+	FBXO28	1	224079087	224172047	461	19	10164	-0.079809	0.53181
-	LOC1053784	10	110137955	110202921	175	28	10164	-0.08	0.53188
-	AIFM2	10	70102267	70167934	224	32	10164	-0.080255	0.53198
-	OTP	5	77618712	77673697	125	22	10164	-0.080466	0.53207
-	FGF20	8	16982825	17037165	202	28	10164	-0.080546	0.5321

STRAND	SYMBOL	CHR	START	STOP	NSNPS	NPARAM	N	ZSTAT	P
-	PYCRL	8	143593913	143644614	134	26	10164	-0.08093	0.53225
-	GTF2E2	8	30568514	30693241	264	31	10164	-0.081194	0.53236
+	SYT12	11	66971773	67060863	151	36	10164	-0.081338	0.53241
-	FAM83E	19	48590600	48649841	132	18	10164	-0.081592	0.53251
-	BMP7	20	57158753	57301651	344	26	10164	-0.081761	0.53258
-	OR2T35	1	248628287	248677735	39	3	10164	-0.081761	0.53258
-	KIFC3	16	57748217	57898053	478	47	10164	-0.081785	0.53259
-	THYN1	11	134238278	134288401	95	14	10164	-0.081772	0.53259
+	FDPS	1	155273748	155330666	103	13	10164	-0.081911	0.53264
-	FCRL2	1	157735733	157812439	137	19	10164	-0.082198	0.53276
-	GXYLT1	12	42071845	42179871	293	22	10164	-0.082253	0.53278
-	TRPV5	7	142898101	142968723	100	4	10164	-0.082374	0.53283
+	IGFBP6	12	53062652	53112344	112	15	10164	-0.082613	0.53292
+	WDTC1	1	27199516	27318633	140	14	10164	-0.082692	0.53295
+	MTA3	2	42459569	42805030	1176	51	10164	-0.082877	0.53303
-	C3orf22	3	126511997	126593965	200	21	10164	-0.082933	0.53305
+	ARID1B	6	156742892	157220779	512	38	10164	-0.083218	0.53316
+	CALML3	10	5489889	5536268	207	55	10164	-0.083287	0.53319
-	DGKI	7	137377654	137881863	870	57	10164	-0.083299	0.53319
+	ACER2	9	19371011	19462505	161	13	10164	-0.083908	0.53344
+	WDR7	18	56616343	57039805	979	35	10164	-0.084161	0.53354
+	OR12D2	6	29361639	29407671	332	30	10164	-0.084523	0.53368
+	PELI2	14	56083323	56311581	822	31	10164	-0.084621	0.53372
-	ZFP57	6	29662392	29716146	749	30	10164	-0.084666	0.53374
+	SLC35D3	6	136887241	136935660	80	8	10164	-0.084733	0.53376
+	LPIN3	20	41305780	41370585	200	33	10164	-0.084746	0.53377
+	SPACA3	17	32956864	33007874	33	11	10164	-0.084877	0.53382
+	VARS2	6	30879208	30936459	326	25	10164	-0.084979	0.53386
-	YIPF1	1	53839790	53924820	255	22	10164	-0.085179	0.53394
+	LEUTX	19	39741594	39796135	162	12	10164	-0.08518	0.53394
-	LOC401052	3	9996418	10046095	227	18	10164	-0.08518	0.53394
+	RARS	5	168451458	168529306	352	25	10164	-0.085624	0.53412
-	TRIM10	6	30141939	30198440	433	36	10164	-0.08604	0.53428
+	CCNDBP1	15	43150118	43207177	79	12	10164	-0.086021	0.53428
+	ABCC2	10	99747598	99862576	193	14	10164	-0.086433	0.53444
+	ZNF609	15	64425743	64696068	332	13	10164	-0.086535	0.53448
-	DOK2	8	21898873	21948743	120	17	10164	-0.086831	0.5346
+	DCBLD1	6	117418819	117579858	659	45	10164	-0.086917	0.53463
-	XRRA1	11	74830910	74984187	204	17	10164	-0.086957	0.53465
+	WDR5	9	134100679	134169972	22	5	10164	-0.086996	0.53466
+	SPATA18	4	52016331	52107305	160	15	10164	-0.087125	0.53471
-	ZCRB1	12	42302086	42361130	214	16	10164	-0.087162	0.53473
+	MGME1	20	17933853	18001122	239	15	10164	-0.08723	0.53476
+	ZNF860	3	31946774	32001736	202	19	10164	-0.087251	0.53476
-	ARFGAP3	22	42786524	42892402	363	28	10164	-0.08728	0.53478
-	OCSTAMP	20	46531031	46585574	128	18	10164	-0.087353	0.5348
-	KCNG3	2	42432017	42529097	372	45	10164	-0.087539	0.53488
-	MB	22	35596764	35658354	127	15	10164	-0.087598	0.5349
+	OLFML1	11	7450369	7521897	320	40	10164	-0.087821	0.53499
+	SLC24A5	15	48085972	48152392	46	9	10164	-0.087929	0.53503
+	SGMS2	4	107789335	107925047	222	27	10164	-0.088102	0.5351
+	COL1A2	7	94359561	94441232	271	42	10164	-0.088118	0.53511
+	NT5DC4	2	112685810	112752879	151	19	10164	-0.088243	0.53516
-	OSCAR	19	54084668	54137686	72	8	10164	-0.088283	0.53517
+	UPP1	7	48053603	48118733	138	21	10164	-0.088365	0.53521
+	LOC1027238	7	90474145	90523397	142	17	10164	-0.088831	0.53539
-	SOSTDC1	7	16451481	16500849	205	20	10164	-0.089303	0.53558
-	TMEM55B	14	20447853	20496612	128	25	10164	-0.089328	0.53559
+	C7orf55	7	139305450	139356319	87	5	10164	-0.089704	0.53574
-	C19orf54	19	40730856	40785713	97	10	10164	-0.090083	0.53589
+	MCPH1	8	6371592	6653619	1790	86	10164	-0.090379	0.53601
+	GRIA1	5	153454696	153823873	1140	73	10164	-0.090493	0.53605
+	OVOL3	19	36073829	36123720	117	17	10164	-0.090479	0.53605

STRAND	SYMBOL	CHR	START	STOP	NSNPS	NPARAM	N	ZSTAT	P
+	TAS2R40	7	143187079	143233050	10	2	10164	-0.090624	0.5361
-	NKX2-5	5	173222104	173270312	55	10	10164	-0.091012	0.53626
+	OR52B6	11	5545877	5591884	299	17	10164	-0.091187	0.53633
+	ARHGEF17	11	73270939	73379380	196	13	10164	-0.091216	0.53634
+	S100P	4	6658839	6707170	106	15	10164	-0.091312	0.53638
-	OR7C1	19	14787568	14870376	245	37	10164	-0.091581	0.53648
-	SCLT1	4	128864418	129128607	908	24	10164	-0.091611	0.5365
+	MDH1	2	63553609	63617197	145	19	10164	-0.091771	0.53656
+	ANKRD20A4	9	64334563	64422691	5	2	10164	-0.091888	0.53661
-	SUMO3	21	44795617	44853129	203	27	10164	-0.091956	0.53663
+	RNF187	1	228452367	228506188	196	33	10164	-0.091994	0.53665
-	ITGB6	2	160089666	160235313	449	31	10164	-0.092115	0.5367
-	FRYL	4	48487363	48815312	537	38	10164	-0.092168	0.53672
-	MOB3C	1	46597715	46651891	179	22	10164	-0.09219	0.53673
+	FAH	15	80117891	80196582	296	34	10164	-0.092335	0.53678
-	C22orf24	22	31915316	31980362	218	40	10164	-0.092333	0.53678
+	ZNF440	19	11779284	11845617	14	4	10164	-0.092634	0.5369
+	FAM47E-STE	4	76216700	76321130	447	29	10164	-0.092843	0.53699
+	TMEM57	1	25395858	25510209	118	2	10164	-0.092909	0.53701
-	GIT2	12	109919802	110031389	200	16	10164	-0.093127	0.5371
-	NAA38	17	7846685	7920283	106	19	10164	-0.093309	0.53717
-	CDH10	5	24477100	24680024	520	26	10164	-0.093477	0.53724
+	CNNM1	10	99294099	99404330	319	27	10164	-0.093521	0.53726
-	HSPA13	21	14361115	14418188	216	24	10164	-0.093729	0.53734
-	WLS	1	68088459	68268000	652	75	10164	-0.093727	0.53734
-	RP9	7	33084797	33144390	214	23	10164	-0.09381	0.53737
+	KIAA0355	19	34219551	34365566	232	20	10164	-0.094026	0.53746
+	OXSM	3	25755072	25804534	76	8	10164	-0.094138	0.5375
+	PRMT8	12	3346349	3603973	365	57	10164	-0.094367	0.53759
-	UQCR11	19	1587155	1640484	62	9	10164	-0.094457	0.53763
-	TMEM237	2	201610184	201678529	205	48	10164	-0.094582	0.53768
+	C11orf53	11	111210704	111296406	277	40	10164	-0.094687	0.53772
+	P2RX6	22	20979451	21038013	193	26	10164	-0.094769	0.53775
-	GAS2L2	17	35734511	35788745	132	16	10164	-0.094782	0.53776
+	TEX40	11	64265391	64314767	146	24	10164	-0.095025	0.53785
+	RUNDC3A	17	44272918	44328670	138	23	10164	-0.095268	0.53795
-	SOX18	20	64037726	64084626	91	16	10164	-0.095487	0.53804
-	EPS8	12	15610141	15824576	337	22	10164	-0.095576	0.53807
+	ARHGEF38	4	105517522	105692539	408	46	10164	-0.095579	0.53807
-	CCBL1	9	128822942	128917513	180	17	10164	-0.095647	0.5381
-	C7orf50	7	968498	1173275	1002	32	10164	-0.095814	0.53817
-	AMPH	7	38373694	38666567	552	43	10164	-0.096094	0.53828
-	PHF12	17	28895247	28986605	86	15	10164	-0.096147	0.5383
+	TCTN1	12	110579027	110659130	77	16	10164	-0.096213	0.53832
+	RNF8	6	37318972	37404738	213	35	10164	-0.096836	0.53857
+	SEC14L1	17	77053643	77227101	525	52	10164	-0.097129	0.53869
+	SLC35A3	1	99933887	100036979	213	17	10164	-0.097227	0.53873
-	HOXB5	17	48581257	48628741	168	36	10164	-0.097294	0.53875
-	SMYD3	1	245739340	246542342	3316	103	10164	-0.097297	0.53875
-	LOC1053712	16	51116627	51170774	74	15	10164	-0.097286	0.53875
+	MLLT10	10	21498823	21753633	292	51	10164	-0.097466	0.53882
-	NMRK1	9	75050577	75123319	203	21	10164	-0.097793	0.53895
-	CXCL17	19	42418543	42477984	59	9	10164	-0.097823	0.53896
+	RIMS2	8	103465642	104264429	2450	51	10164	-0.097913	0.539
+	CFAP58	10	104303558	104465090	365	42	10164	-0.097993	0.53903
-	CHST10	2	100381860	100452668	298	22	10164	-0.098196	0.53911
-	KIAA0895L	16	67165602	67218980	84	13	10164	-0.098205	0.53912
-	STAT1	2	190959036	191049250	235	43	10164	-0.098316	0.53916
+	FAM173A	16	685581	732590	139	19	10164	-0.098428	0.5392
-	GUCA1C	3	108897644	108990188	336	25	10164	-0.098733	0.53933
-	NGEF	2	232868686	233048241	736	71	10164	-0.098832	0.53936
-	ZBTB46	20	63733668	63867344	544	22	10164	-0.098896	0.53939
+	NDUFA3	19	54067852	54116974	44	6	10164	-0.099034	0.53944

STRAND	SYMBOL	CHR	START	STOP	NSNPS	NPARAM	N	ZSTAT	P
-	TARM1	19	54059487	54116365	44	6	10164	-0.099034	0.53944
+	TIMM22	17	962117	1012167	148	21	10164	-0.099133	0.53948
-	C1orf61	1	156394256	156469325	109	30	10164	-0.099291	0.53955
+	LOC1005065	4	56373789	56439005	194	23	10164	-0.099328	0.53956
+	C2orf78	2	73749189	73827148	22	3	10164	-0.099379	0.53958
-	ZMYND12	1	42420329	42491267	186	12	10164	-0.099633	0.53968
+	LUZP2	11	24461488	25092640	1918	79	10164	-0.099664	0.53969
+	METTL21B	12	57737600	57792541	99	14	10164	-0.099672	0.5397
+	DLEC1	3	38003605	38132737	315	24	10164	-0.099781	0.53974
-	PPP1CC	12	110698970	110777978	140	11	10164	-0.09995	0.53981
+	INPP4A	2	98409858	98604067	369	17	10164	-0.10011	0.53987
-	FAM133B	7	92550758	92625394	149	25	10164	-0.1002	0.53991
-	FRAT2	10	97322497	97369701	128	14	10164	-0.10087	0.54017
-	AP1B1	22	29317680	29423583	260	35	10164	-0.10096	0.54021
+	FAR2	12	29114003	29345616	1082	56	10164	-0.10105	0.54024
-	GIGYF1	7	100669507	100729268	103	13	10164	-0.1011	0.54027
-	CKAP5	11	46733534	46881308	222	20	10164	-0.10115	0.54029
-	CLMP	11	123062312	123230299	645	56	10164	-0.10121	0.54031
+	GOLGA6L4	15	84200773	84255368	1	1	10164	-0.10126	0.54033
-	OXGR1	13	96975719	97030826	94	12	10164	-0.10139	0.54038
+	USP17L30	4	9328129	9374721	65	7	10164	-0.10139	0.54038
+	RGP1	9	35714280	35763267	71	25	10164	-0.10148	0.54042
-	CHERP	19	16507889	16577452	77	7	10164	-0.10152	0.54043
-	TCEB3B	18	47022572	47070617	163	26	10164	-0.10168	0.5405
+	C14orf178	14	77725830	77779742	202	19	10164	-0.10195	0.5406
+	ZNF189	9	101363808	101420660	154	24	10164	-0.10199	0.54062
-	CTSG	14	24563518	24611260	159	20	10164	-0.10235	0.54076
+	AK7	14	96357111	96499427	280	38	10164	-0.10234	0.54076
+	IRX1	5	3561054	3611403	122	21	10164	-0.10238	0.54077
-	TPRN	9	137181617	137235711	123	18	10164	-0.10243	0.54079
+	ING3	7	120915763	120985657	135	8	10164	-0.10251	0.54082
+	SCHIP1	3	159238247	159907366	1521	95	10164	-0.10272	0.54091
-	LHB	19	49005980	49052090	156	18	10164	-0.10289	0.54097
-	SIPA1L2	1	232387965	232665696	944	75	10164	-0.10294	0.541
-	GOLPH3L	1	150636225	150732956	223	10	10164	-0.1031	0.54106
+	SLAMF8	1	159791650	159847497	163	22	10164	-0.10323	0.54111
+	SULT1C4	2	108342957	108398989	160	11	10164	-0.10355	0.54124
-	ANO7P1	1	16205907	16262036	103	9	10164	-0.10372	0.54131
-	SDC4	20	45315285	45383424	199	31	10164	-0.10378	0.54133
-	PPP4R1	18	9536791	9650276	282	29	10164	-0.10401	0.54142
+	OR4N5	14	20108736	20154662	21	6	10164	-0.10406	0.54144
+	SAMD4B	19	39307459	39400760	36	9	10164	-0.10421	0.5415
+	CIDEA	18	12219319	12287595	198	39	10164	-0.10445	0.54159
-	TFB1M	6	155237238	155349497	364	27	10164	-0.10509	0.54185
+	SMOC1	14	69844388	70042366	548	53	10164	-0.10516	0.54188
-	EIF1AD	11	65986545	66037166	194	25	10164	-0.10522	0.5419
-	PRDM14	8	70041126	70106327	180	26	10164	-0.1053	0.54193
-	C10orf54	10	71737556	71808580	222	28	10164	-0.10534	0.54195
-	ABHD14B	3	51958510	52009630	74	11	10164	-0.10542	0.54198
+	PTEN	10	87828438	87981930	434	18	10164	-0.10575	0.54211
+	PPP1R26	9	135444699	135498894	149	32	10164	-0.1058	0.54213
+	ST6GAL1	3	186895343	187088553	759	93	10164	-0.10588	0.54216
+	LOC1053773	4	82434579	82490384	91	14	10164	-0.10597	0.5422
+	TRIB2	2	12681872	12752734	111	13	10164	-0.10622	0.5423
-	TNFRSF18	1	1193508	1241709	189	12	10164	-0.1063	0.54233
-	NFAM1	22	42370407	42472083	247	26	10164	-0.10644	0.54238
+	TRAPPC2L	16	88822098	88872678	278	27	10164	-0.10652	0.54242
-	CRYGS	3	186528443	186579378	87	6	10164	-0.10668	0.54248
-	LRP4	11	46846717	46953622	141	15	10164	-0.10685	0.54255
-	PAM16	16	4330251	4386372	5	3	10164	-0.10693	0.54258
+	PGAM5	12	132675807	132732737	142	15	10164	-0.10703	0.54262
+	SPATA31A7	9	61155036	61206280	2	1	10164	-0.10716	0.54267
-	C1orf111	1	162363725	162411925	199	31	10164	-0.10719	0.54268

STRAND	SYMBOL	CHR	START	STOP	NSNPS	NPARAM	N	ZSTAT	P
-	ZNF132	19	58422807	58475222	106	11	10164	-0.10725	0.5427
-	KIAA0930	22	45182242	45275769	475	46	10164	-0.10725	0.54271
+	RHOT1	17	32107454	32263374	24	10	10164	-0.10738	0.54276
+	DEFB127	20	122470	169163	151	11	10164	-0.10753	0.54281
-	LOC1053692	9	88026866	88074819	179	17	10164	-0.10769	0.54288
+	CA3	8	85403827	85459040	104	14	10164	-0.1079	0.54296
-	RLN2	9	5289864	5374412	271	32	10164	-0.10798	0.54299
-	IGFALS	16	1780413	1829908	469	26	10164	-0.10804	0.54302
-	DESI1	22	41588028	41656061	147	14	10164	-0.1084	0.54316
+	EEPD1	7	36118227	36311543	611	50	10164	-0.10841	0.54316
-	ANAPC2	9	137164784	137223605	120	18	10164	-0.10855	0.54322
+	USP47	11	11806423	11969325	493	35	10164	-0.10855	0.54322
-	RHPN2	19	32968592	33099918	521	44	10164	-0.10901	0.5434
-	AP3B2	15	82649281	82744908	186	31	10164	-0.10908	0.54343
-	CPLX4	18	59285402	59353649	163	24	10164	-0.10944	0.54357
+	ZNF471	19	56472843	56540212	227	22	10164	-0.10951	0.5436
+	RAB33B	4	139418232	139485916	144	9	10164	-0.10964	0.54365
+	DCK	4	70958548	71040914	122	15	10164	-0.10988	0.54375
-	OMA1	1	58470719	58581799	440	18	10164	-0.10991	0.54376
-	LOC1053734	2	5686237	5735950	132	17	10164	-0.1099	0.54376
+	GUCY2D	17	7967670	8030340	102	18	10164	-0.1101	0.54384
-	ABCC5	3	183909934	184053010	415	29	10164	-0.11027	0.5439
+	OR5H15	3	98133700	98179641	229	8	10164	-0.11031	0.54392
-	C1orf168	1	56708804	56854696	350	57	10164	-0.11049	0.54399
-	PNPT1	2	55624063	55728910	373	25	10164	-0.11052	0.544
+	SYT11	1	155824469	155895199	97	22	10164	-0.11064	0.54405
+	OR4C15	11	55519307	55565419	352	16	10164	-0.11075	0.54409
-	PIGC	1	172431457	172479090	59	4	10164	-0.11087	0.54414
+	RRP15	1	218250285	218347983	220	26	10164	-0.11089	0.54415
-	CDKN2AIPNL	5	134392065	134449603	80	12	10164	-0.11091	0.54415
+	MYBL2	20	43632019	43726496	218	11	10164	-0.11151	0.5444
-	USP7	16	8882094	8998484	289	30	10164	-0.11158	0.54442
+	SLC25A27	6	46617915	46688190	137	16	10164	-0.11185	0.54453
-	LOC1010600	5	134071431	134117822	152	23	10164	-0.11185	0.54453
-	GTF2A2	15	59628062	59692539	241	10	10164	-0.11208	0.54462
+	H2AFJ	12	14739336	14788002	194	27	10164	-0.11213	0.54464
+	RAB8B	15	63154529	63277776	317	28	10164	-0.11247	0.54477
+	BRINP2	1	177135922	177292422	257	27	10164	-0.11264	0.54484
+	HMCES	3	129243816	129316186	159	6	10164	-0.11268	0.54486
+	SF3B3	16	70488788	70587668	113	8	10164	-0.11299	0.54498
+	CLEC12B	12	9974047	10028800	231	25	10164	-0.113	0.54498
-	CIB4	2	26563764	26676411	258	32	10164	-0.11312	0.54503
-	HEY1	8	79754010	79802863	107	24	10164	-0.11356	0.54521
-	ZNF385D	3	21402222	22407641	4690	268	10164	-0.11357	0.54521
+	PKNOX2	11	125129663	125443389	823	89	10164	-0.11365	0.54524
-	GLB1L	2	219226598	219280429	100	8	10164	-0.11363	0.54524
-	C19orf67	19	14071601	14120795	25	6	10164	-0.11366	0.54525
-	C3orf33	3	155752612	155841287	287	39	10164	-0.11373	0.54527
+	MEI1	22	41664501	41809456	398	18	10164	-0.11395	0.54536
+	MCCC2	5	71552288	71668706	300	18	10164	-0.11396	0.54537
+	CENPQ	6	49428341	49503107	171	18	10164	-0.11443	0.54555
-	TYSND1	10	70127981	70181740	176	17	10164	-0.11452	0.54559
+	IZUMO4	19	2061598	2109587	108	29	10164	-0.11455	0.5456
+	PIK3C2G	12	18207965	18709660	1365	77	10164	-0.11461	0.54562
+	FNDC7	1	108677416	108753262	115	22	10164	-0.11465	0.54564
+	ACO2	22	41434125	41538989	175	21	10164	-0.11567	0.54604
+	INAFM2	15	40289037	40336715	140	33	10164	-0.11591	0.54614
+	PRDM11	11	45059013	45245124	454	54	10164	-0.11598	0.54617
+	SNX27	1	151576984	151709083	285	27	10164	-0.11647	0.54636
+	ZNF207	17	32315138	32418085	100	8	10164	-0.11697	0.54656
+	LEMD3	12	65134571	65258361	177	26	10164	-0.11726	0.54667
-	GRB2	17	75308076	75440709	342	19	10164	-0.11738	0.54672
+	SULT2B1	19	48517172	48609427	314	30	10164	-0.1174	0.54673

STRAND	SYMBOL	CHR	START	STOP	NSNPS	NPARAM	N	ZSTAT	P
+	VASH2	1	212900543	213001585	289	38	10164	-0.11744	0.54674
+	QSER1	11	32858178	32990270	133	21	10164	-0.11748	0.54676
-	PLA2G4F	15	42131134	42191641	177	28	10164	-0.11764	0.54682
-	OR1G1	17	3116505	3163253	137	14	10164	-0.11781	0.54689
+	NOD2	16	50658581	50743081	196	27	10164	-0.11803	0.54698
+	ATF7IP2	16	10351055	10493638	31	3	10164	-0.11818	0.54704
+	KCNK4	11	64256321	64310031	152	26	10164	-0.11822	0.54705
-	RBM34	1	235121183	235196616	64	3	10164	-0.11827	0.54707
-	PLCB2	15	40274260	40342973	184	40	10164	-0.11834	0.5471
-	RTKN2	10	62173035	62303863	331	13	10164	-0.11836	0.54711
+	STXBP5L	3	120873203	121434761	1731	34	10164	-0.11842	0.54713
-	ZNF229	19	44416251	44483725	277	28	10164	-0.11845	0.54715
+	PDLIM5	4	94416857	94678227	721	56	10164	-0.11846	0.54715
-	MEA1	6	42970344	43052087	199	19	10164	-0.11849	0.54716
+	CCIN	9	36134392	36181334	123	13	10164	-0.11857	0.54719
+	COPS6	7	100053960	100102200	85	15	10164	-0.1188	0.54728
+	ATP6V1G1	9	114552714	114608872	191	26	10164	-0.11885	0.5473
+	OPA1	3	193558144	193707811	539	24	10164	-0.11886	0.54731
-	URGCP	7	43865894	43961411	279	28	10164	-0.119	0.54736
+	ADAM7	8	24405996	24522760	257	19	10164	-0.11929	0.54748
-	FSIP1	15	39589529	39817838	734	37	10164	-0.11931	0.54749
-	OR2T27	1	248639931	248685884	75	3	10164	-0.11937	0.54751
-	OR14A16	1	247804800	247869858	189	14	10164	-0.11962	0.54761
-	RORC	1	151796071	151867238	179	32	10164	-0.11981	0.54768
+	ST3GAL3	1	43670824	43941165	566	25	10164	-0.12017	0.54783
+	R3HDML	20	44302158	44360792	151	24	10164	-0.1203	0.54788
-	C12orf66	12	64176312	64272341	124	11	10164	-0.12034	0.54789
+	LOC1005058	5	122094546	122192663	121	20	10164	-0.12044	0.54793
-	C10orf105	10	71701701	71772824	197	30	10164	-0.12056	0.54798
+	SLC2A11	22	23821703	23896112	388	44	10164	-0.1206	0.548
+	ZC3H12A	1	37438455	37494377	189	30	10164	-0.12066	0.54802
-	HRC	19	49141198	49190429	136	15	10164	-0.12074	0.54805
+	ENOPH1	4	82395480	82471175	156	18	10164	-0.1212	0.54823
-	SLC25A30	13	45383316	45453455	182	22	10164	-0.12171	0.54843
+	SSTR2	17	73130021	73181955	60	7	10164	-0.12178	0.54847
+	OR2D3	11	6886002	6931994	196	11	10164	-0.12206	0.54858
-	MEF2BNB	19	19166904	19227591	205	29	10164	-0.12253	0.54876
+	GBAS	7	55929577	56010182	252	29	10164	-0.12269	0.54882
+	C1orf167	1	11727183	11799585	198	15	10164	-0.12267	0.54882
+	KRTAP10-12	21	44662172	44708044	250	14	10164	-0.12271	0.54883
+	GALT	9	34611589	34660598	108	20	10164	-0.12288	0.5489
+	TLX1	10	101095780	101147789	155	24	10164	-0.12302	0.54895
-	THOC3	5	175928810	176003537	18	7	10164	-0.12301	0.54895
+	FBXL2	3	33242442	33413554	356	22	10164	-0.12312	0.54899
+	TSPAN10	17	81602171	81658755	196	7	10164	-0.12334	0.54908
+	FER1L6	8	123816987	124130061	680	48	10164	-0.1234	0.54911
-	ZXDC	3	126427601	126510933	268	26	10164	-0.12351	0.54915
-	UBP1	3	33378336	33475813	215	15	10164	-0.1238	0.54926
+	PCDHB15	5	141210349	141258234	118	12	10164	-0.12378	0.54926
+	CDH23	10	71361934	71825947	1471	137	10164	-0.12399	0.54934
-	KIAA0825	5	94140851	94653604	753	72	10164	-0.12414	0.5494
+	CXCR5	11	118848766	118906271	173	25	10164	-0.12422	0.54943
+	ROR1	1	63739019	64191498	728	74	10164	-0.12428	0.54945
+	WFIKKN2	17	50800244	50852348	143	13	10164	-0.12431	0.54946
-	SLC17A3	6	25835100	25909243	295	33	10164	-0.12444	0.54952
+	FMNL1	17	45186906	45257320	154	29	10164	-0.12456	0.54956
-	BLMH	17	30238195	30327166	130	12	10164	-0.12464	0.54959
+	NAGK	2	71033241	71088868	315	26	10164	-0.12486	0.54968
+	RNASEH2B	13	50874678	50980462	255	37	10164	-0.12498	0.54973
-	DAPK2	15	63897036	64081455	415	30	10164	-0.1251	0.54978
+	TMEM14A	6	52636086	52696588	227	27	10164	-0.12583	0.55007
+	IQCH	15	67219774	67516612	886	40	10164	-0.12584	0.55007
-	WDR31	9	113305265	113375340	259	25	10164	-0.12605	0.55015

STRAND	SYMBOL	CHR	START	STOP	NSNPS	NPARAM	N	ZSTAT	P
+	DLD	7	107856107	107931198	182	11	10164	-0.12622	0.55022
+	COG2	1	230607456	230703985	291	50	10164	-0.12658	0.55036
-	ZFC3H1	12	71599599	71698969	162	15	10164	-0.12664	0.55039
-	DAP	5	10669230	10796275	299	22	10164	-0.12674	0.55043
+	SLC22A1	6	160086789	160169198	292	63	10164	-0.12702	0.55054
+	AMTN	4	70483572	70542743	356	17	10164	-0.12722	0.55062
-	SRGAP3	3	8970591	9397929	1224	144	10164	-0.12725	0.55063
-	RAB6B	3	133814235	133930847	338	46	10164	-0.12738	0.55068
+	P2RY2	11	73181448	73252427	116	33	10164	-0.12754	0.55074
+	CDK5R1	17	32452087	32501254	51	7	10164	-0.12756	0.55075
-	SF3A3	1	37946975	38025490	23	7	10164	-0.12755	0.55075
-	LYPLA1	8	54036367	54137017	109	12	10164	-0.12822	0.55101
-	BCAR3	1	93551786	93882150	315	22	10164	-0.12824	0.55102
+	LOC1053789	1	1850960	1903010	16	2	10164	-0.12835	0.55106
+	PSD4	2	113122399	113213229	295	44	10164	-0.12835	0.55107
+	PDE4B	1	65757510	66384579	1727	109	10164	-0.12871	0.5512
+	TSPAN1	1	46140077	46206489	171	28	10164	-0.12869	0.5512
+	TYRO3	15	41522871	41589338	183	22	10164	-0.1288	0.55124
+	TRPM4	19	49122759	49221841	231	25	10164	-0.12879	0.55124
-	ERVFRD-1	6	11092489	11146838	220	22	10164	-0.12883	0.55125
-	MAP3K14	17	45253119	45352064	210	30	10164	-0.1289	0.55128
+	PPCS	1	42421341	42483381	164	12	10164	-0.12889	0.55128
-	PRRT3	3	9935542	9987394	160	13	10164	-0.12889	0.55128
-	GJB7	6	87272978	87364278	322	14	10164	-0.1291	0.55136
+	NIP7	16	69304512	69353111	124	20	10164	-0.12936	0.55146
-	ICMT	1	6211193	6270984	127	13	10164	-0.12951	0.55152
-	OR6M1	11	123795408	123841349	180	22	10164	-0.12961	0.55156
+	C8orf44	8	66632552	66691149	66	6	10164	-0.12975	0.55162
+	NIPAL3	1	24378674	24482983	299	19	10164	-0.13005	0.55173
-	SPG20	13	36291638	36405180	230	12	10164	-0.13008	0.55175
-	TAF2	8	119720773	119867858	473	24	10164	-0.13024	0.55181
-	TIFAB	5	135438868	135487399	6	4	10164	-0.13026	0.55182
-	ZNF202	11	123713927	123776683	244	25	10164	-0.13049	0.55191
-	PKD1L1	7	47764652	47999635	831	58	10164	-0.13072	0.552
-	ZNF677	19	53225385	53289858	185	20	10164	-0.13071	0.552
-	CALML5	10	5488695	5534570	210	56	10164	-0.13076	0.55202
-	ZNF497	19	58344357	58397848	95	8	10164	-0.13082	0.55204
-	HTR4	5	148441032	148689527	695	49	10164	-0.13118	0.55218
-	ZNF670	1	247024638	247113813	333	15	10164	-0.13117	0.55218
-	ZMAT3	3	179007223	179107279	200	28	10164	-0.1312	0.55219
-	TMPRSS11A	4	67900301	67998514	256	16	10164	-0.13119	0.55219
-	LOC1053769	3	12872360	12920099	111	16	10164	-0.13134	0.55225
+	RUSC1	1	155285849	155341118	94	15	10164	-0.13144	0.55229
-	DLX2	2	172089438	172137750	175	27	10164	-0.13163	0.55236
-	WDR41	5	77420933	77655611	704	36	10164	-0.13189	0.55246
+	FAM71F2	7	128636636	128712262	232	15	10164	-0.13199	0.5525
-	DDX23	12	48819756	48887174	122	19	10164	-0.13238	0.55266
+	LMO4	1	87293468	87358923	152	19	10164	-0.13258	0.55274
+	SPATS2L	2	200270881	200492263	620	59	10164	-0.13277	0.55281
+	PLCL1	2	197769602	198159884	557	18	10164	-0.13287	0.55285
-	BAP1	3	52391004	52445105	110	15	10164	-0.13296	0.55289
+	SOCS5	2	46663960	46772788	420	27	10164	-0.13307	0.55293
-	SNX5	20	17931596	18003991	258	16	10164	-0.13334	0.55304
-	FBXO11	2	47796920	47941535	213	63	10164	-0.13341	0.55307
+	DEFB135	8	11947321	11994590	27	5	10164	-0.13347	0.55309
+	CPQ	8	96610227	97153503	924	52	10164	-0.13352	0.55311
+	HTRA3	4	8234754	8317111	292	32	10164	-0.13355	0.55312
+	DLX6	7	96970978	97021040	29	11	10164	-0.13368	0.55317
-	LOC1019274	16	14885762	14948685	3	1	10164	-0.13369	0.55318
-	PPP3CA	4	101013430	101382471	922	55	10164	-0.13377	0.55321
+	INPP5F	10	119669395	119839278	453	44	10164	-0.13389	0.55326
-	MMP12	11	102852733	102910034	213	44	10164	-0.13416	0.55336
+	FAM65A	16	67493814	67556788	67	12	10164	-0.13418	0.55337

STRAND	SYMBOL	CHR	START	STOP	NSNPS	NPARAM	N	ZSTAT	P
+	GLIPR1	12	75445733	75511936	180	11	10164	-0.13423	0.55339
-	C17orf102	17	34564123	34614369	163	34	10164	-0.13424	0.55339
+	FTMT	5	121816955	121862833	143	17	10164	-0.1343	0.55342
+	AQP10	1	154286067	154335325	117	16	10164	-0.13443	0.55347
+	GALNT5	2	157222828	157321401	190	13	10164	-0.13466	0.55356
+	MEDAG	13	30871175	30935572	153	19	10164	-0.1347	0.55358
+	SAP30BP	17	75632116	75718062	23	5	10164	-0.13474	0.55359
-	KLF9	9	70374597	70449657	200	28	10164	-0.13478	0.55361
-	ZNF496	1	247287413	247368376	165	17	10164	-0.13483	0.55362
+	LOC200726	2	206607418	206659449	91	21	10164	-0.1353	0.55381
-	TACR2	10	69394202	69451918	167	13	10164	-0.13531	0.55382
-	CPLX1	4	774957	861157	326	31	10164	-0.13533	0.55382
-	KBTBD2	7	32858166	32927144	132	14	10164	-0.13539	0.55385
+	TMBIM6	12	49706510	49774934	192	14	10164	-0.13541	0.55386
-	TEAD3	6	35463597	35532084	346	51	10164	-0.13547	0.55388
+	MCCD1	6	31493962	31540231	536	43	10164	-0.13553	0.5539
+	PDPK1	16	2502964	2613191	12	5	10164	-0.13554	0.55391
+	IQCJ-SCHIP1	3	159034252	159907366	1992	103	10164	-0.13563	0.55394
+	MUC5AC	11	1122953	1211138	207	7	10164	-0.13591	0.55405
-	CREBBP	16	3715054	3915120	337	33	10164	-0.13593	0.55406
-	YRDC	1	37792942	37843193	72	11	10164	-0.13597	0.55408
+	FSD1L	9	105407242	105562433	212	21	10164	-0.13615	0.55415
-	GCH1	14	54832005	54937885	270	26	10164	-0.13671	0.55437
+	ZNF853	7	6580719	6634290	114	14	10164	-0.13692	0.55445
+	EXOC1	4	55818636	55915078	210	32	10164	-0.13699	0.55448
-	RHAG	6	49595175	49671874	304	22	10164	-0.13703	0.5545
+	GRHL3	1	24284322	24374482	181	14	10164	-0.13704	0.5545
+	PAPSS2	10	87624719	87757705	361	38	10164	-0.13707	0.55451
-	DUSP6	12	89337825	89387859	48	4	10164	-0.13715	0.55454
+	COX10	17	14034402	14227922	450	33	10164	-0.13729	0.5546
+	IGL	22	21991076	22932913	1673	104	10164	-0.13731	0.55461
+	ICT1	17	74977664	75031261	75	10	10164	-0.13734	0.55462
+	NBPF12	1	146903545	147006093	41	4	10164	-0.13751	0.55469
+	FBRSL1	12	132455428	132595188	599	50	10164	-0.13763	0.55473
-	TBCCD1	3	186536067	186605543	128	8	10164	-0.13766	0.55475
-	PVALB	22	36790701	36854473	166	24	10164	-0.13769	0.55476
+	SNRK	3	43251512	43361143	112	17	10164	-0.13776	0.55478
-	ZNF577	19	51845803	51925916	291	14	10164	-0.13791	0.55484
-	CCDC173	2	169635425	169729421	351	18	10164	-0.13789	0.55484
+	TNFAIP8L2-S	1	151121629	151180297	70	14	10164	-0.138	0.55488
+	PARK7	1	7926654	7995283	126	11	10164	-0.13805	0.5549
-	OR2T34	1	248563801	248609757	4	2	10164	-0.13832	0.55501
+	CYP4F3	19	15605897	15670760	454	19	10164	-0.13848	0.55507
-	IQGAP3	1	156515405	156607604	280	29	10164	-0.13883	0.55521
-	PARN	16	14425700	14665275	53	21	10164	-0.13951	0.55548
+	STARD3	17	39602080	39674201	111	15	10164	-0.13965	0.55553
+	HAT1	2	171887425	171993686	239	28	10164	-0.13987	0.55562
-	AQP12B	2	240666416	240720768	115	24	10164	-0.13989	0.55563
-	MCCC1	3	183005218	183134587	249	28	10164	-0.14012	0.55572
-	ZEB2	2	144374375	144555393	184	39	10164	-0.14041	0.55583
-	DARS	2	135895881	136020684	197	17	10164	-0.14047	0.55586
+	PALLD	4	168462064	168938457	1219	114	10164	-0.14062	0.55592
-	RICTOR	5	38927920	39109419	506	33	10164	-0.14077	0.55598
+	XKR8	1	27924462	27978096	154	15	10164	-0.14091	0.55603
-	GIMD1	4	106347335	106403788	154	17	10164	-0.14093	0.55604
+	TPH2	12	71903846	72045297	459	30	10164	-0.14132	0.55619
+	NISCH	3	52420508	52503072	166	18	10164	-0.14142	0.55623
-	EPPK1	8	143847319	143913464	73	14	10164	-0.14142	0.55623
+	CNTN2	1	205008202	205088043	283	35	10164	-0.14144	0.55624
+	SCG3	15	51646353	51731026	321	26	10164	-0.14153	0.55627
-	FA2H	16	74702955	74809831	312	34	10164	-0.14174	0.55636
+	SNX29	16	11941734	12584289	3186	163	10164	-0.14176	0.55636
+	CELA3A	1	21966656	22022542	334	21	10164	-0.14197	0.55645

STRAND	SYMBOL	CHR	START	STOP	NSNPS	NPARAM	N	ZSTAT	P
-	APOL4	22	36179128	36239833	321	25	10164	-0.14205	0.55648
-	FAM175A	4	83450939	83520163	183	9	10164	-0.14226	0.55656
+	RIPK1	6	3028967	3125187	233	26	10164	-0.14265	0.55672
-	RTP2	3	187688259	187751720	188	21	10164	-0.14275	0.55676
+	BARX2	11	129340238	129462279	418	62	10164	-0.14324	0.55695
+	THAP2	12	71628897	71690648	84	10	10164	-0.14324	0.55695
+	LOC1053763	9	83885901	83934902	115	15	10164	-0.1435	0.55705
+	OTUD6B	8	91035196	91097095	130	18	10164	-0.14361	0.55709
-	TBPL2	14	55404210	55475545	289	25	10164	-0.14371	0.55714
-	EMC3	3	9952682	10046116	390	17	10164	-0.14373	0.55715
+	TMEM39B	1	32037031	32112866	70	9	10164	-0.14377	0.55716
+	OR2H1	6	29422133	29474322	494	30	10164	-0.14385	0.55719
+	LOC1002872	3	158697441	158794039	298	21	10164	-0.14391	0.55722
-	GNB1L	22	19778411	19889939	347	37	10164	-0.14407	0.55728
+	C1QTNF9	13	24272166	24332531	167	33	10164	-0.1442	0.55733
-	PFN2	3	149954904	150005954	127	20	10164	-0.14444	0.55742
-	VAMP2	17	8149147	8197975	128	20	10164	-0.14448	0.55744
+	GML	8	142798998	142927044	162	18	10164	-0.14461	0.55749
-	PFN1	17	4935650	4984086	120	24	10164	-0.14463	0.5575
-	DPAGT1	11	119086503	119137414	160	22	10164	-0.1447	0.55753
+	TMEM263	12	106918415	106984035	167	17	10164	-0.14471	0.55753
+	TRIM43	2	95557018	95609771	43	19	10164	-0.14504	0.55766
+	CCNE1	19	29776898	29834312	167	23	10164	-0.14521	0.55773
+	CHP2	16	23719627	23768951	58	6	10164	-0.14521	0.55773
+	SLIT2	4	20216925	20630561	926	93	10164	-0.1455	0.55784
+	ITPR1	3	4458348	4857840	1075	133	10164	-0.14579	0.55796
+	MGAT3	22	39412182	39502194	192	17	10164	-0.14588	0.55799
-	FAM161A	2	61734556	61890402	403	21	10164	-0.14609	0.55807
+	FZD9	7	73398779	73446120	3	1	10164	-0.14642	0.5582
+	GCDH	19	12856129	12924630	85	8	10164	-0.14647	0.55822
-	DMWD	19	45773006	45827802	154	18	10164	-0.1465	0.55824
-	KLK5	19	50933299	50988088	229	55	10164	-0.14685	0.55838
-	CA11	19	48628015	48681194	91	14	10164	-0.14688	0.55839
-	HK3	5	176870869	176934373	119	20	10164	-0.14699	0.55843
+	BCAN	1	156606948	156669532	103	23	10164	-0.1472	0.55851
-	PPTC7	12	110523245	110618259	103	17	10164	-0.14724	0.55853
-	TVP23C	17	15492264	15598631	241	26	10164	-0.14726	0.55854
+	CASC4	15	44253616	44425761	292	16	10164	-0.14734	0.55857
+	CTTNBP2NL	1	112361178	112471164	195	9	10164	-0.14739	0.55859
+	HELT	4	184983841	185030804	223	22	10164	-0.14767	0.5587
-	DUSP7	3	52038919	52091445	111	19	10164	-0.14808	0.55886
-	FITM2	20	44296557	44346249	128	17	10164	-0.14835	0.55897
+	TMEM63C	14	77146759	77269495	562	51	10164	-0.14848	0.55902
-	TC2N	14	91769751	91902536	298	27	10164	-0.14869	0.5591
+	AKR1B10	7	134492592	134551414	262	25	10164	-0.14931	0.55935
-	S100A4	1	153533619	153580806	47	7	10164	-0.14947	0.55941
+	ASAP2	2	9171765	9415683	398	19	10164	-0.14951	0.55942
-	TMEM60	7	77783728	77833430	94	11	10164	-0.1495	0.55942
+	DAW1	2	227836054	227934347	449	20	10164	-0.14963	0.55947
+	MGAT2	14	49585771	49633481	164	14	10164	-0.14964	0.55948
+	DPRX	19	53566119	53647038	111	17	10164	-0.14998	0.55961
+	ARG1	6	131538203	131594332	126	14	10164	-0.15048	0.55981
-	PLD1	3	171590405	171845494	687	29	10164	-0.15049	0.55981
+	SYNGR4	19	48329281	48386377	139	17	10164	-0.15058	0.55985
+	CLIC6	21	34634292	34728227	247	21	10164	-0.15085	0.55995
+	AP2B1	17	35551899	35736417	503	27	10164	-0.151	0.56001
-	P2RY13	3	151316308	151364549	158	19	10164	-0.15111	0.56005
+	ZNF345	19	36815358	36903218	265	29	10164	-0.15122	0.5601
+	TPD52L2	20	63830228	63901545	201	26	10164	-0.15124	0.56011
+	NDFIP2	13	79445722	79566077	206	18	10164	-0.15133	0.56014
+	SIDT1	3	113497371	113642097	459	40	10164	-0.15141	0.56017
-	MECR	1	29157696	29265958	213	18	10164	-0.15146	0.5602
-	FANCA	16	89727551	89851658	810	27	10164	-0.15169	0.56028

STRAND	SYMBOL	CHR	START	STOP	NSNPS	NPARAM	N	ZSTAT	P
-	CHST9	18	26898754	27220338	1055	57	10164	-0.1517	0.56029
-	TIPIN	15	66326191	66421737	296	26	10164	-0.15173	0.5603
+	NCSTN	1	160308273	160368952	128	16	10164	-0.152	0.56041
-	CITED4	1	40851056	40897346	162	18	10164	-0.152	0.56041
-	MGAT1	5	180780541	180850652	142	6	10164	-0.15223	0.5605
+	IL15	4	141601596	141743987	327	25	10164	-0.15262	0.56065
+	SLC51A	3	196181512	196243430	243	29	10164	-0.15261	0.56065
-	OR10AG1	11	55957558	56003463	363	8	10164	-0.15262	0.56065
+	MYEOV	11	69259138	69307287	229	42	10164	-0.15266	0.56067
+	DPY19L3	19	32370749	32495893	190	24	10164	-0.15288	0.56076
+	ATP5C1	10	7753130	7817801	292	39	10164	-0.15297	0.56079
+	DYNLRB2	16	80505258	80560811	215	26	10164	-0.15319	0.56088
-	WAPAL	10	86425256	86556816	281	21	10164	-0.15326	0.5609
+	LOC1053778	6	79502645	79641131	480	35	10164	-0.15325	0.5609
+	COMT	22	19906740	19979975	383	47	10164	-0.15327	0.56091
-	AGMAT	1	15561699	15620110	232	13	10164	-0.15346	0.56098
+	CA4	17	60114941	60169546	68	13	10164	-0.15347	0.56099
+	HIF3A	19	46262046	46353433	299	20	10164	-0.15351	0.561
+	FOXB1	15	59969222	60015943	92	13	10164	-0.15364	0.56105
-	PTPRK	6	127958779	128555847	801	31	10164	-0.1539	0.56115
+	PAPD4	5	79577416	79696648	332	15	10164	-0.15396	0.56118
-	SEC31B	10	100476646	100554838	143	6	10164	-0.15418	0.56126
+	PYROXD1	12	21402604	21481250	287	14	10164	-0.15435	0.56133
-	ANAPC7	12	110362900	110438730	125	11	10164	-0.1544	0.56135
-	NMB	15	84645129	84693571	121	21	10164	-0.15441	0.56136
-	MYL3	3	46847867	46898483	116	28	10164	-0.15466	0.56146
-	ALG8	11	78090942	78174660	321	9	10164	-0.15469	0.56147
+	ABCG8	2	43793954	43890189	373	32	10164	-0.15479	0.56151
+	EGFL7	9	136623856	136682678	10	1	10164	-0.15503	0.5616
-	IFNA13	9	21357372	21403076	123	26	10164	-0.15504	0.56161
+	ZNF223	19	44017011	44077994	160	15	10164	-0.15507	0.56162
+	DNAJC16	1	15491813	15581733	266	13	10164	-0.15552	0.56179
-	HLA-DQB2	6	32746094	32798553	787	29	10164	-0.15606	0.56201
-	SALL4	20	51774009	51948245	731	64	10164	-0.15611	0.56203
-	ADCY3	2	24809169	24954733	431	23	10164	-0.15625	0.56208
-	SLC25A16	10	68463387	68562523	273	16	10164	-0.15624	0.56208
+	MEIS1	2	66400125	66582759	320	38	10164	-0.15629	0.5621
-	ST6GALNAC	17	76555379	76621066	157	15	10164	-0.1564	0.56214
+	EP400	12	131914920	132090466	81	14	10164	-0.1564	0.56214
+	RBPMS	8	30349465	30582261	502	35	10164	-0.15647	0.56217
+	PSMD8	19	38339550	38393824	228	37	10164	-0.15658	0.56221
-	DCLK3	3	36702422	36799204	225	46	10164	-0.1567	0.56226
-	KRTAP12-1	21	44671576	44717163	200	13	10164	-0.1568	0.5623
-	CHCHD2	7	56091573	56141494	174	21	10164	-0.15688	0.56233
+	RFXANK	19	19157199	19211869	208	22	10164	-0.15705	0.5624
+	CRIM1	2	36320771	36561135	705	64	10164	-0.15765	0.56263
+	ZFPL1	11	65049222	65098402	107	20	10164	-0.15766	0.56264
-	POLR2L	11	829721	877529	134	15	10164	-0.15773	0.56266
-	HIST3H2A	1	228447364	228492859	166	32	10164	-0.1579	0.56273
-	ZDHHC7	16	84964460	85046732	332	24	10164	-0.1581	0.56281
+	UBTD1	10	97463882	97581210	267	22	10164	-0.1581	0.56281
+	ARL14EP	11	30288099	30348458	189	17	10164	-0.15813	0.56282
+	VPS13A	9	77142353	77427483	862	33	10164	-0.15818	0.56284
-	ANO8	19	17313223	17369834	208	29	10164	-0.15838	0.56292
-	THAP7	22	20989772	21037115	173	22	10164	-0.15875	0.56307
+	CD9	12	6164844	6248271	279	27	10164	-0.159	0.56316
+	TRIM46	1	155138787	155194971	89	10	10164	-0.15898	0.56316
-	NTN5	19	48651407	48708081	115	15	10164	-0.15917	0.56323
-	TMEM9	1	201124771	201206582	341	44	10164	-0.15934	0.5633
+	MRGPRX4	11	18137837	18184280	214	6	10164	-0.15939	0.56332
-	ARL16	17	81671155	81718924	162	6	10164	-0.15947	0.56335
-	TXN2	22	36457036	36517025	152	9	10164	-0.15955	0.56338
+	ADIPOQ	3	186807668	186868463	154	25	10164	-0.1597	0.56344

STRAND	SYMBOL	CHR	START	STOP	NSNPS	NPARAM	N	ZSTAT	P
-	GPR75	2	53842913	53895033	149	20	10164	-0.15989	0.56351
+	ABHD16B	20	63826213	63872988	154	19	10164	-0.15999	0.56356
-	PROK2	3	71761655	71820206	80	13	10164	-0.16014	0.56361
+	PRDM6	5	123000985	123204266	700	52	10164	-0.16037	0.5637
-	TAS2R8	12	10796051	10841980	92	7	10164	-0.16051	0.56376
+	LOC1053777	8	2966061	3034227	299	21	10164	-0.16062	0.5638
-	EEF1A2	20	63478012	63534315	292	39	10164	-0.16078	0.56387
-	OR5I1	11	55925456	55971400	336	6	10164	-0.16078	0.56387
-	TAS2R7	12	10791532	10837627	113	7	10164	-0.16113	0.564
-	HOPX	4	56637988	56716706	265	33	10164	-0.1616	0.56419
-	KRTAP12-2	21	44656189	44701927	245	13	10164	-0.16175	0.56425
+	BLVRA	7	43723192	43817342	226	25	10164	-0.16191	0.56431
-	LOC1019297	9	92795385	92843644	109	13	10164	-0.16198	0.56434
+	SLC22A3	6	160313373	160462581	598	40	10164	-0.16255	0.56456
+	SFXN2	10	102679508	102749189	170	28	10164	-0.16265	0.5646
+	TBC1D22A	22	46727617	47213479	1884	96	10164	-0.16299	0.56474
+	ADPRH	3	119544433	119599945	229	15	10164	-0.16311	0.56478
-	SLC25A2	5	141292629	141339063	147	21	10164	-0.16373	0.56503
+	C6orf163	6	87308080	87375553	244	18	10164	-0.164	0.56513
+	GNLY	2	85659291	85708854	204	22	10164	-0.16409	0.56517
+	LOC1053758	8	60023460	60126407	256	25	10164	-0.16412	0.56518
+	CD44	11	35103870	35242402	500	79	10164	-0.16418	0.56521
+	MRPS22	3	139308994	139367129	125	17	10164	-0.16428	0.56524
-	KRTAP12-4	21	44644213	44689659	262	14	10164	-0.16443	0.5653
+	ABCB5	7	20580622	20767014	701	56	10164	-0.1645	0.56533
-	CPNE8	12	38642200	38941135	1026	48	10164	-0.16454	0.56535
+	IGSF21	1	18072746	18388484	759	92	10164	-0.16462	0.56538
-	TM4SF18	3	149308498	149368761	131	17	10164	-0.1648	0.56545
-	TAS2R50	12	10975913	11021912	206	5	10164	-0.16485	0.56547
-	TSTA3	8	143602618	143653043	130	28	10164	-0.16508	0.56556
-	LMOD1	1	201886456	201981588	292	59	10164	-0.16507	0.56556
-	SDC1	2	20190797	20260433	157	18	10164	-0.16547	0.56571
-	FGR	1	27602289	27670561	82	8	10164	-0.16595	0.5659
-	CLK1	2	200843009	200899744	129	43	10164	-0.16599	0.56592
-	KRTAP19-5	21	30491872	30537090	187	10	10164	-0.16601	0.56592
-	NEFL	8	24940955	24991869	123	10	10164	-0.16608	0.56595
-	CHMP3	2	86493430	86598497	189	11	10164	-0.16607	0.56595
-	CALHM1	10	103443387	103493891	137	27	10164	-0.16619	0.566
-	INTS12	4	105672627	105743729	115	14	10164	-0.16647	0.56611
-	LOC1053714	17	1215891	1264724	77	10	10164	-0.16652	0.56612
-	SULF2	20	47647406	47821629	674	96	10164	-0.16675	0.56622
+	ADCY1	7	45539140	45733116	614	55	10164	-0.16678	0.56623
+	ZNF791	19	12575918	12639862	103	5	10164	-0.16685	0.56626
-	SEMA6C	1	151121685	151181664	71	14	10164	-0.16693	0.56629
+	C18orf54	18	54322905	54395218	288	21	10164	-0.16698	0.56631
-	CDC42BPA	1	226979858	227353492	1952	33	10164	-0.16708	0.56635
-	PSG6	19	42892082	42952924	148	6	10164	-0.16717	0.56638
+	SPIN1	9	88353382	88488708	15	8	10164	-0.16721	0.5664
-	RB1CC1	8	52612458	52749466	112	17	10164	-0.16732	0.56644
+	KATNB1	16	57700720	57767250	234	33	10164	-0.16749	0.56651
-	LOC1053719	17	62453848	62514796	63	8	10164	-0.16756	0.56653
-	HIBCH	2	190194634	190355045	472	18	10164	-0.16775	0.56661
-	NOSIP	19	49545468	49615601	195	18	10164	-0.16798	0.5667
+	KLC1	14	103594188	103711551	473	23	10164	-0.16803	0.56672
-	ADORA3	1	111489429	111539121	142	18	10164	-0.16807	0.56673
-	GEMIN4	17	734416	787872	193	26	10164	-0.16811	0.56675
-	CDCP1	3	45072274	45181422	402	40	10164	-0.1681	0.56675
-	PGF	14	74931830	74990764	98	13	10164	-0.16816	0.56677
-	MLC1	22	50049391	50120929	361	17	10164	-0.16815	0.56677
+	MIS12	17	5451374	5500814	180	20	10164	-0.16853	0.56692
-	STARD10	11	72744729	72828705	183	19	10164	-0.16863	0.56696
-	COPA	1	160278587	160378564	205	21	10164	-0.16882	0.56703
-	TKT	3	53214707	53291114	319	27	10164	-0.16887	0.56705

STRAND	SYMBOL	CHR	START	STOP	NSNPS	NPARAM	N	ZSTAT	P
-	JAKMIP2	5	147575439	147817848	728	39	10164	-0.16891	0.56707
-	TRIM3	11	6438613	6509459	201	21	10164	-0.16895	0.56708
+	TBC1D29	17	30524566	30575667	73	7	10164	-0.16895	0.56708
-	SNW1	14	77707599	77796218	343	20	10164	-0.16916	0.56716
+	POLR1E	9	37450935	37513697	205	15	10164	-0.16918	0.56717
-	SFTPB	2	85647317	85703741	207	25	10164	-0.16922	0.56719
+	AHCYL2	7	129190014	129440211	371	22	10164	-0.1693	0.56722
-	ROCK1	18	20939740	21146851	137	8	10164	-0.1695	0.5673
-	LINGO2	9	27927617	29248981	4743	190	10164	-0.16951	0.5673
+	ZNF446	19	58439543	58499533	112	11	10164	-0.16973	0.56739
-	SLC7A10	19	33198664	33260850	215	49	10164	-0.16975	0.5674
-	C10orf82	10	116652211	116705269	142	14	10164	-0.16983	0.56743
-	GPR161	1	168069542	168172667	149	10	10164	-0.16992	0.56746
+	FOXB2	9	76984655	77030953	183	17	10164	-0.17012	0.56754
-	DNAJC28	21	33478055	33526747	108	14	10164	-0.17013	0.56755
-	ENTHD1	22	39733044	39928806	281	29	10164	-0.1702	0.56757
-	ATG9A	2	219209380	219264688	94	8	10164	-0.17037	0.56764
-	SNX6	14	34551409	34665160	250	21	10164	-0.17056	0.56772
-	LYAR	4	4257701	4325169	16	6	10164	-0.17112	0.56794
+	CD4	12	6754472	6830810	170	25	10164	-0.17137	0.56803
-	OXTR	3	8740408	8889549	570	49	10164	-0.17183	0.56822
-	FRZB	2	182823275	182901770	141	11	10164	-0.17216	0.56834
+	NCAM1	11	112926247	113288436	1393	79	10164	-0.17221	0.56836
+	MRPL12	17	81668370	81717526	176	7	10164	-0.17219	0.56836
+	PRRG2	19	49545617	49601008	156	15	10164	-0.17231	0.5684
+	TRIM32	9	116652302	116711300	166	22	10164	-0.1724	0.56844
+	ANKRD49	11	94458987	94509578	157	22	10164	-0.17263	0.56853
-	GNB5	15	52110926	52226368	422	25	10164	-0.17267	0.56854
+	DEF8	16	89913697	89978060	270	24	10164	-0.17277	0.56858
-	REN	1	204144816	204201337	164	21	10164	-0.17283	0.56861
+	SEPT9	17	77246410	77510596	677	63	10164	-0.17298	0.56866
+	ZNF84	12	133002286	133073304	58	8	10164	-0.17312	0.56872
-	ECD	10	73124524	73203095	190	3	10164	-0.17317	0.56874
-	H2AFX	11	119083874	119130467	145	22	10164	-0.17329	0.56879
+	TMEM42	3	44826723	44875667	49	7	10164	-0.17329	0.56879
+	ZNF284	19	44037144	44097470	170	16	10164	-0.1735	0.56887
-	SPATA31C2	9	88119305	88169985	148	15	10164	-0.17349	0.56887
+	ZNF140	12	133044687	133118803	89	6	10164	-0.17369	0.56894
+	FGFR3	4	1758299	1818872	135	24	10164	-0.17388	0.56902
+	CCDC27	1	3717398	3781645	181	21	10164	-0.17394	0.56904
+	LOC1053794	16	20639418	20728430	188	17	10164	-0.17419	0.56914
-	LONP1	19	5681834	5755452	190	24	10164	-0.17457	0.56929
-	MAP7	6	136332281	136585819	576	20	10164	-0.17479	0.56938
-	ADAM8	10	133252416	133311903	57	7	10164	-0.175	0.56946
+	AGPAT9	4	83500914	83615875	354	14	10164	-0.17509	0.5695
-	GBP5	1	89248950	89307861	91	19	10164	-0.1753	0.56958
-	KANSL3	2	96583155	96673379	143	34	10164	-0.17569	0.56973
-	AGR3	7	16844728	16916989	346	41	10164	-0.1757	0.56973
-	SLC22A25	11	63152266	63278516	239	9	10164	-0.17601	0.56986
+	PTGR2	14	73816831	73895465	359	23	10164	-0.17615	0.56991
-	HORMAD1	1	150688059	150755909	141	9	10164	-0.17631	0.56997
-	DPH7	9	137544899	137613935	93	6	10164	-0.17666	0.57011
-	TBXA2R	19	3584506	3641851	117	13	10164	-0.17671	0.57013
+	GLIPR1L2	12	75356070	75442688	266	16	10164	-0.1768	0.57017
-	KCTD8	4	44163903	44483885	701	50	10164	-0.17687	0.57019
-	ZNF180	19	44464428	44536750	295	26	10164	-0.17694	0.57022
+	CHRNG	2	232504727	232556328	181	26	10164	-0.17697	0.57023
+	PNRC2	1	23924811	23973462	81	8	10164	-0.17711	0.57029
+	CASC5	15	40559249	40672683	376	23	10164	-0.17712	0.57029
+	GNAI2	3	50191688	50269354	115	18	10164	-0.17716	0.57031
-	EMILIN3	20	41349966	41401858	162	29	10164	-0.17725	0.57034
+	TRIM67	1	231127624	231231568	326	51	10164	-0.17747	0.57043
-	DXO	6	31959811	32007290	116	36	10164	-0.17751	0.57045

STRAND	SYMBOL	CHR	START	STOP	NSNPS	NPARAM	N	ZSTAT	P
-	IMPDH1	7	128382277	128444987	168	19	10164	-0.17764	0.5705
+	EVI5L	19	7795273	7874976	141	15	10164	-0.17771	0.57053
+	GRPEL2	5	149310414	149364583	69	11	10164	-0.17788	0.57059
-	TNIP3	4	121121408	121262609	274	27	10164	-0.17794	0.57061
+	ELMO3	16	67164111	67214029	78	12	10164	-0.17818	0.57071
+	COQ10B	2	197418507	197485135	55	7	10164	-0.17823	0.57073
-	TMEM258	11	61779130	61827613	56	5	10164	-0.17826	0.57074
-	NR1I3	1	161219666	161273598	148	19	10164	-0.17839	0.57079
+	SNRPA	19	40715854	40775392	98	11	10164	-0.17854	0.57085
-	ZSCAN23	6	28415531	28478974	266	38	10164	-0.17869	0.57091
+	MORN5	9	122124858	122210088	249	35	10164	-0.1787	0.57091
+	OR2W5	1	247456068	247502409	212	9	10164	-0.17879	0.57095
-	ZNF354A	5	178701512	178765702	229	21	10164	-0.17887	0.57098
-	NELFE	6	31942087	31994087	179	49	10164	-0.17899	0.57103
+	ASB15	7	123531999	123647900	200	22	10164	-0.17916	0.57109
-	CACNA2D4	12	1781957	1955865	425	52	10164	-0.1792	0.57111
+	RASGRP3	2	33401349	33574731	642	106	10164	-0.17933	0.57116
-	APCDD1L	20	58442469	58550393	189	34	10164	-0.17932	0.57116
+	IFT88	13	20531446	20701437	424	24	10164	-0.17945	0.57121
+	MAPK8	10	48271639	48449360	456	23	10164	-0.17978	0.57134
-	C5	9	120942335	121109925	419	24	10164	-0.17991	0.57139
-	COL9A2	1	40290487	40352690	132	23	10164	-0.18014	0.57148
+	NHLH1	1	160251369	160382848	300	22	10164	-0.18018	0.57149
+	TBC1D23	3	100225817	100335252	219	21	10164	-0.18019	0.5715
+	RPL7A	9	133313214	133361425	135	15	10164	-0.18044	0.5716
+	MRPL40	22	19397513	19446077	137	17	10164	-0.18055	0.57164
-	AGT	1	230692523	230749590	301	27	10164	-0.18102	0.57183
+	RASSF4	10	44924750	45004724	288	54	10164	-0.18106	0.57184
+	KLF11	2	10007985	10064836	109	23	10164	-0.18115	0.57187
+	STRA8	7	135196979	135268661	197	24	10164	-0.18114	0.57187
+	C9orf171	9	132375043	132583319	526	77	10164	-0.18133	0.57194
-	GPATCH4	1	156584308	156636496	152	16	10164	-0.18135	0.57195
+	SLC35F1	6	117872231	118327676	1313	75	10164	-0.18138	0.57197
-	OR4K13	14	20023844	20069758	69	7	10164	-0.18165	0.57207
+	NDUFB4	3	120561281	120612411	87	9	10164	-0.18201	0.57221
+	HIF1AN	10	100500884	100563924	129	6	10164	-0.18219	0.57228
-	SFMBT2	10	7148624	7446486	534	49	10164	-0.18218	0.57228
+	ITFG3	16	199546	279943	250	30	10164	-0.18232	0.57234
-	CCSAP	1	229311005	229377941	116	13	10164	-0.18234	0.57234
-	GCM2	6	10862724	10921430	206	39	10164	-0.18254	0.57242
+	DUSP22	6	257057	361355	190	33	10164	-0.18264	0.57246
-	DNAAF3	19	55148661	55201722	228	22	10164	-0.18272	0.57249
+	CHTOP	1	153598982	153656306	90	7	10164	-0.18302	0.57261
+	OR2W3	1	247860587	247906531	159	17	10164	-0.18325	0.5727
-	C1orf35	1	228090727	228140411	167	23	10164	-0.18328	0.57271
+	MARCH9	12	57718622	57770410	99	21	10164	-0.18341	0.57276
-	C21orf2	21	44318944	44374402	117	10	10164	-0.18344	0.57277
-	SLC35B3	6	8403067	8470567	366	15	10164	-0.1836	0.57283
-	LPAR6	13	48401045	48479704	121	18	10164	-0.18379	0.57291
-	ZNF300	5	150884383	150939983	184	11	10164	-0.18381	0.57292
+	DNAJB2	2	219244318	219296900	97	11	10164	-0.18415	0.57305
+	LRRC17	7	102877897	102955111	150	15	10164	-0.18415	0.57305
-	TTC9B	19	40206058	40253399	69	13	10164	-0.18416	0.57306
+	ZFYVE21	14	103680744	103743668	235	25	10164	-0.18425	0.57309
-	ZNF318	6	43317165	43404459	173	25	10164	-0.18438	0.57314
+	HSF2	6	122364551	122443119	281	24	10164	-0.18472	0.57327
-	PIEZO2	18	10660134	11183762	1319	106	10164	-0.18499	0.57338
-	KLK1	19	50809146	50858787	224	42	10164	-0.18523	0.57347
+	POGK	1	166804449	166864472	125	11	10164	-0.18527	0.57349
+	UNC79	14	93298219	93717878	940	37	10164	-0.18591	0.57374
-	ZIC4	3	147376048	147442476	109	17	10164	-0.18645	0.57395
-	AGAP2	12	57713761	57777201	126	23	10164	-0.18658	0.57401
-	GPR31	6	167146872	167192831	92	12	10164	-0.18662	0.57402

STRAND	SYMBOL	CHR	START	STOP	NSNPS	NPARAM	N	ZSTAT	P
+	GLIPR2	9	36101536	36173913	179	21	10164	-0.18688	0.57412
+	UCK2	1	165792495	165921618	315	38	10164	-0.18709	0.5742
-	SPRY4	5	142300427	142360055	192	27	10164	-0.18724	0.57427
-	ZHX3	20	41168449	41352618	492	29	10164	-0.18742	0.57433
-	LOC1027244	13	113139633	113188986	101	11	10164	-0.18746	0.57435
-	TAS2R20	12	10985962	11032875	218	6	10164	-0.18768	0.57444
-	RECQL5	17	75616845	75702191	24	5	10164	-0.18773	0.57446
-	KIF27	9	83826698	83956467	287	21	10164	-0.18787	0.57451
+	PTPRO	12	15287257	15608331	845	52	10164	-0.18805	0.57458
+	HSD3B7	16	30950110	30999152	44	4	10164	-0.18817	0.57463
-	HCLS1	3	121621398	121695948	185	21	10164	-0.18868	0.57483
+	MARCO	2	118907166	119004664	284	25	10164	-0.18872	0.57484
+	APOPT1	14	103527957	103602187	228	26	10164	-0.1888	0.57488
-	RNF122	8	33537754	33602128	179	13	10164	-0.18902	0.57496
-	ELK4	1	205583466	205669085	262	32	10164	-0.18909	0.57499
-	TRPC6	11	101441564	101619018	710	39	10164	-0.18912	0.575
-	SAFB2	19	5576993	5657927	170	16	10164	-0.18931	0.57507
-	ADCY8	8	130770300	131076598	827	53	10164	-0.18933	0.57508
+	PRKAR2B	7	107009728	107171811	330	22	10164	-0.18946	0.57513
-	ARHGEF39	9	35648875	35710738	193	33	10164	-0.18948	0.57514
+	CPA3	3	148830256	148907087	251	19	10164	-0.18963	0.5752
-	SERPINA11	14	94432464	94487850	232	24	10164	-0.18966	0.57521
+	ITIH2	10	7668273	7759520	417	46	10164	-0.1897	0.57523
+	SLC5A1	22	32008032	32123029	168	30	10164	-0.1897	0.57523
+	GNG10	9	111626571	111680246	222	23	10164	-0.18974	0.57524
-	MTMR7	8	17286797	17448531	872	69	10164	-0.18988	0.5753
-	GOLGA8A	15	34369068	34472466	129	2	10164	-0.18991	0.57531
+	ZNF530	19	57564885	57622722	118	15	10164	-0.18994	0.57532
+	IGF2BP1	17	48962412	49066145	238	39	10164	-0.1903	0.57546
-	ABR	17	993518	1222322	521	84	10164	-0.1905	0.57554
+	HAPLN2	1	156583801	156635725	149	15	10164	-0.19086	0.57568
-	NDC1	1	53755460	53873552	213	24	10164	-0.19141	0.5759
-	FAM189A1	15	29110252	29605723	938	48	10164	-0.19169	0.57601
-	TRIM33	1	114382777	114546160	208	15	10164	-0.19216	0.57619
-	ELMO1	7	36842906	37484409	1639	136	10164	-0.19218	0.5762
+	MTDH	8	97609147	97740260	262	22	10164	-0.19256	0.57635
+	C4orf26	4	75520510	75575893	142	19	10164	-0.19266	0.57639
-	FGF10	5	44293541	44424780	386	29	10164	-0.19286	0.57647
-	LILRA6	19	54226592	54277768	69	11	10164	-0.19298	0.57651
-	SIRPD	20	1524223	1594006	116	11	10164	-0.19339	0.57667
-	VCPIP1	8	66620253	66702217	97	6	10164	-0.19361	0.57676
+	COL14A1	8	120090108	120383573	686	46	10164	-0.19368	0.57679
+	GABRD	1	1984329	2040753	212	38	10164	-0.19382	0.57684
-	LOC1053695	11	126101558	126175687	302	39	10164	-0.19384	0.57685
-	HIST1H2AA	6	25716063	25761562	260	20	10164	-0.19391	0.57688
-	NCKIPSD	3	48663839	48720933	90	9	10164	-0.19395	0.57689
-	SPHKAP	2	227969954	228216962	758	28	10164	-0.19414	0.57697
-	KDELC1	13	102774281	102834054	203	25	10164	-0.19424	0.57701
-	CHML	1	241618864	241670930	104	23	10164	-0.19442	0.57708
+	CNTN5	11	98985976	100368885	5631	231	10164	-0.19454	0.57712
-	RNASE1	14	20791356	20837877	57	14	10164	-0.19483	0.57724
-	PICALM	11	85947171	86104881	527	32	10164	-0.19484	0.57724
-	CEACAM1	19	42497306	42563509	58	6	10164	-0.19485	0.57725
+	CAP1	1	40005240	40082649	307	12	10164	-0.19488	0.57726
+	KATNAL2	18	46882602	47112243	556	43	10164	-0.19494	0.57728
-	CCDC114	19	48286452	48357795	197	16	10164	-0.19523	0.57739
+	F7	13	113070788	113130681	199	30	10164	-0.19545	0.57748
+	GMPS	3	155834430	155949608	401	22	10164	-0.19545	0.57748
-	SMURF2	17	64532282	64697268	180	5	10164	-0.19564	0.57755
+	CMTM1	16	66531391	66589135	131	6	10164	-0.19564	0.57755
-	LEMD1	1	205371378	205484950	235	26	10164	-0.19584	0.57763
-	SLC17A7	19	49419398	49476551	137	17	10164	-0.19586	0.57764
+	MPZL1	1	167686950	167801919	231	22	10164	-0.19618	0.57776

STRAND	SYMBOL	CHR	START	STOP	NSNPS	NPARAM	N	ZSTAT	P
+	SPINK6	5	148167794	148225137	262	13	10164	-0.19626	0.5778
-	LOC1053737	2	177201394	177261835	131	16	10164	-0.19661	0.57793
+	BACE2	21	41132801	41292534	402	61	10164	-0.19664	0.57794
-	TMEM189-UE	20	50071124	50188798	330	21	10164	-0.1968	0.57801
-	TAS2R9	12	10799094	10845168	85	7	10164	-0.19685	0.57803
-	HDDC2	6	125265350	125337141	251	22	10164	-0.19703	0.5781
+	TANC2	17	62931664	63437706	841	27	10164	-0.19705	0.57811
-	SYNRG	17	37504797	37644496	243	35	10164	-0.19715	0.57814
-	MTERF4	2	241064680	241137359	293	22	10164	-0.19717	0.57815
-	OR14I1	1	248473422	248733899	218	5	10164	-0.19746	0.57826
+	TLX3	5	171274284	171322134	81	7	10164	-0.19751	0.57829
-	DCXR	17	82025881	82072697	53	6	10164	-0.19753	0.57829
-	CPNE5	6	36730772	36874444	603	73	10164	-0.19765	0.57834
-	ANXA2R	5	43029080	43075345	86	14	10164	-0.19784	0.57842
-	LOC1019270	13	63822066	63868720	86	6	10164	-0.19789	0.57844
-	CUL3	2	224460150	224620397	434	25	10164	-0.19798	0.57847
-	RNASE13	14	21022820	21069785	101	27	10164	-0.19805	0.5785
-	KIAA1324L	7	86866906	87094698	617	29	10164	-0.19812	0.57853
-	TMEM245	9	109005135	109154945	402	29	10164	-0.19817	0.57855
+	SLC26A2	5	149925737	149997400	146	22	10164	-0.19821	0.57856
+	INTS3	1	153693050	153784808	103	24	10164	-0.19825	0.57857
+	ZNF511	10	133273889	133323162	52	5	10164	-0.19823	0.57857
+	TTYH3	7	2596969	2674802	237	33	10164	-0.19834	0.57861
-	MFNG	22	37459063	37521440	134	17	10164	-0.19853	0.57868
+	MT2A	16	56573566	56619497	210	39	10164	-0.1986	0.57871
+	DCLRE1B	1	113870293	113924086	125	26	10164	-0.19899	0.57887
-	TMED5	1	93139742	93215689	157	15	10164	-0.19911	0.57891
+	SRSF8	11	95031877	95081227	100	11	10164	-0.19914	0.57892
-	C17orf75	17	32318441	32385047	63	6	10164	-0.19943	0.57904
-	EFCAB11	14	89784984	89989777	628	32	10164	-0.19967	0.57913
-	BHLHE23	20	62995979	63042035	176	11	10164	-0.1997	0.57914
-	ABCG5	2	43796155	43873865	304	23	10164	-0.19971	0.57915
+	DKK1	10	52279281	52327657	104	19	10164	-0.19977	0.57917
-	HIST2H3D	1	149803225	149848681	10	2	10164	-0.20006	0.57928
-	CLEC1B	12	9975697	10036865	244	28	10164	-0.20016	0.57932
-	FOXP1	3	70944714	71618989	1376	157	10164	-0.20025	0.57936
-	C19orf12	19	29688886	29750789	273	39	10164	-0.20065	0.57951
+	WDR70	5	37344289	37762674	865	23	10164	-0.20076	0.57956
-	HOMER3	19	18919201	18976258	110	29	10164	-0.20114	0.5797
+	LOC1053781	6	168929400	169073791	672	46	10164	-0.20122	0.57974
+	PDK1	2	172520373	172680881	708	36	10164	-0.20144	0.57982
-	NMRAL1	16	4451689	4509895	2	1	10164	-0.20144	0.57982
+	AMPD3	11	10415321	10517579	223	23	10164	-0.20163	0.5799
+	CLCA2	1	86389086	86466558	326	17	10164	-0.20165	0.5799
-	PCID2	13	113167539	113244466	176	11	10164	-0.20173	0.57994
+	TLDC2	20	36841121	36904235	127	23	10164	-0.20175	0.57995
+	PEA15	1	160170319	160225376	163	31	10164	-0.20202	0.58005
-	LIMD2	17	63685889	63736769	92	9	10164	-0.20205	0.58006
+	MAPKBP1	15	41739434	41837855	186	23	10164	-0.20228	0.58015
+	LOC1053699	12	94613497	94661091	81	18	10164	-0.20266	0.5803
+	MYCBPAP	17	50473384	50541502	158	18	10164	-0.20272	0.58032
+	OR52I1	11	4559039	4605013	157	24	10164	-0.20285	0.58037
-	SPATA25	20	45876486	45923653	134	31	10164	-0.20298	0.58042
+	SPOPL	2	138466780	138583235	149	20	10164	-0.20304	0.58045
+	OAZ1	19	2234486	2283488	152	24	10164	-0.20317	0.5805
+	HOXD9	2	176087685	176134917	157	44	10164	-0.20347	0.58062
-	DNASE2	19	12865211	12916521	61	7	10164	-0.2036	0.58067
+	SMAD1	4	145446306	145569176	214	9	10164	-0.20369	0.5807
-	C8orf87	8	93124095	93201850	143	20	10164	-0.2037	0.5807
+	LARP7	4	112601964	112667592	138	21	10164	-0.20371	0.58071
-	TMPRSS11B	4	68216653	68280694	68	13	10164	-0.20376	0.58073
+	ZNF444	19	56105951	56170893	98	10	10164	-0.2039	0.58078
-	CYB561A3	11	61338748	61397299	53	10	10164	-0.20393	0.5808

STRAND	SYMBOL	CHR	START	STOP	NSNPS	NPARAM	N	ZSTAT	P
+	CD27	12	6409885	6461718	150	27	10164	-0.204	0.58083
+	POP7	7	100671053	100717500	90	13	10164	-0.20407	0.58085
-	FLCN	17	17196924	17272188	158	11	10164	-0.20469	0.58109
+	ARNTL	11	13242727	13397811	447	54	10164	-0.20474	0.58111
+	ZNF567	19	36652564	36733358	240	22	10164	-0.20477	0.58112
-	EDEM3	1	184680491	184789907	425	16	10164	-0.20477	0.58113
+	BANK1	4	101755607	102084812	950	36	10164	-0.2048	0.58114
+	FXYD3	19	35080816	35134324	131	15	10164	-0.20496	0.5812
+	XPO7	8	21884669	22016585	296	22	10164	-0.20514	0.58127
-	APOB	2	20989218	21079073	156	22	10164	-0.20518	0.58128
-	TEX15	8	30821423	30948015	300	18	10164	-0.20521	0.5813
-	ZNF385C	17	42014876	42107630	134	19	10164	-0.20539	0.58137
-	TP53INP1	8	94915972	94984387	247	16	10164	-0.20578	0.58152
+	C4A	6	31947057	32012680	170	48	10164	-0.20598	0.5816
-	FEZ2	2	36542239	36633190	339	28	10164	-0.20614	0.58166
+	DDTL	22	23931838	23982559	38	5	10164	-0.20613	0.58166
-	PARS2	1	54745519	54799553	182	30	10164	-0.20624	0.5817
-	SPRR2F	1	153102124	153148515	144	10	10164	-0.20626	0.58171
-	HCAR2	12	122691293	122738357	48	2	10164	-0.20648	0.58179
+	ACAP1	17	7301511	7361478	129	25	10164	-0.20697	0.58198
-	GBP3	1	88996677	89057880	182	18	10164	-0.20699	0.58199
-	LRRC20	10	70288970	70417650	469	38	10164	-0.20703	0.58201
-	SIT1	9	35639298	35685950	176	30	10164	-0.20707	0.58202
-	ABCC8	11	17382885	17511845	496	57	10164	-0.20737	0.58214
+	GPBAR1	2	218224496	218273861	77	8	10164	-0.20738	0.58214
-	PGBD5	1	230311646	230460928	344	25	10164	-0.20785	0.58233
-	LOC1053723	19	35032534	35090066	141	31	10164	-0.20797	0.58237
+	CEACAM5	19	41673611	41740433	106	11	10164	-0.20798	0.58238
+	TMEM106C	12	47928547	47978878	208	33	10164	-0.20807	0.58241
+	ZNF713	7	55852277	55952225	292	35	10164	-0.20809	0.58242
-	MMD	17	55382613	55456993	141	22	10164	-0.20834	0.58252
+	TSPAN31	12	57709090	57758243	103	21	10164	-0.20839	0.58254
+	RBM7	11	114365529	114420610	78	9	10164	-0.20857	0.58261
-	XDH	2	31324309	31449749	435	49	10164	-0.20865	0.58264
-	TEX30	13	102755888	102808821	146	18	10164	-0.20881	0.5827
-	AQR	15	34846351	35004794	296	15	10164	-0.20882	0.58271
-	RP1L1	8	10596349	10690107	383	62	10164	-0.20898	0.58277
-	SLC7A9	19	32820511	32905167	351	21	10164	-0.20917	0.58284
+	MIR205HG	1	209393823	209442552	102	21	10164	-0.20918	0.58285
-	FMOD	1	203330621	203386429	148	26	10164	-0.20921	0.58286
-	PEBP4	8	22703251	23034754	842	115	10164	-0.20954	0.58299
-	C2orf68	2	85595253	85647056	116	16	10164	-0.20955	0.58299
-	ULK2	17	19760828	19902926	323	17	10164	-0.20983	0.5831
+	DHRS9	2	169032035	169106167	271	23	10164	-0.20983	0.5831
+	FMO1	1	171213471	171295978	118	10	10164	-0.20989	0.58312
-	NUFIP1	13	44929249	45024478	162	24	10164	-0.21001	0.58317
-	PIK3AP1	10	96583312	96755522	313	38	10164	-0.21004	0.58318
-	ZBTB44	11	130216677	130349769	176	19	10164	-0.21007	0.58319
-	FBXO21	12	117133780	117225495	315	19	10164	-0.21029	0.58328
+	CREM	10	35091841	35222958	296	9	10164	-0.21048	0.58335
+	CASP8	2	201198443	201297711	308	46	10164	-0.21059	0.5834
-	ACD	16	67647512	67695815	54	12	10164	-0.21074	0.58345
-	DPPA2	3	109283788	109351535	188	15	10164	-0.2114	0.58371
-	SLFNL1	1	41005597	41058444	176	26	10164	-0.21144	0.58373
+	CHRND	2	232491160	232546665	165	27	10164	-0.21175	0.58385
+	TTC17	11	43323885	43504933	390	39	10164	-0.21179	0.58387
-	LOC1053736	2	126525789	126675247	273	23	10164	-0.21191	0.58391
+	ARL5B	10	18624335	18691639	191	19	10164	-0.21212	0.58399
-	MAD2L1	4	120049422	120101858	28	3	10164	-0.21214	0.584
-	ANAPC1	2	111757637	111919186	162	12	10164	-0.21223	0.58404
-	ARFGAP2	11	47154298	47212503	106	14	10164	-0.21232	0.58407
-	LOC1019286	19	2898628	2961778	160	12	10164	-0.21235	0.58408
+	RAD51	15	40659774	40742158	241	24	10164	-0.21252	0.58415

STRAND	SYMBOL	CHR	START	STOP	NSNPS	NPARAM	N	ZSTAT	P
+	CNTF	11	58587673	58635733	102	16	10164	-0.21264	0.5842
+	OR10P1	12	55601892	55647833	161	17	10164	-0.21273	0.58423
-	TRIM63	1	26041304	26102634	142	12	10164	-0.21288	0.58429
+	PALMD	1	99610875	99704541	172	30	10164	-0.213	0.58433
+	STK16	2	219210469	219260337	81	8	10164	-0.21305	0.58436
+	BICC1	10	58477916	58841437	55	13	10164	-0.21314	0.58439
-	TMTC4	13	100593836	100710089	276	24	10164	-0.21323	0.58442
+	ZNF502	3	44677643	44733831	63	9	10164	-0.2134	0.58449
+	NOP56	20	2617532	2668393	265	18	10164	-0.2135	0.58453
-	ZNF100	19	21714041	21802628	322	23	10164	-0.21356	0.58455
-	EIF3G	19	10105014	10154923	121	17	10164	-0.21362	0.58458
-	IP6K2	3	48678003	48752278	105	11	10164	-0.21396	0.58471
-	NUAK2	1	205292059	205356791	153	24	10164	-0.214	0.58473
-	CYS1	2	10046799	10115411	129	17	10164	-0.21418	0.5848
+	CYP4A22	1	47102435	47159738	155	16	10164	-0.21419	0.5848
+	HAUS5	19	35577744	35635349	121	21	10164	-0.21421	0.58481
-	RNF103	2	86593393	86658877	133	16	10164	-0.21434	0.58486
-	CRYGD	2	208111607	208159589	204	20	10164	-0.21444	0.5849
-	C11orf40	11	4561423	4612820	155	24	10164	-0.21448	0.58492
-	NDUFA5	7	123526997	123636651	201	22	10164	-0.21455	0.58494
+	RALB	2	120205070	120304711	280	23	10164	-0.21455	0.58494
+	LOC1005057	18	72832267	72891399	203	29	10164	-0.21457	0.58495
+	LOC1053720	18	5594967	5640924	131	36	10164	-0.21459	0.58496
-	ZNF184	6	27440742	27508118	277	25	10164	-0.21463	0.58497
-	CDK5	7	151043812	151092965	124	23	10164	-0.21476	0.58502
+	POLH	6	43541141	43630523	111	12	10164	-0.21486	0.58506
+	RPS5	19	58352269	58404804	105	9	10164	-0.21484	0.58506
+	ACY1	3	51948284	51999202	69	11	10164	-0.215	0.58512
+	CLTA	9	36155856	36222062	147	12	10164	-0.215	0.58512
+	FUT8	14	65375592	65754121	1132	36	10164	-0.21565	0.58537
-	PRR4	12	10835849	10884476	93	6	10164	-0.21571	0.58539
+	ZNF473	19	49990383	50058776	170	20	10164	-0.21572	0.5854
+	YAE1D1	7	39531376	39622089	270	22	10164	-0.21572	0.5854
-	LRP12	8	104479231	104624024	314	26	10164	-0.21586	0.58545
+	HSPA2	14	64505468	64553237	124	13	10164	-0.216	0.58551
+	OR4A15	11	55332884	55378918	174	15	10164	-0.21612	0.58555
-	PIGH	14	67579306	67635300	271	26	10164	-0.21632	0.58563
-	KRT80	12	52158996	52227000	86	13	10164	-0.21644	0.58568
+	FKTN	9	105523119	105651118	199	21	10164	-0.21683	0.58583
+	SMPDL3B	1	27899948	27969157	213	14	10164	-0.21689	0.58585
-	CDK20	9	87956441	88009780	133	14	10164	-0.2172	0.58597
+	PTPRJ	11	47945558	48180842	461	35	10164	-0.21743	0.58607
-	LOC285423	4	149140322	149850835	1414	55	10164	-0.21787	0.58624
+	RNF144A	2	6882392	7086197	554	42	10164	-0.21817	0.58635
+	PRR7	5	177411344	177466701	60	6	10164	-0.21827	0.58639
+	GOLGA7	8	41455562	41520980	149	13	10164	-0.21841	0.58645
+	BRCA2	13	32279862	32409850	368	35	10164	-0.21874	0.58657
-	KCNT2	1	196215779	196643560	674	24	10164	-0.21887	0.58662
-	CACTIN	19	3600628	3661815	150	14	10164	-0.21898	0.58667
-	LOC1053714	16	89836860	89889276	201	16	10164	-0.21903	0.58668
-	RASGRP4	19	38399058	38461305	310	39	10164	-0.21908	0.58671
-	SDC3	1	30859466	30944735	161	26	10164	-0.21918	0.58674
+	ADGRA2	8	37761883	37853986	169	38	10164	-0.21921	0.58676
+	PIP	7	143097081	143149741	55	2	10164	-0.21939	0.58683
+	SLC17A4	6	25718359	25791175	391	28	10164	-0.21949	0.58687
+	LIN52	14	74049836	74210414	581	24	10164	-0.21993	0.58704
+	CCDC137	17	81631731	81683907	212	7	10164	-0.21997	0.58705
+	ZNF93	19	19865913	19945998	205	20	10164	-0.22001	0.58707
-	FOXA2	20	22571004	22620463	100	7	10164	-0.22023	0.58715
-	TTC7B	14	90530588	90851479	771	64	10164	-0.22025	0.58716
+	UBE4B	1	9997983	10191239	307	43	10164	-0.22056	0.58728
+	SCNM1	1	151131022	151180297	60	13	10164	-0.22055	0.58728
+	ITGA6	2	172392586	172516453	557	43	10164	-0.22062	0.58731

STRAND	SYMBOL	CHR	START	STOP	NSNPS	NPARAM	N	ZSTAT	P
+	LOC1053767	16	45887	93044	126	13	10164	-0.22067	0.58732
+	FAM185A	7	102713944	102922918	489	22	10164	-0.22095	0.58743
+	TYW3	1	74698143	74776677	217	19	10164	-0.2213	0.58757
-	GRIN2A	16	9743404	10218364	1654	68	10164	-0.22141	0.58761
-	IMPG1	6	75911114	76107678	506	50	10164	-0.22152	0.58766
+	RBM25	14	73023436	73131368	306	26	10164	-0.22152	0.58766
+	B3GALT6	1	1197249	1245041	176	10	10164	-0.22162	0.58769
+	FANCD2	3	9991387	10111932	474	20	10164	-0.22202	0.58785
-	C1orf229	1	247100160	247147417	110	11	10164	-0.2222	0.58792
-	EDEM2	20	35105357	35182358	230	20	10164	-0.22224	0.58794
-	RFC4	3	186779892	186841695	173	20	10164	-0.22245	0.58802
-	ATP6V0D1	16	67428014	67516186	83	13	10164	-0.22281	0.58816
-	LOC1053782	10	45170356	45229262	220	19	10164	-0.22294	0.58821
-	LGR4	11	27355961	27507787	287	46	10164	-0.22328	0.58834
+	ABHD5	3	43655883	43732725	82	7	10164	-0.22336	0.58837
+	DDR2	1	162596265	162796591	675	35	10164	-0.22346	0.58841
+	TENM3	4	182288984	182813024	1231	105	10164	-0.22364	0.58848
-	UGT3A1	5	35943089	36036045	333	37	10164	-0.22365	0.58848
+	KIAA0922	4	153431346	153647608	456	50	10164	-0.22394	0.5886
-	LOC1053719	17	77363730	77412341	167	19	10164	-0.2241	0.58866
+	ZNF593	1	26134897	26180873	82	13	10164	-0.22426	0.58872
-	C8orf86	8	38494582	38563665	214	31	10164	-0.22426	0.58872
+	GIMAP4	7	150532370	150583955	298	15	10164	-0.22452	0.58883
-	CD300LD	17	74569972	74627231	238	29	10164	-0.22458	0.58885
-	VSTM1	19	54030825	54098966	19	3	10164	-0.22481	0.58894
+	NCOA1	2	24457050	24780702	543	24	10164	-0.22487	0.58896
-	TMEM9B	11	8937201	9000011	162	14	10164	-0.22493	0.58898
-	EXOC3L1	16	67174379	67225204	89	13	10164	-0.22493	0.58898
+	NPIPA1	16	14900310	14962074	1	1	10164	-0.22498	0.589
+	RWDD1	6	116536367	116603601	200	25	10164	-0.22497	0.589
-	RBM28	7	128300383	128378913	108	22	10164	-0.22497	0.589
+	MAP2K5	15	67507606	67817117	951	39	10164	-0.22508	0.58904
-	MYL10	7	101603325	101664296	202	22	10164	-0.22518	0.58908
-	BCL6	3	187711377	187780725	179	31	10164	-0.22539	0.58916
-	CCNE2	8	94870224	94931653	187	24	10164	-0.2254	0.58917
-	TRPC4	13	37623872	37904802	1012	67	10164	-0.22546	0.58919
-	WDR73	15	84631495	84689343	151	22	10164	-0.22579	0.58932
-	ORMDL1	2	189757220	189819371	162	19	10164	-0.22608	0.58943
-	SPTY2D1	11	18596401	18669786	204	25	10164	-0.22611	0.58944
-	ZZZ3	1	77551521	77718426	239	21	10164	-0.22617	0.58947
-	LIM2	19	51369909	51422956	93	21	10164	-0.2263	0.58952
+	ZBTB49	4	4255197	4331786	27	9	10164	-0.22657	0.58962
+	CGB5	19	49008845	49055311	168	22	10164	-0.22678	0.5897
-	UBR4	1	19064506	19245319	353	19	10164	-0.22727	0.58989
+	SLC27A6	5	128930475	129043642	760	20	10164	-0.22726	0.58989
+	MRPS18C	4	83420932	83471723	78	6	10164	-0.22736	0.58993
-	NVL	1	224217334	224365201	331	18	10164	-0.22739	0.58994
+	XPNPEP3	22	40822081	40942819	170	32	10164	-0.22753	0.58999
-	WRAP73	1	3620767	3685107	33	7	10164	-0.22767	0.59005
+	METAP1D	2	171964897	172092430	336	29	10164	-0.22771	0.59007
-	PCBP4	3	51947454	52002466	72	12	10164	-0.22774	0.59008
-	GART	21	33493931	33577917	130	15	10164	-0.22781	0.5901
-	LOC1001303	6	13269295	13332297	327	20	10164	-0.22807	0.59021
+	RNF167	17	4905033	4955222	165	27	10164	-0.22811	0.59022
-	NUFIP2	17	29245836	29329148	152	15	10164	-0.22835	0.59031
-	LMO3	12	16538372	16645130	129	31	10164	-0.2285	0.59037
-	KRTAP19-1	21	30470045	30515317	132	10	10164	-0.22854	0.59039
-	ATP6V1H	8	53705543	53878311	230	16	10164	-0.22916	0.59063
+	LSM14A	19	34137447	34239515	62	12	10164	-0.22926	0.59067
-	B3GALNT1	3	161073883	161140630	266	24	10164	-0.22933	0.59069
+	KREMEN2	16	2929216	2978383	175	17	10164	-0.22934	0.5907
-	CTCFL	20	57485965	57560652	257	31	10164	-0.22957	0.59079
+	DEFB107B	8	7460846	7519311	1	1	10164	-0.2297	0.59084

STRAND	SYMBOL	CHR	START	STOP	NSNPS	NPARAM	N	ZSTAT	P
-	DEFB106B	8	7472504	7521387	1	1	10164	-0.2297	0.59084
+	PRR23D1	8	7501780	7552450	1	1	10164	-0.2297	0.59084
-	GMDS	6	1613800	2280634	1617	122	10164	-0.22983	0.59089
+	LOC1053743	3	150055269	150161046	343	45	10164	-0.22985	0.5909
-	FGL1	8	17854391	17930538	386	19	10164	-0.23022	0.59104
+	IL1RL1	2	102276502	102362366	336	21	10164	-0.2304	0.59111
+	NUPL1	13	25266084	25359795	91	9	10164	-0.23046	0.59113
-	CPT1A	11	68744620	68876931	505	57	10164	-0.23058	0.59118
-	S100A5	1	153527147	153577175	56	7	10164	-0.23096	0.59133
-	DRGX	10	49354066	49431016	207	24	10164	-0.23098	0.59133
+	RNASE10	14	20475472	20521122	51	10	10164	-0.23102	0.59135
-	TUBGCP6	22	50207689	50280024	268	34	10164	-0.23118	0.59141
+	OCM	7	5824804	5896363	307	36	10164	-0.23121	0.59143
-	CCDC53	12	102002840	102097124	146	15	10164	-0.23124	0.59144
+	PRR13	12	53406649	53456643	72	6	10164	-0.23168	0.59161
-	AARS	16	70242295	70324550	57	18	10164	-0.23196	0.59172
+	MCM8	20	5915652	6005206	362	25	10164	-0.23202	0.59174
-	ANKRD22	10	88809899	88886975	260	25	10164	-0.23226	0.59183
+	REC114	15	73341209	73570013	490	45	10164	-0.23226	0.59183
-	UBE2D3	4	102784383	102903893	258	11	10164	-0.23243	0.5919
-	ZP4	1	237867864	237925922	116	8	10164	-0.2325	0.59192
+	PCBP2	12	53417102	53491162	102	11	10164	-0.23253	0.59194
+	SYS1	20	45326937	45386802	157	22	10164	-0.23261	0.59197
-	RARG	12	53200566	53267256	150	28	10164	-0.23268	0.59199
-	ABI1	10	26736596	26896087	569	28	10164	-0.23268	0.59199
-	OR56A5	11	5957553	6003494	135	21	10164	-0.23266	0.59199
+	OR4F15	15	101783187	101829125	70	6	10164	-0.23285	0.59206
-	LYVE1	11	10547866	10603818	46	5	10164	-0.23292	0.59209
-	GRM5	11	88494576	89100961	2389	69	10164	-0.23299	0.59212
+	CISD2	4	102833978	102902807	131	8	10164	-0.23305	0.59214
+	CHI3L2	1	111192659	111253440	212	23	10164	-0.23307	0.59215
+	FAM110D	1	26124008	26172628	93	12	10164	-0.23342	0.59228
-	MZF1	19	58551917	58609439	46	7	10164	-0.23344	0.59229
-	TXLNB	6	139149160	139359011	560	78	10164	-0.23348	0.59231
-	COX17	3	119659525	119712396	181	16	10164	-0.23359	0.59235
-	ECE1	1	21207247	21380541	325	38	10164	-0.23361	0.59236
+	HINFP	11	119086523	119145055	179	23	10164	-0.23372	0.5924
-	PSORS1C2	6	31127534	31174350	635	47	10164	-0.23436	0.59265
-	TNNI3	19	55141767	55192732	221	22	10164	-0.23476	0.5928
+	KLF3	4	38629169	38711508	285	52	10164	-0.23482	0.59282
+	NECAB1	8	90756693	90969402	480	24	10164	-0.23498	0.59289
+	SPDYE5	7	75458182	75514304	45	8	10164	-0.23508	0.59293
+	RABGAP1	9	122906009	123114868	321	16	10164	-0.23518	0.59296
-	GSTA4	6	52967948	53030380	139	11	10164	-0.23527	0.593
+	MAT2A	2	85503978	85555280	180	21	10164	-0.23549	0.59309
-	RPA2	1	27881524	27949797	227	13	10164	-0.23555	0.59311
+	DYRK4	12	4555072	4623888	189	29	10164	-0.23575	0.59319
+	MMP14	14	22801533	22857600	226	39	10164	-0.23602	0.59329
-	GHRL	3	10275750	10327947	185	28	10164	-0.23607	0.59331
-	OR13G1	1	247662118	247708041	143	6	10164	-0.23613	0.59333
+	NDUFS7	19	1348884	1405589	7	1	10164	-0.23631	0.5934
+	TMEM260	14	56544791	56661884	355	24	10164	-0.23632	0.59341
+	SHISA4	1	201853669	201902587	108	29	10164	-0.23647	0.59347
-	GALNT14	2	30900465	31196688	937	68	10164	-0.23666	0.59354
-	SMIM21	18	75399476	75490653	275	30	10164	-0.23666	0.59354
+	CHST13	3	126489288	126553291	186	19	10164	-0.23672	0.59356
+	LIAS	4	39424024	39487653	240	31	10164	-0.23683	0.59361
+	DUSP28	2	240525054	240574014	122	22	10164	-0.23688	0.59362
-	SLC25A29	14	100269177	100341547	177	39	10164	-0.23699	0.59367
-	DHFR	5	80616226	80689981	440	17	10164	-0.23701	0.59368
+	ABLIM3	5	149106483	149270439	480	38	10164	-0.23721	0.59375
-	KCNH4	17	42146892	42216278	111	19	10164	-0.23739	0.59382
-	BHLHE41	12	26110026	26160070	145	22	10164	-0.23758	0.5939

STRAND	SYMBOL	CHR	START	STOP	NSNPS	NPARAM	N	ZSTAT	P
+	LOC390877	19	6358250	6422404	61	1	10164	-0.23768	0.59393
-	LOC1053714	1	149843544	149895683	16	7	10164	-0.23776	0.59397
+	STAMBPL1	10	88844368	88933502	319	21	10164	-0.23785	0.594
+	CPO	2	206844383	206981064	371	26	10164	-0.23795	0.59404
-	ABCC11	16	48156910	48270177	195	5	10164	-0.2384	0.59421
-	ZNF490	19	12566106	12645809	119	6	10164	-0.23869	0.59433
+	ZFP91	11	58544114	58631550	173	23	10164	-0.23876	0.59435
+	LOC1019283	17	74564840	74635910	315	28	10164	-0.23878	0.59436
+	ZNF354C	5	179025606	179096298	190	15	10164	-0.23883	0.59438
-	SLC37A3	7	140323752	140433639	74	15	10164	-0.23919	0.59452
-	LMNTD1	12	25466082	25683594	902	62	10164	-0.23923	0.59454
+	NRROS	3	196604696	196672004	32	5	10164	-0.23929	0.59456
+	SPHAR	1	229269382	229315504	79	6	10164	-0.23947	0.59463
+	RAB11FIP3	16	390592	532485	275	26	10164	-0.23949	0.59464
-	MAP2K2	19	4080321	4159184	169	31	10164	-0.23963	0.59469
+	PSMD11	17	32409463	32493319	65	8	10164	-0.24002	0.59484
+	TMEM248	7	66886216	66968551	232	23	10164	-0.24005	0.59485
+	LOC1053763	10	3208059	3263291	166	20	10164	-0.24008	0.59487
-	PLEC	8	143905147	144011792	162	6	10164	-0.24023	0.59492
-	SIRT7	17	81901939	81953182	1	1	10164	-0.24028	0.59494
-	ZNF394	7	99483231	99535254	67	11	10164	-0.24027	0.59494
+	LOC388282	16	57775066	57826927	179	26	10164	-0.24029	0.59495
-	POC1B	12	89409718	89561262	165	13	10164	-0.24041	0.59499
-	CEMP1	16	2520035	2566408	6	3	10164	-0.24044	0.59501
+	TDRD15	2	21071652	21157393	176	13	10164	-0.2405	0.59503
-	EVI5	1	92498696	92827404	839	21	10164	-0.24056	0.59505
+	TMEM133	11	100957080	101003935	116	13	10164	-0.24055	0.59505
-	SVOP	12	108898129	109056240	432	29	10164	-0.24077	0.59513
+	NCLN	19	3150877	3219575	213	28	10164	-0.24077	0.59513
+	ANKRD2	10	97537441	97593884	128	13	10164	-0.24087	0.59517
-	BCL2L15	1	113866814	113922547	122	26	10164	-0.24097	0.59521
-	SP110	2	230157842	230260729	380	33	10164	-0.24114	0.59528
+	PCP4L1	1	161223727	161295450	187	21	10164	-0.24118	0.59529
-	APOD	3	195558702	195619205	46	5	10164	-0.24119	0.5953
-	NDUFB8	10	100513729	100564923	115	6	10164	-0.24143	0.59539
+	TTC14	3	180567130	180628347	36	16	10164	-0.2415	0.59541
+	FAM92A1	8	93665252	93739253	149	23	10164	-0.24151	0.59542
+	PNLIPRP1	10	116555415	116619175	103	19	10164	-0.24156	0.59544
-	ZNF32	10	43633859	43683878	154	15	10164	-0.24156	0.59544
+	HNF4G	8	75372681	75576843	943	47	10164	-0.24158	0.59545
+	TLX2	2	74479469	74527148	21	1	10164	-0.24176	0.59552
-	ST20-MTHFS	15	79833547	79958106	444	25	10164	-0.24231	0.59573
-	LOC1002872	7	56926027	56980473	3	2	10164	-0.2424	0.59577
+	CCRL2	3	46372166	46419523	137	17	10164	-0.24244	0.59578
-	PRSS8	16	31121433	31170830	36	7	10164	-0.24257	0.59583
+	NFKBIZ	3	101792990	101871025	131	28	10164	-0.24256	0.59583
-	KCNE3	11	74444841	74502557	148	17	10164	-0.24273	0.59589
+	BST1	4	15667950	15784190	435	38	10164	-0.24283	0.59593
+	SLC25A10	17	81677236	81731016	146	6	10164	-0.24285	0.59594
+	SIDT2	11	117144223	117207445	197	20	10164	-0.243	0.596
-	CABLES2	20	62378630	62442283	271	38	10164	-0.24312	0.59604
+	DUSP14	17	37453561	37523501	162	20	10164	-0.24318	0.59607
-	LOC1053696	12	14723577	14805662	304	29	10164	-0.24339	0.59615
-	HOMER2	15	82826946	83021161	407	47	10164	-0.2435	0.59619
+	TLK2	17	62442081	62625481	189	15	10164	-0.24361	0.59623
-	PCP2	19	7621614	7671967	210	21	10164	-0.24377	0.59629
+	TDP1	14	89919935	90054764	400	16	10164	-0.24403	0.5964
-	ZNF416	19	57561010	57614180	106	14	10164	-0.24429	0.5965
-	CHSY1	15	101165723	101286932	535	34	10164	-0.24477	0.59668
-	COG4	16	70470569	70558554	88	7	10164	-0.24534	0.5969
+	ADORA2A	22	24388597	24452360	91	9	10164	-0.24548	0.59696
-	PDE2A	11	72566140	72709453	384	27	10164	-0.24568	0.59703
-	GALNS	16	88803734	88891966	465	28	10164	-0.24576	0.59707

STRAND	SYMBOL	CHR	START	STOP	NSNPS	NPARAM	N	ZSTAT	P
-	BRAT1	7	2527810	2590758	151	16	10164	-0.24582	0.59709
-	GAB4	22	16951936	17043278	332	33	10164	-0.246	0.59716
+	UNC45B	17	35112798	35199345	304	42	10164	-0.24614	0.59721
-	PER1	17	8130470	8191405	172	28	10164	-0.24625	0.59726
-	AFAP1L2	10	114270730	114440143	347	41	10164	-0.24628	0.59727
+	NFE2L3	7	26117227	26197137	199	34	10164	-0.24634	0.59729
-	C8orf59	8	85204053	85255422	56	6	10164	-0.24647	0.59734
+	LOC1053734	2	95172503	95232682	71	24	10164	-0.2466	0.59739
-	SPATA32	17	45237274	45297112	130	24	10164	-0.24676	0.59745
-	KCNJ1	11	128828014	128902373	153	34	10164	-0.24703	0.59756
+	ZNF165	6	28045704	28099563	253	28	10164	-0.24715	0.5976
+	LGI1	10	93722809	93808159	138	22	10164	-0.24713	0.5976
-	SLC41A2	12	104792553	104993740	550	26	10164	-0.24713	0.5976
+	CRB3	19	6428777	6477221	13	1	10164	-0.24727	0.59765
+	EIF1	17	41653875	41701646	110	2	10164	-0.24732	0.59767
-	BEND4	4	42100853	42187878	220	16	10164	-0.24749	0.59773
-	ZNF597	16	3422422	3478537	262	29	10164	-0.24755	0.59776
-	KIAA1143	3	44738744	44796697	56	6	10164	-0.24765	0.5978
-	DEXI	16	10918891	10977400	104	9	10164	-0.24768	0.59781
+	IL20RB	3	136922865	137021085	202	16	10164	-0.24768	0.59781
-	CCHCR1	6	31132439	31193238	850	53	10164	-0.24799	0.59793
+	TTLL10	1	1138898	1207935	195	20	10164	-0.248	0.59793
+	CHCHD7	8	56176756	56228619	77	12	10164	-0.24802	0.59794
+	TMEM174	5	73138196	73185143	154	11	10164	-0.24812	0.59798
-	PBX4	19	19551707	19653916	319	30	10164	-0.24819	0.59801
-	TSHZ3	19	31264945	31384284	268	42	10164	-0.24835	0.59807
-	KCNK12	2	47506574	47605331	274	59	10164	-0.24838	0.59808
+	DCPS	11	126268752	126355754	236	25	10164	-0.24844	0.5981
+	COQ4	9	128287512	128344072	95	9	10164	-0.24853	0.59814
-	RPL29	3	51983628	52030942	98	18	10164	-0.24926	0.59842
+	STT3B	3	31497501	31647622	267	24	10164	-0.24936	0.59846
-	JUND	19	18269694	18316656	148	21	10164	-0.24958	0.59854
+	UFL1	6	96486826	96565276	118	15	10164	-0.24975	0.59861
+	GHR	5	42388775	42731878	896	46	10164	-0.24995	0.59869
+	FOXE3	1	47381072	47428052	137	24	10164	-0.25011	0.59875
-	NDUFC1	4	139279917	139337551	130	11	10164	-0.2501	0.59875
-	CGA	6	87075498	87130147	191	22	10164	-0.25059	0.59893
-	KMT2C	7	152124925	152471638	382	15	10164	-0.25063	0.59895
-	PGR	11	101019624	101165668	443	21	10164	-0.25076	0.599
+	C6orf99	6	158834855	158928005	361	39	10164	-0.25077	0.59901
+	IRX6	16	55288962	55340760	206	22	10164	-0.25085	0.59904
+	OR4A16	11	55288661	55354225	234	18	10164	-0.251	0.59909
+	TELO2	16	1458344	1520459	309	41	10164	-0.2513	0.59921
+	WRNIP1	6	2730341	2795745	212	26	10164	-0.25152	0.59929
-	CHKA	11	68042859	68156391	176	19	10164	-0.25153	0.5993
-	ECSIT	19	11495916	11564172	153	11	10164	-0.25162	0.59933
+	EFNB3	17	7670202	7721375	129	22	10164	-0.25215	0.59954
+	ZNF324	19	58432045	58483578	95	11	10164	-0.25241	0.59964
-	TUBB8	10	36638	111455	125	12	10164	-0.25248	0.59966
-	DNAJC18	5	139400203	139474525	76	12	10164	-0.25273	0.59976
+	TRIM6	11	5561101	5622958	355	39	10164	-0.2529	0.59983
+	ENPEP	4	110441073	110573337	290	39	10164	-0.25299	0.59986
+	ZYG11B	1	52691459	52837342	440	23	10164	-0.25298	0.59986
-	MTHFS	15	79833547	79932285	380	23	10164	-0.25314	0.59992
+	CTTN	11	70363506	70446584	150	15	10164	-0.25349	0.60005
+	STK19	6	31936175	31991446	202	50	10164	-0.25378	0.60017
+	RNF144B	6	18242515	18478874	795	37	10164	-0.2538	0.60017
+	ADCK3	1	226905237	226997545	459	46	10164	-0.25388	0.60021
+	EIF2S1	14	67325317	67396516	234	18	10164	-0.25399	0.60025
+	LELP1	1	153168430	153215125	109	19	10164	-0.25403	0.60027
-	CUL2	10	34998551	35161899	381	10	10164	-0.25421	0.60033
+	SLCO2B1	11	75115987	75216400	311	27	10164	-0.2542	0.60033
+	SPIRE2	16	89782477	89881319	475	24	10164	-0.2542	0.60033

STRAND	SYMBOL	CHR	START	STOP	NSNPS	NPARAM	N	ZSTAT	P
+	LSM6	4	146140683	146200061	107	10	10164	-0.25429	0.60036
+	SDC2	8	96458351	96621809	468	65	10164	-0.25439	0.6004
+	SORBS3	8	22509990	22585495	34	9	10164	-0.25459	0.60048
-	KIAA0196	8	125014260	125126819	352	15	10164	-0.25468	0.60051
-	MBD3	19	1566671	1627761	63	9	10164	-0.25472	0.60053
+	ZNF584	19	58372873	58428327	164	15	10164	-0.25474	0.60054
-	SLC35E1	19	16539837	16607382	81	5	10164	-0.25486	0.60058
+	EME2	16	1738228	1786238	128	22	10164	-0.25494	0.60062
-	UBE2M	19	58545712	58593976	54	8	10164	-0.25501	0.60064
-	PDE6G	17	81640459	81692054	214	7	10164	-0.25512	0.60069
+	ASNSD1	2	189626399	189680831	139	20	10164	-0.25529	0.60075
+	PRPF31	19	54080376	54141719	80	10	10164	-0.25547	0.60082
-	NGLY1	3	25708944	25825039	160	13	10164	-0.25554	0.60085
+	TBCB	19	36079986	36135948	155	21	10164	-0.25569	0.6009
-	FAM205A	9	34713053	34764538	156	19	10164	-0.25618	0.60109
-	ATP6V1D	14	67327864	67395003	221	18	10164	-0.2567	0.6013
+	GCNT1	9	76406666	76517416	394	32	10164	-0.25719	0.60149
+	TRNP1	1	26958667	27010886	42	11	10164	-0.2573	0.60152
+	LIPN	10	88722226	88788891	294	22	10164	-0.25738	0.60156
+	CPT2	1	53161429	53224197	226	47	10164	-0.25743	0.60158
-	SOS1	2	38971549	39159744	410	20	10164	-0.25744	0.60158
-	LCORL	4	17831199	18056860	472	15	10164	-0.25743	0.60158
+	CKLF	16	66517563	66576287	117	6	10164	-0.25747	0.60159
+	ST6GALNAC	1	76832441	77079167	414	32	10164	-0.25748	0.6016
+	PXMP2	12	132652606	132714991	141	10	10164	-0.25752	0.60161
-	F2RL2	5	76605482	76658434	318	24	10164	-0.25767	0.60167
-	DDX41	5	177501577	177551966	65	6	10164	-0.25767	0.60167
+	CIAO1	2	96231146	96284179	106	28	10164	-0.25769	0.60168
-	C17orf47	17	58530681	58579372	85	13	10164	-0.25774	0.60169
-	LOC1053724	20	1017720	1153497	286	44	10164	-0.25785	0.60174
+	PEX7	6	136787220	136923934	219	11	10164	-0.25806	0.60182
+	MUCL1	12	54795357	54868393	120	19	10164	-0.25815	0.60185
+	FAF2	5	176413306	176520074	291	40	10164	-0.25816	0.60186
-	PAIP1	5	43516267	43592419	137	11	10164	-0.25819	0.60187
+	ZNF410	14	73851615	73942288	393	16	10164	-0.25821	0.60188
+	TNNI3K	1	74200387	74554432	462	37	10164	-0.25849	0.60198
-	PABPC4L	4	134186333	134236748	118	7	10164	-0.25848	0.60198
-	TAS1R2	1	18829599	18894661	180	26	10164	-0.25875	0.60209
+	TMEM247	2	46444497	46494579	187	17	10164	-0.25884	0.60212
-	TMEM50B	21	33422486	33515009	257	18	10164	-0.25907	0.60221
-	LARP6	15	70821524	70889265	125	8	10164	-0.25913	0.60223
-	PARK2	6	161337417	162762802	5622	283	10164	-0.25918	0.60225
-	ALG2	9	99206425	99256964	30	5	10164	-0.25927	0.60229
-	TRIP11	14	91955991	92075140	457	18	10164	-0.25952	0.60238
+	CCND1	11	69606105	69664474	179	30	10164	-0.25964	0.60243
+	RNASE7	14	21007226	21054233	107	30	10164	-0.25965	0.60243
+	LSM8	7	118149032	118214039	256	16	10164	-0.25973	0.60247
+	DDI1	11	104001580	104049194	153	22	10164	-0.25977	0.60248
-	PTRHD1	2	24780267	24828382	131	14	10164	-0.25995	0.60255
-	SLC16A4	1	110352851	110426409	178	20	10164	-0.26011	0.60261
+	KLHL29	2	23350427	23718613	994	90	10164	-0.26014	0.60262
-	UACA	15	70644554	70798511	194	22	10164	-0.26023	0.60266
-	ACP6	1	147619286	147705512	192	25	10164	-0.26041	0.60273
-	RBM22	5	150680790	150736107	107	8	10164	-0.26046	0.60275
-	DPH6	15	35294893	35581222	589	28	10164	-0.26075	0.60286
-	BCL9L	11	118886140	118960618	165	30	10164	-0.26074	0.60286
-	OXLD1	17	81655036	81701637	192	6	10164	-0.26089	0.60291
+	SMTNL2	17	4548981	4618319	124	21	10164	-0.26096	0.60294
-	KIAA0430	16	15584369	15678166	172	23	10164	-0.26115	0.60301
-	ONECUT1	15	52746963	52825277	193	33	10164	-0.26118	0.60303
-	BARX1	9	93941627	93990326	146	20	10164	-0.26128	0.60306
-	ADRA2B	2	96102875	96151245	110	26	10164	-0.26139	0.6031
+	LOC1053724	19	56828396	56904601	83	10	10164	-0.26149	0.60314

STRAND	SYMBOL	CHR	START	STOP	NSNPS	NPARAM	N	ZSTAT	P
-	ICE2	15	60409609	60514160	152	11	10164	-0.26154	0.60316
-	MRFAP1L1	4	6697701	6744879	118	21	10164	-0.26158	0.60318
-	SMEK2	2	55537292	55652725	345	29	10164	-0.26166	0.60321
+	WEE2	7	141673353	141741271	130	17	10164	-0.26185	0.60328
+	VRK2	2	57872651	58169920	782	51	10164	-0.26186	0.60329
+	REC8	14	24136853	24190408	83	16	10164	-0.26188	0.60329
-	REEP4	8	22128020	22176935	37	8	10164	-0.26231	0.60346
+	SLC6A11	3	10781200	10951471	625	58	10164	-0.2624	0.60349
+	FAM179A	2	28946298	29062230	476	43	10164	-0.26262	0.60358
+	G6PC3	17	44035730	44086344	66	13	10164	-0.26272	0.60362
-	PSPN	19	6365294	6410849	51	1	10164	-0.26277	0.60364
+	PPHLN1	12	42291137	42460965	551	25	10164	-0.26305	0.60374
-	UBTD2	5	172199645	172318791	325	14	10164	-0.26333	0.60385
-	TLE1	9	81573683	81724535	359	60	10164	-0.26342	0.60389
-	XRCC3	14	103687608	103750486	230	24	10164	-0.26357	0.60394
+	PRR12	19	49556655	49636439	224	16	10164	-0.26385	0.60405
+	SLC17A8	12	100322079	100432059	150	23	10164	-0.26386	0.60405
+	SEPT2	2	241280187	241364027	240	26	10164	-0.26428	0.60422
+	SMOX	20	4113779	4197747	412	50	10164	-0.26449	0.6043
+	NDOR1	9	137170667	137229361	130	18	10164	-0.26472	0.60439
+	GOLGA4	3	37208191	37376879	217	14	10164	-0.26487	0.60444
+	C11orf73	11	86267211	86355943	308	21	10164	-0.26489	0.60445
-	RADIL	7	4789109	4918704	341	33	10164	-0.26551	0.60469
+	HGS	17	81648932	81712121	250	8	10164	-0.26567	0.60475
+	ISLR2	15	74093203	74151841	58	23	10164	-0.26569	0.60476
+	LANCL2	7	55330448	55443742	444	22	10164	-0.26584	0.60482
+	KCNJ5	11	128856418	128931163	207	40	10164	-0.26605	0.6049
+	WNT3A	1	227972022	228076711	421	33	10164	-0.2662	0.60496
+	PROSER2	10	11788349	11882277	225	42	10164	-0.26639	0.60503
+	UGT8	4	114563403	114688225	165	15	10164	-0.26662	0.60512
+	SNAPC1	14	61727357	61806428	272	29	10164	-0.26677	0.60518
+	C7orf65	7	47620244	47671648	188	23	10164	-0.26683	0.6052
+	RBMXL2	11	7053934	7101148	134	10	10164	-0.26691	0.60523
-	NOTCH4	6	32184843	32259067	691	70	10164	-0.267	0.60527
+	EIF4E2	2	232515057	232593645	267	27	10164	-0.26704	0.60528
-	MFSD7	4	664690	724468	109	23	10164	-0.26711	0.60531
-	MRPL10	17	47813272	47866541	194	29	10164	-0.26715	0.60532
-	GTF2F1	19	6369569	6428280	39	1	10164	-0.26726	0.60536
-	CCT8L2	22	16580758	16627810	54	10	10164	-0.26727	0.60537
-	NBPF9	1	149044033	149125861	1	1	10164	-0.26731	0.60539
+	HSPE1	2	197464997	197513463	47	6	10164	-0.26745	0.60544
+	LOC1005061	11	76346325	76418132	216	36	10164	-0.26772	0.60554
-	RPL18	19	48605327	48654418	92	13	10164	-0.2678	0.60557
+	ZSWIM4	19	13760434	13842254	192	22	10164	-0.26786	0.6056
-	OR2A4	6	131689644	131736401	65	5	10164	-0.26834	0.60578
+	ZNF225	19	44077076	44144816	198	19	10164	-0.26859	0.60588
-	MFGE8	15	88888683	88948454	214	27	10164	-0.26868	0.60591
-	TNFRSF4	1	1201326	1249638	162	8	10164	-0.26874	0.60593
+	CKLF-CMTM	16	66517563	66589135	150	7	10164	-0.2688	0.60596
-	SMEK1	14	91447481	91545554	238	19	10164	-0.26884	0.60597
-	PTH2	19	49412414	49461263	107	17	10164	-0.26927	0.60614
+	RASSF10	11	12974423	13022106	157	13	10164	-0.26948	0.60622
-	DLEU7	13	50685550	50878933	604	48	10164	-0.26966	0.60629
-	GPR18	13	99244713	99297448	116	23	10164	-0.26972	0.60631
-	STARD6	18	54314592	54392890	319	19	10164	-0.26988	0.60638
+	MLIP	6	53975979	54276280	787	53	10164	-0.27004	0.60643
-	SYNPO2L	10	73634881	73691105	106	13	10164	-0.27008	0.60645
+	FGFR1OP2	12	26903372	26976648	168	35	10164	-0.27009	0.60646
-	ANKS6	9	98722009	98831531	252	21	10164	-0.27009	0.60646
+	RBP7	1	9962197	10026020	94	15	10164	-0.27082	0.60674
-	TWF2	3	52218610	52274167	110	18	10164	-0.27091	0.60677
+	GRAPL	17	19092469	19168835	1	1	10164	-0.27092	0.60677
-	SGK1	6	134159246	134353112	356	37	10164	-0.27108	0.60684

STRAND	SYMBOL	CHR	START	STOP	NSNPS	NPARAM	N	ZSTAT	P
-	IMPAD1	8	56947929	57028871	148	20	10164	-0.27113	0.60685
-	TMEM230	20	5089838	5148087	92	16	10164	-0.27124	0.6069
-	GABPB1	15	50267192	50390408	408	27	10164	-0.27152	0.607
-	ZNF148	3	125215669	125410354	350	15	10164	-0.27165	0.60705
-	PHB	17	49394048	49449905	117	24	10164	-0.27186	0.60714
+	E2F3	6	20366906	20503714	371	58	10164	-0.27191	0.60715
+	FAM120A	9	93416696	93576115	456	31	10164	-0.27219	0.60726
-	SYDE2	1	85147673	85236704	221	22	10164	-0.27219	0.60726
+	CDCA8	1	37657401	37719719	169	20	10164	-0.27247	0.60737
+	PIK3CA	3	179113114	179250084	311	14	10164	-0.27273	0.60747
-	SLC24A2	9	19497452	19824695	716	56	10164	-0.27306	0.60759
+	PLCG1	20	41102519	41185721	230	21	10164	-0.27306	0.6076
+	LOC1053767	16	84256444	84304838	310	42	10164	-0.27312	0.60762
+	TRA	14	21586904	22562132	3401	179	10164	-0.27328	0.60768
-	VPS29	12	110481523	110537142	60	7	10164	-0.27333	0.6077
-	ADAM30	1	119883533	119931524	83	11	10164	-0.2736	0.60781
+	FAHD1	16	1792224	1850207	594	19	10164	-0.27362	0.60781
+	ADGRG1	16	57584535	57675039	308	44	10164	-0.27367	0.60783
-	COX7A1	19	36140922	36187869	164	31	10164	-0.27378	0.60787
-	SGTB	5	65655928	65757821	257	23	10164	-0.27376	0.60787
+	PPM1E	17	58720869	58995179	438	26	10164	-0.27448	0.60814
-	LOC1010603	2	939634	989998	210	11	10164	-0.27452	0.60816
+	LIG3	17	34945498	35018448	112	31	10164	-0.27491	0.60831
-	EFS	14	23346400	23400633	121	23	10164	-0.27494	0.60832
-	NT5C2	10	103078017	103228306	479	40	10164	-0.27497	0.60833
+	RELB	19	44966449	45048198	181	28	10164	-0.27501	0.60834
+	C7orf26	7	6554368	6618726	144	14	10164	-0.27509	0.60838
-	SDR9C7	12	56913154	56969405	167	18	10164	-0.27538	0.60849
+	BCL2L14	12	12014880	12109695	480	78	10164	-0.27553	0.60855
-	IFT57	3	108150812	108257570	444	12	10164	-0.27563	0.60858
-	MKRN2OS	3	12529717	12596056	176	20	10164	-0.27566	0.6086
-	C5orf46	5	147875908	147941589	246	30	10164	-0.27585	0.60867
-	CST1	20	23737553	23785937	159	18	10164	-0.27588	0.60868
+	UBAC2	13	99165425	99396499	678	34	10164	-0.27606	0.60875
-	TAS2R38	7	141962631	142008773	9	4	10164	-0.27626	0.60883
-	CCDC88C	14	91261323	91452844	481	60	10164	-0.27628	0.60884
-	RPL22	1	6175020	6234619	111	14	10164	-0.27641	0.60888
+	SMPDL3A	6	122753456	122819720	204	45	10164	-0.27686	0.60906
+	METRN	16	680173	727480	137	19	10164	-0.27689	0.60907
-	GPAM	10	112139864	112250395	181	22	10164	-0.27703	0.60912
-	RFX8	2	101387344	101510096	512	30	10164	-0.27704	0.60912
+	RPL7L1	6	42844917	42899889	117	16	10164	-0.27721	0.60919
-	ZNF836	19	52144612	52206667	177	21	10164	-0.27724	0.6092
+	VCAN	5	83436674	83592303	512	71	10164	-0.27725	0.60921
-	LIMA1	12	50165780	50318570	253	19	10164	-0.27734	0.60924
+	SUB1	5	32550499	32614079	207	23	10164	-0.27743	0.60928
-	TNNC1	3	52441091	52489041	95	9	10164	-0.27761	0.60934
+	TTC23L	5	34804164	34909650	276	39	10164	-0.27761	0.60934
-	OR1N1	9	122516358	122562293	243	21	10164	-0.27773	0.60939
+	DPCR1	6	30906000	30964221	339	30	10164	-0.27779	0.60941
+	BCAS4	20	50759894	50887854	428	48	10164	-0.27789	0.60945
-	TLDC1	16	84466360	84589045	804	75	10164	-0.27789	0.60945
+	WDR17	4	176030834	176192828	465	21	10164	-0.27805	0.60951
+	UBR3	2	169792470	170094131	836	25	10164	-0.27821	0.60957
+	RGS7BP	5	64470539	64624657	304	27	10164	-0.27824	0.60959
-	OR10G3	14	21559803	21605744	231	31	10164	-0.27828	0.6096
+	PAK1IP1	6	10655865	10719782	117	4	10164	-0.27832	0.60962
-	ELOVL5	6	53257398	53384179	328	16	10164	-0.27845	0.60967
+	SON	21	33508038	33587514	102	15	10164	-0.27856	0.60971
+	MFAP3	5	154003959	154067454	206	15	10164	-0.27863	0.60974
-	NHEJ1	2	219065324	219195865	238	16	10164	-0.27889	0.60983
+	GFY	19	49386318	49438977	116	13	10164	-0.27907	0.6099
+	AIFM3	22	20930130	20991360	92	14	10164	-0.27913	0.60993

STRAND	SYMBOL	CHR	START	STOP	NSNPS	NPARAM	N	ZSTAT	P
-	A1BG	19	58336806	58388499	85	9	10164	-0.2796	0.61011
-	QRICH2	17	76264049	76343690	268	24	10164	-0.27971	0.61015
+	NKD2	5	973820	1048812	243	27	10164	-0.27977	0.61017
+	FAM131A	3	184300929	184356275	93	23	10164	-0.27982	0.61019
+	IL19	1	206763870	206852981	292	50	10164	-0.28001	0.61026
-	PPM1H	12	62633982	62969885	662	92	10164	-0.28019	0.61033
+	PHF11	13	49460610	49538987	295	20	10164	-0.28026	0.61036
-	PLTP	20	45888620	45947364	164	35	10164	-0.2804	0.61042
+	CREBRF	5	173021349	173149293	229	16	10164	-0.28052	0.61046
+	RCSD1	1	167595237	167718696	360	49	10164	-0.28086	0.61059
+	LOC1053703	14	22521953	22569793	161	30	10164	-0.281	0.61064
+	GPR146	7	1009573	1069269	272	32	10164	-0.2811	0.61068
-	SERINC5	5	80101227	80291082	576	61	10164	-0.2812	0.61072
-	DUXB	16	75683729	75736418	168	16	10164	-0.28123	0.61073
-	ORAOV1	11	69643420	69710397	166	23	10164	-0.28131	0.61076
-	MDN1	6	89632775	89862961	504	31	10164	-0.28134	0.61078
-	TXNRD3NB	3	126561779	126643555	158	13	10164	-0.28153	0.61085
+	SHISA6	17	11206417	11574063	887	67	10164	-0.28174	0.61093
-	CASZ1	1	10626604	10831676	593	100	10164	-0.28207	0.61106
+	ZNF236	18	76787561	76980732	628	30	10164	-0.28223	0.61112
+	RCCD1	15	90919695	90973543	150	18	10164	-0.28244	0.6112
-	SLC12A4	16	67933474	68003694	110	14	10164	-0.28246	0.61121
-	FAM217A	6	4058352	4122344	170	10	10164	-0.2825	0.61122
+	MEI4	6	77615233	77937045	1310	43	10164	-0.28256	0.61124
+	PPP2R5C	14	101725573	101937992	632	30	10164	-0.28267	0.61129
+	LRRC48	17	17937780	18026889	242	25	10164	-0.2827	0.6113
+	CNGA3	2	98311155	98409154	240	36	10164	-0.28273	0.61131
-	KRT9	17	41555841	41607058	98	3	10164	-0.28287	0.61136
-	STAG1	3	136326234	136787403	765	35	10164	-0.28288	0.61136
-	NEUROG2	4	112503516	112551172	102	15	10164	-0.28322	0.6115
+	MAP2K7	19	7868780	7924483	138	26	10164	-0.28359	0.61164
+	C1orf112	1	169760005	169863088	444	25	10164	-0.28392	0.61177
+	RBM20	10	110609397	110849471	850	65	10164	-0.28409	0.61183
+	RIPPLY3	21	36971156	37029659	256	20	10164	-0.28436	0.61193
+	DHRS4L1	14	23972009	24061377	228	27	10164	-0.28453	0.612
+	RFC5	12	117981701	118043564	140	22	10164	-0.28469	0.61206
+	OR5K3	3	98355666	98401631	170	24	10164	-0.28474	0.61208
-	KRI1	19	10543085	10601031	245	27	10164	-0.2849	0.61214
-	RAD54B	8	94361960	94510115	447	21	10164	-0.28492	0.61215
-	CYGB	17	76517348	76592597	37	7	10164	-0.28496	0.61216
+	MRPL42	12	93432490	93513772	210	15	10164	-0.28499	0.61217
-	ADCY6	12	48756192	48824037	110	12	10164	-0.2851	0.61222
+	CCT2	12	69550428	69611577	143	15	10164	-0.2851	0.61222
-	SLC16A8	22	38068137	38118163	144	28	10164	-0.28515	0.61223
-	FAM92B	16	85088352	85147508	254	46	10164	-0.28518	0.61225
+	RHOJ	14	63169362	63303512	407	36	10164	-0.28538	0.61232
-	LIF	22	30230447	30292923	156	55	10164	-0.28574	0.61246
+	NAA15	4	139266467	139400781	261	16	10164	-0.28574	0.61246
+	DUSP12	1	161714768	161767164	185	23	10164	-0.28591	0.61253
+	GREB1	2	11499116	11652788	234	28	10164	-0.28608	0.61259
-	MRPL28	16	357384	405569	247	31	10164	-0.28641	0.61272
+	F10	13	113087799	113159529	176	35	10164	-0.28654	0.61277
-	TRPC7	5	136202765	136400476	505	26	10164	-0.28657	0.61278
+	CDH1	16	68702292	68845542	469	28	10164	-0.28664	0.6128
-	KCNK16	6	39304698	39357985	220	33	10164	-0.28663	0.6128
-	CDCA7L	7	21890899	21980924	417	57	10164	-0.28664	0.61281
-	ASTL	2	96112817	96178077	158	39	10164	-0.28676	0.61285
-	HAO1	20	7872984	7975446	268	27	10164	-0.28718	0.61301
+	GPR21	9	122995013	123049101	82	8	10164	-0.28736	0.61308
-	FBXO2	1	11638361	11689831	137	26	10164	-0.28745	0.61311
-	PPT1	1	40062710	40132470	273	26	10164	-0.28745	0.61312
+	TDRD3	13	60361457	60583879	414	25	10164	-0.28747	0.61312
+	C19orf53	19	13739443	13788773	147	15	10164	-0.2875	0.61313

STRAND	SYMBOL	CHR	START	STOP	NSNPS	NPARAM	N	ZSTAT	P
-	SLC38A9	5	55615845	55747335	683	16	10164	-0.28769	0.61321
-	C10orf88	10	122920903	122989403	148	17	10164	-0.28789	0.61329
+	IQCK	16	19681456	19867585	319	17	10164	-0.28798	0.61332
+	DES	2	219383377	219436739	77	8	10164	-0.28838	0.61347
+	ATP1A4	1	160116562	160196977	282	54	10164	-0.2884	0.61348
+	MVB12B	9	126291844	126517041	672	42	10164	-0.2884	0.61348
-	PKLR	1	155279293	155343709	108	16	10164	-0.28856	0.61354
+	AKAP1	17	57050192	57131349	133	15	10164	-0.28855	0.61354
-	SLC9B1	4	102875048	103054739	332	10	10164	-0.28887	0.61366
-	GTF2IRD2	7	74786144	74886792	16	3	10164	-0.28893	0.61368
-	C11orf85	11	64928230	65007112	169	25	10164	-0.28919	0.61378
-	ABCB6	2	219199766	219253990	89	9	10164	-0.28923	0.6138
-	SGPP1	14	63674216	63763095	163	12	10164	-0.28935	0.61384
-	LOC1005055	10	31352778	31399077	67	17	10164	-0.28946	0.61389
+	MRPL4	19	10216964	10270060	126	22	10164	-0.29016	0.61415
+	RRAGA	9	19014374	19061025	178	16	10164	-0.29081	0.6144
+	TAS1R1	1	6520278	6589757	260	17	10164	-0.2909	0.61443
+	UPP2	2	157960179	158146154	408	29	10164	-0.29114	0.61453
-	THSD1	13	52367167	52441494	169	8	10164	-0.29123	0.61456
-	COL4A3BP	5	75358486	75546981	539	19	10164	-0.29136	0.61461
-	RANBP6	9	6001019	6050640	135	22	10164	-0.29143	0.61464
-	TP53BP1	15	43393064	43545728	336	15	10164	-0.29147	0.61465
-	GOLM1	9	86016141	86135201	338	19	10164	-0.29165	0.61472
-	SEMA6A	5	116433555	116609926	425	55	10164	-0.29168	0.61473
+	ATXN7L3B	12	74502771	74551452	168	16	10164	-0.29174	0.61476
+	CCDC126	7	23562379	23654708	202	28	10164	-0.2918	0.61478
+	NME4	16	361755	410754	243	31	10164	-0.29193	0.61483
-	RPL22L1	3	170854875	170905256	96	14	10164	-0.29224	0.61495
+	SRSF6	20	43422864	43473605	165	27	10164	-0.29255	0.61507
+	YIPF4	2	32242889	32317364	133	24	10164	-0.29266	0.61511
+	OR1I1	19	15052066	15098133	110	16	10164	-0.29266	0.61511
-	PLAGL1	6	143930300	144099599	542	54	10164	-0.29272	0.61513
-	EDIL3	5	83930595	84419867	943	72	10164	-0.29306	0.61526
-	CDK11A	1	1692730	1759352	2	1	10164	-0.29308	0.61527
+	SLC31A2	9	113115958	113174142	176	28	10164	-0.29323	0.61533
-	ZDHHC21	9	14545501	14728482	655	50	10164	-0.29333	0.61537
-	LOC1027246	5	178681534	178743586	231	20	10164	-0.29344	0.61541
+	MAPK4	18	50525114	50741826	653	65	10164	-0.29358	0.61546
+	MLST8	16	2170177	2219417	97	15	10164	-0.29371	0.61551
-	PVRL1	11	119628098	119763725	385	33	10164	-0.2939	0.61558
-	FAM114A2	5	153981599	154073937	284	17	10164	-0.29446	0.6158
-	MSC	8	71831542	71879496	135	18	10164	-0.29462	0.61586
+	BIVM	13	102764049	102851538	286	33	10164	-0.29466	0.61587
-	PTGIS	20	49493874	49603170	159	31	10164	-0.29468	0.61588
-	WNT3	17	46752506	46853760	193	30	10164	-0.29473	0.6159
-	LYSMD3	5	90505626	90564584	142	20	10164	-0.29502	0.61601
+	MSH3	5	80619648	80886815	988	44	10164	-0.29521	0.61608
-	PTGES3	12	56653341	56723408	106	11	10164	-0.29521	0.61608
-	TAF10	11	6600817	6647244	118	19	10164	-0.29542	0.61616
+	TRIM48	11	55227182	55281119	280	19	10164	-0.29548	0.61618
-	CD300E	17	74599883	74658758	234	34	10164	-0.29545	0.61618
+	APELA	4	164841988	164907527	142	27	10164	-0.29547	0.61618
+	PDX1	13	27884994	27936314	193	15	10164	-0.29553	0.6162
-	LOC732265	15	74078446	74135467	42	19	10164	-0.29554	0.61621
-	PRX	19	40383764	40449718	130	17	10164	-0.29562	0.61624
+	NSUN4	1	46305177	46375152	415	9	10164	-0.2957	0.61627
-	UBE3D	6	82882398	83100841	686	38	10164	-0.29578	0.6163
-	IPO8	12	30618981	30730995	288	14	10164	-0.29587	0.61634
-	GMIP	19	19619473	19678667	163	28	10164	-0.29592	0.61635
-	OR10H1	19	15796957	15843759	196	14	10164	-0.29611	0.61643
+	ARHGAP5	14	32041458	32169728	155	45	10164	-0.29615	0.61644
-	CYP39A1	6	46539580	46687847	330	27	10164	-0.29627	0.61649
+	NUTM2A	10	87188041	87245907	50	11	10164	-0.29643	0.61655

STRAND	SYMBOL	CHR	START	STOP	NSNPS	NPARAM	N	ZSTAT	P
-	GNE	9	36204441	36312056	263	20	10164	-0.29645	0.61656
+	ANG	14	20649177	20704186	106	18	10164	-0.29672	0.61666
-	OR5B12	11	58429120	58475173	150	14	10164	-0.29672	0.61666
-	CCNI	4	77037155	77111013	111	16	10164	-0.29736	0.61691
+	RAD9B	12	110467200	110542086	88	8	10164	-0.29737	0.61691
-	XPO5	6	43512330	43611075	131	12	10164	-0.2974	0.61692
-	CRIP3	6	43289741	43344614	102	18	10164	-0.29752	0.61696
-	SLC22A2	6	160206762	160293931	482	37	10164	-0.29777	0.61706
+	VKORC1L1	7	65801336	65969563	412	26	10164	-0.29779	0.61707
-	CHMP2A	19	58541566	58590128	57	9	10164	-0.29785	0.61709
-	PIK3R5	17	8868916	9000712	175	24	10164	-0.29786	0.6171
+	AHRR	5	269176	448291	767	58	10164	-0.29845	0.61732
+	PLD4	14	104889821	104947788	214	25	10164	-0.2985	0.61734
+	GOLGA8J	15	30047375	30106701	17	3	10164	-0.29855	0.61736
+	DNAH6	2	84424540	84829589	827	25	10164	-0.29865	0.6174
-	RNF103-CHM	2	86493430	86756122	369	20	10164	-0.29908	0.61756
-	CTNNA3	10	65902518	67798610	7513	205	10164	-0.29917	0.61759
-	OR51B5	11	5330479	5540652	1382	54	10164	-0.29925	0.61762
+	SULT1C2	2	108253639	108320569	154	15	10164	-0.29929	0.61764
-	MTF1	1	37799567	37894624	112	13	10164	-0.29948	0.61771
+	URI1	19	29888644	30026612	251	19	10164	-0.29997	0.6179
+	SKIV2L2	5	55272748	55435581	378	14	10164	-0.30012	0.61796
+	CTAGE5	14	39230272	39361193	396	26	10164	-0.30017	0.61798
-	DEFB128	20	177853	224681	104	10	10164	-0.30019	0.61798
-	KCNIP2	10	101815974	101878920	107	26	10164	-0.3002	0.61799
+	CABIN1	22	23976192	24188628	307	27	10164	-0.30022	0.618
-	BTNL10	1	228498027	228549017	206	20	10164	-0.30026	0.61801
-	SCRN2	17	47827681	47876333	168	34	10164	-0.30049	0.6181
+	GSTM5	1	109676761	109728268	44	5	10164	-0.30062	0.61815
+	LOC338667	11	125912424	125967040	167	23	10164	-0.30068	0.61817
+	NRF1	7	129576702	129767082	488	28	10164	-0.3008	0.61822
+	C6orf89	6	36836846	36938964	435	30	10164	-0.30106	0.61831
-	LOC1053790	5	73104872	73155456	158	15	10164	-0.30114	0.61835
+	ZNF138	7	64759388	64863800	398	20	10164	-0.30141	0.61845
+	HLA-A	6	29907470	29955884	1309	39	10164	-0.30155	0.6185
-	C16orf74	16	85697510	85786083	337	40	10164	-0.3017	0.61856
+	MLF1	3	158536163	158616460	257	21	10164	-0.30181	0.6186
-	PRKD3	2	37240502	37359772	324	19	10164	-0.302	0.61867
+	MPL	1	43302418	43364464	71	13	10164	-0.3021	0.61871
-	USP54	10	73487538	73626342	202	10	10164	-0.30227	0.61878
+	C2orf48	2	10106382	10221730	338	29	10164	-0.30243	0.61884
+	IL7R	5	35821875	35889603	307	26	10164	-0.30265	0.61892
-	ZNF77	19	2923218	2980279	77	14	10164	-0.3028	0.61898
-	SMAD9	13	36805914	36955721	198	25	10164	-0.3029	0.61902
-	ST20	15	79888840	79958754	281	24	10164	-0.3031	0.61909
+	LOC1053793	8	30697477	30744324	228	17	10164	-0.30308	0.61909
+	SPTAN1	9	128517558	128643665	219	16	10164	-0.30314	0.61911
+	PAFAH1B2	11	117109284	117188173	245	24	10164	-0.30316	0.61912
-	SAMD3	6	130123203	130400425	989	40	10164	-0.30341	0.61921
-	GP6	19	55003705	55073264	323	20	10164	-0.30351	0.61925
+	PRSS21	16	2782163	2831722	23	2	10164	-0.3037	0.61932
+	SCGB2A2	11	62234933	62283160	177	27	10164	-0.30376	0.61935
-	EPYC	12	90953679	91042906	343	13	10164	-0.30385	0.61938
-	LOC1027237	7	836962	883411	148	12	10164	-0.30394	0.61941
+	HIGD1B	17	44809815	44860480	135	20	10164	-0.30395	0.61942
-	MYO1E	15	59125969	59407872	728	69	10164	-0.30398	0.61943
-	OR2D2	11	6881574	6927500	198	12	10164	-0.304	0.61944
-	ZNF565	19	36172060	36250084	259	23	10164	-0.30409	0.61947
-	OR1S2	11	58193204	58239181	201	23	10164	-0.30411	0.61948
-	ZNF688	16	30559677	30607495	12	3	10164	-0.30416	0.6195
-	RPS13	11	17064392	17112673	102	16	10164	-0.30424	0.61953
+	TANC1	2	158933634	159242663	906	55	10164	-0.30424	0.61953
-	FGF23	12	4358227	4414728	168	26	10164	-0.30428	0.61954

STRAND	SYMBOL	CHR	START	STOP	NSNPS	NPARAM	N	ZSTAT	P
-	MYL2	12	110900819	110955600	113	11	10164	-0.30433	0.61956
+	RAD51AP1	12	4503746	4570047	97	13	10164	-0.30463	0.61968
+	RNASE4	14	20649213	20710602	118	21	10164	-0.30498	0.61981
+	UBE2K	4	39663044	39792792	389	24	10164	-0.30539	0.61997
+	TMEM222	1	27287145	27346400	54	8	10164	-0.30556	0.62003
+	IBSP	4	87764550	87822449	199	37	10164	-0.30562	0.62005
-	TMEM8A	16	360776	416950	259	32	10164	-0.30579	0.62012
+	UBL5	19	9792880	9840121	77	12	10164	-0.3059	0.62016
-	LOC1009967	12	131653774	131700416	123	10	10164	-0.30616	0.62026
+	CUL4A	13	113173193	113275078	221	14	10164	-0.30624	0.62029
-	CDCA5	11	65051009	65119150	149	25	10164	-0.30623	0.62029
-	TMEM209	7	130154713	130241197	122	10	10164	-0.30641	0.62035
+	CYP2B6	19	40956229	41028398	184	17	10164	-0.30647	0.62038
+	ACTRT2	1	2986482	3032903	46	3	10164	-0.30653	0.6204
-	SPRED2	2	65297435	65467637	398	26	10164	-0.30694	0.62055
+	TMEM63B	6	44091757	44165519	19	7	10164	-0.30711	0.62062
+	CNN1	19	11503764	11560323	119	10	10164	-0.30719	0.62065
+	IPO9	1	201794160	201894294	219	40	10164	-0.30728	0.62069
+	TAAR8	6	132517693	132563721	165	19	10164	-0.30782	0.62089
-	ANKRD1	10	90902100	90956275	162	17	10164	-0.30809	0.62099
-	ASUN	12	26895057	26973326	183	35	10164	-0.30837	0.6211
-	HHIPL2	1	222512260	222583102	204	22	10164	-0.30845	0.62113
-	CNN3	1	94886949	94962223	223	37	10164	-0.30875	0.62124
-	PSIP1	9	15454066	15546005	214	20	10164	-0.3088	0.62126
+	RNASE6	14	20746051	20792467	319	34	10164	-0.30881	0.62127
-	LAMB3	1	209604870	209687475	127	27	10164	-0.30887	0.62129
+	RNF213	17	80225840	80408786	479	54	10164	-0.30923	0.62143
+	RNF181	2	85560268	85607708	142	16	10164	-0.30951	0.62153
-	ARFIP2	11	6464683	6516479	160	14	10164	-0.30971	0.62161
-	RNF212	4	1046248	1148794	303	19	10164	-0.30972	0.62161
+	MTUS2	13	28785348	29515947	2255	129	10164	-0.30985	0.62166
-	OR10K2	1	158409928	158455866	123	4	10164	-0.30989	0.62168
+	TMEM167B	1	109055762	109106934	80	10	10164	-0.31023	0.62181
-	ZNF704	8	80618451	80909781	253	16	10164	-0.31034	0.62185
-	FXYD2	11	117810075	117863092	222	56	10164	-0.3105	0.62191
+	C17orf64	17	60386085	60441426	52	3	10164	-0.31054	0.62193
+	TMEFF1	9	100438238	100587636	354	22	10164	-0.31059	0.62194
+	TMOD2	15	51716561	51826363	434	24	10164	-0.31058	0.62194
+	FSD1	19	4269594	4333846	100	17	10164	-0.31089	0.62206
+	PWWP2B	10	132362197	132427863	85	16	10164	-0.31097	0.62209
-	FAM171A1	10	15201643	15406059	768	62	10164	-0.31098	0.62209
-	TBCC	6	42734496	42781146	89	14	10164	-0.31121	0.62218
-	GORASP1	3	39086599	39143363	148	17	10164	-0.31208	0.62251
+	HS3ST2	16	22779203	22926338	332	43	10164	-0.31211	0.62252
+	HEXB	5	74605023	74731288	150	22	10164	-0.31233	0.6226
-	CCDC121	2	27615638	27664031	86	17	10164	-0.31234	0.62261
-	TRIM5	11	5580982	5720109	638	61	10164	-0.31256	0.62269
-	ITGB7	12	53181318	53242310	151	31	10164	-0.31275	0.62277
-	TADA1	1	166846510	166911417	114	10	10164	-0.31284	0.6228
-	BRF2	8	37833880	37884913	55	16	10164	-0.31305	0.62288
+	ADRBK2	22	25482856	25739294	732	42	10164	-0.3131	0.6229
-	NT5C1B-RDH	2	18544723	18624580	150	21	10164	-0.31317	0.62293
-	METTL5	2	169801757	169859931	169	21	10164	-0.3134	0.62301
-	ARHGAP32	11	128955060	129302015	871	42	10164	-0.31342	0.62302
-	MCUR1	6	13776549	13849563	124	20	10164	-0.31341	0.62302
-	TMEM127	2	96240208	96301013	124	32	10164	-0.31351	0.62306
-	RPUSD4	11	126192094	126246692	136	18	10164	-0.31361	0.62309
-	ABCG2	4	88080264	88266417	471	46	10164	-0.31368	0.62312
+	LOC1053778	17	22357722	22409180	31	1	10164	-0.31378	0.62316
+	APEX1	14	20420131	20467772	205	37	10164	-0.31393	0.62321
-	OLFM3	1	101792567	102032234	648	45	10164	-0.31392	0.62321
-	PLSCR2	3	146381363	146531012	444	24	10164	-0.31407	0.62327
-	CCND3	6	41924933	42083894	464	58	10164	-0.31417	0.6233

STRAND	SYMBOL	CHR	START	STOP	NSNPS	NPARAM	N	ZSTAT	P
-	WBP11	12	14776478	14838467	173	29	10164	-0.31454	0.62345
-	ABCA5	17	69234435	69362182	254	29	10164	-0.31461	0.62347
-	ANKRD40	17	50683190	50742909	41	7	10164	-0.31475	0.62353
+	JUNB	19	12756496	12803311	10	5	10164	-0.3148	0.62354
-	HOXB6	17	48584095	48639992	201	41	10164	-0.31512	0.62367
-	MYLK3	16	46692282	46798289	24	5	10164	-0.31526	0.62372
-	ZGRF1	4	112529333	112671995	295	22	10164	-0.31549	0.6238
-	BAG5	14	103546544	103597814	133	15	10164	-0.31556	0.62383
-	PKD2L1	10	100278145	100381524	260	43	10164	-0.3157	0.62388
+	LRRC70	5	62543735	62591448	82	13	10164	-0.31568	0.62388
+	FOXO6	1	41326931	41393591	214	31	10164	-0.3158	0.62392
+	SLC16A7	12	59560934	59799855	795	52	10164	-0.31587	0.62395
+	INTS8	8	94778311	94890493	343	29	10164	-0.3164	0.62415
+	CA13	8	85210487	85294073	106	10	10164	-0.31647	0.62418
-	HSF5	17	58410167	58523401	129	17	10164	-0.31652	0.62419
+	AKIP1	11	8876118	8930079	136	15	10164	-0.31655	0.62421
-	GLO1	6	38665925	38738176	296	41	10164	-0.31657	0.62422
+	SOCS2	12	93534822	93636236	261	42	10164	-0.3166	0.62423
-	FUCA2	6	143484812	143546883	201	38	10164	-0.3168	0.6243
-	KLF6	10	3765996	3820281	171	19	10164	-0.31683	0.62431
-	CRYZ	1	74695482	74768408	193	19	10164	-0.31684	0.62432
+	ARF5	7	127553352	127601705	128	22	10164	-0.31721	0.62446
-	COL1A1	17	50174096	50236648	85	22	10164	-0.3173	0.62449
-	MAPK9	5	180223594	180327071	505	43	10164	-0.31751	0.62457
-	RALGDS	9	133087720	133184220	221	30	10164	-0.3176	0.6246
+	LOC441098	5	100353304	100402932	63	12	10164	-0.31758	0.6246
-	PSMD6	3	64000549	64059010	143	23	10164	-0.31781	0.62468
-	MEMO1	2	31857188	32046052	436	24	10164	-0.3179	0.62472
-	NREP	5	111719303	112011931	996	58	10164	-0.318	0.62476
-	ARHGAP19	10	97212173	97327673	312	18	10164	-0.31801	0.62476
+	LOC1027237	1	211794718	211862415	152	16	10164	-0.31814	0.62481
-	KCNK17	6	39289001	39349461	260	39	10164	-0.31822	0.62484
-	SHPK	17	3598262	3671322	256	29	10164	-0.31831	0.62488
+	NMD3	3	161186262	161262294	363	26	10164	-0.31835	0.62489
-	DUSP15	20	31851067	31905676	53	11	10164	-0.31836	0.62489
-	R3HDM2	12	57243764	57466000	393	27	10164	-0.31862	0.62499
+	FAM3B	21	41269223	41367727	276	36	10164	-0.31867	0.62501
-	TMED7-TICA	5	115568496	115661179	270	17	10164	-0.31866	0.62501
+	RMI2	16	11310438	11361763	237	23	10164	-0.31869	0.62502
+	INHBC	12	57399685	57462062	113	19	10164	-0.31883	0.62507
-	LOC1053781	13	112852586	112900576	147	20	10164	-0.3189	0.6251
+	KAZN	1	13858515	15128048	4516	347	10164	-0.31908	0.62517
-	ZMAT5	22	29720956	29802068	248	24	10164	-0.31909	0.62517
+	PARD3B	2	204510793	205630162	1851	115	10164	-0.3192	0.62521
+	SPATA20	17	50512089	50565852	155	16	10164	-0.31931	0.62526
+	HLA-DPB1	6	33040926	33099696	1318	38	10164	-0.31956	0.62535
+	LOC1027247	12	89490664	89608063	165	17	10164	-0.31961	0.62537
+	CLDN18	3	137963816	138043652	68	5	10164	-0.31968	0.62539
-	MYL1	2	210280144	210350171	148	13	10164	-0.31982	0.62545
-	FOXJ3	1	42166539	42370877	417	23	10164	-0.32023	0.6256
-	DNAH3	16	20923111	21194441	610	31	10164	-0.3203	0.62563
-	ZNF706	8	101187038	101241064	110	12	10164	-0.32035	0.62565
+	STMND1	6	17067258	17141372	178	25	10164	-0.32046	0.62569
-	SPRR2E	1	153083135	153129528	156	14	10164	-0.32052	0.62571
-	TLR9	3	52211080	52261163	100	18	10164	-0.32052	0.62571
+	ANKDD1B	5	75576218	75681846	326	19	10164	-0.32062	0.62575
+	IQCE	7	2523972	2624734	299	24	10164	-0.32136	0.62603
+	ZNF324B	19	58383560	58467832	225	17	10164	-0.32135	0.62603
+	SLC2A4RG	20	63704858	63754050	162	11	10164	-0.32141	0.62605
-	SHQ1	3	72739277	72883475	40	6	10164	-0.32178	0.62619
-	POTEF	2	130063479	130164222	68	3	10164	-0.32194	0.62625
+	RAPGEF4	2	172700279	173062893	1209	108	10164	-0.32197	0.62626
+	OR51B6	11	5316508	5362446	311	23	10164	-0.32202	0.62628

STRAND	SYMBOL	CHR	START	STOP	NSNPS	NPARAM	N	ZSTAT	P
+	NKIRAS2	17	41982561	42035641	51	8	10164	-0.32211	0.62631
+	CMPK1	1	47298797	47388839	276	21	10164	-0.32213	0.62632
+	PPP2R5A	1	212250537	212371863	343	28	10164	-0.32218	0.62634
-	ATP13A5	3	193264789	193413824	276	27	10164	-0.32242	0.62643
+	UBE2D4	7	43891436	43966136	238	25	10164	-0.32247	0.62645
+	NRTN	19	5770359	5838324	217	27	10164	-0.32283	0.62659
-	NXPE1	11	114507732	114594895	126	13	10164	-0.32289	0.62661
+	LRRCC1	8	85072088	85156080	103	9	10164	-0.32293	0.62662
+	OR1N2	9	122518170	122564162	255	21	10164	-0.32293	0.62663
+	CHMP6	17	80956841	81010133	200	21	10164	-0.32296	0.62664
+	ZNF501	3	44694606	44747083	65	7	10164	-0.32308	0.62668
+	ZNF146	19	36179602	36248774	228	21	10164	-0.32311	0.62669
+	COPG1	3	129214575	129287773	157	8	10164	-0.32309	0.62669
-	PLLP	16	57246097	57319672	215	18	10164	-0.32313	0.6267
+	FABP5	8	81245483	81294777	14	2	10164	-0.32333	0.62678
+	ZNF292	6	87120551	87273690	477	21	10164	-0.32336	0.62679
-	RCHY1	4	75469037	75549744	162	17	10164	-0.32336	0.62679
+	RBM42	19	35594078	35647685	111	15	10164	-0.32379	0.62695
+	SH2D6	2	85383732	85447029	207	28	10164	-0.32378	0.62695
-	PAPSS1	4	107603666	107755263	376	24	10164	-0.32387	0.62698
+	HMGN4	6	26503344	26556937	190	21	10164	-0.32389	0.62699
-	IGSF10	3	151425990	151654926	836	54	10164	-0.32405	0.62705
-	TAS2R10	12	10805851	10863965	100	8	10164	-0.32415	0.62709
-	ZNF479	7	57109574	57177003	216	15	10164	-0.3242	0.62711
+	CD2AP	6	47442746	47637263	394	13	10164	-0.32456	0.62724
-	THAP5	7	108552144	108604768	149	12	10164	-0.32474	0.62731
-	GPR83	11	94367311	94436419	277	32	10164	-0.32529	0.62752
-	NEURL2	20	45878472	45926287	138	33	10164	-0.32536	0.62754
+	ZGPAT	20	63672442	63746142	261	23	10164	-0.32537	0.62755
-	KRTAP5-11	11	71571855	71617875	63	13	10164	-0.32539	0.62756
-	PLAC4	21	41165231	41220239	110	17	10164	-0.32566	0.62766
+	SMARCA5	4	143478463	143567489	162	12	10164	-0.32577	0.6277
-	ATPAF2	17	17967781	18074169	256	23	10164	-0.32591	0.62776
+	CHST3	10	71929362	72023564	297	25	10164	-0.32604	0.6278
-	OSGEPL1	2	189736660	189798198	143	18	10164	-0.32611	0.62783
+	LOC403312	11	125460214	125509526	117	19	10164	-0.32613	0.62784
+	PPM1M	3	52210725	52260599	100	19	10164	-0.32656	0.628
-	STK32C	10	132197489	132367256	513	56	10164	-0.32658	0.62801
+	BOK	2	241523064	241584138	213	36	10164	-0.32662	0.62802
-	C17orf58	17	67981099	68031784	93	9	10164	-0.32666	0.62804
-	HARBI1	11	46593306	46652894	60	11	10164	-0.32671	0.62806
-	ROPN1	3	123959006	124027170	165	22	10164	-0.32678	0.62808
-	JUP	17	41744607	41821712	105	19	10164	-0.32709	0.6282
+	SH3RF2	5	145901563	146091773	432	24	10164	-0.3271	0.6282
-	CD200R1L	3	112802239	112881889	365	26	10164	-0.3274	0.62832
-	FAM161B	14	73922031	73985414	251	26	10164	-0.32751	0.62836
+	CCAR2	8	22569632	22630471	115	10	10164	-0.3276	0.62839
+	ZNF765	19	53352860	53422009	29	10	10164	-0.3277	0.62843
-	OR1L8	9	122536236	122728914	623	50	10164	-0.32774	0.62845
+	AEBP2	12	19404492	19532239	618	23	10164	-0.3278	0.62847
+	ASL	7	66040789	66103343	206	23	10164	-0.32834	0.62867
+	MRPL52	14	22794851	22845037	218	31	10164	-0.32853	0.62874
-	ERAP1	5	96750810	96970924	826	30	10164	-0.3287	0.62881
+	SPATS2	12	49331905	49537426	354	29	10164	-0.3287	0.62881
-	POLR2J3	7	102511171	102607660	1	1	10164	-0.32947	0.6291
+	RABL2A	2	113592237	113653398	63	10	10164	-0.32965	0.62917
+	GLTSCR1	19	47573196	47713277	405	30	10164	-0.32966	0.62917
+	MFSD1	3	158766450	158839719	234	29	10164	-0.32992	0.62927
+	MED17	11	93749239	93823330	164	13	10164	-0.33	0.6293
-	MUL1	1	20489448	20543181	102	13	10164	-0.33002	0.62931
-	PUS7	7	105446511	105557242	396	28	10164	-0.33013	0.62935
+	ABHD2	15	89053150	89212360	444	40	10164	-0.33027	0.6294
-	OR2B11	1	247441029	247486982	258	13	10164	-0.3306	0.62953

STRAND	SYMBOL	CHR	START	STOP	NSNPS	NPARAM	N	ZSTAT	P
-	SDF4	1	1206908	1267067	170	8	10164	-0.33069	0.62956
+	C2CD2L	11	119072341	119128347	169	26	10164	-0.33072	0.62957
+	GSTT2	22	23945028	23993915	44	7	10164	-0.3312	0.62975
-	CENPK	5	65498067	65598189	284	15	10164	-0.33133	0.6298
-	NRP1	10	33167491	33369905	687	44	10164	-0.33147	0.62985
+	ACTL7A	9	108827228	108873755	260	37	10164	-0.33151	0.62987
-	TAS2R14	12	10928254	10974207	120	3	10164	-0.33158	0.62989
-	ECHDC1	6	127278712	127379069	271	27	10164	-0.33182	0.62999
+	SPESP1	15	68895500	68956811	84	9	10164	-0.33182	0.62999
-	NLRP13	19	55881692	55967336	435	44	10164	-0.33223	0.63014
+	PRKCD	3	53126207	53202717	209	34	10164	-0.33235	0.63019
+	CFD	19	824665	873624	19	4	10164	-0.3324	0.63021
+	OR4F6	15	101770720	101816658	65	5	10164	-0.33243	0.63022
-	NDST4	4	114817775	115148876	938	47	10164	-0.33251	0.63025
+	MAD2L1BP	6	43594542	43650952	78	12	10164	-0.33267	0.63031
-	LOC1053695	11	121468813	121517907	80	12	10164	-0.33269	0.63032
-	STIL	1	47240139	47349781	345	20	10164	-0.33305	0.63045
+	CALB2	16	71323713	71400438	260	31	10164	-0.33314	0.63049
-	CLC	19	39721253	39773029	181	8	10164	-0.33314	0.63049
+	VN1R2	19	53223292	53269602	112	10	10164	-0.33323	0.63052
-	ANKRD27	19	32587001	32710196	633	32	10164	-0.33331	0.63055
+	CHGA	14	92887589	92945293	191	32	10164	-0.33373	0.63071
+	RBM27	5	146168550	146299223	211	6	10164	-0.33376	0.63072
+	MGST2	4	139630768	139750745	395	55	10164	-0.33406	0.63083
-	RNGTT	6	88599897	88998716	740	21	10164	-0.33406	0.63083
+	GAST	17	41677326	41725969	142	9	10164	-0.33428	0.63092
-	FSCB	14	44494149	44542296	249	22	10164	-0.33432	0.63093
-	TMEM52	1	1907589	1954301	47	3	10164	-0.33434	0.63094
-	ZNF644	1	90905298	91057255	269	20	10164	-0.33444	0.63098
-	ASB17	1	75908873	75967433	158	15	10164	-0.33444	0.63098
+	KRTAP9-7	17	41240698	41286207	95	6	10164	-0.33445	0.63098
-	DCTN4	5	150698747	150794095	223	9	10164	-0.33502	0.63119
-	OVGP1	1	111404315	111462777	155	16	10164	-0.33529	0.6313
-	VPS36	13	52402602	52485678	157	9	10164	-0.33531	0.6313
+	CLEC6A	12	8420995	8488330	45	2	10164	-0.33533	0.63131
-	FNBP1	9	129877187	130078194	554	29	10164	-0.33553	0.63139
+	KRTAP5-8	11	71503025	71549207	169	20	10164	-0.33553	0.63139
+	MS4A8	11	60664574	60725811	196	8	10164	-0.33561	0.63142
-	EIF4ENIF1	22	31426963	31524950	271	32	10164	-0.33578	0.63148
-	FBXL13	7	102690062	103109841	774	28	10164	-0.33585	0.63151
+	KLHL20	1	173679912	173796822	130	11	10164	-0.33595	0.63154
-	LIX1	5	97081870	97177816	184	27	10164	-0.33629	0.63167
+	METTL22	16	8586640	8671208	250	21	10164	-0.33631	0.63168
+	TMEM33	4	41900120	41970807	146	4	10164	-0.33656	0.63177
-	LRP8	1	53232364	53363149	411	54	10164	-0.33664	0.6318
-	HVCN1	12	110638686	110739950	146	12	10164	-0.33691	0.63191
+	TMEM219	16	29927030	29983052	48	2	10164	-0.33701	0.63194
-	MRE11A	11	94405578	94547185	508	31	10164	-0.33709	0.63197
-	LOC1027247	16	89823423	89870956	209	19	10164	-0.3371	0.63198
-	CARD10	22	37480362	37554203	207	22	10164	-0.33714	0.63199
+	MYO1H	12	109353277	109459004	357	20	10164	-0.33716	0.632
-	NEURL4	17	7305632	7364319	121	27	10164	-0.3372	0.63202
-	EPHX3	19	15216919	15268047	36	7	10164	-0.33727	0.63204
+	WHAMM	15	82773169	82844861	174	26	10164	-0.33735	0.63207
-	MEF2B	19	19135567	19205289	277	27	10164	-0.33735	0.63207
-	OSBP	11	59564398	59651144	149	12	10164	-0.33754	0.63215
+	THRAP3	1	36189416	36315357	194	29	10164	-0.33767	0.63219
-	FBN2	5	128247909	128573042	886	41	10164	-0.33775	0.63222
-	CLTCL1	22	19169473	19326725	569	24	10164	-0.33829	0.63243
-	KLHL14	18	32662668	32808011	87	9	10164	-0.33834	0.63245
-	SST	3	187658906	187705413	159	19	10164	-0.33849	0.6325
-	TGFB3	14	75948097	76017991	150	34	10164	-0.33848	0.6325
+	DNAJB6	7	157302016	157427439	552	41	10164	-0.3385	0.63251

STRAND	SYMBOL	CHR	START	STOP	NSNPS	NPARAM	N	ZSTAT	P
-	C9orf66	9	203108	250893	181	15	10164	-0.33875	0.6326
+	GRID2	4	92269399	93784556	3868	136	10164	-0.33884	0.63264
+	MGAT5	2	134084930	134464621	1189	117	10164	-0.33885	0.63264
-	KLHL33	14	20417651	20471310	221	40	10164	-0.33919	0.63277
+	ABTB1	3	127637938	127690926	95	18	10164	-0.33943	0.63286
-	ATP7B	13	51881086	52047099	355	30	10164	-0.33965	0.63294
-	HEPACAM	11	124909193	124971412	189	26	10164	-0.33965	0.63294
-	AAMP	2	218254129	218305209	104	6	10164	-0.33984	0.63301
-	CEBPZ	2	37191629	37266597	162	21	10164	-0.34	0.63307
-	TMEM167A	5	83042846	83112453	258	27	10164	-0.34002	0.63308
+	SLCO1C1	12	20660355	20763386	320	22	10164	-0.34007	0.6331
+	AOC3	17	42816123	42868130	64	10	10164	-0.3402	0.63315
-	TMEM150C	4	82474451	82596973	157	20	10164	-0.34024	0.63316
+	CDH3	16	68609248	68737577	384	30	10164	-0.34082	0.63338
-	LOC1027239	1	149709896	149784184	22	3	10164	-0.34097	0.63344
-	HSPD1	2	197476584	197535274	52	7	10164	-0.34169	0.63371
-	FSTL5	4	161373892	162199034	2366	133	10164	-0.34174	0.63373
+	INVS	9	100064185	100348617	590	17	10164	-0.34182	0.63376
-	FGF14	13	101709784	102436774	1765	108	10164	-0.34233	0.63395
-	JAKMIP1	4	6016199	6235591	312	52	10164	-0.34235	0.63396
+	CDC42SE2	5	131229009	131404690	240	27	10164	-0.34251	0.63402
+	SLC39A7	6	33165826	33214437	258	45	10164	-0.34259	0.63405
-	EPHA5	4	65309563	65705495	1211	66	10164	-0.34271	0.63409
+	FPGT-TNNI3I	1	74163212	74554432	504	42	10164	-0.34282	0.63413
-	METTL8	2	171307402	171469802	557	28	10164	-0.343	0.6342
-	PLGRKT	9	5347966	5473456	464	50	10164	-0.34301	0.63421
-	CAMKK2	12	121227692	121333308	259	38	10164	-0.34305	0.63422
+	KIF15	3	44726714	44878656	144	11	10164	-0.34312	0.63424
+	TRIP4	15	64352804	64465303	121	9	10164	-0.34313	0.63425
-	PIGM	1	160017672	160066993	112	19	10164	-0.34326	0.6343
-	C1orf195	1	15154196	15206624	162	16	10164	-0.34332	0.63432
-	NUDT18	8	22096872	22145355	72	5	10164	-0.34334	0.63433
+	HIF1A	14	61660401	61758259	325	26	10164	-0.34362	0.63443
+	PTMA	2	231673525	231723541	107	14	10164	-0.34388	0.63453
-	TAF13	1	109054376	109111002	92	11	10164	-0.34387	0.63453
-	HIST2H2BF	1	149745104	149848123	34	9	10164	-0.34397	0.63456
+	PGA4	11	61187331	61241707	13	5	10164	-0.34409	0.63461
+	OR51Q1	11	5387111	5433206	384	16	10164	-0.34418	0.63464
-	SYCP2	20	59853557	59968681	3	2	10164	-0.34419	0.63465
+	VWA3A	16	22056914	22166966	102	19	10164	-0.34437	0.63471
+	RNF207	1	6170775	6231299	124	14	10164	-0.34453	0.63478
-	CCER1	12	90942215	90990176	134	11	10164	-0.34458	0.6348
-	UROC1	3	126471165	126552773	242	28	10164	-0.34476	0.63486
-	COL4A1	13	110138958	110342157	1157	166	10164	-0.34507	0.63498
+	IL34	16	70544895	70670684	305	20	10164	-0.34511	0.63499
+	AGFG1	2	227437172	227571222	362	14	10164	-0.34517	0.63501
+	BSPRY	9	113314532	113381233	275	30	10164	-0.34516	0.63501
+	ZNF559	19	9288772	9353845	101	6	10164	-0.34529	0.63506
+	ACSF2	17	50391158	50484845	208	17	10164	-0.3453	0.63507
-	NDUFA8	9	122122466	122194819	226	31	10164	-0.3454	0.6351
+	ETS2	21	38770307	38834955	172	16	10164	-0.34542	0.63511
+	TMEM254	10	80043646	80102557	147	11	10164	-0.34541	0.63511
+	CENPF	1	214568179	214674571	333	19	10164	-0.34591	0.63529
-	GGA2	16	23453542	23545494	189	12	10164	-0.34603	0.63534
+	EFNA2	19	1251154	1311431	18	6	10164	-0.34649	0.63551
+	RNF225	19	58360657	58408954	128	12	10164	-0.34679	0.63563
+	HIPK3	11	33221672	33367023	154	11	10164	-0.34684	0.63564
-	ZNF878	19	12033648	12087967	153	14	10164	-0.34685	0.63565
-	EXOSC6	16	70240231	70286930	33	14	10164	-0.34728	0.63581
+	GOSR2	17	46888114	46977000	203	14	10164	-0.34761	0.63593
-	DLK2	6	43440352	43491632	88	17	10164	-0.34765	0.63595
+	FAM71F1	7	128674061	128741743	227	24	10164	-0.34774	0.63598
+	MSANTD3-TI	9	100406906	100587636	402	23	10164	-0.34788	0.63604

STRAND	SYMBOL	CHR	START	STOP	NSNPS	NPARAM	N	ZSTAT	P
+	TAF8	6	42015513	42097462	289	48	10164	-0.34795	0.63606
-	RPUSD3	3	9827849	9879018	197	38	10164	-0.34802	0.63609
-	CCL15	17	35987582	36037038	132	20	10164	-0.34812	0.63613
-	LOC1019277	1	149782523	149828847	14	5	10164	-0.34845	0.63625
+	C19orf80	19	11204619	11251943	80	8	10164	-0.3488	0.63638
+	C17orf104	17	44621394	44688306	161	14	10164	-0.34882	0.63639
+	SP4	7	21393071	21524822	576	48	10164	-0.34893	0.63643
-	OSBPL1A	18	24152045	24432882	277	36	10164	-0.34903	0.63646
-	ENG	9	127805012	127889773	194	30	10164	-0.34984	0.63677
-	HLCS	21	36740888	37025246	824	43	10164	-0.34998	0.63682
+	LOC1005096	2	94552234	94629833	59	20	10164	-0.34998	0.63682
-	MEF2D	1	156453721	156535842	168	21	10164	-0.34999	0.63683
-	PYGM	11	64736389	64795715	91	13	10164	-0.35011	0.63687
-	NUP37	12	102064189	102155139	171	16	10164	-0.3502	0.63691
+	TRIM8	10	102607903	102668319	169	27	10164	-0.35031	0.63695
+	ZKSCAN5	7	99466297	99544107	102	16	10164	-0.35044	0.637
-	TMEM107	17	8162978	8211396	128	23	10164	-0.35084	0.63715
+	CNBD1	8	86831448	87392727	1750	84	10164	-0.35085	0.63715
-	PUM1	1	30921506	31100717	147	10	10164	-0.35151	0.6374
-	C10orf76	10	101835599	102091232	367	36	10164	-0.35152	0.6374
+	C8orf58	8	22564601	22614150	73	8	10164	-0.3518	0.63751
-	ARHGAP1	11	46667075	46735665	118	17	10164	-0.35231	0.6377
+	NELL1	11	20634551	21585686	2096	184	10164	-0.35234	0.63771
+	MRTO4	1	19216581	19270128	163	16	10164	-0.35234	0.63771
+	RMDN2	2	37885845	38079970	827	41	10164	-0.35238	0.63772
+	OR51D1	11	4604715	4650838	119	15	10164	-0.35275	0.63786
-	TFPT	19	54097013	54150675	86	10	10164	-0.35321	0.63803
-	SHCBP1	16	46570554	46657810	10	4	10164	-0.35332	0.63807
+	RPSA	3	39371689	39422542	236	14	10164	-0.35367	0.63821
+	SGSM3	22	40335562	40420289	177	22	10164	-0.35394	0.63831
-	KRTAP19-4	21	30486856	30532110	183	10	10164	-0.35409	0.63837
-	LPGAT1	1	211733457	211865832	326	17	10164	-0.35421	0.63841
+	ANO5	11	22157513	22293367	319	12	10164	-0.35437	0.63847
-	DNER	2	229347629	229749570	1016	84	10164	-0.35465	0.63858
-	SATB1	3	18335806	18480588	320	44	10164	-0.3552	0.63878
-	S100A6	1	153524600	153571241	56	7	10164	-0.35525	0.6388
-	FOXD4L5	9	65272101	65320209	2	1	10164	-0.35533	0.63883
+	SPZ1	5	80284971	80331842	181	18	10164	-0.35536	0.63884
-	PAG1	8	80957810	81147068	43	12	10164	-0.35562	0.63894
+	OR9A4	7	141838479	141996420	321	16	10164	-0.35585	0.63902
+	PIK3C3	18	41920199	42094606	299	20	10164	-0.35586	0.63903
+	CKAP2	13	52420360	52486631	126	8	10164	-0.35587	0.63903
+	IZUMO1R	11	94269561	94317762	159	28	10164	-0.35594	0.63906
-	GDF1	19	18858546	18931144	158	27	10164	-0.35603	0.63909
-	CERS1	19	18858546	18931727	158	27	10164	-0.35603	0.63909
+	PLCL2	3	16849959	17100606	623	30	10164	-0.35603	0.63909
+	OSBP2	22	30658782	30917824	617	50	10164	-0.35612	0.63912
+	LOC1019288	6	87117459	87165589	152	13	10164	-0.35616	0.63914
-	SEPHS1	10	13307428	13383298	224	26	10164	-0.35622	0.63916
-	OTUD4	4	145123650	145214973	83	16	10164	-0.35622	0.63916
+	GPR137B	1	236107502	236218909	334	38	10164	-0.35623	0.63917
-	HCN4	15	73309859	73404264	254	40	10164	-0.35637	0.63922
+	FAM189A2	9	69289572	69402456	401	41	10164	-0.35644	0.63925
+	WWOX	16	78064413	79222667	6359	395	10164	-0.35646	0.63925
-	NLRP7	19	54913509	55004965	268	33	10164	-0.35645	0.63925
+	LOC1053718	17	48588142	48635737	167	37	10164	-0.35658	0.6393
-	FREM3	4	143567407	143735675	456	26	10164	-0.35661	0.63931
-	CLOCK	4	55417901	55581909	574	15	10164	-0.35675	0.63936
-	FGFBP1	4	15925569	15973740	183	31	10164	-0.35684	0.6394
-	HNRNPLL	2	38551978	38638036	360	17	10164	-0.3569	0.63942
-	ISY1	3	129117416	129196230	123	23	10164	-0.357	0.63945
+	IFNGR2	21	33367896	33447521	160	18	10164	-0.3571	0.63949
+	MICALCL	11	12251900	12369144	458	42	10164	-0.35726	0.63955

STRAND	SYMBOL	CHR	START	STOP	NSNPS	NPARAM	N	ZSTAT	P
-	NRN1	6	5987999	6042605	211	22	10164	-0.3573	0.63957
+	MTMR14	3	9614433	9712394	186	18	10164	-0.35755	0.63966
+	DNAJC15	13	42988226	43119170	438	32	10164	-0.35757	0.63967
-	FABP2	4	119307250	119357161	309	9	10164	-0.35772	0.63972
-	CGB	19	49012869	49059375	169	25	10164	-0.35778	0.63974
-	ZNF708	19	21281160	21364439	383	14	10164	-0.35796	0.63981
+	ZNF705A	12	8122012	8190046	100	7	10164	-0.35807	0.63985
-	URAD	13	27968106	28023637	172	30	10164	-0.35815	0.63988
+	AAR2	20	36201417	36266941	66	18	10164	-0.35817	0.63989
-	MRPL53	2	74461958	74507815	24	2	10164	-0.35835	0.63996
+	C6orf58	6	127542174	127601818	132	22	10164	-0.35835	0.63996
-	LOC1053759	8	60899752	60945855	141	17	10164	-0.35838	0.63997
-	RSPH6A	19	45785710	45850347	230	19	10164	-0.35868	0.64008
-	S100A16	1	153596886	153648038	88	8	10164	-0.35916	0.64026
-	MYOC	1	171625417	171687633	311	27	10164	-0.35931	0.64032
-	MTIF2	2	55226620	55304303	257	32	10164	-0.3594	0.64035
-	PRKRIR	11	76339956	76415965	223	36	10164	-0.35938	0.64035
-	KIAA0040	1	175146987	175228093	234	30	10164	-0.35938	0.64035
-	NDRG3	20	36641766	36781138	84	25	10164	-0.3595	0.64039
-	IBTK	6	82159987	82282754	336	24	10164	-0.35962	0.64043
+	EFNA3	1	155043872	155097538	81	20	10164	-0.35983	0.64051
-	TTBK2	15	42728734	42956007	445	29	10164	-0.35982	0.64051
+	ELSPBP1	19	47959650	48035156	292	41	10164	-0.35999	0.64057
+	CD177	19	43318659	43373172	129	18	10164	-0.36004	0.64059
+	KCTD11	17	7316889	7364944	104	26	10164	-0.36008	0.64061
-	KIAA0319	6	24534104	24681172	584	63	10164	-0.36018	0.64064
+	GMPPA	2	219463865	219516996	83	8	10164	-0.36044	0.64074
-	ETNPPL	4	108732040	108798079	172	15	10164	-0.36052	0.64077
-	NEUROG1	5	135524282	135570949	46	8	10164	-0.36076	0.64086
-	LYSMD2	15	51713064	51786453	305	22	10164	-0.36084	0.64089
-	SLC15A3	11	60927083	60987526	89	9	10164	-0.36087	0.6409
-	CCDC71L	7	106646765	106696188	78	7	10164	-0.36117	0.64102
-	CLEC5A	7	141917357	141982007	84	9	10164	-0.36125	0.64104
-	AJUBA	14	22961174	23017642	77	12	10164	-0.36133	0.64107
-	COMMD7	20	32692691	32779008	252	21	10164	-0.36149	0.64113
-	REXO1	19	1805246	1883488	149	18	10164	-0.36151	0.64114
+	SCGB1D2	11	62206630	62254812	163	24	10164	-0.36158	0.64117
-	SLC1A2	11	35241205	35455063	692	52	10164	-0.36175	0.64123
+	TSPAN15	10	69390218	69517669	396	16	10164	-0.36202	0.64133
-	RCC2	1	17396755	17474754	238	22	10164	-0.36221	0.6414
-	SPATA6	1	48250553	48507208	757	28	10164	-0.36223	0.64141
-	ABHD17A	19	1866976	1920519	150	16	10164	-0.36223	0.64141
+	NLRP8	19	55912832	55998629	472	46	10164	-0.36232	0.64144
-	DIS3	13	72745402	72817153	175	23	10164	-0.3625	0.64151
-	NDUFC2	11	78058347	78115219	219	12	10164	-0.36262	0.64156
+	CAV2	7	116464601	116518541	174	9	10164	-0.36297	0.64169
-	SFTA2	6	30921350	30967175	279	43	10164	-0.36302	0.64171
-	CD93	20	23069356	23121340	139	14	10164	-0.36305	0.64172
+	SFTPC	8	22126671	22174479	38	8	10164	-0.36311	0.64174
-	DHX57	2	38787729	38910883	362	24	10164	-0.36317	0.64176
+	CIRBP	19	1234266	1284810	46	13	10164	-0.3632	0.64177
-	LOC1053743	4	1022699	1074509	215	18	10164	-0.36321	0.64177
+	SLCO3A1	15	91818708	92182435	1064	119	10164	-0.36337	0.64183
-	BET1L	11	192924	242422	194	17	10164	-0.36346	0.64187
+	FCRL6	1	159765511	159826257	169	24	10164	-0.3636	0.64192
+	ARMC9	2	231163546	231404991	436	39	10164	-0.36364	0.64194
+	PTBP2	1	96686605	96825049	269	15	10164	-0.36368	0.64195
+	WEE1	11	9538650	9603458	164	30	10164	-0.36371	0.64196
+	EXO5	1	40473719	40527725	148	16	10164	-0.36388	0.64203
+	DEFB126	20	107553	155751	137	14	10164	-0.36396	0.64206
-	MTMR6	13	25236201	25322566	93	8	10164	-0.36404	0.64209
+	SGK2	20	43523995	43595633	101	17	10164	-0.36405	0.64209
-	DOK3	5	177491904	177545522	61	7	10164	-0.36418	0.64214

STRAND	SYMBOL	CHR	START	STOP	NSNPS	NPARAM	N	ZSTAT	P
-	RTN4	2	54962189	55147616	654	50	10164	-0.36433	0.64219
+	SPHK2	19	48584291	48640407	136	22	10164	-0.36439	0.64222
-	BUD13	11	116738170	116807998	201	34	10164	-0.36473	0.64234
-	ESYT2	7	158720998	158864628	470	18	10164	-0.36482	0.64238
+	LURAP1L	9	12739990	12833060	250	34	10164	-0.36501	0.64245
-	TXNRD2	22	19865518	19976992	555	64	10164	-0.36513	0.64249
-	FBXO8	4	174226658	174319263	99	13	10164	-0.36534	0.64257
-	LOC1053773	4	111816331	111879523	187	14	10164	-0.3654	0.64259
-	ZNF737	19	20521224	20600847	473	19	10164	-0.36551	0.64264
-	XAB2	19	7609525	7664553	236	21	10164	-0.36568	0.6427
-	DCANP1	5	135434214	135482348	7	4	10164	-0.36572	0.64271
-	ZNF799	19	12380014	12448983	71	7	10164	-0.36577	0.64273
-	TIMP4	3	12143068	12194351	126	18	10164	-0.36581	0.64275
+	ZNF844	19	12029731	12087812	161	14	10164	-0.36582	0.64275
-	SEMA3G	3	52423252	52480027	113	12	10164	-0.36609	0.64285
-	TMEM185B	2	120207471	120258408	131	19	10164	-0.36622	0.6429
+	CYTH2	19	48434208	48492314	138	15	10164	-0.36684	0.64313
+	IFIT1B	10	89343056	89395205	187	21	10164	-0.36684	0.64313
-	LOC1027242	2	65422010	65469846	128	13	10164	-0.36687	0.64314
-	FAM220A	7	6319409	6383959	246	33	10164	-0.36694	0.64317
+	HSD17B7	1	162755702	162822818	133	23	10164	-0.36704	0.6432
+	PACS1	11	66035353	66254747	539	38	10164	-0.36729	0.6433
+	MKRN2	3	12522014	12593713	204	23	10164	-0.36752	0.64338
+	NMNAT1	1	9907923	9995501	162	21	10164	-0.36778	0.64348
-	NUDT3	6	34277194	34427680	626	24	10164	-0.36786	0.64351
+	HIST3H2BB	1	228423107	228468558	175	28	10164	-0.36794	0.64354
-	GPR1	2	206165316	206253047	238	26	10164	-0.36796	0.64355
+	ZNF491	19	11763477	11819622	70	4	10164	-0.36799	0.64356
-	ITIH5	10	7549270	7701998	455	55	10164	-0.36809	0.6436
-	DONSON	21	33567551	33623708	51	10	10164	-0.36813	0.64361
-	MTFR2	6	136221029	136288615	82	15	10164	-0.36816	0.64362
+	TET3	2	73948335	74118176	149	12	10164	-0.36824	0.64365
+	UTP3	4	70653479	70700551	28	10	10164	-0.36825	0.64366
+	C11orf49	11	46901528	47174381	339	17	10164	-0.36828	0.64367
+	NCK1	3	136827208	136961610	308	9	10164	-0.36866	0.64381
-	MED23	6	131563966	131663239	120	20	10164	-0.36894	0.64391
+	NPBWR1	8	52904908	52950894	98	14	10164	-0.3692	0.64401
+	INMT	7	30717135	30767602	155	23	10164	-0.36938	0.64408
-	SNX3	6	108201217	108296260	125	11	10164	-0.36954	0.64414
+	ARID5B	10	61866254	62106948	510	74	10164	-0.36962	0.64417
-	OR5F1	11	55983681	56029625	341	9	10164	-0.36992	0.64428
-	STRBP	9	123099494	123303576	263	35	10164	-0.37	0.64431
+	TTLL2	6	167290086	167352689	290	33	10164	-0.37024	0.6444
-	KCNA10	1	110507217	110554175	137	18	10164	-0.37033	0.64443
+	C9orf16	9	128124425	128173928	56	8	10164	-0.37036	0.64444
+	OR4D5	11	123904617	123950573	244	25	10164	-0.37037	0.64445
-	OLFM2	19	9843718	9971552	378	42	10164	-0.37047	0.64449
+	LHX8	1	75093434	75171533	140	18	10164	-0.3707	0.64457
+	LOC1019278	22	20302984	20371250	32	13	10164	-0.37071	0.64457
-	CLEC7A	12	10106777	10165273	199	20	10164	-0.37073	0.64458
+	EBAG9	8	109504700	109575996	160	13	10164	-0.37128	0.64478
-	TFB2M	1	246530561	246601319	248	30	10164	-0.37128	0.64479
+	FAM50B	6	3814398	3861317	168	30	10164	-0.37141	0.64483
-	ASCL5	1	201103953	201150372	216	32	10164	-0.3715	0.64487
-	PPP3R1	2	68168857	68287519	200	11	10164	-0.37158	0.6449
-	CCDC153	11	119180226	119241286	150	17	10164	-0.372	0.64505
+	FZD7	2	201999587	202048437	138	19	10164	-0.37206	0.64508
-	TNR	1	175312799	175778702	1385	112	10164	-0.37225	0.64515
-	CCDC142	2	74462832	74518230	30	2	10164	-0.37226	0.64515
-	OR6F1	1	247701829	247747755	92	10	10164	-0.37245	0.64522
-	PYROXD2	10	98373565	98450221	234	16	10164	-0.37251	0.64524
+	LCN2	9	128114453	128163455	51	9	10164	-0.37272	0.64532
-	BTG1	12	92130278	92180897	81	14	10164	-0.37273	0.64533

STRAND	SYMBOL	CHR	START	STOP	NSNPS	NPARAM	N	ZSTAT	P
+	COQ6	14	73914934	73973112	234	25	10164	-0.37274	0.64533
+	RPE	2	209967565	210034599	81	14	10164	-0.37304	0.64544
+	CRYBG3	3	97786937	97954986	272	25	10164	-0.37309	0.64546
-	PEMT	17	17495563	17626703	422	40	10164	-0.37331	0.64554
+	C9orf3	9	94691604	95157311	1423	42	10164	-0.37334	0.64555
-	FOXRED2	22	36477186	36542101	97	7	10164	-0.37345	0.64559
+	PKHD1L1	8	109327477	109542686	581	20	10164	-0.37395	0.64578
+	LGR5	12	71404162	71596310	563	33	10164	-0.37415	0.64585
+	GOLGA6C	15	75223558	75283455	45	26	10164	-0.37414	0.64585
+	FCAR	19	54839093	54901420	231	15	10164	-0.37438	0.64594
-	PEG3	19	56800077	56875726	100	10	10164	-0.37439	0.64594
-	CCDC59	12	82342304	82393805	17	9	10164	-0.37437	0.64594
+	CCAR1	10	68686144	68802377	289	24	10164	-0.37448	0.64598
+	NPW	16	1984520	2030755	138	13	10164	-0.37452	0.64599
+	CLDN23	8	8667156	8714107	220	13	10164	-0.37454	0.646
-	ZNF837	19	58357623	58416022	154	15	10164	-0.37461	0.64603
+	ZNF257	19	22013132	22101109	382	17	10164	-0.37498	0.64616
+	GIMAP1-GIM	7	150681557	150753649	229	24	10164	-0.37503	0.64618
+	FOXRED1	11	126234040	126288132	121	17	10164	-0.3753	0.64628
-	DAGLB	7	6399116	6483012	313	27	10164	-0.37538	0.64631
+	EIF4EBP2	10	70369105	70438618	189	23	10164	-0.3761	0.64658
+	ADAT3	19	1870372	1923447	153	17	10164	-0.37611	0.64658
-	CRMP1	4	5810764	5928083	102	19	10164	-0.3762	0.64662
-	PGLYRP1	19	46009153	46058298	145	22	10164	-0.3764	0.64669
+	RCL1	9	4757834	4871077	442	39	10164	-0.37639	0.64669
-	C8orf76	8	123209956	123276398	21	12	10164	-0.37677	0.64683
-	RARS2	6	87489347	87625017	462	23	10164	-0.3769	0.64688
+	PALM2-AKAF	9	109745297	110182512	905	72	10164	-0.37714	0.64697
+	TCAP	17	39630346	39676554	85	11	10164	-0.37734	0.64704
+	LOC1019299	16	8847334	8902650	193	36	10164	-0.37764	0.64715
-	ZNF680	7	64491290	64598127	427	22	10164	-0.37784	0.64722
+	GATA6	18	22134437	22212528	132	18	10164	-0.3779	0.64725
-	RUVBL1	3	128054877	128188909	422	35	10164	-0.37801	0.64729
-	KLK14	19	51067879	51121458	230	57	10164	-0.3781	0.64732
+	VGLL2	6	117230558	117283565	166	28	10164	-0.37814	0.64734
-	CSDE1	1	114706913	114793050	97	11	10164	-0.37829	0.64739
+	TCF19	6	31123526	31174215	728	53	10164	-0.37845	0.64745
+	PADI6	1	17337196	17411699	326	20	10164	-0.37931	0.64777
+	CENPO	2	24758306	24832376	184	19	10164	-0.37949	0.64784
-	UBE2V1	20	50071124	50150959	183	23	10164	-0.37962	0.64789
+	ERAL1	17	28820025	28871054	88	9	10164	-0.37967	0.6479
+	PSMB6	17	4761144	4808503	151	30	10164	-0.37967	0.64791
+	TUB	11	7984180	8116241	303	41	10164	-0.3798	0.64795
-	NHLH2	1	115821911	115876127	160	19	10164	-0.37986	0.64798
-	GSR	8	30668061	30762969	332	22	10164	-0.38018	0.6481
-	POLE	12	132613762	132722524	298	11	10164	-0.38028	0.64813
+	CLEC3A	16	77987546	78042106	423	26	10164	-0.38085	0.64834
+	MFSD6	2	190373355	190512315	344	22	10164	-0.38151	0.64859
-	NFIL3	9	91399045	91459626	231	37	10164	-0.38156	0.6486
-	TMPRSS5	11	113677546	113749727	256	26	10164	-0.38173	0.64867
-	MYO10	5	16651907	16971276	907	108	10164	-0.38181	0.6487
-	EEF1G	11	62549601	62608988	38	8	10164	-0.38184	0.64871
-	HMGN3	6	79191245	79269738	119	15	10164	-0.38235	0.6489
-	S100A3	1	153537333	153584258	48	8	10164	-0.38254	0.64897
+	AP4S1	14	30990106	31106450	341	29	10164	-0.38258	0.64899
-	DEFB112	6	50033575	50083651	72	6	10164	-0.38269	0.64902
-	ZNF260	19	36500687	36563346	190	25	10164	-0.38302	0.64915
+	DECR2	16	366858	422487	228	29	10164	-0.38315	0.64919
-	ANKRD13C	1	70249002	70389788	309	38	10164	-0.38314	0.64919
+	NXPE3	3	101744185	101838231	171	21	10164	-0.38328	0.64924
-	MYOZ1	10	73621612	73676757	124	17	10164	-0.38385	0.64946
+	GJD4	10	35570410	35618935	92	15	10164	-0.38423	0.6496
+	MBNL2	13	97106820	97404120	699	58	10164	-0.38438	0.64965

STRAND	SYMBOL	CHR	START	STOP	NSNPS	NPARAM	N	ZSTAT	P
+	DCAF17	2	171399173	171501027	367	20	10164	-0.3844	0.64966
+	ODF3	11	161641	210258	56	10	10164	-0.3844	0.64966
+	GRAMD1A	19	34959784	35036471	259	28	10164	-0.38446	0.64968
+	LOC1001299	1	42752990	42808230	99	14	10164	-0.38445	0.64968
+	DPH3P1	20	62809566	62856191	152	26	10164	-0.38465	0.64975
+	HSD17B8	6	33169637	33216831	246	44	10164	-0.38468	0.64976
+	ESPN	1	6389776	6470944	188	14	10164	-0.38467	0.64976
-	RPL17	18	49478481	49527565	187	14	10164	-0.38487	0.64983
+	MAMDC2	9	70008519	70236972	733	25	10164	-0.38497	0.64987
-	LOC1053692	9	87920514	87974304	76	11	10164	-0.38499	0.64988
+	TOP1	20	40993822	41134487	367	24	10164	-0.38507	0.64991
-	MTHFSD	16	86520176	86590235	333	28	10164	-0.38524	0.64997
-	TMEM155	4	121748930	121801042	172	20	10164	-0.38528	0.64998
-	CTAGE9	6	131698441	131746017	64	7	10164	-0.38543	0.65004
+	TMC1	9	72486801	72846351	904	21	10164	-0.38562	0.65011
-	LOC1006527	5	151759793	151806508	118	13	10164	-0.38585	0.6502
-	DHCR24	1	54839627	54922248	366	29	10164	-0.38602	0.65026
-	AUNIP	1	25821913	25894458	93	8	10164	-0.38602	0.65026
+	CPED1	7	120953697	121307444	675	36	10164	-0.38605	0.65027
-	CORO7-PAM	16	4330251	4451961	11	5	10164	-0.38609	0.65028
+	TAF3	10	7783477	8026631	942	55	10164	-0.3861	0.65029
+	NDUFB3	2	201036739	201095750	116	28	10164	-0.38614	0.6503
-	TNFSF14	19	6648135	6705588	208	28	10164	-0.38619	0.65032
+	PRRC2B	9	131395090	131510191	208	13	10164	-0.38675	0.65053
-	CELF6	15	72274727	72355184	169	14	10164	-0.38756	0.65083
-	NLRC4	2	32214449	32300743	166	22	10164	-0.3876	0.65084
-	PSMA2	7	42906861	42967206	113	6	10164	-0.38802	0.651
-	RCBTB2	13	48478963	48568256	181	23	10164	-0.38816	0.65105
-	SHC3	9	88995771	89217072	371	31	10164	-0.38818	0.65106
-	IL26	12	68153987	68260791	389	38	10164	-0.38841	0.65114
-	PSAP	10	71806298	71886325	228	30	10164	-0.38847	0.65117
+	SMO	7	129153872	129223546	173	21	10164	-0.38855	0.65119
+	ITPKA	15	41458858	41513559	95	20	10164	-0.38869	0.65125
-	NMUR2	5	152381541	152440281	267	14	10164	-0.38874	0.65126
-	KLF1	19	12874422	12922203	61	7	10164	-0.38874	0.65127
-	BTBD7	14	93227550	93368092	414	23	10164	-0.38892	0.65133
+	GTF2I	7	74622665	74770692	113	11	10164	-0.38899	0.65136
-	ERLIN1	10	100140090	100221057	214	20	10164	-0.38916	0.65142
-	CTSO	4	155914118	155988896	329	30	10164	-0.3893	0.65147
-	OR5W2	11	55903650	55949582	330	7	10164	-0.38935	0.65149
+	GPR180	13	94566850	94644645	142	9	10164	-0.38938	0.6515
+	GRHL2	8	101457406	101688098	700	59	10164	-0.38963	0.65159
-	FBXL12	19	9800267	9854425	101	18	10164	-0.38993	0.65171
+	RINT1	7	105497085	105577678	349	17	10164	-0.39007	0.65176
+	H1FNT	12	48293980	48340279	149	20	10164	-0.39037	0.65187
+	NFE2L1	17	48013324	48071545	185	37	10164	-0.39039	0.65188
-	CYP19A1	15	51198057	51373598	668	54	10164	-0.39042	0.65189
+	GRAMD1C	3	113793182	113957174	354	46	10164	-0.39061	0.65196
-	PIK3C2A	11	17076561	17242996	357	25	10164	-0.39069	0.65199
-	CCDC127	5	194760	253182	341	13	10164	-0.39088	0.65206
-	IL20RA	6	136989971	137080366	172	26	10164	-0.39097	0.65209
-	AZI2	3	28312353	28384424	110	10	10164	-0.39096	0.65209
+	ULBP1	6	149929007	149983710	257	17	10164	-0.39098	0.6521
-	EFEMP1	2	55855962	55959163	355	39	10164	-0.39103	0.65211
+	FBXO4	5	41890252	42050228	320	19	10164	-0.39112	0.65214
+	LOC1053714	1	153765481	153814151	90	23	10164	-0.39132	0.65222
-	OR51E2	11	4670171	4732846	215	21	10164	-0.39168	0.65235
+	CES2	16	66899444	66955096	88	17	10164	-0.39175	0.65238
-	RNF141	11	10501678	10576227	50	5	10164	-0.39174	0.65238
+	CEP97	3	101689084	101780562	187	26	10164	-0.3922	0.65255
-	LPA	6	160521483	160701375	445	50	10164	-0.3924	0.65262
+	LIME1	20	63701626	63749107	156	11	10164	-0.39247	0.65265
-	KCND3	1	111765822	112024576	694	77	10164	-0.39255	0.65267

STRAND	SYMBOL	CHR	START	STOP	NSNPS	NPARAM	N	ZSTAT	P
+	FCGR1A	1	149746275	149802518	16	5	10164	-0.39272	0.65274
+	LOC1019289	4	9497033	9670852	539	25	10164	-0.39286	0.65279
-	MYBL1	8	66552175	66648249	108	5	10164	-0.39288	0.6528
-	CPN2	3	194329765	194386345	123	9	10164	-0.39301	0.65284
+	LOC1053703	13	112701753	112748367	138	12	10164	-0.39301	0.65285
+	PIN1	19	9800207	9859689	108	19	10164	-0.39306	0.65286
+	GLYCTK	3	52252820	52305256	92	14	10164	-0.3932	0.65291
-	PPP3CB	10	73426428	73531024	76	7	10164	-0.39321	0.65292
+	USP39	2	85567842	85659284	232	27	10164	-0.39333	0.65296
-	CTAGE8	7	144256674	144304784	1	1	10164	-0.39345	0.65301
+	GRAP2	22	39858808	39983343	165	31	10164	-0.39372	0.65311
-	ZNF442	19	12339371	12400985	62	10	10164	-0.39431	0.65332
+	SCG5	15	32606669	32707097	261	56	10164	-0.39447	0.65338
-	AASDH	4	56327687	56424418	330	20	10164	-0.39462	0.65344
-	CDSN	6	31105088	31155475	847	57	10164	-0.39486	0.65353
+	CEP44	4	174248677	174344355	101	13	10164	-0.39492	0.65355
+	ARHGAP23	17	38384286	38522392	234	34	10164	-0.39496	0.65356
+	UMODL1	21	41996463	42152996	642	74	10164	-0.39495	0.65356
-	CYP2R1	11	14867436	14929034	100	26	10164	-0.39525	0.65367
+	DIO1	1	53859188	53921086	197	16	10164	-0.39548	0.65375
-	XRCC2	7	152636498	152711165	247	30	10164	-0.39564	0.65381
+	FAM216A	12	110433427	110500387	100	10	10164	-0.39577	0.65386
+	CCDC176	14	73984350	74088351	479	28	10164	-0.39576	0.65386
-	NOL9	1	6511347	6589598	295	15	10164	-0.39589	0.65391
-	MUC15	11	26549032	26607268	289	38	10164	-0.39589	0.65391
-	ERI3	1	44211070	44390279	372	29	10164	-0.39606	0.65397
-	CPPED1	16	12649799	12838887	435	34	10164	-0.39608	0.65398
+	DNM2	19	10683053	10841910	348	23	10164	-0.39616	0.65401
-	ELF5	11	34468795	34548800	214	26	10164	-0.39616	0.65401
-	GSK3B	3	119811321	120129417	896	38	10164	-0.39645	0.65411
-	PARG	10	49808275	49977027	198	17	10164	-0.39662	0.65418
-	SYT13	11	45230302	45321333	312	20	10164	-0.39704	0.65433
-	KRTAP19-2	21	30477190	30522348	163	10	10164	-0.39707	0.65434
-	MTRNR2L4	16	3361053	3407283	163	16	10164	-0.39707	0.65434
-	ZNF266	19	9402426	9470578	256	15	10164	-0.39711	0.65436
-	PLCH1	3	155469881	155780089	967	34	10164	-0.39725	0.65441
-	LRRC47	1	3770220	3831504	281	18	10164	-0.39758	0.65453
+	DMRT3	9	941968	1001736	148	13	10164	-0.39766	0.65456
+	SUN1	7	780557	884920	302	15	10164	-0.39769	0.65457
+	SH2D4A	8	19278443	19406218	411	47	10164	-0.39774	0.65459
-	AXIN1	16	277440	396497	624	40	10164	-0.39791	0.65465
-	SPATA19	11	133825930	133880538	177	13	10164	-0.39794	0.65466
+	ARMS2	10	122419663	122467352	137	22	10164	-0.39796	0.65467
-	LSM12	17	44024635	44102619	111	17	10164	-0.39799	0.65468
-	VWF	12	5938874	6159670	721	75	10164	-0.39829	0.65479
+	APH1B	15	63242519	63319126	183	31	10164	-0.39831	0.6548
-	ABHD15	17	29550661	29602024	75	19	10164	-0.39852	0.65488
-	RLN1	9	5303627	5374873	234	27	10164	-0.39856	0.65489
+	PPFIBP2	11	7478765	7677344	828	86	10164	-0.39864	0.65492
-	EPB41L4A	5	112132319	112454490	1034	53	10164	-0.39868	0.65494
+	TEX29	13	111281185	111354247	256	39	10164	-0.39895	0.65503
-	TRIM7	5	181183924	181240293	211	24	10164	-0.39895	0.65504
-	WDFY1	2	223865343	223980387	321	17	10164	-0.39901	0.65506
+	LOC1053788	1	94890901	94941255	127	26	10164	-0.39902	0.65506
+	VAMP5	2	85549408	85603388	160	18	10164	-0.39904	0.65507
+	PRAMEF5	1	13219198	13273435	11	1	10164	-0.39936	0.65518
-	LOC391003	1	13212695	13261154	11	1	10164	-0.39936	0.65518
-	GLDC	9	6522464	6680692	591	53	10164	-0.39944	0.65522
+	EHBP1L1	11	65541038	65602650	141	31	10164	-0.39965	0.65529
-	LRCOL1	12	132592224	132645451	229	18	10164	-0.40003	0.65543
-	NDUFB1	14	92106123	92156809	208	19	10164	-0.40009	0.65545
+	KIZ	20	21090983	21256622	271	17	10164	-0.40017	0.65549
+	KDM4B	19	4934112	5163598	637	33	10164	-0.40028	0.65553

STRAND	SYMBOL	CHR	START	STOP	NSNPS	NPARAM	N	ZSTAT	P
-	HSDL1	16	84112139	84180195	242	36	10164	-0.40064	0.65566
+	PHOSPHO2-I	2	169659454	169761886	442	27	10164	-0.40067	0.65567
+	OR5AS1	11	55995419	56041393	366	9	10164	-0.40083	0.65573
-	LOC1027234	4	38584516	38645466	230	39	10164	-0.40098	0.65578
-	TMED10	14	75121468	75211646	168	10	10164	-0.401	0.65579
+	AGAP3	7	151050831	151154436	248	32	10164	-0.40107	0.65582
+	CLPTM1	19	44919585	45003346	235	19	10164	-0.40118	0.65585
-	C18orf32	18	49471178	49522274	201	15	10164	-0.40141	0.65594
-	DUSP4	8	29323062	29385750	132	15	10164	-0.40147	0.65596
-	RPL17-C18or	18	49471178	49527565	216	15	10164	-0.40152	0.65598
+	TDRD5	1	179556613	179701272	473	29	10164	-0.40167	0.65604
-	ACADL	2	210177990	210260491	84	28	10164	-0.402	0.65616
-	PCDH15	10	53792771	54836291	4297	138	10164	-0.40217	0.65622
+	CRKL	22	20882398	20963749	65	4	10164	-0.40243	0.65631
+	GPALPP1	13	44954538	45043611	175	19	10164	-0.40265	0.6564
+	SLC20A1	2	112610857	112673825	105	15	10164	-0.40299	0.65652
+	ARHGEF7	13	111080277	111315734	693	61	10164	-0.40299	0.65652
+	NDUFAF7	2	37196631	37259160	132	18	10164	-0.40304	0.65654
+	TNRC6A	16	24694714	24836227	316	13	10164	-0.4032	0.6566
+	RGPD5	2	109725607	109867691	22	8	10164	-0.40337	0.65666
-	PJA2	5	109324709	109444974	396	16	10164	-0.40343	0.65669
-	NOL4L	20	32433059	32620073	336	28	10164	-0.40345	0.65669
+	THNSL1	10	24981579	25036664	79	4	10164	-0.4038	0.65682
-	WNT11	11	76176326	76249008	270	50	10164	-0.40398	0.65689
+	OR6Q1	11	57995953	58041906	236	22	10164	-0.40403	0.6569
+	ZNF133	20	18253283	18326996	159	15	10164	-0.40409	0.65693
+	PLEKHH1	14	67498199	67599538	397	51	10164	-0.40412	0.65694
+	AKR1D1	7	138041432	138128304	212	24	10164	-0.40416	0.65695
-	POLM	7	44062062	44117540	137	22	10164	-0.40439	0.65704
+	ERCC5	13	102810841	102886001	256	31	10164	-0.40446	0.65706
+	SCGB1C1	11	152899	204575	38	9	10164	-0.40446	0.65706
-	CLEC1A	12	10059504	10134065	244	12	10164	-0.40468	0.65715
-	FAM196B	5	169851291	170015740	424	60	10164	-0.40472	0.65716
+	TUSC3	8	15505087	15776649	1531	60	10164	-0.40489	0.65722
-	ADAMTS9	3	64505654	64723000	595	57	10164	-0.40503	0.65727
+	LOC1009966	2	219459300	219508287	72	8	10164	-0.40505	0.65728
-	HOXB13	17	48714763	48763749	161	46	10164	-0.40518	0.65733
+	TOM1L1	17	54865691	54971967	499	62	10164	-0.4055	0.65744
+	HSD17B6	12	56717449	56797790	135	15	10164	-0.40568	0.65751
+	TAOK2	16	29938641	30002261	54	2	10164	-0.40569	0.65751
-	ELAVL3	19	11441326	11516046	140	13	10164	-0.40572	0.65753
-	VAMP4	1	171690156	171777239	266	20	10164	-0.40573	0.65753
+	BEST1	11	61911715	61974463	158	28	10164	-0.40607	0.65765
-	CDH11	16	64935226	65161112	723	50	10164	-0.40617	0.65769
-	LPXN	11	58516871	58613892	186	22	10164	-0.40624	0.65772
-	ASB6	9	129624604	129677169	101	23	10164	-0.40625	0.65772
-	LRRTM4	2	76737457	77557376	2900	111	10164	-0.40639	0.65777
-	ATP5I	4	662436	709338	68	16	10164	-0.40658	0.65784
-	STAU2	8	73410369	73782708	1179	53	10164	-0.40689	0.65795
+	NDUFAF6	8	94860451	95068716	561	29	10164	-0.40686	0.65795
-	ZAR1L	13	32293719	32347582	177	23	10164	-0.40687	0.65795
+	BLK	8	11458991	11574599	451	37	10164	-0.40694	0.65797
-	GNB1	1	1775285	1926117	61	4	10164	-0.40692	0.65797
+	CCNY	10	35211981	35582670	575	23	10164	-0.40713	0.65805
+	ZSCAN22	19	58292019	58352349	78	10	10164	-0.40713	0.65805
+	UNG	12	109062594	109120993	115	25	10164	-0.40717	0.65806
+	FER1L5	2	96607711	96716220	178	46	10164	-0.4074	0.65814
-	RCVRN	17	9887708	9940367	73	11	10164	-0.40741	0.65815
-	CCDC89	11	85673844	85721277	89	18	10164	-0.40768	0.65825
+	LOC1053717	17	44075810	44130524	87	13	10164	-0.40768	0.65825
-	TEX10	9	100292075	100387977	223	18	10164	-0.40777	0.65828
-	CECR1	22	17168790	17256854	241	45	10164	-0.40781	0.65829
-	CALCOCO1	12	53701118	53762523	195	36	10164	-0.4079	0.65833

STRAND	SYMBOL	CHR	START	STOP	NSNPS	NPARAM	N	ZSTAT	P
+	S100PBP	1	32781767	32868879	110	8	10164	-0.40797	0.65835
-	VOPP1	7	55424406	55608355	432	36	10164	-0.40832	0.65848
-	PCDH12	5	141933585	141994062	118	19	10164	-0.40842	0.65852
-	CLK2	1	155252868	155308529	97	9	10164	-0.40857	0.65857
+	STAB2	12	103552255	103776729	713	81	10164	-0.40865	0.6586
-	STK38	6	36483889	36582479	232	16	10164	-0.40884	0.65867
+	TTC31	2	74448032	74504564	29	1	10164	-0.40888	0.65869
+	GPATCH11	2	37049451	37109244	90	19	10164	-0.40904	0.65875
-	CCL21	9	34699005	34745167	145	20	10164	-0.40908	0.65876
-	DCAF5	14	69040920	69188393	232	23	10164	-0.40912	0.65878
+	AGBL1	15	86044787	87041178	4160	201	10164	-0.40913	0.65878
+	HES1	3	194101142	194148612	36	6	10164	-0.40921	0.65881
+	PDXK	21	43684097	43772307	250	29	10164	-0.40948	0.65891
-	SLC7A1	13	29499410	29630688	401	31	10164	-0.40953	0.65892
+	MTMR3	22	29848165	30040868	503	36	10164	-0.40987	0.65905
+	MAPKAPK5	12	111807228	111911619	255	12	10164	-0.41007	0.65912
+	C8orf34	8	68295722	68829023	1227	78	10164	-0.41022	0.65918
+	ARG2	14	67584862	67661720	320	25	10164	-0.41034	0.65922
-	ADAM2	8	39733735	39873289	388	28	10164	-0.41064	0.65933
-	LOC1006531	15	32479200	32526804	13	3	10164	-0.41116	0.65952
-	NPBWR2	20	64095830	64141831	91	14	10164	-0.41128	0.65957
-	CASKIN1	16	2167183	2231464	120	18	10164	-0.41132	0.65958
+	ANKAR	2	189639292	189773058	300	23	10164	-0.41138	0.6596
+	STRADB	2	201416669	201490851	190	37	10164	-0.41143	0.65962
+	CTDP1	18	79644801	79764510	464	29	10164	-0.41172	0.65973
+	TMEM235	17	78196310	78250987	253	36	10164	-0.41183	0.65977
-	GLYR1	16	4793203	4882333	98	5	10164	-0.41203	0.65984
-	EDAR	2	108884471	109024372	391	26	10164	-0.41212	0.65987
+	SWT1	1	185122023	185301781	423	17	10164	-0.41253	0.66002
+	ACTL8	1	17677000	17837063	486	49	10164	-0.41255	0.66003
-	UQCRFS1	19	29197260	29248229	120	14	10164	-0.41319	0.66027
+	DNAJA3	16	4390805	4466775	6	3	10164	-0.41333	0.66032
+	LOC1027241	17	43894879	43953989	167	17	10164	-0.41338	0.66033
-	ZNF461	19	36626683	36702131	216	21	10164	-0.4134	0.66034
+	WWP2	16	69727284	69951741	491	28	10164	-0.41345	0.66036
-	ATXN3	14	92048552	92141621	476	24	10164	-0.41349	0.66038
+	KPNA6	1	32073043	32186568	136	9	10164	-0.41357	0.6604
-	CDH16	16	66898122	66953984	88	17	10164	-0.4138	0.66049
-	RNF186	1	19804029	19850278	189	36	10164	-0.41384	0.6605
+	TRIM41	5	181188263	181245808	213	23	10164	-0.41383	0.6605
+	LOC1053722	19	9400607	9472529	264	15	10164	-0.41386	0.66051
+	TTC19	17	15964380	16039409	177	10	10164	-0.4139	0.66053
-	HELQ	4	83397343	83490883	149	12	10164	-0.41416	0.66062
+	GPR22	7	107435057	107486212	91	9	10164	-0.41428	0.66067
+	POLB	8	42303455	42381813	116	10	10164	-0.41445	0.66073
-	TM6SF2	19	19254365	19308265	149	23	10164	-0.41469	0.66081
+	IREB2	15	78403176	78511456	401	40	10164	-0.4147	0.66082
-	DST	6	56447987	56989628	976	38	10164	-0.41475	0.66084
+	CTIF	18	48504056	48873217	862	103	10164	-0.41484	0.66087
+	LST1	6	31551179	31598909	311	54	10164	-0.41492	0.6609
-	STOML3	13	38955925	39025859	220	17	10164	-0.41552	0.66112
+	GLIPR1L1	12	75299639	75380390	228	23	10164	-0.41563	0.66116
+	OR52K1	11	4453879	4499908	185	21	10164	-0.41562	0.66116
+	DNM1L	12	32644200	32755650	518	11	10164	-0.41577	0.66121
-	HIST2H2AB	1	149877469	149922916	22	11	10164	-0.4162	0.66137
-	POU5F1	6	31154337	31205693	753	39	10164	-0.41648	0.66147
+	CCDC172	10	116289104	116390029	268	26	10164	-0.41665	0.66153
-	ACTBL2	5	57470016	57517809	131	20	10164	-0.4174	0.66181
-	HOXB7	17	48597232	48646021	177	34	10164	-0.41754	0.66186
+	LOC1053722	19	13971900	14018223	44	10	10164	-0.41774	0.66193
-	LRRC66	4	51983640	52055311	92	10	10164	-0.41781	0.66196
+	OAS2	12	112935819	113021723	216	25	10164	-0.41792	0.662
+	TNFSF13	17	7523292	7571608	180	28	10164	-0.41792	0.662

STRAND	SYMBOL	CHR	START	STOP	NSNPS	NPARAM	N	ZSTAT	P
+	ALDH16A1	19	49418216	49481050	158	20	10164	-0.4183	0.66214
-	ARHGAP22	10	48420318	48691265	677	40	10164	-0.41834	0.66215
-	AES	19	3042910	3098107	156	31	10164	-0.4186	0.66224
-	TAF1L	9	32619454	32670669	65	9	10164	-0.41869	0.66228
+	TMUB2	17	44151969	44201731	85	10	10164	-0.4189	0.66236
-	MOGS	2	74451057	74500410	26	1	10164	-0.41897	0.66238
-	RSF1	11	77650009	77855869	404	13	10164	-0.41936	0.66252
-	LOC1053789	1	110512234	110560306	143	17	10164	-0.41934	0.66252
-	PAQR7	1	25851484	25911707	81	8	10164	-0.4194	0.66254
-	FAM117A	17	49700325	49799156	126	31	10164	-0.41959	0.66261
-	KIF5B	10	31999010	32091443	222	22	10164	-0.41975	0.66267
+	ZNF789	7	99437829	99497600	112	14	10164	-0.41976	0.66267
+	UPB1	22	24459467	24538390	148	19	10164	-0.41989	0.66272
+	MAL	2	94990655	95063992	99	34	10164	-0.41994	0.66274
+	FAM188B	7	30736417	30902387	530	34	10164	-0.41995	0.66274
-	DPY30	2	32011080	32074840	155	18	10164	-0.42	0.66276
+	LOC1019272	8	102619318	102740395	380	60	10164	-0.42007	0.66278
+	SCAF8	6	154698378	154844244	63	16	10164	-0.42014	0.66281
-	DUSP18	22	30642051	30702890	130	25	10164	-0.42019	0.66283
-	MBIP	14	36288558	36355676	120	11	10164	-0.42045	0.66292
+	FBXL21	5	135895317	135951678	167	22	10164	-0.42066	0.663
-	HDAC7	12	47772722	47855612	303	54	10164	-0.4207	0.66301
-	RBM4B	11	66654994	66712921	74	23	10164	-0.42081	0.66305
-	SLBP	4	1682800	1747694	258	17	10164	-0.42089	0.66308
-	GLOD4	17	749309	817331	237	38	10164	-0.42091	0.66309
-	TM9SF1	14	24179143	24230736	99	17	10164	-0.42099	0.66312
+	TAGLN	11	117164324	117214792	152	19	10164	-0.42104	0.66314
+	CSK	15	74747084	74813198	240	34	10164	-0.42113	0.66317
-	ARAP3	5	141643401	141717233	209	44	10164	-0.42114	0.66317
-	ADAM19	5	157467304	157610823	487	33	10164	-0.42153	0.66332
-	KRTAP19-3	21	30481464	30526957	181	10	10164	-0.42165	0.66336
+	C12orf29	12	88000491	88060160	118	18	10164	-0.42195	0.66347
-	ZNFX1	20	49235897	49313244	286	18	10164	-0.42221	0.66356
-	ZNF728	19	22964883	23038208	203	7	10164	-0.42257	0.6637
+	SGCA	17	50130975	50185932	122	21	10164	-0.42262	0.66371
-	SMCO3	12	14793723	14849182	157	24	10164	-0.42263	0.66372
+	CDC7	1	91465814	91535764	236	36	10164	-0.42271	0.66375
-	AHI1	6	135273972	135532765	367	24	10164	-0.42287	0.66381
-	TMEM115	3	50344749	50394508	78	16	10164	-0.42326	0.66395
-	ZSWIM7	17	15966560	16034692	164	10	10164	-0.42334	0.66397
-	ARPC3	12	110424890	110485411	94	9	10164	-0.42354	0.66405
-	TUSC2	3	50314909	50363238	64	10	10164	-0.42393	0.66419
-	CAMK2N1	1	20472391	20521235	77	13	10164	-0.42409	0.66425
+	SRGAP2	1	206168464	206474443	198	15	10164	-0.42412	0.66426
-	THBD	20	23035633	23084664	137	22	10164	-0.4246	0.66444
+	NAA60	16	3408654	3496963	410	33	10164	-0.42485	0.66453
-	SLC13A1	7	123103534	123234975	291	26	10164	-0.42517	0.66464
+	SSB	2	169763812	169822064	222	18	10164	-0.4253	0.66469
-	WDR82	3	52244422	52322876	149	20	10164	-0.42543	0.66474
-	DLG2	11	83445012	85663534	5722	198	10164	-0.42559	0.6648
+	ZNF572	8	124813552	124989511	572	50	10164	-0.42561	0.66481
+	TRIM58	1	247822199	247890138	229	19	10164	-0.42571	0.66484
-	NME9	3	138251437	138365149	81	6	10164	-0.42583	0.66488
+	PGA5	11	61206157	61261457	28	5	10164	-0.42618	0.66501
-	ZNF208	19	21956095	22045943	308	16	10164	-0.42629	0.66505
+	ARPC2	2	218182094	218264348	140	12	10164	-0.42638	0.66508
+	GPR107	9	130018706	130150169	305	19	10164	-0.42637	0.66508
-	CD63	12	55715443	55764673	51	13	10164	-0.42649	0.66513
-	RHBDF1	16	48059	111355	151	16	10164	-0.42668	0.66519
-	LHFPL2	5	78475215	78683895	372	19	10164	-0.42712	0.66535
+	SPINK5	5	147990688	148147366	653	18	10164	-0.42734	0.66544
-	MYH4	17	10433290	10504559	107	9	10164	-0.42749	0.66549
-	PDSS2	6	107142557	107494702	843	48	10164	-0.42767	0.66555

STRAND	SYMBOL	CHR	START	STOP	NSNPS	NPARAM	N	ZSTAT	P
+	GS1-259H13.	7	99562956	99620833	88	12	10164	-0.42778	0.6656
+	MTRNR2L1	17	22488111	22534665	48	2	10164	-0.42799	0.66567
-	DIRC1	2	188723738	188874420	384	14	10164	-0.42835	0.6658
-	FANCL	2	58149243	58276681	353	35	10164	-0.42853	0.66587
+	NUDT13	10	73075375	73141828	154	4	10164	-0.4286	0.66589
+	SCP2	1	52892229	53061617	629	21	10164	-0.4287	0.66593
-	SLC35B1	17	49690934	49743135	79	19	10164	-0.429	0.66604
+	NUP205	7	135522914	135658753	648	27	10164	-0.429	0.66604
-	ADAMTS2	5	179100851	179380430	589	66	10164	-0.42923	0.66612
-	TMEM129	4	1705952	1756357	230	16	10164	-0.42929	0.66615
-	C10orf55	10	73899969	73957777	137	28	10164	-0.42942	0.66619
+	C1orf122	1	37772801	37819454	94	12	10164	-0.42958	0.66625
-	NRIP3	11	8970576	9039049	150	17	10164	-0.43006	0.66642
+	TBC1D22B	6	37222704	37342970	255	20	10164	-0.43031	0.66651
+	GPR141	7	37648625	37752310	273	32	10164	-0.43037	0.66654
+	CAV1	7	116489785	116571185	217	17	10164	-0.43043	0.66656
+	RNF14	5	141931837	142000291	130	21	10164	-0.43087	0.66672
-	FNDC5	1	32852268	32907492	60	12	10164	-0.4309	0.66673
+	VSX2	14	74204472	74272738	303	30	10164	-0.43093	0.66674
+	CDS1	4	84547904	84661340	11	5	10164	-0.43098	0.66676
-	DOCK6	19	11189293	11297501	111	13	10164	-0.43102	0.66677
+	HTR3D	3	183996544	184049369	174	23	10164	-0.43127	0.66687
+	DHX8	17	43448917	43536796	165	12	10164	-0.43131	0.66688
+	TACC3	4	1686490	1755178	265	17	10164	-0.43155	0.66697
-	KPNA7	7	99163574	99254333	274	17	10164	-0.43156	0.66697
+	CPA2	7	130231863	130299798	123	14	10164	-0.43213	0.66718
-	VIMP	15	101260909	101312520	196	21	10164	-0.43227	0.66723
-	HIST4H4	12	14760720	14806131	149	25	10164	-0.43258	0.66734
+	ARL14	3	160642160	160688448	154	12	10164	-0.43295	0.66748
+	EMX1	2	72876148	72944891	67	10	10164	-0.43318	0.66756
-	LOC1053763	10	2412114	2536968	625	45	10164	-0.43351	0.66768
+	LOC728485	19	36738118	36787078	164	22	10164	-0.43354	0.66769
+	CCNYL1	2	207676540	207766174	214	9	10164	-0.43359	0.66771
+	LOC1053743	2	28323011	28375746	195	24	10164	-0.43372	0.66775
+	RNASE8	14	21022822	21068455	100	27	10164	-0.43379	0.66778
+	GPN1	2	27593648	27660846	131	20	10164	-0.43382	0.66779
+	WDR60	7	158821578	158956192	256	22	10164	-0.43386	0.66781
+	SCCPDH	1	246689076	246778137	272	23	10164	-0.43393	0.66783
+	SDHA	5	183223	274816	475	16	10164	-0.43401	0.66786
+	BAHD1	15	40404721	40478242	185	29	10164	-0.43403	0.66787
-	LECT1	13	52693264	52774812	160	19	10164	-0.43451	0.66804
+	ADCK1	14	77765065	77943954	780	48	10164	-0.43455	0.66806
-	PAXBP1	21	32723899	32806858	158	18	10164	-0.43458	0.66807
-	ANKRD31	5	75058297	75271887	641	21	10164	-0.43461	0.66808
-	LOC1053722	19	12369217	12421256	50	8	10164	-0.43466	0.66809
-	CSNK1A1L	13	37093260	37140664	121	6	10164	-0.43508	0.66825
-	SYT4	18	43257892	43312650	96	24	10164	-0.43526	0.66831
+	LRRN3	7	111056006	111135454	113	12	10164	-0.43535	0.66834
+	FAM35A	10	87060196	87201469	250	18	10164	-0.43556	0.66842
-	VEZF1	17	57961547	58023254	9	5	10164	-0.43557	0.66843
-	FBXO16	8	28418408	28525318	263	43	10164	-0.4356	0.66844
-	LOC1019279	11	9694617	9741902	262	11	10164	-0.43574	0.66849
-	PSG9	19	43243282	43304563	153	14	10164	-0.43579	0.66851
+	MTFP1	22	30390623	30439054	117	19	10164	-0.43607	0.66861
+	PDGFRA	4	54194097	54308245	278	16	10164	-0.43611	0.66862
-	MEAF6	1	37479960	37549819	190	26	10164	-0.43616	0.66864
-	C2orf54	2	240876048	240931889	313	21	10164	-0.43618	0.66865
-	EXPH5	11	108495431	108628765	393	48	10164	-0.4362	0.66866
+	WDR25	14	100341417	100540303	521	44	10164	-0.43633	0.6687
+	TMEM95	17	7320134	7367219	102	28	10164	-0.43705	0.66896
-	NAPEPLD	7	103089576	103184322	148	16	10164	-0.43765	0.66918
+	CREB3L3	19	4118601	4183054	79	19	10164	-0.43792	0.66928
+	CCL25	19	8017340	8072663	166	22	10164	-0.43797	0.6693

STRAND	SYMBOL	CHR	START	STOP	NSNPS	NPARAM	N	ZSTAT	P
+	KCTD20	6	36407767	36501143	237	13	10164	-0.43799	0.6693
-	LARS	5	146103026	146217787	296	3	10164	-0.4383	0.66942
+	TANGO6	16	68808553	69095182	699	42	10164	-0.43837	0.66944
+	TGFBR2	3	30571493	30704142	549	77	10164	-0.43847	0.66948
+	AMHR2	12	53388855	53441534	101	6	10164	-0.43876	0.66958
-	LOC1053743	4	5997972	6054180	25	4	10164	-0.43883	0.66961
+	DOK1	2	74513205	74567551	17	3	10164	-0.4389	0.66963
-	PLD5	1	242078387	242565544	1553	110	10164	-0.439	0.66967
+	CD81	11	2341177	2407419	295	22	10164	-0.43902	0.66968
-	GPR155	2	174421571	174522088	149	16	10164	-0.43912	0.66971
-	EIF2AK2	2	37094683	37192065	350	26	10164	-0.43916	0.66973
+	ARHGEF10L	1	17485197	17707875	579	68	10164	-0.4392	0.66974
+	PRR35	16	525422	575529	97	18	10164	-0.43951	0.66985
+	ZNRF2	7	30249307	30377692	181	13	10164	-0.44013	0.67008
-	TACR3	4	103579468	103754816	499	31	10164	-0.44037	0.67016
-	WDR24	16	674622	725649	145	20	10164	-0.44035	0.67016
-	KATNBL1	15	34130674	34245096	304	10	10164	-0.44041	0.67018
+	UBE2U	1	64151828	64277368	320	20	10164	-0.44059	0.67024
-	OR10G2	14	21623836	21669940	165	21	10164	-0.44067	0.67027
+	LEPR	1	65385652	65647493	764	38	10164	-0.44077	0.67031
+	PNP	14	20434379	20488006	203	34	10164	-0.44081	0.67032
-	YBX3	12	10689077	10758448	205	24	10164	-0.44092	0.67037
+	SMYD2	1	214246222	214347134	219	22	10164	-0.44127	0.67049
+	MAP1A	15	43482608	43541620	102	13	10164	-0.44138	0.67053
-	ARSA	22	50612754	50663173	41	10	10164	-0.44147	0.67056
+	BPIFB1	20	33248135	33319878	178	21	10164	-0.44155	0.67059
+	IFI27	14	94075733	94126699	208	25	10164	-0.44179	0.67068
+	ZNF90	19	20042994	20131168	295	17	10164	-0.44188	0.67071
-	ZNF529	19	36524518	36640411	331	24	10164	-0.4419	0.67072
-	SPATA31A3	9	66976301	67027550	3	2	10164	-0.44198	0.67075
+	GUCY1B3	4	155723973	155817642	166	21	10164	-0.44201	0.67076
+	FAM216B	13	42746550	42801549	173	19	10164	-0.44205	0.67077
-	HES2	1	6405232	6459664	111	10	10164	-0.44208	0.67078
+	TMEM151B	6	44235743	44289445	146	20	10164	-0.44216	0.67081
-	SMYD4	17	1769535	1864881	370	27	10164	-0.44272	0.67101
+	RNF2	1	185010419	185112608	200	18	10164	-0.44284	0.67106
+	C6orf201	6	4044206	4140766	232	14	10164	-0.44285	0.67106
+	BTG2	1	203270536	203319602	146	23	10164	-0.44332	0.67123
-	LBX2	2	74487517	74538316	26	2	10164	-0.44334	0.67124
+	GPR25	1	200837955	200884178	147	23	10164	-0.44356	0.67132
+	TULP4	6	158197236	158521828	1098	30	10164	-0.44359	0.67133
-	RSG1	1	16221687	16272452	90	7	10164	-0.44358	0.67133
-	OR5B17	11	58348125	58394069	134	10	10164	-0.4438	0.67141
+	NTPCR	1	232915536	232988799	227	18	10164	-0.44412	0.67152
-	UGT2A3	4	68918459	68986798	401	17	10164	-0.44426	0.67157
+	SH3BP2	4	2758023	2851096	348	33	10164	-0.44436	0.67161
+	GPR160	3	170002543	170095418	351	30	10164	-0.44436	0.67161
+	NPM1	5	171352648	171420884	202	16	10164	-0.44459	0.67169
-	OR7G3	19	9116012	9161950	140	14	10164	-0.44469	0.67173
+	PIAS1	15	68019234	68201459	497	25	10164	-0.44473	0.67174
+	SERTM1	13	36638912	36707839	14	3	10164	-0.44475	0.67175
-	ATP5J2	7	99448161	99501201	92	14	10164	-0.44477	0.67176
-	DCUN1D2	13	113445819	113525709	397	19	10164	-0.445	0.67184
+	RXFP2	13	31704542	31812872	347	30	10164	-0.44519	0.67191
-	ZNF423	16	49477531	49892919	1033	107	10164	-0.44522	0.67192
+	FRG2C	3	75611783	75677217	135	15	10164	-0.44537	0.67197
+	CPSF2	14	92086954	92174199	344	20	10164	-0.44546	0.67201
+	CLCNKA	1	15986991	16044050	334	17	10164	-0.4457	0.67209
-	DHX33	17	5430912	5504060	258	27	10164	-0.44579	0.67213
+	HTRA2	2	74494405	74543556	24	2	10164	-0.44589	0.67216
-	PCGF1	2	74495043	74542694	24	2	10164	-0.44589	0.67216
+	MOB1B	4	70866974	70998174	186	15	10164	-0.44597	0.67219
-	SYT7	11	61503716	61623396	93	28	10164	-0.44603	0.67221

STRAND	SYMBOL	CHR	START	STOP	NSNPS	NPARAM	N	ZSTAT	P
-	TNK2	3	195853364	195944009	167	27	10164	-0.44602	0.67221
-	TMEM128	4	4225542	4283242	11	3	10164	-0.44606	0.67222
+	CRYBB2	22	25176670	25241869	272	41	10164	-0.4461	0.67224
+	GIMAP2	7	150650706	150703641	213	23	10164	-0.44615	0.67226
-	IMMP1L	11	31422399	31544632	154	10	10164	-0.44622	0.67228
-	SRBD1	2	45378680	45646295	697	32	10164	-0.44636	0.67233
-	TRMT5	14	60961441	61016170	110	12	10164	-0.44636	0.67233
-	OR10AD1	12	48191772	48240682	226	15	10164	-0.4466	0.67242
-	EXD1	15	41172728	41266074	379	15	10164	-0.44666	0.67244
-	CLCC1	1	108919508	108998499	139	16	10164	-0.44698	0.67255
+	ADAMTSL3	15	83619086	84049842	1278	68	10164	-0.44703	0.67257
-	COL11A2	6	33152692	33228152	440	49	10164	-0.44705	0.67258
+	CD82	11	44529427	44630363	374	42	10164	-0.4471	0.6726
-	STX18	4	4408968	4577232	415	25	10164	-0.44725	0.67265
-	B3GAT1	11	134368504	134446987	109	22	10164	-0.44779	0.67285
+	TNF	6	31540567	31588336	292	55	10164	-0.44787	0.67287
+	LOC1053734	1	146903328	146970004	31	3	10164	-0.44787	0.67288
+	PARPBP	12	102085171	102207520	233	19	10164	-0.44799	0.67292
+	TMOD3	15	51794628	51922138	532	26	10164	-0.44814	0.67297
+	CDV3	3	133538590	133600274	164	21	10164	-0.44825	0.67301
-	ITPRIP	10	104299696	104373493	153	20	10164	-0.44823	0.67301
+	FIBIN	11	26959081	27007087	118	14	10164	-0.44843	0.67308
+	ID4	6	19802370	19852200	188	24	10164	-0.44861	0.67314
-	COPZ2	17	48016167	48083091	220	37	10164	-0.44904	0.6733
-	GPR183	13	99284535	99342495	174	30	10164	-0.44913	0.67333
-	HOXA9	7	27152438	27200530	70	18	10164	-0.44942	0.67343
-	ATP10A	15	25656794	25900158	881	104	10164	-0.44949	0.67346
-	TMEM2	9	71673366	71804466	394	23	10164	-0.44954	0.67348
-	KRT75	12	52414070	52469326	258	16	10164	-0.44964	0.67351
-	LOC285095	2	241883988	241938279	202	12	10164	-0.44969	0.67353
+	COL6A1	21	45946749	46015049	289	33	10164	-0.44993	0.67362
-	KRTAP29-1	17	41291826	41337851	144	10	10164	-0.45003	0.67365
+	PGPEP1	19	18305582	18379953	185	28	10164	-0.45051	0.67383
+	SLC27A4	9	128305560	128371470	112	12	10164	-0.45096	0.67399
-	GRM4	6	34008643	34190622	1062	77	10164	-0.45121	0.67408
-	TMEM242	6	157279022	157359259	5	3	10164	-0.45159	0.67422
+	IL6R	1	154370193	154479450	326	32	10164	-0.45172	0.67427
-	ANKRD29	18	23588926	23697892	252	42	10164	-0.45174	0.67427
-	SIGLEC8	19	51440997	51493454	242	22	10164	-0.45184	0.67431
+	SUSD2	22	24146476	24199106	84	10	10164	-0.452	0.67437
-	KPNA4	3	160484995	160600588	238	24	10164	-0.45215	0.67442
+	CCDC74A	2	131487641	131543670	176	1	10164	-0.45271	0.67462
+	CYC1	8	144060035	144107527	37	1	10164	-0.45283	0.67467
+	LRRC14B	5	156511	205353	213	22	10164	-0.45334	0.67485
+	S100A9	1	153322854	153371027	31	7	10164	-0.4534	0.67487
-	NSUN6	10	18535207	18694309	474	22	10164	-0.45347	0.67489
+	RAD54L	1	46212695	46288473	211	11	10164	-0.45354	0.67492
+	CNOT8	5	154822975	154886792	141	17	10164	-0.45388	0.67504
-	PRSS58	7	142242138	142293058	236	12	10164	-0.45396	0.67507
+	MAF1	8	144069402	144117612	31	1	10164	-0.45403	0.6751
+	E4F1	16	2188488	2245742	122	16	10164	-0.45435	0.67521
-	FBXW9	19	12678916	12731868	14	5	10164	-0.45436	0.67522
-	DQX1	2	74508131	74561281	20	2	10164	-0.45479	0.67537
-	TRAPPC6A	19	45152928	45213243	158	28	10164	-0.45493	0.67542
-	PIH1D1	19	49436293	49487028	139	15	10164	-0.45509	0.67548
-	GMFG	19	39318357	39371086	20	5	10164	-0.45548	0.67562
+	GYG1	3	148956408	149037669	184	27	10164	-0.45566	0.67568
-	TK2	16	66498003	66585412	171	9	10164	-0.45566	0.67568
-	IGH	14	105556277	106914844	1375	22	10164	-0.45577	0.67572
+	MARCH2	19	8378272	8449017	120	12	10164	-0.45595	0.67579
-	PTGES2	9	128110693	128163462	53	9	10164	-0.45611	0.67585
-	DNAJC7	17	41964005	42056371	108	11	10164	-0.45618	0.67587
+	C11orf58	11	16703601	16768354	107	17	10164	-0.4562	0.67588

STRAND	SYMBOL	CHR	START	STOP	NSNPS	NPARAM	N	ZSTAT	P
+	UTP14C	13	51989691	52043600	101	10	10164	-0.45625	0.67589
-	MBTPS1	16	84043761	84151943	379	33	10164	-0.45632	0.67592
-	YTHDF1	20	63185429	63251186	193	14	10164	-0.45633	0.67592
-	GUCA2A	1	42152691	42199724	115	20	10164	-0.45654	0.676
-	SLC25A11	17	4927130	4975167	117	26	10164	-0.45663	0.67603
+	OR1L6	9	122714848	122760783	27	11	10164	-0.4567	0.67606
+	MANEAL	1	37758820	37811606	125	13	10164	-0.45694	0.67614
+	CDA	1	20553951	20628908	390	28	10164	-0.45698	0.67616
-	CYP8B1	3	42862192	42911141	142	21	10164	-0.457	0.67616
+	BIRC7	20	63200883	63250507	151	13	10164	-0.45707	0.67619
+	MRPL15	8	54100221	54158514	92	7	10164	-0.45716	0.67622
+	PRSS35	6	83477475	83535704	253	29	10164	-0.45728	0.67626
+	MRPL33	2	27736717	27789741	58	10	10164	-0.45736	0.67629
-	SSR2	1	155999048	156055991	96	18	10164	-0.45738	0.6763
+	VAT1L	16	77753564	77990107	898	84	10164	-0.45746	0.67633
-	SPG11	15	44552696	44698678	270	22	10164	-0.45769	0.67641
+	SPRY1	4	123361791	123413760	84	14	10164	-0.45777	0.67644
+	IFT52	20	43555613	43657227	225	12	10164	-0.45778	0.67644
-	ADGRL1	19	14137737	14241248	13	5	10164	-0.45778	0.67645
+	LGALSL	2	64419193	64471383	67	11	10164	-0.45788	0.67648
+	AURKC	19	57195360	57245550	141	14	10164	-0.45801	0.67653
-	DERL2	17	5461251	5521512	264	24	10164	-0.45835	0.67665
+	ADM	11	10269980	10317402	148	16	10164	-0.45851	0.67671
-	COX7A2L	2	42340501	42404010	191	32	10164	-0.45867	0.67676
-	GSAP	7	77300751	77451408	469	39	10164	-0.45872	0.67678
-	MGARP	4	139256163	139315338	134	11	10164	-0.45894	0.67686
-	PTPRS	19	5195503	5375803	444	57	10164	-0.45971	0.67714
-	PZP	12	9132819	9243370	301	26	10164	-0.46004	0.67726
+	KNDC1	10	133125467	133236412	362	20	10164	-0.46009	0.67727
+	TBC1D3P6	1	15941131	16007206	231	23	10164	-0.46021	0.67732
+	FAM26D	6	116493999	116571123	240	20	10164	-0.46028	0.67734
-	ISM2	14	77464394	77533867	209	25	10164	-0.46062	0.67746
+	RNF151	16	1931823	1978975	145	31	10164	-0.46072	0.6775
+	GPAA1	8	144047621	144096216	40	1	10164	-0.46078	0.67752
+	WDR63	1	85027298	85143138	365	37	10164	-0.46088	0.67756
-	CRISP2	6	49666659	49749198	275	26	10164	-0.46097	0.67759
+	C8orf46	8	66458256	66528524	151	17	10164	-0.46098	0.67759
-	PHKG1	7	56070283	56128396	202	23	10164	-0.46118	0.67766
-	SEPT4	17	58510250	58575818	108	15	10164	-0.46122	0.67768
+	OLAH	10	15008896	15083852	339	27	10164	-0.46125	0.67769
-	SLC40A1	2	189550590	189615811	124	23	10164	-0.46129	0.6777
+	ASB16	17	44135706	44189083	89	8	10164	-0.4613	0.67771
-	NACAD	7	45070437	45123961	188	22	10164	-0.46153	0.67779
+	SEC61A1	3	128017369	128081683	228	15	10164	-0.46176	0.67787
-	STOM	9	121328988	121405304	191	26	10164	-0.46215	0.67801
-	ZBTB17	1	15931869	16011132	257	24	10164	-0.46224	0.67804
-	MROH8	20	37091226	37214588	265	12	10164	-0.46226	0.67805
-	FAM46A	6	81735730	81787711	166	39	10164	-0.46256	0.67816
-	NEK3	13	52122643	52194861	135	16	10164	-0.46284	0.67826
+	HMX3	10	123101051	123147731	138	29	10164	-0.46285	0.67827
+	HEPN1	11	124884250	124930677	142	24	10164	-0.46292	0.67829
-	IMPG2	3	101212546	101355575	322	22	10164	-0.46294	0.6783
-	UNC13D	17	75817225	75879717	98	9	10164	-0.46295	0.6783
+	NFATC1	18	79360772	79539323	487	40	10164	-0.46304	0.67833
+	CACNA1G	17	50526068	50639625	251	29	10164	-0.46303	0.67833
+	DMAP1	1	44178432	44230679	95	17	10164	-0.46362	0.67854
-	LRRC26	9	137158758	137205039	75	10	10164	-0.4637	0.67857
-	ISY1-RAB43	3	129077569	129196230	173	22	10164	-0.46405	0.67869
-	PLD6	17	17190995	17241332	105	11	10164	-0.4641	0.67871
-	TMEM143	19	48322356	48399237	174	21	10164	-0.46418	0.67874
-	ELOVL6	4	110039073	110234199	688	81	10164	-0.46434	0.6788
+	WFDC1	16	84259706	84339851	421	57	10164	-0.46452	0.67886
-	ZNF682	19	19994418	20074526	327	11	10164	-0.46532	0.67915

STRAND	SYMBOL	CHR	START	STOP	NSNPS	NPARAM	N	ZSTAT	P
+	MYRFL	12	69789569	69968724	611	40	10164	-0.46538	0.67917
-	CNOT1	16	58509946	58664886	339	15	10164	-0.46542	0.67918
-	C4orf3	4	119286419	119339445	245	12	10164	-0.46569	0.67928
-	THOC5	22	29498167	29588760	317	23	10164	-0.4657	0.67929
+	GET4	7	841554	906434	197	18	10164	-0.46581	0.67932
+	PPP1R9A	7	94872202	95306415	1100	72	10164	-0.46592	0.67936
-	FAXDC2	5	154807034	154885653	162	19	10164	-0.46597	0.67938
-	AVPR1B	1	206099849	206152048	101	5	10164	-0.4662	0.67946
-	GPRC5D	12	12930554	13014517	220	23	10164	-0.46622	0.67947
+	CD46	1	207717038	207805516	189	19	10164	-0.46631	0.6795
-	OPLAH	8	144041266	144099026	41	1	10164	-0.46653	0.67958
+	NR1D2	3	23910260	23990618	297	19	10164	-0.46671	0.67965
-	CD8A	2	86774605	86843396	38	5	10164	-0.46674	0.67966
-	CCDC115	2	130327933	130377810	20	3	10164	-0.4668	0.67968
+	C2CD4A	15	62031977	62080917	125	12	10164	-0.46685	0.6797
+	MND1	4	153309649	153425095	330	23	10164	-0.46694	0.67973
+	KRT7	12	52198170	52262667	127	20	10164	-0.46699	0.67975
-	ATXN7L3	17	44181805	44234701	71	9	10164	-0.46722	0.67983
-	CYTH1	17	78664047	78817342	245	16	10164	-0.46736	0.67988
+	PIK3R1	5	68180756	68311821	296	41	10164	-0.46739	0.67989
+	RPN2	20	37144051	37251623	271	12	10164	-0.4674	0.67989
+	GTF3C4	9	132635035	132700084	180	18	10164	-0.46749	0.67992
+	RNF26	11	119299500	119347314	136	21	10164	-0.46767	0.67999
+	TOMM20L	14	58360926	58427080	71	16	10164	-0.4679	0.68007
-	TRMT1L	1	185108085	185192098	167	15	10164	-0.46793	0.68008
-	MEX3D	19	1544669	1603058	44	7	10164	-0.46821	0.68018
-	LOC644070	17	9791473	9844311	3	1	10164	-0.46841	0.68025
+	GALNTL6	4	171778424	173050408	2579	110	10164	-0.46841	0.68026
-	CCDC12	3	46911730	47017018	196	27	10164	-0.46849	0.68028
+	SURF2	9	133321545	133371165	129	12	10164	-0.46876	0.68038
+	GIMAP5	7	150702348	150753649	163	16	10164	-0.46878	0.68039
+	TROAP	12	49287138	49341733	185	16	10164	-0.46886	0.68041
+	ADD1	4	2808727	2940076	267	34	10164	-0.46906	0.68049
+	FNDC1	6	159134397	159282108	587	18	10164	-0.46917	0.68053
+	ZNF35	3	44613741	44670791	54	9	10164	-0.46925	0.68056
-	APOA2	1	161212293	161258628	128	15	10164	-0.46979	0.68075
+	ROBO2	3	75871675	77659964	5235	252	10164	-0.46984	0.68076
+	CD3E	11	118269580	118326175	194	12	10164	-0.46992	0.68079
-	OSGEP	14	20437048	20490108	195	32	10164	-0.47008	0.68085
+	MBD3L5	19	6994930	7043011	80	14	10164	-0.47017	0.68088
-	YARS	1	32765239	32853032	116	8	10164	-0.47029	0.68092
-	POPDC2	3	119632055	119695590	239	17	10164	-0.47042	0.68097
+	AXDND1	1	179330718	179564735	1272	25	10164	-0.47063	0.68105
+	FUT2	19	48660971	48715934	120	15	10164	-0.47109	0.68121
-	NFATC2	20	51376957	51597858	440	70	10164	-0.47111	0.68122
+	RPH3A	12	112756538	112908881	233	31	10164	-0.47112	0.68122
-	PMEPA1	20	57638392	57746536	193	51	10164	-0.47124	0.68127
-	LOC1053767	15	75215896	75267485	91	40	10164	-0.47167	0.68142
-	TRIM9	14	50965261	51130704	673	63	10164	-0.47169	0.68143
+	FRS2	12	69435349	69589793	345	26	10164	-0.47203	0.68155
+	PNRC1	6	89045710	89095160	184	15	10164	-0.47208	0.68156
-	TSPAN2	1	115038011	115124500	265	26	10164	-0.47231	0.68165
-	GINS2	16	85667674	85723982	184	21	10164	-0.47251	0.68172
-	CCNJL	5	160241659	160348703	182	20	10164	-0.47254	0.68173
-	MAP4K2	11	64779137	64838248	134	32	10164	-0.47262	0.68176
+	ADRM1	20	62267056	62318862	173	26	10164	-0.47278	0.68181
+	BTNL3	5	180953845	181016727	40	6	10164	-0.47301	0.6819
-	C14orf105	14	57459300	57528891	187	17	10164	-0.47333	0.68201
-	GTSF1L	20	43716161	43762012	68	17	10164	-0.4734	0.68204
-	HIGD1A	3	42772908	42839535	155	26	10164	-0.47344	0.68205
-	OR10J5	1	159525078	159571007	137	18	10164	-0.47356	0.68209
-	BLOC1S2	10	100263278	100321712	143	19	10164	-0.47417	0.68231
+	ABAT	16	8639587	8794575	670	75	10164	-0.47431	0.68236

STRAND	SYMBOL	CHR	START	STOP	NSNPS	NPARAM	N	ZSTAT	P
+	ATP2B4	1	203591787	203754081	360	40	10164	-0.47461	0.68247
-	OLFML2B	1	161973192	162059465	248	53	10164	-0.47476	0.68252
-	TPP1	11	6602766	6654461	127	20	10164	-0.47479	0.68253
+	ABCG4	11	119114040	119172666	197	19	10164	-0.47514	0.68266
-	MEOX1	17	43630389	43696954	169	23	10164	-0.47519	0.68267
+	SOX11	2	5657667	5711385	105	18	10164	-0.47523	0.68269
-	GFRA1	10	116046925	116308614	375	35	10164	-0.47534	0.68273
-	CATSPER2	15	43620562	43683845	83	10	10164	-0.47541	0.68275
+	ERICH2	2	170735541	170808971	219	15	10164	-0.47542	0.68276
+	CARF	2	202877218	202997857	247	16	10164	-0.47556	0.6828
+	LOC1053750	6	41215077	41263515	243	19	10164	-0.47556	0.68281
+	KDM4E	11	94990258	95037596	70	13	10164	-0.47591	0.68293
-	DACT2	6	168282830	168354754	293	29	10164	-0.47592	0.68294
-	SEPT8	5	132740817	132813284	148	19	10164	-0.47616	0.68302
-	JSRP1	19	2242251	2291423	144	22	10164	-0.47669	0.68321
-	SAR1A	10	70140204	70205529	215	21	10164	-0.47673	0.68322
+	SEC24A	5	134613785	134737911	209	26	10164	-0.47688	0.68328
+	CLEC17A	19	14547914	14621144	128	24	10164	-0.47691	0.68329
-	TNFRSF11B	8	118913557	118987144	242	24	10164	-0.4771	0.68335
+	C1orf64	1	15969236	16016695	221	20	10164	-0.47712	0.68336
-	PRKAG1	12	48992272	49053980	57	8	10164	-0.47714	0.68337
+	CLCA4	1	86512076	86590758	131	19	10164	-0.47714	0.68337
+	EPB41L1	20	36029891	36242799	298	26	10164	-0.4773	0.68343
-	PPP1R12A	12	79763563	79970455	219	7	10164	-0.47748	0.68349
-	POU6F1	12	51176936	51252693	191	31	10164	-0.47756	0.68352
-	CITED2	6	139362255	139409650	111	24	10164	-0.47774	0.68358
+	SLC6A1	3	10957729	11049249	286	55	10164	-0.47789	0.68364
-	NPTN	15	73550003	73668412	241	26	10164	-0.47789	0.68364
+	ST6GALNAC	1	76039704	76644601	1593	124	10164	-0.47821	0.68375
+	ANK2	4	112783083	113393740	1029	95	10164	-0.47857	0.68388
+	HPCA	1	32851087	32904646	61	12	10164	-0.47877	0.68395
+	RPS6KC1	1	213016233	213283465	406	22	10164	-0.47884	0.68397
-	FZD4	11	86935675	86990398	125	15	10164	-0.47887	0.68398
-	FEZ1	11	125435745	125531310	220	26	10164	-0.47889	0.68399
+	KCNJ12	17	21341387	21429872	73	10	10164	-0.47899	0.68403
-	RAPGEF1	9	131566770	131774944	659	40	10164	-0.47902	0.68404
+	AMER3	2	130720504	130778134	3	2	10164	-0.47905	0.68405
+	ZG16B	16	2795172	2842284	35	2	10164	-0.47907	0.68406
-	ANPEP	15	89774895	89849854	133	12	10164	-0.47951	0.68421
+	LOC1053719	17	35337833	35384437	128	20	10164	-0.47953	0.68422
+	KSR1	17	27421464	27636441	400	30	10164	-0.4796	0.68424
+	ZNF684	1	40496155	40558169	228	17	10164	-0.47965	0.68426
+	MON2	12	62431817	62607583	484	26	10164	-0.47968	0.68427
+	BPGM	7	134611779	134689816	294	19	10164	-0.48018	0.68445
-	GGCX	2	85534855	85596534	194	20	10164	-0.48026	0.68448
-	SCARB1	12	124767628	124898973	449	58	10164	-0.48028	0.68449
-	LOC1053707	14	92498774	92549640	175	39	10164	-0.48034	0.68451
+	SMAP2	1	40309844	40433326	233	37	10164	-0.4805	0.68457
+	CCDC103	17	44864712	44913679	151	15	10164	-0.48051	0.68457
-	STAT3	17	42303324	42423503	312	20	10164	-0.48114	0.68479
+	SENP3	17	7526992	7581969	197	32	10164	-0.48117	0.6848
-	KRT15	17	41503745	41557413	34	8	10164	-0.48159	0.68495
-	ZNF616	19	52104400	52174938	183	28	10164	-0.48188	0.68506
+	TTC16	9	127681064	127741600	130	17	10164	-0.48196	0.68508
-	FEZF1	7	122291309	122346853	111	13	10164	-0.48225	0.68519
-	SLC27A5	19	58488333	58547065	70	11	10164	-0.48262	0.68532
+	SP140	2	230167742	230326616	528	41	10164	-0.48288	0.68541
-	ZNF16	8	144920358	144985888	173	13	10164	-0.48306	0.68547
+	MALRD1	10	19011931	19744478	2820	92	10164	-0.48343	0.6856
+	VAPB	20	58354119	58461101	240	23	10164	-0.48364	0.68568
-	C6orf15	6	31101223	31147555	828	55	10164	-0.48364	0.68568
+	PPM1A	14	60210752	60309087	238	21	10164	-0.48411	0.68585
-	NLRP12	19	53783584	53859403	47	5	10164	-0.48442	0.68595

STRAND	SYMBOL	CHR	START	STOP	NSNPS	NPARAM	N	ZSTAT	P
+	LOC1053724	19	58326589	58377615	93	10	10164	-0.4846	0.68602
-	PYCARD	16	31191486	31237776	21	4	10164	-0.48493	0.68614
-	ENKUR	10	24971979	25097279	188	9	10164	-0.48521	0.68624
+	FAM83B	6	54811643	54955099	593	39	10164	-0.48535	0.68628
+	TMEM140	7	135113014	135176215	189	12	10164	-0.48535	0.68629
+	KAZALD1	10	101026141	101078131	114	28	10164	-0.48581	0.68645
-	SRD5A2	2	31512488	31735784	527	33	10164	-0.48591	0.68648
-	LOC1027246	6	92634473	92679805	128	17	10164	-0.48617	0.68658
-	TMIGD3	1	111473348	111599003	351	42	10164	-0.48652	0.6867
+	MAPK15	8	143681302	143732463	117	18	10164	-0.48652	0.6867
+	HILPDA	7	128420830	128468418	135	18	10164	-0.4866	0.68673
-	C12orf76	12	110018042	110117224	135	19	10164	-0.4866	0.68673
-	STAT5B	17	42189177	42311460	205	22	10164	-0.48664	0.68674
-	RPS10-NUDT	6	34277194	34461125	795	41	10164	-0.48682	0.68681
+	LAP3	4	17542304	17617967	231	16	10164	-0.48686	0.68682
+	TECTB	10	112248345	112315035	121	16	10164	-0.48707	0.68689
+	TIRAP	11	126246893	126304933	146	22	10164	-0.48704	0.68689
-	NR0B2	1	26901484	26949076	43	9	10164	-0.48757	0.68707
-	TPT1	13	45323471	45376284	108	11	10164	-0.48764	0.6871
+	TMEM138	11	61327001	61386502	59	8	10164	-0.48778	0.68715
+	INSL4	9	5196419	5243967	114	9	10164	-0.48782	0.68716
+	ACOT11	1	54513134	54644744	413	44	10164	-0.48823	0.68731
+	PPP6R3	11	68425718	68625334	544	31	10164	-0.48829	0.68733
-	BDH2	4	103067625	103134867	146	17	10164	-0.48847	0.68739
-	MCMBP	10	119819404	119908628	338	14	10164	-0.48847	0.68739
-	SDR16C5	8	56290011	56355776	171	25	10164	-0.48848	0.6874
-	TRIM4	7	99880407	99954600	150	10	10164	-0.4887	0.68747
-	GSTT2B	22	23947414	23996234	54	9	10164	-0.48907	0.6876
-	SNAP91	6	83542885	83744408	450	35	10164	-0.48908	0.68761
-	ZNF217	20	53557065	53643969	138	26	10164	-0.48913	0.68763
+	C6orf165	6	87372972	87474473	268	15	10164	-0.48916	0.68764
-	OR51B2	11	5313298	5359352	300	23	10164	-0.48923	0.68766
-	LAMA5	20	62299060	62402320	453	38	10164	-0.48945	0.68774
-	TMEFF2	2	191939046	192229940	387	44	10164	-0.48966	0.68781
-	HPS1	10	98406198	98481963	278	26	10164	-0.48985	0.68788
-	VTI1B	14	67641149	67709885	214	26	10164	-0.48984	0.68788
-	ZBTB5	9	37428102	37500410	203	20	10164	-0.48988	0.68789
+	PRH2	12	10894235	10944845	97	3	10164	-0.48997	0.68792
+	SH3BGRL2	6	79596283	79713670	388	43	10164	-0.49004	0.68795
-	HDC	15	50231945	50301091	230	30	10164	-0.49015	0.68799
+	DIS3L	15	66258256	66343898	210	31	10164	-0.49015	0.68799
+	LRRC3B	3	26587809	26720776	362	33	10164	-0.49029	0.68804
+	STK32B	4	5016211	5510998	1933	69	10164	-0.49046	0.6881
+	ZNF862	7	149803420	149877479	69	5	10164	-0.49053	0.68812
-	PQLC1	18	79892420	79986664	195	19	10164	-0.49063	0.68815
+	OR56B1	11	5701448	5747538	187	15	10164	-0.49082	0.68822
-	FCRL1	1	157782249	157855150	150	21	10164	-0.49084	0.68823
-	TP53	17	7658402	7722550	169	28	10164	-0.4909	0.68825
-	TRAP1	16	3648037	3752597	266	47	10164	-0.49091	0.68825
-	CRISP3	6	49717376	49779455	178	20	10164	-0.49105	0.6883
-	FASTK	7	151066620	151115883	115	17	10164	-0.49137	0.68842
-	CYP11A1	15	74327762	74402740	275	40	10164	-0.49168	0.68853
-	BCL2A1	15	79950890	80006301	139	19	10164	-0.49176	0.68855
-	SLC9A9	3	143255221	143883531	1796	160	10164	-0.49185	0.68859
-	SLC25A23	19	6426081	6495221	16	2	10164	-0.49205	0.68866
+	ARF1	1	228047660	228109212	236	24	10164	-0.49209	0.68867
-	MSS51	10	73413579	73468561	42	7	10164	-0.4923	0.68875
-	SFPQ	1	35166380	35228158	176	16	10164	-0.49236	0.68877
-	MYL7	7	44128864	44176388	120	20	10164	-0.49237	0.68877
+	HSD17B12	11	43645593	43866619	603	25	10164	-0.49243	0.68879
-	JMJD1C	10	63157221	63501001	849	31	10164	-0.49245	0.6888
-	KRT85	12	52350006	52402525	180	14	10164	-0.49256	0.68884
+	RBM15	1	110303426	110383218	221	23	10164	-0.49267	0.68888

STRAND	SYMBOL	CHR	START	STOP	NSNPS	NPARAM	N	ZSTAT	P
-	LOC1053756	7	129599399	129647722	150	19	10164	-0.4927	0.68889
+	TF	3	133626998	133789006	570	53	10164	-0.4929	0.68896
+	KRTAP9-9	17	41220384	41266364	121	11	10164	-0.49309	0.68903
-	MYH1	17	10482307	10553542	124	10	10164	-0.49328	0.68909
+	QPCT	2	37309610	37383322	161	18	10164	-0.4936	0.68921
-	KRTCAP2	1	155159408	155208328	70	11	10164	-0.4936	0.68921
+	MYO7A	11	77093214	77225241	332	31	10164	-0.49369	0.68924
+	ITGBL1	13	101417593	101730857	1016	63	10164	-0.49378	0.68927
+	CDC20	1	43323955	43373203	69	11	10164	-0.49387	0.6893
+	DAP3	1	155654089	155749010	160	20	10164	-0.49395	0.68933
-	TPRA1	3	127563064	127630059	64	17	10164	-0.4941	0.68938
-	KRTAP16-1	17	41297700	41344253	129	10	10164	-0.49422	0.68942
+	CMTR1	6	37398131	37492827	200	24	10164	-0.49448	0.68952
+	EPHX4	1	91994976	92073536	114	15	10164	-0.49449	0.68952
+	DUT	15	48296100	48353373	92	10	10164	-0.49468	0.68959
+	KDM4D	11	94938681	95009514	137	15	10164	-0.49532	0.68981
-	SSX2IP	1	84633707	84725757	225	24	10164	-0.49586	0.69
+	PEX26	22	18042920	18101031	66	12	10164	-0.49609	0.69009
-	GJC1	17	44783954	44865811	207	28	10164	-0.49626	0.69015
+	NNT	5	43567689	43715566	297	11	10164	-0.49672	0.69031
-	ELMSAN1	14	73705122	73825285	353	54	10164	-0.49689	0.69037
+	TNFSF11	13	42527736	42618013	244	24	10164	-0.49702	0.69041
-	HTR2A	13	46821542	46932076	322	32	10164	-0.49725	0.69049
+	THAP6	4	75474860	75554102	177	18	10164	-0.49752	0.69059
+	ACVR2B	3	38419299	38503142	178	15	10164	-0.49762	0.69062
+	DRG1	22	31364553	31444186	196	32	10164	-0.49775	0.69067
-	IMMP2L	7	110652645	111597531	1629	90	10164	-0.49793	0.69073
-	ZNF428	19	43597224	43654862	148	25	10164	-0.49798	0.69075
-	DHRS7C	17	9761434	9826577	28	4	10164	-0.49799	0.69075
-	TRUB2	9	128299117	128357418	96	10	10164	-0.49829	0.69086
-	PRSS54	16	58269997	58330047	265	20	10164	-0.49872	0.69101
+	PRPF4B	6	3986329	4074983	195	14	10164	-0.49873	0.69102
-	TAS2R13	12	10897926	10944562	92	3	10164	-0.49874	0.69102
-	RAPGEF6	5	131413921	131670236	410	19	10164	-0.49879	0.69104
-	TEX14	17	58546677	58727079	356	21	10164	-0.4988	0.69104
-	KRR1	12	75487639	75546638	135	14	10164	-0.49891	0.69108
+	GALNTL5	7	151921379	152029934	427	48	10164	-0.49897	0.6911
+	CMIP	16	81410170	81721762	1178	125	10164	-0.49908	0.69114
-	TRANK1	3	36816817	37025981	345	19	10164	-0.49924	0.6912
-	DUSP2	2	96133169	96180440	111	31	10164	-0.49955	0.6913
+	SETDB2	13	49409056	49505003	315	34	10164	-0.49981	0.6914
-	CSNK1E	22	38280691	38353084	178	50	10164	-0.49987	0.69142
-	LOC1053769	19	27510104	27677524	922	25	10164	-0.50013	0.69151
+	SPP2	2	234015690	234087134	284	34	10164	-0.50033	0.69158
-	AGBL4	1	48522834	50058954	2441	59	10164	-0.50037	0.69159
-	PGLYRP3	1	153287589	153347975	81	7	10164	-0.50061	0.69168
-	AP5B1	11	65763898	65815591	191	40	10164	-0.50075	0.69173
+	SERPINB13	18	63552202	63609199	153	8	10164	-0.50082	0.69175
+	PPWD1	5	65528236	65597549	198	12	10164	-0.5009	0.69178
+	AEBP1	7	44069320	44124565	133	23	10164	-0.50104	0.69183
-	POMZP3	7	76599986	76662303	54	7	10164	-0.50106	0.69183
-	OR5B2	11	58412265	58458313	187	10	10164	-0.50103	0.69183
+	OR9Q2	11	58155434	58201518	144	22	10164	-0.5012	0.69188
-	RAB3IL1	11	61887240	61981311	231	39	10164	-0.50122	0.69189
+	C2orf82	2	232834119	232888695	175	19	10164	-0.50133	0.69193
-	LIFR	5	38464963	38630405	173	13	10164	-0.50151	0.69199
+	LRFN4	11	66821480	66870475	66	28	10164	-0.50176	0.69208
+	C8orf31	8	143004209	143064303	22	8	10164	-0.50191	0.69213
-	SIK3	11	116833402	117133453	1113	34	10164	-0.502	0.69217
+	XPR1	1	180597010	180900279	410	26	10164	-0.5022	0.69224
-	CHRNA4	20	63333310	63396396	160	22	10164	-0.50224	0.69225
-	F5	1	169501954	169621531	469	36	10164	-0.5023	0.69227
-	ZCCHC10	5	132986985	133061617	179	12	10164	-0.5024	0.69231

STRAND	SYMBOL	CHR	START	STOP	NSNPS	NPARAM	N	ZSTAT	P
-	ZNF669	1	247089962	247139400	132	9	10164	-0.50251	0.69235
-	SLC6A12	12	177458	249205	240	46	10164	-0.50264	0.69239
-	FRMD3	9	83232990	83573433	1246	86	10164	-0.50293	0.69249
+	KCTD7	7	66593881	66653229	216	22	10164	-0.50305	0.69254
-	LGMN	14	92693807	92783702	223	48	10164	-0.50326	0.69261
-	SLC45A3	1	205647851	205715502	176	29	10164	-0.50326	0.69261
+	CLDN25	11	113744796	113790485	108	19	10164	-0.50328	0.69262
-	MYH13	17	10290866	10408005	299	40	10164	-0.50355	0.69271
-	DEPDC1	1	68464152	68532221	197	21	10164	-0.50366	0.69275
+	CLEC9A	12	9995677	10076030	225	35	10164	-0.50368	0.69276
-	YAF2	12	42147104	42273352	285	28	10164	-0.50418	0.69293
-	ACAA2	18	49773504	49848881	289	37	10164	-0.50426	0.69296
+	NDUFV1	11	67571852	67622541	183	18	10164	-0.50437	0.693
-	FCN1	9	134899585	134952960	195	32	10164	-0.50453	0.69305
+	CYBRD1	2	171487247	171568133	378	36	10164	-0.50453	0.69306
-	AUP1	2	74516648	74564897	16	3	10164	-0.50456	0.69307
+	MPP5	14	67206295	67346061	485	17	10164	-0.50471	0.69312
+	CTAGE15	7	143536801	143584387	4	2	10164	-0.50482	0.69316
-	ANAPC5	12	121298245	121389209	233	25	10164	-0.50491	0.69319
+	SPRED3	19	38353741	38409884	225	36	10164	-0.50518	0.69328
+	NEDD1	12	96872223	96963696	262	23	10164	-0.5053	0.69333
-	PLAC8L1	5	146074149	146139383	161	3	10164	-0.5055	0.6934
-	TNFRSF10A	8	23181457	23260167	357	45	10164	-0.50553	0.69341
-	SRPR	11	126252919	126303982	130	20	10164	-0.50556	0.69342
+	MSRB2	10	23060498	23132013	193	32	10164	-0.50576	0.69349
+	CALCB	11	15038600	15088631	138	10	10164	-0.50605	0.69359
+	DYNC2H1	11	103074431	103489863	1912	42	10164	-0.50619	0.69364
-	TRAK2	2	201367207	201486596	331	34	10164	-0.50648	0.69374
-	MUC16	19	8838844	9016342	436	35	10164	-0.50659	0.69378
-	POTEI	2	130452245	130544355	1	1	10164	-0.50661	0.69378
-	LOC1053770	3	32804869	32852341	119	16	10164	-0.50665	0.6938
+	SYT14	1	209903174	210174291	616	27	10164	-0.50687	0.69388
-	DPY19L2	12	63548913	63704201	530	23	10164	-0.50702	0.69393
+	KDM4C	9	6685863	7185648	2214	119	10164	-0.50729	0.69402
-	KCNMB3	3	179229749	179302050	281	32	10164	-0.50729	0.69402
-	ADA	20	44609522	44687233	194	35	10164	-0.50744	0.69408
+	JAKMIP3	10	132001107	132194809	903	70	10164	-0.50755	0.69412
-	MTMR4	17	58479532	58553110	99	14	10164	-0.50777	0.69419
+	ATP9B	18	79012594	79388283	1052	25	10164	-0.50783	0.69421
-	LOC1053722	19	12677444	12723820	14	4	10164	-0.50786	0.69422
+	EPHA8	1	22528508	22613594	319	35	10164	-0.50813	0.69432
-	LAPTM4A	2	20022650	20087028	239	30	10164	-0.50822	0.69435
-	KCNJ13	2	232755802	232811565	133	16	10164	-0.50841	0.69442
-	HERC2	15	28101037	28357172	206	15	10164	-0.50842	0.69442
-	ROBO4	11	124874218	124932935	168	26	10164	-0.50846	0.69443
-	CD72	9	35599979	35681857	246	44	10164	-0.50858	0.69448
-	KANSL1L	2	210010711	210206344	195	30	10164	-0.50865	0.6945
+	ACOT6	14	73577296	73629908	217	27	10164	-0.50897	0.69461
-	ERGIC2	12	29330646	29416210	305	23	10164	-0.50899	0.69462
-	CGB1	19	49025569	49071934	157	27	10164	-0.50901	0.69463
+	SCAMP4	19	1870374	1936013	192	25	10164	-0.50923	0.6947
+	SIN3B	19	16794387	16890355	65	8	10164	-0.50937	0.69475
-	SURF6	9	133320707	133371210	132	13	10164	-0.50957	0.69482
-	XPNPEP1	10	109854766	109958553	222	23	10164	-0.50956	0.69482
-	EFHB	3	19869472	19982456	333	25	10164	-0.50956	0.69482
-	CCDC85C	14	99501266	99639390	215	7	10164	-0.50985	0.69492
+	SCN2A	2	165095149	165402310	889	45	10164	-0.50988	0.69493
-	ASB8	12	48137788	48192594	172	25	10164	-0.51083	0.69526
-	HES5	1	2518745	2565245	85	14	10164	-0.51084	0.69527
+	ZNF662	3	42870910	42929333	132	18	10164	-0.51087	0.69528
+	SLC4A10	2	161589335	161995276	807	21	10164	-0.51094	0.6953
+	SKA1	18	50340022	50404168	287	14	10164	-0.51101	0.69533
-	ZNF541	19	47510685	47608238	151	20	10164	-0.51109	0.69536

STRAND	SYMBOL	CHR	START	STOP	NSNPS	NPARAM	N	ZSTAT	P
+	HMCN1	1	185699381	186200953	545	29	10164	-0.51124	0.69541
-	LAMP3	3	183112215	183197879	302	24	10164	-0.51132	0.69544
-	ACSL1	4	184745595	184861114	314	34	10164	-0.51137	0.69545
+	DMTF1	7	87117361	87206332	252	18	10164	-0.51139	0.69546
-	CABP2	11	67508947	67558614	119	18	10164	-0.51193	0.69565
+	TTC21A	3	39072661	39148994	207	21	10164	-0.51203	0.69569
+	LOC1019282	7	29685497	29752604	207	22	10164	-0.51205	0.69569
+	PDZRN4	12	41152850	41584745	1848	61	10164	-0.51216	0.69573
+	NUDT9	4	87387557	87469454	238	28	10164	-0.51241	0.69582
+	PSMD4	1	151219700	151277479	79	10	10164	-0.51256	0.69587
+	POMT1	9	131467895	131533806	173	11	10164	-0.51326	0.69612
-	PPIL1	6	36844829	36910024	282	33	10164	-0.51335	0.69615
+	CACNG5	17	66800117	66897296	317	50	10164	-0.51342	0.69617
+	LOC1053727	20	33105337	33173020	226	20	10164	-0.51391	0.69634
+	FAM57A	17	697607	752835	219	29	10164	-0.51414	0.69642
-	PPIL3	2	200860907	200924333	130	29	10164	-0.51447	0.69654
-	LOC1053699	12	58313535	58367034	161	22	10164	-0.51497	0.69671
-	EDN2	1	41468775	41519683	185	30	10164	-0.51501	0.69673
+	CCDC184	12	48148583	48195926	168	23	10164	-0.51543	0.69687
+	ZCCHC7	9	37085169	37368149	423	26	10164	-0.51548	0.69689
+	GRWD1	19	48410773	48463907	130	20	10164	-0.51554	0.69691
-	C21orf62	21	32780673	32849181	160	20	10164	-0.51557	0.69692
-	POFUT2	21	45253928	45322899	226	17	10164	-0.51574	0.69698
+	MAPK14	6	35992635	36121236	456	35	10164	-0.51662	0.69729
-	LPAR2	19	19613655	19663930	128	29	10164	-0.51663	0.69729
+	P2RX7	12	121097819	121196551	446	19	10164	-0.5168	0.69735
+	VN1R5	1	247221072	247267145	137	14	10164	-0.51694	0.6974
-	SOX30	5	157614796	157707756	252	21	10164	-0.51738	0.69756
-	MBOAT2	2	8842690	9038747	211	13	10164	-0.51741	0.69756
-	ZNF852	3	44488970	44545640	58	9	10164	-0.51751	0.6976
-	GDPD5	11	75424640	75560920	361	35	10164	-0.51775	0.69769
-	TAS2R43	12	11081287	11127313	145	3	10164	-0.51776	0.69769
-	SLC9C1	3	112130898	112397986	1284	53	10164	-0.51802	0.69778
+	ZEB1	10	31283783	31539814	406	21	10164	-0.51809	0.6978
+	HRH2	5	175622796	175720756	89	12	10164	-0.51825	0.69786
+	GLCE	15	69125560	69282207	263	19	10164	-0.5184	0.69791
+	AUTS2	7	69563475	70803068	2758	79	10164	-0.51864	0.69799
-	SYMPK	19	45805442	45898290	350	25	10164	-0.51876	0.69803
+	KCNJ9	1	160044953	160100563	112	16	10164	-0.5188	0.69805
+	ACKR2	3	42774472	42877283	258	32	10164	-0.51891	0.69809
-	NOP2	12	6546870	6603332	89	11	10164	-0.51922	0.69819
+	BPIFA2	20	33126836	33191417	178	14	10164	-0.51928	0.69822
-	PYDC1	16	31205962	31252093	27	5	10164	-0.5193	0.69822
+	FZD10	12	130127459	130175740	240	45	10164	-0.51935	0.69824
+	SOAT2	12	53067504	53134539	169	17	10164	-0.51946	0.69828
-	DCAKD	17	45013338	45096141	321	27	10164	-0.51946	0.69828
-	FAM72A	1	206176182	206239919	1	1	10164	-0.51959	0.69833
-	KCNJ10	1	160027467	160105261	146	23	10164	-0.51974	0.69838
-	DUS3L	19	5775140	5826238	160	20	10164	-0.51995	0.69845
-	SYBU	8	109564176	109726791	454	28	10164	-0.51999	0.69847
+	CFHR1	1	196784731	196842189	8	3	10164	-0.52023	0.69855
+	PDCD1LG2	9	5475491	5581282	375	43	10164	-0.52023	0.69855
+	DUSP5	10	110462867	110521544	166	22	10164	-0.5205	0.69864
-	CCDC88A	2	55277842	55454921	628	28	10164	-0.52056	0.69866
+	OR9G1	11	56665388	56711305	163	23	10164	-0.52056	0.69866
+	RIC8A	11	172511	225175	114	15	10164	-0.52071	0.69872
+	ARHGEF33	2	38884363	38985449	213	31	10164	-0.52081	0.69875
+	CAPNS2	16	55531672	55577680	122	9	10164	-0.52088	0.69877
+	CLDN11	3	170383865	170444691	171	32	10164	-0.52106	0.69884
+	C3orf79	3	153449495	153512697	209	19	10164	-0.52112	0.69886
-	PRIM1	12	56721580	56787362	85	13	10164	-0.5212	0.69889
+	METTL2A	17	62388885	62460093	105	8	10164	-0.52122	0.69889
+	TNFSF12	17	7514058	7567890	222	28	10164	-0.52148	0.69899

STRAND	SYMBOL	CHR	START	STOP	NSNPS	NPARAM	N	ZSTAT	P
+	BHLHA9	17	1235564	1281271	39	8	10164	-0.5216	0.69902
-	CLDN24	4	183311764	183357426	132	22	10164	-0.52187	0.69912
-	DSC1	18	31119236	31197856	202	14	10164	-0.52193	0.69914
+	G3BP1	5	151736915	151821356	196	17	10164	-0.52196	0.69915
+	BTN1A1	6	26464306	26520425	228	31	10164	-0.52234	0.69928
-	SDAD1	4	75939906	76025962	205	21	10164	-0.52239	0.6993
-	LRRC59	17	50371233	50432553	181	15	10164	-0.52282	0.69945
-	WDR49	3	167468684	167688501	482	43	10164	-0.52282	0.69945
+	WRAP53	17	7651071	7713502	146	25	10164	-0.52299	0.69951
+	ZNF774	15	90317245	90371483	297	19	10164	-0.52306	0.69953
+	DNALI1	1	37521919	37576857	124	20	10164	-0.52331	0.69962
-	MYH8	17	10380325	10456950	133	13	10164	-0.52404	0.69987
-	YY1AP1	1	155649442	155724032	124	19	10164	-0.52403	0.69987
-	GPAT2	2	96011946	96070245	42	12	10164	-0.52414	0.69991
-	FANCD2OS	3	10071320	10143339	314	16	10164	-0.52426	0.69995
+	LOC1019297	22	30632582	30679016	138	19	10164	-0.52438	0.69999
-	VPS9D1	16	89697133	89756272	354	28	10164	-0.52479	0.70014
+	PPP2R5D	6	42949499	43022345	210	20	10164	-0.52482	0.70015
-	TEKT1	17	6787993	6866741	241	31	10164	-0.52492	0.70018
-	DDB1	11	61289447	61368212	79	5	10164	-0.52518	0.70027
-	ZNF598	16	1987652	2044821	139	16	10164	-0.52521	0.70028
+	BCL7A	12	121986886	122072044	271	31	10164	-0.52527	0.7003
-	FIGLA	2	70767310	70825643	238	26	10164	-0.52533	0.70032
+	CAV3	3	8698800	8756765	112	23	10164	-0.52546	0.70037
-	SEMA3C	7	80732538	80954351	472	50	10164	-0.52546	0.70037
-	NACA	12	56702427	56761275	80	9	10164	-0.52551	0.70038
+	PMS1	2	189749085	189887629	351	28	10164	-0.52565	0.70043
+	UBALD2	17	76230205	76281298	128	14	10164	-0.52562	0.70043
+	VAV1	19	6737668	6867366	139	22	10164	-0.52567	0.70044
-	SERPINB4	18	63627259	63679319	38	8	10164	-0.52577	0.70048
+	POTEE	2	131176134	131275448	51	4	10164	-0.52602	0.70056
-	KLHDC8A	1	205326065	205392090	169	21	10164	-0.52611	0.70059
+	PRDM13	6	99571774	99625578	151	28	10164	-0.52624	0.70064
-	SERPINB6	6	2938159	3007165	321	32	10164	-0.52634	0.70067
-	C1orf174	1	3879133	3935293	210	32	10164	-0.52636	0.70068
-	UGT3A2	5	36025017	36101921	263	34	10164	-0.5266	0.70077
-	PHF21A	11	45919319	46156434	355	28	10164	-0.52669	0.7008
+	RPL37A	2	216463797	216511467	160	16	10164	-0.52677	0.70082
-	SLC9A3	5	460510	605428	629	57	10164	-0.52729	0.701
+	SLC35E4	22	30600806	30657875	227	19	10164	-0.52745	0.70106
-	PDLIM7	5	177473394	177532619	61	10	10164	-0.52765	0.70113
-	SPON2	4	1156932	1243962	207	29	10164	-0.52776	0.70117
-	MCAM	11	119298524	119352130	139	21	10164	-0.5278	0.70118
+	ZNF451	6	57055010	57180305	146	10	10164	-0.52787	0.70121
-	MTX3	5	79966716	80026265	130	14	10164	-0.52796	0.70124
-	TIGD1	2	232537970	232585590	160	17	10164	-0.52803	0.70126
+	AIM1	6	106325808	106580451	1174	85	10164	-0.52806	0.70127
+	HOOK1	1	59779861	59886378	250	25	10164	-0.52812	0.70129
+	KCNC4	1	110175714	110292617	325	30	10164	-0.52826	0.70134
+	CBR3	21	36099900	36156562	209	16	10164	-0.52852	0.70143
+	NPNT	4	105859526	105981671	155	22	10164	-0.52851	0.70143
-	SIAH3	13	45770270	45886711	445	42	10164	-0.52864	0.70147
+	SUPT5H	19	39410546	39486670	198	13	10164	-0.529	0.7016
+	KIAA1024	15	79378494	79482304	363	38	10164	-0.52914	0.70164
-	MTRNR2L8	11	10497887	10544176	27	6	10164	-0.52912	0.70164
+	GLCCI1	7	7933743	8099080	546	41	10164	-0.52922	0.70167
-	LOC1053732	1	246184292	246236730	330	14	10164	-0.52922	0.70167
+	TRAPPC13	5	65589717	65676127	262	14	10164	-0.52927	0.70169
+	METTL25	12	82323406	82489239	45	21	10164	-0.52929	0.7017
+	TCN2	22	30572083	30637060	230	24	10164	-0.52933	0.70171
-	PRAC1	17	48711719	48757520	149	41	10164	-0.52936	0.70172
-	WDR92	2	68120149	68192560	123	9	10164	-0.52964	0.70182
-	CDK6	7	92594921	92871627	376	44	10164	-0.52994	0.70192

STRAND	SYMBOL	CHR	START	STOP	NSNPS	NPARAM	N	ZSTAT	P
+	MVB12A	19	17370686	17435339	189	20	10164	-0.53006	0.70197
-	IQUB	7	123442181	123569664	199	16	10164	-0.53016	0.702
+	C9orf9	9	132843895	132900034	112	17	10164	-0.53038	0.70208
+	METTL7B	12	55646546	55694611	145	33	10164	-0.53048	0.70211
-	ALDH9A1	1	165652212	165733663	336	22	10164	-0.53053	0.70213
-	DEGS2	14	100136416	100201882	190	34	10164	-0.53058	0.70215
+	CRB2	9	123319459	123390335	220	41	10164	-0.5311	0.70232
+	ADD3	10	109961365	110145565	281	23	10164	-0.53116	0.70235
-	HIRA	22	19320701	19466696	369	24	10164	-0.53118	0.70235
-	SUCLG1	2	84413523	84494462	174	12	10164	-0.5312	0.70236
+	SPTBN4	19	40431107	40586459	246	36	10164	-0.53121	0.70236
+	PNMT	17	39632981	39680475	101	9	10164	-0.5314	0.70243
+	TRIM34	11	5584944	5654398	293	37	10164	-0.5314	0.70243
+	ZDHHC4	7	6542412	6598979	133	14	10164	-0.53141	0.70243
-	CLDN22	4	183308067	183355774	137	22	10164	-0.53147	0.70245
-	NACC2	9	135996537	136130285	129	29	10164	-0.5331	0.70302
+	SIVA1	14	104717420	104769659	195	22	10164	-0.53322	0.70306
+	ZNF224	19	44059329	44119831	181	19	10164	-0.53343	0.70313
+	BDKRB1	14	96221210	96274763	167	22	10164	-0.53355	0.70318
-	TCEB3CL2	18	46952768	46999644	32	10	10164	-0.5336	0.70319
+	KIAA0586	14	58392385	58558831	161	29	10164	-0.53384	0.70327
+	SLC25A38	3	39348324	39407328	240	28	10164	-0.53397	0.70332
-	POT1	7	124812386	124964983	487	9	10164	-0.53407	0.70335
+	CDC42EP1	22	37524778	37579405	143	21	10164	-0.53409	0.70336
+	PLA1A	3	119562848	119639811	288	26	10164	-0.53435	0.70345
-	LRFN1	19	39296817	39355854	18	7	10164	-0.53449	0.7035
-	KRT19	17	41513617	41563389	27	5	10164	-0.53482	0.70361
+	FAM76A	1	27690979	27772915	155	4	10164	-0.53491	0.70364
-	FASN	17	82068338	82133289	168	5	10164	-0.53493	0.70365
-	CAMK2D	4	113441032	113797133	760	48	10164	-0.53506	0.70369
+	TSPAN13	7	16718726	16794536	327	39	10164	-0.53505	0.70369
-	PLSCR4	3	146182335	146286179	391	20	10164	-0.53514	0.70372
+	LRRC8E	19	7853505	7912023	157	27	10164	-0.53562	0.70389
-	CRISP1	6	49824257	49912096	172	41	10164	-0.53567	0.70391
-	TXNRD3	3	126597052	126690124	199	10	10164	-0.53589	0.70398
-	HEBP1	12	12964864	13035309	219	23	10164	-0.53598	0.70401
+	ZNF382	19	36570305	36638597	187	21	10164	-0.536	0.70402
-	CARHSP1	16	8842942	8904012	230	36	10164	-0.53627	0.70411
-	TRIM35	8	27274886	27346319	195	24	10164	-0.53653	0.70421
-	RPS10	6	34407454	34461125	265	41	10164	-0.5366	0.70423
-	LBR	1	225391502	225463855	229	32	10164	-0.53703	0.70438
-	KRT6B	12	52436651	52487126	285	13	10164	-0.53715	0.70442
+	CBL	11	119171276	119318149	323	27	10164	-0.5373	0.70447
-	BRD3	9	134020305	134103533	315	13	10164	-0.53737	0.70449
+	ESPL1	12	53233266	53303643	130	15	10164	-0.53756	0.70456
+	SSNA1	9	137153602	137200370	70	10	10164	-0.53771	0.70461
-	CA1	8	85318229	85413154	151	18	10164	-0.53788	0.70467
+	DYNLL1	12	120434857	120508495	228	24	10164	-0.53796	0.7047
+	KRTAP9-6	17	41230378	41275860	95	7	10164	-0.538	0.70471
-	S1PR2	19	10211433	10266807	142	25	10164	-0.53807	0.70474
+	AFM	4	73446745	73514001	158	15	10164	-0.53851	0.70489
+	NKX2-3	10	99497933	99546524	186	19	10164	-0.53859	0.70491
+	ADARB1	21	45039578	45236563	579	37	10164	-0.53863	0.70493
-	LUM	12	91093455	91146765	129	20	10164	-0.53882	0.705
-	PRR36	19	7858719	7909441	135	24	10164	-0.53888	0.70502
-	IRX3	16	54273300	54321856	115	20	10164	-0.53895	0.70504
-	TCF15	20	593993	645266	147	27	10164	-0.53898	0.70505
-	HACE1	6	104672659	104894919	548	25	10164	-0.5391	0.70509
+	UQCR10	22	29732369	29780413	160	17	10164	-0.53917	0.70512
-	ADAMTS7	15	78749203	78846464	502	35	10164	-0.53924	0.70514
-	ECI2	6	4105693	4170597	182	17	10164	-0.53928	0.70515
+	PRDM12	9	130629594	130692997	163	33	10164	-0.53937	0.70519
-	LOC1053759	9	961948	1011558	144	16	10164	-0.53945	0.70521

STRAND	SYMBOL	CHR	START	STOP	NSNPS	NPARAM	N	ZSTAT	P
+	KRTAP5-7	11	71492267	71538164	181	21	10164	-0.53968	0.70529
+	CD274	9	5415381	5480567	213	41	10164	-0.53981	0.70534
+	NUDT1	7	2207222	2261145	153	21	10164	-0.53986	0.70535
-	ARVCF	22	19956603	20051802	405	41	10164	-0.53989	0.70536
+	MEP1B	18	32155024	32230404	144	22	10164	-0.53993	0.70538
+	PROZ	13	113120061	113182386	133	21	10164	-0.53998	0.70539
-	SRSF10	1	23954347	24015463	123	6	10164	-0.54032	0.70551
-	DEAF1	11	634220	730754	353	31	10164	-0.54041	0.70554
-	NARS	18	57590656	57656945	225	31	10164	-0.54047	0.70557
-	AKT3	1	243478233	243886079	526	25	10164	-0.5406	0.70561
+	BIVM-ERCC5	13	102772146	102886001	367	34	10164	-0.54072	0.70565
-	ADRA1A	8	26732847	26906008	772	67	10164	-0.541	0.70575
-	ZNF385B	2	179431982	179897321	1448	97	10164	-0.54101	0.70575
-	TTC22	1	54769712	54836717	311	39	10164	-0.54109	0.70578
+	PLA2G4B	15	41803813	41858148	165	22	10164	-0.54112	0.70579
-	BSPH1	19	47958046	48027170	217	32	10164	-0.54132	0.70586
+	IMP4	2	130307897	130356625	70	7	10164	-0.54141	0.70589
-	TRIM37	17	58972638	59141905	241	25	10164	-0.54145	0.7059
-	C19orf43	19	12720640	12769715	9	5	10164	-0.54144	0.7059
-	CD47	3	108033094	108126088	180	23	10164	-0.54165	0.70597
-	MEF2BNB-M	19	19135567	19227591	330	29	10164	-0.54174	0.706
-	APP	21	25870550	26206128	1030	89	10164	-0.54185	0.70604
-	KRT84	12	52367812	52421906	212	16	10164	-0.54213	0.70613
-	TUBD1	17	59849479	59927945	142	12	10164	-0.54217	0.70615
+	LZTR1	22	20947269	21009037	145	27	10164	-0.54239	0.70623
+	C15orf61	15	67486184	67537303	164	19	10164	-0.54245	0.70624
+	MTMR9	8	11249491	11338146	314	25	10164	-0.54253	0.70627
-	CCDC166	8	143696694	143743109	91	11	10164	-0.54275	0.70635
+	IFI27L1	14	94046293	94112714	255	24	10164	-0.54293	0.70641
+	CAMTA1	1	6750324	7779706	2794	209	10164	-0.54314	0.70648
-	FGFBP2	4	15950240	15998236	185	40	10164	-0.54325	0.70652
+	LOC1019284	7	143585924	143653937	2	1	10164	-0.54337	0.70656
-	VN1R4	19	53256760	53302907	150	22	10164	-0.54351	0.70661
+	TNFAIP8	5	119233692	119404604	612	55	10164	-0.5437	0.70668
-	HPGDS	4	94288556	94377876	384	19	10164	-0.54384	0.70672
-	PLEKHM1	17	45424247	45525780	143	10	10164	-0.5439	0.70674
-	UNC5C	4	95152504	95584210	1377	137	10164	-0.54404	0.70679
+	RABEP1	17	5247263	5396340	537	29	10164	-0.54407	0.7068
-	HSD17B11	4	87326522	87426303	315	34	10164	-0.54413	0.70682
-	SCMH1	1	41017199	41277159	763	30	10164	-0.54459	0.70698
-	MRPL27	17	50357867	50408201	150	14	10164	-0.54482	0.70706
-	HIPK4	19	40369271	40425221	89	19	10164	-0.54482	0.70706
+	ZFHX4	8	76646219	76877285	587	51	10164	-0.54522	0.7072
+	GRN	17	44310086	44363106	88	16	10164	-0.5453	0.70723
+	NDUFB9	8	124504102	124559986	140	21	10164	-0.54532	0.70723
+	NEO1	15	73016715	73315206	675	28	10164	-0.54536	0.70725
+	NTNG2	9	132126652	132254534	261	42	10164	-0.54543	0.70727
-	NRAS	1	114694464	114751894	66	10	10164	-0.5456	0.70733
+	AGPAT6	8	41543188	41635001	271	19	10164	-0.54597	0.70746
-	CWC15	11	94952622	95008612	88	9	10164	-0.54604	0.70748
-	ADH5	4	99060978	99123788	53	8	10164	-0.54617	0.70753
-	STMN1	1	25874186	25941877	109	9	10164	-0.54654	0.70765
+	VAMP8	2	85542491	85592033	153	17	10164	-0.5466	0.70767
-	TIMM13	19	2415624	2462916	166	17	10164	-0.5467	0.70771
-	ATP8A1	4	42398373	42692105	1087	59	10164	-0.54674	0.70772
-	ARMC1	8	65592456	65669217	276	16	10164	-0.54685	0.70776
+	ITGAD	16	31358241	31436513	82	14	10164	-0.54696	0.7078
+	EPS8L2	11	671120	737727	234	35	10164	-0.54707	0.70784
+	OR13C8	9	104534168	104580130	107	16	10164	-0.54727	0.7079
-	DEFB133	6	49936101	49984444	84	7	10164	-0.54735	0.70793
-	RSPO1	1	37601350	37669923	186	39	10164	-0.54737	0.70794
+	IMPA2	18	11946428	12040886	448	34	10164	-0.54743	0.70796
-	C4orf50	4	5947112	6043060	207	20	10164	-0.54775	0.70807

STRAND	SYMBOL	CHR	START	STOP	NSNPS	NPARAM	N	ZSTAT	P
+	ANKRD7	7	118189658	118252731	250	17	10164	-0.54802	0.70816
+	MOV10L1	22	50055006	50171705	606	18	10164	-0.54821	0.70823
+	LOC1053724	19	31314235	31393997	174	19	10164	-0.54838	0.70828
-	TTC28	22	27968014	28714865	1672	69	10164	-0.54846	0.70831
-	LY6H	8	143147914	143195636	110	14	10164	-0.54853	0.70833
-	LOC1053788	1	86693498	86739754	83	12	10164	-0.54872	0.7084
-	TECPR1	7	98205443	98287251	302	30	10164	-0.54886	0.70845
+	DAB2IP	9	121531883	121795530	836	116	10164	-0.54887	0.70845
+	TAF1B	2	9808442	9944416	489	17	10164	-0.54892	0.70847
+	STXBP2	19	7602101	7657874	239	19	10164	-0.54908	0.70852
+	SMIM1	1	3737761	3785982	150	17	10164	-0.54912	0.70854
+	OTX1	2	63015057	63067831	64	29	10164	-0.54918	0.70856
-	AATK	17	81107295	81201072	289	41	10164	-0.54943	0.70864
+	NGFR	17	49460293	49525020	205	49	10164	-0.54963	0.70871
+	RAVER2	1	64709964	64843232	176	13	10164	-0.54998	0.70883
+	PAPD5	16	50117918	50245310	210	19	10164	-0.55006	0.70886
+	ESPNL	2	238065310	238143287	331	32	10164	-0.55022	0.70891
+	SZRD1	1	16332030	16408148	120	7	10164	-0.55038	0.70897
+	PTPN20	10	46876413	47041830	10	2	10164	-0.55043	0.70899
-	LRRC58	3	120314729	120384361	239	29	10164	-0.55045	0.70899
-	CNBP	3	129157815	129218967	103	18	10164	-0.55058	0.70904
-	MARCH1	4	163514298	164418989	3308	158	10164	-0.55067	0.70907
-	TVP23C-CDF	17	15426015	15598631	458	36	10164	-0.55071	0.70908
-	LOC1019285	4	1190379	1236262	130	18	10164	-0.551	0.70918
-	N4BP2L1	13	32390723	32464614	145	28	10164	-0.55117	0.70924
+	ZKSCAN3	6	28314913	28379177	288	41	10164	-0.55137	0.70931
+	POLK	5	75476043	75609823	381	15	10164	-0.55142	0.70933
+	GABRG3	15	26936282	27543895	1600	155	10164	-0.55159	0.70939
-	POM121C	7	75406781	75521288	107	14	10164	-0.55182	0.70947
-	UQCC1	20	35292566	35447142	352	19	10164	-0.55195	0.70951
+	HPCAL1	2	10267889	10437617	541	79	10164	-0.55242	0.70967
+	PHACTR3	20	59542509	59857711	654	76	10164	-0.55245	0.70968
+	NFIC	19	3324563	3479217	196	23	10164	-0.55249	0.70969
-	DGKQ	4	948884	1008560	206	37	10164	-0.55275	0.70978
+	PSTK	10	122919235	123007519	187	21	10164	-0.55283	0.70981
+	RIPPLY2	6	83818266	83867515	100	6	10164	-0.55416	0.71027
+	ME2	18	50843604	50959795	300	20	10164	-0.55421	0.71028
+	ATG13	11	46582276	46686019	111	17	10164	-0.55419	0.71028
+	SWAP70	11	9629077	9762991	482	21	10164	-0.55421	0.71028
-	RASAL3	19	15441624	15499571	80	13	10164	-0.55455	0.7104
-	ARID4B	1	235156895	235363298	345	7	10164	-0.55461	0.71042
+	SAG	2	233269300	233357055	313	25	10164	-0.55465	0.71043
-	NAAA	4	75900655	75976013	195	22	10164	-0.55472	0.71046
-	ABCC12	16	48072973	48181770	36	3	10164	-0.55479	0.71048
+	COX8C	14	93312191	93358354	170	10	10164	-0.55486	0.7105
+	WNT8B	10	100428055	100493642	133	4	10164	-0.55544	0.7107
+	ALPK1	4	112262336	112452618	470	61	10164	-0.55556	0.71075
-	MAFG	17	81908269	81966560	7	1	10164	-0.55563	0.71077
-	PYCR1	17	81922384	81972328	7	1	10164	-0.55563	0.71077
-	SETD4	21	36024541	36095818	228	21	10164	-0.55563	0.71077
+	TMCO3	13	113455993	113560229	559	21	10164	-0.55566	0.71078
+	OSGIN2	8	89866868	89937868	223	36	10164	-0.55573	0.7108
-	GDI2	10	5755223	5848549	377	42	10164	-0.55607	0.71092
+	DNAAF1	16	84110260	84187918	294	35	10164	-0.55626	0.71098
-	PTPN2	18	12775478	12919335	402	36	10164	-0.5563	0.711
+	MCM2	3	127563357	127632436	66	18	10164	-0.55665	0.71112
+	PNO1	2	68122873	68185959	105	8	10164	-0.55693	0.71121
-	IRF2BP2	1	234594269	234644525	93	11	10164	-0.55706	0.71126
+	EDNRA	4	147445917	147554954	260	19	10164	-0.55716	0.71129
-	OR52R1	11	4793433	4839380	158	10	10164	-0.55723	0.71132
+	KRTAP5-6	11	1662195	1707755	177	28	10164	-0.55756	0.71143
+	CDH5	16	66331622	66414786	190	28	10164	-0.55787	0.71153
-	ZNF770	15	34968341	35023296	141	16	10164	-0.55796	0.71156

STRAND	SYMBOL	CHR	START	STOP	NSNPS	NPARAM	N	ZSTAT	P
-	TIFA	4	112265597	112320903	135	19	10164	-0.5581	0.71161
-	SOX15	17	7578180	7625170	108	22	10164	-0.55819	0.71164
+	PTPRF	1	43487238	43633672	355	25	10164	-0.55821	0.71165
-	SNX31	8	100562882	100698410	330	29	10164	-0.55842	0.71172
+	OR2F2	7	143900233	143946186	124	11	10164	-0.55872	0.71182
+	FASLG	1	172624008	172676873	147	17	10164	-0.55891	0.71189
-	LOC1053769	19	41125638	41228052	255	44	10164	-0.55893	0.71189
-	PARD3	10	34099560	34850325	1507	38	10164	-0.55896	0.7119
+	API5	11	43276955	43354530	170	31	10164	-0.559	0.71192
-	C9orf153	9	86210265	86294657	185	25	10164	-0.55911	0.71196
-	ZSCAN31	6	28302837	28389506	376	38	10164	-0.55921	0.71199
+	VPS51	11	65061115	65121860	126	21	10164	-0.55924	0.712
+	SSMEM1	7	130170403	130226844	82	7	10164	-0.5593	0.71202
+	DENND3	8	141093621	141205804	209	35	10164	-0.55942	0.71206
+	ZIK1	19	57548642	57603888	114	15	10164	-0.55963	0.71213
+	AP1AR	4	112196737	112282715	194	21	10164	-0.55965	0.71214
-	ITGB3BP	1	63430770	63564159	295	12	10164	-0.55991	0.71223
+	ZNF267	16	31838758	31927308	28	5	10164	-0.56014	0.71231
-	DEFB110	6	49999138	50056981	99	7	10164	-0.56032	0.71237
-	TMEM101	17	44001188	44059260	102	12	10164	-0.56035	0.71238
+	LRRC43	12	122132719	122213471	247	27	10164	-0.56049	0.71243
-	TRDN	6	123206339	123672283	1211	38	10164	-0.56059	0.71246
+	GOLGA5	14	92759231	92849963	311	16	10164	-0.56062	0.71247
+	IFITM2	11	273107	319410	94	18	10164	-0.56095	0.71258
-	ALOX12B	17	8062636	8122703	195	56	10164	-0.56099	0.7126
+	NPIPA2	16	14710595	14775448	1	1	10164	-0.56099	0.7126
-	LOC1053767	16	14724887	14777760	1	1	10164	-0.56099	0.7126
+	COX6A1	12	120403090	120450742	161	28	10164	-0.56106	0.71262
+	ATG4C	1	62749106	62875270	276	12	10164	-0.56132	0.71271
-	M1AP	2	74547883	74683852	51	5	10164	-0.56133	0.71271
-	SERINC1	6	122433348	122506807	193	21	10164	-0.56182	0.71288
-	ESRRG	1	216493246	217172755	2128	177	10164	-0.56218	0.713
+	SLC9A8	20	49777713	49902242	404	34	10164	-0.56264	0.71316
+	DNAJB13	11	73915319	73980287	158	21	10164	-0.56265	0.71316
+	HIC1	17	2020099	2069687	96	19	10164	-0.56306	0.7133
-	STRN3	14	30883779	31061528	522	33	10164	-0.56354	0.71347
+	RDH12	14	67666886	67744451	256	28	10164	-0.56356	0.71347
-	ETS1	11	128448761	128622584	360	58	10164	-0.56363	0.7135
-	DNMT3A	2	25222961	25377590	306	46	10164	-0.56376	0.71354
+	CNTD1	17	42763667	42821587	96	9	10164	-0.5638	0.71355
-	RAB43	3	129077569	129157150	105	14	10164	-0.56382	0.71356
-	ZNF705G	8	7347976	7397968	6	2	10164	-0.56396	0.71361
-	PEX6	6	42953873	43014243	180	18	10164	-0.56398	0.71362
-	DNASE1L3	3	58182626	58246003	115	9	10164	-0.56444	0.71377
-	GDE1	16	19491693	19557128	238	19	10164	-0.56461	0.71383
+	NDE1	16	15608267	15736353	277	26	10164	-0.56471	0.71386
+	AREG	4	74410098	74465009	172	14	10164	-0.56542	0.7141
-	EXD3	9	137296894	137458268	220	37	10164	-0.56555	0.71415
+	SMOC2	6	168406151	168677994	1275	99	10164	-0.56559	0.71416
+	CYP21A2	6	32003316	32051670	38	17	10164	-0.5656	0.71417
+	MCMDC2	8	66835749	66936386	134	10	10164	-0.56571	0.7142
-	RBMXL1	1	88969456	89027960	166	19	10164	-0.56583	0.71425
-	SLC25A28	10	99600518	99694339	236	14	10164	-0.56596	0.71429
+	PDLIM2	8	22543741	22608025	67	9	10164	-0.5661	0.71434
-	PMCH	12	102186459	102232836	103	14	10164	-0.56618	0.71436
+	CBR1	21	36034924	36083177	160	17	10164	-0.5664	0.71444
-	LOC1019293	22	17570157	17624192	233	14	10164	-0.56639	0.71444
-	MZT2A	2	131459724	131527719	161	1	10164	-0.56645	0.71445
+	SLC14A2	18	45177957	45693107	1732	130	10164	-0.56652	0.71448
+	KLHL24	3	183600011	183694519	302	9	10164	-0.56669	0.71454
+	PSME3	17	42798142	42853760	70	13	10164	-0.56695	0.71463
-	ATF2	2	175062250	175203206	295	13	10164	-0.56718	0.7147
+	ABCC10	6	43392546	43461980	167	27	10164	-0.56724	0.71472

STRAND	SYMBOL	CHR	START	STOP	NSNPS	NPARAM	N	ZSTAT	P
+	SELO	22	50165979	50227616	219	32	10164	-0.56736	0.71476
+	CCR5	3	46335142	46386206	169	20	10164	-0.56748	0.71481
+	DMXL1	5	119036014	119259129	482	35	10164	-0.56758	0.71484
+	CLUL1	18	561998	660298	219	49	10164	-0.56807	0.71501
+	LURAP1	1	46168334	46231256	167	19	10164	-0.56811	0.71502
-	COMMD6	13	75515214	75584439	140	12	10164	-0.56817	0.71504
-	LOC1053789	1	44127796	44176631	119	21	10164	-0.5682	0.71505
+	CEBPG	19	33338669	33392686	214	19	10164	-0.56826	0.71507
-	FBXO27	19	39014021	39067596	229	11	10164	-0.56827	0.71507
+	ZNF135	19	58024239	58079755	107	11	10164	-0.5684	0.71512
-	MINA	3	97931817	98007451	140	27	10164	-0.5685	0.71515
+	ATP5L	11	118366389	118419847	88	12	10164	-0.56858	0.71518
-	STK24	13	98440199	98612151	638	36	10164	-0.56884	0.71527
+	DOK6	18	69366048	69859087	1621	133	10164	-0.56921	0.71539
+	SH3GLB1	1	86669570	86758184	158	17	10164	-0.56932	0.71543
+	ZNF197	3	44590019	44658471	73	10	10164	-0.56938	0.71545
-	UFD1L	22	19439941	19514296	206	25	10164	-0.56963	0.71554
-	FNDC4	2	27481883	27530296	107	17	10164	-0.56963	0.71554
-	C5orf34	5	43476701	43550171	147	11	10164	-0.56967	0.71555
+	OR1Q1	9	122533380	122625830	460	37	10164	-0.5697	0.71556
+	CFAP52	17	9541627	9653459	78	14	10164	-0.56978	0.71558
+	LYPLAL1	1	219138831	219222865	182	19	10164	-0.56986	0.71562
-	CYTIP	2	157404619	157479092	72	13	10164	-0.56992	0.71563
-	ANGPT4	20	861243	951375	209	42	10164	-0.57003	0.71567
+	RAD23B	9	107248236	107342194	255	19	10164	-0.57006	0.71568
+	VASH1	14	76726892	76793022	191	21	10164	-0.57008	0.71569
-	PIGF	2	46571274	46652112	236	23	10164	-0.57014	0.71571
-	PRH1	12	10870961	11206623	1069	9	10164	-0.57016	0.71571
-	SLFN14	17	35535734	35595836	207	24	10164	-0.57018	0.71572
+	PPRC1	10	102097994	102160333	77	31	10164	-0.57026	0.71575
-	TRAM1L1	4	117073554	117120580	134	25	10164	-0.57033	0.71577
-	ZNF550	19	57531836	57596647	113	16	10164	-0.57074	0.71591
+	AQP9	15	58103209	58195911	303	32	10164	-0.57079	0.71593
-	FTSJ2	7	2224291	2277198	146	19	10164	-0.57081	0.71594
-	RASSF6	4	73561550	73655631	247	24	10164	-0.57091	0.71597
+	DACT1	14	58599068	58658321	121	21	10164	-0.57115	0.71605
+	MON1B	16	77155919	77209646	236	28	10164	-0.57174	0.71625
-	PIP5K1C	19	3620181	3735492	176	24	10164	-0.57173	0.71625
+	SMIM19	8	42506155	42562997	77	9	10164	-0.57219	0.7164
-	GGT6	17	4546927	4595596	89	14	10164	-0.57224	0.71642
+	LOC1053720	18	4419758	4495704	72	10	10164	-0.57229	0.71644
+	HORMAD2	22	30045069	30220882	415	34	10164	-0.5726	0.71654
-	DPYD	1	97067743	97956059	2275	148	10164	-0.57262	0.71655
-	LRRC32	11	76647524	76705946	190	31	10164	-0.57273	0.71659
-	GUSB	7	65950684	66017314	209	22	10164	-0.57286	0.71663
+	FHOD3	18	36262696	36790059	1172	88	10164	-0.57297	0.71667
-	CCBL2	1	88925773	89027960	295	22	10164	-0.57302	0.71669
-	TRIM23	5	65579680	65659360	247	14	10164	-0.57324	0.71676
+	SSTR4	20	23000420	23046677	134	15	10164	-0.57349	0.71684
+	ZSCAN9	6	28177665	28243487	254	41	10164	-0.57361	0.71688
-	C17orf70	17	81529885	81587403	189	19	10164	-0.57396	0.717
+	CCDC134	22	41765622	41842164	199	20	10164	-0.57429	0.71711
+	MTX1	1	155173699	155223839	47	9	10164	-0.57441	0.71715
+	FCGR2A	1	161470415	161534048	117	25	10164	-0.57457	0.71721
+	TM7SF2	11	65076854	65126235	98	21	10164	-0.57459	0.71721
-	OR52L1	11	5975892	6021985	187	20	10164	-0.57485	0.7173
+	NRP2	2	205647449	205808133	260	61	10164	-0.57486	0.71731
+	CAPN9	1	230712334	230812003	507	56	10164	-0.57489	0.71732
-	SNIP1	1	37524449	37589344	159	21	10164	-0.57503	0.71736
+	ERAP2	5	96840940	96929703	381	24	10164	-0.57542	0.7175
-	LOC1053752	7	57199802	57257887	269	25	10164	-0.57555	0.71754
-	ACTN1	14	68864123	69014366	510	45	10164	-0.57566	0.71758
+	HMBOX1	8	28855394	29074939	237	23	10164	-0.57572	0.7176

STRAND	SYMBOL	CHR	START	STOP	NSNPS	NPARAM	N	ZSTAT	P
+	ZNF333	19	14654787	14743746	288	27	10164	-0.57587	0.71765
-	NALCN	13	101043776	101452205	1540	97	10164	-0.57593	0.71767
+	MRPL44	2	223915890	223977714	155	15	10164	-0.57597	0.71768
+	CCP110	16	19488857	19563408	271	21	10164	-0.57603	0.7177
+	CACHD1	1	64435793	64703058	400	54	10164	-0.5766	0.71789
-	ZPR1	11	116768560	116823023	161	32	10164	-0.57663	0.71791
+	FRAT1	10	97284265	97331915	170	15	10164	-0.57664	0.71791
-	OR10T2	1	158388522	158434466	122	9	10164	-0.57686	0.71798
-	RBFOX3	17	79079345	79646101	1649	195	10164	-0.57685	0.71798
-	ZNF514	2	95120110	95200187	90	20	10164	-0.57702	0.71804
-	SLC25A45	11	65363053	65418701	122	47	10164	-0.57727	0.71812
-	ZNF665	19	53153299	53228396	144	20	10164	-0.57736	0.71815
-	WDR74	11	62822908	62875572	107	8	10164	-0.57744	0.71818
-	COA4	11	73862667	73911845	132	13	10164	-0.57749	0.7182
+	HMG20B	19	3537945	3589083	31	4	10164	-0.57768	0.71826
-	UBTF	17	44195033	44256626	81	9	10164	-0.57773	0.71828
+	MIEF2	17	18225534	18275781	130	19	10164	-0.57792	0.71834
+	IVL	1	152873563	152921886	169	13	10164	-0.57801	0.71837
-	PATL1	11	59626716	59704040	149	12	10164	-0.57812	0.71841
+	INO80E	16	29960690	30015790	51	2	10164	-0.57813	0.71841
+	TNFSF12-TN	17	7514058	7571608	232	30	10164	-0.57851	0.71854
-	ANKRD9	14	102493649	102544791	187	28	10164	-0.57864	0.71858
-	DDX5	17	64488256	64541924	68	3	10164	-0.57887	0.71866
+	RING1	6	33173509	33222722	240	43	10164	-0.579	0.7187
-	KRTAP17-1	17	41304917	41350695	119	11	10164	-0.57899	0.7187
+	HMOX2	16	4439703	4520347	5	3	10164	-0.57919	0.71877
+	ZNF30	19	34891903	34955172	190	12	10164	-0.57944	0.71885
-	C1orf43	1	154196701	154255797	87	14	10164	-0.5797	0.71894
-	TMBIM1	2	218264194	218327557	148	8	10164	-0.57996	0.71903
+	CCNT2	2	134883822	134969345	205	20	10164	-0.57999	0.71904
-	DIAPH1	5	141505016	141654055	296	27	10164	-0.58041	0.71918
-	NDUFA4L2	12	57224903	57275762	84	16	10164	-0.58058	0.71924
-	ZNF841	19	52054363	52130773	295	16	10164	-0.58063	0.71926
+	UBAP2L	1	154184951	154281510	146	19	10164	-0.58068	0.71927
+	SYCE1L	16	77164452	77223079	298	25	10164	-0.58072	0.71929
+	TMEM178B	7	141038311	141507881	971	92	10164	-0.58088	0.71934
-	C10orf120	10	122687709	122734822	136	18	10164	-0.58093	0.71935
-	PGLYRP2	19	15458645	15514504	108	14	10164	-0.58104	0.71939
-	NPTXR	22	38808451	38879012	164	21	10164	-0.58109	0.71941
-	DENND1A	9	123369654	123965138	1517	59	10164	-0.5813	0.71948
-	DBN1	5	177446613	177515336	71	10	10164	-0.58133	0.71949
+	C6orf141	6	49515400	49570572	144	26	10164	-0.58174	0.71963
-	NPRL3	16	74271	173698	236	34	10164	-0.58186	0.71967
+	WDR66	12	121883557	122013927	428	25	10164	-0.58239	0.71985
-	SERINC4	15	43784174	43835097	91	10	10164	-0.58244	0.71987
-	FEM1C	5	115510908	115579894	162	21	10164	-0.5828	0.71999
+	CNKSR1	1	26142490	26199884	124	18	10164	-0.58321	0.72013
-	LOC1053747	5	34646306	34693489	174	25	10164	-0.58327	0.72014
+	LOC1053771	22	20325709	20372095	11	6	10164	-0.58348	0.72021
+	FOXA3	19	45829260	45883797	180	18	10164	-0.5835	0.72022
+	SLC34A2	4	25620813	25688748	87	12	10164	-0.58418	0.72045
-	TRIM26	6	30174453	30248494	483	38	10164	-0.58423	0.72047
+	KCNT1	9	135667185	135803147	235	32	10164	-0.58462	0.7206
+	DNAJC21	5	34894593	34968964	154	25	10164	-0.585	0.72073
+	TIMM23B	10	49907033	49998839	121	14	10164	-0.5853	0.72083
+	CCM2	7	44964746	45086470	299	21	10164	-0.58539	0.72086
-	OR5B21	11	58497176	58543105	80	15	10164	-0.58547	0.72088
+	C12orf57	12	6908438	6956003	22	3	10164	-0.58564	0.72094
+	DYNC1I2	2	171652409	171760158	274	37	10164	-0.58583	0.72101
+	BPTF	17	67790528	67994378	387	14	10164	-0.58643	0.72121
-	FAM222B	17	28745978	28889985	242	16	10164	-0.58643	0.72121
+	TCP11L2	12	106267784	106357587	233	19	10164	-0.58646	0.72122
+	MESDC1	15	80965954	81014004	131	8	10164	-0.58653	0.72124

STRAND	SYMBOL	CHR	START	STOP	NSNPS	NPARAM	N	ZSTAT	P
-	CALM2	2	47150082	47211601	161	22	10164	-0.58656	0.72125
+	CENPH	5	69154548	69220357	173	11	10164	-0.58663	0.72127
-	EIF4E3	3	71669289	71789773	277	30	10164	-0.5867	0.7213
+	LOC1053739	2	231366613	231418342	82	11	10164	-0.58697	0.72139
-	POLD2	7	44104680	44158668	141	19	10164	-0.5871	0.72143
-	APOA1	11	116825751	116872647	159	23	10164	-0.58715	0.72145
+	IFNA8	9	21374147	21420185	139	29	10164	-0.58786	0.72169
-	WSB2	12	118022687	118097174	174	26	10164	-0.58789	0.7217
-	TTC30A	2	177604298	177653966	265	22	10164	-0.588	0.72173
-	CENPE	4	103095806	103233409	358	20	10164	-0.58813	0.72178
+	STMN2	8	79575814	79676175	289	45	10164	-0.58838	0.72186
+	ALG11	13	51977387	52039648	135	12	10164	-0.58891	0.72204
+	CD37	19	49300365	49350606	66	7	10164	-0.58901	0.72207
-	PLEKHG5	1	6456092	6555061	273	21	10164	-0.58912	0.72211
+	RSRC1	3	158075052	158554835	1662	31	10164	-0.58945	0.72222
-	TMEM210	9	137160928	137207046	77	10	10164	-0.58955	0.72226
-	PMVK	1	154914732	154972310	77	14	10164	-0.5897	0.7223
+	FAM83D	20	38890078	38963060	138	11	10164	-0.58969	0.7223
-	LRFN2	6	40381099	40622464	893	65	10164	-0.58987	0.72236
-	TM9SF3	10	96508110	96622052	223	11	10164	-0.58998	0.7224
-	LZIC	1	9912110	9978402	128	17	10164	-0.59007	0.72243
+	PFKFB3	10	6109880	6245545	956	98	10164	-0.59019	0.72247
-	ZBP1	20	57593846	57655576	82	14	10164	-0.59057	0.7226
+	CCDC63	12	110809486	110917535	234	25	10164	-0.59081	0.72268
-	SIRPB1	20	1554383	1661497	157	19	10164	-0.59097	0.72273
-	C21orf59	21	32591674	32647608	158	26	10164	-0.59101	0.72274
-	LRRN4	20	6029745	6089080	227	28	10164	-0.59104	0.72275
+	FBXO18	10	5854572	5947595	456	35	10164	-0.59118	0.7228
+	CFTR	7	117443340	117678665	370	22	10164	-0.59135	0.72286
-	HLA-DPA1	6	33054569	33115778	1454	34	10164	-0.59143	0.72288
-	TGM5	15	43222595	43302023	163	18	10164	-0.59152	0.72291
+	FAM213A	10	80372829	80442997	279	22	10164	-0.59171	0.72298
+	GJA10	6	89859469	89906100	155	21	10164	-0.59213	0.72312
-	RDH11	14	67666800	67730793	219	25	10164	-0.59245	0.72323
+	ZNF483	9	111489299	111587844	278	39	10164	-0.59272	0.72332
-	TMEM262	11	65078212	65124990	94	21	10164	-0.59278	0.72333
+	SLC5A7	2	107951487	108024207	141	19	10164	-0.593	0.72341
-	RASSF1	3	50319786	50375936	81	13	10164	-0.59308	0.72344
+	MYO1B	2	191210185	191435389	400	12	10164	-0.59329	0.72351
+	MARK4	19	45216258	45315283	277	30	10164	-0.59353	0.72359
-	MRPL21	11	68881272	68938835	252	34	10164	-0.59356	0.7236
-	LOC1053763	9	107257050	107318631	167	15	10164	-0.5936	0.72361
+	TSEN2	3	12449432	12549622	389	22	10164	-0.5937	0.72364
+	RBM46	4	154746252	154838813	180	15	10164	-0.5939	0.72371
-	GAL3ST1	22	30544635	30609588	147	32	10164	-0.59398	0.72374
+	TTYH2	17	74178557	74272020	185	19	10164	-0.59435	0.72386
+	GDPGP1	15	90199255	90252080	215	26	10164	-0.59442	0.72389
+	NME2	17	51130435	51181744	170	19	10164	-0.59449	0.72391
+	SLC9A2	2	102584689	102721350	318	27	10164	-0.59449	0.72391
-	DTWD2	5	118826074	119023545	439	25	10164	-0.59451	0.72391
+	SERTAD4	1	210197850	210256858	155	20	10164	-0.59454	0.72392
-	HIST3H3	1	228414845	228460325	160	26	10164	-0.59456	0.72393
+	LOC1053714	1	149580928	149635411	16	1	10164	-0.5948	0.72401
+	ODF4	17	8304837	8356048	195	20	10164	-0.59524	0.72416
-	RAD52	12	901259	1025608	318	37	10164	-0.59531	0.72418
+	ORC3	6	87555067	87677454	326	19	10164	-0.59554	0.72426
+	BPIFB6	20	32995977	33054047	187	29	10164	-0.59577	0.72434
-	TCEB3CL	18	46958695	47004912	15	6	10164	-0.59619	0.72448
+	ANKZF1	2	219194757	219246669	91	12	10164	-0.59629	0.72451
-	CDRT4	17	15426015	15502611	250	27	10164	-0.59658	0.72461
+	LTV1	6	143808371	143873806	208	27	10164	-0.59662	0.72462
-	OR10X1	1	158568919	158614899	96	8	10164	-0.59675	0.72466
-	ZMAT4	8	40520590	40932824	1229	89	10164	-0.59697	0.72474

STRAND	SYMBOL	CHR	START	STOP	NSNPS	NPARAM	N	ZSTAT	P
+	DNM1	9	128168355	128265249	198	23	10164	-0.59712	0.72479
+	NFE4	7	102938437	102988667	75	11	10164	-0.59713	0.72479
+	CRY2	11	45812118	45893248	188	23	10164	-0.5973	0.72485
+	FLJ45513	17	49809715	49858837	56	15	10164	-0.59731	0.72485
-	IDH3B	20	2648394	2699219	188	26	10164	-0.59762	0.72495
-	TSPYL6	2	54243178	54291272	177	23	10164	-0.59797	0.72507
+	ACSBG2	19	6100633	6203103	96	8	10164	-0.59813	0.72512
-	MUC1	1	155175824	155227915	48	9	10164	-0.59817	0.72514
-	SLC20A2	8	42406462	42577213	233	32	10164	-0.59818	0.72514
-	GPN3	12	110442486	110503721	82	9	10164	-0.59833	0.72519
-	MYH2	17	10511148	10584700	153	12	10164	-0.59846	0.72523
-	GTF2H2	5	71025124	71102670	1	1	10164	-0.59849	0.72524
+	NAV2	11	19310247	20131601	2060	181	10164	-0.59849	0.72524
+	SLC50A1	1	155100344	155148858	89	11	10164	-0.59867	0.7253
+	SLC30A6	2	32130841	32234112	264	25	10164	-0.59871	0.72532
-	LOC1053738	2	190615330	190684638	182	29	10164	-0.59881	0.72535
-	ADRB3	8	37952995	38001666	55	10	10164	-0.59887	0.72537
-	NPHS2	1	179540539	179610976	273	31	10164	-0.59887	0.72537
-	ASB14	3	57258351	57327720	177	24	10164	-0.59923	0.72549
+	PDE8B	5	77145262	77438256	792	74	10164	-0.59937	0.72554
-	IFNA2	9	21374255	21420397	139	29	10164	-0.5994	0.72555
+	OR10Z1	1	158571439	158617380	101	8	10164	-0.59941	0.72555
-	GAMT	19	1387026	1436570	26	3	10164	-0.59945	0.72556
-	ADAMTS3	4	72270969	72603799	504	28	10164	-0.59993	0.72572
+	C19orf81	19	50597767	50669310	205	37	10164	-0.60015	0.7258
-	MPZL2	11	118243416	118299536	237	17	10164	-0.60035	0.72586
-	EYA1	8	71187433	71583109	853	87	10164	-0.60043	0.72589
+	HSP90B1	12	103895334	103957930	283	28	10164	-0.60049	0.72591
+	SLC38A6	14	60946114	61093733	269	21	10164	-0.60052	0.72592
+	MAPRE1	20	32784780	32860405	198	23	10164	-0.60055	0.72593
+	KCNQ1	11	2409991	2859110	1085	126	10164	-0.60087	0.72604
-	CST5	20	23865935	23914743	119	15	10164	-0.60093	0.72606
-	STARD7	2	96174859	96243846	153	31	10164	-0.60137	0.7262
-	NEK5	13	52026411	52165366	266	15	10164	-0.60142	0.72622
+	IDNK	9	83588049	83654130	173	24	10164	-0.60144	0.72623
+	PRSS56	2	232485463	232535716	142	28	10164	-0.60164	0.72629
-	CYP24A1	20	54143449	54208985	312	33	10164	-0.60168	0.72631
+	KAT7	17	49753619	49839096	92	24	10164	-0.60181	0.72635
-	C6orf52	6	10661418	10729797	154	7	10164	-0.60199	0.72641
-	NOS2	17	27746766	27839341	305	39	10164	-0.60202	0.72642
+	NOX5	15	68895500	69067161	284	23	10164	-0.60205	0.72643
+	FOXP2	7	114051310	114703772	749	57	10164	-0.60222	0.72649
-	SLC16A1	1	112901847	112991353	85	15	10164	-0.6024	0.72655
+	NDEL1	17	8400852	8481708	146	20	10164	-0.60248	0.72657
-	PPM1J	1	112699994	112750328	90	15	10164	-0.60248	0.72657
-	APOA5	11	116779370	116827420	149	26	10164	-0.60259	0.72661
-	FBXO42	1	16236840	16387491	252	9	10164	-0.60278	0.72667
-	AASS	7	122063544	122179290	224	25	10164	-0.60287	0.7267
-	HR	8	22104419	22166053	79	9	10164	-0.60286	0.7267
+	TXNDC2	18	9850726	9898159	69	9	10164	-0.60289	0.72671
+	IFI16	1	158964973	159065155	328	12	10164	-0.60303	0.72676
-	IL17B	5	149364267	149439244	221	36	10164	-0.60326	0.72683
+	APBB1IP	10	26403203	26577879	613	42	10164	-0.60325	0.72683
-	TOMM20	1	235099341	235163941	43	4	10164	-0.60337	0.72687
-	WDR78	1	66802885	66960095	254	33	10164	-0.60351	0.72691
+	CFAP36	2	55484595	55555080	197	28	10164	-0.60348	0.72691
+	ADGRL3	4	61348124	62088335	1822	98	10164	-0.60372	0.72699
-	ARFRP1	20	63688642	63743013	174	13	10164	-0.60378	0.72701
-	NR2C2AP	19	19191409	19238435	116	21	10164	-0.6043	0.72718
-	CD84	1	160531094	160614516	278	19	10164	-0.60465	0.7273
+	CASP8AP2	6	89794900	89884436	254	26	10164	-0.60481	0.72735
+	CARKD	13	110580460	110649996	320	27	10164	-0.605	0.72741
+	CKMT2	5	81198320	81276398	199	28	10164	-0.60508	0.72744

STRAND	SYMBOL	CHR	START	STOP	NSNPS	NPARAM	N	ZSTAT	P
-	C12orf4	12	4477730	4573508	149	16	10164	-0.60539	0.72754
+	GLRB	4	157041125	157182099	446	22	10164	-0.60568	0.72764
+	LOC1053772	4	69159353	69210539	348	13	10164	-0.60586	0.72769
-	LOC1053695	11	119307224	119356858	128	20	10164	-0.60588	0.7277
-	ATP13A4	3	193388967	193590122	544	43	10164	-0.60642	0.72788
-	AP3B1	5	77992326	78329755	713	22	10164	-0.60648	0.7279
+	SRP19	5	112826188	112903079	244	16	10164	-0.6067	0.72797
+	TMIE	3	46658842	46720923	208	32	10164	-0.60711	0.72811
+	C4B	6	31979795	32045418	14	7	10164	-0.6076	0.72827
+	POM121	7	72844335	72961440	62	14	10164	-0.60774	0.72832
-	EPOR	19	11367205	11419342	61	3	10164	-0.60798	0.7284
+	COL26A1	7	101327820	101569024	1144	59	10164	-0.6083	0.72851
-	SMIM24	19	3464407	3515542	2	1	10164	-0.60839	0.72854
+	TRPM2	21	44315126	44453081	206	14	10164	-0.60876	0.72866
-	KRT37	17	41410557	41459570	231	18	10164	-0.60879	0.72867
-	PPP1R9B	17	50123735	50186022	141	24	10164	-0.60885	0.72869
-	COA3	17	42787618	42833725	65	10	10164	-0.60902	0.72874
-	LAIR1	19	54343624	54405556	51	6	10164	-0.60925	0.72882
-	DSC2	18	31055974	31137432	94	12	10164	-0.60956	0.72892
+	CPLX3	15	74791610	74841795	168	27	10164	-0.60967	0.72896
+	MACROD2	20	13960500	16063197	5835	327	10164	-0.60984	0.72902
-	PRSS36	16	31128923	31185094	38	9	10164	-0.60992	0.72904
+	RAP1GDS1	4	98226376	98453861	429	17	10164	-0.61011	0.72911
-	NSL1	1	212716153	212826797	468	16	10164	-0.61024	0.72915
+	MT1M	16	56597622	56643986	214	39	10164	-0.61034	0.72918
+	FDCSP	4	70191071	70245252	213	9	10164	-0.61045	0.72922
+	SHISA9	16	12866620	13572919	2119	137	10164	-0.61045	0.72922
+	SMIM11	21	34340450	34399154	93	15	10164	-0.61068	0.72929
-	MMP13	11	102932992	102990734	187	38	10164	-0.61096	0.72939
+	ADAM28	8	24259040	24369018	352	15	10164	-0.61115	0.72945
+	BMPR2	2	202341310	202577751	310	22	10164	-0.61133	0.72951
-	DSCR4	21	37996553	38156360	350	35	10164	-0.61136	0.72952
-	YDJC	22	21618089	21665325	99	15	10164	-0.61177	0.72966
-	PXT1	6	36380551	36477889	274	16	10164	-0.61193	0.72971
+	C1orf21	1	184352016	184639021	633	35	10164	-0.61197	0.72972
-	MFRP	11	119328934	119381673	72	14	10164	-0.61197	0.72972
-	C1QTNF5	11	119328934	119381673	72	14	10164	-0.61197	0.72972
+	GSTP1	11	67548595	67596653	174	16	10164	-0.612	0.72973
+	ATG7	3	11237324	11567665	731	38	10164	-0.61238	0.72986
+	HMHA1	19	1030923	1097831	300	28	10164	-0.61246	0.72988
+	RPS6KB1	17	59858046	59960426	169	14	10164	-0.61261	0.72993
+	SASH1	6	148158440	148562049	1450	143	10164	-0.61263	0.72994
+	SPAST	2	32028552	32167637	363	20	10164	-0.61294	0.73004
+	ADM2	22	50446556	50496437	148	25	10164	-0.61295	0.73004
+	USP15	12	62225340	62419721	514	33	10164	-0.61296	0.73005
-	VAC14	16	70677439	70836166	221	19	10164	-0.61328	0.73015
+	CDKN2C	1	50933695	50984637	51	8	10164	-0.61341	0.7302
-	CYB561	17	63422304	63481361	101	22	10164	-0.6135	0.73023
-	USP32	17	60167231	60457470	190	7	10164	-0.61351	0.73023
-	CKM	19	45296413	45357977	163	28	10164	-0.61389	0.73035
-	IDH2	15	90073978	90137554	239	25	10164	-0.61395	0.73038
-	SUPT3H	6	44797499	45413051	1838	32	10164	-0.61402	0.7304
+	CFHR5	1	196940037	197019678	162	12	10164	-0.6143	0.73049
+	LOC1053790	5	88968917	89478238	611	48	10164	-0.61444	0.73054
-	TBC1D2	9	98188998	98290721	439	28	10164	-0.6145	0.73056
+	TMEM67	8	93719844	93842649	150	18	10164	-0.61461	0.73059
-	HACD4	9	20993621	21066670	283	32	10164	-0.6147	0.73062
-	CARD8	19	48180371	48291277	435	30	10164	-0.61493	0.7307
-	TMEM55A	8	90984271	91075975	179	24	10164	-0.61501	0.73073
+	CPTP	1	1289756	1338896	93	9	10164	-0.61512	0.73076
-	EXTL2	1	100862372	100930323	50	13	10164	-0.61558	0.73091
+	FJX1	11	35583187	35630873	133	20	10164	-0.6159	0.73102
+	KIAA1468	18	62152244	62317122	746	38	10164	-0.6161	0.73108

STRAND	SYMBOL	CHR	START	STOP	NSNPS	NPARAM	N	ZSTAT	P
+	RFESD	5	95611741	95668082	67	10	10164	-0.61614	0.7311
-	CD80	3	119513948	119594634	337	38	10164	-0.61624	0.73113
+	FAM81B	5	95349802	95460441	284	34	10164	-0.61632	0.73116
+	TSSK4	14	24170530	24218248	102	16	10164	-0.61631	0.73116
-	ATOX1	5	151732822	151793649	157	14	10164	-0.61638	0.73118
+	MRPS36	5	69182746	69240158	143	10	10164	-0.61657	0.73124
+	HMGA1	6	34201800	34256231	229	31	10164	-0.61701	0.73139
+	PROK1	1	110416166	110467354	153	26	10164	-0.61723	0.73146
-	LITAF	16	11537722	11671377	411	39	10164	-0.61749	0.73154
-	PES1	22	30566625	30642013	260	26	10164	-0.61757	0.73157
-	LOC1053781	6	168909702	168999340	526	37	10164	-0.61769	0.73161
+	ZNF655	7	99523422	99586453	69	12	10164	-0.61777	0.73164
+	VPS11	11	119032752	119091978	189	17	10164	-0.61783	0.73166
-	C4orf36	4	86866205	86932123	179	23	10164	-0.61801	0.73171
-	LRTM1	3	54908237	55002088	279	31	10164	-0.61808	0.73174
+	RMND5A	2	86685291	86788041	52	6	10164	-0.6181	0.73175
+	BAG2	6	57137306	57195215	53	5	10164	-0.61824	0.73179
-	PSD	10	102392617	102455161	110	26	10164	-0.61828	0.73181
-	GON4L	1	155739658	155894400	237	35	10164	-0.61835	0.73183
+	KCNIP3	2	95262324	95396077	216	33	10164	-0.61853	0.73189
+	ZNF222	19	43990332	44043110	164	19	10164	-0.61882	0.73198
-	C7orf77	7	124767292	124825810	127	8	10164	-0.61891	0.73201
-	DEDD	1	161110977	161167783	63	7	10164	-0.61894	0.73202
+	OR5AN1	11	59329459	59375394	65	7	10164	-0.61908	0.73207
-	PROM1	4	15958226	16154884	677	80	10164	-0.61922	0.73212
-	DNAH7	2	195727703	196110186	809	30	10164	-0.61923	0.73212
+	NEU2	2	232988056	233045532	203	34	10164	-0.61955	0.73222
+	NPTX2	7	98582285	98639869	66	13	10164	-0.61958	0.73223
-	MORN1	1	2311253	2426751	446	57	10164	-0.61965	0.73226
-	ITSN2	2	24192866	24395528	264	25	10164	-0.6197	0.73227
-	HHATL	3	42682663	42737848	153	21	10164	-0.62004	0.73239
-	LOC1053701	13	40071592	40515889	1193	144	10164	-0.6203	0.73247
+	PALM2	9	109605788	109961476	978	65	10164	-0.62052	0.73254
+	H6PD	1	9199776	9281337	294	28	10164	-0.62059	0.73257
-	DHPS	19	12662858	12716887	18	5	10164	-0.62073	0.73261
+	HSPA4	5	133016970	133115017	227	15	10164	-0.62073	0.73261
+	OR4E2	14	21630083	21676024	174	19	10164	-0.62071	0.73261
+	SPATA31D3	9	81908500	81960094	17	3	10164	-0.62127	0.73279
-	TCTE1	6	44270160	44332721	135	14	10164	-0.62136	0.73282
-	RFNG	17	82037902	82086774	64	9	10164	-0.62174	0.73294
-	TOB1	17	50852223	50902978	93	10	10164	-0.62176	0.73295
+	HUNK	21	31838315	32014064	433	63	10164	-0.62176	0.73295
+	JMJD7-PLA2	15	41793085	41858148	181	23	10164	-0.62179	0.73296
-	LDB1	10	102096558	102155453	78	31	10164	-0.62203	0.73304
+	PKDCC	2	42013021	42068528	208	14	10164	-0.62222	0.7331
+	RNF212B	14	23150334	23284285	413	39	10164	-0.62229	0.73313
+	PUS1	12	131894200	131953861	73	7	10164	-0.6226	0.73323
-	ALKBH2	12	109078188	109128631	132	24	10164	-0.62276	0.73328
+	LOC1005055	11	1652506	1699058	177	31	10164	-0.62277	0.73328
-	LGALS4	19	38791671	38848544	193	25	10164	-0.62302	0.73336
+	UMPS	3	124695366	124759273	278	29	10164	-0.62333	0.73347
-	LHX5	12	113452889	113507072	4	1	10164	-0.62365	0.73357
-	DDN	12	48985149	49036091	45	7	10164	-0.62375	0.7336
-	CDC42EP4	17	73273624	73347004	90	13	10164	-0.62437	0.73381
+	TKFC	11	61298182	61363139	67	4	10164	-0.62438	0.73381
+	CLDN10	13	95398599	95589756	670	50	10164	-0.62452	0.73386
-	CLDN14	21	36450621	36615085	514	34	10164	-0.62459	0.73388
+	EFCAB7	1	63488301	63595365	240	15	10164	-0.62459	0.73388
+	UBXN8	8	30694107	30777006	312	18	10164	-0.62469	0.73391
-	KLF16	19	1842399	1898565	148	17	10164	-0.62471	0.73392
-	POPDC3	6	105147900	105215009	175	25	10164	-0.62481	0.73395
+	IFNA1	9	21405454	21451316	173	31	10164	-0.62486	0.73397
-	ASPM	1	197074127	197181694	214	10	10164	-0.62498	0.73401

STRAND	SYMBOL	CHR	START	STOP	NSNPS	NPARAM	N	ZSTAT	P
-	HAGH	16	1799103	1862194	618	15	10164	-0.62513	0.73406
+	LOC1019295	1	39753982	39800171	162	13	10164	-0.62518	0.73407
-	PIGS	17	28543388	28606869	52	18	10164	-0.62549	0.73418
-	CFDP1	16	75283710	75468512	552	9	10164	-0.6257	0.73424
-	CORO7	16	4344542	4451961	9	4	10164	-0.62603	0.73435
-	RCAN2	6	46210730	46527067	524	27	10164	-0.6263	0.73444
-	C1RL	12	7083668	7144278	127	17	10164	-0.62631	0.73444
-	NDNL2	15	29258149	29304816	120	15	10164	-0.62682	0.73461
+	SLC25A48	5	135799676	135898637	284	30	10164	-0.62683	0.73461
+	MTFR1	8	65608873	65781252	395	23	10164	-0.62685	0.73462
-	FAM200A	7	99536300	99594395	74	12	10164	-0.62685	0.73462
+	SPIC	12	101440323	101497566	169	22	10164	-0.62699	0.73467
+	CASP2	7	143253215	143317696	8	2	10164	-0.62723	0.73474
+	TRIM39-RPP	6	30294311	30356858	271	36	10164	-0.62722	0.73474
+	CCR2	3	46318744	46370940	188	20	10164	-0.62741	0.7348
+	PNPLA3	22	43888739	43957568	204	24	10164	-0.62753	0.73484
-	SLTM	15	58869045	58969313	136	13	10164	-0.62762	0.73487
-	KEL	7	142931114	142997416	52	3	10164	-0.62766	0.73489
+	HLF	17	55229960	55335065	211	45	10164	-0.62774	0.73491
+	DAO	12	108845030	108911043	165	23	10164	-0.62785	0.73495
+	LOC1053724	20	1150661	1201878	66	14	10164	-0.62818	0.73506
+	ZNF559-ZNF	19	9289226	9392617	173	6	10164	-0.62825	0.73508
+	CYP4Z1	1	47032448	47128320	138	8	10164	-0.62858	0.73519
+	HMHB1	5	143777161	143830719	228	22	10164	-0.62872	0.73523
+	AGAP6	10	49938060	50020499	101	12	10164	-0.62872	0.73523
+	ABI2	2	203292874	203442173	263	32	10164	-0.62895	0.73531
+	ZNF253	19	19830830	19904449	154	19	10164	-0.6292	0.73539
+	CDK13	7	39914838	40107134	280	14	10164	-0.62924	0.73541
+	CRCP	7	66079818	66164568	295	19	10164	-0.62927	0.73541
+	SYT17	16	19132976	19278334	103	13	10164	-0.62942	0.73546
+	LRRC8D	1	89786014	89946432	209	33	10164	-0.62976	0.73557
-	LOC1053706	14	97650793	97697502	27	4	10164	-0.62973	0.73557
+	ZBTB38	3	141289213	141459792	427	61	10164	-0.62991	0.73562
+	TMEM117	12	43760764	44447808	1868	52	10164	-0.62998	0.73565
+	LOC1019298	5	630244	678743	204	22	10164	-0.63002	0.73566
+	RELL2	5	141601946	141651064	124	25	10164	-0.63013	0.73569
+	KRTAP9-4	17	41214687	41260653	129	12	10164	-0.63017	0.73571
-	CD200R1	3	112911057	113010122	396	30	10164	-0.63033	0.73576
-	STAC3	12	57233453	57286193	82	15	10164	-0.63038	0.73578
+	GPR150	5	95583296	95631672	66	15	10164	-0.63046	0.7358
-	DGCR14	22	19120279	19179726	207	36	10164	-0.63058	0.73584
+	CCNG1	5	163402270	163455016	104	22	10164	-0.63095	0.73596
+	SCP2D1	20	18778726	18824392	192	37	10164	-0.63101	0.73598
+	METTL12	11	62630263	62677451	45	8	10164	-0.63116	0.73603
+	STX12	1	27738183	27834452	111	5	10164	-0.63124	0.73606
+	C16orf62	16	19520073	19711163	653	42	10164	-0.63129	0.73608
+	TUBA3D	2	131441007	131492934	4	1	10164	-0.63135	0.73609
-	C20orf78	20	18799351	18865203	186	34	10164	-0.63188	0.73627
+	PGM1	1	63558276	63670245	288	38	10164	-0.632	0.73631
+	ZAN	7	100698583	100807797	254	34	10164	-0.63214	0.73635
-	GHRH	20	37241065	37296878	67	16	10164	-0.63236	0.73642
+	NUDT19	19	32631296	32723796	437	32	10164	-0.63261	0.73651
+	OR51F2	11	4786386	4832414	176	11	10164	-0.63271	0.73654
+	ALG10B	12	38281686	38339726	227	23	10164	-0.63281	0.73657
+	ZCCHC4	4	25277774	25383176	346	31	10164	-0.63303	0.73664
-	LRRC15	3	194345247	194404743	73	3	10164	-0.63303	0.73664
+	ZBTB42	14	104765596	104814712	162	29	10164	-0.63304	0.73665
-	ABCB11	2	168912802	169069890	592	40	10164	-0.63328	0.73672
+	TRIM6-TRIM3	11	5561635	5654398	461	48	10164	-0.63334	0.73675
-	POMGNT1	1	46178681	46255305	201	15	10164	-0.63354	0.73681
+	RCOR1	14	102557639	102740576	366	22	10164	-0.63364	0.73684
-	CYP2J2	1	59883308	59961773	219	24	10164	-0.63371	0.73686
-	GCG	2	162132869	162187404	102	15	10164	-0.63369	0.73686

STRAND	SYMBOL	CHR	START	STOP	NSNPS	NPARAM	N	ZSTAT	P
-	LOC1006529	22	25333582	25380261	119	11	10164	-0.63384	0.73691
-	RAD1	5	34895260	34950675	116	22	10164	-0.63422	0.73703
-	PREX1	20	48614249	48862883	826	34	10164	-0.63424	0.73704
+	C15orf43	15	44921633	44989223	129	12	10164	-0.63425	0.73704
-	OR56A4	11	5992051	6038148	190	19	10164	-0.6344	0.73709
-	OR1E2	17	3422870	3468841	170	12	10164	-0.63493	0.73726
-	ST5	11	8683352	8945951	625	35	10164	-0.63546	0.73743
+	CTR9	11	10715987	10789755	96	18	10164	-0.63597	0.7376
+	UHMK1	1	162462174	162539629	315	27	10164	-0.63611	0.73765
-	MRPL16	11	59796135	59845872	93	9	10164	-0.63618	0.73767
-	NUDCD1	8	109230919	109369121	386	25	10164	-0.63616	0.73767
+	EIF3J	15	44502068	44572803	167	18	10164	-0.63624	0.73769
+	GIPC3	19	3550541	3603541	51	8	10164	-0.63646	0.73776
-	RGS16	1	182588623	182639413	113	21	10164	-0.6366	0.73781
-	ANO10	3	43356326	43726594	421	19	10164	-0.63672	0.73785
+	HIST2H2AC	1	149851975	149897411	9	5	10164	-0.63683	0.73788
-	HIST2H4B	1	149850764	149896159	9	5	10164	-0.63683	0.73788
+	PTPN6	12	6911576	6971316	36	4	10164	-0.63701	0.73794
+	MRPL49	11	65087183	65137369	109	25	10164	-0.63712	0.73798
+	TRIM39	6	30291844	30353729	278	36	10164	-0.63721	0.73801
-	KRT82	12	52383951	52441392	205	20	10164	-0.63733	0.73804
-	BMP8B	1	39747182	39823861	261	20	10164	-0.63753	0.73811
-	GUCY2C	12	14602632	14731618	302	28	10164	-0.63772	0.73817
+	B3GNT7	2	231360624	231418801	87	12	10164	-0.63776	0.73819
+	SPTLC3	20	12973979	13180944	679	55	10164	-0.63781	0.7382
+	MRPS11	15	88432453	88488630	184	11	10164	-0.63786	0.73822
-	ABCD2	12	39540353	39686328	409	46	10164	-0.63789	0.73823
+	ZCWPW2	3	28314149	28535142	295	15	10164	-0.63828	0.73835
-	MN1	22	27738277	27836498	279	63	10164	-0.63835	0.73838
-	ATP5J2-PTC	7	99406739	99501201	183	21	10164	-0.63837	0.73838
+	LACTB	15	63086800	63152065	326	27	10164	-0.63895	0.73857
+	NF1	17	30972873	31387677	413	12	10164	-0.63981	0.73885
+	NR2F2	15	96290928	96350263	92	16	10164	-0.63982	0.73885
+	LOC1053757	8	126522485	126644564	385	40	10164	-0.63989	0.73888
+	C15orf27	15	76024946	76214963	469	51	10164	-0.64	0.73891
-	NRARP	9	137289631	137337251	70	9	10164	-0.64002	0.73892
-	JMJD8	16	671667	719441	141	21	10164	-0.64018	0.73897
+	RPP21	6	30310129	30356858	195	33	10164	-0.64022	0.73898
+	CUL5	11	107973682	108117766	356	30	10164	-0.64028	0.739
+	LOC1053776	2	23347827	23412338	213	33	10164	-0.64027	0.739
-	CST4	20	23675640	23724025	74	10	10164	-0.64044	0.73906
-	LRRC41	1	46268400	46338366	276	11	10164	-0.64055	0.73909
-	LOC400927-(	22	38280691	38433522	347	54	10164	-0.64053	0.73909
+	KLHL41	2	169474702	169536262	124	14	10164	-0.64056	0.7391
-	FAM19A2	12	61698248	62295064	1760	103	10164	-0.64077	0.73916
+	SEMA4F	2	74619228	74704897	71	3	10164	-0.64087	0.7392
+	PODXL2	3	127594159	127682810	112	23	10164	-0.64106	0.73926
+	YWHAH	22	31909493	31967603	194	37	10164	-0.64142	0.73938
-	CRYGC	2	208118137	208174738	233	22	10164	-0.64168	0.73946
-	POLDIP3	22	42573721	42649962	233	28	10164	-0.64173	0.73947
+	KCNE4	2	223016930	223065637	211	25	10164	-0.64216	0.73962
-	TBC1D20	20	425477	497553	181	26	10164	-0.64217	0.73962
-	MTHFR	1	11775730	11841103	374	15	10164	-0.64223	0.73964
-	LOC1053768	18	11625709	11676066	40	4	10164	-0.64232	0.73967
-	NDUFB7	19	14556078	14607077	88	16	10164	-0.64242	0.7397
+	ZNF76	6	35224488	35305987	294	32	10164	-0.64247	0.73972
-	HYOU1	11	119034185	119092246	186	17	10164	-0.64248	0.73972
+	C5orf52	5	157636237	157690176	141	15	10164	-0.64279	0.73982
-	ATP6V1G2	6	31534451	31581848	311	41	10164	-0.64285	0.73984
-	SACS	13	23318823	23468728	455	54	10164	-0.64296	0.73987
+	KRBOX1	3	42901342	42952792	89	9	10164	-0.64295	0.73987
-	SCAP	3	47403694	47512126	122	17	10164	-0.64299	0.73988
+	PLEKHA5	12	19094692	19386400	1028	43	10164	-0.64301	0.73989

STRAND	SYMBOL	CHR	START	STOP	NSNPS	NPARAM	N	ZSTAT	P
+	OCIAD1	4	48796043	48871817	131	25	10164	-0.64306	0.73991
-	NUDT6	4	122882644	122958004	193	19	10164	-0.64313	0.73993
+	SPATA7	14	88350638	88480350	188	21	10164	-0.6432	0.73995
+	KCNS3	2	17842847	17942961	335	30	10164	-0.64359	0.74008
+	UBE3C	7	157103959	157279372	529	21	10164	-0.64369	0.74011
+	SSPO	7	149741042	149843965	138	18	10164	-0.64434	0.74032
+	CYSTM1	5	140140068	140253789	225	14	10164	-0.64437	0.74033
+	TPM4	19	16032507	16113005	201	19	10164	-0.64446	0.74036
+	KCNJ14	19	48420707	48476110	141	18	10164	-0.64468	0.74043
-	FLII	17	18234815	18293916	150	24	10164	-0.64477	0.74046
+	LMAN1L	15	74777853	74835758	193	29	10164	-0.64484	0.74048
-	HAUS8	19	17039761	17110608	347	28	10164	-0.64492	0.74051
-	R3HDM4	19	886503	948225	71	10	10164	-0.64507	0.74056
+	SORL1	11	121417203	121643762	324	32	10164	-0.64517	0.74059
+	SNX9	6	157681046	157955077	857	42	10164	-0.64523	0.74061
+	CEP95	17	64471762	64547951	99	3	10164	-0.64521	0.74061
-	TNNI1	1	201393767	201456746	200	33	10164	-0.6456	0.74073
+	CAMSAP2	1	200704558	200870707	486	31	10164	-0.6457	0.74076
-	KRCC1	2	88017203	88090801	22	5	10164	-0.64584	0.74081
-	MARCH11	5	16057365	16214788	233	32	10164	-0.64585	0.74081
+	ST8SIA2	15	92358910	92478728	422	47	10164	-0.6464	0.74099
+	HTR3C	3	184018047	184070673	188	29	10164	-0.64652	0.74103
+	FOLR1	11	72154558	72206323	185	22	10164	-0.64676	0.74111
+	ZNF493	19	21362119	21437495	249	14	10164	-0.64699	0.74118
-	BECN1	17	42800132	42859292	69	13	10164	-0.64702	0.74119
+	EMP3	19	48290370	48340555	136	11	10164	-0.64704	0.7412
-	ABCC9	12	21787389	21976863	200	27	10164	-0.64722	0.74125
+	TBX2	17	61359698	61419466	145	20	10164	-0.64726	0.74127
-	DENND2D	1	111175969	111239791	165	29	10164	-0.64727	0.74127
-	COPS5	8	67033079	67097327	86	7	10164	-0.64742	0.74132
-	SIKE1	1	114759479	114815687	74	15	10164	-0.64744	0.74133
+	OPRL1	20	64045118	64110643	124	19	10164	-0.6475	0.74135
+	ALAS1	3	52163083	52224327	111	23	10164	-0.64773	0.74142
-	RNLS	10	88170643	88618854	1312	95	10164	-0.64792	0.74148
-	RBKS	2	27771364	27925396	269	20	10164	-0.64796	0.7415
+	BGLAP	1	156207160	156253332	107	15	10164	-0.64839	0.74163
+	FGFR1OP	6	166964317	167052418	454	13	10164	-0.64922	0.7419
+	DCAF4L1	4	41946696	41996467	86	6	10164	-0.64943	0.74197
+	FEM1B	15	68242756	68305865	161	13	10164	-0.64954	0.74201
-	CD3D	11	118329074	118377744	104	13	10164	-0.64994	0.74214
-	HOOK2	19	12753003	12813484	17	7	10164	-0.65	0.74215
+	UQCRH	1	46268613	46326777	178	11	10164	-0.65035	0.74227
-	OR4K14	14	20004261	20050193	95	10	10164	-0.65035	0.74227
-	ATP10B	5	160553120	160964368	1525	105	10164	-0.65045	0.7423
-	POLG2	17	64466994	64532066	94	3	10164	-0.65057	0.74234
-	CLDN17	21	30155923	30201653	95	15	10164	-0.65066	0.74237
-	ESM1	5	54967867	55020586	171	20	10164	-0.65094	0.74246
+	ZNF276	16	89685195	89750925	367	31	10164	-0.65104	0.74249
+	PSMA8	18	26098836	26203355	224	14	10164	-0.65139	0.7426
+	LOC1053728	21	43156604	43205876	3	2	10164	-0.65183	0.74275
-	KDSR	18	63317738	63402273	182	28	10164	-0.65191	0.74277
+	LOC643355	1	111954995	112008842	132	23	10164	-0.65204	0.74281
-	C2orf83	2	227600090	227668320	360	30	10164	-0.65212	0.74284
+	PLEK	2	68330190	68407453	402	18	10164	-0.65266	0.74301
+	NMRK2	19	3898103	3952416	52	7	10164	-0.65268	0.74302
+	FGGY	1	59261405	59772730	1143	83	10164	-0.65276	0.74304
+	ZNF101	19	19633153	19693506	183	29	10164	-0.65296	0.74311
-	NDUFAF4	6	96879311	96932891	174	27	10164	-0.65299	0.74312
+	EXOSC4	8	144029087	144090648	39	1	10164	-0.65305	0.74314
+	NAB1	2	190613895	190702766	235	33	10164	-0.65325	0.7432
+	RPS28	19	8286500	8332396	65	3	10164	-0.65368	0.74334
+	AMZ2	17	68170997	68267164	424	24	10164	-0.65375	0.74336
-	NOD1	7	30414527	30513797	203	22	10164	-0.65462	0.74364

STRAND	SYMBOL	CHR	START	STOP	NSNPS	NPARAM	N	ZSTAT	P
+	CRYBB3	22	25163686	25217363	210	23	10164	-0.65493	0.74374
-	IPO4	14	24170219	24223918	111	18	10164	-0.65505	0.74378
+	CHMP4B	20	33776304	33864367	142	24	10164	-0.65506	0.74379
+	CYB561D2	3	50315695	50378197	86	13	10164	-0.65516	0.74382
-	GFM2	5	74711204	74802371	159	12	10164	-0.65525	0.74385
-	PXDN	2	1621887	1779852	421	44	10164	-0.65533	0.74387
+	APCS	1	159552826	159598871	141	17	10164	-0.65535	0.74388
+	METTL15	11	28073251	28537041	690	24	10164	-0.65546	0.74392
-	ZNF648	1	182043766	182097505	159	20	10164	-0.65583	0.74403
+	MTRNR2L9	6	61539103	61584629	239	11	10164	-0.6559	0.74405
+	LOC1053734	2	696290	789111	298	10	10164	-0.6559	0.74405
-	SLC25A51	9	37867575	37939353	184	15	10164	-0.65618	0.74415
-	SOGA1	20	36767442	36898684	175	32	10164	-0.65621	0.74416
-	P4HA1	10	72997217	73131974	261	6	10164	-0.65648	0.74424
-	HSPB7	1	16004028	16053796	286	26	10164	-0.65655	0.74426
-	CD163L1	12	7316484	7479185	374	41	10164	-0.65654	0.74426
-	WDR59	16	74863568	75020123	385	45	10164	-0.65663	0.74429
+	TSTD3	6	99485827	99589288	273	36	10164	-0.65668	0.74431
-	SLC25A39	17	44309625	44359857	87	15	10164	-0.65682	0.74435
-	LGI3	8	22136830	22191831	53	10	10164	-0.65685	0.74436
-	AMOTL2	3	134345345	134410417	130	21	10164	-0.65698	0.7444
-	PIF1	15	64805630	64862154	140	11	10164	-0.65724	0.74449
-	C22orf39	22	19430886	19483232	142	21	10164	-0.65737	0.74453
-	CCNC	6	99532387	99603973	235	29	10164	-0.65786	0.74469
+	RPRD1B	20	37998487	38102364	172	17	10164	-0.65786	0.74469
-	MGEA5	10	101774443	101853709	130	28	10164	-0.65796	0.74472
-	LOC1019278	17	61351594	61418553	156	23	10164	-0.6581	0.74476
+	NLRX1	11	119133334	119194016	199	17	10164	-0.65847	0.74488
+	ADAL	15	43295356	43364555	162	14	10164	-0.65848	0.74488
+	GDAP1L1	20	44212099	44290917	289	26	10164	-0.65896	0.74504
+	ATP11A	13	112655049	112897168	668	43	10164	-0.659	0.74505
-	ERMP1	9	5754061	5868081	331	27	10164	-0.65906	0.74507
-	SETSIP	1	92064436	92110472	60	11	10164	-0.65907	0.74507
-	SMAGP	12	51235349	51305418	203	27	10164	-0.65915	0.7451
+	DTX1	12	113021714	113108028	172	22	10164	-0.65917	0.74511
-	RASGRP2	11	64716911	64780456	99	12	10164	-0.65922	0.74512
+	P2RX2	12	132583780	132632462	240	25	10164	-0.65923	0.74513
+	ANKS1A	6	34854261	35108010	945	52	10164	-0.6593	0.74515
-	C5orf63	5	127025666	127108539	198	11	10164	-0.65929	0.74515
-	C6orf1	6	34236380	34284108	206	24	10164	-0.65956	0.74523
-	LOC1001311	1	152069413	152115924	108	7	10164	-0.65958	0.74524
-	CALHM3	10	103462804	103514240	132	23	10164	-0.65995	0.74536
+	LOC1053753	7	39913746	39960638	76	9	10164	-0.65996	0.74536
-	CRYGN	7	151419970	151483419	177	31	10164	-0.6602	0.74544
+	SV2C	5	76048414	76363939	1079	80	10164	-0.66037	0.74549
-	VPS37C	11	61120256	61196444	103	8	10164	-0.66044	0.74551
+	CLCN6	1	11771096	11853144	455	20	10164	-0.6605	0.74554
+	THAP11	16	67807310	67854195	50	10	10164	-0.66066	0.74559
+	BEST2	19	12716676	12768458	10	5	10164	-0.66127	0.74578
+	UBE2E2	3	23168007	23600805	1428	28	10164	-0.66148	0.74585
-	COL21A1	6	56046590	56429122	1481	50	10164	-0.6615	0.74586
+	LOC1053781	6	83673512	83746441	145	27	10164	-0.66184	0.74596
-	TRPV6	7	142861203	142920745	83	3	10164	-0.66193	0.74599
-	MAP1LC3C	1	241985490	242036399	69	17	10164	-0.66193	0.74599
+	KLC3	19	45305740	45361559	152	26	10164	-0.66198	0.74601
-	NPIPB4	16	21824568	21915845	8	5	10164	-0.66199	0.74601
+	DCUN1D4	4	51808000	51926837	149	10	10164	-0.66225	0.7461
+	SNRNP35	12	123423026	123482362	39	6	10164	-0.66229	0.74611
-	ZC3H13	13	45945684	46087761	309	14	10164	-0.66231	0.74611
-	USP40	2	233465519	233601787	396	15	10164	-0.66233	0.74612
+	PSMD13	11	201808	262984	284	22	10164	-0.66237	0.74613
-	C1QTNF6	22	37170166	37233301	230	23	10164	-0.66254	0.74619
+	ATP6V0E2	7	149837968	149890713	14	2	10164	-0.66266	0.74622

STRAND	SYMBOL	CHR	START	STOP	NSNPS	NPARAM	N	ZSTAT	P
+	IGHMBP2	11	68868851	68950601	369	28	10164	-0.66268	0.74623
-	ARSB	5	78767209	79021534	1026	73	10164	-0.66276	0.74626
-	SUPT7L	2	27632568	27698973	79	19	10164	-0.663	0.74634
-	AMN1	12	31661137	31765392	321	36	10164	-0.66307	0.74636
-	KIAA0101	15	64354994	64422687	84	7	10164	-0.66351	0.7465
-	THBS3	1	155185584	155244180	46	9	10164	-0.66358	0.74652
+	FAM71D	14	67154393	67238550	259	19	10164	-0.66362	0.74653
+	NEURL1B	5	172606247	172701540	271	39	10164	-0.66373	0.74657
+	COL8A1	3	99603596	99806733	532	42	10164	-0.66388	0.74662
+	ALB	4	73369255	73431412	104	11	10164	-0.66408	0.74668
+	PROX1	1	213948226	214051510	212	33	10164	-0.66476	0.7469
+	HSPA6	1	161489540	161536897	90	16	10164	-0.66482	0.74692
-	NEUROD6	7	31327461	31375924	52	10	10164	-0.66502	0.74698
-	EPS15L1	19	16345244	16507012	278	17	10164	-0.66517	0.74703
-	RELN	7	103461784	104024516	2373	132	10164	-0.66533	0.74708
+	PTPN18	2	130321007	130385409	35	4	10164	-0.6654	0.7471
+	LOC1027248	3	194035145	194133474	55	12	10164	-0.6655	0.74713
+	SPINK13	5	148233860	148296210	233	17	10164	-0.66572	0.7472
-	EXOC6B	2	72165984	72861048	880	17	10164	-0.66587	0.74725
-	ZNF91	19	23347696	23430560	329	18	10164	-0.66632	0.7474
-	BAIAP2L1	7	98281650	98436115	535	35	10164	-0.66643	0.74743
+	PABPC1L	20	44875058	44968801	188	19	10164	-0.66643	0.74743
+	TMEM132B	12	125151774	125672377	776	66	10164	-0.66644	0.74744
+	PAAF1	11	73841699	73937736	287	16	10164	-0.66659	0.74748
+	ACADM	1	75689347	75773679	251	17	10164	-0.66663	0.7475
+	POU3F3	2	104820511	104867013	8	4	10164	-0.66737	0.74773
+	CTDSPL2	15	44392329	44537257	311	16	10164	-0.66774	0.74785
+	RAP1B	12	68575839	68670605	119	18	10164	-0.6679	0.7479
-	EPHA10	1	37703886	37800152	329	26	10164	-0.66792	0.74791
+	FCRLB	1	161686544	161738143	212	30	10164	-0.66799	0.74793
+	MMP2	16	55443830	55516691	311	42	10164	-0.66825	0.74801
+	FAM26E	6	116476645	116528546	204	21	10164	-0.66826	0.74802
+	ATRAID	2	27177031	27227178	117	15	10164	-0.6685	0.74809
+	SPECC1L	22	24235817	24427740	310	15	10164	-0.66874	0.74817
+	CACNG4	17	66929864	67043402	202	24	10164	-0.66887	0.74821
+	POM121L12	7	53000656	53046925	192	25	10164	-0.66899	0.74825
-	EPS8L3	1	109740080	109799022	49	17	10164	-0.66916	0.7483
+	TBC1D14	4	6874444	7043118	495	29	10164	-0.66927	0.74834
-	PDE5A	4	119484395	119663826	515	18	10164	-0.66941	0.74838
+	LYRM7	5	131135914	131215426	133	10	10164	-0.66954	0.74842
+	MEPCE	7	100393698	100444126	82	11	10164	-0.66979	0.74851
+	HHLA3	1	70319797	70378023	122	24	10164	-0.66996	0.74856
-	DRD1	5	175430672	175479160	177	23	10164	-0.67005	0.74859
+	MS4A18	11	60689009	60754213	146	15	10164	-0.67057	0.74875
-	UBE2N	12	93398312	93478445	138	16	10164	-0.67059	0.74876
+	TRAF3IP3	1	209721032	209792323	222	23	10164	-0.67073	0.7488
-	GADL1	3	30716200	30929661	952	77	10164	-0.67072	0.7488
+	SPRR4	1	152935653	152982574	188	12	10164	-0.67086	0.74885
+	LOC1053725	20	22995889	23049219	153	16	10164	-0.67089	0.74886
+	LOC1053728	21	43702000	43748097	112	14	10164	-0.67091	0.74886
-	SIGLEC12	19	51481227	51536789	325	25	10164	-0.67122	0.74896
+	TMPRSS4	11	118042012	118135505	446	36	10164	-0.67132	0.74899
-	MYADML2	17	81929645	81982233	11	2	10164	-0.6715	0.74905
+	TRIM28	19	58509469	58560715	45	9	10164	-0.67179	0.74914
-	ZNF785	16	30563219	30621421	18	4	10164	-0.67186	0.74916
-	CD48	1	160668746	160746851	42	9	10164	-0.6721	0.74924
+	C17orf82	17	61376751	61423280	132	18	10164	-0.67215	0.74925
-	ELAC2	17	12981612	13053064	350	28	10164	-0.67221	0.74927
+	LOC1053789	1	39749636	39818042	231	18	10164	-0.67218	0.74927
-	LOC1019294	15	85195389	85269795	33	8	10164	-0.67246	0.74935
-	CHMP1A	16	89634431	89692785	293	24	10164	-0.67247	0.74936
-	SYT2	1	202577637	202745444	449	53	10164	-0.67253	0.74938
+	CRYBA4	22	26572987	26640672	263	34	10164	-0.67325	0.74961

STRAND	SYMBOL	CHR	START	STOP	NSNPS	NPARAM	N	ZSTAT	P
+	DZIP3	3	108554490	108704846	226	18	10164	-0.67328	0.74961
+	BMS1	10	42747506	42844937	247	19	10164	-0.67325	0.74961
-	DENND1C	19	6457204	6516808	17	2	10164	-0.67339	0.74965
-	LOC1053789	2	91488319	91541909	7	4	10164	-0.67366	0.74973
-	LOC1053789	2	91497532	91548275	7	4	10164	-0.67366	0.74973
+	GAA	17	80066531	80129881	312	18	10164	-0.67368	0.74974
+	UBXN2B	8	58376264	58461501	326	35	10164	-0.67373	0.74976
+	PIK3CG	7	106830278	106918978	174	18	10164	-0.67382	0.74979
-	DEPDC1B	5	60586912	60735166	333	19	10164	-0.67382	0.74979
-	PDZRN3	3	73372430	73659921	717	66	10164	-0.67394	0.74983
+	RANBP17	5	170826882	171310450	955	15	10164	-0.67401	0.74985
-	TRPM3	9	70519063	71481924	3080	117	10164	-0.67414	0.74989
+	LOC646730	3	141625344	141740357	492	46	10164	-0.67439	0.74997
-	IVNS1ABP	1	185286388	185352343	119	20	10164	-0.67441	0.74998
+	RHBDD1	2	226800936	227009210	433	20	10164	-0.67442	0.74998
+	ADCYAP1	18	869345	922172	194	13	10164	-0.67459	0.75003
-	LOC1053723	19	17379069	17429158	140	14	10164	-0.67465	0.75005
-	PDCL	9	122808097	122863656	80	16	10164	-0.67484	0.75011
+	EIF4A1	17	7537706	7589006	167	27	10164	-0.6749	0.75013
+	RYR1	19	38398700	38597564	845	82	10164	-0.67505	0.75018
-	PCNXL2	1	232974101	233330713	1155	88	10164	-0.67515	0.75021
-	LOC1027242	9	89654915	89707876	239	31	10164	-0.67516	0.75021
-	LRRIQ3	1	74016015	74233201	352	13	10164	-0.67537	0.75028
+	NR2F1	5	93548337	93604611	48	8	10164	-0.67545	0.7503
-	CRY1	12	106981364	107128857	410	22	10164	-0.67554	0.75033
+	TMEM202	15	72363101	72418367	91	8	10164	-0.67555	0.75034
+	LOC1053731	1	228612614	228660007	49	10	10164	-0.67557	0.75034
+	CSPP1	8	67027417	67206614	212	11	10164	-0.67572	0.75039
+	ALPPL2	2	232371842	232420714	143	17	10164	-0.67589	0.75044
-	HEXA	15	72333437	72411179	137	12	10164	-0.67607	0.7505
+	LOC1053696	12	10688107	10737487	151	16	10164	-0.67636	0.75059
+	ACSL5	10	112339158	112438380	198	17	10164	-0.67648	0.75063
+	NEU4	2	241774065	241827413	86	11	10164	-0.67658	0.75066
-	NPLOC4	17	81546885	81672153	461	18	10164	-0.67668	0.7507
-	KRTAP10-1	21	44529185	44575195	140	20	10164	-0.67679	0.75073
+	WNT5B	12	1494891	1657213	482	81	10164	-0.67727	0.75088
-	C6orf226	6	42880265	42925816	126	10	10164	-0.67726	0.75088
+	C19orf24	19	1240521	1289244	30	10	10164	-0.67735	0.75091
+	MOG	6	29621981	29682372	434	46	10164	-0.67739	0.75092
-	NCOR2	12	124314411	124602464	820	120	10164	-0.6776	0.75099
+	FIG4	6	109656221	109835431	507	44	10164	-0.67793	0.75109
-	CCDC6	10	59778747	59941656	547	27	10164	-0.6782	0.75118
-	F13B	1	197029191	197102267	176	9	10164	-0.67826	0.7512
+	SLC30A9	4	41955472	42097534	356	7	10164	-0.67839	0.75124
+	PPP2R1A	19	52154802	52236425	281	21	10164	-0.67843	0.75125
-	BAIAP2L2	22	38074883	38146304	224	33	10164	-0.67859	0.7513
+	OR1J4	9	122484141	122530082	118	24	10164	-0.67875	0.75135
-	FADS6	17	74866776	74928811	143	17	10164	-0.67874	0.75135
+	GUCA2B	1	42118421	42165824	116	14	10164	-0.67887	0.75139
-	VWCE	11	61248286	61330316	100	7	10164	-0.67889	0.7514
+	AHSG	3	186578061	186631318	120	14	10164	-0.67945	0.75157
+	CBWD2	2	113402691	113506204	73	4	10164	-0.67954	0.7516
-	SFTPA2	10	79545852	79595459	192	16	10164	-0.68026	0.75183
+	CCDC102B	18	68680254	69181081	1554	83	10164	-0.68037	0.75187
-	C12orf80	12	52195581	52245855	89	13	10164	-0.68082	0.75201
-	HDAC5	17	44066753	44158646	142	20	10164	-0.68092	0.75204
-	PRPH2	6	42686595	42757989	197	23	10164	-0.68111	0.7521
-	SEMA4D	9	89350791	89533014	581	56	10164	-0.68117	0.75212
+	PAK6	15	40182428	40287487	227	44	10164	-0.68127	0.75215
-	ERBB4	2	211365717	212574155	3070	153	10164	-0.68133	0.75217
+	ZNF474	5	122094513	122163571	73	11	10164	-0.68136	0.75218
-	SLC25A13	7	96110220	96357147	490	28	10164	-0.68148	0.75222
-	SRSF9	12	120451668	120504755	166	20	10164	-0.68153	0.75223

STRAND	SYMBOL	CHR	START	STOP	NSNPS	NPARAM	N	ZSTAT	P
-	NEDD9	6	11173298	11417348	795	96	10164	-0.68189	0.75235
+	AKAP11	13	42236481	42333267	333	26	10164	-0.6824	0.75251
-	MYOG	1	203073129	203121038	120	25	10164	-0.68246	0.75253
-	C12orf56	12	64254762	64425584	416	27	10164	-0.68296	0.75269
+	PLEKHH2	2	43602300	43777987	760	46	10164	-0.68299	0.75269
+	GFI1B	9	132910545	133003434	235	41	10164	-0.68304	0.75271
+	NCOA7	6	125746131	125942030	467	39	10164	-0.68304	0.75271
+	RIIAD1	1	151686537	151739606	120	21	10164	-0.68306	0.75272
-	RTTN	18	69993804	70240726	62	18	10164	-0.68313	0.75274
+	GRIA2	4	157185584	157376075	291	25	10164	-0.68317	0.75275
+	TPST1	7	66170272	66370451	505	27	10164	-0.68333	0.7528
+	IFITM1	11	278991	325272	98	20	10164	-0.68334	0.7528
+	AFF1	4	86899511	87151054	688	43	10164	-0.68336	0.75281
-	KCNK10	14	88170108	88361912	774	77	10164	-0.68372	0.75293
-	PECAM1	17	64309415	64448801	113	10	10164	-0.68377	0.75294
+	SLX1A	16	30158843	30207566	12	2	10164	-0.68387	0.75297
+	DNAJC5	20	63860102	63946031	212	18	10164	-0.68393	0.75299
+	MRI1	19	13729492	13784282	157	17	10164	-0.68414	0.75306
-	MRGPRE	11	3217811	3267386	146	19	10164	-0.68418	0.75307
-	RAB35	12	120085094	120151839	122	21	10164	-0.68442	0.75314
+	MTF2	1	93044235	93149081	194	19	10164	-0.68446	0.75316
-	TSSC1	2	3178970	3412882	876	38	10164	-0.68453	0.75318
+	SLC9A3R2	16	1991868	2049026	122	16	10164	-0.68453	0.75318
-	USP33	1	77685987	77794879	147	22	10164	-0.68476	0.75325
-	MICU2	13	21482689	21639216	263	12	10164	-0.68507	0.75335
+	SPATA33	16	89622744	89680458	299	26	10164	-0.68523	0.7534
-	USP45	6	99422306	99550777	276	38	10164	-0.68537	0.75344
+	UBE2I	16	1274153	1337018	139	21	10164	-0.68537	0.75345
-	PHF5A	22	41449717	41503704	86	17	10164	-0.68543	0.75346
+	KLHL23	2	169698846	169761886	282	31	10164	-0.68543	0.75346
+	SMAD3	15	67030627	67205195	621	93	10164	-0.68575	0.75356
-	FXYD6	11	117826976	117912486	460	47	10164	-0.68586	0.7536
-	EFTUD2	17	44840287	44934625	273	28	10164	-0.6861	0.75367
-	ENAH	1	225476832	225696676	538	35	10164	-0.68608	0.75367
+	OTOP2	17	74887963	74943913	120	17	10164	-0.68621	0.75371
-	LOC1002874	19	50048266	50095497	68	11	10164	-0.68638	0.75376
-	ALDOC	17	28563115	28611948	39	17	10164	-0.68643	0.75378
-	ANGEL1	14	76777243	76863473	219	26	10164	-0.68678	0.75389
+	TSKU	11	76747313	76808154	69	10	10164	-0.68696	0.75395
-	MEIS3	19	47393118	47457265	161	18	10164	-0.68764	0.75416
+	FOXP4	6	41511426	41612384	247	31	10164	-0.68808	0.7543
+	HHIP	4	144610996	144751400	244	21	10164	-0.68819	0.75433
+	ZNF155	19	43932703	44008326	300	23	10164	-0.68838	0.75439
+	TATDN3	1	212756828	212826825	311	12	10164	-0.68851	0.75443
+	STH	17	45964250	46009694	8	3	10164	-0.68959	0.75477
+	GRAMD1B	11	123323784	123637771	568	82	10164	-0.68968	0.7548
-	SERBP1	1	67397810	67465440	95	14	10164	-0.69009	0.75493
+	ATN1	12	6889463	6952321	21	5	10164	-0.6903	0.755
+	CCDC107	9	35623282	35671503	164	30	10164	-0.69046	0.75505
-	SNX13	7	17780761	17975518	403	18	10164	-0.69049	0.75506
+	SIGLEC9	19	51089880	51151020	196	48	10164	-0.69052	0.75507
+	CADM2	3	84923982	86084429	3761	80	10164	-0.69095	0.7552
+	PHLDB1	11	118571439	118668038	194	24	10164	-0.69099	0.75522
-	ZMYND10	3	50331106	50381028	78	13	10164	-0.69101	0.75522
-	TAS2R31	12	11020387	11066407	125	7	10164	-0.6915	0.75537
-	CMA1	14	24495346	24543690	186	20	10164	-0.69154	0.75539
+	CR1	1	207461128	207651765	383	35	10164	-0.69169	0.75543
+	TAS1R3	1	1293999	1345320	97	8	10164	-0.69169	0.75543
+	TUBB6	18	12272669	12339826	241	18	10164	-0.6918	0.75547
-	LOC1019274	7	87141423	87191676	132	10	10164	-0.6919	0.7555
-	ENTPD5	14	73949467	74054326	471	34	10164	-0.69234	0.75564
-	TCHHL1	1	152074144	152124064	97	7	10164	-0.69239	0.75565
-	LOC1053751	6	35674467	35724106	161	30	10164	-0.69261	0.75572

STRAND	SYMBOL	CHR	START	STOP	NSNPS	NPARAM	N	ZSTAT	P
-	SEPT1	16	30368133	30417850	3	2	10164	-0.69268	0.75574
-	UGT2A2	4	69578417	69674642	397	32	10164	-0.69265	0.75574
+	CRIPT	2	46582172	46640176	160	18	10164	-0.69269	0.75575
+	MED18	1	28294002	28345967	73	12	10164	-0.69289	0.75581
-	SLC10A7	4	146243981	146556971	957	58	10164	-0.69289	0.75581
+	PDZD3	11	119150436	119200223	160	13	10164	-0.69291	0.75582
-	NADK2	5	36182589	36277279	332	16	10164	-0.693	0.75584
-	ADCY10	1	167799080	167950526	142	26	10164	-0.69339	0.75597
+	C11orf88	11	111479785	111547031	74	14	10164	-0.6936	0.75603
-	C2orf61	2	47076991	47190378	356	34	10164	-0.69368	0.75606
+	C7orf73	7	135627473	135686412	256	24	10164	-0.69412	0.7562
+	TUBA1C	12	49192926	49283338	179	30	10164	-0.69424	0.75624
-	RAB28	4	13286017	13519365	207	20	10164	-0.69451	0.75632
+	MCHR1	22	40644178	40692814	103	16	10164	-0.69472	0.75638
-	LOC1053713	16	75704024	75767740	194	19	10164	-0.69476	0.7564
-	LOC1053785	10	131820526	131868634	98	14	10164	-0.69498	0.75647
-	MC4R	18	60361331	60407768	99	12	10164	-0.69509	0.7565
-	CIB1	15	90220245	90300759	265	28	10164	-0.69528	0.75656
-	SH3GL1	19	4350367	4435568	143	13	10164	-0.69531	0.75657
-	PCOLCE2	3	142807860	142924203	203	34	10164	-0.69542	0.7566
+	OVOL1	11	65752034	65807219	177	34	10164	-0.6958	0.75672
-	POLG	15	89306305	89369795	162	24	10164	-0.69597	0.75678
-	XYLT1	16	17092324	17505881	1192	115	10164	-0.69598	0.75678
-	YPEL3	16	30082314	30131216	45	6	10164	-0.6962	0.75685
-	STX5	11	62796860	62867091	131	8	10164	-0.69626	0.75687
-	BATF3	1	212676417	212734985	221	28	10164	-0.69627	0.75687
-	ADSS	1	244398492	244487134	200	29	10164	-0.69646	0.75693
+	CISD3	17	38695257	38745605	71	19	10164	-0.6965	0.75694
+	SPATA13	13	23944626	24317074	1596	164	10164	-0.69665	0.75699
+	AFP	4	73388104	73465785	150	15	10164	-0.6967	0.757
+	NFKBIL1	6	31511851	31568829	447	47	10164	-0.69674	0.75702
+	JADE1	4	128774623	128885224	264	27	10164	-0.6968	0.75704
+	NHLRC4	16	531995	579495	104	18	10164	-0.69685	0.75705
+	HMGCR	5	75301334	75372101	236	23	10164	-0.69826	0.75749
+	MYC	8	127701069	127751434	121	49	10164	-0.69872	0.75764
+	SLC15A2	3	121859324	121954187	301	12	10164	-0.69908	0.75775
+	OR8H3	11	56087373	56133311	333	8	10164	-0.69932	0.75782
-	STPG2	4	97431146	98178470	2402	43	10164	-0.69933	0.75783
+	LOC1019274	10	130170332	130506873	1653	100	10164	-0.69937	0.75784
-	THEM4	1	151860866	151944885	217	17	10164	-0.69941	0.75785
-	LOC1005065	1	228063909	228113006	184	22	10164	-0.6994	0.75785
-	PDRG1	20	31934955	32003710	89	12	10164	-0.69987	0.75799
+	LOC1019281	1	154926560	154972625	63	13	10164	-0.69984	0.75799
-	HOXB8	17	48602346	48650112	180	33	10164	-0.69995	0.75802
-	MAP3K9	14	70712526	70844269	230	15	10164	-0.70042	0.75817
+	ACKR4	3	132562237	132612644	92	16	10164	-0.70054	0.7582
+	BBS5	2	169444496	169516655	225	23	10164	-0.70056	0.75821
+	SPINK14	5	148133546	148185619	264	14	10164	-0.70158	0.75853
-	POM121L2	6	27299063	27347232	157	28	10164	-0.70161	0.75854
+	TAX1BP1	7	27704373	27839767	348	34	10164	-0.7017	0.75857
-	MGLL	3	127679062	127858250	494	72	10164	-0.70255	0.75883
-	C9orf156	9	97866324	97957570	244	18	10164	-0.70261	0.75885
-	OR3A2	17	3267890	3313974	230	11	10164	-0.70272	0.75888
-	NR2C1	12	95010229	95108628	286	32	10164	-0.70287	0.75893
+	HPS6	10	102030367	102078038	70	19	10164	-0.70288	0.75893
+	MED28	4	17579628	17635628	148	6	10164	-0.70308	0.759
+	RAD51C	17	58657536	58744342	168	21	10164	-0.70316	0.75902
+	NKAPL	6	28224297	28270958	164	32	10164	-0.70323	0.75904
+	ALDH1B1	9	38357664	38408665	190	37	10164	-0.70329	0.75906
-	SGCB	4	52010695	52073319	75	10	10164	-0.70333	0.75908
-	CLEC4E	12	8523305	8575962	193	21	10164	-0.70338	0.75909
+	C17orf53	17	44106906	44172477	103	10	10164	-0.70349	0.75913
+	NCK2	2	105709942	105904274	834	45	10164	-0.7036	0.75916

STRAND	SYMBOL	CHR	START	STOP	NSNPS	NPARAM	N	ZSTAT	P
+	PSMA4	15	78505405	78559220	203	21	10164	-0.70378	0.75922
+	PTPRC	1	198603968	198767476	412	42	10164	-0.70379	0.75922
+	ZP3	7	76362524	76452071	175	14	10164	-0.70384	0.75924
+	GPSM2	1	108840350	108940422	217	20	10164	-0.70443	0.75942
+	OR8D4	11	123871432	123917376	176	26	10164	-0.70448	0.75943
+	DAZAP1	19	1372569	1445687	34	3	10164	-0.70498	0.75959
+	PCNXL4	14	60056827	60144814	260	14	10164	-0.70514	0.75964
-	PNISR	6	99388655	99460342	162	19	10164	-0.70522	0.75966
+	DYNC2LI1	2	43739039	43837950	441	25	10164	-0.70534	0.7597
+	PRSS16	6	27212723	27266620	237	35	10164	-0.70548	0.75974
-	CTU1	19	51087606	51143390	187	45	10164	-0.70557	0.75977
-	GGN	19	38374349	38423583	192	40	10164	-0.7057	0.75981
+	MANF	3	51350237	51399397	77	12	10164	-0.7059	0.75987
-	TTI1	20	37973007	38068468	159	18	10164	-0.70592	0.75988
+	HCRTR2	6	55139273	55292620	599	36	10164	-0.70597	0.7599
+	CCNI2	5	132712411	132766255	100	12	10164	-0.70625	0.75998
+	ASPA	17	3439110	3509406	292	30	10164	-0.70639	0.76003
+	LOC1053698	12	89317603	89465111	104	10	10164	-0.70643	0.76004
-	MDM1	12	68284566	68367381	163	25	10164	-0.70647	0.76005
-	CASP3	4	184617696	184684475	210	18	10164	-0.70661	0.7601
-	DUPD1	10	75027472	75108632	256	33	10164	-0.70685	0.76017
-	CYYR1	21	26456209	26608284	259	31	10164	-0.70694	0.7602
+	CGNL1	15	57341505	57560727	911	83	10164	-0.70707	0.76024
+	SLC18A3	10	49575301	49622720	128	25	10164	-0.70715	0.76026
-	SLC38A8	16	83999667	84078097	448	43	10164	-0.70717	0.76027
+	FOXN1	17	28471229	28548900	128	20	10164	-0.70774	0.76045
-	BST2	19	17392939	17440648	134	14	10164	-0.70785	0.76048
+	CTSL	9	87690519	87741469	118	16	10164	-0.70797	0.76052
+	IFI30	19	18138780	18188124	136	17	10164	-0.70813	0.76057
-	CRYZL1	21	33579341	33676854	95	14	10164	-0.70827	0.76061
+	MED30	8	117485713	117550262	220	35	10164	-0.70868	0.76074
+	TTC39C	18	23957773	24145610	377	50	10164	-0.70895	0.76082
+	FUBP3	9	130543857	130648352	327	26	10164	-0.70896	0.76083
+	SMIM20	4	25879192	25939879	129	14	10164	-0.70911	0.76087
-	C6orf222	6	36305757	36371977	226	48	10164	-0.70925	0.76092
+	SEC61A2	10	12094641	12179958	231	24	10164	-0.70934	0.76094
+	TADA2B	4	7008429	7067952	149	17	10164	-0.70932	0.76094
+	CNOT6	5	180459373	180588405	462	22	10164	-0.70981	0.76109
+	BEAN1	16	66392297	66503529	232	32	10164	-0.71004	0.76116
+	HAX1	1	154237563	154285875	83	13	10164	-0.71036	0.76126
+	NAV1	1	201504102	201836974	988	118	10164	-0.71036	0.76126
-	TOP2A	17	40378521	40452950	55	7	10164	-0.71051	0.76131
-	TREML1	6	41139260	41189349	112	10	10164	-0.71051	0.76131
+	OR4A5	11	54671832	54717902	160	15	10164	-0.71088	0.76142
+	KCNMB4	12	70331223	70444292	427	41	10164	-0.71097	0.76145
-	SPDYE17	7	77013688	77066631	12	3	10164	-0.71133	0.76156
-	DNAJC14	12	55810960	55864636	53	16	10164	-0.71152	0.76162
-	HDDC3	15	90920918	90967569	128	15	10164	-0.71159	0.76164
+	CLCA1	1	86433843	86510294	247	35	10164	-0.71175	0.76169
+	CHP1	15	41196149	41291887	353	19	10164	-0.71192	0.76174
+	ZNF454	5	178906193	178976218	217	23	10164	-0.7119	0.76174
+	TACC1	8	38693186	38863028	308	36	10164	-0.712	0.76177
-	FAF1	1	50431263	50995264	731	25	10164	-0.71215	0.76181
-	EGR4	2	73280929	73328701	3	1	10164	-0.71258	0.76195
-	SPDYE16	7	76523538	76576484	22	5	10164	-0.71294	0.76206
-	ACTR1A	10	102469229	102537755	225	16	10164	-0.71298	0.76207
-	SLC30A2	1	26028022	26081138	122	12	10164	-0.71321	0.76214
+	EPHX1	1	225775074	225855563	397	49	10164	-0.71327	0.76216
-	SLC16A6	17	68257026	68326264	140	22	10164	-0.71354	0.76225
+	BLM	15	90682327	90825462	453	34	10164	-0.71377	0.76231
-	ICOSLG	21	44206971	44276447	59	9	10164	-0.71374	0.76231
+	SLC35C1	11	45769072	45823016	146	22	10164	-0.71376	0.76231
+	MT1E	16	56590673	56637112	207	40	10164	-0.71378	0.76232

STRAND	SYMBOL	CHR	START	STOP	NSNPS	NPARAM	N	ZSTAT	P
+	EMX2	10	117507445	117559546	165	16	10164	-0.71385	0.76234
-	UBE2Q1	1	154538575	154593644	104	17	10164	-0.71396	0.76237
-	DDX24	14	94040922	94116212	286	27	10164	-0.71403	0.76239
-	PGBD3	10	49505105	49559281	148	14	10164	-0.71411	0.76242
+	CPSF4	7	99403940	99467373	138	18	10164	-0.71434	0.76249
+	FAM47E	4	76179040	76293783	527	36	10164	-0.71508	0.76272
-	MRPS5	2	95077207	95157009	72	19	10164	-0.71513	0.76274
+	PI4K2B	4	25199031	25289209	269	29	10164	-0.71523	0.76277
-	KIAA1147	7	141646728	141737169	168	20	10164	-0.71533	0.7628
-	THADA	2	43220836	43631056	1171	35	10164	-0.71539	0.76282
-	B4GALT4	3	119201742	119275905	315	30	10164	-0.71554	0.76286
-	SLC25A52	18	31749696	31795880	96	15	10164	-0.71617	0.76306
-	C1orf189	1	154189085	154241365	70	13	10164	-0.71624	0.76308
-	OXCT2	1	39759525	39806348	168	13	10164	-0.71637	0.76312
+	PTGER2	14	52279298	52338606	174	27	10164	-0.71647	0.76315
-	TRAPPC3L	6	116486483	116580610	310	21	10164	-0.71651	0.76316
-	TAS2R19	12	11011619	11057620	137	7	10164	-0.71653	0.76317
+	CCNB2	15	59070085	59135045	170	10	10164	-0.71663	0.7632
-	LOC1053766	11	30353241	30409007	141	21	10164	-0.71678	0.76325
+	MRPL1	4	77826955	77962790	220	11	10164	-0.71703	0.76332
+	OR52K2	11	4414295	4460361	172	29	10164	-0.71713	0.76335
+	SUFU	10	102467874	102643457	509	26	10164	-0.71717	0.76337
-	HMGB1	13	30448740	30500944	35	6	10164	-0.71752	0.76347
+	SIM2	21	36664133	36760219	90	19	10164	-0.7175	0.76347
+	UBE4A	11	118324581	118409211	163	17	10164	-0.71761	0.7635
+	RIC1	9	5594030	5788015	540	37	10164	-0.7178	0.76356
+	IQGAP2	5	76368255	76718132	1118	100	10164	-0.71788	0.76358
-	SIRT3	11	205030	271362	279	21	10164	-0.71809	0.76365
-	WDR12	2	202870600	202947226	163	12	10164	-0.71815	0.76367
-	OR5A2	11	59411979	59457953	117	9	10164	-0.71814	0.76367
+	IDUA	4	951997	1014557	218	39	10164	-0.71828	0.76371
-	KIAA0020	9	2794152	2879130	216	28	10164	-0.71836	0.76373
+	HCST	19	35867480	35914271	94	8	10164	-0.71847	0.76377
-	PGK2	6	49775651	49822340	115	21	10164	-0.71879	0.76386
-	PTCD1	7	99406739	99473839	147	19	10164	-0.71878	0.76386
-	ANKRD16	10	5851726	5924897	354	22	10164	-0.71885	0.76388
-	N4BP1	16	48528726	48645214	196	6	10164	-0.71908	0.76395
+	TNK1	17	7345559	7399774	132	30	10164	-0.7191	0.76396
-	C14orf93	14	22976901	23045201	111	19	10164	-0.71942	0.76406
+	NDRG4	16	58428645	58523619	235	48	10164	-0.71942	0.76406
-	HYI	1	43440607	43490882	78	10	10164	-0.7195	0.76408
-	H1FX	3	129304771	129351277	149	18	10164	-0.71965	0.76413
-	SLC5A6	2	27189587	27247307	138	14	10164	-0.7197	0.76415
+	C14orf132	14	96004324	96103971	301	25	10164	-0.71977	0.76417
-	SUGP1	19	19266511	19355512	224	21	10164	-0.72027	0.76432
+	KCNE2	21	34329024	34381141	120	19	10164	-0.72041	0.76436
+	FLT3LG	19	49439172	49496234	154	15	10164	-0.72051	0.7644
-	LOC1053713	16	87690832	87738017	234	23	10164	-0.72063	0.76443
-	STAT4	2	191019576	191281172	491	53	10164	-0.7207	0.76445
+	TRIM72	16	31179021	31235189	25	5	10164	-0.72093	0.76452
-	CLUH	17	2679386	2746633	89	17	10164	-0.72105	0.76456
+	CCL18	17	36029272	36081481	148	23	10164	-0.7212	0.76461
+	BACH1	21	29263795	29371896	177	24	10164	-0.7214	0.76467
+	MAP7D1	1	36120965	36190849	167	16	10164	-0.72144	0.76468
-	THY1	11	119407945	119458536	79	3	10164	-0.72152	0.76471
+	GABRA6	5	161650652	161712592	189	21	10164	-0.72161	0.76473
+	MMAA	4	145584388	145670035	11	5	10164	-0.72198	0.76485
-	ZNF850	19	36733478	36807814	263	28	10164	-0.72222	0.76492
-	CCZ1B	7	6788935	6861295	107	7	10164	-0.7223	0.76495
+	GOLGA6L3	15	85205410	85257187	27	9	10164	-0.72256	0.76503
+	LTA	6	31525550	31584324	388	49	10164	-0.72297	0.76515
+	ZNF131	5	43085888	43186324	318	15	10164	-0.72297	0.76515
-	SELPLG	12	108611895	108668954	111	22	10164	-0.7231	0.76519

STRAND	SYMBOL	CHR	START	STOP	NSNPS	NPARAM	N	ZSTAT	P
-	TRIM68	11	4588672	4643259	117	13	10164	-0.72327	0.76524
-	MRGPRX2	11	19044455	19106978	234	24	10164	-0.72349	0.76531
+	NOL7	6	13580327	13642524	93	15	10164	-0.72367	0.76536
+	LOC1053761	9	83672835	83723496	163	16	10164	-0.72368	0.76537
+	ATG16L2	11	72779406	72852705	111	12	10164	-0.72447	0.76561
+	DDX10	11	108630025	108950930	607	21	10164	-0.72464	0.76566
-	MMP28	17	35746249	35830707	185	15	10164	-0.72473	0.76569
-	DNAJA2	16	46945362	47008714	13	4	10164	-0.72482	0.76572
+	BUB1B	15	40126009	40231136	208	13	10164	-0.72503	0.76578
+	S100A1	1	153593397	153642037	91	9	10164	-0.72516	0.76582
+	RABGEF1	7	66647091	66821464	710	26	10164	-0.72519	0.76583
-	CREG1	1	167531013	167588819	253	29	10164	-0.72542	0.7659
-	WBP2	17	75835699	75890420	114	8	10164	-0.72542	0.7659
+	MORF4L1	15	78837781	78907739	297	15	10164	-0.72564	0.76597
-	IL11	19	55354382	55405463	172	25	10164	-0.72572	0.766
+	AGO1	1	35834791	35934298	107	14	10164	-0.72589	0.76605
+	RER1	1	2356314	2415444	170	35	10164	-0.72596	0.76607
-	SLAMF1	1	160598111	160682537	174	26	10164	-0.72627	0.76616
+	RFPL4B	6	112312330	112361296	127	28	10164	-0.72637	0.76619
-	LAMTOR5	1	110391253	110442924	136	20	10164	-0.72638	0.7662
+	PLB1	2	28453719	28653788	612	59	10164	-0.72651	0.76624
-	CDON	11	125946818	126098292	470	50	10164	-0.72721	0.76645
-	TIMM9	14	58398494	58462614	71	19	10164	-0.72762	0.76658
+	AKAP13	15	85345616	85759358	1434	51	10164	-0.72767	0.76659
+	FOCAD	9	20623309	21005955	1155	51	10164	-0.72766	0.76659
-	GEMIN5	5	154877416	154973216	276	25	10164	-0.72777	0.76662
-	EAPP	14	34505929	34574737	149	14	10164	-0.72778	0.76663
+	MNAT1	14	60699741	60978680	510	20	10164	-0.72781	0.76664
+	RGS13	1	192601138	192670311	344	26	10164	-0.72782	0.76664
-	CEP89	19	32868998	33007029	590	37	10164	-0.72784	0.76664
+	SZT2	1	43349830	43464247	199	16	10164	-0.72793	0.76667
-	CEP112	17	65625540	66227131	2289	48	10164	-0.72797	0.76668
+	ITPRIPL1	2	96290324	96338353	74	17	10164	-0.72812	0.76673
-	PPP1R1A	12	54569240	54623677	138	21	10164	-0.7282	0.76675
-	FGF12	3	192129244	192762599	1351	82	10164	-0.7282	0.76676
-	TNPO2	19	12689194	12758996	17	6	10164	-0.72822	0.76676
-	GABBR2	9	98278082	98744197	1570	118	10164	-0.72827	0.76678
+	POLE4	2	74923648	74979732	102	12	10164	-0.72852	0.76685
-	APOA4	11	116810702	116858295	171	23	10164	-0.72875	0.76692
-	PGLYRP4	1	153317380	153383841	73	9	10164	-0.7288	0.76694
-	MRPL46	15	88449478	88502402	185	15	10164	-0.72886	0.76696
+	CDC45	22	19444385	19530612	250	29	10164	-0.72892	0.76697
-	MRPL14	6	44103635	44162454	13	4	10164	-0.72891	0.76697
-	COMP	19	18772773	18826305	107	19	10164	-0.72905	0.76701
+	FGF18	5	171384663	171467626	195	21	10164	-0.72903	0.76701
+	NME1-NME2	17	51118536	51181744	195	24	10164	-0.72911	0.76703
-	PPP1R3B	8	9126254	9186642	177	27	10164	-0.72929	0.76709
-	PDGFD	11	103897186	104199299	1091	75	10164	-0.72939	0.76712
-	SERPINC1	1	173893800	173952378	127	14	10164	-0.72941	0.76713
+	HIST1H2BA	6	25691909	25737345	219	22	10164	-0.7295	0.76715
-	CRYL1	13	20393667	20560873	591	50	10164	-0.72962	0.76719
+	EFNA1	1	155092873	155144910	98	11	10164	-0.72983	0.76725
-	DSEL	18	67496582	67551730	124	22	10164	-0.72989	0.76727
+	KLB	4	39371853	39461533	254	35	10164	-0.7299	0.76728
+	RAB11A	15	65834459	65901991	96	12	10164	-0.73	0.76731
+	ZMYM4	1	35233714	35431944	337	18	10164	-0.7302	0.76737
+	PI16	6	36913263	36974837	320	40	10164	-0.73021	0.76737
+	SHROOM3	4	76400100	76793253	1065	71	10164	-0.73024	0.76738
+	CALD1	7	134744413	134980729	635	63	10164	-0.73048	0.76745
-	NDUFC2-KC⁻	11	78005715	78115219	410	19	10164	-0.73052	0.76746
+	RAB3IP	12	69703400	69833204	294	24	10164	-0.73053	0.76747
-	CDX2	13	27950057	28004368	146	22	10164	-0.73135	0.76772
+	CHRM2	7	136833652	137030255	454	35	10164	-0.73146	0.76775

STRAND	SYMBOL	CHR	START	STOP	NSNPS	NPARAM	N	ZSTAT	P
-	LACRT	12	54620839	54669879	131	27	10164	-0.73168	0.76782
+	CES4A	16	66953589	67019758	58	16	10164	-0.73178	0.76785
+	ZNF2	2	95130417	95194317	74	18	10164	-0.73192	0.76789
-	ETV4	17	43517843	43581432	139	12	10164	-0.73197	0.76791
+	FOXN2	2	48279644	48389295	282	21	10164	-0.732	0.76792
-	PRMT9	4	147627785	147719230	125	8	10164	-0.732	0.76792
+	NEXN	1	77853515	77958643	153	20	10164	-0.73206	0.76793
+	PLEKHF1	19	29630420	29685477	164	34	10164	-0.73213	0.76795
-	LCAT	16	67929884	67979112	64	7	10164	-0.73215	0.76796
-	NBPF7	1	119823730	119879880	85	9	10164	-0.73223	0.76798
+	ANGPTL3	1	62562487	62616305	75	9	10164	-0.73243	0.76805
-	PDE1A	2	182130035	182557845	1030	46	10164	-0.7327	0.76813
+	AGFG2	7	100504211	100578220	117	18	10164	-0.73287	0.76818
-	HOXB9	17	48605128	48661473	218	37	10164	-0.73345	0.76836
+	LTBP1	2	32912300	33409509	2322	118	10164	-0.73373	0.76844
-	DKK4	8	42363194	42426754	122	12	10164	-0.73382	0.76847
+	HELB	12	66267555	66353643	294	21	10164	-0.73405	0.76854
+	UTP11L	1	37977712	38034825	29	7	10164	-0.73408	0.76855
-	LOC1019290	19	2501218	2548571	163	19	10164	-0.7342	0.76859
-	SNRNP200	2	96264336	96340569	134	27	10164	-0.7344	0.76865
-	SLC25A32	8	103388638	103450335	178	11	10164	-0.73449	0.76868
+	CATSPERD	19	5685401	5788734	272	40	10164	-0.73455	0.76869
-	AKIRIN2	6	87664860	87737267	201	22	10164	-0.73463	0.76872
+	TJAP1	6	43442523	43516556	120	17	10164	-0.73485	0.76878
+	RBM44	2	237763050	237852808	290	28	10164	-0.73524	0.7689
+	FAM98C	19	38368125	38419088	193	38	10164	-0.73527	0.76891
-	LOC1005075	7	143369155	143415795	102	26	10164	-0.73532	0.76893
-	NUCKS1	1	205702819	205785244	200	22	10164	-0.73557	0.769
-	LOC1027244	10	47736653	47798592	3	1	10164	-0.73555	0.769
-	FADS1	11	61789625	61852057	94	9	10164	-0.7356	0.76901
-	EPB42	15	43187227	43260861	141	16	10164	-0.73626	0.76921
-	ULBP3	6	150053264	150104213	261	31	10164	-0.73626	0.76921
-	SRL	16	4179374	4277080	185	29	10164	-0.73661	0.76932
-	TNFRSF21	6	47221527	47344976	169	27	10164	-0.73698	0.76943
+	FAM73A	1	77744622	77889540	209	32	10164	-0.73705	0.76945
+	DNMT3B	20	32727385	32819356	270	26	10164	-0.73716	0.76949
+	UCHL3	13	75513867	75616020	231	14	10164	-0.73728	0.76952
+	TIMM21	18	74113511	74168969	196	9	10164	-0.73735	0.76954
-	LRRC19	9	26983136	27040693	124	18	10164	-0.73734	0.76954
-	LOC1019290	7	153134205	153179588	335	18	10164	-0.73737	0.76955
-	SALL2	14	21511080	21572216	178	23	10164	-0.73745	0.76958
+	FADS2	11	61781203	61877354	199	21	10164	-0.73801	0.76975
-	POMGNT2	3	43069229	43141083	149	17	10164	-0.73814	0.76978
+	EPN1	19	55640195	55705767	145	16	10164	-0.73837	0.76985
+	FXN	9	69000563	69110178	402	29	10164	-0.73849	0.76989
+	EIF4G1	3	184279495	184345358	103	20	10164	-0.73853	0.7699
-	PLET1	11	112238153	112295860	158	7	10164	-0.73883	0.76999
+	UTF1	10	133195274	133241558	127	13	10164	-0.73885	0.77
-	SCYL3	1	169843074	169929267	335	23	10164	-0.73886	0.77
-	DDX59	1	200630807	200704998	250	21	10164	-0.7389	0.77002
-	A1CF	10	50789409	50920675	216	37	10164	-0.73922	0.77011
-	CAPS2	12	75247036	75425928	463	32	10164	-0.73928	0.77013
+	NRSN2	20	311726	364868	79	5	10164	-0.73931	0.77014
-	NOTUM	17	81942507	81996823	13	2	10164	-0.73932	0.77014
-	LOC1001445	2	154425853	154492438	217	37	10164	-0.73956	0.77022
+	UQCC3	11	62636654	62683690	49	9	10164	-0.73964	0.77024
-	LOC1053748	2	77977876	78034030	255	21	10164	-0.73969	0.77026
-	SCN3B	11	123619187	123689607	318	28	10164	-0.73977	0.77028
+	ATP5F1	1	111414121	111471918	133	15	10164	-0.7401	0.77038
-	N6AMT1	21	28862191	28920373	337	17	10164	-0.74048	0.7705
-	GABBR1	6	29592228	29668185	475	50	10164	-0.74052	0.77051
+	ACE	17	63442061	63508380	131	20	10164	-0.74086	0.77061
+	ANGPTL4	19	8329127	8384375	56	11	10164	-0.7413	0.77074

STRAND	SYMBOL	CHR	START	STOP	NSNPS	NPARAM	N	ZSTAT	P
-	CPSF3L	1	1301585	1359687	103	7	10164	-0.7414	0.77077
-	ZNF124	1	247111975	247207438	321	26	10164	-0.74191	0.77093
+	SLC26A4	7	107625635	107727809	110	14	10164	-0.74214	0.771
+	ZNF485	10	43571407	43627904	129	22	10164	-0.74221	0.77102
-	SLC35F5	2	113702792	113791823	306	20	10164	-0.74239	0.77107
+	SCIN	7	12535349	12663603	523	55	10164	-0.74238	0.77107
-	DHH	12	49079421	49129819	90	15	10164	-0.74259	0.77113
+	NGRN	15	90230663	90282211	160	14	10164	-0.74302	0.77126
+	BRDT	1	91914371	92024428	287	14	10164	-0.74306	0.77128
+	LOC1053733	2	4061389	4150262	126	8	10164	-0.74312	0.77129
-	CA8	8	60177347	60316395	580	39	10164	-0.74371	0.77148
+	ZIC2	13	99946772	99996765	51	16	10164	-0.74374	0.77148
-	MTERF2	12	106967291	107022166	148	20	10164	-0.74384	0.77151
-	BACH2	6	89916528	90331908	1061	106	10164	-0.74403	0.77157
-	ABRA	8	106749483	106805244	176	29	10164	-0.74459	0.77174
+	SLC18A2	10	117206073	117289430	199	29	10164	-0.74465	0.77176
+	EMC4	15	34189997	34240165	143	9	10164	-0.74477	0.7718
+	EDARADD	1	236359380	236494708	392	25	10164	-0.74494	0.77185
-	OR10S1	11	123966696	124012691	215	28	10164	-0.74518	0.77192
+	RNF169	11	74713855	74852413	214	15	10164	-0.7453	0.77195
+	TMEM19	12	71651098	71714060	104	14	10164	-0.74556	0.77203
-	ADAT2	6	143412832	143485750	207	18	10164	-0.7456	0.77204
-	FAM166B	9	35551830	35598899	104	24	10164	-0.74662	0.77235
+	RAD21L1	20	1191043	1271279	95	7	10164	-0.74666	0.77237
-	SPTBN2	11	66672496	66764237	123	36	10164	-0.74673	0.77239
+	CUTC	10	99697201	99766137	99	12	10164	-0.74674	0.77239
-	OR2V1	5	181114357	181160304	126	19	10164	-0.74691	0.77244
+	SMAP1	6	70632771	70872015	409	24	10164	-0.74697	0.77246
+	RNF7	3	141703209	141756803	179	22	10164	-0.74714	0.77251
+	TXNRD1	12	104180779	104360307	557	29	10164	-0.74722	0.77253
-	CAMK2A	5	150209491	150324840	270	57	10164	-0.74734	0.77257
+	FAM163A	1	179692763	179826198	405	30	10164	-0.74735	0.77257
+	CTNS	17	3601468	3673103	235	26	10164	-0.74742	0.77259
-	PRKCQ	10	6383595	6615646	837	100	10164	-0.74772	0.77268
-	ZFR	5	32344350	32479761	337	32	10164	-0.74771	0.77268
+	EMG1	12	6935781	6986003	38	3	10164	-0.74777	0.7727
+	MIOX	22	50448988	50500321	155	27	10164	-0.74811	0.7728
+	MILR1	17	64412249	64489383	37	3	10164	-0.74822	0.77284
-	GJB2	13	20177463	20227975	81	8	10164	-0.74866	0.77297
-	FAM126B	2	200963718	201106671	264	45	10164	-0.7487	0.77298
+	KPNA2	17	68000732	68056854	132	8	10164	-0.74872	0.77299
-	MFSD12	19	3528273	3592588	33	4	10164	-0.74895	0.77306
-	YIPF3	6	43501827	43551990	73	9	10164	-0.74943	0.7732
+	CDK15	2	201755459	201905550	331	34	10164	-0.74956	0.77324
-	IFNG	12	68144770	68194741	180	17	10164	-0.74971	0.77329
+	JMJD7	15	41793085	41847587	134	15	10164	-0.74995	0.77336
+	FAM118B	11	126176450	126272984	294	29	10164	-0.74998	0.77337
-	ST3GAL1	8	133444848	133607454	535	77	10164	-0.75013	0.77341
-	OR5P2	11	7785974	7831942	177	16	10164	-0.75012	0.77341
+	STK11IP	2	219562871	219626451	58	12	10164	-0.7502	0.77343
+	RPL34	4	108585566	108640484	105	5	10164	-0.75032	0.77347
-	MICAL3	22	17777649	18059559	903	63	10164	-0.75033	0.77347
-	C9orf41	9	74971020	75063486	167	14	10164	-0.75046	0.77351
+	PPM1B	2	44133803	44254425	305	26	10164	-0.75067	0.77357
+	VDAC3	8	42356624	42415937	110	12	10164	-0.75072	0.77359
-	TRAPPC3	1	36126569	36191053	155	16	10164	-0.75071	0.77359
+	EFCAB3	17	62335218	62426479	206	28	10164	-0.75091	0.77365
+	ADRA2A	10	111042032	111090907	113	19	10164	-0.75106	0.77369
+	RPL28	19	55350101	55413245	200	28	10164	-0.75121	0.77374
+	CSRNP3	2	165434647	165699407	636	51	10164	-0.75127	0.77375
-	SYNJ1	21	32618759	32763040	223	25	10164	-0.75135	0.77378
+	CNNM2	10	102883293	103088587	657	30	10164	-0.75162	0.77386
-	CDAN1	15	42713557	42772219	165	20	10164	-0.7518	0.77391

STRAND	SYMBOL	CHR	START	STOP	NSNPS	NPARAM	N	ZSTAT	P
+	ABCC3	17	50599843	50701704	70	20	10164	-0.75188	0.77394
+	LOC1002870	16	89286133	89335110	156	35	10164	-0.75212	0.77401
-	COG7	16	23378493	23488210	160	12	10164	-0.75224	0.77405
+	RAB4A	1	229236062	229315894	142	12	10164	-0.75229	0.77406
+	C19orf38	19	10801653	10879791	141	17	10164	-0.75233	0.77408
+	OSTC	4	108615585	108677822	62	8	10164	-0.75242	0.7741
-	OR1D2	17	3082058	3127996	95	5	10164	-0.7527	0.77418
-	FBXL20	17	39242644	39437661	416	14	10164	-0.75269	0.77418
+	FAM198A	3	42944267	43067715	314	21	10164	-0.75282	0.77422
-	TEKT3	17	15293811	15376889	266	26	10164	-0.75287	0.77424
+	NTNG1	1	107104918	107494899	726	81	10164	-0.7529	0.77425
+	COG3	13	45429895	45546698	390	24	10164	-0.75291	0.77425
-	BID	22	17724140	17809665	282	60	10164	-0.75307	0.7743
-	CWC22	2	179934876	180042297	274	28	10164	-0.75308	0.7743
-	CCDC64B	16	3017710	3071963	31	5	10164	-0.75322	0.77434
-	SCAF4	21	31661000	31767118	143	14	10164	-0.75326	0.77435
-	SHH	7	155792864	155847273	142	17	10164	-0.75334	0.77438
+	RPL13A	19	49452554	49502308	132	15	10164	-0.75362	0.77446
+	IFIT3	10	89292819	89350968	202	20	10164	-0.75425	0.77465
-	AMIGO2	12	47065707	47115826	141	36	10164	-0.75456	0.77474
+	COX7C	5	86582967	86630766	108	6	10164	-0.75464	0.77477
+	UNC93A	6	167236174	167326029	484	39	10164	-0.75494	0.77486
-	C14orf142	14	93192892	93242114	146	22	10164	-0.75518	0.77493
+	SULT1A3	16	30164228	30214329	9	2	10164	-0.75531	0.77497
+	MISP	19	713421	774318	33	7	10164	-0.75537	0.77499
+	PLEKHA1	10	122339578	122442355	299	24	10164	-0.75547	0.77502
+	OR11H6	14	20188710	20234702	57	11	10164	-0.75551	0.77503
-	SKIDA1	10	21500754	21562188	76	20	10164	-0.75586	0.77513
-	LOXL3	2	74522258	74590887	20	3	10164	-0.75596	0.77516
-	C22orf42	22	32139006	32195432	224	19	10164	-0.75593	0.77516
+	MAP3K10	19	40156650	40225580	190	20	10164	-0.75646	0.77531
-	FPR1	19	51735770	51786897	200	39	10164	-0.75697	0.77546
-	NFYB	12	104107080	104173262	197	26	10164	-0.75708	0.7755
-	KRT31	17	41383724	41432592	127	15	10164	-0.7573	0.77557
+	LOC1019290	7	45734085	45815139	235	13	10164	-0.75764	0.77567
+	AOC2	17	42809592	42860707	63	13	10164	-0.75767	0.77568
+	STXBP5	6	147169358	147400476	202	15	10164	-0.75774	0.7757
-	RPA3	7	7626944	7753607	563	33	10164	-0.75785	0.77573
-	PLA2G12B	10	72925180	72989883	134	22	10164	-0.75795	0.77576
+	SLC16A13	17	7001075	7050121	137	16	10164	-0.75808	0.7758
+	DIEXF	1	209792967	209867565	259	21	10164	-0.75813	0.77581
-	KRT6C	12	52458516	52508785	294	11	10164	-0.75829	0.77586
+	SPATA31D1	9	81952667	82005256	38	9	10164	-0.75828	0.77586
-	SYN3	22	32498022	33093391	2063	144	10164	-0.75839	0.77589
-	CARS2	13	110631410	110748601	468	24	10164	-0.75843	0.7759
+	SLC6A7	5	150154957	150221072	223	37	10164	-0.7585	0.77592
-	IKZF3	17	39747715	39899188	135	12	10164	-0.75865	0.77597
-	CHRNA3	15	78583052	78656295	261	35	10164	-0.75883	0.77602
-	ALS2CL	3	46658995	46728704	235	33	10164	-0.75888	0.77604
+	C11orf97	11	94477529	94542123	250	26	10164	-0.75895	0.77606
+	TRB	7	142264011	142823287	896	33	10164	-0.75982	0.77632
+	ARGFX	3	121527303	121600622	191	25	10164	-0.75994	0.77635
+	NTRK1	1	156780750	156891850	207	28	10164	-0.76003	0.77638
-	MATK	19	3767969	3836812	104	15	10164	-0.76031	0.77647
+	IDO2	8	39899955	40026391	570	47	10164	-0.76104	0.77668
+	NSA2	5	74730429	74786912	93	9	10164	-0.76107	0.77669
+	PSMB11	14	23007167	23054060	94	21	10164	-0.76137	0.77678
-	ZSCAN32	16	3372081	3436065	214	22	10164	-0.76173	0.77689
-	S100A2	1	153551108	153600830	46	7	10164	-0.76177	0.7769
-	HCAR3	12	122704756	122751892	30	4	10164	-0.7618	0.77691
+	KIAA1551	12	31924419	32003107	331	48	10164	-0.76188	0.77693
-	CBLN2	18	72525898	72673477	285	23	10164	-0.76197	0.77696
-	RAB11FIP1	8	37848947	37934497	69	18	10164	-0.76198	0.77697

STRAND	SYMBOL	CHR	START	STOP	NSNPS	NPARAM	N	ZSTAT	P
+	SLC6A2	16	55620604	55716192	312	34	10164	-0.76222	0.77703
+	LDLR	19	11054362	11143830	350	36	10164	-0.76229	0.77706
+	SNX25	4	185169242	185373969	621	26	10164	-0.76236	0.77708
-	CLRN1	3	150908911	151008020	108	14	10164	-0.76242	0.7771
+	GTPBP10	7	90311665	90401455	274	26	10164	-0.76257	0.77714
-	MESDC2	15	80937326	81024878	192	12	10164	-0.76267	0.77717
+	ALPP	2	232343534	232392889	142	25	10164	-0.76297	0.77726
-	HUS1B	6	645939	691964	150	15	10164	-0.76301	0.77727
-	IGFBP5	2	216662105	216730549	231	31	10164	-0.76313	0.77731
-	MRPL11	11	66425075	66473848	75	18	10164	-0.76314	0.77731
+	GINS4	8	41494206	41555046	161	20	10164	-0.76318	0.77732
+	SRRD	22	26448884	26501938	136	15	10164	-0.76326	0.77735
+	CCR6	6	167076807	167149141	280	34	10164	-0.76336	0.77737
-	ADH6	4	99192638	99254246	111	11	10164	-0.76352	0.77742
-	ZFHX2	14	23510855	23590939	86	21	10164	-0.76376	0.77749
+	SEPT7	7	35765986	35925763	259	14	10164	-0.7638	0.77751
+	STAC	3	36345424	36558011	738	41	10164	-0.76401	0.77757
+	F2RL3	19	16854015	16902019	54	9	10164	-0.76401	0.77757
+	CDYL	6	4671159	4965544	635	54	10164	-0.76446	0.7777
+	EIF5AL1	10	79477601	79526440	103	7	10164	-0.76457	0.77774
+	STAT5A	17	42252547	42321943	150	16	10164	-0.76466	0.77776
+	PRCD	17	76492586	76563580	86	9	10164	-0.76468	0.77777
+	DCAF7	17	63515435	63604279	86	13	10164	-0.76474	0.77779
-	RNASE11	14	20572892	20625258	191	34	10164	-0.76488	0.77783
-	GDPD3	16	30094810	30148943	47	7	10164	-0.76508	0.77789
+	ARHGAP26	5	142735380	143239007	1546	116	10164	-0.76513	0.7779
-	SCAMP3	1	155245979	155297434	92	8	10164	-0.7655	0.77801
+	RHOD	11	67021818	67082017	125	27	10164	-0.76568	0.77807
+	TESK1	9	35570284	35620041	107	23	10164	-0.76582	0.77811
+	NUTF2	16	67811916	67882567	73	11	10164	-0.76587	0.77812
-	AMER2	13	25151679	25207324	91	18	10164	-0.766	0.77816
-	RPL3L	16	1934579	1989678	191	32	10164	-0.76606	0.77818
+	MAU2	19	19285817	19368754	223	20	10164	-0.76609	0.77819
+	CEP295	11	93626650	93740358	303	14	10164	-0.76631	0.77825
-	FBLN5	14	91859411	91982702	394	49	10164	-0.76637	0.77827
-	ACAP3	1	1282384	1342925	115	11	10164	-0.76636	0.77827
-	PFDN1	5	140235050	140338104	184	14	10164	-0.76661	0.77834
+	LRRC52	1	165509241	165573948	217	19	10164	-0.76671	0.77837
+	SIRT4	12	120257199	120323242	178	25	10164	-0.76676	0.77839
+	C1orf177	1	54771063	54852264	409	40	10164	-0.76678	0.77839
+	RHO	3	129493639	129545344	140	21	10164	-0.76691	0.77843
-	NPHP4	1	5852808	6027505	441	20	10164	-0.76689	0.77843
+	SLC12A1	15	48171301	48314078	196	24	10164	-0.76742	0.77858
+	SRP54	14	34947695	35039567	351	14	10164	-0.76787	0.77872
+	RGS9BP	19	32640407	32688300	255	25	10164	-0.76791	0.77873
-	EIF4G2	11	10787046	10844035	79	5	10164	-0.76808	0.77878
-	GFI1	1	92463043	92521876	103	12	10164	-0.76838	0.77887
+	CLCNKB	1	16008736	16067326	351	26	10164	-0.7684	0.77888
-	LIG1	19	48105444	48205603	401	24	10164	-0.76853	0.77891
-	LOC1053787	1	65038336	65104390	172	18	10164	-0.76854	0.77892
-	TGDS	13	94564054	94631275	146	10	10164	-0.76863	0.77894
+	ACOT4	14	73556706	73605766	198	28	10164	-0.76877	0.77898
-	FAM169B	15	98427162	98549382	392	34	10164	-0.76891	0.77903
+	RIMKLB	12	8646611	8793098	307	10	10164	-0.76925	0.77913
+	TRABD	22	50150905	50209598	239	36	10164	-0.7693	0.77914
+	PAPL	19	39049305	39121489	325	28	10164	-0.76948	0.7792
-	HYAL2	3	50307790	50357850	71	10	10164	-0.76965	0.77925
+	PGA3	11	61168495	61222878	35	5	10164	-0.76966	0.77925
+	PTCRA	6	42880807	42935838	139	13	10164	-0.76973	0.77927
+	MMP25	16	3010963	3070729	30	5	10164	-0.77	0.77935
+	PRKAA2	1	56610317	56725335	180	22	10164	-0.77014	0.77939
-	ZIC5	13	99953021	100006924	73	15	10164	-0.77014	0.77939
-	JCHAIN	4	70645541	70711901	63	15	10164	-0.77068	0.77955

STRAND	SYMBOL	CHR	START	STOP	NSNPS	NPARAM	N	ZSTAT	P
-	OR51F1	11	4758979	4804917	151	9	10164	-0.77073	0.77957
+	ACACB	12	109076184	109278226	529	53	10164	-0.77078	0.77958
+	RBBP8	18	22879121	23036488	444	26	10164	-0.77096	0.77964
-	PRSS12	4	118270038	118387815	292	34	10164	-0.77114	0.77969
-	C1QL2	2	119146243	119193895	113	12	10164	-0.77115	0.77969
-	TMEM229A	7	124020916	124068469	126	12	10164	-0.77115	0.77969
-	LRPPRC	2	43876224	44031005	507	25	10164	-0.77133	0.77974
-	RPS16	19	39423207	39471020	129	8	10164	-0.7715	0.77979
-	TNC	9	115009575	115153257	579	56	10164	-0.77157	0.77981
+	LOC642515	9	131514603	131569199	220	32	10164	-0.77154	0.77981
-	SENP7	3	101314189	101548883	676	14	10164	-0.77172	0.77986
-	WDR1	4	10064339	10151949	535	25	10164	-0.77194	0.77992
+	PPARD	6	35307558	35438191	622	46	10164	-0.77195	0.77993
-	LOC1053712	1	146333119	146379870	1	1	10164	-0.77211	0.77997
-	STAP2	19	4314043	4373877	98	13	10164	-0.77235	0.78005
-	NPM3	10	101771325	101818413	57	18	10164	-0.77251	0.78009
-	SPAG17	1	117943665	118220225	638	21	10164	-0.77258	0.78011
+	FFAR4	10	93531204	93600072	159	20	10164	-0.77273	0.78016
-	CORO1C	12	108635109	108766550	220	18	10164	-0.77291	0.78021
-	C12orf42	12	103069645	103560254	1480	95	10164	-0.77314	0.78028
+	PIK3R2	19	18118178	18180533	196	21	10164	-0.77319	0.7803
+	CCDC15	11	124919121	125051489	386	20	10164	-0.77323	0.78031
-	HTR1A	5	63950048	63997292	103	16	10164	-0.77344	0.78037
+	ARSK	5	95520111	95615102	155	16	10164	-0.77346	0.78038
-	CAPRIN2	12	30699552	30858235	470	34	10164	-0.77364	0.78043
-	SLC4A11	20	3217417	3276482	201	23	10164	-0.77375	0.78046
+	MANBAL	20	37254648	37327260	71	10	10164	-0.77378	0.78047
+	LHFPL3	7	104293656	104918558	2203	107	10164	-0.77423	0.7806
+	RNPEPL1	2	240533541	240588732	134	27	10164	-0.77433	0.78063
+	BTN3A3	6	26405472	26463415	261	20	10164	-0.77436	0.78064
-	LDLRAD1	1	53997298	54053186	145	15	10164	-0.77459	0.78071
+	RRM2	2	10087568	10141419	91	20	10164	-0.77491	0.7808
-	LOC1027244	19	20387440	20433579	85	13	10164	-0.77512	0.78087
+	PDE1B	12	54514393	54589239	154	35	10164	-0.77531	0.78092
+	LOC1053737	2	177667404	177738138	232	26	10164	-0.77543	0.78096
+	KMT2B	19	35682995	35748880	201	27	10164	-0.77574	0.78105
+	TMEM184C	4	147582371	147645523	157	15	10164	-0.77677	0.78135
-	SLC35B2	6	44244101	44292890	135	17	10164	-0.77686	0.78138
-	MED22	9	133330901	133383156	107	10	10164	-0.7773	0.78151
+	APOC3	11	116794908	116843071	171	23	10164	-0.77738	0.78153
+	IL17D	13	20667875	20733100	191	17	10164	-0.77737	0.78153
+	C3orf58	3	143936798	144002368	247	8	10164	-0.77827	0.78179
+	PRR15	7	29528811	29577300	192	17	10164	-0.77833	0.78181
-	ERVMER34-1	4	52728952	52786640	138	10	10164	-0.77836	0.78182
-	RSPH1	21	42462486	42531354	176	33	10164	-0.77925	0.78208
+	TTLL3	3	9774960	9846356	249	29	10164	-0.77937	0.78212
-	LOC1019297	5	96924080	96971094	149	13	10164	-0.77958	0.78218
+	NDUFV3	21	42858268	42919670	219	10	10164	-0.77998	0.7823
-	SCUBE1	22	43193223	43378388	886	116	10164	-0.78023	0.78237
-	NR3C2	4	148068764	148480278	996	78	10164	-0.78042	0.78243
+	OXR1	8	106235178	106762694	1535	90	10164	-0.78046	0.78244
-	CES5A	16	55836154	55991031	574	64	10164	-0.7809	0.78257
+	ZNF174	16	3366190	3440851	273	23	10164	-0.78105	0.78261
-	ARHGEF35	7	144176083	144230698	6	3	10164	-0.78118	0.78265
-	C1QL1	17	44949693	45003276	184	37	10164	-0.78159	0.78277
+	SLC25A37	8	23493850	23582550	575	63	10164	-0.7816	0.78277
-	ZMYND8	20	47199214	47391889	233	28	10164	-0.78162	0.78278
-	TGM7	15	43266281	43337255	144	16	10164	-0.78162	0.78278
+	PELI3	11	66431327	66487337	85	16	10164	-0.78165	0.78279
-	MRPS15	1	36445761	36499439	160	17	10164	-0.78178	0.78283
+	GGPS1	1	235293438	235354532	110	6	10164	-0.78187	0.78285
-	IFITM5	11	288200	334526	87	17	10164	-0.78195	0.78288
-	C10orf95	10	102439837	102486543	147	21	10164	-0.78216	0.78294

STRAND	SYMBOL	CHR	START	STOP	NSNPS	NPARAM	N	ZSTAT	P
+	RPS15	19	1403364	1450494	33	4	10164	-0.78228	0.78298
-	MNT	17	2374060	2435964	83	10	10164	-0.78304	0.7832
+	MT1X	16	56647470	56694196	232	36	10164	-0.78319	0.78324
+	BRI3	7	98246667	98333430	317	21	10164	-0.78337	0.7833
-	CYP2A6	19	40833538	40885447	78	15	10164	-0.78349	0.78333
+	PRNP	20	4651151	4711588	174	35	10164	-0.78372	0.7834
-	GJC3	7	99913269	99970091	111	11	10164	-0.78376	0.78341
+	SLC4A1AP	2	27628471	27704980	92	20	10164	-0.78401	0.78348
+	PRKAR1A	17	68378623	68561316	490	42	10164	-0.78414	0.78352
+	SLC3A2	11	62821012	62898883	143	14	10164	-0.78414	0.78352
-	SPC25	2	168855144	168925434	194	25	10164	-0.78434	0.78358
-	PREPL	2	44307607	44396862	259	21	10164	-0.78487	0.78373
+	NME1	17	51118559	51172089	162	21	10164	-0.78488	0.78374
-	PCSK7	11	117195071	117267525	236	39	10164	-0.78521	0.78383
+	UFC1	1	161118744	161168856	54	6	10164	-0.78532	0.78387
+	NYNRIN	14	24363786	24429288	151	36	10164	-0.78532	0.78387
-	SERPINA9	14	94452721	94511333	225	33	10164	-0.78553	0.78393
-	DENND6B	22	50301963	50362089	193	20	10164	-0.78556	0.78394
+	ADSL	22	40311500	40397408	149	20	10164	-0.78567	0.78397
-	ATP6V1E1	22	17582136	17663822	350	21	10164	-0.78569	0.78398
-	TAS2R30	12	11123285	11169244	189	5	10164	-0.78599	0.78406
+	AS3MT	10	102834453	102911899	285	29	10164	-0.78652	0.78422
-	TYMSOS	18	639620	693340	265	32	10164	-0.78656	0.78423
-	MTNR1A	4	186523000	186590383	314	29	10164	-0.78669	0.78427
+	GDF11	12	55708278	55767466	67	20	10164	-0.78707	0.78438
-	C12orf50	12	87970039	88070403	209	26	10164	-0.78713	0.7844
+	PHACTR4	1	28334582	28510369	338	6	10164	-0.78716	0.78441
-	PLCD1	3	37997496	38064663	183	26	10164	-0.78724	0.78443
-	TMED1	19	10822067	10871307	82	12	10164	-0.78808	0.78467
+	PTGFR	1	78441240	78550701	274	40	10164	-0.78847	0.78479
-	SH3BP5	3	15244353	15376394	356	51	10164	-0.78849	0.78479
-	TAS2R16	7	122984705	123030700	135	17	10164	-0.78874	0.78487
-	FBXO15	18	74063343	74182865	364	11	10164	-0.7889	0.78491
+	ADGRB3	6	68600282	69399511	2226	91	10164	-0.78892	0.78492
-	PRICKLE2	3	64083850	64480476	1217	109	10164	-0.78913	0.78498
+	LOC1019295	3	11835884	11911607	228	27	10164	-0.78916	0.78499
+	PEX3	6	143415781	143500616	235	22	10164	-0.78924	0.78501
+	AIMP1	4	106280610	106359225	144	10	10164	-0.78932	0.78504
-	GCM1	6	53116961	53183826	181	20	10164	-0.7894	0.78506
+	LOC1053733	2	3985896	4035307	83	6	10164	-0.78941	0.78506
-	PODXL	7	131490262	131591617	252	49	10164	-0.78979	0.78517
-	OR5B3	11	58392465	58438409	227	7	10164	-0.78982	0.78518
-	NFKBIA	14	35391510	35439754	166	35	10164	-0.79006	0.78525
-	LCMT2	15	43317776	43365622	121	11	10164	-0.7903	0.78532
+	ASXL3	18	33543577	33761195	443	34	10164	-0.79027	0.78532
-	MUC13	3	124895442	124969751	210	43	10164	-0.79035	0.78534
+	LRRK2	12	40190011	40379285	740	42	10164	-0.79049	0.78538
+	ENTPD7	10	99624506	99721245	213	12	10164	-0.79063	0.78542
-	SLC9A1	1	27088809	27190130	178	6	10164	-0.79068	0.78543
+	IL16	15	81147600	81322763	473	39	10164	-0.79112	0.78556
-	MT1G	16	56656735	56703065	210	38	10164	-0.79147	0.78567
+	TACO1	17	63565872	63618365	27	10	10164	-0.79205	0.78583
+	RNF214	11	117197625	117295688	293	36	10164	-0.79206	0.78584
-	MFAP5	12	8635943	8697888	130	12	10164	-0.79215	0.78586
+	CHURC1	14	64879361	64945368	280	17	10164	-0.79219	0.78587
+	CLASRP	19	45004040	45080956	112	23	10164	-0.79222	0.78588
-	GFAP	17	44895626	44950552	171	28	10164	-0.79237	0.78593
+	SHBG	17	7579064	7643383	178	27	10164	-0.79242	0.78594
-	AMPD1	1	114663098	114730618	100	16	10164	-0.79267	0.78601
-	TOP3A	17	18263921	18350007	255	23	10164	-0.79326	0.78619
-	APOH	17	66202029	66264438	275	29	10164	-0.79332	0.7862
+	GPT2	16	46849396	46941289	9	3	10164	-0.79338	0.78622
+	PLEKHO2	15	64806883	64878002	175	12	10164	-0.79354	0.78627

STRAND	SYMBOL	CHR	START	STOP	NSNPS	NPARAM	N	ZSTAT	P
+	PDP1	8	93881855	93936068	148	16	10164	-0.79375	0.78633
-	UBQLN1	9	83649963	83743253	274	23	10164	-0.79383	0.78635
-	NCL	2	231444748	231499494	206	25	10164	-0.79386	0.78636
-	NPRL2	3	50337487	50386055	72	13	10164	-0.79409	0.78643
+	LGR6	1	202158990	202329785	566	57	10164	-0.7941	0.78643
-	SLFN12L	17	35464923	35526208	182	36	10164	-0.79428	0.78648
-	SCN1A	2	165979160	166184272	829	47	10164	-0.79435	0.7865
+	RPS11	19	49461365	49509712	125	14	10164	-0.7944	0.78652
-	CDK2AP1	12	123250970	123307316	148	9	10164	-0.79471	0.78661
+	NCAPH	2	96300741	96387095	160	30	10164	-0.79486	0.78665
-	ANXA2	15	60337151	60432986	208	28	10164	-0.7949	0.78666
+	ADAM23	2	206408544	206631130	342	34	10164	-0.79494	0.78668
-	TICAM2	5	115568496	115637479	142	15	10164	-0.79522	0.78676
-	CCDC23	1	42797052	42852388	87	15	10164	-0.79552	0.78684
+	CDK10	16	89637247	89706364	353	28	10164	-0.79554	0.78685
+	C7orf72	7	50060861	50169256	321	11	10164	-0.7959	0.78695
+	C5orf22	5	31497266	31565058	204	24	10164	-0.79622	0.78705
+	MPND	19	4308527	4370086	96	12	10164	-0.79664	0.78717
-	DDIT4L	4	100175110	100225782	242	24	10164	-0.79665	0.78717
+	LIN28A	1	26375778	26439728	164	22	10164	-0.79721	0.78733
-	ITGA8	10	15503949	15755335	699	47	10164	-0.79727	0.78735
-	TMUB1	7	151071085	151118533	104	16	10164	-0.7975	0.78742
-	SESN3	11	95155513	95267541	172	30	10164	-0.79759	0.78745
+	FLAD1	1	154948294	155003111	68	12	10164	-0.79769	0.78748
+	DOCK1	10	126870409	127462517	1824	129	10164	-0.79774	0.78749
-	YTHDC1	4	68300387	68385106	140	14	10164	-0.79781	0.78751
-	OR7G2	19	9092269	9138306	136	10	10164	-0.79795	0.78755
+	C4orf22	4	80300326	80973756	1445	38	10164	-0.79817	0.78761
+	ARID3A	19	891037	982816	154	17	10164	-0.79822	0.78763
+	HMGB4	1	33825475	33874791	164	13	10164	-0.79834	0.78766
-	PTPRN	2	219279623	219344573	170	13	10164	-0.79847	0.7877
-	GOLIM4	3	167998669	168130924	424	32	10164	-0.79856	0.78773
-	FBN3	19	8055402	8184012	544	59	10164	-0.79859	0.78774
-	SLN	11	107697375	107747061	84	10	10164	-0.79894	0.78784
+	SMIM10L1	12	11136181	11186020	203	6	10164	-0.79912	0.78789
-	ERG	21	38357261	38696833	861	91	10164	-0.79916	0.7879
+	C1orf162	1	111432487	111488512	128	22	10164	-0.79923	0.78792
+	OR2A2	7	144074583	144120539	78	6	10164	-0.79927	0.78793
-	SMPD3	16	68348327	68483506	263	22	10164	-0.79941	0.78797
-	GSC	14	94758223	94805162	184	41	10164	-0.7994	0.78797
-	CXCL2	4	74087035	74134280	60	13	10164	-0.79949	0.788
-	SLC12A8	3	125072636	125247765	637	81	10164	-0.79971	0.78806
+	KBTBD12	3	127880232	127997685	426	49	10164	-0.79994	0.78813
+	CARTPT	5	71684163	71731048	153	18	10164	-0.80022	0.78821
+	APOC4	19	44907238	44955496	130	15	10164	-0.80029	0.78823
-	CCL24	7	75801429	75858356	200	21	10164	-0.80064	0.78833
+	CPLX2	5	175761539	175894021	335	43	10164	-0.80078	0.78837
+	NDST3	4	117998826	118268634	739	22	10164	-0.8008	0.78838
-	TCF24	8	66936501	66997590	67	5	10164	-0.80086	0.78839
+	RBM14	11	66581582	66639934	82	26	10164	-0.80112	0.78847
+	OTULIN	5	14629674	14722867	173	17	10164	-0.80112	0.78847
-	MEN1	11	64793514	64846294	139	32	10164	-0.8012	0.78849
+	ULK1	12	131859734	131933162	109	10	10164	-0.8012	0.78849
-	DROSHA	5	31390494	31567175	505	40	10164	-0.80121	0.78849
-	LOC1053728	21	42906803	42954021	180	16	10164	-0.80135	0.78854
-	ANKMY1	2	240469422	240596068	410	51	10164	-0.80152	0.78858
-	RNASE12	14	20580080	20625823	189	32	10164	-0.80183	0.78868
+	MGST3	1	165595873	165666136	329	22	10164	-0.80194	0.78871
+	ERMAP	1	42782105	42854989	110	17	10164	-0.80202	0.78873
-	LYZL4	3	42350768	42445652	232	34	10164	-0.80202	0.78873
-	TBX18	6	84677351	84799730	261	22	10164	-0.80267	0.78892
-	POLR2I	19	36103709	36150304	118	24	10164	-0.8029	0.78899
-	SHC4	15	48813737	48998444	411	55	10164	-0.80301	0.78902

STRAND	SYMBOL	CHR	START	STOP	NSNPS	NPARAM	N	ZSTAT	P
-	SMG1	16	18794853	18961409	243	9	10164	-0.80319	0.78907
-	MRPL38	17	75888643	75940100	81	10	10164	-0.80319	0.78907
+	DHX35	20	38927338	39049723	209	14	10164	-0.80333	0.78911
-	HIST2H2BE	1	149874460	149921682	22	11	10164	-0.80352	0.78916
-	CALCA	11	14956669	15007286	120	14	10164	-0.80355	0.78917
-	TRABD2A	2	84811650	84916975	176	20	10164	-0.80355	0.78917
+	TDO2	4	155868693	155930406	181	22	10164	-0.80361	0.78919
-	RAPGEF3	12	47724670	47806035	301	39	10164	-0.80364	0.7892
-	DDX39B	6	31520219	31577475	411	49	10164	-0.80371	0.78922
-	MREG	2	215932590	216048623	323	45	10164	-0.80376	0.78923
-	SYTL2	11	85684221	85846159	431	35	10164	-0.80383	0.78925
-	SLC17A1	6	25771454	25867070	363	28	10164	-0.80525	0.78966
+	RBM15B	3	51356268	51407908	80	11	10164	-0.80568	0.78979
-	COL11A1	1	102866467	103143576	1045	25	10164	-0.80574	0.7898
-	C9orf69	9	136100220	136153885	192	25	10164	-0.80571	0.7898
+	C7orf71	7	26602488	26661531	266	35	10164	-0.80573	0.7898
-	TRIAP1	12	120433961	120481412	141	21	10164	-0.80586	0.78984
+	OCEL1	19	17191204	17239219	235	34	10164	-0.80609	0.7899
-	OR6B3	2	240034636	240088711	5	2	10164	-0.80612	0.78991
+	GLYATL3	6	49464958	49538064	178	19	10164	-0.80609	0.78991
-	PRR21	2	240031813	240077982	5	2	10164	-0.80612	0.78991
+	CAD	2	27182390	27253792	176	17	10164	-0.80629	0.78996
+	AKAP2	9	110013598	110182512	222	22	10164	-0.8064	0.78999
-	COX15	10	99686129	99767666	127	13	10164	-0.80641	0.79
+	RUSC2	9	35454838	35571898	259	47	10164	-0.80645	0.79001
+	TGFB2	1	218310334	218454619	409	50	10164	-0.80656	0.79004
+	OR6B1	7	143968997	144014932	99	12	10164	-0.80667	0.79007
+	HMBS	11	119049876	119103549	166	19	10164	-0.80668	0.79008
-	NUDT12	5	103538855	103597804	190	29	10164	-0.80701	0.79017
-	DNMT1	19	10123345	10230079	278	27	10164	-0.80724	0.79024
-	CCDC43	17	44667437	44724797	94	19	10164	-0.80736	0.79027
+	ZNF512	2	27547969	27633215	166	18	10164	-0.80741	0.79028
+	GSTO2	10	104233416	104314948	167	25	10164	-0.80742	0.79029
+	SMIM23	5	171737642	171801136	231	30	10164	-0.80766	0.79036
+	TAC1	7	97696959	97750472	33	4	10164	-0.80805	0.79047
-	ARHGAP27	17	45383902	45467916	189	13	10164	-0.80828	0.79053
+	GPR158	10	25139737	25612233	1514	46	10164	-0.80836	0.79056
+	PILRA	7	100338445	100410099	122	11	10164	-0.80893	0.79072
+	CFHR2	1	196908758	196969226	144	15	10164	-0.80905	0.79076
+	PADI3	1	17214082	17294233	248	30	10164	-0.80911	0.79077
+	ZNF660	3	44549849	44609699	89	7	10164	-0.80914	0.79078
+	POLR3B	12	106322658	106520198	421	17	10164	-0.8092	0.7908
-	NRCAM	7	108137626	108491397	1086	62	10164	-0.8093	0.79083
+	ATP2C2	16	84333523	84474187	871	87	10164	-0.80932	0.79083
-	TBCK	4	106034317	106351704	532	12	10164	-0.80945	0.79087
-	NOXO1	16	1968917	2016549	158	14	10164	-0.80974	0.79095
+	DNAJC22	12	49311917	49363684	159	21	10164	-0.80992	0.79101
-	ENTPD4	8	23419152	23492731	292	54	10164	-0.80996	0.79102
+	HBZ	16	107053	164505	144	26	10164	-0.81007	0.79105
+	EME1	17	50338220	50391752	156	13	10164	-0.81012	0.79106
-	PUS7L	12	43718025	43793817	185	15	10164	-0.81024	0.7911
+	DOCK3	3	50639969	51394198	1480	19	10164	-0.81072	0.79124
+	ADIG	20	38546195	38598463	59	10	10164	-0.81082	0.79127
-	INIP	9	112675564	112753107	175	26	10164	-0.81097	0.79131
-	CRHR2	7	30641843	30735103	218	37	10164	-0.81107	0.79134
-	STAM2	2	152106801	152210992	248	24	10164	-0.8113	0.7914
+	FMO2	1	171150208	171222683	276	22	10164	-0.81147	0.79145
+	TMEM165	4	55360913	55462974	299	17	10164	-0.81148	0.79145
-	BRICD5	16	2199253	2248540	104	16	10164	-0.81146	0.79145
+	RABGAP1L	1	174124414	175005308	1608	24	10164	-0.81172	0.79152
-	TGM2	20	38118461	38201509	253	27	10164	-0.81178	0.79154
+	RALYL	8	84148218	84931844	1149	44	10164	-0.81189	0.79157
+	PGBD1	6	28246537	28312549	240	43	10164	-0.81213	0.79164

STRAND	SYMBOL	CHR	START	STOP	NSNPS	NPARAM	N	ZSTAT	P
+	SRSF5	14	69732095	69782005	76	6	10164	-0.81235	0.7917
-	EGLN3	14	33914215	33986083	188	29	10164	-0.81237	0.79171
+	DNASE1L2	16	2201252	2248711	98	16	10164	-0.81296	0.79188
-	AP1G2	14	23549563	23604545	37	8	10164	-0.81317	0.79194
+	ZNF524	19	55564755	55613138	135	15	10164	-0.81322	0.79195
+	OR5A1	11	59403057	59454188	123	11	10164	-0.81332	0.79198
-	PSMA5	1	109389031	109461486	86	5	10164	-0.81338	0.792
+	KLHL36	16	84613511	84677686	158	16	10164	-0.81343	0.79201
+	TLR4	9	117669175	117727491	133	22	10164	-0.81349	0.79203
+	SCFD1	14	30587254	30745828	340	19	10164	-0.8136	0.79206
-	RAB1A	2	65076854	65165301	219	13	10164	-0.81412	0.79221
+	SLC6A19	5	1166595	1235117	309	51	10164	-0.81412	0.79221
-	SUCLA2	13	47932656	48036326	511	21	10164	-0.81435	0.79228
+	CHRNA5	15	78530520	78605269	261	31	10164	-0.81464	0.79236
-	PRSS42	3	46820404	46869095	124	17	10164	-0.81476	0.7924
-	RC3H1	1	173921084	174028072	202	13	10164	-0.81496	0.79245
+	OR8H2	11	56070043	56115981	338	8	10164	-0.81518	0.79251
-	DDX31	9	132582997	132705401	299	24	10164	-0.81543	0.79259
+	HNF4A	20	44320801	44442845	430	70	10164	-0.81548	0.7926
-	AP3S2	15	89820599	89929385	106	5	10164	-0.81597	0.79274
+	PMF1-BGLAI	1	156177988	156253332	178	17	10164	-0.81631	0.79284
-	SPATA22	17	3430011	3548852	562	64	10164	-0.81634	0.79285
-	CNGB1	16	57872340	58014295	466	70	10164	-0.81656	0.79291
+	CES3	16	66926229	66985149	63	13	10164	-0.81657	0.79291
-	RPTN	1	152143595	152194228	132	20	10164	-0.81663	0.79293
+	KHDRBS3	8	135422461	135666516	476	29	10164	-0.81708	0.79306
+	CDK7	5	69199795	69287430	237	15	10164	-0.81736	0.79314
+	PAPLN	14	73200843	73284640	267	43	10164	-0.81759	0.79321
+	ACTL6A	3	179527880	179598405	116	14	10164	-0.81797	0.79331
-	TOPBP1	3	133590605	133696918	333	25	10164	-0.81804	0.79333
-	SLCO1A2	12	21254600	21438657	505	38	10164	-0.81813	0.79336
+	C1QB	1	22618179	22671552	204	24	10164	-0.81827	0.7934
-	IL22	12	68238245	68288501	174	18	10164	-0.81839	0.79343
-	UCK1	9	131513796	131566275	212	30	10164	-0.81845	0.79345
-	COG5	7	107191744	107599514	721	23	10164	-0.81886	0.79357
+	CAPN5	11	77031945	77136155	158	27	10164	-0.81909	0.79363
+	COX4I1	16	85764567	85817002	160	18	10164	-0.81914	0.79365
-	CD320	19	8292127	8343356	73	3	10164	-0.81937	0.79371
+	NLRP5	19	55951783	56071813	603	61	10164	-0.81942	0.79373
+	DPYSL2	8	26479193	26668177	735	40	10164	-0.81947	0.79374
+	PMF1	1	156177988	156250077	173	16	10164	-0.81948	0.79374
+	FAR1	11	13633659	13742346	233	24	10164	-0.81968	0.7938
+	ELAC1	18	50933017	50998120	123	16	10164	-0.8199	0.79386
-	N6AMT2	13	20718931	20808956	223	15	10164	-0.81988	0.79386
+	GID4	17	18004297	18078405	173	21	10164	-0.82034	0.79399
+	ZNF726	19	23879882	23943967	272	24	10164	-0.82071	0.7941
-	DOCK10	2	224755090	225077613	809	53	10164	-0.82101	0.79418
-	TBRG4	7	45090100	45146747	193	25	10164	-0.82169	0.79437
+	MPRIP	17	17007468	17202648	323	11	10164	-0.82169	0.79437
-	TMIGD1	17	30306329	30369076	122	11	10164	-0.82198	0.79446
-	CPN1	10	100032308	100116901	240	27	10164	-0.82203	0.79447
-	CYB5R3	22	42607840	42684399	328	20	10164	-0.82237	0.79457
-	MYO5A	15	52297283	52564050	1128	26	10164	-0.82251	0.79461
-	CXXC4	4	104458306	104529901	158	34	10164	-0.82284	0.7947
+	CENPA	2	26751014	26804589	81	9	10164	-0.82307	0.79477
-	ANKRD36C	2	95817619	96027320	260	31	10164	-0.82319	0.7948
+	LOC1009962	17	77404912	77456373	94	12	10164	-0.82326	0.79482
+	XKR7	20	31933002	32008453	100	12	10164	-0.82368	0.79494
+	RAB21	12	71719850	71797370	217	22	10164	-0.82374	0.79496
-	CXCL9	4	75991342	76042523	191	11	10164	-0.8239	0.795
+	CSN3	4	70203306	70261432	278	11	10164	-0.82394	0.79501
-	OR1J1	9	122466958	122512926	137	25	10164	-0.82401	0.79503
+	BTN2A1	6	26422904	26486621	279	35	10164	-0.82452	0.79518

STRAND	SYMBOL	CHR	START	STOP	NSNPS	NPARAM	N	ZSTAT	P
-	PSMB10	16	67924504	67971877	56	8	10164	-0.8249	0.79529
+	UBR7	14	93172056	93239215	194	30	10164	-0.82496	0.7953
+	SMIM3	5	150742946	150806736	170	10	10164	-0.82514	0.79535
+	ADAM18	8	39549568	39740064	402	23	10164	-0.82519	0.79537
-	C1QL4	12	49322416	49372188	126	22	10164	-0.8258	0.79554
+	ELP3	8	28055231	28201153	287	30	10164	-0.82592	0.79557
-	GNG4	1	235537685	235685754	316	25	10164	-0.8262	0.79565
+	TNFRSF8	1	12028330	12154207	174	22	10164	-0.82649	0.79574
+	VASN	16	4336848	4393528	4	2	10164	-0.8267	0.7958
-	SYCP3	12	101718648	101774513	127	16	10164	-0.82673	0.79581
-	OR13C9	9	104607248	104653204	163	9	10164	-0.82687	0.79585
-	ERICH1	8	604737	766239	744	39	10164	-0.82713	0.79592
+	DEF6	6	35262818	35331771	310	32	10164	-0.82725	0.79595
-	RANGAP1	22	41234777	41337210	235	29	10164	-0.82734	0.79598
+	DGKD	2	233319507	233482098	498	41	10164	-0.82738	0.79599
+	BLOC1S3	19	45143745	45227078	258	40	10164	-0.82755	0.79604
+	OR4D6	11	59421961	59467905	131	9	10164	-0.82774	0.79609
+	RHOU	1	228609647	228756669	333	31	10164	-0.82777	0.7961
-	DCD	12	54634591	54683493	165	27	10164	-0.82777	0.7961
+	C6orf223	6	43965597	44015958	70	11	10164	-0.82776	0.7961
+	C9orf91	9	114568942	114656423	371	26	10164	-0.82796	0.79615
-	TLR5	1	223099404	223178282	249	12	10164	-0.82835	0.79626
-	SPRR2G	1	153139582	153185951	149	12	10164	-0.82875	0.79638
+	IPO7	11	9349622	9458127	230	15	10164	-0.82882	0.7964
-	CLDN15	7	101222092	101273820	64	17	10164	-0.82898	0.79644
-	RNF24	20	3919918	4050569	296	23	10164	-0.82935	0.79655
+	PET100	19	7594785	7641624	202	18	10164	-0.82945	0.79657
-	AGR2	7	16781811	16840114	349	27	10164	-0.82946	0.79658
+	CCL22	16	57323783	57376190	60	11	10164	-0.82989	0.7967
+	NLN	5	65687196	65839283	424	32	10164	-0.82997	0.79672
-	ASAH1	8	18046416	18119998	404	39	10164	-0.83009	0.79675
+	MROH1	8	144113016	144271940	17	1	10164	-0.83122	0.79707
+	NUBP1	16	10708321	10779351	89	12	10164	-0.83124	0.79708
-	MED8	1	43373908	43424812	102	12	10164	-0.83148	0.79715
-	TRIM43B	2	95466967	95519731	39	14	10164	-0.83147	0.79715
-	AUH	9	91203815	91396939	528	28	10164	-0.83163	0.79719
-	RHEBL1	12	49054676	49105025	58	14	10164	-0.83169	0.79721
+	FRMD8	11	65351570	65423525	157	54	10164	-0.83179	0.79724
+	IL12RB2	1	67272351	67406900	332	29	10164	-0.83186	0.79726
+	RNF111	15	58952666	59107419	321	16	10164	-0.83206	0.79731
+	TRIM2	4	153117469	153349322	392	37	10164	-0.8323	0.79738
+	GTF2IRD2B	7	75057396	75159817	8	2	10164	-0.83239	0.79741
+	RAI14	5	34621328	34842612	858	86	10164	-0.83267	0.79748
+	B2M	15	44676487	44728159	86	14	10164	-0.83287	0.79754
+	XYLB	3	38311760	38463041	318	31	10164	-0.83299	0.79757
+	SLC11A1	2	218346766	218406894	146	24	10164	-0.83301	0.79758
-	VPS41	7	38713943	38944200	310	23	10164	-0.83302	0.79758
+	STX3	11	59720059	59815882	266	9	10164	-0.83333	0.79767
+	MYO3B	2	170143145	170665164	1509	125	10164	-0.83347	0.79771
-	TBX6	16	30075793	30126937	43	7	10164	-0.83409	0.79788
-	ADGRE2	19	14714170	14813541	389	37	10164	-0.83431	0.79795
-	PUS3	11	125883485	125938369	167	27	10164	-0.83464	0.79804
-	TMTC1	12	29490813	29819759	1108	76	10164	-0.83464	0.79804
-	LOC1053732	1	247690079	247784120	245	23	10164	-0.83465	0.79804
-	ENSA	1	150612123	150664622	97	14	10164	-0.8347	0.79806
+	DARS2	1	173789645	173868544	111	14	10164	-0.83477	0.79808
-	KIAA0368	9	111350693	111519745	483	35	10164	-0.83574	0.79835
-	CRYBB1	22	26589278	26653087	248	34	10164	-0.83612	0.79846
-	TM4SF1	3	149359018	149412860	96	17	10164	-0.83638	0.79853
+	DCAF4	14	72891332	72972098	350	33	10164	-0.83654	0.79857
+	ZNF736	7	64278873	64366634	473	25	10164	-0.8366	0.79859
-	NT5DC3	12	103753312	103876245	580	33	10164	-0.83687	0.79867
-	LOC1053732	1	220865193	220914330	160	21	10164	-0.83699	0.7987

STRAND	SYMBOL	CHR	START	STOP	NSNPS	NPARAM	N	ZSTAT	P
+	GPS1	17	82016351	82067470	66	7	10164	-0.83707	0.79872
+	BRK1	3	10080649	10137190	249	17	10164	-0.83724	0.79877
-	CENPT	16	67818157	67882458	67	10	10164	-0.83743	0.79883
-	HNRNPD	4	82343314	82408996	139	18	10164	-0.83756	0.79886
+	PDCD6	5	236621	324974	405	19	10164	-0.8384	0.7991
+	VWC2L	2	214376127	214589037	467	22	10164	-0.8384	0.7991
-	LRRC73	6	43496965	43545343	76	9	10164	-0.83893	0.79925
-	LAMB4	7	107993986	108165356	351	40	10164	-0.83913	0.7993
+	CETN1	18	545343	592020	37	6	10164	-0.83916	0.79931
-	NCOA6	20	34704774	34860630	287	31	10164	-0.83929	0.79935
-	CCKAR	4	26471396	26525420	183	24	10164	-0.83952	0.79941
+	TRD	14	22387546	22476577	246	25	10164	-0.83959	0.79943
-	PRSS43	3	46743043	46847420	128	15	10164	-0.83958	0.79943
-	KRT71	12	52533909	52588147	284	48	10164	-0.83974	0.79947
+	PPP1R8	1	27795741	27861672	70	5	10164	-0.83975	0.79948
+	RPS3	11	75364491	75432302	125	16	10164	-0.83983	0.7995
-	USH1C	11	17483895	17579416	245	40	10164	-0.83984	0.7995
-	WASH1	9	4511	65487	4	2	10164	-0.8399	0.79952
+	RAD54L2	3	51503683	51678660	242	22	10164	-0.84052	0.79969
-	ERCC6	10	49444480	49574123	314	20	10164	-0.84058	0.79971
+	MCM5	22	35365063	35464889	230	21	10164	-0.84058	0.79971
-	CHRDL2	11	74686428	74766385	140	19	10164	-0.84066	0.79973
+	KLF2	19	16289840	16337534	27	4	10164	-0.84075	0.79976
+	ADRA1B	5	159881733	159996178	237	37	10164	-0.84096	0.79982
-	ANKRD50	4	124654048	124748220	264	42	10164	-0.84124	0.79989
+	SLC35F6	2	26729274	26791231	91	9	10164	-0.84127	0.7999
+	LAPTM4B	8	97740581	97862602	571	30	10164	-0.84202	0.80011
+	NRXN3	14	77986758	79878290	5778	315	10164	-0.84207	0.80013
-	FAM212B	1	111712063	111790797	242	30	10164	-0.84317	0.80043
+	LOC1053794	16	30359113	30405749	6	2	10164	-0.84327	0.80046
+	CENPW	6	126304789	126493320	148	9	10164	-0.84364	0.80056
-	FAM89A	1	231008958	231075249	273	34	10164	-0.84368	0.80057
+	METTL2B	7	128441729	128512924	183	20	10164	-0.84374	0.80059
-	OR10V1	11	59702916	59748845	125	7	10164	-0.84386	0.80063
+	PLEKHB2	2	131069735	131159852	80	3	10164	-0.84396	0.80066
-	OR51I1	11	5430542	5476553	252	32	10164	-0.8443	0.80075
+	PSMD2	3	184264098	184319054	103	20	10164	-0.84448	0.8008
-	PURG	8	30985802	31068715	176	31	10164	-0.84474	0.80087
+	SNRPD3	22	24520650	24584964	126	11	10164	-0.84477	0.80088
-	SVEP1	9	110355248	110614880	933	69	10164	-0.84494	0.80093
+	GRAMD4	22	46541017	46692758	517	28	10164	-0.84521	0.801
-	MRGPRF	11	68994394	69048382	187	47	10164	-0.84523	0.80101
+	ZNF407	18	74562870	75075672	907	37	10164	-0.84549	0.80108
-	OSCP1	1	36407906	36485485	296	13	10164	-0.84563	0.80112
+	ID2	2	8646983	8694453	11	3	10164	-0.8457	0.80114
-	PRPSAP1	17	76300787	76389149	262	26	10164	-0.84581	0.80117
-	MYO1G	7	44952661	45014105	104	19	10164	-0.84604	0.80123
+	L1TD1	1	62159802	62222328	308	26	10164	-0.84611	0.80126
-	CRH	8	66166377	66213611	40	9	10164	-0.84619	0.80128
+	ZSCAN16	6	28089556	28140086	228	26	10164	-0.84634	0.80132
+	FURIN	15	90833592	90893458	137	25	10164	-0.84641	0.80134
-	ABCA10	17	69138007	69279815	317	18	10164	-0.84706	0.80152
+	G6PC2	2	168866240	168920000	147	22	10164	-0.84705	0.80152
+	GIGYF2	2	232662305	232870577	616	31	10164	-0.84737	0.80161
-	INSL5	1	66787741	66836259	88	14	10164	-0.84744	0.80163
+	NMT1	17	45026283	45119016	352	30	10164	-0.8477	0.8017
+	GATC	12	120411438	120473753	197	28	10164	-0.84816	0.80183
-	CUBN	10	16813966	17165249	1181	68	10164	-0.84831	0.80187
-	TPST2	22	26515748	26625125	458	49	10164	-0.84839	0.80189
+	ZNF341	20	33696760	33802269	199	32	10164	-0.8484	0.80189
+	RAB42	1	28557200	28604586	53	8	10164	-0.84839	0.80189
-	FBXO31	16	87316987	87427107	217	47	10164	-0.84862	0.80195
+	CAMLG	5	134702927	134762160	108	16	10164	-0.8488	0.802

STRAND	SYMBOL	CHR	START	STOP	NSNPS	NPARAM	N	ZSTAT	P
+	FUT4	11	94508851	94559898	207	23	10164	-0.84957	0.80222
+	PPOX	1	161130728	161188277	65	8	10164	-0.84991	0.80231
+	ADRB1	10	114009047	114056908	148	8	10164	-0.84996	0.80233
-	SLC7A14	3	170449554	170621074	425	51	10164	-0.85016	0.80238
-	CETN3	5	90383335	90444786	83	24	10164	-0.85024	0.8024
-	BRAP	12	111632146	111720996	126	23	10164	-0.85045	0.80246
-	CCL1	17	34350328	34398233	154	14	10164	-0.85051	0.80248
-	CYP2D6	22	42115656	42170378	117	13	10164	-0.85068	0.80253
+	IRAK4	12	43723944	43799543	173	14	10164	-0.85092	0.80259
+	NPAS2	2	100785151	101006827	877	79	10164	-0.85095	0.8026
+	RPS3A	4	151064573	151114652	90	11	10164	-0.85102	0.80262
-	LOC1027241	17	7572170	7618814	111	23	10164	-0.85104	0.80263
+	ROM1	11	62577741	62625120	47	7	10164	-0.85116	0.80266
+	LGALS14	19	39669306	39719448	135	14	10164	-0.85117	0.80266
-	MPP4	2	201634874	201733834	345	56	10164	-0.85191	0.80287
+	TRIM71	3	32783018	32902279	273	38	10164	-0.85209	0.80292
-	TMEM225	11	123872920	123920650	185	27	10164	-0.85232	0.80298
+	LAX1	1	203730146	203786372	179	29	10164	-0.85235	0.80299
-	WDR86	7	151368541	151445705	253	44	10164	-0.85255	0.80305
+	CD300LG	17	43811777	43873639	65	22	10164	-0.85262	0.80306
+	TMEM251	14	93149951	93197086	177	15	10164	-0.85264	0.80307
+	RNASE2	14	20920471	20966435	120	15	10164	-0.85284	0.80313
-	NEURL3	2	96487643	96543136	88	26	10164	-0.85293	0.80315
+	SLC35G2	3	136784019	136865892	199	10	10164	-0.85334	0.80327
+	NEDD4L	18	58009233	58411541	881	83	10164	-0.85353	0.80332
-	ZKSCAN4	6	28231705	28294271	213	41	10164	-0.85371	0.80337
+	RAB5A	3	19912080	19995175	254	22	10164	-0.85399	0.80344
-	NBPF4	1	108212464	108279081	30	6	10164	-0.85424	0.80351
-	NBAS	2	15156908	15596348	1635	39	10164	-0.85451	0.80359
-	MRPL36	5	1788385	1836423	174	14	10164	-0.85456	0.8036
+	ZNF254	19	23998405	24139968	538	21	10164	-0.85493	0.80371
+	TRAF3	14	102742475	102921500	382	20	10164	-0.85522	0.80378
-	NDST2	10	73791911	73846831	90	12	10164	-0.85525	0.80379
+	SHKBP1	19	40541851	40601399	71	9	10164	-0.85528	0.8038
-	ETV5	3	186036317	186144112	175	21	10164	-0.85543	0.80384
+	CWH43	4	48951247	49072079	251	23	10164	-0.85582	0.80395
+	NLRP2	19	54930284	55011142	202	21	10164	-0.85591	0.80398
+	NUTM2B	10	79664700	79725486	145	9	10164	-0.85613	0.80404
-	LOC1053703	13	96076390	96125864	95	10	10164	-0.85626	0.80407
+	C1orf185	1	51067234	51158082	160	9	10164	-0.85636	0.8041
-	PBXIP1	1	154934080	154991091	84	16	10164	-0.85651	0.80414
+	HYKK	15	78472564	78547373	273	35	10164	-0.85676	0.80421
-	ZNF579	19	55567523	55615899	123	13	10164	-0.857	0.80428
-	FYB	5	39095252	39309523	771	53	10164	-0.85706	0.80429
-	SLC52A3	20	750080	810985	240	28	10164	-0.85709	0.8043
+	DDX11	12	31038581	31114799	242	18	10164	-0.8573	0.80436
+	COX5B	2	97611058	97658194	47	9	10164	-0.85745	0.8044
+	SNX7	1	98626662	98770500	445	24	10164	-0.85746	0.8044
-	RHBDF2	17	76460893	76536437	97	11	10164	-0.85784	0.80451
-	SBF2	11	9768667	10329207	1955	42	10164	-0.85794	0.80454
+	EBF4	20	2657878	2770108	324	40	10164	-0.85816	0.8046
-	PLXNA4	7	132113332	132683688	1299	129	10164	-0.85821	0.80461
-	MPP2	17	43865357	43944711	167	18	10164	-0.8584	0.80467
-	SERPINB3	18	63645197	63696963	73	12	10164	-0.85863	0.80473
+	LOC1053792	1	226936537	226987442	268	36	10164	-0.85918	0.80488
+	KIF26B	1	245119985	245719432	1773	166	10164	-0.85934	0.80492
+	IRGM	5	150811523	150912400	309	9	10164	-0.85933	0.80492
+	MTHFD1	14	64353041	64470007	351	38	10164	-0.85946	0.80496
+	PTPN23	3	47345982	47423441	122	22	10164	-0.85962	0.805
-	NT5C1B	2	18552871	18624580	134	18	10164	-0.85964	0.80501
-	SPTLC2	14	77495997	77651768	576	42	10164	-0.85978	0.80505
-	VNN2	6	132733870	132798459	196	18	10164	-0.85999	0.8051
-	MPZL3	11	118216690	118287368	271	21	10164	-0.86	0.80511

STRAND	SYMBOL	CHR	START	STOP	NSNPS	NPARAM	N	ZSTAT	P
-	PRLR	5	35038756	35265589	790	58	10164	-0.86011	0.80514
+	LCOR	10	96797260	96974441	210	21	10164	-0.86044	0.80523
-	CLDN1	3	190295701	190357446	320	21	10164	-0.86087	0.80535
-	SFRP2	4	153770590	153824076	180	16	10164	-0.86102	0.80539
-	MASP2	1	11016523	11082239	195	15	10164	-0.86133	0.80547
-	CCDC87	11	66580169	66628083	68	24	10164	-0.86157	0.80554
+	FAM83F	22	39959949	40040039	162	30	10164	-0.86168	0.80557
-	FXYD6-FXYD	11	117810075	117912031	549	79	10164	-0.86179	0.8056
+	OR8I2	11	56058308	56104240	321	8	10164	-0.86183	0.80561
-	TMEM161A	19	19109616	19173554	217	26	10164	-0.86252	0.8058
+	COX20	1	244800337	244855057	8	1	10164	-0.86274	0.80586
+	ARHGAP35	19	46883567	47015077	177	19	10164	-0.86302	0.80594
-	C1orf87	1	59980394	60108770	352	45	10164	-0.86317	0.80598
+	LIPC	15	58375754	58578874	731	97	10164	-0.86331	0.80602
-	UGT2A1	4	69578417	69688249	438	36	10164	-0.86335	0.80603
-	LY75	2	159793355	159939756	465	29	10164	-0.86346	0.80606
+	SLC16A10	6	111052503	111233405	321	32	10164	-0.86364	0.80611
-	ACAD11	3	132548138	132695131	267	29	10164	-0.8637	0.80612
-	MCFD2	2	46891870	46976855	268	22	10164	-0.86403	0.80622
+	TMEM213	7	138762915	138816024	199	29	10164	-0.86412	0.80624
-	CXCL14	5	135560679	135614279	111	7	10164	-0.8644	0.80632
+	OMP	11	77067840	77113331	55	13	10164	-0.86452	0.80635
-	ZNHIT2	11	65106403	65152738	98	27	10164	-0.86511	0.80651
+	DCAF13	8	103379714	103453453	220	14	10164	-0.86509	0.80651
+	SERPINE3	13	51303519	51375454	204	20	10164	-0.86544	0.8066
+	TNRC6B	22	40009817	40345808	774	34	10164	-0.86557	0.80664
+	DDX20	1	111720568	111777577	194	24	10164	-0.86589	0.80673
+	BTN3A1	6	26367237	26425216	343	24	10164	-0.86598	0.80675
+	CEP63	3	134450733	134585013	349	12	10164	-0.86598	0.80675
+	BTN3A2	6	26330159	26461653	681	35	10164	-0.86604	0.80677
+	AKR1B15	7	134511245	134589861	373	28	10164	-0.86608	0.80678
+	LENEP	1	154958586	155004315	59	9	10164	-0.86652	0.8069
-	PPP1R2	3	195504422	195578401	35	4	10164	-0.86671	0.80695
+	C8orf44-SGK	8	66632552	66872022	370	11	10164	-0.86691	0.807
-	SORT1	1	109299565	109432941	156	7	10164	-0.86706	0.80705
+	GPD2	2	156400290	156596403	411	30	10164	-0.86715	0.80707
+	INSC	11	15076430	15257208	583	67	10164	-0.86737	0.80713
+	CTPS1	1	40944299	41022565	297	32	10164	-0.86767	0.80721
-	DLGAP5	14	55138116	55226678	252	23	10164	-0.86768	0.80722
-	SLFN11	17	35340305	35408853	200	30	10164	-0.86772	0.80722
-	ZNF667	19	56428512	56513065	198	22	10164	-0.86827	0.80738
-	TREM2	6	41148506	41198198	121	13	10164	-0.8686	0.80747
-	INTS5	11	62636848	62688302	57	11	10164	-0.86913	0.80761
-	APPBP2	17	60433149	60561240	59	8	10164	-0.86928	0.80765
+	SEC61B	9	99187288	99240619	61	9	10164	-0.86964	0.80775
+	PKIG	20	44496781	44629037	207	17	10164	-0.86969	0.80776
-	JAK1	1	64823223	65001910	325	15	10164	-0.86983	0.8078
-	GPHA2	11	64924471	64973132	93	19	10164	-0.86987	0.80781
-	SYCE2	19	12889076	12954343	69	6	10164	-0.87001	0.80785
-	TRAF3IP2	6	111545378	111641874	310	32	10164	-0.87003	0.80786
-	PLXDC1	17	39053303	39186649	507	49	10164	-0.87006	0.80786
+	TMEM132E	17	34545749	34649319	303	54	10164	-0.87009	0.80787
+	LOC1053775	4	173492382	173593168	167	31	10164	-0.87026	0.80792
+	C11orf44	11	130637956	130727352	35	8	10164	-0.87049	0.80798
+	PDIA2	16	248118	297209	221	41	10164	-0.87065	0.80803
+	NLRP3	1	247380904	247459108	276	36	10164	-0.87073	0.80805
-	ZNF695	1	246935547	247043093	316	23	10164	-0.87082	0.80807
+	NUDT7	16	77687492	77752260	387	37	10164	-0.87092	0.8081
+	DCLK2	4	150043274	150267457	632	39	10164	-0.87094	0.80811
-	C6orf106	6	34577280	34731850	514	41	10164	-0.87147	0.80825
-	GNL2	1	37556812	37630985	190	27	10164	-0.87156	0.80828
+	MYPN	10	68071117	68222017	523	40	10164	-0.87161	0.80829
-	SV2A	1	149893318	149952882	27	9	10164	-0.87181	0.80834

STRAND	SYMBOL	CHR	START	STOP	NSNPS	NPARAM	N	ZSTAT	P
-	YIPF2	19	10912768	10964012	135	18	10164	-0.87222	0.80845
-	LSM10	1	36383420	36432959	232	14	10164	-0.87292	0.80865
-	DNAJB7	22	40849549	40897126	67	18	10164	-0.87301	0.80867
+	GNA11	19	3059410	3134002	254	34	10164	-0.87306	0.80869
+	TIMP3	22	32765816	32873041	384	39	10164	-0.87307	0.80869
-	C11orf24	11	68251335	68307001	169	24	10164	-0.87314	0.80871
+	BPIFA3	20	33182310	33237806	98	18	10164	-0.8733	0.80875
-	SLC6A16	19	49279632	49366701	137	10	10164	-0.87332	0.80876
+	DGUOK	2	73891826	73968961	109	6	10164	-0.87356	0.80882
+	CCNJ	10	96006774	96070870	101	11	10164	-0.87376	0.80888
+	CNTN4	3	2063866	3067961	3121	270	10164	-0.8738	0.80889
+	NEUROD4	12	54984945	55040017	160	23	10164	-0.87402	0.80895
-	TMEM116	12	111884930	112048219	324	13	10164	-0.87402	0.80895
+	IFT43	14	75950753	76094073	346	36	10164	-0.87475	0.80914
+	SQRDL	15	45596148	45701294	375	43	10164	-0.87514	0.80925
+	PRIMPOL	4	184614613	184704959	315	20	10164	-0.87537	0.80931
+	TBL3	16	1937063	1988750	177	30	10164	-0.87552	0.80935
-	SEC16B	1	177919107	178004915	369	47	10164	-0.87557	0.80937
-	DCLK1	13	35758652	36167020	1265	91	10164	-0.87565	0.80939
-	CASC10	10	21484492	21532284	52	15	10164	-0.87589	0.80946
+	SETD8	12	123348778	123419358	152	8	10164	-0.8761	0.80951
-	EPB41L2	6	130818173	131098322	931	29	10164	-0.8762	0.80954
+	WBP2NL	22	41960346	42069574	226	17	10164	-0.87627	0.80956
+	CEP57L1	6	109060153	109173912	127	13	10164	-0.87652	0.80963
+	RTP5	2	241833319	241883823	181	12	10164	-0.87658	0.80964
-	FUT1	19	48738011	48790390	67	9	10164	-0.87666	0.80966
-	NXF1	11	62782126	62840492	92	7	10164	-0.87682	0.80971
+	ZKSCAN7	3	44520175	44593464	106	7	10164	-0.87691	0.80973
+	ONECUT2	18	57400685	57501298	333	29	10164	-0.87693	0.80974
+	GSTM4	1	109621076	109675501	89	14	10164	-0.87718	0.80981
+	TEAD4	12	2924312	3050676	280	32	10164	-0.87719	0.80981
-	FDXR	17	74852497	74908031	163	17	10164	-0.8773	0.80984
+	XCL1	1	168541061	168592077	156	14	10164	-0.87731	0.80984
+	PAIP2	5	139306832	139379720	76	10	10164	-0.87746	0.80988
-	PSPH	7	56000363	56086604	253	29	10164	-0.87752	0.8099
+	TUBGCP4	15	43336059	43417095	192	15	10164	-0.87758	0.80991
-	FAM71E2	19	55344908	55398252	168	27	10164	-0.87765	0.80993
-	CTSE	1	205999264	206058909	9	4	10164	-0.87792	0.81001
-	SEMA5A	5	9025026	9581121	1805	162	10164	-0.878	0.81003
+	ZNF469	16	88198015	88450757	596	43	10164	-0.87831	0.81011
-	ABCB10	1	229506582	229593695	142	14	10164	-0.87865	0.8102
+	MROH9	1	170900471	171075985	241	25	10164	-0.87874	0.81023
+	RALGAPB	20	38437806	38588861	147	15	10164	-0.87882	0.81025
-	LOC1019287	13	21277321	21333115	226	12	10164	-0.87909	0.81032
+	CYP2U1	4	107896549	107963461	151	10	10164	-0.8791	0.81033
-	TRIP12	2	229753837	229958234	231	21	10164	-0.87918	0.81035
+	OR5L1	11	55776467	55822402	157	18	10164	-0.87923	0.81036
+	VANGL2	1	160365574	160438678	203	21	10164	-0.8796	0.81046
+	DEFB129	20	192258	239886	134	14	10164	-0.87978	0.81051
+	SNTG1	8	49874789	50806442	3092	56	10164	-0.88023	0.81063
+	ZNF317	19	9105380	9173415	223	23	10164	-0.88038	0.81067
+	BRIX1	5	34880715	34935682	120	21	10164	-0.88052	0.81071
-	ARL5C	17	39146894	39201161	142	25	10164	-0.8807	0.81076
-	KIF14	1	200541497	200655791	292	30	10164	-0.88103	0.81085
-	PI4KB	1	151281797	151362715	113	17	10164	-0.88107	0.81086
-	KLHL25	15	85749326	85829958	450	43	10164	-0.88112	0.81087
+	MSH6	2	47748082	47816953	106	34	10164	-0.88118	0.81089
+	SERPINH1	11	75527056	75582804	205	37	10164	-0.88137	0.81094
+	GNG2	14	51812131	51979800	732	78	10164	-0.88158	0.811
+	CD3G	11	118309316	118363782	123	16	10164	-0.88168	0.81103
+	DVL3	3	184120311	184183526	109	16	10164	-0.8817	0.81103
-	BCL2	18	63113346	63354778	693	107	10164	-0.88191	0.81109
-	FAM13C	10	59236129	59398181	230	28	10164	-0.882	0.81111

STRAND	SYMBOL	CHR	START	STOP	NSNPS	NPARAM	N	ZSTAT	P
+	KRTAP9-8	17	41203018	41249004	127	16	10164	-0.88205	0.81113
-	KRT1	12	52664736	52715407	186	27	10164	-0.88255	0.81126
+	CEP55	10	93461612	93539094	282	26	10164	-0.88265	0.81129
+	C2orf73	2	54295013	54393673	270	37	10164	-0.88264	0.81129
-	PGP	16	2201602	2249821	100	16	10164	-0.88277	0.81132
-	ZNF322	6	26624383	26694752	189	25	10164	-0.88308	0.8114
-	RXRB	6	33183585	33235853	252	55	10164	-0.88319	0.81143
+	BLOC1S1	12	55681034	55729707	89	12	10164	-0.88326	0.81145
+	TMEM180	10	102426381	102490118	190	25	10164	-0.88356	0.81153
+	ZNF766	19	52234571	52302726	294	23	10164	-0.88415	0.81169
+	PCM1	8	17887857	18039948	578	27	10164	-0.88421	0.81171
+	LOC1053740	3	126900167	126960505	103	7	10164	-0.88424	0.81172
-	KDM5B	1	202717403	202843468	367	22	10164	-0.88444	0.81177
+	RPAIN	17	5384641	5443020	261	13	10164	-0.88446	0.81177
+	MICALL1	22	37871148	37952458	116	17	10164	-0.88459	0.81181
+	SPR	2	72852383	72902160	66	6	10164	-0.88461	0.81182
+	ZSCAN26	6	28232010	28288224	190	38	10164	-0.88461	0.81182
-	ICA1L	2	202763150	202906985	312	18	10164	-0.88465	0.81183
+	SGK3	8	66677418	66872022	323	9	10164	-0.88485	0.81188
-	GANAB	11	62614826	62681726	58	10	10164	-0.88553	0.81206
-	AKT1	14	104759349	104830743	217	39	10164	-0.88574	0.81212
+	TNFRSF19	13	23535370	23686105	590	44	10164	-0.88593	0.81217
-	CSF3R	1	36456043	36518314	162	27	10164	-0.88596	0.81218
-	UGDH	4	39488755	39562598	286	38	10164	-0.88598	0.81219
-	PDE7A	8	65704334	65876734	241	25	10164	-0.88604	0.8122
+	TEX9	15	56209085	56469738	685	21	10164	-0.88607	0.81221
-	INTS6	13	51343862	51488236	415	18	10164	-0.88623	0.81225
+	KIAA1804	1	233292743	233395148	364	19	10164	-0.88651	0.81233
-	KMT2D	12	49008975	49096895	84	12	10164	-0.88657	0.81234
+	NUDT15	13	48002567	48057222	250	24	10164	-0.88658	0.81235
-	ALDH3B2	11	67652155	67716214	241	34	10164	-0.88673	0.81239
-	SLC35E2	1	1714838	1780999	10	2	10164	-0.88711	0.81249
-	ACOX2	3	58495136	58572348	128	16	10164	-0.88738	0.81256
-	EML5	14	88602431	88827752	446	28	10164	-0.88765	0.81264
+	RIMS1	6	71851703	72413143	1433	65	10164	-0.88769	0.81265
+	SYT16	14	61777729	62127043	1040	73	10164	-0.8877	0.81265
-	PLEKHA7	11	16767297	17049417	799	46	10164	-0.88772	0.81265
+	SEC23IP	10	119857573	119954658	374	15	10164	-0.88782	0.81268
+	ENY2	8	109299323	109355960	162	23	10164	-0.88828	0.81281
+	SCUBE3	6	35179047	35263079	263	39	10164	-0.88841	0.81284
-	MDGA1	6	37622508	37732990	424	58	10164	-0.88857	0.81288
-	FBXW4	10	101600664	101730037	236	40	10164	-0.88898	0.81299
-	SPAG5	17	28567565	28634038	55	18	10164	-0.88896	0.81299
-	TACR1	2	75036463	75234519	482	66	10164	-0.88906	0.81301
-	DHRS7	14	60134778	60200493	200	15	10164	-0.88906	0.81302
-	LYSMD4	15	99706804	99768458	115	11	10164	-0.88921	0.81305
-	RNF44	5	176516699	176578413	259	33	10164	-0.88928	0.81307
+	XYLT2	17	50311032	50371185	145	18	10164	-0.88936	0.8131
+	THRSP	11	78028861	78078357	195	14	10164	-0.88948	0.81313
+	RABGGTB	1	75751194	75805090	168	18	10164	-0.88956	0.81315
-	GRTP1	13	113314164	113399148	215	24	10164	-0.88968	0.81318
+	HCN3	1	155242427	155299848	107	9	10164	-0.88987	0.81323
-	ATP2B1	12	89578049	89744353	180	28	10164	-0.89021	0.81332
+	LOC1027251	7	12461425	12554195	604	41	10164	-0.89027	0.81334
+	UGP2	2	63805950	63901562	194	32	10164	-0.89047	0.81339
-	KERA	12	91040491	91093354	223	23	10164	-0.89084	0.81349
-	SLC25A12	2	171773405	171929306	373	26	10164	-0.89136	0.81363
-	NAT8B	2	73690509	73736340	8	2	10164	-0.89137	0.81363
+	TMEM126B	11	85593573	85646539	123	17	10164	-0.89138	0.81364
+	CCZ1	7	5863710	5935976	224	31	10164	-0.89141	0.81365
-	NARFL	16	719753	776038	143	24	10164	-0.89145	0.81366
+	LOC1019299	2	126863878	126912097	95	9	10164	-0.89156	0.81368
-	FAU	11	65110627	65157200	97	27	10164	-0.89175	0.81374

STRAND	SYMBOL	CHR	START	STOP	NSNPS	NPARAM	N	ZSTAT	P
-	FAM189B	1	155237205	155290483	100	9	10164	-0.89192	0.81378
+	APOC2	19	44910982	44959565	135	15	10164	-0.89194	0.81379
+	STARD3NL	7	38143206	38240671	79	9	10164	-0.89226	0.81387
-	TMEM211	22	24924773	24974347	131	17	10164	-0.89248	0.81393
+	EML4	2	42134338	42342548	795	35	10164	-0.89251	0.81394
+	C14orf159	14	91079013	91235396	238	38	10164	-0.8926	0.81396
-	KIAA1524	3	108535752	108624644	154	17	10164	-0.89316	0.81411
-	TCHH	1	152096317	152150454	95	9	10164	-0.89318	0.81412
+	CACNB2	10	18105677	18551759	1884	148	10164	-0.89323	0.81413
+	GHITM	10	84104429	84163555	216	21	10164	-0.89322	0.81413
+	EEFSEC	3	128118457	128436193	881	42	10164	-0.89341	0.81418
-	TUT1	11	62565045	62626637	49	8	10164	-0.89355	0.81422
-	ANAPC13	3	134467704	134521023	151	10	10164	-0.89358	0.81423
+	CCDC155	19	49353218	49427999	216	10	10164	-0.89408	0.81436
+	ADAD2	16	84156117	84207166	318	30	10164	-0.89409	0.81436
-	GADD45GIP1	19	12944156	12992254	31	8	10164	-0.8943	0.81442
+	DPP3	11	66445013	66519659	159	18	10164	-0.89472	0.81453
+	OR1L4	9	122688990	122734925	44	11	10164	-0.895	0.81461
-	SLC22A6	11	62966597	63020023	121	23	10164	-0.89536	0.8147
-	TSC22D4	7	100456519	100514279	96	12	10164	-0.89561	0.81477
+	DGKE	17	56799099	56879567	90	4	10164	-0.89569	0.81479
+	CACNA1B	9	137842789	138134624	416	26	10164	-0.89589	0.81485
-	GPCPD1	20	5534434	5646026	268	25	10164	-0.89596	0.81486
+	MT1H	16	56634814	56681129	236	30	10164	-0.89669	0.81506
+	ACADSB	10	122973913	123068290	252	22	10164	-0.89693	0.81512
-	TNS3	7	47265144	47617553	955	104	10164	-0.89748	0.81527
-	CEACAM8	19	42570243	42629939	18	6	10164	-0.89755	0.81529
-	SRSF12	6	89085957	89153081	230	14	10164	-0.89758	0.81529
-	RGMA	15	93033406	93124214	362	60	10164	-0.89775	0.81534
+	CFHR3	1	196739800	196804073	47	7	10164	-0.89786	0.81537
-	CTGF	6	131938176	131986378	104	16	10164	-0.89805	0.81542
-	SH3TC2	5	148972150	149098174	345	21	10164	-0.89867	0.81558
-	GLUD1	10	87040202	87130019	184	17	10164	-0.89877	0.81561
+	WIPF3	7	29771554	29927066	589	39	10164	-0.8989	0.81565
-	SLC6A13	12	210621	299308	304	31	10164	-0.89911	0.8157
+	FOXD4L1	2	113464084	113511150	32	3	10164	-0.89971	0.81586
+	NOLC1	10	102117176	102173871	87	29	10164	-0.90044	0.81606
-	SVIP	11	22812052	22864836	212	22	10164	-0.90046	0.81606
-	KIF9	3	47218026	47317847	127	20	10164	-0.9006	0.8161
-	PLA2G1B	12	120312111	120362789	137	16	10164	-0.90077	0.81614
+	DDX46	5	134723734	134841138	213	20	10164	-0.90076	0.81614
+	ATF1	12	50728710	50831167	192	18	10164	-0.90108	0.81623
-	SPATA16	3	172879357	173176268	537	45	10164	-0.90118	0.81625
+	KCNA5	12	5008919	5056788	119	26	10164	-0.90156	0.81636
+	VPREB1	22	22209780	22255515	137	10	10164	-0.90162	0.81637
-	KIAA0319L	1	35423490	35592670	264	22	10164	-0.90205	0.81648
-	LOC728392	17	5489427	5535999	269	24	10164	-0.90209	0.8165
-	TRIM17	1	228397935	228451882	181	27	10164	-0.90223	0.81653
-	OR6P1	1	158552651	158598604	91	7	10164	-0.90284	0.81669
-	TGFA	2	70437280	70589015	595	64	10164	-0.90286	0.8167
+	SMC2	9	104058848	104151419	465	18	10164	-0.90301	0.81674
-	TADA3	3	9769964	9828011	200	26	10164	-0.90315	0.81678
+	CD68	17	7544487	7592111	139	25	10164	-0.90333	0.81683
+	LGALS13	19	39567359	39617474	158	12	10164	-0.90357	0.81689
+	SMARCAD1	4	94172608	94301292	354	17	10164	-0.90389	0.81697
+	C2orf88	2	189844553	190213484	793	57	10164	-0.90446	0.81713
-	SLC13A4	7	135671231	135763185	321	31	10164	-0.90458	0.81716
+	LEPROT	1	65385448	65446007	165	26	10164	-0.90524	0.81733
-	GRIN2C	17	74832021	74896514	175	16	10164	-0.90564	0.81744
-	TMEM200C	18	5879412	5932101	101	16	10164	-0.90564	0.81744
+	VTA1	6	142112162	142230948	263	19	10164	-0.90609	0.81756
-	SHARPIN	8	144088633	144139237	28	2	10164	-0.90609	0.81756
-	SAR1B	5	134591149	134667843	108	20	10164	-0.90625	0.8176

STRAND	SYMBOL	CHR	START	STOP	NSNPS	NPARAM	N	ZSTAT	P
+	THEG5	19	31553189	31603550	31	3	10164	-0.90629	0.81761
-	C16orf46	16	81043497	81112267	327	14	10164	-0.90645	0.81765
-	PYGO2	1	154947026	154996782	65	12	10164	-0.90659	0.81769
-	MOAP1	14	93172196	93219904	136	24	10164	-0.90698	0.81779
+	SNRPB2	20	16694964	16751772	198	29	10164	-0.9072	0.81785
+	LOC1001296	16	88905039	88961524	227	13	10164	-0.90728	0.81787
-	LOC646588	7	25645209	25705674	203	15	10164	-0.90733	0.81788
+	TRAF4	17	28709005	28760958	59	10	10164	-0.90801	0.81806
+	ADAMTS16	5	5105330	5330299	922	100	10164	-0.90822	0.81812
-	CRYGB	2	208132573	208181153	205	20	10164	-0.90847	0.81818
+	SMYD1	2	88032780	88123384	118	19	10164	-0.90847	0.81818
-	ASNS	7	97842117	97907542	157	21	10164	-0.90848	0.81819
-	DAB1	1	56987906	58285539	3322	218	10164	-0.90851	0.8182
+	C9orf89	9	93061129	93123283	196	19	10164	-0.90868	0.81824
+	SPAM1	7	123890232	123981410	122	19	10164	-0.90887	0.81829
-	ORC2	2	200900171	200998703	164	32	10164	-0.90908	0.81835
-	ANKRD18A	9	38532965	38655655	140	23	10164	-0.90909	0.81835
+	MYOCD	17	12630890	12778949	551	66	10164	-0.90972	0.81851
-	RBM19	12	113806738	114001371	892	52	10164	-0.9101	0.81861
-	OR4C12	11	49971473	50017535	147	13	10164	-0.9102	0.81864
+	FPR2	19	51725920	51780670	215	47	10164	-0.9105	0.81872
-	LRG1	19	4527215	4575024	5	1	10164	-0.91085	0.81881
+	ZNF85	19	20888221	20960697	419	18	10164	-0.9116	0.81901
+	LOC1053761	9	104235224	104291266	187	12	10164	-0.912	0.81912
+	GPHN	14	66472407	67191808	1740	26	10164	-0.91205	0.81913
+	NCF1	7	74738962	74799376	5	3	10164	-0.91232	0.8192
+	MT1F	16	56622943	56669303	244	28	10164	-0.91243	0.81923
+	DAND5	19	12934618	12984753	34	8	10164	-0.91251	0.81925
-	KRT36	17	41476136	41529015	116	22	10164	-0.91293	0.81936
+	UBN1	16	4812446	4892401	100	5	10164	-0.91304	0.81939
-	PAK7	20	9527389	9874039	983	80	10164	-0.91317	0.81942
+	TMTC3	12	88107296	88209887	184	14	10164	-0.9133	0.81946
+	MRPL48	11	73752872	73874611	345	19	10164	-0.91348	0.81951
-	ADGRL4	1	78879764	79041810	656	32	10164	-0.91348	0.81951
-	ZNF800	7	127336721	127427724	269	21	10164	-0.9137	0.81956
+	GDPD1	17	59185467	59285970	204	15	10164	-0.91368	0.81956
-	GPR153	1	6237346	6295990	134	16	10164	-0.9137	0.81956
+	PWP2	21	44072290	44141181	51	6	10164	-0.91372	0.81957
+	CCDC67	11	93294952	93448470	424	22	10164	-0.91406	0.81966
-	COQ3	6	99359401	99429218	186	24	10164	-0.91413	0.81967
+	ENPP1	6	131773016	131905155	321	42	10164	-0.91476	0.81984
+	SLC25A44	1	156158932	156222796	147	14	10164	-0.91484	0.81986
+	DDX27	20	49184295	49254077	201	16	10164	-0.91524	0.81997
-	PARP12	7	140013744	140098828	155	18	10164	-0.9154	0.82001
+	STEAP1	7	90119375	90174827	160	16	10164	-0.91557	0.82005
-	UBA6	4	67605761	67736171	191	10	10164	-0.91574	0.8201
-	OR8B12	11	124532722	124578654	186	20	10164	-0.91586	0.82013
+	NUP35	2	183082490	183171684	245	11	10164	-0.91604	0.82018
+	CEP68	2	65021361	65097008	123	9	10164	-0.9163	0.82024
-	RBFOX2	22	35728736	36063892	352	16	10164	-0.91626	0.82024
+	DRC1	2	26366916	26466713	174	20	10164	-0.91639	0.82027
-	DOCK4	7	111716108	112241407	1117	108	10164	-0.91645	0.82028
-	RAB6A	11	73665638	73796156	285	23	10164	-0.91655	0.82031
-	OR7G1	19	9104828	9150763	152	18	10164	-0.91672	0.82035
-	STK17B	2	196123583	196211503	301	20	10164	-0.91706	0.82044
+	CABP1	12	120605619	120677324	225	29	10164	-0.91732	0.82051
-	CNRIP1	2	68274171	68355051	208	24	10164	-0.91735	0.82052
+	GPR39	2	132381161	132656596	368	41	10164	-0.91773	0.82062
-	PTP4A2	1	31896421	31973387	49	6	10164	-0.91791	0.82067
+	KLHL18	3	47247840	47356816	142	24	10164	-0.91795	0.82068
+	MSGN1	2	17781519	17827100	224	16	10164	-0.91843	0.8208
-	CUZD1	10	122822155	122881175	132	13	10164	-0.91864	0.82086
-	ERCC6-PGBI	10	49505105	49574538	198	19	10164	-0.91891	0.82093

STRAND	SYMBOL	CHR	START	STOP	NSNPS	NPARAM	N	ZSTAT	P
-	UBR1	15	42932897	43141088	293	26	10164	-0.91906	0.82097
-	HS3ST5	6	114045586	114377433	767	88	10164	-0.91946	0.82107
-	TASP1	20	13379389	13673936	747	22	10164	-0.91952	0.82109
-	PSRC1	1	109269554	109318168	87	16	10164	-0.91959	0.82111
+	ANO6	12	45180987	45450404	676	23	10164	-0.91965	0.82112
+	S1PR1	1	101201749	101251520	157	29	10164	-0.9197	0.82113
+	NBEA	13	34907287	35682737	1889	63	10164	-0.91981	0.82116
-	CBX1	17	48060052	48136521	291	26	10164	-0.91987	0.82118
-	LOC1053711	16	668507	715527	140	20	10164	-0.91989	0.82118
-	NARR	17	28705993	28752890	54	10	10164	-0.91989	0.82119
+	HSBP1	16	83772903	83823002	261	29	10164	-0.92034	0.8213
-	LOC1027244	1	247555639	247676191	397	24	10164	-0.92091	0.82145
+	BZW2	7	16611134	16716523	357	42	10164	-0.92118	0.82152
+	DMBX1	1	46454837	46524226	265	39	10164	-0.92118	0.82152
+	KIF4B	5	154978700	155028125	218	19	10164	-0.92119	0.82152
-	TTC37	5	95453895	95590005	292	15	10164	-0.92142	0.82158
-	COA1	7	43598452	43764541	492	21	10164	-0.92147	0.8216
+	ZC3H14	14	88527909	88623509	165	18	10164	-0.92172	0.82166
+	MTFR1L	1	25784906	25842942	20	3	10164	-0.92182	0.82169
+	RPLP1	15	69417820	69465545	56	9	10164	-0.92187	0.8217
-	IFRD2	3	50277732	50327595	76	9	10164	-0.92217	0.82178
+	GSG2	17	3688903	3736699	178	8	10164	-0.92232	0.82182
-	VWDE	7	12320883	12439226	670	36	10164	-0.92238	0.82183
+	LOC1053719	17	59990994	60050475	26	2	10164	-0.92244	0.82185
+	CD276	15	73649281	73724518	184	21	10164	-0.92262	0.8219
-	LKAAEAR1	20	64073376	64120144	82	13	10164	-0.92302	0.822
-	TLCD2	17	1692790	1745368	133	19	10164	-0.92315	0.82204
+	ZBTB16	11	114024579	114260676	740	94	10164	-0.92319	0.82205
+	KCNJ16	17	70040225	70145608	250	23	10164	-0.92327	0.82207
+	SYPL2	1	109431478	109492142	85	3	10164	-0.92365	0.82216
-	RNF219	13	78604286	78694179	205	21	10164	-0.9237	0.82218
-	POU1F1	3	87249633	87311587	288	20	10164	-0.92375	0.82219
+	WDR48	3	39016986	39106671	246	24	10164	-0.92405	0.82227
+	OR5D18	11	55783023	55830591	164	18	10164	-0.92433	0.82234
+	MTNR1B	11	92934623	92996240	119	11	10164	-0.92455	0.8224
+	YWHAB	20	44850599	44918532	155	8	10164	-0.92469	0.82244
-	SBDS	7	66977703	67030601	168	26	10164	-0.92484	0.82248
-	ACTL6B	7	100633097	100691461	95	16	10164	-0.92516	0.82256
-	PDCD1	2	241839881	241893908	206	12	10164	-0.92543	0.82263
+	RHBDL3	17	32231131	32334661	162	21	10164	-0.92567	0.82269
-	NAT6	3	50286402	50334421	73	9	10164	-0.92581	0.82273
+	MEP1A	6	46758357	46855987	229	23	10164	-0.92597	0.82277
-	MIS18A	21	32258219	32314065	115	25	10164	-0.92611	0.82281
+	KLC2	11	66222294	66277861	102	20	10164	-0.9262	0.82283
+	C9orf170	9	87113644	87169726	192	17	10164	-0.92623	0.82284
-	LOC1053742	3	195637421	195688263	6	2	10164	-0.92654	0.82292
-	ADH1A	4	99266366	99326028	118	11	10164	-0.92663	0.82294
+	SMARCAL1	2	216377414	216493053	418	25	10164	-0.92684	0.823
-	EPHA1	7	143380782	143443892	183	36	10164	-0.927	0.82304
+	BCAS3	17	60642811	61402838	1198	50	10164	-0.9272	0.82309
-	SSC4D	7	76379329	76444695	142	12	10164	-0.92738	0.82314
-	MNS1	15	56418731	56500137	199	28	10164	-0.92748	0.82316
+	PTP4A3	8	141356993	141442454	230	29	10164	-0.92756	0.82318
-	IFT140	16	1500427	1647108	488	27	10164	-0.92757	0.82319
+	MAB21L2	4	150546925	150594693	104	16	10164	-0.92759	0.82319
-	EZH2	7	148797372	148919349	145	13	10164	-0.92778	0.82324
+	RDH5	12	55685367	55734742	83	13	10164	-0.92783	0.82325
+	ARMC12	6	35698805	35758913	261	49	10164	-0.92784	0.82325
-	BRF1	14	105199286	105350577	130	23	10164	-0.92791	0.82327
-	PLIN4	19	4492180	4553541	31	4	10164	-0.9281	0.82332
+	EDC4	16	67838023	67894514	64	9	10164	-0.9282	0.82335
+	YKT6	7	44165968	44224294	149	18	10164	-0.92824	0.82336
+	CETP	16	56926923	56993844	291	55	10164	-0.92836	0.82339

STRAND	SYMBOL	CHR	START	STOP	NSNPS	NPARAM	N	ZSTAT	P
+	BOLA1	1	149864602	149910795	18	10	10164	-0.92844	0.82341
-	CENPL	1	173789550	173859639	86	13	10164	-0.92842	0.82341
-	FNIP1	5	131631714	131832063	304	19	10164	-0.92845	0.82341
-	CTBS	1	84543121	84609480	198	21	10164	-0.92847	0.82342
-	KRT74	12	52555722	52609092	293	33	10164	-0.92879	0.8235
-	VAV3	1	107561160	107999923	1363	82	10164	-0.92884	0.82351
+	CARM1	19	10836577	10932775	167	19	10164	-0.92885	0.82352
-	CXCL12	10	44360153	44420097	320	39	10164	-0.92916	0.8236
+	SMR3B	4	70348078	70400244	218	14	10164	-0.92947	0.82368
-	TBC1D9	4	140610782	140791317	376	29	10164	-0.9296	0.82371
-	FSTL4	5	133184827	133877066	2173	189	10164	-0.92974	0.82375
+	EHBP1	2	62638851	63056487	779	65	10164	-0.92984	0.82377
-	KRT3	12	52779685	52840717	260	64	10164	-0.93004	0.82383
-	KCNN3	1	154687462	154905278	500	89	10164	-0.93036	0.82391
+	TMEM14B	6	10712759	10769774	153	25	10164	-0.9304	0.82392
-	DUS1L	17	82047872	82100821	80	13	10164	-0.93056	0.82396
+	ASNA1	19	12702492	12758323	14	6	10164	-0.93069	0.82399
-	C5orf28	5	43434252	43520178	188	13	10164	-0.93068	0.82399
+	SFR1	10	104087058	104136385	88	9	10164	-0.93069	0.82399
+	BPIFA1	20	33200996	33253309	98	13	10164	-0.93071	0.824
+	CD1C	1	158254773	158304774	69	11	10164	-0.93078	0.82402
+	APPL1	3	57192737	57283471	128	22	10164	-0.93081	0.82402
-	SLC35G3	17	35182520	35229393	217	33	10164	-0.93081	0.82402
-	SAXO1	9	18917660	19084354	763	32	10164	-0.93098	0.82407
+	EPDR1	7	37885561	37961941	336	35	10164	-0.93103	0.82408
+	INPP5D	2	233024967	233217903	752	66	10164	-0.93111	0.8241
+	SRR	17	2268371	2335264	160	12	10164	-0.93132	0.82416
-	ZNF790	19	36808322	36885787	237	24	10164	-0.93142	0.82418
-	SLC18B1	6	132759368	132839931	280	16	10164	-0.93176	0.82427
+	PTMS	12	6731139	6780952	111	10	10164	-0.93184	0.82429
-	IL9	5	135882246	135930827	120	18	10164	-0.932	0.82433
+	KHDRBS1	1	31978694	32070859	81	8	10164	-0.93198	0.82433
+	PLEKHG4B	5	19015	199972	464	26	10164	-0.93222	0.82439
+	LOC1019291	17	57953110	57999295	16	6	10164	-0.93263	0.82449
+	SLC35G6	17	7446402	7493064	166	19	10164	-0.93274	0.82452
-	LOC1053724	19	57254033	57315483	206	25	10164	-0.93279	0.82454
-	SLC7A13	8	86191763	86265380	241	25	10164	-0.93301	0.82459
+	SLC30A7	1	100861073	101006913	70	20	10164	-0.93319	0.82464
-	KRT77	12	52679612	52738534	280	42	10164	-0.93319	0.82464
+	CALR	19	12903600	12954490	52	6	10164	-0.93334	0.82468
+	SP100	2	230381156	230555602	627	42	10164	-0.93335	0.82468
+	C11orf54	11	93706594	93773102	183	12	10164	-0.93334	0.82468
+	CNOT3	19	54102689	54165708	99	13	10164	-0.93389	0.82482
+	DMRT2	9	968242	1067554	305	29	10164	-0.93391	0.82482
+	MYH14	19	50153186	50320544	303	48	10164	-0.93416	0.82489
-	ANKRA2	5	73542200	73600705	150	11	10164	-0.93449	0.82497
-	ELP6	3	47485640	47548722	95	18	10164	-0.9345	0.82498
-	LOC1053776	3	38940448	39087002	407	22	10164	-0.93501	0.82511
-	CALR3	19	16469057	16531192	135	11	10164	-0.93523	0.82516
-	OPRK1	8	53215716	53286697	287	18	10164	-0.93524	0.82517
+	CTCF	16	67527407	67649185	111	15	10164	-0.93585	0.82533
+	TMED3	15	79276062	79394721	487	44	10164	-0.93632	0.82545
-	USP28	11	113787875	113910570	296	21	10164	-0.93644	0.82548
-	POU4F1	13	78589092	78638560	97	9	10164	-0.93662	0.82552
+	AKR1E2	10	4789560	4864131	484	24	10164	-0.93671	0.82555
-	FGF8	10	101760130	101815369	62	22	10164	-0.93677	0.82556
-	TMEM168	7	112752382	112825713	89	5	10164	-0.93751	0.82575
-	PTGDR2	11	60840925	60890971	94	14	10164	-0.93773	0.82581
-	LOC1053780	6	159041766	159156467	408	27	10164	-0.93775	0.82581
+	PNKD	2	218235392	218356793	276	18	10164	-0.93779	0.82582
+	RET	10	43042027	43140351	334	24	10164	-0.93801	0.82588
-	GBA	1	155224448	155279862	80	9	10164	-0.93842	0.82599
-	GPR37	7	124735601	124801025	92	13	10164	-0.93856	0.82602

STRAND	SYMBOL	CHR	START	STOP	NSNPS	NPARAM	N	ZSTAT	P
-	PRUNE2	9	76601376	76941220	924	65	10164	-0.93858	0.82603
-	IRF2	4	184377713	184509580	223	51	10164	-0.93867	0.82605
-	RPN1	3	128609970	128685876	247	20	10164	-0.93934	0.82622
-	ZNF347	19	53128300	53198938	171	23	10164	-0.93943	0.82624
+	OR51I2	11	5418408	5464477	288	33	10164	-0.93961	0.82629
+	GLT1D1	12	128818440	128994968	543	68	10164	-0.93989	0.82636
-	TYROBP	19	35894401	35943309	92	12	10164	-0.94038	0.82649
-	FXR2	17	7581230	7649897	217	22	10164	-0.94044	0.82651
+	SP1	12	53345195	53426446	121	9	10164	-0.94049	0.82652
+	SNX11	17	48072554	48132745	230	22	10164	-0.94051	0.82652
+	LOC729468	2	113506191	113580962	50	11	10164	-0.94078	0.82659
-	FILIP1L	3	99823144	100149513	483	30	10164	-0.94094	0.82663
+	TRIM24	7	138425216	138595588	272	14	10164	-0.94115	0.82669
-	GPM6A	4	175622937	176037691	824	89	10164	-0.94144	0.82676
-	ZW10	11	113723183	113808763	222	28	10164	-0.94149	0.82677
-	IRF6	1	209775623	209841175	228	17	10164	-0.94162	0.82681
+	ZNF664-FAM	12	123938215	124326024	1051	82	10164	-0.94211	0.82693
-	CBX8	17	79784377	79832116	113	9	10164	-0.94231	0.82698
-	SI	3	164968893	165113495	391	18	10164	-0.94246	0.82702
+	BRPF3	6	36161773	36242790	195	39	10164	-0.94255	0.82704
-	SLC22A8	11	62982824	63050845	182	35	10164	-0.94273	0.82709
+	LPP	3	188117143	188900671	1546	166	10164	-0.94325	0.82722
-	GAB2	11	78205290	78452822	485	12	10164	-0.94349	0.82728
-	LPCAT1	5	1451427	1560730	401	59	10164	-0.94413	0.82745
-	DENND2C	1	114574575	114705111	186	18	10164	-0.9445	0.82754
-	DACH1	13	71427966	71904113	933	68	10164	-0.94452	0.82755
+	SMC5	9	70223962	70364873	457	22	10164	-0.94471	0.8276
+	SLCO1B1	12	21096194	21249796	634	31	10164	-0.94481	0.82762
-	MKRN1	7	140443040	140514569	64	8	10164	-0.94496	0.82766
+	G2E3	14	30524123	30629840	221	28	10164	-0.94499	0.82767
-	USP36	17	78777385	78875898	192	29	10164	-0.94505	0.82768
+	UBIAD1	1	11238198	11306595	144	13	10164	-0.94517	0.82771
+	OR5L2	11	55792219	55838154	163	17	10164	-0.94519	0.82772
+	GALNT16	14	69224546	69389904	475	56	10164	-0.9452	0.82772
+	C10orf11	10	75396458	76567375	2633	177	10164	-0.9454	0.82777
-	PPP1R13L	19	45369634	45441349	180	29	10164	-0.9459	0.8279
+	TOM1	22	35264275	35357994	100	18	10164	-0.94622	0.82798
+	BCAP29	7	107544977	107633317	137	12	10164	-0.94642	0.82803
-	VAT1	17	43004605	43057442	94	10	10164	-0.94657	0.82807
+	TTC27	2	32593020	32831051	596	36	10164	-0.94661	0.82808
-	WDR77	1	111429890	111484208	119	17	10164	-0.9474	0.82828
-	KCTD14	11	78005715	78081191	287	22	10164	-0.94756	0.82832
-	HDAC3	5	141610876	141671856	162	31	10164	-0.94795	0.82842
-	MCL1	1	150564551	150614738	108	17	10164	-0.94823	0.82849
+	IGLON5	19	51276848	51340848	179	25	10164	-0.94821	0.82849
-	DPP4	2	161982241	162109542	301	36	10164	-0.94828	0.82851
-	ANKMY2	7	16589776	16680817	308	36	10164	-0.94832	0.82852
+	PARVG	22	44135227	44218469	178	25	10164	-0.94835	0.82852
-	MYEF2	15	48129428	48213400	22	7	10164	-0.94841	0.82854
-	SURF1	9	133341805	133391485	104	9	10164	-0.94866	0.8286
+	HS3ST3B1	17	14266050	14359406	111	18	10164	-0.94891	0.82867
+	CHAF1B	21	36342057	36426827	87	13	10164	-0.94955	0.82883
+	TAAR9	6	132503288	132549336	161	21	10164	-0.94964	0.82885
-	FAM49B	8	129829593	130051697	660	26	10164	-0.94986	0.82891
+	TOR1B	9	129768148	129821284	204	16	10164	-0.94999	0.82894
-	PPA2	4	105359077	105509070	511	22	10164	-0.95023	0.829
+	LOC1053691	21	16006292	16650637	799	66	10164	-0.95028	0.82902
+	ITM2C	2	230829598	230889254	143	23	10164	-0.95035	0.82903
-	RTN1	14	59585976	59905839	832	41	10164	-0.95039	0.82904
-	C7orf61	7	100446615	100499271	84	11	10164	-0.95043	0.82905
-	CXCL10	4	76011116	76058536	311	8	10164	-0.95125	0.82926
+	CAPN10	2	240551716	240609109	192	23	10164	-0.9517	0.82937
-	IFFO1	12	6529528	6591083	132	18	10164	-0.9519	0.82943

STRAND	SYMBOL	CHR	START	STOP	NSNPS	NPARAM	N	ZSTAT	P
+	VEZT	12	95182746	95312790	444	34	10164	-0.95211	0.82948
-	TEAD2	19	49330595	49397457	195	9	10164	-0.95214	0.82949
+	ACOT2	14	73532620	73585658	107	20	10164	-0.95268	0.82962
+	DNAI2	17	74239232	74324884	315	26	10164	-0.95301	0.82971
-	LRP1B	2	140221427	142167474	5831	250	10164	-0.9532	0.82975
+	ADGRE5	19	14346144	14418725	48	16	10164	-0.95331	0.82978
+	CTXN2	15	48143355	48213754	21	7	10164	-0.95338	0.8298
+	GNB2	7	100638740	100689169	76	13	10164	-0.9535	0.82983
-	GLG1	16	74437427	74642144	418	15	10164	-0.95354	0.82984
+	ATHL1	11	252832	305688	156	22	10164	-0.95365	0.82987
+	CKS2	9	89276195	89326703	69	17	10164	-0.95376	0.8299
+	DTD1	20	18552912	18773917	902	28	10164	-0.95428	0.83003
-	CD74	5	150391637	150447936	156	21	10164	-0.95447	0.83008
-	ZCWPW1	7	100390872	100464414	114	15	10164	-0.95455	0.8301
+	EDDM3B	14	20733427	20780948	342	33	10164	-0.95483	0.83017
+	CNIH2	11	66243201	66294214	95	22	10164	-0.9551	0.83024
-	SS18	18	26006253	26126212	225	14	10164	-0.95523	0.83027
+	LOC1053728	1	207927873	207977460	174	7	10164	-0.95526	0.83028
-	UXS1	2	106083303	106229339	275	19	10164	-0.95543	0.83032
+	SECISBP2	9	89283473	89377117	155	23	10164	-0.95565	0.83037
+	MAGI3	1	113355474	113695923	826	39	10164	-0.95589	0.83044
-	SYT10	12	33365413	33474819	310	16	10164	-0.95591	0.83044
-	HS1BP3	2	20607804	20686104	267	35	10164	-0.95609	0.83049
+	TMEM151A	11	66256902	66306664	106	27	10164	-0.95617	0.83051
+	ITGAM	16	31224967	31342892	213	13	10164	-0.95643	0.83057
-	SAT2	17	7616234	7662876	168	15	10164	-0.95654	0.8306
-	TATDN1	8	124478494	124574088	266	33	10164	-0.9567	0.83064
-	TSC1	9	132881348	132980370	214	25	10164	-0.95677	0.83066
+	ZNF212	7	149204648	149265609	180	28	10164	-0.957	0.83072
+	IL10RA	11	117951391	118011483	171	22	10164	-0.95718	0.83076
+	CDK2	12	55931769	55982789	58	17	10164	-0.9572	0.83077
-	GSG1L	16	27777529	28098509	802	100	10164	-0.95756	0.83086
+	MAL2	8	119173371	119255673	263	29	10164	-0.95838	0.83107
-	CTNNAL1	9	108932569	109053802	301	22	10164	-0.95858	0.83111
-	ACTR3C	7	149877178	150358669	448	25	10164	-0.95875	0.83116
+	CEACAM18	19	51443547	51501261	292	26	10164	-0.95876	0.83116
+	AGO3	1	35888403	36066747	201	15	10164	-0.95897	0.83121
-	AK1	9	127856480	127912743	139	18	10164	-0.95938	0.83132
+	BORA	13	72692749	72766198	204	21	10164	-0.95994	0.83146
-	TMEM131	2	97746336	98030932	646	28	10164	-0.96014	0.83151
-	SYNDIG1L	14	74395893	74472631	188	25	10164	-0.96062	0.83163
+	HSPB8	12	119143790	119204746	163	27	10164	-0.96065	0.83164
-	ZNF438	10	30834634	31067407	827	31	10164	-0.96072	0.83165
+	PAX7	1	18596006	18758866	176	25	10164	-0.96073	0.83166
-	AADAT	4	170050222	170126699	92	11	10164	-0.96119	0.83177
+	PODN	1	53027052	53095502	394	24	10164	-0.96143	0.83183
-	THAP10	15	70871342	70927433	57	11	10164	-0.96151	0.83185
-	OR51B4	11	5291014	5336946	250	27	10164	-0.9615	0.83185
+	BCL2L13	22	17593855	17740855	604	26	10164	-0.96176	0.83191
-	MAP3K5	6	136547046	136828051	416	42	10164	-0.96194	0.83196
-	TUBAL3	10	5383098	5439830	228	23	10164	-0.96249	0.8321
-	CTBP1	4	1201440	1285248	273	27	10164	-0.96285	0.83219
+	PSMB3	17	38717713	38774231	104	21	10164	-0.96316	0.83227
+	C19orf52	19	10893748	10940241	95	14	10164	-0.96318	0.83227
-	RRBP1	20	17603678	17717283	183	26	10164	-0.96327	0.83229
+	IL32	16	3030312	3079667	46	7	10164	-0.96328	0.8323
-	PRSS27	16	2702418	2755578	3	1	10164	-0.96345	0.83234
-	IFI6	1	27656061	27707213	86	3	10164	-0.96348	0.83235
+	GCKR	2	27461839	27533689	146	19	10164	-0.96349	0.83235
-	RAB34	17	28704281	28753268	56	10	10164	-0.9636	0.83238
+	PAK4	19	39090780	39189406	282	28	10164	-0.96368	0.8324
-	ANKRD11	16	89257619	89525574	810	51	10164	-0.96394	0.83246
+	ARPC4-TTLL	3	9757548	9846356	292	33	10164	-0.96395	0.83246

STRAND	SYMBOL	CHR	START	STOP	NSNPS	NPARAM	N	ZSTAT	P
-	LSM4	19	18296230	18358191	153	23	10164	-0.96416	0.83252
+	C19orf44	19	16461311	16531369	145	11	10164	-0.96446	0.83259
+	HEBP2	6	138369199	138431545	132	4	10164	-0.96522	0.83278
+	GIPR	19	45633193	45692504	240	27	10164	-0.9656	0.83288
+	ACAT1	11	108086531	108158168	176	29	10164	-0.9657	0.8329
-	C14orf1	14	75640890	75696195	107	14	10164	-0.96597	0.83297
-	LMBR1L	12	49087139	49145900	110	12	10164	-0.96596	0.83297
-	C7orf43	7	100144420	100193721	89	14	10164	-0.96612	0.83301
-	ISPD	7	15906851	16565558	2219	93	10164	-0.96635	0.83306
-	MRPS28	8	79908860	80065271	200	24	10164	-0.96642	0.83308
+	HGH1	8	144102769	144150843	19	1	10164	-0.96657	0.83312
+	C5orf24	5	134810680	134869735	100	13	10164	-0.9667	0.83315
-	SOX5	12	23519495	24597669	2977	256	10164	-0.96702	0.83323
+	TMPO	12	98480573	98560379	254	30	10164	-0.96703	0.83324
+	BTN2A2	6	26347886	26404874	351	29	10164	-0.9673	0.8333
-	SSTR3	22	37194237	37255447	225	26	10164	-0.96732	0.83331
-	LIN54	4	82914603	83047941	1	1	10164	-0.96752	0.83336
-	HSPH1	13	31124974	31198109	219	22	10164	-0.96854	0.83361
+	CEP78	9	78201062	78281883	153	25	10164	-0.96894	0.83371
+	CORO1A	16	30148410	30199076	27	2	10164	-0.96896	0.83372
-	WDR4	21	42833080	42914589	284	13	10164	-0.96904	0.83374
+	CDHR2	5	176507511	176605768	395	40	10164	-0.96935	0.83382
+	USE1	19	17180346	17229829	255	29	10164	-0.97001	0.83398
+	USP21	1	161124464	161175726	61	7	10164	-0.97038	0.83407
+	GNAI1	7	80099824	80229409	301	34	10164	-0.97053	0.83411
+	CSRP2BP	20	18107224	18198387	253	28	10164	-0.9711	0.83425
-	KRTAP4-8	17	41086981	41133123	306	7	10164	-0.97108	0.83425
+	CCT6A	7	56016685	56073989	165	24	10164	-0.97113	0.83426
+	LOC1053756	8	101179031	101331227	301	27	10164	-0.97125	0.83429
-	HYAL1	3	50289889	50347419	91	10	10164	-0.97131	0.8343
+	VIM	10	17192935	17247593	188	23	10164	-0.97137	0.83432
-	TNS4	17	40465828	40536681	68	16	10164	-0.97136	0.83432
-	A2M	12	9057708	9150962	188	14	10164	-0.97149	0.83435
+	TAL2	9	105627457	105673112	94	14	10164	-0.9716	0.83438
-	MRGPRG	11	3207944	3253813	170	23	10164	-0.9717	0.8344
-	DNMBP	10	99865576	100044948	572	28	10164	-0.97191	0.83445
+	DKKL1	19	49325495	49385119	159	8	10164	-0.97196	0.83447
+	ERP29	12	111978348	112033220	99	9	10164	-0.97205	0.83449
-	PDAP1	7	99384673	99443682	113	17	10164	-0.97245	0.83459
+	SYDE1	19	15072364	15124988	113	17	10164	-0.97273	0.83466
+	PFDN5	12	53260451	53309450	80	10	10164	-0.97299	0.83472
+	SLC25A26	3	66098610	66390021	947	57	10164	-0.97301	0.83473
+	AMPD2	1	109584659	109642055	118	19	10164	-0.97318	0.83477
-	UBE2J2	1	1243912	1308854	118	13	10164	-0.97324	0.83478
+	AQP2	12	49915741	49968881	156	41	10164	-0.97328	0.83479
+	OR52N4	11	5687259	5765958	379	26	10164	-0.97329	0.8348
+	ZNF518A	10	96094200	96173793	118	9	10164	-0.97342	0.83483
-	SPTLC1	9	92021134	92150474	398	26	10164	-0.97358	0.83487
+	PRR27	4	70119187	70176609	187	21	10164	-0.97371	0.8349
+	THBS4	5	79956311	80093637	343	19	10164	-0.97378	0.83492
+	MT1A	16	56603666	56650087	206	36	10164	-0.97386	0.83494
-	EMC8	16	85768624	85834555	194	23	10164	-0.97387	0.83494
-	KIF13B	8	29057278	29298304	481	29	10164	-0.97395	0.83496
-	PCGF2	17	38723897	38784817	116	24	10164	-0.97418	0.83502
-	CAMK2G	10	73802501	73909591	229	30	10164	-0.97463	0.83513
-	PYCR2	1	225909877	225959340	105	24	10164	-0.9747	0.83515
-	RPLP0	12	120186700	120236211	58	11	10164	-0.97497	0.83521
+	BMP1	8	22130140	22222326	88	17	10164	-0.97553	0.83535
+	DOLPP1	9	129046104	129100438	51	8	10164	-0.97566	0.83538
-	FCGR3A	1	161531759	161585623	55	9	10164	-0.97569	0.83539
+	KCMF1	2	84936108	85069472	181	16	10164	-0.97568	0.83539
+	CLDN12	7	90368334	90425954	155	22	10164	-0.97629	0.83554
-	CCDC13	3	42694118	42808253	258	25	10164	-0.97633	0.83555

STRAND	SYMBOL	CHR	START	STOP	NSNPS	NPARAM	N	ZSTAT	P
-	RGS19	20	64063181	64115003	95	14	10164	-0.97646	0.83558
+	ZNF691	1	42811573	42862477	85	17	10164	-0.97665	0.83563
+	BAG3	10	119616347	119687819	285	47	10164	-0.97701	0.83572
+	INSIG2	2	118053474	118120027	151	24	10164	-0.97734	0.8358
+	SPEG	2	219399192	219503632	133	20	10164	-0.97771	0.83589
+	SPRR1A	1	152949088	152995814	194	12	10164	-0.97774	0.8359
-	TWF1	12	43783723	43841375	106	12	10164	-0.97777	0.83591
-	MAFB	20	40675848	40724240	110	16	10164	-0.97795	0.83595
+	CMTM5	14	23341808	23389772	112	21	10164	-0.97798	0.83596
+	ACMSD	2	134803348	134912076	254	29	10164	-0.978	0.83596
+	TXLNA	1	32144695	32208285	71	7	10164	-0.97802	0.83597
+	LOC1005062	12	12076002	12121688	215	37	10164	-0.97808	0.83598
+	IFIT1	10	89357546	89416487	196	25	10164	-0.97837	0.83605
+	SYN2	3	11969360	12202032	565	27	10164	-0.97844	0.83607
+	TRIM51	11	55848297	55901810	203	15	10164	-0.97857	0.8361
-	CXCR1	2	218152845	218201993	107	19	10164	-0.97859	0.83611
+	LOC1053769	19	55570753	55620280	123	13	10164	-0.97897	0.8362
+	C16orf86	16	67631806	67678758	52	14	10164	-0.97913	0.83624
+	PROKR1	2	68610822	68665576	93	16	10164	-0.97918	0.83625
-	CHD6	20	41392101	41653494	694	24	10164	-0.97918	0.83625
-	CSRP1	1	201473530	201542259	248	20	10164	-0.9797	0.83638
-	HAND2	4	173516501	173565227	101	15	10164	-0.9798	0.83641
-	VSX1	20	25060885	25117379	88	7	10164	-0.97992	0.83644
+	POR	7	75880102	75996855	146	25	10164	-0.98009	0.83648
-	PLEKHA6	1	204208851	204412763	520	40	10164	-0.98008	0.83648
+	RPS9	19	54165557	54217647	90	14	10164	-0.98019	0.8365
+	KCNH6	17	63488334	63558977	112	13	10164	-0.98021	0.83651
+	AGK	7	141516278	141664409	210	13	10164	-0.98033	0.83654
-	PPP1R42	8	66978131	67063551	123	8	10164	-0.98036	0.83654
+	EMP1	12	13161668	13226774	157	30	10164	-0.98064	0.83661
-	LRBA	4	150254659	151050727	1795	51	10164	-0.98118	0.83675
+	FOSB	19	45432995	45485179	170	27	10164	-0.98132	0.83678
+	GDF7	2	20631664	20681490	117	31	10164	-0.98132	0.83678
-	LOC1053739	2	118067311	118123892	128	22	10164	-0.98158	0.83685
+	ARL8B	3	5087245	5190916	283	25	10164	-0.98174	0.83689
+	DLK1	14	100691865	100745130	213	37	10164	-0.98179	0.8369
+	RAD23A	19	12910814	12963643	50	6	10164	-0.98195	0.83694
+	TECTA	11	121067666	121200806	229	19	10164	-0.98199	0.83695
+	TRPM8	2	233882362	234029522	636	64	10164	-0.98263	0.83711
+	FOXD3	1	63288059	63335126	114	26	10164	-0.98271	0.83712
-	MTSS1L	16	70651204	70721051	127	15	10164	-0.98277	0.83714
+	LOC1053749	6	7877481	7926025	133	17	10164	-0.98323	0.83725
+	ZNF654	3	88104112	88154664	165	16	10164	-0.9833	0.83727
-	MTA2	11	62583203	62636840	51	8	10164	-0.98339	0.83729
-	BCAS2	1	114557557	114616644	67	10	10164	-0.98366	0.83736
-	MED1	17	39394285	39486280	177	11	10164	-0.98369	0.83737
+	CGRRF1	14	54474869	54549309	145	9	10164	-0.98383	0.8374
+	AQP5	12	49926496	49975682	124	37	10164	-0.98422	0.8375
-	RHOBTB1	10	60859438	61036468	428	42	10164	-0.98456	0.83758
-	CREBZF	11	85647564	85700138	101	17	10164	-0.98513	0.83772
+	SHISA3	4	42362839	42412487	211	31	10164	-0.98515	0.83772
-	GFPT2	5	180290698	180388336	561	56	10164	-0.9854	0.83779
-	KDELR2	7	6451081	6519218	189	24	10164	-0.98554	0.83782
+	ATP6V0C	16	2478726	2530223	7	3	10164	-0.98562	0.83784
-	TTLL11	9	121805674	122128606	1322	107	10164	-0.98575	0.83787
+	C19orf68	19	48135662	48207621	310	23	10164	-0.98584	0.83789
+	RBM11	21	14181106	14238372	289	27	10164	-0.986	0.83793
+	LAMTOR4	7	100113907	100164210	88	21	10164	-0.98616	0.83797
-	LHX3	9	136186250	136240109	105	15	10164	-0.98663	0.83809
-	ITGA7	12	55674568	55751037	116	19	10164	-0.98668	0.8381
+	VSTM2B	19	29490431	29574551	358	39	10164	-0.98667	0.8381
+	NIM1K	5	43157054	43290850	332	23	10164	-0.98678	0.83812
+	OOSP2	11	60005275	60058044	115	14	10164	-0.98679	0.83813

STRAND	SYMBOL	CHR	START	STOP	NSNPS	NPARAM	N	ZSTAT	P
+	RPL23A	17	28684982	28734356	53	8	10164	-0.98713	0.83821
+	LRRC72	7	16491827	16591554	318	34	10164	-0.9876	0.83833
-	UCP2	11	73964671	74017844	116	15	10164	-0.98771	0.83835
+	PTGFRN	1	116874923	117000358	273	22	10164	-0.98807	0.83844
-	CNTN3	3	74252568	74649445	913	85	10164	-0.98814	0.83846
-	MED4	13	48065728	48130141	294	20	10164	-0.98822	0.83848
-	OR2AG2	11	6757345	6961426	892	31	10164	-0.98827	0.83849
-	CCDC149	4	24796117	25015204	518	61	10164	-0.98847	0.83854
+	C14orf119	14	23060474	23110456	147	20	10164	-0.98864	0.83858
-	OR2A7	7	144248696	144294628	8	3	10164	-0.98932	0.83875
-	OR2A42	7	144221911	144267843	8	3	10164	-0.98932	0.83875
-	DHX37	12	124936824	125024131	332	30	10164	-0.99069	0.83908
-	LARGE	22	33263076	33957841	1910	160	10164	-0.99075	0.8391
-	OR6N2	1	158766682	158812635	122	21	10164	-0.99102	0.83916
+	PDLIM4	5	132222658	132283454	246	28	10164	-0.99108	0.83918
-	DPP7	9	137100540	137152980	98	20	10164	-0.99108	0.83918
+	LOC1053732	1	239351518	239397227	93	13	10164	-0.99109	0.83918
-	TMEM74	8	108773117	108822541	95	11	10164	-0.99128	0.83923
-	FCHSD1	5	141629302	141686428	160	33	10164	-0.99136	0.83925
+	TRUB1	10	114903193	114987680	186	17	10164	-0.99149	0.83928
+	BHMT2	5	79034724	79100074	217	15	10164	-0.99157	0.8393
-	TMEM261	9	7786490	7834817	139	24	10164	-0.9916	0.8393
+	DNAJC3	13	95642139	95804989	240	33	10164	-0.99163	0.83931
-	NKX6-3	8	41636310	41682359	147	19	10164	-0.99176	0.83934
+	ZNF302	19	34642639	34696397	211	20	10164	-0.99185	0.83937
+	LOC1053733	2	600637	656098	375	14	10164	-0.99214	0.83943
+	IL36G	2	112937329	112995672	181	16	10164	-0.99241	0.8395
-	ZC3H7A	16	11740586	11832258	266	27	10164	-0.99246	0.83951
-	LEFTY2	1	225926598	225976383	111	33	10164	-0.99258	0.83954
+	LOC1053719	17	77218764	77280801	106	14	10164	-0.99273	0.83958
+	HPN	19	35005506	35076577	195	24	10164	-0.99285	0.83961
-	DCTD	4	182880087	182952477	214	20	10164	-0.99291	0.83962
+	FBXO24	7	100551333	100611117	69	21	10164	-0.99309	0.83967
-	IGFL3	19	46109863	46159705	89	12	10164	-0.99319	0.83969
+	LMO7DN	13	75835571	75893812	211	22	10164	-0.99353	0.83977
-	ARL6IP1	16	18781669	18836535	69	4	10164	-0.9936	0.83979
+	CX3CL1	16	57337461	57395048	91	15	10164	-0.99382	0.83985
-	NMBR	6	142064484	142182118	295	17	10164	-0.99445	0.84
+	HCK	20	32017188	32111856	178	23	10164	-0.9945	0.84001
-	CLRN3	10	127867841	127927947	186	35	10164	-0.99494	0.84012
-	HYAL3	3	50282828	50334468	79	9	10164	-0.99508	0.84015
+	SAMD5	6	147473648	147747541	1029	87	10164	-0.99521	0.84018
+	CCDC40	17	80001632	80110613	438	28	10164	-0.99565	0.84029
-	LOC1009965	9	133048029	133096567	131	21	10164	-0.99564	0.84029
+	NCDN	1	35522792	35576779	66	10	10164	-0.99583	0.84033
-	SGK494	17	28597964	28649193	46	17	10164	-0.99587	0.84034
+	KCNC1	11	17700910	17874288	309	45	10164	-0.99594	0.84036
+	MLYCD	16	83864125	83926182	304	26	10164	-0.99637	0.84046
-	FAM153A	5	177712380	177818398	50	5	10164	-0.99658	0.84052
+	SUV420H2	19	55304210	55358121	141	23	10164	-0.99675	0.84056
+	MRPL22	5	154906073	154979411	197	21	10164	-0.9972	0.84067
+	CTAGE4	7	144148455	144196080	69	5	10164	-0.99735	0.8407
-	ZC3HAV1	7	139033520	139144720	166	18	10164	-0.99767	0.84078
-	VPS4B	18	63379190	63457519	195	15	10164	-0.99771	0.84079
+	OR5D14	11	55760556	55806500	213	13	10164	-0.99786	0.84083
-	LOC1027236	10	96820632	96867393	65	7	10164	-0.99802	0.84086
+	TMEM132C	12	128232403	128717915	657	83	10164	-0.99815	0.8409
-	ZSCAN29	15	43348172	43405648	134	14	10164	-0.99819	0.84091
+	SOX14	3	137729292	137776334	76	10	10164	-0.99838	0.84095
+	RPS12	6	132779569	132827564	168	12	10164	-0.99854	0.84099
+	BAG4	8	38141588	38223301	118	12	10164	-0.99912	0.84113
+	SLC44A3	1	94785342	94905247	394	51	10164	-0.99933	0.84118
-	PFDN2	1	161090556	161153076	79	9	10164	-0.99937	0.84119

STRAND	SYMBOL	CHR	START	STOP	NSNPS	NPARAM	N	ZSTAT	P
+	SLC37A1	21	42464632	42591440	401	35	10164	-0.9994	0.8412
-	BIRC8	19	53279601	53326622	146	26	10164	-0.99984	0.84131
-	ARL17B	17	46264184	46397064	5	2	10164	-1.0002	0.84139
+	FAM228A	2	24140043	24201698	190	16	10164	-1.0004	0.84143
+	ZBTB37	1	173833082	173897458	142	16	10164	-1.0004	0.84145
-	TBX20	7	35192430	35289100	301	13	10164	-1.0005	0.84146
+	KRTAP4-9	17	41070389	41116488	300	7	10164	-1.0011	0.8416
+	HEXIM2	17	45125026	45180040	121	25	10164	-1.0011	0.84161
+	ATP1A2	1	160080730	160153591	182	23	10164	-1.0012	0.84165
+	OR4C45	11	49997874	50043794	157	12	10164	-1.0014	0.84167
-	SERGEF	11	17778048	18048162	532	45	10164	-1.0015	0.84171
+	LOC1053714	16	89261085	89310962	162	23	10164	-1.0016	0.84173
+	FUS	16	31144704	31204871	27	5	10164	-1.002	0.84182
+	PPFIBP1	12	27489112	27705564	755	77	10164	-1.0021	0.84184
+	ZPBP2	17	39833164	39887896	72	6	10164	-1.0021	0.84185
-	VWA8	13	41556825	41996103	1338	64	10164	-1.0022	0.84187
-	TRDMT1	10	17127802	17237071	373	20	10164	-1.0023	0.84189
+	OSMR	5	38810858	38955579	502	48	10164	-1.0025	0.84195
+	BEND6	6	56919975	57037652	213	15	10164	-1.0029	0.84204
-	ELOVL1	1	43353397	43403074	75	10	10164	-1.0033	0.84214
+	MS4A15	11	60721867	60786733	98	19	10164	-1.0033	0.84214
-	ZNF746	7	149462793	149532837	143	17	10164	-1.0034	0.84216
+	CDK12	17	39426486	39577559	293	12	10164	-1.0036	0.84221
+	AKAP5	14	64430499	64484503	160	17	10164	-1.0038	0.84225
+	IDO1	8	39878809	39938790	180	18	10164	-1.004	0.84232
-	AVPR1A	12	63132287	63188860	182	11	10164	-1.0041	0.84234
+	SEPT3	22	41941927	42008221	177	15	10164	-1.0041	0.84234
+	EI24	11	125534216	125594689	147	19	10164	-1.0043	0.84237
+	TTC9	14	70606787	70685360	257	48	10164	-1.0044	0.8424
-	ZCCHC6	9	86277733	86389501	281	19	10164	-1.0047	0.84247
+	MIIP	1	11984242	12042049	205	12	10164	-1.0049	0.84253
+	CMSS1	3	99782834	100188632	598	28	10164	-1.0049	0.84253
-	GTF2B	1	88842638	88926944	244	15	10164	-1.0049	0.84254
+	SLC36A1	5	151376344	151571961	873	48	10164	-1.0052	0.84259
-	CD163	12	7460816	7538818	206	32	10164	-1.0053	0.84262
-	UQCC2	6	33686761	33746751	364	33	10164	-1.0053	0.84263
+	SERPINF1	17	1726965	1787565	214	30	10164	-1.0057	0.84271
-	ZBTB45	19	58503530	58555823	51	10	10164	-1.0058	0.84275
+	ABHD10	3	111943876	112003368	255	36	10164	-1.0059	0.84277
-	TBC1D8	2	100997228	101230326	687	56	10164	-1.0061	0.84282
-	ERCC2	19	45341391	45405646	168	24	10164	-1.007	0.84303
-	OSBPL10	3	31650825	32016850	1142	112	10164	-1.0071	0.84307
-	ZNF675	19	23642781	23722215	171	20	10164	-1.0072	0.84308
-	SPATA2L	16	89686357	89736713	255	35	10164	-1.0082	0.84332
-	SOHLH2	13	36158208	36249615	298	24	10164	-1.0084	0.84337
+	CNOT10	3	32650145	32783875	403	14	10164	-1.0087	0.84344
-	RGS11	16	258301	310943	253	46	10164	-1.0088	0.84345
-	NKX1-2	10	124437429	124484981	181	33	10164	-1.0089	0.84349
-	CXCL11	4	76023682	76071197	352	9	10164	-1.0092	0.84355
-	FARSA	19	12912470	12968744	52	6	10164	-1.0094	0.8436
+	OR11G2	14	20162336	20208373	62	7	10164	-1.0095	0.84363
+	DYNC1H1	14	101929528	102060798	371	20	10164	-1.0097	0.84369
+	MAPK13	6	36095484	36154524	154	38	10164	-1.01	0.84375
+	POLR1C	6	43482039	43560984	126	11	10164	-1.0102	0.84381
+	LOC1053743	4	7875373	7929635	331	19	10164	-1.0106	0.84388
+	UBA5	3	132619446	132688253	114	20	10164	-1.0106	0.84391
+	PCED1B	12	47044603	47246663	833	77	10164	-1.011	0.84398
+	CHRNB2	1	154532781	154589878	119	15	10164	-1.0112	0.84403
-	HAP1	17	41712639	41769646	108	8	10164	-1.0112	0.84405
-	CREB3L2	7	137864979	138037101	391	47	10164	-1.0113	0.84406
+	EVC	4	5676189	5839045	631	63	10164	-1.0113	0.84407
-	LIG4	13	108197442	108253368	142	17	10164	-1.0114	0.84408
+	PVRIG	7	100181717	100231490	111	8	10164	-1.0117	0.84416

STRAND	SYMBOL	CHR	START	STOP	NSNPS	NPARAM	N	ZSTAT	P
+	AADAC	3	151779025	151838488	241	17	10164	-1.0118	0.84418
-	FAM169A	5	74767574	74901838	244	13	10164	-1.0119	0.84422
-	NOX3	6	155385368	155490903	308	54	10164	-1.0121	0.84425
-	HAUS6	9	19043137	19137942	298	27	10164	-1.0121	0.84426
+	SIPA1	11	65603107	65660930	132	37	10164	-1.0123	0.84431
+	SLC22A20	11	65178840	65252757	181	28	10164	-1.0124	0.84432
+	WBP1	2	74423400	74470891	29	3	10164	-1.013	0.84446
-	ADAMTSL5	19	1495018	1548189	81	11	10164	-1.0134	0.84458
-	NACA2	17	61580433	61626202	95	11	10164	-1.0135	0.84459
-	RNFT1	17	59942362	59999756	50	9	10164	-1.0136	0.84462
-	MBOAT7	19	54163412	54224882	97	16	10164	-1.0137	0.84464
+	MUC19	12	40358394	40580757	1218	40	10164	-1.0141	0.84473
+	KRTAP4-7	17	41049207	41095144	232	12	10164	-1.0147	0.84487
+	ERCC6L2	9	95840618	96130476	762	51	10164	-1.0147	0.84488
-	BMF	15	40077890	40143884	177	25	10164	-1.0148	0.8449
-	C17orf67	17	56781913	56869062	80	3	10164	-1.0149	0.84493
+	COL13A1	10	69766863	69969148	819	120	10164	-1.0152	0.84498
+	CHIA	1	111255852	111330566	407	28	10164	-1.0153	0.84501
-	HPGD	4	174480177	174557898	234	35	10164	-1.0153	0.84502
+	MATR3	5	139238752	139341677	90	9	10164	-1.0154	0.84505
+	KLRG1	12	8954625	9020747	156	9	10164	-1.0155	0.84506
+	SMCR8	17	18280280	18338056	171	16	10164	-1.0156	0.84509
-	KITLG	12	88482793	88615473	304	23	10164	-1.0159	0.84515
-	RNF152	18	61798067	61929432	496	57	10164	-1.0158	0.84515
-	RPL6	12	112395190	112453838	61	18	10164	-1.0161	0.84522
-	RANBP3	19	5906139	6013309	104	12	10164	-1.0162	0.84523
+	PSMD1	2	231021864	231182827	257	24	10164	-1.0164	0.84527
-	SLC10A2	13	103033998	103101846	228	26	10164	-1.0164	0.84527
+	SAMD8	10	75064592	75192123	353	26	10164	-1.0168	0.84538
-	DNPEP	2	219363458	219435017	123	14	10164	-1.0169	0.8454
-	DET1	15	88501482	88581698	242	17	10164	-1.0169	0.8454
+	UTP15	5	73530741	73593377	166	13	10164	-1.0176	0.84555
+	DNAJC5B	8	65980042	66110520	347	23	10164	-1.0175	0.84555
+	FBXL15	10	102384061	102433138	83	21	10164	-1.0176	0.84556
+	BECN2	1	241922767	241969062	224	19	10164	-1.0176	0.84557
-	TAX1BP3	17	3652893	3703679	152	19	10164	-1.0178	0.84562
-	NPTX1	17	80456833	80511604	82	13	10164	-1.0183	0.84573
-	B3GNTL1	17	82932149	83086845	751	52	10164	-1.0184	0.84577
+	MSLN	16	725732	778865	133	23	10164	-1.0186	0.84581
+	EID3	12	104268732	104315205	139	17	10164	-1.0192	0.84593
+	CNTRL	9	121039955	121187610	365	20	10164	-1.0193	0.84596
+	ALG6	1	63332590	63448562	288	33	10164	-1.0193	0.84597
+	PATE3	11	125753111	125801600	114	17	10164	-1.02	0.84613
-	P2RX5	17	3663227	3731404	214	13	10164	-1.0204	0.84623
-	AFF3	2	99535864	100177583	1255	76	10164	-1.0205	0.84625
+	TCF21	6	133854121	133905537	73	7	10164	-1.0205	0.84626
+	RNMTL1	17	747273	802509	199	29	10164	-1.0206	0.84628
-	ZNF280D	15	56620176	56769086	279	23	10164	-1.021	0.84637
+	FCHO2	5	72920981	73100522	312	16	10164	-1.021	0.84638
+	MS4A7	11	60343485	60405954	212	21	10164	-1.0214	0.84647
-	ERP27	12	14904027	14973549	277	33	10164	-1.0221	0.84663
-	DIO2	14	80187525	80266054	103	8	10164	-1.0221	0.84664
+	IL24	1	206862098	206914139	110	15	10164	-1.0222	0.84666
+	NIF3L1	2	200854327	200913932	130	34	10164	-1.0223	0.84667
-	CELF3	1	151690052	151751821	137	24	10164	-1.0228	0.84679
+	CBLL1	7	107708497	107772011	65	4	10164	-1.023	0.84684
-	SCN11A	3	38835769	38988651	361	19	10164	-1.023	0.84685
-	INTS2	17	61855367	61963030	193	21	10164	-1.0232	0.84689
-	MPHOSPH9	12	123142324	123279014	238	12	10164	-1.0234	0.84693
-	ARL8A	1	202123404	202179743	184	38	10164	-1.0234	0.84694
-	SDS	12	113382445	113438887	1	1	10164	-1.0236	0.84698
+	SDSL	12	113387268	113448277	1	1	10164	-1.0236	0.84698
-	PRR22	19	5772960	5819765	129	14	10164	-1.0236	0.847

STRAND	SYMBOL	CHR	START	STOP	NSNPS	NPARAM	N	ZSTAT	P
-	YARS2	12	32715091	32790961	270	23	10164	-1.024	0.84708
-	KRTAP2-3	17	41049240	41095092	230	12	10164	-1.0242	0.84714
+	RCOR3	1	211224366	211326385	240	24	10164	-1.0243	0.84715
-	CEP290	12	88039013	88177216	220	17	10164	-1.0244	0.84718
-	FKBP5	6	35563585	35763583	702	66	10164	-1.0246	0.84723
-	KIF18B	17	44914711	44982745	207	35	10164	-1.0249	0.84728
+	HTR3E	3	184062064	184116995	160	16	10164	-1.0249	0.84729
-	NEUROD1	2	181666106	181715665	109	11	10164	-1.0253	0.8474
-	NFKBID	19	35877129	35937334	115	12	10164	-1.0253	0.8474
+	CDK3	17	75965906	76015999	132	15	10164	-1.0254	0.84742
-	C11orf94	11	45896534	45942282	65	13	10164	-1.0255	0.84743
-	LOC1019286	15	58203535	58367001	778	69	10164	-1.0259	0.84754
+	ITPR3	6	33586379	33706574	743	77	10164	-1.0261	0.84759
+	KRTAP9-2	17	41191648	41237652	106	17	10164	-1.0265	0.84766
-	TMEM17	2	62437595	62541469	435	70	10164	-1.0264	0.84766
-	EML3	11	62592218	62647765	49	9	10164	-1.0264	0.84766
+	KIF5C	2	148840223	149036759	337	21	10164	-1.0267	0.84773
-	TXNDC8	9	110266799	110372887	287	32	10164	-1.0272	0.84784
-	JAG2	14	105130981	105203824	181	19	10164	-1.0275	0.84791
-	RUNX1T1	8	91944967	92138226	145	7	10164	-1.0277	0.84796
-	SIX2	2	44995182	45044610	240	37	10164	-1.028	0.84802
-	CCDC141	2	178819757	179085118	856	58	10164	-1.0283	0.84809
+	SMDT1	22	42044691	42094284	97	10	10164	-1.0284	0.84812
-	ART4	12	14819311	14878479	222	15	10164	-1.0285	0.84813
+	GRIN2D	19	48359875	48454937	214	35	10164	-1.0285	0.84814
+	INSM1	20	20333121	20380949	38	10	10164	-1.0288	0.84821
-	PHB2	12	6955352	7005823	32	4	10164	-1.029	0.84827
+	ATP6V1C2	2	10685974	10795110	387	41	10164	-1.0294	0.84835
+	RIC8B	12	106739285	106899316	214	25	10164	-1.0295	0.84837
+	NPM2	8	21988708	22046897	119	18	10164	-1.0296	0.8484
+	LSMEM2	3	50242915	50298114	76	11	10164	-1.0299	0.84847
+	TMEM126A	11	85612919	85666553	101	13	10164	-1.03	0.8485
-	IZUMO2	19	50142548	50198281	61	14	10164	-1.03	0.8485
+	UNC13B	9	35126982	35415337	684	43	10164	-1.0303	0.84856
-	FUT3	19	5832888	5886474	119	24	10164	-1.0304	0.84859
-	CES1	16	55792851	55868186	128	18	10164	-1.0305	0.84862
-	EYS	6	63709980	65742225	7083	155	10164	-1.0311	0.84876
-	KRT35	17	41466689	41517743	177	16	10164	-1.0312	0.84878
-	TMEM160	19	47035909	47083784	85	13	10164	-1.0312	0.84878
+	PTPRE	10	127872061	128095916	784	104	10164	-1.0316	0.84886
-	KLK4	19	50896352	50945749	275	33	10164	-1.0318	0.84892
-	TMEM100	17	55709627	55767121	75	14	10164	-1.0322	0.84902
+	ZNF783	7	149227171	149294994	236	30	10164	-1.0322	0.84902
-	SPATA2	20	49893391	49950543	200	18	10164	-1.0323	0.84904
+	TBCE	1	235332360	235458968	258	19	10164	-1.0327	0.84912
+	OR2A14	7	144094113	144140045	127	5	10164	-1.033	0.8492
+	STK17A	7	43548093	43637379	268	26	10164	-1.0331	0.84922
-	PATL2	15	44655732	44746392	155	18	10164	-1.0331	0.84922
+	AHSA2	2	61142420	61198573	155	11	10164	-1.0332	0.84925
+	MT1B	16	56616899	56663204	242	29	10164	-1.0334	0.84928
+	FICD	12	108480211	108531210	205	24	10164	-1.0335	0.8493
+	C12orf65	12	123198297	123267959	121	6	10164	-1.0338	0.84939
+	ARHGAP42	11	100652676	101000925	1032	38	10164	-1.034	0.84943
+	APC	5	112672505	112856239	479	22	10164	-1.0341	0.84946
-	HIST1H2AM	6	27882699	27928185	217	34	10164	-1.0344	0.84952
-	TAF9	5	69354743	69405013	76	11	10164	-1.0344	0.84953
+	SFXN1	5	175443511	175539742	361	29	10164	-1.0348	0.84961
-	OR6K3	1	158707168	158753115	158	9	10164	-1.0348	0.84961
-	ASB5	4	176203673	176312571	327	26	10164	-1.0348	0.84962
+	ALKBH3	11	43845807	43930275	305	25	10164	-1.0352	0.84971
+	ZNF865	19	55578593	55628545	119	14	10164	-1.0353	0.84973
+	RHOH	4	40156011	40254764	309	52	10164	-1.0354	0.84976
+	CCDC113	16	58161964	58293836	588	30	10164	-1.0354	0.84977

STRAND	SYMBOL	CHR	START	STOP	NSNPS	NPARAM	N	ZSTAT	P
-	TSPYL5	8	97263486	97312948	132	17	10164	-1.0363	0.84997
+	GALNT12	9	98772699	98860081	238	32	10164	-1.0364	0.85
-	TRIM47	17	75864164	75913600	97	8	10164	-1.0369	0.8501
-	DCHS2	4	154224375	154526778	738	56	10164	-1.0371	0.85015
+	ZNF805	19	57205685	57272738	206	18	10164	-1.0377	0.85029
-	MNX1	7	156994853	157045653	114	22	10164	-1.0377	0.8503
-	KIAA1429	8	94477777	94588518	349	18	10164	-1.0377	0.8503
+	DLX1	2	172050480	172099674	122	27	10164	-1.0378	0.85032
-	C2CD2	21	41875110	41988957	384	64	10164	-1.0378	0.85032
-	HDHD2	18	47097410	47185515	332	24	10164	-1.0378	0.85032
+	ZBTB1	14	64468950	64543690	195	16	10164	-1.0379	0.85035
+	TMEM190	19	55341836	55388244	127	22	10164	-1.0381	0.8504
+	ERICH5	8	98029522	98103610	136	21	10164	-1.0383	0.85044
-	MRPL9	1	151749643	151798916	150	18	10164	-1.0386	0.8505
+	OR2A5	7	144015402	144061337	199	11	10164	-1.04	0.85082
-	FAM132A	1	1232446	1282218	83	8	10164	-1.0401	0.85085
+	POP4	19	29571263	29627255	273	33	10164	-1.0402	0.85087
-	PHYKPL	5	178198474	178267822	264	47	10164	-1.0405	0.85094
-	SH3PXD2A	10	103584027	103890482	762	72	10164	-1.0408	0.85102
+	NCCRP1	19	39161964	39211882	90	9	10164	-1.041	0.85105
-	PPARGC1A	4	23782021	24507771	2052	150	10164	-1.0413	0.85113
+	FBXO40	3	121558323	121640292	271	38	10164	-1.0415	0.85119
-	ACIN1	14	23048564	23130614	229	37	10164	-1.0417	0.85122
+	RFX4	12	106547907	106772804	423	35	10164	-1.0417	0.85123
+	MGC57346-C	17	45585329	45845828	32	12	10164	-1.042	0.85129
+	SH3BP4	2	234916984	235065714	496	65	10164	-1.0427	0.85146
-	APOF	12	56350571	56397799	39	5	10164	-1.0428	0.85148
+	TMEM51	1	15117532	15230478	378	23	10164	-1.0433	0.85161
+	MKL2	16	14036321	14276776	303	6	10164	-1.0436	0.85167
-	ST13	22	40814535	40892008	127	23	10164	-1.0437	0.85168
-	ACTR1B	2	97645939	97699098	98	15	10164	-1.0438	0.8517
+	OR4C6	11	55623587	55676189	5	3	10164	-1.0438	0.85172
+	SIGLEC7	19	51107294	51163526	130	34	10164	-1.044	0.85175
+	BUD31	7	99373641	99429616	115	16	10164	-1.0443	0.85182
+	NEFH	22	29445192	29501290	163	30	10164	-1.0444	0.85185
+	ALPI	2	232421112	232470745	233	23	10164	-1.0448	0.85194
-	RPL30	8	98031710	98080590	120	17	10164	-1.0448	0.85195
+	EHD2	19	47678344	47753134	136	19	10164	-1.045	0.85199
+	FANCI	15	89208948	89327131	373	18	10164	-1.0451	0.852
-	PPIB	15	64145815	64198155	46	7	10164	-1.0452	0.85202
+	FAM109B	22	42039251	42089438	89	10	10164	-1.0454	0.85208
+	CNIH3	1	224399790	224750567	1192	77	10164	-1.0455	0.85211
-	RFK	9	76375517	76429528	126	8	10164	-1.0459	0.85219
+	MAST3	19	18062764	18161692	332	26	10164	-1.0462	0.85225
+	DDX25	11	125869366	125933110	175	28	10164	-1.0462	0.85227
+	RAB19	7	140368822	140436878	10	3	10164	-1.0463	0.85228
+	PM20D2	6	89061753	89175569	374	16	10164	-1.0464	0.85232
+	TAS2R60	7	143408453	143454409	143	27	10164	-1.0465	0.85233
+	EXD2	14	69156477	69254020	129	14	10164	-1.0467	0.85239
+	DBF4B	17	44673608	44768852	168	26	10164	-1.047	0.85245
-	LRIT2	10	84210485	84260589	174	19	10164	-1.047	0.85246
+	OR2A12	7	144060108	144106040	95	11	10164	-1.0478	0.85263
+	LOC1019281	8	143045043	143093478	15	3	10164	-1.0479	0.85265
+	EIF4H	7	74139376	74207101	67	5	10164	-1.048	0.85267
-	S100A11	1	152022506	152072035	103	11	10164	-1.048	0.85268
+	NRBP1	2	27393606	27452259	84	8	10164	-1.0497	0.85308
+	EPHB3	3	184526799	184592408	192	21	10164	-1.0503	0.85321
+	N4BP2	4	40021212	40195798	557	39	10164	-1.0503	0.85321
+	LNPEP	5	96900642	97039411	433	19	10164	-1.0507	0.8533
-	MAMSTR	19	48702722	48754778	150	13	10164	-1.0508	0.85333
+	FPGT	1	74163212	74218702	99	7	10164	-1.0515	0.85349
+	CPNE3	8	86479427	86571498	252	23	10164	-1.052	0.85359
-	SLC36A4	11	93134171	93232975	189	18	10164	-1.052	0.8536

STRAND	SYMBOL	CHR	START	STOP	NSNPS	NPARAM	N	ZSTAT	P
+	GZMA	5	55067646	55120252	91	7	10164	-1.0528	0.85377
-	HIST1H3J	6	27880315	27925792	219	32	10164	-1.0529	0.85381
-	OR5R1	11	56407258	56453232	200	17	10164	-1.0531	0.85384
-	MYH15	3	108370368	108584465	462	31	10164	-1.0531	0.85385
+	NPFFR2	4	71996804	72158201	482	14	10164	-1.0533	0.85389
-	PTPN21	14	88455778	88589779	274	19	10164	-1.0537	0.85399
+	MS4A10	11	60750304	60811305	77	16	10164	-1.0537	0.85399
-	RNF43	17	58343676	58452620	157	24	10164	-1.0545	0.85418
-	SBNO1	12	123279109	123400525	315	9	10164	-1.0546	0.85419
-	SSR3	3	156530140	156590184	277	34	10164	-1.0546	0.85421
-	PTPN7	1	202137012	202196592	212	29	10164	-1.0549	0.85427
-	ITLN2	1	160935020	160992378	204	16	10164	-1.055	0.8543
-	METTL3	14	21488131	21546375	67	12	10164	-1.0567	0.85468
-	NPAT	11	108147215	108257638	210	23	10164	-1.0572	0.85478
+	PATE1	11	125711293	125759848	167	15	10164	-1.0572	0.85479
+	LDLRAP1	1	25508580	25601287	75	7	10164	-1.0576	0.85489
+	GALNT13	2	153836898	154463977	2275	74	10164	-1.0582	0.85502
+	PHGDH	1	119676737	119754226	180	29	10164	-1.0583	0.85503
-	PANK4	1	2498531	2561628	134	15	10164	-1.0588	0.85515
-	TMEM14E	3	152329698	152375990	100	21	10164	-1.0588	0.85515
-	ZNF564	19	12515370	12586542	83	12	10164	-1.0589	0.85518
-	HESX1	3	57187838	57262632	97	15	10164	-1.059	0.85519
-	CDRT15	17	14225856	14271833	51	6	10164	-1.059	0.85519
-	CEP104	1	3802081	3892387	463	26	10164	-1.0591	0.85521
-	PLA2G6	22	38101495	38227109	498	28	10164	-1.0592	0.85525
-	TAF11	6	34867778	34923071	215	27	10164	-1.0595	0.85532
+	ARHGEF5	7	144320396	144390632	24	5	10164	-1.0597	0.85536
-	LOC1053765	11	6723720	6781480	262	24	10164	-1.0597	0.85536
-	RBPMS2	15	64729894	64810571	141	6	10164	-1.0599	0.85541
-	FAM193B	5	177509789	177589547	134	13	10164	-1.0601	0.85545
+	STXBP1	9	127577207	127702716	259	28	10164	-1.0603	0.8555
-	OR52B2	11	6159355	6205326	129	14	10164	-1.0604	0.85552
-	GRID2IP	7	6486778	6586436	232	35	10164	-1.0607	0.85558
-	ADNP	20	50878918	50965990	196	22	10164	-1.0609	0.85564
-	FIS1	7	101229612	101280090	75	17	10164	-1.0617	0.85581
+	LOC1053700	12	124260091	124311978	254	27	10164	-1.0621	0.85589
-	LOC1053720	18	6396091	6449772	52	10	10164	-1.0622	0.85593
-	GYPA	4	144099303	144175752	198	20	10164	-1.0623	0.85596
+	SERPINB12	18	63484090	63579329	337	22	10164	-1.0624	0.85597
-	CTSF	11	66553463	66603576	122	20	10164	-1.0624	0.85598
-	OR5M10	11	56566774	56612721	165	18	10164	-1.0627	0.85604
+	SETMAR	3	4268304	4327265	178	24	10164	-1.0632	0.85615
+	OR4C13	11	49917391	49963419	177	16	10164	-1.0633	0.85618
-	MEX3C	18	51164550	51232681	106	9	10164	-1.0638	0.85629
-	OR13C5	9	104588457	104634413	205	6	10164	-1.0638	0.85629
-	OR5M1	11	56602555	56648502	123	15	10164	-1.0639	0.85632
+	PLK5	19	1489074	1545456	95	11	10164	-1.0642	0.85638
+	EFHD1	2	232571057	232692781	411	55	10164	-1.0643	0.8564
-	GBE1	3	81479699	81796799	572	34	10164	-1.0645	0.85644
+	PIGQ	16	534968	594136	161	17	10164	-1.0645	0.85646
+	FAM86C1	11	71752511	71811234	272	18	10164	-1.0646	0.85647
+	NRN1L	16	67849878	67896368	55	9	10164	-1.0648	0.85652
-	NLRP9	19	55698432	55780528	294	41	10164	-1.0648	0.85652
-	TFIP11	22	26481927	26547496	210	36	10164	-1.0659	0.85676
+	ADGRA1	10	133035929	133141675	195	23	10164	-1.0662	0.85684
-	TBCA	5	77681169	77811361	496	25	10164	-1.0666	0.85693
+	COCH	14	30839535	30905079	189	32	10164	-1.0669	0.857
-	SPIRE1	18	12436512	12693102	778	38	10164	-1.0678	0.85719
-	SULT1E1	4	69831212	69895765	183	36	10164	-1.0679	0.85721
-	NODAL	10	70421936	70476709	116	20	10164	-1.0681	0.85726
+	OAS3	12	112903433	112983249	231	11	10164	-1.0681	0.85726
+	RGPD4	2	107791932	107902544	72	10	10164	-1.0681	0.85726
+	MPDU1	17	7548647	7598212	128	24	10164	-1.0684	0.85733

STRAND	SYMBOL	CHR	START	STOP	NSNPS	NPARAM	N	ZSTAT	P
+	CLVS2	6	122960971	123082920	313	23	10164	-1.0689	0.85744
+	TMEM179B	11	62752399	62800400	60	7	10164	-1.0692	0.85752
+	DUS4L	7	107528957	107588523	104	8	10164	-1.0694	0.85755
-	MPC1	6	166354919	166418013	5	3	10164	-1.0695	0.85757
-	CRACR2A	12	3602930	3788200	680	64	10164	-1.0695	0.85757
-	M6PR	12	8930361	8984761	132	9	10164	-1.0695	0.85759
-	GOT1L1	8	37924281	37975157	91	12	10164	-1.0695	0.85759
-	GPC2	7	100159606	100212426	100	10	10164	-1.0697	0.85763
-	IL12RB1	19	18048995	18133986	338	28	10164	-1.0699	0.85766
+	UST	6	148711962	149086990	1000	121	10164	-1.0701	0.85772
+	TRAPPC10	21	43977325	44116552	287	19	10164	-1.0706	0.85781
+	SNAI1	20	49947976	49998886	239	32	10164	-1.0714	0.858
+	GLTPD2	17	4753422	4800592	161	31	10164	-1.0714	0.85801
-	MZT1	13	72698357	72762800	175	17	10164	-1.0719	0.85811
-	STRC	15	43589243	43746486	182	17	10164	-1.0729	0.85835
-	TRIM65	17	75870379	75932003	113	10	10164	-1.073	0.85837
-	CLK4	5	178592664	178665639	221	14	10164	-1.0732	0.85841
+	SLC6A18	5	1190355	1256189	295	49	10164	-1.0732	0.85842
-	OR2C3	1	247520132	247568839	141	21	10164	-1.0733	0.85844
+	CBX3	7	26166211	26223607	149	27	10164	-1.0735	0.85848
-	FGD6	12	95066749	95252464	632	34	10164	-1.0739	0.85857
+	LOC1053763	9	121159174	121222539	162	22	10164	-1.0741	0.8586
-	RAC2	22	37215261	37279299	195	23	10164	-1.0741	0.85861
+	ZPLD1	3	102322481	102489841	41	15	10164	-1.0742	0.85864
+	FARS2	6	5226018	5781583	1630	146	10164	-1.0744	0.85868
+	CABP7	22	29685090	29741833	183	32	10164	-1.0744	0.85868
-	GUCD1	22	24530423	24590935	116	12	10164	-1.0747	0.85876
+	EXOC3L4	14	103059410	103120562	179	28	10164	-1.0748	0.85878
-	SIAH1	16	48350531	48483440	147	19	10164	-1.0751	0.85883
+	LILRA2	19	54537997	54597560	220	18	10164	-1.0751	0.85883
+	NWD1	19	16684853	16827963	128	17	10164	-1.0752	0.85886
-	MATN3	2	19982052	20047694	191	27	10164	-1.0753	0.85888
+	C8A	1	56819770	56928221	223	24	10164	-1.0755	0.85893
+	IL25	14	23337809	23386403	105	21	10164	-1.0755	0.85893
-	KCNK7	11	65582855	65630996	118	29	10164	-1.0756	0.85895
-	OR3A1	17	3278318	3421209	655	26	10164	-1.0763	0.8591
+	MLXIP	12	122043728	122157347	369	14	10164	-1.0763	0.85911
-	HAS1	19	51703112	51758992	229	56	10164	-1.0766	0.85918
+	RAD51B	14	67784779	68693210	2970	141	10164	-1.0767	0.85919
-	EIF5A2	3	170878415	170943637	126	14	10164	-1.0768	0.85921
+	PLXNC1	12	94113723	94317675	584	75	10164	-1.077	0.85926
+	OR13D1	9	104659422	104705462	162	13	10164	-1.0772	0.8593
-	C11orf21	11	2286277	2336913	295	25	10164	-1.0774	0.85936
-	C19orf73	19	49108397	49154140	127	13	10164	-1.0784	0.85957
-	RBM43	2	151238214	151296901	215	19	10164	-1.0786	0.85962
+	MSH4	1	75761871	75923238	532	19	10164	-1.0789	0.85969
+	APLP1	19	35833499	35889797	84	13	10164	-1.0792	0.85974
-	SFRP4	7	37895932	37951923	254	32	10164	-1.0792	0.85974
+	SLC25A42	19	19028966	19123030	151	12	10164	-1.0792	0.85976
+	TMEM80	11	660591	715028	198	22	10164	-1.0793	0.85977
-	CLASP2	3	33486245	33753356	577	26	10164	-1.0794	0.85979
-	NOP14	4	2927936	2998506	174	15	10164	-1.0795	0.85981
-	KDELC2	11	108462105	108533519	162	29	10164	-1.0795	0.85983
-	OARD1	6	41056792	41107449	151	12	10164	-1.0795	0.85983
-	AIM1L	1	26311859	26389130	241	23	10164	-1.0797	0.85986
+	HEXIM1	17	45112317	45162101	118	25	10164	-1.0798	0.85988
+	CHAT	10	49574095	49675104	267	53	10164	-1.08	0.85992
-	KRTAP2-4	17	41055116	41100879	263	11	10164	-1.0801	0.85995
-	B3GAT3	11	62605296	62657175	40	9	10164	-1.0804	0.86002
-	SYVN1	11	65117279	65170178	111	30	10164	-1.0805	0.86004
+	ADHFE1	8	66397483	66478809	235	23	10164	-1.0807	0.86008
+	TMEM14C	6	10687915	10741129	135	8	10164	-1.0809	0.86012
-	PDLIM3	4	185490660	185570607	209	20	10164	-1.0809	0.86014

STRAND	SYMBOL	CHR	START	STOP	NSNPS	NPARAM	N	ZSTAT	P
+	ANKRD34C	15	79247804	79308239	253	23	10164	-1.081	0.86015
+	CACNB3	12	48779432	48838943	91	13	10164	-1.0811	0.86017
+	C2orf16	2	27541522	27592722	89	12	10164	-1.0812	0.86019
-	BVES	6	105086822	105172174	214	14	10164	-1.0815	0.86026
+	LOC1019295	17	39202786	39261019	99	19	10164	-1.0816	0.86028
-	KDELR1	19	48372570	48432689	125	24	10164	-1.0818	0.86033
-	HMGN1	21	39332315	39384121	155	17	10164	-1.0821	0.8604
-	LOC1053762	9	106847663	106896009	85	14	10164	-1.0823	0.86044
+	RBM24	6	17246492	17303875	159	19	10164	-1.0825	0.86048
+	WDR3	1	117894749	117970426	203	17	10164	-1.0827	0.86052
-	LOC1053725	20	24458226	24510800	87	20	10164	-1.0828	0.86055
+	VMP1	17	59672502	59850593	241	18	10164	-1.0833	0.86067
-	PEX16	11	45899669	45953123	70	13	10164	-1.084	0.86082
-	TAS2R46	12	11051365	11097294	62	6	10164	-1.0841	0.86083
+	TLE4	9	79536773	79736882	438	39	10164	-1.0841	0.86084
+	PPAPDC1A	10	120421954	120599855	415	54	10164	-1.0841	0.86084
+	CHMP7	8	23208637	23271999	302	41	10164	-1.0842	0.86086
-	LENG9	19	54451796	54498711	73	7	10164	-1.0842	0.86086
+	EMC6	17	3633723	3679669	163	14	10164	-1.0844	0.8609
-	LRP5L	22	25341418	25416989	148	17	10164	-1.0844	0.86091
-	WDR89	14	63587039	63676923	245	15	10164	-1.0844	0.86091
-	SUGP2	19	18980888	19068571	137	12	10164	-1.0846	0.86096
+	C3orf30	3	119111150	119161455	220	29	10164	-1.0847	0.86098
+	CR1L	1	207610113	207733691	442	26	10164	-1.0849	0.86101
+	KCNF1	2	10876937	10924225	129	12	10164	-1.0849	0.86102
+	VDAC2	10	75175154	75241448	136	19	10164	-1.085	0.86105
-	FAM150A	8	52524037	52600461	150	17	10164	-1.0852	0.86108
+	MMP9	20	45973908	46026561	172	22	10164	-1.0858	0.86122
-	CIZ1	9	128156065	128239383	165	17	10164	-1.0859	0.86123
+	RAMP1	2	237824544	237922114	397	63	10164	-1.0861	0.86128
+	PLAU	10	73874182	73927501	151	21	10164	-1.0861	0.86129
+	SLC3A1	2	44240458	44330824	225	16	10164	-1.0861	0.86129
-	CEP120	5	123334885	123458592	378	19	10164	-1.0863	0.86133
+	OAS1	12	112871934	112942049	166	6	10164	-1.0865	0.86136
-	ADH1B	4	99296387	99356442	207	13	10164	-1.0866	0.86138
+	AFAP1L1	5	149236838	149353637	215	34	10164	-1.0868	0.86144
+	PUSL1	1	1273571	1321677	102	12	10164	-1.0869	0.86146
+	EDN3	20	59265424	59335992	70	10	10164	-1.087	0.86149
-	RTKN	2	74415861	74476933	36	4	10164	-1.087	0.86149
-	PAQR3	4	78877223	78974428	178	9	10164	-1.0871	0.86151
-	CSNK1G1	15	64155517	64391243	248	9	10164	-1.0874	0.86158
-	NUP88	17	5376026	5454739	326	16	10164	-1.0877	0.86163
+	LOC1005063	19	56229260	56320454	203	30	10164	-1.0877	0.86163
-	LOC1053694	11	66711448	66762977	75	31	10164	-1.0877	0.86165
+	MOCS3	20	50923814	50971863	88	17	10164	-1.0882	0.86175
-	SEMA6B	19	4532587	4616602	14	2	10164	-1.0885	0.86182
+	OBSCN	1	228173072	228388876	955	47	10164	-1.0889	0.86191
-	ZNF516	18	76347682	76530234	230	23	10164	-1.089	0.86192
-	SNRPA1	15	101271510	101330277	216	27	10164	-1.0892	0.86197
-	ERMN	2	157308613	157362713	88	6	10164	-1.0895	0.86204
-	LOC1053767	15	89077561	89129089	110	11	10164	-1.0897	0.86207
-	ABHD12	20	25284743	25425982	391	13	10164	-1.09	0.86214
-	ARHGAP18	6	129566145	129745225	610	60	10164	-1.0902	0.86218
-	NAA11	4	79307118	79361017	51	13	10164	-1.0902	0.86219
-	RNASEH2C	11	65707673	65755938	170	29	10164	-1.0907	0.8623
+	TMEM135	11	87002844	87338834	1623	35	10164	-1.0909	0.86234
-	PRPS1L1	7	18016774	18062863	112	14	10164	-1.091	0.86237
-	C1QBP	17	5422779	5474151	146	21	10164	-1.0911	0.86238
-	DCAF4L2	8	87860743	87909068	82	25	10164	-1.0913	0.86244
+	WDR76	15	43791914	43878419	148	14	10164	-1.0915	0.86248
+	TREML4	6	41193324	41248382	277	23	10164	-1.0916	0.8625
+	WDYHV1	8	123381725	123458380	264	8	10164	-1.0918	0.86253
-	MFAP1	15	43794535	43859753	107	11	10164	-1.093	0.8628

STRAND	SYMBOL	CHR	START	STOP	NSNPS	NPARAM	N	ZSTAT	P
-	GAL3ST4	7	100149242	100203750	96	13	10164	-1.0933	0.86287
+	KRTAP9-3	17	41197463	41243454	124	18	10164	-1.0933	0.86287
+	GPR27	3	71719050	71765177	93	8	10164	-1.0935	0.86291
+	SLC48A1	12	47718842	47792753	279	41	10164	-1.0935	0.86292
-	IL6ST	5	55925095	56029993	168	15	10164	-1.0938	0.86297
+	CD3EAP	19	45371209	45420766	131	23	10164	-1.0938	0.86299
-	IWS1	2	127470807	127561886	167	9	10164	-1.0941	0.86304
-	OR1C1	1	247747462	247793406	192	14	10164	-1.0942	0.86307
-	C11orf45	11	128889565	128944717	200	36	10164	-1.0945	0.86313
-	REG4	1	119784018	119846580	168	14	10164	-1.095	0.86324
-	LNX1	4	53450270	53687514	802	58	10164	-1.0952	0.86328
+	RAB1B	11	66233585	66287492	98	20	10164	-1.0952	0.86329
+	IFNAR1	21	33289443	33369823	226	17	10164	-1.0954	0.86333
-	RIMBP2	12	130386133	130751351	1166	121	10164	-1.0961	0.86348
-	MBP	18	76968833	77168708	718	98	10164	-1.0962	0.86351
+	C14orf169	14	73455934	73503401	133	29	10164	-1.0963	0.86352
-	TK1	17	78164079	78222204	288	41	10164	-1.0963	0.86353
-	SLA	8	133026728	133138066	355	16	10164	-1.0963	0.86354
+	LOC1019276	15	101723394	101772342	37	4	10164	-1.0965	0.86356
+	CNTNAP4	16	76242278	76570884	1471	66	10164	-1.0966	0.86358
+	CR2	1	207419300	207499897	177	19	10164	-1.0967	0.86362
-	LOC1019279	16	73599525	73846427	704	81	10164	-1.0968	0.86363
-	PRSS45	3	46732091	46779755	106	10	10164	-1.0973	0.86374
+	RNF114	20	49901377	49963885	222	19	10164	-1.0974	0.86377
+	C9orf172	9	136809415	136857345	58	9	10164	-1.0975	0.86379
+	EPGN	4	74273270	74327298	103	8	10164	-1.0976	0.8638
-	KCTD13	16	29896335	29961232	60	2	10164	-1.0977	0.86382
+	MMP17	12	131793396	131861771	164	24	10164	-1.098	0.86389
-	SHANK2	11	70457856	71287738	1591	157	10164	-1.098	0.8639
+	SEC14L5	16	4923317	5029155	130	16	10164	-1.098	0.86391
-	HNRNPUL2	11	62702625	62762385	90	10	10164	-1.0984	0.86397
+	PLS1	3	142561387	142723664	362	22	10164	-1.0985	0.864
-	NCOR1	17	16020094	16251746	435	15	10164	-1.0985	0.86401
-	PIGK	1	77078981	77254447	434	25	10164	-1.0985	0.86401
-	TIAL1	10	119563465	119632029	211	26	10164	-1.0991	0.86414
-	CLEC4F	2	70798643	70858764	230	17	10164	-1.0993	0.86418
-	PTPRR	12	70628073	70955804	797	71	10164	-1.1	0.86434
-	CGB8	19	49037638	49084111	144	21	10164	-1.1001	0.86436
+	RBM14-RBM4	11	66581582	66656473	106	30	10164	-1.1004	0.86441
+	LAG3	12	6737483	6788455	110	13	10164	-1.1009	0.86454
-	MLF2	12	6737992	6788470	110	13	10164	-1.1009	0.86454
+	LOC1053767	15	101720720	101768532	35	4	10164	-1.1012	0.8646
+	SFSWAP	12	131676087	131809738	251	21	10164	-1.1018	0.86472
-	LOC1027238	1	219399459	219592280	625	44	10164	-1.1018	0.86472
-	DMGDH	5	78987564	79104674	388	24	10164	-1.1019	0.86474
+	NET1	10	5377551	5469056	415	27	10164	-1.1021	0.8648
-	NOBOX	7	144386914	144445227	144	14	10164	-1.1027	0.86492
+	MPV17L2	19	18158230	18206742	142	18	10164	-1.1028	0.86495
+	OTOP3	17	74900802	74959992	105	27	10164	-1.1036	0.86512
-	GNG7	19	2501220	2737748	363	39	10164	-1.1039	0.86518
-	B4GALT3	1	161161310	161212968	65	12	10164	-1.1041	0.86522
-	SPATA4	4	176174574	176230671	161	20	10164	-1.1041	0.86522
-	ANHX	12	133208312	133271220	45	4	10164	-1.1041	0.86523
-	STAC2	17	39200536	39260787	100	19	10164	-1.1043	0.86527
-	PTCH1	9	95432982	95551965	456	41	10164	-1.1044	0.86529
+	USP1	1	62401304	62461804	116	14	10164	-1.1047	0.86535
-	REEP5	5	112866384	112957334	287	20	10164	-1.1047	0.86536
+	LRRC74A	14	76791376	76880302	252	32	10164	-1.1049	0.86539
+	CAMKMT	2	44326904	44782592	1251	50	10164	-1.1051	0.86543
-	GCN1L1	12	120117210	120229710	151	13	10164	-1.1051	0.86544
-	NFKBIE	6	44248166	44300788	122	13	10164	-1.1054	0.86551
-	TMX4	20	7967353	8054829	270	21	10164	-1.1055	0.86552
+	POU3F2	6	98799704	98848790	93	16	10164	-1.1058	0.86559

STRAND	SYMBOL	CHR	START	STOP	NSNPS	NPARAM	N	ZSTAT	P
+	UNC45A	15	90895008	90964093	187	21	10164	-1.106	0.86564
-	SPOP	17	49588884	49713163	179	38	10164	-1.1066	0.86576
-	PRDM5	4	120674928	120957866	1074	35	10164	-1.1066	0.86576
+	ARMC10	7	103039727	103109763	69	17	10164	-1.1068	0.8658
-	OSBPL5	11	3077115	3200352	486	58	10164	-1.1068	0.86582
-	SLC26A3	7	107755467	107838233	237	37	10164	-1.1073	0.86593
+	C9orf117	9	127671988	127726002	101	15	10164	-1.1073	0.86593
-	TMEM18	2	657973	712439	86	13	10164	-1.1081	0.86609
+	ITGB8	7	20294803	20425759	428	38	10164	-1.1082	0.8661
+	OR2B6	6	27922241	27968182	186	30	10164	-1.1085	0.86617
-	AKR1B1	7	134432351	134494136	167	32	10164	-1.1089	0.86626
+	KCTD15	19	33761846	33825761	117	21	10164	-1.109	0.86628
+	APC2	19	1411268	1483244	52	5	10164	-1.1092	0.86632
+	MUSK	9	110633545	110816542	660	65	10164	-1.1094	0.86638
+	MRAP	21	32256813	32324784	158	29	10164	-1.1098	0.86646
+	LDLRAD4	18	13183730	13662754	1218	149	10164	-1.1099	0.86649
+	GCSAML	1	247472058	247587690	308	28	10164	-1.11	0.8665
+	SLCO4A1	20	62607445	62695830	255	52	10164	-1.1102	0.86654
+	GRIN1	9	137104112	137178762	114	26	10164	-1.1102	0.86655
+	SPINK9	5	148300559	148349849	186	16	10164	-1.1105	0.86661
+	TFAM	10	58350143	58409230	74	9	10164	-1.1109	0.8667
+	GSX2	4	54065081	54111955	85	16	10164	-1.111	0.86672
-	SH3PXD2B	5	172315181	172489523	505	25	10164	-1.1114	0.86679
+	CNPY3	6	42894122	42949270	150	17	10164	-1.1116	0.86684
+	LIN37	19	35713361	35764519	127	21	10164	-1.1121	0.86696
-	ABO	9	133245176	133310214	401	15	10164	-1.1123	0.86699
-	SYT3	19	50611860	50693127	256	52	10164	-1.1129	0.86712
+	LOC1053771	3	48389610	48439742	69	9	10164	-1.1129	0.86713
-	WWTR1	3	149507235	149738273	817	71	10164	-1.1133	0.8672
-	IFT46	11	118534528	118607965	148	8	10164	-1.1134	0.86723
+	CKS1B	1	154939642	154989249	72	16	10164	-1.1135	0.86726
-	AARSD1	17	42940526	42999498	50	7	10164	-1.1135	0.86726
+	C15orf39	15	75163887	75222169	119	22	10164	-1.1138	0.86732
-	RABAC1	19	41946681	41994376	51	13	10164	-1.114	0.86736
-	KHDC1L	6	73213544	73260452	139	22	10164	-1.114	0.86737
-	RAET1L	6	150008342	150060596	216	20	10164	-1.1142	0.86739
+	GFER	16	1949149	1997749	165	22	10164	-1.1144	0.86745
-	UBE2O	17	76379532	76488206	133	10	10164	-1.1146	0.86749
-	CLDN6	16	3004712	3053187	8	3	10164	-1.1152	0.86761
-	MRPS23	17	57828926	57885072	91	17	10164	-1.1152	0.86762
-	OR5M11	11	56532340	56578257	189	16	10164	-1.1152	0.86762
+	SSPN	12	26086951	26244777	471	46	10164	-1.1153	0.86765
-	TMEM176B	7	150781287	150836360	288	33	10164	-1.1154	0.86766
-	DOC2B	17	133688	216630	12	2	10164	-1.1155	0.86768
-	C1orf74	1	209772317	209819545	150	12	10164	-1.1157	0.86773
+	GATAD2A	19	19350833	19518932	432	25	10164	-1.116	0.86778
-	NEK2	1	211648256	211710630	188	24	10164	-1.1163	0.86785
+	ZKSCAN8	6	28101743	28169472	306	23	10164	-1.1163	0.86785
-	GCK	7	44134271	44224423	238	33	10164	-1.1164	0.86787
-	MYH9	22	36271277	36423067	355	23	10164	-1.1166	0.86791
-	LRRN4CL	11	62676402	62724899	68	7	10164	-1.117	0.868
-	STX8	17	9240471	9610958	766	89	10164	-1.1173	0.86806
+	HRH4	18	24425568	24497427	184	22	10164	-1.1174	0.86809
+	KIAA1107	1	92132052	92194723	98	13	10164	-1.1175	0.8681
-	ZBTB25	14	64439106	64540213	256	23	10164	-1.1176	0.86813
-	IKZF5	10	122980806	123043850	171	15	10164	-1.1183	0.86827
-	CDH24	14	23037061	23092619	145	28	10164	-1.1185	0.86833
+	NFIX	19	12960756	13108796	47	6	10164	-1.1188	0.86838
-	LPCAT3	12	6966185	7053538	45	7	10164	-1.119	0.86842
-	ABHD8	19	17282131	17338473	221	22	10164	-1.12	0.86864
-	AKNA	9	114324149	114433551	337	30	10164	-1.1201	0.86865
+	GKN1	2	68939573	68990980	184	22	10164	-1.1202	0.8687
-	SURF4	9	133351449	133412949	177	7	10164	-1.1218	0.86903

STRAND	SYMBOL	CHR	START	STOP	NSNPS	NPARAM	N	ZSTAT	P
-	HPS4	22	26433613	26518863	226	22	10164	-1.1219	0.86904
-	DYDC1	10	80326102	80391787	248	17	10164	-1.1219	0.86905
-	COL2A1	12	47962965	48060286	370	33	10164	-1.1221	0.86909
+	NANOG	12	7753426	7806061	228	27	10164	-1.1222	0.86912
+	SUMF2	7	56029007	56090672	184	25	10164	-1.1233	0.86934
-	SHC1	1	154952298	155009492	78	14	10164	-1.1234	0.86937
-	KRTAP4-11	17	41107181	41153354	198	8	10164	-1.1235	0.86938
-	GJD3	17	40350652	40399693	24	8	10164	-1.1235	0.86939
+	RNF32	7	156605286	156687893	310	37	10164	-1.1235	0.8694
+	CBX2	17	79742188	79797650	23	8	10164	-1.1238	0.86945
-	RILPL2	12	123400076	123471733	76	10	10164	-1.124	0.8695
+	USP14	18	123483	223739	234	22	10164	-1.1241	0.86951
+	GSTM2	1	109633022	109693997	70	10	10164	-1.1253	0.86976
+	ASIC4	2	219479170	219548772	132	15	10164	-1.1253	0.86977
-	TRIM11	1	228383672	228441877	182	29	10164	-1.1258	0.86989
-	C9orf40	9	74936583	74987886	78	9	10164	-1.126	0.86991
+	SCNN1D	1	1245415	1302029	94	12	10164	-1.1261	0.86994
+	FABP7	6	122744501	122794074	208	39	10164	-1.1264	0.86999
-	ASB18	2	236184872	236299345	7	2	10164	-1.1266	0.87003
-	WARS	14	100323788	100411343	280	25	10164	-1.1271	0.87015
-	PPP2CB	8	30775610	30847836	168	18	10164	-1.1273	0.87019
+	SERPINF2	17	1707807	1765265	158	19	10164	-1.1274	0.87022
+	CYB5R4	6	83824651	83970427	266	10	10164	-1.1276	0.87025
-	REV3L	6	111289031	111518715	535	36	10164	-1.1279	0.87033
+	VIPR1	3	42454299	42547573	314	40	10164	-1.128	0.87034
-	DVL1	1	1325278	1384158	177	7	10164	-1.1284	0.87043
-	MAPK3	16	30104105	30158309	54	8	10164	-1.1285	0.87044
+	SETD6	16	58480479	58530527	94	18	10164	-1.1285	0.87045
-	RBP5	12	7109074	7165236	116	14	10164	-1.1285	0.87045
-	ZBTB24	6	109452516	109518237	154	18	10164	-1.1286	0.87047
-	COL8A2	1	36085236	36160770	183	22	10164	-1.1288	0.87052
+	IMPACT	18	24391645	24463531	223	16	10164	-1.1288	0.87052
+	TYMS	18	622604	683499	256	37	10164	-1.129	0.87055
-	RNF222	17	8380704	8432833	139	21	10164	-1.129	0.87055
-	PXN	12	120200439	120300771	190	24	10164	-1.1291	0.87057
+	C2orf72	2	231002566	231059714	193	22	10164	-1.1292	0.8706
+	FETUB	3	186600832	186663008	177	25	10164	-1.1293	0.87061
-	TSTD1	1	161027631	161073984	84	12	10164	-1.1293	0.87061
-	C5orf15	5	133945507	134003715	109	16	10164	-1.1294	0.87065
-	KRT39	17	40948417	41001892	342	18	10164	-1.1298	0.87073
-	OR5M3	11	56459488	56505538	181	17	10164	-1.1299	0.87074
-	SMG6	17	2049839	2338813	611	16	10164	-1.1304	0.87085
+	MEGF10	5	127193947	127471222	894	27	10164	-1.1305	0.87086
-	CFAP20	16	58103592	58164392	240	24	10164	-1.1308	0.87093
-	CD247	1	167420640	167553616	402	70	10164	-1.1316	0.87109
+	FMNL2	2	152300170	152659834	1198	70	10164	-1.1315	0.87109
-	ZNF99	19	22742183	22819171	355	14	10164	-1.1316	0.8711
-	P2RY12	3	151326843	151419812	322	27	10164	-1.1318	0.87114
+	DHDDS	1	26397282	26481306	148	20	10164	-1.1321	0.8712
-	AMICA1	11	118183727	118260094	294	19	10164	-1.1321	0.87121
+	CPNE9	3	9668812	9739908	113	36	10164	-1.1323	0.87124
-	ERICH6B	13	45531241	45650739	271	37	10164	-1.1326	0.87131
-	SUV420H1	11	68144863	68248783	225	23	10164	-1.1328	0.87135
-	DSC3	18	30979365	31077815	121	17	10164	-1.1332	0.87144
+	COLEC10	8	118917240	119118455	812	28	10164	-1.1332	0.87144
-	AQP7	9	33374947	33437645	164	26	10164	-1.1334	0.87147
-	VPS53	17	498668	749856	931	38	10164	-1.1334	0.87148
+	CKMT1A	15	43657886	43709222	20	7	10164	-1.1334	0.87148
-	BLOC1S5	6	8003567	8099414	467	32	10164	-1.1337	0.87155
-	TREML2	6	41179749	41236192	255	24	10164	-1.1337	0.87155
+	GPR35	2	240570408	240641259	250	29	10164	-1.1338	0.87156
+	ZNF330	4	141185294	141244697	150	7	10164	-1.134	0.87159
+	HIP1R	12	122799498	122872961	116	3	10164	-1.1341	0.87162

STRAND	SYMBOL	CHR	START	STOP	NSNPS	NPARAM	N	ZSTAT	P
-	KIAA0408	6	127428406	127494390	162	17	10164	-1.1341	0.87162
-	KIAA2018	3	113638385	113731646	177	21	10164	-1.1344	0.87168
-	FGF19	11	69688238	69739338	158	32	10164	-1.1344	0.87169
-	FIZ1	19	55581371	55634696	116	15	10164	-1.1344	0.87169
-	KRTAP2-2	17	41044498	41090211	219	14	10164	-1.135	0.87182
-	PDLIM1	10	95227568	95326148	317	40	10164	-1.1351	0.87183
-	TOPORS	9	32530544	32587628	107	20	10164	-1.1352	0.87185
+	HRASLS	3	193206128	193310939	181	11	10164	-1.1352	0.87186
-	PPM1G	2	27371199	27444683	119	10	10164	-1.1359	0.872
+	DSP	6	7506575	7596713	319	34	10164	-1.1364	0.87211
+	OR2AG1	11	6750017	6796014	217	21	10164	-1.1365	0.87213
+	OR1J2	9	122413953	122590751	658	47	10164	-1.1366	0.87214
+	PILRB	7	100323003	100377831	96	9	10164	-1.1373	0.87229
+	CABYR	18	24103956	24171600	42	10	10164	-1.1375	0.87234
+	CRTC1	19	18648615	18792333	295	21	10164	-1.1376	0.87235
-	ILKAP	2	238160401	238238702	324	22	10164	-1.1376	0.87235
-	IGSF8	1	160081339	160133860	109	17	10164	-1.1377	0.87238
+	ARHGDIG	16	245606	293003	210	38	10164	-1.1379	0.87241
-	SCARA5	8	27859882	28027852	541	53	10164	-1.1381	0.87247
+	SEPN1	1	25765176	25828222	32	4	10164	-1.1383	0.8725
+	FGFRL1	4	974979	1036898	236	44	10164	-1.1386	0.87256
-	MB21D2	3	192786815	192953161	202	14	10164	-1.1386	0.87256
+	OR10K1	1	158430562	158476503	126	7	10164	-1.14	0.87286
-	IGLL1	22	23563125	23615472	73	19	10164	-1.1402	0.87291
-	ZC3H4	19	47054187	47148752	141	19	10164	-1.1403	0.87292
-	ADH1C	4	99326492	99388045	354	14	10164	-1.1404	0.87293
-	COX6B2	19	55339702	55389814	137	23	10164	-1.1404	0.87294
+	THEMIS2	1	27837543	27896685	126	14	10164	-1.1406	0.87299
-	OR10W1	11	58256792	58303260	196	15	10164	-1.1406	0.87299
-	PAX5	9	36823275	37070319	783	79	10164	-1.1407	0.873
-	RNASEL	1	182563634	182624285	137	30	10164	-1.1407	0.87301
-	SUSD4	1	223210819	223399202	125	8	10164	-1.1408	0.87301
-	UBE2J1	6	89316625	89387900	186	24	10164	-1.1408	0.87303
-	USH1G	17	74906083	74958263	100	26	10164	-1.1412	0.8731
+	LOC1053753	7	87023643	87121964	291	24	10164	-1.1411	0.8731
+	PPP1R1C	2	181919241	182141382	468	44	10164	-1.1413	0.87313
+	KTN1	14	55545207	55694584	536	22	10164	-1.1418	0.87323
+	SPACA4	19	48571743	48617714	187	30	10164	-1.1418	0.87323
+	ATP1B2	17	7611627	7667771	191	19	10164	-1.1419	0.87324
+	HTR3A	11	113940075	114000313	164	36	10164	-1.1425	0.87338
-	SAMD1	19	14078417	14125420	25	6	10164	-1.1435	0.87359
+	AANAT	17	76417853	76480790	77	8	10164	-1.144	0.87368
-	TAF1D	11	93725929	93776537	130	10	10164	-1.1441	0.8737
-	OTOS	2	240129029	240175656	28	5	10164	-1.1444	0.87376
-	LOC1053789	5	61311490	61367216	67	11	10164	-1.1444	0.87376
+	SYK	9	90766680	90908560	661	62	10164	-1.1445	0.87379
+	LOC1053775	4	170057143	170117095	61	9	10164	-1.1448	0.87385
+	PPM1L	3	160720532	161088902	1111	54	10164	-1.145	0.8739
-	SULT2A1	19	47860466	47921397	175	23	10164	-1.1454	0.87398
+	DCSTAMP	8	104304087	104366689	278	33	10164	-1.1457	0.87404
+	C1orf100	1	244317069	244399874	252	39	10164	-1.146	0.87411
+	IL10RB	21	33231360	33307234	230	26	10164	-1.1462	0.87414
+	URB2	1	229591205	229670887	166	12	10164	-1.1463	0.87417
-	LOC1001281	15	101714664	101782827	61	5	10164	-1.1464	0.87418
-	NLRP1	17	5491399	5619512	601	25	10164	-1.1466	0.87422
-	C2orf66	2	196794415	196845276	98	6	10164	-1.147	0.87431
-	NDUFA7	19	8298762	8356396	89	5	10164	-1.1471	0.87433
+	CALML6	1	1878752	1927294	31	2	10164	-1.1471	0.87434
-	YIF1A	11	66274580	66324167	112	30	10164	-1.1474	0.87439
-	OR13C2	9	104594671	104640627	202	6	10164	-1.1479	0.87449
-	KRT32	17	41449513	41504252	275	23	10164	-1.1484	0.8746
+	STAG3	7	100142563	100224387	170	15	10164	-1.1485	0.87462
+	OGFRL1	6	71253774	71318953	297	20	10164	-1.1492	0.87477

STRAND	SYMBOL	CHR	START	STOP	NSNPS	NPARAM	N	ZSTAT	P
-	HSPB6	19	35744566	35792029	165	25	10164	-1.1494	0.8748
-	CD164	6	109356514	109417559	202	20	10164	-1.1498	0.87489
+	IGFBP4	17	40408424	40467730	52	8	10164	-1.1509	0.87512
+	ENPP3	6	131602235	131757413	215	20	10164	-1.1513	0.8752
-	MAK	6	10752723	10873605	258	43	10164	-1.1516	0.87526
+	LOC1002937	12	123328059	123375789	148	7	10164	-1.1516	0.87526
+	STKLD1	9	133341413	133416096	212	9	10164	-1.1518	0.8753
-	HIVEP3	1	41496365	42070931	1363	102	10164	-1.152	0.87534
+	ARHGEF28	5	73591158	73951993	509	56	10164	-1.1521	0.87536
+	CTNNB1	3	41164451	41250448	144	17	10164	-1.1523	0.8754
+	MBNL1	3	152209321	152475780	522	55	10164	-1.1524	0.87541
-	ANKUB1	3	149750648	149827843	203	36	10164	-1.1524	0.87543
-	RD3	1	211466522	211527917	299	26	10164	-1.1525	0.87544
-	CTRL	16	67919570	67966875	57	10	10164	-1.1528	0.8755
+	IRAK1BP1	6	78832085	78946160	424	17	10164	-1.1528	0.87551
+	INSIG1	7	155262776	155320235	28	5	10164	-1.1529	0.87553
+	FOXK1	7	4609691	4781443	66	7	10164	-1.1533	0.87561
-	GNAT2	1	109592906	109654662	124	22	10164	-1.1538	0.87571
+	TRIM69	15	44701362	44777829	205	14	10164	-1.1543	0.87581
+	LRP5	11	68263866	68459275	601	67	10164	-1.1543	0.87582
+	ZNF316	7	6602322	6668279	172	25	10164	-1.1544	0.87583
-	PDE6A	5	149847956	149994518	371	50	10164	-1.1551	0.87598
+	RUFY4	2	217999934	218100625	321	30	10164	-1.1557	0.87609
+	FAAH	1	46359267	46423848	291	15	10164	-1.1565	0.87625
+	GPR52	1	174413074	174459545	84	10	10164	-1.1566	0.87627
-	C10orf25	10	44983932	45035910	185	26	10164	-1.1571	0.87637
+	ORMDL2	12	55783022	55831175	51	9	10164	-1.1577	0.8765
-	ANKH	5	14694800	14906778	507	51	10164	-1.1578	0.87652
-	LOC1053711	16	29120314	29167441	118	5	10164	-1.1578	0.87653
+	RAC3	17	81996656	82044204	33	3	10164	-1.1578	0.87654
+	ZNF143	11	9425965	9538524	167	13	10164	-1.158	0.87658
-	MAML3	4	139706391	140189079	952	116	10164	-1.1581	0.87659
+	PTPRM	18	7532316	8416861	2392	186	10164	-1.1584	0.87664
-	RAB17	2	237564322	237626126	282	33	10164	-1.159	0.87676
-	C12orf79	12	91974976	92177671	273	43	10164	-1.1591	0.87679
-	TNFSF15	9	114774635	114841128	150	26	10164	-1.1594	0.87685
-	STX6	1	180939690	181058121	248	30	10164	-1.1597	0.87692
+	SLC25A36	3	140906820	140989943	189	8	10164	-1.1598	0.87693
+	CSF1	1	109875611	109940994	171	32	10164	-1.1599	0.87695
-	TWIST1	7	19103047	19152672	174	21	10164	-1.1609	0.87715
-	CXCR4	2	136104349	136153155	98	20	10164	-1.1614	0.87726
+	ABHD13	13	108183415	108244255	154	20	10164	-1.1615	0.87729
+	LRRC45	17	81988250	82041151	33	3	10164	-1.1617	0.87732
-	DLG5	10	77780787	77961590	73	7	10164	-1.1617	0.87733
+	PHPT1	9	136813816	136861043	57	9	10164	-1.1623	0.87743
-	ATP5L2	22	42629803	42675601	211	17	10164	-1.1623	0.87744
+	LOC1053711	16	30335114	30383010	11	3	10164	-1.1628	0.87755
-	HCAR1	12	122717606	122765582	41	3	10164	-1.1629	0.87757
+	RASGRF2	5	80925672	81240162	1098	108	10164	-1.1641	0.87782
+	NYAP1	7	100448862	100504802	89	11	10164	-1.1642	0.87783
+	RCE1	11	66808412	66856532	50	21	10164	-1.1644	0.87787
+	LOC1053694	11	93162639	93213569	102	13	10164	-1.1644	0.87787
-	LOC1053785	10	119416744	119464244	168	20	10164	-1.165	0.87798
-	CHRNA10	11	3655587	3708208	86	11	10164	-1.1651	0.87802
-	GKN2	2	68935232	68987970	162	23	10164	-1.1654	0.87808
+	CLCN3	4	169585521	169733187	173	8	10164	-1.1655	0.87809
+	CHCHD6	3	126669220	126970420	413	14	10164	-1.1655	0.87809
+	SPHK1	17	76346567	76397860	113	12	10164	-1.1658	0.87814
+	C17orf89	17	81204311	81251298	124	17	10164	-1.1658	0.87815
-	ZNF566	19	36426034	36524902	306	26	10164	-1.1661	0.87821
+	SERF2	15	43742096	43806089	121	16	10164	-1.1665	0.87829
+	CHD2	15	92865321	93038007	397	26	10164	-1.1665	0.8783
+	SLC9A4	2	102438282	102543974	510	27	10164	-1.1673	0.87846

STRAND	SYMBOL	CHR	START	STOP	NSNPS	NPARAM	N	ZSTAT	P
-	OR2B2	6	27901185	27947396	206	35	10164	-1.1674	0.87847
-	RASL12	15	65035582	65111670	169	23	10164	-1.1675	0.8785
-	RFX7	15	56080533	56278536	475	15	10164	-1.1676	0.87851
-	FAT1	4	186577783	186761915	455	50	10164	-1.1677	0.87853
-	SART3	12	108512214	108596389	264	25	10164	-1.1678	0.87856
+	CLEC4D	12	8478482	8532366	58	8	10164	-1.1682	0.87863
-	NAA50	3	113706460	113781273	154	14	10164	-1.1682	0.87864
-	SCUBE2	11	9009500	9126603	231	47	10164	-1.1684	0.87868
+	FBXO38	5	148348935	148452836	230	22	10164	-1.1689	0.87877
+	FGD3	9	92912319	93046236	382	31	10164	-1.1691	0.87882
-	LOC283710	15	31211813	31265847	168	20	10164	-1.1691	0.87882
+	ZSWIM6	5	61297273	61556172	355	17	10164	-1.1695	0.87889
-	SPATA45	1	212820141	212882649	274	12	10164	-1.1695	0.87889
+	AKR1C4	10	5161809	5230633	347	15	10164	-1.1696	0.87891
-	RNF126	19	637526	698313	36	7	10164	-1.1696	0.87893
-	ANKS1B	12	98719904	100019727	3178	169	10164	-1.17	0.879
+	GP1BB	22	19688543	19734774	47	10	10164	-1.1703	0.87906
+	BTBD16	10	122235828	122348161	386	29	10164	-1.1706	0.87912
-	PHLDA3	1	201455479	201504171	145	27	10164	-1.171	0.8792
+	WBP4	13	41026561	41094006	195	20	10164	-1.1712	0.87925
+	GAD1	2	170778210	170871151	270	36	10164	-1.1718	0.87937
+	BIRC5	17	78179196	78235635	276	36	10164	-1.1725	0.87951
-	SLC2A12	6	133977581	134087668	189	30	10164	-1.1728	0.87956
-	AGAP5	10	73664275	73732796	84	12	10164	-1.1729	0.87958
+	ZCCHC3	20	262563	310321	215	24	10164	-1.173	0.8796
+	OSBPL2	20	62203485	62306213	294	27	10164	-1.1731	0.87963
+	ERGIC3	20	35507029	35567634	136	13	10164	-1.1731	0.87963
+	PDE7B	6	135773752	136205571	802	81	10164	-1.1735	0.8797
+	NPY	7	24249188	24301865	221	22	10164	-1.1741	0.87982
+	MYCN	2	15905438	15957007	118	15	10164	-1.1742	0.87984
+	ESYT3	3	138399573	138489942	112	8	10164	-1.1747	0.87994
+	NBEAL2	3	46944683	47019704	135	21	10164	-1.1752	0.88004
-	CDYL2	16	80587899	80840043	1148	84	10164	-1.1753	0.88005
-	TBC1D10B	16	30347101	30405258	8	2	10164	-1.1754	0.88009
-	ZNF232	17	5095734	5158102	165	22	10164	-1.1758	0.88016
+	OVCA2	17	2006983	2053431	116	20	10164	-1.176	0.88019
-	TOMM5	9	37578413	37627639	86	9	10164	-1.1761	0.88021
-	PTPRH	19	55171247	55244506	225	31	10164	-1.1763	0.88026
+	PEAR1	1	156858692	156926434	140	15	10164	-1.1763	0.88026
-	ABCA1	9	104771002	104963246	686	83	10164	-1.1769	0.88039
+	SETD1B	12	121768530	121842656	140	19	10164	-1.1774	0.88049
-	KCNB1	20	49353877	49519042	379	36	10164	-1.1777	0.88055
-	POLQ	3	121421420	121581006	355	28	10164	-1.1778	0.88057
-	FHL3	1	37986770	38040515	38	8	10164	-1.1779	0.88058
-	MFAP3L	4	169976597	170068028	143	10	10164	-1.1779	0.88058
+	GNMT	6	42904415	42973880	203	22	10164	-1.1781	0.88062
-	ZBTB4	17	7449366	7519249	258	24	10164	-1.1784	0.88069
-	CELSR1	22	46350834	46572502	516	48	10164	-1.1787	0.88073
-	SGK223	8	8307736	8421443	463	37	10164	-1.1787	0.88075
+	PLEKHG3	14	64669413	64756905	210	28	10164	-1.1793	0.88086
-	ANP32C	4	164187007	164232711	141	28	10164	-1.1794	0.88087
-	GDAP2	1	117853485	117964679	349	17	10164	-1.1801	0.88102
-	NPPB	1	11847464	11893935	192	24	10164	-1.1805	0.8811
-	DOCK7	1	62444726	62723368	451	17	10164	-1.1805	0.8811
+	CLYBL	13	99571664	99919459	992	85	10164	-1.1806	0.88111
-	MICAL1	6	109434062	109500968	157	19	10164	-1.1807	0.88113
-	ACOT7	1	6254272	6428766	517	22	10164	-1.1807	0.88115
+	LOC1027250	15	31179222	31229598	113	13	10164	-1.1809	0.88117
+	ZNF408	11	46665767	46715916	74	15	10164	-1.181	0.88119
-	ASIC5	4	155819729	155901273	265	25	10164	-1.181	0.8812
-	CTSB	8	11832524	11903137	315	20	10164	-1.1811	0.88121
+	LOC1027237	2	1513525	1566488	194	20	10164	-1.1812	0.88124
+	TEX264	3	51636175	51714323	88	15	10164	-1.1818	0.88136

STRAND	SYMBOL	CHR	START	STOP	NSNPS	NPARAM	N	ZSTAT	P
-	MFHAS1	8	8773354	8928621	718	46	10164	-1.1831	0.88162
+	ZNF595	4	18285	98211	28	2	10164	-1.1833	0.88166
+	WDR64	1	241617278	241812745	603	62	10164	-1.1834	0.88167
+	ARPC4	3	9757495	9817105	172	25	10164	-1.184	0.88179
-	GNPDA2	4	44672411	44761634	236	13	10164	-1.184	0.8818
-	CNIH1	14	54416929	54476430	166	15	10164	-1.1841	0.88182
-	ERI2	16	20770193	20841473	96	10	10164	-1.1845	0.88189
+	GALNT2	1	230022789	230292130	599	49	10164	-1.185	0.88198
-	A4GALT	22	42682117	42756301	393	36	10164	-1.1853	0.88205
+	LRRC30	18	7196139	7242044	41	4	10164	-1.1854	0.88208
+	OR4D10	11	59442430	59488365	113	13	10164	-1.1857	0.88212
-	BBIP1	10	110888730	110954366	136	20	10164	-1.1858	0.88215
+	PVRL3	3	111036759	111279283	499	43	10164	-1.1864	0.88226
+	OR52D1	11	5453685	5499749	211	27	10164	-1.1864	0.88226
+	OR1A2	17	3162517	3208448	135	23	10164	-1.1864	0.88227
+	TCF23	2	27113933	27163576	85	13	10164	-1.1865	0.88228
-	IP6K3	6	33711637	33781985	436	43	10164	-1.187	0.88239
-	SHB	9	37905898	38104213	646	34	10164	-1.1871	0.8824
+	GARS	7	30559565	30644033	136	15	10164	-1.1884	0.88265
+	DDA1	19	17274528	17333298	248	26	10164	-1.1883	0.88265
-	SEPP1	5	42789880	42846922	182	17	10164	-1.1885	0.88268
+	ANGPTL7	1	11154289	11205981	140	9	10164	-1.1885	0.88268
-	LOC1019295	9	93657342	93708250	235	30	10164	-1.1885	0.88268
-	ZBED3	5	77066707	77122205	147	25	10164	-1.1891	0.88281
-	PROCA1	17	28693197	28747071	54	10	10164	-1.1893	0.88285
+	PDE8A	15	84945513	85149145	505	27	10164	-1.1897	0.88291
+	STOX1	10	68792537	68905453	304	25	10164	-1.1896	0.88291
-	GCSAM	3	112110841	112171705	187	31	10164	-1.1897	0.88292
-	IZUMO3	9	24533214	24580930	47	19	10164	-1.1898	0.88294
+	EEF2K	16	22171271	22298745	163	16	10164	-1.1901	0.88299
-	SLC29A2	11	66352521	66407476	143	13	10164	-1.1901	0.883
+	ARMC3	10	22893024	23048523	323	27	10164	-1.1903	0.88304
-	HSD17B13	4	87293789	87357906	162	26	10164	-1.1905	0.88308
+	CCDC171	9	15517874	15981899	1270	24	10164	-1.1906	0.8831
+	PPIE	1	39703845	39773914	120	17	10164	-1.1907	0.88311
+	POLR2A	17	7449379	7524618	285	26	10164	-1.1909	0.88315
+	FAM162A	3	122349176	122420114	209	25	10164	-1.1913	0.88323
+	HHIPL1	14	99569438	99690569	40	7	10164	-1.1915	0.88326
+	UNC119B	12	120675435	120733640	175	22	10164	-1.1917	0.88332
+	PARP8	5	50630899	50856522	554	30	10164	-1.1918	0.88333
-	TSPAN3	15	77034017	77106228	165	28	10164	-1.1918	0.88334
+	AKR1C1	10	4928262	4987966	238	22	10164	-1.1921	0.88339
-	PCSK4	19	1471428	1525888	88	11	10164	-1.1922	0.88341
-	SLC22A17	14	23336311	23389976	122	23	10164	-1.1923	0.88342
-	SUMF1	3	4055934	4502282	1722	74	10164	-1.1923	0.88343
+	TLR2	4	153649256	153716091	174	26	10164	-1.1924	0.88345
-	HNRNPA2B1	7	26179927	26235793	135	25	10164	-1.1925	0.88347
+	RRS1	8	66394028	66440733	126	16	10164	-1.1926	0.88348
+	FAM221A	7	23644463	23713249	170	20	10164	-1.1928	0.88353
-	C1orf216	1	35703875	35754189	59	7	10164	-1.193	0.88356
+	NOP58	2	202230792	202313661	167	15	10164	-1.193	0.88357
-	TMEM86B	19	55216634	55264264	115	17	10164	-1.1931	0.88358
+	FAM117B	2	202600178	202779757	271	37	10164	-1.1932	0.88361
+	SEMA3B	3	50232558	50287171	73	13	10164	-1.1933	0.88363
+	TMA7	3	48405276	48454129	77	8	10164	-1.1934	0.88365
-	ALX3	1	110050375	110105700	109	15	10164	-1.1935	0.88366
-	YPEL1	22	21687536	21770834	192	26	10164	-1.194	0.88375
-	MYBBP1A	17	4528896	4590386	104	16	10164	-1.1941	0.88378
+	OR5D16	11	55803752	55849738	158	17	10164	-1.1944	0.88384
-	DUSP26	8	33581330	33635028	110	14	10164	-1.1946	0.88387
+	KAT5	11	65676996	65729606	216	28	10164	-1.1947	0.8839
-	ANKRD63	15	40268369	40317987	141	29	10164	-1.1952	0.88399
-	PTPRT	20	42062752	43224917	2880	237	10164	-1.1953	0.88401

STRAND	SYMBOL	CHR	START	STOP	NSNPS	NPARAM	N	ZSTAT	P
+	TMEM171	5	73085561	73141817	135	20	10164	-1.1955	0.88406
-	BSCL2	11	62680262	62744619	98	9	10164	-1.1957	0.8841
-	CGGBP1	3	88041950	88184866	499	19	10164	-1.1958	0.88411
-	ALDH7A1	5	126531841	126630390	279	34	10164	-1.1959	0.88414
+	PIP5K1A	1	151162954	151259535	119	14	10164	-1.1959	0.88414
-	LY75-CD302	2	159758628	159939756	563	37	10164	-1.1968	0.88431
-	HTR2B	2	231098230	231160778	76	12	10164	-1.1969	0.88434
-	OR5M9	11	56452469	56498401	166	18	10164	-1.1971	0.88437
+	ZFP2	5	178860915	178943209	212	18	10164	-1.1983	0.8846
+	C1orf137	1	116659112	116716603	58	9	10164	-1.1987	0.88467
-	TDRKH	1	151760264	151825534	188	22	10164	-1.1989	0.88473
-	CNPY1	7	155491258	155568844	178	30	10164	-1.1995	0.88484
-	SLIT1	10	96988038	97220926	610	75	10164	-1.1996	0.88486
+	TIGD3	11	65319811	65367613	108	19	10164	-1.1998	0.88489
-	PSMB5	14	23006543	23070220	129	30	10164	-1.1999	0.8849
+	KIAA2012	2	202038253	202215195	481	40	10164	-1.1999	0.8849
-	HIVEP2	6	142741467	142980201	635	60	10164	-1.2009	0.88511
+	PPARG	3	12252850	12481013	621	46	10164	-1.2013	0.88519
+	HMGN2	1	26437411	26486642	75	12	10164	-1.2016	0.88523
+	ZNF141	4	302807	383959	124	6	10164	-1.2018	0.88528
-	IL22RA2	6	137133820	137208648	148	26	10164	-1.202	0.88532
-	PITX2	4	110607423	110677123	109	9	10164	-1.2021	0.88534
-	KRT38	17	41426369	41476344	294	18	10164	-1.2022	0.88535
-	DGCR2	22	19026282	19157454	477	19	10164	-1.2027	0.88546
-	CACNB4	2	151822771	152134988	744	46	10164	-1.2036	0.88562
+	FBXW12	3	48337175	48404791	152	14	10164	-1.2038	0.88567
-	IZUMO1	19	48730888	48782650	81	9	10164	-1.2045	0.8858
-	HYDIN	16	70792084	71265722	354	27	10164	-1.205	0.88591
+	SLC12A5	20	45986690	46070150	266	31	10164	-1.2053	0.88595
-	PXMP4	20	33692744	33755330	121	22	10164	-1.2054	0.88598
+	CCNB1	5	69132010	69188245	149	14	10164	-1.2056	0.88602
+	BMPER	7	33869911	34165872	447	49	10164	-1.2056	0.88602
+	TINAGL1	1	31541384	31597686	37	10	10164	-1.2057	0.88604
+	NLRP6	11	243365	295388	170	21	10164	-1.206	0.88609
-	SEL1L2	20	13839188	14030333	529	39	10164	-1.2066	0.8862
-	PAX8	2	113205997	113313950	345	23	10164	-1.207	0.88629
-	HSPA8	11	123047492	123097366	214	21	10164	-1.2074	0.88636
+	NR4A3	9	99786855	99876893	63	5	10164	-1.2074	0.88636
+	LOC402096	2	106334738	106382422	109	15	10164	-1.2091	0.88668
+	CCDC62	12	122739509	122837385	126	3	10164	-1.2095	0.88677
+	ACCS	11	44031179	44094237	286	47	10164	-1.2106	0.88697
+	PHF2	9	93541627	93689587	661	37	10164	-1.2109	0.88703
-	HNRNPF	10	43375617	43444248	201	16	10164	-1.2114	0.88712
-	TMEM161B	5	88175575	88303879	236	28	10164	-1.2116	0.88716
+	PFKM	12	48070402	48156404	303	22	10164	-1.2119	0.88723
-	SCN10A	3	38686891	38829010	482	44	10164	-1.212	0.88724
-	VPRBP	3	51385867	51540618	270	22	10164	-1.212	0.88725
+	GAL	11	68649515	68701175	181	29	10164	-1.212	0.88725
-	SLC52A1	17	5022602	5070458	197	28	10164	-1.2123	0.88731
-	SLC8A2	19	47418017	47507050	163	18	10164	-1.2125	0.88734
+	COPS8	2	237050441	237108846	80	10	10164	-1.2126	0.88736
+	UNC80	2	209736993	210009300	472	35	10164	-1.2128	0.88739
-	RUNX3	1	24889511	25000157	287	39	10164	-1.2128	0.8874
+	CACNA1E	1	181448311	181816784	856	77	10164	-1.2142	0.88767
+	TBX19	1	168246040	168324426	149	15	10164	-1.2143	0.88768
-	LOC1001303	3	63739816	63820022	231	30	10164	-1.2144	0.88771
-	RRAD	16	66911679	66960536	64	13	10164	-1.2146	0.88774
+	FAM134A	2	219143217	219195475	98	10	10164	-1.2147	0.88775
+	NFYA	6	41037968	41112407	229	15	10164	-1.215	0.88782
+	KLRF1	12	9792392	9855005	151	22	10164	-1.215	0.88782
-	GATS	7	100190653	100307232	188	22	10164	-1.2151	0.88784
+	NXPH3	17	49540936	49593810	105	25	10164	-1.2152	0.88785
-	TMEM192	4	165066078	165147913	253	24	10164	-1.2156	0.88794

STRAND	SYMBOL	CHR	START	STOP	NSNPS	NPARAM	N	ZSTAT	P
-	ANKRD44	2	196952617	197345797	736	48	10164	-1.2157	0.88795
-	RSPO2	8	107889316	108118685	586	36	10164	-1.2159	0.88798
-	ZNF337	20	25663195	25731904	200	13	10164	-1.2164	0.88808
-	MYO5C	15	52182318	52330798	528	51	10164	-1.2165	0.88811
-	MTOR	1	11096531	11297557	509	17	10164	-1.2167	0.88813
+	OR1A1	17	3180621	3226550	200	20	10164	-1.217	0.8882
+	KIF23	15	69379246	69458427	111	12	10164	-1.217	0.8882
+	OLFM4	13	52993741	53062061	225	52	10164	-1.217	0.8882
-	OR2S2	9	35947108	35993154	160	17	10164	-1.2172	0.88824
-	LOC1006528	5	99512014	99561049	132	13	10164	-1.2172	0.88824
-	SLC45A4	8	141197166	141343310	452	42	10164	-1.2176	0.88831
+	WDR11	10	120816175	120919526	396	25	10164	-1.2176	0.88832
-	NKX2-2	20	21489260	21549026	129	22	10164	-1.218	0.88838
+	MR1	1	180998379	181071938	184	26	10164	-1.218	0.88839
+	RAN	12	130836994	130887678	171	18	10164	-1.218	0.88839
-	KRT33A	17	41335705	41385812	82	15	10164	-1.2182	0.88842
-	ATP13A1	19	19635198	19702823	221	34	10164	-1.2182	0.88842
+	SPACA1	6	88012035	88076832	25	2	10164	-1.2182	0.88842
-	LINGO4	1	151790264	151840442	121	22	10164	-1.2182	0.88843
+	BRE	2	27855615	28348901	1102	50	10164	-1.2185	0.88848
-	OR2K2	9	111317370	111365173	108	20	10164	-1.2189	0.88856
+	DENR	12	122717821	122781064	54	3	10164	-1.2194	0.88865
-	RRAGD	6	89354616	89447276	263	35	10164	-1.2195	0.88867
-	ATG5	6	106174476	106360820	552	34	10164	-1.2198	0.88873
-	IFT172	2	27434373	27524811	154	17	10164	-1.22	0.88876
-	OR4C46	11	54593069	54638998	173	17	10164	-1.2201	0.88879
-	DLX3	17	49967079	50030224	211	26	10164	-1.2205	0.88887
+	CAPN1	11	65146215	65222006	187	35	10164	-1.2207	0.88891
-	FANCF	11	22612533	22660841	110	12	10164	-1.2207	0.88891
-	CDC42EP5	19	54455025	54508290	90	8	10164	-1.2209	0.88894
+	BRPF1	3	9696729	9758015	106	31	10164	-1.2212	0.88899
-	PPP1R35	7	100425277	100471615	62	9	10164	-1.2213	0.88902
+	CDKN1B	12	12682270	12732383	116	18	10164	-1.2215	0.88904
-	AK6	5	69341726	69405013	100	13	10164	-1.2216	0.88908
-	KRTAP2-1	17	41036541	41082316	202	18	10164	-1.2219	0.88912
-	ATAD2	8	123309850	123431465	143	15	10164	-1.222	0.88914
+	ARPP21	3	35604174	35804496	481	34	10164	-1.2224	0.88922
-	LOC1053781	6	169320544	169375581	93	7	10164	-1.2225	0.88924
-	PIANP	12	6680934	6735843	108	23	10164	-1.2229	0.88932
-	MMP20	11	102566835	102660332	516	44	10164	-1.2231	0.88936
-	GRSF1	4	70805782	70878274	135	10	10164	-1.2232	0.88937
+	GPX4	19	1068926	1116789	185	30	10164	-1.2233	0.8894
+	ARHGAP33	19	35740515	35798822	202	28	10164	-1.2236	0.88945
+	CDK18	1	205469556	205542793	251	46	10164	-1.224	0.88953
-	HEATR6	17	60033191	60113931	78	3	10164	-1.224	0.88953
+	ING2	4	183470050	183522428	43	7	10164	-1.2242	0.88955
+	KRTCAP3	2	27407351	27456481	82	9	10164	-1.2241	0.88955
-	KCTD21	11	78161243	78223817	121	18	10164	-1.2254	0.88979
+	MVP	16	29785394	29858039	1	1	10164	-1.2255	0.88981
-	BRMS1	11	66327333	66380111	127	18	10164	-1.2256	0.88982
-	C1orf198	1	230827119	230904589	281	45	10164	-1.2265	0.89
+	HMGXB4	22	35206535	35305807	55	6	10164	-1.2271	0.8901
+	ALKBH5	17	18148553	18219954	192	20	10164	-1.2275	0.89019
+	OLIG2	21	32990908	33039196	153	28	10164	-1.2277	0.89021
-	FBXO33	14	39386373	39467500	250	24	10164	-1.2282	0.89032
-	UFSP2	4	185389540	185460985	113	10	10164	-1.2286	0.8904
+	CCS	11	66558162	66616019	124	21	10164	-1.2288	0.89043
+	PCGF5	10	91128012	91294331	380	34	10164	-1.229	0.89046
+	HTR3B	11	113863923	113956561	214	29	10164	-1.2291	0.89048
+	PNPLA6	19	7499152	7571767	245	29	10164	-1.2297	0.8906
+	UNC13C	15	53943203	54638608	3366	159	10164	-1.2301	0.89068
-	TMEM130	7	98836488	98905050	64	14	10164	-1.2306	0.89076
-	ZNF384	12	6656477	6724572	99	25	10164	-1.2307	0.89078

STRAND	SYMBOL	CHR	START	STOP	NSNPS	NPARAM	N	ZSTAT	P
-	ST6GAL2	2	106784128	106922265	439	34	10164	-1.2308	0.8908
-	CCDC58	3	122349589	122418281	203	24	10164	-1.2309	0.89083
+	MSANTD3	9	100392213	100461734	108	13	10164	-1.2314	0.89091
-	PPIP5K1	15	43523462	43625272	100	13	10164	-1.2314	0.89092
+	D2HGDH	2	241699579	241778816	19	3	10164	-1.2314	0.89092
-	LOC1019289	17	2714411	2784562	155	19	10164	-1.2317	0.89096
-	RMDN1	8	86457398	86549338	235	13	10164	-1.2325	0.89113
-	SPRR2B	1	153060228	153106608	184	15	10164	-1.2328	0.89118
+	VENTX	10	133202904	133251930	118	15	10164	-1.2333	0.89126
+	NQO2	6	2953640	3029876	419	36	10164	-1.2337	0.89134
+	CEP192	18	12956321	13135053	566	30	10164	-1.234	0.89139
-	SBF1	22	50434630	50515296	265	46	10164	-1.234	0.8914
-	NCKAP5L	12	49781139	49863750	240	28	10164	-1.2343	0.89146
+	PREX2	8	67916983	68241662	956	68	10164	-1.2343	0.89146
+	KCTD5	16	2647494	2719030	25	2	10164	-1.2349	0.89156
+	BZW1	2	200776544	200833846	139	28	10164	-1.2351	0.8916
-	CD207	2	70815208	70871601	217	17	10164	-1.2354	0.89167
+	TEF	22	41332333	41409328	204	20	10164	-1.2358	0.89174
-	TYW1B	7	72564507	72863234	526	11	10164	-1.2361	0.89179
-	RPS15A	16	18772955	18825334	71	4	10164	-1.2362	0.89181
+	TMC2	20	2501607	2651869	568	47	10164	-1.2374	0.89203
-	RPS2	16	1952061	1999826	170	21	10164	-1.2376	0.89206
+	BTBD8	1	92045305	92157840	160	16	10164	-1.2376	0.89207
-	SRSF1	17	57990919	58042346	6	4	10164	-1.2378	0.8921
+	AOC1	7	150791393	150871291	413	28	10164	-1.238	0.89214
-	KRTAP4-12	17	41113091	41159167	161	9	10164	-1.2386	0.89225
+	CYP4V2	4	186156411	186223463	244	26	10164	-1.2387	0.89228
-	CEP76	18	12651750	12737777	259	19	10164	-1.2392	0.89236
+	HLX	1	220844401	220895059	158	22	10164	-1.24	0.89251
+	PYGB	20	25213070	25308012	298	15	10164	-1.24	0.89252
-	GMFB	14	54464484	54524067	124	8	10164	-1.2403	0.89257
+	GYLTL1B	11	45885974	45939096	84	16	10164	-1.2413	0.89275
-	CCDC169-SC	13	36158208	36332855	590	19	10164	-1.2415	0.89278
-	LOC1019286	4	1313178	1362620	194	19	10164	-1.2418	0.89285
-	XRN1	3	142296607	142483062	278	25	10164	-1.2424	0.89295
+	ZNF397	18	35205718	35268433	121	17	10164	-1.2424	0.89296
-	SVOPL	7	138584285	138736354	456	70	10164	-1.2428	0.89304
+	CEL	9	133026978	133081863	141	17	10164	-1.2429	0.89305
-	ATP1A3	19	41956582	42029276	60	17	10164	-1.2437	0.8932
-	ATP6V0A4	7	138696294	138834632	510	75	10164	-1.2438	0.89321
+	PSENEN	19	35710577	35757155	116	21	10164	-1.2445	0.89335
+	ZBTB7B	1	154967627	155028525	92	19	10164	-1.2449	0.89341
+	IVD	15	40370485	40445948	210	25	10164	-1.2452	0.89347
+	HDAC11	3	13445171	13516424	165	22	10164	-1.2457	0.89356
-	APBA1	9	69417532	69708617	597	42	10164	-1.2458	0.89358
-	GPX2	14	64929152	64977905	141	19	10164	-1.2462	0.89366
+	ADAM11	17	44724037	44791846	161	24	10164	-1.2468	0.89376
+	SEPT5	22	19679464	19733322	68	12	10164	-1.2468	0.89377
+	FAM160A1	4	151374156	151680503	635	14	10164	-1.2468	0.89377
+	CRTC3	15	90494886	90655345	662	40	10164	-1.247	0.8938
-	TTC32	2	19886753	19936986	139	20	10164	-1.2471	0.89383
+	IFT81	12	110089335	110228795	155	23	10164	-1.2472	0.89384
+	LOC400863	21	33912934	33975164	46	7	10164	-1.2481	0.894
+	RUFY3	4	70669686	70818622	280	17	10164	-1.2483	0.89404
-	U2AF1L4	19	35732527	35780442	161	25	10164	-1.2486	0.89409
+	CAPNS1	19	36104926	36160353	169	38	10164	-1.2491	0.89419
-	SKAP1	17	48123438	48465235	1031	47	10164	-1.2492	0.8942
-	FBXO48	2	68452366	68502316	99	4	10164	-1.2497	0.89429
+	HCFC2	12	104029458	104116525	237	40	10164	-1.25	0.89435
+	PPP1R36	14	64514902	64599386	228	26	10164	-1.25	0.89435
-	ZNF732	4	260675	340321	103	8	10164	-1.2504	0.89443
-	KCNQ2	20	63390208	63507640	491	48	10164	-1.2505	0.89444
+	ST7	7	116918327	117240023	526	60	10164	-1.2506	0.89446

STRAND	SYMBOL	CHR	START	STOP	NSNPS	NPARAM	N	ZSTAT	P
+	OR3A3	17	3385424	3431666	152	22	10164	-1.2507	0.89447
-	OR5P3	11	7815037	7860972	244	16	10164	-1.251	0.89454
-	KRTAP4-2	17	41167446	41213208	142	7	10164	-1.2513	0.89459
+	DMP1	4	87615302	87674361	176	25	10164	-1.2515	0.89462
+	LGALS16	19	39620894	39670647	129	10	10164	-1.2515	0.89462
+	OTOR	20	16713353	16762164	164	38	10164	-1.2516	0.89464
+	TSSK2	22	19095808	19142623	184	15	10164	-1.2524	0.89479
+	THNSL2	2	88135295	88196637	151	17	10164	-1.2524	0.89479
-	TMEM232	5	110377273	110773921	1050	33	10164	-1.2529	0.89487
-	TAF7	5	141308490	141355784	107	16	10164	-1.2529	0.89489
+	C5orf51	5	41869289	41931636	98	12	10164	-1.2535	0.89498
-	SKA2	17	59099947	59190439	132	20	10164	-1.2535	0.89499
-	UNC5CL	6	41016901	41074199	148	9	10164	-1.2541	0.89509
-	RAB15	14	64935814	65007290	163	26	10164	-1.254	0.89509
-	UNC119	17	28536707	28587628	42	15	10164	-1.2541	0.8951
+	OR8J1	11	56325215	56371288	167	14	10164	-1.2547	0.8952
+	BANF2	20	17658675	17745872	196	23	10164	-1.255	0.89526
-	FGF4	11	69749043	69810403	313	50	10164	-1.2554	0.89533
-	LOC1053693	11	67748563	67797743	222	24	10164	-1.2555	0.89534
+	SYNDIG1	20	24434199	24676617	596	63	10164	-1.2555	0.89535
-	TBX3	12	114660254	114719164	170	38	10164	-1.2559	0.89542
-	OXCT1	5	41720065	41905689	270	22	10164	-1.2563	0.8955
+	LOC1053742	3	192531796	192584213	79	13	10164	-1.2564	0.89552
-	CYB5RL	1	54162336	54235073	208	34	10164	-1.2569	0.89561
+	SIX3	2	44906729	44976751	325	46	10164	-1.2572	0.89565
-	CADPS2	7	122308424	122921759	1327	64	10164	-1.2579	0.89579
+	PHACTR2	6	143573180	143841185	1044	79	10164	-1.2582	0.89584
+	S100Z	5	76815001	76962882	459	45	10164	-1.2582	0.89584
-	SMAD2	18	47823095	47966193	243	20	10164	-1.2584	0.89587
-	F11R	1	160985211	161056343	151	11	10164	-1.2584	0.89587
-	ROBO1	3	78587238	79802998	3064	126	10164	-1.2584	0.89588
-	CSTF3	11	33074584	33196491	221	9	10164	-1.2586	0.89592
+	UPF1	19	18796935	18878232	150	26	10164	-1.2587	0.89593
+	SMARCA4	19	10925922	11072282	373	34	10164	-1.2588	0.89594
+	TMEM139	7	143249899	143298049	6	2	10164	-1.2588	0.89594
+	COQ7	16	19032595	19090095	137	13	10164	-1.2594	0.89605
+	DSPP	4	87573529	87626873	157	28	10164	-1.2598	0.89613
+	ADAMTSL2	9	133497164	133585519	105	18	10164	-1.26	0.89617
+	DYDC2	10	80309745	80378118	221	16	10164	-1.2606	0.89627
-	MXRA8	1	1342689	1398541	154	6	10164	-1.2614	0.89641
+	NFKB2	10	102359110	102412529	87	26	10164	-1.2617	0.89648
+	CAMK1G	1	209548700	209623939	123	11	10164	-1.2625	0.89662
-	PPIL6	6	109380215	109476171	257	23	10164	-1.2626	0.89662
+	MAP2	2	209389047	209744118	705	24	10164	-1.263	0.8967
-	UBE2G2	21	44758580	44836836	263	29	10164	-1.2637	0.89682
-	CLTB	5	176382455	176451569	159	37	10164	-1.2638	0.89684
-	GPR4	19	45579764	45637208	167	36	10164	-1.2638	0.89685
+	HTT	4	3039510	3253960	557	30	10164	-1.2639	0.89688
-	BAZ1A	14	34742731	34910647	480	24	10164	-1.2644	0.89696
+	MUC20	3	195685882	195743553	97	4	10164	-1.2647	0.89702
-	POLR3A	10	77965149	78064540	90	5	10164	-1.2649	0.89704
+	SPRR3	1	152966747	153013856	229	8	10164	-1.2651	0.89708
-	WDR83OS	19	12658067	12704651	17	4	10164	-1.2656	0.89717
+	PCNXL3	11	65579322	65647439	167	39	10164	-1.2658	0.8972
+	DGKH	13	42005036	42266584	1086	67	10164	-1.2658	0.89721
+	RXFP1	4	158486714	158663372	136	15	10164	-1.266	0.89724
+	TET1	10	68525360	68704487	548	26	10164	-1.2664	0.89732
+	ZCCHC9	5	81266583	81323344	127	15	10164	-1.2669	0.89741
+	PPIG	2	169549340	169651406	336	17	10164	-1.267	0.89742
-	LOC1053717	17	32131226	32178117	2	1	10164	-1.2671	0.89744
+	ACAD10	12	111651053	111767107	168	24	10164	-1.2678	0.89757
-	KLHL8	4	87150103	87255608	245	37	10164	-1.2682	0.89764
+	FAM19A5	22	48454460	48761935	1478	110	10164	-1.2683	0.89766

STRAND	SYMBOL	CHR	START	STOP	NSNPS	NPARAM	N	ZSTAT	P
-	MECOM	3	169073499	169698786	1953	137	10164	-1.2685	0.89769
-	KCNK5	6	39178971	39264475	287	46	10164	-1.2685	0.8977
+	RIPK2	8	89722733	89801064	304	19	10164	-1.2692	0.89781
+	DDX50	10	68863972	68956847	207	20	10164	-1.2692	0.89781
+	THEM6	8	142692203	142746932	144	13	10164	-1.2692	0.89782
-	USP44	12	95506560	95586529	310	38	10164	-1.2694	0.89785
-	SLC26A1	4	969073	1029217	226	40	10164	-1.27	0.89796
+	ACADS	12	120690738	120750008	193	23	10164	-1.2706	0.89806
+	IER5	1	181053502	181100843	134	13	10164	-1.2709	0.89811
-	HCFC1R1	16	3012620	3059296	11	2	10164	-1.271	0.89814
+	RPS24	10	77998760	78066813	55	3	10164	-1.2712	0.89817
-	GJA3	13	20128255	20196385	140	22	10164	-1.2717	0.89826
-	SH2D1B	1	162385266	162447138	229	24	10164	-1.272	0.89831
-	WIZ	19	15411507	15484956	65	13	10164	-1.2722	0.89835
-	PIGN	18	62034224	62222118	976	37	10164	-1.2723	0.89837
-	TLCD1	17	28714348	28762935	61	11	10164	-1.2724	0.89838
-	LOC1019274	1	228350910	228403332	199	27	10164	-1.2724	0.89839
+	HNRNPM	19	8409575	8499118	112	9	10164	-1.2726	0.89842
+	ATXN7L2	1	109448911	109502804	80	3	10164	-1.2726	0.89842
-	EVI2A	17	31306410	31356749	58	4	10164	-1.2732	0.89853
+	TNKS2	10	91763394	91875475	237	16	10164	-1.2733	0.89854
+	UPK1B	3	119138578	119215153	334	42	10164	-1.2736	0.8986
+	VWA3B	2	98052132	98340681	652	49	10164	-1.2736	0.8986
-	ZNF697	1	119609377	119683170	220	35	10164	-1.2737	0.89861
+	LOC1053710	16	4219514	4265336	83	15	10164	-1.274	0.89867
-	ZNF683	1	26351632	26409533	200	23	10164	-1.2741	0.89868
-	IGSF22	11	18694305	18761230	187	29	10164	-1.2741	0.89869
-	CD248	11	66304487	66352044	113	20	10164	-1.2744	0.89874
+	NDUFB5	3	179569787	179634500	116	10	10164	-1.2752	0.89889
-	RFPL2	22	32180435	32242236	256	20	10164	-1.2753	0.8989
-	GABRR3	3	97976683	98070304	297	28	10164	-1.2753	0.8989
-	NFIB	9	14071843	14433983	731	99	10164	-1.276	0.89902
-	NAF1	4	163099133	163201921	206	25	10164	-1.2761	0.89903
+	MARVELD2	5	69380112	69452063	126	8	10164	-1.276	0.89903
+	C1QC	1	22608625	22658110	199	25	10164	-1.2769	0.89918
+	SERPINB5	18	63441911	63515085	337	27	10164	-1.2774	0.89928
+	ELP4	11	31474729	31795252	397	11	10164	-1.2781	0.89939
-	EML2	19	45599400	45680601	305	34	10164	-1.2782	0.89941
+	LYRM1	16	20863805	20935006	100	10	10164	-1.2784	0.89945
-	LOC1053768	1	26155313	26204802	124	18	10164	-1.2784	0.89945
-	TPK1	7	144441941	144871411	776	62	10164	-1.2795	0.89964
+	ZNF230	19	43967925	44023926	222	28	10164	-1.2796	0.89966
-	GATA5	20	62453497	62510970	142	34	10164	-1.2797	0.89967
-	ANKRD54	22	37820854	37883621	82	12	10164	-1.2798	0.8997
-	C11orf98	11	62652817	62700216	65	10	10164	-1.2804	0.89979
-	RHOF	12	121767754	121828688	124	18	10164	-1.2804	0.8998
-	KRTAP11-1	21	30870644	30916555	91	16	10164	-1.2805	0.89982
-	LOC1053760	9	38610801	38657698	142	21	10164	-1.2805	0.89982
+	LRCH1	13	46518138	46763041	442	38	10164	-1.2806	0.89983
-	YWHAQ	2	9573967	9666055	97	11	10164	-1.2809	0.89988
-	ZNF626	19	20609939	20696596	426	19	10164	-1.2809	0.89988
+	LOC1019284	6	16725058	16774994	151	29	10164	-1.2809	0.89989
+	MRPL34	19	17257778	17316847	250	30	10164	-1.2811	0.89992
-	BMP10	2	68855481	68906517	124	15	10164	-1.2815	0.89999
-	ADAMTS12	5	33513535	33927194	993	71	10164	-1.2824	0.90015
+	GAP43	3	115588304	115731487	289	65	10164	-1.2826	0.90018
-	USF1	1	161029251	161080979	90	13	10164	-1.2826	0.90018
+	RCN2	15	76896621	76960260	123	22	10164	-1.2826	0.90019
-	ARHGEF11	1	156924840	157081640	509	25	10164	-1.283	0.90026
-	LOC1053715	17	2696477	2743492	52	10	10164	-1.2837	0.90037
+	MBD3L1	19	8807593	8853340	95	8	10164	-1.2841	0.90045
+	CHMP5	9	33229879	33292069	78	8	10164	-1.2845	0.90052
-	TTC29	4	146697027	146980882	857	46	10164	-1.2845	0.90052

STRAND	SYMBOL	CHR	START	STOP	NSNPS	NPARAM	N	ZSTAT	P
-	IER5L	9	129165552	129213261	55	9	10164	-1.2847	0.90055
-	ABCA6	17	69068089	69176992	205	14	10164	-1.2849	0.90058
+	C3orf38	3	88114743	88167965	156	13	10164	-1.2849	0.90059
+	BTAF1	10	91888769	92040998	315	19	10164	-1.2851	0.90063
+	SNRPF	12	95823931	95876460	192	25	10164	-1.2861	0.9008
+	HNMT	2	137929238	138026364	237	26	10164	-1.2866	0.90088
+	PROSER3	19	35723143	35779371	178	27	10164	-1.2869	0.90093
-	IGFBPL1	9	38396528	38459447	271	26	10164	-1.2873	0.90101
+	ZAP70	2	97678568	97754327	187	28	10164	-1.2874	0.90103
-	SFRP1	8	41251957	41344471	195	28	10164	-1.2875	0.90104
+	CCDC150	2	196604632	196742806	311	14	10164	-1.2875	0.90104
+	APOBEC2	6	41018201	41074891	149	9	10164	-1.2877	0.90108
-	LOC1019274	2	64512189	64559611	118	14	10164	-1.2885	0.90121
+	MRFAP1	4	6605091	6652745	86	11	10164	-1.2886	0.90122
-	NMI	2	151260465	151324916	277	20	10164	-1.2886	0.90123
+	FLVCR1	1	212823255	212909363	391	12	10164	-1.2886	0.90123
-	TMPRSS2	21	41454551	41543158	404	40	10164	-1.289	0.90129
-	DGKB	7	14135049	15009777	2507	166	10164	-1.2892	0.90134
+	EID1	15	48843093	48890183	112	16	10164	-1.2893	0.90136
-	CCDC169	13	36217042	36332855	458	7	10164	-1.2894	0.90136
+	POLR2G	11	62726539	62776715	81	10	10164	-1.2902	0.90152
-	SLC9B2	4	103010230	103112371	173	25	10164	-1.2905	0.90156
-	C1QTNF3	5	34007858	34078266	152	18	10164	-1.2909	0.90162
+	KCNK13	14	90026765	90195857	555	52	10164	-1.2912	0.90168
+	MAMDC4	9	136817367	136870799	67	10	10164	-1.2912	0.90169
+	GBF1	10	102210498	102392899	286	33	10164	-1.2913	0.90171
-	MTRF1	13	41206369	41298797	212	18	10164	-1.2914	0.90171
+	NCAPH2	22	50473215	50534780	160	35	10164	-1.2915	0.90174
-	KIF28P	1	246760907	246874587	306	30	10164	-1.2915	0.90174
+	B4GALNT2	17	49097460	49179989	284	35	10164	-1.2916	0.90176
+	ALDH2	12	111731887	111819985	126	17	10164	-1.2918	0.90179
-	HINT1	5	131149283	131200348	77	8	10164	-1.292	0.90181
+	OR2A25	7	144039220	144085152	158	12	10164	-1.2921	0.90184
-	RGPD6	2	110503802	110645888	26	6	10164	-1.2924	0.9019
+	KIAA1919	6	111224279	111279060	143	28	10164	-1.2927	0.90194
+	PARD6A	16	67625948	67672778	51	17	10164	-1.2931	0.90201
-	IGFLR1	19	35729250	35777450	157	25	10164	-1.2936	0.9021
-	IER3IP1	18	47145019	47211374	261	26	10164	-1.2938	0.90214
-	BAG1	9	33242471	33299761	57	8	10164	-1.2944	0.90224
+	GZMK	5	54989279	55044132	144	18	10164	-1.2949	0.90233
-	ZNF777	7	149421363	149495962	136	26	10164	-1.2956	0.90245
+	LENG8	19	54413719	54472046	57	6	10164	-1.2957	0.90246
-	TNFRSF13C	22	41915032	41961817	133	14	10164	-1.2958	0.90248
-	FERD3L	7	19134782	19180421	223	21	10164	-1.2959	0.90249
-	FBF1	17	75900537	75976038	147	14	10164	-1.2962	0.90255
+	SPDYE3	7	100271812	100332196	67	12	10164	-1.2962	0.90255
-	NUMA1	11	71992864	72115693	307	15	10164	-1.2965	0.90259
+	MUC3A	7	100914117	100978347	175	34	10164	-1.2967	0.90263
-	MRPL47	3	179578465	179639646	112	11	10164	-1.2971	0.9027
+	CTDSPL	3	37827178	37994469	364	28	10164	-1.2983	0.9029
+	OR2V2	5	181119943	181165890	132	20	10164	-1.2984	0.90292
+	TRAF5	1	211291579	211384946	184	24	10164	-1.2984	0.90293
+	OR2C1	16	3320889	3366924	191	28	10164	-1.2985	0.90294
+	STRIP1	1	109996577	110064641	70	16	10164	-1.2987	0.90297
-	NDUFB6	9	32543526	32608184	106	23	10164	-1.2987	0.90298
+	MICAL2	11	12075576	12291016	834	99	10164	-1.2989	0.90302
+	PPP1R16B	20	38770705	38933024	123	17	10164	-1.2992	0.90306
-	OLIG3	6	137482199	137529394	185	32	10164	-1.2992	0.90306
-	TFR2	7	100610416	100677765	111	16	10164	-1.2998	0.90317
-	PXDC1	6	3712602	3787012	341	22	10164	-1.3	0.9032
+	MLANA	9	5855909	5919822	203	31	10164	-1.3003	0.90326
+	ESYT1	12	56093202	56154676	75	10	10164	-1.3006	0.9033
-	GRAP	17	19010677	19082023	10	1	10164	-1.3007	0.90332

STRAND	SYMBOL	CHR	START	STOP	NSNPS	NPARAM	N	ZSTAT	P
-	UBC	12	124901645	124950041	112	21	10164	-1.3008	0.90333
-	EVI2B	17	31292870	31349389	65	5	10164	-1.3008	0.90334
-	RFWD3	16	74611394	74701892	222	18	10164	-1.3011	0.90339
+	LOC389199	4	7903921	7950296	255	27	10164	-1.3014	0.90344
-	EIF2B4	2	27354352	27405457	76	10	10164	-1.3019	0.90352
-	GALNT3	2	165737803	165829650	218	32	10164	-1.3021	0.90356
-	USP46	4	52580960	52694335	182	11	10164	-1.303	0.90372
+	LRFN5	14	41572561	41914549	942	41	10164	-1.3036	0.90382
-	PRM1	16	11270836	11316335	199	16	10164	-1.3039	0.90386
-	HMGCS1	5	43277470	43348512	182	6	10164	-1.3045	0.90397
-	GNG13	16	788041	835733	234	25	10164	-1.3045	0.90397
+	MAP3K3	17	63587209	63706310	116	10	10164	-1.3047	0.90401
+	DNAJC5G	2	27240421	27291430	111	18	10164	-1.3052	0.90408
+	IL1RAP	3	190479051	190667197	258	45	10164	-1.3054	0.90413
+	USP35	11	78153812	78227644	138	19	10164	-1.3056	0.90416
-	SP3	2	173896459	174000702	253	28	10164	-1.3057	0.90418
+	LOC1053703	13	111588249	111652014	275	31	10164	-1.3057	0.90418
-	DNAJC12	10	67786665	67873191	212	21	10164	-1.3058	0.9042
+	POU4F2	4	146603893	146652471	168	21	10164	-1.306	0.90423
-	ZP2	16	21187452	21249510	140	27	10164	-1.3061	0.90423
-	ANKRD55	5	56089680	56268359	443	51	10164	-1.3064	0.9043
-	ELL3	15	43762600	43812304	90	12	10164	-1.3065	0.9043
+	BRSK1	19	55249166	55322562	156	24	10164	-1.3066	0.90433
-	LOC1053791	5	127254453	127326123	271	17	10164	-1.3067	0.90433
+	ZBED6CL	7	150294849	150342721	116	8	10164	-1.3067	0.90435
-	TTC21B	2	165863362	165988838	377	30	10164	-1.3068	0.90436
-	GNAQ	9	77706274	78066449	727	39	10164	-1.3073	0.90444
+	KBTBD13	15	65041816	65089938	135	19	10164	-1.3075	0.90447
+	SIMC1	5	176203367	176355989	356	26	10164	-1.3077	0.90451
-	CHD4	12	6560082	6642439	113	13	10164	-1.308	0.90456
+	LETM2	8	38351429	38419527	93	9	10164	-1.308	0.90456
-	CDH9	5	26870600	27156198	515	31	10164	-1.3082	0.90459
+	TYW1	7	66961805	67249520	1459	40	10164	-1.3085	0.90464
+	NAA16	13	41276205	41387030	264	17	10164	-1.3087	0.90468
+	ZNF177	19	9328020	9392617	113	4	10164	-1.3091	0.90476
+	RIN3	14	92478781	92698994	780	102	10164	-1.3095	0.90481
-	C16orf87	16	46792047	46866162	10	4	10164	-1.3095	0.90483
-	ZBTB3	11	62740963	62789184	72	9	10164	-1.31	0.9049
-	FAM168B	2	131037876	131128431	84	3	10164	-1.3101	0.90491
+	CRB1	1	197166462	197488455	599	25	10164	-1.3102	0.90494
-	CAPSL	5	35894295	35973779	322	36	10164	-1.3108	0.90504
-	PTRH2	17	59687306	59742495	80	14	10164	-1.3109	0.90506
-	AK8	9	132715574	132913811	443	32	10164	-1.311	0.90507
+	CIITA	16	10831212	10951449	265	37	10164	-1.3111	0.90509
-	SLFN13	17	35425096	35484008	205	31	10164	-1.3112	0.90511
-	LRIT1	10	84221520	84281493	217	30	10164	-1.3115	0.90515
-	VSIG10L	19	51321541	51377124	222	36	10164	-1.3116	0.90517
-	SSH1	12	108772690	108892583	217	30	10164	-1.3118	0.90521
-	CENPU	4	184684065	184769132	365	14	10164	-1.312	0.90523
+	DNAJC24	11	31334815	31442835	156	15	10164	-1.312	0.90523
+	PI15	8	74789469	74865044	176	19	10164	-1.3123	0.90529
-	ARPIN	15	89886532	89947990	76	7	10164	-1.3126	0.90534
-	MAN2B1	19	12636508	12701777	35	6	10164	-1.3126	0.90535
+	OAZ3	1	151727969	151781330	145	20	10164	-1.3136	0.90551
+	KIRREL	1	157958273	158110262	329	57	10164	-1.3145	0.90566
+	TMC7	16	18948934	19073942	268	17	10164	-1.3146	0.90568
-	STX7	6	132447524	132548198	296	30	10164	-1.3147	0.9057
+	UBASH3A	21	42368862	42457688	268	33	10164	-1.3155	0.90584
+	AHCYL1	1	109949741	110033742	97	26	10164	-1.3163	0.90597
+	KCNN2	5	114021006	114506500	1564	86	10164	-1.3173	0.90613
+	INO80B	2	74420023	74467960	28	3	10164	-1.3175	0.90616
+	TXNDC15	5	134838770	134911633	114	20	10164	-1.3175	0.90617
-	SNAI2	8	48907677	48956440	87	25	10164	-1.3181	0.90627

STRAND	SYMBOL	CHR	START	STOP	NSNPS	NPARAM	N	ZSTAT	P
-	SLC35E2B	1	1650025	1727804	2	1	10164	-1.3184	0.90631
-	SLC47A2	17	19668315	19753842	195	20	10164	-1.3184	0.90632
+	INAFM1	19	47239402	47285723	98	8	10164	-1.3185	0.90633
-	GDF5	20	35423347	35476387	123	11	10164	-1.3189	0.9064
+	ZYX	7	143343329	143401111	103	27	10164	-1.319	0.90642
+	PLEKHF2	8	95098721	95166685	85	25	10164	-1.3195	0.9065
+	TOMM40L	1	161190939	161240746	100	18	10164	-1.3195	0.90651
+	GDNF-AS1	5	37805438	37885799	247	26	10164	-1.3198	0.90656
-	ATG14	14	55356391	55446880	335	21	10164	-1.3199	0.90657
+	MFSD2B	2	23975083	24035874	170	23	10164	-1.3199	0.90657
+	CRISPLD1	8	74949473	75044558	293	21	10164	-1.3208	0.90672
-	KRT13	17	41490981	41540613	65	19	10164	-1.3209	0.90673
+	ADGRD2	9	124416087	124487771	188	35	10164	-1.3214	0.90681
+	TEN1	17	75944217	76010586	167	16	10164	-1.3219	0.9069
+	PDIA3	15	43711392	43782606	129	16	10164	-1.322	0.90691
+	SIPA1L1	14	71286396	71751229	628	30	10164	-1.3219	0.90691
+	TRIM42	3	140643024	140711150	114	9	10164	-1.322	0.90692
-	ERCC1	19	45397333	45513820	338	36	10164	-1.3222	0.90696
-	ENTHD2	17	81218277	81274091	124	16	10164	-1.3224	0.90699
-	PRSS50	3	46702116	46752883	194	18	10164	-1.3225	0.907
+	PRDM1	6	106051320	106119939	143	27	10164	-1.3229	0.90706
+	OR52W1	11	6164224	6210186	117	12	10164	-1.3234	0.90715
+	ADCY2	5	7361230	7840081	783	80	10164	-1.324	0.90725
-	ZNF652	17	49279206	49397473	291	27	10164	-1.324	0.90725
-	TXNDC5	6	7871250	7945814	207	26	10164	-1.3242	0.90728
+	ITGAX	16	31320134	31392997	149	11	10164	-1.3249	0.90739
-	SERINC3	20	44486223	44557085	113	12	10164	-1.3248	0.90739
+	TTC9C	11	62693078	62748638	89	9	10164	-1.3255	0.90749
+	SPATA31E1	9	87847857	87898899	242	24	10164	-1.3257	0.90753
-	ALS2CR11	2	201477421	201654183	478	34	10164	-1.3259	0.90757
+	ST3GAL4	11	126320645	126662917	664	87	10164	-1.326	0.90759
-	GYPB	4	143985188	144054427	218	18	10164	-1.3262	0.90762
-	ABCC6	16	16139565	16258673	112	13	10164	-1.3263	0.90763
+	ACSM3	16	20728990	20807157	135	9	10164	-1.3265	0.90766
+	C12orf10	12	53264686	53317181	90	10	10164	-1.3265	0.90766
+	MAVS	20	3811799	3886123	188	36	10164	-1.3265	0.90767
+	ACOT1	14	73458234	73553799	188	40	10164	-1.3265	0.90767
+	PML	15	73959673	74057819	287	29	10164	-1.3266	0.90768
-	EFCAB6	22	43518561	43847337	961	36	10164	-1.3267	0.9077
-	GDNF	5	37802677	37874680	211	21	10164	-1.327	0.90775
+	ALOX15B	17	8004040	8059134	168	31	10164	-1.3271	0.90776
+	IFIT5	10	89379568	89431002	150	19	10164	-1.3272	0.90778
-	CAMK2N2	3	184249215	184296463	94	17	10164	-1.3285	0.90799
+	C2orf74	2	61110091	61174830	174	11	10164	-1.3285	0.908
+	SYNGR3	16	1954945	2004275	184	21	10164	-1.3295	0.90816
+	LOC1053714	16	89666491	89713524	208	29	10164	-1.331	0.9084
+	SREBF2	22	41798079	41917308	308	28	10164	-1.3312	0.90844
-	IRX2	5	2731863	2786655	306	32	10164	-1.3315	0.90849
+	ROBO3	11	124830006	124891474	194	37	10164	-1.3318	0.90853
-	CCDC178	18	32927402	33476040	961	20	10164	-1.3319	0.90855
+	CATSPER4	1	26155587	26212542	137	20	10164	-1.3319	0.90856
+	VWA5B2	3	184194588	184252330	138	24	10164	-1.332	0.90857
-	GLRA1	5	151812513	151959866	416	21	10164	-1.3325	0.90865
-	WDR35	2	19900260	20025123	332	24	10164	-1.3329	0.90871
-	RNASE9	14	20546093	20595931	100	20	10164	-1.3329	0.90872
+	C1S	12	7025676	7081032	27	7	10164	-1.333	0.90873
+	CLCN1	7	143281126	143363862	71	9	10164	-1.333	0.90874
+	CCM2L	20	31975379	32042182	106	11	10164	-1.3331	0.90875
-	SESN1	6	108976437	109129505	274	11	10164	-1.3334	0.9088
-	KRTAP1-1	17	41030559	41076461	196	19	10164	-1.3338	0.90887
-	PABPC1	8	100692916	100757998	144	9	10164	-1.3339	0.90888
+	FAM101A	12	124254164	124326024	326	28	10164	-1.334	0.90889
-	TGOLN2	2	85308018	85363296	140	10	10164	-1.3343	0.90895

STRAND	SYMBOL	CHR	START	STOP	NSNPS	NPARAM	N	ZSTAT	P
-	PATE2	11	125762311	125813834	119	19	10164	-1.3349	0.90905
+	PCMTD2	20	64220695	64286226	182	23	10164	-1.3352	0.90909
+	LOC1019287	5	1046751	1093738	134	19	10164	-1.3352	0.90909
+	UBN2	7	139194565	139339421	141	9	10164	-1.3357	0.90917
-	C20orf203	20	32621625	32686981	161	18	10164	-1.3358	0.9092
+	CEP57	11	95755461	95842693	271	19	10164	-1.3361	0.90924
-	S100A10	1	151972910	152029238	187	14	10164	-1.3361	0.90925
+	PPARA	22	46115547	46253756	140	11	10164	-1.3365	0.9093
-	ARSI	5	150286346	150337962	124	26	10164	-1.3372	0.90943
+	EXTL1	1	25986741	26046464	101	10	10164	-1.3374	0.90945
+	ZNF460	19	57244408	57304075	225	29	10164	-1.3384	0.90961
+	C16orf92	16	29988334	30034702	50	2	10164	-1.3388	0.90969
+	ANKLE1	19	17246645	17297646	222	30	10164	-1.3389	0.9097
+	ARHGEF18	19	7313911	7486990	653	34	10164	-1.339	0.90972
+	CCR3	3	46175695	46276706	278	21	10164	-1.3392	0.90975
-	GCSH	16	81071947	81131375	356	18	10164	-1.3395	0.90979
-	CDC25A	3	48147178	48223658	118	21	10164	-1.3396	0.90982
+	RBM4	11	66603617	66678385	112	30	10164	-1.3398	0.90985
+	WDR81	17	1681523	1748599	186	28	10164	-1.3398	0.90985
-	ASAP1	8	130042104	130478660	1213	39	10164	-1.3402	0.9099
+	ANKRD37	4	185361686	185410243	114	19	10164	-1.3404	0.90994
-	CYFIP1	15	22859465	23015419	215	33	10164	-1.3407	0.91
+	PIGL	17	16182195	16359164	339	17	10164	-1.3409	0.91002
+	LOC1053772	3	88024221	88139791	398	22	10164	-1.3411	0.91005
-	COL17A1	10	104021288	104120880	222	31	10164	-1.3411	0.91006
-	DCUN1D3	16	20848074	20935239	120	11	10164	-1.3414	0.91011
+	FCN2	9	134829156	134899286	165	29	10164	-1.3416	0.91013
+	SMARCD1	12	50050200	50110711	89	12	10164	-1.3416	0.91013
-	SDF2	17	28638356	28697189	56	13	10164	-1.3422	0.91023
-	MAP3K11	11	65587755	65649338	154	38	10164	-1.3425	0.91029
+	ZSWIM8	10	73750577	73811798	74	10	10164	-1.3429	0.91034
-	FERMT1	20	6064845	6158544	313	22	10164	-1.343	0.91036
+	OR8K1	11	56311039	56356998	156	13	10164	-1.3431	0.91037
-	KBTBD7	13	41181575	41229566	87	17	10164	-1.3435	0.91044
-	SENP1	12	48032898	48141308	382	23	10164	-1.3438	0.91049
-	CPAMD8	19	16882951	17061818	554	87	10164	-1.344	0.91052
-	LOC1053753	7	26362438	26411794	190	33	10164	-1.344	0.91053
-	LOC1053710	15	101040701	101186837	533	75	10164	-1.3444	0.91058
-	MLN	6	33784672	33839016	346	37	10164	-1.3447	0.91063
+	BCL6B	17	6988050	7039642	140	15	10164	-1.3453	0.91074
+	SGCG	13	23125506	23335165	1178	95	10164	-1.3457	0.9108
-	GPR19	12	12650884	12752272	244	38	10164	-1.3462	0.91088
+	SPDYC	11	65134994	65183368	101	29	10164	-1.3462	0.91088
+	CD109	6	73637182	73838317	674	50	10164	-1.3464	0.91091
+	PCOLCE	7	100567259	100618175	63	20	10164	-1.3466	0.91094
-	C16orf13	16	624427	671366	179	19	10164	-1.3466	0.91094
+	CLEC4A	12	8088632	8148607	45	4	10164	-1.3468	0.91098
+	WARSP1	11	59220468	59268657	141	16	10164	-1.347	0.91101
+	GLRX5	14	95499986	95554718	201	32	10164	-1.3472	0.91104
-	SYCN	19	39192922	39239266	71	5	10164	-1.3473	0.91106
+	RASSF8	12	25924031	26089892	699	36	10164	-1.3477	0.91112
+	ATP6V1A	3	113712019	113822058	218	18	10164	-1.3482	0.91121
+	MSX1	4	4824665	4873936	232	36	10164	-1.349	0.91133
-	C3AR1	12	8048323	8101359	40	9	10164	-1.3495	0.91141
-	RAB14	9	121168137	121237087	169	26	10164	-1.3495	0.91141
+	FXYD4	10	43336644	43386335	134	16	10164	-1.3496	0.91143
-	EFCAB12	3	129391321	129463651	292	24	10164	-1.3497	0.91145
+	TVP23B	17	18746145	18816714	17	8	10164	-1.3499	0.91148
+	OR5AR1	11	56628686	56674618	150	21	10164	-1.3502	0.91152
-	FBXO47	17	38926278	39002486	187	16	10164	-1.3505	0.91157
+	RAD17	5	69334297	69424801	153	13	10164	-1.3507	0.9116
+	DOPEY2	21	36121783	36304274	560	40	10164	-1.3508	0.91161
-	PCLO	7	82744005	83197881	1130	53	10164	-1.3508	0.91162

STRAND	SYMBOL	CHR	START	STOP	NSNPS	NPARAM	N	ZSTAT	P
+	HYPK	15	43765421	43812589	88	13	10164	-1.351	0.91166
+	PHAX	5	126565915	126637252	197	18	10164	-1.352	0.91182
+	TAF6L	11	62736303	62797342	86	11	10164	-1.3521	0.91183
+	TMEM86A	11	18663735	18714785	138	28	10164	-1.3527	0.91192
-	DPM1	20	50924855	50993561	141	28	10164	-1.3527	0.91193
-	AK9	6	109482855	109726212	815	23	10164	-1.3536	0.91206
-	ANXA13	8	123670794	123772407	287	48	10164	-1.3538	0.91209
-	SARNP	12	55742463	55852756	106	16	10164	-1.3537	0.91209
-	ANXA5	4	121657997	121731992	237	21	10164	-1.3538	0.9121
+	NEK8	17	28690897	28752766	66	11	10164	-1.3538	0.9121
-	HIST1H4L	6	27863148	27908511	221	37	10164	-1.3538	0.91211
+	AP5S1	20	3785524	3835307	110	15	10164	-1.3543	0.91218
-	CDIP1	16	4500675	4573815	8	4	10164	-1.3544	0.9122
+	ADAMDEC1	8	24349285	24416013	113	14	10164	-1.3547	0.91224
+	COPS7A	12	6688984	6741875	133	17	10164	-1.355	0.91228
-	TPD52	8	80024868	80206659	448	20	10164	-1.3558	0.91242
+	DNAJB4	1	77944217	78028721	147	16	10164	-1.3561	0.91247
+	PPME1	11	74135891	74264703	253	17	10164	-1.3564	0.91251
-	C2orf91	2	41925368	41988803	277	34	10164	-1.3565	0.91253
-	CD2BP2	16	30340766	30390361	9	2	10164	-1.3567	0.91256
+	MYLPF	16	30339802	30387989	9	2	10164	-1.3567	0.91256
+	ZNF48	16	30343312	30410108	9	2	10164	-1.3567	0.91256
-	RPAP3	12	47651932	47741061	248	30	10164	-1.3568	0.91258
+	TRAFD1	12	112090545	112163604	105	13	10164	-1.3571	0.91262
-	EXOC3L2	19	45202621	45269211	238	31	10164	-1.3571	0.91262
-	KCNA4	11	29999741	30052030	92	11	10164	-1.3573	0.91266
+	RPS21	20	62352065	62398520	222	25	10164	-1.3576	0.91271
-	CACNA1A	19	13196442	13541460	489	63	10164	-1.3577	0.91272
-	POLRMT	19	607223	668592	34	6	10164	-1.3577	0.91272
+	ATF7IP	12	14330632	14512935	480	27	10164	-1.3578	0.91274
-	SCN9A	2	166185185	166410987	835	72	10164	-1.358	0.91278
-	OCIAD2	4	48875380	48941828	120	20	10164	-1.3588	0.91289
+	NEURL1	10	103458890	103602552	376	41	10164	-1.3589	0.9129
+	OSGIN1	16	83918222	83976332	245	34	10164	-1.359	0.91293
+	ZNF639	3	179287991	179345535	156	19	10164	-1.3597	0.91304
+	RFPL4A	19	55722430	55773419	195	32	10164	-1.3598	0.91306
+	GRK4	4	2928505	3050752	310	19	10164	-1.3603	0.91313
+	MYL6B	12	56117420	56167987	64	10	10164	-1.3607	0.9132
-	SLC23A2	20	4842356	5045293	346	46	10164	-1.361	0.91324
-	RETSAT	2	85331955	85389698	157	13	10164	-1.3614	0.9133
+	GUF1	4	44643395	44710928	200	12	10164	-1.3614	0.91331
-	CBFA2T3	16	88864855	89012199	475	24	10164	-1.3619	0.91339
+	CC2D2B	10	95939029	96045750	211	25	10164	-1.3622	0.91343
+	CSTL1	20	23404639	23471091	212	17	10164	-1.3623	0.91345
-	GFRA4	20	3649292	3698399	168	32	10164	-1.3624	0.91346
+	KIR3DL2	19	54815443	54877215	245	15	10164	-1.3624	0.91347
-	QSOX2	9	136196333	136280841	125	16	10164	-1.3629	0.91354
-	IFITM3	11	309673	355914	104	13	10164	-1.3633	0.9136
+	GRINA	8	143955058	144003419	66	3	10164	-1.3633	0.91361
+	NECAP1	12	8047211	8107777	44	9	10164	-1.3635	0.91364
+	LOC1053751	6	27277169	27324612	154	40	10164	-1.3636	0.91366
+	PRR11	17	59120499	59216709	159	21	10164	-1.3639	0.91369
+	PSKH1	16	67858272	67939678	94	13	10164	-1.3641	0.91373
-	KRTAP4-3	17	41157231	41203172	132	7	10164	-1.3642	0.91374
+	PSMG2	18	12623739	12735740	358	18	10164	-1.3645	0.9138
-	GPR68	14	91222532	91305790	163	22	10164	-1.3646	0.91382
+	EIF3B	7	2319540	2390745	143	17	10164	-1.3647	0.91382
+	HERC5	4	88422117	88516170	50	15	10164	-1.3653	0.91393
-	THOC1	18	204520	303059	264	26	10164	-1.3655	0.91396
-	KRTAP4-1	17	41174100	41219895	137	9	10164	-1.3659	0.91401
-	TMEM150B	19	55298886	55369043	185	29	10164	-1.3658	0.91401
+	DFFB	1	3822143	3895429	389	27	10164	-1.3664	0.91409
+	RGS6	14	71897100	72576530	2488	146	10164	-1.3664	0.91409

STRAND	SYMBOL	CHR	START	STOP	NSNPS	NPARAM	N	ZSTAT	P
-	KRT25	17	40738021	40790542	118	13	10164	-1.3666	0.91413
+	KIAA1614	1	180878177	180956103	146	32	10164	-1.3672	0.91422
+	CHAF1A	19	4367612	4458308	161	10	10164	-1.3676	0.91427
+	DLGAP2	8	984561	1718476	3963	204	10164	-1.3678	0.91431
+	UNK	17	75749839	75835805	61	8	10164	-1.3685	0.91443
-	HDAC4	2	239038168	239436647	957	61	10164	-1.3693	0.91455
-	BRIP1	17	61669186	61898937	423	35	10164	-1.3698	0.91463
+	APLF	2	68432559	68590162	371	11	10164	-1.3698	0.91463
-	MRPS31	13	40719296	40806223	269	23	10164	-1.3699	0.91464
-	OR1E1	17	3387466	3433410	159	24	10164	-1.37	0.91465
+	TNFAIP2	14	103086147	103147439	191	24	10164	-1.37	0.91466
+	UBXN2A	2	23892271	24011000	382	17	10164	-1.3701	0.91467
-	USP2	11	119345215	119416726	28	8	10164	-1.3708	0.91479
-	KRTAP1-3	17	41023884	41069855	220	19	10164	-1.3711	0.91482
-	FAM196A	10	127125426	127231216	217	26	10164	-1.3713	0.91485
-	PRSS46	3	46709583	46771431	175	16	10164	-1.3714	0.91488
+	FLNB	3	57973400	58182255	569	49	10164	-1.3722	0.915
+	NIPA2	15	22803641	22878384	72	8	10164	-1.3725	0.91505
+	MAEA	4	1254851	1350148	439	14	10164	-1.3729	0.91511
-	SEPT14	7	55783544	55897789	315	25	10164	-1.3752	0.91546
-	GYPE	4	143860866	143940563	174	14	10164	-1.3752	0.91547
-	DHCR7	11	71424411	71483431	221	16	10164	-1.3755	0.91552
-	GK2	4	79396353	79443218	41	13	10164	-1.3756	0.91552
+	PTPN12	7	77502456	77650071	328	19	10164	-1.3761	0.91561
-	OR6S1	14	20630696	20676691	123	11	10164	-1.3764	0.91566
-	GPR176	15	39789022	39955892	485	61	10164	-1.3766	0.91569
+	TET2	4	105110875	105289803	421	40	10164	-1.3767	0.91569
-	LBHD1	11	62652817	62706769	79	12	10164	-1.3768	0.91571
+	PANK2	20	3853839	3933891	229	31	10164	-1.3768	0.91572
+	ITSN1	21	33607400	33899305	317	34	10164	-1.3771	0.91576
+	ADGRE1	19	6852549	6950453	295	13	10164	-1.3775	0.91582
-	OR5M8	11	56480435	56526370	167	17	10164	-1.3775	0.91583
+	COL6A5	3	130310516	130494846	674	50	10164	-1.3777	0.91585
-	HIRIP3	16	29982321	30031096	52	2	10164	-1.3782	0.91593
-	IGSF11	3	118890630	119181068	949	51	10164	-1.3785	0.91598
-	PGAP1	2	196823004	196961995	217	16	10164	-1.3789	0.91604
-	CEP128	14	80466988	80994501	1453	52	10164	-1.379	0.91605
-	TNS1	2	217789789	218013827	462	70	10164	-1.3793	0.91609
-	HOXA11	7	27171157	27220216	70	12	10164	-1.3794	0.91612
-	PLAC8	4	83080048	83149758	48	4	10164	-1.3798	0.91618
-	FASTKD1	2	169517880	169608921	251	21	10164	-1.3802	0.91623
+	ADRA2C	4	3731569	3778526	48	7	10164	-1.3808	0.91633
-	AFAP1	4	7748713	7974926	1297	44	10164	-1.3814	0.91642
-	C19orf25	19	1463201	1514229	42	3	10164	-1.3816	0.91645
-	SMUG1	12	54162031	54223994	162	29	10164	-1.382	0.91651
-	CCR1	3	46191709	46243341	149	10	10164	-1.3822	0.91654
+	GNG3	11	62671422	62719206	64	7	10164	-1.3824	0.91658
+	FAM210B	20	56323927	56378663	106	8	10164	-1.3825	0.91659
-	PP2D1	3	19969961	20047780	176	22	10164	-1.3826	0.9166
-	KIAA2026	9	5849595	6043479	619	36	10164	-1.3829	0.91665
+	KIAA1586	6	57011532	57069326	75	9	10164	-1.383	0.91666
+	NCS1	9	130137578	130247304	299	28	10164	-1.383	0.91667
+	KCND2	7	120238641	120760333	684	27	10164	-1.3832	0.91669
+	LLGL1	17	18190593	18254875	186	23	10164	-1.3832	0.91669
+	C7orf69	7	47760291	47829847	317	23	10164	-1.3832	0.9167
-	THUMPD1	16	20723664	20776964	104	7	10164	-1.3833	0.91671
-	RPS6KA5	14	90858122	91095649	582	39	10164	-1.3836	0.91676
-	TEFM	17	30888983	30941775	79	5	10164	-1.3842	0.91685
-	C20orf194	20	3239302	3442662	688	23	10164	-1.3846	0.91691
+	CYB561D1	1	109459036	109510441	84	5	10164	-1.3846	0.91691
+	POU2F1	1	167185829	167437345	392	28	10164	-1.3849	0.91696
-	CENPB	20	3773851	3821690	103	14	10164	-1.3853	0.91701
-	LOC1053758	8	47078584	47127604	94	13	10164	-1.3856	0.91706

STRAND	SYMBOL	CHR	START	STOP	NSNPS	NPARAM	N	ZSTAT	P
+	HRG	3	186630958	186688235	166	21	10164	-1.3859	0.91711
-	KIAA0100	17	28604440	28680159	66	19	10164	-1.386	0.91713
+	OR6K6	1	158719816	158765847	182	13	10164	-1.386	0.91713
-	LRRC10	12	69598564	69646162	139	23	10164	-1.3864	0.91718
-	C15orf38-AP3	15	89820599	89947990	137	9	10164	-1.3883	0.91748
+	PHACTR1	6	12681606	13300244	1543	99	10164	-1.3886	0.91752
+	RASSF3	12	64575513	64707567	291	29	10164	-1.3887	0.91753
-	AP2S1	19	46828158	46885995	70	21	10164	-1.3896	0.91768
+	DEFB132	20	222736	271096	225	17	10164	-1.3898	0.9177
+	LOC1053730	22	41934247	41985904	132	14	10164	-1.3902	0.91777
-	LRCH4	7	100564011	100621188	69	20	10164	-1.3906	0.91783
-	MAN1A1	6	119167201	119386633	1006	34	10164	-1.3906	0.91783
-	UCN	2	27297397	27343262	50	14	10164	-1.3906	0.91783
-	LCP1	13	46115923	46217324	204	31	10164	-1.3908	0.91786
+	FAM64A	17	6409205	6461065	148	25	10164	-1.3913	0.91794
-	ATR	3	142439235	142613826	299	19	10164	-1.3917	0.91799
+	VILL	3	37952975	38017185	157	17	10164	-1.3917	0.918
-	OBP2B	9	133195279	133288397	503	31	10164	-1.3921	0.91805
-	ST8SIA5	18	46669118	46792169	439	65	10164	-1.3923	0.91808
+	TIMM50	19	39445412	39503018	160	12	10164	-1.3926	0.91813
+	FHL5	6	96527548	96628620	223	17	10164	-1.3927	0.91814
+	RGR	10	84202083	84269347	239	28	10164	-1.3927	0.91815
-	ADH4	4	99113681	99179314	77	9	10164	-1.3928	0.91816
+	PPP2R5B	11	64882553	64944478	99	31	10164	-1.3937	0.9183
-	NPFFR1	10	70244957	70318694	246	39	10164	-1.3938	0.91831
-	C6orf118	6	165269664	165344647	141	11	10164	-1.3943	0.91838
+	AGXT	2	240833745	240889119	279	21	10164	-1.3944	0.91839
+	ARPC1B	7	99339675	99404801	123	17	10164	-1.3944	0.91841
-	HECTD4	12	112150188	112417092	404	19	10164	-1.3945	0.91841
+	MRPL32	7	42897205	42947857	95	3	10164	-1.3946	0.91843
+	ASPSCR1	17	81942550	82027563	37	3	10164	-1.3948	0.91846
-	AIPL1	17	6413737	6470199	167	30	10164	-1.3957	0.91859
+	DECR1	8	89966352	90062004	301	20	10164	-1.3961	0.91866
-	WTH3DI	2	131350492	131399158	82	2	10164	-1.3962	0.91867
+	MGAM2	7	142076722	142233533	257	33	10164	-1.3965	0.91872
+	TNN	1	175032858	175158066	405	33	10164	-1.3966	0.91873
+	ZP1	11	60832542	60885693	115	16	10164	-1.3979	0.91893
-	EBF2	8	25831730	26080124	518	51	10164	-1.3981	0.91895
+	VRTN	14	74268076	74370008	244	35	10164	-1.3982	0.91897
+	TAGLN3	3	111963739	112023888	316	39	10164	-1.3984	0.919
-	ZDHHC20	13	21362571	21494370	165	11	10164	-1.3984	0.919
-	JUN	1	58770791	58819113	116	18	10164	-1.3987	0.91905
-	NAPB	20	23364519	23456519	290	21	10164	-1.3994	0.91916
-	NPPA	1	11835709	11882783	205	34	10164	-1.4003	0.91929
-	SLC22A23	6	3258973	3491587	580	44	10164	-1.4006	0.91933
-	C1orf194	1	109095951	109148870	134	14	10164	-1.4006	0.91934
+	MAN2B2	4	6540174	6632403	171	18	10164	-1.4008	0.91937
-	SLC43A2	17	1559254	1665018	242	46	10164	-1.4012	0.91943
-	NAGA	22	42048334	42105871	119	8	10164	-1.4016	0.91949
+	ABT1	6	26561943	26610050	195	23	10164	-1.4018	0.91952
+	MUC17	7	100985076	101068859	258	27	10164	-1.4019	0.91953
+	MIPOL1	14	37162913	37588857	616	19	10164	-1.4019	0.91953
+	CLEC19A	16	19250755	19334537	189	21	10164	-1.4023	0.91959
-	SEC14L4	22	30478890	30540711	233	23	10164	-1.4024	0.9196
+	BAMBI	10	28642495	28692939	122	16	10164	-1.4025	0.91962
-	FREM1	9	14724666	14946647	765	95	10164	-1.4027	0.91965
-	NAA25	12	112016689	112144022	181	15	10164	-1.4029	0.91967
+	TMEM178A	2	39630498	39727963	92	17	10164	-1.4029	0.91968
+	CLSTN3	12	7095371	7168945	166	19	10164	-1.4031	0.9197
+	AMDHD2	16	2485362	2540954	10	4	10164	-1.4034	0.91975
+	EIF3L	22	37813448	37898782	117	13	10164	-1.4037	0.91979
+	NSMCE2	8	125056818	125377125	733	46	10164	-1.4038	0.91981
-	GPR156	3	120155481	120320124	583	35	10164	-1.4039	0.91983

STRAND	SYMBOL	CHR	START	STOP	NSNPS	NPARAM	N	ZSTAT	P
+	TMSB10	2	84870639	84916675	31	8	10164	-1.4042	0.91987
+	CHCHD1	10	73747050	73793648	61	9	10164	-1.4044	0.9199
-	TRIM21	11	4374897	4428712	266	17	10164	-1.4045	0.91991
-	ZNF239	10	43546344	43609618	132	10	10164	-1.405	0.91999
-	RASA3	13	113967783	114167620	734	34	10164	-1.4055	0.92007
+	SHOC2	10	110884543	111023667	249	28	10164	-1.4056	0.92008
+	MIR1-1HG	20	62515453	62580764	250	20	10164	-1.4056	0.92008
+	ARCN1	11	118537387	118613033	147	9	10164	-1.406	0.92014
+	PURA	5	140079123	140129416	42	7	10164	-1.4061	0.92015
-	HOXA10	7	27160591	27215261	83	16	10164	-1.4061	0.92016
+	LOC1053707	14	23021023	23068476	105	26	10164	-1.4062	0.92016
+	LOC81691	16	20771429	20859668	115	12	10164	-1.4062	0.92017
+	EFHD2	1	15374895	15440343	136	17	10164	-1.4073	0.92033
-	HIST1H3I	6	27861845	27907321	225	37	10164	-1.4083	0.92048
+	ELOVL3	10	102190358	102239589	84	21	10164	-1.4084	0.9205
+	DESI2	1	244618048	244719034	188	23	10164	-1.4088	0.92055
-	DCBLD2	3	98785970	98936689	470	27	10164	-1.409	0.92058
+	HYLS1	11	125848614	125910646	163	24	10164	-1.409	0.92059
+	KRTAP9-1	17	41154887	41200639	134	8	10164	-1.4091	0.92059
+	LPPR4	1	99227953	99319584	258	29	10164	-1.4093	0.92063
-	KRBA2	17	8358637	8411711	123	20	10164	-1.4099	0.92071
-	OR6Y1	1	158537128	158583105	97	4	10164	-1.4104	0.92078
+	PIWIL4	11	94532308	94631421	321	29	10164	-1.4105	0.9208
+	LOC1053734	1	162385082	162457346	273	26	10164	-1.4105	0.9208
+	MAPK8IP1	11	45850496	45916465	123	24	10164	-1.4106	0.92082
-	AQP4	18	26842043	26900752	147	32	10164	-1.4109	0.92086
+	TSEN34	19	54154517	54204536	99	15	10164	-1.4113	0.92091
+	CATIP	2	218321832	218378110	159	23	10164	-1.4113	0.92092
-	SUN5	20	32973773	33039449	232	25	10164	-1.4115	0.92095
-	LOC1053741	3	130133281	130180044	163	8	10164	-1.4115	0.92095
+	DIRC2	3	122760054	122891139	245	24	10164	-1.4117	0.92098
-	USHBP1	19	17240021	17299796	244	32	10164	-1.4121	0.92104
-	CCL28	5	43346972	43447391	233	18	10164	-1.413	0.92118
-	SCAPER	15	76338185	76940455	1316	28	10164	-1.4132	0.9212
+	PSMB4	1	151364565	151411936	64	17	10164	-1.4133	0.92122
-	C20orf96	20	260863	325778	235	27	10164	-1.4138	0.92129
+	CEP250	20	35420139	35527531	234	17	10164	-1.4139	0.9213
-	NDUFA12	12	94961328	95038713	323	24	10164	-1.4146	0.92141
+	PDE6B	4	552325	680892	120	6	10164	-1.415	0.92146
-	LRPAP1	4	3493597	3567497	220	23	10164	-1.4152	0.92149
+	FAM207A	21	44904998	44986976	247	24	10164	-1.4153	0.92151
-	TNP2	16	11257857	11304303	186	17	10164	-1.4154	0.92152
+	SPATA1	1	84471301	84566495	275	19	10164	-1.4158	0.92159
-	POLR2E	19	1076579	1130392	216	31	10164	-1.4162	0.92165
+	FSTL3	19	641389	693392	33	6	10164	-1.4163	0.92166
-	ZBTB21	21	41976831	42045387	169	27	10164	-1.4171	0.92177
+	LRRC1	6	53759497	53934610	694	23	10164	-1.4172	0.92178
+	TRIM73	7	75360629	75415613	4	2	10164	-1.4173	0.9218
-	OR5AP2	11	56631489	56677439	155	22	10164	-1.4186	0.92199
+	LOC1053708	15	53802628	53926058	602	53	10164	-1.4189	0.92203
+	SLC25A33	1	9504469	9592773	175	19	10164	-1.4191	0.92206
-	C7orf25	7	42899273	42947090	93	3	10164	-1.4196	0.92214
+	GNAI3	1	109513564	109605843	193	22	10164	-1.4199	0.92219
+	CLN8	8	1720778	1796570	235	18	10164	-1.4204	0.92225
-	KRT23	17	40912696	40972643	288	31	10164	-1.4206	0.92228
+	PET117	20	18102855	18153169	135	19	10164	-1.4205	0.92228
+	LOC1053723	19	9370651	9418256	110	4	10164	-1.4208	0.92231
+	UVSSA	4	1310658	1398049	305	20	10164	-1.4209	0.92232
-	BRINP3	1	190087662	190513404	973	46	10164	-1.4216	0.92243
+	SMAD6	15	66667100	66792000	407	47	10164	-1.422	0.92249
+	CADM3	1	159136587	159213142	137	38	10164	-1.4222	0.92252
-	TMEM223	11	62780315	62827014	66	6	10164	-1.4224	0.92255
+	OGG1	3	9714944	9798246	201	33	10164	-1.4235	0.9227

STRAND	SYMBOL	CHR	START	STOP	NSNPS	NPARAM	N	ZSTAT	P
-	C10orf113	10	21115763	21181559	178	20	10164	-1.4237	0.92273
+	LOC1027239	9	135579222	135629893	135	27	10164	-1.4239	0.92277
+	CCDC130	19	13696763	13773292	147	15	10164	-1.4242	0.9228
-	CEP131	17	81179593	81257994	223	25	10164	-1.4245	0.92285
+	FGF2	4	122791708	122908235	335	27	10164	-1.4247	0.92287
-	KRTAP1-4	17	41019677	41065125	227	19	10164	-1.4248	0.92289
-	PEX10	1	2394802	2448780	128	31	10164	-1.4248	0.9229
-	CBX6	22	38851216	38907334	214	8	10164	-1.4253	0.92296
-	CGB7	19	49044274	49092788	142	20	10164	-1.4253	0.92296
+	OR8U1	11	56340624	56386553	161	15	10164	-1.4253	0.92297
-	NUMB	14	73265154	73493582	714	40	10164	-1.4254	0.92298
-	ADAM33	20	3657973	3717131	200	32	10164	-1.4255	0.92299
+	SYNPO	5	150551037	150669230	293	35	10164	-1.4255	0.923
-	HNRNPL	19	38826378	38887339	155	19	10164	-1.426	0.92307
+	GALNT10	5	154155735	154430984	943	67	10164	-1.4262	0.92309
-	WNT2	7	117266631	117358289	182	34	10164	-1.4273	0.92325
+	CAMP	3	48188347	48235491	67	16	10164	-1.4291	0.92352
+	MAPK1IP1L	14	55016644	55080194	124	15	10164	-1.4292	0.92352
-	GSPT1	16	11858128	11951662	197	19	10164	-1.4292	0.92353
-	BEST3	12	69633508	69734416	357	25	10164	-1.4296	0.92359
-	MAT1A	10	80261820	80324979	195	26	10164	-1.4299	0.92363
-	CTAGE1	18	22403601	22452915	110	11	10164	-1.43	0.92364
+	FAM111A	11	59107845	59165039	172	14	10164	-1.4304	0.9237
-	COMTD1	10	75223971	75271057	103	20	10164	-1.4305	0.92371
+	ISCU	12	108526463	108579384	179	15	10164	-1.4306	0.92372
+	WSCD2	12	108094343	108260537	114	29	10164	-1.4308	0.92376
-	SAP25	7	100562226	100608864	51	18	10164	-1.4321	0.92394
+	TCP11L1	11	33004417	33089454	335	9	10164	-1.4322	0.92395
-	PLCD3	17	45101640	45167533	163	33	10164	-1.4322	0.92395
+	IL17A	6	52151387	52200638	183	23	10164	-1.4327	0.92403
-	LOC1053778	13	113997613	114044124	178	16	10164	-1.433	0.92408
-	MAGOHB	12	10593765	10648609	173	16	10164	-1.4341	0.92422
+	RNF121	11	71893701	72007597	407	14	10164	-1.4343	0.92425
+	TSHR	14	80919989	81156302	832	52	10164	-1.4345	0.92428
-	CAMKK1	17	3850315	3929891	150	32	10164	-1.4346	0.9243
+	GINS1	20	25372444	25458555	234	12	10164	-1.435	0.92435
+	PANX2	22	50135731	50190295	243	30	10164	-1.4349	0.92435
+	WFIKKN1	16	596012	644116	186	18	10164	-1.4353	0.9244
-	RAPGEF5	7	22108290	22392133	799	67	10164	-1.4354	0.92441
-	SLC19A1	21	45492517	45598126	281	30	10164	-1.4357	0.92445
-	FMO5	1	147174300	147260798	274	24	10164	-1.4357	0.92446
+	RFXAP	13	36784202	36839603	101	20	10164	-1.4359	0.92448
+	ATM	11	108187500	108379102	372	21	10164	-1.436	0.9245
-	MTPAP	10	30299801	30384338	286	36	10164	-1.4365	0.92457
-	CERS5	12	50119306	50202533	142	12	10164	-1.4366	0.92458
+	IFI44	1	78613943	78674078	124	22	10164	-1.4366	0.92459
-	DUSP13	10	75084432	75144221	155	19	10164	-1.4366	0.92459
-	PTPN5	11	18717928	18827721	324	49	10164	-1.4367	0.92459
-	ZSCAN5B	19	56179689	56243038	34	5	10164	-1.4366	0.92459
-	KRTAP19-8	21	31028159	31073476	202	9	10164	-1.4367	0.9246
+	LOC1053709	15	39747415	39795617	120	12	10164	-1.4367	0.9246
-	IGFL4	19	46029748	46092132	177	28	10164	-1.4368	0.92461
-	CTSH	15	78911750	78980089	211	28	10164	-1.4373	0.92469
+	ACTN3	11	66511395	66573329	182	20	10164	-1.4376	0.92472
+	GAS2	11	22592053	22823001	727	52	10164	-1.4377	0.92474
-	LILRB5	19	54240405	54292308	84	11	10164	-1.4378	0.92476
-	DDX58	9	32445302	32561324	255	29	10164	-1.4386	0.92487
-	C20orf173	20	35510741	35564652	117	13	10164	-1.4387	0.92488
+	IFT74	9	26912039	27076129	430	25	10164	-1.4394	0.92498
+	BMP2K	4	78741378	78926365	229	7	10164	-1.4395	0.92499
-	LOC1005054	9	114656434	114717089	282	32	10164	-1.4394	0.92499
+	GSTK1	7	143228429	143279129	9	2	10164	-1.4399	0.92505
-	SEMA3E	7	83353906	83684163	1054	53	10164	-1.44	0.92507

STRAND	SYMBOL	CHR	START	STOP	NSNPS	NPARAM	N	ZSTAT	P
-	FAM184B	4	17622073	17816527	577	44	10164	-1.4408	0.92518
-	SPTA1	1	158600498	158721716	227	18	10164	-1.4412	0.92524
+	WWC2	4	183063635	183330777	514	40	10164	-1.4413	0.92524
+	NENF	1	212397887	212456379	177	27	10164	-1.4421	0.92536
-	PYGO1	15	55528884	55623852	233	37	10164	-1.4426	0.92544
-	SNCB	5	176610209	176665556	163	33	10164	-1.4433	0.92553
-	ACOX1	17	75931507	76014434	197	20	10164	-1.4433	0.92554
+	TRIB1	8	125395321	125448405	53	15	10164	-1.4444	0.92569
+	MFN2	1	11945181	12023515	254	20	10164	-1.4445	0.9257
-	GPR20	8	141343407	141402271	143	17	10164	-1.4447	0.92572
+	HSPA12B	20	3697670	3763111	171	24	10164	-1.4447	0.92573
-	LOC1019286	7	51376082	51422506	222	25	10164	-1.446	0.92591
+	C10orf35	10	69595243	69643599	126	27	10164	-1.4464	0.92597
+	TP53I13	17	29532180	29590820	91	22	10164	-1.447	0.92606
+	SNURF	15	24919923	24988582	4	2	10164	-1.4474	0.9261
+	RGS2	1	192774039	192822277	155	17	10164	-1.4475	0.92612
-	LOC1053760	9	30261682	30384696	356	18	10164	-1.4479	0.92618
-	AKR1C2	10	4977775	5053033	345	28	10164	-1.4484	0.92624
+	BABAM1	19	17232376	17289353	232	33	10164	-1.4485	0.92626
-	PHIP	6	78924419	79113294	716	20	10164	-1.4487	0.92629
-	ZNF681	19	23729195	23793891	139	20	10164	-1.4488	0.9263
+	SLC24A3	20	19177646	19732897	2014	136	10164	-1.4489	0.92631
-	TMEM206	1	212354474	212450508	267	34	10164	-1.449	0.92633
-	CCDC125	5	69263101	69367852	216	15	10164	-1.4501	0.92649
+	RAB27B	18	54682866	54905516	566	36	10164	-1.4511	0.92662
+	NUP107	12	68651951	68752693	170	21	10164	-1.4513	0.92666
-	CDRT1	17	15555482	15654704	200	31	10164	-1.4516	0.9267
-	FAM76B	11	95758942	95825396	211	17	10164	-1.4519	0.92673
+	LBH	2	30196531	30270033	204	33	10164	-1.4525	0.92682
-	ATAD2B	2	23675229	23962114	838	24	10164	-1.4527	0.92684
+	RFPL4AL1	19	55734118	55783423	203	33	10164	-1.4532	0.92692
+	CFAP69	7	90210174	90334208	365	24	10164	-1.4536	0.92697
+	GABRG2	5	162032642	162165539	283	55	10164	-1.4538	0.92699
-	GOT1	10	99386870	99465773	117	22	10164	-1.4538	0.92699
+	PYHIN1	1	158896547	158998872	273	27	10164	-1.454	0.92703
-	CBS	21	43043191	43111868	1	1	10164	-1.4546	0.92712
+	SLFN5	17	35208067	35277749	305	37	10164	-1.4547	0.92712
-	CHD5	1	6091787	6215134	332	31	10164	-1.4551	0.92717
-	TMIGD2	19	4282227	4337582	62	8	10164	-1.4551	0.92718
+	LOC1001292	11	71843453	71894561	289	12	10164	-1.4552	0.92719
-	FUBP1	1	77934055	78014205	140	15	10164	-1.4556	0.92725
-	NUDT14	14	105162937	105216323	151	22	10164	-1.4556	0.92725
-	ITGB2	21	44875949	44963873	263	43	10164	-1.4557	0.92726
+	POU5F1B	8	127380612	127427210	217	38	10164	-1.4558	0.92727
+	SPNS3	17	4398924	4498204	159	22	10164	-1.4562	0.92733
+	KIAA1671	22	24917702	25207448	924	71	10164	-1.4568	0.92741
-	ADGRF3	2	26298173	26381817	183	21	10164	-1.4567	0.92741
-	SMIM14	4	39537297	39673861	409	42	10164	-1.4568	0.92742
-	TULP1	6	35487874	35547902	325	39	10164	-1.4571	0.92745
+	NIPA1	15	22751225	22839789	12	4	10164	-1.4573	0.92749
-	ABCD4	14	74275277	74338064	151	20	10164	-1.4575	0.92751
+	APOL5	22	35673424	35740325	203	16	10164	-1.4611	0.92801
+	ENTPD6	20	25160670	25236724	225	19	10164	-1.4613	0.92804
+	ODAM	4	70160726	70214576	178	20	10164	-1.4616	0.92808
+	TENM2	5	166944078	168274157	2747	247	10164	-1.4618	0.9281
-	ELF1	13	40921919	41096378	549	28	10164	-1.462	0.92813
-	CD302	2	159758628	159833255	258	25	10164	-1.462	0.92813
+	SYNPR	3	63165409	63626924	1829	99	10164	-1.4622	0.92816
-	SDR42E1	16	81987616	82046488	143	8	10164	-1.4623	0.92818
-	PREB	2	27120756	27169674	86	14	10164	-1.4624	0.92819
+	HTR1F	3	87757767	88004856	968	38	10164	-1.4626	0.92822
-	GNRHR	4	67727375	67791086	95	11	10164	-1.4628	0.92824
-	SH2D2A	1	156796243	156851862	123	15	10164	-1.4629	0.92825

STRAND	SYMBOL	CHR	START	STOP	NSNPS	NPARAM	N	ZSTAT	P
-	FMN1	15	32755544	33229784	1747	118	10164	-1.4634	0.92831
-	TRIQK	8	92873530	93001144	192	35	10164	-1.4639	0.92839
+	OR10J1	1	159362494	159450967	270	29	10164	-1.4648	0.92851
+	CDKN3	14	54361934	54430218	197	24	10164	-1.465	0.92854
-	DTYMK	2	241665742	241721968	122	8	10164	-1.4655	0.92861
-	ATAD1	10	87741851	87876349	294	30	10164	-1.4659	0.92866
+	ANTXR1	2	68978144	69259327	1079	80	10164	-1.4661	0.92868
+	C8orf4	8	40118468	40165308	93	14	10164	-1.4662	0.9287
-	CSNK2A1	20	462969	578838	263	36	10164	-1.4663	0.92872
-	TBL1XR1	3	177010754	177232260	606	32	10164	-1.4666	0.92876
+	ARSG	17	68222159	68460773	606	57	10164	-1.4668	0.92878
+	IFT122	3	129405036	129530507	446	21	10164	-1.4671	0.92883
-	ZNF558	19	8799574	8867304	149	9	10164	-1.4678	0.92892
+	PADI4	1	17273195	17374004	436	23	10164	-1.4682	0.92897
-	MSI1	12	120329650	120404180	179	22	10164	-1.4683	0.92899
-	DSN1	20	36741791	36808827	50	16	10164	-1.4684	0.929
+	PNLDC1	6	159765249	159830704	197	15	10164	-1.4685	0.92902
-	CDH2	18	27922879	28212481	774	71	10164	-1.4686	0.92903
-	DEFA6	8	6914694	6961076	184	29	10164	-1.4693	0.92913
+	LCE5A	1	152475844	152522177	136	15	10164	-1.4707	0.92931
+	PKN2	1	88649193	88846255	398	22	10164	-1.4711	0.92936
+	PATE4	11	125798316	125850072	106	16	10164	-1.471	0.92936
-	CCDC34	11	27328514	27398248	177	20	10164	-1.4715	0.92942
+	FCER1A	1	159248888	159318224	224	25	10164	-1.4718	0.92946
-	RNASET2	6	166919516	166991589	303	25	10164	-1.4721	0.9295
-	RASIP1	19	48710585	48775813	127	12	10164	-1.4721	0.9295
-	B4GAT1	11	66335372	66382690	111	15	10164	-1.4722	0.92951
-	LOC1053719	17	28212783	28263461	73	17	10164	-1.4721	0.92951
+	NFASC	1	204793654	205032822	841	61	10164	-1.4726	0.92957
+	TMEM106B	7	12176222	12247264	539	35	10164	-1.4734	0.92967
+	COQ10A	12	56231858	56280966	55	5	10164	-1.4734	0.92968
+	WDFY2	13	51549194	51776799	281	38	10164	-1.4736	0.9297
+	C20orf202	20	1168454	1218274	51	8	10164	-1.4737	0.92972
+	USP6	17	5081438	5185032	268	32	10164	-1.474	0.92976
-	FAM131B	7	143343400	143417304	150	38	10164	-1.474	0.92976
-	TLE2	19	2987638	3082635	308	49	10164	-1.4744	0.92982
-	CUEDC2	10	102413245	102467666	119	30	10164	-1.4748	0.92987
+	SMPD2	6	109405646	109453922	129	16	10164	-1.4752	0.92992
-	TMEM229B	14	67460266	67568739	391	47	10164	-1.4756	0.92997
-	CAMK1	3	9747345	9804987	166	26	10164	-1.4763	0.93007
+	WDR83	19	12631804	12685832	36	5	10164	-1.4764	0.93008
-	LMF2	22	50492947	50542840	131	19	10164	-1.4771	0.93017
-	COTL1	16	84555596	84653096	353	43	10164	-1.4771	0.93018
+	MIEF1	22	39465100	39528134	108	15	10164	-1.4771	0.93018
+	ZNF492	19	22599324	22677670	429	20	10164	-1.4775	0.93023
-	KRT34	17	41367650	41418816	97	16	10164	-1.4783	0.93033
+	TMEM201	1	9553853	9624877	129	14	10164	-1.4789	0.93042
-	ATP5O	21	33893453	33950854	29	7	10164	-1.479	0.93043
+	UAP1L1	9	137042465	137094538	89	10	10164	-1.4795	0.9305
+	MYOZ3	5	150625841	150689700	194	17	10164	-1.48	0.93056
-	PMEL	12	55944105	56001712	102	19	10164	-1.4806	0.93064
+	LOC1027240	11	66312544	66373764	143	21	10164	-1.4808	0.93067
+	ADAP2	17	30886685	30969398	101	7	10164	-1.4815	0.93076
-	MFSD10	4	2920560	2969868	103	16	10164	-1.4825	0.9309
-	LMBRD2	5	36088419	36186913	220	31	10164	-1.4825	0.9309
+	LOC1019282	7	30425051	30479422	112	11	10164	-1.4825	0.9309
-	GTF3C2	2	27315849	27392034	104	17	10164	-1.4827	0.93093
+	SMAD4	18	50995213	51095042	127	12	10164	-1.4829	0.93095
-	FAM83G	17	18961068	19039747	22	1	10164	-1.483	0.93097
-	KRTAP4-4	17	41149653	41195731	125	7	10164	-1.4842	0.93112
+	EHD3	2	31199014	31279451	262	32	10164	-1.4845	0.93116
-	ZCCHC24	10	79372325	79480627	309	39	10164	-1.4849	0.93121
-	UBXN1	11	62666498	62714117	66	8	10164	-1.4849	0.93122

STRAND	SYMBOL	CHR	START	STOP	NSNPS	NPARAM	N	ZSTAT	P
+	RLTPR	16	67610004	67667569	54	15	10164	-1.4851	0.93124
+	RAB5B	12	55938913	56006683	113	21	10164	-1.4852	0.93126
+	YY1	14	100203765	100289034	146	17	10164	-1.4861	0.93138
-	TRIM74	7	72944849	73004681	3	2	10164	-1.4862	0.93139
+	CKMT1B	15	43557862	43609406	23	4	10164	-1.4875	0.93156
-	MPEG1	11	59198510	59248021	128	15	10164	-1.4878	0.9316
+	ANKS4B	16	21198695	21262429	143	30	10164	-1.4883	0.93167
-	ZNF319	16	57984668	58035684	130	20	10164	-1.4888	0.93173
-	LAPTM5	1	30722468	30792836	161	27	10164	-1.4889	0.93175
-	LEMD2	6	33761213	33824163	355	29	10164	-1.489	0.93175
+	CLVS1	8	60931438	61511645	1537	100	10164	-1.4898	0.93187
+	DDX21	10	68921123	68995069	154	17	10164	-1.4902	0.93191
+	NF2	22	29568556	29708600	369	32	10164	-1.4906	0.93197
-	C2CD3	11	74002714	74206210	430	20	10164	-1.4907	0.93198
+	RHOT2	16	633083	684174	181	19	10164	-1.491	0.93202
+	FGF21	19	48720559	48768330	97	10	10164	-1.492	0.93215
+	LSM3	3	14143728	14208369	153	19	10164	-1.4921	0.93216
-	C11orf65	11	108321426	108502547	480	27	10164	-1.4925	0.93221
-	HMGCS2	1	119737996	119804125	219	18	10164	-1.4926	0.93224
+	LOC1027249	19	28571688	28625229	121	28	10164	-1.4927	0.93224
-	FBXO10	9	37500892	37611311	293	22	10164	-1.4928	0.93225
+	LOC1053746	5	2882838	2975527	334	31	10164	-1.4928	0.93226
-	LOC1053769	19	22445376	22567491	511	46	10164	-1.494	0.93241
+	ABHD1	2	27088780	27140991	86	12	10164	-1.4948	0.93251
-	ITPKB	1	226621690	226774327	437	46	10164	-1.4953	0.93258
+	LRTOMT	11	72045331	72120782	174	9	10164	-1.4953	0.93259
+	GALNT15	3	16139579	16256333	406	46	10164	-1.4956	0.93262
-	SLITRK6	13	85782787	85834348	174	13	10164	-1.4958	0.93265
-	ANTXR2	4	79891617	80108472	522	32	10164	-1.4961	0.93269
+	MLEC	12	120652124	120711864	182	23	10164	-1.4962	0.9327
-	GPR63	6	96784122	96872492	273	24	10164	-1.4962	0.9327
+	TNFRSF17	16	11930107	11978068	166	16	10164	-1.4975	0.93287
+	GRM2	3	51672033	51728616	67	14	10164	-1.4991	0.93308
-	NCOA2	8	70099770	70440437	677	27	10164	-1.4997	0.93315
-	MED13	17	61932605	62100311	291	29	10164	-1.4997	0.93316
-	PNPLA5	22	43869678	43927013	132	23	10164	-1.4997	0.93316
-	LRP2BP	4	185353878	185431935	161	20	10164	-1.4998	0.93317
-	TVP23A	16	10751364	10853859	162	25	10164	-1.4999	0.93317
+	RNF217	6	124927486	125102633	349	37	10164	-1.5005	0.93326
+	ANKRD20A1	9	67824286	67912394	1	1	10164	-1.5019	0.93344
+	KCNK1	1	233579004	233682512	371	54	10164	-1.5029	0.93357
+	PIFO	1	111289515	111363017	388	30	10164	-1.5041	0.93372
+	LPCAT2	16	55474001	55596670	385	32	10164	-1.5042	0.93374
+	SNX17	2	27335496	27387533	69	14	10164	-1.5047	0.93379
-	STRA13	17	82008703	82057930	55	5	10164	-1.5052	0.93386
-	PRM3	16	11263199	11308595	181	16	10164	-1.5053	0.93387
+	SUPT6H	17	28627203	28712237	76	16	10164	-1.5054	0.93389
-	IFT27	22	36748202	36811133	120	17	10164	-1.5054	0.93389
-	ADAMTS4	1	161179748	161234055	93	17	10164	-1.5063	0.934
-	RPL26	17	8367516	8418247	150	23	10164	-1.5063	0.93401
-	C17orf85	17	3792160	3881251	104	24	10164	-1.5064	0.93401
+	ECE2	3	184214657	184303031	189	25	10164	-1.507	0.9341
+	LIN7B	19	49079361	49128460	108	18	10164	-1.5072	0.93412
-	CECR5	22	17127520	17200287	315	32	10164	-1.5081	0.93424
-	GLT6D1	9	135613656	135676216	139	20	10164	-1.5081	0.93424
+	LMO7	13	75585434	75869870	745	48	10164	-1.5085	0.93428
+	PDCD4	10	110836795	110910006	134	26	10164	-1.5089	0.93433
-	TAS2R42	12	11175993	11221937	150	12	10164	-1.509	0.93435
-	CEBPA	19	33289934	33337564	145	14	10164	-1.5093	0.93438
+	CSF2	5	132038792	132086170	123	19	10164	-1.51	0.93448
+	PRDM8	4	80148741	80214329	208	16	10164	-1.5104	0.93452
-	SH2D3A	19	6742160	6802512	86	9	10164	-1.5109	0.93459
-	LOC1053777	8	852574	917930	255	36	10164	-1.5113	0.93464

STRAND	SYMBOL	CHR	START	STOP	NSNPS	NPARAM	N	ZSTAT	P
+	DSTN	20	17534173	17618242	155	23	10164	-1.5117	0.9347
+	LOC1053792	8	11229160	11294725	260	39	10164	-1.5118	0.93471
-	SNX8	7	2241770	2349475	294	28	10164	-1.5121	0.93474
+	KCNK3	2	26657713	26741198	172	22	10164	-1.5123	0.93477
-	ADGRF5	6	46842505	46989954	480	38	10164	-1.5127	0.93482
-	SLC6A15	12	84849488	84948615	139	16	10164	-1.513	0.93486
-	SLC46A3	13	28690080	28754013	128	11	10164	-1.5134	0.93491
-	SHISA8	22	41899533	41950093	143	12	10164	-1.5135	0.93492
+	LOC339862	3	17927572	18278922	787	41	10164	-1.5143	0.93503
-	TAF1C	16	84167847	84222070	331	39	10164	-1.5146	0.93506
-	PDS5A	4	39812863	40012956	643	34	10164	-1.5147	0.93508
+	TTC8	14	88789153	88887996	200	23	10164	-1.5154	0.93516
-	ARFGEF1	8	67165296	67378897	284	18	10164	-1.5158	0.93522
-	TCP1	6	159768498	159824703	173	13	10164	-1.516	0.93523
+	NDUFS6	5	1766382	1826051	233	22	10164	-1.516	0.93524
-	ACSL6	5	131939973	132047068	206	15	10164	-1.5161	0.93525
-	SCARB2	4	76148737	76248899	470	32	10164	-1.5162	0.93526
+	LOC1005069	3	88303295	88478050	861	46	10164	-1.5164	0.93529
-	LOC1053769	19	49046589	49095965	145	20	10164	-1.5166	0.93532
-	LRRC75A	17	16432005	16527191	172	19	10164	-1.5171	0.93538
-	SRM	1	11044592	11095034	163	9	10164	-1.5173	0.9354
+	RNF13	3	149777484	149972139	378	30	10164	-1.5176	0.93544
+	LCN9	9	135628322	135676769	108	14	10164	-1.5181	0.9355
-	MEIKIN	5	131796990	131980698	268	16	10164	-1.5184	0.93554
-	PRKAB2	1	147145106	147207589	135	17	10164	-1.5193	0.93566
-	PRTFDC1	10	24802957	24988751	536	52	10164	-1.5193	0.93566
-	TUBA1B	12	49117782	49166521	108	10	10164	-1.5195	0.93568
+	ZNF326	1	89960119	90038538	35	4	10164	-1.5195	0.93569
+	MYOM2	8	2010043	2155456	736	48	10164	-1.5198	0.93571
-	PRDM10	11	129889706	130038050	507	36	10164	-1.5201	0.93576
+	WDR90	16	614363	677833	255	24	10164	-1.5204	0.9358
+	KLKB1	4	186175753	186268474	362	27	10164	-1.5206	0.93582
+	LOC1053733	2	1763741	1821738	224	26	10164	-1.5208	0.93584
-	MFSD6L	17	8787110	8834349	134	26	10164	-1.5211	0.93588
-	STX10	19	13134058	13186334	21	2	10164	-1.5215	0.93593
-	PHC2	1	33313623	33465610	451	26	10164	-1.5228	0.9361
+	BPIFB2	20	32972578	33033709	215	22	10164	-1.5231	0.93613
-	KIF3A	5	132679161	132772633	214	18	10164	-1.5231	0.93614
-	PERP	6	138078505	138142523	239	30	10164	-1.5236	0.93619
-	ZASP	7	100564035	100609733	47	18	10164	-1.5236	0.9362
+	SERPINB7	18	63718047	63815376	248	18	10164	-1.5244	0.9363
-	GJB6	13	20211962	20267395	186	23	10164	-1.5249	0.93635
-	RACGAP1	12	49979162	50068136	187	20	10164	-1.5253	0.93641
+	BMP2	20	6732694	6790263	74	20	10164	-1.5263	0.93654
-	SIGLEC1	20	3676970	3747617	191	23	10164	-1.5276	0.93669
+	FAHD2A	2	95367691	95426609	73	25	10164	-1.5282	0.93676
+	ELMOD3	2	85319720	85401752	217	17	10164	-1.5282	0.93677
-	C2orf81	2	74404176	74452717	33	3	10164	-1.5284	0.93679
+	LGALS3	14	55094217	55155430	174	20	10164	-1.5287	0.93682
-	PLOD3	7	101195977	101252730	155	19	10164	-1.5288	0.93684
+	CRCT1	1	152479502	152526010	137	14	10164	-1.5288	0.93685
-	ZBTB41	1	197143684	197235542	204	13	10164	-1.5291	0.93688
+	NADSYN1	11	71418171	71511538	371	18	10164	-1.5296	0.93694
+	AACS	12	125030379	125153327	340	30	10164	-1.5311	0.93713
+	RAB40C	16	554357	639273	293	17	10164	-1.5316	0.93718
+	MCF2L	13	112859378	113109739	913	90	10164	-1.5317	0.9372
+	SCGN	6	25617201	25711783	429	41	10164	-1.5317	0.93721
-	SPDYE4	17	8736868	8794412	156	20	10164	-1.532	0.93723
-	RIN1	11	66322064	66372133	120	17	10164	-1.5322	0.93726
-	FGFR1	8	38401138	38503834	207	35	10164	-1.5324	0.93729
-	ZDHHC24	11	66511379	66581200	206	23	10164	-1.5325	0.9373
-	NKX6-1	4	84481987	84534141	52	9	10164	-1.5332	0.93738
+	C1QA	1	22601625	22649682	192	26	10164	-1.5333	0.93739

STRAND	SYMBOL	CHR	START	STOP	NSNPS	NPARAM	N	ZSTAT	P
-	SLITRK3	3	165176720	165231681	90	13	10164	-1.5339	0.93748
+	DLX4	17	49934198	49984959	143	32	10164	-1.5349	0.9376
+	ARRB2	17	4675494	4731500	126	13	10164	-1.5351	0.93761
-	PRM2	16	11265636	11311480	194	16	10164	-1.5355	0.93766
+	FNBP1L	1	93413076	93580905	236	27	10164	-1.5355	0.93767
+	ATRIP	3	48411710	48475655	93	10	10164	-1.5357	0.93769
-	PABPN1L	16	88852623	88905156	216	17	10164	-1.5359	0.93771
+	FUT11	10	73737175	73789075	70	11	10164	-1.536	0.93773
+	PPP1R21	2	48405769	48525392	287	37	10164	-1.5372	0.93787
+	AFTPH	2	64489305	64603005	327	18	10164	-1.5376	0.93792
+	CNTN6	3	973135	1413610	1869	156	10164	-1.5381	0.93799
+	GSTM1	1	109652796	109703745	37	5	10164	-1.5382	0.938
+	NFYC	1	40656570	40781603	374	24	10164	-1.5389	0.93809
+	NDUFS2	1	161162419	161224395	91	16	10164	-1.5397	0.93818
-	NEBL	10	20769971	21209187	1369	90	10164	-1.5397	0.93818
-	LOC1019277	15	100907250	100953889	143	22	10164	-1.5398	0.9382
-	MYH10	17	8464205	8666012	332	28	10164	-1.54	0.93821
+	SUCNR1	3	151838643	151892088	124	19	10164	-1.5399	0.93821
-	AICDA	12	8592166	8647888	55	9	10164	-1.5405	0.93828
-	TIGD7	16	3288808	3340439	92	19	10164	-1.5424	0.93851
+	FGF9	13	21636076	21714501	139	24	10164	-1.5425	0.93853
-	TERT	5	1243167	1330047	260	65	10164	-1.5426	0.93853
-	ALDH18A1	10	95595929	95691810	347	24	10164	-1.543	0.93859
+	TSPAN17	5	176612387	176669058	172	34	10164	-1.5432	0.93861
+	FZD6	8	103263433	103342866	242	24	10164	-1.5433	0.93862
-	WHSC1L1	8	38259699	38417354	201	14	10164	-1.5436	0.93866
+	MUC12	7	100934623	101028949	198	30	10164	-1.5438	0.93868
+	MRPL37	1	54165167	54236682	201	32	10164	-1.544	0.9387
-	OR10J3	1	159303670	159349659	137	21	10164	-1.544	0.93871
-	DUSP1	5	172758090	172806200	183	31	10164	-1.5452	0.93885
-	LOC1053751	6	354143	415671	202	45	10164	-1.5453	0.93887
-	OTOP1	4	4178803	4261894	9	2	10164	-1.5456	0.9389
+	WNT10A	2	218839323	218903931	100	24	10164	-1.5457	0.93891
+	DPH1	17	1995137	2053431	150	23	10164	-1.5471	0.93908
+	ING5	2	241665593	241739481	125	9	10164	-1.5474	0.93911
+	SAE1	19	47095823	47220636	149	23	10164	-1.5474	0.93912
+	C9orf62	9	135308249	135356562	56	6	10164	-1.5474	0.93912
-	TEX36	10	125566513	125718154	360	48	10164	-1.5474	0.93912
-	EHD1	11	64842727	64914713	125	17	10164	-1.5486	0.93926
+	MPPED1	22	43377014	43517808	606	69	10164	-1.549	0.93931
+	CD5	11	61067458	61137852	136	12	10164	-1.5505	0.93948
+	LOC1053697	12	37506740	37587517	332	21	10164	-1.5506	0.9395
+	VPS37A	8	17211892	17308024	288	31	10164	-1.5513	0.93958
+	RPL26L1	5	172919294	172979771	181	23	10164	-1.5521	0.93968
+	RBM45	2	178077417	178139656	150	23	10164	-1.5539	0.9399
-	NKX1-1	4	1392932	1441442	96	14	10164	-1.5545	0.93997
-	LOC1053719	17	35231324	35279151	213	37	10164	-1.555	0.94003
+	JAK2	9	4950245	5138183	619	35	10164	-1.5551	0.94004
-	OR8J3	11	56126771	56172718	154	16	10164	-1.5552	0.94005
-	CMKLR1	12	108278044	108374317	262	41	10164	-1.5555	0.94008
-	CHCHD4	3	14102077	14159871	134	22	10164	-1.5556	0.94009
+	SULT1C3	2	108204974	108275608	186	9	10164	-1.5561	0.94016
+	AGGF1	5	76995385	77075234	216	33	10164	-1.5563	0.94018
-	STX19	3	94004365	94063776	98	34	10164	-1.5563	0.94019
+	PRR5	22	44633547	44747681	420	55	10164	-1.5567	0.94022
-	LOC1053791	5	103422459	103538988	287	28	10164	-1.557	0.94027
-	HRH3	20	62204766	62255278	135	25	10164	-1.5578	0.94036
+	C14orf28	14	44862304	44917257	144	14	10164	-1.5579	0.94037
+	CLEC18A	16	69915394	69974347	111	13	10164	-1.5582	0.9404
-	FAM96B	16	66922055	66969423	49	11	10164	-1.5587	0.94047
+	DUSP27	1	167059077	167139897	187	17	10164	-1.5594	0.94055
-	ECEL1	2	232469827	232522859	160	32	10164	-1.5595	0.94056
-	FAM46B	1	26995020	27047842	35	15	10164	-1.5596	0.94057

STRAND	SYMBOL	CHR	START	STOP	NSNPS	NPARAM	N	ZSTAT	P
+	ATG4D	19	10508894	10563423	248	29	10164	-1.5603	0.94065
-	HEATR4	14	73468484	73593947	310	47	10164	-1.5609	0.94073
+	CFHR4	1	196853014	196928812	209	21	10164	-1.5611	0.94075
-	IL5RA	3	3056324	3145374	374	70	10164	-1.5621	0.94086
+	NPAS1	19	46984820	47055775	102	21	10164	-1.5623	0.94089
+	NIT1	1	161083072	161135445	69	11	10164	-1.5626	0.94093
-	MOGAT3	7	101185729	101236060	153	19	10164	-1.5627	0.94094
+	PTK2B	8	27276482	27469391	648	37	10164	-1.5631	0.94098
-	DCDC1	11	31252624	31404810	227	16	10164	-1.5632	0.941
-	OR5T2	11	56222106	56268185	147	14	10164	-1.5634	0.94102
+	C17orf49	17	6979737	7027524	127	15	10164	-1.5641	0.9411
+	ADAM32	8	39072531	39294917	435	20	10164	-1.565	0.94121
+	FCER1G	1	161180297	161229248	82	16	10164	-1.5651	0.94122
+	ACTR2	2	65192695	65281253	188	16	10164	-1.566	0.94133
+	PCBD2	5	134870120	134972646	184	18	10164	-1.5661	0.94134
-	ETFB	19	51335155	51401418	214	34	10164	-1.5679	0.94155
+	KCNG2	18	79762963	79913195	387	40	10164	-1.5683	0.94159
+	SPATA31C1	9	87878936	87933657	127	26	10164	-1.5684	0.94161
-	CENPM	22	41917763	41982164	165	16	10164	-1.5691	0.94169
+	NFX1	9	33255420	33381157	115	15	10164	-1.5692	0.9417
+	ADIRF	10	86933431	86980909	173	25	10164	-1.5699	0.94178
-	CST11	20	23440404	23487845	164	10	10164	-1.5702	0.94181
-	OMG	17	31284650	31332362	60	5	10164	-1.5704	0.94184
-	OR6N1	1	158755744	158801682	154	20	10164	-1.5707	0.94188
-	ADCY5	3	123272296	123483638	541	51	10164	-1.5712	0.94193
+	AP5Z1	7	4740631	4804395	73	3	10164	-1.5711	0.94193
+	TMEM43	3	14089940	14153680	146	21	10164	-1.5712	0.94193
-	MMD2	7	4882245	4994213	371	32	10164	-1.5712	0.94193
+	ZNF286A	17	15664577	15730787	113	19	10164	-1.5722	0.94204
+	OR5T3	11	56217200	56263222	159	15	10164	-1.5724	0.94207
+	HOXD4	2	176115394	176163223	146	33	10164	-1.5726	0.94209
-	YIPF7	4	44611970	44686641	292	22	10164	-1.5726	0.9421
+	MORC3	21	36285189	36386646	145	21	10164	-1.5729	0.94212
-	HECW2	2	196189000	196628692	1155	69	10164	-1.5728	0.94212
+	TAS2R39	7	143148419	143194435	8	3	10164	-1.5742	0.94228
-	KIAA2013	1	11909588	11961428	191	23	10164	-1.5746	0.94233
-	LOC1009966	14	68595297	68721949	597	84	10164	-1.575	0.94237
+	GAD2	10	26181307	26314562	348	14	10164	-1.5751	0.94238
+	HOXD8	2	176094694	176142695	147	39	10164	-1.5751	0.94239
+	TP63	3	189531861	189907279	1261	107	10164	-1.5754	0.94242
-	PROS1	3	93863037	94009090	218	51	10164	-1.5756	0.94244
+	PLXNA1	3	126947119	127047392	196	17	10164	-1.5767	0.94256
+	WSCD1	17	6034106	6134427	138	25	10164	-1.5769	0.94259
-	C22orf34	22	49404524	49692542	651	46	10164	-1.5769	0.94259
-	CGREF1	2	27089353	27154127	109	13	10164	-1.5772	0.94263
+	OR5D13	11	55738438	55784382	415	7	10164	-1.5787	0.9428
+	ZC3H7B	22	41266503	41370147	221	27	10164	-1.5789	0.94282
+	CTHRC1	8	103336509	103393005	228	23	10164	-1.5795	0.94289
+	LOC1009965	22	41334082	41380396	108	14	10164	-1.5799	0.94294
+	LOC1053714	16	88890447	88940657	272	14	10164	-1.5803	0.94298
-	FABP1	2	88112982	88163131	140	19	10164	-1.5811	0.94307
+	KALRN	3	123998339	124736325	2544	129	10164	-1.5812	0.94309
-	HPSE2	10	98447077	99270875	2067	50	10164	-1.5815	0.94312
+	TAF15	17	35774455	35857242	167	16	10164	-1.5816	0.94313
-	SOD2	6	159669116	159728321	173	16	10164	-1.5817	0.94315
-	ACTC1	15	34778096	34830726	129	34	10164	-1.5819	0.94317
+	ASPHD2	22	26394314	26455012	192	26	10164	-1.5831	0.9433
-	SAAL1	11	18070343	18141091	183	11	10164	-1.5834	0.94333
+	SLC25A15	13	40754411	40822460	224	21	10164	-1.5834	0.94334
-	APBA3	19	3740773	3796698	43	8	10164	-1.5839	0.94339
-	DMRTA2	1	50407550	50458447	72	10	10164	-1.584	0.94341
-	AAAS	12	53297456	53356628	97	11	10164	-1.5847	0.94348
+	AKAP12	6	151204999	151368559	736	99	10164	-1.5847	0.94348

STRAND	SYMBOL	CHR	START	STOP	NSNPS	NPARAM	N	ZSTAT	P
-	CLMN	14	95171939	95354908	552	49	10164	-1.5857	0.94359
-	GOLPH3	5	32114718	32209319	133	13	10164	-1.5858	0.94361
-	HOXA13	7	27183419	27235266	63	10	10164	-1.5859	0.94362
-	TARSL2	15	101643752	101759442	129	8	10164	-1.5859	0.94362
-	STARD4	5	111486033	111547536	151	18	10164	-1.5868	0.94372
+	ERI1	8	8967804	9110256	633	36	10164	-1.5872	0.94377
+	ASB4	7	95426542	95550231	335	40	10164	-1.5873	0.94378
-	TRIM45	1	117101055	117156789	50	6	10164	-1.588	0.94386
+	C5orf38	5	2716943	2765397	182	24	10164	-1.5881	0.94387
+	UNC5A	5	176775559	176890898	196	33	10164	-1.5886	0.94393
+	TBXAS1	7	139743248	140030325	682	92	10164	-1.5889	0.94396
+	NNMT	11	114260813	114322516	116	21	10164	-1.589	0.94397
+	JPH3	16	87566835	87708156	658	67	10164	-1.589	0.94397
+	BBS1	11	66475648	66543613	169	16	10164	-1.5893	0.944
-	MEX3B	15	82031778	82081143	133	26	10164	-1.5902	0.94411
-	SCFD2	4	52862982	53401111	1202	53	10164	-1.5916	0.94426
-	KRT2	12	52634558	52687175	254	30	10164	-1.5918	0.94428
-	CADM4	19	43612367	43674839	177	38	10164	-1.592	0.9443
-	KRTAP1-5	17	41016026	41062202	218	20	10164	-1.5923	0.94434
+	ARL13B	3	93945134	94065678	182	46	10164	-1.5924	0.94435
-	SERPINA12	14	94471651	94552844	236	42	10164	-1.5932	0.94444
-	PITX3	10	102220189	102276474	94	13	10164	-1.5947	0.94462
+	B4GALT7	5	177565118	177620347	127	10	10164	-1.5951	0.94465
-	COMMD2	3	149728470	149787510	171	26	10164	-1.5951	0.94465
+	TIGIT	3	114258986	114320288	62	17	10164	-1.595	0.94465
-	ETV3L	1	157082043	157134808	153	24	10164	-1.5952	0.94467
-	SETD2	3	47006408	47198977	300	20	10164	-1.5953	0.94468
-	LRRTM1	2	80278280	80339593	163	32	10164	-1.5953	0.94468
+	SMG8	17	59175010	59225250	105	12	10164	-1.5963	0.94479
+	WDR54	2	74386733	74435755	39	3	10164	-1.5963	0.94479
-	KIF21A	12	39283228	39478390	481	37	10164	-1.597	0.94486
+	IGFL2	19	46043528	46205097	359	35	10164	-1.5971	0.94487
-	GDPD4	11	77206558	77336699	314	17	10164	-1.5974	0.94491
+	TMEM176A	7	150765525	150815120	202	32	10164	-1.5975	0.94493
-	DYTN	2	206641621	206753396	356	31	10164	-1.5983	0.94501
-	MTMR2	11	95822880	95959207	359	22	10164	-1.5987	0.94505
+	CILP2	19	19503265	19556659	90	18	10164	-1.5989	0.94508
+	WNT16	7	121290367	121351104	121	10	10164	-1.5993	0.94512
-	NCKAP1	2	182914851	183073858	197	13	10164	-1.5998	0.94518
+	TPO	2	1378461	1552727	724	53	10164	-1.6007	0.94528
+	AKR1C3	10	5013766	5117686	476	34	10164	-1.6009	0.9453
+	TFDP1	13	113549688	113651473	398	21	10164	-1.6012	0.94534
-	LEFTY1	1	225876282	225924146	181	26	10164	-1.6014	0.94536
-	LOC1053758	8	47090920	47203769	212	20	10164	-1.6015	0.94537
-	CCL14	17	35973656	36021728	122	15	10164	-1.6016	0.94538
-	RYK	3	134147133	134285843	294	15	10164	-1.6021	0.94543
+	CEP72	5	577290	673500	384	24	10164	-1.6021	0.94543
+	ATP2A2	12	110246227	110361093	120	19	10164	-1.6029	0.94552
+	FAM195A	16	606849	658474	192	21	10164	-1.6031	0.94554
-	SMCO4	11	93468472	93621855	539	48	10164	-1.6035	0.94558
+	SNRNP70	19	49050140	49118613	187	26	10164	-1.6035	0.94559
+	COL27A1	9	114095331	114322516	647	101	10164	-1.6036	0.94559
+	PDK2	17	50059737	50121369	170	22	10164	-1.6038	0.94562
-	JPH4	14	23558035	23613800	29	8	10164	-1.6038	0.94562
+	ART3	4	75976180	76122802	771	23	10164	-1.6042	0.94567
+	ARMC6	19	18998578	19068178	105	8	10164	-1.6049	0.94575
+	XKR4	8	55067457	55536155	1591	99	10164	-1.6052	0.94578
+	RFPL3	22	32319885	32371161	337	27	10164	-1.6053	0.94579
+	CCDC9	19	47221474	47281953	115	11	10164	-1.6074	0.94602
+	ARV1	1	230944075	231010733	237	20	10164	-1.6078	0.94606
-	TFCP2L1	2	121206587	121320209	199	27	10164	-1.608	0.94608
-	LOC1001290	8	54455594	54505426	177	26	10164	-1.6082	0.9461
-	TMPRSS13	11	117890641	117964453	327	43	10164	-1.6086	0.94615

STRAND	SYMBOL	CHR	START	STOP	NSNPS	NPARAM	N	ZSTAT	P
-	LOC1053719	17	29358857	29427458	133	15	10164	-1.6089	0.94618
-	SAPCD2	9	137052124	137105576	93	12	10164	-1.609	0.94619
+	SEC24C	10	73709281	73782175	121	15	10164	-1.6095	0.94625
-	OR10Q1	11	58217882	58263918	213	17	10164	-1.6102	0.94632
-	FAM198B	4	158114580	158208050	158	10	10164	-1.6115	0.94647
+	CABS1	4	70299954	70347116	169	10	10164	-1.6116	0.94647
+	PRPSAP2	17	18821299	18941287	69	2	10164	-1.6125	0.94657
+	FAM167B	1	32212217	32258860	64	5	10164	-1.613	0.94662
+	PPFIA3	19	49084389	49161030	182	21	10164	-1.6132	0.94665
-	ZNF717	3	75706445	75820583	486	18	10164	-1.6132	0.94665
+	LOC1009962	18	79109752	79159693	170	15	10164	-1.6132	0.94665
-	SH3RF1	4	169084256	169306098	348	24	10164	-1.6138	0.94671
-	ZNF24	18	35322214	35379462	125	13	10164	-1.614	0.94674
+	POU6F2	7	38942904	39478601	1613	81	10164	-1.615	0.94684
-	OBSL1	2	219539403	219606577	110	15	10164	-1.6161	0.94696
+	YJEFN3	19	19493861	19547584	108	18	10164	-1.6163	0.94699
-	EXOSC10	1	11056613	11134910	252	13	10164	-1.6168	0.94704
+	PAPOLG	2	60721230	60812086	221	13	10164	-1.6168	0.94704
-	DENND5B	12	31372223	31626018	895	33	10164	-1.6168	0.94704
-	MPV17	2	27299490	27360680	74	16	10164	-1.6171	0.94708
-	VDAC1	5	133961875	134040389	182	21	10164	-1.6173	0.94709
+	DPF2	11	65298754	65362980	144	28	10164	-1.6174	0.9471
+	CRIPAK	4	1356552	1405994	145	19	10164	-1.6174	0.9471
+	TNFAIP6	2	151322583	151391245	221	27	10164	-1.6179	0.94716
+	MYOZ2	4	119100784	119197789	336	27	10164	-1.6183	0.9472
-	NAMPT	7	106238285	106320732	133	15	10164	-1.6184	0.94721
-	RGL3	19	11384056	11454342	87	7	10164	-1.6186	0.94724
+	USP13	3	179618145	179799401	282	40	10164	-1.6194	0.94732
+	KCNAB2	1	5957298	6111193	355	40	10164	-1.6196	0.94734
-	XKR5	8	6798517	6870644	286	42	10164	-1.6198	0.94736
-	SLC45A2	5	33934616	34020144	219	39	10164	-1.6203	0.94741
+	USP20	9	129800417	129891838	530	20	10164	-1.6203	0.94742
+	MALT1	18	58636386	58760139	307	27	10164	-1.6204	0.94743
-	NTF4	19	49051080	49100076	147	20	10164	-1.6206	0.94745
+	DCDC2B	1	32174094	32226196	63	7	10164	-1.6209	0.94748
+	RAP1GAP2	17	2720698	3047741	885	108	10164	-1.6219	0.94759
-	TTLL7	1	83859374	84034167	370	16	10164	-1.6226	0.94766
+	LILRB4	19	54608835	54680359	244	21	10164	-1.6249	0.94791
-	KANK3	19	8312582	8378262	71	10	10164	-1.625	0.94792
-	ECH1	19	38805422	38866857	182	21	10164	-1.6253	0.94795
-	FUT7	9	137020174	137067840	100	13	10164	-1.626	0.94803
+	SKP2	5	36116989	36202788	225	29	10164	-1.626	0.94803
+	ELK3	12	96159382	96279835	211	36	10164	-1.6268	0.94811
-	VAV2	9	133751894	134027374	868	86	10164	-1.6271	0.94814
+	GALNT7	4	173133753	173333967	354	19	10164	-1.6271	0.94814
-	EFCAB1	8	48704915	48770311	128	28	10164	-1.6277	0.9482
+	SNX10	7	26256895	26384383	491	47	10164	-1.6278	0.94822
-	TTC13	1	230896241	231013872	407	18	10164	-1.6279	0.94822
-	SCD5	4	82619537	82833857	602	24	10164	-1.628	0.94823
-	STAT2	12	56331597	56395253	58	6	10164	-1.6282	0.94825
+	ZC3H10	12	56083220	56132497	73	13	10164	-1.6282	0.94826
-	PLXNB1	3	48393854	48465324	103	9	10164	-1.6286	0.9483
+	RUNDC1	17	42945510	43003696	55	7	10164	-1.6287	0.94832
-	TMC4	19	54150095	54208250	112	18	10164	-1.6295	0.94839
+	LOC1053769	1	26010895	26059057	98	9	10164	-1.6295	0.9484
-	KRT20	17	40865889	40920243	171	28	10164	-1.6299	0.94843
-	PRR30	2	27126847	27174464	79	14	10164	-1.6308	0.94853
-	NBN	8	89923336	90019733	319	22	10164	-1.6308	0.94854
-	KCNH7	2	162361407	162873747	1299	66	10164	-1.631	0.94856
-	MBD4	3	129420944	129475179	191	18	10164	-1.6323	0.94869
+	TMED9	5	177557212	177606107	104	10	10164	-1.6327	0.94874
+	ADGRG3	16	57633192	57699378	238	41	10164	-1.6327	0.94874
+	PPARGC1B	5	149695252	149867874	606	46	10164	-1.633	0.94877

STRAND	SYMBOL	CHR	START	STOP	NSNPS	NPARAM	N	ZSTAT	P
-	DEFA1	8	6967649	7015122	36	8	10164	-1.6331	0.94878
-	FGF3	11	69799968	69854424	305	29	10164	-1.6335	0.94882
+	MRPL18	6	159755460	159808429	175	10	10164	-1.6335	0.94882
-	PDZD8	10	117270489	117410467	314	14	10164	-1.6338	0.94885
-	TGFBR3	1	91670343	91941002	1075	82	10164	-1.6339	0.94886
+	LRRC71	1	156885608	156952683	178	16	10164	-1.6339	0.94886
+	RNASEK	17	6977417	7024533	133	16	10164	-1.6342	0.94889
-	LOC1053691	2	74397553	74449171	36	4	10164	-1.6342	0.94889
-	LOC1053707	14	71206320	71263231	174	27	10164	-1.6344	0.94891
+	ARRDC4	15	97925703	97983838	190	19	10164	-1.6346	0.94893
-	TRMT6	20	5927833	5985558	252	24	10164	-1.6349	0.94896
+	DAOA	13	105430867	105501034	205	12	10164	-1.6348	0.94896
-	AURKAIP1	1	1363730	1410438	133	7	10164	-1.635	0.94897
-	ALDH3A1	17	19727984	19783433	158	19	10164	-1.6355	0.94903
-	NDUFAF1	15	41377349	41437554	126	17	10164	-1.6356	0.94904
+	TBC1D3K	17	37888805	37945371	12	2	10164	-1.636	0.94908
+	PACRG	6	162692084	163325492	2166	134	10164	-1.6362	0.94911
-	KRT6A	12	52477174	52528397	255	19	10164	-1.6366	0.94914
-	NUGGC	8	28011964	28119063	203	32	10164	-1.6368	0.94916
+	CCDC61	19	45960451	46028616	231	31	10164	-1.6369	0.94917
-	LOC1053718	17	59171320	59218020	101	14	10164	-1.6369	0.94917
+	MRPL45	17	38262023	38333218	26	8	10164	-1.6378	0.94927
+	POLA2	11	65226852	65315598	180	28	10164	-1.6381	0.9493
+	GALNT1	18	35592436	35721834	416	17	10164	-1.6392	0.94941
+	IL18BP	11	71962944	72012919	161	14	10164	-1.6394	0.94944
+	IRF4	6	356739	421443	214	45	10164	-1.6398	0.94947
+	RHBDL1	16	640666	688271	178	19	10164	-1.6398	0.94948
+	TTPAL	20	44440883	44504603	122	18	10164	-1.64	0.94949
-	INSL6	9	4981949	5220710	730	25	10164	-1.6401	0.94951
+	GALNT11	7	151990674	152132347	205	16	10164	-1.6402	0.94951
+	ATF4	22	39485559	39532686	83	9	10164	-1.6409	0.94959
-	SRSF7	2	38733599	38786494	136	20	10164	-1.6413	0.94964
+	LOC1027246	6	111837732	111887015	128	18	10164	-1.6418	0.94969
-	ACRV1	11	125662334	125715898	181	19	10164	-1.6422	0.94973
-	SPINK8	3	48296846	48363341	183	13	10164	-1.6427	0.94977
-	LOC1053715	17	18092005	18152789	158	25	10164	-1.6427	0.94978
+	PLOD1	1	11899667	11985542	325	27	10164	-1.6435	0.94986
+	GNA15	19	3101032	3173769	312	42	10164	-1.6439	0.9499
+	LRRFIP1	2	237592581	237791647	1022	46	10164	-1.6441	0.94993
+	HTR1E	6	86902306	87026679	332	23	10164	-1.6442	0.94994
+	IQCC	1	32170635	32218687	54	7	10164	-1.6446	0.94997
-	RELA	11	65643596	65697972	168	25	10164	-1.6447	0.94999
+	MYL5	4	639212	692028	51	6	10164	-1.6449	0.95001
-	ZNF816	19	52939379	52997911	168	19	10164	-1.645	0.95001
+	PRSS1	7	142705235	142763076	99	7	10164	-1.6453	0.95004
-	MYBPHL	1	109282365	109342041	86	10	10164	-1.6454	0.95005
+	PPP6R2	22	50308317	50455090	538	42	10164	-1.6462	0.95014
-	SKOR2	18	47196203	47284630	304	30	10164	-1.6463	0.95015
-	DNMT3L	21	44236339	44302138	121	16	10164	-1.6464	0.95016
+	PTPN11	12	112383732	112519918	141	28	10164	-1.6466	0.95018
+	ANO3	11	26153821	26678409	2353	53	10164	-1.6468	0.9502
-	VPS54	2	63882150	64054080	461	34	10164	-1.6474	0.95026
+	TRPV2	17	16380542	16447003	66	5	10164	-1.6475	0.95027
+	SDR42E2	16	22127438	22201856	89	15	10164	-1.6475	0.95027
+	SLC35E3	12	68711148	68776073	120	23	10164	-1.6475	0.95028
-	OR52N5	11	5767634	5813667	180	11	10164	-1.6482	0.95034
+	TSPO2	6	41006370	41054337	118	9	10164	-1.6484	0.95037
+	LAIR2	19	54467797	54520690	101	10	10164	-1.6488	0.9504
+	DDHD2	8	38196343	38283885	84	7	10164	-1.6488	0.9504
-	VDR	12	47831537	47940031	414	64	10164	-1.6489	0.95041
-	PCSK1	5	96380336	96468281	220	35	10164	-1.6491	0.95043
-	EDF1	9	136852114	136901336	82	5	10164	-1.6494	0.95047
+	TRRAP	7	98843490	99023243	165	13	10164	-1.6497	0.95049

STRAND	SYMBOL	CHR	START	STOP	NSNPS	NPARAM	N	ZSTAT	P
+	MME	3	154989124	155193729	295	42	10164	-1.6498	0.95051
+	HRH1	3	11102093	11273253	533	64	10164	-1.65	0.95053
+	MDFIC	7	114887154	115029916	217	26	10164	-1.6513	0.95066
+	SERPINB11	18	63667304	63733893	235	12	10164	-1.6514	0.95067
-	STRN	2	36827698	37001557	462	24	10164	-1.6518	0.95072
+	VCAM1	1	100684640	100749045	103	27	10164	-1.6522	0.95075
+	RPL41	12	56081590	56127832	74	13	10164	-1.6526	0.95079
+	RIF1	2	151374883	151544202	481	27	10164	-1.6533	0.95087
-	SCRN1	7	29910103	30025289	234	21	10164	-1.6537	0.95091
+	ZRANB1	10	124881899	124998059	478	41	10164	-1.655	0.95104
+	ACAT2	6	159726957	159789112	145	11	10164	-1.6566	0.9512
+	GPR61	1	109504872	109557260	126	15	10164	-1.6568	0.95122
-	BTBD9	6	38158451	38675547	1625	88	10164	-1.6572	0.95126
+	MATN2	8	97834056	98046720	679	67	10164	-1.6575	0.95129
-	NUP54	4	76104659	76183515	510	12	10164	-1.6578	0.95132
+	ZNF740	12	53145751	53200870	138	18	10164	-1.6581	0.95136
+	LPIN1	2	11642545	11837409	186	19	10164	-1.6587	0.95141
-	OVOS2	12	31101657	31267532	592	44	10164	-1.6592	0.95146
-	STOML1	15	73973218	74029622	173	17	10164	-1.6594	0.95148
+	TEX101	19	43353611	43428615	177	16	10164	-1.6597	0.95151
+	ZNF589	3	48206106	48280990	90	13	10164	-1.6599	0.95153
-	CAPN14	2	31163056	31268971	433	32	10164	-1.6607	0.95161
+	REST	4	56872876	56945845	377	22	10164	-1.6608	0.95163
+	MTURN	7	30099936	30172765	165	29	10164	-1.6609	0.95163
-	SPAG9	17	50952174	51155865	358	28	10164	-1.6612	0.95167
-	TSR1	17	2312678	2372384	103	18	10164	-1.6617	0.95171
-	ETV3	1	157111191	157173591	136	17	10164	-1.6618	0.95172
+	SPERT	13	45667292	45724559	120	20	10164	-1.6625	0.95179
+	ACBD4	17	45097600	45154181	150	31	10164	-1.6627	0.95182
-	GTF3C3	2	196753032	196834768	148	8	10164	-1.6632	0.95186
-	CDIPT	16	29848356	29898288	28	2	10164	-1.6632	0.95187
+	LILRA1	19	54558576	54612724	266	23	10164	-1.6633	0.95187
+	FAM105A	5	14546782	14626180	120	23	10164	-1.6635	0.95189
+	CCDC152	5	42721818	42812437	291	16	10164	-1.6643	0.95197
+	METAP1	4	98960637	99072809	145	11	10164	-1.6644	0.95198
+	TNFRSF10C	8	23067814	23127437	244	44	10164	-1.665	0.95204
+	C4orf47	4	185370235	185459828	172	19	10164	-1.665	0.95204
+	HIST1H2AL	6	27830329	27875798	176	40	10164	-1.6656	0.9521
-	PPP6R1	19	55219779	55293670	149	22	10164	-1.6657	0.95211
-	SLC30A3	2	27244572	27310366	135	19	10164	-1.6662	0.95216
+	RFPL1	22	29399530	29452455	182	30	10164	-1.6667	0.95222
+	ZNF462	9	106825165	107022455	271	39	10164	-1.6668	0.95222
+	PCDH7	4	30685319	31156801	1497	61	10164	-1.668	0.95234
-	ENDOU	12	47699734	47760572	206	28	10164	-1.6683	0.95237
+	PLEKHA8	7	29993361	30140483	354	16	10164	-1.6685	0.95239
-	TLK1	2	170980823	171266314	748	40	10164	-1.6688	0.95242
-	NKG7	19	51361615	51407728	111	24	10164	-1.669	0.95244
+	PAPOLA	14	96467376	96577116	145	9	10164	-1.6696	0.9525
+	EIF4A2	3	186748572	186799897	157	33	10164	-1.6707	0.95261
-	KCNIP4	4	20718606	21983801	4828	192	10164	-1.6711	0.95265
+	SCN1B	19	34995688	35050449	145	13	10164	-1.6714	0.95268
-	CLEC14A	14	38244000	38291370	94	12	10164	-1.6718	0.95272
-	TCTN3	10	95653396	95729143	233	22	10164	-1.6721	0.95275
-	TDP2	6	24639977	24701989	257	30	10164	-1.6724	0.95278
+	MOSPD3	7	100577102	100625384	60	15	10164	-1.6727	0.95281
-	INSRR	1	156830873	156893920	98	19	10164	-1.6728	0.95282
-	C10orf10	10	44966261	45013882	150	31	10164	-1.6729	0.95282
-	PDE10A	6	165317287	165746153	1312	112	10164	-1.6733	0.95287
+	MSANTD1	4	3206598	3275085	236	21	10164	-1.6734	0.95288
-	MGP	12	14871181	14920919	237	14	10164	-1.6742	0.95295
+	ASIC1	12	50022637	50093622	121	18	10164	-1.6744	0.95298
-	PSMA7	20	62126727	62178458	187	9	10164	-1.6751	0.95304
-	KIAA1755	20	38200505	38295772	265	37	10164	-1.6763	0.95316

STRAND	SYMBOL	CHR	START	STOP	NSNPS	NPARAM	N	ZSTAT	P
+	ALDOA	16	30018090	30080420	58	5	10164	-1.677	0.95323
-	APOL2	22	36216209	36274954	216	28	10164	-1.677	0.95323
+	ZNF331	19	53468880	53590269	486	39	10164	-1.6775	0.95328
-	HIST1H4K	6	27821174	27866527	167	37	10164	-1.6786	0.95338
-	SPINK2	4	56799860	56857176	301	26	10164	-1.6788	0.95341
-	TFF1	21	42352282	42401535	142	29	10164	-1.6789	0.95342
+	MAFF	22	38166932	38226511	250	20	10164	-1.6801	0.95353
+	CHPT1	12	101662639	101739074	288	37	10164	-1.6803	0.95355
-	TPH1	11	18010537	18075788	113	12	10164	-1.6804	0.95356
+	GTPBP3	19	17299982	17352731	199	23	10164	-1.6828	0.9538
-	ALS2	2	201690263	201816172	380	23	10164	-1.6829	0.95381
-	XCL2	1	168530765	168578997	138	10	10164	-1.6832	0.95383
-	RLBP1	15	89199867	89256909	168	19	10164	-1.6834	0.95385
+	FAM206A	9	108899393	108961041	185	20	10164	-1.6835	0.95386
+	LINC00452	13	113848717	113934812	224	28	10164	-1.6837	0.95388
-	SULT4A1	22	43814507	43897498	259	22	10164	-1.6838	0.95389
-	SERAC1	6	158099504	158203280	175	9	10164	-1.6844	0.95395
+	MED11	17	4696408	4743610	143	18	10164	-1.6848	0.95399
-	ALG3	3	184232329	184284525	102	16	10164	-1.6849	0.954
-	RSL1D1	16	11824196	11886585	145	14	10164	-1.685	0.95401
+	TEX43	5	126596722	126646282	128	12	10164	-1.6853	0.95403
+	ELAVL4	1	50013014	50213786	245	38	10164	-1.6854	0.95405
+	LOC1053710	15	89364901	89447853	224	34	10164	-1.6855	0.95406
-	FOPNL	16	15855719	15923649	259	6	10164	-1.6856	0.95407
+	HIST1H3H	6	27775064	27820536	178	33	10164	-1.6868	0.95418
+	NDUFA13	19	19481210	19538204	118	19	10164	-1.6868	0.95418
+	PPIP5K2	5	103085254	103213523	223	10	10164	-1.6879	0.95428
-	RETNLB	3	108745639	108792283	114	16	10164	-1.6883	0.95433
+	ALLC	2	3623184	3712671	317	31	10164	-1.6885	0.95434
+	LRRC37A	17	46204319	46347794	11	2	10164	-1.6889	0.95438
+	PCNX	14	70872405	71125382	535	24	10164	-1.6897	0.95445
+	NAT2	8	18356245	18411219	305	23	10164	-1.6903	0.95452
-	EOMES	3	27705949	27757715	163	24	10164	-1.6904	0.95452
-	PDGFC	4	156751611	157006394	480	36	10164	-1.6905	0.95453
-	ZSCAN30	18	35241058	35325259	141	16	10164	-1.6907	0.95455
+	UHRF1BP1	6	34757017	34887514	556	35	10164	-1.6918	0.95465
+	ANXA8L1	10	46340590	46401784	11	3	10164	-1.6923	0.9547
-	OFCC1	6	9692023	9877630	467	47	10164	-1.6926	0.95473
+	SP140L	2	230292166	230413730	470	36	10164	-1.6929	0.95476
+	KNG1	3	186682309	186754410	295	20	10164	-1.6937	0.95484
+	GPR6	6	109943256	109990720	77	21	10164	-1.694	0.95487
+	CHD9	16	53019998	53337502	641	26	10164	-1.6945	0.95492
+	ZNF735	7	64172203	64230290	314	14	10164	-1.695	0.95496
+	DPEP1	16	89578308	89650862	366	40	10164	-1.6963	0.95509
-	HEATR5B	2	36970999	37119375	350	35	10164	-1.6963	0.95509
-	TUSC1	9	25666389	25713858	95	20	10164	-1.6968	0.95513
-	DCTN1	2	74351154	74427087	63	3	10164	-1.6973	0.95518
-	CDH12	5	21740864	22888622	3096	125	10164	-1.6974	0.95519
+	SPCS3	4	176284939	176342245	204	20	10164	-1.6981	0.95526
+	GEN1	2	17718627	17797104	224	17	10164	-1.6989	0.95533
-	CLSPN	1	35710691	35804997	105	9	10164	-1.699	0.95534
-	ASCL2	11	2258498	2305952	284	39	10164	-1.6991	0.95535
-	SLC1A7	1	53077179	53177632	579	60	10164	-1.7004	0.95547
-	PTGES	9	129728336	129812327	335	36	10164	-1.7004	0.95547
+	SGSM2	17	2302512	2391074	154	24	10164	-1.7009	0.95552
-	CCL16	17	35966493	36017889	151	18	10164	-1.7013	0.95555
-	C9orf78	9	129817285	129870293	366	16	10164	-1.7014	0.95556
-	GNG5	1	84488323	84541579	149	13	10164	-1.7016	0.95559
-	SOCS1	16	11244417	11291182	171	18	10164	-1.7017	0.95559
+	SOX12	20	290571	340228	128	17	10164	-1.7029	0.95571
+	SLC39A10	2	195540977	195747702	525	54	10164	-1.7035	0.95576
+	TSPAN14	10	80419278	80532635	329	25	10164	-1.7035	0.95577
-	KRTAP4-6	17	41129433	41175487	112	13	10164	-1.7039	0.9558

STRAND	SYMBOL	CHR	START	STOP	NSNPS	NPARAM	N	ZSTAT	P
-	DCUN1D5	11	103040684	103127215	180	34	10164	-1.7039	0.9558
+	LCNL1	9	136947993	136995758	134	13	10164	-1.7042	0.95582
+	RTF1	15	41382104	41493563	205	21	10164	-1.7055	0.95595
+	LRRC61	7	150287639	150348156	167	12	10164	-1.7055	0.95595
+	VPS8	3	184777137	185062614	623	36	10164	-1.7057	0.95597
-	MPLKIP	7	40122743	40169652	33	2	10164	-1.7058	0.95597
+	CRLS1	20	5971093	6050072	249	32	10164	-1.7069	0.95608
-	SMC6	2	17653812	17788829	353	15	10164	-1.7072	0.95611
-	CAAP1	9	26830685	26927828	292	35	10164	-1.7073	0.95612
+	NEIL3	4	177274837	177372938	323	46	10164	-1.7075	0.95614
-	SPPL3	12	120752510	120939352	568	23	10164	-1.7078	0.95617
+	REL	2	60846495	60938171	161	19	10164	-1.7081	0.95619
+	EIF3I	1	32186867	32241604	78	7	10164	-1.7081	0.95619
+	ISOC1	5	129059749	129124028	211	30	10164	-1.7082	0.9562
-	PCSK6	15	101293928	101524984	946	96	10164	-1.7091	0.95628
-	ZFYVE26	14	67719569	67851611	448	35	10164	-1.7104	0.95641
-	C6orf62	6	24694861	24755836	182	36	10164	-1.7123	0.95658
+	PDZD2	5	31604363	32120932	1469	163	10164	-1.7124	0.95659
-	ZBED2	3	111582900	111630335	93	22	10164	-1.7132	0.95666
+	GAL3ST2	2	241741825	241814287	34	9	10164	-1.7137	0.95671
+	RAB11FIP4	17	31356624	31548217	480	54	10164	-1.7146	0.95679
+	RNF139	8	124439767	124498618	118	14	10164	-1.7147	0.9568
+	CRHBP	5	76917835	76991147	228	33	10164	-1.7148	0.95681
-	KRT72	12	52575589	52637944	376	31	10164	-1.7153	0.95685
+	TPCN2	11	69013882	69170413	793	69	10164	-1.7154	0.95686
+	DRG2	17	18052867	18117985	178	23	10164	-1.7155	0.95687
+	LOC554223	6	29756906	29807807	998	32	10164	-1.7156	0.95688
-	KRT5	12	52504575	52555459	220	25	10164	-1.716	0.95692
+	SPINK7	5	148277427	148325919	171	22	10164	-1.716	0.95692
-	TMEM74B	20	1170561	1223128	51	8	10164	-1.7166	0.95698
-	GGT7	20	34834720	34907858	132	14	10164	-1.7171	0.95702
-	FGFBP3	10	91896588	91944501	103	15	10164	-1.7182	0.95712
+	RGS9	17	65102338	65237703	246	19	10164	-1.7191	0.9572
+	LY6K	8	142664928	142714170	169	15	10164	-1.7192	0.95721
+	HTR5A	7	155035324	155097392	218	19	10164	-1.7205	0.95733
-	PPBP	4	73976439	74023190	92	17	10164	-1.7207	0.95734
+	C11orf80	11	66709736	66853516	186	48	10164	-1.7206	0.95734
-	RESP18	2	219317409	219371785	151	17	10164	-1.7208	0.95735
-	SPEF1	20	3767504	3816466	114	18	10164	-1.721	0.95738
+	HSD3B1	1	119472124	119525060	273	21	10164	-1.7227	0.95753
-	KRTAP4-5	17	41138924	41184802	136	9	10164	-1.7231	0.95756
-	KANSL1	17	46019916	46260374	29	5	10164	-1.723	0.95756
-	GLRX	5	95803849	95857873	129	16	10164	-1.7234	0.95759
+	ZNHIT1	7	101182704	101234192	162	20	10164	-1.7236	0.95761
-	DLGAP3	1	34852120	34964585	131	28	10164	-1.724	0.95764
-	SLC6A3	5	1382790	1480428	378	47	10164	-1.7248	0.95772
+	OR56B4	11	6072684	6118835	261	5	10164	-1.7273	0.95794
+	UBR2	6	42528952	42703505	334	16	10164	-1.7275	0.95796
-	PHOSPHO1	17	49213366	49266692	143	21	10164	-1.73	0.95819
-	SLC15A1	13	98673801	98787675	450	67	10164	-1.7304	0.95822
-	SAMD14	17	50101309	50164882	145	27	10164	-1.7309	0.95826
+	ZFAND2B	2	219171784	219219651	80	11	10164	-1.7319	0.95835
+	TSPAN32	11	2267013	2328201	356	35	10164	-1.7338	0.95853
-	DEFA4	8	6925820	6973338	202	28	10164	-1.734	0.95854
+	PEX13	2	60982677	61061990	131	26	10164	-1.7351	0.95864
-	GAS6	13	113810549	113899103	252	27	10164	-1.7356	0.95868
-	POU3F1	1	38033851	38081778	99	9	10164	-1.7357	0.95869
+	CHST12	7	2368526	2444607	107	13	10164	-1.7368	0.95879
+	RAP2B	3	153127212	153180624	189	21	10164	-1.7376	0.95886
-	ALPK2	18	58471247	58664188	616	67	10164	-1.7376	0.95886
+	KNSTRN	15	40347721	40404288	162	27	10164	-1.741	0.95916
-	RPUSD1	16	774974	823387	187	21	10164	-1.7415	0.9592
-	RFX5	1	151330640	151382293	86	15	10164	-1.7418	0.95923

STRAND	SYMBOL	CHR	START	STOP	NSNPS	NPARAM	N	ZSTAT	P
-	PLBD1	12	14493663	14602857	200	31	10164	-1.7426	0.95929
+	BTBD6	14	105213542	105261093	57	13	10164	-1.7426	0.9593
-	GPR139	16	20021455	20108778	287	30	10164	-1.7432	0.95935
-	TM2D1	1	61671046	61760423	52	10	10164	-1.7436	0.95938
+	PEX5	12	7154163	7228574	192	22	10164	-1.7437	0.95939
+	DCDC2C	2	3668592	3857411	741	57	10164	-1.7441	0.95943
+	C10orf131	10	95872965	95948658	211	21	10164	-1.7447	0.95948
-	SCARF1	17	1623858	1680789	125	11	10164	-1.745	0.95951
-	KRT33B	17	41353494	41404800	93	15	10164	-1.7452	0.95952
-	PPP1R3C	10	91618440	91668101	153	14	10164	-1.7458	0.95958
+	LOC1027247	8	67308647	67385213	118	10	10164	-1.7461	0.9596
-	TMEM234	1	32194806	32257353	87	7	10164	-1.7471	0.95969
-	CMPK2	2	6830417	6901635	44	7	10164	-1.7474	0.95972
-	FAIM2	12	49856896	49938977	242	41	10164	-1.7476	0.95973
+	C4orf32	4	112110397	112199081	218	19	10164	-1.7479	0.95976
+	SAP30	4	173335942	173387532	53	14	10164	-1.7486	0.95982
-	GIN1	5	103076000	103155169	178	6	10164	-1.7486	0.95982
-	STYK1	12	10608923	10709318	310	26	10164	-1.7494	0.95989
-	A3GALT2	1	33296766	33356098	145	18	10164	-1.7498	0.95992
+	LOC1053743	2	27681068	27771921	111	21	10164	-1.7498	0.95992
+	GEMIN6	2	38743185	38792456	144	19	10164	-1.7506	0.96
+	TMEM214	2	26997906	27051697	70	9	10164	-1.7509	0.96002
+	OR10A4	11	6841625	6887619	277	14	10164	-1.751	0.96003
+	FZD1	7	91229468	91278817	106	16	10164	-1.7528	0.96018
-	HIST1H2AK	6	27827880	27873339	178	39	10164	-1.7536	0.96025
-	ECI1	16	2229872	2286601	61	10	10164	-1.7541	0.96029
-	NR2F6	19	17221885	17280342	266	42	10164	-1.7543	0.96031
+	PAN3	13	28101717	28305338	355	19	10164	-1.7543	0.96031
-	CSAD	12	53147663	53215909	151	21	10164	-1.7549	0.96036
-	RIT2	18	42733218	43150692	1033	65	10164	-1.7551	0.96038
-	HIST1H1B	6	27856792	27902581	217	38	10164	-1.7562	0.96047
+	LOC1053742	3	195780607	195828776	117	9	10164	-1.7563	0.96048
+	ACOT13	6	24632035	24715069	321	28	10164	-1.7567	0.96051
+	HIST1H2BM	6	27780044	27825489	175	32	10164	-1.7568	0.96052
-	KIF1A	2	240703764	240856025	506	49	10164	-1.7571	0.96055
+	HIST1H2AI	6	27773199	27818667	181	33	10164	-1.7586	0.96067
+	SMR3A	4	70325776	70377106	247	10	10164	-1.7591	0.96072
-	FKBP14	7	30000583	30061801	133	11	10164	-1.7592	0.96073
-	NRIP1	21	14951235	15100903	365	27	10164	-1.7593	0.96074
+	PIGG	4	464200	549921	216	8	10164	-1.7593	0.96074
-	KCNA7	19	49057418	49107941	111	17	10164	-1.7602	0.96081
-	LOC1053692	6	71241239	71363267	479	31	10164	-1.7603	0.96082
+	ZNF22	10	44965825	45015329	164	31	10164	-1.761	0.96088
+	CSTA	3	122290164	122351969	210	21	10164	-1.7612	0.9609
-	RNF175	4	153697672	153795235	322	22	10164	-1.7613	0.96091
+	FIP1L1	4	53342646	53472611	408	20	10164	-1.762	0.96096
+	STUB1	16	645111	692768	174	19	10164	-1.7624	0.961
+	DRC7	16	57659801	57741455	275	39	10164	-1.7626	0.96101
-	CSNK2A2	16	58147907	58232878	364	26	10164	-1.7628	0.96103
-	UGGT2	13	95791580	96088482	551	21	10164	-1.7628	0.96103
-	TMEM63A	1	225830570	225917720	333	29	10164	-1.763	0.96105
-	VGF	7	101152509	101204935	154	22	10164	-1.7637	0.96111
-	LILRA5	19	54297070	54348139	72	6	10164	-1.7638	0.96112
-	TOR1A	9	129802942	129859245	313	15	10164	-1.7639	0.96113
+	FES	15	90849435	90905776	118	17	10164	-1.7639	0.96113
-	SELP	1	169578849	169665139	300	31	10164	-1.7652	0.96124
-	UBALD1	16	4598883	4649926	8	3	10164	-1.7656	0.96127
+	SPATA3	2	230961124	231035056	263	31	10164	-1.7657	0.96127
-	TM2D3	15	101623226	101687391	58	5	10164	-1.7658	0.96128
+	SLC39A5	12	56195036	56247846	70	11	10164	-1.7668	0.96137
+	OR52N2	11	5773905	5831400	245	14	10164	-1.7668	0.96137
-	ZNF816-ZNF3	19	52917135	52997911	173	20	10164	-1.7669	0.96138
-	OR52N1	11	5777854	5823816	220	13	10164	-1.7676	0.96143

STRAND	SYMBOL	CHR	START	STOP	NSNPS	NPARAM	N	ZSTAT	P
+	SNX30	9	112714725	112885851	509	25	10164	-1.768	0.96147
-	FLT3	13	27993274	28135592	584	45	10164	-1.7687	0.96153
+	LRFN3	19	35902120	35955195	124	12	10164	-1.7687	0.96153
-	SCN2B	11	118152804	118211622	263	32	10164	-1.7692	0.96157
+	FOXE1	9	97818255	97866715	154	12	10164	-1.7696	0.9616
-	DOC2A	16	29995514	30058280	67	3	10164	-1.7708	0.9617
+	HERC6	4	88343535	88453097	173	40	10164	-1.7716	0.96177
-	FAM109A	12	111350651	111404121	103	18	10164	-1.7733	0.96191
-	CRYM	16	21248518	21338136	174	20	10164	-1.7741	0.96198
+	ANO4	12	100682463	101138641	1436	97	10164	-1.7741	0.96198
+	HIST1H2BO	6	27858425	27903891	217	37	10164	-1.7757	0.96211
+	ANKDD1A	15	64876902	64968700	222	25	10164	-1.7764	0.96217
+	MAN1B1	9	137051862	137119187	131	15	10164	-1.7775	0.96226
+	APOL1	22	36218071	36277531	218	29	10164	-1.778	0.96229
+	DAZAP2	12	51203724	51256717	126	17	10164	-1.7778	0.96229
+	MEF2A	15	99530533	99726483	308	16	10164	-1.7782	0.96231
+	RND2	17	42990241	43042041	89	14	10164	-1.7812	0.96256
-	SUMO1	2	202196180	202273599	155	16	10164	-1.7815	0.96258
-	TENM4	11	78643283	79475989	1944	178	10164	-1.7828	0.96269
-	HIPK2	7	139551570	139812894	429	47	10164	-1.7828	0.96269
-	KRT40	17	40967716	41022139	377	22	10164	-1.7839	0.96278
-	MYEOV2	2	240116563	240171347	22	2	10164	-1.7839	0.96278
+	SIRT1	10	67849662	67928390	227	19	10164	-1.7843	0.96282
-	KEAP1	19	10476120	10538378	217	37	10164	-1.785	0.96287
-	LMO2	11	33848576	33927289	130	28	10164	-1.7861	0.96296
-	HEATR1	1	236526173	236639541	584	22	10164	-1.7863	0.96297
+	TEKT2	1	36049075	36098276	76	11	10164	-1.7866	0.963
+	RAB32	6	146508692	146564950	82	12	10164	-1.7885	0.96315
-	E2F2	1	23495974	23566250	143	21	10164	-1.7886	0.96316
+	LOC1053694	11	59988803	60040834	149	12	10164	-1.7887	0.96317
-	PTGES3L-AA	17	42940526	43015528	81	8	10164	-1.7894	0.96323
+	BCO1	16	81203642	81301142	473	42	10164	-1.7895	0.96324
-	HEATR9	17	35844946	35903891	145	13	10164	-1.7902	0.96329
+	MMP15	16	57990378	58056901	169	26	10164	-1.7904	0.96331
+	LEKR1	3	156791307	157057659	783	57	10164	-1.7912	0.96337
+	SORCS3	10	104605837	105275250	1769	61	10164	-1.7918	0.96342
+	LOC1053738	2	201746052	201792110	141	18	10164	-1.7919	0.96343
-	DPH5	1	100979623	101060806	21	10	10164	-1.7929	0.96351
+	AADACL2	3	151698916	151768016	238	27	10164	-1.7933	0.96354
-	TMEM163	2	134445759	134771424	888	68	10164	-1.7941	0.9636
+	NWD2	4	37210068	37459465	847	85	10164	-1.7942	0.96361
+	TEPP	16	57941435	57998354	190	30	10164	-1.7946	0.96364
+	SNCG	10	86923531	86973260	166	28	10164	-1.7952	0.96369
+	POLR3G	5	90439229	90524677	180	29	10164	-1.7954	0.9637
+	FAM25A	10	86985289	87034730	126	21	10164	-1.7961	0.96376
-	ALOX15	17	4620919	4676665	74	16	10164	-1.7966	0.9638
-	HMGB2	4	173321376	173369444	56	8	10164	-1.7968	0.96381
+	STEAP3	2	119187731	119275652	208	22	10164	-1.7972	0.96385
+	LOC1019291	17	18799662	18875564	18	4	10164	-1.798	0.96392
+	C16orf96	16	4503709	4610455	11	5	10164	-1.7985	0.96395
+	LGALS8	1	236483214	236562981	400	25	10164	-1.7986	0.96396
-	PKP2	12	32780746	32931846	585	50	10164	-1.7992	0.96401
+	FRMD6	14	51454100	51740727	1008	116	10164	-1.7993	0.96401
-	COBL	7	51006212	51351818	922	55	10164	-1.7999	0.96406
-	NOA1	4	56953350	57012660	186	26	10164	-1.8002	0.96408
+	HIST1H4J	6	27789125	27834480	170	30	10164	-1.801	0.96415
+	REEP6	19	1456166	1507925	52	5	10164	-1.8014	0.96418
+	METTL20	12	31612160	31679086	278	26	10164	-1.8013	0.96418
-	RIMS3	1	40610680	40727084	349	31	10164	-1.8021	0.96424
-	CNPPD1	2	219161897	219213164	88	11	10164	-1.8033	0.96433
-	S1PR5	19	10502742	10552992	216	30	10164	-1.8034	0.96434
+	ING1	13	110677623	110731074	166	19	10164	-1.8036	0.96435
+	NUDT4	12	93342923	93413248	153	22	10164	-1.8036	0.96435

STRAND	SYMBOL	CHR	START	STOP	NSNPS	NPARAM	N	ZSTAT	P
-	ZNF467	7	149754182	149811323	40	6	10164	-1.8045	0.96442
-	TSSK6	19	19504219	19550660	80	16	10164	-1.8045	0.96443
+	BRI3BP	12	124958648	125061845	377	41	10164	-1.8048	0.96444
+	EVX1	7	27207545	27257819	76	13	10164	-1.8054	0.9645
-	PELP1	17	4661384	4739337	163	18	10164	-1.8057	0.96452
+	TRIO	5	14108708	14520204	911	57	10164	-1.8061	0.96455
-	ZNF12	7	6678433	6741969	192	13	10164	-1.8066	0.96459
+	AP1S1	7	101119405	101171276	166	30	10164	-1.808	0.9647
-	HSPBAP1	3	122729997	122830216	233	21	10164	-1.8094	0.96481
+	FLRT2	14	85495105	85638696	443	48	10164	-1.8099	0.96485
-	TMX3	18	68663688	68750298	200	18	10164	-1.8102	0.96487
-	OR52B4	11	4357263	4403386	163	17	10164	-1.8121	0.96501
+	ANKRD30A	10	37090742	37245831	238	26	10164	-1.8126	0.96505
+	CAND1	12	67234281	67324692	228	14	10164	-1.813	0.96508
-	SLC38A2	12	46348188	46407862	164	22	10164	-1.8137	0.96513
-	CMTR2	16	71272300	71324606	188	20	10164	-1.814	0.96516
+	IL3	5	132025654	132073203	130	14	10164	-1.8144	0.96519
+	SPATA17	1	217596324	217878151	792	38	10164	-1.8143	0.96519
-	XPC	3	14135147	14213672	200	26	10164	-1.8144	0.9652
-	MMP8	11	102701795	102759964	288	37	10164	-1.8156	0.96528
-	PDYN	20	1968756	2029285	114	15	10164	-1.8159	0.96531
-	PPM1K	4	88247609	88319831	174	26	10164	-1.817	0.96539
-	GIF	11	59819268	59880537	60	12	10164	-1.8173	0.96541
+	MARCH7	2	159677457	159778583	358	20	10164	-1.8173	0.96541
+	TG	8	132831935	133144902	925	40	10164	-1.8175	0.96543
-	H3F3C	12	31781185	31827241	249	27	10164	-1.8176	0.96544
+	OR10A2	11	6834755	6880666	281	14	10164	-1.8179	0.96546
-	PRKAR1B	7	539196	762676	137	16	10164	-1.818	0.96547
+	DEFB108B	11	71798200	71847562	247	16	10164	-1.818	0.96547
+	PRR5-ARHG/	22	44667198	44872784	684	86	10164	-1.819	0.96554
-	MEGF11	15	65885296	66288737	1040	108	10164	-1.8195	0.96558
-	MAGI1	3	65320764	66073836	2343	176	10164	-1.8205	0.96566
-	RNF38	9	36326396	36522182	247	28	10164	-1.8205	0.96566
+	MUM1	19	1319977	1388431	34	2	10164	-1.8212	0.96571
-	LPCAT4	15	34348888	34402196	33	7	10164	-1.8213	0.96572
+	PPP4C	16	30040976	30095377	41	6	10164	-1.8244	0.96595
-	FAM135B	8	138120023	138531822	1095	95	10164	-1.8245	0.96596
-	ANKRD52	12	56227807	56293359	77	6	10164	-1.8245	0.96596
-	NTRK3	15	87864999	88291796	1300	104	10164	-1.8249	0.96599
-	SORBS1	10	95301773	95596420	960	70	10164	-1.8259	0.96606
-	SPOCK3	4	166723384	167269796	1806	100	10164	-1.8264	0.9661
-	LOC1010603	7	55736453	55785700	158	14	10164	-1.8267	0.96613
-	NOL4	18	33841100	34259834	638	48	10164	-1.8269	0.96614
-	LGI2	4	24988849	25065792	327	42	10164	-1.8268	0.96614
-	AMACR	5	33976986	34043115	177	18	10164	-1.8273	0.96617
-	SLC39A6	18	36098531	36164814	197	18	10164	-1.8286	0.96627
+	MKRN3	15	23530307	23578020	87	10	10164	-1.8287	0.96628
+	KIR2DL1	19	54734805	54794326	4	2	10164	-1.8294	0.96633
+	NLRP4	19	55801086	55891855	461	66	10164	-1.8313	0.96647
-	PLAA	9	26893370	26982470	295	21	10164	-1.8318	0.96651
+	DUSP23	1	159745940	159792543	91	22	10164	-1.8327	0.96657
-	PSMB7	9	124343465	124450473	187	28	10164	-1.8333	0.96662
-	ANAPC15	11	72099587	72147776	178	22	10164	-1.8335	0.96664
-	LY6D	8	142774882	142821592	191	11	10164	-1.8339	0.96667
-	KRT73	12	52592883	52655116	378	36	10164	-1.8347	0.96672
-	ASZ1	7	117353222	117462523	148	17	10164	-1.8355	0.96678
+	B3GNT6	11	76999341	77051973	109	10	10164	-1.8356	0.96679
-	UBQLN4	1	156023135	156088825	94	29	10164	-1.8362	0.96683
-	KANK4	1	62226165	62354412	364	27	10164	-1.8363	0.96684
-	AMIGO1	1	109494178	109544717	128	14	10164	-1.8367	0.96687
+	DEPDC7	11	32980864	33043582	165	9	10164	-1.837	0.96689
-	ATG2A	11	64884532	64952250	107	31	10164	-1.8376	0.96694
+	NECAB2	16	83930185	84012776	437	37	10164	-1.8379	0.96696

STRAND	SYMBOL	CHR	START	STOP	NSNPS	NPARAM	N	ZSTAT	P
+	STEAP2	7	90176686	90247678	213	18	10164	-1.8381	0.96698
-	SLC4A5	2	74206242	74378407	160	14	10164	-1.8384	0.967
+	NPAS4	11	66386004	66436706	121	16	10164	-1.8384	0.967
+	SFTPA1	10	79575939	79625443	193	16	10164	-1.839	0.96704
+	POLR2C	16	57427287	57482009	119	18	10164	-1.8402	0.96713
-	FLT1	13	28290346	28530128	513	56	10164	-1.841	0.96719
+	TAOK1	17	29355925	29561904	371	16	10164	-1.8419	0.96725
+	DLL3	19	39463917	39518481	131	13	10164	-1.8423	0.96728
+	ST8SIA3	18	57317490	57381800	146	19	10164	-1.8428	0.96732
-	HIST1H2BL	6	27797479	27842931	165	32	10164	-1.843	0.96733
-	ZNF513	2	27367231	27415866	73	8	10164	-1.8433	0.96736
-	CLDND2	19	51356957	51404241	131	26	10164	-1.8439	0.9674
+	CCDC174	3	14616746	14682659	185	21	10164	-1.8446	0.96745
-	MRPL13	8	120385844	120480407	147	11	10164	-1.845	0.96748
+	TNFAIP8L1	19	4604514	4665568	14	3	10164	-1.8454	0.96751
-	PF4	4	73970825	74017124	105	20	10164	-1.8461	0.96756
+	SPRR1B	1	152996203	153042900	242	6	10164	-1.8465	0.96759
+	ESRRB	14	76336299	76511839	434	51	10164	-1.8472	0.96764
-	CAMK2B	7	44207150	44360994	271	32	10164	-1.8499	0.96784
+	FOLR3	11	72100682	72149892	192	24	10164	-1.8508	0.9679
+	WTAP	6	159691998	159766319	183	15	10164	-1.8516	0.96796
+	OR10R2	1	158444878	158490885	126	10	10164	-1.8517	0.96797
-	PAFAH2	1	25949764	26033581	167	11	10164	-1.8526	0.96803
-	TAF5L	1	229583111	229661047	160	12	10164	-1.8532	0.96807
-	ENTPD2	9	137038098	137089069	99	12	10164	-1.8555	0.96824
+	LOC1053695	11	125792568	125838337	88	14	10164	-1.8556	0.96824
-	SOGA3	6	127428406	127554355	355	34	10164	-1.8562	0.96828
-	TNFRSF10B	8	22996383	23104187	420	64	10164	-1.8566	0.96832
-	SPTB	14	64736283	64914893	704	52	10164	-1.8576	0.96839
-	ZNF484	9	92832573	92913058	171	18	10164	-1.8576	0.96839
-	PDE11A	2	177613249	178143339	1966	55	10164	-1.859	0.96849
+	TRIM54	2	27247429	27317439	126	18	10164	-1.8593	0.9685
-	KRT76	12	52758155	52812345	236	46	10164	-1.8598	0.96855
+	ZDHHC13	11	19082099	19186420	294	25	10164	-1.8601	0.96857
-	MMP27	11	102681510	102740755	314	28	10164	-1.861	0.96862
-	LDAH	2	20674014	20858137	285	26	10164	-1.8615	0.96867
-	C3	19	6667835	6755682	193	27	10164	-1.862	0.9687
+	OR5T1	11	56240639	56286619	138	13	10164	-1.862	0.9687
+	PPIL2	22	21630984	21707913	194	18	10164	-1.8622	0.96871
+	PRR25	16	770443	823861	206	23	10164	-1.8622	0.96871
+	ALOX12	17	6959196	7020754	166	22	10164	-1.8624	0.96872
+	APOLD1	12	12690917	12801466	253	47	10164	-1.8625	0.96873
+	ZDHHC2	8	17121020	17232732	337	50	10164	-1.8626	0.96874
+	NSF	17	46555669	46767464	74	6	10164	-1.8632	0.96878
+	TMEM204	16	1493741	1565580	285	28	10164	-1.8656	0.96895
+	MYL6	12	56123261	56171582	58	11	10164	-1.8657	0.96896
-	EID2	19	39528806	39575198	123	16	10164	-1.866	0.96898
+	ENTPD1	10	95675906	95887266	569	24	10164	-1.8666	0.96902
+	MRGPRX3	11	18068652	18148491	210	14	10164	-1.8667	0.96903
-	C20orf27	20	3743499	3803388	113	20	10164	-1.8669	0.96904
-	PRODH	22	18902695	18971553	19	2	10164	-1.8676	0.96909
+	ELP2	18	36094874	36184725	256	22	10164	-1.869	0.96919
+	TTYH1	19	54380426	54446719	78	9	10164	-1.8696	0.96923
-	TMPRSS15	21	18259107	18438806	508	54	10164	-1.8698	0.96924
+	C2orf40	2	106028294	106088155	183	30	10164	-1.8712	0.96934
-	KRT12	17	40851178	40902210	120	19	10164	-1.8721	0.96941
-	TXNDC11	16	11669080	11777878	288	26	10164	-1.8724	0.96943
+	VASP	19	45472430	45536989	196	29	10164	-1.8739	0.96952
-	CCDC110	4	185435182	185506759	204	22	10164	-1.8739	0.96953
-	PAXIP1	7	154933687	155038178	295	22	10164	-1.8757	0.96965
+	ADGRG6	6	142266919	142456266	306	31	10164	-1.8756	0.96965
+	RPS6KA1	1	26494758	26585030	221	26	10164	-1.8768	0.96973
+	SUSD3	9	93023688	93095138	225	29	10164	-1.8776	0.96978

STRAND	SYMBOL	CHR	START	STOP	NSNPS	NPARAM	N	ZSTAT	P
-	DLGAP1	18	3486032	4490310	1446	162	10164	-1.8782	0.96982
+	PMPCB	7	103262426	103339902	81	16	10164	-1.8791	0.96989
-	DHFRL1	3	94047922	94098389	84	36	10164	-1.8809	0.97001
+	TNFSF13B	13	108234420	108318484	184	34	10164	-1.8817	0.97006
+	C3orf56	3	127158131	127208185	113	19	10164	-1.8828	0.97014
-	C20orf62	20	44451763	44500344	90	15	10164	-1.883	0.97015
+	ACSS2	20	34839942	34937966	169	14	10164	-1.8836	0.97019
-	DEFB114	6	49950292	49999105	79	9	10164	-1.8836	0.97019
-	SLURP1	8	142730944	142777411	124	16	10164	-1.8852	0.9703
+	GSN	9	121175803	121342844	434	30	10164	-1.8854	0.97031
-	STAU1	20	49103339	49223370	263	19	10164	-1.8855	0.97032
-	PSMG1	21	39165446	39218514	168	18	10164	-1.8855	0.97032
-	CERKL	2	181526672	181692107	423	29	10164	-1.8856	0.97033
-	RARRES2	7	150328318	150376674	180	14	10164	-1.8893	0.97057
+	CDC25B	20	3751772	3816121	153	25	10164	-1.8894	0.97058
+	CLSTN2	3	139900185	140578077	1761	146	10164	-1.8898	0.97061
+	CMTM8	3	32203679	32380321	527	73	10164	-1.8899	0.97061
+	IL17RA	22	17049959	17125694	240	30	10164	-1.8901	0.97063
-	DAD1	14	22554907	22624237	209	29	10164	-1.8907	0.97067
+	SEH1L	18	12912984	12997537	278	38	10164	-1.8911	0.97069
-	GNGT2	17	49194442	49245574	152	25	10164	-1.8931	0.97083
+	MAN2A2	15	90867218	90932585	140	24	10164	-1.8941	0.97089
+	ARFGEF2	20	48886721	49046693	429	19	10164	-1.8944	0.97091
+	SOCS4	14	54992126	55059489	116	12	10164	-1.8959	0.97101
+	RNF135	17	30923921	31009911	37	16	10164	-1.8967	0.97107
+	CHMP2B	3	87192263	87265548	248	37	10164	-1.8977	0.97114
+	C1orf101	1	244417100	244650362	427	35	10164	-1.8981	0.97116
-	SAYSD1	6	39094063	39150186	201	23	10164	-1.8988	0.97121
+	CCL8	17	34284047	34331402	114	11	10164	-1.8998	0.97127
-	CCL5	17	35861491	35915373	160	11	10164	-1.9002	0.9713
+	LILRB1	19	54581930	54648167	311	23	10164	-1.9005	0.97132
+	PTGDS	9	136942504	136991742	149	13	10164	-1.9009	0.97134
+	ST3GAL6	3	98697236	98807054	339	38	10164	-1.9027	0.97146
+	EIF2B5	3	184099435	184155311	133	19	10164	-1.9028	0.97147
+	PPM1N	19	45462975	45512510	159	23	10164	-1.9032	0.97149
+	SERPINE1	7	101092089	101149266	198	29	10164	-1.9033	0.9715
+	RGS4	1	163033606	163086802	174	24	10164	-1.9047	0.97159
+	LOC1019292	19	23797189	23881182	238	26	10164	-1.9051	0.97162
+	GTF3C5	9	132995675	133068503	234	27	10164	-1.9058	0.97166
-	LAMTOR1	11	72087292	72138387	164	16	10164	-1.9063	0.97169
-	SKA3	13	21143595	21211602	152	17	10164	-1.9063	0.97169
-	RTN2	19	45475288	45532055	177	27	10164	-1.9077	0.97178
-	CCDC167	6	37472920	37534924	215	30	10164	-1.908	0.9718
-	CCK	3	42247826	42301170	202	24	10164	-1.9082	0.97182
+	BMPR1A	10	86720786	86935188	503	28	10164	-1.9087	0.97185
-	GRIP1	12	66337398	67104267	2421	185	10164	-1.9093	0.97189
+	FBXO34	14	55236303	55363611	348	19	10164	-1.9097	0.97192
-	QRFPR	4	121317211	121416059	473	26	10164	-1.9098	0.97192
-	DEFB1	8	6860575	6913007	339	21	10164	-1.9104	0.97196
+	NABP2	12	56187093	56239854	74	8	10164	-1.9104	0.97196
+	HIST1H2BN	6	27803662	27849110	167	29	10164	-1.9106	0.97197
-	XPO1	2	61467934	61573522	194	15	10164	-1.9107	0.97198
+	CCDC106	19	55612568	55663161	77	13	10164	-1.9111	0.972
-	TMEM179	14	104580864	104639787	158	14	10164	-1.9116	0.97203
+	SLC35F3	1	233869712	234334518	1422	111	10164	-1.9122	0.97207
-	DOCK9	13	98783487	99123618	995	27	10164	-1.9156	0.97229
-	ZNF396	18	35356697	35412337	115	14	10164	-1.9171	0.97239
-	LYSMD1	1	151146810	151200948	53	16	10164	-1.9177	0.97243
+	CFH	1	196616878	196757504	376	21	10164	-1.9179	0.97244
-	CXCL5	4	73985642	74033729	79	17	10164	-1.9181	0.97245
+	PA2G4	12	56069319	56123910	84	14	10164	-1.9203	0.97259
+	ZMYND15	17	4705015	4756119	164	27	10164	-1.9203	0.97259
+	CCDC28B	1	32165386	32215390	62	7	10164	-1.9206	0.97261

STRAND	SYMBOL	CHR	START	STOP	NSNPS	NPARAM	N	ZSTAT	P
+	CRADD	12	93642351	93904742	777	91	10164	-1.9211	0.97264
-	NOVA2	19	45923734	46008399	240	33	10164	-1.9217	0.97268
-	SLC23A3	2	219151459	219205095	104	11	10164	-1.9219	0.97269
+	INHA	2	219537232	219585713	106	13	10164	-1.9221	0.9727
+	RSAD2	2	6830840	6908232	50	8	10164	-1.9226	0.97274
+	PAX2	10	100700709	100839941	160	21	10164	-1.9237	0.97281
-	SLFN12	17	35399650	35468229	234	36	10164	-1.9238	0.97281
+	ZNF181	19	34698316	34752872	208	22	10164	-1.925	0.97288
-	REXO4	9	133396058	133453172	167	12	10164	-1.9253	0.97291
+	ZFP3	17	5043459	5106374	237	28	10164	-1.9254	0.97291
+	ITPRIPL2	16	19078932	19131630	29	6	10164	-1.9259	0.97294
+	USB1	16	57964600	58031623	188	26	10164	-1.926	0.97295
+	SMIM5	17	75598434	75651406	49	9	10164	-1.9272	0.97302
+	METRNL	17	83044691	83105129	274	32	10164	-1.9276	0.97305
+	CECR2	22	17324949	17568155	950	91	10164	-1.9278	0.97306
-	DPY19L1	7	34918879	35073041	444	18	10164	-1.9282	0.97308
+	WISP1	8	133156039	133241690	177	31	10164	-1.9283	0.97309
+	ATAD5	17	30796476	30905869	247	6	10164	-1.9286	0.97311
-	NDUFA6	22	42075526	42125884	124	8	10164	-1.9289	0.97313
+	OR8K3	11	56283307	56329245	168	15	10164	-1.9292	0.97315
-	GOLGA8M	15	28688695	28753510	34	3	10164	-1.9299	0.97319
-	SEC14L6	22	30514606	30581717	176	33	10164	-1.9305	0.97323
-	PLOD2	3	146059436	146196495	362	19	10164	-1.9313	0.97327
-	BSDC1	1	32354633	32429461	35	10	10164	-1.932	0.97332
-	TMEM8C	9	133504586	133563606	51	9	10164	-1.9343	0.97346
-	TRIM16	17	15617966	15717879	156	23	10164	-1.9346	0.97348
+	FBXO36	2	229887491	230023109	174	19	10164	-1.9358	0.97355
+	ZNF358	19	7480282	7531025	114	21	10164	-1.936	0.97357
-	SLC10A6	4	86777607	86884360	324	32	10164	-1.936	0.97357
-	PVRL4	1	161060991	161124595	91	18	10164	-1.9364	0.97359
+	ARPC1A	7	99290873	99376262	145	17	10164	-1.9395	0.97378
-	FUT5	19	5855826	5905540	107	9	10164	-1.94	0.97381
+	SPEF2	5	35582779	35824611	941	54	10164	-1.9406	0.97384
-	PEX11G	19	7466870	7532485	162	29	10164	-1.9408	0.97386
-	INHBA	7	41679003	41738108	104	15	10164	-1.9413	0.97389
-	SLC25A24	1	108124722	108235358	278	27	10164	-1.9415	0.9739
+	SNAP25	20	10183829	10317420	324	46	10164	-1.9426	0.97396
-	WDHD1	14	54928938	55062101	245	19	10164	-1.9425	0.97396
+	SMARCA2	9	1980219	2203624	799	104	10164	-1.9434	0.97401
+	PGM2	4	37791660	37872938	417	22	10164	-1.9433	0.97401
-	PPAPDC1B	8	38251790	38304437	60	8	10164	-1.9432	0.97401
-	H3F3B	17	75766434	75814935	43	6	10164	-1.9434	0.97402
+	SRSF3	6	36559313	36614467	128	13	10164	-1.9437	0.97403
-	RTL1	14	100870655	100919847	48	14	10164	-1.9442	0.97406
+	CCL2	17	34220277	34267203	69	7	10164	-1.9448	0.9741
-	SFXN5	2	72932036	73106836	234	12	10164	-1.9463	0.97419
-	C7orf62	7	88784106	88830773	187	23	10164	-1.9469	0.97423
+	SEMA6D	15	47149101	47784223	2221	102	10164	-1.9473	0.97425
+	ZNF679	7	64193474	64276931	404	27	10164	-1.9476	0.97427
+	INPP1	2	190308470	190381665	202	19	10164	-1.9482	0.9743
+	MYO15A	17	18073706	18190225	310	37	10164	-1.9494	0.97438
+	C11orf42	11	6170043	6241450	161	17	10164	-1.9495	0.97438
+	RSPH10B2	7	6719108	6808775	93	7	10164	-1.951	0.97447
+	NCAPG	4	17775813	17854865	176	18	10164	-1.9512	0.97449
+	KIR2DL4	19	54768612	54824517	111	7	10164	-1.9514	0.9745
+	DUSP19	2	183043224	183110008	109	8	10164	-1.9524	0.97455
+	UCN3	10	5330013	5384206	298	36	10164	-1.9525	0.97456
-	OPA3	19	45516863	45619864	298	47	10164	-1.9535	0.97462
-	GLT8D2	12	103978984	104099183	356	42	10164	-1.9541	0.97465
-	PRKACA	19	14081688	14152747	37	10	10164	-1.9551	0.97472
-	GPRIN1	5	176585802	176652338	177	43	10164	-1.9553	0.97473
+	LTBP4	19	40557888	40639820	127	10	10164	-1.9555	0.97474
-	CRLF3	17	30772684	30859760	246	5	10164	-1.9557	0.97475

STRAND	SYMBOL	CHR	START	STOP	NSNPS	NPARAM	N	ZSTAT	P
-	LYRM4	6	5021753	5295950	1053	62	10164	-1.9563	0.97479
-	DCAF16	4	17790655	17845769	119	13	10164	-1.9585	0.97491
+	FAM43A	3	194650893	194699037	177	12	10164	-1.9585	0.97491
+	CHD1L	1	147207641	147305766	398	21	10164	-1.9586	0.97492
+	TMEM144	4	158166604	158265287	61	14	10164	-1.9604	0.97502
-	ABCA3	16	2265878	2375746	39	7	10164	-1.9607	0.97504
-	LOC1053765	10	3522040	3591794	214	26	10164	-1.962	0.97512
+	CXCR2	2	218090290	218147253	131	12	10164	-1.9624	0.97514
-	NME6	3	48282317	48337246	145	7	10164	-1.9626	0.97516
+	NUSAP1	15	41297694	41391050	217	21	10164	-1.9627	0.97516
+	LCK	1	32216239	32296170	98	12	10164	-1.9649	0.97529
-	MYO5B	18	49812786	50230081	1668	87	10164	-1.9654	0.97531
+	TIPARP	3	156639416	156716770	249	28	10164	-1.9667	0.97539
-	UCP3	11	73990281	74044237	111	20	10164	-1.9668	0.9754
+	IRGC	19	43681058	43730036	175	24	10164	-1.9672	0.97542
+	SH3GL3	15	83412123	83628743	514	86	10164	-1.9674	0.97543
-	OR8H1	11	56280127	56326062	162	15	10164	-1.9674	0.97543
+	DRAXIN	1	11651647	11730612	206	39	10164	-1.9687	0.9755
-	C17orf51	17	21429840	21586678	36	5	10164	-1.97	0.97558
+	TRMT12	8	124415807	124463026	172	24	10164	-1.9703	0.9756
-	FBXW5	9	136930433	136979774	156	13	10164	-1.9705	0.97561
-	DNAJC2	7	103302474	103379873	79	15	10164	-1.9719	0.97569
+	B3GALT5	21	39577940	39672889	285	50	10164	-1.9731	0.97576
+	LOC1053771	22	35279113	35324890	26	4	10164	-1.9731	0.97576
+	ABHD4	14	22563238	22622923	164	27	10164	-1.9739	0.9758
+	PRMT6	1	107021645	107069294	81	6	10164	-1.9752	0.97588
-	TCEA3	1	23370909	23459779	200	22	10164	-1.9765	0.97595
+	ADPRHL2	1	36053852	36103932	87	13	10164	-1.9766	0.97596
+	EFCAB2	1	244934739	245135228	438	19	10164	-1.9782	0.97604
-	ANXA11	10	80142610	80240677	264	25	10164	-1.9783	0.97605
-	MRGPRD	11	68970021	69015986	166	25	10164	-1.9792	0.97611
-	FRRS1	1	99698632	99801630	293	10	10164	-1.9793	0.97611
+	TUSC5	17	1244663	1310987	100	16	10164	-1.9797	0.97613
-	KCNC2	12	75030078	75244748	499	38	10164	-1.9798	0.97614
-	SMARCC2	12	56151852	56224567	95	9	10164	-1.9801	0.97616
-	FAM57B	16	30014427	30088012	70	5	10164	-1.9804	0.97617
+	RYR3	15	33275783	33876103	1922	187	10164	-1.9831	0.97632
-	MAGEF1	3	184700367	184747048	122	27	10164	-1.9838	0.97636
-	LIPI	21	14098463	14246040	753	45	10164	-1.9842	0.97638
-	NR3C1	5	143267931	143470512	523	33	10164	-1.9844	0.97639
-	SLC38A1	12	46173055	46304425	314	45	10164	-1.9852	0.97644
+	PCDH10	4	133114290	133218606	275	27	10164	-1.9856	0.97646
+	NKAIN2	6	123768837	124835640	2465	175	10164	-1.9867	0.97652
+	ARL10	5	176330474	176425463	270	49	10164	-1.9871	0.97654
-	SELENBP1	1	151354302	151407734	86	21	10164	-1.9874	0.97656
+	CHRM3	1	239351565	239921462	1044	91	10164	-1.989	0.97665
-	MAD2L2	1	11664480	11726788	181	36	10164	-1.9897	0.97669
+	PRR31	9	136933886	136986820	158	14	10164	-1.9898	0.97669
+	TRAT1	3	108787784	108864867	227	24	10164	-1.9914	0.97678
+	HHLA2	3	108261485	108398971	398	19	10164	-1.9929	0.97686
-	MMRN2	10	86925540	87004953	252	34	10164	-1.9935	0.9769
-	CHPF	2	219528947	219578765	118	15	10164	-1.9938	0.97691
-	ARHGEF3	3	56717418	57114308	1393	107	10164	-1.9955	0.97701
+	AGAP11	10	86935741	87020203	261	33	10164	-1.9967	0.97707
-	FAM83H	8	143713933	143773030	94	6	10164	-1.9995	0.97722
-	HIST1H2AJ	6	27804302	27849740	166	30	10164	-2.0009	0.9773
+	FAM110A	20	798697	856279	103	18	10164	-2.001	0.9773
-	BUB1	2	110627527	110713107	101	17	10164	-2.0011	0.97731
-	IL17F	6	52226681	52280689	179	26	10164	-2.0022	0.97737
+	LOC1053761	9	89619416	89672115	184	30	10164	-2.0021	0.97737
-	CNOT7	8	17216030	17281878	180	26	10164	-2.0031	0.97742
+	SMCO2	12	27411744	27512322	413	24	10164	-2.0035	0.97744
-	NAT16	7	101160493	101230908	225	28	10164	-2.0046	0.97749

STRAND	SYMBOL	CHR	START	STOP	NSNPS	NPARAM	N	ZSTAT	P
+	SPNS2	17	4463834	4549933	132	24	10164	-2.0051	0.97752
+	MET	7	116637405	116808386	298	37	10164	-2.006	0.97757
-	IGFBP7	4	57021071	57145385	574	59	10164	-2.0067	0.97761
+	C19orf45	19	7462559	7525233	157	28	10164	-2.007	0.97762
+	SPATA12	3	57025441	57086068	234	38	10164	-2.0074	0.97765
-	RNF41	12	56192504	56256982	86	11	10164	-2.0077	0.97766
+	STT3A	11	125557795	125632759	261	24	10164	-2.0094	0.97775
-	CCDC51	3	48422170	48476078	77	7	10164	-2.0107	0.97782
+	SLC25A46	5	110703136	110775161	176	26	10164	-2.0113	0.97785
-	C7orf60	7	112809147	112974877	160	13	10164	-2.0118	0.97788
+	TREX1	3	48430520	48477645	64	6	10164	-2.0145	0.97802
+	TSC22D2	3	150373335	150470119	64	7	10164	-2.0149	0.97804
-	OR6K2	1	158689678	158735652	116	10	10164	-2.0153	0.97807
-	MIS18BP1	14	45193190	45288402	190	29	10164	-2.0159	0.9781
-	ASF1B	19	14109509	14171628	24	7	10164	-2.019	0.97826
+	ZNF580	19	55599903	55653470	84	16	10164	-2.0198	0.9783
+	PPP3CC	8	22405970	22551144	8	3	10164	-2.0215	0.97838
-	MPO	17	58259855	58319313	106	16	10164	-2.0227	0.97845
-	PEX5L	3	179784958	180072053	176	22	10164	-2.0227	0.97845
-	PRKCDBP	11	6308946	6355510	99	13	10164	-2.0233	0.97848
-	OSR1	2	19341485	19393611	79	16	10164	-2.0246	0.97854
+	AMZ1	7	2644522	2775679	512	50	10164	-2.0246	0.97855
-	ZNF436	1	23349448	23404864	140	16	10164	-2.0259	0.97861
-	TMPRSS3	21	42361887	42431846	214	27	10164	-2.0261	0.97862
-	KIAA1191	5	176336061	176396807	170	32	10164	-2.0271	0.97867
+	ARHGAP8	22	44717558	44872784	510	70	10164	-2.0297	0.97881
+	TGFBR1	9	99069038	99164192	184	21	10164	-2.0303	0.97884
-	PUM2	2	20238692	20387229	190	15	10164	-2.0303	0.97884
+	IL20	1	206830354	206879223	102	22	10164	-2.0314	0.97889
-	ZNF705E	11	71804045	71856547	255	18	10164	-2.0315	0.9789
-	DPYSL3	5	147380808	147545260	354	24	10164	-2.0356	0.9791
-	CD244	1	160820158	160897902	187	9	10164	-2.0356	0.97911
-	SCN4B	11	118123377	118187915	261	39	10164	-2.0377	0.97921
-	IPCEF1	6	154144483	154391802	692	68	10164	-2.0377	0.97921
+	MRAS	3	138312648	138415535	138	7	10164	-2.0383	0.97924
+	SNRPC	6	34722094	34783857	299	17	10164	-2.0384	0.97925
+	S1PR4	19	3143738	3190332	155	29	10164	-2.0393	0.97929
-	UBE2E2-AS1	3	23185070	23238045	162	19	10164	-2.0395	0.9793
+	LOC642574	8	143731664	143800701	88	7	10164	-2.0411	0.97938
+	CASC3	17	40105254	40182178	38	12	10164	-2.0414	0.97939
-	TRPC4AP	20	34992404	35127837	405	18	10164	-2.0417	0.97941
-	COL23A1	5	178227616	178625555	1414	133	10164	-2.0419	0.97942
-	KIRREL3	11	126413493	127038460	1403	133	10164	-2.0422	0.97943
-	WIPI1	17	68411281	68492512	202	27	10164	-2.0428	0.97946
+	KBTBD8	3	66963303	67021211	81	9	10164	-2.045	0.97957
+	CCL17	16	57361190	57426063	156	21	10164	-2.0461	0.97963
+	ATG4B	2	241602612	241683857	181	16	10164	-2.0468	0.97966
+	ARHGEF10	8	1788983	1968641	435	44	10164	-2.0473	0.97968
-	LOC1053790	5	77762649	77811194	208	25	10164	-2.0476	0.9797
-	IFT22	7	101303367	101356823	212	21	10164	-2.0494	0.97979
-	KCNH1	1	210668314	211169115	1575	96	10164	-2.0501	0.97982
+	GZF1	20	23326639	23383046	184	18	10164	-2.0504	0.97984
+	MCOLN1	19	7487610	7544009	132	24	10164	-2.0506	0.97985
+	LHX2	9	123976610	124043163	171	24	10164	-2.051	0.97986
-	EID2B	19	39521533	39567867	123	16	10164	-2.051	0.97987
+	EPT1	2	26311086	26405891	184	18	10164	-2.0538	0.98
-	MARCKSL1	1	32323829	32371239	21	8	10164	-2.0548	0.98005
+	TSSK3	1	32327261	32374325	21	8	10164	-2.0548	0.98005
-	B3GALNT2	1	235430660	235539481	247	11	10164	-2.0549	0.98005
-	GTF2A1	14	81165452	81256231	206	23	10164	-2.0554	0.98008
+	CDH26	20	59920831	60044338	61	5	10164	-2.0565	0.98013
-	RTN4R	22	20231415	20303293	89	17	10164	-2.0566	0.98014
-	PRKACG	9	69002529	69049123	141	24	10164	-2.0569	0.98015

STRAND	SYMBOL	CHR	START	STOP	NSNPS	NPARAM	N	ZSTAT	P
+	ARHGAP24	4	85112742	86012670	2478	92	10164	-2.0599	0.9803
-	LOC1053781	6	170120197	170177348	278	17	10164	-2.0604	0.98032
+	EIF4E1B	5	176595640	176656641	155	38	10164	-2.0607	0.98033
+	C16orf72	16	9056680	9129698	294	20	10164	-2.061	0.98035
-	CUEDC1	17	57851243	57990323	122	27	10164	-2.0625	0.98042
-	CSRP3	11	19172030	19245571	190	35	10164	-2.0631	0.98045
+	USP17L1	8	7297387	7343979	2	1	10164	-2.0642	0.9805
+	USP17L4	8	7302115	7348707	2	1	10164	-2.0642	0.9805
+	PTPN1	20	50475321	50594762	252	31	10164	-2.0649	0.98053
+	VRK1	14	96762347	96891614	348	25	10164	-2.0649	0.98053
-	PTGES3L	17	42958088	43015528	66	8	10164	-2.0648	0.98053
+	SUOX	12	55962259	56015525	101	16	10164	-2.0652	0.98055
+	STK31	7	23675167	23842513	566	27	10164	-2.066	0.98058
+	NUP93	16	56695105	56854950	391	36	10164	-2.0661	0.98059
-	KDM5A	12	270057	424455	536	31	10164	-2.0665	0.98061
+	LOC1019281	9	133154786	133202966	144	19	10164	-2.0673	0.98065
+	EYA2	20	46859624	47198844	509	51	10164	-2.0686	0.98071
-	PAK1	11	77311551	77509063	377	20	10164	-2.0688	0.98072
-	MBLAC2	5	90448203	90509768	127	22	10164	-2.07	0.98077
-	PIK3R6	17	8792722	8902697	236	41	10164	-2.0703	0.98079
-	NPDC1	9	137029457	137081224	110	14	10164	-2.0713	0.98084
+	KIF1BP	10	68953721	69026983	189	20	10164	-2.0721	0.98087
+	CPEB2	4	14967451	15080153	270	23	10164	-2.0723	0.98088
+	STAMBP	2	73793916	73883656	60	4	10164	-2.0726	0.9809
+	CD96	3	111507079	111675996	346	39	10164	-2.0733	0.98093
-	IGSF3	1	116564398	116702755	105	20	10164	-2.0757	0.98104
-	SLC15A4	12	128783194	128858996	299	20	10164	-2.076	0.98105
+	LOC1019298	19	54480664	54537372	145	16	10164	-2.0764	0.98107
+	DTX4	11	59136430	59218587	241	24	10164	-2.0767	0.98109
+	GTF2H5	6	158133347	158209344	141	10	10164	-2.0769	0.9811
+	REPIN1	7	150333229	150384044	169	14	10164	-2.0777	0.98113
-	CDC42BPG	11	64813809	64879673	145	21	10164	-2.0776	0.98113
-	HSPBP1	19	55252222	55315383	119	21	10164	-2.0781	0.98115
-	MBL2	10	52755380	52807841	337	23	10164	-2.0802	0.98125
-	KY	3	134589923	134686677	240	20	10164	-2.0809	0.98128
-	LENG1	19	54145639	54194743	116	18	10164	-2.0821	0.98134
+	CHTF18	16	753622	808074	183	19	10164	-2.0827	0.98136
-	LYRM9	17	27868314	27928383	108	25	10164	-2.0827	0.98136
-	LYZL6	17	35924518	35978710	233	21	10164	-2.084	0.98142
-	EPHA4	2	221408027	221609202	484	43	10164	-2.0848	0.98146
-	THAP4	2	241574405	241672525	165	19	10164	-2.0869	0.98155
+	POLR2B	4	56943640	57041162	335	28	10164	-2.0871	0.98156
+	LCN12	9	136914195	136965498	145	13	10164	-2.0871	0.98156
-	SP7	12	53316575	53379793	91	14	10164	-2.0905	0.98171
-	ETV7	6	36344221	36422800	285	37	10164	-2.093	0.98182
+	TBC1D26	17	15697277	15754784	64	15	10164	-2.0932	0.98183
-	CCDC182	17	57734481	57780329	150	22	10164	-2.0953	0.98193
+	CHN2	7	29111547	29524335	1257	141	10164	-2.0972	0.98201
-	KRT10	17	40808117	40857618	118	15	10164	-2.0981	0.98205
-	ZNF80	3	114224631	114272578	72	13	10164	-2.099	0.98209
+	FGF17	8	22004709	22058809	120	28	10164	-2.0996	0.98212
+	RAB25	1	156026175	156080504	82	27	10164	-2.0997	0.98212
+	LRRK1	15	100884215	101080112	791	57	10164	-2.1003	0.98215
-	NIPSNAP1	22	29544808	29616337	235	20	10164	-2.1011	0.98218
+	BCL2L2	14	23271762	23321759	88	20	10164	-2.1016	0.98221
+	ERBB3	12	56045025	56113507	94	15	10164	-2.1022	0.98223
-	LOXL2	8	23286897	23439209	1086	75	10164	-2.1026	0.98225
+	GPD1	12	50068819	50121320	78	10	10164	-2.1043	0.98232
+	KIR3DL1	19	54781438	54840778	166	9	10164	-2.1042	0.98232
-	BZRAP1	17	58291227	58363795	159	27	10164	-2.1048	0.98234
-	C12orf73	12	103940202	103992215	203	24	10164	-2.1056	0.98238
+	ETV6	12	11614854	11905402	914	136	10164	-2.1067	0.98243
-	SLC25A21	14	36667318	37207660	1621	104	10164	-2.1068	0.98243

STRAND	SYMBOL	CHR	START	STOP	NSNPS	NPARAM	N	ZSTAT	P
-	ARHGAP30	1	161036942	161104971	120	20	10164	-2.1072	0.98245
-	GPR12	13	26745200	26795785	86	15	10164	-2.1075	0.98246
+	ATP6V0E1	5	172948760	173044897	201	24	10164	-2.1074	0.98246
-	CIAPIN1	16	57418174	57482457	143	20	10164	-2.1074	0.98246
+	IGIP	5	140090936	140138806	33	7	10164	-2.1085	0.98251
+	KIAA1841	2	61030870	61167942	269	23	10164	-2.1089	0.98252
+	C20orf197	20	60020925	60082953	66	12	10164	-2.1088	0.98252
+	PADI1	1	17170123	17256006	236	25	10164	-2.1122	0.98266
+	LAMTOR2	1	156019726	156068510	67	20	10164	-2.1123	0.98267
-	ATXN2	12	111442214	111634676	257	27	10164	-2.1127	0.98269
-	ALG5	13	36939770	37034367	84	13	10164	-2.1134	0.98272
+	AIRE	21	44250838	44308219	162	20	10164	-2.1146	0.98277
-	SEZ6L2	16	29861159	29934264	64	4	10164	-2.1151	0.98279
-	LRIF1	1	110937190	110999197	86	11	10164	-2.1162	0.98284
-	FAM172A	5	93607725	94146699	659	20	10164	-2.1176	0.9829
+	ACTR3B	7	152724749	152897759	109	13	10164	-2.1181	0.98291
+	PSMC2	7	103309405	103379395	75	14	10164	-2.1196	0.98298
-	WIPF1	2	174549574	174717899	204	27	10164	-2.1198	0.98299
-	RPL39L	3	187110953	187174475	137	15	10164	-2.122	0.98308
-	CCT8	21	29046322	29108797	141	18	10164	-2.1223	0.98309
+	ZNF845	19	53298714	53364869	198	31	10164	-2.123	0.98312
-	DEFB113	6	49958677	50004625	76	10	10164	-2.1235	0.98314
-	LSM1	8	38153321	38211730	96	9	10164	-2.1245	0.98318
+	SNAPC3	9	15387696	15476751	279	18	10164	-2.1266	0.98327
-	GBGT1	9	133142948	133198945	135	23	10164	-2.1269	0.98328
+	LOC1053728	21	44438512	44487220	28	5	10164	-2.1281	0.98334
-	FKBP3	14	45105599	45169529	120	17	10164	-2.1293	0.98338
-	TUBA1A	12	49174795	49224324	87	12	10164	-2.1292	0.98338
-	RILP	17	1636150	1685098	106	8	10164	-2.1302	0.98342
+	EXOSC8	13	36965373	37019614	54	10	10164	-2.1312	0.98346
+	ZNF775	7	150344301	150408635	191	17	10164	-2.1313	0.98347
-	FAM129A	1	184781025	185010138	609	43	10164	-2.1328	0.98353
-	SUPT20H	13	36999312	37094713	84	16	10164	-2.1332	0.98355
+	C8G	9	136909875	136956974	132	13	10164	-2.1378	0.98373
+	RAB31	18	9673231	9872556	590	82	10164	-2.1389	0.98378
-	CALB1	8	90048608	90117881	193	23	10164	-2.1425	0.98392
+	NSUN3	3	94028011	94137740	170	54	10164	-2.1424	0.98392
+	LARP1	5	154647663	154827607	526	56	10164	-2.1445	0.984
-	INTS9	8	28757658	28925389	255	23	10164	-2.1444	0.984
+	GMNN	6	24739931	24796099	210	18	10164	-2.1454	0.98404
-	LOC1053736	2	131372330	131429225	66	4	10164	-2.1454	0.98404
+	TMEM198	2	219508663	219560595	112	15	10164	-2.1456	0.98405
+	F2RL1	5	76784008	76845315	177	20	10164	-2.1466	0.98409
+	CCSER2	10	84293586	84528521	776	29	10164	-2.1471	0.98411
-	BCDIN3D	12	49826043	49878129	139	18	10164	-2.1481	0.98415
+	RPL19	17	39165283	39214727	104	26	10164	-2.1486	0.98417
+	C3orf20	3	14639225	14783036	376	38	10164	-2.1486	0.98417
-	CEP41	7	130383771	130476741	194	21	10164	-2.149	0.98418
-	IL17RD	3	57079982	57205317	330	28	10164	-2.1497	0.98421
-	RPS19BP1	22	39519093	39567855	127	17	10164	-2.1505	0.98424
-	ARMC4	10	27802164	28034067	745	41	10164	-2.1507	0.98425
+	FGF22	19	604895	653703	36	8	10164	-2.1512	0.98427
+	CCDC77	12	354350	452642	369	43	10164	-2.1514	0.98428
+	GORASP2	2	170893438	170977135	182	20	10164	-2.1533	0.98435
-	BBC3	19	47210822	47267996	94	17	10164	-2.1535	0.98436
-	CLPTM1L	5	1307755	1380065	292	47	10164	-2.1538	0.98437
+	GALP	19	56141020	56195775	118	10	10164	-2.1547	0.98441
-	PLAUR	19	43636095	43705346	247	43	10164	-2.1551	0.98442
+	ASPHD1	16	29865826	29930112	61	4	10164	-2.1552	0.98443
-	ARC	8	142601044	142649472	160	19	10164	-2.1567	0.98449
+	NTRK2	9	84633458	85037070	1221	80	10164	-2.1581	0.98454
+	LSM14B	20	62087459	62145378	116	10	10164	-2.1592	0.98458
+	CXCL8	4	73705506	73753716	130	13	10164	-2.16	0.98461

STRAND	SYMBOL	CHR	START	STOP	NSNPS	NPARAM	N	ZSTAT	P
-	ITLN1	1	160866540	160920170	138	8	10164	-2.1601	0.98462
+	GABRA1	5	161812191	161909959	337	28	10164	-2.1611	0.98465
+	LOC1053767	17	5059894	5121791	206	26	10164	-2.1627	0.98472
-	PUS10	2	60928352	61055031	206	39	10164	-2.1653	0.98482
+	SMPD5	8	144012114	144061294	14	3	10164	-2.1668	0.98488
-	ADTRP	6	11702895	11814628	381	40	10164	-2.1683	0.98493
-	GALK1	17	75747937	75800199	25	5	10164	-2.1706	0.98502
-	ABCA2	9	136997234	137063974	123	16	10164	-2.1715	0.98505
+	EDDM3A	14	20710940	20758380	249	34	10164	-2.1727	0.9851
-	CACNB1	17	39163456	39232703	123	32	10164	-2.1729	0.98511
-	PPL	16	4872507	4972135	85	7	10164	-2.1748	0.98518
-	C22orf29	22	19836138	19889848	179	18	10164	-2.1773	0.98527
+	ABCA13	7	48136448	48657497	2025	89	10164	-2.1773	0.98527
-	PRPF8	17	1640629	1719882	177	20	10164	-2.1782	0.9853
+	LOC1053692	20	62192876	62245050	179	30	10164	-2.178	0.9853
-	SCN7A	2	166393468	166529258	440	32	10164	-2.1816	0.98543
+	U2AF2	19	55619050	55684716	123	14	10164	-2.1825	0.98546
-	RSPO4	20	948452	1037284	298	36	10164	-2.1835	0.9855
-	CDH19	18	66491187	66639138	415	42	10164	-2.1848	0.98555
+	DYRK2	12	67613732	67672664	125	23	10164	-2.185	0.98556
-	SPG21	15	64953021	65024946	230	22	10164	-2.1891	0.9857
+	TDG	12	103930815	103998878	271	30	10164	-2.1892	0.98571
+	REEP3	10	63486353	63635123	389	29	10164	-2.1924	0.98582
+	RHOB	2	20412071	20459445	92	16	10164	-2.1936	0.98587
-	LZTS1	8	20236165	20338981	368	61	10164	-2.1941	0.98589
+	ACKR1	1	159169013	159216500	101	25	10164	-2.1972	0.986
-	CD58	1	116504534	116606093	92	13	10164	-2.1978	0.98602
-	MEIS2	15	36881021	37136334	254	57	10164	-2.2033	0.98621
+	UFM1	13	38314771	38373007	21	12	10164	-2.2035	0.98622
+	LOC1053781	14	23263916	23317276	95	21	10164	-2.2038	0.98623
+	ERGIC1	5	172799220	172962685	433	61	10164	-2.2043	0.98625
+	ACER3	11	76825873	77036797	517	10	10164	-2.2071	0.98635
-	NHSL1	6	138412043	138727616	1012	77	10164	-2.2076	0.98636
+	SLC51B	15	65010370	65063396	152	28	10164	-2.2091	0.98641
-	HNRNPR	1	23294688	23379650	109	13	10164	-2.2091	0.98642
+	IFI35	17	42971725	43024459	70	10	10164	-2.2099	0.98644
-	CYP4A11	1	46919174	46976499	172	17	10164	-2.2123	0.98653
-	SPOCK1	5	136965298	137534329	1312	69	10164	-2.2137	0.98658
-	GSS	20	34918433	34990992	181	20	10164	-2.2151	0.98662
+	SS18L1	20	62108720	62192510	282	14	10164	-2.215	0.98662
-	WNT5A	3	55455715	55540261	144	18	10164	-2.2151	0.98663
+	R3HCC1	8	23253092	23306279	288	63	10164	-2.2172	0.9867
+	KIF11	10	92558068	92665395	369	22	10164	-2.2176	0.98671
+	TRAF2	9	136846933	136936621	180	8	10164	-2.2198	0.98678
+	IFIT2	10	89266949	89319276	105	22	10164	-2.2208	0.98682
-	LOC1019273	18	58660009	58706873	121	19	10164	-2.2207	0.98682
-	SLC5A4	22	32208421	32388008	978	55	10164	-2.2227	0.98688
+	SAP18	13	21105514	21159085	211	30	10164	-2.2232	0.9869
-	CLIC3	9	136984608	137031572	76	8	10164	-2.2237	0.98692
-	BRWD1	21	39174176	39348819	553	22	10164	-2.2241	0.98693
-	CYTL1	4	5004586	5054470	12	6	10164	-2.2266	0.98701
-	CLHC1	2	55162547	55267563	357	31	10164	-2.2267	0.98702
-	NANP	20	25602935	25659012	158	10	10164	-2.2295	0.98711
+	COQ9	16	57412425	57471275	139	21	10164	-2.2324	0.9872
+	COX14	12	50074635	50130457	92	10	10164	-2.2332	0.98723
-	IDE	10	92441684	92609095	436	28	10164	-2.2362	0.98733
-	GPR108	19	6719914	6772622	93	12	10164	-2.2366	0.98734
-	ZNF366	5	72429899	72542422	261	19	10164	-2.2369	0.98735
+	FANCM	14	45100933	45210890	207	24	10164	-2.2378	0.98738
+	FLI1	11	128651535	128823267	475	53	10164	-2.2442	0.98759
-	SHE	1	154459637	154537050	223	16	10164	-2.2442	0.98759
-	F13A1	6	6134078	6356013	571	78	10164	-2.2445	0.9876
+	CDC42EP2	11	65279818	65332429	102	30	10164	-2.2466	0.98767

STRAND	SYMBOL	CHR	START	STOP	NSNPS	NPARAM	N	ZSTAT	P
-	ZNF721	4	429984	534171	163	11	10164	-2.2472	0.98768
-	CHD8	14	21375194	21472298	28	3	10164	-2.2512	0.98781
+	C9orf142	9	136957418	137003976	123	11	10164	-2.2513	0.98782
-	WNT7A	3	13808217	13915121	285	44	10164	-2.2516	0.98783
+	CAPZA2	7	116827509	116929259	196	24	10164	-2.2529	0.98787
+	KCNQ4	1	40749012	40850452	334	56	10164	-2.2543	0.98791
+	CACNA1I	22	39522969	39699739	374	36	10164	-2.2544	0.98792
-	RWDD2B	21	28994778	29054371	107	18	10164	-2.2546	0.98792
+	PALD1	10	70423545	70578450	519	62	10164	-2.256	0.98796
-	SPRR2D	1	153029725	153076118	220	11	10164	-2.2613	0.98813
+	PPP2R4	9	129075945	129158946	176	8	10164	-2.2625	0.98817
-	PKD1L2	16	81090879	81255370	940	75	10164	-2.2625	0.98817
-	PARP10	8	143967152	144021467	57	2	10164	-2.2669	0.9883
+	LRIG2	1	113038170	113135202	136	14	10164	-2.2685	0.98835
+	NOMO3	16	16197532	16304811	4	2	10164	-2.2706	0.98841
+	TJP3	19	3673337	3760813	106	18	10164	-2.2727	0.98848
-	REG3A	2	79147006	79194754	174	23	10164	-2.2756	0.98857
-	SERPINE2	2	223965048	224074319	427	37	10164	-2.2757	0.98857
+	GULP1	2	188256669	188605926	778	30	10164	-2.2761	0.98858
-	PRKAG3	2	218812383	218866789	85	19	10164	-2.2783	0.98865
+	C10orf99	10	84138764	84195294	152	32	10164	-2.2799	0.98869
-	ARRDC3	5	91358724	91418359	117	16	10164	-2.2838	0.98881
+	TDRD10	1	154467219	154558147	249	17	10164	-2.2838	0.98881
-	MCM3	6	52254014	52319881	227	29	10164	-2.2851	0.98885
+	CXCL1	4	73834392	73881302	154	25	10164	-2.2856	0.98886
+	AGTRAP	1	11700474	11760771	81	20	10164	-2.2863	0.98888
-	NINL	20	25442697	25620531	446	19	10164	-2.2867	0.98889
-	JRK	8	142641501	142705132	279	20	10164	-2.2874	0.98891
-	LILRB2	19	54263812	54316178	134	12	10164	-2.2891	0.98896
-	GLIS3	9	3814127	4345506	2192	199	10164	-2.2895	0.98897
-	BFSP1	20	17483905	17604220	328	46	10164	-2.2916	0.98903
+	ALOX5AP	13	30678478	30774428	374	34	10164	-2.2922	0.98905
+	MRPL57	13	21141233	21189084	111	14	10164	-2.293	0.98908
+	GPRC5A	12	12856022	12923666	127	27	10164	-2.295	0.98913
+	PLCH2	1	2380782	2515530	288	59	10164	-2.2966	0.98918
-	LYNX1	8	142754334	142812848	219	18	10164	-2.2967	0.98918
-	COL25A1	4	108798725	109337752	1445	83	10164	-2.2984	0.98923
-	NRBP2	8	143820016	143875974	51	9	10164	-2.3021	0.98933
+	CD2	1	116719435	116779229	75	13	10164	-2.3029	0.98936
+	AQP6	12	49937837	49987139	131	37	10164	-2.3041	0.98939
+	PABPN1	14	23285188	23336185	94	19	10164	-2.3079	0.9895
-	NOP16	5	176373937	176423762	126	38	10164	-2.3117	0.9896
-	RYBP	3	72364593	72481623	180	30	10164	-2.3119	0.98961
-	ASAP3	1	23418563	23519567	178	16	10164	-2.3125	0.98963
+	OR5J2	11	56141618	56187556	135	16	10164	-2.3127	0.98963
-	PLIN2	9	19098388	19162606	186	33	10164	-2.3134	0.98965
+	CLIC4	1	24710269	24854324	172	13	10164	-2.3134	0.98965
-	ZNF395	8	28335585	28421460	143	15	10164	-2.318	0.98978
-	NDUFA11	19	5881276	5939013	94	6	10164	-2.3183	0.98978
-	FOXO1	13	40545664	40701660	448	26	10164	-2.3188	0.9898
-	SPRR2A	1	153046120	153092512	193	15	10164	-2.3188	0.9898
+	LOC1053713	16	83896236	83961460	338	29	10164	-2.3198	0.98982
+	HDAC1	1	32257107	32343628	73	15	10164	-2.3205	0.98984
-	ZFR2	19	3794024	3904029	248	26	10164	-2.3227	0.9899
-	LOC1005077	13	113946426	114009083	224	19	10164	-2.3229	0.98991
+	ABI3	17	49175227	49233225	168	22	10164	-2.328	0.99004
+	LOC1053739	2	111665614	111732334	53	6	10164	-2.329	0.99007
+	CNTN1	12	40657442	41082412	1377	55	10164	-2.3305	0.99011
-	TMOD4	1	151159986	151211304	54	12	10164	-2.3312	0.99013
-	ARL4C	2	234483041	234532053	103	25	10164	-2.3316	0.99014
+	NDST1	5	150450823	150568211	317	20	10164	-2.3376	0.9903
+	NXT1	20	23315736	23364771	175	22	10164	-2.3379	0.9903
+	CLTC	17	59584689	59706956	153	11	10164	-2.3387	0.99032

STRAND	SYMBOL	CHR	START	STOP	NSNPS	NPARAM	N	ZSTAT	P
+	COL15A1	9	98908348	99080792	497	50	10164	-2.339	0.99033
-	NEK1	4	169383270	169647629	360	7	10164	-2.3398	0.99035
+	CAPS	19	5878648	5926211	87	5	10164	-2.3402	0.99036
-	ING4	12	6640538	6698149	60	12	10164	-2.3402	0.99036
+	ITGB4	17	75686342	75767819	19	7	10164	-2.3421	0.99041
+	B4GALNT3	12	425377	571892	598	84	10164	-2.3445	0.99047
+	MFN1	3	179312692	179403226	149	14	10164	-2.3454	0.9905
-	HOMEZ	14	23263635	23321100	108	24	10164	-2.3454	0.9905
+	PNOC	8	28281991	28353355	214	26	10164	-2.3461	0.99052
-	AVP	20	3072555	3128521	45	13	10164	-2.348	0.99056
-	PPP1R3E	14	23285921	23337848	96	20	10164	-2.3482	0.99057
+	SMLR1	6	130792405	130847135	192	23	10164	-2.351	0.99064
+	VMAC	19	5869841	5920252	105	6	10164	-2.3517	0.99066
-	FAM107B	10	14508557	14809897	1267	113	10164	-2.3555	0.99075
+	DPYSL5	2	26813101	26960351	306	12	10164	-2.3597	0.99085
+	OPRM1	6	153975496	154256867	758	55	10164	-2.3605	0.99087
+	FASTKD2	2	206730388	206806189	253	19	10164	-2.3635	0.99095
-	HJURP	2	233826701	233889566	358	29	10164	-2.3634	0.99095
+	ADAMTS13	9	133379339	133469403	228	14	10164	-2.3639	0.99096
-	RTN4RL1	17	1924677	2060345	428	52	10164	-2.3649	0.99098
+	LOC1019279	18	76456970	76503918	68	13	10164	-2.3649	0.99098
-	MDH1B	2	206727763	206800549	243	18	10164	-2.3657	0.991
+	CUX2	12	110999024	111360554	510	63	10164	-2.368	0.99106
+	LOC1053730	22	43801042	43848860	124	19	10164	-2.3706	0.99112
-	SMURF1	7	99017435	99179120	214	26	10164	-2.3708	0.99113
+	C8orf48	8	13531843	13578288	297	30	10164	-2.3719	0.99115
+	OTOG	11	17512373	17655944	215	33	10164	-2.3737	0.99119
+	LOC1019293	10	42207499	42287067	17	4	10164	-2.3746	0.99122
-	ZNF888	19	52895893	52958452	120	16	10164	-2.3764	0.99126
+	OR2G3	1	247570586	247616515	119	13	10164	-2.3767	0.99127
-	FAM3D	3	58623943	58701844	230	22	10164	-2.3771	0.99127
-	USP34	2	61177455	61505714	664	21	10164	-2.3787	0.99131
+	CSE1L	20	49011246	49106960	245	17	10164	-2.3803	0.99135
+	AGO4	1	35772630	35867890	109	12	10164	-2.3813	0.99137
-	RNF150	4	140849807	141247869	912	82	10164	-2.3814	0.99138
+	RNF4	4	2434068	2525859	245	40	10164	-2.3826	0.9914
+	PARP14	3	122645763	122740840	344	27	10164	-2.3841	0.99144
-	CCNL2	1	1375711	1434338	131	6	10164	-2.3845	0.99145
-	OR6A2	11	6784525	6830908	150	13	10164	-2.3851	0.99146
-	SEC14L3	22	30437959	30507047	252	30	10164	-2.385	0.99146
+	HOXD3	2	176101921	176183102	272	50	10164	-2.3861	0.99149
+	MYH7B	20	34920835	35012437	243	24	10164	-2.3861	0.99149
+	DPP6	7	153852097	154904290	1736	142	10164	-2.3936	0.99166
-	FZD8	10	35628249	35676434	87	10	10164	-2.3944	0.99168
-	VPS72	1	151166300	151225213	74	12	10164	-2.4009	0.99182
+	DNAAF5	7	691701	796479	209	11	10164	-2.4044	0.9919
+	FAM91A1	8	123733439	123825452	184	16	10164	-2.4071	0.99196
+	ITGA3	17	50020976	50100485	306	39	10164	-2.4119	0.99206
+	DCP2	5	112941702	113032195	261	27	10164	-2.4115	0.99206
-	SCG2	2	223586940	223637499	169	20	10164	-2.4122	0.99207
+	MRPL54	19	3727667	3777565	81	12	10164	-2.414	0.99211
+	IKZF4	12	55972647	56048435	114	19	10164	-2.4143	0.99212
+	HLA-G	6	29791979	29841122	983	31	10164	-2.4199	0.99224
-	LYPD2	8	142740210	142787534	134	20	10164	-2.4216	0.99227
+	PDE9A	21	42618752	42785520	626	54	10164	-2.4227	0.9923
-	HERC4	10	67911899	68110348	366	27	10164	-2.4238	0.99232
+	LOC1053776	3	58136777	58191840	90	13	10164	-2.4263	0.99237
+	CCL13	17	34321452	34368610	128	13	10164	-2.4279	0.99241
+	PSCA	8	142635308	142692727	270	18	10164	-2.4327	0.99251
-	NLRP11	19	55775397	55871762	502	72	10164	-2.4333	0.99252
-	LTN1	21	28918144	29027956	242	30	10164	-2.4372	0.9926
-	SUPT4H1	17	58335175	58387238	92	18	10164	-2.44	0.99266
+	HIGD2A	5	176353783	176399750	116	32	10164	-2.4417	0.99269

STRAND	SYMBOL	CHR	START	STOP	NSNPS	NPARAM	N	ZSTAT	P
-	LOC1053728	22	18986513	19033725	156	17	10164	-2.444	0.99274
-	SHANK1	19	50649264	50754783	306	70	10164	-2.4474	0.99281
+	SH2B3	12	111370108	111461624	147	28	10164	-2.4528	0.99291
+	RPL27	17	42963429	43012959	59	7	10164	-2.4531	0.99292
+	C18orf65	18	76460521	76508088	62	12	10164	-2.4535	0.99293
+	TM4SF5	17	4736892	4793211	186	36	10164	-2.4541	0.99294
-	FAM60A	12	31270422	31361225	257	24	10164	-2.4548	0.99295
+	ZNF581	19	55608620	55655623	68	11	10164	-2.4563	0.99298
+	HOXD1	2	176153579	176200907	163	26	10164	-2.4595	0.99304
+	SNRNP48	6	7555199	7621967	175	25	10164	-2.4606	0.99306
+	SLC5A10	17	18915676	19030691	58	5	10164	-2.469	0.99323
+	IQGAP1	15	90353241	90512243	626	26	10164	-2.4702	0.99325
+	KIR2DS4	19	54797676	54858569	189	11	10164	-2.4759	0.99335
+	LRRC3	21	44420486	44468856	13	3	10164	-2.4798	0.99343
+	LOC1027237	22	42094871	42142085	95	6	10164	-2.4803	0.99344
+	NYAP2	2	225364710	225713653	585	32	10164	-2.4872	0.99356
-	KRTAP3-1	17	40998521	41044114	250	24	10164	-2.4876	0.99357
-	SULT1B1	4	69716120	69795712	224	22	10164	-2.4903	0.99362
+	WSB1	17	27259080	27323844	11	7	10164	-2.4943	0.99369
-	OR8K5	11	56149394	56195317	136	16	10164	-2.4946	0.99369
-	FYN	6	111650332	111908452	701	36	10164	-2.4962	0.99372
+	USP16	21	28989617	29064488	134	21	10164	-2.4984	0.99376
+	EHF	11	34586041	34673288	199	33	10164	-2.4987	0.99377
-	LONRF1	8	12711897	12790485	124	16	10164	-2.4994	0.99378
+	CAB39	2	230677842	230831075	261	23	10164	-2.5036	0.99385
+	CXCL6	4	73801556	73848760	72	11	10164	-2.5044	0.99387
-	MUC4	3	195736765	195846973	236	14	10164	-2.5089	0.99394
-	FAM229A	1	32351270	32397243	21	7	10164	-2.509	0.99395
+	IER2	19	13115468	13164908	36	3	10164	-2.5111	0.99398
-	LMO1	11	8214304	8303635	210	35	10164	-2.5126	0.99401
-	CD34	1	207871028	207946338	248	24	10164	-2.514	0.99403
+	FDX1	11	110394937	110474884	222	13	10164	-2.514	0.99403
-	CRBN	3	3139633	3214717	314	26	10164	-2.5138	0.99403
-	TCN1	11	59842808	59901568	40	15	10164	-2.515	0.99405
-	KRT26	17	40756238	40807159	129	22	10164	-2.5189	0.99411
+	TTC26	7	139098370	139201986	171	9	10164	-2.5232	0.99418
+	P2RX4	12	121174861	121244106	254	23	10164	-2.5249	0.99421
+	HS3ST4	16	25657026	26147688	1109	121	10164	-2.5249	0.99421
-	RELL1	4	37578059	37721377	323	31	10164	-2.5255	0.99422
+	PRPF39	14	45049099	45125601	147	16	10164	-2.5259	0.99423
+	WNT6	2	218824823	218884233	97	23	10164	-2.5279	0.99426
+	MTG2	20	62148025	62213568	291	25	10164	-2.528	0.99427
-	PON2	7	95394862	95470072	240	37	10164	-2.5298	0.99429
+	DHX40	17	59530525	59618352	86	10	10164	-2.5311	0.99431
+	HSD17B2	16	82000237	82108534	300	22	10164	-2.5384	0.99443
+	CYP4B1	1	46763995	46829349	189	24	10164	-2.5485	0.99459
-	GNB4	3	179386088	179486583	141	19	10164	-2.5554	0.9947
+	TMEM99	17	40784092	40846274	159	21	10164	-2.5567	0.99472
-	PAX6	11	31774792	31852961	92	11	10164	-2.5577	0.99473
+	OR10A5	11	6810683	6856636	206	12	10164	-2.5575	0.99473
-	RDM1	17	35908080	35965811	243	22	10164	-2.5634	0.99482
-	ZNF784	19	55610741	55659575	76	13	10164	-2.566	0.99486
-	MLLT1	19	6200381	6314948	36	2	10164	-2.5667	0.99487
-	FOXA1	14	37579552	37630120	67	11	10164	-2.5674	0.99488
-	PPP1R3D	20	59926832	59975297	1	1	10164	-2.5699	0.99491
-	CRAT	9	129084794	129145791	134	6	10164	-2.5725	0.99495
+	MAP3K7CL	21	29042471	29185889	446	25	10164	-2.576	0.995
-	MTSS1	8	124540770	124763507	815	86	10164	-2.5783	0.99504
-	KRT28	17	40782196	40834959	140	20	10164	-2.5805	0.99507
+	IFI44L	1	78585382	78656145	117	24	10164	-2.5825	0.9951
+	MTBP	8	120410398	120533635	234	9	10164	-2.5825	0.9951
+	C9orf139	9	136992464	137047767	94	12	10164	-2.5827	0.9951
-	RNPS1	16	2243116	2303412	49	5	10164	-2.5946	0.99526

STRAND	SYMBOL	CHR	START	STOP	NSNPS	NPARAM	N	ZSTAT	P
-	CPXM1	20	2784069	2835646	144	23	10164	-2.5945	0.99526
+	CLEC11A	19	50688348	50735724	93	24	10164	-2.5987	0.99532
+	ACTG2	2	73857966	73929653	76	6	10164	-2.6024	0.99537
-	MTFMT	15	64991512	65064639	211	32	10164	-2.604	0.99539
+	PLCXD2	3	111639676	111856447	609	47	10164	-2.6045	0.9954
-	KRTAP3-3	17	40983430	41029133	278	24	10164	-2.6089	0.99546
-	KRTAP3-2	17	40989193	41034886	283	24	10164	-2.6101	0.99547
+	BCL2L2-PAB	14	23271762	23336185	122	25	10164	-2.6133	0.99552
-	KLHL28	14	44914319	45013290	223	17	10164	-2.6163	0.99556
+	MSL1	17	40086988	40146794	52	15	10164	-2.6175	0.99557
+	RNASE3	14	20856403	20902348	81	10	10164	-2.6208	0.99561
+	OR2G2	1	247553360	247599313	136	18	10164	-2.623	0.99564
+	AGBL5	2	27016423	27080622	85	12	10164	-2.6297	0.99573
-	EIF4A3	17	80125214	80182183	204	22	10164	-2.6316	0.99575
-	ACRBP	12	6628076	6682414	68	10	10164	-2.6374	0.99582
-	LOC1019287	9	136242193	136301525	26	5	10164	-2.6476	0.99595
+	ZNF107	7	64631083	64721582	350	25	10164	-2.6496	0.99597
+	METAP2	12	95439046	95525839	316	21	10164	-2.6521	0.996
+	B3GALTL	13	31164975	31342276	439	31	10164	-2.6562	0.99605
-	OIP5	15	41299268	41367729	167	18	10164	-2.661	0.9961
-	NR1D1	17	40082784	40135725	52	15	10164	-2.6645	0.99614
+	FAM179B	14	44927213	45084431	319	16	10164	-2.666	0.99616
+	PPP1R3G	6	5050486	5097221	141	18	10164	-2.6659	0.99616
+	GREM1	15	32682974	32744669	223	37	10164	-2.6691	0.9962
+	HDGFRP2	19	4437196	4512220	139	13	10164	-2.669	0.9962
-	PUF60	8	143806344	143864386	46	8	10164	-2.6728	0.99624
+	CCDC86	11	60806956	60861088	166	14	10164	-2.6728	0.99624
+	KLHDC9	1	161063361	161110348	69	14	10164	-2.679	0.99631
+	RPS27A	2	55196903	55245853	159	30	10164	-2.6814	0.99633
+	SMIM6	17	75609822	75657977	13	4	10164	-2.6884	0.99641
+	FMO3	1	171055877	171127821	165	15	10164	-2.6903	0.99643
+	CNGA4	11	6199774	6255924	92	13	10164	-2.6936	0.99647
-	LOC1053738	2	190332271	190378960	129	11	10164	-2.6947	0.99648
+	LOC1053723	19	35924291	35974064	111	12	10164	-2.6982	0.99651
-	SLC2A6	9	133461094	133514154	106	16	10164	-2.699	0.99652
-	CCL3	17	36078256	36125160	153	13	10164	-2.7022	0.99656
+	SELV	19	39480113	39530686	121	18	10164	-2.7024	0.99656
+	CYP2F1	19	41079292	41138381	262	28	10164	-2.7037	0.99657
-	LPAR5	12	6608835	6671131	77	11	10164	-2.7031	0.99657
-	SLC5A12	11	26657019	26758356	344	40	10164	-2.7041	0.99658
+	SPINK4	9	33205198	33258567	114	14	10164	-2.708	0.99662
+	KHK	2	27051743	27110751	74	10	10164	-2.7104	0.99664
-	POU5F2	5	93730309	93776603	67	13	10164	-2.7101	0.99664
+	LAMP1	13	113262121	113333431	132	5	10164	-2.7122	0.99666
-	CXCL16	17	4723529	4774928	182	34	10164	-2.7213	0.99675
+	CYP2A13	19	41053451	41106195	156	16	10164	-2.7221	0.99676
+	PRSS2	7	142735943	142784564	90	3	10164	-2.7223	0.99676
+	LOC401040	2	239543396	239596088	119	27	10164	-2.733	0.99686
-	SCRIB	8	143780920	143850379	69	13	10164	-2.7466	0.99699
-	ITGB5	3	124751948	124922538	649	34	10164	-2.7484	0.99701
-	LSG1	3	194630788	194707477	272	23	10164	-2.7504	0.99702
-	C16orf95	16	87292798	87352420	174	20	10164	-2.7631	0.99714
-	PID1	2	229013973	229306341	818	72	10164	-2.7645	0.99715
+	RPS26	12	56006902	56054223	41	11	10164	-2.7809	0.99729
+	CCL4	17	36068827	36115621	157	11	10164	-2.7815	0.99729
+	RHBDD2	7	75843999	75898926	62	10	10164	-2.7822	0.9973
-	ZNF599	19	34748074	34808229	136	24	10164	-2.7838	0.99731
+	DPP10	2	114407322	115855752	3275	116	10164	-2.7845	0.99732
-	LOC1053766	13	110700644	110749084	117	13	10164	-2.7871	0.99734
+	FAM111B	11	59072185	59137415	139	18	10164	-2.7953	0.99741
+	SCNN1B	16	23267270	23391299	173	34	10164	-2.7963	0.99742
-	CS	12	56261699	56335391	93	5	10164	-2.7978	0.99743
-	UBXN6	19	4435006	4492794	111	11	10164	-2.8004	0.99745

STRAND	SYMBOL	CHR	START	STOP	NSNPS	NPARAM	N	ZSTAT	P
-	LYPD1	2	132634764	132706497	112	6	10164	-2.8007	0.99745
+	MAPRE3	2	26935371	27037219	143	9	10164	-2.8024	0.99746
-	TMEM184B	22	38206377	38308034	317	50	10164	-2.8017	0.99746
-	KCTD1	18	26444910	26692401	672	91	10164	-2.8057	0.99749
-	ADRA1D	20	4210631	4284012	288	33	10164	-2.8179	0.99758
-	CCDC151	19	11410605	11470782	50	6	10164	-2.8172	0.99758
+	CHEK1	11	125589920	125686259	314	21	10164	-2.8195	0.99759
+	LPO	17	58202737	58278536	101	16	10164	-2.82	0.9976
-	MRPL20	1	1391896	1442834	87	5	10164	-2.8362	0.99772
+	TMEM88B	1	1391128	1437787	87	5	10164	-2.8362	0.99772
+	TMEM239	20	2780874	2828062	132	19	10164	-2.8361	0.99772
+	CYP4X1	1	46926364	47060751	335	22	10164	-2.8384	0.99773
+	ORM2	9	114294789	114343256	151	30	10164	-2.8534	0.99784
+	HHEX	10	92654924	92705651	120	12	10164	-2.8664	0.99792
+	PRKCB	16	23800979	24230611	874	84	10164	-2.8656	0.99792
+	PHLDB2	3	111697480	111986517	1016	59	10164	-2.8685	0.99794
+	ATXN7	3	63829557	64013462	341	28	10164	-2.8848	0.99804
-	TMEM44	3	194577673	194668780	371	28	10164	-2.8909	0.99808
+	PSTPIP1	15	76960124	77047337	245	41	10164	-2.8947	0.9981
-	CCDC42	17	8719928	8779836	165	23	10164	-2.8953	0.99811
+	PPM1D	17	60565183	60676280	110	10	10164	-2.9042	0.99816
+	TRIM56	7	101050424	101101738	186	20	10164	-2.9068	0.99817
+	C20orf141	20	2779987	2825830	133	19	10164	-2.9096	0.99819
-	MKS1	17	58195436	58254605	77	13	10164	-2.9177	0.99824
+	INPP5A	10	132502784	132793480	631	24	10164	-2.9205	0.99825
+	EMILIN1	2	27043567	27096404	69	10	10164	-2.9309	0.99831
+	NACC1	19	13081847	13151147	26	3	10164	-2.9366	0.99834
+	SNN	16	11633433	11689159	180	26	10164	-2.9522	0.99842
-	H2AFY	5	135324381	135435147	62	3	10164	-2.9526	0.99842
-	CECR6	22	17106299	17156367	214	32	10164	-2.9529	0.99843
-	ZNF320	19	52866172	52932693	115	14	10164	-2.9592	0.99846
-	KRT27	17	40766808	40817534	130	23	10164	-2.9644	0.99848
+	CACFD1	9	133424965	133480787	102	14	10164	-2.9891	0.9986
+	ARHGAP10	4	147697063	148082779	1021	71	10164	-2.9959	0.99863
+	EDEM1	3	5152674	5229965	121	15	10164	-3.0237	0.99875
+	VWA1	1	1400523	1452882	79	5	10164	-3.0227	0.99875
-	E2F7	12	77011246	77100580	321	40	10164	-3.0358	0.9988
+	TOX4	14	21442176	21509177	41	3	10164	-3.0533	0.99887
-	RAB2B	14	21449020	21511973	42	3	10164	-3.0576	0.99888
-	LOC1053693	11	59250051	59319695	162	26	10164	-3.0566	0.99888
+	ATAD3C	1	1406301	1480158	72	5	10164	-3.0688	0.99893
-	ANKRD65	1	1408420	1456820	65	4	10164	-3.0695	0.99893
+	PF4V1	4	73818296	73864481	97	16	10164	-3.0727	0.99894
+	TRNT1	3	3091916	3162503	327	49	10164	-3.0758	0.99895
+	PDCL3	2	100527956	100586739	175	16	10164	-3.0842	0.99898
-	WDFY3	4	84659537	85001391	98	3	10164	-3.09	0.999
+	PRKCSH	19	11400257	11460968	48	6	10164	-3.0951	0.99902
+	LOC1001308	7	137918348	137967966	128	14	10164	-3.0992	0.99903
+	PFKL	21	44265034	44337381	165	20	10164	-3.1135	0.99908
-	TNFSF8	9	114883343	114965595	249	30	10164	-3.1179	0.99909
+	CDHR1	10	84159635	84229621	225	30	10164	-3.1391	0.99915
-	PON1	7	95288357	95359572	257	35	10164	-3.1548	0.9992
+	TLE6	19	2942415	3005184	101	24	10164	-3.1571	0.9992
-	OST4	2	27060472	27106699	57	7	10164	-3.165	0.99922
-	LYL1	19	13087188	13138160	19	3	10164	-3.1655	0.99923
+	SPATC1	8	143975993	144057114	54	2	10164	-3.1735	0.99925
+	TRIP10	19	6704682	6761526	78	16	10164	-3.1834	0.99927
-	TESPA1	12	54938303	55020161	386	27	10164	-3.2202	0.99936
+	LOC1053724	19	43652591	43710078	233	33	10164	-3.2252	0.99937
+	ATCAY	19	3845620	3938082	164	24	10164	-3.2336	0.99939
+	IL23A	12	56299158	56350410	61	3	10164	-3.254	0.99943
+	ORM1	9	114288023	114336479	168	35	10164	-3.2659	0.99945
-	PON3	7	95349872	95431375	272	27	10164	-3.2773	0.99948

STRAND	SYMBOL	CHR	START	STOP	NSNPS	NPARAM	N	ZSTAT	P
-	TRMT1	19	13094900	13152567	25	3	10164	-3.2894	0.9995
-	TMEM154	4	152616114	152715165	92	11	10164	-3.3071	0.99953
-	CNPY2	12	56300428	56351344	65	4	10164	-3.3114	0.99954
-	ADAMTS18	16	77272128	77470114	1168	70	10164	-3.3184	0.99955
-	THOC7	3	63823870	63899399	138	21	10164	-3.3612	0.99961
-	RPS14	5	150434229	150484756	163	18	10164	-3.4565	0.99973
+	CCL11	17	34250668	34298180	82	6	10164	-3.4978	0.99977
+	CCL7	17	34235216	34282242	68	5	10164	-3.6684	0.99988
-	FAM107A	3	58554112	58662610	329	30	10164	-3.7205	0.9999
+	EGFLAM	5	38223409	38475480	677	67	10164	-3.7305	0.9999
-	PAN2	12	56306223	56369053	71	5	10164	-3.7597	0.99991
-	FAM160A2	11	6201334	6269711	136	16	10164	-3.7683	0.99992
+	PABPC3	13	25061138	25108567	147	21	10164	-3.8665	0.99994
+	CCKBR	11	6224611	6282127	119	18	10164	-3.9759	0.99996
+	ARMC2	6	108813414	109008506	470	33	10164	-4.0397	0.99997

Supplementary Table 7. MAGMA results for iCOGS imputed by SG10K.

STRAND	SYMBOL	CHR	START	STOP	NSNPS	NPARAM	N	ZSTAT	P
+	ZNF532	18	58827600	58996477	423	35	10164	4.2902	8.926e-06
-	FILIP1	6	75284948	75528829	436	29	10164	3.7998	7.241e-05
-	FGFR2	10	121468330	121633656	643	87	10164	3.6733	0.00011973
+	TAB2	6	149182924	149421613	684	36	10164	3.6381	0.00013735
-	PRIMA1	14	93708298	93824035	342	56	10164	3.5717	0.00017737
-	DCAF12	9	34076383	34161773	214	15	10164	3.5537	0.00018989
-	CADM1	11	115163625	115539523	424	48	10164	3.5287	0.00020879
-	PKD1L3	16	71919538	72034978	428	32	10164	3.5221	0.00021405
+	LSP1	11	1817970	1902263	445	74	10164	3.5111	0.00022315
-	UBAP2	9	33911693	34083949	295	13	10164	3.5055	0.00022786
-	ADGRE3	19	14609117	14709918	83	9	10164	3.4965	0.0002357
+	IST1	16	71859408	71940639	187	21	10164	3.4949	0.0002371
-	TOX3	16	52426425	52582802	348	39	10164	3.465	0.00026508
-	ZNF821	16	71849680	71919867	150	18	10164	3.4583	0.00027175
-	SLC5A8	12	101146216	101245274	164	24	10164	3.4526	0.00027756
-	AP1G1	16	71719000	71844073	223	17	10164	3.4526	0.00027757
-	IMPA1	8	81646916	81721354	184	15	10164	3.442	0.00028868
+	ATXN1L	16	71810991	71867333	103	16	10164	3.4394	0.00029145
+	LAMA2	6	128848141	129526569	1568	92	10164	3.4025	0.00033384
-	PHLPP2	16	71634924	71759701	214	12	10164	3.3706	0.00037505
-	SLC10A5	8	81683656	81729972	151	11	10164	3.3605	0.00038902
-	COL12A1	6	75074326	75241202	318	20	10164	3.351	0.00040264
-	TMEM30A	6	75242922	75319916	111	11	10164	3.2991	0.0004849
+	CHMP4C	8	81697453	81769515	183	16	10164	3.268	0.00054153
-	ZFAND1	8	81691331	81756304	177	13	10164	3.2265	0.00062651
-	COX7A2	6	75227675	75289885	108	7	10164	3.2098	0.00066415
+	PAPPA	9	116118792	116412321	332	54	10164	3.1651	0.00077514
-	SBK2	19	55519336	55572174	89	17	10164	3.1474	0.0008236
-	SHCBP1L	1	182889865	182988418	139	9	10164	3.1416	0.00084003
-	SEL1L	14	81461547	81568861	223	19	10164	3.0912	0.00099677
+	PLEKHS1	10	113716262	113792433	280	28	10164	3.0901	0.0010005
+	MARVELD3	16	71591138	71651965	100	9	10164	3.0889	0.0010046
-	ABHD17B	9	71852452	71946233	25	2	10164	3.0888	0.0010049
-	TAT	16	71556851	71612095	106	10	10164	3.0714	0.0010654
+	CCDC170	6	151459040	151631193	812	65	10164	3.0475	0.0011536
-	CDH22	20	46163733	46343829	136	24	10164	3.0472	0.001155
+	C9orf85	9	71876321	71983459	37	3	10164	3.0135	0.0012913
-	GNPNAT1	14	52765193	52826668	127	18	10164	3.0063	0.0013223
-	SORCS1	10	106563663	107216126	1235	77	10164	2.9975	0.0013609
-	CDKN2A	9	21957752	22030043	194	26	10164	2.9973	0.0013621
+	NUBPL	14	31526385	31871224	822	14	10164	2.995	0.0013722
-	EEA1	12	92762509	92964331	289	17	10164	2.9916	0.0013877
+	MYO6	6	75714176	75929537	498	24	10164	2.9869	0.0014089
-	SUN2	22	38724714	38791019	99	16	10164	2.9781	0.0014501
-	CPM	12	68841175	68998240	423	51	10164	2.9629	0.0015239
+	CORT	1	10414719	10462003	126	16	10164	2.9592	0.0015423
+	HABP2	10	113515831	113599602	337	39	10164	2.958	0.0015482
-	CCDC122	13	43813931	43914690	372	21	10164	2.9429	0.0016257
-	CPXM2	10	123712291	123926984	340	33	10164	2.9385	0.0016492
-	ADAMTS10	19	8570240	8645753	18	3	10164	2.9381	0.0016512
+	SPON1	11	13927637	14278133	538	35	10164	2.9304	0.0016929
-	TOP2B	3	25587905	25699905	227	17	10164	2.91	0.0018069
+	TEX37	2	88489317	88539585	145	10	10164	2.9022	0.0018525
+	LACC1	13	43844284	43903933	213	20	10164	2.8882	0.0019374
+	STYX	14	52695165	52784989	156	18	10164	2.874	0.0020268
+	FEM1A	19	4756716	4805559	167	19	10164	2.8738	0.0020277
-	MEGF6	1	3477942	3659757	87	10	10164	2.8668	0.0020731
-	KRIT1	7	92188969	92281128	92	10	10164	2.8491	0.0021918
+	ANKIB1	7	92211216	92411770	197	16	10164	2.8469	0.0022076
-	SBSPON	8	73054543	73128272	220	23	10164	2.8301	0.0023268
+	CROCC	1	16860707	16982979	49	4	10164	2.8211	0.0023929
+	GAR1	4	109780510	109834737	192	17	10164	2.8186	0.0024113
+	GORAB	1	170497122	170563834	34	6	10164	2.8112	0.0024681
+	B3GNT5	3	183218226	183283391	28	3	10164	2.7938	0.002605
-	CFI	4	109721221	109837225	378	26	10164	2.7899	0.0026361
+	TSNAX	1	231493653	231576524	41	6	10164	2.7854	0.0026732
-	MIER3	5	56909602	56987130	276	34	10164	2.7816	0.0027046
+	SPATA8	15	96748407	96795615	98	12	10164	2.7804	0.002715
+	ISLR	15	74138746	74186871	53	19	10164	2.769	0.0028112
+	SENP6	6	75566509	75728285	357	18	10164	2.7596	0.0028936
+	XKR9	8	70634363	71075788	797	7	10164	2.7582	0.0029059
+	SETD9	5	56874259	56935532	186	23	10164	2.7568	0.0029187

STRAND	SYMBOL	CHR	START	STOP	NSNPS	NPARAM	N	ZSTAT	P
+	UBE2D2	5	139526166	139638434	7	2	10164	2.7455	0.003021
-	ZNF814	19	57859379	57924074	92	6	10164	2.734	0.0031288
-	JOSD1	22	38675543	38736418	116	9	10164	2.7277	0.0031892
+	GTPBP1	22	38669557	38748173	154	10	10164	2.7268	0.0031973
-	ATP2A3	17	3913869	3999464	64	9	10164	2.7209	0.0032553
-	RASSF2	20	4770023	4858645	352	47	10164	2.6969	0.003499
+	CHRNB3	8	42662419	42747407	65	2	10164	2.6959	0.0035099
+	ICOS	2	203901731	203971579	206	24	10164	2.6944	0.0035259
+	KLHL5	4	39009801	39152823	367	9	10164	2.6817	0.0036623
-	LOC105378948	1	1055635	1104326	48	3	10164	2.6773	0.0037112
+	APITD1-CORT	1	10395102	10462003	166	19	10164	2.6768	0.003716
-	RNF223	1	1061746	1109307	62	3	10164	2.6765	0.0037195
-	MTERF1	7	91862876	91915720	112	10	10164	2.676	0.0037252
+	ADGRL2	1	81271199	82002738	1993	154	10164	2.6752	0.0037348
-	MLLT3	9	20331665	20657543	446	39	10164	2.6693	0.0038008
+	LOC105369174	1	1026207	1076390	28	2	10164	2.6593	0.0039152
+	APITD1	1	10395102	10452815	148	16	10164	2.6546	0.0039704
+	SSC5D	19	55453497	55529099	293	33	10164	2.6504	0.0040193
-	CDKN2B	9	21992903	22044313	155	18	10164	2.646	0.0040727
-	HRK	12	116850420	116916427	123	19	10164	2.6447	0.0040885
+	IYD	6	150333861	150414629	399	24	10164	2.6444	0.0040923
+	GOLGA7B	10	97815238	97881578	123	14	10164	2.6419	0.0041222
+	CNDP2	18	74461265	74533454	99	5	10164	2.6414	0.0041283
-	LACTB2	8	70627266	70704212	148	6	10164	2.6414	0.0041286
+	RRH	4	109792994	109854707	188	16	10164	2.6385	0.0041639
-	PLA2G16	11	63564462	63651926	17	3	10164	2.63	0.0042696
-	ADAMTS6	5	65138736	65517027	445	28	10164	2.622	0.0043705
-	METTL21A	2	207570635	207660928	88	7	10164	2.6139	0.0044758
-	ZNF417	19	57895774	57951616	78	5	10164	2.6098	0.0045304
+	CORO2B	15	68524275	68737806	222	28	10164	2.6084	0.004549
+	RNFT2	12	116703291	116863631	368	29	10164	2.6068	0.00457
+	WIPF2	17	40184304	40294136	10	1	10164	2.5998	0.0046633
-	EGR3	8	22677659	22728302	74	9	10164	2.5968	0.0047052
+	RAPGEFL1	17	40142010	40205656	9	1	10164	2.5878	0.0048296
+	ATOH1	4	93793927	93839991	119	11	10164	2.579	0.0049545
-	DFFA	1	10450531	10507556	137	19	10164	2.5658	0.0051467
-	COX7B2	4	46724827	46944267	524	20	10164	2.5605	0.0052255
-	TAAR1	6	132634984	132681003	212	19	10164	2.5573	0.0052739
-	LAMA4	6	112097931	112289722	524	58	10164	2.5509	0.0053725
+	LOC105372267	19	8595645	8687993	75	10	10164	2.5423	0.0055066
-	PTCH2	1	44809844	44877944	163	12	10164	2.5388	0.0055615
-	NXPH2	2	138659157	138815241	196	17	10164	2.5331	0.0056533
+	KIRREL2	19	35816635	35877146	51	10	10164	2.5316	0.0056769
+	ZFP1	16	75097509	75182234	250	21	10164	2.5294	0.0057124
+	DAAM2	6	39757366	39914877	429	42	10164	2.5221	0.0058325
+	RNMT	18	13691647	13774556	115	10	10164	2.5113	0.0060145
+	CNDP1	18	74499457	74595025	124	18	10164	2.5086	0.0060603
-	MEGF9	9	120590918	120749487	165	8	10164	2.5085	0.0060628
-	CHRNA6	8	42742620	42803786	63	2	10164	2.4949	0.0062988
-	TAAR2	6	132589627	132659358	360	33	10164	2.4949	0.0062993
-	FAM227A	22	38568120	38691629	280	12	10164	2.4926	0.0063412
+	AGRN	1	985101	1066119	37	2	10164	2.4904	0.0063808
+	LBP	20	38311411	38387011	326	42	10164	2.4815	0.0065418
-	ZFAND4	10	45604567	45707962	243	8	10164	2.4679	0.0067961
-	TICAM1	19	4805924	4866742	171	21	10164	2.4656	0.0068393
-	NPHS1	19	35815372	35886993	58	11	10164	2.4602	0.0069438
-	TMEM156	4	38956016	39067669	142	9	10164	2.4534	0.0070751
-	FABP12	8	81514981	81625596	249	20	10164	2.4534	0.0070758
+	CLEC2L	7	139488928	139554985	30	6	10164	2.4525	0.0070925
+	UBLCP1	5	159228081	159296040	128	18	10164	2.4454	0.0072338
+	PHC1	12	8879684	8951467	155	12	10164	2.4449	0.0072455
-	TRPM1	15	30991061	31196273	504	34	10164	2.4434	0.0072751
-	LHFP	13	39332892	39638219	711	68	10164	2.4421	0.0073017
+	FAM21C	10	45692200	45802964	225	8	10164	2.4322	0.0075036
-	CCDC112	5	115257188	115331761	124	20	10164	2.431	0.0075281
-	HES4	1	988962	1036285	28	2	10164	2.4297	0.0075562
+	CBY1	22	38621653	38683850	185	8	10164	2.4268	0.0076171
-	USH2A	1	215612894	216458396	1500	89	10164	2.4263	0.0076261
+	ZHX2	8	122746349	122984515	411	74	10164	2.4256	0.0076407
-	C1orf159	1	1071818	1151356	137	6	10164	2.424	0.0076746
+	TOMM22	22	38646949	38694760	150	7	10164	2.42	0.0077613
-	ZNF418	19	57911884	57970408	94	8	10164	2.4192	0.0077777
-	AGAP4	10	45815592	45888877	21	3	10164	2.4181	0.0078011
+	OR8G1	11	124214676	124260611	319	11	10164	2.4178	0.0078081

STRAND	SYMBOL	CHR	START	STOP	NSNPS	NPARAM	N	ZSTAT	P
+	ATPIF1	1	28201091	28248105	92	3	10164	2.416	0.0078455
+	KIF2C	1	44704712	44777767	135	13	10164	2.4149	0.0078702
+	ST14	11	130124787	130220362	89	13	10164	2.4105	0.0079643
-	TCTEX1D4	1	44795908	44842442	109	11	10164	2.4101	0.0079732
-	LMOD3	3	69098672	69157595	212	16	10164	2.4047	0.0080918
-	MORN4	10	97604553	97669156	149	15	10164	2.4047	0.0080927
-	PPP3R2	9	101581615	101630001	170	21	10164	2.4032	0.0081251
+	A2ML1	12	8787554	8896918	178	29	10164	2.4013	0.0081674
+	TLL1	4	165838238	166115178	418	29	10164	2.3974	0.0082558
+	PTER	10	16401943	16523745	348	28	10164	2.3924	0.0083684
+	NTS	12	85839295	85892992	128	4	10164	2.3911	0.0084
-	PDCD2	6	170565572	170619774	63	3	10164	2.3893	0.0084408
-	MARCH8	10	45444585	45629906	580	14	10164	2.383	0.0085861
-	PTAFR	1	28137166	28228936	89	7	10164	2.3787	0.0086878
-	MYSM1	1	58644739	58735092	356	17	10164	2.3726	0.0088319
+	OR8G5	11	124206158	124333224	659	18	10164	2.3707	0.0088766
-	THAP1	8	42826674	42878331	1	1	10164	2.3677	0.0089484
-	RNF170	8	42839637	42932931	1	1	10164	2.3677	0.0089484
-	ELOVL7	5	60741789	60879283	125	13	10164	2.3675	0.0089554
-	EPHA2	1	16114337	16191087	115	13	10164	2.3627	0.0090722
-	EIF4G3	1	20796292	21212320	557	17	10164	2.3625	0.0090772
+	LOC389602	7	155927632	155976343	38	4	10164	2.3551	0.0092578
+	SMARCA5	4	143478463	143567489	16	4	10164	2.355	0.0092611
-	NFXL1	4	47837233	47949667	198	12	10164	2.3548	0.009267
-	VPS28	8	144413601	144463563	110	8	10164	2.3401	0.0096385
+	TMEM183A	1	202972383	203034848	156	24	10164	2.3353	0.0097631
+	RPS8	1	44740574	44788740	79	8	10164	2.3336	0.009808
-	PSMB1	6	170525116	170588329	93	3	10164	2.3335	0.0098107
-	NRXN1	2	49908505	51067536	3481	142	10164	2.3335	0.0098109
-	OR51G1	11	4913374	4959339	148	16	10164	2.3334	0.0098123
+	ACTN4	19	38612616	38740531	339	28	10164	2.3324	0.009841
+	LOC105378276	10	43877405	43924496	169	24	10164	2.3305	0.0098909
+	DHRS7B	17	21091275	21201972	104	9	10164	2.3286	0.00994
-	ZNF622	5	16441519	16505327	129	15	10164	2.3244	0.010052
-	RNF166	16	88686495	88741421	165	22	10164	2.3222	0.01011
+	TBP	6	170519333	170582870	91	3	10164	2.3152	0.0103
-	DPP9	19	4665226	4758843	68	4	10164	2.3131	0.01036
-	RORA	15	60478284	61264303	1801	199	10164	2.3128	0.010367
-	HRASLS2	11	63542759	63600089	33	5	10164	2.3124	0.010377
+	MYZAP	15	57556904	57695364	600	41	10164	2.3113	0.010409
-	PRODH2	19	35789990	35848299	115	25	10164	2.3111	0.010413
-	CARD17	11	105082469	105136465	142	7	10164	2.3099	0.010448
-	COX5A	15	74910275	74973154	157	28	10164	2.3086	0.010483
+	PFKP	10	3032474	3146805	393	38	10164	2.3083	0.010491
-	PGGT1B	5	115190241	115297887	164	21	10164	2.3058	0.01056
+	PSMC6	14	52672163	52737998	152	25	10164	2.3045	0.010596
-	RAB27A	15	55192966	55324828	272	19	10164	2.3044	0.010601
-	ST8SIA6	10	17305414	17490510	695	30	10164	2.3014	0.010684
-	PIGZ	3	196936343	197003871	12	3	10164	2.3007	0.010705
-	RASSF9	12	85790703	85871540	178	2	10164	2.2966	0.01082
+	ISG15	1	978467	1024540	16	1	10164	2.2921	0.010951
-	SLC39A4	8	144402414	144453034	83	5	10164	2.2908	0.010989
+	DHODH	16	71973744	72035417	243	28	10164	2.2874	0.011087
+	BPI	20	38269150	38347505	229	38	10164	2.2869	0.011099
-	CYB5R1	1	202951873	203002276	134	22	10164	2.2869	0.0111
+	CHRNA7	15	31995483	32182521	285	21	10164	2.2836	0.011198
-	TONSL	8	144418780	144479488	125	9	10164	2.2797	0.011313
+	GPR89B	1	147893393	148003521	31	5	10164	2.2785	0.01135
-	HERC1	15	63598618	63868949	561	19	10164	2.2779	0.011366
+	STON1	2	48495169	48608515	246	23	10164	2.2771	0.01139
+	BSND	1	54963944	55018792	78	14	10164	2.2681	0.011662
+	NOS3	7	150956056	151024599	112	17	10164	2.268	0.011664
-	RPP25	15	74945102	74992434	109	17	10164	2.2654	0.011743
-	LRIG3	12	58862149	58955538	322	32	10164	2.2646	0.01177
-	NUP153	6	17605035	17741834	91	5	10164	2.2581	0.01197
-	HP1BP3	1	20732677	20822310	115	9	10164	2.2557	0.012045
+	PLCG2	16	81744258	81972693	768	73	10164	2.2534	0.012118
+	ATRNL1	10	115058365	115958999	1613	26	10164	2.2515	0.012178
-	DBNDD1	16	89994865	90055131	259	27	10164	2.2514	0.012181
+	LOC644277	11	111410886	111466605	204	30	10164	2.2472	0.012313
+	GCOM1	15	57556904	57727557	654	49	10164	2.2441	0.012414
+	GBP6	1	89328877	89398160	158	16	10164	2.2436	0.012428
+	CLIP4	2	29062676	29193813	374	29	10164	2.2406	0.012528
-	RSL24D1	15	55171314	55232033	232	11	10164	2.2349	0.012712

STRAND	SYMBOL	CHR	START	STOP	NSNPS	NPARAM	N	ZSTAT	P
+	FAM120B	6	170255703	170417071	131	10	10164	2.2341	0.012738
-	RASGRF1	15	78949947	79125873	236	36	10164	2.2325	0.012791
+	TSLP	5	111035080	111088024	111	26	10164	2.2313	0.01283
-	CPSF1	8	144383231	144444400	70	4	10164	2.231	0.012841
+	ALDH5A1	6	24459969	24547207	470	36	10164	2.2296	0.012886
+	ATP1A1	1	116337185	116414774	21	3	10164	2.2262	0.012999
+	CDC40	6	110145421	110242232	163	8	10164	2.224	0.013073
-	FBXL17	5	107849033	108417098	1753	57	10164	2.2233	0.013097
+	RALGPS1	9	126879774	127233166	625	22	10164	2.2228	0.013114
-	FRG2B	10	133614796	133662886	67	8	10164	2.2205	0.013194
-	CD300LF	17	74684308	74748059	132	21	10164	2.2203	0.0132
-	LOC105374638	5	6329488	6404751	138	11	10164	2.2188	0.013249
-	OR51G2	11	4904719	4950663	152	21	10164	2.2186	0.013259
-	LOC105370579	14	77022368	77069940	154	27	10164	2.2182	0.013271
+	NELFCD	20	58946208	59005134	18	1	10164	2.2179	0.01328
-	PERM1	1	965199	1017117	5	1	10164	2.2177	0.013288
+	CIPC	14	77063235	77127287	179	29	10164	2.2137	0.013425
+	TPSD1	16	1221272	1268493	40	3	10164	2.2106	0.013532
-	RNF19A	8	100247059	100371218	290	22	10164	2.2049	0.013729
-	LOC105372192	18	74444602	74491948	87	12	10164	2.2049	0.013732
-	DNAJC8	1	28190278	28268031	147	4	10164	2.2025	0.013815
-	CMTM6	3	32471312	32537911	100	12	10164	2.2015	0.013851
+	KIFC2	8	144431149	144484116	107	8	10164	2.1994	0.013924
-	CCR10	17	42669402	42716827	43	7	10164	2.1982	0.013969
-	PPP2R2C	4	6310578	6598600	615	55	10164	2.1972	0.014002
+	TBRG1	11	124587599	124645926	114	15	10164	2.1957	0.014058
-	NGF	1	115275916	115373384	254	38	10164	2.1932	0.014145
-	CEP162	6	84114241	84262890	169	14	10164	2.1925	0.014171
-	TPSB2	16	1218336	1265184	54	4	10164	2.1922	0.014184
-	KRTAP5-11	11	71571855	71617875	1	1	10164	2.1921	0.014185
-	MED10	5	6361926	6413526	120	10	10164	2.1887	0.014309
+	C17orf112	17	52948991	52997658	118	13	10164	2.188	0.014336
-	DTNBP1	6	15512801	15698058	98	10	10164	2.1863	0.014398
-	DMC1	22	38499154	38605286	135	12	10164	2.184	0.014479
-	PRSS22	16	2842727	2893630	97	8	10164	2.1831	0.014513
-	TPSG1	16	1211488	1261384	68	4	10164	2.1826	0.014533
+	PI4K2A	10	97605686	97686434	198	16	10164	2.1773	0.014729
-	C2orf71	2	29051373	29109838	186	22	10164	2.1764	0.014763
-	BLNK	10	96181699	96306576	136	8	10164	2.1748	0.014823
+	TDGF1	3	46539555	46592463	238	17	10164	2.1744	0.014839
-	HS3ST6	16	1901463	1956422	131	11	10164	2.1743	0.014841
-	SNAI3	16	88667682	88721474	116	20	10164	2.1716	0.014942
+	BAIAP2	17	81000144	81127432	269	34	10164	2.166	0.015155
-	C1QTNF9B	13	23881099	23933503	130	14	10164	2.1658	0.015162
-	SERPINA6	14	94294248	94358351	195	28	10164	2.1641	0.015227
+	GRM1	6	145929769	146447598	465	16	10164	2.1612	0.015339
-	NEK10	3	27097336	27404460	996	33	10164	2.1609	0.015351
-	PSMD5	9	120804779	120878021	102	10	10164	2.1603	0.015377
+	PAK2	3	196704857	196842647	292	18	10164	2.1578	0.015472
+	FBXW10	17	18705223	18789349	3	1	10164	2.1567	0.015514
-	SYCE1	10	133543900	133603291	234	6	10164	2.1554	0.015564
+	CARD6	5	40806308	40865354	129	22	10164	2.155	0.015581
-	OR51A4	11	4936159	4982100	145	15	10164	2.1533	0.015646
+	CTU2	16	88671483	88725386	121	20	10164	2.1525	0.015678
+	SPDL1	5	169548634	169614778	120	8	10164	2.1523	0.015686
+	PEX14	1	10439946	10640758	458	36	10164	2.1472	0.015889
+	ADAD1	4	122343966	122439815	142	16	10164	2.1461	0.015933
+	FAM19A1	3	67956592	68555621	1191	58	10164	2.146	0.015936
+	ACAN	15	88768442	88885354	14	6	10164	2.1459	0.01594
+	IFRD1	7	112388144	112487203	216	18	10164	2.144	0.016018
+	BTBD19	1	44772457	44824199	96	11	10164	2.14	0.016176
-	FAM210A	18	13653347	13761592	146	10	10164	2.1395	0.016199
-	DEFA3	8	7005869	7053339	184	15	10164	2.1388	0.016225
+	BCL2L11	2	111085618	111178445	203	26	10164	2.1383	0.016248
+	TPSAB1	16	1205677	1252554	75	5	10164	2.1382	0.016249
+	SCPEP1	17	56943107	57016768	84	7	10164	2.1369	0.016302
+	KAAG1	6	24321903	24368284	88	10	10164	2.1368	0.016306
+	CDH6	5	31158655	31339146	451	56	10164	2.1355	0.016362
+	PVR	19	44608798	44676161	107	10	10164	2.1346	0.016397
+	SRGN	10	69053072	69114811	98	10	10164	2.1332	0.016453
+	MMP26	11	4953194	5002429	168	24	10164	2.1328	0.016469
+	C18orf8	18	23468470	23541807	149	18	10164	2.132	0.016504
-	C11orf95	11	63749892	63803641	5	1	10164	2.1274	0.016694
-	LOC105378161	6	170451022	170498114	128	10	10164	2.1257	0.016764

STRAND	SYMBOL	CHR	START	STOP	NSNPS	NPARAM	N	ZSTAT	P
-	ZZEF1	17	3994445	4178020	459	17	10164	2.1244	0.016818
+	FBXL22	15	63559830	63612428	109	12	10164	2.123	0.016877
+	LOC100996813	17	19542660	19643825	39	5	10164	2.1228	0.016885
+	SLC47A1	17	19498854	19589033	43	5	10164	2.1211	0.016956
-	PTHLH	12	27948084	28007731	236	29	10164	2.1206	0.016977
+	GJA8	1	147867820	147925287	89	13	10164	2.1177	0.017101
+	LDLRAD3	11	35908981	36242136	1281	131	10164	2.1172	0.01712
-	LRRC2	3	46505385	46615060	428	25	10164	2.1172	0.017123
+	TRMU	22	46300401	46367340	85	3	10164	2.1152	0.017207
+	TMEM253	14	21063937	21113724	29	3	10164	2.1121	0.017338
+	LMX1B	9	126579443	126711032	367	34	10164	2.1096	0.017445
+	C1orf228	1	44639694	44735591	91	17	10164	2.1053	0.017631
+	PANX3	11	124576557	124630356	120	16	10164	2.1051	0.01764
+	GAS8	16	89984629	90054971	309	27	10164	2.1046	0.017663
-	TRIM25	17	56877909	56949048	39	6	10164	2.1043	0.017676
-	IL2	4	122441470	122491495	95	14	10164	2.1017	0.017791
+	TMCC2	1	205192910	205283343	247	22	10164	2.101	0.017819
-	BEST4	1	44772045	44827828	103	11	10164	2.0995	0.017887
+	CPS1	2	210442682	210689107	453	25	10164	2.0982	0.017944
+	DNASE2B	1	84363532	84425895	74	9	10164	2.0981	0.017949
-	GABRA4	4	46908900	47029407	360	27	10164	2.0976	0.017972
-	LOC729159	16	60348455	60394763	99	17	10164	2.0928	0.018185
+	SOD1	21	31624622	31678931	67	8	10164	2.0902	0.018302
-	FAM69C	18	74425728	74499715	147	23	10164	2.0893	0.018341
+	HOXC13	12	53903792	53956544	131	19	10164	2.0883	0.018383
-	KRT222	17	40645620	40700164	58	4	10164	2.0883	0.018386
-	DUSP8	11	1544051	1607831	202	17	10164	2.0849	0.018539
+	STON1-GTF2A1L	2	48494925	48786517	770	60	10164	2.0848	0.018543
+	SND1	7	127617119	128102607	806	32	10164	2.0835	0.018605
-	HMGCLL1	6	55424373	55714668	1021	44	10164	2.0821	0.018669
-	GPLD1	6	24415832	24530043	705	38	10164	2.0818	0.018682
-	IKZF2	2	212989684	213189266	543	38	10164	2.0783	0.018843
-	DEFA5	8	7045300	7091739	101	11	10164	2.0782	0.018844
-	LANCL1	2	210421249	210512618	226	16	10164	2.0781	0.018852
+	OPTC	1	203459143	203518949	145	17	10164	2.0752	0.018986
-	PRB1	12	11341823	11390590	43	5	10164	2.0714	0.019163
-	SMARCE1	17	40617720	40682851	80	6	10164	2.0695	0.019252
-	PHF19	9	120845651	120929896	196	16	10164	2.0669	0.01937
+	OR6C4	12	55516227	55562156	134	4	10164	2.0641	0.019506
-	AHNAK	11	62423542	62581860	157	31	10164	2.0638	0.01952
+	ADCY7	16	50209749	50328135	133	18	10164	2.0632	0.019548
+	HEPHL1	11	93986212	94124208	340	27	10164	2.0631	0.01955
-	CANT1	17	78981716	79044817	187	25	10164	2.062	0.019603
-	KCNH2	7	150934956	151013314	129	18	10164	2.0617	0.019619
-	OR2B3	6	29076208	29122313	184	28	10164	2.0604	0.01968
-	TFCP2	12	51083756	51208143	135	9	10164	2.0596	0.019718
+	OR2J2	6	29138534	29184574	229	21	10164	2.0591	0.019741
-	SH2D5	1	20709731	20767640	125	14	10164	2.0591	0.019744
+	PLK3	1	44764952	44815995	86	10	10164	2.0571	0.019836
-	DAPK3	19	3948453	4006123	32	2	10164	2.0564	0.019873
+	CXXC5	5	139613716	139693882	49	5	10164	2.0562	0.019881
+	ESR1	6	151655496	152113274	2174	136	10164	2.0561	0.019886
-	ACTA2	10	88925074	89026390	302	26	10164	2.0558	0.019903
-	METTL24	6	110235946	110393272	207	17	10164	2.0553	0.019923
+	OR2J3	6	29076810	29122884	185	27	10164	2.0538	0.019998
+	FOXF2	6	1354834	1405597	66	15	10164	2.0533	0.020023
+	USP5	12	6817118	6876632	22	6	10164	2.0531	0.02003
-	TEC	4	48125783	48304797	594	30	10164	2.0527	0.02005
+	LOC105375003	6	29073630	29124417	201	28	10164	2.0527	0.020052
+	HOGA1	10	97549345	97622802	167	22	10164	2.0471	0.020325
+	ARL6IP5	3	69049939	69116088	157	16	10164	2.0458	0.020386
-	DNAJB3	2	233732750	233779015	178	25	10164	2.0435	0.020503
+	CYB5D2	17	4108168	4167701	149	12	10164	2.0423	0.020559
+	LOC105378423	10	89754148	89814646	194	12	10164	2.041	0.020625
-	NPC1	18	23496184	23621617	225	23	10164	2.0404	0.020657
+	LUC7L3	17	50684565	50762711	3	1	10164	2.0403	0.020662
+	IGSF23	19	44578590	44646781	125	14	10164	2.0395	0.020701
-	C5orf67	5	56501394	56641232	474	68	10164	2.0389	0.020732
+	LRRC8B	1	89489144	89611539	291	19	10164	2.0387	0.020741
+	GLP2R	17	9790543	9902104	81	2	10164	2.0344	0.020954
+	SIGLECL1	19	51216026	51279330	182	26	10164	2.032	0.021078
+	COASY	17	42527074	42576281	81	7	10164	2.0288	0.021238
-	LOC102723456	17	36851674	36898150	82	13	10164	2.0262	0.02137
+	MLX	17	42532060	42583203	78	7	10164	2.0248	0.021443

STRAND	SYMBOL	CHR	START	STOP	NSNPS	NPARAM	N	ZSTAT	P
-	TPTE2	13	19412877	19596574	295	8	10164	2.0245	0.021457
-	TXK	4	48056393	48169256	231	26	10164	2.0235	0.021511
+	FLYWCH2	16	2847804	2909382	153	9	10164	2.0231	0.021529
+	UBE2R2	9	33782164	33930403	128	6	10164	2.0224	0.021567
-	ZNF34	8	144762224	144822359	84	5	10164	2.021	0.021641
-	ATL2	2	38284094	38413139	347	11	10164	2.0204	0.021669
+	LSMEM1	7	112445238	112501062	140	10	10164	2.0193	0.02173
-	PITX1	5	135017734	135069274	74	13	10164	2.0145	0.021978
+	RAB44	6	36662827	36743183	375	46	10164	2.0142	0.021992
-	MCF2L2	3	183168004	183463619	333	22	10164	2.0136	0.022026
+	SARS	1	109178893	109248182	116	13	10164	2.0135	0.022031
-	PLEKHH3	17	42657914	42712030	59	9	10164	2.0132	0.022048
+	MAFK	7	1495732	1553043	124	4	10164	2.0114	0.022143
-	EBF1	5	158685908	159134780	971	76	10164	2.0113	0.022147
+	CELF2	10	10969641	11346675	824	79	10164	2.0109	0.022167
+	LOC101928868	6	154475097	154532300	177	23	10164	2.0095	0.02224
-	LOC101927562	11	1544607	1591457	135	10	10164	2.0089	0.022275
-	KLF7	2	207064137	207208851	322	30	10164	2.0088	0.022277
-	SHPRH	6	145852721	145999285	177	5	10164	2.0085	0.022296
+	LOC105378578	10	85113131	85166883	179	7	10164	2.0083	0.022305
-	FNBP4	11	47706517	47802460	65	2	10164	2.0075	0.022349
+	CELSR2	1	109214715	109285756	95	12	10164	2.0065	0.0224
-	LOC101927579	15	30529463	30660778	43	1	10164	2.0056	0.022449
-	EMB	5	50386192	50478307	125	1	10164	2.0055	0.022454
-	UBA3	3	69044730	69115373	161	16	10164	2.0044	0.022514
-	SCTR	2	119429843	119559452	271	26	10164	2.0043	0.022518
+	GOLGA8H	15	30510870	30627827	24	1	10164	2.0035	0.02256
+	CYP2E1	10	133492363	133549116	154	9	10164	2.0029	0.022592
+	ARHGAP11B	15	30591131	30648810	36	1	10164	2.0027	0.022607
+	AMBN	4	70557258	70617288	117	13	10164	2.0013	0.022681
+	NUS1	6	117640454	117720727	84	13	10164	2.0001	0.022746
+	XIRP2	2	166853487	167269753	1597	53	10164	1.9967	0.02293
+	PRKCI	3	170187432	170315982	524	29	10164	1.996	0.022965
-	POU6F1	12	51176936	51252693	14	3	10164	1.9951	0.023017
-	STON2	14	81250650	81462404	520	40	10164	1.9932	0.023122
+	HEXDC	17	82383376	82452640	124	13	10164	1.9917	0.023201
+	UBE2F	2	237931945	238052782	270	33	10164	1.9916	0.023209
-	C21orf58	21	46290430	46358899	224	23	10164	1.9907	0.023259
+	PCNT	21	46289100	46455769	588	24	10164	1.9904	0.023276
-	ABCB4	7	87392044	87512523	269	23	10164	1.9898	0.023308
-	GFRA2	8	21682014	21824294	239	36	10164	1.9884	0.023384
-	COL24A1	1	85719233	86191791	1856	72	10164	1.9883	0.023387
-	ARL5A	2	151790966	151863495	45	7	10164	1.9881	0.0234
+	PCSK5	9	75855601	76372339	859	89	10164	1.988	0.023403
-	HSD17B3	9	96225306	96348983	349	32	10164	1.9871	0.023456
+	MAN2A1	5	109654366	109879625	464	13	10164	1.9865	0.023487
-	APOBEC1	12	7639400	7705599	155	22	10164	1.9864	0.023496
+	HSD17B1	17	42516943	42565214	78	10	10164	1.9852	0.023559
-	RPL8	8	144779767	144827420	80	6	10164	1.9846	0.023592
-	CAPN12	19	38720192	38779474	234	17	10164	1.9844	0.023608
+	TMEM177	2	119644167	119692118	89	7	10164	1.9841	0.023624
-	LOC105370940	15	32018535	32065596	130	9	10164	1.9834	0.023663
+	TRIM16L	17	18662998	18746118	72	6	10164	1.9827	0.023698
-	ZNF286B	17	18648429	18717259	72	6	10164	1.9827	0.023698
-	FBXW2	9	120746976	120828462	83	7	10164	1.9806	0.023816
+	JARID2	6	15210975	15532042	420	29	10164	1.9795	0.023881
-	PLA2G2E	1	19910307	19958617	140	18	10164	1.9792	0.023896
-	NUP160	11	47768113	47883505	81	2	10164	1.9781	0.023957
+	OR51L1	11	4963983	5009930	161	26	10164	1.9753	0.024118
+	FAS	10	88934801	89027059	282	27	10164	1.9748	0.024146
-	ST3GAL5	2	85829147	85924034	243	13	10164	1.9747	0.024153
-	LCTL	15	66537468	66600979	242	36	10164	1.9745	0.024165
-	DEFB136	8	11963937	12009599	19	3	10164	1.9737	0.02421
+	C11orf70	11	102012411	102094560	157	15	10164	1.971	0.024365
-	INTS1	7	1460277	1539382	186	4	10164	1.9707	0.02438
-	AP3M1	10	74110257	74186068	69	3	10164	1.9693	0.024462
-	IL12B	5	159304783	159365473	121	19	10164	1.9688	0.024488
+	GPX7	1	52567371	52619051	61	4	10164	1.9669	0.024598
+	ZNF517	8	144763876	144819144	75	5	10164	1.9664	0.024625
+	OR8G2	11	124189639	124235663	245	16	10164	1.9661	0.024643
+	BIRC2	11	102312182	102388670	179	15	10164	1.9638	0.024774
+	KIAA1109	4	122117333	122372759	404	17	10164	1.9637	0.024781
-	SIAE	11	124625789	124711303	146	12	10164	1.9637	0.024782
+	ACSM2A	16	20416526	20497669	101	3	10164	1.9632	0.024811

STRAND	SYMBOL	CHR	START	STOP	NSNPS	NPARAM	N	ZSTAT	P
+	PANO1	11	762511	809185	49	5	10164	1.9624	0.024858
+	C10orf2	10	100952378	101004403	73	6	10164	1.9622	0.024871
+	AGTR1	3	148662871	148753003	126	12	10164	1.9614	0.024914
+	LOC105375039	6	36643946	36691307	246	26	10164	1.9614	0.024917
+	C16orf78	16	49338808	49409431	108	7	10164	1.9587	0.025076
-	DSTYK	1	205132503	205246599	276	18	10164	1.9585	0.025088
+	FN3KRP	17	82681706	82738017	51	6	10164	1.9578	0.025127
-	DCDC2	6	24161755	24418292	674	37	10164	1.9569	0.025182
+	PLEKHO1	1	150114434	150170065	1	1	10164	1.9565	0.025204
+	GMCL1	2	69794642	69891386	267	21	10164	1.9563	0.025213
+	CDKN1A	6	36641460	36697339	266	29	10164	1.9562	0.025222
-	MRPL43	10	100967822	101022515	97	10	10164	1.9556	0.025257
+	UGT1A3	2	233694127	233783299	373	27	10164	1.9551	0.025283
-	OR8D1	11	124299840	124345766	159	14	10164	1.9539	0.025354
-	PDDC1	11	757217	812501	52	5	10164	1.9524	0.025448
-	ZNF692	1	248840004	248895798	47	6	10164	1.9483	0.025692
-	LOC105376791	17	82691905	82740329	62	6	10164	1.9482	0.025695
-	SLC46A2	9	112868920	112925942	219	28	10164	1.9479	0.025713
+	OGDH	7	44571522	44719070	218	14	10164	1.9475	0.025735
+	MANEA	6	95542497	95619452	203	11	10164	1.9471	0.025761
-	ZNF219	14	21080046	21139704	62	8	10164	1.9461	0.025822
+	LOC105370748	15	30764309	30810017	117	4	10164	1.9454	0.025866
-	PSMC3IP	17	42562310	42612831	66	5	10164	1.9439	0.025952
+	LOC105369190	11	744359	790898	32	4	10164	1.9435	0.025977
+	OR2AP1	12	55539415	55585344	212	10	10164	1.9428	0.02602
-	YIPF5	5	144148159	144205714	24	3	10164	1.9414	0.026105
-	VIPR2	7	159018175	159179957	373	17	10164	1.9411	0.026125
+	UGT1A1	2	233725273	233783299	231	28	10164	1.9408	0.02614
-	EIF2B3	1	44840522	45021722	483	15	10164	1.9394	0.026225
+	PLEKHG7	12	92701489	92782745	121	10	10164	1.9389	0.026259
-	WASF1	6	110089817	110224874	252	8	10164	1.9382	0.026302
-	KIAA0226L	13	46332000	46425042	180	13	10164	1.9339	0.026565
+	SESN2	1	28224452	28292491	127	5	10164	1.9324	0.026653
+	PIM3	22	49925495	49974072	88	8	10164	1.9322	0.026666
-	CCL27	9	34651883	34697692	101	10	10164	1.9313	0.026726
+	UGT1A4	2	233683792	233783299	423	28	10164	1.9296	0.026831
-	APTX	9	32962606	33060138	366	13	10164	1.9289	0.026872
+	RAP2A	13	97399221	97477998	43	10	10164	1.9253	0.027097
+	ZNF578	19	52418506	52526878	186	18	10164	1.9243	0.027156
+	DNAJA1	9	32990211	33049064	266	11	10164	1.9238	0.027193
+	SPA17	11	124638844	124707545	114	10	10164	1.9232	0.027228
-	RPL37	5	40821328	40870285	113	18	10164	1.9227	0.027261
-	HLA-B	6	31343868	31392212	1783	55	10164	1.9226	0.027266
+	TMEM91	19	41328966	41394083	64	4	10164	1.9192	0.027481
-	FUT10	8	33298611	33508478	421	23	10164	1.9179	0.027561
-	GRIP2	3	14479111	14637996	513	64	10164	1.9174	0.027596
-	PRKAA1	5	40749379	40833195	168	20	10164	1.9165	0.027652
+	SLC6A17	1	110115510	110212202	155	13	10164	1.9164	0.027659
+	C11orf63	11	122846639	122969722	388	45	10164	1.9158	0.027696
+	C20orf144	20	33627286	33673915	48	9	10164	1.9155	0.027715
-	B9D2	19	41344417	41399540	69	3	10164	1.9154	0.027721
-	RSRC2	12	122494643	122562013	6	1	10164	1.9143	0.02779
-	CADPS	3	62388346	62910389	1467	87	10164	1.9139	0.027814
+	LZTS2	10	100962107	101017836	91	8	10164	1.9126	0.027897
-	LRRC4	7	128017071	128067107	75	15	10164	1.9122	0.027926
-	STK39	2	167944020	168282595	941	41	10164	1.9117	0.027959
-	ATXN1	6	16289112	16796490	1344	177	10164	1.9108	0.028012
+	MICA	6	31364784	31425315	1596	36	10164	1.9096	0.028093
+	PGBD2	1	248837850	248929146	52	6	10164	1.9093	0.028109
-	LOC101927216	13	80031700	80110306	194	24	10164	1.9071	0.028251
+	GPR26	10	123631355	123707399	287	38	10164	1.9066	0.028286
-	OR13J1	9	35859463	35905401	118	16	10164	1.9046	0.028416
+	OR6C68	12	55457378	55503316	244	12	10164	1.9039	0.028463
-	PPP2R2B	5	146579504	147116520	980	61	10164	1.9028	0.028533
+	C6orf136	6	30612039	30663210	220	42	10164	1.9023	0.028568
+	CNTNAP1	17	42647614	42709993	79	8	10164	1.8993	0.02876
-	LOC105376341	10	941571	991386	98	13	10164	1.8983	0.028828
-	IARS	9	92200207	92328756	76	4	10164	1.8978	0.028861
-	VNN3	6	132712787	132769765	18	7	10164	1.8975	0.028879
+	SPAG1	8	100123003	100251904	284	18	10164	1.8974	0.028889
-	HS6ST1	2	128255480	128353597	92	12	10164	1.8967	0.028935
+	RAB37	17	74636117	74757335	282	48	10164	1.8958	0.028992
+	PLCB4	20	9033710	9490816	555	51	10164	1.8941	0.029109
+	MRPS26	20	3011029	3058250	109	14	10164	1.8918	0.02926

STRAND	SYMBOL	CHR	START	STOP	NSNPS	NPARAM	N	ZSTAT	P
+	SEMA4G	10	100934482	100995616	74	8	10164	1.8912	0.029301
-	VAX1	10	117118521	117173301	49	4	10164	1.8885	0.02948
-	ATOH7	10	68220595	68267113	110	8	10164	1.8875	0.029545
+	UGGT1	2	128056180	128205677	363	10	10164	1.8862	0.029633
+	ADAMTS14	10	70637539	70772441	635	55	10164	1.886	0.029649
+	GINM1	6	149531392	149600931	66	6	10164	1.8846	0.029739
-	SERPINA2	14	94354318	94401702	135	13	10164	1.884	0.029786
+	MOBP	3	39432573	39539497	264	15	10164	1.8814	0.029956
+	ZSCAN2	15	84565986	84633718	40	6	10164	1.8783	0.030168
-	ADIPOR1	1	202930825	202993572	192	28	10164	1.8777	0.030212
+	TMEM61	1	54945595	55002293	132	10	10164	1.8772	0.030248
-	OGFOD3	17	82379210	82453637	142	12	10164	1.8771	0.030249
-	ACSBG1	15	78160845	78269707	377	47	10164	1.876	0.030331
-	TGFB1	19	41320531	41388933	73	6	10164	1.8754	0.030372
+	ZNF507	19	32310608	32397667	16	2	10164	1.8743	0.030444
-	LDHD	16	75101860	75151772	166	20	10164	1.8739	0.03047
+	UGT1A5	2	233677992	233783299	458	29	10164	1.8735	0.030502
-	MTMR10	15	30909764	31026604	201	8	10164	1.8726	0.030561
-	ENPP2	8	119547077	119708404	366	40	10164	1.8723	0.030581
-	LOC105371907	17	77247737	77317699	191	19	10164	1.8711	0.030663
+	TRIM64	11	89931087	89984072	1	1	10164	1.8705	0.030707
-	EPM2A	6	145490777	145771303	676	12	10164	1.8703	0.030719
+	C1orf54	1	150234004	150290924	70	9	10164	1.8702	0.030728
-	RAB40B	17	82644959	82733722	92	15	10164	1.8699	0.030748
+	GADD45A	1	67650177	67698338	142	18	10164	1.8692	0.0308
+	FAN1	15	30868852	30953108	97	5	10164	1.8687	0.03083
+	LOC105376149	9	92586621	92641713	17	2	10164	1.8681	0.030872
-	IRF2BPL	14	77014543	77063699	171	29	10164	1.8678	0.030892
+	SENP5	3	196832856	196944714	102	4	10164	1.8673	0.030928
-	MAP3K12	12	53469669	53535136	62	11	10164	1.867	0.03095
+	VPS37D	7	73632844	73682110	49	6	10164	1.8636	0.031187
+	STK32A	5	147199963	147411533	496	34	10164	1.8633	0.031212
+	CD19	16	28896916	28949347	1	1	10164	1.8632	0.031219
+	HSPB9	17	42087738	42133353	59	4	10164	1.8601	0.031434
-	SLC39A8	4	102241041	102380498	420	31	10164	1.8596	0.031474
-	TRIM27	6	28893002	28958991	328	30	10164	1.8588	0.031529
-	C8orf37	8	95234913	95305337	241	19	10164	1.8586	0.031541
+	JDP2	14	75392806	75482701	209	33	10164	1.8571	0.031649
-	LOC105377805	13	113731875	113787839	103	7	10164	1.8562	0.031715
+	CA14	1	150222159	150275078	56	6	10164	1.8559	0.031735
-	ELMO2	20	46356049	46442051	87	6	10164	1.8548	0.031812
+	GRM7	3	6826097	7751531	2044	149	10164	1.8545	0.031833
-	COIL	17	56928199	56996050	77	6	10164	1.8535	0.031905
-	PRKG2	4	81077370	81251743	410	25	10164	1.8515	0.032051
+	SRGAP1	12	63809512	64157863	362	32	10164	1.8504	0.03213
-	FAM134C	17	42569508	42644427	102	5	10164	1.8482	0.032285
+	LOC100506388	17	296205	343488	17	4	10164	1.8475	0.032334
-	ERO1L	14	52629915	52730931	232	34	10164	1.847	0.032373
+	TUBG1	17	42574340	42625238	62	4	10164	1.8469	0.032378
-	GPR84	12	54340784	54400253	77	12	10164	1.8468	0.03239
+	NEFM	8	24878761	24929093	45	3	10164	1.8466	0.0324
-	SFXN4	10	119130913	119200709	212	22	10164	1.8463	0.032426
+	ATAT1	6	30591836	30656823	310	35	10164	1.8462	0.032429
+	OR6C65	12	55365529	55411467	124	6	10164	1.8459	0.032452
+	ISG20	15	88600622	88666344	152	20	10164	1.8452	0.032502
-	ASXL2	2	25723753	25913474	270	9	10164	1.8437	0.032611
-	ANP32E	1	150208417	150271156	59	5	10164	1.8435	0.032629
+	SDHAF2	11	61395125	61456767	46	2	10164	1.8433	0.032644
+	TPI1	12	6832420	6880946	6	1	10164	1.8416	0.03277
+	TALDO1	11	712432	775024	33	8	10164	1.8405	0.032847
-	PNMAL2	19	46481191	46530912	103	13	10164	1.8396	0.032911
-	ZNF251	8	144710909	144790585	104	6	10164	1.8396	0.032912
-	CDCA3	12	6834798	6887066	5	1	10164	1.8394	0.032925
-	B4GALT1	9	33100637	33202358	222	20	10164	1.8394	0.032931
-	ZDHHC22	14	77121270	77179268	226	43	10164	1.8385	0.032992
+	GTSE1	22	46261014	46343699	114	2	10164	1.8371	0.0331
-	LRRC31	3	169829241	169904935	270	37	10164	1.8369	0.033114
-	VNN1	6	132670858	132749055	201	17	10164	1.8365	0.033143
-	TNFRSF1A	12	6318757	6377117	175	43	10164	1.835	0.033256
+	SETBP1	18	44645173	45078510	861	96	10164	1.8347	0.033277
+	TMEM216	11	61356687	61408863	7	1	10164	1.8344	0.033295
+	ASB7	15	100567550	100661701	237	24	10164	1.8342	0.033313
-	GUCY1A2	11	106664012	107053445	905	54	10164	1.8329	0.033406
+	LOC102724135	15	84407434	84482780	7	3	10164	1.8321	0.033466

STRAND	SYMBOL	CHR	START	STOP	NSNPS	NPARAM	N	ZSTAT	P
+	NAGLU	17	42500933	42554449	70	11	10164	1.8313	0.033526
-	CPSF7	11	61392648	61464992	51	2	10164	1.8308	0.033569
+	GTF2A1L	2	48582780	48689609	219	18	10164	1.8296	0.033654
-	MORC2	22	30915138	31003497	110	10	10164	1.8294	0.033671
+	RTN3	11	63646360	63769891	5	2	10164	1.8292	0.033687
-	BLID	11	122105354	122151215	89	10	10164	1.829	0.033701
-	CASKIN2	17	75490260	75550583	159	14	10164	1.8278	0.033789
+	USP3	15	63469511	63604640	352	23	10164	1.8276	0.033807
+	DISP1	1	222779514	223015995	11	2	10164	1.8268	0.033865
+	RPLP2	11	774936	822876	51	5	10164	1.8266	0.033877
+	VPS13D	1	12195039	12522047	457	20	10164	1.8264	0.033893
+	GNRH2	20	3004061	3055860	122	14	10164	1.8244	0.034046
-	SERPINA10	14	94273313	94328271	174	31	10164	1.8242	0.034059
+	PARVA	11	12341441	12545356	354	47	10164	1.8234	0.034118
-	INPP4B	4	142018028	142881535	1115	56	10164	1.8227	0.034174
+	OR6C76	12	55391254	55437192	125	8	10164	1.8207	0.034329
+	CAPN2	1	223666593	223786018	384	27	10164	1.8192	0.034444
-	LDOC1L	22	44482570	44533125	187	12	10164	1.8185	0.034492
+	SALL3	18	78945275	79008969	138	16	10164	1.8181	0.034522
+	MOGAT2	11	75682819	75741286	227	30	10164	1.8179	0.034537
+	LOC105372824	21	43702000	43748097	124	15	10164	1.8173	0.034586
+	HRCT1	9	35871192	35917141	105	15	10164	1.816	0.034689
+	C10orf142	10	44257750	44304649	189	17	10164	1.8156	0.034718
+	ENAM	4	70593744	70656819	36	10	10164	1.8142	0.034822
+	FOXG1	14	28732072	28780277	128	17	10164	1.8131	0.03491
+	DNTT	10	96269328	96348564	137	7	10164	1.8128	0.034932
+	STK35	20	2064799	2158555	199	9	10164	1.8125	0.034953
+	CFAP54	12	96454546	96885555	1403	37	10164	1.8122	0.034978
+	SOX4	6	21558741	21608619	53	5	10164	1.8113	0.035047
-	FBXO30	6	145788121	145849786	56	4	10164	1.8105	0.035108
+	FBXW8	12	116875951	117042498	182	17	10164	1.8104	0.035117
+	UGT1A7	2	233646938	233783299	536	35	10164	1.8092	0.035209
-	CALN1	7	71769491	72482151	791	32	10164	1.8075	0.035342
+	MIA3	1	222583102	222678012	113	9	10164	1.807	0.035383
-	DHX58	17	42091404	42147733	91	5	10164	1.8059	0.035465
-	DEDD2	19	42188593	42255152	51	3	10164	1.8044	0.035588
-	GRIN3A	9	101559352	101773580	751	34	10164	1.8026	0.035726
-	ABCA12	2	214921542	215173580	127	12	10164	1.8005	0.035887
+	ATP1B1	1	169071709	169142722	134	22	10164	1.8	0.035931
+	LOC105374874	6	562631	612734	217	23	10164	1.7993	0.035989
-	ZNF827	4	145747627	145973455	235	30	10164	1.7991	0.036003
-	SDCBP2	20	1299909	1364235	143	19	10164	1.7987	0.03603
+	CEACAM19	19	44631592	44694359	88	9	10164	1.798	0.03609
+	GBA3	4	22657914	22829575	141	15	10164	1.7977	0.036113
+	CBFA2T2	20	33455122	33660031	192	16	10164	1.797	0.036172
+	ACTL10	20	33631498	33678525	54	10	10164	1.7969	0.036176
+	ZNF526	19	42185340	42238201	40	3	10164	1.7951	0.036318
+	FAM46C	1	117570982	117638389	241	28	10164	1.7947	0.036348
+	FAT4	4	125280897	125502932	684	51	10164	1.7943	0.036383
-	CD59	11	33693010	33771479	76	8	10164	1.7935	0.03645
-	OR51A2	11	4944772	4990713	126	17	10164	1.7934	0.036455
+	CACNA1D	3	53459976	53823151	889	90	10164	1.7933	0.036461
-	CCDC82	11	96342765	96424931	210	23	10164	1.7931	0.036476
-	ZNF311	6	28984785	29040628	243	36	10164	1.7931	0.036479
+	IRAK2	3	10129879	10253745	508	54	10164	1.7922	0.036553
+	LOC105375612	7	158398391	158457848	9	1	10164	1.7915	0.03661
-	TCERG1L	10	131082391	131346721	1316	128	10164	1.79	0.036724
-	LRRD1	7	92134884	92200276	105	11	10164	1.7892	0.036789
-	LOC105379194	5	138734429	138789097	7	1	10164	1.788	0.036888
+	ZBTB34	9	126825665	126895878	139	14	10164	1.7875	0.036929
+	LOC105371906	17	77218764	77280801	85	9	10164	1.7874	0.036933
-	LOC105378803	1	74469955	74572447	83	9	10164	1.7865	0.037007
+	LOC101927789	11	65419421	65476720	142	19	10164	1.7861	0.03704
-	MOB2	11	1459448	1521779	148	8	10164	1.7859	0.037055
+	STOX2	4	183761331	184033526	383	55	10164	1.7858	0.037063
-	IPPK	9	92603184	92705265	28	4	10164	1.7853	0.037109
+	C1QTNF1	17	78987934	79059788	206	28	10164	1.7852	0.037114
-	GATA2	3	128469422	128528187	192	33	10164	1.7847	0.037155
-	CHFR	12	132830352	132922618	114	7	10164	1.7845	0.03717
+	PTPRG	3	61526569	62307609	2145	155	10164	1.7833	0.037267
-	OR8D2	11	124309262	124355197	174	14	10164	1.7823	0.037354
+	MTRNR2L5	10	55563990	55610728	76	5	10164	1.7822	0.037357
-	NPFF	12	53496690	53542638	57	9	10164	1.781	0.03746
-	PPIL4	6	149494495	149581102	85	5	10164	1.7805	0.0375

STRAND	SYMBOL	CHR	START	STOP	NSNPS	NPARAM	N	ZSTAT	P
+	DNAJA4	15	78229145	78292196	212	25	10164	1.7768	0.037802
+	KIAA1324	1	109078963	109216781	186	18	10164	1.7765	0.037823
+	OR2AK2	1	247930332	247976339	61	5	10164	1.7758	0.037887
+	OR5AK2	11	56953914	56999843	141	13	10164	1.7755	0.037904
-	C10orf128	10	49144724	49223585	181	17	10164	1.7726	0.038145
+	NFKB1	4	102466329	102627302	303	14	10164	1.7721	0.038187
-	TNPO3	7	128944180	129090173	237	17	10164	1.7719	0.038209
+	MTAP	9	21767636	21875971	439	31	10164	1.7718	0.038214
-	ACBD5	10	27185214	27277139	476	14	10164	1.7716	0.03823
+	TRIB3	20	345629	407559	42	11	10164	1.7704	0.038327
-	KDR	4	55068259	55160595	233	25	10164	1.7698	0.038378
-	BICD2	9	92701363	92799801	19	2	10164	1.7695	0.038403
-	SBK3	19	55530559	55580543	63	13	10164	1.769	0.038445
-	CYHR1	8	144439582	144500691	94	7	10164	1.7672	0.038594
-	NEB	2	151475339	151769487	143	6	10164	1.767	0.038612
+	NR2C2	3	14912584	15059279	179	23	10164	1.7656	0.038729
-	CEP295NL	17	78880579	78938217	347	35	10164	1.7651	0.038773
+	SLC9A3R1	17	74713612	74779360	129	19	10164	1.7648	0.038796
-	PPP1R10	6	30590400	30653607	307	34	10164	1.7646	0.038815
-	TMC6	17	78102918	78167407	125	27	10164	1.7646	0.038818
+	PKN3	9	128667499	128730920	27	2	10164	1.7632	0.038933
-	MMS19	10	97448324	97533794	152	14	10164	1.7632	0.038933
-	CLASP1	2	121327776	121684476	435	6	10164	1.7632	0.038936
-	TNFRSF13B	17	16929084	17007088	214	30	10164	1.7626	0.038987
+	AP1M1	19	16162854	16245345	2	1	10164	1.7623	0.039005
+	FSCN1	7	5557805	5616656	77	10	10164	1.7623	0.039012
+	UGT1A9	2	233636898	233783305	557	36	10164	1.7621	0.039026
-	CST9L	20	23554732	23603749	113	2	10164	1.7608	0.039139
-	CEND1	11	777110	825126	48	5	10164	1.7588	0.039303
+	TUBG2	17	42624248	42677006	91	8	10164	1.757	0.03946
+	FBXO7	22	32439682	32508831	255	28	10164	1.7568	0.039474
-	KCNK9	8	139590838	139738068	269	33	10164	1.7567	0.039482
+	DISC1	1	231591815	232051272	1041	99	10164	1.7564	0.039508
-	RAB3GAP2	1	220138268	220307501	350	29	10164	1.754	0.039715
-	PAX3	2	222189887	222356263	276	28	10164	1.7537	0.039738
+	NHLRC2	10	113819632	113922506	116	26	10164	1.7528	0.039815
+	B3GALT1	2	167258061	167880856	760	66	10164	1.7525	0.039841
-	TRIM14	9	98073189	98154408	79	15	10164	1.7525	0.039841
+	CFAP221	2	119509423	119670321	206	17	10164	1.7522	0.039871
+	FGF5	4	80231588	80301017	77	12	10164	1.7515	0.039934
-	MLXIPL	7	73583194	73659540	15	2	10164	1.7509	0.039984
+	UGT1A6	2	233656675	233783305	515	29	10164	1.7501	0.040054
+	JRKL	11	96354994	96403563	120	16	10164	1.7492	0.040127
-	PANK1	10	89569497	89680572	408	27	10164	1.7485	0.040185
-	CDC23	5	138177648	138248343	49	2	10164	1.7475	0.040272
+	HIST1H4C	6	26068948	26114337	185	26	10164	1.7474	0.040286
+	NBL1	1	19608229	19668456	128	20	10164	1.7458	0.040423
+	VWA5B1	1	20255907	20370221	128	14	10164	1.7456	0.04044
+	UROD	1	44977133	45025669	143	10	10164	1.7427	0.040692
+	UGT2B10	4	68780993	68842017	19	3	10164	1.7427	0.040694
+	MED15	22	20472542	20597632	54	2	10164	1.7411	0.04083
-	NR6A1	9	124507275	124806310	149	13	10164	1.7411	0.040834
-	FAM219B	15	74889987	74942307	135	23	10164	1.7407	0.040864
+	ARID2	12	45694689	45918037	265	19	10164	1.7407	0.040864
-	SNRPB	20	2451635	2505853	105	14	10164	1.7391	0.041011
-	KLRB1	12	9585274	9642901	51	3	10164	1.7388	0.041035
-	MYH11	16	15693135	15892033	384	35	10164	1.738	0.041107
+	KIF20A	5	138143728	138197715	33	3	10164	1.738	0.041108
-	ATP4B	13	113638804	113693198	39	10	10164	1.7376	0.041136
+	ITGB3	17	47218842	47322711	340	22	10164	1.7371	0.041184
+	POMP	13	28624004	28688957	163	15	10164	1.7341	0.041452
+	PCNP	3	101538534	101604437	144	13	10164	1.7336	0.041494
+	WDR75	2	189406433	189485562	251	11	10164	1.733	0.041546
-	IGFBP3	7	45902245	45956272	159	19	10164	1.7324	0.041597
-	IFI27L2	14	94117781	94164620	142	22	10164	1.7323	0.041609
+	LRIT3	4	109813184	109882315	197	17	10164	1.7306	0.041762
+	HIST1H2AC	6	26089145	26134690	194	23	10164	1.7305	0.041767
+	CACNG8	19	53928036	54000215	1	1	10164	1.7298	0.041832
-	LOC101929552	17	82703905	82751473	89	8	10164	1.7294	0.041868
-	MYH6	14	23371990	23443277	127	19	10164	1.7286	0.041939
+	CETN1	18	545343	592020	37	6	10164	1.7275	0.042035
-	TCF20	22	42150013	42318922	258	17	10164	1.7245	0.042312
+	C15orf41	15	36544603	36820260	529	48	10164	1.7243	0.042328
-	PTPRB	12	70506850	70672440	450	51	10164	1.7241	0.042347

STRAND	SYMBOL	CHR	START	STOP	NSNPS	NPARAM	N	ZSTAT	P
-	SLC4A7	3	27362721	27519420	506	30	10164	1.7226	0.042481
+	LIPG	18	49525699	49602908	187	26	10164	1.722	0.042538
+	PCMT1	6	149714695	149821421	197	5	10164	1.7207	0.042651
+	TMEM87B	2	112020223	112129318	102	10	10164	1.7204	0.04268
-	PRDX3	10	119157699	119213865	157	14	10164	1.7201	0.04271
+	ESRP1	8	94606136	94717466	344	26	10164	1.7201	0.04271
+	IQCF2	3	51826629	51873424	16	1	10164	1.7198	0.042738
-	ZNF256	19	57930833	57982747	88	7	10164	1.7197	0.042746
+	GOSR2	17	46888114	46977000	24	3	10164	1.7195	0.042761
-	GPR151	5	146504854	146551113	113	15	10164	1.719	0.042805
-	NEUROD1	2	181666106	181715665	28	7	10164	1.7187	0.042832
+	ZNF587	19	57814813	57875123	101	7	10164	1.7186	0.042846
+	IRG1	13	76913559	76968659	114	19	10164	1.7178	0.042919
+	ITGA9	3	37417322	37829790	774	58	10164	1.7168	0.04301
+	PRELP	1	203440755	203501352	171	16	10164	1.7153	0.043142
+	CYP27A1	2	218746749	218825293	171	6	10164	1.7138	0.043283
+	FAM32A	19	16150184	16202046	9	1	10164	1.7137	0.043292
-	CIB3	19	16151368	16208525	9	1	10164	1.7137	0.043292
-	C16orf47	16	73116638	73179447	140	14	10164	1.7128	0.043371
+	UGT1A10	2	233601477	233783305	636	34	10164	1.7123	0.043425
-	DSCAML1	11	117417772	117832261	1612	150	10164	1.7114	0.043502
+	POTEA	8	43257370	43376407	1	1	10164	1.7102	0.043618
+	LOC105371432	17	319716	367581	31	9	10164	1.7079	0.043823
+	IRF5	7	128902737	128960042	112	16	10164	1.7079	0.043828
-	GGH	8	63005079	63074445	230	20	10164	1.7077	0.04385
-	ORC4	2	147920397	148056604	236	15	10164	1.7075	0.043864
+	OLFML2A	9	124742158	124824891	10	3	10164	1.7053	0.044068
+	R3HDM1	2	135496455	135735270	383	22	10164	1.7048	0.044111
-	CARD18	11	105127721	105174734	142	5	10164	1.7039	0.0442
+	PPP1R32	11	61446113	61500928	43	2	10164	1.7031	0.044271
-	DIP2C	10	264190	724689	576	31	10164	1.7018	0.044396
-	ZCCHC8	12	122461599	122536073	2	1	10164	1.7015	0.044428
-	AP1G2	14	23549563	23604545	20	4	10164	1.7004	0.044528
+	NXPH1	7	8398955	8762963	1076	99	10164	1.7003	0.044541
-	CSRNP2	12	51051205	51118671	101	9	10164	1.6998	0.044584
+	NRG1	8	31604304	32777957	3180	121	10164	1.6989	0.044673
-	KAT2A	17	42103111	42156393	94	6	10164	1.6974	0.044809
-	UEVLD	11	18519609	18623938	196	17	10164	1.6962	0.044927
-	FYCO1	3	45907899	46030824	291	20	10164	1.6955	0.044986
+	UBE2Q2L	15	84137490	84192233	35	11	10164	1.6946	0.045076
-	EEF2	19	3966056	4020463	43	2	10164	1.6944	0.045095
+	DNAI1	9	34423752	34530989	156	15	10164	1.6938	0.045151
-	CLDN7	17	7249903	7298193	82	14	10164	1.6902	0.0455
+	FST	5	53445341	53497134	164	27	10164	1.6898	0.045532
-	PIEZO1	16	88705338	88819964	419	46	10164	1.6888	0.045627
-	CAGE1	6	7316654	7424709	161	7	10164	1.6882	0.045687
+	OXT	20	3033871	3082517	62	9	10164	1.688	0.045707
+	TARBP2	12	53465203	53516431	27	8	10164	1.6879	0.045715
+	SCARA3	8	27598894	27722743	386	26	10164	1.6876	0.045742
-	SPATA6L	9	4585837	4701801	552	50	10164	1.6875	0.045751
+	LOC100132146	3	46577435	46636543	297	27	10164	1.6871	0.045791
-	DEFB134	8	11983177	12035752	31	3	10164	1.6867	0.045835
+	ARMC4P1	10	27245762	27339145	409	17	10164	1.6862	0.04588
+	LHX1	17	36902475	36954615	59	8	10164	1.6858	0.045915
-	HECTD3	1	44992546	45046355	111	10	10164	1.6856	0.045934
-	LOC105379290	8	12975151	13073442	642	40	10164	1.6832	0.046166
-	BMP4	14	53939736	53991836	135	28	10164	1.6816	0.046325
-	KCNH5	14	62696573	63080256	986	52	10164	1.6809	0.046395
-	OR2W1	6	29034213	29080175	228	30	10164	1.6805	0.046434
-	NKX3-2	4	13530830	13579490	28	8	10164	1.6804	0.046438
+	SAA1	11	18231261	18279977	112	14	10164	1.6789	0.046589
-	HSPG2	1	21812232	21972297	62	6	10164	1.6788	0.046595
+	MPHOSPH8	13	19598647	19683459	131	7	10164	1.6781	0.046661
-	SLC16A12	10	89420294	89570556	182	31	10164	1.678	0.046677
-	BTG4	11	111374095	111549733	432	39	10164	1.6777	0.046701
-	LDB2	4	16491534	16933809	661	73	10164	1.6771	0.046758
-	IL21	4	122602628	122656057	117	18	10164	1.6766	0.046812
-	STC2	5	173304723	173364503	52	8	10164	1.6761	0.046863
+	TMEM125	1	43234951	43284002	23	2	10164	1.6745	0.047011
+	FAM71A	1	212589447	212636778	143	24	10164	1.673	0.047163
-	DND1	5	140660796	140708586	70	4	10164	1.6725	0.047213
+	OR5K2	3	98462681	98508631	105	17	10164	1.6715	0.047309
+	ARF6	14	49858018	49907054	12	3	10164	1.6709	0.047374
-	BPIFC	22	32403845	32499469	302	40	10164	1.6682	0.047642

STRAND	SYMBOL	CHR	START	STOP	NSNPS	NPARAM	N	ZSTAT	P
-	PLAT	8	42164718	42242676	139	22	10164	1.6679	0.047665
-	C1orf210	1	43271865	43320840	7	2	10164	1.6679	0.047667
-	NLRP10	11	7949023	8000424	111	17	10164	1.6678	0.047674
-	LOC105376779	16	88376927	88426933	1	1	10164	1.6677	0.047683
+	WBSCR22	7	73648566	73708221	89	7	10164	1.6676	0.047694
-	CNKSR3	6	154395299	154545619	513	63	10164	1.6673	0.047729
-	PSKH2	8	86037109	86104622	121	9	10164	1.667	0.047759
-	ME3	11	86431108	86707636	1171	50	10164	1.6669	0.047767
+	GAB1	4	143301762	143484565	190	14	10164	1.6668	0.047774
-	SHISA7	19	55418738	55477863	131	23	10164	1.6668	0.04778
-	SLC25A22	11	780475	833269	56	7	10164	1.6653	0.047923
+	SLC2A4	17	7246735	7298048	86	14	10164	1.664	0.048054
-	SAP130	2	127931217	128063093	228	16	10164	1.6639	0.048071
-	CCR7	17	40543769	40600484	19	3	10164	1.6638	0.048073
+	TTLL4	2	218675835	218765416	134	3	10164	1.6637	0.048089
-	HARS	5	140663904	140726727	97	5	10164	1.6634	0.048116
+	SH3GL2	9	17543954	17807129	900	44	10164	1.663	0.048158
+	ZMAT2	5	140663743	140716663	78	4	10164	1.6626	0.048192
-	CYP4F11	19	15902370	15969866	138	7	10164	1.6626	0.0482
-	HIST1H1T	6	26097412	26143136	188	22	10164	1.6625	0.048211
-	TSG101	11	18470311	18561956	201	13	10164	1.662	0.048259
+	TIE1	1	43265895	43333110	10	2	10164	1.6612	0.048338
+	HARS2	5	140656426	140709318	74	4	10164	1.661	0.048357
+	IRF8	16	85864168	85932606	322	42	10164	1.6609	0.048363
+	UTP20	12	101245127	101396619	332	17	10164	1.6591	0.048546
-	ADAMTS20	12	43342843	43586940	266	14	10164	1.6587	0.048588
-	BRD8	5	138129766	138213669	53	3	10164	1.6575	0.048707
+	ZNF534	19	52394321	52461939	132	14	10164	1.657	0.048764
+	ZFP36	19	39371847	39419412	18	2	10164	1.6568	0.048778
+	UGT1A8	2	233582645	233783299	671	39	10164	1.6565	0.04881
-	FAM184A	6	118949829	119184193	489	39	10164	1.6562	0.048838
-	LOC101927764	2	119700868	119795452	108	11	10164	1.6553	0.048929
-	SAA2	11	18228233	18283674	122	16	10164	1.6553	0.048931
-	PAF1	19	39375629	39426195	22	3	10164	1.6545	0.04901
-	ASTN1	1	176847305	177199904	456	30	10164	1.654	0.049067
-	RERG	12	15097782	15256477	373	29	10164	1.6538	0.049085
-	CHCHD3	7	132774862	133117079	535	23	10164	1.6534	0.049124
-	SDPR	2	191824305	191882280	7	2	10164	1.6528	0.049187
+	HOXC12	12	53919930	53966566	74	10	10164	1.6527	0.049197
+	FCGR2B	1	161628115	161688654	60	10	10164	1.6524	0.049225
-	EOGT	3	68965212	69048961	217	19	10164	1.6522	0.049244
+	ATP11AUN	13	112612044	112694497	47	5	10164	1.6514	0.049331
-	SLC44A5	1	75192131	75759322	1210	35	10164	1.6511	0.04936
-	CLIC5	6	45888451	46115348	710	67	10164	1.6508	0.049386
-	FRMD4B	3	69158782	69576340	1066	75	10164	1.6507	0.049396
-	CSNK1A1	5	149485894	149586552	206	22	10164	1.6504	0.049432
-	BARD1	2	214715646	214844711	385	20	10164	1.6499	0.04948
-	MFSD8	4	127907805	128000984	91	3	10164	1.6472	0.049759
+	SLC22A14	3	38246430	38328522	214	12	10164	1.6464	0.049842
+	ZNRF4	19	5420415	5466856	71	17	10164	1.6458	0.049898
-	LOC101927824	10	3042510	3104273	62	10	10164	1.6452	0.049969
+	CACNG3	16	24220553	24372416	378	61	10164	1.6443	0.050058
+	MROH2A	2	233740662	233843423	453	68	10164	1.6439	0.050099
+	C10orf12	10	96946284	96995828	6	1	10164	1.6421	0.050289
+	LOC105378722	1	52517934	52575732	45	3	10164	1.6417	0.050322
+	LOC102724957	11	15588774	15715368	225	14	10164	1.6411	0.050388
+	PPFIA4	1	202991521	203088736	279	51	10164	1.6404	0.050457
+	CIART	1	150247533	150297093	75	9	10164	1.6387	0.050642
-	C12orf49	12	116703320	116773070	135	24	10164	1.6371	0.05081
+	NLRC5	16	56954547	57093526	424	79	10164	1.6365	0.050865
+	PIAS4	19	3972598	4049386	49	2	10164	1.6363	0.050887
+	RMND5B	5	178095532	178158570	83	10	10164	1.6361	0.050905
+	VIT	2	36661690	36824794	271	28	10164	1.6316	0.051377
+	SLC6A5	11	20564400	20669285	466	33	10164	1.6311	0.051437
-	SERPINB1	6	2822332	2877049	205	20	10164	1.6299	0.051561
-	ACOT12	5	81319991	81429169	262	38	10164	1.6289	0.051666
+	HSPA4L	4	127747209	127844073	83	5	10164	1.6276	0.05181
+	BAALC	8	103105693	103240305	435	40	10164	1.6269	0.051876
+	TMEM98	17	32892910	32951649	16	2	10164	1.6261	0.051968
-	FAM13B	5	137927949	138086961	122	7	10164	1.625	0.052081
-	WASF2	1	27394226	27525187	100	2	10164	1.6249	0.052092
-	NME5	5	138105172	138174443	51	4	10164	1.6242	0.052166
+	MYL12B	18	3227113	3288284	178	13	10164	1.6235	0.052237
+	OR10H4	19	15914008	15959958	75	3	10164	1.6235	0.05224

STRAND	SYMBOL	CHR	START	STOP	NSNPS	NPARAM	N	ZSTAT	P
-	AIDA	1	222658013	222747901	77	8	10164	1.6227	0.052326
+	TERF1	8	72973862	73058123	191	18	10164	1.6225	0.052352
+	CABLES1	18	23099564	23270470	265	24	10164	1.6224	0.05236
-	AGO2	8	140510156	140677326	320	27	10164	1.6214	0.052463
-	ZNF20	19	12121350	12175407	70	7	10164	1.6214	0.052467
+	UNC5B	10	71177535	71312879	400	49	10164	1.621	0.052511
-	CSPG4	15	75664322	75747848	160	15	10164	1.6202	0.052597
-	ARL1	12	101383120	101442820	109	12	10164	1.62	0.052621
+	PLEKHG2	19	39377582	39438415	24	3	10164	1.6192	0.052705
+	NNAT	20	37486205	37533690	92	12	10164	1.6182	0.05281
+	FOXI2	10	127702274	127751186	112	7	10164	1.6181	0.052815
-	ECM2	9	92483547	92571092	128	6	10164	1.6179	0.052839
+	APOBEC3A	22	38922522	38973183	115	9	10164	1.6176	0.052871
+	SLC2A8	9	127362138	127417898	175	23	10164	1.6174	0.052892
-	S100A7L2	1	153426995	153475027	89	6	10164	1.6174	0.052897
-	NSMCE1	16	27214994	27303809	129	10	10164	1.6156	0.053086
+	ACSM5	16	20374520	20450987	114	15	10164	1.6155	0.053105
-	PPP5D1	19	46470788	46636200	230	26	10164	1.6153	0.053128
+	IL36RN	2	113023638	113074744	233	20	10164	1.6151	0.053149
+	SLC29A4	7	5239374	5314073	87	16	10164	1.6117	0.053508
-	SLC35C2	20	46339528	46399458	63	8	10164	1.6115	0.053531
+	PLK4	4	127845861	127909224	63	4	10164	1.6109	0.053603
+	IL17RB	3	53811550	53875800	156	24	10164	1.6109	0.053606
+	TTC1	5	159974100	160075545	25	2	10164	1.6099	0.053708
+	DEFB135	8	11947321	11994590	21	4	10164	1.6098	0.053724
+	LCMT1	16	25076726	25188231	145	10	10164	1.6089	0.053819
-	ESF1	20	13704322	13819933	96	9	10164	1.6088	0.05383
+	SNRNP27	2	69858943	69915236	116	15	10164	1.6083	0.053885
+	MYF5	12	80681929	80729668	70	3	10164	1.6078	0.053939
-	ZDHHC11	5	785605	893973	141	10	10164	1.6069	0.054037
-	CDK5RAP2	9	120378869	120615170	149	8	10164	1.6069	0.054043
+	VCL	10	73963078	74130160	205	15	10164	1.6065	0.054082
+	RHPN1	8	143333842	143394220	145	14	10164	1.6063	0.054103
-	GPR149	3	154327672	154464715	167	17	10164	1.606	0.054141
-	C9orf57	9	72041381	72095605	41	2	10164	1.6049	0.054256
-	NUP43	6	149714315	149781572	120	4	10164	1.6047	0.054275
-	KCTD9	8	25417842	25493468	176	7	10164	1.6035	0.054413
-	NRL	14	24068693	24149802	81	12	10164	1.6029	0.05448
-	GZMB	14	24620948	24669226	182	24	10164	1.6023	0.054544
-	LOC105375396	7	92402550	92472456	123	20	10164	1.6022	0.054558
+	RNF20	9	101498849	101573344	183	19	10164	1.6013	0.054658
-	GABARAP	17	7230419	7277434	62	12	10164	1.6011	0.054678
-	S100A7	1	153447744	153495661	104	6	10164	1.6003	0.054764
-	CSTB	21	43763665	43811375	158	13	10164	1.5993	0.054875
-	BEGAIN	14	100527147	100622404	286	36	10164	1.5988	0.054931
-	FASTKD5	20	3136519	3194886	63	7	10164	1.5983	0.054989
-	BRD7	16	50309018	50403966	148	16	10164	1.5975	0.055073
+	NPEPL1	20	58654131	58725844	160	14	10164	1.5973	0.055103
+	CACNA1H	16	1118241	1231772	104	11	10164	1.5964	0.055205
+	DGCR8	22	20045232	20121877	206	20	10164	1.596	0.05524
+	KL	13	32981063	33076145	312	32	10164	1.5958	0.055264
-	CFAP46	10	132798392	132978163	458	19	10164	1.5945	0.055414
-	FAM134B	5	16462816	16652058	345	29	10164	1.5941	0.05546
-	TIMP2	17	78842977	78960390	546	55	10164	1.5937	0.055503
+	CROT	7	87310635	87409796	182	22	10164	1.5934	0.055537
+	ZNF808	19	52492652	52580178	170	5	10164	1.5933	0.055547
+	HTR6	1	19630287	19690966	157	21	10164	1.5931	0.055569
-	CLU	8	27586917	27650031	201	27	10164	1.593	0.055579
+	INTU	4	127588271	127726779	207	13	10164	1.5927	0.055612
+	SRC	20	37309685	37415432	147	14	10164	1.5914	0.055759
-	AK3	9	4699556	4777043	413	52	10164	1.591	0.055802
+	HFE	6	26052281	26106117	197	28	10164	1.5907	0.05584
-	MICALL2	7	1418465	1494497	135	6	10164	1.5906	0.055846
+	KIF20B	10	89666590	89784943	415	15	10164	1.5906	0.055854
-	LOC101927016	13	63822066	63868720	2	1	10164	1.5903	0.055887
-	NCBP2	3	196925402	196977593	18	2	10164	1.5896	0.055962
+	RTP4	3	187333380	187381584	131	17	10164	1.5888	0.05605
-	HIST1H4H	6	26275126	26320499	186	16	10164	1.5872	0.056236
+	OR4D10	11	59442430	59488365	6	2	10164	1.5871	0.056248
-	LOC643802	16	53359362	53406129	129	21	10164	1.5867	0.056289
+	LDLRAD2	1	21777265	21835221	26	3	10164	1.5855	0.056432
+	MRRF	9	122229603	122335297	109	17	10164	1.5853	0.056449
+	RIOK1	6	7354419	7428037	131	5	10164	1.5852	0.056455
-	GSTA3	6	52886639	52944798	63	5	10164	1.5847	0.056521

STRAND	SYMBOL	CHR	START	STOP	NSNPS	NPARAM	N	ZSTAT	P
+	PSD2	5	139686903	139854466	165	17	10164	1.5837	0.056635
+	TMEM260	14	56544791	56661884	192	16	10164	1.5831	0.056702
-	EXOC7	17	76070993	76138787	72	6	10164	1.5812	0.056921
+	MYF6	12	80672629	80719478	81	3	10164	1.5803	0.057016
+	SCGB1A1	11	62384035	62433206	192	23	10164	1.5788	0.057186
-	RAB20	13	110513066	110596737	383	45	10164	1.5785	0.057225
+	OR4D9	11	59479913	59525857	47	2	10164	1.578	0.057279
-	ZNF521	18	25051924	25387250	359	37	10164	1.5773	0.057368
+	RRP1B	21	43624551	43706079	149	9	10164	1.577	0.057393
+	OR4D11	11	59468576	59514511	39	2	10164	1.5758	0.057534
+	INHBB	2	120311143	120361807	99	23	10164	1.5757	0.057543
+	RRP1	21	43754535	43814102	195	17	10164	1.5749	0.057634
+	GNG11	7	93886704	93936514	17	4	10164	1.5742	0.057722
+	TBC1D10C	11	67368686	67420090	96	14	10164	1.5735	0.057801
+	ATP5S	14	50277329	50336715	54	2	10164	1.5731	0.057853
-	NRIP2	12	2814882	2870536	93	8	10164	1.5729	0.057867
+	TMCO2	1	40212901	40261693	71	2	10164	1.5722	0.057951
-	SLC25A40	7	87823568	87911377	168	15	10164	1.5719	0.057991
-	STRA6	15	74169466	74247259	173	26	10164	1.5718	0.058001
+	NDUFB10	16	1924516	1971975	78	13	10164	1.5711	0.058079
+	SYCP2L	6	10851831	10984309	541	55	10164	1.5709	0.0581
+	GABRB1	4	46958858	47437343	1038	62	10164	1.5705	0.058148
+	SAMD7	3	169870106	169949175	320	29	10164	1.5703	0.058171
-	LZTS3	20	3152617	3208592	94	10	10164	1.5702	0.058188
-	HSF2BP	21	43519186	43694499	149	8	10164	1.569	0.05832
-	CD69	12	9742486	9795901	147	9	10164	1.5688	0.058343
-	MYH7	14	23402738	23470686	118	11	10164	1.5687	0.058355
+	ADAM22	7	87899157	88212889	451	26	10164	1.5676	0.058491
-	SAA2-SAA4	11	18221355	18283674	143	19	10164	1.5676	0.058491
+	DNAJB5	9	34954369	35008433	119	27	10164	1.5674	0.058512
+	CDK14	7	90561362	91220590	1620	56	10164	1.5659	0.058681
+	ANLN	7	36354803	36463791	277	28	10164	1.5659	0.058685
-	CLDND1	3	98505473	98558066	112	22	10164	1.5659	0.058689
+	RTP3	3	46462995	46510949	115	15	10164	1.5653	0.058755
+	HSCB	22	28707001	28767518	154	24	10164	1.5649	0.058807
+	C15orf53	15	38661598	38710038	112	19	10164	1.5645	0.058856
+	AKAP9	7	91905867	92120673	368	13	10164	1.5635	0.058962
-	CEP152	15	48652534	48846918	194	21	10164	1.5635	0.058962
-	HIST1H2BC	6	26113467	26158904	150	24	10164	1.5631	0.05902
+	CDCA2	8	25423574	25521308	209	9	10164	1.5626	0.059076
-	PRB4	12	11297081	11345435	11	2	10164	1.5625	0.05909
-	OR5AU1	14	21144937	21191025	73	6	10164	1.5617	0.059178
+	CD38	4	15743298	15859083	319	15	10164	1.5617	0.05918
-	NTHL1	16	2029815	2082866	39	11	10164	1.5615	0.059209
+	FN3K	17	82700576	82761197	102	8	10164	1.561	0.059256
+	PPP4R4	14	94139312	94289755	378	22	10164	1.5607	0.059298
-	IQCF5	3	51863721	51910584	1	1	10164	1.5604	0.059337
-	KRTAP5-1	11	1574342	1620283	193	20	10164	1.5603	0.05935
-	EQTN	9	27274654	27332139	110	13	10164	1.5598	0.059405
-	CLPS	6	35784982	35832344	161	26	10164	1.5593	0.05946
+	PDXK	21	43684097	43772307	249	26	10164	1.5586	0.059541
+	STX11	6	144105051	144201939	320	31	10164	1.5575	0.059681
-	SNTA1	20	33397957	33478892	94	12	10164	1.5571	0.059722
-	EXT1	8	117789363	118146819	702	48	10164	1.5568	0.059758
-	GDF6	8	96132330	96195792	4	1	10164	1.5567	0.059767
+	ISX	22	35031136	35097387	224	12	10164	1.556	0.059853
-	HEPACAM2	7	93178586	93267297	122	16	10164	1.5551	0.059964
-	SRP68	17	76028775	76107526	88	2	10164	1.555	0.059973
+	CALM3	19	46566255	46620782	26	5	10164	1.5538	0.060119
+	COL6A6	3	130482177	130688135	552	54	10164	1.5534	0.060161
+	SPO11	20	57294759	57353993	95	9	10164	1.5525	0.060267
+	CCDC7	10	32411082	32892864	1349	23	10164	1.5524	0.06028
+	C9orf106	9	129286016	129332603	1	1	10164	1.5517	0.060369
-	TJP1	15	29689367	30003799	680	43	10164	1.5511	0.060435
-	LOC105375131	7	4116653	4172174	223	23	10164	1.5511	0.060443
+	ANKRD66	6	46711917	46769218	102	17	10164	1.5508	0.060474
-	ZNF605	12	132911433	132991306	11	3	10164	1.5507	0.060482
-	LRRC20	10	70288970	70417650	335	23	10164	1.5503	0.060533
+	LOC105372444	19	51236237	51305425	199	26	10164	1.5486	0.060742
-	DIRAS1	19	2704567	2756392	48	2	10164	1.5484	0.060761
-	AGBL2	11	47649169	47750382	75	3	10164	1.5483	0.060773
+	RASAL2	1	178058729	178489514	575	18	10164	1.5483	0.060781
-	PLA2G3	22	31124807	31175757	86	18	10164	1.5479	0.060822
+	CELA2A	1	15421728	15482091	109	11	10164	1.5469	0.060949

STRAND	SYMBOL	CHR	START	STOP	NSNPS	NPARAM	N	ZSTAT	P
+	STK38L	12	27209145	27335959	297	24	10164	1.546	0.061051
+	TCERG1	5	146412310	146521508	246	18	10164	1.5457	0.06109
+	ZACN	17	76044182	76092804	58	2	10164	1.5452	0.061143
-	RGS22	8	99950936	100141116	383	25	10164	1.5452	0.061149
+	ZNF7	8	144792518	144857508	137	8	10164	1.5449	0.061185
+	ZNF701	19	52535273	52597174	73	3	10164	1.5445	0.061228
-	NINJ2	12	554296	698741	386	31	10164	1.5438	0.061315
+	ZMPSTE24	1	40223050	40304184	69	2	10164	1.5437	0.061335
-	HIST1H3F	6	26240142	26285607	143	22	10164	1.5432	0.061396
-	CTDNEP1	17	7233587	7286940	76	13	10164	1.5425	0.061472
-	TBC1D32	6	121069494	121369956	1194	24	10164	1.5421	0.061526
+	CERS6	2	168421249	168785134	861	45	10164	1.5407	0.061695
-	TMF1	3	69009827	69087333	132	12	10164	1.5407	0.061698
-	ZDHHC12	9	128710869	128759141	22	3	10164	1.5405	0.061724
+	PRSS3	9	33715466	33809231	118	7	10164	1.5401	0.06177
+	C15orf26	15	81064408	81159175	92	9	10164	1.5389	0.061913
-	LOC101927403	13	33002924	33051442	195	25	10164	1.5384	0.061973
+	ACVR2A	2	147809517	147940827	233	20	10164	1.538	0.062028
-	STK25	2	241484707	241544730	182	23	10164	1.5376	0.062068
+	NEK11	3	130991850	131360465	648	23	10164	1.5357	0.062308
-	HPCAL4	1	39668648	39726710	107	17	10164	1.5331	0.06262
+	GALNT8	12	4685586	4782726	247	15	10164	1.5329	0.062646
-	OR6C70	12	55459200	55694844	891	27	10164	1.5313	0.062851
-	FBXO3	11	33730944	33809531	123	11	10164	1.5305	0.062943
+	GLIS2	16	4281181	4349597	83	11	10164	1.5304	0.062959
-	NDUFA2	5	140635363	140682785	64	4	10164	1.53	0.063004
-	PFN3	5	177390107	177435636	31	5	10164	1.5296	0.063063
+	KCNIP1	5	170318487	170746632	1122	133	10164	1.5289	0.063144
+	PCK2	14	24059131	24114132	63	15	10164	1.5287	0.063172
-	LOC101928726	5	177098798	177156834	93	9	10164	1.5283	0.063215
+	RARRES3	11	63501801	63556458	47	4	10164	1.5281	0.063244
-	PTGIR	19	46600040	46660118	86	9	10164	1.528	0.063261
+	GRK5	10	119172685	119465619	913	68	10164	1.5264	0.063449
-	RAB5C	17	42114976	42190044	119	11	10164	1.5264	0.063451
+	LARS2	3	45353561	45558842	418	38	10164	1.5263	0.063472
-	EZH1	17	42690275	42780078	108	9	10164	1.5259	0.063512
+	SLC34A1	5	177349431	177408848	39	4	10164	1.5258	0.063526
+	INA	10	103242163	103300351	143	16	10164	1.5257	0.063547
-	LOC105374156	3	150861019	150913949	47	9	10164	1.5256	0.06356
-	TESK2	1	45333883	45526342	535	22	10164	1.5252	0.063604
+	DBF4	7	87841229	87919541	139	13	10164	1.525	0.063631
-	SPTY2D1	11	18596401	18669786	63	9	10164	1.5248	0.06366
+	SEPT9	17	77246410	77510596	635	57	10164	1.5245	0.063695
+	RPS19	19	41824918	41881416	72	4	10164	1.5233	0.063845
+	ZNF672	1	248803178	248859517	52	4	10164	1.5226	0.063927
+	USP38	4	143149917	143233874	6	2	10164	1.5219	0.064019
-	SRPK1	6	35823034	35956180	363	22	10164	1.5217	0.064042
+	MMACHC	1	45465184	45521067	155	15	10164	1.5208	0.064152
+	LOC105371558	17	15782863	15940886	129	2	10164	1.5197	0.064295
+	SLC44A1	9	105209608	105448504	24	2	10164	1.5196	0.064309
+	FAM231B	1	16503724	16550035	1	1	10164	1.5195	0.064313
+	LOC102724970	1	16500773	16546956	1	1	10164	1.5195	0.064313
-	TMEM2	9	71673366	71804466	194	28	10164	1.5192	0.064354
-	UBOX5	20	3097573	3194910	77	10	10164	1.5191	0.064367
+	RPEL1	10	103210887	103258016	85	17	10164	1.519	0.064376
+	MRPS18B	6	30582320	30636397	262	32	10164	1.5179	0.06452
+	DCAF11	14	24079697	24135242	61	9	10164	1.5178	0.06453
+	WHSC1	4	1836396	1992207	82	6	10164	1.5176	0.064558
+	LOC105370399	14	22521953	22569793	131	22	10164	1.5168	0.064654
-	LOC105369145	6	9835518	10194438	1182	70	10164	1.5168	0.064661
-	SNX16	8	81789583	81877325	226	8	10164	1.5165	0.064693
+	PWP1	12	107650813	107722480	188	22	10164	1.5164	0.064703
-	PLEKHM3	2	207811288	208075372	363	18	10164	1.5159	0.064777
+	H2AFY2	10	70017601	70122284	256	28	10164	1.5154	0.064834
-	PRC1	15	90956038	91029651	193	15	10164	1.515	0.064881
-	PLXNA2	1	208012242	208279384	887	81	10164	1.5145	0.064954
+	NXPE2	11	114613490	114717117	332	17	10164	1.5143	0.064969
+	KLF13	15	31291855	31445665	144	12	10164	1.5141	0.064995
-	SLC39A3	19	2722524	2775076	69	3	10164	1.5139	0.065029
+	MRAP2	6	83989786	84156282	264	26	10164	1.5139	0.06503
+	N4BP3	5	178078506	178137034	86	13	10164	1.5135	0.065081
-	LINS	15	100559223	100637240	251	21	10164	1.5135	0.065082
+	CCDC70	13	51826981	51876236	74	9	10164	1.5132	0.065116
-	NT5C3B	17	41815082	41871271	119	4	10164	1.5131	0.065128

STRAND	SYMBOL	CHR	START	STOP	NSNPS	NPARAM	N	ZSTAT	P
-	EDAR	2	108884471	109024372	165	20	10164	1.513	0.065144
-	ZWINT	10	56347438	56396302	28	3	10164	1.513	0.065144
+	IARS2	1	220059113	220158041	189	21	10164	1.5114	0.06534
-	ZNF671	19	57709747	57762627	84	9	10164	1.5098	0.065545
-	HTR1B	6	77452231	77498403	152	24	10164	1.5097	0.065561
+	CCDC109B	4	109525199	109698645	639	36	10164	1.5081	0.065758
-	FBXO43	8	100123360	100185569	139	12	10164	1.508	0.065779
+	ITM2B	13	48198138	48272096	121	17	10164	1.5079	0.065796
+	LOC105370939	15	31356503	31414730	81	9	10164	1.5077	0.065821
+	RLF	1	40126369	40250921	231	4	10164	1.5076	0.065824
-	WDR61	15	78273233	78334726	149	25	10164	1.5076	0.065831
+	PGD	1	10363992	10430511	159	13	10164	1.507	0.065911
+	LOC105378056	6	150565272	150611061	155	32	10164	1.5069	0.065923
+	ZDBF2	2	206239641	206324438	117	9	10164	1.5066	0.065952
-	RANBP9	6	13611498	13746580	186	18	10164	1.5065	0.065966
-	KIAA0895	7	36314150	36425125	248	24	10164	1.506	0.066038
+	DDX18	2	117779679	117842377	177	16	10164	1.5053	0.066122
-	PPID	4	158699127	158758400	39	4	10164	1.5053	0.066123
-	ZCCHC11	1	52413275	52588463	116	4	10164	1.5042	0.066271
-	ATP5G2	12	53655160	53711728	153	16	10164	1.504	0.066292
+	MPI	15	74855011	74909457	128	24	10164	1.5037	0.066333
-	TTC33	5	40701576	40790970	191	18	10164	1.503	0.06642
-	TREH	11	118648232	118714690	240	10	10164	1.5013	0.06664
+	GALR2	17	76037088	76087541	53	2	10164	1.501	0.066672
+	WDR55	5	140629799	140682345	71	4	10164	1.5006	0.066734
-	IGSF6	16	21631284	21687666	78	7	10164	1.4999	0.066815
+	ADORA2B	17	15892783	15985896	64	2	10164	1.4996	0.066862
+	PCP4	21	39832421	39939397	376	38	10164	1.4986	0.066992
+	STX16	20	58616253	58689526	127	18	10164	1.4982	0.067043
+	BROX	1	222677553	222745196	52	6	10164	1.4968	0.067217
-	LRP11	6	149785188	149899348	258	15	10164	1.4966	0.067244
-	CTTNBP2	7	117700648	117908507	207	25	10164	1.4963	0.067293
-	SPTBN5	15	41838146	41929077	289	38	10164	1.4959	0.067343
-	SH3BP5L	1	248800446	248860955	54	4	10164	1.4955	0.067392
-	LYPD5	19	43785927	43862199	289	23	10164	1.4944	0.067533
-	EIF3H	8	116634816	116790823	128	10	10164	1.4943	0.067552
+	ZNF283	19	43792292	43858898	259	21	10164	1.494	0.067592
+	GTPBP4	10	953409	1027768	174	16	10164	1.4935	0.067655
+	CD22	19	35294166	35357361	185	39	10164	1.4927	0.067762
+	LLGL2	17	75490080	75585209	310	21	10164	1.4923	0.067807
-	ARHGAP17	16	24909384	25050601	227	17	10164	1.4918	0.067872
-	ASTN2	9	116415225	117450039	3292	222	10164	1.4918	0.067878
+	CD79A	19	41842120	41891372	54	4	10164	1.4911	0.067962
-	LYPD4	19	41823686	41879862	75	4	10164	1.4906	0.068035
-	ZNF625	19	12134890	12191731	74	6	10164	1.4902	0.068083
+	IL13	5	132623173	132671109	105	15	10164	1.4899	0.068123
+	FAM159A	1	52596439	52679120	114	15	10164	1.4898	0.068133
-	GNRH1	8	25409258	25460040	113	6	10164	1.4894	0.068196
+	LDHAL6B	15	59171816	59218588	87	7	10164	1.4887	0.068278
-	GNG8	19	46623934	46670534	60	7	10164	1.4881	0.068358
+	ZNF215	11	6891418	7015354	141	17	10164	1.4881	0.06836
+	ABCD3	1	94383086	94528663	169	22	10164	1.488	0.068369
+	CFAP57	1	43137330	43264358	156	3	10164	1.4874	0.068456
-	KLHL12	1	202881096	202963644	248	25	10164	1.4868	0.068537
+	KNTC1	12	122492249	122637311	57	5	10164	1.4861	0.068627
-	TRG	7	38230024	38403055	72	8	10164	1.486	0.068633
+	LOC100996273	17	77404912	77456373	124	12	10164	1.485	0.068771
-	LATS1	6	149648153	149753256	139	5	10164	1.485	0.068774
-	LOC105372022	18	23123586	23170761	80	9	10164	1.4848	0.068799
+	ABRACL	6	138993682	139053302	159	28	10164	1.4844	0.068856
-	C7orf31	7	25115200	25215748	201	20	10164	1.4834	0.068986
-	SLC38A11	2	164887745	164990560	299	24	10164	1.4807	0.069337
-	SLC2A3	12	7909228	7971296	98	19	10164	1.4804	0.069389
+	DSG3	18	31412769	31488702	207	21	10164	1.4802	0.069414
+	CCDC129	7	31479071	31668720	386	16	10164	1.4784	0.069652
+	KIAA0195	17	75406159	75510452	192	14	10164	1.4778	0.069727
-	PDE6D	2	231722425	231816327	92	2	10164	1.4765	0.069911
+	TMEM255B	13	113724210	113823797	224	14	10164	1.4764	0.069916
-	APH1A	1	150255399	150304209	89	9	10164	1.4759	0.069987
+	ATG101	12	52034974	52087495	42	3	10164	1.4758	0.070004
+	REP15	12	27661495	27707633	103	22	10164	1.4752	0.070078
-	FKBP1A	20	1358977	1428172	217	18	10164	1.4746	0.070163
-	IQSEC1	3	12887043	13317998	837	87	10164	1.474	0.070241
+	FAM222A	12	109679382	109780509	65	7	10164	1.4739	0.07025

STRAND	SYMBOL	CHR	START	STOP	NSNPS	NPARAM	N	ZSTAT	P
-	PWWP2A	5	160033562	160155808	45	3	10164	1.4736	0.070289
-	WDR26	1	224375143	224469299	177	16	10164	1.4735	0.070308
+	C4orf29	4	127930261	128046326	116	4	10164	1.4728	0.070402
+	C11orf84	11	63778374	63837718	40	7	10164	1.4726	0.070424
+	PINLYP	19	43541800	43592104	167	29	10164	1.4705	0.070715
-	LGALS3BP	17	78961253	79014979	176	29	10164	1.4699	0.070789
-	EGLN1	1	231353751	231460044	73	3	10164	1.4698	0.070813
+	SEMG2	20	45186369	45234458	39	3	10164	1.4695	0.070845
+	NUPL2	7	23146827	23211011	84	6	10164	1.4689	0.070928
-	CLPB	11	72282425	72469680	419	31	10164	1.4687	0.070961
-	ERCC8	5	60863832	60980078	129	9	10164	1.4683	0.071008
-	HIST1H4G	6	26236611	26281977	139	22	10164	1.4676	0.0711
-	WASL	7	123671927	123784071	4	1	10164	1.4675	0.071124
-	CD164L2	1	27369100	27418333	29	2	10164	1.4673	0.071143
+	ACTR3	2	113854934	113971554	392	19	10164	1.4672	0.07116
+	OGFOD2	12	122939703	122990041	6	1	10164	1.4669	0.0712
-	POLDIP2	17	28336628	28392589	75	4	10164	1.4659	0.071334
-	BLCAP	20	37507417	37562931	141	10	10164	1.4657	0.071362
+	HIST1H2BI	6	26237976	26283412	135	22	10164	1.4657	0.071363
+	YBEY	21	46251310	46323225	270	21	10164	1.4653	0.071414
+	LOC101060569	15	30480862	30528467	8	1	10164	1.4644	0.071541
+	MYB	6	135145981	135229173	129	10	10164	1.4641	0.07158
-	RCAN1	21	34506442	34650142	644	31	10164	1.4637	0.071644
+	TRHDE	12	72237580	72675644	586	17	10164	1.4633	0.07169
-	POLD4	11	67340765	67388596	63	10	10164	1.463	0.07174
-	SCAMP2	15	74833730	74908365	183	33	10164	1.4626	0.071784
+	FAM8A1	6	17565287	17621719	89	9	10164	1.4624	0.071813
+	GNAS	20	58804740	58921195	124	17	10164	1.4622	0.071842
+	CCDC132	7	93197340	93371123	226	28	10164	1.4622	0.071847
-	ADD2	2	70651202	70803243	561	38	10164	1.4619	0.07189
-	CRK	17	1411353	1491267	109	9	10164	1.4615	0.071945
-	CHDH	3	53806297	53881393	177	27	10164	1.4614	0.071953
+	CHST4	16	71491120	71548590	89	10	10164	1.461	0.072006
-	LOC102724965	2	45638733	45685986	157	28	10164	1.4606	0.072064
+	THUMPD3	3	9327976	9396791	156	17	10164	1.4599	0.072152
+	ANKHD1	5	140366814	140549856	109	11	10164	1.4597	0.072192
+	KLRD1	12	10269446	10370876	113	10	10164	1.4594	0.072232
+	FAM84A	2	14597686	14650046	61	12	10164	1.459	0.072278
+	GFRAL	6	55292469	55412493	748	32	10164	1.4586	0.072332
+	NKD1	16	50513330	50659249	123	10	10164	1.4586	0.072339
+	ZNF793	19	37471447	37553337	160	20	10164	1.4585	0.072353
+	OR6C75	12	55330111	55376049	173	8	10164	1.4579	0.072433
+	MYRIP	3	39773914	40270321	1302	57	10164	1.4578	0.072452
+	LRCH3	3	197756226	197894310	159	10	10164	1.4576	0.072471
+	POLD3	11	74557530	74679341	312	13	10164	1.4574	0.072497
+	ASH2L	8	38069965	38150080	65	4	10164	1.4573	0.072516
+	TBX1	22	19721703	19793593	144	18	10164	1.456	0.072695
+	FAM20C	7	157777	270774	228	20	10164	1.4556	0.072745
+	ZNF136	19	12128057	12199249	94	7	10164	1.4556	0.072757
+	TMEM138	11	61327001	61386502	9	1	10164	1.4537	0.07301
-	DHX16	6	30643119	30708053	354	27	10164	1.4532	0.073079
-	ABCB9	12	122908708	123010125	23	2	10164	1.4531	0.073099
-	TNNT2	1	201349008	201412765	193	27	10164	1.453	0.073115
-	SLC7A10	19	33198664	33260850	156	34	10164	1.4524	0.073197
-	PTAR1	9	69699522	69795044	147	19	10164	1.4522	0.073218
+	RUNDC3B	7	87592675	87842298	438	22	10164	1.4521	0.073243
+	KLHL10	17	41802490	41858347	117	5	10164	1.4509	0.073397
+	LOC105377594	4	186069788	186134062	217	22	10164	1.4507	0.073428
+	MSRA	8	10019224	10438891	1774	94	10164	1.4507	0.073433
+	LOC105372271	19	10002577	10051022	10	2	10164	1.4507	0.073438
+	MSI2	17	57221570	57710477	800	113	10164	1.4503	0.073491
-	EBNA1BP2	1	43154174	43207570	100	1	10164	1.4501	0.073514
+	WWC1	5	168257060	168482303	458	60	10164	1.4485	0.073739
+	ANKHD1-EIF4EB	5	140366814	140559578	118	11	10164	1.4484	0.07375
-	C12orf43	12	120992422	121051502	223	26	10164	1.4478	0.073838
+	OSBP2	22	30658782	30917824	120	20	10164	1.4476	0.073871
-	TUBE1	6	112060657	112122548	168	10	10164	1.447	0.073947
-	IQCB1	3	121759763	121870079	328	11	10164	1.4469	0.07396
+	MED29	19	39356323	39410563	5	1	10164	1.4454	0.074173
+	ADK	10	74116185	74719303	664	9	10164	1.4449	0.074236
-	RBM18	9	122229555	122299864	91	14	10164	1.4448	0.074259
+	SFXN3	10	100996229	101051241	105	23	10164	1.4441	0.07436
-	GPHB5	14	63302835	63353879	139	19	10164	1.444	0.074372
-	ZSWIM5	1	45006399	45241578	395	17	10164	1.4438	0.074403

STRAND	SYMBOL	CHR	START	STOP	NSNPS	NPARAM	N	ZSTAT	P
+	MAG	19	35257086	35323807	194	32	10164	1.4434	0.074451
+	SLC2A10	20	46673347	46746347	29	2	10164	1.4429	0.074525
-	TP53RK	20	46674365	46724637	29	2	10164	1.4429	0.074525
-	FAM219A	9	34388184	34493610	136	12	10164	1.4428	0.074541
-	ASPN	9	92446205	92517562	110	6	10164	1.4427	0.074555
-	TWISTNB	7	19685462	19744037	148	15	10164	1.4426	0.074569
-	LOC105375938	8	60899752	60945855	117	14	10164	1.4426	0.074569
+	SLC45A1	1	8283085	8354167	5	1	10164	1.4423	0.074612
+	KDM8	16	27168486	27232305	52	7	10164	1.4423	0.074615
+	RPIA	2	88656648	88760935	215	7	10164	1.4412	0.074764
+	MRPS21	1	150258842	150318979	129	11	10164	1.4407	0.074837
+	CENPP	9	92290457	92625164	435	10	10164	1.4406	0.074844
+	KCTD20	6	36407767	36501143	119	10	10164	1.4406	0.074855
-	NOL8	9	92287358	92360994	74	6	10164	1.4399	0.074947
-	POC5	5	75664198	75752488	389	22	10164	1.4395	0.075003
+	ODF3L1	15	75688944	75737688	88	11	10164	1.4391	0.075055
+	YTHDF2	1	28701621	28779775	62	2	10164	1.4391	0.075065
+	ZNF79	9	127389374	127456964	194	17	10164	1.4388	0.075099
+	UHRF2	9	6378148	6517056	238	12	10164	1.4387	0.075117
-	GIP	17	48948556	49003593	111	13	10164	1.4386	0.075129
+	FAM45A	10	119069065	119147984	196	14	10164	1.4378	0.075238
+	CASS4	20	56376548	56469340	81	18	10164	1.4378	0.075248
-	ENPP6	4	184078706	184252961	315	44	10164	1.4374	0.075299
+	C17orf99	17	78111040	78176904	144	26	10164	1.4371	0.075338
-	FBXO41	2	73244682	73319453	24	4	10164	1.436	0.075498
+	BCKDHA	19	41362789	41435005	109	3	10164	1.4345	0.075718
+	SCAMP5	15	74960535	75031495	220	22	10164	1.4345	0.075719
-	NHP2	5	178139463	178188960	100	13	10164	1.4339	0.075795
-	SAMSN1	21	14475228	14618402	357	32	10164	1.4339	0.075805
-	PPP1R18	6	30666389	30722895	353	26	10164	1.4338	0.075815
+	EIF3F	11	7952320	8006171	113	18	10164	1.4337	0.07583
-	SMU1	9	33031852	33111716	217	20	10164	1.4336	0.075838
+	C1orf106	1	200856074	200925736	207	40	10164	1.4335	0.07586
-	SLPI	20	45242239	45289564	149	3	10164	1.4328	0.075953
+	FCGR2C	1	161546339	161611220	33	4	10164	1.4319	0.076081
-	AURKA	20	56359389	56427295	82	19	10164	1.4315	0.076144
+	WDR18	19	949328	1004570	100	14	10164	1.4315	0.076146
+	C14orf177	14	98676613	98727766	206	22	10164	1.4313	0.07617
-	GFOD1	6	13347830	13522637	345	25	10164	1.4311	0.0762
-	DHX36	3	154265668	154359497	112	8	10164	1.431	0.07622
+	LOC105369970	12	45955062	46014827	68	9	10164	1.4305	0.076293
+	PARP2	14	20308614	20367910	202	19	10164	1.4302	0.076331
-	KRTAP26-1	21	30309131	30355289	245	16	10164	1.4299	0.07638
-	FIGNL2	12	51807892	51883760	50	6	10164	1.4297	0.076405
-	TMEM196	7	19709315	19808576	395	19	10164	1.4296	0.076419
+	PPP1R16A	8	144442987	144512121	88	6	10164	1.4295	0.076431
-	NDUFA4	7	10921953	10975186	164	21	10164	1.4295	0.076436
+	ZNF527	19	37336048	37403066	140	12	10164	1.4293	0.076459
+	FAM177B	1	222702216	222763727	35	4	10164	1.4281	0.076632
-	TMEM114	16	8516549	8625193	449	39	10164	1.4272	0.076754
+	TMEM199	17	28322581	28373683	54	5	10164	1.4272	0.076756
-	MED31	17	6633313	6686634	170	18	10164	1.426	0.076935
-	FAM188A	10	15768169	15895520	16	4	10164	1.4256	0.076997
-	NSMCE4A	10	121947088	122010228	245	22	10164	1.4255	0.077008
+	PNPLA2	11	783891	835573	65	8	10164	1.4243	0.077179
+	LMOD2	7	123620807	123674093	2	1	10164	1.4236	0.077278
-	KATNA1	6	149584873	149684018	157	4	10164	1.4233	0.077328
-	SEBOX	17	28354268	28400151	75	5	10164	1.4231	0.077356
-	SHFM1	7	96678767	96744891	121	13	10164	1.4228	0.077402
-	CYB561A3	11	61338748	61397299	5	1	10164	1.4216	0.077567
+	UBAP1	9	34144005	34262523	101	9	10164	1.4214	0.077601
-	P3H4	17	41791952	41847616	107	6	10164	1.421	0.077661
-	GMPPB	3	49711476	49758974	8	2	10164	1.4207	0.077701
-	L2HGDH	14	50232434	50347389	137	6	10164	1.4207	0.077704
+	ZNF430	19	20985620	21070055	186	11	10164	1.4206	0.07771
-	GSK3A	19	42220186	42277584	53	2	10164	1.4203	0.077764
-	VTN	17	28357277	28405352	75	5	10164	1.4203	0.077764
+	CLEC17A	19	14547914	14621144	24	5	10164	1.4199	0.077821
+	RAB3GAP1	2	135017265	135185584	277	14	10164	1.4196	0.077861
+	KIF2B	17	53787878	53835212	183	16	10164	1.4193	0.077908
-	WFDC8	20	45541152	45614350	377	17	10164	1.4183	0.078054
-	TMEM123	11	102386325	102488044	177	29	10164	1.4181	0.078088
-	LATS2	13	20963036	21096583	304	16	10164	1.4173	0.0782
-	TRAM2	6	52487402	52612064	427	37	10164	1.4171	0.07823

STRAND	SYMBOL	CHR	START	STOP	NSNPS	NPARAM	N	ZSTAT	P
+	ABCB8	7	150993422	151057782	132	30	10164	1.4169	0.078253
+	LETMD1	12	51013230	51077369	99	10	10164	1.4167	0.078284
-	ZNF404	19	43862363	43918964	126	7	10164	1.4167	0.078292
+	RPS6KB2	11	67393460	67445408	93	14	10164	1.4166	0.078294
-	KNCN	1	46535638	46586657	6	2	10164	1.4156	0.078441
+	MINOS1-NBL1	1	19561977	19668456	229	22	10164	1.4153	0.078494
+	RAB18	10	27469174	27552237	222	18	10164	1.4151	0.078523
+	GRIN3B	19	965438	1020223	193	25	10164	1.4146	0.078591
-	VPS37B	12	122855328	122931220	15	2	10164	1.4144	0.078622
-	PHF23	17	7225028	7274717	68	13	10164	1.4133	0.07878
-	MUTYH	1	45319242	45375470	136	15	10164	1.4133	0.078786
-	PPP2R3C	14	35075472	35157501	200	16	10164	1.4132	0.078799
+	ATP6V0A1	17	42423844	42532579	168	19	10164	1.4128	0.078853
-	GJA5	1	147746199	147816067	194	28	10164	1.4126	0.078883
+	ARL6IP4	12	122945060	122992913	3	1	10164	1.4125	0.078895
-	SLC2A14	12	7802512	7926196	298	40	10164	1.4125	0.078901
+	FAM229B	6	112052471	112112790	165	11	10164	1.4108	0.079152
+	SARM1	17	28336662	28411045	102	6	10164	1.4102	0.079245
+	CREB1	2	207494892	207615560	104	9	10164	1.41	0.079265
+	SET	9	128648432	128706396	38	5	10164	1.4088	0.079451
-	PTCHD3	10	27388188	27449368	253	21	10164	1.4087	0.07946
+	PTH2R	2	208324845	208504506	333	14	10164	1.4082	0.079529
+	WNT8A	5	138048892	138106730	69	4	10164	1.4074	0.079658
+	COL19A1	6	69831556	70222454	1150	72	10164	1.4074	0.079661
-	FBXW7	4	152311258	152571095	372	9	10164	1.4058	0.079898
+	HKR1	19	37299678	37374455	162	12	10164	1.4056	0.079919
+	MAK16	8	33450167	33511260	79	4	10164	1.4056	0.079923
-	PEX2	8	76970258	77036044	122	13	10164	1.4052	0.079974
+	IK	5	140612799	140672480	97	6	10164	1.405	0.080007
+	SPSB1	1	9257882	9379532	222	31	10164	1.4048	0.080042
+	NDUFAF5	20	13750026	13828421	60	8	10164	1.4036	0.080214
+	RAMP2	17	42726194	42773041	63	7	10164	1.4025	0.080376
+	ANKRD30B	18	14713160	14865465	168	3	10164	1.4025	0.080377
+	WDR19	4	39147189	39295810	266	11	10164	1.4025	0.080384
-	ZC3H3	8	143427655	143577245	494	33	10164	1.4014	0.080543
+	MAP3K1	5	56780073	56906152	378	26	10164	1.3994	0.080847
-	SCAF4	21	31661000	31767118	134	10	10164	1.3994	0.080848
-	GPANK1	6	31651229	31701283	245	54	10164	1.399	0.080901
-	CCDC73	11	32592080	32829658	370	10	10164	1.3987	0.080956
+	TRIM60	4	164990762	165051818	131	7	10164	1.3984	0.080994
+	LYRM5	12	25160216	25215015	113	8	10164	1.398	0.081056
-	SGTA	19	2744713	2818356	57	2	10164	1.3978	0.081083
+	NTM	11	131335361	132346822	2190	179	10164	1.3977	0.081096
+	SMIM4	3	52500986	52550570	90	10	10164	1.3974	0.081153
-	ERC2	3	55498308	56503366	2454	102	10164	1.397	0.081204
-	LIPT2	11	74480727	74528719	123	10	10164	1.3968	0.081231
+	LYZ	12	69313354	69364233	27	2	10164	1.3963	0.081317
-	CYP1B1	2	38057603	38111181	238	28	10164	1.3963	0.081319
+	MLNR	13	49185338	49232377	34	5	10164	1.395	0.081508
-	IFT20	17	28318325	28370489	55	5	10164	1.395	0.081511
-	PHOX2A	11	72229077	72279176	111	17	10164	1.3949	0.081521
-	LOC105369195	1	223941266	224018034	6	2	10164	1.3929	0.081819
-	OPALIN	10	96333216	96394365	95	9	10164	1.3905	0.082188
-	ZER1	9	128719786	128807422	66	6	10164	1.3903	0.082214
-	CDK5RAP1	20	33348839	33436902	112	10	10164	1.3895	0.082347
+	C15orf54	15	39215669	39264845	62	14	10164	1.3893	0.082369
-	TRABD2B	1	47750528	48032371	767	50	10164	1.3891	0.082406
+	LOC101927375	13	24506663	24577321	193	13	10164	1.389	0.082419
+	TNNT3	11	1884562	1948706	263	48	10164	1.3885	0.082485
+	MYL12A	18	3212482	3266237	116	19	10164	1.3882	0.082531
+	OTOA	16	21629155	21770729	112	10	10164	1.3878	0.0826
-	CCNB1IP1	14	20301368	20368312	222	20	10164	1.3877	0.082612
+	SELT	3	150568279	150640447	121	11	10164	1.3873	0.082678
-	TSC22D1	13	44422143	44611565	297	24	10164	1.3868	0.082759
+	LOC105370980	15	93171194	93216739	168	27	10164	1.3866	0.082785
-	DEFB116	20	31293212	31343585	3	1	10164	1.3864	0.082813
+	ZC3H18	16	88535379	88641964	165	11	10164	1.3864	0.082816
+	VSNL1	2	17505540	17666439	335	12	10164	1.3862	0.082839
-	CLECL1	12	9705860	9768299	191	11	10164	1.386	0.082879
+	ETNK1	12	22590142	22700674	179	16	10164	1.3859	0.082883
-	CNGA1	4	47925015	48051679	350	20	10164	1.3847	0.083071
-	ABCC11	16	48156910	48270177	162	4	10164	1.3845	0.083106
+	ITFG2	12	2777621	2846956	117	9	10164	1.3843	0.083127
-	HIST1H3B	6	26021589	26067060	146	23	10164	1.3843	0.083134

STRAND	SYMBOL	CHR	START	STOP	NSNPS	NPARAM	N	ZSTAT	P
-	ME1	6	83200389	83466741	708	25	10164	1.3842	0.083148
-	DNAJC30	7	73670918	73718451	65	4	10164	1.3841	0.083157
+	SNCAIP	5	122276349	122474099	267	21	10164	1.3837	0.083221
-	MTF1	1	37799567	37894624	2	1	10164	1.3826	0.083391
+	PTK7	6	43041268	43171720	273	29	10164	1.3821	0.083471
+	ARHGEF40	14	21035226	21100248	58	13	10164	1.382	0.083491
-	C10orf111	10	15085385	15132319	151	18	10164	1.3814	0.083584
+	SMIM17	19	56608159	56667246	89	11	10164	1.3812	0.083608
-	HIST1H3G	6	26260918	26306384	159	17	10164	1.3804	0.083729
+	USP18	22	18114959	18187397	10	2	10164	1.3792	0.08392
-	GZMH	14	24596480	24644774	168	27	10164	1.3788	0.083974
+	EIF4EBP3	5	140512666	140559578	39	6	10164	1.3787	0.084
-	RSU1	10	16580616	16852454	473	44	10164	1.3786	0.08401
+	HOXC11	12	53938126	53986419	43	7	10164	1.3786	0.084013
+	KDM2A	11	67084268	67268082	217	22	10164	1.3785	0.084032
+	LRSAM1	9	127416486	127513501	284	19	10164	1.3776	0.084165
-	IL36B	2	113012091	113087867	348	23	10164	1.3776	0.084168
-	DDX17	22	38473438	38541340	83	8	10164	1.3773	0.08421
+	HPR	16	72028226	72087246	126	14	10164	1.3765	0.084326
-	NT5C1A	1	39649121	39707038	137	22	10164	1.3758	0.084434
-	TXNL1	18	56592387	56673689	146	12	10164	1.3757	0.084457
+	CUL9	6	43147174	43234587	166	18	10164	1.3752	0.084527
+	CTNNA1	5	138718396	138945034	53	2	10164	1.3752	0.084534
+	ITPA	20	3173868	3237449	154	16	10164	1.3751	0.084547
-	ATP6V1G3	1	198513222	198575945	130	15	10164	1.3751	0.084557
+	TUBA8	22	18075687	18141731	17	2	10164	1.3749	0.084579
-	GOLGA3	12	132758909	132864118	111	9	10164	1.3745	0.084639
+	ZNF776	19	57711796	57768159	95	10	10164	1.3739	0.084735
+	PPIH	1	42622770	42691654	123	13	10164	1.3737	0.084775
+	C1S	12	7025676	7081032	1	1	10164	1.3732	0.084852
-	LPCAT3	12	6966185	7053538	1	1	10164	1.3732	0.084852
-	TRIM61	4	164944446	165012666	198	8	10164	1.3727	0.084917
+	LOC105375116	7	141197	190728	159	13	10164	1.3725	0.084961
-	KIAA0753	17	6568151	6676744	290	17	10164	1.3714	0.085123
+	PTGER4	5	40644498	40751764	303	17	10164	1.3713	0.085135
-	HIST1H2BB	6	26033227	26078657	153	21	10164	1.3708	0.08522
+	PNPO	17	47906523	47959308	127	17	10164	1.3698	0.085375
-	AMIGO3	3	49706834	49754805	9	2	10164	1.3691	0.085477
+	RIMBP3B	22	21348751	21399169	1	1	10164	1.3673	0.08576
+	FOXC1	6	1575446	1623897	100	29	10164	1.3665	0.085888
+	MT4	16	56530049	56578957	239	44	10164	1.3663	0.085926
-	NXPE4	11	114560591	114630840	149	15	10164	1.3654	0.086067
+	LOC729254	2	191757840	191830249	100	4	10164	1.3647	0.086181
+	RPP14	3	58271245	58330193	145	4	10164	1.3642	0.086258
+	GPR3	1	27357644	27405814	22	2	10164	1.364	0.086291
+	NSD1	5	177098025	177310213	262	14	10164	1.3638	0.086313
+	HIST1H4I	6	27104309	27149678	57	13	10164	1.3638	0.086318
+	GABARAPL1	12	10177841	10233128	183	23	10164	1.3637	0.086328
-	ANGPT2	8	6489651	6598263	681	68	10164	1.3635	0.086367
-	REPS1	6	138894015	139023282	379	19	10164	1.3632	0.086415
-	DKK3	11	11952996	12044370	349	69	10164	1.363	0.086447
+	POLD1	19	50349290	50428018	245	27	10164	1.3621	0.086579
-	PLA2G7	6	46694316	46770701	130	22	10164	1.362	0.086597
-	LAMB2	3	49111114	49168166	8	2	10164	1.36	0.086922
-	RAPH1	2	203423682	203570335	173	12	10164	1.3599	0.086936
-	CBR4	4	168884623	169045317	56	6	10164	1.3595	0.086991
-	FOXJ1	17	76126333	76176299	75	8	10164	1.3595	0.086995
-	TRMT10A	4	99536707	99599057	192	15	10164	1.359	0.087066
+	TMEM191C	22	21432140	21479940	17	2	10164	1.3587	0.087123
-	RPL12	9	127437674	127486432	150	12	10164	1.3585	0.087152
+	PLEKHG6	12	6275436	6338506	215	29	10164	1.3584	0.087163
-	SERPINA1	14	94366747	94425692	161	21	10164	1.3578	0.087267
+	IL17C	16	88603593	88650474	73	8	10164	1.3575	0.087316
+	PKD2L2	5	137854211	137952747	53	5	10164	1.3572	0.087354
-	MFI2	3	196977559	197064816	8	2	10164	1.3571	0.087369
+	IL23R	1	67103640	67269979	369	28	10164	1.3565	0.08747
-	DARS	2	135895881	136020684	67	4	10164	1.3556	0.087607
+	TUBB	6	30685201	30735426	319	25	10164	1.3555	0.08763
-	MSANTD4	11	105997902	106057254	109	12	10164	1.3552	0.087672
-	LOC105377621	4	102408707	102536891	257	34	10164	1.3545	0.087795
-	PTPRCAP	11	67425510	67472682	63	9	10164	1.3543	0.087815
-	PNMAL1	19	46456491	46506563	133	11	10164	1.3541	0.087859
-	AMT	3	49406778	49457678	68	8	10164	1.3531	0.088016
+	DLX6	7	96970978	97021040	6	2	10164	1.3525	0.0881

STRAND	SYMBOL	CHR	START	STOP	NSNPS	NPARAM	N	ZSTAT	P
+	IL1F10	2	113032970	113085850	273	26	10164	1.3525	0.088115
+	MC5R	18	13790544	13836862	204	16	10164	1.3523	0.088134
-	KRTAP13-2	21	30361391	30407239	135	22	10164	1.3518	0.088218
-	RBBP5	1	205076142	205157022	217	20	10164	1.3518	0.088219
-	NRM	6	30678047	30726420	287	31	10164	1.3516	0.088255
+	GDAP1	8	74314251	74377110	205	24	10164	1.3515	0.088271
-	CD14	5	140621728	140668701	83	6	10164	1.3514	0.088283
-	DYNC1LI1	3	32515971	32605874	167	18	10164	1.3511	0.088336
-	DAZL	3	16576792	16640499	254	25	10164	1.351	0.088349
+	GPR15	3	98496899	98543150	96	18	10164	1.3496	0.088564
+	AIP	11	67448029	67501108	64	14	10164	1.3496	0.088578
-	TDRP	8	447018	580781	476	34	10164	1.3493	0.088622
-	NOXRED1	14	77384021	77461011	184	11	10164	1.349	0.088674
+	PRAMEF20	1	13375450	13431354	4	1	10164	1.3489	0.088682
+	FAM178A	10	100877569	100975134	141	13	10164	1.3488	0.088697
+	PDPN	1	13548757	13627957	317	53	10164	1.3486	0.088728
-	CORO1B	11	67428047	67478821	71	11	10164	1.3479	0.088849
+	ARMT1	6	151417258	151480101	244	28	10164	1.3476	0.0889
+	KCNB2	8	72502391	72948349	941	38	10164	1.3468	0.089023
-	ATF7	12	53497856	53661415	313	16	10164	1.3464	0.08909
-	KLRG2	7	139416933	139518711	53	5	10164	1.3461	0.089143
+	TMCO6	5	140561540	140657248	144	10	10164	1.3456	0.089213
+	LRP1	12	57093499	57223359	40	5	10164	1.3456	0.089215
+	KRTAP13-1	21	30361074	30406822	136	22	10164	1.345	0.089314
-	KLHL11	17	41838518	41900431	97	4	10164	1.3448	0.08934
+	LY6G5B	6	31635951	31682450	263	55	10164	1.3447	0.089363
+	CABP4	11	67417415	67471774	80	12	10164	1.3444	0.089405
-	FCN3	1	27359110	27409825	21	2	10164	1.3441	0.089454
+	DGKZ	11	46297905	46390554	89	11	10164	1.3429	0.08965
+	IGF1R	15	98613539	98974530	1012	96	10164	1.3427	0.089683
-	HIST1H4B	6	26016896	26062252	154	24	10164	1.3422	0.089765
+	RAD50	5	132521924	132654621	306	22	10164	1.3419	0.089813
-	STAT6	12	57085404	57146433	41	5	10164	1.3416	0.089862
+	MFSD11	17	76701565	76814356	133	18	10164	1.3415	0.08988
+	GARNL3	9	127189471	127403549	474	40	10164	1.3415	0.089887
+	LRMP	12	25017101	25118335	260	33	10164	1.3405	0.090044
+	RAB11FIP3	16	390592	532485	10	4	10164	1.3401	0.09011
-	MDC1	6	30689807	30752966	412	31	10164	1.3397	0.090174
+	OR4A47	11	48448466	48499774	128	9	10164	1.3396	0.090191
-	INO80C	18	35458327	35532991	93	11	10164	1.3392	0.090257
+	RPL35A	3	197915181	197965851	137	12	10164	1.3384	0.090378
-	EXOSC5	19	41376370	41432351	93	2	10164	1.3373	0.090566
+	DNAH8	6	38680332	39040798	1543	66	10164	1.3373	0.09057
+	TMED2	12	123549529	123608144	1	1	10164	1.3367	0.090652
+	SLC22A5	5	132334704	132405614	286	26	10164	1.3367	0.090656
-	CYP4F2	19	15868024	15933120	260	11	10164	1.3358	0.090815
-	PDIA4	7	148993051	149063690	146	14	10164	1.3357	0.090826
-	EXOC2	6	475138	728141	877	59	10164	1.3357	0.09083
+	LRRC55	11	57146747	57201714	99	13	10164	1.3356	0.090841
+	TCEA2	20	64007350	64082347	73	7	10164	1.3355	0.090855
+	GPR37L1	1	202083705	202141425	199	28	10164	1.3349	0.090949
+	COPS7B	2	231746671	231819254	69	2	10164	1.3343	0.091057
+	SLC22A11	11	64520626	64582875	127	13	10164	1.3339	0.091125
+	RNASEH2A	19	12767054	12823648	5	1	10164	1.3336	0.091165
+	UCHL1	4	41221881	41278429	103	10	10164	1.3335	0.091187
+	METTL9	16	21562221	21667473	128	7	10164	1.3333	0.091219
+	RAE1	20	57316089	57388463	129	7	10164	1.333	0.091272
-	IP6K1	3	49714295	49821540	78	8	10164	1.3325	0.091343
-	GFRA3	5	138242380	138309564	80	7	10164	1.3321	0.091416
+	ZNF749	19	57393568	57457485	114	19	10164	1.332	0.091426
+	LOC105370059	12	126390958	126444206	156	10	10164	1.3316	0.091489
+	TXNDC17	17	6605902	6654541	119	8	10164	1.3315	0.091516
-	OR8B4	11	124413942	124459871	207	23	10164	1.3306	0.091655
-	CHRNA1	2	174737592	174799472	86	10	10164	1.3302	0.091725
-	OR52A1	11	5141431	5187369	271	15	10164	1.33	0.091752
-	AGMO	7	15149124	15597015	683	43	10164	1.3298	0.091785
-	C6orf47	6	31648298	31695772	236	54	10164	1.3292	0.091887
-	LOC105378579	10	119188557	119242578	163	16	10164	1.3292	0.091896
+	GRIK4	11	120476746	120998805	1083	107	10164	1.3291	0.091909
-	AQP3	9	33431154	33482633	54	10	10164	1.3289	0.091947
+	NIPAL1	4	47981774	48050173	230	20	10164	1.3286	0.091991
+	MRPS35	12	27675773	27766304	211	20	10164	1.3284	0.09202
-	NICN1	3	49412333	49464324	69	7	10164	1.3272	0.09222
-	FAM110C	2	28814	82115	5	2	10164	1.3267	0.092308

STRAND	SYMBOL	CHR	START	STOP	NSNPS	NPARAM	N	ZSTAT	P
-	HNRNPA0	5	137741384	137789350	18	2	10164	1.326	0.092426
-	KIF17	1	20651643	20753017	268	49	10164	1.326	0.092426
+	APBA2	15	28850994	29128315	461	19	10164	1.3258	0.092457
-	LRRTM2	5	138859346	138910368	5	1	10164	1.3256	0.092492
-	CASC1	12	25097057	25230162	280	12	10164	1.3241	0.092731
+	ZNF778	16	89167038	89242637	41	3	10164	1.324	0.092747
+	THOP1	19	2750508	2823601	56	2	10164	1.3236	0.092817
-	GOLGA1	9	124868294	124976114	136	11	10164	1.3232	0.092892
-	GDF3	12	7679785	7730764	94	17	10164	1.3225	0.092994
-	TRPM7	15	50547158	50721815	480	17	10164	1.3225	0.092997
+	CCDC50	3	191294082	191408670	436	25	10164	1.3224	0.093018
+	C10orf62	10	97554693	97600934	100	12	10164	1.3217	0.093128
-	MCM3AP	21	46225125	46321559	343	20	10164	1.3211	0.093241
+	LOC105376780	16	89197741	89244453	37	2	10164	1.3209	0.093261
-	LOC101927254	19	31139950	31267298	163	10	10164	1.3207	0.093307
+	KLHDC8B	3	49136120	49186486	19	3	10164	1.3206	0.093315
-	OTUD7A	15	31471682	31905685	771	16	10164	1.3205	0.09334
-	FBL	19	39824453	39881414	148	16	10164	1.3204	0.093351
+	PPP1R37	19	45058173	45157285	192	20	10164	1.3199	0.09344
+	CDKN2AIP	4	183409635	183459064	133	5	10164	1.3198	0.093458
-	PRDX2	19	12786820	12836910	9	1	10164	1.3193	0.093529
-	C2orf80	2	208155347	208225258	152	19	10164	1.3185	0.093664
-	TRAM1	8	70563218	70643459	79	5	10164	1.3178	0.093783
-	EYA3	1	27960344	28123675	212	17	10164	1.3176	0.093826
+	MAP3K4	6	160956727	161127385	380	32	10164	1.3175	0.093832
+	DPPA3	12	7676454	7727559	95	17	10164	1.3175	0.093839
-	SECISBP2L	15	48978638	49081563	117	9	10164	1.3174	0.093854
-	TUBGCP3	13	112475005	112641442	82	5	10164	1.3171	0.093903
+	TCTA	3	49377206	49426476	79	8	10164	1.3171	0.093909
-	JPH2	20	44101697	44222578	299	32	10164	1.317	0.093918
-	PPP2R5E	14	63362479	63578361	447	22	10164	1.3166	0.09398
-	PLIN3	19	4828334	4902768	114	15	10164	1.3165	0.094002
-	COMMD5	8	144827978	144888640	111	6	10164	1.3164	0.094025
-	SEMA3D	7	84985192	85221855	225	19	10164	1.3164	0.094027
+	DSG1	18	31283089	31369246	326	20	10164	1.3161	0.094076
+	TSEN54	17	75480707	75534739	103	9	10164	1.316	0.094095
-	TMEM134	11	67454348	67504277	58	13	10164	1.3156	0.094157
-	KCNE1	21	34408715	34547275	187	14	10164	1.3155	0.094178
+	ASIP	20	34151493	34279344	126	8	10164	1.3153	0.094198
-	C3orf84	3	49167539	49227181	23	2	10164	1.3149	0.094279
-	DEFB112	6	50033575	50083651	13	2	10164	1.3147	0.094312
+	NUB1	7	151306699	151388461	186	29	10164	1.3147	0.094313
-	DEFB110	6	49999138	50056981	10	2	10164	1.3139	0.09444
-	ADCY8	8	130770300	131076598	597	35	10164	1.3139	0.094447
+	AP2A2	11	890809	1022245	235	4	10164	1.3133	0.094539
+	DYNLL2	17	58048419	58100257	33	2	10164	1.313	0.09459
+	HIC2	22	21382404	21461463	11	1	10164	1.3129	0.094613
-	USP50	15	50483681	50582439	219	13	10164	1.3126	0.094654
-	RMND1	6	151394761	151487181	373	31	10164	1.312	0.094764
-	SAMD10	20	63964113	64015986	78	4	10164	1.3118	0.094795
-	MFAP2	1	16964500	17016643	69	6	10164	1.3117	0.094804
-	TIAM1	21	31108418	31594977	890	75	10164	1.3114	0.094858
-	CCDC71	3	49152535	49201352	21	3	10164	1.3112	0.094891
-	TMEM158	3	45214464	45261322	226	24	10164	1.3109	0.094944
+	TOE1	1	45304670	45353978	109	14	10164	1.3108	0.094969
+	SOCS6	18	70253901	70340199	231	25	10164	1.3101	0.095084
-	PAH	12	102828321	102952603	358	21	10164	1.3098	0.095135
-	ZRANB3	2	135186969	135566322	575	11	10164	1.309	0.095262
-	SLC6A20	3	45745449	45831561	165	43	10164	1.3087	0.095323
-	RHOA	3	49349136	49447093	136	10	10164	1.3084	0.09536
-	SH3GLB2	9	128997792	129063353	12	1	10164	1.3071	0.095595
+	FAM183A	1	43107979	43166396	39	1	10164	1.3068	0.095637
-	PXT1	6	36380551	36477889	179	11	10164	1.3068	0.095641
-	ATP9A	20	51586775	51803411	629	29	10164	1.3061	0.095756
-	THOC3	5	175928810	176003537	13	6	10164	1.3061	0.095764
-	CTXN1	19	7914496	7961166	92	6	10164	1.3059	0.09579
-	PPEF2	4	75849872	75937528	184	23	10164	1.3059	0.095797
+	PTTG2	4	37925435	37971010	106	20	10164	1.3054	0.095883
-	ZNF154	19	57686275	57744211	106	13	10164	1.3049	0.09597
-	OASL	12	121010292	121074242	197	31	10164	1.3047	0.096005
+	CTNNBL1	20	37658955	37882129	554	24	10164	1.3044	0.09604
-	BDH1	3	197499783	197608323	321	30	10164	1.304	0.096118
+	ZNF528	19	52362868	52428408	185	27	10164	1.3038	0.096158
-	LOC105373957	2	237475700	237522338	272	31	10164	1.3037	0.096161

STRAND	SYMBOL	CHR	START	STOP	NSNPS	NPARAM	N	ZSTAT	P
-	DDRGK1	20	3180366	3239649	154	17	10164	1.3033	0.096238
+	KIAA0141	5	141888785	141961465	144	15	10164	1.3031	0.096263
+	SLC25A31	4	127695375	127784299	95	7	10164	1.3031	0.096271
-	HFM1	1	91250766	91443007	495	35	10164	1.3026	0.096358
-	SORBS2	4	185575444	185991716	922	67	10164	1.3022	0.096417
-	PIWIL3	22	24709034	24809720	389	26	10164	1.3022	0.096424
-	EVA1A	2	75482318	75604722	316	42	10164	1.3018	0.096494
-	GRB14	2	164482406	164656850	301	30	10164	1.301	0.096626
-	CBX4	17	79823156	79874414	175	25	10164	1.3009	0.096648
-	DPF1	19	38201006	38264714	276	61	10164	1.3008	0.096665
+	RARB	3	25139332	25607932	1542	106	10164	1.3003	0.096753
+	HIST1H2AG	6	27098038	27143535	57	12	10164	1.3002	0.096759
-	RNF130	5	179910270	180107130	459	30	10164	1.3	0.096797
+	SLC6A6	3	14367576	14499349	209	33	10164	1.3	0.096805
+	B3GNT3	19	17759828	17823576	117	14	10164	1.2988	0.097
-	SLC22A31	16	89185756	89236671	38	2	10164	1.2986	0.097039
-	TNP1	2	216849458	216895059	165	30	10164	1.2984	0.097071
-	KRT24	17	40687991	40738750	87	10	10164	1.2982	0.097116
-	GRIK3	1	36785527	37069548	189	15	10164	1.2974	0.097245
+	ASRGL1	11	62302302	62411378	405	18	10164	1.2974	0.097248
+	STK11	19	1170799	1238435	130	17	10164	1.2973	0.097255
+	PHF14	7	10922892	11179828	613	22	10164	1.2972	0.097274
+	C5orf66	5	134998280	135354680	487	47	10164	1.297	0.097322
+	OR6C2	12	55417214	55463152	136	10	10164	1.2958	0.097528
-	PAIP2B	2	71172738	71262114	137	14	10164	1.2956	0.097557
+	AIG1	6	143024363	143350343	864	48	10164	1.2949	0.097676
-	ZBTB12	6	31889204	31937367	260	70	10164	1.294	0.097832
+	HIST1H1E	6	26121331	26167115	155	25	10164	1.2934	0.097939
+	FNIP2	4	158734030	158918050	192	9	10164	1.2934	0.097939
-	LGI3	8	22136830	22191831	31	6	10164	1.2933	0.097946
-	REEP4	8	22128020	22176935	31	6	10164	1.2931	0.097981
-	RGPD8	2	112358369	112468645	30	7	10164	1.293	0.098009
+	MAML1	5	179697850	179787286	87	5	10164	1.2929	0.098021
-	OPN1SW	7	128762489	128810790	48	4	10164	1.2929	0.098024
+	C7orf33	7	148555565	148625860	130	22	10164	1.2925	0.098095
+	LOC105369971	12	51812781	51863515	24	2	10164	1.2924	0.098109
+	B3GNT2	2	62161127	62234731	136	17	10164	1.2916	0.098244
-	BPNT1	1	220047482	220124927	152	20	10164	1.2915	0.098258
-	OLA1	2	174062447	174283637	385	10	10164	1.2914	0.09828
-	KLHL22	22	20431519	20530883	16	2	10164	1.2912	0.098319
-	GPR152	11	67441301	67487729	60	12	10164	1.2912	0.098323
+	RNF185	22	31125152	31217019	142	19	10164	1.2909	0.098372
+	ZNF548	19	57354850	57412551	91	9	10164	1.2908	0.098384
+	HIVEP1	6	11972653	12174999	626	45	10164	1.2901	0.098509
-	PRR14L	22	31671347	31785173	204	14	10164	1.289	0.098706
+	MYBPC2	19	50397903	50476326	275	50	10164	1.2876	0.098949
-	HIST1H2AB	6	26023092	26068568	147	23	10164	1.2874	0.098973
-	MAP3K6	1	27345179	27403014	30	4	10164	1.2871	0.099023
+	STXBP4	17	54933735	55174285	892	34	10164	1.287	0.09904
+	DMRTC2	19	41809743	41862330	80	3	10164	1.2868	0.099087
+	CALCOCO2	17	48795988	48875245	218	21	10164	1.2865	0.099143
-	ERF	19	42237561	42290164	49	1	10164	1.2861	0.099198
+	C17orf100	17	6616739	6663298	127	8	10164	1.2858	0.099262
-	PDHB	3	58417630	58468852	93	6	10164	1.2851	0.099376
-	CYP51A1	7	92102149	92169745	116	9	10164	1.2851	0.099387
-	LOC105378979	5	56760327	56808143	129	20	10164	1.2844	0.099492
+	PRPF6	20	63946069	64043100	131	7	10164	1.284	0.099579
-	MMP1	11	102779910	102833235	290	52	10164	1.2838	0.099606
-	ICA1	7	8103184	8297687	342	49	10164	1.2829	0.09976
+	OR6C3	12	55296701	55342636	125	13	10164	1.2827	0.099801
+	ZNF419	19	57452711	57504680	198	10	10164	1.2819	0.099938
+	SPACA7	13	112340881	112444695	260	14	10164	1.2813	0.10004
+	MED21	12	26987522	27040673	138	21	10164	1.2806	0.10017
-	TFPI2	7	93875397	93925991	16	4	10164	1.2792	0.10041
-	TNFSF10	3	172495508	172558507	235	28	10164	1.2779	0.10065
-	NSFL1C	20	1432162	1502720	195	14	10164	1.2774	0.10073
-	SCAF11	12	45909131	46027040	130	13	10164	1.2772	0.10076
+	RNF17	13	24712672	24899262	593	34	10164	1.2763	0.10092
+	HOXC4	12	53981852	54066030	75	10	10164	1.276	0.10098
+	IL1R1	2	102034638	102189874	523	58	10164	1.276	0.10099
+	TOR4A	9	137242828	137292641	88	5	10164	1.2754	0.10109
+	NUP85	17	75170450	75245759	138	9	10164	1.2753	0.1011
+	UTRN	6	144250298	144863034	1301	63	10164	1.2751	0.10114
-	LAMA1	18	6931744	7152814	289	36	10164	1.2743	0.10128

STRAND	SYMBOL	CHR	START	STOP	NSNPS	NPARAM	N	ZSTAT	P
+	MEPE	4	87786398	87856817	322	40	10164	1.2743	0.10129
+	PXK	3	58297890	58436127	284	10	10164	1.2742	0.1013
+	PDE4B	1	65757510	66384579	1396	79	10164	1.2736	0.1014
+	FMN2	1	240056885	240485189	1147	88	10164	1.2732	0.10148
+	CCDC36	3	49163428	49268157	39	2	10164	1.2729	0.10152
+	AP4E1	15	50873625	51015900	332	13	10164	1.2727	0.10155
+	NANS	9	98021677	98093083	119	12	10164	1.2725	0.1016
+	HIST1H3C	6	26010411	26055869	148	22	10164	1.2724	0.10162
-	MASP1	3	187206085	187327022	339	46	10164	1.2715	0.10178
+	FAM212A	3	49768233	49815033	59	6	10164	1.2711	0.10185
+	TCEB3	1	23708366	23772059	28	1	10164	1.2706	0.10194
+	LGALS7B	19	38754210	38801754	151	13	10164	1.2692	0.10218
+	PGAP2	11	3762724	3836371	40	6	10164	1.2691	0.1022
+	ARHGEF1	19	41848173	41917454	81	5	10164	1.2687	0.10228
-	C3orf62	3	49258597	49312075	30	3	10164	1.2683	0.10234
+	PARD6B	20	50696544	50763741	38	5	10164	1.2682	0.10236
-	ALDH1L1	3	126093561	126216526	467	28	10164	1.2682	0.10237
-	KCNC3	19	50301942	50368515	86	12	10164	1.268	0.10241
-	BRD9	5	853735	927824	148	8	10164	1.2678	0.10244
+	C4orf19	4	37418454	37603510	545	47	10164	1.2677	0.10245
+	FABP6	5	160152367	160248722	80	7	10164	1.2674	0.10251
+	NUTM1	15	34308251	34367737	130	4	10164	1.2673	0.10253
-	RAX2	19	3759089	3807221	21	4	10164	1.2667	0.10263
-	EPSTI1	13	42876388	43027271	436	51	10164	1.2659	0.10277
+	CCDC136	7	128756410	128832133	61	5	10164	1.2656	0.10284
+	OR2L13	1	247902029	248110922	377	18	10164	1.2653	0.10288
+	CARNS1	11	67380678	67435607	103	15	10164	1.2651	0.10292
+	TSN	2	121720545	121777853	59	4	10164	1.2646	0.10301
-	CBLN3	14	24416532	24464525	275	14	10164	1.2637	0.10316
-	NPIPA5	16	15353624	15416972	6	2	10164	1.2636	0.10319
-	SLC8A1	2	40102146	40547486	1325	66	10164	1.2635	0.1032
-	DYRK1B	19	39815347	39869233	153	14	10164	1.2634	0.10322
-	ATP13A3	3	194392674	194521654	27	3	10164	1.2632	0.10325
+	MCTP2	15	94196538	94493952	330	29	10164	1.2632	0.10326
+	LIPJ	10	88548277	88616976	173	13	10164	1.2631	0.10328
-	LOC105370295	13	24528602	24576913	131	5	10164	1.2628	0.10333
-	FBXL14	12	1555993	1629180	191	38	10164	1.2627	0.10336
-	INTS4	11	77868720	78029842	355	19	10164	1.2621	0.10346
+	LNP1	3	100366193	100466326	188	22	10164	1.2621	0.10346
-	USP4	3	49267144	49375103	90	7	10164	1.2618	0.1035
-	ASB2	14	93924153	94011786	169	29	10164	1.2616	0.10354
-	AHCY	20	34224840	34346976	143	8	10164	1.2611	0.10365
-	SAA4	11	18221355	18271837	117	17	10164	1.2608	0.10369
-	TIMM44	19	7916718	7978823	119	7	10164	1.2603	0.10379
-	TRAF1	9	120892393	120964173	172	11	10164	1.2587	0.10407
+	SETD5	3	9362700	9488154	246	16	10164	1.2587	0.10407
-	DNPH1	6	43215629	43264473	113	17	10164	1.2581	0.10418
-	NMU	4	55585229	55671698	227	17	10164	1.2579	0.10421
-	ZNF569	19	37400876	37504064	159	14	10164	1.2579	0.10421
-	C8B	1	56919210	57001140	261	20	10164	1.2579	0.10422
-	HOXB2	17	48532655	48580031	144	33	10164	1.2566	0.10445
+	CXCR6	3	45905399	45958354	114	10	10164	1.2563	0.1045
+	EMC10	19	50441457	50515909	248	35	10164	1.2559	0.10457
-	NIFK	2	121716945	121771927	66	3	10164	1.2554	0.10467
-	MTRNR2L3	20	57348440	57394822	82	6	10164	1.2552	0.10471
+	C2orf57	2	231557864	231604283	123	12	10164	1.2551	0.10473
+	F11	4	186230945	186299681	234	33	10164	1.2548	0.10477
+	CSNK2B	6	31630880	31680070	289	58	10164	1.2536	0.10499
-	APPL2	12	105163297	105271230	412	23	10164	1.2534	0.10503
+	CIC	19	42233537	42305796	63	2	10164	1.2532	0.10507
+	XRCC6BP1	12	57906541	57967269	135	10	10164	1.2529	0.10512
+	SFTPC	8	22126671	22174479	32	6	10164	1.2526	0.10518
+	TMC8	17	78093851	78152968	111	24	10164	1.2523	0.10523
+	SRD5A1	5	6598360	6679562	283	24	10164	1.2511	0.10544
+	OR9K2	12	55094769	55140776	207	3	10164	1.2509	0.10549
+	ZNF540	19	37516371	37624179	219	21	10164	1.2507	0.10553
+	LOC105373984	2	105107926	105155520	43	7	10164	1.2504	0.10558
-	ZNF117	7	64964452	65026036	58	3	10164	1.2492	0.1058
-	PMP2	8	81430326	81482484	36	2	10164	1.2491	0.10581
-	FCGBP	19	39853323	39969626	184	22	10164	1.2491	0.10582
-	TAAR5	6	132578592	132624738	226	34	10164	1.2489	0.10584
+	BBX	3	107487936	107821329	478	28	10164	1.2489	0.10585
-	TXN	9	110233812	110291640	102	17	10164	1.2486	0.10591
-	DENND4A	15	65649123	65827293	284	11	10164	1.2483	0.10597

STRAND	SYMBOL	CHR	START	STOP	NSNPS	NPARAM	N	ZSTAT	P
-	CCBE1	18	59420939	59732643	790	70	10164	1.2473	0.10614
+	DAAM1	14	59153611	59381405	610	30	10164	1.2471	0.10618
+	KHNYN	14	24388171	24451342	234	23	10164	1.2464	0.10631
-	CHEK2	22	28677743	28776905	275	38	10164	1.2462	0.10634
+	POLR2K	8	100115611	100164002	113	10	10164	1.2459	0.10639
+	LOC653602	2	14600091	14660819	69	11	10164	1.2455	0.10648
-	XKR6	8	10886045	11236381	1144	66	10164	1.2452	0.10652
+	GATAD1	7	92412448	92501328	165	21	10164	1.2451	0.10655
-	USP19	3	49098046	49155938	7	2	10164	1.245	0.10657
+	AHSA1	14	77422875	77479472	161	11	10164	1.2434	0.10687
+	BCL2L12	19	49630142	49683916	68	12	10164	1.2432	0.10689
-	IL2RB	22	37115838	37184922	110	21	10164	1.2428	0.10697
+	FZD2	17	44522444	44571262	62	13	10164	1.2426	0.10701
-	PTK2	8	140648382	141036313	506	14	10164	1.2425	0.10703
+	ZFPM1	16	88416431	88545166	71	4	10164	1.2416	0.1072
+	INPPL1	11	72188866	72249147	148	24	10164	1.2412	0.10727
-	MRPL51	12	6482150	6528305	182	15	10164	1.241	0.10729
-	LRRN2	1	204607170	204720733	291	40	10164	1.2401	0.10747
+	TRIOBP	22	37661988	37786556	174	15	10164	1.2395	0.10758
-	ILF2	1	153651788	153706028	27	2	10164	1.2391	0.10765
+	NPR1	1	153643617	153703992	27	2	10164	1.2391	0.10765
-	MAP3K19	2	134954290	135082444	276	22	10164	1.2389	0.10769
-	DDHD1	14	53026740	53188328	318	17	10164	1.2385	0.10776
-	SETSIP	1	92064436	92110472	10	5	10164	1.2381	0.10784
-	KRAS	12	25194789	25285931	215	20	10164	1.2376	0.10794
+	STAB1	3	52460338	52534495	82	11	10164	1.2373	0.10799
+	LTBR	12	6340368	6401571	216	53	10164	1.2369	0.10806
+	UBXN4	2	135706619	135795063	204	21	10164	1.2365	0.10813
-	NOTCH1	9	136484444	136580786	86	14	10164	1.2363	0.10817
-	DOLK	9	128935530	128982733	39	1	10164	1.2362	0.10818
+	PLA2G2F	1	20104326	20160386	89	24	10164	1.2361	0.1082
-	UCMA	10	13211766	13269328	184	32	10164	1.2361	0.10822
+	CNIH4	1	224321811	224389452	77	19	10164	1.2359	0.10825
-	GLO1	6	38665925	38738176	279	42	10164	1.235	0.10841
-	PPP1CA	11	67388181	67436905	95	14	10164	1.2346	0.10848
+	MAP2K4	17	11985818	12153831	355	23	10164	1.2343	0.10855
+	MRPS7	17	75226668	75276376	127	6	10164	1.2338	0.10865
-	SLC12A6	15	34219996	34373064	266	7	10164	1.2336	0.10867
+	NPY5R	4	163308868	163362403	162	29	10164	1.2334	0.10871
-	ABL2	1	179089327	179264693	398	19	10164	1.2334	0.10872
+	SRF	6	43136295	43191506	109	11	10164	1.233	0.10878
-	HIST1H1A	6	26007032	26052812	150	23	10164	1.2326	0.10886
+	MITF	3	69704435	69978337	188	23	10164	1.2318	0.10901
-	PRF1	10	70587348	70637775	133	18	10164	1.2317	0.10903
-	NKPD1	19	45139111	45197841	120	19	10164	1.2314	0.10908
-	ERV3-1	7	64980355	65041746	60	3	10164	1.2313	0.1091
-	ELP6	3	47485640	47548722	2	1	10164	1.2312	0.10912
-	VIPAS39	14	77416675	77492640	207	14	10164	1.2311	0.10914
+	NR1H2	19	50341423	50393028	120	14	10164	1.2308	0.10919
-	IQCG	3	197877208	197995015	281	20	10164	1.2307	0.10921
+	LOC105376909	19	43866660	43945219	202	14	10164	1.2307	0.10921
+	CCDC81	11	86339736	86433109	347	39	10164	1.23	0.10935
-	CLCF1	11	67354168	67409177	94	12	10164	1.2298	0.10939
-	GPR33	14	31472944	31522945	152	19	10164	1.2287	0.10959
-	JAK3	19	17814780	17883071	127	12	10164	1.2284	0.10965
+	SLC4A9	5	140323559	140385137	56	8	10164	1.2279	0.10975
+	COPZ1	12	54290090	54361851	89	9	10164	1.2274	0.10984
+	TRIP13	5	857854	929072	138	8	10164	1.2272	0.10987
+	CSTF1	20	56357371	56414526	83	17	10164	1.2271	0.10989
+	SPIB	19	50383938	50441053	202	31	10164	1.2266	0.10998
+	JUNB	19	12756496	12803311	1	1	10164	1.2266	0.11
-	HOOK2	19	12753003	12813484	1	1	10164	1.2266	0.11
-	YES1	18	711588	847326	464	24	10164	1.2253	0.11023
+	FCHO1	19	17712718	17798568	189	14	10164	1.2247	0.11034
+	RAD9A	11	67356952	67408412	88	12	10164	1.2246	0.11037
+	KIAA0754	1	39374504	39426482	41	4	10164	1.2239	0.11049
+	LOC646670	15	72602065	72659548	41	7	10164	1.2238	0.11052
+	SEPT11	4	76914742	77050154	255	23	10164	1.2235	0.11057
-	SERP1	3	150531993	150638177	177	12	10164	1.2235	0.11058
+	CDC123	10	12160962	12260590	268	26	10164	1.2234	0.11059
+	MINOS1	1	19561977	19639821	158	12	10164	1.2231	0.11064
-	GNG12	1	67691466	67868779	460	37	10164	1.2227	0.11071
-	GTSF1	12	54445952	54508602	108	8	10164	1.2223	0.1108
+	LOC105373990	2	121575402	121659344	142	4	10164	1.2217	0.11091

STRAND	SYMBOL	CHR	START	STOP	NSNPS	NPARAM	N	ZSTAT	P
-	EXOSC1	10	97425909	97481011	67	10	10164	1.2216	0.11092
+	ROPN1L	5	10406862	10492298	11	1	10164	1.2216	0.11092
-	PDZD7	10	100997683	101066157	135	32	10164	1.2215	0.11096
+	AADACL4	1	12609547	12677086	61	12	10164	1.2213	0.11099
+	SSH3	11	67268448	67322607	86	13	10164	1.2212	0.111
-	NFAM1	22	42370407	42472083	86	7	10164	1.2211	0.11102
+	PTDSS2	11	413271	501399	192	14	10164	1.221	0.11105
-	SERPINB9	6	2877265	2938312	167	24	10164	1.22	0.11123
+	ARHGEF4	2	130801916	131057253	263	30	10164	1.2199	0.11125
+	PRMT2	21	46600153	46675685	218	25	10164	1.2198	0.11127
-	GALNT18	11	11260874	11657045	1591	188	10164	1.2193	0.11136
+	SPP1	4	87940650	87993411	191	21	10164	1.2183	0.11155
+	RBM33	7	155609494	155791485	384	6	10164	1.2181	0.1116
+	KCNK15	20	44710847	44762313	66	7	10164	1.2179	0.11162
+	CD33	19	51180537	51250019	201	37	10164	1.2176	0.11168
-	DVL2	17	7215342	7269548	67	11	10164	1.2173	0.11175
+	ELP5	17	7217053	7269940	67	11	10164	1.2173	0.11175
+	PICK1	22	38021420	38085701	113	19	10164	1.2171	0.11178
-	NDUFA5	7	123526997	123636651	1	1	10164	1.2168	0.11184
+	ASB15	7	123531999	123647900	1	1	10164	1.2168	0.11184
+	GPR137C	14	52518148	52647713	366	33	10164	1.2167	0.11186
-	SPRY2	13	80325976	80375951	135	26	10164	1.2157	0.11205
-	RAB38	11	88103247	88210467	347	18	10164	1.2155	0.11208
-	OR8B8	11	124430150	124476085	187	20	10164	1.2155	0.11208
+	HN1L	16	1643277	1712072	116	9	10164	1.2155	0.11208
+	GNGT1	7	93871508	93921173	17	4	10164	1.2154	0.1121
+	CD36	7	80567188	80689277	1	1	10164	1.2153	0.11212
+	C15orf32	15	92436654	92511117	220	37	10164	1.2153	0.11213
-	MTCH2	11	47595204	47677654	55	4	10164	1.2151	0.11216
+	LEAP2	5	132838666	132884890	104	12	10164	1.2148	0.11223
+	BMP8A	1	39456634	39539869	86	7	10164	1.2146	0.11225
-	KRTAP25-1	21	30279145	30324514	208	21	10164	1.2145	0.11227
-	FUT6	19	5820626	5874753	87	13	10164	1.2144	0.1123
-	PLSCR5	3	146566608	146641216	249	17	10164	1.2144	0.11231
-	DCLRE1A	10	113824724	113889404	75	21	10164	1.2142	0.11234
-	FAM187B	19	35214801	35263725	197	24	10164	1.2126	0.11264
-	NCOR2	12	124314411	124602464	494	72	10164	1.2125	0.11266
+	MERTK	2	111863479	112049953	67	7	10164	1.2124	0.11269
-	PADI2	1	17056761	17154453	204	6	10164	1.2123	0.1127
+	PRMT1	19	49642152	49698450	76	14	10164	1.212	0.11276
+	SOX9	17	72086020	72136420	121	16	10164	1.212	0.11276
-	KRTAP21-2	21	30736951	30782202	61	2	10164	1.2115	0.11285
-	POU2F2	19	42076110	42167473	46	2	10164	1.211	0.11295
-	MMADHC	2	149559633	149622816	89	5	10164	1.2106	0.11303
+	NXNL2	9	88500073	88594466	28	3	10164	1.2098	0.11317
+	SLC35A4	5	140529565	140579104	44	6	10164	1.2097	0.1132
-	HPS5	11	18268670	18357498	131	10	10164	1.2095	0.11324
-	C17orf62	17	82432586	82485831	82	7	10164	1.2093	0.11328
-	OR10A3	11	7928576	7974520	153	18	10164	1.2088	0.11338
+	PCDHA1	5	140751136	141022344	393	10	10164	1.2084	0.11344
-	SCNN1A	12	6336843	6412357	248	60	10164	1.2084	0.11345
-	SNF8	17	48920096	48980414	176	11	10164	1.2079	0.11354
+	DAG1	3	49433703	49545618	130	9	10164	1.2076	0.1136
+	APOBEC3G	22	39042005	39097743	88	7	10164	1.2074	0.11363
-	IFNLR1	1	24144157	24222275	223	35	10164	1.2074	0.11364
-	LOC441239	7	64877751	64925100	142	8	10164	1.2074	0.11364
+	NR4A1	12	51987832	52069507	76	13	10164	1.2067	0.11377
-	KRTAP27-1	21	30327013	30372694	206	17	10164	1.2066	0.1138
+	CH17-360D5.1	10	47879012	47936069	7	1	10164	1.2061	0.11388
+	NUDT16	3	131346671	131398830	146	10	10164	1.205	0.11411
-	LOC105373985	2	110012300	110064293	7	2	10164	1.2049	0.11412
-	ADPGK	15	72741294	72818785	108	7	10164	1.2044	0.11421
+	RBPJL	20	45271824	45329677	176	9	10164	1.2043	0.11425
-	NAPSA	19	50348472	50404331	167	17	10164	1.2041	0.11427
-	RND3	2	150458193	150522695	131	16	10164	1.2033	0.11442
-	APMAP	20	24952925	25027974	164	6	10164	1.203	0.11449
-	DEFB124	20	31455506	31508013	26	2	10164	1.2027	0.11454
+	PRDM16	1	3033227	3448621	975	109	10164	1.2024	0.1146
+	EIF3M	11	32548767	32614498	97	10	10164	1.2019	0.1147
+	TRMT10C	3	101526836	101576446	108	10	10164	1.2016	0.11476
-	HBEGF	5	140322843	140381603	55	8	10164	1.2012	0.11484
-	CTSZ	20	58985187	59042254	12	2	10164	1.2011	0.11485
-	IL5	5	132531444	132591890	135	17	10164	1.2011	0.11485
-	MORN3	12	121641118	121707639	60	8	10164	1.2008	0.11491

STRAND	SYMBOL	CHR	START	STOP	NSNPS	NPARAM	N	ZSTAT	P
+	GOLGA6L4	15	84200773	84255368	3	2	10164	1.2003	0.11502
+	DHX38	16	72058665	72122912	171	13	10164	1.2	0.11506
+	LHFPL5	6	35762420	35834075	301	37	10164	1.1996	0.11514
+	OPTN	10	13065075	13148291	195	24	10164	1.1994	0.11518
+	C18orf21	18	35937625	35989287	86	4	10164	1.1992	0.11523
+	BMP3	4	80995622	81067531	123	9	10164	1.1991	0.11524
-	PRTG	15	55601540	55778649	242	8	10164	1.1987	0.11531
-	EIF2AK3	2	88546741	88662576	191	7	10164	1.1987	0.11532
+	DEFB118	20	31333618	31383923	28	2	10164	1.1981	0.11544
+	ZNF17	19	57376161	57431953	76	9	10164	1.1976	0.11553
-	ASIC2	17	33003087	34191806	2487	184	10164	1.1975	0.11556
+	LARP1B	4	128026266	128232931	251	6	10164	1.1962	0.11582
+	DIP2A	21	46423942	46594263	609	22	10164	1.196	0.11585
-	FAM170B	10	49121154	49169020	168	14	10164	1.1957	0.1159
+	HPDL	1	45291873	45338675	118	19	10164	1.1956	0.11592
+	SLC14A1	18	45688756	45762520	317	17	10164	1.1955	0.11596
+	C5orf58	5	170197552	170256231	171	26	10164	1.1943	0.11618
-	ZNF19	16	71464073	71524351	97	10	10164	1.1936	0.11632
+	SYNGR2	17	78133551	78182928	142	24	10164	1.1935	0.11633
+	SLC30A8	8	116915180	117186714	589	37	10164	1.1935	0.11633
-	WARS2	1	119021216	119175788	507	35	10164	1.1932	0.11639
-	ZSWIM2	2	186817480	186884170	204	12	10164	1.1928	0.11648
+	DUOXA2	15	45079325	45128106	62	4	10164	1.1928	0.11648
-	FOXH1	8	144463732	144511335	42	4	10164	1.1927	0.1165
-	VMO1	17	4775285	4821434	74	15	10164	1.1923	0.11658
-	BTBD17	17	74346416	74398652	62	13	10164	1.1917	0.11669
-	LOC105378249	12	51829120	51881959	29	3	10164	1.1916	0.11671
-	HIST1H1C	6	26045740	26091471	165	21	10164	1.1915	0.11672
+	PIWIL2	8	22240297	22367568	102	5	10164	1.1916	0.11672
+	NCAPD2	12	6459132	6541966	312	18	10164	1.1905	0.11693
+	C12orf75	12	105295636	105381518	297	30	10164	1.1905	0.11693
+	MPV17L	16	15360698	15419686	13	2	10164	1.1902	0.11699
+	FOXQ1	6	1277440	1324758	91	16	10164	1.1899	0.11704
+	RGS1	1	192540727	192590029	123	17	10164	1.1896	0.1171
-	ALAD	9	113376312	113436338	144	20	10164	1.1895	0.11712
+	MAPKAPK2	1	206649912	206744285	157	16	10164	1.1892	0.11719
-	NECAB3	20	33647087	33709458	86	16	10164	1.1892	0.11719
+	MLPH	2	237451410	237565318	608	36	10164	1.1881	0.1174
-	NT5DC2	3	52514369	52570077	95	8	10164	1.1875	0.11752
-	RPS6KA2	6	166399366	166897550	1189	104	10164	1.1859	0.11783
-	MCM9	6	118803449	118970162	390	9	10164	1.1859	0.11783
-	PRKDC	8	47763108	47995183	146	18	10164	1.1855	0.11791
-	KRTAP23-1	21	30338399	30383606	170	17	10164	1.1851	0.118
-	PRB2	12	11381540	11430564	16	5	10164	1.1848	0.11805
+	MAST4	5	66561348	67179598	1253	76	10164	1.1837	0.11827
-	LY6G5C	6	31666684	31715373	234	58	10164	1.1836	0.11829
+	PTPRA	20	2829195	3048669	513	24	10164	1.1835	0.11831
+	GRB7	17	39702909	39757285	45	7	10164	1.1834	0.11832
+	TRIM44	11	35627692	35821053	332	20	10164	1.1832	0.11836
-	SRA1	5	140540067	140593093	47	7	10164	1.183	0.1184
+	LTC4S	5	179758985	179806512	59	6	10164	1.1827	0.11846
+	CCNA1	13	36396030	36452882	24	1	10164	1.1826	0.11848
+	HIST1H2BD	6	26122843	26181349	185	29	10164	1.1819	0.11862
+	LRRN1	3	3764402	3859834	369	48	10164	1.1817	0.11866
-	ZNF772	19	57459586	57512570	234	11	10164	1.1817	0.11867
-	CASP5	11	104982633	105058221	71	13	10164	1.1816	0.11869
+	TDRD9	14	103893438	104062667	47	2	10164	1.1814	0.11871
-	MYCBP2	13	77034657	77362042	507	23	10164	1.1814	0.11872
+	TUBB1	20	58984254	59036654	11	2	10164	1.1811	0.11878
-	FAM71E1	19	50456643	50511746	187	30	10164	1.1804	0.11893
+	SLC25A34	1	15700088	15751392	134	12	10164	1.1802	0.11896
-	TFAP4	16	4247186	4308000	132	15	10164	1.1801	0.11898
+	FRY	13	31996300	32307498	664	80	10164	1.1799	0.11902
-	LOC285423	4	149140322	149850835	440	37	10164	1.1795	0.1191
-	TESC	12	117028923	117134446	79	14	10164	1.1791	0.11918
+	ZNF570	19	37432465	37495358	101	11	10164	1.1791	0.11918
+	SLC39A14	8	22332249	22444129	33	2	10164	1.1788	0.11924
+	HK1	10	69234984	69411882	418	50	10164	1.1786	0.11927
+	C3orf80	3	160188784	160300143	167	27	10164	1.1781	0.11937
-	EHHADH	3	185180624	185317886	239	22	10164	1.1778	0.11944
-	RPL35	9	124847879	124896961	63	7	10164	1.1778	0.11944
-	ZNF44	19	12237244	12329899	195	7	10164	1.1773	0.11953
+	C4BPB	1	207053850	207109993	75	10	10164	1.1766	0.11968
-	TXNL4B	16	72074857	72129316	151	11	10164	1.1762	0.11975

STRAND	SYMBOL	CHR	START	STOP	NSNPS	NPARAM	N	ZSTAT	P
-	TTC39B	9	15160844	15342360	438	47	10164	1.1762	0.11977
+	TMPRSS12	12	50807918	50897884	173	11	10164	1.1757	0.11985
+	HNRNPAB	5	178169507	178221183	123	26	10164	1.1756	0.11988
+	OTUD3	1	19846702	19922945	175	17	10164	1.1755	0.11989
-	SNRNP40	1	31249568	31331797	25	1	10164	1.1745	0.12009
-	VN1R1	19	57445174	57491739	169	10	10164	1.1742	0.12016
+	RBL2	16	53399439	53501648	171	10	10164	1.1741	0.12018
+	BNIP3L	8	26348007	26423129	89	15	10164	1.1736	0.12027
+	LOC105375205	7	27079130	27126665	103	21	10164	1.1729	0.12041
-	SMARCD3	7	151228973	151312149	171	19	10164	1.1729	0.12043
+	ADGB	6	146563965	146825462	247	28	10164	1.1728	0.12044
-	PSMD12	17	67330486	67401605	16	2	10164	1.1722	0.12056
-	KCNMB1	5	170368161	170424677	229	38	10164	1.1718	0.12063
+	JAM3	11	134033925	134161757	300	14	10164	1.1713	0.12073
-	TOP1MT	8	143299324	143394977	281	22	10164	1.1712	0.12076
+	PPP1R15A	19	48837392	48886062	122	14	10164	1.1711	0.12078
-	GDF9	5	132851181	132901884	107	12	10164	1.171	0.12081
-	KRTAP21-1	21	30745139	30790378	47	2	10164	1.1708	0.12083
+	PIKFYVE	2	208230753	208368751	265	11	10164	1.1706	0.12089
+	PTPRQ	12	80409274	80690189	325	11	10164	1.1705	0.12091
-	OBFC1	10	103860258	103953287	243	22	10164	1.1704	0.12092
+	MDK	11	46345784	46393837	33	4	10164	1.1699	0.12103
-	XRCC1	19	43533312	43610578	240	38	10164	1.1698	0.12104
-	APBB3	5	140548268	140599604	57	7	10164	1.1694	0.12112
-	BAG6	6	31629028	31695767	370	54	10164	1.1689	0.12122
+	AREG	4	74410098	74465009	162	12	10164	1.1685	0.1213
+	CCR9	3	45851504	45913177	126	15	10164	1.1684	0.12133
-	HELZ	17	67060438	67280963	216	10	10164	1.1675	0.1215
-	CERS3	15	100390395	100579786	919	36	10164	1.1668	0.12165
+	AP3M2	8	42117946	42181183	100	11	10164	1.1667	0.12166
-	C1orf52	1	85239953	85294672	165	23	10164	1.1664	0.12173
+	ABCF1	6	30536393	30601532	257	43	10164	1.1662	0.12176
+	PSME1	14	24101158	24148967	50	6	10164	1.1662	0.12177
-	LOC102724378	22	37719416	37782434	62	3	10164	1.166	0.12181
+	MARCH6	5	10318639	10450388	78	2	10164	1.1657	0.12186
+	CDK5RAP3	17	47932904	47991786	173	22	10164	1.1656	0.12188
+	PAX9	14	36622568	36687807	227	21	10164	1.1656	0.12189
-	ZNF883	9	112987120	113047192	69	9	10164	1.1656	0.12189
+	NMRK2	19	3898103	3952416	34	6	10164	1.1655	0.12191
-	GSDMC	8	129695903	129821888	439	28	10164	1.1645	0.1221
-	CRTAC1	10	97844711	98065828	294	24	10164	1.1644	0.12213
-	DEPDC1B	5	60586912	60735166	163	7	10164	1.1642	0.12217
+	FAM81A	15	59367276	59533552	446	49	10164	1.1638	0.12226
+	FITM1	14	24096466	24142849	50	6	10164	1.1638	0.12226
-	TBC1D28	17	18624434	18679433	37	4	10164	1.1638	0.12226
-	RFT1	3	53061152	53165459	251	18	10164	1.1637	0.12227
+	DTNA	18	34458290	34901844	507	38	10164	1.1632	0.12237
+	GABRA5	15	26831719	26959210	128	12	10164	1.1631	0.1224
+	CEMIP	15	80744343	80961662	455	77	10164	1.1628	0.12245
+	LGALS1	22	37640606	37689802	111	13	10164	1.1626	0.12249
-	UBXN7	3	196343490	196489318	131	14	10164	1.1626	0.12249
-	LOC105372109	18	49007679	49054771	65	14	10164	1.1625	0.12251
+	CSNK1G3	5	123477099	123627045	271	23	10164	1.1625	0.12252
-	FAM126A	7	22885848	23049195	355	12	10164	1.1619	0.12265
+	ZCCHC17	1	31261982	31375722	52	1	10164	1.1615	0.12272
+	CACNA1C	12	1935786	2707949	1364	123	10164	1.1601	0.123
-	ACLY	17	41856916	41965542	136	6	10164	1.159	0.12324
+	RBM38	20	57356398	57419333	68	8	10164	1.1587	0.12328
-	SEP15	1	86852445	86949424	169	4	10164	1.1586	0.12332
-	ZNF780A	19	40059152	40126736	176	4	10164	1.1584	0.12335
-	NDRG2	14	21006763	21105872	132	32	10164	1.1582	0.12339
-	P2RY14	3	151202115	151313628	227	20	10164	1.1581	0.12341
+	ITCH	20	34328235	34521393	156	4	10164	1.1581	0.12342
+	DCC	18	52305172	53545903	4104	102	10164	1.1579	0.12346
+	ZNF273	7	64835177	64942239	274	13	10164	1.1571	0.12362
+	ZNF761	19	53396974	53468261	45	5	10164	1.1569	0.12366
-	ANO9	11	407930	477011	105	11	10164	1.1559	0.12387
-	SNTB1	8	120525745	120847069	839	58	10164	1.1557	0.1239
+	OSBPL9	1	51581874	51799219	231	13	10164	1.1556	0.12391
-	CPA6	8	67412170	67781571	591	37	10164	1.1554	0.12397
+	KREMEN1	22	29038078	29178333	464	42	10164	1.155	0.12404
+	SLC17A8	12	100322079	100432059	96	13	10164	1.1543	0.12418
-	POLL	10	101568882	101623270	83	10	10164	1.1541	0.12424
+	FGF7	15	49388178	49497326	313	19	10164	1.1538	0.12428

STRAND	SYMBOL	CHR	START	STOP	NSNPS	NPARAM	N	ZSTAT	P
-	SUMO2	17	75157730	75218003	80	9	10164	1.1538	0.12429
-	RASD1	17	17484437	17531395	62	9	10164	1.1537	0.12432
-	DIAPH3	13	59655583	60198985	1300	32	10164	1.1534	0.12437
+	APLP2	11	130034821	130154811	215	12	10164	1.1529	0.12449
-	NOC3L	10	94304907	94397976	189	25	10164	1.1526	0.12453
-	RNF145	5	159147409	159245053	188	22	10164	1.1524	0.12459
+	KCTD6	3	58457096	58512360	57	6	10164	1.1519	0.12469
-	MED13L	12	115945771	116312203	548	41	10164	1.1515	0.12476
+	LAMA3	18	23654443	23965066	535	25	10164	1.1514	0.12478
-	VAMP1	12	6452237	6505899	227	15	10164	1.1514	0.12478
+	MYL4	17	47174013	47237697	84	14	10164	1.1513	0.12481
+	SQSTM1	5	179771388	179848078	166	25	10164	1.1511	0.12485
+	LDHAL6A	11	18420827	18489600	191	11	10164	1.1509	0.12488
+	TPRG1	3	189064459	189333482	269	28	10164	1.1509	0.12488
-	CDKL1	14	50319404	50432404	25	3	10164	1.1503	0.12502
-	MPDZ	9	13095296	13314662	82	3	10164	1.1496	0.12516
+	LDHA	11	18359389	18418218	142	12	10164	1.1486	0.12536
-	GPX1	3	49347176	49393358	53	5	10164	1.1482	0.12544
-	ZGLP1	19	10294803	10344557	117	13	10164	1.1477	0.12554
-	TNFAIP8L3	15	51046596	51140276	205	21	10164	1.1473	0.12563
-	MGAT4B	5	179787597	179841952	136	23	10164	1.1472	0.12566
-	HIST1H1D	6	26224212	26269988	154	19	10164	1.1469	0.12571
-	STX1A	7	73689205	73754702	40	6	10164	1.1464	0.12582
+	NDST1	5	150450823	150568211	137	12	10164	1.1458	0.12594
+	LOC105374986	6	26222024	26272753	180	21	10164	1.1457	0.12597
-	FLOT1	6	30717734	30777685	418	41	10164	1.1453	0.12603
+	TACC2	10	121954174	122264545	1104	118	10164	1.1451	0.12608
+	LOC100996637	2	459739	506631	90	11	10164	1.1451	0.12609
+	RPL5	1	92797037	92851924	79	7	10164	1.1448	0.12614
-	LOC105372204	18	76043684	76091412	232	24	10164	1.1439	0.12634
-	TREM1	6	41257385	41321719	177	22	10164	1.1431	0.1265
+	NPSR1	7	34623239	34888332	859	46	10164	1.143	0.12651
-	C9orf92	9	16193935	16311313	538	76	10164	1.1423	0.12666
-	HIP1	7	75523298	75773972	474	37	10164	1.1422	0.12668
-	PLA2G12A	4	109699989	109765086	156	32	10164	1.1422	0.12668
+	CLEC3B	3	44991267	45046071	120	8	10164	1.1419	0.12675
+	DERA	12	15876172	16047381	174	16	10164	1.1419	0.12675
-	C1orf141	1	67082176	67176645	248	19	10164	1.1413	0.12687
+	LSR	19	35213656	35277964	243	28	10164	1.1411	0.12691
+	HIST1H2AH	6	27112082	27157562	63	14	10164	1.1411	0.12691
-	LLPH	12	66113069	66165753	89	6	10164	1.1409	0.12696
-	SIGLEC14	19	51632553	51681879	133	14	10164	1.1408	0.12697
+	LOC105373551	2	110281888	110357069	10	2	10164	1.1407	0.12699
+	PLEKHM2	1	15649267	15744769	231	14	10164	1.1398	0.12719
-	USP31	16	23051406	23184270	304	13	10164	1.1393	0.12729
-	DUOX2	15	45082653	45149161	68	7	10164	1.139	0.12735
-	CSMD3	8	112212928	113472467	2184	48	10164	1.1383	0.12751
-	OMD	9	92404245	92459554	115	6	10164	1.1382	0.12752
+	CATSPER3	5	134932906	135021707	185	21	10164	1.1379	0.12758
-	ANXA8	10	47457993	48028768	44	2	10164	1.1376	0.12764
+	HS2ST1	1	86879652	87119998	178	5	10164	1.1368	0.1278
+	WNT2B	1	112431541	112531288	165	18	10164	1.1365	0.12788
+	SMC4	3	160364304	160444962	138	15	10164	1.1363	0.12792
+	C9orf43	9	113375644	113439684	153	21	10164	1.1359	0.12799
+	LOC105374860	2	38040387	38105569	291	34	10164	1.1355	0.12809
+	PGBD4	15	34067073	34114390	62	5	10164	1.1354	0.1281
-	IRF7	11	602553	650999	74	5	10164	1.1353	0.12813
-	LOC105376911	1	28667907	28716528	40	2	10164	1.1352	0.12814
+	UBTD1	10	97463882	97581210	230	16	10164	1.1351	0.12817
+	ZMIZ2	7	44713547	44779881	91	12	10164	1.1346	0.12827
+	CNN2	19	991275	1049068	322	26	10164	1.1343	0.12834
+	LOC101060022	1	227934793	227981378	277	18	10164	1.1337	0.12847
-	CRNKL1	20	20024361	20091046	164	14	10164	1.1334	0.12852
+	SDHAF1	19	35960188	36006318	24	4	10164	1.1334	0.12853
-	MAGI2	7	78007055	79488805	4989	283	10164	1.1329	0.12864
-	SPPL2A	15	50697533	50801158	309	16	10164	1.1324	0.12873
-	RHOG	11	3816978	3875983	89	12	10164	1.1323	0.12876
+	RBCK1	20	373065	440966	114	19	10164	1.1323	0.12876
-	WNT4	1	22107305	22178981	195	29	10164	1.1321	0.12879
+	VWA3A	16	22056914	22166966	5	1	10164	1.1319	0.12883
-	PKHD1	6	51604685	52122625	1495	62	10164	1.1312	0.12899
-	XCR1	3	46010799	46062487	184	10	10164	1.1308	0.12907
+	AQP8	16	25181916	25238932	71	10	10164	1.1306	0.12911
-	FABP9	8	81448383	81496523	46	2	10164	1.1302	0.12919

STRAND	SYMBOL	CHR	START	STOP	NSNPS	NPARAM	N	ZSTAT	P
-	SIRPB2	20	1460688	1526587	210	16	10164	1.1302	0.1292
-	JOSD2	19	50495997	50546355	167	39	10164	1.1293	0.12938
+	DAPP1	4	99781824	99882249	232	24	10164	1.129	0.12944
-	ELOVL2	6	10970759	11079391	302	21	10164	1.1288	0.12949
+	SYNGR1	22	39314949	39395588	235	27	10164	1.1288	0.1295
+	TANK	2	161101927	161246176	137	3	10164	1.1284	0.12957
+	RCAN3	1	24467351	24547020	112	11	10164	1.1283	0.1296
+	B3GNT7	2	231360624	231418801	29	3	10164	1.1276	0.12974
+	LOC105373926	2	231366613	231418342	29	3	10164	1.1276	0.12974
-	ZNF334	20	46471908	46548581	98	9	10164	1.1275	0.12976
-	SGSH	17	80196966	80255400	192	16	10164	1.1274	0.12978
+	SLC5A5	19	17836411	17905174	92	6	10164	1.127	0.12988
+	DRD4	11	602305	650706	74	5	10164	1.1264	0.13001
+	NUP188	9	128912693	129017096	95	4	10164	1.1262	0.13005
-	CBLB	3	105645461	105904552	711	29	10164	1.1259	0.1301
+	RXFP4	1	155906689	155952834	53	6	10164	1.1257	0.13014
+	ICAM4	19	10251967	10298584	78	14	10164	1.1249	0.13033
+	HP	16	72019609	72071056	113	13	10164	1.1247	0.13035
-	TTC5	14	20279131	20340994	200	21	10164	1.1247	0.13035
+	PCDHB16	5	141146690	141196221	147	10	10164	1.1245	0.1304
-	RNH1	11	484512	542283	237	14	10164	1.1244	0.13043
+	TMEM8B	9	35794225	35864847	102	11	10164	1.1243	0.13045
+	LHX4	1	180193803	180285053	175	22	10164	1.1241	0.13048
-	ITGA2B	17	44362181	44424505	54	5	10164	1.1239	0.13053
-	LZTFL1	3	45813316	45950724	254	23	10164	1.1237	0.13057
+	CGN	1	151475877	151548692	96	11	10164	1.1236	0.13059
+	LRRC10B	11	61473800	61521018	37	6	10164	1.1234	0.13064
-	KCNE3	11	74444841	74502557	129	13	10164	1.1231	0.13069
+	LIMD1	3	45559188	45691263	369	26	10164	1.1229	0.13074
-	OR56A1	11	6016671	6062741	105	14	10164	1.1224	0.13084
-	ATP5E	20	59018678	59067367	25	2	10164	1.1217	0.13099
+	NOL12	22	37651337	37703478	117	14	10164	1.1217	0.13099
-	TMEM170A	16	75433244	75499737	28	4	10164	1.1217	0.13099
-	TBC1D9B	5	179852071	179942856	157	16	10164	1.1211	0.13113
+	CCT7	2	73198425	73263022	78	5	10164	1.1208	0.13119
+	ZNF714	19	21047147	21135094	250	8	10164	1.1205	0.13125
+	ICAM5	19	10254435	10306778	89	15	10164	1.1204	0.13127
+	USO1	4	75689522	75824289	225	13	10164	1.1203	0.13129
+	MAST2	1	45751992	46046124	605	12	10164	1.12	0.13135
+	AIF1L	9	131061476	131133152	206	22	10164	1.1199	0.13137
-	ST3GAL2	16	70369435	70474088	1	1	10164	1.1197	0.13141
+	ARL11	13	49593299	49643872	200	21	10164	1.1197	0.13142
-	RRAS	19	49625295	49675143	64	12	10164	1.1195	0.13146
+	C2orf49	2	105302359	105369940	217	23	10164	1.1192	0.13154
+	LOC105376339	10	630959	676681	43	2	10164	1.1189	0.13159
-	TNIP1	5	151019943	151122660	374	43	10164	1.1188	0.13162
-	C9orf72	9	27536546	27608866	194	24	10164	1.1185	0.13167
+	UQCRQ	5	132831627	132878844	96	11	10164	1.1183	0.13172
-	DACT3	19	46637564	46696138	61	11	10164	1.1178	0.13184
+	TMEM37	2	119394912	119448520	133	17	10164	1.1175	0.13188
-	RPRM	2	153467338	153513808	100	19	10164	1.1173	0.13193
+	RNF31	14	24112450	24170661	60	9	10164	1.1167	0.13205
+	KLHL2	4	165172618	165333156	300	19	10164	1.1167	0.13206
-	SLC30A1	1	211565039	211613757	151	31	10164	1.1163	0.13215
+	TARS2	1	150452364	150517609	179	13	10164	1.1162	0.13217
+	MTRR	5	7816186	7911124	169	19	10164	1.1162	0.13218
-	JMJD6	17	76702832	76761799	78	12	10164	1.1155	0.13232
-	MKKS	20	10394780	10469239	308	14	10164	1.1148	0.13247
+	PCDHA2	5	140759859	141022344	352	9	10164	1.1145	0.13253
-	ANGPT4	20	861243	951375	198	38	10164	1.1137	0.1327
-	SSBP3	1	54215431	54448485	654	71	10164	1.1135	0.13274
+	RAC1	7	6339495	6413967	168	14	10164	1.1134	0.13277
+	TBC1D13	9	128752207	128820432	73	7	10164	1.1134	0.13277
-	SPC24	19	11135498	11191002	94	17	10164	1.1134	0.13277
+	CRABP1	15	78305324	78358230	115	26	10164	1.1133	0.13279
-	B4GALNT1	12	57615895	57668239	126	10	10164	1.1131	0.13284
+	PTPMT1	11	47530430	47583461	31	3	10164	1.1119	0.13309
+	CAPN5	11	77031945	77136155	39	5	10164	1.1114	0.13319
+	NARF	17	82423184	82498267	118	10	10164	1.1115	0.13319
+	MAP3K10	19	40156650	40225580	148	13	10164	1.1109	0.1333
-	TMBIM4	12	66126936	66205072	176	14	10164	1.1102	0.13346
+	APAF1	12	98610228	98745433	309	20	10164	1.1098	0.13354
+	FKBP10	17	41777262	41833217	80	7	10164	1.1088	0.13376
+	UTS2R	17	82339277	82385586	78	2	10164	1.1085	0.13382

STRAND	SYMBOL	CHR	START	STOP	NSNPS	NPARAM	N	ZSTAT	P
+	TNFAIP1	17	28300522	28357009	41	6	10164	1.1078	0.13398
-	DCTN2	12	57520050	57582331	83	12	10164	1.1073	0.13409
-	NUP155	5	37281633	37406455	55	4	10164	1.1072	0.13411
-	LOC101060181	19	12237244	12302038	144	6	10164	1.107	0.13415
-	ZBED8	5	160383148	160435097	51	9	10164	1.1062	0.13432
+	LOC101927503	11	1004200	1065749	56	7	10164	1.1062	0.13432
-	IKBIP	12	98603404	98680051	194	20	10164	1.106	0.13437
-	NSUN2	5	6589239	6668360	299	19	10164	1.1057	0.13444
-	CDHR5	11	606565	660067	83	6	10164	1.1055	0.13447
+	RAB10	2	25998860	26147454	152	2	10164	1.105	0.13457
+	RPL18A	19	17824878	17873324	79	7	10164	1.105	0.13459
-	GPATCH8	17	44385277	44538602	151	5	10164	1.1048	0.13463
+	ADM5	19	49653685	49700990	68	13	10164	1.1047	0.13465
+	SCNN1G	16	23147719	23226879	165	12	10164	1.1043	0.13474
+	ANKRD13D	11	67254291	67312484	89	15	10164	1.104	0.13479
+	PDXP	22	37623730	37676932	118	11	10164	1.104	0.1348
-	SFMBT1	3	52889982	53081655	480	25	10164	1.1039	0.13481
-	VPS33B	15	90988416	91057621	171	21	10164	1.1034	0.13493
-	DERL1	8	123003164	123077423	134	20	10164	1.1031	0.13499
-	MGARP	4	139256163	139315338	35	5	10164	1.1025	0.13512
-	C14orf2	14	103902288	103956566	13	1	10164	1.1021	0.13522
-	GGA3	17	75215480	75297393	223	9	10164	1.1019	0.13526
+	C5orf47	5	173954159	174016140	127	11	10164	1.1017	0.13529
+	IFNK	9	27489314	27536498	107	11	10164	1.1013	0.13539
-	NELFA	4	1972714	2076914	58	11	10164	1.1012	0.13541
+	POLR3F	20	18432389	18494648	216	21	10164	1.1011	0.13543
+	LOC101059906	11	493484	542263	193	13	10164	1.1009	0.13547
-	FAM221B	9	35807017	35868805	92	11	10164	1.0996	0.13575
+	HAMP	19	35247507	35295143	149	20	10164	1.0996	0.13576
+	PTTG1	5	160386807	160438744	51	8	10164	1.0991	0.13587
-	GPR157	1	9093391	9164170	147	21	10164	1.099	0.13589
+	BTRC	10	101319033	101567321	485	17	10164	1.0985	0.13599
+	SGK2	20	43523995	43595633	34	6	10164	1.0982	0.13606
+	BBS4	15	72651179	72748476	81	8	10164	1.0973	0.13626
+	QKI	6	163379643	163588596	81	8	10164	1.0973	0.13626
-	TMEM252	9	68520662	68575886	89	13	10164	1.0973	0.13626
-	ATG9B	7	151002209	151059499	128	29	10164	1.097	0.13632
+	LGALS9	17	27596148	27659560	127	7	10164	1.0968	0.13637
-	RIT1	1	155887808	155946402	73	7	10164	1.0967	0.13639
-	ZNF571	19	37554253	37629792	147	15	10164	1.0963	0.13647
+	NIPAL4	5	157425019	157484722	142	12	10164	1.0962	0.13649
+	ASCL1	12	102922674	102970516	108	16	10164	1.0959	0.13655
-	MYT1L	2	1779113	2366375	1224	85	10164	1.0959	0.13656
-	ANKRD23	2	96827914	96879021	54	9	10164	1.0958	0.13659
-	PIP4K2A	10	22524837	22749574	550	28	10164	1.0956	0.13662
-	FAM171A2	17	44343733	44398867	35	5	10164	1.0946	0.13684
-	IL22RA1	1	24109771	24178285	193	27	10164	1.0945	0.13687
+	CREB3L1	11	46242638	46331422	91	11	10164	1.0939	0.137
-	LRRC40	1	70134802	70240678	133	12	10164	1.0932	0.13715
-	IGF2BP3	7	23300209	23505527	322	31	10164	1.0929	0.13722
+	LONP2	16	48209167	48363979	210	4	10164	1.0928	0.13724
-	NSMAF	8	58573504	58694845	431	41	10164	1.092	0.13741
+	GADD45G	9	89570012	89616555	178	16	10164	1.092	0.13743
+	PCDHA3	5	140766198	141022344	341	9	10164	1.0919	0.13745
+	FFAR1	19	35316542	35362464	137	31	10164	1.0917	0.13749
+	HSPA1A	6	31780514	31827942	195	65	10164	1.091	0.13763
-	HOXB1	17	48519445	48565910	137	27	10164	1.0906	0.13772
+	DTWD1	15	49585980	49664448	91	10	10164	1.0902	0.13782
-	IFT80	3	160246986	160434532	272	23	10164	1.0902	0.13782
-	HEYL	1	39613431	39674676	120	19	10164	1.0898	0.13791
+	TKFC	11	61298182	61363139	32	1	10164	1.0896	0.13795
+	EFCAB5	17	29894856	30118452	162	5	10164	1.0889	0.1381
+	LOC105370815	15	51059593	51106596	106	12	10164	1.0888	0.13812
+	PSMB6	17	4761144	4808503	75	17	10164	1.0868	0.13857
+	RANBP1	22	20080938	20137357	152	13	10164	1.0866	0.1386
-	OR6C6	12	55284288	55330232	102	13	10164	1.0864	0.13866
+	OR14J1	6	29271690	29317655	242	31	10164	1.0862	0.1387
+	MAP1LC3B	16	87357195	87414774	57	14	10164	1.086	0.13874
+	GSE1	16	85135003	85686206	935	111	10164	1.0857	0.13881
-	CYCS	7	25108651	25160361	83	5	10164	1.0856	0.13882
+	POLR2M	15	57671521	57727557	99	16	10164	1.0851	0.13894
-	LOC105378950	1	39478834	39526993	42	4	10164	1.0848	0.139
-	ZC3H8	2	112205677	112290101	54	4	10164	1.0839	0.13921
-	PRELID2	5	145327932	145870383	1288	39	10164	1.0838	0.13922

STRAND	SYMBOL	CHR	START	STOP	NSNPS	NPARAM	N	ZSTAT	P
+	LOC101928596	1	169068700	169116473	104	14	10164	1.0836	0.13927
-	OGN	9	92373967	92439655	112	6	10164	1.0832	0.13936
-	OR6X1	11	123743580	123789518	123	11	10164	1.0831	0.13938
+	SUGCT	7	40099965	40870767	700	43	10164	1.0831	0.13939
+	PCDHB8	5	141142790	141190500	140	10	10164	1.0828	0.13944
+	ZNF596	8	197137	257340	130	13	10164	1.0828	0.13945
+	UPF3A	13	114246584	114315817	206	22	10164	1.0826	0.1395
-	JAZF1	7	27820573	28215818	1378	119	10164	1.0825	0.13951
-	PC	11	66838522	66993376	210	23	10164	1.0825	0.13953
+	LOC105373982	2	96834182	96880390	50	9	10164	1.0823	0.13955
-	FAM104A	17	73197353	73267394	196	7	10164	1.0823	0.13957
+	FAM218A	4	164921948	164969121	121	8	10164	1.0817	0.1397
+	CYP46A1	14	99649262	99737321	10	3	10164	1.0816	0.13971
+	PROSC	8	37727583	37789768	20	5	10164	1.0816	0.13971
+	CYP26C1	10	93026264	93078697	104	11	10164	1.0815	0.13973
-	PCDH8	13	52833974	52883640	86	11	10164	1.0813	0.13979
-	CENPJ	13	24872274	24957889	455	24	10164	1.0811	0.13983
+	NDFIP1	5	142073759	142164443	292	22	10164	1.0809	0.13986
+	C9orf135	9	69785800	69916237	357	32	10164	1.0808	0.13989
-	FLG2	1	152338735	152395010	102	7	10164	1.0808	0.13989
+	ACVR1B	12	51916667	52007079	120	17	10164	1.0807	0.13991
-	KRTAP21-3	21	30708525	30753777	179	4	10164	1.0802	0.14003
-	VTCN1	1	117133587	117305368	243	19	10164	1.0799	0.1401
-	TMEM105	17	81295814	81365674	98	6	10164	1.0797	0.14013
-	TMEM11	17	21187950	21249595	75	7	10164	1.0794	0.1402
-	SEMA3C	7	80732538	80954351	272	29	10164	1.0794	0.14021
-	GAREM	18	32253521	32505496	649	33	10164	1.0793	0.14022
-	UBE2S	19	55391282	55443146	147	26	10164	1.0791	0.14027
+	AKAP6	14	32294273	32843999	1610	141	10164	1.0787	0.14036
-	MED27	9	131850110	132114887	315	16	10164	1.0787	0.14037
-	TSTD2	9	97590080	97668680	212	7	10164	1.0785	0.14042
-	CD7	17	82304865	82352604	36	1	10164	1.0782	0.14046
-	ANGPT1	8	107239482	107533026	955	65	10164	1.0782	0.14048
+	COL20A1	20	63258169	63344847	89	6	10164	1.0777	0.14057
-	CCNL1	3	157136364	157195693	181	15	10164	1.0776	0.1406
+	CPSF6	12	69204537	69284358	26	1	10164	1.0761	0.14094
-	IFNW1	9	21130632	21177145	182	33	10164	1.0757	0.14104
-	ZNF563	19	12307477	12383130	50	3	10164	1.0755	0.14107
+	NRSN1	6	24091186	24157529	163	32	10164	1.0753	0.14111
+	MLLT4	6	167791922	167982023	123	4	10164	1.0748	0.14123
+	SLITRK5	13	87636232	87689616	42	4	10164	1.0745	0.14131
+	SACM1L	3	45654262	45755409	298	13	10164	1.0735	0.14152
+	PCDHB9	5	141152127	141201538	142	10	10164	1.073	0.14163
-	SLU7	5	160391641	160454161	62	11	10164	1.0724	0.14177
+	AMDHD1	12	95908293	95978592	314	19	10164	1.0724	0.14177
+	GNB3	12	6805854	6857395	45	6	10164	1.072	0.14186
-	CXCR4	2	136104349	136153155	52	6	10164	1.072	0.14187
+	PCDHA4	5	140772074	141022344	335	9	10164	1.0718	0.14191
-	HAND1	5	154464972	154513264	154	27	10164	1.0717	0.14193
+	CCDC140	2	222263147	222315217	66	9	10164	1.0708	0.14214
-	RD3L	14	103930426	103977308	18	1	10164	1.0703	0.14225
-	AGPAT4	6	161120020	161309075	565	81	10164	1.0702	0.14226
+	GSDMD	8	143518387	143573062	201	21	10164	1.0701	0.14228
-	FRRS1L	9	109126188	109202291	204	32	10164	1.0695	0.14242
-	DDB1	11	61289447	61368212	37	1	10164	1.0693	0.14247
-	PLA2G2D	1	20102650	20154566	86	24	10164	1.0693	0.14247
-	FAM25C	10	47985322	48034791	22	2	10164	1.0688	0.14258
+	OTUD1	10	23404269	23452381	56	11	10164	1.0687	0.14261
-	LOC101059914	4	164919491	164965626	118	7	10164	1.0685	0.14264
-	DEFB119	20	31367163	31425649	100	5	10164	1.0684	0.14267
+	SAMD15	14	77342419	77401244	141	15	10164	1.0682	0.14272
+	COMMD10	5	116048645	116422683	2023	47	10164	1.0681	0.14274
+	NIT2	3	100299718	100365634	120	17	10164	1.0679	0.14278
-	SCGB1D4	11	62286282	62334064	202	14	10164	1.0678	0.14281
-	FAM227B	15	49318393	49655931	868	24	10164	1.0676	0.14284
-	TMEM184A	7	1532235	1595821	30	6	10164	1.0677	0.14284
+	REG1A	2	79085458	79133419	174	28	10164	1.0676	0.14285
+	GCA	2	162283793	162389989	112	4	10164	1.0671	0.14296
-	TSPYL5	8	97263486	97312948	130	15	10164	1.0671	0.14296
-	NOP10	15	34331716	34378161	62	4	10164	1.067	0.14299
-	FAM166A	9	137233583	137286193	112	8	10164	1.067	0.14299
-	COL22A1	8	138578235	138949094	797	66	10164	1.0666	0.14308
+	TRIM42	3	140643024	140711150	2	1	10164	1.0665	0.14309
+	USF2	19	35233993	35289815	194	22	10164	1.0662	0.14316

STRAND	SYMBOL	CHR	START	STOP	NSNPS	NPARAM	N	ZSTAT	P
-	POLE3	9	113397235	113445749	84	7	10164	1.0662	0.14317
-	QARS	3	49085932	49140129	8	2	10164	1.0661	0.14319
-	PGC	6	41726711	41782401	110	23	10164	1.0659	0.14323
-	SECTM1	17	82311024	82369045	53	2	10164	1.0657	0.14329
+	TEX19	17	82324247	82373776	53	2	10164	1.0657	0.14329
+	NGDN	14	23434628	23488193	104	5	10164	1.0655	0.14333
+	OR10H2	19	15693024	15739052	257	14	10164	1.0651	0.14341
+	OPRD1	1	28777142	28873696	143	12	10164	1.065	0.14344
-	LSM2	6	31787392	31841984	246	73	10164	1.0644	0.14357
-	GPR135	14	59442773	59500396	181	11	10164	1.0643	0.14361
+	COMMD4	15	75300996	75351927	137	13	10164	1.0642	0.14362
-	MIEN1	17	39719156	39765541	36	6	10164	1.0638	0.14371
-	WDSUB1	2	159225793	159321793	352	17	10164	1.0638	0.14371
+	CD177	19	43318659	43373172	48	5	10164	1.0637	0.14374
+	CNNM3	2	96779952	96847466	83	8	10164	1.0635	0.14377
+	PTBP1	19	762392	822327	78	13	10164	1.0632	0.14385
-	RNF25	2	218653864	218707058	68	4	10164	1.0632	0.14385
+	PRKAB1	12	119632864	119691624	92	10	10164	1.063	0.14389
-	YRDC	1	37792942	37843193	3	2	10164	1.0626	0.14398
+	C1orf122	1	37772801	37819454	3	2	10164	1.0626	0.14398
-	EMC7	15	34074022	34136852	102	12	10164	1.062	0.14411
+	CCDC117	22	28737674	28799301	171	25	10164	1.0619	0.14415
-	FDX1L	19	10300211	10351015	110	13	10164	1.0617	0.14419
+	NDUFS7	19	1348884	1405589	2	1	10164	1.0613	0.14427
-	HOXA1	7	27082993	27131006	106	19	10164	1.0611	0.14432
-	OXER1	2	42752499	42799261	216	23	10164	1.061	0.14435
+	GPR142	17	74332407	74383008	97	19	10164	1.0608	0.1444
+	ARHGAP15	2	143035968	143778352	1421	104	10164	1.0606	0.14445
-	SLC1A5	19	46749698	46823649	123	14	10164	1.0603	0.14449
-	SLMO2	20	59023145	59077846	40	2	10164	1.0601	0.14455
+	WDR37	10	1021836	1142297	384	32	10164	1.0594	0.14472
+	RPE	2	209967565	210034599	39	3	10164	1.0591	0.14477
-	MAFA	8	143418060	143465432	182	18	10164	1.059	0.14481
+	METTL23	17	76690873	76743881	107	14	10164	1.0589	0.14483
+	SNTG2	2	915868	1377645	1724	51	10164	1.0588	0.14484
-	SAMD9L	7	93120054	93183393	105	13	10164	1.058	0.14503
+	MARS	12	57452953	57526655	134	15	10164	1.0579	0.14505
-	LOC105376847	17	76690190	76742304	107	14	10164	1.0574	0.14516
-	BBS2	16	56460389	56555096	325	28	10164	1.0572	0.1452
-	KCNA3	1	110643560	110710033	27	4	10164	1.0571	0.14524
-	KRTAP7-1	21	30819039	30864732	119	16	10164	1.0569	0.14528
+	GNL3	3	52650920	52704497	79	7	10164	1.0568	0.1453
+	C17orf80	17	73197233	73258959	183	6	10164	1.0564	0.1454
-	FKBPL	6	32118707	32165308	166	56	10164	1.0563	0.14541
+	BLZF1	1	169332956	169406544	152	10	10164	1.0563	0.14542
-	TGFBRAP1	2	105239404	105364714	391	33	10164	1.056	0.14547
-	ZBBX	3	167168425	167415405	623	22	10164	1.056	0.14547
+	HNF1A	12	120942787	121012512	243	32	10164	1.056	0.14548
-	GLT8D1	3	52684484	52741083	90	7	10164	1.0555	0.1456
-	C22orf31	22	29048672	29108966	241	27	10164	1.0547	0.14578
-	FCRL5	1	157503377	157587520	281	18	10164	1.0546	0.1458
-	CWC25	17	38790434	38860350	147	13	10164	1.0542	0.14589
-	LETM1	4	1801479	1891247	64	5	10164	1.054	0.14595
+	CLIP2	7	74254475	74415943	5	2	10164	1.0538	0.14598
-	LRRC34	3	169783006	169847866	164	20	10164	1.0529	0.1462
+	NDUFS3	11	47544010	47594563	31	4	10164	1.0525	0.14628
+	HIST1H2BE	6	26148796	26194230	157	20	10164	1.0524	0.1463
-	UHRF1BP1L	12	100026794	100177864	253	12	10164	1.0524	0.1463
+	NPY2R	4	155138754	155227096	205	10	10164	1.0515	0.14651
+	OR4D6	11	59421961	59467905	2	1	10164	1.0515	0.14651
+	NAA20	20	19982290	20043629	119	16	10164	1.0515	0.14652
-	C4orf27	4	169719468	169792942	27	1	10164	1.0515	0.14652
-	TOMM70A	3	100353459	100436398	153	17	10164	1.0514	0.14653
+	SUDS3	12	118338189	118428035	165	11	10164	1.0513	0.14656
+	FERMT3	11	64171655	64233891	153	22	10164	1.0513	0.14657
+	SNAPC1	14	61727357	61806428	263	27	10164	1.0511	0.14661
-	PBLD	10	68272660	68367928	215	12	10164	1.051	0.14662
+	OR5A1	11	59403057	59454188	2	1	10164	1.0507	0.14669
+	C6orf48	6	31799915	31849766	232	70	10164	1.0504	0.14678
+	MTTP	4	99529078	99633997	322	19	10164	1.0501	0.14683
+	HSPA1B	6	31792735	31840254	224	68	10164	1.0501	0.14684
-	DRICH1	22	23570872	23672723	226	14	10164	1.05	0.14685
-	PITPNM3	17	6440344	6591557	184	29	10164	1.0498	0.14691
+	LRRIQ4	3	169777947	169848007	169	20	10164	1.0494	0.14701

STRAND	SYMBOL	CHR	START	STOP	NSNPS	NPARAM	N	ZSTAT	P
-	LHX6	9	122175652	122263893	168	31	10164	1.0492	0.14705
+	ANXA4	2	69609100	69836464	712	42	10164	1.0489	0.14712
-	KCNN4	19	43756533	43817139	190	22	10164	1.0486	0.14718
-	CXCL3	4	74026589	74073773	41	13	10164	1.0485	0.14721
-	GSTA2	6	52740087	52798563	39	2	10164	1.0481	0.1473
-	TBC1D4	13	75274664	75517646	578	62	10164	1.048	0.14731
-	TMED8	14	77325021	77412053	185	18	10164	1.0479	0.14734
-	OR5A2	11	59411979	59457953	3	1	10164	1.0473	0.14748
+	OR10A7	12	55186025	55231975	139	5	10164	1.0473	0.14749
+	STIP1	11	64150272	64214548	153	21	10164	1.0472	0.1475
+	ASF1A	6	118859077	118919171	120	6	10164	1.0472	0.14751
-	KRTAP5-2	11	1587177	1633294	245	22	10164	1.0468	0.14759
-	TAF1A	1	222547902	222625096	56	7	10164	1.0468	0.1476
-	FAM180A	7	135719598	135783867	144	20	10164	1.0467	0.14762
+	HSD3B2	1	119379931	119433039	181	18	10164	1.0464	0.14768
-	TCL1A	14	95699967	95749196	129	11	10164	1.0461	0.14775
+	LOC105378386	10	80614552	80663734	44	6	10164	1.0459	0.1478
-	DYNLT1	6	158626474	158679786	81	11	10164	1.0456	0.14786
-	IRF3	19	49649569	49700875	74	14	10164	1.0455	0.14791
-	EVPL	17	75996845	76062452	88	12	10164	1.0451	0.14799
+	THEG5	19	31553189	31603550	25	1	10164	1.0448	0.14806
+	LOC105377771	5	181260347	181328407	121	9	10164	1.0447	0.14808
+	UVRAG	11	75780167	76154238	790	41	10164	1.0444	0.14815
-	LOC105377716	5	170632233	170716437	167	19	10164	1.044	0.14824
-	LOC105377623	4	143167737	143220297	4	1	10164	1.0438	0.14829
+	PRPF3	1	150286479	150363228	211	14	10164	1.0437	0.14831
+	SUMO4	6	149365359	149411046	113	9	10164	1.0437	0.14832
-	KIAA0907	1	155903043	155969442	81	7	10164	1.0429	0.14849
-	DDO	6	110382180	110450550	82	12	10164	1.0429	0.1485
+	APOBEC3F	22	39005604	39065972	89	7	10164	1.0425	0.1486
+	EAF2	3	121800187	121896526	239	14	10164	1.0422	0.14865
+	HADH	4	107954714	108045175	197	9	10164	1.0418	0.14876
+	KIAA1522	1	32706911	32784970	95	7	10164	1.0417	0.14877
+	NCKAP1L	12	54462711	54553115	157	19	10164	1.0412	0.1489
-	SCAP	3	47403694	47512126	5	2	10164	1.041	0.14895
-	LOC105370098	13	18932475	18979377	47	5	10164	1.0408	0.14899
+	FGD4	12	32364541	32656050	1111	59	10164	1.0403	0.14909
+	SCGB1D1	11	62155238	62203537	211	20	10164	1.0401	0.14914
-	CBY3	5	179668558	179715974	68	7	10164	1.0401	0.14914
+	CHRM5	15	33933245	34075094	234	18	10164	1.04	0.14917
+	SRSF11	1	70170682	70262020	112	10	10164	1.0398	0.14923
+	ZNF587B	19	57795294	57856238	91	7	10164	1.0393	0.14932
-	LPAR1	9	110863261	111074243	287	31	10164	1.039	0.14941
-	SLC7A4	22	21018718	21067558	78	13	10164	1.0389	0.14942
-	GLI3	7	41950949	42299112	625	57	10164	1.0388	0.14944
+	TTC25	17	41895617	41975651	93	6	10164	1.0388	0.14946
+	SAMD13	1	84239952	84360798	59	7	10164	1.0388	0.14946
+	MALSU1	7	23264321	23319561	94	7	10164	1.0387	0.14947
+	NOTO	2	73167258	73221212	100	6	10164	1.0382	0.1496
+	ZDHHC8	22	20096841	20158007	190	15	10164	1.0381	0.14961
+	IGF2BP1	17	48962412	49066145	171	23	10164	1.0381	0.14962
+	SLC5A9	1	48187270	48258644	201	17	10164	1.0376	0.14972
+	NDUFA9	12	4614098	4697554	273	28	10164	1.0367	0.14994
+	C5AR1	19	47272745	47332070	55	2	10164	1.0365	0.14999
-	SIN3A	15	75359379	75490815	157	16	10164	1.0361	0.15008
-	OR8B3	11	124386410	124434522	206	20	10164	1.036	0.15011
+	LOC102724668	7	32737427	32843146	343	15	10164	1.0352	0.15028
-	TOMM7	7	22802632	22857852	164	19	10164	1.0349	0.15037
+	BTBD8	1	92045305	92157840	26	10	10164	1.0346	0.15043
+	PDIA5	3	123032009	123172106	414	56	10164	1.0344	0.15047
+	JKAMP	14	59449443	59515407	203	11	10164	1.0342	0.15052
-	ZNF343	20	2470071	2559522	216	33	10164	1.034	0.15058
+	TCF7L1	2	85098460	85320388	609	48	10164	1.0335	0.15068
-	UGT2A3	4	68918459	68986798	75	5	10164	1.0334	0.15071
+	ISL2	15	76300942	76352476	104	14	10164	1.033	0.1508
-	PAQR9	3	142951231	142998336	1	1	10164	1.0328	0.15084
-	ZDHHC11B	5	700355	819749	11	3	10164	1.0328	0.15085
-	ZNF780B	19	40018260	40094027	142	6	10164	1.0328	0.15086
-	PCDH9	13	66292834	67265336	1938	78	10164	1.0327	0.15088
+	FGD5	3	14783962	14944565	485	23	10164	1.0324	0.15095
-	SMIM15	5	61147709	61197475	85	9	10164	1.0323	0.15096
-	CDKL3	5	134226120	134406469	239	6	10164	1.0322	0.151
-	RIMS4	20	44741804	44845338	56	12	10164	1.032	0.15103
+	MRS2	6	24367908	24436194	282	21	10164	1.0315	0.15115

STRAND	SYMBOL	CHR	START	STOP	NSNPS	NPARAM	N	ZSTAT	P
+	ANKRD42	11	83158739	83266099	204	22	10164	1.0315	0.15115
+	ERICH4	19	41408158	41454765	78	5	10164	1.0315	0.15116
+	PRKCD	3	53126207	53202717	149	20	10164	1.0312	0.15122
+	EIF5A	17	7271999	7322463	110	22	10164	1.0311	0.15125
-	LOC101929469	12	2838243	2892215	105	11	10164	1.0311	0.15125
-	CCDC181	1	169384870	169495669	223	18	10164	1.031	0.15127
+	CACNG1	17	67009536	67066797	77	15	10164	1.0309	0.1513
+	LOC105370296	13	39566324	39650412	126	20	10164	1.0306	0.15137
+	FAM180B	11	47551678	47599194	22	3	10164	1.0305	0.15138
-	NEGR1	1	71392942	72317847	1675	87	10164	1.03	0.15152
+	PSMB9	6	32819161	32869851	525	71	10164	1.0296	0.1516
+	CYR61	1	85545761	85593967	143	29	10164	1.0295	0.15162
-	PAWR	12	79577791	79726010	101	4	10164	1.0292	0.15169
+	APOBEC3B	22	38947399	39002779	75	13	10164	1.0291	0.15172
-	CYP26B1	2	72119238	72182862	80	10	10164	1.0288	0.15178
-	MAN2C1	15	75345792	75404114	61	10	10164	1.0288	0.15179
-	XIRP1	3	39173215	39227620	106	22	10164	1.0284	0.15187
-	MSRB1	16	1928233	1978293	73	13	10164	1.0281	0.15194
+	TMEM212	3	171808349	171877846	151	25	10164	1.0281	0.15196
-	SULT1E1	4	69831212	69895765	148	26	10164	1.0277	0.15204
-	FASTKD3	5	7849159	7904037	149	17	10164	1.0275	0.15208
+	OR6C74	12	55204762	55258226	144	6	10164	1.0275	0.15209
-	SEC63	6	107857756	107993278	196	14	10164	1.0269	0.15223
+	CMAS	12	22011174	22075674	24	1	10164	1.0269	0.15223
+	EXO1	1	241812967	241919165	415	39	10164	1.0268	0.15225
-	SPAG8	9	35797785	35847272	74	8	10164	1.0266	0.15232
-	PHOX2B	4	41734082	41783970	154	11	10164	1.0265	0.15233
+	GAPDH	12	6499405	6548375	153	19	10164	1.0264	0.15236
+	THSD4	15	71080628	71793383	1506	114	10164	1.0263	0.15236
+	S100A1	1	153593397	153642037	1	1	10164	1.0263	0.15237
+	CHTOP	1	153598982	153656306	1	1	10164	1.0263	0.15237
-	S100A14	1	153604255	153651332	1	1	10164	1.0263	0.15237
-	S100A16	1	153596886	153648038	1	1	10164	1.0263	0.15237
+	PRMT8	12	3346349	3603973	271	46	10164	1.0263	0.15239
+	FNDC3A	13	48940912	49219779	291	17	10164	1.0255	0.15256
+	ZNF470	19	56532081	56598903	104	13	10164	1.0253	0.15262
-	LOC101930023	12	92136489	92398858	190	21	10164	1.0252	0.15263
+	C11orf86	11	66940283	66987008	42	11	10164	1.025	0.15268
-	PIP4K2B	17	38755689	38834965	133	13	10164	1.025	0.15269
+	CWC27	5	64733918	65028763	529	20	10164	1.025	0.15269
-	PPAP2B	1	56484747	56614584	307	34	10164	1.0245	0.15279
-	PBRM1	3	52535352	52720898	310	9	10164	1.0239	0.15295
-	ITGB1	10	32890318	32993365	284	30	10164	1.0237	0.15298
+	RIN2	20	19722610	20012459	738	77	10164	1.0234	0.15306
-	NAIP	5	70958483	71060114	1	1	10164	1.0233	0.15309
-	SCAND1	20	35943617	35990359	78	9	10164	1.0226	0.15324
-	ABTB2	11	34140987	34393008	305	34	10164	1.0225	0.15327
+	OR6C1	12	55285600	55331538	104	13	10164	1.0223	0.15331
-	EPB41L4B	9	109161974	109355964	504	67	10164	1.0223	0.15332
+	APOM	6	31617410	31668210	306	49	10164	1.0223	0.15333
+	SCN8A	12	51555193	51822864	151	11	10164	1.0212	0.15357
-	FAM69A	1	92822729	92996522	107	6	10164	1.0205	0.15375
-	RUNX1	21	34777801	35084310	460	64	10164	1.0204	0.15376
+	PPFIA1	11	70235687	70394501	264	9	10164	1.0204	0.15377
-	CEP83	12	94255652	94494988	203	17	10164	1.0203	0.15378
+	GNAL	18	11654015	11895685	433	24	10164	1.0202	0.15382
-	BTBD2	19	1975438	2050703	181	27	10164	1.02	0.15388
-	ZNF280B	22	22474421	22544187	70	2	10164	1.0199	0.1539
-	ZFHX2	14	23510855	23590939	62	14	10164	1.0197	0.15394
+	LY6G6F	6	31671907	31720595	239	55	10164	1.0196	0.15397
+	EGFR	7	54984032	55217338	699	82	10164	1.0192	0.15406
-	EPS15	1	51344263	51554354	117	10	10164	1.0192	0.15407
+	STK36	2	218637026	218712717	92	5	10164	1.0189	0.15412
-	ANKRD60	20	58206126	58263653	51	7	10164	1.0183	0.15426
+	ARHGEF37	5	149516632	149644968	364	27	10164	1.0182	0.15429
+	ZNF678	1	227528519	227687443	522	24	10164	1.0177	0.1544
+	ARHGEF16	1	3419583	3491115	200	21	10164	1.0177	0.15442
-	AVPI1	10	97667424	97722258	139	18	10164	1.0173	0.1545
-	NOL10	2	10560766	10724987	621	21	10164	1.0173	0.15451
+	CCDC116	22	21597147	21647327	49	3	10164	1.0171	0.15455
-	PTGR1	9	111539722	111634855	308	32	10164	1.017	0.15458
-	CSRP2	12	76848715	76914046	85	9	10164	1.0169	0.1546
-	TMEM110	3	52826756	52932581	270	18	10164	1.0168	0.15463
+	GPC5	13	91363619	92877237	1969	87	10164	1.0166	0.15467

STRAND	SYMBOL	CHR	START	STOP	NSNPS	NPARAM	N	ZSTAT	P
+	GLRX3	10	130101375	130190382	254	27	10164	1.0165	0.15469
-	APIP	11	34872295	34951411	400	10	10164	1.0164	0.15472
-	HOXA4	7	27118507	27165780	99	11	10164	1.0164	0.15473
-	CCDC14	3	123877353	123996409	213	10	10164	1.0163	0.15474
+	NCBP1	9	97598423	97683748	231	7	10164	1.016	0.15481
-	PGPEP1L	15	98958230	99042795	302	21	10164	1.0159	0.15484
-	MRPL3	3	131452201	131538016	136	16	10164	1.0158	0.15486
-	NMB	15	84645129	84693571	94	13	10164	1.0155	0.15493
+	KIF5A	12	57515064	57594771	120	17	10164	1.0154	0.15496
+	LOC105376714	15	45413410	45479925	82	11	10164	1.0151	0.15504
+	RTFDC1	20	56433585	56528886	75	6	10164	1.0149	0.15507
-	PPAP2A	5	55414842	55570370	290	15	10164	1.0148	0.15509
+	MYOT	5	137832856	137897851	29	3	10164	1.0149	0.15509
-	MVD	16	88641935	88698157	80	19	10164	1.0147	0.15513
+	HMP19	5	174010604	174119179	30	6	10164	1.0145	0.15516
+	P3H3	12	6793373	6849854	69	10	10164	1.0145	0.15517
-	AGXT2	5	34988101	35083188	311	27	10164	1.0144	0.1552
+	ZC2HC1A	8	78631047	78729765	163	7	10164	1.0143	0.15523
+	ATIC	2	215276956	215359773	208	14	10164	1.014	0.15529
-	SIGIRR	11	395716	452454	80	10	10164	1.0134	0.15544
+	ECM1	1	150473011	150523789	127	11	10164	1.0133	0.15546
+	NUCB1	19	48865050	48933283	185	15	10164	1.0131	0.1555
-	SLC2A7	1	8950853	9061384	185	34	10164	1.0129	0.15555
-	ACBD7	10	15065475	15123776	182	20	10164	1.0128	0.15557
-	MUC6	11	1002824	1071706	80	9	10164	1.0127	0.1556
+	GNPAT	1	231206173	231287973	169	18	10164	1.0125	0.15565
-	MRPS25	3	15032251	15100311	113	8	10164	1.0122	0.15573
+	ATP6V0D2	8	86063910	86164225	263	13	10164	1.012	0.15578
-	LOC105373986	2	110031483	110090097	11	2	10164	1.012	0.15578
+	BHLHE40	3	4944412	4995181	58	3	10164	1.0119	0.15579
-	MMP3	11	102825797	102878611	194	34	10164	1.0117	0.15585
+	BCL9	1	147506401	147636219	230	30	10164	1.0115	0.15589
-	DZANK1	20	18373367	18502185	462	25	10164	1.0114	0.15592
+	IRAK3	12	66154198	66264622	215	24	10164	1.011	0.15601
-	MITD1	2	99151427	99216061	69	7	10164	1.011	0.15602
+	PRRC1	5	127482609	127565089	128	4	10164	1.0107	0.15609
-	SLC11A2	12	50969410	51063335	180	12	10164	1.0102	0.15619
-	GRM8	7	126428598	127288294	2240	79	10164	1.0102	0.1562
-	ZNF512B	20	63946704	64004870	88	6	10164	1.01	0.15626
+	CCDC138	2	108751750	108886769	145	7	10164	1.0098	0.15629
-	RAET1E	6	149854397	149933321	212	14	10164	1.0097	0.15632
+	PMAIP1	18	59864960	59914306	134	20	10164	1.0097	0.15633
-	CHTF8	16	69108009	69167590	81	13	10164	1.0096	0.15634
-	L3HYPDH	14	59455511	59519438	202	10	10164	1.0096	0.15635
-	CELF1	11	47455937	47600569	67	5	10164	1.0087	0.15657
-	IRS2	13	109743837	109821567	235	30	10164	1.0086	0.15659
-	PRADC1	2	73218006	73268228	45	4	10164	1.0084	0.15664
-	ORC1	1	52362829	52439471	40	3	10164	1.0083	0.15665
+	ANKFN1	17	55930604	56521407	827	33	10164	1.0081	0.15671
+	ATP5G1	17	48857786	48905871	128	13	10164	1.0073	0.1569
+	MED25	19	49783279	49850384	141	17	10164	1.0069	0.15699
-	TRAF6	11	36473767	36545313	195	31	10164	1.0066	0.15707
-	PKDREJ	22	46245663	46298322	94	2	10164	1.0066	0.15708
-	TSSK1B	5	113422554	113470031	191	16	10164	1.0062	0.15715
-	ZNF142	2	218627916	218694632	94	4	10164	1.0062	0.15716
-	TBC1D16	17	79922343	80070867	216	25	10164	1.0062	0.15716
-	TMEM246	9	101463158	101522221	183	21	10164	1.0061	0.15719
-	PITPNM2	12	122973480	123185810	24	4	10164	1.006	0.1572
+	FLYWCH1	16	2876921	2961208	314	21	10164	1.006	0.15721
-	TP53BP2	1	223769893	223880981	222	17	10164	1.0058	0.15724
-	GGACT	13	100520164	100623792	106	12	10164	1.0058	0.15727
+	C16orf52	16	21973135	22094654	18	2	10164	1.0052	0.1574
+	DMBT1	10	122525665	122653736	160	14	10164	1.0051	0.15743
+	DYRK3	1	206600534	206659197	89	16	10164	1.0048	0.1575
-	RFC2	7	74221485	74289458	1	1	10164	1.0047	0.15752
-	PREP	6	105267567	105438124	381	35	10164	1.0047	0.15753
+	DDX55	12	123567092	123630938	7	2	10164	1.0042	0.15764
-	APBB2	4	40800027	41251714	885	70	10164	1.004	0.15768
+	GUCA1A	6	42120386	42190056	64	12	10164	1.004	0.15769
+	CRELD2	22	49883630	49937540	286	15	10164	1.004	0.15769
-	MXRA7	17	76663650	76746005	167	18	10164	1.004	0.15769
-	GMEB2	20	63577602	63662028	293	30	10164	1.0038	0.15773
-	ATP5SL	19	41421318	41482827	117	12	10164	1.0037	0.15775
-	PRR34	22	46040459	46089144	18	4	10164	1.0035	0.15782

STRAND	SYMBOL	CHR	START	STOP	NSNPS	NPARAM	N	ZSTAT	P
+	TMEM69	1	45653175	45704436	125	9	10164	1.003	0.15794
-	FUZ	19	49796866	49852376	112	14	10164	1.0029	0.15796
+	ARHGEF25	12	57575180	57627245	94	14	10164	1.0022	0.15812
-	ITGA11	15	68287428	68467160	404	31	10164	1.002	0.15816
+	FAM209A	20	56489729	56536152	37	4	10164	1.002	0.15817
+	CNTNAP5	2	123990287	124931201	2386	126	10164	1.0018	0.15822
+	ZMYM2	13	19922432	20101845	128	4	10164	1.0011	0.15839
-	LOXHD1	18	46466961	46692114	746	74	10164	1.001	0.15842
+	LOC285500	4	177409513	177687123	810	55	10164	1.0009	0.15844
+	DPCD	10	101553332	101619653	115	13	10164	1.0008	0.15847
+	LOC105373989	2	120293666	120356060	109	26	10164	1.0008	0.15847
-	EIF3A	10	119025029	119115822	170	12	10164	1.0007	0.15849
-	TFEB	6	41673978	41771259	278	43	10164	1.0003	0.15859
+	PCDHB11	5	141164775	141213045	137	10	10164	0.99989	0.15868
+	EIF3J	15	44502068	44572803	4	2	10164	0.99984	0.15869
+	GPR88	1	100503172	100552027	117	15	10164	0.99966	0.15874
-	SDR39U1	14	24429766	24477905	376	14	10164	0.99956	0.15876
-	WDR73	15	84631495	84689343	105	13	10164	0.99934	0.15882
+	ZNF586	19	57734652	57790616	90	8	10164	0.99928	0.15883
+	MRPL30	2	99146079	99209557	75	7	10164	0.99903	0.15889
+	LFNG	7	2477529	2539177	69	10	10164	0.99868	0.15898
-	GATM	15	45351124	45432043	182	9	10164	0.99852	0.15901
-	TTN	2	178515989	178865754	515	34	10164	0.99847	0.15903
+	ZNF365	10	62339157	62682011	1256	111	10164	0.99836	0.15905
+	DAPL1	2	158760317	158837457	148	15	10164	0.99838	0.15905
+	CANX	5	179643646	179741641	144	18	10164	0.99819	0.15909
+	MRTO4	1	19216581	19270128	67	5	10164	0.99804	0.15913
-	DPH3	3	16247061	16299989	165	14	10164	0.9979	0.15916
+	ZNF383	19	37182926	37253926	116	14	10164	0.9978	0.15919
+	RPL27A	11	8647448	8699872	60	6	10164	0.99777	0.1592
+	ICAM1	19	10235841	10296615	101	18	10164	0.99771	0.15921
+	DSE	6	116245120	116453475	511	33	10164	0.99755	0.15925
+	PHRF1	11	541220	622222	201	19	10164	0.99744	0.15928
-	ZNF585B	19	37171579	37245549	110	13	10164	0.99725	0.15932
-	GPBP1L1	1	45617304	45723110	275	13	10164	0.9971	0.15936
+	NELFB	9	137220307	137283548	120	9	10164	0.99694	0.1594
-	ULK3	15	74826116	74878211	119	27	10164	0.99615	0.15959
+	C1orf94	1	34131883	34229131	227	25	10164	0.99615	0.15959
-	PIK3R3	1	46030140	46211495	387	25	10164	0.99598	0.15963
+	PIP4K2C	12	57556159	57613428	97	13	10164	0.99581	0.15967
+	NT5DC4	2	112685810	112752879	122	13	10164	0.99575	0.15969
-	CORIN	4	47583998	47873106	593	23	10164	0.99569	0.1597
-	MCOLN2	1	84915583	85032122	252	15	10164	0.99537	0.15978
-	TRIM66	11	8602037	8717703	150	13	10164	0.9952	0.15982
-	TRIML2	4	188081255	188144603	46	3	10164	0.99483	0.15991
+	TRIML1	4	188103189	188157743	46	3	10164	0.99483	0.15991
-	ADCY9	16	3943387	4151185	429	45	10164	0.99473	0.15993
-	PCGF6	10	103292796	103386134	139	19	10164	0.99471	0.15994
-	ZBTB2	6	151354115	151426700	270	40	10164	0.99451	0.15999
+	TMEM108	3	133003288	133407775	1060	36	10164	0.99445	0.16
+	FBLN7	2	112103385	112198218	121	13	10164	0.99416	0.16007
-	CDC42EP3	2	37631882	37707535	236	23	10164	0.99392	0.16013
+	ADRBK1	11	67231400	67296558	77	13	10164	0.99368	0.16019
-	SLC26A9	1	205903048	205978472	150	20	10164	0.99349	0.16024
+	DNAJC10	2	182681041	182790025	154	8	10164	0.99308	0.16034
-	KRTAP10-3	21	44548023	44593760	212	17	10164	0.99291	0.16038
+	PELO	5	52752940	52812617	250	28	10164	0.99279	0.16041
+	RGS3	9	113409729	113607743	163	8	10164	0.99271	0.16042
-	CRYGA	2	208150740	208198573	136	16	10164	0.99265	0.16044
-	NT5C	17	75120225	75166795	35	6	10164	0.99251	0.16047
+	FFAR3	19	35323209	35370489	136	32	10164	0.9925	0.16048
+	ZNF576	19	43561392	43610435	138	28	10164	0.99229	0.16053
-	TMEM110-MUST	3	52823115	52932581	286	18	10164	0.99208	0.16058
-	C19orf18	19	57948437	58009534	142	8	10164	0.99136	0.16075
-	TYW5	2	199918911	199990736	126	18	10164	0.99136	0.16076
-	GFPT1	2	69309769	69422254	257	13	10164	0.99087	0.16087
+	PCDHB10	5	141157379	141205640	146	10	10164	0.99039	0.16099
+	PRPF38A	1	52369547	52428320	33	3	10164	0.99008	0.16107
-	LRRC38	1	13464977	13548781	356	50	10164	0.99005	0.16108
-	KIAA1755	20	38200505	38295772	182	24	10164	0.98999	0.16109
+	MAPK8IP3	16	1671183	1780317	194	18	10164	0.98941	0.16123
-	UBE2W	8	73770097	73913910	146	4	10164	0.98928	0.16126
+	EIF2A	3	150511690	150596016	122	10	10164	0.98912	0.1613
+	PHYHIPL	10	59138371	59257774	58	2	10164	0.98914	0.1613

STRAND	SYMBOL	CHR	START	STOP	NSNPS	NPARAM	N	ZSTAT	P
-	OR13A1	10	45292294	45350608	325	11	10164	0.98894	0.16135
+	ZNF773	19	57464854	57528437	325	9	10164	0.9888	0.16138
+	GIMAP8	7	150415630	150489395	126	9	10164	0.98848	0.16146
+	CHST2	3	143084776	143134014	7	1	10164	0.98776	0.16163
+	LRRC56	11	484000	564916	332	15	10164	0.98773	0.16164
+	PCDHB7	5	141137621	141186376	132	10	10164	0.9876	0.16167
+	UNC5D	8	35200457	35806550	272	16	10164	0.98744	0.16171
+	MTF2	1	93044235	93149081	47	6	10164	0.98737	0.16173
-	ERCC3	2	127247290	127329176	37	3	10164	0.98679	0.16187
-	LIN7A	12	80782520	80972923	467	27	10164	0.98673	0.16189
-	FBXO48	2	68452366	68502316	88	2	10164	0.98668	0.1619
+	LOC105378684	1	42944698	42995699	170	15	10164	0.98648	0.16195
+	FAM53C	5	138302535	138359729	91	9	10164	0.9863	0.16199
+	TCAF2	7	143585952	143740080	32	5	10164	0.9861	0.16204
+	SMPD1	11	6355301	6404998	46	6	10164	0.98595	0.16208
+	NPR2	9	35717945	35819731	123	17	10164	0.9859	0.16209
+	TMEM175	4	897387	968656	115	16	10164	0.98591	0.16209
+	LMNB1	5	126741623	126847020	65	5	10164	0.98553	0.16218
-	RABIF	1	202868282	202924257	147	13	10164	0.98529	0.16224
-	SEC16A	9	136430096	136519549	182	16	10164	0.98529	0.16224
-	CNGB3	8	86563843	86778675	650	44	10164	0.98513	0.16228
+	TBKBP1	17	47659065	47722063	93	13	10164	0.98492	0.16233
-	CDHR4	3	49780732	49838164	66	7	10164	0.98478	0.16237
-	SYT6	1	114079292	114188850	244	40	10164	0.98466	0.1624
-	HOXA2	7	27090354	27137775	96	14	10164	0.98456	0.16242
-	PRR23C	3	139032102	139079892	78	5	10164	0.98422	0.1625
-	PITRM1	10	3127727	3207841	332	18	10164	0.9842	0.16251
-	TRMT2A	22	20101866	20152295	158	12	10164	0.98411	0.16253
+	C4BPA	1	207069238	207154972	81	11	10164	0.98327	0.16274
-	SLC2A2	3	170985365	171061979	122	7	10164	0.98325	0.16274
-	CBLN2	18	72525898	72673477	6	3	10164	0.9832	0.16275
-	GABRB3	15	26533546	26808788	503	42	10164	0.983	0.1628
-	POU2AF1	11	111342251	111414432	247	21	10164	0.98273	0.16287
+	SPCS1	3	52670841	52718182	72	6	10164	0.98243	0.16294
+	UBE2Z	17	48873369	48939060	189	12	10164	0.98245	0.16294
+	MPZL1	1	167686950	167801919	185	16	10164	0.98188	0.16308
+	LARP4	12	50365809	50490005	124	4	10164	0.9817	0.16312
+	FOLR2	11	72181775	72231950	128	20	10164	0.98166	0.16313
-	RNASE1	14	20791356	20837877	46	10	10164	0.98169	0.16313
-	FERMT2	14	52847271	52986097	355	26	10164	0.98144	0.16319
-	SERPINI2	3	167431789	167509132	113	5	10164	0.98128	0.16323
+	CHAC1	15	40918438	40966519	112	14	10164	0.98125	0.16323
-	PRR15L	17	47941967	47992894	153	20	10164	0.98117	0.16325
-	LOC100996574	9	133048029	133096567	75	11	10164	0.9812	0.16325
+	DYNLRB1	20	34481355	34550958	43	4	10164	0.98094	0.16331
+	ZNF547	19	57328435	57389559	178	14	10164	0.98095	0.16331
-	LOC105378189	14	60565033	60678945	147	8	10164	0.98086	0.16333
+	RPRD2	1	150329111	150486566	470	16	10164	0.98073	0.16336
-	BBS7	4	121814329	121905497	176	24	10164	0.98075	0.16336
+	GPR45	2	105206743	105253467	138	16	10164	0.98067	0.16338
-	PRDM7	16	90046566	90112339	139	10	10164	0.98038	0.16345
-	TTI1	20	37973007	38068468	104	10	10164	0.98032	0.16347
+	GPR17	2	127610864	127662639	136	15	10164	0.98022	0.16349
+	GOLGA6B	15	72573556	72677397	69	9	10164	0.97983	0.16358
+	SHKBP1	19	40541851	40601399	37	5	10164	0.97962	0.16364
+	DTX3	12	57569327	57619804	93	13	10164	0.97945	0.16368
-	IFIH1	2	162257079	162353764	129	4	10164	0.97917	0.16375
+	PFKFB2	1	206999416	207091023	195	21	10164	0.97874	0.16385
+	SORCS2	4	7157607	7752837	1984	209	10164	0.97872	0.16386
+	NDUFAF2	5	60910129	61163037	274	15	10164	0.97853	0.16391
-	LHCGR	2	48676774	48790741	388	39	10164	0.97843	0.16393
+	DHX9	1	182804304	182897982	74	3	10164	0.9784	0.16394
+	C2orf47	2	199920317	199974125	96	15	10164	0.97833	0.16396
-	RAD51AP2	2	17500584	17555580	43	6	10164	0.97773	0.1641
+	PCDHA5	5	140786776	141022344	308	9	10164	0.97765	0.16412
-	MC2R	18	13872044	13950707	266	17	10164	0.97698	0.16429
-	SLC2A1	1	42915375	42994176	222	20	10164	0.97618	0.16449
+	ABHD6	3	58202506	58304738	209	8	10164	0.97617	0.16449
-	LOC105378105	6	163701976	163749377	148	10	10164	0.97614	0.1645
-	MCAT	22	43122206	43178397	248	52	10164	0.97566	0.16462
-	CASP4	11	104932866	105003598	201	24	10164	0.97537	0.16469
-	C15orf40	15	82978308	83047298	96	13	10164	0.97528	0.16471
-	PDILT	16	20349170	20439742	175	24	10164	0.97525	0.16472
+	CTRC	1	15403442	15459247	83	13	10164	0.97477	0.16484

STRAND	SYMBOL	CHR	START	STOP	NSNPS	NPARAM	N	ZSTAT	P
-	C4orf45	4	158883532	159117867	273	29	10164	0.97456	0.16489
+	GMEB1	1	28633722	28725603	72	4	10164	0.97388	0.16506
-	NAP1L1	12	76034892	76119958	65	4	10164	0.97385	0.16507
-	PDZK1IP1	1	47173589	47225099	76	8	10164	0.97372	0.1651
-	UBA7	3	49795205	49848958	54	6	10164	0.97367	0.16511
+	EPS8L1	19	55040853	55097923	101	13	10164	0.97364	0.16512
-	EPM2AIP1	3	36975866	37028304	65	4	10164	0.97359	0.16513
-	VGLL4	3	11546067	11755768	522	46	10164	0.97314	0.16524
+	GLS	2	190845821	190975552	252	25	10164	0.97279	0.16533
-	B3GNT8	19	41415359	41463856	78	6	10164	0.97257	0.16538
+	ZNRF1	16	74964017	75120994	295	19	10164	0.9723	0.16545
+	ACPT	19	50755415	50805224	242	41	10164	0.97221	0.16547
+	TMEM82	1	15707492	15757797	138	14	10164	0.97222	0.16547
-	BTBD1	15	83006423	83102434	153	16	10164	0.97142	0.16567
-	MSMP	9	35742990	35789277	53	11	10164	0.97132	0.16569
-	COQ2	4	83128601	83320089	280	14	10164	0.97126	0.16571
-	FNDC5	1	32852268	32907492	28	3	10164	0.97104	0.16576
+	LOC102724371	12	24867309	24914261	109	11	10164	0.97107	0.16576
+	ZNF546	19	39960630	40031041	145	6	10164	0.97088	0.1658
-	MANSC4	12	27752641	27808083	187	22	10164	0.97075	0.16584
+	ARL4A	7	12651827	12700934	155	20	10164	0.96978	0.16608
-	SPATA32	17	45237274	45297112	116	20	10164	0.96944	0.16616
+	DGKA	12	55896162	55964027	48	5	10164	0.96927	0.16621
-	MOXD1	6	132286055	132436534	362	28	10164	0.96884	0.16631
-	ARHGEF2	1	155936640	156037635	121	17	10164	0.96863	0.16636
+	ADO	10	62769756	62818479	123	24	10164	0.9682	0.16647
-	KRTAP8-1	21	30802697	30848252	124	13	10164	0.96781	0.16657
-	CCNI	4	77037155	77111013	89	12	10164	0.9674	0.16667
+	DEFB123	20	31405519	31460257	113	8	10164	0.96737	0.16668
+	DNAJC25	9	111596352	111664351	258	29	10164	0.96733	0.16669
+	CCDC30	1	42428318	42664664	434	14	10164	0.96719	0.16672
+	HLA-DQA1	6	32602396	32664774	2903	36	10164	0.96688	0.1668
+	TRMT13	1	100098147	100160498	24	7	10164	0.96661	0.16687
+	FAM73B	9	129001605	129082082	18	3	10164	0.96589	0.16705
-	WIBG	12	55891413	55962913	50	5	10164	0.96572	0.16709
+	LOC101928021	17	67208959	67282108	85	7	10164	0.96552	0.16714
-	HSPA1L	6	31799619	31856999	287	78	10164	0.96534	0.16719
-	SLC38A4	12	46754761	46867422	329	26	10164	0.96516	0.16723
-	LIM2	19	51369909	51422956	11	6	10164	0.96515	0.16724
-	HAAO	2	42757089	42828138	311	28	10164	0.96506	0.16726
-	GHDC	17	42179087	42229532	53	8	10164	0.96493	0.16729
-	SCT	11	614989	662240	61	7	10164	0.96477	0.16733
+	UBE2V2	8	47963288	48074708	67	13	10164	0.96468	0.16735
-	SEC61G	7	54742247	54794246	178	12	10164	0.96471	0.16735
-	C1orf131	1	231213763	231276187	119	12	10164	0.96468	0.16735
-	NDRG1	8	133227171	133332587	175	25	10164	0.96461	0.16737
-	IL2RA	10	6000694	6097370	528	73	10164	0.96447	0.16741
+	MEGF8	19	42290609	42388769	82	3	10164	0.9643	0.16745
+	ZNF138	7	64759388	64863800	164	12	10164	0.96399	0.16752
+	HIST1H2BF	6	26164559	26209988	211	20	10164	0.96391	0.16755
-	SHROOM1	5	132812141	132866621	101	11	10164	0.96365	0.16761
+	GRK6	5	177391496	177452849	49	6	10164	0.96313	0.16774
+	RXRA	9	134291463	134450586	216	17	10164	0.96313	0.16774
+	ZDHHC16	10	97411131	97467370	79	12	10164	0.96281	0.16782
-	SDHB	1	17008722	17089170	264	9	10164	0.96191	0.16805
-	DEFB121	20	31394845	31454068	133	8	10164	0.96174	0.16809
-	CYTH3	7	6151779	6307611	204	18	10164	0.96157	0.16813
-	ZNF641	12	48325491	48386414	43	7	10164	0.9616	0.16813
+	KLHL7	7	23070734	23185421	209	7	10164	0.96114	0.16824
-	NME7	1	169121998	169402963	691	15	10164	0.9611	0.16825
-	LOC101929806	10	131874018	131924796	37	3	10164	0.96107	0.16826
+	ABHD17C	15	80660311	80765621	316	33	10164	0.9609	0.1683
+	AMH	19	2214114	2262073	66	10	10164	0.9608	0.16833
+	TBC1D12	10	94367429	94546332	352	20	10164	0.96053	0.16839
-	PBK	8	27799621	27873095	272	10	10164	0.96032	0.16845
+	UBE2B	5	134336176	134402108	77	3	10164	0.96002	0.16852
+	EXOSC7	3	44941230	45022668	117	10	10164	0.95989	0.16856
+	TSPO	22	43116514	43173242	238	48	10164	0.9595	0.16865
-	FANCG	9	35063838	35115016	129	22	10164	0.95943	0.16867
+	SNX21	20	45798622	45853275	143	19	10164	0.9593	0.1687
-	BMP5	6	55743653	55910593	409	33	10164	0.95923	0.16872
+	SDK1	7	3266448	4279000	3847	74	10164	0.95922	0.16872
+	TMEM200A	6	130331281	130453424	304	15	10164	0.95915	0.16874
-	VARS	6	31767518	31830935	265	77	10164	0.95844	0.16892

STRAND	SYMBOL	CHR	START	STOP	NSNPS	NPARAM	N	ZSTAT	P
-	TMEM132D	12	129061726	129938730	2270	220	10164	0.95845	0.16892
+	PRMT3	11	20352530	20519333	498	23	10164	0.95816	0.16899
+	CLN5	13	76956924	77012517	138	18	10164	0.9573	0.16921
+	EPHB2	1	22675770	22925330	506	67	10164	0.95717	0.16924
-	RALGDS	9	133087720	133184220	153	18	10164	0.95714	0.16925
-	ARHGAP9	12	57462255	57518959	105	11	10164	0.9571	0.16926
-	C6orf10	6	32278541	32407114	1851	42	10164	0.95618	0.16949
+	IQSEC3	12	31796	188460	409	44	10164	0.95616	0.16949
-	CORO2A	9	98110975	98227674	367	41	10164	0.95574	0.1696
-	DIDO1	20	62867734	62972952	235	20	10164	0.95574	0.1696
+	ARL5B	10	18624335	18691639	122	7	10164	0.9557	0.16961
-	PSMB8	6	32830717	32879935	478	49	10164	0.95545	0.16967
-	RAP1GAP	1	21586215	21704375	166	23	10164	0.95525	0.16973
-	ZBTB22	6	33304405	33352942	179	35	10164	0.95514	0.16975
-	LOC105376824	1	20153076	20198980	54	7	10164	0.95451	0.16991
+	IGSF5	21	39690838	39812096	528	26	10164	0.9543	0.16997
-	NPY1R	4	163313962	163379832	264	36	10164	0.95418	0.17
-	TSPYL4	6	116239964	116289098	144	11	10164	0.95407	0.17002
+	SIX6	14	60474220	60521807	76	4	10164	0.95384	0.17008
+	TTBK1	6	43208484	43298259	209	28	10164	0.95354	0.17016
+	UBE2QL1	5	6402347	6506721	267	35	10164	0.95351	0.17017
-	NDUFB7	19	14556078	14607077	16	2	10164	0.95326	0.17023
-	ASPDH	19	50501600	50549690	166	41	10164	0.95313	0.17026
+	GPATCH2L	14	76116885	76224473	344	20	10164	0.95306	0.17028
-	ERN2	16	23680304	23748500	77	11	10164	0.95283	0.17034
+	REM1	20	31440288	31494905	53	5	10164	0.95198	0.17055
+	SUCO	1	172497349	172621833	206	14	10164	0.95195	0.17056
+	AGAP1	2	235459089	236141800	835	79	10164	0.95196	0.17056
-	TMPRSS11D	4	67810876	67918998	109	9	10164	0.95174	0.17062
+	CEACAM21	19	41514250	41596844	134	12	10164	0.95153	0.17067
-	INSL3	19	17806513	17856574	85	18	10164	0.9514	0.1707
+	HIST1H4E	6	26169645	26215021	207	18	10164	0.95106	0.17079
+	SEMG1	20	45171997	45219773	65	5	10164	0.95026	0.17099
-	TBC1D5	3	17147162	17777739	883	23	10164	0.95004	0.17105
-	NMNAT2	1	183238237	183453499	667	42	10164	0.94974	0.17112
-	NETO1	18	72732314	72902575	426	38	10164	0.94965	0.17114
-	LOC102724404	5	157351616	157495056	311	19	10164	0.94965	0.17115
-	PPP1R14D	15	40805445	40863709	153	22	10164	0.94942	0.1712
+	TOMM6	6	41752443	41799896	89	14	10164	0.94941	0.17121
-	PHF21B	22	44858432	45044928	546	54	10164	0.94937	0.17122
+	CSF3	17	39980361	40027813	161	10	10164	0.94926	0.17124
-	S100A13	1	153608799	153669092	4	1	10164	0.94923	0.17125
+	SNAPIN	1	153623654	153671852	4	1	10164	0.94923	0.17125
+	PPT2	6	32118452	32173681	211	63	10164	0.94901	0.17131
-	AGTPBP1	9	85536539	85777029	175	8	10164	0.9487	0.17139
+	KCNJ12	17	21341387	21429872	42	4	10164	0.94857	0.17142
-	ZNF573	19	37725131	37814636	195	23	10164	0.94848	0.17144
-	KCP	7	128858934	128945719	130	18	10164	0.94844	0.17145
+	LOC105372315	19	20323181	20381817	22	1	10164	0.94791	0.17159
+	BSN	3	49519489	49681549	156	10	10164	0.94762	0.17166
-	ESCO1	18	21519281	21635736	50	3	10164	0.94744	0.17171
+	HIST1H3A	6	25985490	26030958	144	25	10164	0.94738	0.17172
-	ZBTB11	3	101639439	101712144	98	9	10164	0.94699	0.17182
-	FAM220A	7	6319409	6383959	107	10	10164	0.9466	0.17192
+	VPS25	17	42738436	42789600	69	6	10164	0.9464	0.17197
+	HSPA14	10	14803160	14881741	166	26	10164	0.94636	0.17198
-	SIAH2	3	150731123	150798476	111	5	10164	0.94577	0.17213
-	COPRS	17	31841865	31894307	35	1	10164	0.94555	0.17219
+	OXSR1	3	38130260	38265488	99	13	10164	0.94547	0.17221
-	LRRC57	15	42518444	42583804	58	7	10164	0.94491	0.17235
-	LSAMP	3	115792363	116480542	1090	45	10164	0.94479	0.17238
+	SREK1	5	66109218	66193616	91	6	10164	0.94441	0.17248
+	LRRTM3	10	66891034	67111551	515	35	10164	0.94376	0.17265
-	ARF4	3	57561363	57632488	106	7	10164	0.94315	0.1728
+	EIF4B	12	52971258	53052209	272	29	10164	0.94309	0.17282
-	CHST1	11	45637689	45700656	137	15	10164	0.94301	0.17284
+	AP4S1	14	30990106	31106450	305	23	10164	0.94269	0.17292
-	CDC25C	5	138275265	138373355	149	11	10164	0.94176	0.17316
+	ZFP28	19	56502716	56566810	156	14	10164	0.94172	0.17317
-	RPL4	15	66489315	66539855	143	18	10164	0.94163	0.17319
+	CAPN15	16	492756	564636	3	1	10164	0.94155	0.17321
+	HIST1H4A	6	25986679	26032050	147	25	10164	0.94136	0.17326
+	SLC26A11	17	80185401	80263509	242	23	10164	0.94106	0.17334
-	PISD	22	31608491	31697564	232	17	10164	0.94082	0.1734

STRAND	SYMBOL	CHR	START	STOP	NSNPS	NPARAM	N	ZSTAT	P
-	KIF3C	2	25916586	26017574	86	9	10164	0.94044	0.1735
+	SDF2L1	22	21607253	21654299	46	4	10164	0.93994	0.17362
-	HCRT	17	42174060	42220452	45	7	10164	0.93942	0.17376
-	SIX5	19	45754785	45804239	72	8	10164	0.9391	0.17384
-	HN1	17	75125243	75189683	39	6	10164	0.93905	0.17385
-	LARP4B	10	796914	1023323	327	25	10164	0.939	0.17386
-	ST6GALNAC1	17	76607774	76678838	197	16	10164	0.93899	0.17387
+	PCDHB6	5	141115022	141163287	94	12	10164	0.93834	0.17403
-	VWA9	15	65568757	65646289	123	6	10164	0.93823	0.17406
-	TFRC	3	196039284	196117161	238	17	10164	0.9382	0.17407
-	EEF1E1	6	8063360	8137595	265	32	10164	0.938	0.17412
-	BCL11B	14	99159287	99306713	216	31	10164	0.93765	0.17421
+	LOC102724427	19	20212268	20267291	55	1	10164	0.93752	0.17424
+	UBE2L3	22	21514447	21634034	100	2	10164	0.93752	0.17425
-	FKBP7	2	178453664	178515393	82	10	10164	0.93746	0.17426
-	ULK4	3	41229987	41997887	1600	37	10164	0.93741	0.17427
+	PLEKHA3	2	178445410	178515586	91	10	10164	0.9374	0.17428
-	HOXA5	7	27131052	27178668	93	14	10164	0.93735	0.17429
+	LOC105377135	3	52964045	53058123	246	22	10164	0.93735	0.17429
+	WNK2	9	93149155	93330572	432	34	10164	0.93731	0.1743
+	TSHZ2	20	52937309	53505330	613	64	10164	0.93724	0.17432
+	FAM129C	19	17488301	17565545	65	7	10164	0.93724	0.17432
+	AP2A1	19	49731923	49817112	109	14	10164	0.93713	0.17435
+	ARPC5L	9	124827135	124887733	67	8	10164	0.93695	0.17439
+	SLC39A13	11	47372132	47426500	92	6	10164	0.93675	0.17444
-	GJA9	1	38864067	38916626	130	10	10164	0.93654	0.1745
-	PABPC4	1	39550813	39611849	75	6	10164	0.93644	0.17452
+	PCTP	17	55715979	55853209	173	16	10164	0.93612	0.17461
-	EPPIN	20	45530626	45582426	288	8	10164	0.93588	0.17467
+	KIAA1328	18	36794109	37257316	365	3	10164	0.9357	0.17471
+	GABPA	21	25699947	25782460	145	16	10164	0.93566	0.17472
-	CCDC90B	11	83249093	83321407	157	14	10164	0.93564	0.17473
+	COG1	17	73158034	73218507	102	7	10164	0.93553	0.17476
-	EHMT2	6	31869759	31932707	356	66	10164	0.93542	0.17479
+	HAUS2	15	42513813	42579994	57	7	10164	0.93534	0.17481
+	CDH15	16	89136755	89205492	23	1	10164	0.93487	0.17493
+	OR10H3	19	15706393	15752343	206	13	10164	0.93487	0.17493
-	COL5A3	19	9949561	10045538	43	4	10164	0.93447	0.17503
-	CH25H	10	89195937	89242314	128	20	10164	0.93442	0.17504
-	DAXX	6	33308558	33358016	189	39	10164	0.9339	0.17518
+	MLH1	3	36958350	37060846	136	6	10164	0.93375	0.17522
+	NUDT22	11	64191258	64240269	128	23	10164	0.9335	0.17528
+	RTP1	3	187162486	187211465	136	22	10164	0.9335	0.17528
+	SCGB2A1	11	62173668	62223939	218	24	10164	0.93328	0.17534
-	SEC23A	14	39021919	39138243	209	20	10164	0.93326	0.17534
-	CCDC17	1	45610044	45659787	148	10	10164	0.93309	0.17539
-	OSBPL11	3	125518858	125630537	146	10	10164	0.93279	0.17546
-	PAFAH1B3	19	42287033	42337800	26	2	10164	0.9326	0.17551
-	LOC105369246	16	88079867	88125794	36	5	10164	0.9326	0.17551
-	TRIM64C	11	49043714	49094112	51	2	10164	0.9324	0.17556
+	SHD	19	4243601	4300723	76	4	10164	0.93237	0.17557
-	HINT2	9	35802960	35850479	67	9	10164	0.93232	0.17559
+	BHMG1	19	45688892	45774539	131	19	10164	0.93223	0.17561
-	RAET1G	6	149906878	149958152	152	15	10164	0.93212	0.17564
+	PPAPDC2	9	4627294	4675274	342	31	10164	0.93192	0.17569
-	CSGALNACT1	8	19394161	19792271	1364	75	10164	0.93168	0.17575
-	FGF1	5	142582178	142733070	269	36	10164	0.93142	0.17582
+	LOC105373289	1	227751481	227800651	172	26	10164	0.93107	0.17591
+	SPINT1	15	40809018	40867655	147	22	10164	0.93089	0.17595
+	SLC31A1	9	113186528	113274492	228	24	10164	0.93055	0.17604
-	SSH2	17	29615938	29965239	65	2	10164	0.93038	0.17609
+	TMEM147	19	35510600	35557527	152	24	10164	0.93022	0.17613
+	RAB22A	20	58274715	58377507	131	3	10164	0.93009	0.17616
-	ALG12	22	49849147	49953458	483	16	10164	0.93004	0.17618
+	FGFR4	5	177051872	177108142	136	15	10164	0.92971	0.17626
+	LHPP	10	124426772	124624141	999	88	10164	0.92951	0.17631
-	TSPAN5	4	98460367	98693661	564	57	10164	0.9291	0.17642
+	FOLH1B	11	89602520	89708718	437	7	10164	0.92875	0.17651
-	AFF4	5	132865379	132998662	279	17	10164	0.92865	0.17654
+	ZNF304	19	57316245	57369898	202	18	10164	0.92864	0.17654
+	LOC105369869	12	80901093	80947633	113	17	10164	0.92859	0.17655
+	MDFI	6	41602037	41664246	114	25	10164	0.92848	0.17658
-	LXN	3	158656414	158707693	153	8	10164	0.92818	0.17666
+	SNAP47	1	227699996	227791231	324	26	10164	0.92818	0.17666

STRAND	SYMBOL	CHR	START	STOP	NSNPS	NPARAM	N	ZSTAT	P
-	LOC388849	22	20138417	20186259	189	24	10164	0.92814	0.17667
-	IER3	6	30733199	30779550	337	38	10164	0.928	0.1767
-	HES6	2	238228267	238275124	132	11	10164	0.92799	0.17671
-	DNAJC11	1	6624168	6736906	162	8	10164	0.92738	0.17686
+	SPDYA	2	28775834	28860610	118	10	10164	0.92739	0.17686
-	SMIM2	13	44133151	44196257	147	23	10164	0.92737	0.17687
-	CCL19	9	34679570	34726277	109	19	10164	0.9272	0.17691
+	CNTN2	1	205008202	205088043	208	23	10164	0.92712	0.17693
-	ENO1	1	8851000	8914092	110	11	10164	0.92666	0.17705
-	NPC2	14	74469940	74528381	146	15	10164	0.92626	0.17716
+	CDADC1	13	49212875	49303486	116	6	10164	0.92621	0.17717
+	PIGB	15	55283865	55365648	132	9	10164	0.92608	0.1772
+	DDIT4	10	72238919	72286039	75	6	10164	0.9256	0.17733
-	ABCB1	7	87493863	87748323	513	40	10164	0.9249	0.17751
+	TDRD6	6	46652875	46714319	73	13	10164	0.92488	0.17751
+	SV2B	15	91064721	91311309	332	17	10164	0.92454	0.1776
-	NETO2	16	47071520	47179025	43	1	10164	0.92427	0.17767
+	C9orf163	9	136448495	136496067	96	9	10164	0.9242	0.17769
+	SLC22A13	3	38230807	38288315	41	10	10164	0.92413	0.17771
+	ZNF420	19	36972813	37140324	623	12	10164	0.92406	0.17773
-	ACTRT3	3	169756921	169804895	87	9	10164	0.9239	0.17777
+	ANKRD62	18	12037533	12139764	244	7	10164	0.92385	0.17778
+	COMMD1	2	61853728	62146070	480	19	10164	0.92346	0.17788
+	C2orf69	2	199876256	199938273	122	19	10164	0.92334	0.17791
-	TAP1	6	32835209	32888971	545	48	10164	0.92324	0.17794
+	ZBTB18	1	244013939	244067476	8	2	10164	0.9232	0.17795
-	TMEM244	6	129821244	129896503	243	18	10164	0.92308	0.17798
-	PLEKHA4	19	48827092	48903627	199	18	10164	0.92229	0.17819
-	DDIT3	12	57506588	57556698	69	8	10164	0.92208	0.17824
-	TRIM52	5	181246327	181296160	147	10	10164	0.92194	0.17828
-	IPP	1	45684324	45785813	269	15	10164	0.92172	0.17834
-	TTPA	8	63049488	63121100	224	20	10164	0.9217	0.17834
-	TULP2	19	48870822	48935184	171	14	10164	0.92169	0.17835
-	MRPS33	7	140996161	141049981	103	9	10164	0.92168	0.17835
-	RGPD2	2	87747198	88020814	25	1	10164	0.92079	0.17858
+	ARID3B	15	74506177	74608131	184	13	10164	0.92073	0.1786
-	DAB2	5	39361674	39460233	272	35	10164	0.92026	0.17872
+	REG3G	2	78990686	79038504	293	16	10164	0.91995	0.1788
+	KANK1	9	435294	756106	1407	52	10164	0.91978	0.17884
-	L3MBTL4	18	5944703	6449911	361	46	10164	0.91981	0.17884
-	ZFP64	20	52074011	52226985	339	17	10164	0.91957	0.1789
+	ALCAM	3	105331713	105586913	562	31	10164	0.91949	0.17892
-	EPPIN-WFDC6	20	45526280	45582426	297	7	10164	0.91919	0.179
-	SLC17A2	6	25902754	25965726	256	23	10164	0.91896	0.17906
+	FAM102B	1	108525349	108649327	251	13	10164	0.91864	0.17914
+	ULBP2	6	149906938	149959235	153	15	10164	0.91848	0.17918
-	PAX4	7	127600292	127653192	110	17	10164	0.91847	0.17919
+	DNAJC25-GNG1(	9	111596352	111680246	312	26	10164	0.91831	0.17923
+	RECK	9	36001913	36134455	10	3	10164	0.91827	0.17924
+	HPCA	1	32851087	32904646	30	3	10164	0.9182	0.17926
+	NEIL1	15	75311649	75365251	121	11	10164	0.91815	0.17927
-	HOXA3	7	27096190	27187581	179	21	10164	0.91813	0.17928
+	PHF20	20	35737001	35960377	409	12	10164	0.91811	0.17928
-	PPT1	1	40062710	40132470	236	20	10164	0.91766	0.1794
-	MACROD1	11	63988556	64201130	428	48	10164	0.9173	0.17949
-	TMEM8C	9	133504586	133563606	1	1	10164	0.91704	0.17956
+	TEX26	13	30897684	30985502	113	12	10164	0.91668	0.17966
-	SNX20	16	50656300	50716353	112	15	10164	0.91656	0.17969
+	EGFL8	6	32129605	32178285	188	53	10164	0.91628	0.17976
+	NTN1	17	8986542	9254000	664	73	10164	0.91612	0.1798
-	AVEN	15	33856227	34109458	397	27	10164	0.91569	0.17991
+	PPP2R2D	10	131865644	131979980	110	4	10164	0.91484	0.18014
+	CEP126	11	101880015	102011065	280	21	10164	0.91448	0.18023
-	WFDC6	20	45524197	45574495	282	8	10164	0.91445	0.18024
-	TRIM49D1	11	89901409	89955388	5	2	10164	0.91435	0.18027
-	KRTAP10-2	21	44540357	44586505	186	18	10164	0.91398	0.18036
+	SPATA5L1	15	45367273	45431419	130	9	10164	0.91379	0.18041
+	RGS14	5	177322711	177382601	40	3	10164	0.91375	0.18042
-	LMAN2	5	177321562	177386884	42	3	10164	0.91341	0.18051
-	EIF2AK1	7	6012247	6094229	270	24	10164	0.91315	0.18058
-	CDCA4	14	104999573	105056088	190	13	10164	0.91312	0.18059
+	UBXN10	1	20148302	20206048	70	9	10164	0.91305	0.18061
+	FAM159B	5	64655308	64728190	62	5	10164	0.91224	0.18082
-	QRICH1	3	49019707	49129071	19	2	10164	0.91192	0.18091

STRAND	SYMBOL	CHR	START	STOP	NSNPS	NPARAM	N	ZSTAT	P
+	STXBP5	6	147169358	147400476	130	7	10164	0.91177	0.18095
-	ZNF552	19	57797082	57850188	73	6	10164	0.91172	0.18096
-	PLA2G4D	15	42057565	42129559	262	34	10164	0.91136	0.18105
-	F12	5	177392138	177451524	47	6	10164	0.91099	0.18115
+	MDM4	1	204481379	204591681	212	12	10164	0.91069	0.18123
+	USP10	16	84664949	84789922	376	32	10164	0.91044	0.18129
-	CHID1	11	857859	950058	146	8	10164	0.91035	0.18132
+	ALOX5	10	45339176	45456121	497	31	10164	0.90989	0.18144
-	SBNO2	19	1097634	1209283	270	27	10164	0.90957	0.18152
+	TMEM72	10	44876308	44947010	253	25	10164	0.90949	0.18155
-	LOC105373472	1	178528473	178577606	156	8	10164	0.90921	0.18162
-	MOCS1	6	39893705	39969551	234	32	10164	0.90897	0.18168
+	AADACL3	1	12681115	12738759	75	6	10164	0.90848	0.18181
+	LDHC	11	18377306	18461246	260	14	10164	0.90747	0.18208
-	TM7SF3	12	26961570	27049427	210	31	10164	0.90745	0.18208
+	TMEM145	19	42278325	42335064	30	1	10164	0.90627	0.1824
-	HIST1H4D	6	26178710	26224076	233	17	10164	0.90619	0.18242
-	ENPP5	6	46149182	46206010	125	18	10164	0.90576	0.18253
+	TPGS1	19	472497	529654	15	3	10164	0.90576	0.18253
-	LOC105372911	1	214009269	214147021	151	29	10164	0.90554	0.18259
+	NANOS1	10	118994716	119044342	55	7	10164	0.9054	0.18263
+	LUC7L2	7	139305359	139433457	174	3	10164	0.90503	0.18273
+	C7orf55-LUC7L2	7	139306132	139433457	174	3	10164	0.90503	0.18273
-	ANXA6	5	151090706	151192882	439	53	10164	0.90489	0.18276
+	NCR3LG1	11	17316732	17391661	176	16	10164	0.90462	0.18283
+	VEGFB	11	64199584	64249264	135	26	10164	0.90439	0.18289
+	LOC105369201	15	100820307	100887011	144	15	10164	0.9043	0.18292
+	PDE12	3	57521252	57664979	231	12	10164	0.90426	0.18293
+	ZNF628	19	55441332	55494487	131	25	10164	0.90415	0.18296
+	PNPLA1	6	36204766	36323955	397	74	10164	0.90396	0.18301
-	PRRT1	6	32138363	32186943	216	58	10164	0.9039	0.18302
-	H2AFV	7	44816889	44883126	115	7	10164	0.90288	0.18329
-	IGDCC3	15	65317127	65413040	51	8	10164	0.90285	0.1833
+	ORAI1	12	121591550	121652040	12	3	10164	0.90278	0.18332
+	TRIM49B	11	48996600	49047977	103	2	10164	0.90263	0.18336
+	LOC101929301	7	44835946	44882158	70	5	10164	0.90252	0.18339
+	FRMPD1	9	37567978	37756904	482	55	10164	0.90237	0.18343
-	KRTAP19-6	21	30531535	30576864	138	9	10164	0.9019	0.18356
-	CST2	20	23813767	23861675	92	3	10164	0.90179	0.18358
-	NUAK2	1	205292059	205356791	128	17	10164	0.90135	0.1837
-	PTPRK	6	127958779	128555847	631	22	10164	0.90129	0.18372
+	C18orf25	18	46138553	46276992	271	12	10164	0.90115	0.18375
+	MEST	7	130451175	130516297	101	10	10164	0.90101	0.18379
+	CHMP6	17	80956841	81010133	148	15	10164	0.90101	0.18379
+	SLC24A1	15	65576405	65671242	142	7	10164	0.90087	0.18383
+	MSMO1	4	165292666	165353164	132	18	10164	0.90048	0.18393
-	ZNF607	19	37686363	37754790	144	19	10164	0.90017	0.18401
+	VAMP3	1	7736269	7791432	112	17	10164	0.9001	0.18403
+	CALU	7	128704292	128783423	155	17	10164	0.90003	0.18405
-	PTRF	17	42392449	42458320	158	17	10164	0.89963	0.18416
-	TRPV4	12	109773087	109868407	158	18	10164	0.89937	0.18423
+	SNPH	20	1231297	1319327	144	15	10164	0.89912	0.1843
+	PDHX	11	34880829	35006128	535	13	10164	0.89891	0.18435
-	ZKSCAN2	16	25226001	25292889	177	3	10164	0.89886	0.18436
+	LOC105372382	19	35412902	35458862	155	32	10164	0.89886	0.18436
+	SLC12A2	5	128048791	128199688	159	7	10164	0.89867	0.18441
-	LOC105370971	15	91260022	91307820	99	8	10164	0.8982	0.18454
-	LINC00083	1	178468153	178534619	107	16	10164	0.89799	0.1846
-	RGS5	1	163132299	163356791	549	31	10164	0.89741	0.18475
-	GOPC	6	117550269	117637542	151	16	10164	0.89733	0.18477
+	LOC105376785	16	90067673	90116120	91	7	10164	0.89726	0.18479
+	MED9	17	17441986	17503220	100	10	10164	0.89708	0.18484
+	PCDHA6	5	140792922	141022344	295	10	10164	0.89691	0.18488
-	CREG2	2	101338354	101422988	203	18	10164	0.89633	0.18504
-	AGPAT1	6	32158206	32213111	285	72	10164	0.89545	0.18527
-	SLAMF9	1	159941492	159989254	46	9	10164	0.89531	0.18531
+	ERC1	12	956208	1505933	1604	49	10164	0.89526	0.18532
-	RRP7A	22	42498335	42554823	33	12	10164	0.89526	0.18532
-	FABP4	8	81468497	81518238	63	3	10164	0.89513	0.18536
-	GOLGA6L2	15	23429386	23482243	64	4	10164	0.89512	0.18536
-	ALG9	11	111772195	111906581	230	15	10164	0.89498	0.1854
-	PM20D1	1	205818022	205885148	133	19	10164	0.89489	0.18542
+	TSEN15	1	184016651	184105846	122	13	10164	0.89476	0.18546
+	RPRD1B	20	37998487	38102364	86	10	10164	0.89375	0.18573

STRAND	SYMBOL	CHR	START	STOP	NSNPS	NPARAM	N	ZSTAT	P
-	CCDC148	2	158161356	158491824	1335	44	10164	0.89362	0.18576
+	CBLL1	7	107708497	107772011	23	2	10164	0.89325	0.18586
+	CIRH1A	16	69097596	69179034	117	16	10164	0.89318	0.18588
+	SLC39A2	14	20964255	21011875	147	18	10164	0.89296	0.18594
-	TRPT1	11	64213799	64261254	130	26	10164	0.89293	0.18595
-	DALRD3	3	49005488	49056386	9	2	10164	0.8928	0.18598
+	CRACR2B	11	791144	841991	93	8	10164	0.8927	0.18601
+	CCL20	2	227778842	227827564	56	9	10164	0.89263	0.18603
+	VWA5A	11	124080404	124156912	239	40	10164	0.89235	0.1861
-	TAL1	1	47206290	47267373	143	18	10164	0.89234	0.1861
-	KRT79	12	52811447	52869293	306	31	10164	0.89236	0.1861
-	OSBPL8	12	76341797	76594809	81	5	10164	0.89216	0.18615
-	CLDN5	22	19513024	19560337	133	25	10164	0.8921	0.18617
+	FTMT	5	121816955	121862833	86	14	10164	0.89203	0.18619
-	GRIFIN	7	2464636	2511855	62	6	10164	0.89173	0.18627
-	KIF13A	6	17749183	18022629	293	7	10164	0.8913	0.18638
-	RPL22L1	3	170854875	170905256	67	6	10164	0.89133	0.18638
-	UBR4	1	19064506	19245319	69	7	10164	0.89089	0.18649
+	LOC105377132	3	37140864	37206722	80	5	10164	0.89066	0.18656
+	CRISPLD2	16	84784981	84919510	563	65	10164	0.89059	0.18657
+	MUC2	11	1039875	1120508	87	11	10164	0.89056	0.18658
+	ZWILCH	15	66470083	66559485	230	24	10164	0.89045	0.18661
+	TJP2	9	69086264	69265208	587	44	10164	0.89027	0.18666
-	ZNF750	17	82819434	82875055	133	9	10164	0.88967	0.18682
-	HBG1	11	5238272	5284857	94	6	10164	0.88957	0.18685
-	MTPN	7	135916755	136012456	307	21	10164	0.88923	0.18694
-	LUZP6	7	135916755	136012456	307	21	10164	0.88923	0.18694
+	EPHB1	3	134760257	135270467	1046	71	10164	0.88909	0.18698
-	KIAA1715	2	175913862	176040388	465	43	10164	0.88895	0.18702
+	PSMA6	14	35243558	35327479	212	17	10164	0.88867	0.18709
-	LOC100129307	13	77651374	77697803	129	16	10164	0.88853	0.18713
-	AKTIP	16	53481280	53539411	148	23	10164	0.88817	0.18723
-	KRTAP24-1	21	30271309	30317958	192	22	10164	0.88785	0.18731
-	HS3ST1	4	11388364	11464567	258	36	10164	0.88747	0.18741
+	DLGAP4	20	36271323	36538637	58	6	10164	0.88705	0.18753
+	GOLGA6D	15	75247841	75305807	35	13	10164	0.88699	0.18754
+	TRIM38	6	25927689	25997329	260	28	10164	0.88694	0.18756
+	CCDC94	19	4212114	4279088	110	5	10164	0.88688	0.18757
+	CFAP99	4	2383893	2472963	189	17	10164	0.88677	0.1876
-	EEF1G	11	62549601	62608988	5	3	10164	0.88673	0.18761
-	TMEM200B	1	29109425	29164134	93	10	10164	0.88639	0.1877
-	LOC400499	16	11362601	11558533	437	36	10164	0.88639	0.1877
-	LOC105369202	19	15845340	15898863	208	11	10164	0.88634	0.18772
+	KLHL42	12	27744958	27813040	250	30	10164	0.8863	0.18773
-	ZMYND12	1	42420329	42491267	137	8	10164	0.8863	0.18773
-	SH3YL1	2	208136	299866	144	8	10164	0.88613	0.18777
-	RFC1	4	39277449	39401381	171	9	10164	0.88601	0.18781
-	YBX2	17	7278252	7329557	105	20	10164	0.88543	0.18796
-	LCT	2	135777845	135872180	232	19	10164	0.88542	0.18797
-	GPA33	1	167042836	167125631	43	5	10164	0.88528	0.188
-	IMPDH2	3	49014325	49064701	9	2	10164	0.8852	0.18802
+	TES	7	116175493	116268786	278	17	10164	0.88523	0.18802
+	FKBP2	11	64205941	64254135	132	26	10164	0.88516	0.18803
+	ALDH3A2	17	19613136	19687596	2	1	10164	0.8845	0.18821
-	PAPOLB	7	4847738	4896994	113	8	10164	0.88448	0.18822
-	BDNF	11	27644893	27757058	157	18	10164	0.88441	0.18824
-	PRCP	11	82814367	82936691	350	24	10164	0.88384	0.18839
-	C6orf132	6	42091119	42177977	122	14	10164	0.88343	0.1885
-	TSPYL1	6	116264859	116315117	170	16	10164	0.88333	0.18853
+	OR13F1	9	104469263	104515222	85	5	10164	0.88332	0.18853
+	DNAJC4	11	64195234	64244281	126	25	10164	0.88322	0.18856
-	STX2	12	130764883	130874274	129	13	10164	0.88316	0.18857
-	EIF3G	19	10105014	10154923	37	4	10164	0.88315	0.18858
+	RNF5	6	32143385	32190793	207	57	10164	0.88303	0.18861
-	IGF1	12	102385867	102516786	212	19	10164	0.88299	0.18862
-	VWCE	11	61248286	61330316	65	2	10164	0.88294	0.18863
+	PRKCA	17	66267782	66820744	1873	131	10164	0.88286	0.18866
+	UAP1	1	162526506	162609843	312	17	10164	0.88273	0.18869
+	UPK1A	19	35631516	35688485	186	23	10164	0.88269	0.1887
+	C15orf48	15	45395529	45443449	79	8	10164	0.88237	0.18879
-	FBXO46	19	45700629	45765893	96	14	10164	0.88227	0.18881
+	ATP5D	19	1206750	1254825	72	8	10164	0.8821	0.18886
+	EXOC6	10	92799713	93069494	399	18	10164	0.88189	0.18892
-	PSPC1	13	19664752	19818019	131	7	10164	0.88181	0.18894

STRAND	SYMBOL	CHR	START	STOP	NSNPS	NPARAM	N	ZSTAT	P
+	KIAA0391	14	35086283	35284078	535	30	10164	0.88178	0.18895
+	PPCS	1	42421341	42483381	121	8	10164	0.88162	0.18899
+	HIST1H2BH	6	26216651	26262075	179	19	10164	0.8816	0.189
+	CLASRP	19	45004040	45080956	86	17	10164	0.88135	0.18906
+	MIA2	14	39198921	39263371	140	12	10164	0.88131	0.18908
-	KDM7A	7	140074746	140211956	100	4	10164	0.88112	0.18913
-	EMC1	1	19205664	19286559	89	7	10164	0.88084	0.1892
-	SELL	1	169680665	169746702	322	22	10164	0.88048	0.1893
+	RBM17	10	6053986	6127459	403	54	10164	0.88037	0.18933
-	RTBDN	19	12815477	12873267	51	2	10164	0.88008	0.18941
+	MAST1	19	12798934	12884953	63	2	10164	0.87992	0.18945
+	CYP20A1	2	203203441	203315840	145	8	10164	0.87982	0.18948
+	CDC14A	1	100310042	100530277	452	30	10164	0.87971	0.18951
+	XAF1	17	6720572	6785647	178	22	10164	0.87967	0.18952
+	HSH2D	19	16099028	16168575	85	6	10164	0.87943	0.18958
-	SREK1IP1	5	64708148	64803731	130	11	10164	0.87827	0.1899
+	CACNG5	17	66800117	66897296	245	45	10164	0.87822	0.18991
+	CLUL1	18	561998	660298	193	42	10164	0.87824	0.18991
-	CCDC25	8	27723316	27807655	249	11	10164	0.8782	0.18992
-	LOC102724279	19	15841993	15899904	211	11	10164	0.87817	0.18992
+	TEX35	1	178478077	178533500	89	13	10164	0.87768	0.19006
-	SEC11A	15	84659537	84751460	167	19	10164	0.87764	0.19007
+	SPAG16	2	213249379	214420501	1278	36	10164	0.87763	0.19007
-	ACTL9	19	8687397	8733830	80	11	10164	0.87734	0.19015
+	WFDC10A	20	45594746	45641192	149	12	10164	0.87697	0.19025
-	SLC46A1	17	28384642	28441212	68	10	10164	0.87691	0.19027
+	PRICKLE4	6	41745762	41797372	106	16	10164	0.87679	0.1903
-	STK33	11	8381866	8629289	634	17	10164	0.87659	0.19036
+	ZFYVE9	1	52107094	52356686	166	3	10164	0.87622	0.19045
+	ZNF511	10	133273889	133323162	18	2	10164	0.87612	0.19048
-	SDC1	2	20190797	20260433	108	9	10164	0.87548	0.19066
+	C2orf15	2	99106722	99161487	67	6	10164	0.87531	0.1907
-	CC2D1B	1	52340593	52401205	37	2	10164	0.87453	0.19091
+	PCDHA7	5	140799384	141022344	290	12	10164	0.8744	0.19095
-	ISOC2	19	55442978	55496682	134	25	10164	0.87425	0.19099
-	SLITRK1	13	83867205	83917393	192	17	10164	0.87415	0.19102
-	IFNA21	9	21155637	21201660	233	14	10164	0.87369	0.19114
+	TMOD1	9	97466180	97611743	411	42	10164	0.87371	0.19114
+	BOK	2	241523064	241584138	95	13	10164	0.87292	0.19135
+	ACO1	9	32349603	32460836	258	30	10164	0.87219	0.19155
-	PARS2	1	54745519	54799553	122	22	10164	0.87144	0.19176
+	GRHL3	1	24284322	24374482	98	4	10164	0.87139	0.19177
-	NINJ1	9	93111489	93169304	155	21	10164	0.8713	0.1918
-	ASGR2	17	7091322	7150522	230	33	10164	0.87099	0.19188
-	UTP6	17	31853171	31936710	78	2	10164	0.87098	0.19188
-	PFDN1	5	140235050	140338104	121	6	10164	0.87079	0.19193
+	NEIL2	8	11734663	11797345	408	40	10164	0.87076	0.19194
-	UBL7	15	74435977	74496188	134	10	10164	0.87051	0.19201
-	MYCBP	1	38852490	38908378	130	10	10164	0.87008	0.19213
+	BCR	22	23145365	23328037	412	27	10164	0.8699	0.19218
-	PSMC3	11	47408769	47461473	86	5	10164	0.86924	0.19236
-	TFEC	7	115925148	116194891	536	25	10164	0.86913	0.19239
-	C10orf67	10	23192851	23380216	249	15	10164	0.86884	0.19247
+	TEAD1	11	12639422	12954737	432	39	10164	0.86872	0.1925
-	SLC19A2	1	169453909	169521079	144	19	10164	0.86874	0.1925
-	DBX1	11	20146214	20195324	133	28	10164	0.86861	0.19253
+	ACTR6	12	100165087	100234424	132	9	10164	0.86857	0.19254
+	MICALCL	11	12251900	12369144	399	30	10164	0.86841	0.19258
+	GPNMB	7	23211686	23285110	124	8	10164	0.86829	0.19262
-	RPRD1A	18	35979824	36102594	191	12	10164	0.8673	0.19289
-	SGCZ	8	14079864	15273283	7099	226	10164	0.86698	0.19298
+	SF3A2	19	2201817	2258679	82	11	10164	0.86674	0.19304
+	KLK2	19	50838433	50890567	284	42	10164	0.86672	0.19305
+	SNRPE	1	203826585	203881152	80	6	10164	0.86626	0.19317
-	ZBTB7C	18	48016864	48444422	803	99	10164	0.86609	0.19322
+	LINC01272	20	50232483	50289795	170	27	10164	0.86597	0.19325
+	LY96	8	73956329	74042562	54	4	10164	0.86592	0.19327
+	ZNF880	19	52334917	52395795	207	24	10164	0.86587	0.19328
+	MRPL23	11	1912272	2024017	164	36	10164	0.86585	0.19329
-	FEV	2	218971087	219020657	14	6	10164	0.86582	0.1933
+	ANO5	11	22157513	22293367	149	9	10164	0.86579	0.1933
-	RGS7	1	240765514	241392230	923	79	10164	0.86574	0.19332
-	AMMECR1L	2	127851630	127923570	151	18	10164	0.86532	0.19343
+	HOXC6	12	53981852	54040823	38	5	10164	0.8646	0.19363

STRAND	SYMBOL	CHR	START	STOP	NSNPS	NPARAM	N	ZSTAT	P
+	PRR19	19	42267132	42320821	33	1	10164	0.86457	0.19364
-	HBS1L	6	134950378	135089898	185	4	10164	0.86409	0.19377
+	C11orf96	11	43907556	43953883	147	30	10164	0.86385	0.19384
-	HBG2	11	5243191	5289781	95	7	10164	0.86368	0.19388
+	CTLA4	2	203832788	203883960	88	9	10164	0.86291	0.19409
+	PXYLP1	3	141196825	141304903	356	34	10164	0.86259	0.19418
+	ISL1	5	51348124	51404738	54	3	10164	0.86252	0.1942
+	SOD3	4	24760463	24810845	70	10	10164	0.86245	0.19422
+	CHST11	12	104421914	104772014	926	84	10164	0.86223	0.19428
-	FLRT3	20	14313993	14372667	146	16	10164	0.86114	0.19458
-	FDXACB1	11	111864056	111914457	92	9	10164	0.86041	0.19478
-	SEL1L3	4	25711867	25898595	139	24	10164	0.86026	0.19482
-	CER1	9	14709733	14757717	67	9	10164	0.86005	0.19488
-	ZNF385A	12	54359129	54426305	84	10	10164	0.86001	0.19489
-	GCNT4	5	75017464	75065899	124	19	10164	0.85991	0.19492
+	SLC25A4	4	185108263	185160384	105	9	10164	0.85974	0.19497
+	SNTB2	16	69152147	69319052	283	19	10164	0.85958	0.19501
-	ZDHHC6	10	112415439	112482234	66	10	10164	0.85938	0.19506
-	PLEKHJ1	19	2219952	2272405	69	10	10164	0.85895	0.19518
-	KLK12	19	51018960	51070652	221	49	10164	0.85879	0.19523
-	PRKRA	2	178421414	178486231	64	8	10164	0.85827	0.19537
-	GRIN2B	12	13527337	14017002	1212	95	10164	0.85823	0.19538
-	DDX60	4	168206291	168353807	488	20	10164	0.85801	0.19544
-	DNAJA2	16	46945362	47008714	1	1	10164	0.85792	0.19547
+	ZNF277	7	112171588	112353934	268	17	10164	0.85785	0.19549
+	UMODL1	21	41996463	42152996	589	64	10164	0.85779	0.1955
+	KRTAP20-2	21	30600264	30645461	78	6	10164	0.85767	0.19554
+	FFAR2	19	35414715	35461767	162	35	10164	0.85757	0.19556
+	GRAMD3	5	126325096	126504364	452	72	10164	0.85691	0.19575
-	CYBA	16	88633289	88686085	71	20	10164	0.85685	0.19576
+	CSN1S1	4	69896062	69956570	146	16	10164	0.85678	0.19578
-	IDH1	2	208226227	208290143	83	13	10164	0.8568	0.19578
+	KCTD16	5	144135760	144495686	493	18	10164	0.85619	0.19595
-	E2F6	2	11434375	11501177	96	6	10164	0.85583	0.19605
-	CD5L	1	157817071	157876844	150	17	10164	0.85552	0.19613
-	ADGRL1	19	14137737	14241248	1	1	10164	0.85519	0.19622
-	SSR1	6	7271143	7348314	183	19	10164	0.85486	0.19631
-	UCKL1	20	63929829	63994435	83	7	10164	0.85466	0.19637
+	GTF2IRD1	7	74418790	74612605	236	17	10164	0.85445	0.19643
+	GTF3C6	6	110923560	110977888	234	13	10164	0.85443	0.19643
+	GLTPD2	17	4753422	4800592	81	19	10164	0.85421	0.19649
+	HIGD1C	12	50901223	50982571	258	19	10164	0.85423	0.19649
+	PCDHB12	5	141173719	141222126	136	11	10164	0.85396	0.19656
+	ODF1	8	102516620	102571017	284	42	10164	0.85366	0.19665
-	DFNA5	7	24688355	24793020	292	35	10164	0.8536	0.19666
+	KRTAP10-8	21	44577079	44622954	177	5	10164	0.85303	0.19682
-	KANK2	19	11154267	11232567	54	9	10164	0.85278	0.19689
+	ZNF93	19	19865913	19945998	63	5	10164	0.85227	0.19703
-	RIMBP3C	22	21535666	21586461	40	2	10164	0.85172	0.19719
-	CRYBA2	2	218980190	219028556	22	7	10164	0.85145	0.19726
+	CNNM1	10	99294099	99404330	289	21	10164	0.85142	0.19727
+	LRTM2	12	1785267	1846753	129	23	10164	0.85142	0.19727
-	DEFA1B	8	6986766	7034202	54	9	10164	0.85131	0.1973
-	NCAPD3	11	134142442	134260345	174	17	10164	0.85119	0.19733
+	C7	5	40874473	41026004	634	43	10164	0.85112	0.19735
+	GAS2L3	12	100538646	100638288	260	25	10164	0.851	0.19738
-	ZNF18	17	11967439	12032510	133	15	10164	0.85085	0.19743
+	TEX12	11	112132372	112182556	86	10	10164	0.85084	0.19743
-	FAM71B	5	157152332	157201268	142	13	10164	0.85077	0.19745
+	MCM10	10	13126554	13221104	236	37	10164	0.85018	0.19761
-	MTIF3	13	27425639	27485854	169	16	10164	0.85018	0.19761
-	LOC101927147	2	239869393	239948187	302	27	10164	0.84989	0.19769
+	OIT3	10	72858556	72943036	50	4	10164	0.84926	0.19787
-	PRRT4	7	128340325	128397069	56	17	10164	0.84898	0.19795
+	ACPP	3	132282367	132378302	378	22	10164	0.84883	0.19799
-	LOC105374995	6	27115897	27165449	71	14	10164	0.84853	0.19807
+	FAT3	11	92279283	92906470	674	28	10164	0.84838	0.19811
-	NKX2-6	8	23692451	23741598	260	36	10164	0.8484	0.19811
-	FAM124B	2	224368698	224436994	329	27	10164	0.84817	0.19817
+	MAPK4	18	50525114	50741826	596	58	10164	0.8477	0.1983
-	GLYAT	11	58698757	58766974	299	15	10164	0.84736	0.1984
+	LOC105377134	21	13992416	14070261	2	1	10164	0.84721	0.19844
-	COPG2	7	130496238	130703758	360	15	10164	0.84715	0.19846
-	SFT2D1	6	166309728	166377606	85	4	10164	0.84703	0.19849

STRAND	SYMBOL	CHR	START	STOP	NSNPS	NPARAM	N	ZSTAT	P
-	VEPH1	3	157249742	157538626	876	47	10164	0.84668	0.19859
+	ITGAV	2	186555063	186690902	185	11	10164	0.84648	0.19864
-	GTDC1	2	143935787	144367534	263	13	10164	0.84626	0.1987
-	S100B	21	46588618	46640122	233	24	10164	0.8455	0.19892
-	GNAT3	7	80447292	80562915	145	11	10164	0.84515	0.19901
+	MAP1LC3A	20	34511830	34570345	51	5	10164	0.84488	0.19909
-	GNL1	6	30531377	30592594	225	43	10164	0.84479	0.19911
-	UBE2H	7	129820732	129987960	323	7	10164	0.84465	0.19915
-	ADARB2	10	1167313	1772498	1371	129	10164	0.8446	0.19917
+	MYO7B	2	127500693	127647728	284	24	10164	0.84444	0.19921
+	MRPS9	2	105003025	105109960	151	2	10164	0.84414	0.1993
+	RNF34	12	121365083	121434352	169	14	10164	0.8441	0.19931
+	CELA2B	1	15441101	15501400	184	17	10164	0.84381	0.19939
+	SP2	17	47861054	47942271	223	29	10164	0.84337	0.19951
+	BBOX1	11	27005705	27137807	388	29	10164	0.8431	0.19959
+	HDAC9	7	18051942	19009521	2810	242	10164	0.84308	0.19959
+	ZNF804A	2	184563366	184949487	700	22	10164	0.84302	0.19961
-	PATZ1	22	31315804	31381263	26	4	10164	0.84296	0.19963
+	CCND2	12	4238733	4315356	83	19	10164	0.84282	0.19967
+	PSMC4	19	39936005	39991764	109	6	10164	0.84275	0.19969
+	SLC17A6	11	22303121	22389503	37	7	10164	0.84241	0.19978
-	OCM2	7	97974700	98025104	220	33	10164	0.8421	0.19987
+	ESCO2	8	27736477	27822396	279	9	10164	0.84196	0.19991
-	DDX6	11	118737763	118826263	191	14	10164	0.84173	0.19997
+	PTGDR	14	52232256	52286724	103	11	10164	0.84169	0.19998
+	MED17	11	93749239	93823330	50	6	10164	0.84168	0.19998
+	DMRTB1	1	53424399	53477488	192	19	10164	0.84121	0.20011
+	ADIPOR2	12	1655981	1798679	380	22	10164	0.84105	0.20016
+	KRTAP10-4	21	44538729	44585371	185	19	10164	0.84093	0.20019
-	SPDYE6	7	102335752	102392047	44	5	10164	0.84091	0.2002
+	RAG1	11	36475400	36589762	272	37	10164	0.84089	0.20021
+	TOMM40	19	44856220	44913689	158	19	10164	0.84087	0.20021
-	HRAS	11	522242	570561	194	17	10164	0.84084	0.20022
-	PIDD1	11	789179	844501	103	9	10164	0.84082	0.20023
-	KRTAP10-5	21	44569455	44615604	205	8	10164	0.8406	0.20029
+	LOC105377789	8	4493043	4547829	232	37	10164	0.84043	0.20033
+	KIAA1456	8	12910674	13039777	721	42	10164	0.84032	0.20036
-	SIGLEC5	19	51600957	51665474	105	14	10164	0.83992	0.20048
-	AAED1	9	96631251	96690323	6	3	10164	0.83983	0.2005
-	PCMTD1	8	51807575	51934186	411	15	10164	0.83918	0.20068
+	PHF3	6	63600802	63725522	183	12	10164	0.83916	0.20069
-	MEOX2	7	15601212	15721683	332	22	10164	0.83882	0.20079
-	TNNC2	20	45813214	45868323	159	20	10164	0.8388	0.20079
+	AIFM3	22	20930130	20991360	40	7	10164	0.83862	0.20084
+	MSRB3	12	65243643	65476907	334	20	10164	0.83808	0.20099
+	EML1	14	99702722	99952060	583	50	10164	0.83803	0.20101
-	THRB	3	24107153	24530281	1097	129	10164	0.83798	0.20102
-	LGMN	14	92693807	92783702	174	37	10164	0.83788	0.20105
-	PENK	8	56430954	56481723	166	16	10164	0.83769	0.2011
-	ALDOB	9	101410560	101470780	169	21	10164	0.83756	0.20114
-	SYNE4	19	35993000	36043793	65	11	10164	0.83757	0.20114
+	RAB33B	4	139418232	139485916	10	2	10164	0.83734	0.2012
+	RSC1A1	1	15624869	15671722	108	7	10164	0.8373	0.20121
+	GTF2H1	11	18287269	18377043	113	8	10164	0.83716	0.20125
-	SLCO4C1	5	102223986	102331549	82	5	10164	0.83687	0.20133
-	FAM19A4	3	68721764	68967610	755	33	10164	0.83679	0.20136
+	PGA5	11	61206157	61261457	17	2	10164	0.83668	0.20139
-	KRTAP6-1	21	30603686	30648904	71	6	10164	0.83664	0.2014
-	IL18	11	112133251	112199117	119	11	10164	0.83658	0.20141
+	TMEM233	12	119557636	119664442	227	17	10164	0.83646	0.20145
+	PRSS55	8	10490477	10564166	357	22	10164	0.83629	0.2015
+	OR8A1	11	124535003	124612563	259	30	10164	0.83616	0.20153
-	TMEM259	19	999651	1056169	305	27	10164	0.83595	0.20159
+	OR51T1	11	4846819	4892883	85	6	10164	0.83576	0.20164
-	SERPINB4	18	63627259	63679319	4	1	10164	0.83557	0.2017
+	HIST1H2AE	6	26181920	26227483	237	17	10164	0.8353	0.20178
+	KRTAP10-7	21	44565597	44612174	218	9	10164	0.83529	0.20178
+	PAM	5	102720276	103041105	727	20	10164	0.83507	0.20184
+	HSPB3	5	54420601	54466384	175	22	10164	0.83499	0.20186
+	PITHD1	1	23743386	23798232	5	1	10164	0.83492	0.20188
+	ZNF253	19	19830830	19904449	59	7	10164	0.83429	0.20206
-	CCNO	5	55221152	55268717	112	10	10164	0.83313	0.20239
-	C12orf71	12	27071057	27119144	150	14	10164	0.8331	0.20239
+	HOXD10	2	176081764	176129942	170	45	10164	0.83308	0.2024

STRAND	SYMBOL	CHR	START	STOP	NSNPS	NPARAM	N	ZSTAT	P
+	GPT	8	144467221	144517174	44	4	10164	0.83271	0.2025
-	TTLL6	17	48752231	48852107	319	28	10164	0.83231	0.20262
+	DOK5	20	54440472	54661171	210	20	10164	0.83209	0.20268
+	TMEM159	16	21123377	21190616	141	5	10164	0.83202	0.2027
+	SRD5A3	4	55311221	55383100	142	16	10164	0.8319	0.20273
-	LDHB	12	21625341	21692971	73	2	10164	0.83175	0.20277
+	LOC105378101	6	163376037	163424279	17	3	10164	0.83168	0.2028
+	H1F0	22	37770107	37817436	65	3	10164	0.83138	0.20288
-	HGF	7	81689006	81805438	214	19	10164	0.83117	0.20294
-	ADI1	2	3487919	3554579	60	7	10164	0.83115	0.20294
+	GIPC2	1	77989370	78148449	234	16	10164	0.83112	0.20295
-	CHRM4	11	46374792	46421608	18	3	10164	0.8311	0.20296
+	KCNMB2	3	178501298	178854429	875	50	10164	0.83106	0.20297
-	LOC105374752	5	41194	92883	34	4	10164	0.83084	0.20303
-	C11orf68	11	65906810	65954060	90	19	10164	0.8306	0.2031
-	KTI12	1	52022105	52068800	56	2	10164	0.83047	0.20314
+	SRRM4	12	118946486	119173053	821	74	10164	0.83033	0.20318
+	PSMD7	16	74261775	74316288	12	1	10164	0.83024	0.2032
+	ACP1	2	229140	288283	96	7	10164	0.82982	0.20332
-	GSTA5	6	52821742	52881095	111	2	10164	0.82942	0.20343
-	LIMS2	2	127628421	127716786	225	26	10164	0.82887	0.20359
-	LOC105378479	10	110137955	110202921	98	15	10164	0.82882	0.2036
-	UBE3A	15	25327234	25474043	92	2	10164	0.82857	0.20367
-	CEP19	3	196696273	196747294	108	7	10164	0.82858	0.20367
+	CLLU1	12	92386531	92441002	66	10	10164	0.8286	0.20367
+	NAT14	19	55450190	55497568	127	24	10164	0.8285	0.20369
-	CCDC8	19	46400329	46448662	140	16	10164	0.82853	0.20369
+	KSR1	17	27421464	27636441	86	3	10164	0.82799	0.20384
+	PER3	1	7749380	7855181	266	28	10164	0.82769	0.20392
-	ACTA1	1	229421245	229469096	62	3	10164	0.82757	0.20396
+	NECAP2	1	16405672	16470090	82	8	10164	0.82754	0.20397
+	NCMAP	1	24521076	24619328	323	27	10164	0.82699	0.20412
-	EMC9	14	24128965	24176588	56	10	10164	0.82698	0.20413
+	PPIA	7	44761636	44813123	85	6	10164	0.82684	0.20416
-	CPNE1	20	35616031	35699956	170	11	10164	0.82684	0.20416
-	CARS	11	2990922	3092451	249	15	10164	0.82671	0.2042
+	WDR38	9	124818347	124867891	48	5	10164	0.8259	0.20443
+	CNNM4	2	96725902	96821891	118	8	10164	0.82554	0.20453
-	CEP120	5	123334885	123458592	362	17	10164	0.82521	0.20463
+	SH2D4B	10	80502902	80656560	398	31	10164	0.82502	0.20468
+	MCM4	8	47925203	47988160	36	8	10164	0.82472	0.20477
-	BTNL2	6	32383339	32443879	1267	36	10164	0.82466	0.20478
-	MUSTN1	3	52823115	52870219	158	11	10164	0.82468	0.20478
+	LIPT1	2	99119955	99173157	60	5	10164	0.82458	0.2048
+	MMRN1	4	89859876	89964629	195	13	10164	0.82413	0.20493
+	NPL	1	182754449	182840384	84	10	10164	0.82397	0.20498
+	RALGAPB	20	38437806	38588861	107	4	10164	0.82391	0.20499
+	SH3BP1	22	37604677	37666043	114	10	10164	0.82338	0.20515
-	CCNA2	4	121806444	121858933	130	19	10164	0.82331	0.20517
+	LOC400682	19	21602371	21667708	567	17	10164	0.82294	0.20527
+	ARMC7	17	75074952	75140265	119	16	10164	0.8227	0.20534
+	EPHX2	8	27456002	27554922	270	37	10164	0.82186	0.20558
-	NRG2	5	139836779	140078437	339	32	10164	0.82184	0.20558
+	UFL1	6	96486826	96565276	39	5	10164	0.82182	0.20559
-	CCDC108	2	218992846	219076551	130	10	10164	0.82179	0.2056
+	ALMS1	2	73350758	73619919	9	1	10164	0.82175	0.20561
+	MADCAM1	19	461490	515343	9	1	10164	0.82151	0.20568
-	ODF3L2	19	453346	509983	9	1	10164	0.82151	0.20568
+	HMSD	18	63914250	63971821	158	7	10164	0.82126	0.20575
-	MATN4	20	45283446	45343566	155	16	10164	0.82114	0.20578
+	TNS2	12	53011980	53074379	190	20	10164	0.8211	0.20579
-	PCYT1A	3	196228382	196322713	20	2	10164	0.82078	0.20589
-	PDE4C	19	18197961	18283200	232	24	10164	0.82024	0.20604
-	TPR	1	186301652	186410848	107	5	10164	0.8198	0.20616
-	OR51S1	11	4838203	4894600	114	9	10164	0.81974	0.20618
-	NFS1	20	35658688	35734365	187	12	10164	0.81953	0.20624
+	LOC105371729	1	155966153	156043225	89	14	10164	0.81922	0.20633
+	MANEAL	1	37758820	37811606	4	2	10164	0.8187	0.20648
+	SERPINB8	18	63935028	64016821	115	10	10164	0.81862	0.2065
+	ZNRD1	6	30026240	30074909	555	23	10164	0.81849	0.20654
-	C17orf96	17	38661703	38709934	29	5	10164	0.8184	0.20656
-	CX3CR1	3	39253494	39316735	157	24	10164	0.81838	0.20657
-	PPM1H	12	62633982	62969885	339	36	10164	0.81821	0.20662
-	FBXL3	13	76991339	77062196	157	14	10164	0.81791	0.20671

STRAND	SYMBOL	CHR	START	STOP	NSNPS	NPARAM	N	ZSTAT	P
+	LOC101929163	6	32220717	32417822	2697	54	10164	0.81758	0.2068
+	AZU1	19	792831	842017	67	7	10164	0.81734	0.20687
-	SLC44A4	6	31853192	31914046	314	60	10164	0.81721	0.2069
+	ADPRM	17	10662610	10721558	32	2	10164	0.81716	0.20692
+	MIDN	19	1213553	1269143	64	7	10164	0.81681	0.20702
-	WNT9A	1	227908658	227982975	401	30	10164	0.81628	0.20717
+	IRF9	14	24126213	24176565	55	10	10164	0.81594	0.20727
-	LOC105378193	13	112003841	112071593	106	10	10164	0.81576	0.20732
+	C11orf1	11	111844198	111894074	81	9	10164	0.81547	0.2074
+	GAREML	2	26138091	26212529	122	8	10164	0.81549	0.2074
+	NUBP2	16	1747923	1799191	196	20	10164	0.81537	0.20743
+	ATP6V1B1	2	70900868	70975431	206	37	10164	0.81518	0.20748
+	C16orf45	16	15399468	15604689	156	10	10164	0.81513	0.2075
-	EHD4	15	41889440	42007557	378	48	10164	0.81503	0.20753
+	SLC26A10	12	57584910	57636151	93	15	10164	0.81502	0.20753
+	MARVELD1	10	97678708	97728152	122	20	10164	0.81495	0.20755
+	CST7	20	24914230	24969928	285	9	10164	0.81469	0.20763
+	PRSS38	1	227780692	227870439	319	38	10164	0.81396	0.20783
+	GLI1	12	57425135	57482268	96	16	10164	0.81392	0.20785
+	MAP1S	19	17684494	17744515	112	10	10164	0.81378	0.20788
+	DPY19L3	19	32370749	32495893	31	2	10164	0.81378	0.20788
+	IL17RE	3	9867062	9926402	71	4	10164	0.8135	0.20796
+	OMP	11	77067840	77113331	10	1	10164	0.81341	0.20799
+	CYP3A43	7	99793013	99877092	99	9	10164	0.81322	0.20805
+	LYN	8	55843463	56022447	550	38	10164	0.81271	0.20819
-	STEAP4	7	88266429	88341913	88	14	10164	0.81266	0.20821
-	KRTAP6-2	21	30588686	30633874	75	7	10164	0.81244	0.20827
-	RBM12	20	35638925	35699956	129	10	10164	0.81221	0.20833
-	ALKBH8	11	107492726	107600737	264	20	10164	0.81222	0.20833
-	KBTBD3	11	106036806	106112765	120	15	10164	0.81218	0.20834
-	RAPSN	11	47427757	47484178	58	4	10164	0.81176	0.20847
+	DTL	1	212000553	212115006	239	14	10164	0.81171	0.20848
-	KLF14	7	130722554	130769033	30	4	10164	0.81165	0.20849
+	EBI3	19	4194302	4247542	43	3	10164	0.81153	0.20853
+	PLD2	17	4772101	4833432	118	22	10164	0.81112	0.20865
-	SLC7A11	4	138154094	138347660	384	41	10164	0.81105	0.20867
-	CSMD2	1	33503999	34200842	1374	120	10164	0.81105	0.20867
+	RTEL1	20	63622810	63706253	315	31	10164	0.81084	0.20873
-	LAD1	1	201370838	201434541	155	32	10164	0.81062	0.20879
+	CD200	3	112297347	112372812	308	21	10164	0.8105	0.20883
-	CDNF	10	14809245	14873098	134	22	10164	0.81001	0.20897
+	MVK	12	109538353	109607270	226	16	10164	0.80994	0.20899
+	HYAL4	7	123810169	123887481	22	2	10164	0.80965	0.20907
+	METTL7A	12	50888811	50942517	125	13	10164	0.80946	0.20912
-	CYP11B1	8	142862354	142914846	43	1	10164	0.8094	0.20914
+	HADHB	2	26209748	26300465	124	4	10164	0.80929	0.20917
+	OR1L6	9	122714848	122760783	6	3	10164	0.80926	0.20918
-	YARS	1	32765239	32853032	92	4	10164	0.80921	0.2092
+	CD151	11	797952	848835	106	9	10164	0.80896	0.20927
-	COLEC12	18	309355	535729	635	69	10164	0.80858	0.20938
-	APOL3	22	36130323	36201177	237	25	10164	0.80843	0.20942
-	RRAS2	11	14267920	14399506	112	13	10164	0.80834	0.20945
+	BIK	22	43075748	43139712	274	57	10164	0.80826	0.20947
+	SEC23B	20	18472544	18571415	333	16	10164	0.8078	0.2096
+	GLRB	4	157041125	157182099	269	16	10164	0.80771	0.20963
+	PRDX6	1	173442347	173498807	79	6	10164	0.8077	0.20963
+	CARTPT	5	71684163	71731048	102	10	10164	0.80735	0.20973
+	RTCA	1	100231158	100302769	43	12	10164	0.80708	0.20981
+	LOC105374700	5	28774035	28908807	26	3	10164	0.80706	0.20981
-	SLC27A5	19	58488333	58547065	39	4	10164	0.80688	0.20987
+	APOA1BP	1	156556766	156604299	136	16	10164	0.8068	0.20989
-	LOC105376732	15	89077561	89129089	69	10	10164	0.80661	0.20994
-	MMAB	12	109543715	109608553	202	15	10164	0.8064	0.21001
+	ADIG	20	38546195	38598463	27	1	10164	0.80626	0.21005
-	BRINP1	9	119156630	119404461	169	23	10164	0.8062	0.21006
-	TUBB2A	6	3143666	3192549	53	4	10164	0.80615	0.21008
-	ELFN2	22	37330644	37462470	337	37	10164	0.80611	0.21009
-	CYP27C1	2	127173832	127261356	30	3	10164	0.80596	0.21013
-	C16orf93	16	30747423	30797277	7	1	10164	0.80561	0.21023
-	EIF2D	1	206561297	206647551	157	26	10164	0.8053	0.21032
-	ARRB1	11	75250122	75386831	289	58	10164	0.80511	0.21038
-	CALHM2	10	103436785	103487405	101	17	10164	0.80499	0.21041
-	TUBGCP2	10	133268630	133347337	22	2	10164	0.80493	0.21043
-	CAPG	2	85384748	85453467	197	25	10164	0.80491	0.21044

STRAND	SYMBOL	CHR	START	STOP	NSNPS	NPARAM	N	ZSTAT	P
+	HOXC5	12	53981852	54045361	41	5	10164	0.8047	0.2105
+	FPR3	19	51760158	51836474	226	25	10164	0.80415	0.21066
-	TAPBP	6	33289694	33349387	213	41	10164	0.80371	0.21078
+	ZNF839	14	102282377	102353174	104	11	10164	0.80363	0.2108
-	TAP2	6	32811833	32873823	638	67	10164	0.80356	0.21083
-	FRMPD2	10	48140001	48309898	266	19	10164	0.80329	0.2109
-	DDX56	7	44555417	44609538	78	6	10164	0.80326	0.21091
+	RASSF7	11	524776	574025	202	18	10164	0.80282	0.21104
-	SGCE	7	94575224	94691209	209	28	10164	0.80274	0.21106
-	NOL6	9	33451353	33508943	105	9	10164	0.80265	0.21109
-	ANKRD26	10	26956584	27135498	481	18	10164	0.80215	0.21123
-	MST1	3	49673947	49724528	27	4	10164	0.80196	0.21129
-	XKR3	22	16773412	16856694	233	24	10164	0.80193	0.2113
+	ELFN1	7	1631056	1757954	121	9	10164	0.8016	0.21139
+	SMIM20	4	25879192	25939879	84	11	10164	0.80159	0.2114
+	FAM174A	5	100500305	100596741	102	5	10164	0.8011	0.21154
+	FOXR1	11	118936543	118991285	132	15	10164	0.80069	0.21166
-	HADHA	2	26180635	26279726	138	3	10164	0.80061	0.21168
+	MYLK2	20	31784312	31844697	87	6	10164	0.80047	0.21172
-	SIGMAR1	9	34624722	34672826	88	14	10164	0.80044	0.21173
-	OR10A6	11	7917718	7963662	183	17	10164	0.80037	0.21175
+	MARK2	11	63803928	63921020	67	11	10164	0.80026	0.21178
+	MAP3K8	10	30399003	30471833	159	14	10164	0.80018	0.2118
-	OLR1	12	10148300	10211261	218	20	10164	0.80012	0.21182
+	ZNF431	19	21107004	21196002	150	7	10164	0.7998	0.21191
+	PROC	2	127383405	127439246	112	10	10164	0.7997	0.21194
-	RBM39	20	35693609	35777336	203	8	10164	0.79972	0.21194
+	SDC2	8	96458351	96621809	326	41	10164	0.79966	0.21195
-	SAMD12	8	118121828	118656995	1382	99	10164	0.79955	0.21199
-	DSC2	18	31055974	31137432	12	3	10164	0.79928	0.21206
+	LIMK2	22	31177264	31290080	108	9	10164	0.79919	0.21209
-	COL6A3	2	237314012	237449370	478	53	10164	0.79915	0.2121
-	AAGAB	15	67190675	67290198	292	24	10164	0.79909	0.21212
+	ACVRL1	12	51871913	51933361	164	19	10164	0.79906	0.21213
-	ZNF281	1	200394947	200445058	88	14	10164	0.79906	0.21213
+	SEC62	3	169931792	170008373	236	28	10164	0.79814	0.2124
+	PHGR1	15	40316033	40366434	120	24	10164	0.79804	0.21242
+	MBD5	2	147986011	148523477	898	32	10164	0.79803	0.21243
+	YBX1	1	42647389	42712363	82	9	10164	0.79794	0.21245
-	STMN3	20	63629705	63688610	250	32	10164	0.7975	0.21258
+	NKD2	5	973820	1048812	165	18	10164	0.79744	0.2126
-	HOXA6	7	27135583	27182774	87	15	10164	0.79728	0.21265
-	SPOCD1	1	31780422	31851051	22	2	10164	0.79722	0.21266
-	ANGPTL5	11	101880674	101951715	147	17	10164	0.79648	0.21288
-	NOVA1	14	26435883	26632754	4	1	10164	0.79642	0.21289
-	C5orf45	5	179827266	179893840	148	21	10164	0.79616	0.21297
+	LYPLA2	1	23755785	23805539	6	1	10164	0.7961	0.21299
-	ZNF280A	22	22503736	22555281	23	2	10164	0.7961	0.21299
-	HECTD1	14	31090115	31243060	315	8	10164	0.79545	0.21317
+	TMEM70	8	73941142	73992783	54	4	10164	0.79535	0.2132
+	ZNF486	19	20132214	20210490	97	1	10164	0.79538	0.2132
-	PARP9	3	122517910	122599676	249	29	10164	0.79531	0.21322
+	SDCBP	8	58518169	58592860	200	14	10164	0.79514	0.21327
-	PLA2G2C	1	20153991	20210194	62	8	10164	0.79513	0.21327
+	CREB3	9	35697320	35747008	53	13	10164	0.79509	0.21328
-	ANO2	12	5550241	5983791	1072	91	10164	0.79503	0.2133
+	CNP	17	41931741	41987736	65	6	10164	0.7948	0.21337
+	OR2D3	11	6886002	6931994	65	11	10164	0.79451	0.21345
-	ALG1L	3	125918840	125987098	92	10	10164	0.7941	0.21357
+	HSPB2	11	111877736	111924093	85	10	10164	0.79398	0.2136
+	SMN1	5	70889941	70963015	1	1	10164	0.79392	0.21362
-	ALDH1A2	15	57943424	58100923	309	8	10164	0.7939	0.21363
-	ADGRF1	6	46987703	47077363	188	22	10164	0.79383	0.21365
-	PGRMC2	4	128259237	128323829	26	2	10164	0.79355	0.21373
-	COX6A2	16	31417731	31463428	7	2	10164	0.79326	0.21381
+	FREM2	13	38652036	38897131	398	39	10164	0.79324	0.21382
-	GBA2	9	35726866	35784228	61	15	10164	0.79233	0.21409
-	RTTN	18	69993804	70240726	31	2	10164	0.79199	0.21418
+	CYTH4	22	37247454	37325348	138	21	10164	0.79198	0.21418
-	FAM129B	9	127495338	127614007	402	32	10164	0.79195	0.21419
-	TXNDC12	1	52010131	52091171	93	2	10164	0.79087	0.21451
-	PARL	3	183816488	183919905	270	19	10164	0.79086	0.21451
-	FOXS1	20	31834300	31880617	41	6	10164	0.79084	0.21452
-	MYO1D	17	32482522	32912173	210	22	10164	0.79075	0.21454

STRAND	SYMBOL	CHR	START	STOP	NSNPS	NPARAM	N	ZSTAT	P
-	ZNF250	8	144866951	144937305	81	7	10164	0.79043	0.21464
+	LOC105370838	15	59994652	60051899	82	11	10164	0.79029	0.21468
+	MFSD4	1	205533984	205612918	189	32	10164	0.78938	0.21495
+	FHL5	6	96527548	96628620	123	8	10164	0.78919	0.215
-	G3BP2	4	75632769	75759525	201	15	10164	0.78862	0.21517
+	OCLM	1	186365572	186411455	38	3	10164	0.78851	0.2152
-	JMJD4	1	227721189	227770499	172	23	10164	0.78836	0.21524
+	LRRC8C	1	89580873	89729535	229	16	10164	0.78799	0.21535
-	SLC22A24	11	63069255	63179221	446	13	10164	0.788	0.21535
-	SRSF2	17	76724115	76772629	23	5	10164	0.78783	0.2154
+	LOC100289561	7	102328861	102390633	64	6	10164	0.78751	0.21549
+	SUZ12	17	31902025	32011045	52	1	10164	0.78733	0.21554
-	ATG2B	14	96271258	96398341	172	11	10164	0.78733	0.21554
+	FOXI1	5	170070897	170119725	200	25	10164	0.78707	0.21562
+	CCDC24	1	43956608	44006528	59	8	10164	0.78702	0.21563
+	S100PBP	1	32781767	32868879	86	4	10164	0.78696	0.21565
-	ZNF235	19	44276348	44340707	98	8	10164	0.78691	0.21567
-	SLC13A5	17	6674713	6748421	101	18	10164	0.78674	0.21572
+	CDK18	1	205469556	205542793	186	33	10164	0.78667	0.21574
+	LOC645177	12	24962562	25076516	208	31	10164	0.78627	0.21585
+	LOXL1	15	73891448	73962137	237	43	10164	0.786	0.21593
+	FARP2	2	241321249	241504842	431	23	10164	0.78593	0.21595
+	C1orf27	1	186340758	186431375	84	6	10164	0.78508	0.2162
-	SKAP2	7	26657062	26899743	711	17	10164	0.78503	0.21622
+	IGL	22	21991076	22932913	1092	65	10164	0.78475	0.2163
+	NKAIN3	8	62213310	63009652	2360	83	10164	0.78449	0.21638
-	IFNA4	9	21176618	21222599	244	14	10164	0.78442	0.2164
+	BTF3L4	1	52021185	52100716	106	2	10164	0.78401	0.21652
+	RASA1	5	87232801	87401926	207	9	10164	0.78376	0.21659
-	SOX7	8	10713768	10765574	230	20	10164	0.78332	0.21672
-	FAM174B	15	92607448	92847338	681	51	10164	0.78327	0.21673
-	NFU1	2	69386113	69472628	230	16	10164	0.78299	0.21682
-	GPR55	2	230897318	230996227	143	24	10164	0.78296	0.21683
+	HOXD11	2	176072356	176119588	174	42	10164	0.78265	0.21692
+	MGA	15	41586232	41779943	155	16	10164	0.78265	0.21692
+	FAM175B	10	124766785	124846670	203	46	10164	0.78234	0.21701
+	PIM1	6	37135146	37185428	131	16	10164	0.78217	0.21706
-	AP4B1	1	113884748	113940206	96	19	10164	0.78177	0.21717
+	PPIF	10	79312464	79365334	92	12	10164	0.7812	0.21734
-	SCGB3A1	5	180580105	180626487	171	25	10164	0.78102	0.21739
-	PARD6G	18	80147232	80282514	79	6	10164	0.78098	0.21741
-	MT1HL1	1	236994103	237039418	50	11	10164	0.78093	0.21742
-	TNFRSF25	1	6451151	6501195	29	4	10164	0.78081	0.21746
+	ZC3H6	2	112240559	112350063	116	4	10164	0.78011	0.21766
-	CCDC93	2	117905478	118049163	338	14	10164	0.77966	0.2178
+	FAM151B	5	80452981	80552563	200	25	10164	0.77962	0.21781
-	TNFSF18	1	173031220	173085963	91	8	10164	0.779	0.21799
-	ZNF629	16	30768449	30822277	24	1	10164	0.77895	0.21801
-	PTPN9	15	75457121	75614291	196	16	10164	0.77879	0.21805
-	ACOX2	3	58495136	58572348	62	6	10164	0.77868	0.21808
+	CDC16	13	114199845	114282675	272	23	10164	0.77866	0.21809
+	DEPDC5	22	31718951	31917034	338	20	10164	0.7784	0.21817
+	DHDH	19	48898639	48954969	113	18	10164	0.77821	0.21822
-	CPEB3	10	92038638	92326118	323	15	10164	0.77819	0.21823
+	PRAC2	17	48685809	48734758	161	32	10164	0.77817	0.21823
-	SEMA3G	3	52423252	52480027	13	6	10164	0.77808	0.21826
-	MMEL1	1	2580639	2668042	136	4	10164	0.77785	0.21833
-	DHRS12	13	51757993	51839199	90	12	10164	0.77763	0.21839
+	YPEL2	17	59296692	59411734	226	24	10164	0.77718	0.21853
-	CHST6	16	75463124	75530434	81	8	10164	0.77668	0.21867
+	BFSP2	3	133364995	133485212	192	22	10164	0.77638	0.21876
-	ADAM17	2	9479263	9590788	187	12	10164	0.77619	0.21882
-	KRT16	17	41599778	41647827	33	3	10164	0.77526	0.21909
+	PLEKHG4	16	67242820	67299500	18	3	10164	0.77526	0.21909
+	C7orf55	7	139305450	139356319	73	3	10164	0.77476	0.21924
+	MDH2	7	76013019	76077508	114	8	10164	0.7744	0.21935
-	GAB4	22	16951936	17043278	300	27	10164	0.77433	0.21937
+	EPAS1	2	46262402	46396703	420	69	10164	0.77427	0.21939
+	ARFGAP1	20	63237785	63299793	176	15	10164	0.77424	0.21939
-	RBBP9	20	18476540	18532255	178	15	10164	0.7742	0.21941
-	BTBD10	11	13377996	13498297	342	16	10164	0.77416	0.21942
-	DMKN	19	35487217	35548668	223	40	10164	0.77395	0.21948
-	INPP5E	9	136418615	136474859	143	13	10164	0.77384	0.21951
+	AP3S1	5	115806922	115924081	163	16	10164	0.77382	0.21952

STRAND	SYMBOL	CHR	START	STOP	NSNPS	NPARAM	N	ZSTAT	P
+	HIPK1	1	113894197	113987869	174	24	10164	0.77378	0.21953
+	SCEL	13	77500674	77655263	276	27	10164	0.77346	0.21962
+	TRIM6	11	5561101	5622958	352	37	10164	0.77341	0.21964
-	KCTD10	12	109438655	109512350	235	16	10164	0.77335	0.21966
+	OR13D1	9	104659422	104705462	53	5	10164	0.77301	0.21976
-	MAP4K3	2	39239266	39472312	332	17	10164	0.7727	0.21985
-	VCP	9	35046068	35107742	159	22	10164	0.77231	0.21996
+	OPN4	10	86619557	86676460	150	8	10164	0.77227	0.21998
-	SEC24D	4	118712823	118873683	600	21	10164	0.77215	0.22001
+	C2	6	31862785	31955674	487	78	10164	0.77205	0.22004
+	NANOGNB	12	7730216	7784121	13	2	10164	0.77141	0.22023
+	OLFML3	1	113944391	113992254	104	15	10164	0.77138	0.22024
-	CLMP	11	123062312	123230299	449	44	10164	0.77133	0.22026
+	KRTAP20-3	21	30607864	30653136	71	6	10164	0.77132	0.22026
+	SFT2D2	1	168191017	168252850	43	3	10164	0.77122	0.22029
-	SHOX2	3	158086011	158141202	126	10	10164	0.77106	0.22033
-	HENMT1	1	108638288	108696526	42	3	10164	0.77099	0.22036
-	CTDSP2	12	57809927	57881964	108	11	10164	0.77091	0.22038
-	PCDH1	5	141842319	141913813	103	13	10164	0.77088	0.22039
+	LOC101927572	19	35979499	36055997	92	13	10164	0.76999	0.22065
-	ARSA	22	50612754	50663173	1	1	10164	0.76972	0.22073
+	FNDC3B	3	172004628	172410703	678	60	10164	0.76935	0.22084
-	TPMT	6	18118311	18190165	21	1	10164	0.76895	0.22096
+	SYT9	11	7203796	7479045	921	86	10164	0.76867	0.22105
-	AKAP3	12	4605508	4684047	257	25	10164	0.7677	0.22133
-	DNAJC17	15	40757867	40842478	197	24	10164	0.76768	0.22134
+	CEP164	11	117286778	117423266	307	26	10164	0.76739	0.22142
-	FUT3	19	5832888	5886474	46	10	10164	0.76725	0.22146
+	CEACAM3	19	41761590	41821553	80	4	10164	0.76666	0.22164
-	HNRNPK	9	83958083	84015654	92	10	10164	0.76663	0.22165
+	ABCG1	21	42164689	42314387	223	20	10164	0.76624	0.22177
+	PPP1R11	6	30031880	30080333	541	23	10164	0.76613	0.2218
+	NXPH4	12	57181795	57236449	9	2	10164	0.7661	0.22181
-	ERICH3	1	74558111	74708738	155	15	10164	0.76596	0.22185
-	CYP11B2	8	142900558	142952862	10	1	10164	0.76578	0.2219
-	BNC2	9	16399503	16905838	1354	111	10164	0.76571	0.22193
-	PPP2R1B	11	111685074	111801445	207	14	10164	0.76565	0.22194
-	ATP5J	21	25714480	25770654	86	13	10164	0.76558	0.22196
-	OR2T33	1	248262852	248308814	136	1	10164	0.76551	0.22198
+	EED	11	86209544	86295420	203	25	10164	0.76549	0.22199
+	NISCH	3	52420508	52503072	31	12	10164	0.76475	0.22221
-	MARCH10	17	62691314	62843381	172	9	10164	0.7646	0.22226
+	C22orf15	22	23725033	23775863	156	20	10164	0.76425	0.22236
-	TMEM217	6	37202178	37294005	192	19	10164	0.76419	0.22238
+	ACTR10	14	58165115	58245636	53	4	10164	0.76401	0.22243
+	LMTK2	7	98071875	98219636	457	29	10164	0.76361	0.22255
+	SHANK3	22	50639642	50743212	35	6	10164	0.76344	0.2226
-	FAP	2	162160684	162278535	153	5	10164	0.76303	0.22272
+	TANC1	2	158933634	159242663	747	44	10164	0.76273	0.22281
-	SPI1	11	47344858	47430640	141	7	10164	0.76264	0.22284
+	FBXO25	8	371808	479876	367	21	10164	0.76232	0.22293
+	KIF1B	1	10175706	10391603	387	10	10164	0.76176	0.2231
-	FCGR3B	1	161613196	161666963	63	6	10164	0.76138	0.22321
+	SRRD	22	26448884	26501938	117	11	10164	0.76118	0.22328
-	TTI2	8	33488509	33548185	85	8	10164	0.76101	0.22333
+	HECTD2	10	91374280	91524829	165	2	10164	0.76073	0.22341
-	NPVF	7	25214570	25263486	191	23	10164	0.76039	0.22351
-	LRRFIP2	3	37042626	37211817	205	7	10164	0.76006	0.22361
-	KBTBD4	11	47562197	47614015	27	3	10164	0.75994	0.22365
+	B4GALT2	1	43944202	44001171	70	9	10164	0.75987	0.22367
-	ITIH4	3	52802990	52865701	222	13	10164	0.75958	0.22375
-	GSTA1	6	52781380	52838866	81	3	10164	0.75949	0.22378
+	C1QTNF9B-AS1	13	23853889	23902103	123	9	10164	0.75941	0.2238
-	MS4A4E	11	60191253	60278114	199	14	10164	0.75927	0.22385
-	TCFL5	20	62831014	62896763	174	19	10164	0.75903	0.22392
-	KLHL38	8	123635505	123688866	198	27	10164	0.75898	0.22393
+	LGALS12	11	63471052	63526774	55	7	10164	0.75882	0.22398
+	SLC35F1	6	117872231	118327676	1150	66	10164	0.75829	0.22414
-	LOC100507462	6	138715211	138808679	235	25	10164	0.75818	0.22417
+	RHOJ	14	63169362	63303512	360	30	10164	0.75816	0.22418
+	CIDEA	18	12219319	12287595	118	26	10164	0.75788	0.22426
-	ZFP30	19	37622487	37692285	154	17	10164	0.75778	0.22429
-	LOC105369337	11	63826865	63874417	20	5	10164	0.75692	0.22455
-	HIST1H2AD	6	26188784	26234293	232	17	10164	0.75678	0.22459

STRAND	SYMBOL	CHR	START	STOP	NSNPS	NPARAM	N	ZSTAT	P
+	C18orf63	18	74280875	74369187	361	17	10164	0.75669	0.22462
+	BCO2	11	112140485	112228929	160	14	10164	0.75667	0.22463
+	DDIAS	11	82866695	82944659	202	20	10164	0.75653	0.22466
-	ACSS1	20	24996230	25093182	107	11	10164	0.75639	0.22471
+	OS9	12	57658955	57731557	195	14	10164	0.75596	0.22484
+	GINS3	16	58357394	58416144	96	13	10164	0.7559	0.22485
-	CSNK1D	17	82229725	82308742	2	1	10164	0.75573	0.22491
+	ERMARD	6	169716622	169791584	160	9	10164	0.75546	0.22499
+	BMP1	8	22130140	22222326	64	13	10164	0.75514	0.22508
+	ELMOD1	11	107556091	107676779	150	15	10164	0.75502	0.22512
+	TRAPPC5	19	7645821	7692862	66	13	10164	0.75401	0.22542
+	SLK	10	103932182	104039233	308	17	10164	0.75387	0.22546
+	TDRD1	10	114139442	114242669	140	9	10164	0.75343	0.22559
+	TTC38	22	46232964	46304008	127	2	10164	0.75336	0.22562
-	ZFP14	19	36324453	36414203	186	13	10164	0.75322	0.22566
+	RFX6	6	116842213	116942163	341	19	10164	0.75323	0.22566
-	SLC13A3	20	46547823	46719485	161	25	10164	0.75294	0.22574
+	MXD1	2	69880041	69952945	169	17	10164	0.75292	0.22575
-	TM4SF20	2	227352156	227414306	306	32	10164	0.75289	0.22576
+	TGIF1	18	3376927	3468411	106	20	10164	0.75281	0.22578
-	PPY	17	43930804	43979248	73	8	10164	0.75279	0.22579
-	HIST1H3D	6	26186784	26234293	238	17	10164	0.75269	0.22582
+	SLX4IP	20	10399547	10648546	695	39	10164	0.75255	0.22586
+	HIST1H3E	6	26190155	26235616	232	16	10164	0.75197	0.22603
-	MAP3K14	17	45253119	45352064	192	27	10164	0.75194	0.22604
-	TNNC1	3	52441091	52489041	12	6	10164	0.75187	0.22606
-	KRTAP10-6	21	44581268	44627505	147	5	10164	0.75185	0.22607
+	LOC100996573	12	132515729	132564890	176	20	10164	0.75178	0.22609
-	EDEM3	1	184680491	184789907	268	15	10164	0.75152	0.22617
+	INHBE	12	57420313	57468008	77	13	10164	0.75145	0.22619
-	FRS3	6	41760176	41814905	85	12	10164	0.75067	0.22643
+	FOXO3	6	108524823	108694769	215	17	10164	0.75063	0.22644
+	VWA2	10	114204137	114304500	146	11	10164	0.75062	0.22644
-	DHX29	5	55246245	55342693	145	10	10164	0.74994	0.22664
-	STYXL1	7	75985834	76083003	147	10	10164	0.74968	0.22672
-	OR2C3	1	247520132	247568839	117	16	10164	0.74934	0.22683
-	NSUN6	10	18535207	18694309	312	9	10164	0.7492	0.22687
+	DCBLD1	6	117418819	117579858	606	36	10164	0.74905	0.22691
-	PTPN3	9	109365694	109573353	273	23	10164	0.74896	0.22694
-	CDC37	19	10381131	10438595	122	13	10164	0.74895	0.22694
-	CPNE4	3	131523560	132320547	2917	113	10164	0.74864	0.22704
-	GEM	8	94239253	94297319	130	16	10164	0.7484	0.22711
-	ZFP62	5	180835338	180896286	105	5	10164	0.74822	0.22716
+	UGCG	9	111861766	111945374	439	32	10164	0.74816	0.22718
+	TRIM28	19	58509469	58560715	22	4	10164	0.7478	0.22729
+	CEACAM18	19	51443547	51501261	247	19	10164	0.74779	0.22729
+	GPX3	5	150985438	151038993	145	26	10164	0.74761	0.22735
-	CABP5	19	48019383	48079054	221	22	10164	0.7475	0.22738
-	INSR	19	7102255	7329000	578	61	10164	0.74741	0.22741
+	IKZF1	7	50268465	50415101	209	23	10164	0.74717	0.22748
+	HMCN2	9	130230759	130444123	348	44	10164	0.74701	0.22753
-	ATF6B	6	32105268	32163240	210	69	10164	0.74666	0.22763
+	CAPNS2	16	55531672	55577680	94	7	10164	0.74664	0.22764
-	SBSN	19	35513367	35563351	159	26	10164	0.74653	0.22767
+	SC5D	11	121257679	121323410	204	12	10164	0.74617	0.22778
-	WIF1	12	65040624	65156566	28	5	10164	0.74606	0.22782
+	PVRL2	19	44811136	44899228	224	30	10164	0.74589	0.22787
-	TCTE3	6	169715090	169786549	142	8	10164	0.74583	0.22788
+	PTPRZ1	7	121838105	122072036	483	30	10164	0.74582	0.22789
-	GAMT	19	1387026	1436570	16	2	10164	0.74577	0.2279
-	RAVER1	19	10306212	10368638	152	19	10164	0.7456	0.22795
-	RBSN	3	15060069	15134157	162	11	10164	0.74511	0.2281
+	ZSCAN20	1	33437631	33510693	176	20	10164	0.74491	0.22816
+	NASP	1	45548988	45628906	246	15	10164	0.74484	0.22818
+	WISP2	20	44679993	44737812	40	7	10164	0.74418	0.22838
+	AGPAT5	8	6673357	6771503	202	20	10164	0.74415	0.22839
-	ARHGAP21	10	24573609	24758668	258	11	10164	0.74389	0.22847
+	GFM1	3	158609497	158702572	301	10	10164	0.74385	0.22848
-	CDPF1	22	46234013	46285679	97	2	10164	0.74369	0.22853
-	CASQ2	1	115690003	115803805	484	24	10164	0.74341	0.22862
+	IL11RA	9	34617185	34671902	99	14	10164	0.74281	0.2288
+	ISM1	20	13186771	13310650	342	43	10164	0.7427	0.22883
-	KIAA0226	3	197661393	197784727	156	3	10164	0.74265	0.22885
+	PRKRIP1	7	102361357	102436682	60	5	10164	0.74225	0.22897

STRAND	SYMBOL	CHR	START	STOP	NSNPS	NPARAM	N	ZSTAT	P
+	UBE2E1	3	23770893	23901640	267	18	10164	0.74197	0.22905
+	DUS2	16	67987944	68089323	85	3	10164	0.74189	0.22908
+	ZAN	7	100698583	100807797	16	4	10164	0.74157	0.22917
+	SOWAHA	5	132778324	132826797	88	17	10164	0.74159	0.22917
+	NOL11	17	67682833	67754208	25	4	10164	0.74156	0.22918
-	LMNTD2	11	544850	595780	140	19	10164	0.74128	0.22926
+	CCDC33	15	74182197	74346472	514	62	10164	0.74124	0.22928
-	PSMA1	11	14494876	14678634	228	23	10164	0.74119	0.22929
+	MBD6	12	57485710	57540148	94	8	10164	0.74107	0.22933
-	PIK3IP1	22	31271593	31327534	37	5	10164	0.74103	0.22934
+	BSPRY	9	113314532	113381233	271	29	10164	0.74093	0.22937
+	C17orf78	17	37340935	37402708	93	16	10164	0.74092	0.22937
+	PAPSS2	10	87624719	87757705	92	14	10164	0.74083	0.2294
+	GJB5	1	34720046	34768512	3	1	10164	0.74042	0.22952
+	GJB4	1	34724741	34773724	3	1	10164	0.74042	0.22952
+	GNAZ	22	23035483	23135034	140	8	10164	0.74037	0.22954
-	ZDHHC3	3	44905261	45011182	131	8	10164	0.74023	0.22958
-	TCEA1	8	53956554	54057456	70	7	10164	0.74016	0.2296
-	RNF6	13	26122115	26257559	466	38	10164	0.74006	0.22963
+	PCDHB13	5	141178919	141227421	132	11	10164	0.74007	0.22963
-	UBE2M	19	58545712	58593976	21	3	10164	0.73983	0.2297
+	LIPF	10	88629389	88688815	152	20	10164	0.73969	0.22974
+	OR1F1	16	3156095	3215344	90	5	10164	0.73967	0.22975
+	GTF2E1	3	120707711	120793070	126	10	10164	0.73923	0.22988
+	INSM2	14	35499042	35547054	62	12	10164	0.7391	0.22992
+	UBE3B	12	109442410	109553628	353	16	10164	0.73907	0.22993
-	PRR23B	3	139009031	139055926	78	8	10164	0.73907	0.22993
-	PRDX1	1	45501035	45557890	202	16	10164	0.73881	0.23001
+	ISLR2	15	74093203	74151841	44	16	10164	0.73859	0.23008
+	ENPP4	6	46094964	46156699	178	12	10164	0.73834	0.23015
-	RDH13	19	55031319	55104774	140	18	10164	0.73824	0.23018
-	TMED4	7	44567894	44617295	71	6	10164	0.73769	0.23035
+	PEG10	7	94621325	94679695	108	15	10164	0.73757	0.23039
-	MANBA	4	102621486	102795994	396	20	10164	0.73751	0.23041
-	DEPDC4	12	100193669	100302172	174	12	10164	0.73692	0.23059
+	RAMP3	7	45122768	45194250	343	19	10164	0.73649	0.23072
+	LOC105373474	1	210258123	210331710	142	19	10164	0.73635	0.23076
-	ASNS	7	97842117	97907542	111	19	10164	0.73625	0.23079
-	RABL3	3	120676681	120777537	116	9	10164	0.73621	0.2308
+	BPHL	6	3083376	3163198	35	5	10164	0.7359	0.2309
-	MESP1	15	89739867	89786309	137	6	10164	0.73522	0.2311
+	SLC22A4	5	132259384	132354206	378	29	10164	0.73512	0.23113
-	SLC2A5	1	9026943	9106704	272	29	10164	0.73505	0.23115
-	NGB	14	77255491	77306312	116	14	10164	0.73487	0.23121
+	DNAH11	7	21508215	21911568	2151	163	10164	0.73475	0.23125
+	KCNK2	1	214967639	215247093	654	21	10164	0.73455	0.23131
-	LOC101927160	7	76061322	76107392	74	6	10164	0.73447	0.23133
-	ACTB	7	5517148	5565601	23	3	10164	0.73432	0.23138
-	INTS7	1	211930399	212070677	348	12	10164	0.73425	0.2314
+	SLMAP	3	57721221	57940003	247	13	10164	0.73421	0.23141
+	BMPR1A	10	86720786	86935188	248	22	10164	0.73391	0.2315
-	VNN2	6	132733870	132798459	19	3	10164	0.73391	0.2315
+	PRPF38B	1	108657292	108711803	29	2	10164	0.73369	0.23157
-	RERE	1	8342404	8852691	177	8	10164	0.73332	0.23168
-	PDZD9	16	21947540	22036127	10	2	10164	0.73324	0.23171
+	DEFB125	20	52672	106573	19	6	10164	0.73306	0.23176
+	SGPL1	10	70780948	70891189	274	24	10164	0.73302	0.23177
+	AKR1A1	1	45515783	45580051	218	17	10164	0.73292	0.2318
+	HSD17B7	1	162755702	162822818	135	20	10164	0.73287	0.23182
+	ZFYVE19	15	40772076	40824569	135	18	10164	0.73285	0.23182
+	LAMC3	9	130974117	131103059	353	39	10164	0.73282	0.23183
+	LDB3	10	86631717	86746072	141	9	10164	0.73278	0.23185
+	IL24	1	206862098	206914139	99	13	10164	0.73177	0.23215
-	GNB1L	22	19778411	19889939	238	24	10164	0.73167	0.23219
+	EREG	4	74330143	74398760	320	15	10164	0.7312	0.23233
-	ARHGAP29	1	94158907	94310068	206	23	10164	0.73108	0.23236
+	PAFAH1B1	17	2558629	2695617	119	5	10164	0.73107	0.23237
+	SLAIN2	4	48306480	48436201	369	21	10164	0.73105	0.23237
-	LOC730183	16	30687704	30733489	12	1	10164	0.73069	0.23248
+	RBMS2	12	56486824	56606196	56	11	10164	0.7305	0.23254
-	RHEB	7	151456012	151554924	112	13	10164	0.73042	0.23257
-	TRPC3	4	121869028	121986891	191	29	10164	0.73038	0.23258
-	CLDN19	1	42723093	42775254	52	7	10164	0.73034	0.23259
+	NRG3	10	81840309	82997179	3539	214	10164	0.73015	0.23265

STRAND	SYMBOL	CHR	START	STOP	NSNPS	NPARAM	N	ZSTAT	P
+	IAH1	2	9439532	9527208	164	9	10164	0.7301	0.23266
+	PCDHA8	5	140806322	141022344	270	11	10164	0.72981	0.23275
-	RPL3	22	39302882	39354665	213	21	10164	0.72936	0.23289
-	HIST1H2BG	6	26206200	26251644	206	17	10164	0.72903	0.23299
+	AKIRIN1	1	38956244	39016065	132	13	10164	0.72895	0.23302
-	SIGLEC8	19	51440997	51493454	188	13	10164	0.7289	0.23303
-	IFNGR1	6	137187484	137254844	131	25	10164	0.72854	0.23314
+	SH3TC1	4	8147072	8251103	91	18	10164	0.72852	0.23315
-	KIF21B	1	200959386	201058700	275	32	10164	0.72835	0.2332
-	CIDEC	3	9856710	9915262	71	5	10164	0.72826	0.23323
+	KRTAP20-1	21	30581455	30626625	77	8	10164	0.72826	0.23323
-	AREL1	14	74651252	74748115	145	14	10164	0.72797	0.23331
-	SASS6	1	100073544	100168095	64	7	10164	0.72756	0.23344
+	DIS3L2	2	231926583	232353968	587	19	10164	0.72723	0.23354
+	VPS26B	11	134189605	134257792	84	12	10164	0.72713	0.23357
-	RNF183	9	113287093	113338274	119	14	10164	0.72699	0.23362
+	ZNF549	19	57492081	57550886	245	9	10164	0.72649	0.23377
-	STOML2	9	35089776	35138195	138	25	10164	0.72601	0.23392
-	BEND3	6	107055180	107150182	49	7	10164	0.72582	0.23397
+	LOC101060179	11	45178066	45224744	115	16	10164	0.72553	0.23406
+	MUC22	6	30970952	31045570	941	45	10164	0.72533	0.23413
-	MKL1	22	40400281	40671719	384	11	10164	0.72503	0.23422
+	GCAT	22	37772900	37827327	72	4	10164	0.72496	0.23424
-	ATG12	5	115818196	115876851	69	6	10164	0.72469	0.23432
+	CLPSL1	6	35746054	35798064	281	42	10164	0.72446	0.23439
-	DDX28	16	68011274	68058867	36	2	10164	0.72432	0.23443
-	LOC100131303	16	68003436	68050911	39	2	10164	0.72337	0.23472
-	UGT2B4	4	69470165	69561014	532	8	10164	0.72332	0.23474
+	SCAF1	19	49605503	49668649	80	12	10164	0.72265	0.23495
-	PLBD1	12	14493663	14602857	147	19	10164	0.72246	0.23501
-	ADAMTS5	21	26907912	27002120	101	9	10164	0.72241	0.23502
+	GPR107	9	130018706	130150169	130	10	10164	0.72219	0.23509
+	PRRC2C	1	171450513	171603511	402	23	10164	0.7221	0.23512
+	RGSL1	1	182414217	182570597	310	12	10164	0.72199	0.23515
-	NKG7	19	51361615	51407728	42	9	10164	0.72181	0.23521
-	SLC22A16	6	110414685	110511641	288	19	10164	0.72172	0.23523
-	GRB10	7	50580063	50828462	687	27	10164	0.72171	0.23524
+	TSPAN33	7	129109716	129179694	113	13	10164	0.7217	0.23524
-	AGER	6	32170968	32219322	281	64	10164	0.72154	0.23529
-	LOC105378192	13	112852586	112900576	95	11	10164	0.72135	0.23535
+	COLGALT1	19	17520702	17593162	120	11	10164	0.72125	0.23538
-	LMAN2L	2	96695929	96775082	77	6	10164	0.72121	0.23539
-	MMP16	8	87993095	88362488	778	77	10164	0.72118	0.2354
-	FAM186A	12	50316852	50431635	106	6	10164	0.72091	0.23548
-	RFX3	9	3208297	3561001	734	33	10164	0.72082	0.23551
-	PMS2	7	5960925	6044106	234	23	10164	0.72064	0.23557
-	MYOF	10	93296429	93517486	560	51	10164	0.72062	0.23557
+	GAPDHS	19	35498412	35555319	191	32	10164	0.72037	0.23565
+	CNBD2	20	35919562	36040700	172	15	10164	0.72016	0.23571
+	CHRNA9	4	40300329	40365217	168	9	10164	0.72013	0.23572
-	ITFG1	16	47144387	47496274	44	3	10164	0.71937	0.23596
-	EPC1	10	32257716	32413798	355	19	10164	0.71906	0.23605
-	PRICKLE1	12	42448338	42624770	550	41	10164	0.719	0.23607
+	CHD7	8	60643762	60878028	478	29	10164	0.71887	0.23611
+	MESP2	15	89741358	89788754	142	6	10164	0.71842	0.23625
+	SGSM1	22	24771221	24936846	651	65	10164	0.71814	0.23634
-	CNFN	19	42377019	42425317	30	1	10164	0.71805	0.23636
-	XPO4	13	20767329	20937774	333	17	10164	0.71791	0.23641
+	OR1K1	9	122765123	122811073	17	2	10164	0.71789	0.23641
-	C17orf98	17	38815392	38876500	141	15	10164	0.71788	0.23642
-	CLLU1OS	12	92410094	92463545	58	7	10164	0.71783	0.23643
+	PIGW	17	36500002	36549368	22	3	10164	0.71769	0.23647
-	SLC35F4	14	57553922	58017274	1675	82	10164	0.71764	0.23649
-	PRR18	6	166295680	166343383	152	9	10164	0.71708	0.23666
-	COX6C	8	99867995	99929014	78	6	10164	0.71703	0.23668
+	HIST1H4F	6	26205426	26250793	208	17	10164	0.71682	0.23674
-	ENHO	9	34511042	34558039	76	13	10164	0.71673	0.23677
+	ATRN	20	3436018	3661122	264	24	10164	0.71665	0.2368
-	ACBD6	1	180277385	180537967	163	6	10164	0.71647	0.23685
+	ROPN1B	3	125934144	125993454	99	9	10164	0.71633	0.23689
+	PTCD2	5	72285367	72378397	65	16	10164	0.71617	0.23694
-	TRPV3	17	3500502	3592995	246	35	10164	0.71595	0.23701
+	PNN	14	39140183	39193218	100	12	10164	0.71593	0.23702
-	C1orf127	1	10936473	11017037	73	11	10164	0.71585	0.23704

STRAND	SYMBOL	CHR	START	STOP	NSNPS	NPARAM	N	ZSTAT	P
-	SPATA9	5	95642181	95718010	172	11	10164	0.71575	0.23707
+	DMRTA1	9	22411841	22462473	223	23	10164	0.71544	0.23717
-	SLCO5A1	8	69657046	69870064	452	45	10164	0.71508	0.23728
+	ZNF638	2	71296755	71445061	350	16	10164	0.71454	0.23745
-	SLC41A3	3	125996357	126136550	456	19	10164	0.71451	0.23745
-	KCNH4	17	42146892	42216278	78	11	10164	0.71425	0.23754
-	MIF4GD	17	75256228	75306292	149	9	10164	0.71423	0.23754
-	OR2T12	1	248284616	248330578	192	1	10164	0.71422	0.23755
+	SMYD5	2	73179238	73237227	82	4	10164	0.71356	0.23775
-	GTSCR1	18	70620519	70705875	232	24	10164	0.71354	0.23775
+	RHNO1	12	2841258	2899525	142	10	10164	0.71328	0.23784
+	MPP6	7	24538346	24703703	335	29	10164	0.71293	0.23794
+	HEATR3	16	50030970	50115481	202	9	10164	0.71276	0.238
-	GPRIN3	4	89231276	89343010	217	35	10164	0.71274	0.238
+	TOR3A	1	179046977	179105996	186	21	10164	0.71259	0.23805
-	NATD1	17	21228870	21288266	80	12	10164	0.71255	0.23806
+	DGAT2	11	75733733	75811536	199	18	10164	0.71253	0.23807
-	CRP	1	159702289	159749609	128	14	10164	0.71243	0.2381
+	NAT8L	4	2024512	2079089	40	6	10164	0.71237	0.23812
-	GALNT6	12	51341870	51426955	195	31	10164	0.71229	0.23814
+	TLR3	4	186034155	186095098	228	34	10164	0.71225	0.23815
-	MYO19	17	36485755	36570457	29	4	10164	0.71213	0.23819
-	WDR31	9	113305265	113375340	252	24	10164	0.71207	0.23821
-	EPPK1	8	143847319	143913464	27	4	10164	0.71197	0.23824
-	ZNF764	16	30543764	30593321	3	1	10164	0.71165	0.23834
-	XXYLT1	3	195058276	195306166	432	21	10164	0.71139	0.23842
+	LOC105372481	19	58270337	58325653	77	8	10164	0.71099	0.23855
+	RPL10A	6	35433401	35480781	244	45	10164	0.71079	0.23861
+	RNF40	16	30726612	30812340	22	1	10164	0.71045	0.23871
+	MURC	9	100541867	100598402	139	15	10164	0.70993	0.23887
+	AARD	8	116903225	116954000	173	18	10164	0.70987	0.23889
+	ZSCAN4	19	57616504	57689152	194	11	10164	0.7098	0.23891
+	SERPIND1	22	20739095	20797720	4	1	10164	0.70973	0.23894
-	FCMR	1	206893285	206957033	116	14	10164	0.70949	0.23901
-	AP3D1	19	2090988	2186620	227	18	10164	0.70929	0.23907
-	LOC101928344	19	194025	281533	18	2	10164	0.70924	0.23909
-	WDR45B	17	82604562	82683535	125	13	10164	0.70921	0.2391
+	TANGO2	22	19982000	20075926	346	36	10164	0.70913	0.23912
+	SLC1A3	5	36571355	36698334	307	40	10164	0.70903	0.23915
+	CHMP1B	18	11816390	11864449	100	6	10164	0.70859	0.23929
-	CLSTN1	1	9719021	9859526	246	7	10164	0.70843	0.23934
+	CCNF	16	2394394	2468858	1	1	10164	0.70836	0.23936
+	NTN3	16	2436499	2484145	1	1	10164	0.70836	0.23936
+	C16orf59	16	2425111	2474963	1	1	10164	0.70836	0.23936
+	RASA2	3	141452047	141625363	443	20	10164	0.70772	0.23956
+	EXOSC9	4	121766295	121827021	172	21	10164	0.70749	0.23963
+	BCS1L	2	218624656	218673443	75	3	10164	0.70737	0.23967
-	ZBTB44	11	130216677	130349769	154	15	10164	0.7072	0.23972
+	LMLN	3	197925200	198053720	278	19	10164	0.70695	0.2398
-	FOXD4	9	106231	153417	48	4	10164	0.7068	0.23985
+	WIPI2	7	5155204	5243855	144	14	10164	0.70672	0.23987
-	ARAP2	4	36055998	36279357	453	45	10164	0.70645	0.23995
+	GATA3	10	8010428	8085201	275	55	10164	0.70639	0.23997
-	CRABP2	1	156689606	156740816	90	23	10164	0.70629	0.24001
-	RHBDL2	1	38875806	38976830	247	28	10164	0.70619	0.24003
+	RBM48	7	92493692	92549405	108	14	10164	0.7062	0.24003
+	SYNGR4	19	48329281	48386377	47	4	10164	0.70601	0.24009
-	C11orf71	11	114381448	114435550	77	7	10164	0.70595	0.24011
+	SLC30A5	5	69058949	69141072	150	7	10164	0.70586	0.24014
-	TMPRSS6	22	37055436	37145635	91	15	10164	0.7054	0.24028
-	STK10	5	172032070	172223342	305	29	10164	0.70503	0.2404
+	TSC2	16	2012936	2098712	71	17	10164	0.70501	0.2404
-	OGDHL	10	49724641	49797379	215	19	10164	0.70496	0.24042
-	ADH7	4	99402261	99470510	194	19	10164	0.70475	0.24048
+	PHLPP1	18	62680439	62990443	198	11	10164	0.70477	0.24048
+	MCEMP1	19	7642057	7689833	78	14	10164	0.70472	0.24049
-	GALE	1	23785599	23835804	8	1	10164	0.70413	0.24068
-	STARD13	13	33093135	33711835	1654	123	10164	0.70412	0.24068
-	KIAA1598	10	116873377	117161586	287	22	10164	0.70404	0.2407
+	PRKCZ	1	2015470	2195395	604	37	10164	0.70385	0.24076
+	COL18A1	21	45370137	45523720	464	44	10164	0.70386	0.24076
-	LOC105376854	18	498396	566912	7	1	10164	0.70386	0.24076
+	TNRC6C	17	77969170	78118835	272	18	10164	0.70365	0.24083
+	ARMC5	16	31423273	31477167	13	3	10164	0.70363	0.24083

STRAND	SYMBOL	CHR	START	STOP	NSNPS	NPARAM	N	ZSTAT	P
+	BTNL8	5	180864077	180962166	262	10	10164	0.70362	0.24084
-	POLE2	14	49633552	49723417	131	5	10164	0.70347	0.24088
+	METTL14	4	118650364	118722270	151	17	10164	0.70339	0.24091
+	SRCAP	16	30664141	30750129	20	1	10164	0.70333	0.24093
-	POLR2D	2	127836266	127893155	109	13	10164	0.70324	0.24095
+	LOC105373091	22	49814635	49861067	140	13	10164	0.70305	0.24101
+	HOXD12	2	176063513	176113770	204	45	10164	0.70302	0.24102
+	HHAT	1	210293252	210686298	1287	113	10164	0.70284	0.24108
+	PSMD3	17	39945768	40007960	213	16	10164	0.7026	0.24115
-	PYY	17	43942733	44059260	148	13	10164	0.7019	0.24137
-	KRTDAP	19	35477324	35525531	181	36	10164	0.70172	0.24143
-	OR52E2	11	5048650	5094627	266	19	10164	0.70167	0.24144
+	C11orf52	11	111883877	111936871	90	10	10164	0.70153	0.24149
+	GP9	3	129020449	129072411	14	3	10164	0.7014	0.24153
-	BCL2L1	20	31654452	31758964	113	6	10164	0.70093	0.24167
+	DFNB59	2	178415592	178471390	54	7	10164	0.70076	0.24173
+	POLR1D	13	27585743	27677422	231	27	10164	0.70064	0.24176
-	POLN	4	2061958	2277164	506	13	10164	0.70032	0.24186
+	ADGRD1	12	130918907	131151465	619	49	10164	0.70022	0.2419
-	NUAK1	12	106053347	106175033	202	17	10164	0.70014	0.24192
+	VASH1	14	76726892	76793022	89	14	10164	0.70006	0.24195
-	MOK	14	102214450	102340200	216	14	10164	0.69967	0.24207
+	LIMCH1	4	41324607	41710044	392	34	10164	0.69948	0.24212
-	DNAJC19	3	180973709	181024774	59	5	10164	0.69927	0.24219
-	TPM2	9	35671993	35725056	105	21	10164	0.69878	0.24234
-	TCEB1	8	73935138	74007287	62	5	10164	0.69862	0.24239
+	RWDD2A	6	83158313	83208932	114	21	10164	0.69838	0.24247
+	AIMP2	7	5974251	6033834	202	22	10164	0.69835	0.24248
-	UBQLN3	11	5497300	5544985	213	26	10164	0.69831	0.24249
-	FECH	18	57534841	57621737	255	36	10164	0.69821	0.24252
-	CCDC179	11	22836922	22895486	171	11	10164	0.69802	0.24258
+	MUC21	6	30948708	30998637	372	34	10164	0.69766	0.2427
+	SERPINA5	14	94546369	94603120	211	20	10164	0.69726	0.24282
+	LOC101927844	1	87177669	87261331	101	9	10164	0.69721	0.24284
-	LOC105369212	15	96071411	96187989	228	25	10164	0.69635	0.2431
+	SORD	15	44988104	45085089	114	12	10164	0.69629	0.24312
-	LRRC39	1	100138448	100213273	29	6	10164	0.6962	0.24315
-	COL28A1	7	7328549	7572277	812	30	10164	0.6961	0.24318
-	MCHR2	6	99909910	100029238	103	10	10164	0.69595	0.24323
-	CEP70	3	138484339	138629383	2	1	10164	0.69574	0.24329
-	ADAMTS1	21	26826287	26880409	152	16	10164	0.69573	0.2433
-	SMG9	19	43717992	43789990	130	15	10164	0.69562	0.24333
+	TBC1D31	8	123037680	123175173	160	25	10164	0.69559	0.24334
+	CHAMP1	13	114279490	114337328	169	16	10164	0.69543	0.24339
-	APLNR	11	57223577	57272453	98	9	10164	0.69532	0.24343
+	TAC1	7	97696959	97750472	15	2	10164	0.69496	0.24354
+	WASF3	13	26522703	26698945	471	20	10164	0.69496	0.24354
-	FIGNL1	7	50434129	50485390	166	15	10164	0.69493	0.24355
-	TP53I3	2	24067428	24120215	169	7	10164	0.69482	0.24359
-	IZUMO2	19	50142548	50198281	5	3	10164	0.6948	0.24359
-	ILDR1	3	121977323	122095516	236	35	10164	0.69444	0.2437
-	POLR3H	22	41515799	41579606	99	7	10164	0.69424	0.24377
+	FSD1	19	4269594	4333846	22	4	10164	0.69419	0.24378
-	TMEM54	1	32884595	32936435	36	3	10164	0.69421	0.24378
-	OR51V1	11	5189735	5235700	115	11	10164	0.69398	0.24385
+	NFIA	1	61042257	61472788	597	81	10164	0.69362	0.24396
+	MPRIP	17	17007468	17202648	75	2	10164	0.69359	0.24397
+	ABCF3	3	184151075	184204452	59	6	10164	0.69357	0.24398
-	KRTAP22-2	21	30580105	30625397	79	9	10164	0.69346	0.24401
-	TMEM220	17	10703322	10765329	15	1	10164	0.69341	0.24403
-	C15orf56	15	40240664	40287909	122	25	10164	0.69319	0.24409
-	CHMP2A	19	58541566	58590128	22	3	10164	0.69316	0.2441
-	GMNC	3	190833268	190897743	125	14	10164	0.69313	0.24411
+	LOC441098	5	100353304	100402932	54	6	10164	0.69289	0.24419
+	CA6	1	8910785	8985092	159	33	10164	0.69276	0.24423
-	MRPS24	7	43856558	43904546	121	15	10164	0.69274	0.24424
+	GEMIN2	14	39079284	39146973	113	13	10164	0.69263	0.24427
+	FBLN2	3	13514125	13648422	493	77	10164	0.69256	0.24429
+	DLAT	11	111989814	112074278	61	3	10164	0.69204	0.24446
+	FSCN2	17	81478051	81549790	194	13	10164	0.69187	0.24451
+	SNX2	5	122739996	122844539	262	16	10164	0.69179	0.24453
+	ASS1	9	130409707	130511274	247	34	10164	0.69176	0.24454
+	LRRC37B	17	31972372	32063500	8	1	10164	0.69147	0.24464
+	TPX2	20	31698819	31811802	124	6	10164	0.69128	0.2447

STRAND	SYMBOL	CHR	START	STOP	NSNPS	NPARAM	N	ZSTAT	P
+	KCNRG	13	49980254	50030922	79	9	10164	0.69107	0.24476
+	SRRT	7	100839971	100898664	107	13	10164	0.69092	0.24481
-	NRDE2	14	90268054	90367137	2	1	10164	0.6908	0.24485
-	GJD2	15	34742441	34789581	132	16	10164	0.69079	0.24485
-	ARHGAP39	8	144519179	144735520	157	7	10164	0.69072	0.24487
+	ATP10D	4	47450271	47603486	212	23	10164	0.6906	0.24491
+	LOC388780	20	2171468	2268031	240	25	10164	0.69061	0.24491
+	LCN1	9	135486297	135536540	105	9	10164	0.68999	0.2451
-	LYG2	2	99188503	99291732	128	12	10164	0.68978	0.24516
+	AMD1	6	110779621	110905713	208	13	10164	0.68971	0.24519
-	MCTP1	5	94693690	95319575	1375	105	10164	0.68935	0.2453
+	SLC1A4	2	64953445	65033865	76	14	10164	0.68919	0.24535
+	ZIC1	3	147374394	147426719	62	8	10164	0.68898	0.24542
+	COL4A3	2	227129565	227324792	920	82	10164	0.68853	0.24556
+	C14orf166	14	51954510	52014702	173	23	10164	0.68824	0.24565
-	BSPH1	19	47958046	48027170	174	26	10164	0.68803	0.24572
+	ANKRD61	7	5996376	6046552	186	20	10164	0.68791	0.24575
-	ZSCAN5A	19	56211303	56265399	28	4	10164	0.68751	0.24588
+	NFATC3	16	68050366	68239259	175	2	10164	0.68742	0.24591
-	RALGAPA2	20	20379558	20747638	384	9	10164	0.68738	0.24592
+	GUCY1A3	4	155631568	155747062	240	26	10164	0.68716	0.24599
+	RDH8	19	9978249	10032278	6	1	10164	0.68715	0.24599
+	SGPP2	2	222389052	222571842	253	35	10164	0.68718	0.24599
+	CCDC60	12	119299712	119551047	569	80	10164	0.68706	0.24602
-	LIN7C	11	27484418	27541779	83	11	10164	0.68699	0.24605
+	TMEM97	17	28284095	28338685	34	5	10164	0.68689	0.24607
+	BIRC3	11	102282373	102349403	175	19	10164	0.68657	0.24618
-	TMEM41A	3	185472671	185534057	80	5	10164	0.6865	0.2462
+	DYSF	2	71418119	71696763	552	62	10164	0.68627	0.24627
+	SERTAD4	1	210197850	210256858	108	12	10164	0.68606	0.24634
-	HOXB4	17	48565507	48613381	141	29	10164	0.68603	0.24635
-	MYLK	3	123602296	123919302	268	28	10164	0.68597	0.24636
+	CYP26A1	10	93038475	93087884	110	17	10164	0.68596	0.24637
+	CPT1C	19	49655878	49723731	136	18	10164	0.68594	0.24638
+	SLC39A12	10	17916839	18053292	642	45	10164	0.68582	0.24641
-	XPA	9	97644398	97732409	292	14	10164	0.68552	0.24651
+	MFSD5	12	53216251	53264406	82	12	10164	0.6854	0.24655
-	LOC105373780	2	184583627	184633985	91	13	10164	0.68534	0.24656
+	ANKRD6	6	89398178	89643834	464	34	10164	0.6852	0.24661
-	ETFA	15	76206287	76346469	247	16	10164	0.68492	0.2467
-	SOX10	22	37962312	38019532	45	5	10164	0.6845	0.24683
-	ZNF23	16	71437597	71497252	91	12	10164	0.68422	0.24692
+	LOC102723422	10	1003454	1050858	156	22	10164	0.68411	0.24695
-	MYLK4	6	2653629	2805123	535	38	10164	0.68398	0.247
-	CLNK	4	10479900	10769853	1108	76	10164	0.68383	0.24704
+	ZNF831	20	59088428	59269113	142	14	10164	0.68382	0.24704
-	SLC35D2	9	96303437	96418710	175	17	10164	0.68375	0.24706
-	TMEM52	1	1907589	1954301	1	1	10164	0.68347	0.24715
-	HPS4	22	26433613	26518863	192	16	10164	0.68287	0.24734
+	SOCS2	12	93534822	93636236	215	32	10164	0.68267	0.24741
-	TFAP2A	6	10386677	10454624	172	32	10164	0.68243	0.24748
-	FAM65C	20	50576108	50727004	86	19	10164	0.68241	0.24749
-	OTOF	2	26447203	26593698	34	8	10164	0.68221	0.24755
+	PTCHD2	1	11444238	11547583	102	7	10164	0.68221	0.24755
+	FLRT1	11	64000970	64129183	248	48	10164	0.68212	0.24758
-	HACD2	3	123484516	123620077	197	13	10164	0.68194	0.24764
-	BCL10	1	85255776	85311904	176	25	10164	0.68187	0.24766
+	HLF	17	55229960	55335065	187	38	10164	0.68174	0.2477
+	KRTAP5-5	11	1594803	1640930	262	23	10164	0.68166	0.24773
+	LYPD6B	2	149003467	149225262	634	27	10164	0.6816	0.24774
-	ILVBL	19	15104974	15160799	91	6	10164	0.68159	0.24775
-	GOLGA8B	15	34515276	34618651	158	12	10164	0.68131	0.24784
-	C1QTNF4	11	47579664	47629409	24	3	10164	0.68086	0.24798
-	PLXND1	3	129545213	129641739	224	18	10164	0.68041	0.24812
+	SGCD	5	156082934	156777788	1346	54	10164	0.68013	0.24821
-	ELF2	4	139047717	139212218	146	12	10164	0.68012	0.24822
-	HBD	11	5222829	5269628	104	6	10164	0.67979	0.24832
-	ZNF585A	19	37137438	37207741	114	11	10164	0.67955	0.24839
+	CCDC64	12	119954799	120104495	260	15	10164	0.67951	0.24841
+	RALA	7	39587955	39718124	145	13	10164	0.67934	0.24846
+	RPP38	10	15062180	15114257	160	18	10164	0.6793	0.24848
+	HELLS	10	94510767	94612099	213	16	10164	0.67911	0.24853
+	RXFP3	5	33901386	33948918	141	22	10164	0.67894	0.24859
+	TIPRL	1	168143845	168212114	123	4	10164	0.67892	0.2486

STRAND	SYMBOL	CHR	START	STOP	NSNPS	NPARAM	N	ZSTAT	P
-	SYNC	1	32669906	32737760	153	8	10164	0.67888	0.24861
-	DPH6	15	35294893	35581222	408	23	10164	0.67798	0.24889
+	SPX	12	21491322	21541276	172	23	10164	0.67786	0.24893
-	LIPE	19	42391507	42462426	3	1	10164	0.6775	0.24904
-	FHOD1	16	67219389	67282522	26	3	10164	0.67753	0.24904
+	XRN2	20	21268304	21399825	192	9	10164	0.67747	0.24905
+	DOT1L	19	2129127	2242578	267	19	10164	0.67734	0.2491
-	TBC1D27	17	16912923	16964592	135	18	10164	0.67717	0.24915
+	CAP2	6	17358216	17567792	309	35	10164	0.67711	0.24917
+	FAM149A	4	186109287	186182663	258	30	10164	0.67691	0.24923
-	IGF2	11	2119112	2184603	226	38	10164	0.67651	0.24936
-	PPP2CA	5	134186457	134261259	157	8	10164	0.67643	0.24938
+	WISP3	6	112018516	112079686	156	31	10164	0.67639	0.2494
+	DAZAP1	19	1372569	1445687	22	2	10164	0.67634	0.24941
+	LMCD1	3	8466807	8578125	273	20	10164	0.67633	0.24942
+	LOC105376855	18	12396366	12446206	115	20	10164	0.67589	0.24955
-	HLA-C	6	31258749	31307136	1761	44	10164	0.67529	0.24975
-	IPMK	10	58181517	58302937	314	4	10164	0.67515	0.24979
-	DNAL4	22	38768508	38829156	99	13	10164	0.67506	0.24982
+	CSDC2	22	41526010	41586666	99	8	10164	0.67504	0.24982
+	TMEM136	11	120289630	120343688	112	17	10164	0.67484	0.24989
+	UMAD1	7	7605636	7889223	1036	77	10164	0.67477	0.24991
-	WDR34	9	128623661	128691850	62	7	10164	0.67442	0.25002
+	CTTN	11	70363506	70446584	117	9	10164	0.67425	0.25008
+	YEATS4	12	69324700	69434838	123	14	10164	0.67403	0.25015
+	LYRM1	16	20863805	20935006	5	1	10164	0.67403	0.25015
-	LSS	21	46178446	46263824	295	23	10164	0.67385	0.2502
-	MEF2C	5	88708241	88939105	257	20	10164	0.67382	0.25021
-	NKAIN4	20	63230784	63289540	176	16	10164	0.67377	0.25023
+	GPR162	12	6786797	6837418	90	13	10164	0.67372	0.25024
-	RPP40	6	4982414	5039063	157	11	10164	0.6735	0.25031
-	HDGF	1	156732107	156787448	104	12	10164	0.67336	0.25036
+	GLI2	2	120762291	121002653	100	12	10164	0.67328	0.25038
-	ASB3	2	53659980	53822009	473	27	10164	0.6731	0.25044
+	SAMD4B	19	39307459	39400760	8	3	10164	0.67304	0.25046
-	CPNE8	12	38642200	38941135	628	22	10164	0.67303	0.25046
+	NUPL1	13	25266084	25359795	21	2	10164	0.67299	0.25048
+	HOXD13	2	176052505	176105938	234	48	10164	0.67283	0.25053
-	OR2M7	1	248313630	248359568	101	1	10164	0.67248	0.25064
+	PRELID1	5	177268762	177316959	53	5	10164	0.67231	0.25069
+	OR14C36	1	248313775	248359713	100	1	10164	0.67229	0.2507
-	OR1G1	17	3116505	3163253	119	9	10164	0.67192	0.25082
-	TUBA3C	13	19163770	19216852	80	4	10164	0.67179	0.25086
+	FBXO22	15	75868859	75945268	51	8	10164	0.67178	0.25086
+	DNAH14	1	224894595	225409294	1694	35	10164	0.67136	0.251
-	EIF2B1	12	123611023	123668776	55	5	10164	0.67087	0.25115
+	AFMID	17	78150102	78217701	259	36	10164	0.67086	0.25115
+	C2orf70	2	26527556	26589529	28	6	10164	0.67089	0.25115
+	PLCB3	11	64216523	64279452	174	32	10164	0.67083	0.25116
+	PRPF4	9	113240634	113303914	171	17	10164	0.67078	0.25118
-	OSBPL3	7	24786537	25015212	124	21	10164	0.67077	0.25118
+	TRIM13	13	49962007	50028467	98	10	10164	0.67075	0.25119
+	FAM181A	14	93883894	93939608	182	30	10164	0.67061	0.25124
-	TIMD4	5	156909282	157007310	87	6	10164	0.67045	0.25128
-	RGL2	6	33281654	33334452	193	40	10164	0.67023	0.25136
+	ATP6V0B	1	43939648	43988300	66	8	10164	0.67007	0.25141
-	LOC105378592	1	2198942	2250211	97	19	10164	0.67007	0.25141
+	RPS15	19	1403364	1450494	24	2	10164	0.66984	0.25148
-	MAGOH	1	53216892	53273610	110	17	10164	0.6698	0.25149
+	PLG	6	160667193	160764054	332	43	10164	0.66973	0.25152
+	CALR	19	12903600	12954490	4	1	10164	0.66941	0.25162
-	KCTD19	16	67279432	67361761	8	3	10164	0.669	0.25175
-	SF3B6	2	24057584	24111444	165	8	10164	0.66884	0.2518
+	GOLGA6L9	15	82382678	82449387	93	8	10164	0.66872	0.25184
-	ROPN1	3	123959006	124027170	76	8	10164	0.66867	0.25185
-	ACTG1	17	81499971	81547866	138	12	10164	0.66861	0.25187
+	CAPN11	6	44123811	44194402	37	5	10164	0.6686	0.25188
-	ZNF202	11	123713927	123776683	176	14	10164	0.66843	0.25193
+	PHOSPHO2	2	169659454	169711708	8	1	10164	0.66833	0.25196
-	ATP8B4	15	49848238	50216793	954	67	10164	0.66824	0.25199
-	PI4KA	22	20697691	20893812	12	2	10164	0.66811	0.25203
-	LOC105369153	15	45222945	45310437	229	5	10164	0.66799	0.25207
+	GPC1	2	240400698	240478078	297	28	10164	0.66766	0.25218
-	ATP13A2	1	16975958	17046972	124	8	10164	0.66758	0.2522

STRAND	SYMBOL	CHR	START	STOP	NSNPS	NPARAM	N	ZSTAT	P
+	TSGA10IP	11	65910445	65970392	121	20	10164	0.6675	0.25223
-	ZNF835	19	56652652	56706783	79	13	10164	0.66721	0.25232
-	SCO1	17	10670332	10732568	9	1	10164	0.66708	0.25236
-	BARHL2	1	90702022	90752237	16	2	10164	0.66632	0.2526
-	POMT2	14	77264956	77355884	194	18	10164	0.66621	0.25264
+	ECT2L	6	138761051	138914070	438	42	10164	0.66619	0.25265
+	APOC1	19	44879325	44929349	115	16	10164	0.66591	0.25274
-	LOC105370849	15	62239533	62291938	127	14	10164	0.66537	0.25291
+	FAM213B	1	2551460	2601469	125	7	10164	0.66499	0.25303
-	LRRC75B	22	24575623	24628104	81	6	10164	0.66493	0.25305
+	PHKG2	16	30713299	30771176	10	1	10164	0.66478	0.2531
-	FCRL3	1	157666481	157735985	173	19	10164	0.66454	0.25317
-	ACP5	19	11564660	11613986	37	5	10164	0.66445	0.2532
-	ESR2	14	64162925	64373550	446	27	10164	0.66436	0.25323
+	CISD1	10	58234102	58299259	176	3	10164	0.66428	0.25326
-	LPPR3	19	802488	859243	59	6	10164	0.66422	0.25328
+	SLC28A2	15	45217066	45285934	204	4	10164	0.66414	0.2533
+	CYLD	16	50707050	50811936	178	15	10164	0.6636	0.25347
-	SOHLH2	13	36158208	36249615	96	16	10164	0.66356	0.25349
-	SDCCAG3	9	136391922	136445602	188	16	10164	0.66334	0.25356
-	YOD1	1	207033849	207088133	82	11	10164	0.66324	0.25359
-	C9orf114	9	128809651	128864847	74	6	10164	0.66302	0.25366
+	PPCDC	15	74988555	75060726	229	23	10164	0.66283	0.25372
+	PRR3	6	30521709	30574696	199	35	10164	0.66246	0.25384
-	CCDC38	12	95857048	95978254	511	34	10164	0.6623	0.25389
-	USP12	13	27056150	27206896	367	27	10164	0.6621	0.25395
-	UNC13A	19	17591328	17723353	115	15	10164	0.66196	0.254
+	FAM26F	6	116426370	116473782	124	21	10164	0.66194	0.254
-	KRTAP19-7	21	30551098	30596289	132	11	10164	0.6618	0.25405
+	GPR65	14	87970124	88024811	297	21	10164	0.66164	0.2541
-	FABP3	1	31350418	31408283	46	4	10164	0.6616	0.25411
+	C9orf47	9	88955863	89006142	121	11	10164	0.66154	0.25413
-	TRA2B	3	185904568	185973136	132	17	10164	0.66145	0.25416
-	TMED5	1	93139742	93215689	90	6	10164	0.66053	0.25446
-	SCRT2	20	651596	711179	136	26	10164	0.66053	0.25446
+	GTF3A	13	27389544	27445709	180	14	10164	0.66039	0.2545
+	AEN	15	88569681	88642281	187	22	10164	0.66038	0.2545
-	SFRP5	10	97756751	97806999	166	17	10164	0.66025	0.25455
-	ABLIM2	4	7955310	8193832	686	107	10164	0.65985	0.25467
-	NUFIP2	17	29245836	29329148	3	1	10164	0.65966	0.25474
+	ROMO1	20	35664310	35710980	105	10	10164	0.65966	0.25474
-	ASGR1	17	7163431	7214564	73	16	10164	0.65902	0.25494
+	MDM2	12	68773168	68855544	135	17	10164	0.65891	0.25498
-	FARSA	19	12912470	12968744	4	1	10164	0.65873	0.25503
+	RAD23A	19	12910814	12963643	4	1	10164	0.65873	0.25503
+	PRDM2	1	13665240	13835079	392	35	10164	0.65869	0.25505
+	KLHDC1	14	49658105	49763152	100	6	10164	0.65842	0.25513
-	MTMR6	13	25236201	25322566	15	2	10164	0.65837	0.25515
+	SGIP1	1	66498311	66758999	795	30	10164	0.65795	0.25528
-	CLDND2	19	51356957	51404241	65	11	10164	0.6578	0.25533
+	SWSAP1	19	11339707	11386951	9	1	10164	0.65775	0.25535
-	TCF3	19	1599290	1687426	59	9	10164	0.65712	0.25555
-	MFAP5	12	8635943	8697888	41	5	10164	0.65712	0.25555
-	GADD45GIP1	19	12944156	12992254	3	1	10164	0.65687	0.25563
+	DAND5	19	12934618	12984753	3	1	10164	0.65687	0.25563
+	UPK2	11	118921298	118968559	103	20	10164	0.65645	0.25577
-	DENND4B	1	153919501	153981681	60	5	10164	0.65629	0.25582
-	ZNF200	16	3212325	3271221	71	4	10164	0.65615	0.25587
+	DAGLA	11	61645433	61757002	176	21	10164	0.65606	0.25589
-	MKI67	10	128086659	128161363	314	28	10164	0.65599	0.25592
+	MYO16	13	108460729	109218007	2190	153	10164	0.65584	0.25596
+	CFB	6	31910944	31962084	259	66	10164	0.65575	0.25599
-	PHLDA2	11	2918273	2964420	99	9	10164	0.65564	0.25603
+	GSTZ1	14	77285887	77341597	119	13	10164	0.65562	0.25604
-	GRM6	5	178968327	179030123	247	22	10164	0.65541	0.2561
+	KIAA1033	12	105072714	105179135	264	20	10164	0.65522	0.25616
-	ANGPTL2	9	127077349	127157797	132	16	10164	0.65473	0.25632
+	NPNT	4	105859526	105981671	57	6	10164	0.65468	0.25634
-	DCUN1D3	16	20848074	20935239	6	1	10164	0.65431	0.25646
+	ZNF398	7	149091416	149193042	175	6	10164	0.6542	0.25649
-	SPATA21	1	16377117	16472842	122	11	10164	0.65414	0.25651
-	ANGPTL6	19	10082337	10141209	28	4	10164	0.65375	0.25664
+	LOC105375667	8	100071005	100117768	90	11	10164	0.65356	0.2567
+	CD86	3	122020362	122131143	215	36	10164	0.65345	0.25673

STRAND	SYMBOL	CHR	START	STOP	NSNPS	NPARAM	N	ZSTAT	P
+	SPAM1	7	123890232	123981410	84	5	10164	0.65345	0.25673
+	GSKIP	14	96328452	96397290	66	7	10164	0.65345	0.25673
-	OR9I1	11	58108500	58154444	192	24	10164	0.65347	0.25673
+	PMPCA	9	136375573	136433761	233	17	10164	0.65339	0.25675
-	LOC105377819	13	18952129	19054869	338	13	10164	0.65324	0.2568
+	SUPT5H	19	39410546	39486670	51	6	10164	0.65298	0.25689
-	TRAPPC6B	14	39137811	39205430	123	12	10164	0.65269	0.25698
+	CAND2	3	12761653	12844818	287	28	10164	0.65267	0.25699
+	OR56A3	11	5912347	5958294	185	17	10164	0.65198	0.25721
-	TSKS	19	49729753	49798328	63	9	10164	0.6516	0.25733
+	RGS20	8	53816808	53969304	219	22	10164	0.6514	0.25739
-	LGALS7	19	38760968	38808517	117	12	10164	0.65132	0.25742
+	FBXO24	7	100551333	100611117	38	4	10164	0.65113	0.25748
-	EPOR	19	11367205	11419342	21	1	10164	0.65108	0.2575
+	OR52B6	11	5545877	5591884	291	22	10164	0.65088	0.25756
+	EDRF1	10	125684515	125774143	143	18	10164	0.65085	0.25757
+	ZNF211	19	57598167	57652779	155	7	10164	0.65071	0.25762
-	S100A8	1	153380032	153457578	142	6	10164	0.65059	0.25766
-	ZNF112	19	44316553	44391704	139	13	10164	0.65019	0.25779
-	HLA-DOA	6	32994182	33044612	515	50	10164	0.65009	0.25782
-	SNRPD2	19	45677454	45727311	85	16	10164	0.64963	0.25797
+	OR10G8	11	123994623	124040558	246	16	10164	0.64946	0.25802
-	ERMP1	9	5754061	5868081	312	28	10164	0.64944	0.25803
-	TMEM143	19	48322356	48399237	52	4	10164	0.6491	0.25814
-	FAM65B	6	24794281	25077288	1115	101	10164	0.64902	0.25816
-	OR2AT4	11	75078751	75124713	104	7	10164	0.64902	0.25816
+	OR2T4	1	248326581	248372627	43	1	10164	0.64887	0.25821
+	ITK	5	157145896	157265099	272	30	10164	0.64878	0.25824
-	VGLL3	3	86927973	87026287	393	34	10164	0.64862	0.25829
-	DNAH12	3	57283699	57579344	744	12	10164	0.64841	0.25836
+	TMEM265	16	30705191	30755196	9	1	10164	0.64834	0.25838
-	IDI1	10	1030023	1091699	209	27	10164	0.64823	0.25842
-	TBC1D8	2	100997228	101230326	547	45	10164	0.64813	0.25845
-	OR52A5	11	5121692	5167642	140	16	10164	0.648	0.25849
-	ANGPTL1	1	178839535	178906353	113	13	10164	0.64791	0.25852
+	APCDD1	18	10419628	10498701	269	41	10164	0.64708	0.25879
+	FAM122A	9	68745048	68793693	125	21	10164	0.64688	0.25886
+	MYO3A	10	25899073	26222536	22	2	10164	0.64679	0.25888
-	ALKBH6	19	35999116	36049243	64	11	10164	0.64678	0.25889
+	GID8	20	62903089	62958475	113	12	10164	0.64646	0.25899
-	OR2D2	11	6881574	6927500	73	12	10164	0.64622	0.25907
+	IL33	9	6180149	6267983	137	14	10164	0.64605	0.25912
-	ICAM3	19	10323776	10374829	136	16	10164	0.64578	0.25921
+	KCNT1	9	135667185	135803147	78	5	10164	0.64561	0.25927
-	PRR23A	3	138993962	139041268	68	8	10164	0.64548	0.25931
+	LOC441155	6	65266418	65315613	54	4	10164	0.64512	0.25942
-	KRT78	12	52827804	52883994	350	22	10164	0.645	0.25946
+	GEMIN7	19	45040739	45101524	97	7	10164	0.64445	0.25964
+	SPTBN1	2	54421317	54681446	941	50	10164	0.6443	0.25969
+	ZNF551	19	57646969	57699811	113	10	10164	0.6439	0.25982
-	ZNF843	16	31425564	31478027	15	3	10164	0.64351	0.25995
+	PROKR1	2	68610822	68665576	91	14	10164	0.64345	0.25997
+	LOC100506922	2	127343128	127399411	68	9	10164	0.6434	0.25998
+	CD28	2	203671475	203748912	140	18	10164	0.64311	0.26008
-	NRG4	15	75931973	76047444	73	12	10164	0.64295	0.26013
+	HIAT1	1	100003078	100093377	115	5	10164	0.64271	0.26021
-	ENOX1	13	43203130	43821980	1569	90	10164	0.6424	0.26031
+	SLC4A4	4	71152286	71582087	791	54	10164	0.64237	0.26032
-	SAP25	7	100562226	100608864	30	3	10164	0.64231	0.26034
-	AVIL	12	57787372	57854824	112	14	10164	0.64224	0.26036
+	P2RY6	11	73229496	73308625	101	7	10164	0.64213	0.2604
-	DLG4	17	7179890	7255050	95	16	10164	0.6421	0.26041
-	DENND5A	11	9128825	9300335	83	4	10164	0.64204	0.26042
-	IFNE	9	21470839	21517313	121	9	10164	0.64202	0.26043
-	TRIM59	3	160425503	160484838	119	14	10164	0.64199	0.26044
+	NBEAL1	2	202979617	203236353	263	15	10164	0.6418	0.2605
+	TIGD5	8	143562904	143610315	190	20	10164	0.6418	0.2605
-	C9orf116	9	135485180	135536832	111	10	10164	0.64169	0.26054
-	PDGFB	22	39213680	39279952	109	13	10164	0.6415	0.2606
-	C1orf116	1	207008521	207067761	145	14	10164	0.64143	0.26062
-	RNF186	1	19804029	19850278	150	32	10164	0.64128	0.26067
-	NMT2	10	15095700	15203705	324	41	10164	0.64124	0.26068
-	PPP1R14B	11	64234479	64281941	135	26	10164	0.64126	0.26068
-	ZFYVE28	4	2257524	2453690	454	45	10164	0.64103	0.26075

STRAND	SYMBOL	CHR	START	STOP	NSNPS	NPARAM	N	ZSTAT	P
+	TMEM45B	11	129780781	129870003	282	25	10164	0.64091	0.26079
-	KSR2	12	117443012	118004103	1589	167	10164	0.64083	0.26082
-	LOC105375604	7	156927417	156974681	221	19	10164	0.64067	0.26087
+	CREB3L4	1	153932839	153984364	42	5	10164	0.6405	0.26092
-	RASGRP1	15	38478101	38599806	91	12	10164	0.64026	0.261
-	FMOD	1	203330621	203386429	118	20	10164	0.64015	0.26104
+	SNAP23	15	42456244	42543061	108	12	10164	0.64016	0.26104
+	SYNM	15	99070080	99145595	34	2	10164	0.64014	0.26104
-	ZNF787	19	56077363	56156373	63	8	10164	0.6399	0.26112
+	TICRR	15	89540438	89638023	70	2	10164	0.63961	0.26121
-	MOB3A	19	2061032	2131502	126	20	10164	0.63958	0.26122
-	RHOV	15	40862214	40909289	113	10	10164	0.63949	0.26125
+	INTS3	1	153693050	153784808	31	5	10164	0.63937	0.26129
-	C16orf89	16	5032772	5101145	51	6	10164	0.63929	0.26132
+	BLOC1S4	4	6681115	6727660	68	12	10164	0.63917	0.26135
+	C8orf33	8	145017438	145075864	162	6	10164	0.63919	0.26135
-	WDR46	6	33269103	33324527	211	40	10164	0.63912	0.26137
+	GPX5	6	28491012	28544951	218	26	10164	0.63906	0.26139
-	GOLGB1	3	121653199	121785811	285	22	10164	0.63901	0.26141
+	DPH2	1	43934981	43983371	69	8	10164	0.63897	0.26142
-	COL4A4	2	226957532	227199559	775	54	10164	0.63876	0.26149
-	CORO6	17	29604756	29657908	19	1	10164	0.63872	0.2615
-	CCDC92	12	123926409	124007985	94	2	10164	0.63861	0.26154
+	CDC42	1	22017627	22102943	227	17	10164	0.63857	0.26155
-	YWHAZ	8	100908576	100989596	155	13	10164	0.63843	0.2616
-	TRAPPC6A	19	45152928	45213243	131	22	10164	0.63841	0.2616
-	MZF1	19	58551917	58609439	19	3	10164	0.6384	0.26161
-	MTCH1	6	36958135	37021551	300	37	10164	0.63826	0.26165
-	FBXL18	7	5444465	5548805	143	9	10164	0.63804	0.26172
-	ZBTB45	19	58503530	58555823	26	4	10164	0.63797	0.26175
+	ATF3	1	212530334	212630777	260	37	10164	0.63757	0.26188
-	RBP2	3	139442884	139511510	169	11	10164	0.63713	0.26202
+	PRAP1	10	133312340	133362683	5	1	10164	0.63713	0.26202
+	ZNF804B	7	88724368	89347057	2285	102	10164	0.63676	0.26214
+	TRIP6	7	100831221	100883455	90	13	10164	0.63671	0.26216
+	LRRC74B	22	21010960	21074168	118	21	10164	0.63662	0.26219
+	PCF11	11	83122095	83195794	110	14	10164	0.63632	0.26228
-	SLC12A7	5	1040374	1190747	397	50	10164	0.63627	0.2623
-	PRG3	11	57366769	57416150	58	8	10164	0.63612	0.26235
-	ZNF681	19	23729195	23793891	6	1	10164	0.63605	0.26237
-	HIST1H2BJ	6	27122316	27167796	67	14	10164	0.63589	0.26242
-	LAMTOR3	4	99868338	99929546	72	9	10164	0.63584	0.26244
+	TBCD	17	82717060	82953186	564	22	10164	0.63578	0.26246
-	PLA2R1	2	159922002	160097902	582	41	10164	0.63579	0.26246
-	RAB3B	1	51897956	52025764	208	14	10164	0.6357	0.26249
-	TBATA	10	70761238	70820401	257	28	10164	0.63501	0.26271
+	SPIC	12	101440323	101497566	120	13	10164	0.63471	0.26281
-	LSM5	7	32475333	32530258	176	14	10164	0.63431	0.26294
+	PKN1	19	14398053	14481867	168	27	10164	0.63393	0.26306
+	CSNK1G2	19	1906149	1991338	296	29	10164	0.63371	0.26314
-	PIGU	20	34550542	34712285	184	9	10164	0.63352	0.2632
-	RCBTB1	13	49521944	49620606	499	20	10164	0.63347	0.26321
+	ANXA1	9	73116731	73180393	194	23	10164	0.63339	0.26324
+	METTL17	14	20954806	21007035	166	19	10164	0.63333	0.26326
-	WDR47	1	108960211	109077228	28	2	10164	0.63324	0.26329
+	MTBP	8	120410398	120533635	201	6	10164	0.63283	0.26342
+	LOC105370380	13	113782492	113830631	175	14	10164	0.6327	0.26347
+	BLVRA	7	43723192	43817342	136	14	10164	0.63253	0.26352
+	ZNF592	15	84713561	84816432	201	20	10164	0.63248	0.26354
-	EIF5A2	3	170878415	170943637	67	6	10164	0.63223	0.26362
-	UBQLNL	11	5504393	5551726	241	22	10164	0.63218	0.26364
+	NOM1	7	156914723	156983182	308	17	10164	0.632	0.26369
+	COX6B1	19	35613223	35668784	119	18	10164	0.63199	0.2637
+	ETV2	19	35606213	35654871	89	14	10164	0.63128	0.26393
+	C2orf78	2	73749189	73827148	4	1	10164	0.63113	0.26398
-	COLGALT2	1	183919662	184086611	195	26	10164	0.63109	0.26399
-	CDC27	17	47107697	47224312	172	15	10164	0.63086	0.26406
-	AKT1S1	19	49859033	49913356	120	23	10164	0.63075	0.2641
+	ADGRG5	16	57494333	57587210	179	20	10164	0.63052	0.26418
+	LOC105370553	14	70188259	70262008	187	13	10164	0.62996	0.26436
-	DUSP16	12	12463282	12597839	261	29	10164	0.62984	0.2644
-	CAPZB	1	19328773	19520641	533	30	10164	0.62957	0.26449
-	XPO6	16	28087976	28246960	322	6	10164	0.62948	0.26452
-	RBP1	3	139507434	139574829	135	15	10164	0.62939	0.26455

STRAND	SYMBOL	CHR	START	STOP	NSNPS	NPARAM	N	ZSTAT	P
-	MMP7	11	102510508	102565747	199	35	10164	0.62932	0.26457
+	GPBP1	5	57138849	57274679	223	25	10164	0.62921	0.2646
-	AZIN1	8	102816302	102899200	241	19	10164	0.62908	0.26465
-	PTPRD	9	8304246	10647723	9138	445	10164	0.62891	0.2647
-	ST6GALNAC2	17	76555379	76621066	55	3	10164	0.62881	0.26474
-	RGL3	19	11384056	11454342	21	2	10164	0.62823	0.26493
+	LOC105374301	3	195960072	196015093	24	3	10164	0.62822	0.26493
+	FOXN2	2	48279644	48389295	32	4	10164	0.62787	0.26504
+	TRIP4	15	64352804	64465303	3	1	10164	0.62774	0.26509
+	OR1L4	9	122688990	122734925	24	6	10164	0.62702	0.26532
-	COPB2	3	139347587	139424680	187	12	10164	0.62682	0.26539
-	C3orf70	3	185068050	185188014	391	34	10164	0.62679	0.2654
+	RNF165	18	46299224	46471412	265	42	10164	0.62673	0.26542
+	YTHDC2	5	113478683	113605287	485	18	10164	0.62662	0.26545
+	PTP4A1	6	63537012	63593588	60	4	10164	0.62612	0.26562
-	HLA-DOB	6	32802763	32852048	558	55	10164	0.62605	0.26564
-	HOXA7	7	27143716	27191677	89	18	10164	0.62592	0.26568
+	DNTTIP1	20	45756937	45821427	201	18	10164	0.62578	0.26573
-	DBT	1	100176922	100284863	42	9	10164	0.62555	0.26581
-	ZNF70	22	23731584	23786092	177	23	10164	0.62548	0.26583
+	OR10G9	11	123988013	124033948	233	17	10164	0.6248	0.26605
-	KPNA1	3	122411901	122549939	261	18	10164	0.62478	0.26606
-	CHIC2	4	53998373	54100427	95	7	10164	0.62472	0.26608
+	NUDT2	9	34294506	34353713	77	5	10164	0.62456	0.26613
-	AMFR	16	56351452	56460538	300	19	10164	0.62453	0.26614
+	LOC105376667	11	539016	587669	156	19	10164	0.62438	0.26619
+	LCOR	10	96797260	96974441	47	4	10164	0.62436	0.2662
+	LOC113230	19	14038009	14085062	23	2	10164	0.62433	0.26621
-	IFNA7	9	21191469	21237205	250	14	10164	0.62411	0.26628
+	ZFPM2	8	105211436	105814539	1268	91	10164	0.62384	0.26637
-	PBX2	6	32174733	32225186	327	55	10164	0.62375	0.26639
+	PRSS23	11	86756059	86962910	532	45	10164	0.62361	0.26644
-	EVA1B	1	36312030	36359154	59	12	10164	0.62353	0.26647
+	TRH	3	129939393	129987938	200	15	10164	0.62338	0.26652
+	DMRT1	9	806647	979090	565	43	10164	0.62332	0.26654
-	TACR2	10	69394202	69451918	141	9	10164	0.62323	0.26657
-	MROH6	8	143556189	143607758	203	22	10164	0.62297	0.26665
-	REEP1	2	86203993	86373418	466	18	10164	0.62214	0.26693
-	OR1D5	17	3052669	3098607	20	2	10164	0.62164	0.26709
+	DYNC1I1	7	95737506	96120322	989	81	10164	0.62129	0.2672
-	YDJC	22	21618089	21665325	43	6	10164	0.62114	0.26725
-	CRTC2	1	153937672	153993656	40	5	10164	0.62113	0.26726
+	SLC9A5	16	67212551	67282191	31	3	10164	0.62101	0.2673
+	FAM124A	13	51187334	51294241	278	45	10164	0.62083	0.26736
-	CCDC102A	16	57502178	57572130	125	14	10164	0.62035	0.26751
+	CRTAM	11	122803431	122882643	266	38	10164	0.62017	0.26757
-	CCNH	5	87381493	87448033	74	6	10164	0.62008	0.2676
+	RANBP2	2	108684446	108795811	152	6	10164	0.61999	0.26763
-	SLC7A7	14	22763222	22854811	337	49	10164	0.61965	0.26774
+	CASP7	10	113644162	113740909	361	28	10164	0.61948	0.2678
+	KIAA0922	4	153431346	153647608	355	32	10164	0.61922	0.26789
-	RCAN2	6	46210730	46527067	498	26	10164	0.61843	0.26814
-	ZNF606	19	57967072	58038346	166	9	10164	0.61846	0.26814
-	CDKL4	2	39164937	39281810	175	16	10164	0.61841	0.26815
-	RIC3	11	8082965	8204083	351	17	10164	0.61832	0.26818
+	ANKRD33	12	51853009	51901721	80	10	10164	0.61805	0.26827
+	PRKCH	14	61286443	61560980	726	78	10164	0.61796	0.2683
+	TPBGL	11	75205905	75253704	60	8	10164	0.61756	0.26843
-	ZASP	7	100564035	100609733	28	3	10164	0.61749	0.26846
-	TXNDC16	14	52420590	52587583	532	34	10164	0.61745	0.26847
+	NOD2	16	50658581	50743081	155	18	10164	0.61678	0.26869
+	ZNF664	12	123938215	124025439	99	3	10164	0.6167	0.26872
+	BAX	19	48919860	48971798	123	17	10164	0.61666	0.26873
-	C6	5	41132146	41296486	567	42	10164	0.61639	0.26882
+	FCF1	14	74678117	74746691	111	12	10164	0.61615	0.2689
-	CRNN	1	152399243	152449274	71	7	10164	0.61612	0.26891
-	NAP1L4	11	2934430	3027377	252	14	10164	0.6158	0.26901
+	MLLT6	17	38670620	38739803	47	11	10164	0.61575	0.26903
+	ANKRD24	19	4147625	4234814	19	1	10164	0.61571	0.26904
+	ZNRF3	22	28765654	29067488	884	45	10164	0.61563	0.26907
-	SUCLG2	3	67350460	67689614	1174	49	10164	0.61548	0.26912
+	AATF	17	36913875	37066871	174	12	10164	0.61515	0.26923
-	RNASEH1	2	3521813	3593371	57	5	10164	0.6148	0.26934
+	GTF2H3	12	123598739	123672604	63	5	10164	0.61446	0.26945

STRAND	SYMBOL	CHR	START	STOP	NSNPS	NPARAM	N	ZSTAT	P
-	HSD17B14	19	48803017	48871703	66	12	10164	0.61426	0.26952
-	AKR7A2	1	19292708	19347146	122	13	10164	0.61424	0.26953
+	PTPN13	4	86559307	86825179	780	24	10164	0.61397	0.26962
-	ZNF14	19	19700472	19768112	143	16	10164	0.61379	0.26968
-	WDR33	2	127694234	127846187	351	19	10164	0.61367	0.26972
-	STRN4	19	46709509	46781475	133	16	10164	0.61344	0.26979
-	CA12	15	63313531	63417110	288	48	10164	0.61304	0.26992
+	CAPZA3	12	18703111	18749188	84	7	10164	0.61264	0.27006
+	TARS	5	33405696	33478091	164	14	10164	0.61227	0.27018
+	ENDOG	9	128783411	128832678	69	6	10164	0.61142	0.27046
+	RPS7	2	3540263	3590919	47	5	10164	0.61135	0.27048
+	DMBX1	1	46454837	46524226	2	1	10164	0.61125	0.27052
+	CEP350	1	179919773	180124880	381	14	10164	0.61066	0.27071
+	LOC105377310	4	82434579	82490384	24	2	10164	0.61068	0.27071
+	MACF1	1	39046417	39497138	445	20	10164	0.61035	0.27082
+	SERPINB7	18	63718047	63815376	95	5	10164	0.61012	0.27089
+	KCNH8	3	19112093	19545643	359	21	10164	0.61013	0.27089
+	MFSD3	8	144472267	144521228	44	4	10164	0.61	0.27093
+	OR2L8	1	247913858	247959796	51	4	10164	0.60987	0.27097
+	CR1	1	207461128	207651765	290	26	10164	0.60977	0.27101
+	ZSCAN1	19	57998989	58068907	128	12	10164	0.60974	0.27102
-	ZFP82	19	36380332	36453658	147	19	10164	0.60971	0.27103
-	PITPNM1	11	67481767	67540372	78	13	10164	0.60959	0.27107
+	C10orf71	10	49262056	49337492	171	17	10164	0.60929	0.27117
-	C1orf110	1	162836566	162903900	290	33	10164	0.60925	0.27118
-	TACSTD2	1	58565423	58612494	209	21	10164	0.60809	0.27156
-	GRID1	10	85589555	86401493	2140	152	10164	0.60771	0.27169
-	T	6	166147656	166203669	290	32	10164	0.6077	0.27169
+	ABCA7	19	1005103	1075572	329	27	10164	0.60766	0.27171
-	TLR10	4	38762238	38817990	340	23	10164	0.60763	0.27172
-	ATP5H	17	75028860	75081979	90	8	10164	0.60739	0.27179
+	ADNP2	18	80074031	80150348	55	5	10164	0.60736	0.27181
+	LRRC8A	9	128847112	128928042	123	12	10164	0.60728	0.27183
-	LOC105372585	20	24458226	24510800	32	7	10164	0.60719	0.27186
+	SERPINB10	18	63872969	63946111	274	9	10164	0.60717	0.27187
+	SLC7A6	16	68228830	68311823	94	7	10164	0.60704	0.27191
+	LOC105374013	3	101652198	101719759	96	9	10164	0.60638	0.27213
+	MAP2K3	17	21249656	21325240	75	10	10164	0.60599	0.27226
+	SWI5	9	128240384	128298989	90	10	10164	0.60495	0.27261
-	WDR72	15	53503741	53797878	1340	55	10164	0.60458	0.27273
-	THEMIS	6	127698194	127953631	201	12	10164	0.60449	0.27276
-	CACUL1	10	118662993	118790246	118	7	10164	0.6044	0.27279
-	LOC101927763	11	5504393	5556635	278	22	10164	0.60402	0.27292
+	SART1	11	65926689	65990136	161	19	10164	0.60379	0.27299
-	CHIT1	1	203202823	203265099	238	26	10164	0.60377	0.273
-	LOC105379177	5	132948513	132997693	101	9	10164	0.60371	0.27302
-	BSX	11	122967649	123016671	129	16	10164	0.60334	0.27314
-	ZEB2	2	144374375	144555393	99	17	10164	0.60328	0.27316
+	TECTB	10	112248345	112315035	78	8	10164	0.60324	0.27318
+	HKDC1	10	69185303	69277559	243	36	10164	0.60311	0.27322
+	C2orf27A	2	131687491	131777404	121	6	10164	0.60301	0.27325
+	ALPP	2	232343534	232392889	83	10	10164	0.60277	0.27333
-	MRPL13	8	120385844	120480407	119	6	10164	0.60268	0.27336
-	PSD3	8	18517303	19121921	2882	185	10164	0.60249	0.27342
+	C1orf158	1	12711186	12771169	150	8	10164	0.6025	0.27342
-	EEF1D	8	143569697	143632675	189	19	10164	0.60182	0.27365
-	STC1	8	23831921	23889807	195	23	10164	0.60165	0.2737
-	UBALD1	16	4598883	4649926	18	1	10164	0.60133	0.27381
+	ATP1B3	3	141841628	141936540	175	10	10164	0.60116	0.27387
-	MYO1C	17	1454186	1527707	109	12	10164	0.60101	0.27392
-	SGMS1	10	50295585	50658977	874	35	10164	0.60099	0.27392
-	TFPI	2	187454230	187589494	354	27	10164	0.60087	0.27396
-	ROS1	6	117277918	117460922	658	56	10164	0.60057	0.27406
-	MAPK11	22	50253713	50305393	40	6	10164	0.60031	0.27415
-	LMF1	16	843634	1016318	442	13	10164	0.60016	0.2742
-	ANKRD39	2	96837985	96893019	75	12	10164	0.60012	0.27421
-	POP5	12	120569045	120616452	121	21	10164	0.60009	0.27422
+	RNF213	17	80225840	80408786	97	9	10164	0.6	0.27425
-	N4BP2L2	13	32422488	32573885	261	32	10164	0.59995	0.27427
-	YME1L1	10	27100111	27190278	192	11	10164	0.59992	0.27428
-	C15orf52	15	40321452	40375967	143	28	10164	0.59951	0.27442
+	MASTL	10	27119824	27196920	167	10	10164	0.59925	0.2745
+	SRFBP1	5	121926961	122038600	221	8	10164	0.59879	0.27466
-	MRPL2	6	43044029	43095186	105	11	10164	0.59874	0.27467

STRAND	SYMBOL	CHR	START	STOP	NSNPS	NPARAM	N	ZSTAT	P
-	NOB1	16	69731854	69789968	90	8	10164	0.59842	0.27478
-	OTUD4	4	145123650	145214973	80	8	10164	0.59833	0.27481
+	C19orf24	19	1240521	1289244	7	3	10164	0.5982	0.27485
+	SCYL1	11	65490065	65548711	80	25	10164	0.59808	0.27489
-	NAPRT	8	143564664	143614626	204	22	10164	0.59791	0.27495
+	SH3BGR	21	39410856	39525506	260	15	10164	0.59784	0.27497
-	FRK	6	115931528	116135728	473	29	10164	0.59779	0.27499
+	CLDN16	3	190352872	190422143	223	25	10164	0.59778	0.27499
+	RGS4	1	163033606	163086802	111	12	10164	0.5976	0.27505
+	ZNF8	19	58243952	58305888	143	11	10164	0.59759	0.27506
+	SUV39H2	10	14843783	14914315	125	24	10164	0.59759	0.27506
-	WFDC5	20	45099452	45151321	120	10	10164	0.59745	0.2751
+	TTLL5	14	75626208	75965082	514	34	10164	0.59739	0.27512
-	NAPA	19	47477634	47550258	2	1	10164	0.59727	0.27516
-	KPTN	19	47465141	47521791	2	1	10164	0.59727	0.27516
-	C19orf26	19	1219948	1276763	53	6	10164	0.59723	0.27518
+	ANG	14	20649177	20704186	92	16	10164	0.59687	0.2753
-	HBB	11	5215466	5262071	104	8	10164	0.59652	0.27542
-	LOC105372478	19	58009302	58060640	86	9	10164	0.59634	0.27547
+	IL4	5	132638986	132692678	123	22	10164	0.59626	0.2755
-	FAM53A	4	1564062	1719313	286	27	10164	0.59606	0.27557
-	CNTFR	9	34541432	34625140	199	24	10164	0.59582	0.27565
+	DISP2	15	40319359	40381055	153	29	10164	0.59565	0.2757
+	PNRC2	1	23924811	23973462	15	2	10164	0.59535	0.2758
+	G0S2	1	209640325	209686390	31	6	10164	0.59527	0.27583
-	DCHS1	11	6611324	6690849	191	27	10164	0.59504	0.27591
+	TNFRSF11A	18	62290287	62397710	467	57	10164	0.59493	0.27594
+	C5orf64	5	61602781	61740997	395	39	10164	0.59492	0.27595
-	ABHD16A	6	31676949	31738360	272	63	10164	0.59472	0.27602
+	CYSTM1	5	140140068	140253789	147	6	10164	0.59469	0.27602
+	DAPK1	9	87462228	87718634	875	73	10164	0.5946	0.27606
-	MAP6D1	3	183805876	183860605	131	13	10164	0.59426	0.27617
+	IL17RC	3	9882074	9943621	104	10	10164	0.59415	0.27621
-	DENND6A	3	57615454	57728089	176	11	10164	0.59409	0.27622
-	TIMELESS	12	56406373	56484416	99	7	10164	0.59389	0.27629
-	C2orf42	2	70139885	70226019	224	23	10164	0.59389	0.27629
+	PLD3	19	40313425	40388490	157	20	10164	0.59364	0.27638
+	HRH2	5	175622796	175720756	69	7	10164	0.59335	0.27647
-	PARP4	13	24410930	24547993	618	16	10164	0.59322	0.27652
-	VWA7	6	31755401	31812331	233	71	10164	0.5929	0.27662
-	XRRA1	11	74830910	74984187	118	6	10164	0.59288	0.27663
-	SPG20	13	36291638	36405180	79	4	10164	0.59284	0.27664
+	NABP1	2	191643072	191698522	109	15	10164	0.59284	0.27665
-	DCHS2	4	154224375	154526778	280	27	10164	0.5928	0.27666
+	RLN3	19	13993205	14040971	59	5	10164	0.59216	0.27687
+	GRASP	12	51971937	52025889	47	8	10164	0.59212	0.27688
+	PRIM2	6	57279805	57656850	884	23	10164	0.59199	0.27693
+	GCDH	19	12856129	12924630	41	3	10164	0.59191	0.27696
+	FUK	16	70419584	70490274	2	1	10164	0.5917	0.27702
+	GRIA2	4	157185584	157376075	212	20	10164	0.59162	0.27705
+	RMI1	9	83945720	84014074	115	12	10164	0.59007	0.27757
+	KLF17	1	44008927	44145140	437	39	10164	0.58995	0.27761
-	FCRL4	1	157563749	157633080	280	15	10164	0.58983	0.27765
+	ZC3H15	2	186451158	186519361	21	3	10164	0.58971	0.27769
-	TMEM243	7	87186160	87255573	174	16	10164	0.58965	0.27771
-	DDC	7	50448436	50600457	585	22	10164	0.58927	0.27784
-	ZNF296	19	45061500	45111430	98	7	10164	0.58923	0.27785
+	FOXF1	16	86475527	86524464	263	43	10164	0.58921	0.27786
-	RCC2	1	17396755	17474754	222	18	10164	0.58921	0.27786
-	CCBL1	9	128822942	128917513	140	13	10164	0.58917	0.27787
-	DPF3	14	72599032	72929116	1016	97	10164	0.58917	0.27787
-	ALG14	1	94971565	95107964	301	22	10164	0.58911	0.27789
+	SNAP29	22	20824004	20901214	6	2	10164	0.58837	0.27814
-	MTRF1L	6	152977366	153037815	193	27	10164	0.58825	0.27818
+	NPAP1	15	24640394	24693446	160	13	10164	0.58814	0.27822
+	TAF4B	18	26191445	26401685	465	28	10164	0.58802	0.27826
-	ETHE1	19	43496719	43562244	208	28	10164	0.58799	0.27827
-	FAM214B	9	35094121	35151351	177	30	10164	0.58781	0.27833
+	C9orf131	9	35006095	35055991	117	18	10164	0.58747	0.27845
-	TMPRSS11F	4	68043198	68164869	261	9	10164	0.5868	0.27867
+	TBPL1	6	133917170	133997500	141	9	10164	0.58671	0.2787
-	KLHL35	11	75412394	75468444	73	10	10164	0.58652	0.27876
+	GALR3	22	37787009	37835865	58	4	10164	0.58634	0.27882
+	CRAMP1L	16	1579640	1687908	180	10	10164	0.58617	0.27888

STRAND	SYMBOL	CHR	START	STOP	NSNPS	NPARAM	N	ZSTAT	P
+	ZSWIM3	20	45822581	45889131	199	20	10164	0.58616	0.27888
+	C9orf173	9	137215840	137263483	92	10	10164	0.58613	0.2789
+	SPCS2	11	74914247	74989031	73	5	10164	0.58584	0.27899
-	C9orf152	9	110189561	110243133	150	17	10164	0.58567	0.27905
+	PLCE1	10	93958989	94338392	457	54	10164	0.58564	0.27906
-	LOC100652833	5	99512014	99561049	123	7	10164	0.58559	0.27907
+	ZNF282	7	149160462	149236248	129	13	10164	0.58529	0.27918
-	KRTAP19-8	21	31028159	31073476	3	2	10164	0.58516	0.27922
-	TIMM8B	11	112074800	112121798	74	9	10164	0.58508	0.27925
-	C9orf84	9	111676175	111830008	569	26	10164	0.585	0.27928
+	CHST14	15	40435961	40483158	80	18	10164	0.5849	0.27931
+	ALG1	16	5036809	5097379	48	5	10164	0.58463	0.2794
-	C1orf86	1	2174460	2247720	151	29	10164	0.58382	0.27967
+	SOX1	13	112032599	112081706	42	8	10164	0.58331	0.27984
-	FHL2	2	105350815	105473773	325	49	10164	0.58326	0.27986
+	LOC105378254	12	80389259	80445269	23	2	10164	0.58274	0.28003
+	IL27RA	19	13996740	14062919	75	6	10164	0.5826	0.28008
+	SLC16A3	17	82193406	82249499	49	1	10164	0.58237	0.28016
+	RNF182	6	13889446	13990009	133	7	10164	0.58234	0.28017
+	TMEM236	10	17717210	17810887	248	24	10164	0.5821	0.28025
+	PCDHA9	5	140812772	141022344	256	10	10164	0.58188	0.28032
-	C7orf76	7	96471626	96538523	137	17	10164	0.58168	0.28039
+	GPR137B	1	236107502	236218909	160	14	10164	0.58134	0.28051
+	APOE	19	44870749	44919395	106	14	10164	0.5813	0.28052
+	FTL	19	48930309	48976879	129	18	10164	0.58108	0.28059
+	RASSF5	1	206472530	206599284	234	32	10164	0.58103	0.28061
-	EZR	6	158755741	158854424	350	16	10164	0.58094	0.28064
+	NUDT16L1	16	4658558	4705859	58	2	10164	0.58078	0.28069
-	CUL7	6	43027617	43088950	121	12	10164	0.58046	0.2808
-	FOXN3	14	89146172	89654150	748	85	10164	0.58039	0.28083
+	TNNI2	11	1804003	1851680	211	40	10164	0.58017	0.2809
-	C1orf123	1	53204099	53255639	103	19	10164	0.58006	0.28094
+	MGMT	10	129432190	129778042	721	25	10164	0.57983	0.28102
+	LOH12CR1	12	12322079	12479410	456	35	10164	0.57952	0.28112
+	HINT3	6	125921715	125990244	28	5	10164	0.5795	0.28112
-	TOLLIP	11	1264368	1344662	190	25	10164	0.57859	0.28143
+	VIP	6	152712923	152769765	170	21	10164	0.57854	0.28145
+	ASAH2B	10	50704899	50767188	115	7	10164	0.57853	0.28145
-	GSC	14	94758223	94805162	185	42	10164	0.5783	0.28153
-	CA10	17	51620313	52195017	1088	77	10164	0.57793	0.28166
+	KDM1B	6	18120388	18233854	69	5	10164	0.57679	0.28204
-	THPO	3	184361935	184414688	99	23	10164	0.57645	0.28216
+	SPINT2	19	38229458	38302614	345	48	10164	0.57637	0.28218
+	WDR17	4	176030834	176192828	362	12	10164	0.57633	0.2822
+	SMIM13	6	11059033	11148738	303	28	10164	0.57622	0.28223
+	OR2L3	1	248025682	248071620	71	5	10164	0.57604	0.2823
+	GDA	9	72079595	72269059	185	13	10164	0.57566	0.28242
-	DPEP2	16	67977390	68035611	53	5	10164	0.57556	0.28246
+	GLIPR2	9	36101536	36173913	64	8	10164	0.57538	0.28252
-	TSGA10	2	98987261	99189920	364	11	10164	0.57521	0.28257
+	SEMA4B	15	90149920	90239664	341	31	10164	0.57496	0.28266
+	SP4	7	21393071	21524822	556	46	10164	0.5743	0.28288
+	GREB1L	18	21207242	21535423	210	8	10164	0.57422	0.28291
-	FANCC	9	95089054	95352709	544	28	10164	0.57411	0.28295
-	NFE2	12	54282107	54336037	63	7	10164	0.57351	0.28315
-	CEP170	1	243114428	243290406	241	19	10164	0.57337	0.2832
+	ZNF446	19	58439543	58499533	90	7	10164	0.57331	0.28322
-	HSPA12A	10	116661192	116885814	396	44	10164	0.57322	0.28325
-	ANKLE2	12	132715667	132796888	154	19	10164	0.57285	0.28337
-	RSPH10B	7	5916136	6005684	97	15	10164	0.57276	0.2834
+	THTPA	14	23476760	23569581	111	16	10164	0.57275	0.28341
-	AKAP10	17	19894452	20012856	272	10	10164	0.57192	0.28369
+	FYTTD1	3	197714553	197794446	88	2	10164	0.5719	0.28369
-	GBP4	1	89171148	89233950	25	3	10164	0.57153	0.28382
+	RNF123	3	49654143	49731529	46	5	10164	0.57151	0.28383
-	SLIT3	5	168651733	169336129	2103	187	10164	0.57122	0.28392
-	MCM6	2	135829626	135911477	177	15	10164	0.57118	0.28394
-	PPP1R14A	19	38241237	38291591	237	42	10164	0.57099	0.284
+	TMEM169	2	216046866	216112783	121	20	10164	0.57095	0.28402
+	PAEP	9	135526754	135576776	86	11	10164	0.57055	0.28415
+	RAB12	18	8574445	8649383	159	15	10164	0.57056	0.28415
-	IHH	2	219044420	219095516	107	8	10164	0.57053	0.28416
-	SUSD5	3	33140045	33254215	321	27	10164	0.57032	0.28423
-	REG1B	2	79075023	79123024	190	29	10164	0.57027	0.28425

STRAND	SYMBOL	CHR	START	STOP	NSNPS	NPARAM	N	ZSTAT	P
+	C1orf50	1	42732245	42785742	58	8	10164	0.57014	0.28429
+	TRIM77	11	89675299	89727872	131	6	10164	0.5701	0.28431
+	KLRF2	12	9846489	9905833	53	7	10164	0.57004	0.28433
+	FGD2	6	36970647	37039070	329	43	10164	0.56995	0.28435
+	NR2E3	15	71772197	71828666	70	12	10164	0.56982	0.2844
-	NPC1L1	7	44502535	44576330	63	7	10164	0.56979	0.28441
+	PCDHGA12	5	141395591	141522979	220	19	10164	0.56975	0.28442
+	TSPAN15	10	69390218	69517669	348	12	10164	0.56964	0.28446
+	FAIM	3	138573594	138643376	1	1	10164	0.56961	0.28447
-	LINGO1	15	77603024	77855900	631	30	10164	0.56949	0.28451
+	CDC5L	6	44352514	44460424	238	33	10164	0.56925	0.28459
+	OR5C1	9	122753933	122799895	7	2	10164	0.56926	0.28459
-	ESD	13	46761256	46832681	92	8	10164	0.56908	0.28465
+	CPZ	4	8557660	8629761	100	11	10164	0.56874	0.28477
+	LOC105373991	2	236518699	236579257	51	2	10164	0.56869	0.28478
-	PLCXD3	5	41296946	41545628	691	42	10164	0.5686	0.28482
+	C17orf77	17	74549679	74604209	234	23	10164	0.56849	0.28485
+	LGALSL	2	64419193	64471383	4	2	10164	0.56847	0.28486
-	GLB1	3	32986608	33132202	450	31	10164	0.56843	0.28487
+	CCDC28A	6	138738520	138818327	223	22	10164	0.56843	0.28487
-	HRSP12	8	98092339	98152190	56	2	10164	0.56809	0.28499
+	NAA35	9	85906142	86035462	20	4	10164	0.56758	0.28516
+	FAM20B	1	178990809	179086567	290	28	10164	0.56738	0.28523
+	ACOXL	2	110697539	111130610	1227	71	10164	0.5672	0.28529
+	BCKDHB	6	80071627	80356270	867	28	10164	0.56718	0.2853
-	BRAF	7	140705951	140959764	560	16	10164	0.56712	0.28532
-	ELAVL1	19	7948573	8040645	169	10	10164	0.56706	0.28534
+	CMIP	16	81410170	81721762	1105	118	10164	0.56694	0.28538
+	PAPD7	5	6678953	6767048	313	22	10164	0.5669	0.28539
+	LOC105372811	21	26360033	26423109	154	30	10164	0.56679	0.28543
-	EXOC5	14	57192476	57303899	127	3	10164	0.56675	0.28544
-	ZNF425	7	149092784	149161346	124	5	10164	0.56667	0.28547
-	PEX1	7	92477023	92563531	165	18	10164	0.56632	0.28559
+	CCM2	7	44964746	45086470	12	5	10164	0.56628	0.2856
+	BARHL1	9	132547606	132600253	93	19	10164	0.56566	0.28581
+	GGNBP2	17	36509888	36599848	22	3	10164	0.5655	0.28587
-	FCHSD2	11	72826743	73177454	208	22	10164	0.56536	0.28592
-	CLIP3	19	36004660	36067895	90	13	10164	0.5652	0.28597
-	LTA4H	12	95990753	96078520	258	31	10164	0.56506	0.28601
-	ZCCHC14	16	87396246	87527269	249	26	10164	0.56507	0.28601
-	ST8SIA4	5	100796935	100938285	237	12	10164	0.56497	0.28605
-	ZNF688	16	30559677	30607495	6	2	10164	0.56494	0.28606
+	SPINT4	20	45687349	45735696	185	14	10164	0.56482	0.2861
+	GSX1	13	27757643	27803952	70	11	10164	0.56467	0.28615
+	POLR3D	8	22210104	22264355	45	10	10164	0.56429	0.28628
-	RBMS1	2	160262151	160528856	540	31	10164	0.56429	0.28628
-	PHYHIP	8	22209703	22267338	45	10	10164	0.56429	0.28628
+	PPP1CB	2	28716748	28812940	146	12	10164	0.56414	0.28633
-	HSD17B11	4	87326522	87426303	328	27	10164	0.56365	0.2865
-	ARPC5	1	183616193	183670941	324	6	10164	0.56355	0.28653
-	ANKRD28	3	15657236	15894555	535	22	10164	0.56354	0.28654
-	CAMSAP1	9	135798487	135942251	42	5	10164	0.56319	0.28665
-	CALY	10	133314072	133371971	10	1	10164	0.56276	0.2868
+	POP1	8	98082293	98169841	62	2	10164	0.56221	0.28699
+	TMOD3	15	51794628	51922138	401	16	10164	0.56213	0.28701
+	L3MBTL2	22	41170232	41241272	135	9	10164	0.56207	0.28703
+	PTF1A	10	23157531	23204252	24	5	10164	0.5621	0.28703
+	PFDN6	6	33254597	33300934	193	32	10164	0.56203	0.28705
-	TMEM205	19	11332776	11381414	6	2	10164	0.562	0.28706
-	ACOT8	20	45831721	45892409	181	19	10164	0.56196	0.28707
+	CLPSL2	6	35741594	35789552	272	38	10164	0.56188	0.2871
+	HTRA3	4	8234754	8317111	231	25	10164	0.56177	0.28713
-	PINX1	8	10754961	10874899	564	27	10164	0.56164	0.28718
+	AAMDC	11	77786107	77882354	157	8	10164	0.56158	0.2872
+	KRTAP13-4	21	30395276	30440758	129	12	10164	0.56155	0.28721
-	RGS8	1	182633341	182788307	225	22	10164	0.56146	0.28724
-	CBWD1	9	111038	214075	191	11	10164	0.56141	0.28726
+	DUSP22	6	257057	361355	12	9	10164	0.56131	0.28729
-	TRMT1L	1	185108085	185192098	109	8	10164	0.56104	0.28738
+	LOC101927262	2	251352	304727	82	6	10164	0.56084	0.28745
+	POLR2F	22	37918663	38051915	108	9	10164	0.56066	0.28751
-	DNAJB14	4	99886248	99981726	114	13	10164	0.56051	0.28757
+	NAV3	12	77536862	78223012	1098	77	10164	0.56047	0.28758
-	UBL3	13	29754405	29885683	309	17	10164	0.56003	0.28773

STRAND	SYMBOL	CHR	START	STOP	NSNPS	NPARAM	N	ZSTAT	P
-	KCNT2	1	196215779	196643560	135	13	10164	0.55968	0.28785
+	RELB	19	44966449	45048198	164	24	10164	0.55939	0.28795
-	KIAA1683	19	18247096	18309509	145	15	10164	0.55933	0.28797
-	ADCY10	1	167799080	167950526	18	5	10164	0.5589	0.28812
-	INS	11	2149779	2196209	195	24	10164	0.55867	0.28819
+	RRM1	11	4059561	4148881	248	13	10164	0.55859	0.28822
-	MDM1	12	68284566	68367381	167	25	10164	0.55854	0.28824
-	C16orf87	16	46792047	46866162	7	1	10164	0.55854	0.28824
-	DDN	12	48985149	49036091	26	2	10164	0.5585	0.28825
+	TIGIT	3	114258986	114320288	16	3	10164	0.55851	0.28825
-	LOC102723665	10	96820632	96867393	11	2	10164	0.55847	0.28826
-	OR52H1	11	5534561	5580523	284	21	10164	0.55825	0.28834
-	SPINK1	5	147814580	147874231	220	29	10164	0.55813	0.28838
+	PRRC2A	6	31585673	31647777	405	77	10164	0.55805	0.2884
+	FAM21A	10	50032888	50143509	7	4	10164	0.55796	0.28843
-	APOBEC4	1	183636275	183688313	295	5	10164	0.55786	0.28847
+	RRP15	1	218250285	218347983	209	24	10164	0.5573	0.28866
+	LAMC1	1	182988460	183155592	621	9	10164	0.55707	0.28874
-	PIGO	9	35078688	35131601	147	28	10164	0.55673	0.28886
-	CALHM1	10	103443387	103493891	106	19	10164	0.55648	0.28894
-	GBP5	1	89248950	89307861	51	8	10164	0.55629	0.28901
+	SPAG4	20	35580853	35631045	83	8	10164	0.55622	0.28903
-	SLC39A1	1	153949099	154003184	38	6	10164	0.55604	0.28909
+	OSTN	3	191164142	191273338	454	32	10164	0.55604	0.28909
-	LOC105371641	1	182054295	182279444	194	16	10164	0.55595	0.28912
+	PRKACB	1	84042975	84248498	325	18	10164	0.55485	0.2895
-	HDHD3	9	113363416	113412009	141	33	10164	0.55477	0.28952
+	PCSK9	1	55004476	55074853	193	30	10164	0.5546	0.28959
+	ACR	22	50703224	50755299	19	3	10164	0.55442	0.28964
-	MED24	17	40009097	40089636	153	15	10164	0.5544	0.28965
+	PLA2G15	16	68210343	68271062	67	3	10164	0.55409	0.28976
-	ZIC4	3	147376048	147442476	73	9	10164	0.55401	0.28979
+	NRGN	11	124704933	124757206	117	22	10164	0.55357	0.28994
-	TRAIP	3	49818595	49891564	93	8	10164	0.55344	0.28998
+	DDX1	2	15556621	15641111	279	28	10164	0.55338	0.29
+	ZNHIT3	17	36451627	36509310	20	3	10164	0.55329	0.29003
-	LOC105377622	4	139590624	139643579	160	32	10164	0.5533	0.29003
-	CKAP4	12	106227881	106282935	35	3	10164	0.55312	0.29009
-	RNF103	2	86593393	86658877	42	4	10164	0.55309	0.2901
-	HIST1H2BK	6	27128293	27181858	76	14	10164	0.5529	0.29017
-	DTNB	2	25367198	25708647	394	41	10164	0.55281	0.2902
+	KLK3	19	50819915	50887702	344	46	10164	0.55268	0.29024
-	PER2	2	238234038	238325102	217	25	10164	0.55255	0.29029
-	TNFRSF10D	8	23125588	23199030	264	59	10164	0.5524	0.29034
+	BANK1	4	101755607	102084812	796	31	10164	0.55232	0.29036
-	HOXB3	17	48538870	48625282	264	46	10164	0.5517	0.29058
-	NUDT5	10	12155330	12231144	162	21	10164	0.55113	0.29077
+	MRPS12	19	38895708	38943020	106	15	10164	0.55098	0.29082
-	HNRNPDL	4	82412564	82465225	62	3	10164	0.55098	0.29082
-	C19orf47	19	40285271	40383527	200	21	10164	0.55096	0.29083
-	ABCF2	7	151197830	151262374	89	8	10164	0.55069	0.29092
+	LOC105377746	5	175996325	176045274	5	3	10164	0.55067	0.29093
+	PIGX	3	196677374	196746007	103	6	10164	0.55028	0.29106
+	PTPN4	2	119724631	119994899	237	11	10164	0.55012	0.29112
-	SNAPC4	9	136365571	136435208	264	18	10164	0.55011	0.29112
+	LOC81691	16	20771429	20859668	1	1	10164	0.55002	0.29115
-	IFNA2	9	21374255	21420397	110	20	10164	0.54997	0.29117
+	NCOA3	20	47466857	47666877	429	18	10164	0.54995	0.29118
-	NAT9	17	74760547	74811395	117	12	10164	0.54969	0.29127
-	MCEE	2	71099676	71165288	221	17	10164	0.54943	0.29135
-	RPRML	17	46968156	47014248	126	6	10164	0.54944	0.29135
-	NAT8	2	73630723	73677410	1	1	10164	0.54932	0.29139
+	KLC4	6	43024594	43085099	116	11	10164	0.54934	0.29139
+	RBPJ	4	26128494	26445131	397	43	10164	0.54926	0.29141
+	MGRN1	16	4581493	4700974	73	4	10164	0.54921	0.29143
+	OR52J3	11	5011526	5057461	343	13	10164	0.54913	0.29146
-	TRMT61B	2	28839821	28905309	113	11	10164	0.54877	0.29158
-	GLTP	12	109840943	109915488	138	11	10164	0.54857	0.29165
-	RICTOR	5	38927920	39109419	479	31	10164	0.54858	0.29165
-	ADAMTS17	15	99961438	100377087	1971	125	10164	0.54855	0.29166
+	EXT2	11	44060549	44255430	536	45	10164	0.54847	0.29168
-	SKP1	5	134146391	134212033	130	8	10164	0.54849	0.29168
+	USPL1	13	30582693	30669557	94	5	10164	0.54843	0.2917
+	WDR6	3	48972204	49025953	3	1	10164	0.54821	0.29177

STRAND	SYMBOL	CHR	START	STOP	NSNPS	NPARAM	N	ZSTAT	P
-	DRAM2	1	111107332	111175216	98	8	10164	0.54816	0.29179
+	LOC105378190	14	104181110	104228144	15	2	10164	0.54817	0.29179
+	KDM3B	5	138317596	138447027	218	13	10164	0.5481	0.29181
-	CERS2	1	150955173	151010003	71	7	10164	0.54788	0.29189
-	C10orf90	10	126414997	126706038	311	38	10164	0.54787	0.29189
+	IFNA8	9	21374147	21420185	110	20	10164	0.54766	0.29196
+	TCL1B	14	95651417	95702643	104	21	10164	0.54755	0.292
+	GSDMA	17	39927973	39987880	188	16	10164	0.54737	0.29206
-	ESRP2	16	68218547	68271233	61	3	10164	0.5473	0.29209
-	C17orf59	17	8178333	8225246	111	22	10164	0.54717	0.29213
+	AP2M1	3	184139846	184194091	62	7	10164	0.54701	0.29218
-	ADAM8	10	133252416	133311903	25	2	10164	0.547	0.29219
-	DDX39A	19	14398798	14454383	52	14	10164	0.54679	0.29226
-	AXIN2	17	65518563	65596622	127	23	10164	0.54676	0.29227
+	ILF3	19	10619261	10702419	49	6	10164	0.54659	0.29233
+	VPS18	15	40859430	40913975	129	12	10164	0.5463	0.29243
+	TTC12	11	113279529	113395952	325	32	10164	0.54616	0.29248
+	ARID1B	6	156742892	157220779	258	30	10164	0.5461	0.2925
+	CXCR5	11	118848766	118906271	79	10	10164	0.54597	0.29254
-	UQCRHL	1	15797162	15842699	71	7	10164	0.54558	0.29268
+	CNGA3	2	98311155	98409154	133	13	10164	0.54485	0.29293
+	ACSS3	12	81043018	81267795	750	32	10164	0.54447	0.29306
-	C19orf84	19	51378289	51425574	6	2	10164	0.54415	0.29317
+	KRTAP12-3	21	44622932	44668341	129	7	10164	0.54373	0.29331
-	HGD	3	120618168	120717571	36	7	10164	0.5437	0.29332
+	WDR93	15	89655085	89753790	161	5	10164	0.54369	0.29333
+	PRLH	2	237531574	237577175	183	25	10164	0.54362	0.29335
-	GPX6	6	28493296	28550793	228	28	10164	0.54348	0.2934
-	BCL6	3	187711377	187780725	162	27	10164	0.54335	0.29345
+	LOC101930085	7	35659329	35709413	71	6	10164	0.54326	0.29347
+	TSHB	1	114994824	115044309	128	4	10164	0.54323	0.29349
+	SLC24A4	14	92287581	92511481	604	46	10164	0.54281	0.29363
+	DYNAP	18	54497713	54609493	209	23	10164	0.54262	0.2937
+	CYP4F12	19	15638018	15708819	473	24	10164	0.54253	0.29373
+	APOBEC3H	22	39062223	39114067	138	8	10164	0.54228	0.29381
+	DOPEY1	6	83032660	83178471	298	26	10164	0.54216	0.29386
+	PLCD4	2	218572765	218647186	95	4	10164	0.5419	0.29394
+	DEC1	9	115077617	115412644	1245	67	10164	0.54179	0.29398
-	DTD2	14	31436036	31492474	124	14	10164	0.54178	0.29398
-	KLK6	19	50948631	51004673	228	44	10164	0.54147	0.29409
-	ACTL7B	9	108844588	108890995	233	31	10164	0.54149	0.29409
-	PCED1A	20	2825314	2876243	158	17	10164	0.54134	0.29414
+	CPNE6	14	24035837	24088100	72	19	10164	0.54084	0.29431
+	CEBPB	20	50155583	50202690	150	13	10164	0.54066	0.29437
+	MRPS30	5	44773925	44825514	144	14	10164	0.5406	0.29439
-	CDO1	5	115794733	115851954	59	6	10164	0.54057	0.2944
-	FBXO5	6	152960519	153018605	220	24	10164	0.54049	0.29443
-	KIF24	9	34242380	34394914	156	9	10164	0.54031	0.29449
-	CELA1	12	51318443	51381679	137	17	10164	0.54024	0.29451
-	ERVW-1	7	92458380	92512915	109	14	10164	0.53983	0.29466
-	WDR49	3	167468684	167688501	285	26	10164	0.53943	0.2948
+	NAPG	18	10490876	10562769	230	14	10164	0.53888	0.29499
-	KHDRBS2	6	61620484	62321195	1540	32	10164	0.53852	0.29511
-	NES	1	156658763	156712397	107	20	10164	0.53844	0.29514
+	SERPINB11	18	63667304	63733893	25	3	10164	0.53775	0.29537
-	LTF	3	46426005	46500142	264	32	10164	0.53755	0.29544
+	KIAA1217	10	23659746	24557848	2060	98	10164	0.53743	0.29548
+	LOC105372489	19	40408184	40455263	64	6	10164	0.53739	0.2955
+	LOC101928093	5	172720503	172772565	121	22	10164	0.53731	0.29553
+	PKP4	2	158421880	158691429	831	33	10164	0.5369	0.29567
-	ZNF781	19	37657749	37727315	156	16	10164	0.53685	0.29568
-	VPS52	6	33240272	33306965	276	40	10164	0.53667	0.29575
+	ATXN2L	16	28788048	28847237	41	1	10164	0.53646	0.29582
+	KCTD17	22	37016725	37073390	38	3	10164	0.53646	0.29582
+	PRTN3	19	805965	858175	55	6	10164	0.53617	0.29592
+	ZBED4	22	49818849	49900078	307	15	10164	0.53615	0.29593
-	TMEM41B	11	9270654	9349768	20	2	10164	0.53616	0.29593
+	CREB5	7	28264321	28835894	2154	235	10164	0.53565	0.2961
+	PCDHGA11	5	141385970	141522979	242	21	10164	0.53554	0.29614
+	ORM1	9	114288023	114336479	168	32	10164	0.53551	0.29615
+	COL4A2	13	110272284	110523027	1485	180	10164	0.53534	0.29621
+	SLC28A1	15	84849577	84985651	190	31	10164	0.53529	0.29623
+	HAPLN2	1	156583801	156635725	108	12	10164	0.53515	0.29627
-	FSTL5	4	161373892	162199034	1478	83	10164	0.53493	0.29635

STRAND	SYMBOL	CHR	START	STOP	NSNPS	NPARAM	N	ZSTAT	P
+	PQLC2	1	19277246	19342431	126	15	10164	0.53475	0.29641
-	TTLL8	22	50008575	50094132	102	8	10164	0.53453	0.29649
+	RBX1	22	40916347	40983015	49	9	10164	0.53442	0.29653
+	CEPT1	1	111104627	111195102	132	11	10164	0.5339	0.29671
+	SYT8	11	1792479	1847521	236	40	10164	0.53378	0.29675
+	TRHR	8	109051342	109129584	239	21	10164	0.53374	0.29676
+	USP29	19	57085141	57141926	186	19	10164	0.53368	0.29678
+	KRTAP10-11	21	44611414	44657650	85	7	10164	0.53343	0.29687
+	C10orf107	10	61627952	61776766	404	23	10164	0.53332	0.29691
-	FBXL4	6	98863725	98983006	187	14	10164	0.53299	0.29702
+	OR5H6	3	98228260	98275350	278	14	10164	0.53255	0.29717
-	CHMP3	2	86493430	86598497	122	5	10164	0.53233	0.29725
-	TMEM262	11	65078212	65124990	73	13	10164	0.53232	0.29725
+	RALGPS2	1	178690147	178931842	507	27	10164	0.53228	0.29727
+	GADD45B	19	2441125	2488259	168	31	10164	0.53212	0.29732
-	FAM98A	2	33573661	33634362	149	14	10164	0.53176	0.29745
-	CCER2	19	38898980	38949370	105	14	10164	0.5311	0.29767
-	LAMB1	7	107913801	108038359	88	8	10164	0.53103	0.2977
+	RQCD1	2	218533575	218606435	86	4	10164	0.53102	0.2977
+	OGFOD1	16	56416417	56487495	181	21	10164	0.53101	0.29771
+	TINAG	6	54272792	54400152	486	35	10164	0.53044	0.2979
-	TMEM194A	12	57045643	57123857	90	9	10164	0.53042	0.29791
+	PRCC	1	156732482	156810817	160	14	10164	0.52982	0.29812
-	DPEP3	16	67965663	68015549	46	5	10164	0.52915	0.29835
-	COA5	2	98589323	98643518	30	3	10164	0.52896	0.29842
-	CHRM1	11	62898679	62956861	90	19	10164	0.52833	0.29864
-	NEK4	3	52700780	52805949	191	13	10164	0.52825	0.29866
+	TAPBPL	12	6416655	6472322	174	28	10164	0.52803	0.29874
-	EVX2	2	176066021	176120797	207	48	10164	0.52797	0.29876
-	FKBP15	9	113155519	113256361	266	27	10164	0.52773	0.29884
+	UNC5A	5	176775559	176890898	196	32	10164	0.52769	0.29886
-	GIMD1	4	106347335	106403788	105	8	10164	0.52765	0.29887
-	NUDT21	16	56419136	56486349	171	21	10164	0.5276	0.29889
+	PCDHB1	5	141016376	141063962	47	6	10164	0.52757	0.2989
+	VPS4A	16	69276384	69335043	127	15	10164	0.52722	0.29902
-	MCCC1	3	183005218	183134587	186	18	10164	0.52712	0.29906
-	KLF4	9	107474852	107524766	111	13	10164	0.52686	0.29915
+	SCYL2	12	100232137	100351722	179	11	10164	0.52679	0.29917
+	OAZ1	19	2234486	2283488	46	8	10164	0.52663	0.29922
-	CDCA5	11	65051009	65119150	108	15	10164	0.52626	0.29935
+	TMEM104	17	74741483	74849783	185	17	10164	0.52605	0.29943
-	IFNA17	9	21217243	21263222	194	11	10164	0.5259	0.29948
-	C6orf7	6	79793582	79913494	483	49	10164	0.52574	0.29953
+	PCDHGB7	5	141382647	141522981	250	21	10164	0.52572	0.29954
+	TMEM52B	12	10135599	10201804	194	17	10164	0.5257	0.29955
-	HAUS3	4	2221836	2277164	124	11	10164	0.52524	0.29971
+	CYLC2	9	102960311	103028488	271	22	10164	0.52498	0.2998
-	SNCA	4	89714099	89873314	390	14	10164	0.52497	0.2998
-	ASCC2	22	29778608	29873304	209	20	10164	0.52497	0.2998
-	PPP1R12C	19	55080913	55152600	103	15	10164	0.5246	0.29993
-	ZNF582	19	56364832	56428585	101	17	10164	0.5246	0.29993
+	KCNQ5	6	72586280	73208851	1562	122	10164	0.52455	0.29995
+	PTOV1	19	49813844	49870742	121	15	10164	0.52449	0.29997
-	MRPL24	1	156727302	156776266	80	11	10164	0.52429	0.30004
+	SNRPD1	18	21577269	21640247	27	2	10164	0.52418	0.30008
-	MTRNR2L7	10	37591438	37637931	58	5	10164	0.52407	0.30012
-	KLK7	19	50966479	51019064	241	37	10164	0.52404	0.30013
+	PDCD11	10	103361624	103456295	128	18	10164	0.52386	0.30019
+	VPS51	11	65061115	65121860	92	13	10164	0.52362	0.30027
-	PIH1D2	11	112042409	112109171	75	9	10164	0.52303	0.30048
+	SFT2D3	2	127666023	127713833	127	14	10164	0.5224	0.30069
-	KLK11	19	51012231	51063034	244	49	10164	0.52227	0.30074
-	HELZ2	20	63548086	63609239	223	38	10164	0.52193	0.30086
-	DNASE2	19	12865211	12916521	33	3	10164	0.5219	0.30087
-	WFDC9	20	45597939	45666268	231	14	10164	0.52184	0.30089
+	RNASE4	14	20649213	20710602	98	17	10164	0.52149	0.30101
+	H1FNT	12	48293980	48340279	66	6	10164	0.52147	0.30102
+	PCDHB14	5	141187326	141236288	129	12	10164	0.52138	0.30105
+	SLC22A12	11	64555810	64612353	99	13	10164	0.52138	0.30105
+	SH3BGRL3	1	26244722	26291522	107	8	10164	0.52126	0.30109
+	SSTR1	14	38172999	38223063	124	10	10164	0.52095	0.3012
+	PCDHA10	5	140820883	141022344	251	10	10164	0.5206	0.30132
-	MCIDAS	5	55209597	55262315	123	10	10164	0.52025	0.30144
-	PKM	15	72189029	72266812	63	6	10164	0.52019	0.30147

STRAND	SYMBOL	CHR	START	STOP	NSNPS	NPARAM	N	ZSTAT	P
+	RPL21	13	27216555	27266568	66	7	10164	0.51994	0.30155
-	WFDC10B	20	45674651	45740019	257	15	10164	0.51975	0.30162
-	ZNF45	19	43902624	43970280	210	19	10164	0.5196	0.30167
-	FAM92B	16	85088352	85147508	225	39	10164	0.5194	0.30174
-	LOC105374103	3	130133281	130180044	43	4	10164	0.51931	0.30177
+	AASDHPPT	11	106042565	106108692	95	12	10164	0.51911	0.30184
+	TRMT10B	9	37718204	37788972	169	19	10164	0.51901	0.30188
+	LAYN	11	111503985	111571745	103	17	10164	0.51854	0.30204
+	TPM1	15	63007639	63081915	261	30	10164	0.51846	0.30207
-	KLK9	19	50992513	51044634	252	40	10164	0.51839	0.30209
+	ASIC3	7	151013292	151062756	113	25	10164	0.51831	0.30212
-	TM4SF1	3	149359018	149412860	35	5	10164	0.51795	0.30224
+	SPATA13	13	23944626	24317074	1460	151	10164	0.51783	0.30229
+	B3GALT4	6	33242140	33288825	201	29	10164	0.51739	0.30244
+	CRIM1	2	36320771	36561135	580	54	10164	0.5173	0.30247
-	NME3	16	1760320	1806758	249	22	10164	0.51715	0.30253
+	LRFN4	11	66821480	66870475	30	9	10164	0.51707	0.30255
-	PCDH12	5	141933585	141994062	67	14	10164	0.51681	0.30264
-	CYB5R2	11	7655095	7709341	332	30	10164	0.51681	0.30265
+	SLC5A11	16	24810863	24921628	99	17	10164	0.51679	0.30265
+	OTOL1	3	161394601	161513975	376	20	10164	0.51648	0.30276
-	SHISA2	13	26034597	26094587	42	6	10164	0.51631	0.30282
+	NCR2	6	41300790	41360887	262	24	10164	0.51598	0.30293
-	KLK8	19	50986008	51036702	222	31	10164	0.51564	0.30305
+	WNK4	17	42744673	42807066	82	6	10164	0.51556	0.30308
+	SH2B1	16	28811600	28884213	81	1	10164	0.51547	0.30311
+	LOC105372285	19	13971900	14018223	35	6	10164	0.51541	0.30313
+	NDC80	18	2536511	2626635	226	18	10164	0.51526	0.30319
+	NOSTRIN	2	168751539	168875339	269	30	10164	0.51506	0.30326
-	RSPH3	6	158966739	159035432	185	5	10164	0.51477	0.30336
-	RNF216	7	5610041	5816730	562	26	10164	0.51461	0.30341
+	FBRS	16	30623431	30680810	31	1	10164	0.51419	0.30356
+	NDUFS4	5	53525623	53693341	583	31	10164	0.51396	0.30364
+	DNAH10	12	123726133	123945720	272	18	10164	0.51359	0.30377
-	TMEM65	8	124296189	124407699	301	14	10164	0.51347	0.30381
-	MZB1	5	139377568	139424916	45	4	10164	0.51343	0.30383
+	TTC36	11	118492495	118541025	58	6	10164	0.51336	0.30385
+	ENGASE	17	79036523	79098603	151	22	10164	0.51325	0.30389
+	ZNF114	19	48235099	48297608	262	22	10164	0.51318	0.30391
-	SEMA5B	3	122899193	123063605	502	57	10164	0.51314	0.30393
+	PTBP2	1	96686605	96825049	90	4	10164	0.51295	0.30399
-	C8orf82	8	144516219	144564074	24	4	10164	0.51277	0.30405
-	BAK1	6	33562546	33615299	338	38	10164	0.5126	0.30412
+	WFDC2	20	45413648	45491532	226	37	10164	0.5125	0.30415
+	MFSD2A	1	39920112	39979968	89	9	10164	0.51244	0.30417
-	GLDC	9	6522464	6680692	404	36	10164	0.51205	0.30431
-	LOC105370641	14	95453143	95500924	116	15	10164	0.512	0.30433
+	PCDHGA10	5	141378176	141522979	258	21	10164	0.51192	0.30435
+	LPL	8	19904071	19977259	275	19	10164	0.51186	0.30438
+	STATH	4	69960931	70012456	100	10	10164	0.51182	0.30439
+	ACSF3	16	89058809	89165846	68	2	10164	0.51182	0.30439
+	PPP2R2A	8	26256491	26382680	148	20	10164	0.51171	0.30443
-	C2orf61	2	47076991	47190378	249	28	10164	0.51126	0.30458
-	LOC101929901	1	41365004	41415923	167	26	10164	0.5111	0.30464
-	LOC100506504	1	2171811	2219360	108	24	10164	0.51101	0.30467
+	ANXA9	1	150947023	151006063	79	8	10164	0.51082	0.30474
+	LOC105377595	4	186123229	186168843	170	20	10164	0.5108	0.30474
+	NCR1	19	54870992	54948208	183	19	10164	0.51071	0.30478
+	LOC105369188	22	37951323	37998098	42	5	10164	0.51063	0.3048
+	GMPR	6	16203580	16305549	337	28	10164	0.51032	0.30491
+	HLA-E	6	30454406	30504205	369	41	10164	0.50991	0.30506
-	MYCL	1	39885424	39937015	85	10	10164	0.50986	0.30508
+	VHL	3	10106635	10163670	290	21	10164	0.50976	0.30511
+	ST6GALNAC3	1	76039704	76644601	1336	103	10164	0.50972	0.30512
-	POLR1A	2	86015168	86141155	285	15	10164	0.50965	0.30515
+	SPRTN	1	231302936	231380551	64	10	10164	0.50931	0.30527
-	BTC	4	74734759	74829672	303	19	10164	0.50891	0.30541
+	LOC101929355	13	114137978	114233074	390	22	10164	0.5088	0.30545
+	ZNF879	5	178988732	179044387	173	10	10164	0.50858	0.30552
+	ZSCAN22	19	58292019	58352349	15	6	10164	0.50854	0.30554
-	HID1	17	74940742	75007805	46	10	10164	0.50849	0.30555
+	WDR36	5	111057172	111140502	257	25	10164	0.50841	0.30558
+	MIF	22	23859378	23905222	184	27	10164	0.50834	0.30561
+	SPATS1	6	44307660	44387167	166	24	10164	0.50834	0.30561

STRAND	SYMBOL	CHR	START	STOP	NSNPS	NPARAM	N	ZSTAT	P
-	ZSCAN18	19	58073834	58153433	186	6	10164	0.50823	0.30565
-	DMPK	19	45759709	45817557	101	12	10164	0.50777	0.30581
-	PGM3	6	83141972	83228936	204	29	10164	0.50777	0.30581
-	ST7L	1	112507804	112655838	239	17	10164	0.50761	0.30586
+	CASD1	7	94474858	94641872	324	26	10164	0.50751	0.3059
-	RSPH14	22	23049408	23180230	234	9	10164	0.50729	0.30598
-	GFOD2	16	67664533	67754370	57	5	10164	0.50728	0.30598
+	IL20RB	3	136922865	137021085	143	8	10164	0.50713	0.30603
+	RBM5	3	50053908	50128964	135	11	10164	0.50693	0.3061
+	CTTNBP2NL	1	112361178	112471164	161	5	10164	0.50641	0.30629
+	PTDSS1	8	96226886	96344552	74	6	10164	0.50602	0.30642
+	C5AR2	19	47296841	47352080	51	5	10164	0.50602	0.30642
+	TRIM49D2	11	89889064	89943063	7	3	10164	0.50561	0.30657
+	ZNF37A	10	38059336	38133352	133	5	10164	0.50537	0.30665
-	VPREB3	22	23742743	23789443	164	22	10164	0.50528	0.30668
-	BOD1L1	4	13558738	13662705	171	11	10164	0.50527	0.30668
-	CAMK2D	4	113441032	113797133	470	35	10164	0.50521	0.30671
+	LOC105371789	17	44075810	44130524	35	7	10164	0.50518	0.30672
-	HR	8	22104419	22166053	54	4	10164	0.50509	0.30675
-	LOC105378148	6	169320544	169375581	16	3	10164	0.50509	0.30675
-	SMC1B	22	45334063	45448629	335	15	10164	0.50498	0.30679
+	APLF	2	68432559	68590162	224	6	10164	0.5049	0.30681
-	DCLRE1C	10	14894611	14989432	301	31	10164	0.5047	0.30688
+	C3orf35	3	37351269	37445497	80	5	10164	0.50473	0.30688
+	SLC16A5	17	75052727	75116162	137	15	10164	0.50462	0.30691
-	GPR1	2	206165316	206253047	184	18	10164	0.50445	0.30697
-	SLC6A9	1	43986483	44066492	138	30	10164	0.50418	0.30707
-	SCML4	6	107687297	107883659	741	52	10164	0.50415	0.30708
+	IQCJ	3	159034252	159276307	422	26	10164	0.504	0.30713
-	TPGS2	18	36770024	36864393	20	2	10164	0.50386	0.30718
-	TSPEAR	21	44487892	44746580	648	29	10164	0.50351	0.3073
-	TMEM207	3	190418655	190484876	197	23	10164	0.50335	0.30736
+	SERPINA3	14	94577377	94634053	135	19	10164	0.50309	0.30745
+	NTF3	12	5397114	5505299	353	42	10164	0.50309	0.30745
-	PAMR1	11	35421827	35565300	534	66	10164	0.50309	0.30745
-	DUSP11	2	73752198	73815157	3	1	10164	0.50295	0.3075
-	CD300LD	17	74569972	74627231	229	27	10164	0.50254	0.30765
-	FUOM	10	133342775	133393025	19	2	10164	0.50232	0.30772
+	IL37	2	112876064	112928882	128	12	10164	0.50221	0.30776
-	LOC149373	1	231174099	231222627	143	21	10164	0.50207	0.30781
-	RRAGC	1	38828197	38894823	143	12	10164	0.50203	0.30782
-	FAM53B	10	124609294	124779361	589	49	10164	0.50177	0.30791
-	MAPK12	22	50242902	50296759	49	8	10164	0.50162	0.30797
-	ZMYM6NB	1	34971526	35020347	68	2	10164	0.50126	0.30809
-	LSM12	17	44024635	44102619	61	7	10164	0.50087	0.30823
+	TBC1D23	3	100225817	100335252	167	13	10164	0.50081	0.30825
+	SOAT1	1	179258714	179368680	424	22	10164	0.50078	0.30826
-	ZNF786	7	149059641	149125777	95	3	10164	0.50075	0.30827
-	UBAC1	9	135922969	135996380	74	3	10164	0.50041	0.30839
+	ANKRD32	5	94580858	94724918	333	16	10164	0.50033	0.30842
+	BSG	19	536277	593493	189	23	10164	0.50032	0.30843
-	DCP1B	12	1936048	2039511	130	12	10164	0.5002	0.30847
+	C6orf25	6	31685808	31736710	227	56	10164	0.49968	0.30865
-	POC1B	12	89409718	89561262	4	2	10164	0.49968	0.30865
+	HELT	4	184983841	185030804	80	10	10164	0.49946	0.30873
-	ST18	8	52100832	52444902	578	47	10164	0.49935	0.30877
+	LOC105378832	1	87184918	87271689	128	8	10164	0.49934	0.30877
-	SMYD3	1	245739340	246542342	2509	72	10164	0.49907	0.30886
-	DNAJB8	3	128452432	128502248	214	50	10164	0.49899	0.30889
+	STXBP3	1	108711663	108819526	241	10	10164	0.49894	0.30891
+	PNPLA3	22	43888739	43957568	116	8	10164	0.49879	0.30896
+	EEF1B2	2	206124594	206172929	119	8	10164	0.49799	0.30924
-	LOC105375108	6	30454493	30502855	360	40	10164	0.49777	0.30932
-	LILRB3	19	54206278	54258087	37	6	10164	0.49768	0.30936
-	C9orf24	9	34369019	34433577	57	6	10164	0.49746	0.30943
-	RNF103-CHMP3	2	86493430	86756122	173	7	10164	0.49746	0.30943
-	IGSF9	1	159917039	159980596	66	12	10164	0.49727	0.3095
-	COMMD9	11	36262292	36324449	217	31	10164	0.49725	0.30951
-	MYO9A	15	71812291	72153464	366	11	10164	0.49702	0.30959
+	IPO13	1	43911806	43978022	110	10	10164	0.497	0.30959
+	S1PR3	9	88956409	89015155	139	14	10164	0.49685	0.30965
-	DYM	18	49031474	49495709	1055	17	10164	0.49673	0.30969
-	LRP6	12	12106025	12302007	535	46	10164	0.49652	0.30976
+	KRTAP6-3	21	30557440	30603055	124	10	10164	0.49647	0.30978

STRAND	SYMBOL	CHR	START	STOP	NSNPS	NPARAM	N	ZSTAT	P
+	OR5K4	3	98318854	98364819	153	14	10164	0.4961	0.30991
-	OSER1	20	44185939	44245906	143	16	10164	0.49606	0.30993
+	FAM177A1	14	35009907	35093383	217	15	10164	0.49603	0.30994
-	GPATCH4	1	156584308	156636496	111	12	10164	0.49535	0.31018
+	LOC105374086	3	126178254	126257330	383	14	10164	0.49507	0.31027
+	ATP2A1	16	28843488	28914509	47	1	10164	0.4949	0.31034
+	ERCC4	16	13885145	13962348	87	2	10164	0.49475	0.31039
-	FTCD	21	46126151	46190856	280	59	10164	0.49454	0.31046
+	ZBTB38	3	141289213	141459792	399	52	10164	0.49408	0.31062
-	MBD2	18	54141601	54259788	168	14	10164	0.49375	0.31074
-	TLN1	9	35687337	35767395	106	21	10164	0.49356	0.31081
-	KCNA2	1	110583580	110666514	46	5	10164	0.49345	0.31085
-	C22orf24	22	31915316	31980362	169	26	10164	0.4928	0.31108
+	LOC105370943	15	40869922	40918532	106	11	10164	0.4927	0.31111
+	HOXC10	12	53950162	54000279	25	5	10164	0.49266	0.31113
-	IFNA10	9	21196181	21242143	217	13	10164	0.49247	0.31119
-	DCUN1D1	3	182928158	183020947	109	12	10164	0.49248	0.31119
+	LY6G6D	6	31680356	31727804	217	55	10164	0.49236	0.31123
-	TUFM	16	28832411	28881511	51	1	10164	0.49233	0.31124
+	TGFB1I1	16	31437155	31487960	21	4	10164	0.49228	0.31126
-	EBPL	13	49650676	49726487	248	26	10164	0.49223	0.31128
+	PHYHD1	9	128885895	128952041	95	9	10164	0.49224	0.31128
+	TBC1D26	17	15697277	15754784	12	1	10164	0.49187	0.3114
+	OR4C3	11	48289941	48335930	145	8	10164	0.49176	0.31144
+	STAM	10	17609125	17726824	463	24	10164	0.49169	0.31147
+	DTX3L	3	122529338	122585203	142	19	10164	0.49169	0.31147
+	ARHGEF12	11	120301406	120499936	199	13	10164	0.49159	0.3115
+	CCDC84	11	118959353	119025793	199	13	10164	0.49162	0.3115
-	TSR3	16	1339240	1386872	2	1	10164	0.49145	0.31155
+	MAP10	1	232769892	232820346	178	15	10164	0.49096	0.31173
-	CACNG2	22	36550869	36737645	212	27	10164	0.49087	0.31176
-	PTGER3	1	70842353	71082814	534	42	10164	0.49076	0.3118
[truncated: 726,493 more chars]
